# A general method for the metal-free, regioselective, remote C–H halogenation of 8-substituted quinolines†
†Electronic supplementary information (ESI) available. See DOI: 10.1039/c7sc04107a


**DOI:** 10.1039/c7sc04107a

**Published:** 2018-01-05

**Authors:** Damoder Reddy Motati, Dilipkumar Uredi, E. Blake Watkins

**Affiliations:** a Department of Pharmaceutical Sciences , College of Pharmacy , Union University , Jackson , Tennessee , 38305 USA . Email: bwatkins@uu.edu ; Email: dreddy@uu.edu

## Abstract

An operationally simple and metal-free protocol for geometrically inaccessible C5–H halogenation of a range of 8-substituted quinoline derivatives has been established. The reaction proceeds under air, with inexpensive and atom economical trihaloisocyanuric acid as a halogen source (only 0.36 equiv.), at room temperature. Exceptionally high generality with respect to quinoline is observed, and in most instances, the reaction proceeded with complete regioselectivity. Quinoline with a variety of substituents at the 8-position gave, exclusively, the C5-halogenated product in good to excellent yields. Phosphoramidates, tertiary amides, *N*-alkyl/*N*,*N*-dialkyl, and urea derivatives of quinolin-8-amine as well as alkoxy quinolines were halogenated at the C5-position *via* remote functionalization for the first time. This methodology provides a highly economical route to halogenated quinolines with excellent functional group tolerance, thus providing a good complement to existing remote functionalization methods of quinolin-8-amide derivatives and broadening the field of remote functionalization. The utility of the method is further showcased through the synthesis of several compounds of biological and pharmaceutical interest.

## Abstract 2

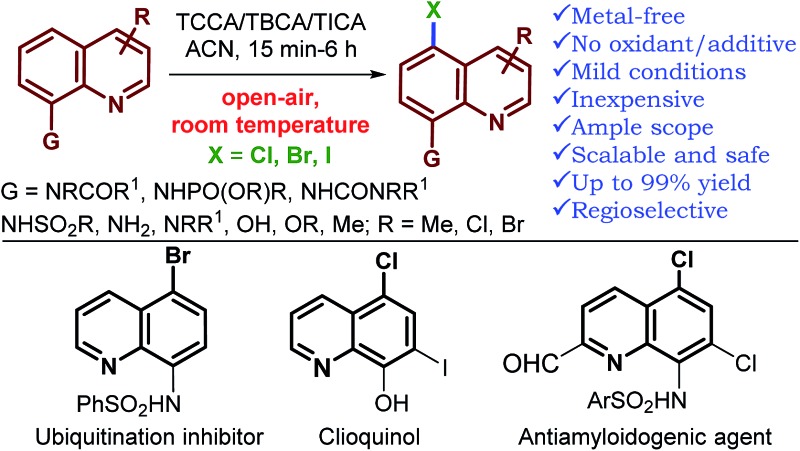
An operationally simple, metal-free protocol for regioselective halogenation of a range of 8-substituted quinolines has been established using recyclable trihaloisocyanuric acids.

## Introduction

Approaches to the functionalization of unactivated carbon-hydrogen (C–H) bonds is an area of great importance. C–H bond activation/functionalization is an atom economical and eco-friendly strategy for streamlining the transformation of one of the most fundamental and ubiquitous linkages in organic molecules into a range of functional groups.^1^ Achieving site selectivity in C–H bond functionalization is a key challenge in organic synthesis due to the subtle differences in the reactivity of various C–H bonds within a given molecule. Recently, remarkable advances have been realized in the highly selective and geometrically accessible C–H bond functionalization of various aromatic/heteroaromatic and aliphatic compounds.^2^ Here cyclometalation is facilitated *via* chelation assistance to achieve regioselectivity (directing group assisted C–H functionalization).^3^ In contrast, functionalization of a regioselective, remote C–H bond is a long-standing challenge and ascendant topic for the chemistry community and would provide access to a wide variety of derivatives.^4^

**Fig. 1:**
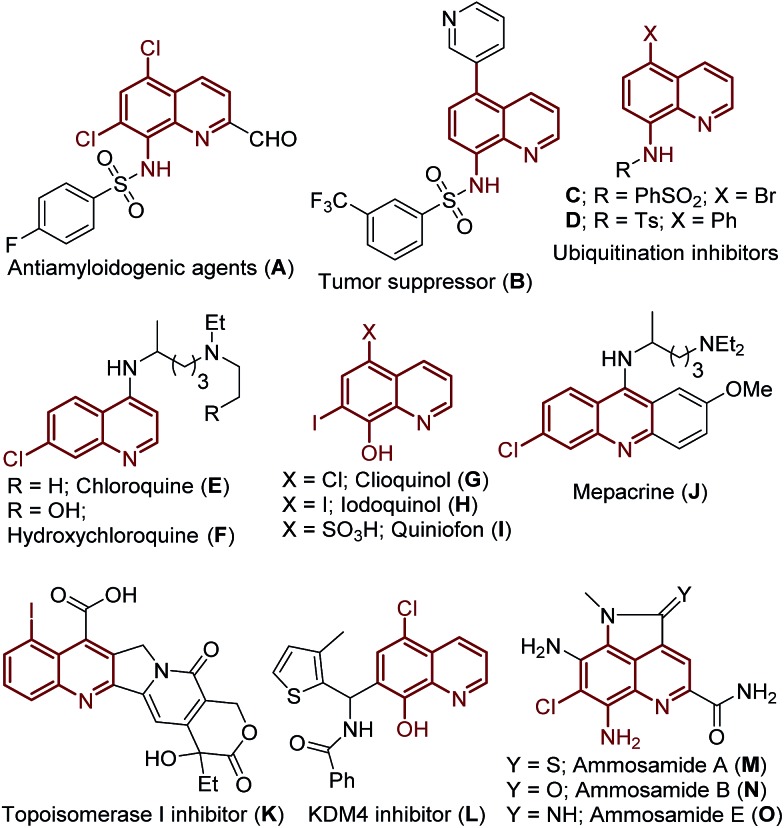
Examples of biologically active compounds and natural products featuring (halo)-quinoline motifs.

The quinoline framework has received significant attention over the past century due to its frequent occurrence in bioactive natural products,^5^ pharmaceuticals,^6^ materials^7^ and agrochemicals^8^ (Fig. 1), including the following drugs: chloroquine (**E**), hydroxychloroquine (**F**), clioquinol (**G**), iodoquinol (**H**), quiniofon (**I**), mepacrine (**J**), tafenoquine and primaquine; medicinally important quinoline motifs: antiamyloidogenic agent (**A**), tumor suppressor (**B**), ubiquitination inhibitors (**C**/**D**), topoisomerase I inhibitor (**K**), KMD4 inhibitor (**L**), and bioactive natural products: ammosamides A, B and E (Fig. 1, **M–O**). Additionally, great advancement has been realized in the valuable applications of the quinoline framework as a bidentate directing group^9^ in the arena of C–H activation/functionalization processes, after the seminal discovery of 8-aminoquinoline as a bidentate directing group by Daugulis in 2005.^10^ Consequently, there is great interest in the development of novel protocols for the preparation of halogenated quinolines.

**Scheme 1:**
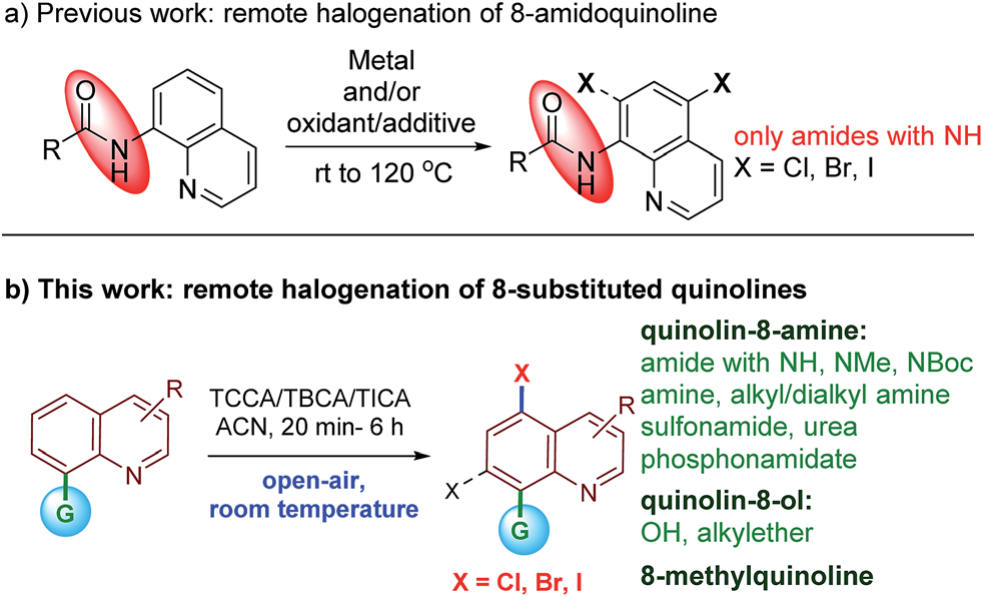
Remote halogenation of quinoline at C5 and/or C7-position.

Early precedent for the regioselective, remote C5-halogenation of *N*-(quinolin-8-yl)benzamide was established by Stahl and co-workers in 2013. In this pioneering study, 8-amidoquinoline was chlorinated under Cu-mediated conditions.^11^ Furthermore, we and others have leveraged the C5-remote functionalization of quinoline amides using sulfonation, halogenation, amination, and carbon–carbon bond formation using different metal catalysts.^12^ Among many others, the halogenation of remote C–H bonds of quinoline continues to hold much appeal due to the large number of halogenated quinolines possessing pharmacological properties (Fig. 1). Subsequently, Cu, Pd, and Fe mediated/catalyzed strategies for remote C5- and/or C7-halogenations have been reported by various groups (Scheme 1).^13^–^15^ Very recently, Li *et al.* reported transition metal-free remote C5-chlorination (at 130 °C) and bromination (at rt) of secondary amides of quinolin-8-amine using oxone and an excess of a halogen source. No iodination was reported under these oxidative conditions.^16^ Similarly, in 2017, Zhang and Ghosh independently reported transition metal-free C5-halogenation of 8-amidoquinolines using K_2_S_2_O_8_ at higher temperatures, affording moderate to good yields.^17^ Although these halogenations of quinoline have been reported, a facile and metal-free reaction for C5-halogenation is still rare.^18^,^19^ Additionally, the reported methods have several limitations. For instance, to the best of our knowledge in the reported examples the substrate scope is largely restricted to 8-*NH*-amides of quinolines. In most cases the reaction proceeded either with metal-mediated/catalyzed and/or oxidant/additive conditions. The reactions involved unfavorable stoichiometric amounts of the halogen source and higher temperatures. They also require an inert atmosphere for the reaction to progress. In addition, these metals/oxidants are often difficult to separate from the reaction mixture and require special attention for waste disposal. These factors limit the practicality for large-scale use. In continuation of our work on C–H bond activation/functionalization reactions;12a,^20^ herein, we report an atom-economical, safe, inexpensive, air- and moisture-tolerant protocol for remote C5-halogenation (chlorination, bromination and iodination) of an array of 8-substituted quinoline derivatives in high yields and with excellent regioselectivity at room temperature under metal-free conditions.

## Results and discussion

We began our investigation into the regioselective, remote halogenation of quinolines with an evaluation of a range of benchmark organic halogen reagents and solvents using *N*-(quinolin-8-yl)acetamide (**1a**) as a model substrate (Table 1). Initially, **1a** was treated with *N*-chlorosuccinimide (NCS) at room temperature in CH_2_Cl_2_ for 24 h. To our delight, the remote C5-chlorination product **2a** was obtained, although in only 15% yield (Table 1, entry 1). The yield of **2a** was slightly improved when acetonitrile was used as a solvent (24%, Table 1, entry 2). Interestingly, treatment of **1a** with 1,3-dichloro-5,5-dimethylhydantoin (DCDMH, 0.55 equiv.) in acetonitrile led to **2a** in excellent yield (86%) at rt under an open-air atmosphere (Table 1, entry 3). Next, **1a** was stirred with 0.36 equivalents of trichloroisocyanuric acid (TCCA) in acetonitrile to afford the desired product **2a** in 98% yield in only 15 min (Table 1, entry 4). Acetonitrile was found to be the most efficient solvent among the various solvents examined under TCCA conditions (Table 1, entries 5–9).

**Table 1:** Optimization of reaction conditions^*a*^

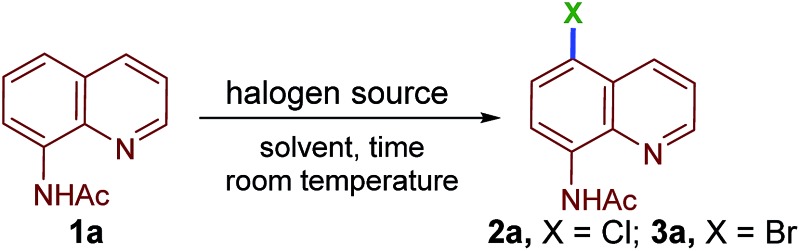				
Entry	Halogen source	Solvent	Time	Yield^*b*^ (%)
1^*c*^	NCS	CH_2_C1_2_	24 h	15
2^*d*^	NCS	CH_3_CN	24 h	24
3	DCDMH	CH_3_CN	30 min	89
**4**	**TCCA**	**CH**_**3**_ **CN**	**15 min**	**98**
5	TCCA	CH_2_Cl_2_	20 min	98
6^*e*^	TCCA	Water	24 h	17
7	TCCA	THF	4 h	56
8	TCCA	EtOH	30 min	96
9	TCCA	MeOH	30 min	97
10	NBS	CH_3_CN	45 min	82
11	DBDMH	CH_3_CN	30 min	95
12	DBCA	CH_3_CN	30 min	92
**13**	**TBCA**	**CH**_**3**_ **CN**	**30 min**	**96**

^*a*^Reaction conditions: **1a** (0.4 mmol) and halogen source: NCS or NBS (0.4 mmol); DCDMH or DBDMH or DBCA (0.22 mmol); TCCA or TBCA (0.145 mmol); solvent (3 mL) room temperature, open-air atmosphere.

^*b*^Isolated yields, entries 1–9: product is **2a**; entries 10–13: product is **3a**.

^*c*^65% of **1a** recovered.

^*d*^52% of **1a** recovered.

^*e*^70% of **1a** recovered.

Having determined the optimal conditions for remote chlorination, we turned our attention toward identifying a suitable reagent for remote C5–H bromination. Quinoline (**1a**) in acetonitrile was stirred in the presence of *N*-bromosuccinimide (NBS), 1,3-dibromo-5,5-dimethylhydantoin (DBDMH), dibromoisocyanuric acid (DBCA) or tribromoisocyanuric acid (TBCA) at rt. The desired product (**3a**) was isolated in excellent yields (Table 1, entries 10–13). The optimal conditions for chlorination were then established as shown in Table 1, entry 4 and bromination as shown in Table 1, entry 13.

Trichloroisocyanuric acid (TCCA) is a safe, easy-to-handle, shelf-stable solid frequently found in commercially available sanitizing agents, used as a disinfectant and preservative.^21^ The byproduct of chlorination *via* TCCA is cyanuric acid, which is easily isolable and can be reused to produce TCCA. From a green perspective, using trihaloisocyanuric acids for halogenation is advantageous for this reason. Additionally, it is the most atom-economical method currently known when compared with other reported methods.

Having identified optimal reaction conditions for halogenation (chlorination and bromination) of *N*-(quinolin-8-yl)acetamide (**1a**) with TCCA/TBCA, we examined the scope of remote halogenation with an array of quinolines. The results are shown in Table 2. A broad range of quinoline substrates readily participated in this mild and versatile halogenation with great efficiency. A variety of substitutions were tolerated under the present reaction conditions.

**Table 2:** Regioselective, C5-chlorination/bromination of diverse quinoline amides^*a*^

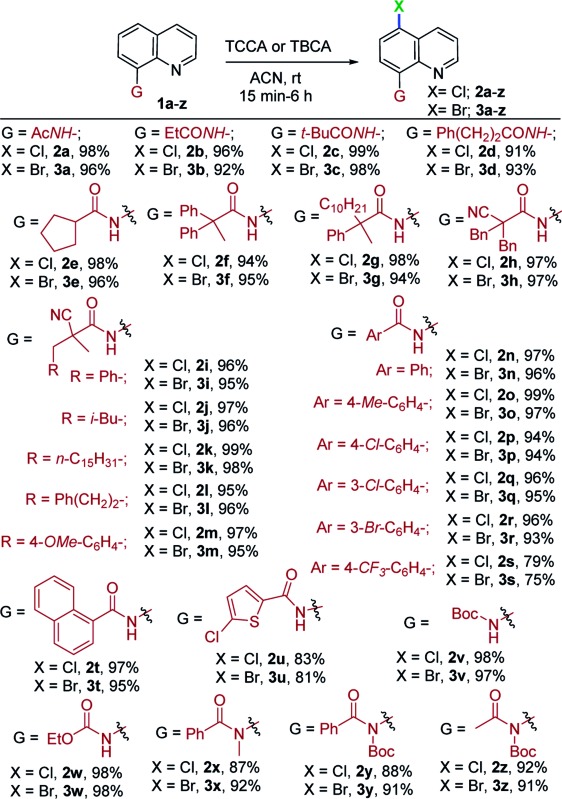

^*a*^Reaction conditions: **1** (0.4 mmol) and TCCA or TBCA (0.145 mmol), acetonitrile (ACN, 3 mL), rt, open-air atmosphere, 15 min to 6 h. Isolated yields.

Initially, the effects of substitution on the amine functionality of 8-aminoquinoline was investigated. Diversely substituted aliphatic and aromatic amides were well tolerated. The linear and branched alkyl amides were successfully converted to the corresponding C5-chlorinated/brominated products in excellent yields (91–99%; **2a–g** and **3a–g**, Table 2). Gratifyingly, α-cyano aliphatic amide (**1h**) proceeded smoothly under mild conditions to give **2h** in 97% and **3h** in 97% yields. Similarly, numerous other α-cyano amides (**1i–m**) with alkyl substitutions were halogenated in synthetically useful yields (95–99%; **2i–m**, **3i–m**). Furthermore, aromatic quinoline amides, including phenyl (**1n**), 4-OMe-(**1o**), halogenated benzamides (**1p–r**) and an electron-withdrawing benzamide (**1s**, 4-CF_3_–C_6_H_4_–) were compatible in this process and delivered corresponding products in good yields (**2n–s** and **3n–s**, 75–99%), thus offering ample opportunity for further derivatization. In addition, the reaction of naphthalene amide (**1t**) with TCCA and TBCA, afforded exclusively the C5-halogenated products (**2t**, 97% and **3t**, 95%), respectively, in excellent yields. Moreover, the heteroaromatic amide (**1u**) served well under the optimal conditions. Interestingly, Boc-protected (**1v**) and ethyl carbamate (**1w**) quinolines were halogenated in excellent yields and exclusive regioselectivity (97–98%, **2v**/**3v** and **2w**/**3w**). Notably, *tert*-amide derivatives (**1x–z**), subjected to the current conditions, gave chlorination and bromination at the C5 position in high yields (87–92%, **2x–z** and **3x–z**). The generation of C5-regioselective chlorination and bromination products of aliphatic/aromatic amides, and secondary as well as *tert*-amides indicated that the current mild, metal-free system is indeed attractive.

To further demonstrate the potential application of this protocol, numerous, variously substituted quinoline derivatives were utilized, as demonstrated in Table 3. *N*-(2-Methylquinolin-8-yl)benzamide (**1aa**) could be halogenated with TCCA or TBCA in 97% (**4a**) and 98% (**5a**) yields, respectively. Substituted urea derivatives of quinoline (**1ab** and **1ac**) were reactive, affording products in very high yields (98–99%, **4b, c** and **5b, c**). Surprisingly, the phosphoramidate scaffold (**1ad**) gave regioselective, halogenated products in 99% (**4d**) and 98% (**5d**) yields, respectively. As expected, the C5 substituted quinoline amide (**1ae**) was subjected to TCCA conditions and afforded the C7-chlorinated compound (**4e**) in 69% yield. Decomposition was observed when **1ae** was treated with TBCA. Furthermore, 5-methoxyquinolin-8-amine (**1af**) underwent halogenation to ultimately give C7-chlorination and bromination products in reasonably good yields (**4f**, 79% and **5f**, 63%). Similarly, *N*-(6-methoxyquinolin-8-yl)acetamide (**1ag**) tolerated the present conditions to afford C5 halogenated derivatives **4g** and **5g** in excellent yields. Next, halogen substitution on the pyridine ring of quinoline amides (**1ah** and **1ai**) were evaluated and generated the expected chlorination/bromination products in good yields (**4h**, **4i** and **5h**, **5i**; 79–90%). To the best of our knowledge, this marks the first report of C5-halogenation on urea and phosphoramidate quinoline derivatives using a remote functionalization protocol. DCDMH/DBDMH was also employed in the chlorination and bromination reactions of representative quinoline derivatives under optimal reaction conditions, to afford the corresponding C5-halogenated compounds in excellent yields (89–96%). It was observed that DBDMH and DCDMH have almost equal reactivity when compared to TCCA and TBCA (see ESI† for details).

**Table 3:** C5- or C7-chlorination/bromination of 8-substituted quinolines^*a*^ ^,^^*b*^

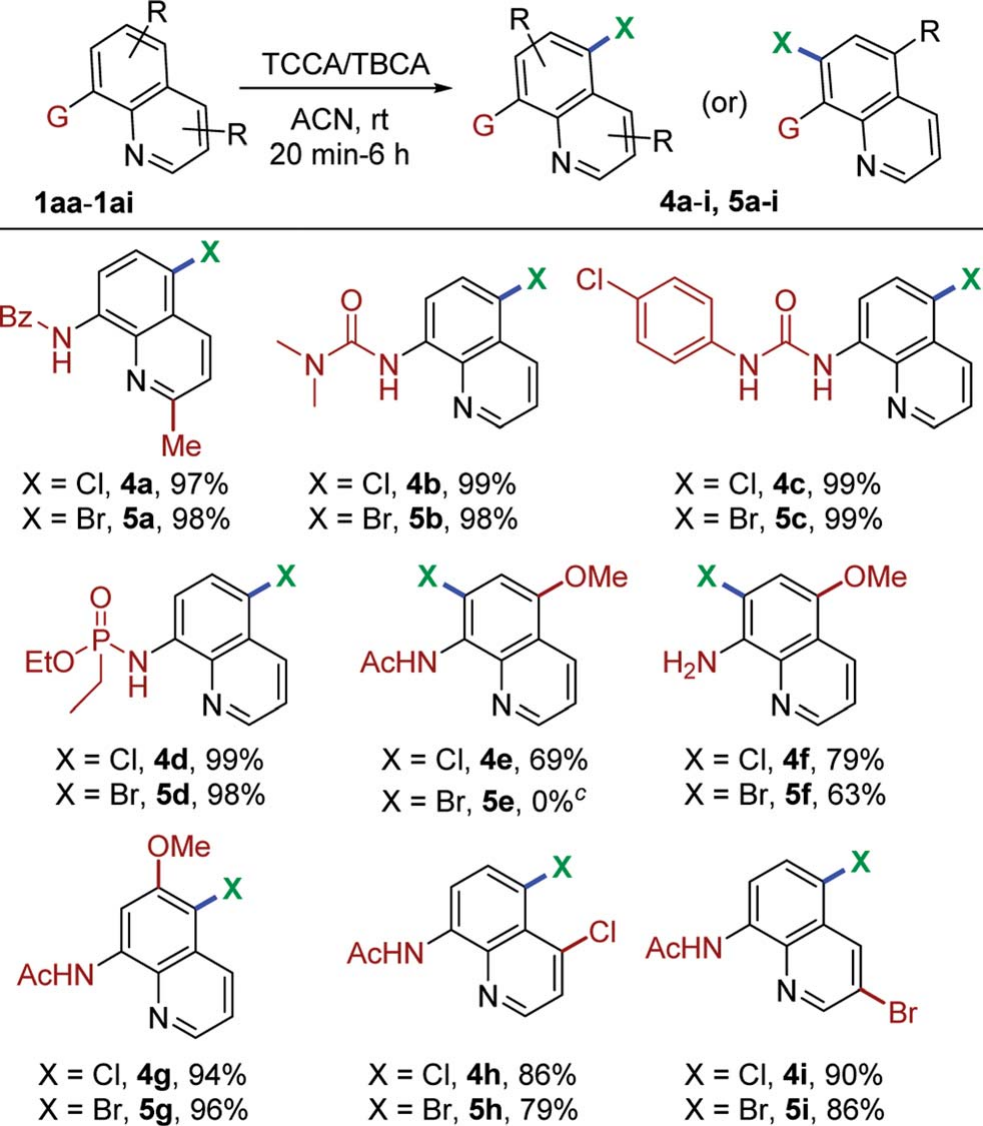

^*a*^All the reactions were conducted with 0.4 mmol of **1**.

^*b*^Isolated yields.

^*c*^Starting material decomposed.

Encouraged by the excellent performance of various 8-aminoquinoline derivatives in this mild and metal-free system for regioselective, remote chlorination/bromination, we continued to attempt halogenation with 8-substituted quinolines. As shown in Table 4, when *N*-(quinolin-8-yl)methanesulfonamide (**1aj**) was used as a substrate with TCCA and TBCA, C5-mono- and C5,C7-dihalogenation occurred to give a separable mixture of **6a** (85%), **7a** (2%), and **8a** (79%) and **9a** (3%), respectively. Other sulfonamide derivatives also underwent chlorination/bromination, giving C5-mono substitution as the major product (**6b**/**c** and **8b**/**c**; 80–82%) and C5,C7-dihalogenated product as a minor component (**7b**/**c** and **9b**/**c**; 5–8%). The monobromination product (**8c**) obtained in this mild and concise route, possesses ubiquitination inhibition activity.^9^ Interestingly, *N*-benzylquinolin-8-amine (**1am**) also worked in this transformation, providing the C5-mono and C5,C7-dihalogenated products in good yields (**6d**, 61%; **7d**, 13%; **8d**, 64%; **9d**, 12%). Similar results were observed in the case of chlorination of *N*,*N*-dibenzylquinolin-8-amine (**1an**). Interestingly, bromination of **1an** proceeded smoothly and afforded, exclusively, the C5-brominated substrate (**8e**) in 86% yield, presumably due to steric hindrance. Dibrominated compound **9e** was prepared separately using an excess of TBCA and longer reaction times (see ESI† for details). In addition, quinolin-8-amine (**1ao**) afforded predominantly the C5-mono halogenation products (**6f**, 70%; **7f**, 11%) (**8f**, 72%; **9f**, 7%), thus offering a straightforward and modular route for halogen substituted 8-aminoquinolines. Moreover, quinolin-8-ol (**1ap**) was also compatible with this mild halogenation procedure for accessing C5-mono- and C5,C7-dichlorinated as well as brominated hydroxy quinolines in moderate to good yields (**6g**, 71%; **7g**, 13%; **8g**, 72%; **9g**, 11%).

**Table 4:** C5-mono and C5,C7-dichlorination/bromination of quinolines^*a*^

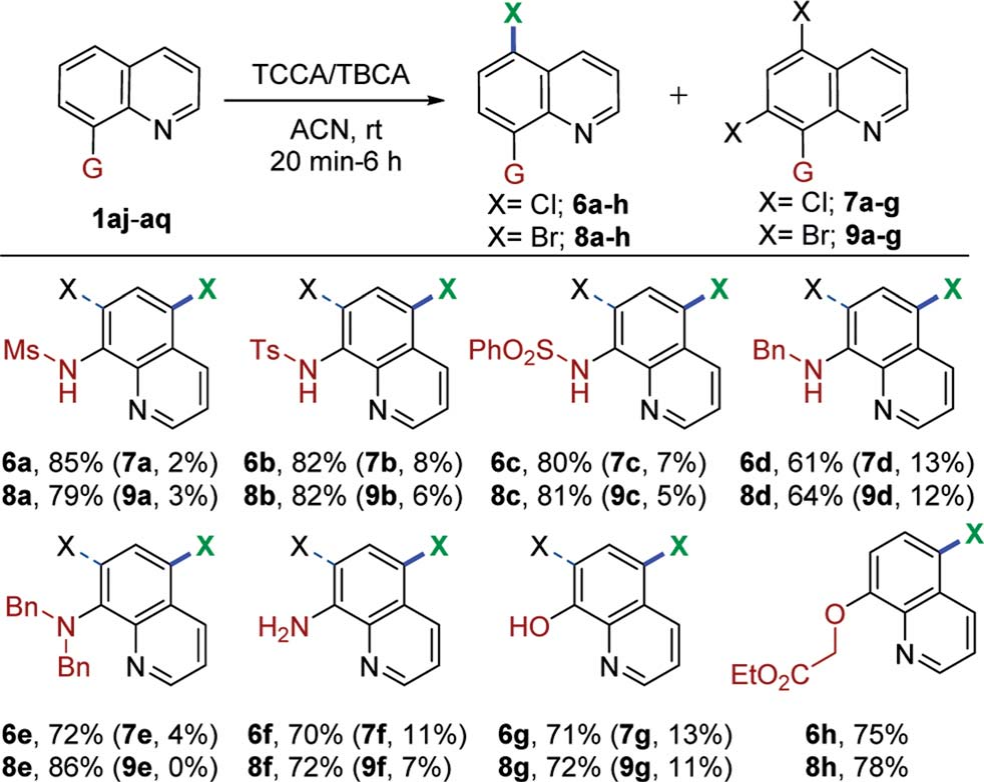

^*a*^Reaction conditions: **1** (0.4 mmol) and TCCA or TBCA (0.145 mmol), acetonitrile (ACN, 3 mL), rt, open-air atmosphere, 15 min to 6 h. The yields in parentheses are of the C5,C7-dihalogenation product obtained as a minor compound (see ESI for details). Isolated yields.

Surprisingly, we observed only C5-halogenation (**6h**, 75%; **8h**, 78%), when *O*-alkylated quinoline (**1aq**) was treated independently with TCCA and TBCA. It was noteworthy that valuable and diverse substrates were also compatible with this mild and metal-free transformation. Additionally, *N*-alkyl, *N*,*N*-dialkyl- and *O*-alkylated quinolines (**1am**, **1an** and **1aq**) were halogenated at the remote C5-position for the first time.

This metal-free, remote chlorination/bromination has proven to be a highly general and versatile method for a range of quinoline derivatives. Having achieved such success, we shifted our attention to remote C5–H iodination to highlight the scope of the present conditions. Initially, brief optimization experiments for iodination were carried out. Thus, **1a** with *N*-iodosuccinimide12*f* in acetonitrile for 24 h failed to produce the expected iodoquinoline derivative **10a**. Switching to 1,3-diiodo-5,5-dimethylhydantoin (DIH), **1a** at rt for 24 h furnished **10a** in 45% yield. Having demonstrated that remote iodination was feasible, we attempted triiodoisocyanuric acid (TICA). We were able to obtain the exclusive C5–H iodination product **10a** in very high yield (96%). With the reaction conditions for C5–H iodination established, a set of quinoline substrates were investigated (Table 5). Alkylated quinoline amides (acyclic, cyclic and α-cyano amide, **1a**, **1c**, **1n**, **1e**, **1i** and **1h**) were treated with TICA conditions, providing **10a–10f** in 88–97% yields. The reaction of *tert*-butyl quinolin-8-ylcarbamate (**1v**) delivered **10g** in 96% yield. Moreover, *tert*-amide derivative (**1x**) also worked in this transformation, affording the anticipated product **10h** in good yield (79%). Urea derivatives (**1ab**) proceeded to give **10i** in 99% yield. Delightfully, quinoline phosphoramidate (**1ad**) was well-suited for this reaction and provided the corresponding C5-iodo compound in excellent yield (**10j**, 98%). Somewhat surprisingly, when **1an** was subjected to the optimal conditions, the reaction progressed neatly and delivered only the C5-iodoquinoline derivative (**10k**) in 84% yield. Additionally, the treatment of substituted quinoline amides (**1ag** and **1ai**) under TICA conditions were successful and delivered the C5 iodination products in synthetically useful yields (**10l**, 95% and **10m**, 81%). Finally, **1aq** was well-tolerated in this system and gave the desired product (**10n**) in 54% yield .

**Table 5:** C5-iodination of various quinoline derivatives^*a*^ ^,^^*b*^

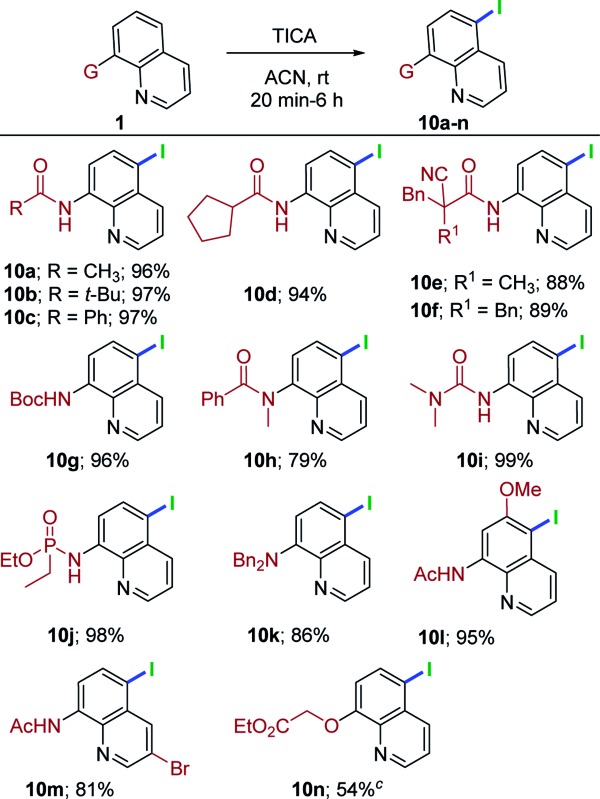

^*a*^Reactions were conducted on 0.4 mmol of **1** and 0.145 mmol of TICA.

^*b*^Isolated yields.

^*c*^24% of **1aq** recovered.

**Scheme 2:**
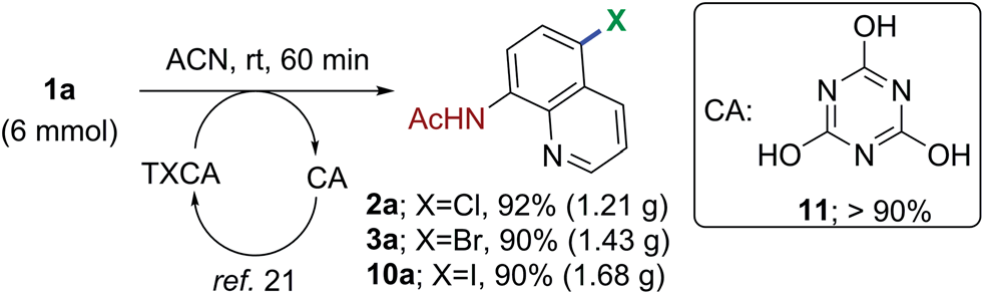
Gram-scale synthesis of halogenated quinolines.

The scalable nature of the remote halogenation was evaluated by conducting the reaction on a 6 mmol scale (Scheme 2). The reaction of **1a** with 2.2 mmol of TCCA/TBCA/TICA, afforded the corresponding halogenation products **2a** in 92%, **3a** in 90% and **10a** in 90% yields, respectively. Upon completion of the reaction, the byproduct, cyanuric acid (**11**, generated from trihaloisocyanuric acid) was precipitated in the reaction mixture and filtered (>90% yield). The recovered cyanuric acid can be reused to generate the trihaloisocyanuric acid.

**Scheme 3:**
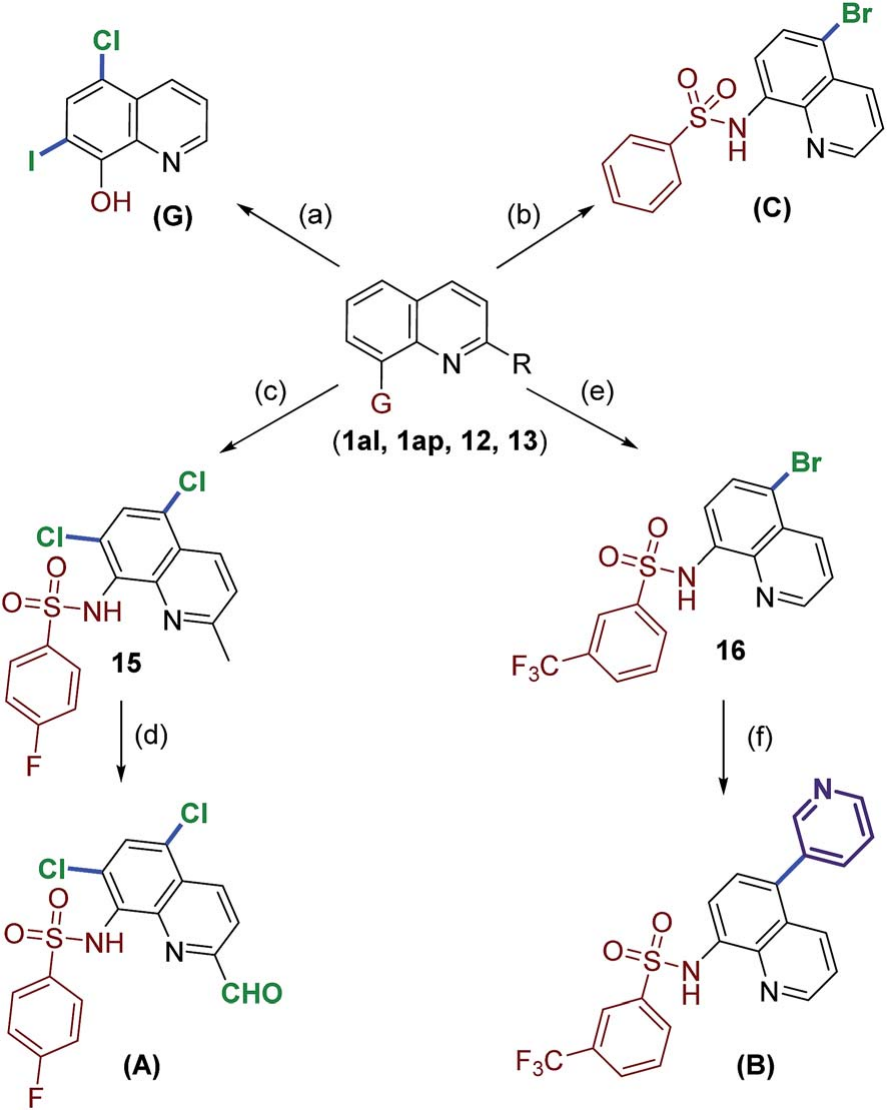
Synthesis of medicinally important quinolines using metal-free halogenation. Reagents and conditions: (a) (i) **1am**, NIS, CHCl_3_, 40 °C, 24 h. (ii) 7-Iodoquinolin-8-ol (**14**), TCCA (1.05 equiv.), CH_3_CN, rt, 2 h. (b) **1ai** TBCA (1.05 equiv.), CH_3_CN, rt, 30 min. (c) **12**, TCCA (2.2 equiv.), CH_3_CN, rt, 6 h. (d) SeO_2_, toluene, reflux, 4 h. (e) **13**, TBCA (1.05 equiv.), CH_3_CN, rt, 20 min. (f) Pyridin-3-ylboronic acid (17), Na_2_CO_3_, PdCl_2_(PPh_3_)_2_, 1,4-dioxane : water (3 : 1), MW, 100 °C, 20 min.

Next, demonstration of the potential synthetic applicability of the current method was attempted. As shown in Scheme 3, several quinoline compounds were successfully converted to medicinally useful candidates. Initially, 7-iodoquinolin-8-ol (**14**) was prepared using a literature procedure.^22^ Compound **14** was treated with TCCA to generate clioquinol (**G**) in 65% yield.

Alternatively, bromosulfonamide **C** (**8c**) was prepared from **1al** under optimal conditions. Compounds **C** exhibits ubiquitination inhibition activity.^9^,19b Moreover, the di-chlorination of 2-methylquinoline sulfonamide **12** gave the corresponding heteroaryl halide in high yield (**15**, 86%). The oxidation of the methyl group in **15** with SeO_2_ conditions afforded the antiamyloidogenic agent **A** in 72% yield.^23^ The power of this mild protocol is further showcased by preparing the tumor suppressor candidate (**B**) in a concise route. Compound **13** under standard conditions with TBCA furnished the C5-brominated substrate (**16**) as the major product. Finally, the coupling reaction of **16** with pyridin-3-ylboronic acid (**17**), gave the tumor suppressor molecule **B** in 79% yield.6f,13d

**Scheme 4:**
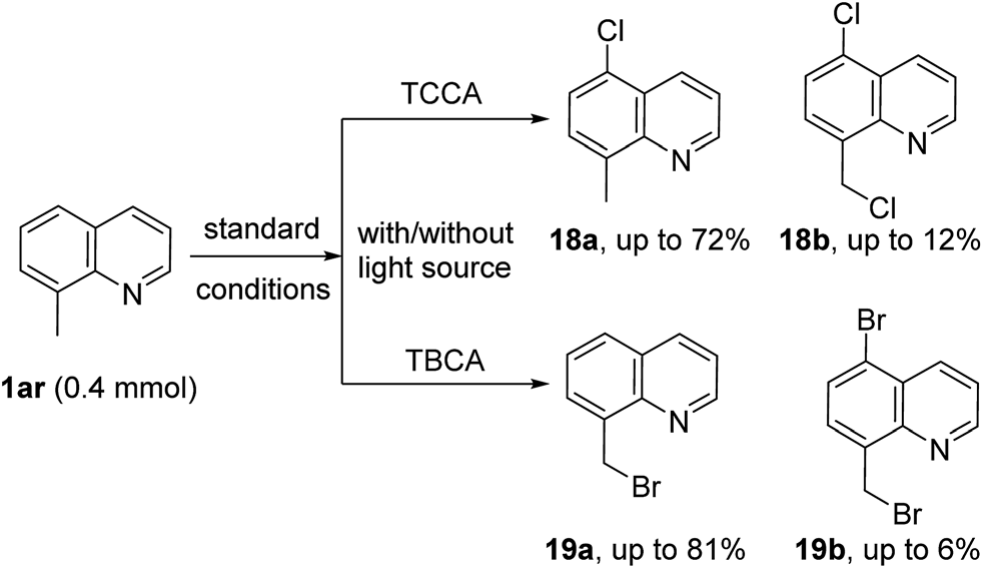
Halogenation of 8-methyl quinoline.

To further explore the scope of this metal-free protocol and to gain insight into the reaction mechanism, halogenation of 8-methyl quinoline (**1ar**) was attempted. As shown in Scheme 4, chlorination of **1ao** with TCCA gave a separable mixture of C5-chlorinated compounds **18a** (major product) and dichlorinated compound **18b** (minor product) under standard reaction conditions with longer reaction times. Likewise, when **1ar** was subjected to TBCA conditions, the methyl brominated product **19a** was isolated as the major product, along with a small amount of the brominated compound **19b**. The reaction times were drastically decreased when these reactions were exposed to a light source (see ESI† for details). Additionally, radical inhibition experiments were also performed. With 3 equivalents of TEMPO, the yield of the halogenated derivatives were lowered significantly (**2a**, 15%; **3a**, 13% and **10a**, 9%). Similar results were obtained with 3 equivalents of BHT as a radical inhibitor (**2a**, 18%; **3a**, 21% and **10a**, 12%). These results are in good agreement with previous reports of C5 halogenation reactions *via* radical mechanisms.12h,12i,13c,13f,13g,^15^,^16^ Based on the above results and literature reports,^19^ a plausible mechanism *via* a radical pathway is proposed (see ESI†).

## Conclusions

In conclusion, we have developed a general, operationally simple and metal-free reaction for the regioselective, remote C5–H halogenation (chlorination, bromination and iodination) of a broad range of 8-substituted quinolines using trihaloisocyanuric acids as an atom efficient halogen source for the first time. The reaction reveals good functional group tolerance and excellent reactivity with short reaction times under open-air conditions. Complete regioselectivity and good to excellent product yields were observed for most substrates. The applicability of this strategy is further showcased by the synthesis of pharmacologically active molecules, particularly antiamyloidogenic agent (**A**), tumor suppressor (**B**) and ubiquitination inhibitor (**C**) and an anti-fungal and protozoal drug, clioquinol (**G**).

## Conflicts of interest

There are no conflicts to declare.

## Supplementary Material

Supplementary information

## References

[cit1] Davies H. M., Du Bois J., Yu J. Q. (2011). Chem. Soc. Rev..

[cit2] Castro L. C. M., Chatani N. (2015). Chem. Lett..

[cit3] Andorfer M. C., Park H. J., Vergara-Coll J., Lewis J. C. (2016). Chem. Sci..

[cit4] Chu L., Shang M., Tanaka K., Chen Q., Pissarnitski N., Streckfuss E., Yu J.-Q. (2015). ACS Cent. Sci..

[cit5] Gaudêncio S. P., MacMillan J. B., Jensen P. R., Fenical W. (2008). Planta Med..

[cit6] Long K., Boyce M., Lin H., Yuan J., Ma D. (2005). Bioorg. Med. Chem. Lett..

[cit7] Xue G., Bradshaw J. S., Dalley N. K., Savage P. B., Izatt R. M., Prodi L., Montalti M., Zaccheroni N. (2002). Tetrahedron.

[cit8] (a) Markert J., Hagen H. and Wuerzer B., Germany Pat., DE 3223884 A1, 1985.

[cit9] Buhr W., Burckhardt S., Duerrenberger F., Funk F., Geisser P. O., Corden V. A., Courtney S. M., Davenport T., Slack M., Ridgill M. P., Yarnold C. J., Dawson G., Boyce S. and Ellenbroek A. A., WO2012110603A1, 2012.

[cit10] Zaitsev V. G., Shabashov D., Daugulis O. (2005). J. Am. Chem. Soc..

[cit11] Suess A. M., Ertem M. Z., Cramer C. J., Stahl S. S. (2013). J. Am. Chem. Soc..

[cit12] Reddy M. D., Fronczek F. R., Watkins E. B. (2016). Org. Lett..

[cit13] Guo H., Chen M., Jiang P., Chen J., Pan L., Wang M., Xie C., Zhang Y. (2015). Tetrahedron.

[cit14] Guan Y., Wang K., Shen J., Xu J., Shen C., Zhang P. (2017). Catal. Lett..

[cit15] Ding J., Zhang Y., Li J. (2017). Org. Chem. Front..

[cit16] Wang Y., Wang Y., Jiang K., Zhang Q., Li D. (2016). Org. Biomol. Chem..

[cit17] Jiao J.-Y., Mao Y.-J., Feng A.-W., Li X.-F., Li M.-T., Zhang X.-H. (2017). Tetrahedron.

[cit18] In 2017, Jinyi Xu and co-workers first reported metal-free C5 halogenation of quinoline amides with *N*-halosuccinimide as a halogen source. The reaction proceeded with 1.5 equiv. of NCS at rt for chlorination, and in the case of bromination and iodination, 3 equiv. of NBS/NIS were used at 100 °C. Acyl protection on aminoquinoline is necessary for mono halogenation under their conditions.19*a* During the preparation of this manuscript, a one-pot, metal-free bromination followed by C-heteroatom bond formation with NBS at 50 °C to 140 °C was reported by Qiu *et al.*19*b*

[cit19] Li Y., Zhu L., Cao X., Au C.-T., Qiu R., Yin S.-F. (2017). Adv. Synth. Catal..

[cit20] Reddy M. D., Watkins E. B. (2015). J. Org. Chem..

[cit21] Gabriela F. M., Marcio C. S. d. M. (2013). Curr. Org. Synth..

[cit22] Babudri F., Cardone A., Cioffi C. T., Farinola G. M., Naso F., Ragni R. (2006). Synthesis.

[cit23] Gautier E. C. L., Barnham K. J., Huggins P. J. and Parsons J. G., WO2008074068A1, 2008.

