# Supplementary material for: A general method for the metal-free, regioselective, remote C–H halogenation of 8-substituted quinolines
Source: Chem Sci. 2018 Jan 5;9(7):1782–8. doi: 10.1039/c7sc04107a (PMC5892134; doi:10.1039/c7sc04107a)

Electronic Supplementary Material (ESI) for Chemical Science.
This journal is © The Royal Society of Chemistry 2018

Supporting Information

for

A General Method for the Metal-free, Regioselective, Remote C-H Halogenation of 8-Substituted Quinolines

M. Damoder Reddy*, Uredi Dilipkumar, E. Blake Watkins*

Department of Pharmaceutical Sciences, College of Pharmacy, Union University, Jackson, Tennessee 38305, United States

E-mail: bwatkins@uu.edu, dreddy@uu.edu

Table of Contents

1) General experimental……………………..……………………………………………………S2

2) Structures of starting materials...S3-S4

3) Mechanistic studies……………………………………………………………………...S5-S9

4) Experimental procedures and characterization of compounds…………………………...…S10-S70

5) References……………………………………………………………………………….S70-S71

6) ^{1}H and $^{13}C\{^{1}H\}$ NMR spectra for new starting materials and all products; DEPT135 NMR and HRESMS for representative compounds………….……………………………..…….S72-S474

EXPERIMENTAL SECTION

General Experimental Procedures. Glassware was dried in an oven (120 °C), heated under reduced pressure, and cooled under argon before use. Unless otherwise noted, materials obtained from commercial suppliers were used without further purification. Reactions were monitored by thin-layer chromatography on Analtech silica gel plates using UV-light and ceric sulfate or *β*-naphthol for visualization. Column chromatography was performed on silica gel (230–400 mesh) using hexanes and ethyl acetate as eluent. Evaporation of solvents was conducted under reduced pressure at 50 °C. FTIR spectra were recorded neat on a Perkin-Elmer Spectrum 65. Microwave reactions were conducted in a Biotage Initiator Classic. NMR spectra were recorded on a Bruker Avance III 400 NMR spectrometer at 400 MHz (^{1}H) and 100 MHz (^{13}C), respectively. Deuterated chloroform was used as the solvent, and spectra were calibrated against the residual solvent peak (7.24 ppm for ^{1}H and 77.0 ppm for ^{13}C) or TMS. Chemical shifts (δ) and coupling constants (J) are given in ppm (parts per million) and Hz (Hertz), respectively. The following abbreviations were used to explain multiplicities: s = singlet, d = doublet, t = triplet, q = quartet, m = multiplet, bs = broad singlet. Low resolution ESI mass spectra were obtained on a Waters Acquity UPLC H-Class with PDA and SQ mass detectors using a Waters BEH C_{18} 1.7 μm column (2.1x50mm). High resolution mass spectra were obtained on VG 70–70H or LC/MSD trapSL spectrometer operating at 70 eV using a direct inlet system.

Figure 1: Structures of starting materials (1a-z, 1aa-ar, 12 and 13):

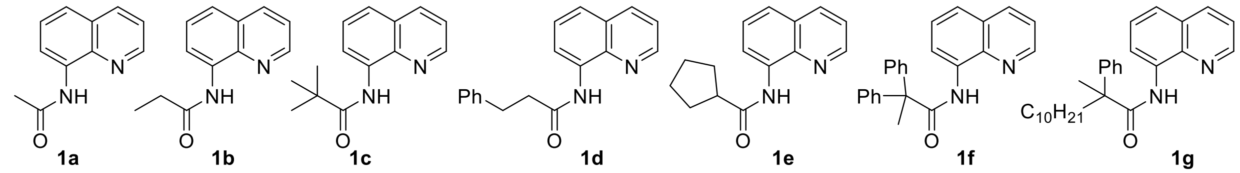

1a **1b** **1c** **1d** **1e** **1f** **1g**

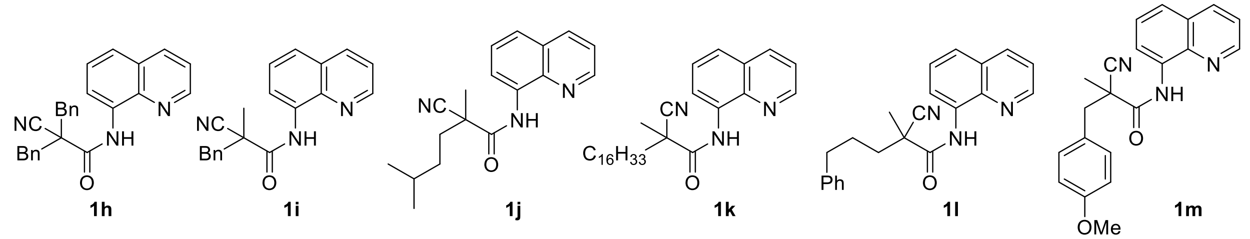

1h **1i** **1j** **1k** **1l** **1m**

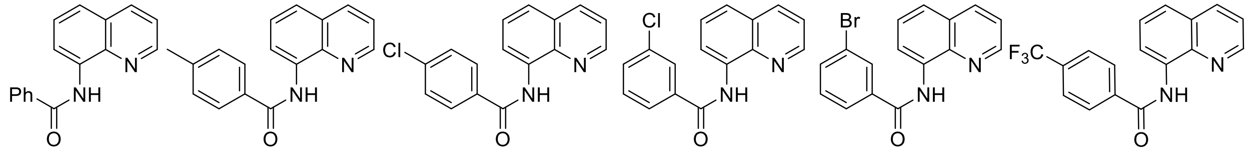

1n **1o** **1p** **1q** **1r** **1s**

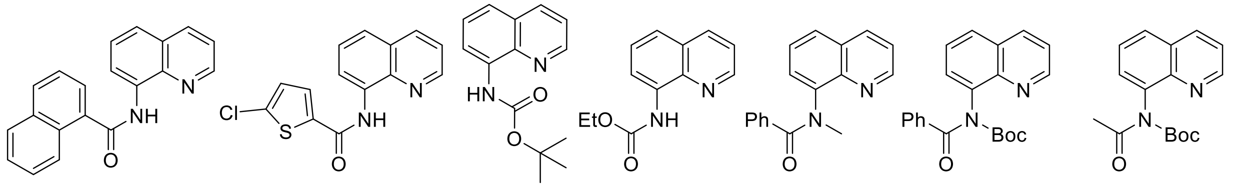

1t **1u** **1v** **1w** **1x** **1y** **1z**

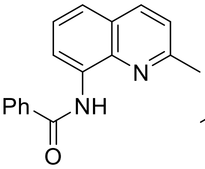

1aa

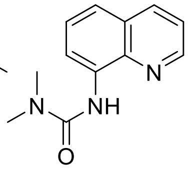

1ab

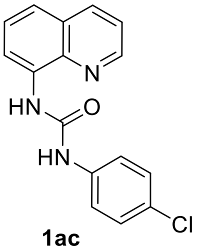

1ac

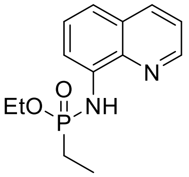

1ad

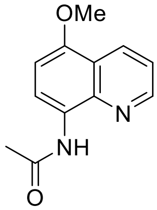

1ae

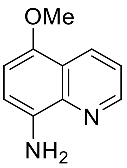

1af

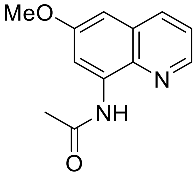

1ag

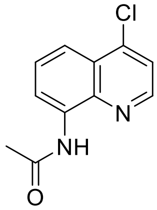

1ah

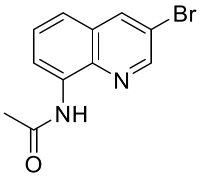

1ai

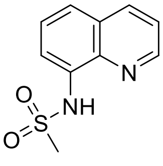

1aj

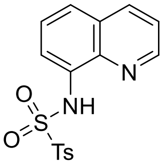

1ak

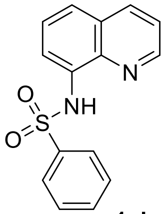

1al

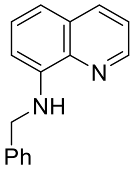

1am

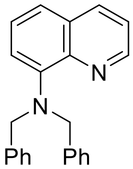

1an

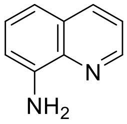

1ao

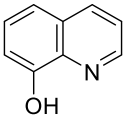

1ap

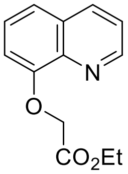

1aq

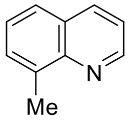

1ar

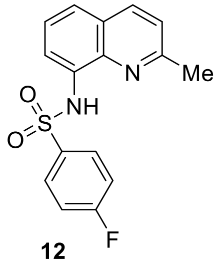

12

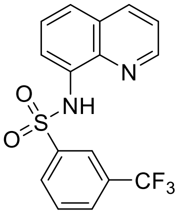

13

1) Mechanistic Studies:

i) Radical inhibition experiments: The general protocol described below was followed.

A 10 mL round bottom flask equipped with a magnetic stir bar was charged with quinoline (**1a**, 0.40 mmol) and acetonitrile (4 mL) under open air conditions at room temperature. To the stirred solution was added radical inhibitor (TEMPO or BHT, 0.4/1.2 mmol), followed by halogen source TCCA/TBCA/TICA (0.145 mmol) in one portion, and the resulting solution was stirred at room temperature for 6 h. The reaction mixture was then evaporated, and the crude residue was purified by flash chromatography (EtOAc/hexanes, 5:95 to 60:40) to yield the corresponding halogenated quinoline derivative **2a**, **3a** and **10a**.

A series of control experiments were performed to explore the reaction mechanism as shown in Scheme 1. In the chlorination reaction of **1a** (0.4 mmol) with TCCA in the presence of 0.4 mmol of TEMPO/BHT, the yield of **2a** was reduced to 51% (eq 1) and 63% (eq 3), respectively. A significant reduction in yield of **2a** was realized with 1.2 mmol of TEMPO/BHT in the chlorination reaction (eqs 2 and 4). Similarly, bromination and iodination of **1a** (0.4 mmol) with TBCA/TICA under TEMPO/BHT (1.2 mmol) conditions gave the C5-brominated and iodinated derivatives in very low yields (eq 5-8). Taken together, these results indicate that the halogenation reaction proceeds through a radical pathway.

a) Radical inhibition of chlorination reaction with TEMPO:

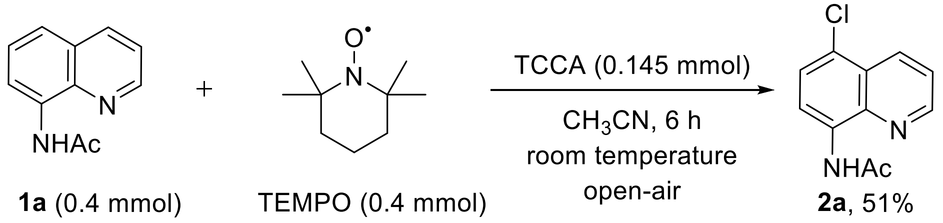

(eq 1)

1a (0.4 mmol) + TEMPO (1.2 mmol) —[TCCA (0.145 mmol); CH_3CN, 6 h; room temperature; open-air]→ **2a**, 15% **(eq 2)**

b) Radical inhibition of chlorination reaction with BHT:

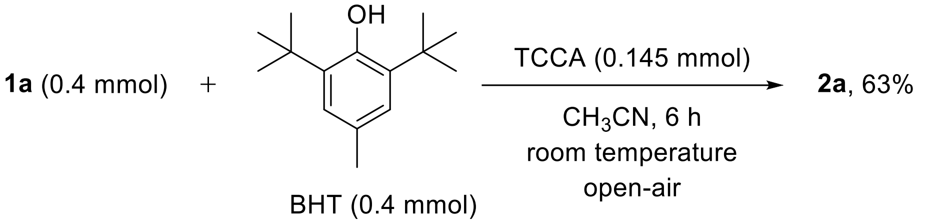

(eq 3)

1a (0.4 mmol) + BHT (1.2 mmol) —[TCCA (0.145 mmol); CH_3CN, 6 h; room temperature; open-air]→ **2a**, 18% **(eq 4)**

c) Radical inhibition of bromination reaction with TEMPO and BHT:

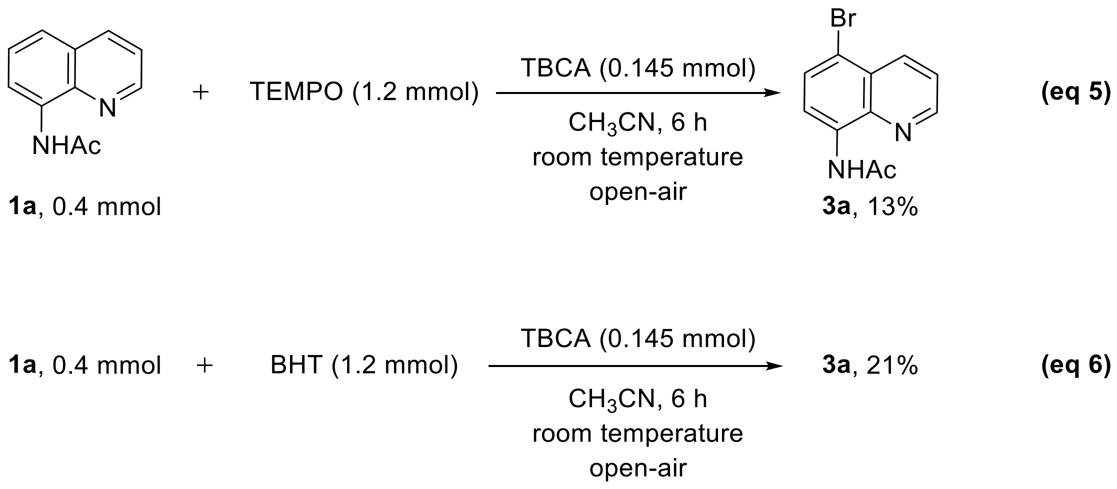

d) Radical inhibition of iodination reaction with TEMPO and BHT:

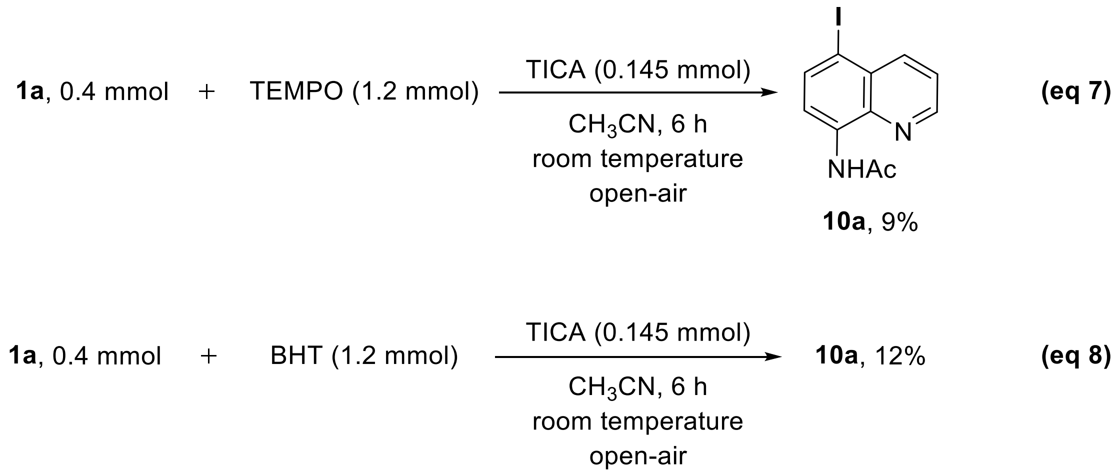

Scheme 1: Halogenation reaction of 1a with radical quenchers.

ii) Halogenation reaction of 8-methylquinoline with TCCA/TBCA:

A 10 mL round bottom flask equipped with a magnetic stir bar was charged with 8-methylquinoline (**1ar**, 0.40 mmol), and acetonitrile (3 mL) under open air conditions at room temperature. To the stirred solution was added the halogen source TCCA/TBCA (0.4 mmol) in one portion, and the resulting solution was stirred at room temperature for 48 hours. The reaction mixture was then evaporated, and the crude residue

was purified by flash chromatography (EtOAc/hexanes, 5:95 to 70:30) to yield the corresponding halogenated quinoline derivatives **18a/18b** and **19a/19b**.

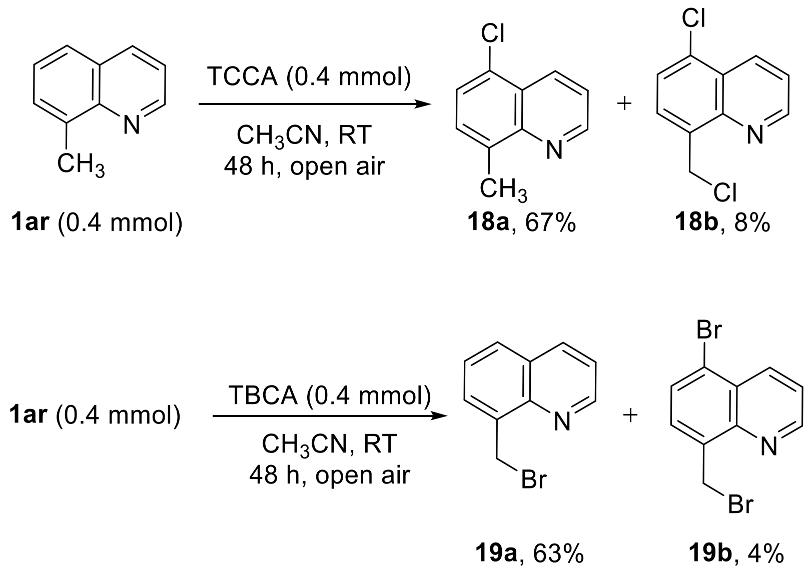

Scheme 2: Halogenation of 1ar under standard reaction conditions.

iii) Halogenation reaction of 8-methylquinoline with TCCA/TBCA under light source:

A 10 mL round bottom flask equipped with a magnetic stir bar was charged with 8-methylquinoline (**1ar**, 0.40 mmol), and acetonitrile (3 mL) under open air conditions at room temperature, and two 100 watt household lights were arranged approximately 15 cm from the reaction. To the stirred solution was added the halogen source TCCA/TBCA (0.4 mmol) in one portion, and the resulting solution was stirred at room temperature for 5-6 hours. The reaction mixture was then evaporated, and the crude residue was purified by flash chromatography (EtOAc/hexanes, 05:95 to 70:30) to yield the corresponding halogenated quinoline derivative **18a/18b** and **19a/19b**.

1ar (0.4 mmol) —[TCCA (0.4 mmol); CH_3CN, RT, 6 h; light source, open air]→ **18a**, 72% + **18b**, 12%

1ar (0.4 mmol) —[TBCA (0.4 mmol); CH_3CN, RT, 5 h; light source, open air]→ **19a**, 81% + **19b**, 6%

Scheme 3: Halogenation of 1ar under light source

The formation of the ring chlorination/bromination products along with benzylic halogenation on **1ar** with TCCA/TBCA suggests the reaction proceeds via a radical mechanism.[1] Additionally, acceleration of the reaction when exposed to light further supports the radical pathway. On the basis of experimental results and literature reports,[2] a plausible reaction mechanism is proposed in Scheme 4. The reaction is likely initiated through homolytic cleavage of the *N*-Cl bond of trichloroisocyanuric acid (**I**) to generate a chlorine radical and the nitrogen-centered radical **II**. The reaction propagates via abstraction of the C5 hydrogen of the quinoline derivative **1a** by **II** to generate intermediate **III**. Quenching of the carbon radical by chloride transfer generates the regioselective C5-chlorinated product **2a**. Product generation continues until the halogen source has been consumed.

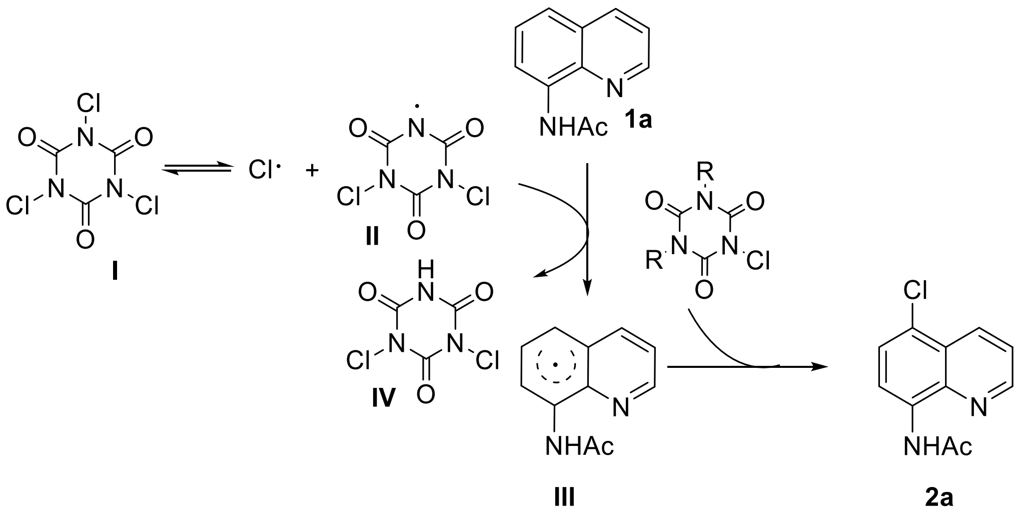

Scheme 4: Plausible reaction mechanism for halogenation of quinoline derivatives

2) Synthesis of 8-substituted quinoline starting materials (1a-z, 1aa-ar, 12 and 13):

Commercially available starting materials (**1af**, **1ag, 1ao**, **1ap** and **1ar**) were used without further purification. The remaining starting materials were synthesized as described below.

8-Amidoquinolines (**1a-z, 1aa-ab, 1ae-1ai**) were synthesized according to literature procedures.[3] Compounds **1a-c, 1e, 1n-u, 1w, 1aa, 1ab 1ae, 1ah** and **1ai** were prepared from the corresponding acid chlorides using Method 1.[3a-c] Other quinoline amides (**1d** and **1f-m**) were synthesized from the corresponding acids using Method 2.[3a, 3c, 3d, 4] Boc- (**1v**), *tert*-amide derivative **1x**, **1y** and **1z**, urea derivative **1ac** were prepared using literature procedures.[3b, 5] Phosphoramidate **1ad** was synthesized according to the method of Daugulis.[6] Sulfonamides **1aj-al, 12** and **13** were synthesized using microwave conditions.[7] *N*-benzyl and *N*,*N*-dibenzyl quinolines **1am** and **1an** were prepared using reported procedures.[5b, 8] Finally, alkylated quinoline **1aq** also prepared with reported method.[9]

The new quinoline derivatives **1g**, **1y**, **1z**, **1ac**, **1ae** and **12** were characterized using NMR, IR and mass spectral data. All other compounds are reported in the literature and their spectral data were in good agreement with reported compounds. (**1c, 1v, 1ab, 1ak, 1am** and **1an**[5b]; **1a, 1n** and **1x**[3b]; **1n-q, 1s** and **1t**[10]; **1h-m**[4]; **1d**[11]; **1e**[12]; **1f**[13]; **1r**[14]; **1c**, **1d**, **1p-r** and **1u**[3c]; **1b**[15]; **1w**[16]; **1aa**[17]; **1ad**[6]; **1aj** and **1ai**[2a]; **1aq**[3d]; **13**[7], **1ah** and **1ai** [18]

Synthesis of 8-amidoquinolines:

Method 1:[3a-c]

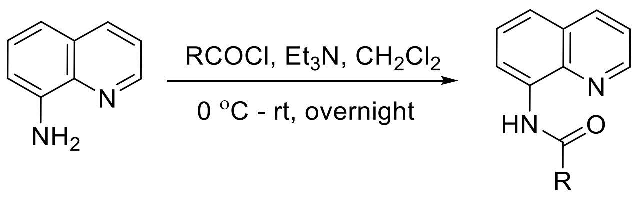

8-Aminoquinoline (4.0 mmol) and triethylamine (6.0 mmol) were dissolved in anhydrous dichloromethane (20 mL), and the mixture was cooled to 0 °C. The corresponding acid chloride (4.4 mmol), dissolved in 5 mL of dichloromethane, was added via syringe pump over 15 minutes. The reaction mixture was warmed to room temperature and stirred overnight. Dichloromethane (20 mL) was added, and the mixture was washed with saturated aqueous sodium bicarbonate (30 mL), 1N HCl (30 mL), and brine (30 mL), dried over $MgSO_4$ and the solvent was removed *in vacuo*. The crude product was purified by flash column chromatography (silica gel, EtOAc/hexanes, 05:95 to 30:70) to give the corresponding quinoline amide product (**1a-c, 1e, 1n-u, 1w, 1aa, 1ab, 1ae, 1ah, 1ae, 1ah** and **1ai**).

Note: For the synthesis of **1a**, **1b**, **1ab, 1ae, 1ah** and **1ai**, after completion of the reaction using Method 1, the mixture was washed with only brine solution (30 mL).

Method 2:[3a, 4]

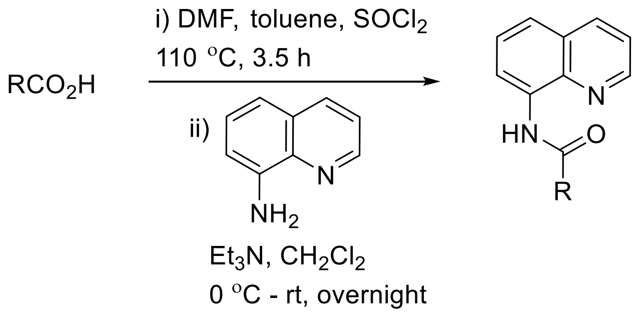

The corresponding carboxylic acid (5.0 mmol) and DMF (50 μL) were dissolved in anhydrous toluene (20 mL). After the addition of thionyl chloride (7.5 mmol), the mixture was heated to 110 °C for 3.5 h, after which the volatiles were removed *in vacuo*. A 100 mL round-bottom flask was charged with 8-aminoquinoline (4.0 mmol), triethylamine (8.0 mmol), and dichloromethane (20 mL) at 0 °C. The solution of the crude acid chloride in 5 mL of dichloromethane was added slowly via a syringe pump over a period of 15 minutes. The reaction mixture was warmed to room temperature and stirred overnight. Dichloromethane (20 mL) was added, and the mixture was washed with saturated aqueous sodium bicarbonate (40 mL), 1N HCl (40 mL), and brine (40 mL), dried over $MgSO_4$ and the solvent was removed

in vacuo. The crude product was purified by flash column chromatography (silica, EtOAc/hexanes 05:95 to 30:70) to afford the corresponding 8-amidoquinoline (**1d** and **1f-m**).

Synthesis of carbamate (1v):[5b]

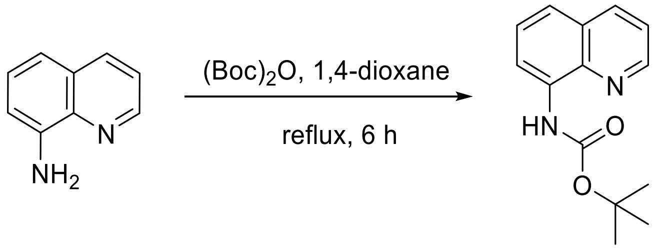

A mixture of 8-aminoquinoline (0.30 g, 2.08 mmol, 1.0 equiv), di-*tert*-butyl dicarbonate (0.908 g, 4.16 mmol, 2.0 equiv), and 1,4-dioxane (10 mL) was stirred at 102 °C for 6 h. The solvent was removed under reduced pressure. The residue was purified by column chromatography on silica gel (EtOAc/hexanes = 10:90) to give **1v** as a brown solid (0.488 g, 96% yield).

Synthesis of *tert*-amide (1x):[3b]

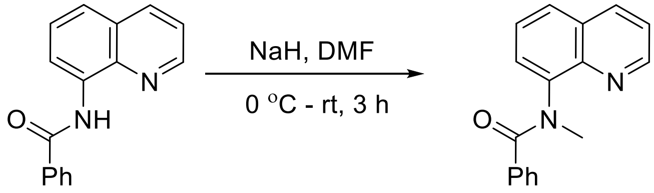

To a suspension of sodium hydride (49.5 mg, 1.24 mmol, 2.05 equiv.) in dry DMF (3 mL), was added a solution of *N*-(quinolin-8-yl)benzamide (**1n**, 150 mg, 0.604 mmol, 1 equiv.) in dry DMF (3 mL) at 0 °C. The reaction mixture was allowed to warm to room temperature and stirred for 2 h. Then, methyl iodide (49 μL, 0.785 mmol, 1.3 equiv.) was added dropwise, and the reaction was stirred at room temperature for 1 h. The reaction was diluted with CH_2Cl_2 (20 mL), and the organic layer was washed with water (3 x 20 mL), dried over anhydrous $MgSO_4$ and concentrated to dryness under reduced pressure. A pale, yellow oil

was obtained and purified by column chromatography on silica gel, eluting with CH_2Cl_2. The *N*-methyl amide (**1x**) was isolated as a white solid (148 mg, 93% yield).

Synthesis of 1y and 1z:[5c]

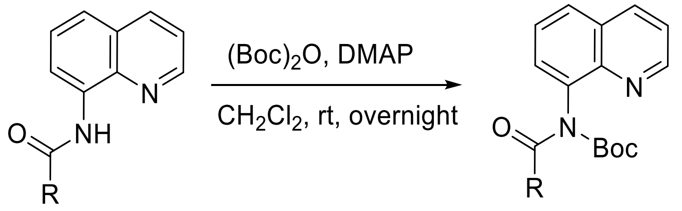

Boc anhydride (6 mmol) was added to a solution of **1n**/**1a** (3 mmol) and DMAP (4.5 mmol) in CH_2Cl_2 (40 mL), and the reaction mixture was stirred overnight. The reaction mixture was quenched with saturated aqueous NH_4Cl (40 mL) and extracted with CH_2Cl_2 (2 × 40 mL). The combined organic extracts were dried ($MgSO_4$), concentrated under reduced pressure, and purified by column chromatography (EtOAc/hexanes = 20:80) to give **1y** and **1z** in good yields.

Synthesis of urea derivative 1ac:[5d]

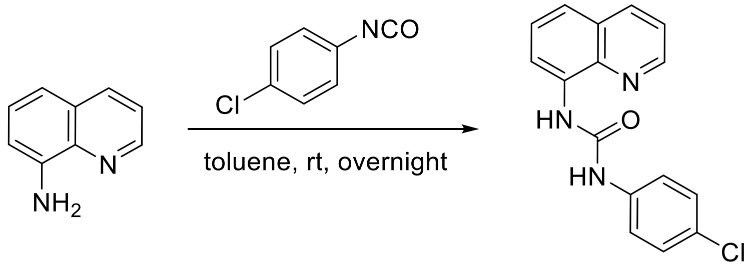

A flame-dried 100 mL round bottom flask with a stir bar was charged with 8-aminoquinoline (0.31 g, 1.96 mmol, 1.0 equiv.) and toluene (30 mL), resulting in a dark brown solution. The flask was fitted with a septum and placed under an atmosphere of argon. 1-Chloro-4-isocyanatobenzene (2.00 mmol, 1.01 equiv.) was added dropwise *via* syringe. This was stirred overnight, resulting in the precipitation of a brown solid, which was filtered under vacuum, yielding product without need for further purification.

Synthesis of ethyl *P*-ethyl-*N*-(quinolin-8-yl)phosphonamidate (1ad):[6]

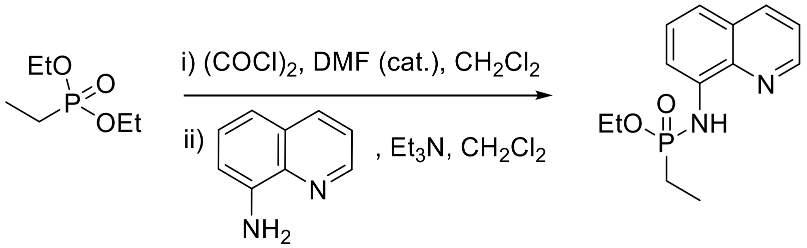

A 50 mL, oven-dried, round bottom flask was charged diethyl ethylphosphonate (3.24 mL, 20 mmol, 1 equiv.), DMF (5 drops), and CH_2Cl_2 (20 mL). The solution was kept under argon and placed into an ice/water bath, followed by slow addition of oxalyl chloride (5.23 mL, 60 mmol, 3 equiv.) over 5 min. The solution was kept at 0 °C for 30 min, warmed to room temperature, and stirred overnight. After completion, the crude mixture was evaporated under vacuum to remove solvent and excess oxalyl chloride. Toluene (10 mL) was added, and the mixture was concentrated again. The crude ethyl ethylphosphonochloridate was then dissolved in CH_2Cl_2 (10 mL). The resulting solution was kept under argon and placed into an ice/water bath followed by slow addition of Et_3N (3.5 mL, 25 mmol, 1.25 equiv.) over 5 min. The brownish solution was vigorously stirred for 30 min then warmed to room temperature for 1 h. The mixture was placed into an ice/water bath again, followed by slow addition of a CH_2Cl_2 (10 mL) solution of 8-aminoquinoline (2.88 g, 20 mmol, 1 equiv.) under nitrogen over 5 min. The suspension was stirred at room temperature overnight. The reaction was quenched by adding saturated aqueous NH_4Cl solution (30 mL) followed by extraction with CH_2Cl_2 (3 x 30 mL). The combined organic layers were dried over $MgSO_4$, filtered, and concentrated. Purification by column chromatography (hexanes/EtOAc from 1:1 to 1:2, then EtOAc/MeOH 100:1) afforded 3.71 g (70% over 2 steps) of ethyl *P*-ethyl-*N*-(quinolin-8-yl)phosphonamidate as an orange oil.

Synthesis of halogenated amides 1ah and 1ai.[18]

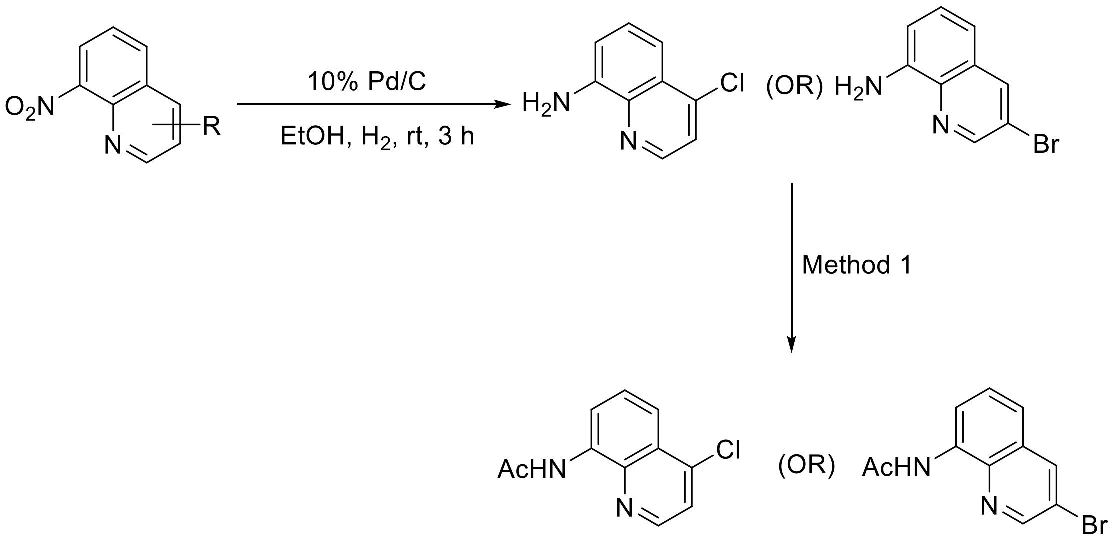

A mixture of 4-chloro-8-nitroquinoline or 3-bromo-8-nitroquinoline (0.2 mmol) and 10% Pd/C (30 mg) in EtOH (10 mL) was stirred under a hydrogen atmosphere (30 psi) at room temperature for 3 h. Upon completion, the reaction mixture was filtered through Celite. The filtrate was evaporated under reduced pressure, and the resulting residue was purified by column chromatography to afford the corresponding amine in good yield (4-chloroquinolin-8-amine, 94%; 3-bromoquinolin-8-amine, 81%).

The halogenated amine was acetylated using method A to afford the corresponding amides, **1ah** and **1ai** in 91% and 89% yield, respectively.

Synthesis of quinoline sulfonamide derivatives (1aj-al):[7]

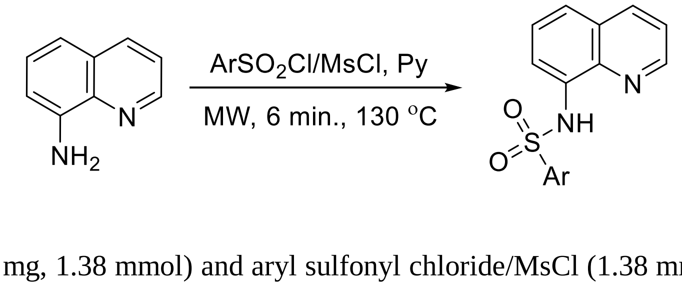

Quinolin-8-amine (200 mg, 1.38 mmol) and aryl sulfonyl chloride/MsCl (1.38 mmol) were dissolved in 5 mL of pyridine. The reaction mixture was irradiated in a microwave at 130 °C for 6 min. The reaction was quenched with 20 mL of water and the resulting precipitate was filtered and washed with cold water (10

mL) and dried under vacuum to give the sulfonamide derivative in moderate to good yield. If no precipitation was observed after addition of water, the resulting mixture was then extracted with CH_2Cl_2 (2 x 15 mL). The organic layer was dried over $MgSO_4$, filtered, and the crude compound was purified by column chromatography (EtOAc/hexanes, 50:50) to afford the product as a brown powder/solid.

Synthesis of *N*-benzyl- and *N,N*-dibenzyl quinolin-8-amines (**1am** and **1an**):[5b, 8]

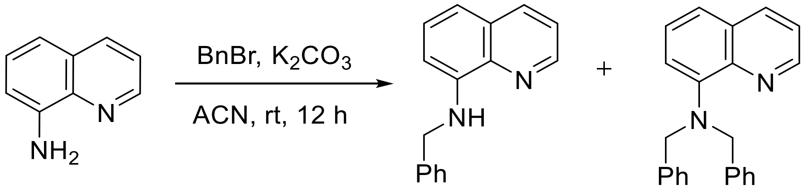

Method (i): To a mixture of 8-aminoquinoline (200 mg, 1.38 mmol), K_2CO_3 (192 mg, 1.38 mmol) in anhydrous ACN (10 mL) at room temperature, benzyl bromide (237 mg, 165 μL, 1.38 mmol) was added. The solution was stirred for 12 h, water was added to reaction mixture (20 mL), extracted with CH_2Cl_2 (2 x 20 mL), dried over $MgSO_4$, filtered and evaporated *in vacuo*. The resulting brown oil was purified by chromatographic column (EtOAc/hexanes 20:80) to give the mono-benzylated product **1am** as a yellow oil (76%) and the di-benzylated quinoline **1an** as a brown solid (8%).

Method (ii): To a mixture of 8-aminoquinoline (200 mg, 1.38 mmol), K_2CO_3 (383 mg, 2.77 mmol) in anhydrous ACN (12 mL) at room temperature, benzyl bromide (475 mg, 330 μL, 2.77 mmol) was added. The solution was stirred for 20 h, water was added to reaction mixture (20 mL), extracted with CH_2Cl_2 (2 x 20 mL), dried over $MgSO_4$, filtered and evaporated *in vacuo*. The resulting brown oil was purified by chromatographic column (EtOAc/hexanes 20:80) to give the di-benzylated quinoline **1an** as a brown solid (92%).

2-Methyl-2-phenyl-*N*-(quinolin-8-yl)dodecanamide (1g):

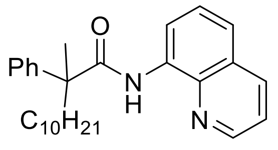

Brown solid, mp = 91-93 °C; ^{1}H NMR (400 MHz, $CDCl_3$): δ 9.87 (s, 1H), 8.77 (dd, J = 7.6, 1.3 Hz, 1H), 8.59 (dd, J = 4.2, 1.7 Hz, 1H), 8.05 (dd, J = 8.3, 1.7 Hz, 1H), 7.50 (ddd, J = 7.7, 4.4, 2.4 Hz, 3H), 7.45-7.24 (m, 5H), 2.34-2.06 (m, 2H), 1.75 (s, 3H), 1.46-1.13 (m, 16H), 0.86 (t, J = 7.0 Hz, 3H); $^{13}C\{^{1}H\}$NMR (100 MHz, $CDCl_3$): δ 175.4, 148.0, 144.2, 138.6, 136.0, 134.6, 128.6, 127.8, 127.3, 126.8, 126.7, 121.4, 121.1, 115.9, 51.9, 39.0, 31.8, 30.2, 29.6, 29.5, 29.4, 29.3, 24.5, 23.6, 22.6, 14.1; FTIR (neat): 3303, 3009, 2892, 1695, 1572, 1448, 1303, 1221, 1013, 932, 814, 712 cm^{-1}; MS (ESI): *m/z* 417 $(M+H)^+$; HRMS (ESI): *m/z* calcd for $C_{28}H_{37}N_2O$ $(M+H)^+$: 417.2900, found: 417.2912.

***tert*-Butyl benzoyl(quinolin-8-yl)carbamate (1y):**

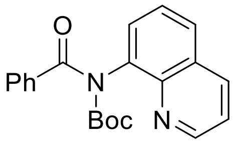

Pale yellow solid, mp = 114-116 °C; ^{1}H NMR (400 MHz, $CDCl_3$): δ 8.88 (dd, J = 4.2, 1.7 Hz, 1H), 8.17 (dd, J = 8.3, 1.7 Hz, 1H), 7.97 -7.91 (m, 2H), 7.83 (dd, J = 8.2, 1.4 Hz, 1H), 7.69 (dd, J = 7.4, 1.4 Hz, 1H), 7.62-7.55 (m, 1H), 7.55-7.49 (m, 1H), 7.49-7.36 (m, 3H), 1.20 (s, 9H); $^{13}C\{^{1}H\}$NMR (100 MHz, $CDCl_3$): δ 173.4, 153.4, 150.4, 144.0, 137.4, 137.2, 136.0, 131.3, 129.1, 129.0, 128.5, 128.2, 127.9, 126.2, 121.6, 83.1, 27.4; FTIR (neat): 3296, 2932, 1732, 1635, 1516, 1395, 1272, 1101, 936, 889, 703 cm^{-1}; MS (ESI): *m/z* 349 $(M+H)^+$; HRMS (ESI): *m/z* calcd for $C_{21}H_{21}N_2O_3$ $(M+H)^+$: 349.1547, found: 349.1542.

***tert*-Butyl acetyl(quinolin-8-yl)carbamate (1z):**

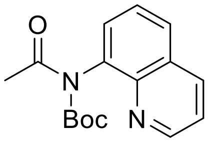

Pale yellow solid, mp = 103-105 °C; ^{1}H NMR (400 MHz, $CDCl_3$): δ 8.90 (dd, J = 4.2, 1.7 Hz, 1H), 8.18 (dd, J = 8.3, 1.3 Hz, 1H), 7.82 (dt, J = 11.6, 5.8 Hz, 1H), 7.60 -7.48 (m, 2H), 7.42 (dd, J = 8.3, 4.2 Hz, 1H), 2.74 (s, 3H), 1.24 (s, 9H); $^{13}C\{^{1}H\}$NMR (100 MHz, $CDCl_3$): δ 173.5, 152.9, 150.4, 144.1, 136.7, 135.9, 128.9, 128.9, 128.1, 126.0, 121.4, 82.6, 27.6, 26.4; FTIR (neat): 3256, 3010, 1738, 1672, 1556, 1465, 1237, 1112, 1036, 872, 756, 699 cm^{-1}; MS (ESI): *m/z* 287 $(M+H)^+$; HRMS (ESI): *m/z* calcd for $C_{16}H_{19}N_2O_3$ $(M+H)^+$: 287.1390, found: 287.1385.

1-(4-Chlorophenyl)-3-(quinolin-8-yl)urea (1ac):

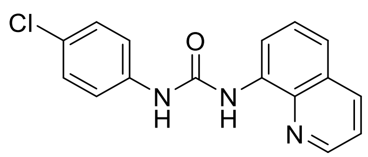

Brown solid, mp = 195-196 °C; ^{1}H NMR (400 MHz, $CDCl_3$: DMSO-*d6*, 8:2): δ 9.61 (s, 1H), 9.43 (s, 1H), 8.80 (dd, J = 4.2, 1.6 Hz, 1H), 8.65 (dd, J = 7.8, 1.1 Hz, 1H), 8.16 (dd, J = 8.3, 1.6 Hz, 1H), 7.59-7.35 (m, 5H), 7.28-7.20 (m, 2H); $^{13}C\{^{1}H\}$NMR (100 MHz, $CDCl_3$: DMSO-*d6*, 8:2): δ 152.8, 147.2, 138.3, 138.2, 136.1, 135.8, 128.4, 127.9, 127.2, 126.4, 121.1, 119.6, 119.5, 114.9; FTIR (neat): 3296, 3013, 2875, 1610, 1526, 1492, 1336, 1293, 1174, 936, 796, 664 cm^{-1}; MS (ESI): *m/z* 398 $(M+H)^+$; HRMS (ESI): *m/z* calcd for $C_{16}H_{13}ClN_3O$ $(M+H)^+$: 298.0742, found: 298.0750.

***N*-(5-Methoxyquinolin-8-yl)acetamide (1ae):**

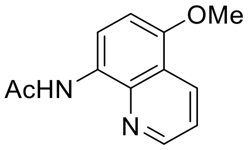

Pale yellow solid, mp = 97-98 °C; ^{1}H NMR (400 MHz, $CDCl_3$): δ 9.53 (s, 1H), 8.80 (dd, J = 4.2, 1.7 Hz, 1H), 8.67 (d, J = 8.5 Hz, 1H), 8.57 (dd, J = 8.4, 1.7 Hz, 1H), 7.43 (dd, J = 8.4, 4.2 Hz, 1H), 6.82 (d, J = 8.6 Hz, 1H), 3.98 (s, 3H), 2.33 (s, J = 3.1 Hz, 3H); $^{13}C\{^{1}H\}$NMR (100 MHz, $CDCl_3$): δ 168.3, 150.1, 148.5, 138.9, 131.2, 127.9, 120.6, 120.3, 116.5, 104.2, 55.7, 25.0; FTIR (neat): 3313, 3056, 2915, 1662, 1587, 1499, 1389, 1292, 1113, 997, 858, 735cm^{-1}; MS (ESI): *m/z* 217 $(M+H)^+$; HRMS (ESI): *m/z* calcd for $C_{12}H_{13}N_2O_2$ $(M+H)^+$: 217.0972, found: 217.0976.

Preparation of Tribromoisocyanuric Acid (TBCA):[19]

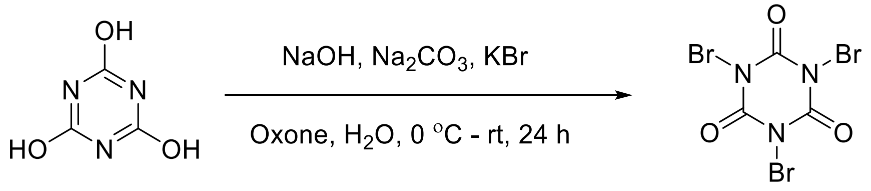

11

To a 500 mL round bottom flask equipped with a magnetic stir bar was added cyanuric acid (1.61 g, 12.5 mmol) and H_2O (180 mL), followed by NaOH (1.50 g, 37.5 mmol), Na_2CO_3 (1.99 g, 18.75 mmol), and KBr (4.46 g, 37.5 mmol). The mixture was stirred at room temperature for 10 min and cooled to 0 °C. A solution of Oxone (23.1 g, 37.5 mmol) in H_2O (150 mL) was added dropwise. During the addition of the oxidant

solution, a white solid precipitate appeared and formed a dense suspension, which was stirred for 24 h at room temperature. The product was isolated by vacuum filtration, washed with cold H_2O (4 x 20 mL), and then dried under vacuum in a desiccator over P_2O_5 to give the product as a white powder (81% yield), which is stored as 0 ^oC.

Preparation of Triiodoisocyanuric Acid (TICA):[20]

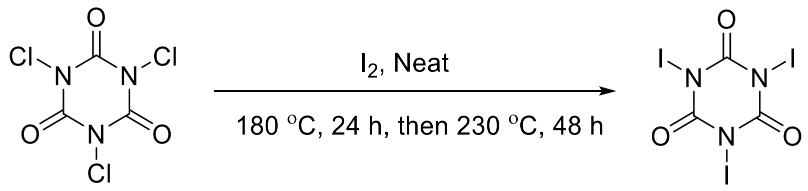

Trichloroisocyanuric acid (13.00 g, 55.93 mmol) and iodine (46.85 g, 184.6 mmol) were added to a 100 mL sealed tube and heated in a sand bath at 180 ^oC for 24 h. The dark red liquid and vapors of ICl were removed under reduced pressure for 1 h, and the sealed tube was heated again to 230 ^oC for 48 h. Evaporation of ICl under reduced pressure and heating (80 ^oC) for 2 h gave triiodoisocyanuric acid as a half white solid in 90% yield. At room temperature TICA decomposes slowly with the formation of I_2. On the other hand, in the presence of light the decomposition is very fast. However, if stored in the dark in a freezer, TICA proved to be quite stable.

3) Synthesis of halogenated quinolines:

General experimental procedure for halogenated quinolines with TXCA (2a-z, 3a-z, 4a-i, 5a-i, 6a-h, 7a-g, 8a-h, 9a-g and 10a-n):

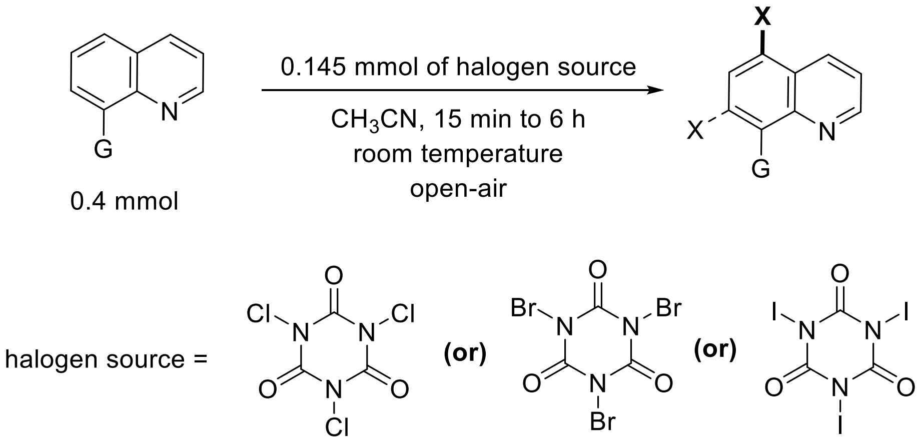

A 10 mL round bottom flask equipped with a magnetic stir bar was charged with 8-substituted quinoline (0.40 mmol, **1a-z, 1aa-ar**), and acetonitrile (3 mL) under open air conditions at room temperature. To the stirred solution was added the halogen source TCCA/TBCA/TICA (0.145 mmol) in one portion, and the resulting solution was stirred at room temperature for 15 minutes to 6 h. During the course of the reaction a color change occurred and cyanuric acid precipitated. The reaction mixture was then evaporated, and the crude residue was purified by flash chromatography (EtOAc/hexanes, 05:95 to 60:40) to yield the corresponding halogenated quinoline derivative **2a-z, 3a-z, 4a-i, 5a-i, 6a-h, 7a-g, 8a-h, 9a-g and 10a-n**.

General experimental procedure for halogenated quinolines with DCDMH/DBDMH:

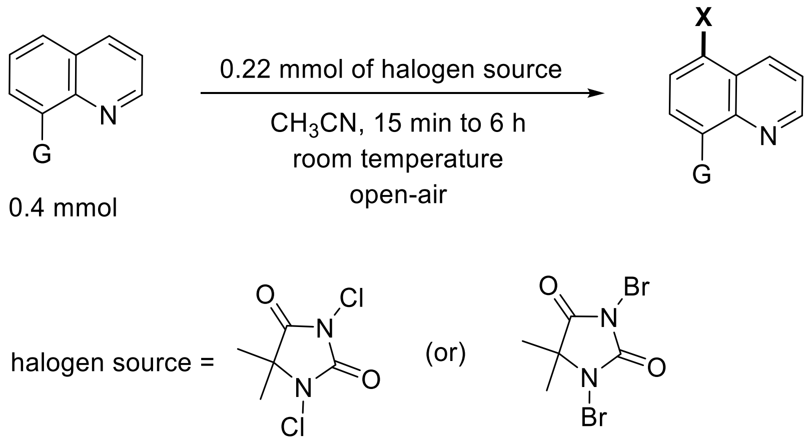

A 10 mL round bottom flask equipped with a magnetic stir bar was charged with 8-substituted quinoline (0.40 mmol, **1**), and acetonitrile (4 mL) under open air conditions at room temperature. To the stirred solution was added the halogen source 1,3-dichloro-5,5-dimethylhydantoin (DCDMH, 0.22 mmol, 0.55 equiv.)/1,3-dibromo-5,5-dimethylhydantoin (DBDMH, 0.22 mmol, 0.55 equiv.) in one portion, and the resulting solution was stirred at room temperature for 15 minutes to 6 h. The reaction mixture was then evaporated, and the crude residue was purified by flash chromatography (EtOAc/hexanes, 5:95 to 60:40) to yield the corresponding halogenated quinoline derivative **2a** (89%), **3a** (95%), **2c** (93%), **3c** (95%), **2e** (94%), **3e** (95%), **2h** (92%), **3h** (94%), **3n** (95%), **3t** (90%), **3v** (94%), **3w** (96%), **5b** (95%).

Gram-scale procedure for halogenated quinolines (2a, 3a and 10a) with TXCA:

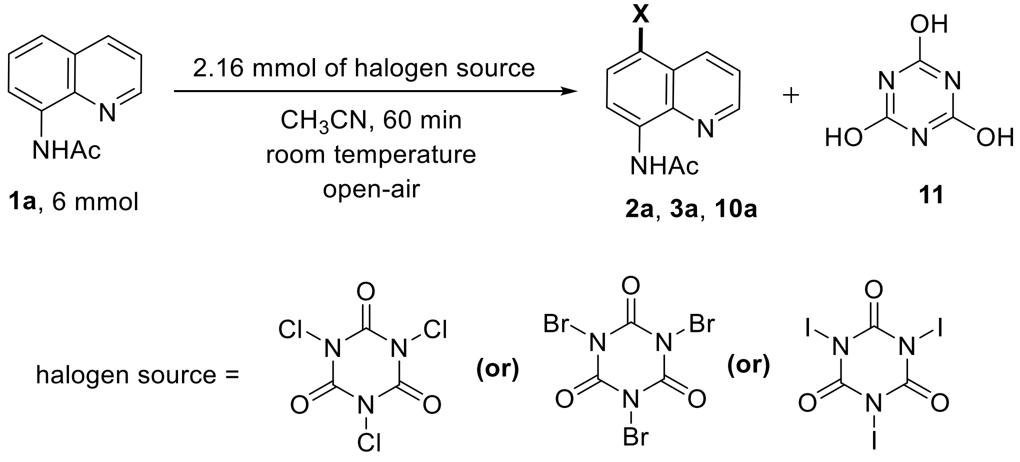

A 100 mL round bottom flask equipped with a magnetic stir bar was charged with *N*-(quinolin-8-yl)acetamide (**1a,** 1.12 g, 6.00 mmol), and acetonitrile (40 mL) under open air conditions at room temperature. To the stirred solution was added TCCA (501 mg, 2.16 mmol)/TBCA (790 mg, 2.16 mmol)/TICA (1.09 g, 2.16 mmol) in one portion, and the resulting solution was stirred at room temperature for 60 minutes. The reaction mixture was then filtered, and the residue was transferred to a round bottom flask, 30 mL of EtOAc was added, stirred for 10 min and filtered. This was repeated twice more (2 x 30 mL). The solid residue was washed with EtOAc (2 x 15 mL). The combined organic layers were evaporated and the crude residue was purified by flash chromatography (EtOAc/hexanes, 10:90 to 60:40) to yield the corresponding halogenated quinoline derivatives (**1a**, **2a** and **10a**) in 90-92% yield. The solid residue was dried under vacuum for 15 h to give compound **11** in approximately 90% yield.

***N*-(5-Chloroquinolin-8-yl)acetamide (2a):**[21]

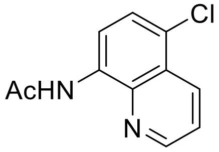

N-(Quinolin-8-yl)acetamide (**1a**, 75 mg, 0.40 mmol) and trichloroisocyanuric acid (34 mg, 0.145 mmol) in acetonitrile (3 mL), 20 min at room temperature. After column chromatography (gradient: EtOAc/hexanes 10:90 to 40:60) 86 mg (98%) of a white solid was obtained. R_f = 0.20 (EtOAc/hexanes 30:70); ^{1}H NMR (400 MHz, $CDCl_3$): δ 9.74 (s, 1H), 8.83 (dd, *J* = 4.2, 1.6 Hz, 1H), 8.70 (d, *J* = 8.4 Hz, 1H), 8.55 (dd, *J* = 8.5, 1.6 Hz, 1H), 7.61-7.52 (m, 2H), 2.35 (s, 3H); ^{13}C{^{1}H}NMR (100 MHz, $CDCl_3$): δ 168.8, 148.5, 138.7, 133.7, 133.4, 127.2, 125.8, 124.2, 122.3, 116.3, 25.1; MS (ESI): *m/z* 221 $(M+H)^+$.

***N*-(5-Bromoquinolin-8-yl)acetamide (3a):**[21]

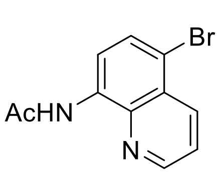

N-(Quinolin-8-yl)acetamide (**1a**, 75 mg, 0.40 mmol) and tribromoisocyanuric acid (53 mg, 0.145 mmol) in acetonitrile (3 mL), 30 min at room temperature. After column chromatography (gradient: EtOAc/hexanes 10:90 to 40:60) 101 mg (96%) of a white solid was obtained. R_f = 0.22 (EtOAc/hexanes 30:70); ^{1}H NMR (400 MHz, $CDCl_3$): δ 9.75 (s, 1H), 8.80 (dd, *J* = 4.2, 1.5 Hz, 1H), 8.64 (d, *J* = 8.4 Hz, 1H), 8.50 (dd, *J* = 8.5, 1.5 Hz, 1H), 7.77 (d, *J* = 8.4 Hz, 1H), 7.55 (dd, *J* = 8.5, 4.2 Hz, 1H), 2.35 (s, 3H); ^{13}C{^{1}H}NMR (100 MHz, $CDCl_3$): δ 168.8, 148.5, 138.8, 136.0, 134.3, 130.8, 127.1, 122.6, 116.9, 114.1, 25.1; MS (ESI): *m/z* 265 $(M+H)^+$.

N-(5-Chloroquinolin-8-yl)propionamide (2b):[15]

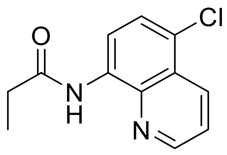

N-(Quinolin-8-yl)propionamide (**1b**, 80 mg, 0.40 mmol) and trichloroisocyanuric acid (34 mg, 0.145 mmol) in acetonitrile (3 mL), 60 min at room temperature. After column chromatography (gradient: EtOAc/hexanes 10:90 to 40:60) 90 mg (96%) of a white solid was obtained. R_f = 0.30 (EtOAc/hexanes 30:70); ^{1}H NMR (400 MHz, $CDCl_3$): δ 9.76 (s, 1H), 8.82 (dd, *J* = 4.2, 1.6 Hz, 1H), 8.72 (d, *J* = 8.4 Hz, 1H), 8.54 (dd, *J* = 8.5, 1.6 Hz, 1H), 7.60-7.49 (m, 2H), 2.60 (q, *J* = 7.6 Hz, 2H), 1.34 (t, *J* = 7.6 Hz, 3H); ^{13}C{^{1}H}NMR (100 MHz, $CDCl_3$): δ 172.4, 148.5, 138.8, 133.7, 133.3, 127.2, 125.8, 123.9, 122.2, 116.2, 31.2, 9.6; FTIR (neat): 3340, 3100, 2991, 1692, 1504, 1482, 1286, 1141, 954, 879, 756, 697 cm^{-1}; MS (ESI): *m/z* 235 $(M+H)^+$.

N-(5-Bromoquinolin-8-yl)propionamide (3b):[15]

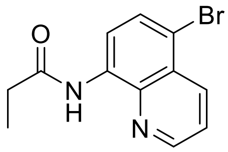

N-(Quinolin-8-yl)propionamide (**1b**, 80 mg, 0.40 mmol) and tribromoisocyanuric acid (53 mg, 0.145 mmol) in acetonitrile (3 mL), 120 min at room temperature. After column chromatography (gradient: EtOAc/hexanes 10:90 to 40:60) 102 mg (92%) of a white solid was obtained. R_f = 0.32 (EtOAc/hexanes 30:70); ^{1}H NMR (400 MHz, $CDCl_3$): δ 9.78 (s, 1H), 8.80 (dd, *J* = 4.2, 1.6 Hz, 1H), 8.67 (d, *J* = 8.4 Hz, 1H), 8.50 (dd, *J* = 8.5, 1.6 Hz, 1H), 7.77 (d, *J* = 8.4 Hz, 1H), 7.55 (dd, *J* = 8.5, 4.2 Hz, 1H), 2.60 (q, *J* = 7.6 Hz, 2H), 1.34 (t, *J* = 7.6 Hz, 3H); ^{13}C{^{1}H}NMR (100 MHz, $CDCl_3$): δ 172.4, 148.5, 138.9, 135.9, 134.4, 130.9, 127.1, 122.6, 116.8, 113.9, 31.2, 9.6; MS (ESI): *m/z* 279 $(M+H)^+$.

N-(5-Chloroquinolin-8-yl)pivalamide (2c):[21]

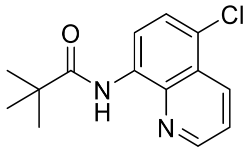

N-(Quinolin-8-yl)pivalamide (**1c**, 91 mg, 0.40 mmol) and trichloroisocyanuric acid (34 mg, 0.145 mmol) in acetonitrile (3 mL), 15 min at room temperature. After column chromatography (gradient: EtOAc/hexanes 10:90 to 30:70) 103 mg (99%) of a white solid was obtained. R_f = 0.30 (EtOAc/hexanes 20:80); ^{1}H NMR (400 MHz, $CDCl_3$): δ 10.21 (s, 1H), 8.85 (dd, *J* = 4.2, 1.6 Hz, 1H), 8.74 (d, *J* = 8.4 Hz, 1H), 8.54 (dd, *J* = 8.5, 1.6 Hz, 1H), 7.63-7.49 (m, 2H), 1.43 (s, 9H); ^{13}C{^{1}H}NMR (100 MHz, $CDCl_3$): δ 177.2, 148.6, 139.2, 133.9, 133.3, 127.2, 125.8, 123.9, 122.2, 116.1, 40.3, 27.6; MS (ESI): *m/z* 263 $(M+H)^+$.

***N*-(5-Bromoquinolin-8-yl)pivalamide (3c):**[21]

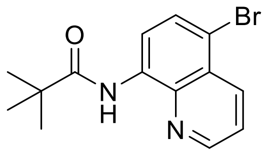

N-(Quinolin-8-yl)pivalamide (**1c**, 91 mg, 0.40 mmol) and tribromoisocyanuric acid (53 mg, 0.145 mmol) in acetonitrile (3 mL), 20 min at room temperature. After column chromatography (gradient: EtOAc/hexanes 10:90 to 30:70) 120 mg (98%) of a white solid was obtained. R_f = 0.31 (EtOAc/hexanes 20:80); ^{1}H NMR (400 MHz, $CDCl_3$): δ 10.21 (s, 1H), 8.78 (dd, J = 4.2, 1.6 Hz, 1H), 8.67 (d, J = 8.4 Hz, 1H), 8.43 (dd, J = 8.5, 1.6 Hz, 1H), 7.74 (d, J = 8.4 Hz, 1H), 7.49 (dd, J = 8.5, 4.2 Hz, 1H), 1.43 (s, 9H); ^{13}C{^{1}H}NMR (100 MHz, $CDCl_3$): δ 177.1, 148.5, 139.2, 135.7, 134.4, 130.7, 126.9, 122.4, 116.5, 113.7, 40.2, 27.6; MS (ESI): *m/z* 307 $(M+H)^+$.

***N*-(5-Chloroquinolin-8-yl)-3-phenylpropanamide (2d):**[11]

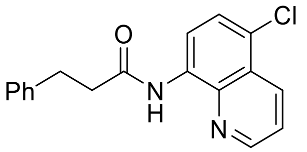

3-Phenyl-*N*-(quinolin-8-yl)propanamide (**1d**, 110 mg, 0.40 mmol) and trichloroisocyanuric acid (34 mg, 0.145 mmol) in acetonitrile (3 mL), 60 min at room temperature. After column chromatography (gradient: EtOAc/hexanes 10:90 to 40:60) 113 mg (91%) of a white solid was obtained. R_f = 0.20 (EtOAc/hexanes 20:80); ^{1}H NMR (400 MHz, $CDCl_3$): δ 9.75 (s, 1H), 8.77 (dd, J = 4.2, 1.6 Hz, 1H), 8.68 (d, J = 8.4 Hz, 1H), 8.51 (dd, J = 8.5, 1.6 Hz, 1H), 7.78 (d, J = 8.4 Hz, 1H), 7.54 (dd, J = 8.5, 4.2 Hz, 1H), 7.31-7.27 (m, 4H), 7.24-7.17 (m, 1H), 3.14 (t, J = 7.4 Hz, 2H), 2.88 (t, J = 7.4 Hz, 2H); ^{13}C{^{1}H}NMR (100 MHz, $CDCl_3$): δ 170.7, 148.5, 140.6, 138.9, 135.9, 134.3, 130.9, 128.5, 128.4, 127.1, 126.3, 122.6, 116.9, 114.1, 39.7, 31.3; MS (ESI): *m/z* 311 $(M+H)^+$.

***N*-(5-Bromoquinolin-8-yl)-3-phenylpropanamide (3d):**[11]

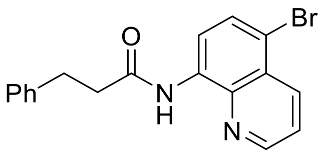

3-Phenyl-*N*-(quinolin-8-yl)propanamide (**1d**, 110 mg, 0.40 mmol) and tribromoisocyanuric acid (53 mg, 0.145 mmol) in acetonitrile (3 mL), 2 h at room temperature. After column chromatography (gradient: EtOAc/hexanes 10:90 to 30:70) 132 mg (93%) of a white solid was obtained. R_f = 0.21 (EtOAc/hexanes 20:80); ^{1}H NMR (400 MHz, $CDCl_3$): δ 9.72 (s, 1H), 8.79 (dd, J = 4.2, 1.6 Hz, 1H), 8.72 (d, J = 8.4 Hz, 1H), 8.54 (dd, J = 8.5, 1.6 Hz, 1H), 7.58 (d, J = 8.4 Hz, 1H), 7.54 (dd, J = 8.5, 4.2 Hz, 1H), 7.32-7.28 (m, 4H), 7.24-7.17 (m, 1H), 3.14 (t, J = 7.4 Hz, 2H), 2.87 (t, J = 7.4 Hz, 2H); ^{13}C{^{1}H}NMR (100 MHz, $CDCl_3$): δ

170.7, 148.5, 140.6, 138.7, 133.6, 133.3, 128.5, 128.4, 127.2, 126.2, 125.8, 124.1, 122.3, 116.3, 39.7, 31.3; MS (ESI): *m/z* 355 $(M+H)^+$.

***N*-(5-Chloroquinolin-8-yl)cyclopentanecarboxamide (2e):**

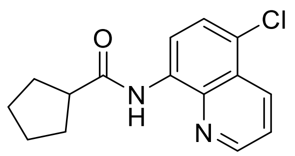

N-(Quinolin-8-yl)cyclopentanecarboxamide (**1e**, 96 mg, 0.40 mmol) and trichloroisocyanuric acid (34 mg, 0.145 mmol) in acetonitrile (3 mL), 90 min at room temperature. After column chromatography (gradient: EtOAc/hexanes 10:90 to 50:50) 107 mg (98%) of a white solid was obtained. R_f = 0.20 (EtOAc/hexanes 20:80), mp = 100-101 °C; ^{1}H NMR (400 MHz, $CDCl_3$): δ 9.81 (s, 1H), 8.82 (dd, *J* = 4.2, 1.6 Hz, 1H), 8.72 (d, *J* = 8.4 Hz, 1H), 8.53 (dd, *J* = 8.5, 1.6 Hz, 1H), 7.59-7.52 (m, 2H), 2.9-2.86 (m, 1H), 2.13-1.93 (m, 4H), 1.89-1.78 (m, 2H), 1.74-1.57 (m, 2H); ^{13}C{^{1}H}NMR (100 MHz, $CDCl_3$): δ 175.0, 148.5, 138.8, 133.9, 133.3, 127.2, 125.8, 123.8, 122.2, 116.1, 47.3, 30.5, 25.9; FTIR (neat): 3327, 2950, 2867, 1677, 1515, 1477, 1451, 1214, 1056, 929, 782, 704 cm^{-1}; MS (ESI): *m/z* 275 $(M+H)^+$; HRMS (ESI): *m/z* calcd for $C_{15}H_{16}ClN_2O$ $(M+H)^+$: 275.0946, found: 275.0949.

***N*-(5-Bromoquinolin-8-yl)cyclopentanecarboxamide (3e):**[12]

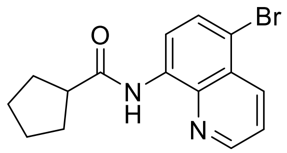

N-(Quinolin-8-yl)cyclopentanecarboxamide (**1e**, 96 mg, 0.40 mmol) and tribromoisocyanuric acid (53 mg, 0.145 mmol) in acetonitrile (3 mL), 2 h at room temperature. After column chromatography (gradient: EtOAc/hexanes 10:90 to 50:50) 122 mg (96%) of a white solid was obtained. R_f = 0.21 (EtOAc/hexanes 20:80); mp = 125-127 °C; ^{1}H NMR (400 MHz, $CDCl_3$) δ 9.81 (s, 1H), 8.78 (dd, *J* = 4.2, 1.6 Hz, 1H), 8.66 (d, *J* = 8.4 Hz, 1H), 8.46 (dd, *J* = 8.5, 1.6 Hz, 1H), 7.75 (d, *J* = 8.4 Hz, 1H), 7.51 (dd, *J* = 8.5, 4.2 Hz, 1H), 3.02-2.84 (m, 1H), 2.12-1.92 (m, 4H), 1.90-1.76 (m, 2H), 1.74-1.60 (m, 2H); ^{13}C{^{1}H}NMR (100 MHz, $CDCl_3$): δ 175.0, 148.4, 138.9, 135.8, 134.5, 130.8, 127.0, 122.5, 116.7, 113.7, 47.3, 30.5, 25.9; MS (ESI): *m/z* 319 $(M+H)^+$.

***N*-(5-Chloroquinolin-8-yl)-2,2-diphenylpropanamide (2f):**

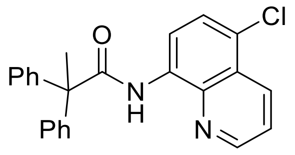

2,2-Diphenyl-*N*-(quinolin-8-yl)propanamide (**1f**, 141 mg, 0.40 mmol) and trichloroisocyanuric acid (34 mg, 0.145 mmol) in acetonitrile (3 mL), 30 min

at room temperature. After column chromatography (gradient: EtOAc/hexanes 10:90 to 50:50) 145 mg (94%) of a white solid was obtained. R_f = 0.25 (EtOAc/hexanes 20:80), mp = 172-174 °C; ^{1}H NMR (400 MHz, $CDCl_3$): δ 10.06 (s, 1H), 8.80 (d, *J* = 8.4 Hz, 1H), 8.54 (dd, *J* = 4.2, 1.6 Hz, 1H), 8.49 (dd, *J* = 8.5, 1.6 Hz, 1H), 7.59 (d, *J* = 8.4 Hz, 1H), 7.45 (dd, *J* = 8.5, 4.2 Hz, 1H), 7.38 (d, *J* = 4.5 Hz, 8H), 7.34-7.29 (m, 2H), 2.16 (s, 3H); ^{13}C{^{1}H}NMR (100 MHz, $CDCl_3$): δ 173.8, 148.5, 144.7, 139.3, 133.7, 133.1, 128.6, 128.3, 127.1, 127.1, 125.8, 124.4, 122.2, 115.9, 58.4, 27.1; FTIR (neat): 3323, 3017, 2896, 1731, 1570, 1496, 1416, 1205, 1041, 996, 918, 803, 699 cm^{-1}; MS (ESI): *m/z* 387 $(M+H)^+$; HRMS (ESI): *m/z* calcd for $C_{24}H_{20}ClN_2O$ $(M+H)^+$: 387.1259, found: 387.1260.

***N*-(5-Bromoquinolin-8-yl)-2,2-diphenylpropanamide (3f):**

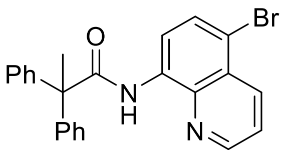

2,2-Diphenyl-*N*-(quinolin-8-yl)propanamide (**1f**, 141 mg, 0.40 mmol) and tribromoisocyanuric acid (53 mg, 0.145 mmol) in acetonitrile (3 mL), 30 min at room temperature. After column chromatography (gradient: EtOAc/hexanes 10:90 to 50:50) 163 mg (95%) of a white solid was obtained. R_f = 0.25 (EtOAc/hexanes 20:80), mp = 152-154 °C; ^{1}H NMR (400 MHz, $CDCl_3$): δ 10.08 (s, 1H), 8.75 (d, *J* = 8.4 Hz, 1H), 8.50 (dd, *J* = 4.2, 1.5 Hz, 1H), 8.42 (dd, *J* = 8.5, 1.5 Hz, 1H), 7.77 (d, *J* = 8.4 Hz, 1H), 7.42 (dd, *J* = 8.5, 4.2 Hz, 1H), 7.40-7.36 (m, 8H), 7.36-7.28 (m, 2H), 2.16 (s, 3H); ^{13}C{^{1}H}NMR (100 MHz, $CDCl_3$): δ 173.8, 148.5, 144.7, 139.4, 135.6, 134.3, 130.8, 128.6, 128.2, 127.0, 127.0, 122.5, 116.4, 114.3, 58.4, 27.0; FTIR (neat): 3305, 3060, 2939, 1666, 1516, 1472, 1316, 1246, 1041, 1001, 934, 843, 754, 692 cm^{-1}; MS (ESI): *m/z* 431 $(M+H)^+$; HRMS (ESI): *m/z* calcd for $C_{24}H_{20}BrN_2O$ $(M+H)^+$: 431.0754, found: 431.0756.

***N*-(5-Chloroquinolin-8-yl)-2-methyl-2-phenyldodecanamide (2g):**

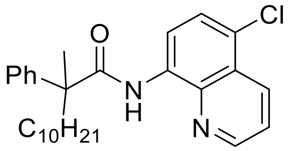

2-Methyl-2-phenyl-*N*-(quinolin-8-yl)dodecanamide (**1g**, 166 mg, 0.40 mmol) and trichloroisocyanuric acid (34 mg, 0.145 mmol) in acetonitrile (3 mL), 60 min at room temperature. After column chromatography (gradient: EtOAc/hexanes 10:90 to 50:50) 176 mg (98%) of a white solid was obtained. R_f = 0.30 (EtOAc/hexanes 20:80), mp = 144-146 °C; ^{1}H NMR (400 MHz, $CDCl_3$): δ 9.78 (s, 1H), 8.71 (d, *J* = 8.4 Hz, 1H), 8.61 (dd,

J = 4.2, 1.5 Hz, 1H), 8.44 (dd, J = 8.5, 1.5 Hz, 1H), 7.55 (d, J = 8.4 Hz, 1H), 7.49 (d, J = 7.4 Hz, 2H), 7.45-7.35 (m, 3H), 7.31-7.23 (m, 1H), 2.31-2.00 (m, 2H), 1.74 (s, 3H), 1.44-1.11 (m, 16H), 0.86 (t, J = 6.9 Hz, 3H); $^{13}C\{^1H\}$NMR (100 MHz, $CDCl_3$): δ 175.5, 148.5, 143.9, 139.1, 133.9, 133.0, 128.7, 127.1, 126.9, 126.6, 125.7, 123.8, 122.0, 115.8, 51.9, 39.0, 31.8, 30.1, 29.6, 29.5, 29.4, 29.3, 24.4, 23.5, 22.6, 14.1; FTIR (neat): 3358, 2920, 2851, 1680, 1520, 1468, 1366, 1134, 1076, 945, 834, 724, 699 cm^{-1}; MS (ESI): *m/z* 451 $(M+H)^+$; HRMS (ESI): *m/z* calcd for $C_{28}H_{36}ClN_2O$ $(M+H)^+$: 451.2511, found: 451.2514.

***N*-(5-Bromoquinolin-8-yl)-2-methyl-2-phenyldodecanamide (3g):**

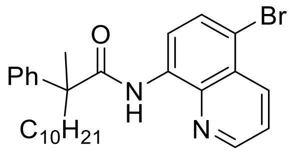

2-Methyl-2-phenyl-*N*-(quinolin-8-yl)dodecanamide (**1g**, 166 mg, 0.40 mmol) and tribromoisocyanuric acid (53 mg, 0.145 mmol) in acetonitrile (3 mL), 2 h at room temperature. After column chromatography (gradient: EtOAc/hexanes 10:90 to 50:50) 184 mg (93%) of a white solid was obtained. R_f = 0.30 (EtOAc/hexanes 20:80), mp = 191-193 °C; ^1H NMR (400 MHz, $CDCl_3$): δ 9.80 (s, 1H), 8.66 (d, J = 8.4 Hz, 1H), 8.58 (dd, J = 4.2, 1.6 Hz, 1H), 8.39 (dd, J = 8.5, 1.6 Hz, 1H), 7.74 (d, J = 8.4 Hz, 1H), 7.52-7.46 (m, 2H), 7.45-7.34 (m, 3H), 7.32-7.26 (m, 1H), 2.29-2.06 (m, 2H), 1.74 (s, 3H), 1.36-1.16 (m, 16H), 0.86 (t, J = 7.0 Hz, 3H); $^{13}C\{^1H\}$NMR (100 MHz, $CDCl_3$): δ 175.5, 148.5, 143.9, 139.2, 135.6, 134.5, 130.7, 128.7, 127.0, 126.9, 126.6, 122.4, 116.4, 113.8, 51.9, 38.9, 31.8, 30.1, 29.5, 29.5, 29.4, 29.2, 24.4, 23.5, 22.6, 14.1; FTIR (neat): 3321, 2861, 2836, 1617, 1505, 1471, 1392, 1182, 1001, 834, 732, 686 cm^{-1}; MS (ESI): *m/z* 495 $(M+H)^+$; HRMS (ESI): *m/z* calcd for $C_{28}H_{36}BrN_2O$ $(M+H)^+$: 495.2006, found: 495.2014.

2-Benzyl-*N*-(5-chloroquinolin-8-yl)-2-cyano-3-phenylpropanamide (2h):

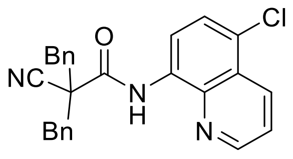

2-Benzyl-2-cyano-3-phenyl-*N*-(quinolin-8-yl)propanamide (**1h**, 156 mg, 0.40 mmol) and trichloroisocyanuric acid (34 mg, 0.145 mmol) in acetonitrile (3 mL), 2 h at room temperature. After column chromatography (gradient: EtOAc/hexanes 10:90 to 50:50) 164 mg (97%) of a white solid was obtained. R_f = 0.30 (EtOAc/hexanes 20:80), mp = 155-157 °C; ^1H NMR (400 MHz, $CDCl_3$): δ 10.24 (s, 1H), 8.67-8.56 (m, 2H), 8.36 (dd, J = 8.5, 1.6 Hz, 1H), 7.53 (d, J = 8.4 Hz, 1H), 7.40-7.32 (m, 5H), 7.24-7.19 (m, 4H), 7.17-7.11 (m, 2H), 3.54

(d, J = 13.4 Hz, 2H), 3.15 (d, J = 13.4 Hz, 2H); $^{13}C\{^1H\}$NMR (100 MHz, $CDCl_3$): δ 164.8, 149.0, 138.9, 134.3, 132.8, 132.3, 130.0, 128.5, 127.6, 126.6, 125.5, 125.4, 122.3, 119.6, 116.4, 54.5, 43.0; FTIR (neat): 3312, 3031, 2916, 1677, 1524, 1480, 1367, 1119, 1005, 844, 760, 694 cm^{-1}; MS (ESI): *m/z* 426 $(M+H)^+$; HRMS (ESI): *m/z* calcd for $C_{26}H_{21}ClN_3O$ $(M+H)^+$: 426.1368, found: 426.1371.

2-Benzyl-*N*-(5-bromoquinolin-8-yl)-2-cyano-3-phenylpropanamide (3h):

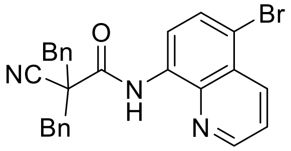

2-Benzyl-2-cyano-3-phenyl-*N*-(quinolin-8-yl)propanamide (**1h**, 156 mg, 0.40 mmol) and tribromoisocyanuric acid (53 mg, 0.145 mmol) in acetonitrile (3 mL), 2 h at room temperature. After column chromatography (gradient: EtOAc/hexanes 10:90 to 50:50) 180 mg (96%) of a white solid was obtained. R_f = 0.30 (EtOAc/hexanes 20:80), mp = 166-168 °C; ^1H NMR (400 MHz, $CDCl_3$): δ 10.27 (s, 1H), 8.61 (dd, J = 4.2, 1.5 Hz, 1H), 8.58 (d, J = 8.4 Hz, 1H), 8.35 (dd, J = 8.5, 1.5 Hz, 1H), 7.74 (d, J = 8.4 Hz, 1H), 7.39 (dd, J = 8.5, 4.2 Hz, 1H), 7.35 (dd, J = 5.1, 3.3 Hz, 4H), 7.25-7.18 (m, 4H), 7.18-7.11 (m, 2H), 3.54 (d, J = 13.4 Hz, 2H), 3.15 (d, J = 13.4 Hz, 2H); $^{13}C\{^1H\}$NMR (100 MHz, $CDCl_3$): δ 164.8, 149.0, 139.0, 135.4, 134.3, 133.0, 130.3, 130.0, 128.5, 127.7, 126.8, 122.7, 119.6, 116.9, 115.5, 54.5, 43.1; FTIR (neat): 3296, 3030, 2961, 1682, 1520, 1476, 1249, 1205, 953, 916, 766, 700 cm^{-1}; MS (ESI): *m/z* 470 $(M+H)^+$; HRMS (ESI): *m/z* calcd for $C_{26}H_{21}BrN_3O$ $(M+H)^+$: 470.0863, found: 470.0865.

***N*-(5-Chloroquinolin-8-yl)-2-cyano-2-methyl-3-phenylpropanamide (2i):**

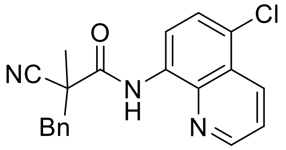

2-Cyano-2-methyl-3-phenyl-*N*-(quinolin-8-yl)propanamide (**1i**, 126 mg, 0.40 mmol) and trichloroisocyanuric acid (34 mg, 0.145 mmol) in acetonitrile (3 mL), 45 min at room temperature. After column chromatography (gradient: EtOAc/hexanes 10:90 to 50:50) 134 mg (96%) of a white solid was obtained. R_f = 0.30 (EtOAc/hexanes 20:80), mp = 147-149 °C; ^1H NMR (400 MHz, $CDCl_3$): δ 10.60 (s, 1H), 8.82 (dd, J = 4.2, 1.6 Hz, 1H), 8.65 (d, J = 8.4 Hz, 1H), 8.52 (dd, J = 8.5, 1.6 Hz, 1H), 7.60 (d, J = 8.4 Hz, 1H), 7.54 (dd, J = 8.5, 4.2 Hz, 1H), 7.38-7.30 (m, 2H), 7.29-7.11 (m, 3H), 3.42 (d, J = 13.5 Hz, 1H), 3.12 (d, J = 13.5 Hz, 1H), 1.78 (s, 3H); $^{13}C\{^1H\}$NMR (100 MHz, $CDCl_3$): δ 165.9, 149.2, 139.1, 134.4, 133.2, 132.6, 130.0, 128.5, 127.7, 126.8,

125.8, 125.5, 122.5, 120.8, 116.6, 46.9, 43.9, 23.7; FTIR (neat): 3291, 3003, 2933, 1682, 1529, 1480, 1253, 1032, 915, 829, 701, 692 cm^{-1}; MS (ESI): *m/z* 350 $(M+H)^+$; HRMS (ESI): *m/z* calcd for $C_{20}H_{17}ClN_3O$ $(M+H)^+$: 350.1055, found: 350.1058.

***N*-(5-Bromoquinolin-8-yl)-2-cyano-2-methyl-3-phenylpropanamide (3i):**

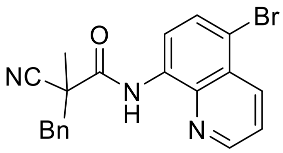

2-Cyano-2-methyl-3-phenyl-*N*-(quinolin-8-yl)propanamide (**1i**, 126 mg, 0.40 mmol) and tribromoisocyanuric acid (53 mg, 0.145 mmol) in acetonitrile (3 mL), 2 h at room temperature. After column chromatography (gradient: EtOAc/hexanes 10:90 to 50:50) 149 mg (95%) of a white solid was obtained. R_f = 0.30 (EtOAc/hexanes 20:80), mp = 145-146 °C; ^{1}H NMR (400 MHz, $CDCl_3$): δ 10.60 (s, 1H), 8.77 (dd, *J* = 4.2, 1.5 Hz, 1H), 8.58 (d, *J* = 8.4 Hz, 1H), 8.43 (dd, *J* = 8.5, 1.6 Hz, 1H), 7.77 (d, *J* = 8.4 Hz, 1H), 7.50 (dd, *J* = 8.5, 4.2 Hz, 1H), 7.38-7.29 (m, 2H), 7.29-7.12 (m, 3H), 3.42 (d, *J* = 13.5 Hz, 1H), 3.11 (d, *J* = 13.5 Hz, 1H), 1.78 (s, 3H); $^{13}C\{^1H\}$NMR (100 MHz, $CDCl_3$): δ 165.9, 149.1, 139.1, 135.6, 134.4, 133.2, 130.4, 129.9, 128.5, 127.7, 126.9, 122.8, 120.7, 117.0, 115.5, 46.9, 43.8, 23.6; FTIR (neat): 3289, 3003, 2933, 1682, 1528, 1476, 1453, 1151, 1048, 835, 827, 700 cm^{-1}; MS (ESI): *m/z* 394 $(M+H)^+$; HRMS (ESI): *m/z* calcd for $C_{20}H_{17}BrN_3O$ $(M+H)^+$: 394.0550, found: 394.0553.

***N*-(5-Chloroquinolin-8-yl)-2-cyano-2,5-dimethylhexanamide (2j):**

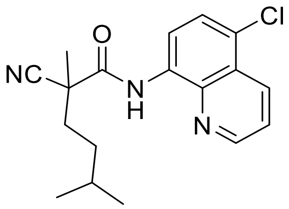

2-Cyano-2,5-dimethyl-*N*-(quinolin-8-yl)hexanamide (**1j**, 118 mg, 0.40 mmol) and trichloroisocyanuric acid (34 mg, 0.145 mmol) in acetonitrile (3 mL), 30 min at room temperature. After column chromatography (gradient: EtOAc/hexanes 10:90 to 50:50) 128 mg (97%) of a white solid was obtained. R_f = 0.30 (EtOAc/hexanes 20:80), mp = 61-62 °C; ^{1}H NMR (400 MHz, $CDCl_3$): δ 10.82 (s, 1H), 8.94 (dd, *J* = 4.2, 1.6 Hz, 1H), 8.67 (d, *J* = 8.4 Hz, 1H), 8.57 (dd, *J* = 8.5, 1.6 Hz, 1H), 7.60 (dd, *J* = 8.4, 3.8 Hz, 2H), 2.19-2.07 (m, 1H), 1.86 (td, *J* = 13.1, 4.3 Hz, 1H), 1.75 (s, 3H), 1.67-1.44 (m, 2H), 1.42-1.29 (m, 1H), 0.91 (dd, *J* = 6.5, 3.5 Hz, 6H); $^{13}C\{^1H\}$NMR (100 MHz, $CDCl_3$): δ 166.6, 149.3, 139.2, 133.3, 132.7, 126.9, 125.8, 125.5, 122.6, 121.3, 116.6, 45.5, 36.5, 34.3, 27.9, 24.1, 22.3, 22.2; FTIR (neat): 3320, 2955, 2870,

1691, 1524, 1482, 1322, 1104, 947, 839, 783, 660 cm^{-1}; MS (ESI): *m/z* 330 (M+H)$^+$; HRMS (ESI): *m/z* calcd for $C_{18}H_{21}ClN_3O$ (M+H)$^+$: 330.1395, found: 330.1397.

***N*-(5-Bromoquinolin-8-yl)-2-cyano-2,5-dimethylhexanamide (3j):**

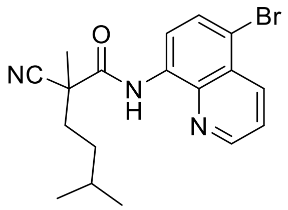

2-Cyano-2,5-dimethyl-*N*-(quinolin-8-yl)hexanamide (**1j**, 118 mg, 0.40 mmol) and tribromoisocyanuric acid (53 mg, 0.145 mmol) in acetonitrile (3 mL), 30 min at room temperature. After column chromatography (gradient: EtOAc/hexanes 10:90 to 50:50) 143 mg (96%) of a white solid was obtained. R_f = 0.31 (EtOAc/hexanes 20:80), mp = 91-92 °C; ^{1}H NMR (400 MHz, $CDCl_3$): δ 10.84 (s, 1H), 8.91 (dd, *J* = 4.2, 1.6 Hz, 1H), 8.62 (d, *J* = 8.4 Hz, 1H), 8.52 (dd, *J* = 8.5, 1.6 Hz, 1H), 7.80 (d, *J* = 8.4 Hz, 1H), 7.59 (dd, *J* = 8.5, 4.2 Hz, 1H), 2.18-2.08 (m, 1H), 1.86 (td, *J* = 13.1, 4.3 Hz, 1H), 1.75 (s, 3H), 1.67-1.46 (m, 2H), 1.40-1.29 (m, 1H), 0.91 (dd, *J* = 6.5, 3.5 Hz, 6H); ^{13}C{^{1}H}NMR (100 MHz, $CDCl_3$): δ 166.6, 149.3, 139.3, 135.8, 133.3, 130.5, 127.1, 122.9, 121.2, 117.1, 115.5, 45.5, 36.5, 34.2, 27.9, 24.0, 22.3, 22.2; FTIR (neat): 3313, 2954, 2870, 1691, 1523, 1478, 1361, 1172, 939, 838, 783, 674 cm^{-1}; MS (ESI): *m/z* 374 (M+H)$^+$; HRMS (ESI): *m/z* calcd for $C_{18}H_{21}BrN_3O$ (M+H)$^+$: 374.0863, found: 374.0865.

***N*-(5-Chloroquinolin-8-yl)-2-cyano-2-methylheptadecanamide (2k):**

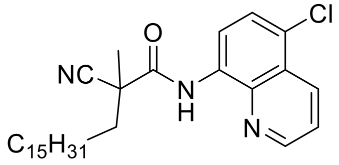

2-Cyano-2-methyl-*N*-(quinolin-8-yl)heptadecanamide (**1k**, 174 mg, 0.40 mmol) and trichloroisocyanuric acid (34 mg, 0.145 mmol) in acetonitrile (3 mL), 2 h at room temperature. After column chromatography (gradient: EtOAc/hexanes 10:90 to 50:50) 191 mg (99%) of a white solid was obtained. R_f = 0.30 (EtOAc/hexanes 20:80), mp = 74-75 °C; ^{1}H NMR (400 MHz, $CDCl_3$): δ 10.83 (s, 1H), 8.94 (dd, *J* = 4.2, 1.6 Hz, 1H), 8.67 (d, *J* = 8.4 Hz, 1H), 8.58 (dd, *J* = 8.5, 1.6 Hz, 1H), 7.67-7.50 (m, 2H), 2.17-2.07 (m, 1H), 1.89-1.80 (m, 1H), 1.74 (s, 3H), 1.70-1.55 (m, 1H), 1.52-1.40 (m, 1H), 1.39-1.18 (m, 26H), 0.87 (t, *J* = 6.9 Hz, 3H); ^{13}C{^{1}H}NMR (100 MHz, $CDCl_3$): δ 166.6, 149.3, 139.2, 133.3, 132.8, 126.9, 125.9, 125.5, 122.6, 121.3, 116.6, 45.5, 38.5, 31.9, 29.7, 29.7, 29.6, 29.6, 29.6, 29.5, 29.4, 29.4, 29.3, 29.2, 29.1, 25.6, 24.0, 22.7, 14.1;

FTIR (neat): 3297, 2915, 2847, 1712, 1693, 1525, 1464, 1247, 1084, 948, 787, 723, 684 cm^{-1}; MS (ESI): *m/z* 484 $(M+H)^+$; HRMS (ESI): *m/z* calcd for $C_{29}H_{43}ClN_3O$ $(M+H)^+$: 484.3089, found: 484.3090.

***N*-(5-Bromoquinolin-8-yl)-2-cyano-2-methylheptadecanamide (3k):**

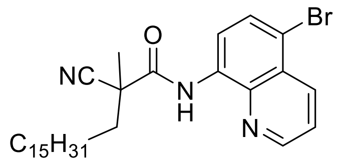

2-Cyano-2-methyl-*N*-(quinolin-8-yl)heptadecanamide (**1k**, 174 mg, 0.40 mmol) and tribromoisocyanuric acid (53 mg, 0.145 mmol) in acetonitrile (3 mL), 2 h at room temperature. After column chromatography (gradient: EtOAc/hexanes 10:90 to 50:50) 206 mg (98%) of a white solid was obtained. R_f = 0.31 (EtOAc/hexanes 20:80), mp = 191-193 °C; ^{1}H NMR (400 MHz, $CDCl_3$): δ 10.84 (s, 1H), 8.91 (dd, *J* = 4.2, 1.6 Hz, 1H), 8.62 (d, *J* = 8.4 Hz, 1H), 8.52 (dd, *J* = 8.5, 1.6 Hz, 1H), 7.80 (d, *J* = 8.4 Hz, 1H), 7.59 (dd, *J* = 8.5, 4.2 Hz, 1H), 2.17-2.07 (m, 1H), 1.90-1.78 (m, 1H), 1.74 (s, 3H), 1.72-1.54 (m, 1H), 1.51-1.40 (m, 1H), 1.39-1.18 (m, 26H), 0.87 (t, *J* = 6.9 Hz, 3H); $^{13}C\{^1H\}$NMR (100 MHz, $CDCl_3$): δ 166.6, 149.3, 139.3, 135.8, 133.4, 130.5, 127.1, 122.9, 121.3, 117.1, 115.5, 45.5, 38.5, 31.9, 29.7, 29.6, 29.6, 29.6, 29.6, 29.5, 29.5, 29.4, 29.3, 29.2 29.1, 25.6, 24.0, 22.6, 14.1; FTIR (neat): 3288, 2914, 2847, 1710, 1693, 1524, 1479, 1167, 929, 837, 723, 686 cm^{-1}; MS (ESI): *m/z* 528 $(M+H)^+$; HRMS (ESI): *m/z* calcd for $C_{29}H_{43}BrN_3O$ $(M+H)^+$: 528.2584, found: 528.2587.

***N*-(5-Chloroquinolin-8-yl)-2-cyano-2-methyl-5-phenylpentanamide (2l):**

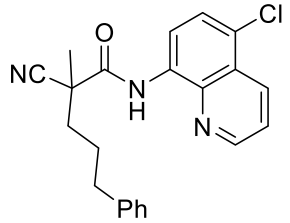

2-Cyano-2-methyl-5-phenyl-*N*-(quinolin-8-yl)pentanamide (**1l**, 137 mg, 0.40 mmol) and trichloroisocyanuric acid (34 mg, 0.145 mmol) in acetonitrile (3 mL), 2 h at room temperature. After column chromatography (gradient: EtOAc/hexanes 10:90 to 50:50) 143 mg (95%) of a white solid was obtained. R_f = 0.31 (EtOAc/hexanes 20:80), mp = 203-205 °C; ^{1}H NMR (400 MHz, $CDCl_3$): δ 10.81 (s, 1H), 8.89 (dd, *J* = 4.2, 1.6 Hz, 1H), 8.64 (d, *J* = 8.4 Hz, 1H), 8.52 (dd, *J* = 8.5, 1.5 Hz, 1H), 7.62-7.50 (m, 2H), 7.28-7.20 (m, 2H), 7.20-7.11 (m, 3H), 2.76-2.59 (m, 2H), 2.22-2.12 (m, 1H), 2.02-1.75 (m, 3H), 1.73 (s, 3H); $^{13}C\{^1H\}$NMR (100 MHz, $CDCl_3$): δ 166.3, 149.2, 140.8, 139.1, 133.2, 132.6, 128.3, 128.2, 126.8, 126.0, 125.7, 125.5, 122.5, 121.1, 116.5, 45.2, 37.9, 35.2, 27.1, 24.0; FTIR (neat): 3307, 2935, 2861, 1689, 1523,

1480, 1385, 1319, 1036, 945, 786, 697 cm^{-1}; MS (ESI): *m/z* 378 (M+H)$^+$; HRMS (ESI): *m/z* calcd for $C_{22}H_{21}ClN_3O$ (M+H)$^+$: 378.1368, found: 378.1370.

***N*-(5-Bromoquinolin-8-yl)-2-cyano-2-methyl-5-phenylpentanamide (3l):**

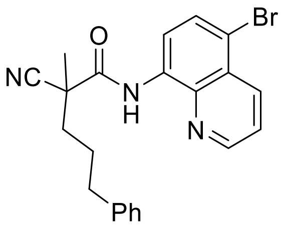

2-Cyano-2-methyl-5-phenyl-*N*-(quinolin-8-yl)pentanamide (**1l**, 137 mg, 0.40 mmol) and tribromoisocyanuric acid (53 mg, 0.145 mmol) in acetonitrile (3 mL), 2 h at room temperature. After column chromatography (gradient: EtOAc/hexanes 10:90 to 50:50) 158 mg (94%) of a white solid was obtained. R_f = 0.31 (EtOAc/hexanes 20:80), mp = 215-217 °C; ^{1}H NMR (400 MHz, $CDCl_3$): δ 10.83 (s, 1H), 8.87 (dd, *J* = 4.2, 1.6 Hz, 1H), 8.59 (d, *J* = 8.4 Hz, 1H), 8.49 (dd, *J* = 8.5, 1.6 Hz, 1H), 7.78 (d, *J* = 8.4 Hz, 1H), 7.56 (dd, *J* = 8.5, 4.2 Hz, 1H), 7.28-7.21 (m, 2H), 7.19-7.10 (m, 3H), 2.75-2.60 (m, 2H), 2.22-2.11 (m, 1H), 2.02-1.76 (m, 3H), 1.73 (s, 3H); ^{13}C{^{1}H}NMR (100 MHz, $CDCl_3$): δ 166.4, 149.3, 140.9, 139.2, 135.8, 133.2, 130.5, 128.3, 128.3, 127.0, 126.0, 122.9, 121.1, 117.1, 115.5, 45.3, 37.9, 35.2, 27.2, 24.0; FTIR (neat): 3305, 3026, 2860, 1689, 1520, 1477, 1385, 1108, 933, 834, 785, 697 cm^{-1}; MS (ESI): *m/z* 422 (M+H)$^+$; HRMS (ESI): *m/z* calcd for $C_{22}H_{21}BrN_3O$ (M+H)$^+$: 422.0863, found: 422.0866.

***N*-(5-Chloroquinolin-8-yl)-2-cyano-3-(4-methoxyphenyl)-2-methylpropanamide (2m):**

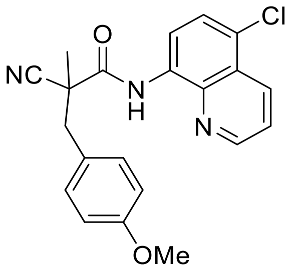

2-Cyano-3-(4-methoxyphenyl)-2-methyl-*N*-(quinolin-8-yl)propanamide (**1m**, 138 mg, 0.40 mmol) and trichloroisocyanuric acid (34 mg, 0.145 mmol) in acetonitrile (3 mL), 60 min at room temperature. After column chromatography (gradient: EtOAc/hexanes 10:90 to 50:50) 147 mg (97%) of a brown solid was obtained. R_f = 0.24 (EtOAc/hexanes 30:70), mp = 137-139 °C; ^{1}H NMR (400 MHz, $CDCl_3$): δ 10.56 (s, 1H), 8.83 (dd, *J* = 4.2, 1.6 Hz, 1H), 8.64 (d, *J* = 8.4 Hz, 1H), 8.54 (dd, *J* = 8.5, 1.6 Hz, 1H), 7.60 (d, *J* = 8.4 Hz, 1H), 7.55 (dd, *J* = 8.5, 4.2 Hz, 1H), 7.29-7.20 (m, 2H), 6.82-6.71 (m, 2H), 3.69 (s, 3H), 3.36 (d, *J* = 13.7 Hz, 1H), 3.07 (d, *J* = 13.7 Hz, 1H), 1.76 (s, 3H); ^{13}C{^{1}H}NMR (100 MHz, $CDCl_3$): δ 166.1, 159.1, 149.2, 139.1, 133.2, 132.6, 131.0, 126.8, 126.4, 125.8, 125.5, 122.5, 120.9, 116.6, 113.9, 55.1, 47.2, 43.2, 23.5; FTIR (neat): 3310, 2937, 2838, 1680, 1517, 1475, 1272, 1207, 948, 832, 785,

742 cm^{-1}; MS (ESI): *m/z* 380 (M+H)$^+$; HRMS (ESI): *m/z* calcd for $C_{21}H_{19}ClN_3O_2$ (M+H)$^+$: 380.1160, found: 380.1161.

***N*-(5-Bromoquinolin-8-yl)-2-cyano-3-(4-methoxyphenyl)-2-methylpropanamide (3m):**

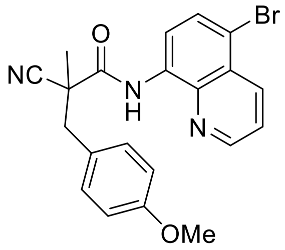

2-Cyano-3-(4-methoxyphenyl)-2-methyl-*N*-(quinolin-8-yl)propanamide (**1m**, 138 mg, 0.40 mmol) and tribromoisocyanuric acid (53 mg, 0.145 mmol) in acetonitrile (3 mL), 2 h at room temperature. After column chromatography (gradient: EtOAc/hexanes 10:90 to 50:50) 122 mg (95%) of a white solid was obtained. R_f = 0.25 (EtOAc/hexanes 30:70), mp = 130-132 °C; ^{1}H NMR (400 MHz, CDCl$_3$): δ 10.58 (s, 1H), 8.79 (dd, *J* = 4.2, 1.6 Hz, 1H), 8.59 (d, *J* = 8.4 Hz, 1H), 8.47 (dd, *J* = 8.5, 1.6 Hz, 1H), 7.79 (d, *J* = 8.4 Hz, 1H), 7.53 (dd, *J* = 8.5, 4.2 Hz, 1H), 7.28-7.21 (m, 2H), 6.83-6.70 (m, 2H), 3.68 (s, 3H), 3.36 (d, *J* = 13.7 Hz, 1H), 3.06 (d, *J* = 13.7 Hz, 1H), 1.76 (s, 3H); ^{13}C{^{1}H}NMR (100 MHz, CDCl$_3$): δ 166.1, 159.1, 149.2, 139.2, 135.7, 133.2, 131.0, 130.5, 127.0, 126.4, 122.8, 120.9, 117.1, 115.5, 113.9, 55.1, 47.2, 43.2, 23.5; FTIR (neat): 3313, 2967, 2838, 1681, 1529, 1516, 1474, 1246, 1174, 1032, 932, 784, 703 cm^{-1}; MS (ESI): *m/z* 426 (M+H)$^+$; HRMS (ESI): *m/z* calcd for $C_{21}H_{19}BrN_3O_2$ (M+H)$^+$: 426.0635, found: 426.0635.

***N*-(5-Chloroquinolin-8-yl)benzamide** (**2n**):[15, 21-22]

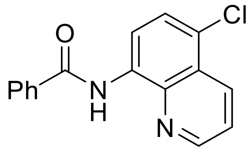

N-(Quinolin-8-yl)benzamide (**1n**, 99 mg, 0.40 mmol) and trichloroisocyanuric acid (34 mg, 0.145 mmol) in acetonitrile (3 mL), 30 min at room temperature. After column chromatography (gradient: EtOAc/hexanes 10:90 to 50:50) 109 mg (97%) of a white solid was obtained. R_f = 0.35 (EtOAc/hexanes 20:80); ^{1}H NMR (400 MHz, CDCl$_3$): δ 10.60 (s, 1H), 8.82 (dd, *J* = 5.0, 3.5 Hz, 2H), 8.49 (dd, *J* = 8.5, 1.6 Hz, 1H), 8.08-7.98 (m, 2H), 7.62-7.45 (m, 5H); ^{13}C{^{1}H}NMR (100 MHz, CDCl$_3$): δ 165.1, 148.6, 139.0, 134.6, 133.6, 133.2, 131.8, 128.7, 127.1, 127.1, 125.7, 124.3, 122.2, 116.2; MS (ESI): *m/z* 283 (M+H)$^+$.

***N*-(5-Bromoquinolin-8-yl)benzamide (3n):**[15, 21]

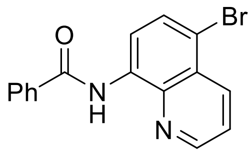

N-(Quinolin-8-yl)benzamide (**1n**, 99 mg, 0.40 mmol) and tribromoisocyanuric acid (53 mg, 0.145 mmol) in acetonitrile (3 mL), 30 min at room temperature. After column chromatography (gradient: EtOAc/hexanes 10:90 to 50:50) 125 mg (96%)

of a white solid was obtained. R_f = 0.36 (EtOAc/hexanes 20:80); ^{1}H NMR (400 MHz, $CDCl_3$): δ 10.70 (s, 1H), 8.86 (dd, J = 4.2, 1.6 Hz, 1H), 8.82 (d, J = 4.5 Hz, 1H), 8.53 (dd, J = 8.5, 1.6 Hz, 1H), 8.11-8.02 (m, 2H), 7.84 (d, J = 4.7 Hz, 1H), 7.62-7.49 (m, 4H); ^{13}C{^{1}H}NMR (100 MHz, $CDCl_3$): δ 165.3, 148.7, 139.3, 135.9, 134.7, 134.4, 132.0, 130.9, 128.8, 127.2, 127.2, 122.7, 116.9, 114.3; MS (ESI): m/z 327 $(M+H)^+$.

***N*-(5-Chloroquinolin-8-yl)-4-methylbenzamide (2o):**[15]

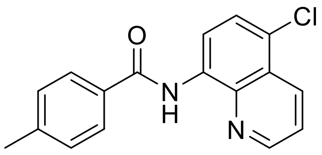

4-Methyl-*N*-(quinolin-8-yl)benzamide (**1o**, 105 mg, 0.40 mmol) and trichloroisocyanuric acid (34 mg, 0.145 mmol) in acetonitrile (3 mL), 30 min at room temperature. After column chromatography (gradient: EtOAc/hexanes 10:90 to 50:50) 117 mg (99%) of a white solid was obtained. R_f = 0.40 (EtOAc/hexanes 20:80); ^{1}H NMR (400 MHz, $CDCl_3$): δ 10.68 (s, 1H), 8.94-8.85 (m, 2H), 8.60 (dd, J = 8.5, 1.6 Hz, 1H), 7.97 (d, J = 8.2 Hz, 2H), 7.66 (d, J = 8.4 Hz, 1H), 7.61 (dd, J = 8.5, 4.2 Hz, 1H), 7.36 (d, J = 7.9 Hz, 2H), 2.46 (s, 3H); ^{13}C{^{1}H}NMR (100 MHz, $CDCl_3$): δ 165.5, 148.7, 142.6, 139.3, 134.0, 133.5, 132.1, 130.7, 129.5, 127.3, 126.0, 124.3, 122.4, 116.4, 21.6; MS (ESI): m/z 297 $(M+H)^+$.

***N*-(5-Bromoquinolin-8-yl)-4-methylbenzamide (3o):**[15]

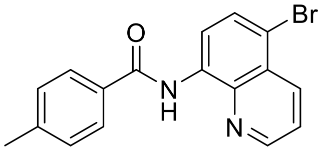

4-Methyl-*N*-benzamide (**1o**, 105 mg, 0.40 mmol) and tribromoisocyanuric acid (53 mg, 0.145 mmol) in acetonitrile (3 mL), 30 min at room temperature. After column chromatography (gradient: EtOAc/hexanes 10:90 to 50:50) 122 mg (97%) of a white solid was obtained. R_f = 0.42 (EtOAc/hexanes 20:80); ^{1}H NMR (400 MHz, $CDCl_3$): δ 10.68 (s, 1H), 8.87-8.81 (m, 2H), 8.54 (dd, J = 8.5, 1.6 Hz, 1H), 7.96 (dd, J = 8.1, 6.5 Hz, 2H), 7.83 (d, J = 7.9 Hz, 1H), 7.57 (dd, J = 8.5, 3.0 Hz, 1H), 7.34 (d, J = 7.9 Hz, 2H), 2.45 (s, 3H); ^{13}C{^{1}H}NMR (100 MHz, $CDCl_3$): δ 165.4, 148.7, 142.5, 139.4, 136.0, 134.6, 132.0, 131.0, 129.5, 127.3, 127.2, 122.7, 116.9, 114.2, 21.5; MS (ESI): m/z 341 $(M+H)^+$.

4-Chloro-*N*-(5-chloroquinolin-8-yl)benzamide (2p):[15]

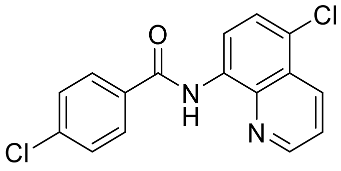

4-Chloro-*N*-(quinolin-8-yl)benzamide (**1p**, 113 mg, 0.40 mmol) and trichloroisocyanuric acid (34 mg, 0.145 mmol) in acetonitrile (3 mL), 2 h at room temperature. After column chromatography (gradient:

EtOAc/hexanes 10:90 to 50:50) 119 mg (94%) of a white solid was obtained. R_f = 0.40 (EtOAc/hexanes 20:80); ^{1}H NMR (400 MHz, $CDCl_3$): δ 10.58 (s, 1H), 8.84 (dd, J = 4.2, 1.5 Hz, 1H), 8.79 (d, J = 8.4 Hz, 1H), 8.53 (dd, J = 8.5, 1.5 Hz, 1H), 7.96 (dd, J = 8.9, 2.1 Hz, 2H), 7.59 (d, J = 8.4 Hz, 1H), 7.55 (dd, J = 8.5, 4.2 Hz, 1H), 7.48 (dd, J = 8.8, 2.1 Hz, 2H); ^{13}C{^{1}H}NMR (100 MHz, $CDCl_3$): δ 164.0, 148.7, 139.0, 138.2, 133.4, 133.4, 133.0, 129.0, 128.6, 127.1, 125.8, 124.6, 122.4, 116.4; MS (ESI): *m/z* 317 $(M+H)^+$.

***N*-(5-Bromoquinolin-8-yl)-4-chlorobenzamide (3p):**[15]

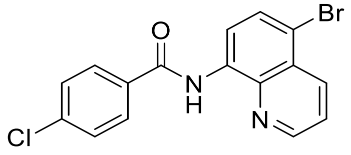

4-Chloro-*N*-(quinolin-8-yl)benzamide (**1p**, 113 mg, 0.40 mmol) and tribromoisocyanuric acid (53 mg, 0.145 mmol) in acetonitrile (3 mL), 2 h at room temperature. After column chromatography (gradient: EtOAc/hexanes 10:90 to 50:50) 135 mg (94%) of a white solid was obtained. R_f = 0.41 (EtOAc/hexanes 20:80); ^{1}H NMR (400 MHz, $CDCl_3$): δ 10.60 (s, 1H), 8.81 (dd, J = 4.2, 1.6 Hz, 1H), 8.73 (d, J = 8.4 Hz, 1H), 8.49 (dd, J = 8.5, 1.6 Hz, 1H), 7.98-7.94 (m, 2H), 7.78 (d, J = 8.4 Hz, 1H), 7.55 (dd, J = 8.5, 4.2 Hz, 1H), 7.52-7.45 (m, 2H); ^{13}C{^{1}H}NMR (100 MHz, $CDCl_3$): δ 164.0, 148.7, 139.2, 138.2, 135.9, 134.1, 133.0, 130.8, 129.0, 128.6, 127.1, 122.7, 116.9, 114.6; MS (ESI): *m/z* 361 $(M+H)^+$.

3-Chloro-*N*-(5-chloroquinolin-8-yl)benzamide (2q):[15]

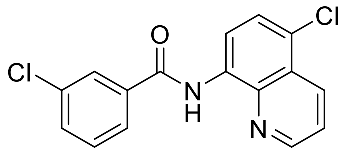

3-Chloro-*N*-(quinolin-8-yl)benzamide (**1q**, 113 mg, 0.40 mmol) and trichloroisocyanuric acid (34 mg, 0.145 mmol) in acetonitrile (3 mL), 60 min at room temperature. After column chromatography (gradient: EtOAc/hexanes 10:90 to 50:50) 121 mg (96%) of a white solid was obtained. R_f = 0.40 (EtOAc/hexanes 20:80); ^{1}H NMR (400 MHz, $CDCl_3$): δ 10.60 (s, 1H), 8.88 (dd, J = 4.2, 1.5 Hz, 1H), 8.81 (d, J = 8.4 Hz, 1H), 8.56 (dd, J = 8.5, 1.5 Hz, 1H), 8.02 (t, J = 1.8 Hz, 1H), 7.93-7.88 (m, 1H), 7.62 (d, J = 8.4 Hz, 1H), 7.60-7.53 (m, 2H), 7.48 (t, J = 7.8 Hz, 1H); ^{13}C{^{1}H}NMR (100 MHz, $CDCl_3$): δ 163.9, 148.9, 139.1, 136.5, 135.0, 133.5, 133.4, 132.0, 130.1, 127.7, 127.2, 125.9, 125.2, 124.8, 122.5, 116.5; MS (ESI): *m/z* 317 $(M+H)^+$.

***N*-(5-Bromoquinolin-8-yl)-3-chlorobenzamide (3q):**[15]

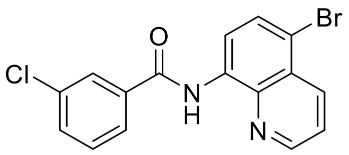

3-Chloro-*N*-(quinolin-8-yl)benzamide (**1q**, 113 mg, 0.40 mmol) and tribromoisocyanuric acid (53 mg, 0.145 mmol) in acetonitrile (3 mL), 60 min at room temperature. After column chromatography (gradient: EtOAc/hexanes 10:90 to 50:50) 137 mg (95%) of a white solid was obtained. R_f = 0.41 (EtOAc/hexanes 20:80); ^{1}H NMR (400 MHz, $CDCl_3$): δ 10.63 (s, 1H), 8.86 (dd, *J* = 4.2, 1.6 Hz, 1H), 8.77 (d, *J* = 8.4 Hz, 1H), 8.53 (dd, *J* = 8.5, 1.6 Hz, 1H), 8.03 (t, *J* = 1.8 Hz, 1H), 7.93-7.90 (m, 1H), 7.82 (d, *J* = 8.4 Hz, 1H), 7.60-7.54 (m, 2H), 7.51-7.46 (m, 1H); ^{13}C{^{1}H}NMR (100 MHz, $CDCl_3$): δ 163.9, 148.8, 139.2, 136.5, 136.0, 135.0, 134.1, 132.0, 130.9, 130.1, 127.6, 127.2, 125.2, 122.8, 117.1, 114.8; FTIR (neat): 3299, 3031, 2896, 1751, 1536, 1499, 1361, 1207, 1001, 956, 773, 694 cm^{-1}; MS (ESI): *m/z* 363 (M+H)$^+$; HRMS (ESI): *m/z* calcd for $C_{16}H_{11}BrClN_2O$ (M+H)$^+$: 362.9715, found: 362.9717.

3-Bromo-*N*-(5-chloroquinolin-8-yl)benzamide (2r):

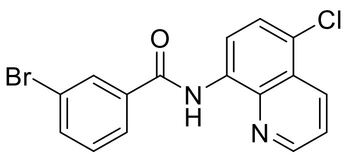

3-Bromo-*N*-(quinolin-8-yl)benzamide (**1r**, 130 mg, 0.40 mmol) and trichloroisocyanuric acid (34 mg, 0.145 mmol) in acetonitrile (3 mL), 4 h at room temperature. After column chromatography (gradient: EtOAc/hexanes 10:90 to 50:50) 138 mg (96%) of a white solid was obtained. R_f = 0.42 (EtOAc/hexanes 20:80), mp = 130-132 °C; ^{1}H NMR (400 MHz, $CDCl_3$): δ 10.61 (s, 1H), 8.90 (dd, *J* = 4.2, 1.6 Hz, 1H), 8.83 (d, *J* = 8.4 Hz, 1H), 8.58 (dd, *J* = 8.5, 1.6 Hz, 1H), 8.19 (t, *J* = 1.8 Hz, 1H), 7.96 (ddd, *J* = 7.8, 1.5, 1.0 Hz, 1H), 7.71 (ddd, *J* = 8.0, 1.9, 0.9 Hz, 1H), 7.64 (d, *J* = 8.4 Hz, 1H), 7.60 (dd, *J* = 8.5, 4.2 Hz, 1H), 7.42 (t, *J* = 7.9 Hz, 1H); ^{13}C{^{1}H}NMR (100 MHz, $CDCl_3$): δ 163.8, 148.8, 139.1, 136.7, 134.9, 133.5, 133.4, 130.6, 130.3, 127.2, 125.9, 125.6, 124.8, 123.1, 122.5, 116.6; FTIR (neat): 3352, 3069, 2923, 1674, 1518, 1479, 1383, 1252, 1049, 923, 825, 773 cm^{-1}; MS (ESI): *m/z* 361 (M+H)$^+$; HRMS (ESI): *m/z* calcd for $C_{16}H_{11}BrClN_2O$ (M+H)$^+$: 360.9738, found: 360.9741.

3-Bromo-*N*-(5-bromoquinolin-8-yl)benzamide (3r):

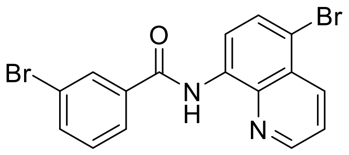

3-Bromo-*N*-(quinolin-8-yl)benzamide (**1r**, 130 mg, 0.40 mmol) and tribromoisocyanuric acid (53 mg, 0.145 mmol) in acetonitrile (3 mL), 4 h at room temperature. After column chromatography (gradient:

EtOAc/hexanes 10:90 to 50:50) 151 mg (93%) of a white solid was obtained. R_f = 0.44 (EtOAc/hexanes 20:80), mp = 138-139 °C; ^{1}H NMR (400 MHz, $CDCl_3$): δ 10.64 (s, 1H), 8.88 (dd, *J* = 4.2, 1.5 Hz, 1H), 8.79 (d, *J* = 8.4 Hz, 1H), 8.55 (dd, *J* = 8.5, 1.6 Hz, 1H), 8.19 (t, *J* = 1.8 Hz, 1H), 7.99-7.94 (m, 1H), 7.84 (d, *J* = 8.4 Hz, 1H), 7.72 (ddd, *J* = 8.0, 1.9, 1.0 Hz, 1H), 7.60 (dd, *J* = 8.3, 3.2 Hz, 1H), 7.43 (t, *J* = 7.9 Hz, 1H); ^{13}C{^{1}H}NMR (100 MHz, $CDCl_3$), δ 163.8, 148.9, 139.3, 136.8, 136.1, 135.0, 134.1, 130.9, 130.6, 130.5, 127.2, 125.7, 123.1, 122.8, 117.2, 114.8; FTIR (neat): 3352, 3069, 2923, 1674, 1518, 1479, 1383, 1252, 1049, 923, 825, 773 cm^{-1}; MS (ESI): *m/z* 407 (M+H)$^+$; HRMS (ESI): *m/z* calcd for $C_{16}H_{11}Br_2N_2O$ (M+H)$^+$: 406.9212, found: 406.9212.

N-(5-Chloroquinolin-8-yl)-4-(trifluoromethyl)benzamide (2s):[15]

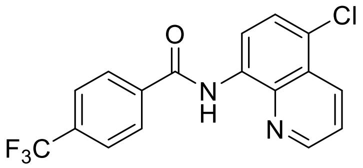

N-(Quinolin-8-yl)-4-(trifluoromethyl)benzamide (**1s**, 126 mg, 0.40 mmol) and trichloroisocyanuric acid (34 mg, 0.145 mmol) in acetonitrile (3 mL), 6 h at room temperature. After column chromatography (gradient: EtOAc/hexanes 10:90 to 50:50) 110 mg (79%) of a white solid was obtained. R_f = 0.40 (EtOAc/hexanes 20:80); ^{1}H NMR (400 MHz, $CDCl_3$): δ 10.70 (s, 1H), 8.88 (dd, *J* = 4.2, 1.6 Hz, 1H), 8.83 (d, *J* = 8.1 Hz, 1H), 8.58 (dd, *J* = 5.7, 2.8 Hz, 1H), 8.16 (d, *J* = 8.1 Hz, 2H), 7.81 (d, *J* = 8.2 Hz, 2H), 7.64 (d, *J* = 8.4 Hz, 1H), 7.60 (dd, *J* = 8.5, 4.2 Hz, 1H); ^{13}C{^{1}H}NMR (100 MHz, $CDCl_3$): δ 163.9, 148.9, 139.1, 138.0, 133.5, 133.4, 133.3, 127.7, 127.2, 126.0, 125.9, 125.8 (q, *J* = 3.7 Hz), 125.0, 122.5, 116.6; MS (ESI): *m/z* 351 (M+H)$^+$;

N-(5-Bromoquinolin-8-yl)-4-(trifluoromethyl)benzamide (3s):[15]

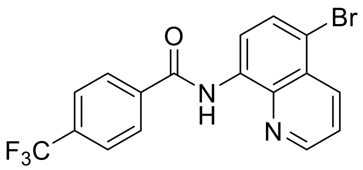

N-(Quinolin-8-yl)-4-(trifluoromethyl)benzamide (**1s**, 126 mg, 0.40 mmol) and tribromoisocyanuric acid (53 mg, 0.145 mmol) in acetonitrile (3 mL), 3 h at room temperature. After column chromatography (gradient: EtOAc/hexanes 10:90 to 50:50) 118 mg (75%) of a white solid was obtained. R_f = 0.41 (EtOAc/hexanes 20:80); ^{1}H NMR (400 MHz, $CDCl_3$): δ 10.74 (s, 1H), 8.87 (dd, *J* = 4.2, 1.6 Hz, 1H), 8.80 (d, *J* = 8.1 Hz, 1H), 8.56 (dd, *J* = 8.5, 1.6 Hz, 1H), 8.17 (d, *J* = 8.1 Hz, 2H), 7.88-7.78 (m, 3H), 7.61 (dd, *J* = 8.5, 4.2 Hz,

1H); $^{13}C\{^1H\}$NMR (100 MHz, $CDCl_3$): δ 164.0, 148.9, 139.3, 138.0, 136.1, 134.0, 130.9, 127.7, 127.3, 126.0, 125.9 (q, J = 3.7 Hz), 125.8, 122.9, 117.2, 115.0; MS (ESI): m/z 395 $(M+H)^+$.

***N*-(5-Chloroquinolin-8-yl)-1-naphthamide (2t):**[23]

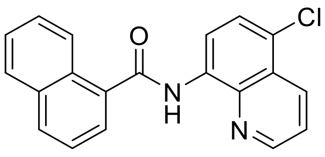

N-(Quinolin-8-yl)-1-naphthamide (**1t**, 119 mg, 0.40 mmol) and trichloroisocyanuric acid (34 mg, 0.145 mmol) in acetonitrile (3 mL), 60 min at room temperature. After column chromatography (gradient: EtOAc/hexanes 10:90 to 50:50) 129 mg (97%) of a white solid was obtained. R_f = 0.40 (EtOAc/hexanes 20:80); ^{1}H NMR (400 MHz, $CDCl_3$): δ 10.38 (s, 1H), 9.00 (d, J = 8.4 Hz, 1H), 8.79 (dd, J = 4.2, 1.6 Hz, 1H), 8.59 (dd, J = 8.5, 1.6 Hz, 1H), 8.55-8.48 (m, 1H), 8.02 (d, J = 8.3 Hz, 1H), 7.96-7.88 (m, 2H), 7.70 (d, J = 8.4 Hz, 1H), 7.62-7.52 (m, 4H); $^{13}C\{^1H\}$NMR (100 MHz, $CDCl_3$): δ 167.7, 148.8, 139.1, 134.3, 134.0, 133.8, 133.4, 131.3, 130.3, 128.4, 127.4, 127.2, 126.6, 126.0, 125.5, 125.4, 124.8, 124.7, 122.4, 116.6; MS (ESI): m/z 333 $(M+H)^+$.

***N*-(5-Bromoquinolin-8-yl)-1-naphthamide (3t):**

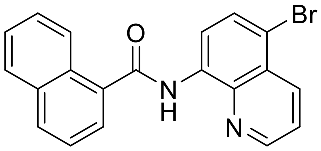

N-(Quinolin-8-yl)-1-naphthamide (**1t**, 119 mg, 0.40 mmol) and tribromoisocyanuric acid (53 mg, 0.145 mmol) in acetonitrile (3 mL), 60 min at room temperature. After column chromatography (gradient: EtOAc/hexanes 10:90 to 50:50) 143 mg (95%) of a white solid was obtained. R_f = 0.41 (EtOAc/hexanes 20:80), mp = 178-179 °C; ^{1}H NMR (400 MHz, $CDCl_3$): δ 10.41 (s, 1H), 8.95 (d, J = 8.4 Hz, 1H), 8.77 (dd, J = 4.2, 1.6 Hz, 1H), 8.56 (dd, J = 8.5, 1.6 Hz, 1H), 8.53-8.50 (m, 1H), 8.02 (d, J = 8.3 Hz, 1H), 7.95-7.87 (m, 3H), 7.62-7.53 (m, 4H); $^{13}C\{^1H\}$NMR (100 MHz, $CDCl_3$): δ 167.7, 148.8, 139.3, 136.0, 134.7, 134.3, 133.8, 131.3, 130.9, 130.3, 128.4, 127.4, 127.3, 126.6, 125.5, 125.4, 124.8, 122.7, 117.2, 114.7; FTIR (neat): 3350, 3049, 1672, 1521, 1473, 1320, 1238, 1065, 915, 829, 772 cm^{-1}; MS (ESI): m/z 377 $(M+H)^+$; HRMS (ESI): m/z calcd for $C_{20}H_{13}BrN_2O$ $(M+H)^+$: 377.0284, found: 377.0283.

5-Chloro-*N*-(5-chloroquinolin-8-yl)thiophene-2-carboxamide (2u):

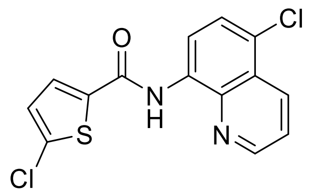

5-Chloro-*N*-(quinolin-8-yl)thiophene-2-carboxamide (**1u**, 115 mg, 0.40 mmol) and trichloroisocyanuric acid (34 mg, 0.145 mmol) in acetonitrile (3 mL), 60 min at room temperature. After column chromatography (gradient: EtOAc/hexanes 10:90 to 50:50) 106 mg (83%) of a white solid was obtained. R_f = 0.35 (EtOAc/hexanes 20:80), mp = 70-71 °C; ^{1}H NMR (400 MHz, $CDCl_3$): δ 10.44 (s, 1H), 8.88 (dd, *J* = 4.2, 1.5 Hz, 1H), 8.73 (d, *J* = 8.4 Hz, 1H), 8.58 (dd, *J* = 8.5, 1.5 Hz, 1H), 7.65-7.55 (m, 3H), 7.00 (d, *J* = 4.0 Hz, 1H); ^{13}C{^{1}H}NMR (100 MHz, $CDCl_3$): δ 158.9, 148.8, 138.9, 138.2, 136.5, 133.5, 133.2, 127.7, 127.7, 127.3, 126.0, 124.7, 122.5, 116.5; FTIR (neat): 3338, 3120, 3063, 1663, 1536, 1524, 1478, 1266, 1072, 995, 803, 781 cm^{-1}; MS (ESI): *m/z* 323 $(M+H)^+$; HRMS (ESI): *m/z* calcd for $C_{14}H_9Cl_2N_2OS$ $(M+H)^+$: 322.9807, found: 322.9808.

***N*-(5-Bromoquinolin-8-yl)-5-chlorothiophene-2-carboxamide (3u):**

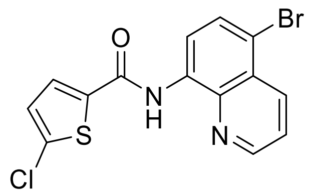

5-Chloro-*N*-(quinolin-8-yl)thiophene-2-carboxamide (**1u**, 115 mg, 0.40 mmol) and tribromoisocyanuric acid (53 mg, 0.145 mmol) in acetonitrile (3 mL), 60 min at room temperature. After column chromatography (gradient: EtOAc/hexanes 10:90 to 50:50) 118 mg (81%) of a white solid was obtained. R_f = 0.36 (EtOAc/hexanes 20:80), mp = 177-178 °C; ^{1}H NMR (400 MHz, $CDCl_3$): δ 10.47 (s, 1H), 8.86 (dd, *J* = 4.2, 1.6 Hz, 1H), 8.68 (d, *J* = 8.4 Hz, 1H), 8.55 (dd, *J* = 8.5, 1.6 Hz, 1H), 7.83 (d, *J* = 8.4 Hz, 1H), 7.63-7.59 (m, 2H), 7.01 (d, *J* = 4.0 Hz, 1H); ^{13}C{^{1}H}NMR (100 MHz, $CDCl_3$): δ 158.9, 148.9, 139.1, 138.2, 136.5, 136.1, 133.9, 131.0, 127.8, 127.3, 127.2, 122.9, 117.0, 114.7; FTIR (neat): 3336, 3121, 3061, 1661, 1537, 1523, 1383, 1217, 994, 838, 738 cm^{-1}; MS (ESI): *m/z* 367 $(M+H)^+$; HRMS (ESI): *m/z* calcd for $C_{14}H_9BrClN_2OS$ $(M+H)^+$: 366.9302, found: 366.9301.

***tert*-Butyl (5-chloroquinolin-8-yl)carbamate (2v):**[24]

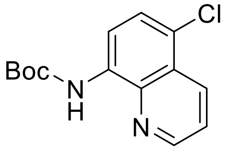

tert-Butyl quinolin-8-ylcarbamate (**1v**, 98 mg, 0.40 mmol) and trichloroisocyanuric acid (34 mg, 0.145 mmol) in acetonitrile (3 mL), 60 min at room temperature. After column chromatography (gradient: EtOAc/hexanes 10:90 to 50:50) 109 mg (98%) of a white solid was obtained. R_f = 0.30 (EtOAc/hexanes 20:80); ^{1}H NMR (400 MHz, $CDCl_3$): δ 8.97 (s, 1H),

8.81 (dd, J = 4.2, 1.6 Hz, 1H), 8.51 (dd, J = 8.5, 1.6 Hz, 1H), 8.34 (d, J = 8.4 Hz, 1H), 7.55 (d, J = 8.4 Hz, 1H), 7.52 (dd, J = 8.5, 4.2 Hz, 1H), 1.58 (s, 9H); $^{13}C\{^1H\}$NMR (100 MHz, $CDCl_3$): δ 152.7, 148.3, 138.6, 134.4, 133.2, 127.1, 125.9, 122.8, 122.2, 114.2, 80.7, 28.3; FTIR (neat): 3381, 2977, 2931, 1722, 1518, 1477, 1383, 1247, 1124, 999, 831, 781, 711 cm^{-1}; MS (ESI): *m/z* 279 $(M+H)^+$; HRMS (ESI): *m/z* calcd for $C_{14}H_{16}ClN_2O_2$ $(M+H)^+$: 279.0895, found: 279.0896.

***tert*-Butyl (5-bromoquinolin-8-yl)carbamate (3v):**[24]

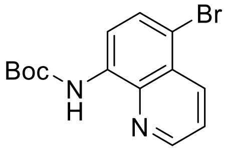

tert-Butyl quinolin-8-ylcarbamate (**1v**, 98 mg, 0.40 mmol) and tribromoisocyanuric acid (53 mg, 0.145 mmol) in acetonitrile (3 mL), 60 min at room temperature. After column chromatography (gradient: EtOAc/hexanes 10:90 to 50:50) 125 mg (97%) of a white solid was obtained. R_f = 0.31 (EtOAc/hexanes 20:80); ^1H NMR (400 MHz, $CDCl_3$): δ 8.99 (s, 1H), 8.78 (dd, J = 4.2, 1.6 Hz, 1H), 8.46 (dd, J = 8.5, 1.6 Hz, 1H), 8.29 (d, J = 8.4 Hz, 1H), 7.74 (d, J = 8.4 Hz, 1H), 7.51 (dd, J = 8.5, 4.2 Hz, 1H), 1.58 (s, 9H); $^{13}C\{^1H\}$NMR (100 MHz, $CDCl_3$): δ 152.6, 148.3, 138.8, 135.7, 135.1, 130.7, 127.1, 122.5, 114.8, 112.6, 80.7, 28.3; MS (ESI): *m/z* 323 $(M+H)^+$.

Ethyl (5-chloroquinolin-8-yl)carbamate (2w):

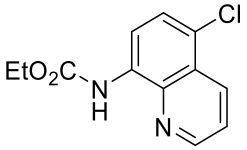

Ethyl quinolin-8-ylcarbamate (**1w**, 86 mg, 0.40 mmol) and trichloroisocyanuric acid (34 mg, 0.145 mmol) in acetonitrile (3 mL), 30 min at room temperature. After column chromatography (gradient: EtOAc/hexanes 10:90 to 50:50) 98 mg (98%) of a white solid was obtained. R_f = 0.20 (EtOAc/hexanes 20:80), mp = 83-85 ^oC; ^1H NMR (400 MHz, $CDCl_3$): δ 9.12 (s, 1H), 8.81 (dd, J = 4.2, 1.6 Hz, 1H), 8.52 (dd, J = 8.5, 1.6 Hz, 1H), 8.33 (dd, J = 20.5, 4.9 Hz, 1H), 7.57 (d, J = 8.4 Hz, 1H), 7.53 (dd, J = 8.5, 4.2 Hz, 1H), 4.30 (q, J = 7.1 Hz, 2H), 1.37 (t, J = 7.1 Hz, 3H); $^{13}C\{^1H\}$NMR (100 MHz, $CDCl_3$): δ 153.5, 148.5, 138.6, 134.1, 133.2, 127.1, 125.9, 123.2, 122.3, 114.4, 61.3, 14.5; FTIR (neat): 3366, 2989, 2907, 1732, 1582, 1525, 1379, 1155, 1043, 934, 831, 762 cm^{-1}; MS (ESI): *m/z* 251 $(M+H)^+$; HRMS (ESI): *m/z* calcd for $C_{12}H_{12}ClN_2O_2$ $(M+H)^+$: 251.0582, found: 251.0585.

Ethyl (5-bromoquinolin-8-yl)carbamate (3w):

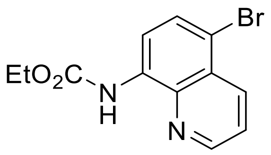

Ethyl quinolin-8-ylcarbamate (**1w**, 86 mg, 0.40 mmol) and tribromoisocyanuric acid (53 mg, 0.145 mmol) in acetonitrile (3 mL), 30 min at room temperature. After column chromatography (gradient: EtOAc/hexanes 10:90 to 50:50) 117 mg (98%) of a white solid was obtained. R_f = 0.21 (EtOAc/hexanes 20:80), mp = 88-90 ^oC; ^1H NMR (400 MHz, $CDCl_3$): δ 9.12 (s, 1H), 8.76 (dd, *J* = 4.2, 1.6 Hz, 1H), 8.45 (dd, *J* = 8.5, 1.6 Hz, 1H), 8.29 (d, *J* = 8.3 Hz, 1H), 7.74 (d, *J* = 8.4 Hz, 1H), 7.50 (dd, *J* = 8.5, 4.2 Hz, 1H), 4.30 (q, *J* = 7.1 Hz, 2H), 1.37 (t, *J* = 7.1 Hz, 3H); $^{113}C\{^1H\}$NMR (100 MHz, $CDCl_3$): δ 153.4, 148.5, 138.7, 135.7, 134.7, 130.7, 127.1, 122.6, 115.0, 113.0, 61.3, 14.6; FTIR (neat): 3365, 3987, 2905, 1731, 1575, 1524, 1465, 1379, 1195, 1043, 992, 780, 762 cm^{-1}; MS (ESI): *m/z* 295 $(M+H)^+$; HRMS (ESI): *m/z* calcd for $C_{12}H_{12}BrN_2O_2$ $(M+H)^+$: 295.0077, found: 295.0081.

***N*-(5-Chloroquinolin-8-yl)-*N*-methylbenzamide (2x):**

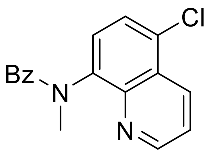

N-Methyl-*N*-(quinolin-8-yl)benzamide (**1x**, 105 mg, 0.40 mmol) and trichloroisocyanuric acid (34 mg, 0.145 mmol) in acetonitrile (3 mL), 6 h at room temperature. After column chromatography (gradient: EtOAc/hexanes 10:90 to 50:50) 103 mg (87%) of a white solid was obtained. R_f = 0.21 (EtOAc/hexanes 20:80), mp = 164-165 ^oC; ^1H NMR (400 MHz, $CDCl_3$): δ 9.03 (dd, *J* = 4.2, 1.7 Hz, 1H), 8.52 (dd, *J* = 8.6, 1.7 Hz, 1H), 7.53 (dd, *J* = 8.6, 4.2 Hz, 1H), 7.45 (d, *J* = 8.0 Hz, 1H), 7.35 (d, *J* = 8.0 Hz, 1H), 7.29 (d, *J* = 7.3 Hz, 2H), 7.11 (t, *J* = 7.4 Hz, 1H), 7.02 (t, *J* = 7.5 Hz, 2H), 3.57 (s, 3H); $^{13}C\{^1H\}$NMR (100 MHz, $CDCl_3$): δ 172.1, 151.0, 144.3, 141.6, 136.4, 133.2, 130.5, 129.4, 128.8, 127.8, 127.5, 127.1, 126.2, 122.4, 38.6; FTIR (neat): 3061, 2925, 1645, 1602, 1577, 1365, 1313, 1105, 838, 786, 727 cm^{-1}; MS (ESI): *m/z* 297 $(M+H)^+$; HRMS (ESI): *m/z* calcd for $C_{17}H_{14}ClN_2O$ $(M+H)^+$: 297.0789, found: 297.0793.

***N*-(5-Bromoquinolin-8-yl)-*N*-methylbenzamide (3x):**

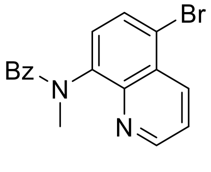

N-Methyl-*N*-(quinolin-8-yl)benzamide (**1x**, 105 mg, 0.40 mmol) and tribromoisocyanuric acid (53 mg, 0.145 mmol) in acetonitrile (3 mL), 6 h at room temperature. After column chromatography (gradient: EtOAc/hexanes 10:90 to 50:50)

125 mg (92%) of a white solid was obtained. R_f = 0.21 (EtOAc/hexanes 20:80), mp = 110-111 °C; ^{1}H NMR (400 MHz, $CDCl_3$): δ 9.00 (dd, J = 4.2, 1.6 Hz, 1H), 8.47 (dd, J = 8.6, 1.6 Hz, 1H), 7.65 (d, J = 8.0 Hz, 1H), 7.52 (dd, J = 8.6, 4.2 Hz, 1H), 7.29 (dd, J = 7.9, 2.3 Hz, 3H), 7.15-7.06 (m, 1H), 7.01 (t, J = 7.5 Hz, 2H), 3.57 (s, 3H); $^{13}C\{^1H\}$NMR (100 MHz, $CDCl_3$): δ 172.0, 151.0, 144.3, 142.3, 136.3, 135.8, 129.9, 129.4, 129.2, 128.4, 127.8, 127.4, 122.7, 120.9, 38.5; FTIR (neat): 3035, 2925, 1633, 1601, 1587, 1390, 1270, 1060, 1002, 787, 729 cm^{-1}; MS (ESI): *m/z* 341 (M+H)$^+$; HRMS (ESI): *m/z* calcd for $C_{17}H_{14}BrN_2O$ (M+H)$^+$: 341.0284, found: 341.0289.

***tert*-Butyl benzoyl(5-chloroquinolin-8-yl)carbamate (2y):**

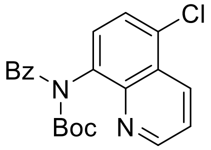

tert-Butyl benzoyl(quinolin-8-yl)carbamate (**1y**, 139 mg, 0.40 mmol) and trichloroisocyanuric acid (34 mg, 0.145 mmol) in acetonitrile (3 mL), 5 h at room temperature. After column chromatography (gradient: EtOAc/hexanes 10:90 to 50:50) 134 mg (88%) of a white solid was obtained. R_f = 0.20 (EtOAc/hexanes 20:80), mp = 170-171 °C; ^{1}H NMR (400 MHz, $CDCl_3$): δ 8.92 (dd, J = 4.2, 1.6 Hz, 1H), 8.58 (dd, J = 8.6, 1.6 Hz, 1H), 7.95-7.87 (m, 2H), 7.68 (d, J = 8.0 Hz, 1H), 7.62 (d, J = 8.0 Hz, 1H), 7.55-7.50 (m, 2H), 7.48-7.43 (m, 2H), 1.19 (s, 9H); $^{13}C\{^1H\}$NMR (100 MHz, $CDCl_3$): δ 173.3, 153.2, 151.0, 144.5, 137.2, 136.4, 133.2, 131.4, 128.9, 128.5, 128.1, 128.0, 127.1, 126.3, 122.4, 83.4, 27.4; FTIR (neat): 3294, 2979, 1730, 1669, 1568, 1496, 1359, 1241, 1149, 995, 872, 724 cm^{-1}; MS (ESI): *m/z* 383 (M+H)$^+$; HRMS (ESI): *m/z* calcd for $C_{21}H_{20}ClN_2O_3$ (M+H)$^+$: 383.1157, found: 383.1158.

***tert*-Butyl benzoyl(5-bromoquinolin-8-yl)carbamate (3y):**

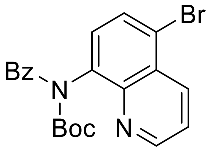

tert-Butyl benzoyl(quinolin-8-yl)carbamate (**1y**, 139 mg, 0.40 mmol) and tribromoisocyanuric acid (53 mg, 0.145 mmol) in acetonitrile (3 mL), 4 h at room temperature. After column chromatography (gradient: EtOAc/hexanes 10:90 to 50:50) 155 mg (91%) of a white solid was obtained. R_f = 0.21 (EtOAc/hexanes 20:80), mp = 192-193 °C; ^{1}H NMR (400 MHz, $CDCl_3$): δ 8.88 (dd, J = 4.2, 1.7 Hz, 1H), 8.57 (dd, J = 8.3, 1.7 Hz, 1H), 7.97-7.91 (m, 2H), 7.83 (dd, J = 8.2, 1.4 Hz, 1H), 7.69 (dd, J = 7.4, 1.4 Hz, 1H), 7.58 (dd, J = 8.1, 7.4 Hz, 1H), 7.54-7.49 (m, 1H),

7.47-7.42 (m, 1H), 7.40 (dd, J = 8.3, 4.2 Hz, 1H), 1.20 (s, 9H); $^{13}C\{^1H\}$NMR (100 MHz, $CDCl_3$): δ 173.4, 153.4, 150.4, 144.0, 137.4, 137.2, 136.0, 131.3, 129.1, 129.0, 128.5, 128.2, 128.0, 126.2, 121.6, 83.1, 27.4; FTIR (neat): 3007, 2976, 1730, 1666, 1566, 1463, 1353, 1149, 925, 869, 785, 695 cm^{-1}; MS (ESI): *m/z* 427 $(M+H)^+$; HRMS (ESI): *m/z* calcd for $C_{21}H_{20}BrN_2O_3$ $(M+H)^+$: 427.0652, found: 427.0653.

***tert*-Butyl acetyl(5-chloroquinolin-8-yl)carbamate (2z):**

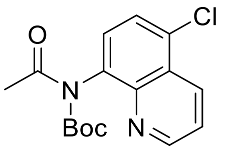

tert-Butyl acetyl(quinolin-8-yl)carbamate (**1z**, 115 mg, 0.40 mmol) and trichloroisocyanuric acid (34 mg, 0.145 mmol) in acetonitrile (3 mL), 5 h at room temperature. After column chromatography (gradient: EtOAc/hexanes 20:80 to 80:20) 118 mg (92%) of a white solid was obtained. R_f = 0.20 (EtOAc/hexanes 40:60), mp = 124-126 °C; ^1H NMR (400 MHz, $CDCl_3$): δ 8.92 (dd, J = 4.2, 1.6 Hz, 1H), 8.55 (dd, J = 8.5, 1.6 Hz, 1H), 7.64 (d, J = 8.0 Hz, 1H), 7.50 (dd, J = 8.5, 4.2 Hz, 1H), 7.47 (d, J = 8.0 Hz, 1H), 2.74 (s, 3H), 1.24 (s, 9H); $^{13}C\{^1H\}$NMR (100 MHz, $CDCl_3$): δ 173.4, 152.6, 150.8, 144.4, 135.9, 132.9, 131.1, 128.7, 126.7, 126.1, 122.1, 82.8, 27.5, 26.3; FTIR (neat): 3303, 2968, 1732, 1690, 1586, 1425, 1311, 1172, 968, 830, 699 cm^{-1}; MS (ESI): *m/z* 321 $(M+H)^+$; HRMS (ESI): *m/z* calcd for $C_{16}H_{18}ClN_2O_3$ $(M+H)^+$: 321.1000, found: 321.1007.

***tert*-Butyl acetyl(5-bromoquinolin-8-yl)carbamate (3z):**

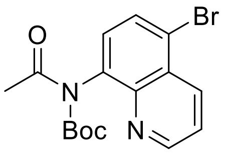

tert-Butyl acetyl(quinolin-8-yl)carbamate (**1z**, 115 mg, 0.40 mmol) and tribromoisocyanuric acid (53 mg, 0.145 mmol) in acetonitrile (3 mL), 4 h at room temperature. After column chromatography (gradient: EtOAc/hexanes 20:80 to 80:20) 132 mg (91%) of a white solid was obtained. R_f = 0.21 (EtOAc/hexanes 40:60), mp = 142-144 °C; ^1H NMR (400 MHz, $CDCl_3$): δ 8.91 (dd, J = 4.2, 1.6 Hz, 1H), 8.53 (dd, J = 8.6, 1.6 Hz, 1H), 7.85 (d, J = 7.9 Hz, 1H), 7.52 (dd, J = 8.6, 4.2 Hz, 1H), 7.41 (d, J = 7.9 Hz, 1H), 2.74 (s, 3H), 1.24 (s, 9H); $^{13}C\{^1H\}$NMR (100 MHz, $CDCl_3$): δ 173.4, 152.6, 151.0, 144.7, 136.7, 135.6, 129.8, 129.3, 128.1, 122.6, 121.7, 82.9, 27.6, 26.3; FTIR (neat): 3260, 3003, 1726, 1696, 1553, 1486, 1298, 1154, 998, 864, 712, 683 cm^{-1}; MS (ESI): *m/z* 365 $(M+H)^+$; HRMS (ESI): *m/z* calcd for $C_{16}H_{18}BrN_2O_3$ $(M+H)^+$: 365.0495, found: 365.0497.

***N*-(5-Chloro-2-methylquinolin-8-yl)benzamide (4a):**

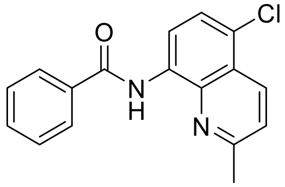

N-(2-Methylquinolin-8-yl)benzamide (**1aa**, 105 mg, 0.40 mmol) and trichloroisocyanuric acid (34 mg, 0.145 mmol) in acetonitrile (3 mL), 30 min at room temperature. After column chromatography (gradient: EtOAc/hexanes 10:90 to 50:50) 115 mg (97%) of a white solid was obtained. R_f = 0.35 (EtOAc/hexanes 20:80), mp = 106-108 °C; ^{1}H NMR (400 MHz, $CDCl_3$): δ 10.69 (s, 1H), 8.80 (d, *J* = 8.4 Hz, 1H), 8.38 (d, *J* = 8.6 Hz, 1H), 8.07-8.01 (m, 2H), 7.66-7.47 (m, 4H), 7.39 (d, *J* = 8.6 Hz, 1H), 2.76 (s, 3H); ^{13}C{^{1}H}NMR (100 MHz, $CDCl_3$): δ 165.1, 157.9, 138.6, 134.9, 133.4, 133.1, 131.9, 128.8, 127.1, 126.1, 124.4, 124.0, 123.1, 116.3, 25.2; FTIR (neat): 3340, 3066, 2919, 1666, 1529, 1484, 1376, 1256, 936, 788, 683 cm^{-1}; MS (ESI): *m/z* 297 $(M+H)^+$; HRMS (ESI): *m/z* calcd for $C_{17}H_{14}ClN_2O$ $(M+H)^+$: 297.0789, found: 297.0798.

***N*-(5-Bromo-2-methylquinolin-8-yl)benzamide (5a):**[25]

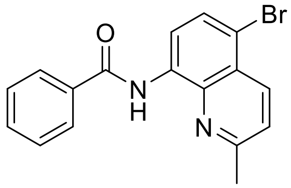

N-(2-Methylquinolin-8-yl)benzamide (**1aa**, 105 mg, 0.40 mmol) and tribromoisocyanuric acid (53 mg, 0.145 mmol) in acetonitrile (3 mL), 30 min at room temperature. After column chromatography (gradient: EtOAc/hexanes 10:90 to 50:50) 133 mg (98%) of a white solid was obtained. R_f = 0.38 (EtOAc/hexanes 20:80); ^{1}H NMR (400 MHz, $CDCl_3$): δ 10.75 (s, 1H), 8.78 (d, *J* = 8.4 Hz, 1H), 8.38 (d, *J* = 8.6 Hz, 1H), 8.08-8.03 (m, 2H), 7.75 (d, *J* = 8.4 Hz, 1H), 7.65-7.51 (m, 3H), 7.42 (d, *J* = 8.6 Hz, 1H), 2.79 (s, 3H); ^{13}C{^{1}H}NMR (100 MHz, $CDCl_3$): δ 165.2, 157.9, 138.8, 136.0, 134.9, 133.8, 131.9, 129.9, 128.8, 127.2, 125.4, 123.5, 116.9, 114.4, 25.2; FTIR (neat): 3318, 3028, 2914, 1671, 1580, 1525, 1480, 1391, 1223, 1093, 827, 789 cm^{-1}; MS (ESI): *m/z* 363 $(M+Na)^+$; HRMS (ESI): *m/z* calcd for $C_{17}H_{14}BrN_2O$ $(M+H)^+$: 341.0284, found: 341.0287.

3-(5-Chloroquinolin-8-yl)-1,1-dimethylurea (4b):

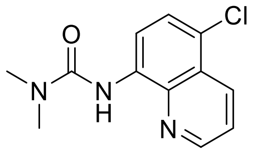

1,1-Dimethyl-3-(quinolin-8-yl)urea (**1ab**, 86 mg, 0.40 mmol) and trichloroisocyanuric acid (34 mg, 0.145 mmol) in acetonitrile (3 mL), 30 min at room temperature. After column chromatography (gradient: EtOAc/hexanes 10:90 to 50:50) 99 mg (99%) of a white solid was obtained. R_f = 0.20 (EtOAc/hexanes 30:70), mp = 68-69 °C; ^{1}H NMR (400 MHz, $CDCl_3$): δ 9.29 (s, 1H), 8.78 (dd, *J* = 4.2, 1.6 Hz, 1H), 8.55-8.47 (m, 2H), 7.56 (d, *J* =

8.5 Hz, 1H), 7.52 (dd, *J* = 8.5, 4.2 Hz, 1H), 3.15 (s, 6H); $^{13}C\{^1H\}$NMR (100 MHz, $CDCl_3$): δ 155.2, 148.1 (2), 138.9, 135.3, 133.2, 127.4, 125.8, 122.0, 114.4, 36.3; FTIR (neat): 3321, 2923, 2854, 1711, 1590, 1505, 1379, 1170, 1088, 908, 833, 730 cm^{-1}; MS (ESI): *m/z* 250 $(M+H)^+$; HRMS (ESI): *m/z* calcd for $C_{12}H_{13}ClN_3O$ $(M+H)^+$: 250.0742, found: 250.0743.

3-(5-Bromoquinolin-8-yl)-1,1-dimethylurea (5b):

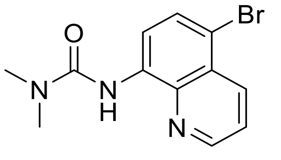

1,1-Dimethyl-3-(quinolin-8-yl)urea (**1ab**, 86 mg, 0.40 mmol) and tribromoisocyanuric acid (53 mg, 0.145 mmol) in acetonitrile (3 mL), 30 min at room temperature. After column chromatography (gradient: EtOAc/hexanes 10:90 to 50:50) 115 mg (98%) of a white solid was obtained. R_f = 0.21 (EtOAc/hexanes 30:70), mp = 84-85 °C; ^1H NMR (400 MHz, $CDCl_3$): δ 9.31 (s, 1H), 8.75 (dd, *J* = 4.2, 1.6 Hz, 1H), 8.52-8.39 (m, 2H), 7.74 (d, *J* = 8.5 Hz, 1H), 7.50 (dd, *J* = 8.5, 4.2 Hz, 1H), 3.15 (s, 6H); $^{13}C\{^1H\}$NMR (100 MHz, $CDCl_3$): δ 155.1, 148.1, 139.0, 135.9, 135.8, 131.0, 127.0, 122.3, 115.0, 111.8, 36.3; FTIR (neat): 3313, 3247, 2923, 1682, 1630, 1571, 1397, 1342, 1167, 909, 839, 762, 710 cm^{-1}; MS (ESI): *m/z* 294 $(M+H)^+$; HRMS (ESI): *m/z* calcd for $C_{12}H_{13}BrN_3O$ $(M+H)^+$: 294.0237, found: 294.0239.

1-(4-Chlorophenyl)-3-(5-chloroquinolin-8-yl)urea (4c):

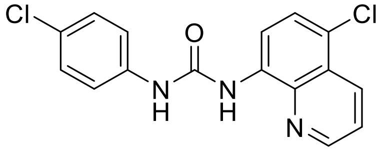

1-(4-Chlorophenyl)-3-(quinolin-8-yl)urea (**1ac**, 119 mg, 0.40 mmol) and trichloroisocyanuric acid (34 mg, 0.145 mmol) in acetonitrile (3 mL), 30 min at room temperature. After column chromatography (gradient: EtOAc/hexanes 10:90 to 50:50) 131 mg (99%) of a white solid was obtained. R_f = 0.20 (EtOAc/hexanes 30:70), mp = 211-213 °C; ^1H NMR (400 MHz, $CDCl_3$ + DMSO-*d6*, 8:2): δ 9.66 (d, *J* = 12.1 Hz, 2H), 8.93-8.85 (m, 1H), 8.62-8.53 (m, 2H), 7.66-7.49 (m, 4H), 7.24 (d, *J* = 8.7 Hz, 2H); $^{13}C\{^1H\}$NMR (100 MHz, $CDCl_3$ + DMSO-*d6*, 8:2): δ 152.0, 147.3, 138.2, 137.8, 134.8, 132.5, 127.8, 126.4, 125.8, 125.1, 121.5, 121.3, 119.0, 114.1; FTIR (neat): 3291, 3071, 2855, 1590, 1505, 1493, 1470, 1379, 1170, 1152, 883, 762, 709 cm^{-1}; MS (ESI): *m/z* 332 $(M+H)^+$; HRMS (ESI): *m/z* calcd for $C_{16}H_{12}Cl_2N_3O$ $(M+H)^+$: 332.0352, found: 332.0358.

1-(5-Bromoquinolin-8-yl)-3-(4-chlorophenyl)urea (5c):

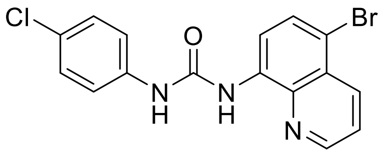

1-(4-Chlorophenyl)-3-(quinolin-8-yl)urea (**1ac**, 119 mg, 0.40 mmol) and tribromoisocyanuric acid (53 mg, 0.145 mmol) in acetonitrile (3 mL), 40 min at room temperature. After column chromatography (gradient: EtOAc/hexanes 10:90 to 50:50) 148 mg (99%) of a white solid was obtained. R_f = 0.20 (EtOAc/hexanes 30:70), mp = 228-229 °C; ^{1}H NMR (400 MHz, $CDCl_3$ + DMSO-*d6*, 8:2): δ 9.72 (d, *J* = 11.7 Hz, 2H), 8.86 (s, 1H), 8.58-8.42 (m, 2H), 7.82-7.67 (m, 1H), 7.69-7.45 (m, 3H), 7.34-7.11 (m, 2H); ^{13}C{^{1}H}NMR (100 MHz, $CDCl_3$ + DMSO-*d6*, 8:2): δ 151.5, 147.0, 137.9, 137.4, 135.1, 134.5, 129.6, 127.4, 125.9, 125.2, 121.5, 118.5, 114.2, 110.6; FTIR (neat): 3299, 3063, 2843, 1597, 1501, 1485, 1379, 1280, 1131, 885, 779, 698 cm^{-1}; MS (ESI): *m/z* 376 (M+H)$^+$; HRMS (ESI): *m/z* calcd for $C_{16}H_{12}BrClN_3O$ (M+H)$^+$: 375.9847, found: 375.9853.

Ethyl *N*-(5-chloroquinolin-8-yl)-P-ethylphosphonamidate (4d):

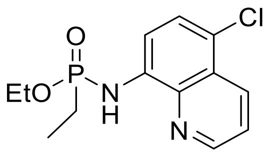

Ethyl *P*-ethyl-*N*-(quinolin-8-yl)phosphonamidate (**1ad**, 106 mg, 0.40 mmol) and trichloroisocyanuric acid (34 mg, 0.145 mmol) in acetonitrile (3 mL), 60 min at room temperature. After column chromatography (gradient: EtOAc/hexanes 10:90 to 60:40) 118 mg (99%) of a white solid was obtained. R_f = 0.22 (EtOAc/hexanes 30:70), mp = 91-93 °C; ^{1}H NMR (400 MHz, $CDCl_3$): δ 8.83 (dd, *J* = 4.2, 1.6 Hz, 1H), 8.51 (dd, *J* = 8.5, 1.6 Hz, 1H), 7.61 (d, *J* = 8.6 Hz, 1H), 7.55 (dd, *J* = 8.5, 4.2 Hz, 1H), 7.49 (d, *J* = 2.0 Hz, 2H), 4.36-4.22 (m, 1H), 4.18-4.06 (m, 1H), 2.10-1.85 (m, 2H), 1.37 (t, *J* = 7.1 Hz, 3H), 1.19 (dt, *J* = 20.3, 7.7 Hz, 3H); ^{13}C{^{1}H}NMR (100 MHz, $CDCl_3$): δ 148.4, 138.9 (d, *J* = 8.1 Hz), 137.2 (d, *J* = 2.5 Hz), 133.2, 126.9, 126.3, 122.5, 121.7, 111.5 (d, *J* = 1.1 Hz), 60.5 (d, *J* = 6.9 Hz), 20.3, 19.0, 16.3 (d, *J* = 6.5 Hz), 6.2 (d, *J* = 6.0 Hz); FTIR (neat): 3310, 3060, 2981, 1724, 1586, 1503, 1468, 1319, 1215, 1033, 948, 782, 694 cm^{-1}; MS (ESI): *m/z* 299 (M+H)$^+$; HRMS (ESI): *m/z* calcd for $C_{13}H_{17}ClN_2O_2P$ (M+H)$^+$: 299.0711, found: 299.0713.

Ethyl *N*-(5-bromoquinolin-8-yl)-P-ethylphosphonamidate (5d):

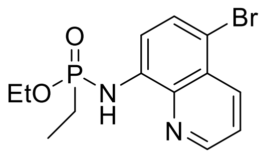

Ethyl *P*-ethyl-*N*-(quinolin-8-yl)phosphonamidate (**1ad**, 106 mg, 0.40 mmol) and tribromoisocyanuric acid (53 mg, 0.145 mmol) in acetonitrile (3 mL), 60 min at room temperature. After column chromatography (gradient: EtOAc/hexanes 10:90 to 60:40) 134 mg (98%) of a white solid was obtained. R_f = 0.22 (EtOAc/hexanes 30:70), mp = 102-103 °C; ^{1}H NMR (400 MHz, $CDCl_3$): δ 8.79 (dd, *J* = 4.2, 1.6 Hz, 1H), 8.45 (dd, *J* = 8.5, 1.6 Hz, 1H), 7.69 (d, *J* = 8.3 Hz, 1H), 7.66 (d, *J* = 8.7 Hz, 1H), 7.53 (dd, *J* = 8.5, 4.2 Hz, 1H), 7.46 (d, *J* = 8.3 Hz, 1H), 4.36-4.23 (m, 1H), 4.19-4.06 (m, 1H), 2.10-1.89 (m, 2H), 1.38 (t, *J* = 7.1 Hz, 3H), 1.19 (dt, *J* = 20.3, 7.7 Hz, 3H); ^{13}C{^{1}H}NMR (100 MHz, $CDCl_3$): δ 148.4, 139.0 (d, *J* = 8.1 Hz), 137.8 (d, *J* = 2.6 Hz), 135.7, 130.5, 127.5, 122.8, 112.2 (d, *J* = 1.2 Hz), 111.4, 60.5 (d, *J* = 6.9 Hz), 20.3, 19.0, 16.3 (d, *J* = 6.5 Hz), 6.2 (d, *J* = 6.0 Hz); FTIR (neat): 3312, 3056, 2979, 1723, 1585, 1498, 1319, 1219, 1020, 943, 816, 703 cm^{-1}; MS (ESI): *m/z* 343 $(M+H)^+$; HRMS (ESI): *m/z* calcd for $C_{13}H_{17}BrN_2O_2P$ $(M+H)^+$: 343.0206, found: 343.0211.

***N*-(7-Chloro-5-methoxyquinolin-8-yl)acetamide (4e):**

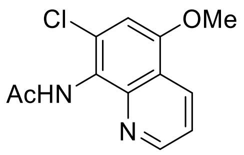

N-(5-Methoxyquinolin-8-yl)acetamide (**1ae**, 86 mg, 0.40 mmol) and trichloroisocyanuric acid (34 mg, 0.145 mmol) in acetonitrile (3 mL), 30 min at room temperature. After column chromatography (gradient: EtOAc/hexanes 10:90 to 60:40) 69 mg (69%) of a white solid was obtained. R_f = 0.30 (EtOAc/hexanes 30:70), mp = 123-124 °C; ^{1}H NMR (400 MHz, $CDCl_3$): δ 9.62 (s, 1H), 8.83-8.75 (m, 2H), 8.44 (dd, *J* = 8.5, 1.7 Hz, 1H), 7.52 (dd, *J* = 8.5, 4.2 Hz, 1H), 3.99 (s, 3H), 2.34 (s, 3H); ^{13}C{^{1}H}NMR (100 MHz, $CDCl_3$): δ 168.7, 148.3, 146.0, 137.7, 131.6, 130.9, 124.3, 123.6, 122.2, 117.9, 61.7, 25.0; FTIR (neat): 3351, 3073, 2923, 1628, 1590, 1493, 1467, 1378, 1292, 1167, 1012, 882, 835, 710 cm^{-1}; MS (ESI): *m/z* 251 $(M+H)^+$; HRMS (ESI): *m/z* calcd for $C_{12}H_{12}ClN_2O_2$ $(M+H)^+$: 251.0582, found: 251.0583.

7-Chloro-5-methoxyquinolin-8-amine (4f):

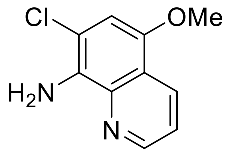

5-Methoxyquinolin-8-amine (**1af**, 70 mg, 0.40 mmol) and trichloroisocyanuric acid (34 mg, 0.145 mmol) in acetonitrile (3 mL), 30 min at room temperature. After column chromatography (gradient: EtOAc/hexanes 10:90 to 60:40) 66 mg (79%) of a

white solid was obtained. R_f = 0.25 (EtOAc/hexanes 30:70); ^{1}H NMR (400 MHz, $CDCl_3$): δ 8.80 (dd, *J* = 4.2, 1.7 Hz, 1H), 8.47 (dd, *J* = 8.4, 1.7 Hz, 1H), 7.38 (dd, *J* = 8.4, 4.2 Hz, 1H), 6.77 (s, 1H), 4.93 (s, 2H), 3.92 (s, 3H); $^{13}C\{^1H\}$NMR (100 MHz, $CDCl_3$): δ 148.9, 146.4, 139.0, 134.2, 131.0, 120.5, 120.0, 114.2, 106.6, 56.0; FTIR (neat): 3430, 3328, 2921, 1609, 1583, 1465, 1260, 1148, 1099, 977, 861, 806, 781 cm^{-1}; MS (ESI): *m/z* 209 $(M+H)^+$; HRMS (ESI): *m/z* calcd for $C_{10}H_{10}ClN_2O$ $(M+H)^+$: 209.0476, found: 209.0479.

7-Bromo-5-methoxyquinolin-8-amine (5f):

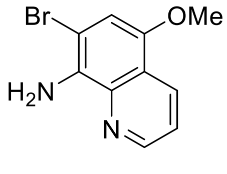

5-Methoxyquinolin-8-amine (**1af**, 70 mg, 0.40 mmol) and tribromoisocyanuric acid (53 mg, 0.145 mmol) in acetonitrile (3 mL), 15 min at room temperature. After column chromatography (gradient: EtOAc/hexanes 10:90 to 60:40) 63 mg (63%) of a white solid was obtained. R_f = 0.25 (EtOAc/hexanes 30:70); ^{1}H NMR (400 MHz, $CDCl_3$): δ 8.77 (dd, *J* = 4.2, 1.7 Hz, 1H), 8.44 (dd, *J* = 8.4, 1.7 Hz, 1H), 7.38 (dd, *J* = 8.4, 4.2 Hz, 1H), 6.88 (s, 1H), 4.99 (s, 2H), 3.90 (s, 3H); $^{13}C\{^1H\}$NMR (100 MHz, $CDCl_3$): δ 148.7, 146.4, 138.7, 135.8, 131.0, 120.6, 120.4, 108.9, 103.8, 55.9; FTIR (neat): 3317, 2923, 2853, 1640, 1590, 1493, 1397, 1283, 1167, 1059, 883, 816, 726 cm^{-1}; MS (ESI): *m/z* 253 $(M+H)^+$; HRMS (ESI): *m/z* calcd for $C_{10}H_{10}BrN_2O$ $(M+H)^+$: 252.9971, found: 252.9972.

***N*-(5-Chloro-6-methoxyquinolin-8-yl)acetamide (4g):**[18a]

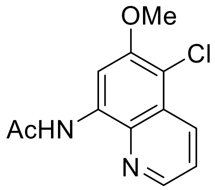

N-(6-Methoxyquinolin-8-yl)acetamide (**1ag**, 86 mg, 0.40 mmol) was treated with trichloroisocyanuric acid (34 mg, 0.145 mmol) in acetonitrile (3 mL) for 45 min at room temperature. After column chromatography (gradient: EtOAc/hexanes 20:80 to 50:50) 94 mg (94%) of a pale-yellow solid was obtained. R_f = 0.20 (EtOAc/hexanes 30:70); ^{1}H NMR (400 MHz, $CDCl_3$): δ 9.77 (s, 1H), 8.77 (s, 1H), 8.65 (dd, *J* = 4.2, 1.5 Hz, 1H), 8.47 (dd, *J* = 8.6, 1.5 Hz, 1H), 7.49 (dd, *J* = 8.6, 4.2 Hz, 1H), 4.06 (s, 3H), 2.36 (s, 3H); $^{13}C\{^1H\}$NMR (100 MHz, $CDCl_3$): δ 168.9, 153.2, 146.0, 134.5, 133.9, 132.1, 126.6, 122.7, 108.4, 104.3, 56.8, 25.2; MS (ESI): *m/z* 251 (M+H)+.

***N*-(5-Bromo-6-methoxyquinolin-8-yl)acetamide (5g):**[18a]

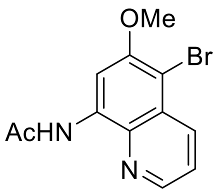

N-(6-Methoxyquinolin-8-yl)acetamide (**1ag**, 86 mg, 0.40 mmol) was treated with tribromoisocyanuric acid (53 mg, 0.145 mmol) in acetonitrile (3 mL) for 30 min at room temperature. After column chromatography (gradient: EtOAc/hexanes 20:80 to 60:40) 113 mg (96%) of a white solid was obtained. R_f = 0.22 (EtOAc/hexanes 30:70); ^{1}H NMR (400 MHz, $CDCl_3$): δ 9.78 (s, 1H), 8.75 (s, 1H), 8.61 (dd, J = 4.2, 1.5 Hz, 1H), 8.45 (dd, J = 8.6, 1.5 Hz, 1H), 7.47 (dd, J = 8.6, 4.2 Hz, 1H), 4.06 (s, 3H), 2.36 (s, 3H); ^{13}C{^{1}H}NMR (100 MHz, $CDCl_3$): δ 168.9, 154.4, 145.9, 135.2, 134.6, 134.2, 127.8, 122.9, 104.2, 99.3, 56.9, 25.2; MS (ESI): *m/z* 295 $(M+H)^+$.

***N*-(4,5-Dichloroquinolin-8-yl)acetamide (4h):**

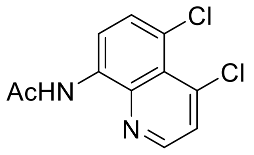

N-(4-Chloroquinolin-8-yl)acetamide (**1ah**, 88 mg, 0.40 mmol) was treated with trichloroisocyanuric acid (34 mg, 0.145 mmol) in acetonitrile (3 mL) for 6 h at room temperature. After column chromatography (gradient: EtOAc/hexanes 20:80 to 50:50) 88 mg (86%) of a pale yellow solid was obtained. R_f = 0.20 (EtOAc/hexanes 30:70); ^{1}H NMR (400 MHz, $CDCl_3$): δ 9.83 (s, 1H), 8.73 (d, J = 8.5 Hz, 1H), 8.62 (d, J = 4.7 Hz, 1H), 7.65 (d, J = 8.5 Hz, 1H), 7.59 (d, J = 4.7 Hz, 1H), 2.34 (s, 3H); ^{13}C{^{1}H}NMR (100 MHz, $CDCl_3$): δ 168.8, 147.2, 142.4, 140.6, 134.4, 131.5, 125.5, 123.4, 122.7, 116.9, 25.2; FTIR (neat): 3340, 3245, 3105, 2927, 1688, 1576, 1515, 1361, 1138, 965, 830, 695 cm^{-1}; MS (ESI): *m/z* 255 $(M+H)^+$.

***N*-(5-Bromo-4-chloroquinolin-8-yl)acetamide (5h):**

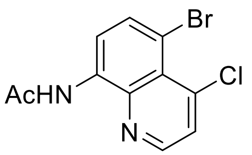

N-(4-Chloroquinolin-8-yl)acetamide (**1ah**, 88 mg, 0.40 mmol) was treated with tribromoisocyanuric acid (53 mg, 0.145 mmol) in acetonitrile (3 mL) for 6 h at room temperature. After column chromatography (gradient: EtOAc/hexanes 20:80 to 60:40) 94 mg (79%) of a white solid was obtained. R_f = 0.25 (EtOAc/hexanes 30:70); ^{1}H NMR (400 MHz, $CDCl_3$): δ 9.85 (s, 1H), 8.65 (d, J = 8.5 Hz, 1H), 8.60 (d, J = 4.6 Hz, 1H), 7.91 (d, J = 8.5 Hz, 1H), 7.61 (d, J = 4.6 Hz, 1H), 2.34 (s, 3H); ^{13}C{^{1}H}NMR (100 MHz, $CDCl_3$): δ 168.8, 147.0, 143.1, 140.5, 135.9, 135.0, 125.4, 124.1, 117.4, 109.4, 25.2; FTIR (neat): 3344, 3102, 2926, 1685, 1520, 1400, 1302, 1198, 1132, 829, 689 cm^{-1}; MS (ESI): *m/z* 301$(M+H)^+$.

***N*-(3-Bromo-5-chloroquinolin-8-yl)acetamide (4i):[18a]**

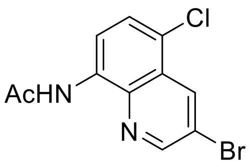

N-(3-Bromoquinolin-8-yl)acetamide (**1ai**, 106 mg, 0.40 mmol) was treated with trichloroisocyanuric acid (34 mg, 0.145 mmol) in acetonitrile (3 mL) for 6 h at room temperature. After column chromatography (gradient: EtOAc/hexanes 10:90 to 40:60) 108 mg (90%) of a pale yellow solid was obtained. R_f = 0.25 (EtOAc/hexanes 30:70); ^{1}H NMR (400 MHz, $CDCl_3$): δ 9.53 (s, 1H), 8.80 (d, *J* = 2.2 Hz, 1H), 8.71 (d, *J* = 8.5 Hz, 1H), 8.68 (d, *J* = 2.2 Hz, 1H), 7.59 (d, *J* = 8.5 Hz, 1H), 2.35 (s, 3H); $^{13}C\{^{1}H\}$NMR (100 MHz, $CDCl_3$): δ 168.7, 149.7, 136.7, 134.9, 133.9, 128.5, 126.8, 123.0, 119.0, 116.6, 25.1; MS (ESI): *m/z* 301 $(M+H)^+$.

***N*-(3,5-Dibromoquinolin-8-yl)acetamide (5i):**[18a]

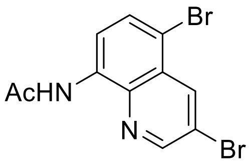

N-(3-Bromoquinolin-8-yl)acetamide (**1ai**, 106 mg, 0.40 mmol) was treated with tribromoisocyanuric acid (53 mg, 0.145 mmol) in acetonitrile (3 mL) for 6 h at room temperature. After column chromatography (gradient: EtOAc/hexanes 20:80 to 60:40) 118 mg (86%) of a pale yellow solid was obtained. R_f = 0.25 (EtOAc/hexanes 30:70); ^{1}H NMR (400 MHz, $CDCl_3$): δ 9.54 (s, 1H), 8.77 (d, *J* = 2.2 Hz, 1H), 8.65 (dd, *J* = 5.2, 3.1 Hz, 2H), 7.78 (d, *J* = 8.4 Hz, 1H), 2.35 (s, 3H); $^{13}C\{^{1}H\}$NMR (100 MHz, $CDCl_3$): δ 168.7, 149.7, 137.4, 136.9, 134.6, 132.1, 128.0, 119.3, 117.2, 112.7, 25.1; MS (ESI): *m/z* 345 $(M+H)^+$.

***N*-(5-Chloroquinolin-8-yl)methanesulfonamide (6a):**

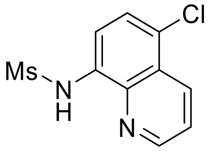

N-(Quinolin-8-yl)methanesulfonamide (**1aj**, 89 mg, 0.40 mmol) and trichloroisocyanuric acid (34 mg, 0.145 mmol) in acetonitrile (3 mL), 30 min at room temperature. After column chromatography (gradient: EtOAc/hexanes 05:95 to 50:50) 85 mg (85%) of a brown solid was obtained. R_f = 0.25 (1:3 EtOH: EtOAc in hexanes and two drops of AcOH, 20:80), R_f = 0.28 (1:3 EtOH: EtOAc in hexanes and two drops of NH_4OH, 20:80); ^{1}H NMR (400 MHz, $CDCl_3$): δ 8.89 (s, 1H), 8.87 (dd, *J* = 4.2, 1.6 Hz, 1H), 8.57 (dd, *J* = 8.5, 1.6 Hz, 1H), 7.79 (d, *J* = 8.3 Hz, 1H), 7.66-7.56 (m, 2H), 3.04 (s, 3H); $^{13}C\{^{1}H\}$NMR (100 MHz, $CDCl_3$): δ 149.4, 139.0, 133.5, 133.3, 126.8, 126.4, 125.5, 122.9, 114.9, 39.3; FTIR (neat): 3262, 3097, 2932, 1667, 1590, 1499, 1328, 1297, 1220, 978, 949, 767, 753 cm^{-1}; MS (ESI): *m/z* 257 $(M+H)^+$; HRMS (ESI): *m/z* calcd for $C_{10}H_{10}ClN_2O_2S$ $(M+H)^+$: 257.0146, found: 257.0147.

N-(5,7-Dichloroquinolin-8-yl)methanesulfonamide (7a):

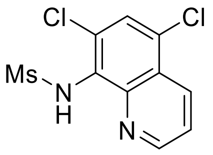

(2.5 mg, 2% yield) as a minor product obtained along with **6a**, R_f = 0.30 (1:3 EtOH/EtOAc in hexanes and two drops of AcOH, 20:80), R_f = 0.35 (1:3 EtOH: EtOAc in hexanes and two drops of NH_4OH, 20:80), ^1H NMR (400 MHz, $CDCl_3$): δ 8.98 (dd, J = 4.2, 1.6 Hz, 1H), 8.56 (dd, J = 8.5, 1.6 Hz, 1H), 7.88 (s, 1H), 7.72 (s, 1H), 7.60 (dd, J = 8.5, 4.2 Hz, 1H), 3.44 (s, 3H); $^{13}C\{^1H\}$NMR (100 MHz, $CDCl_3$): δ 151.1, 143.5, 133.6, 131.2, 129.4, 129.3, 128.3, 125.5, 122.6, 43.0; FTIR (neat): 3327, 3288, 2924, 1713, 1678, 1590, 1493, 1378, 1153, 1088, 1059, 882, 836, 726 cm^{-1}; MS (ESI): *m/z* 291 $(M+H)^+$; HRMS (ESI): *m/z* calcd for $C_{10}H_9Cl_2N_2O_2S$ $(M+H)^+$: 290.9756, found: 290.9759.

N-(5-Bromoquinolin-8-yl)methanesulfonamide (8a):[2a]

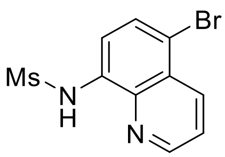

N-(Quinolin-8-yl)methanesulfonamide (**1aj**, 89 mg, 0.40 mmol) and tribromoisocyanuric acid (53 mg, 0.145 mmol) in acetonitrile (3 mL), 30 min at room temperature. After column chromatography (gradient: EtOAc/hexanes 05:95 to 50:50) 95 mg (79%) of a white solid was obtained. R_f = 0.25 (1:3 EtOH: EtOAc in hexanes and two drops of AcOH, 20:80), R_f = 0.28 (1:3 EtOH/EtOAc in hexanes and two drops of NH_4OH, 20:80); ^1H NMR (400 MHz, $CDCl_3$): δ 8.92 (s, 1H), 8.84 (dd, J = 4.2, 1.5 Hz, 1H), 8.53 (dd, J = 8.5, 1.6 Hz, 1H), 7.76 (dd, J = 22.8, 8.3 Hz, 2H), 7.61 (dd, J = 8.5, 4.2 Hz, 1H), 3.05 (s, 3H); $^{13}C\{^1H\}$NMR (100 MHz, $CDCl_3$): δ 149.4, 139.1, 136.0, 134.0, 130.4, 127.6, 123.2, 115.3 (2), 39.3; MS (ESI): *m/z* 301 $(M+H)^+$.

N-(5,7-Dibromoquinolin-8-yl)methanesulfonamide (9a):[2a]

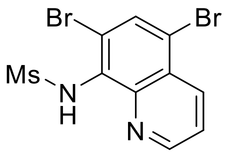

(4.5 mg, 3% yield) as a minor product obtained along with **8a**, R_f = 0.31 (1:3 EtOH/EtOAc in hexanes and two drops of AcOH, 20:80), R_f = 0.35 (1:3 EtOH: EtOAc in hexanes and two drops of NH_4OH, 20:80), ^1H NMR (400 MHz, $CDCl_3$): δ 8.95 (dd, J = 4.2, 1.6 Hz, 1H), 8.53 (dd, J = 8.5, 1.6 Hz, 1H), 8.09 (d, J = 3.3 Hz, 1H), 7.71 (s, 1H), 7.61 (dd, J = 8.5, 4.2 Hz, 1H), 3.46 (s, 3H); $^{13}C\{^1H\}$NMR (100 MHz, $CDCl_3$): δ 151.1, 143.9, 136.3, 134.2, 133.7, 127.3, 123.1, 120.4, 119.8, 43.3; MS (ESI): *m/z* 379 $(M+H)^+$.

N-(5-Chloroquinolin-8-yl)-4-methylbenzenesulfonamide (6b):

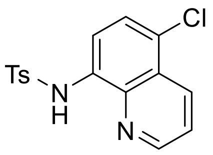

4-Methyl-*N*-(quinolin-8-yl)benzenesulfonamide (**1ak**, 119 mg, 0.40 mmol) and trichloroisocyanuric acid (34 mg, 0.145 mmol) in acetonitrile (3 mL), 20 min at room temperature. After column chromatography (gradient: EtOAc/hexanes 5:95 to 60:40) 109 mg (82%) of a white solid was obtained. R_f = 0.25 (1:3 EtOH/EtOAc in hexanes and two drops of AcOH, 20:80), R_f = 0.28 (1:3 EtOH/EtOAc in hexanes and two drops of NH_4OH, 20:80); ^{1}H NMR (400 MHz, $CDCl_3$): δ 9.18 (s, 1H), 8.80 (dd, *J* = 4.2, 1.6 Hz, 1H), 8.48 (dd, *J* = 8.5, 1.6 Hz, 1H), 7.81-7.73 (m, 3H), 7.56-7.46 (m, 2H), 7.16 (d, *J* = 8.0 Hz, 2H), 2.30 (s, 3H); ^{13}C{^{1}H}NMR (100 MHz, $CDCl_3$): δ 149.1, 143.9, 138.9, 136.1, 133.3, 133.1, 129.6, 127.2, 126.7, 126.2, 125.0, 122.7, 114.7, 21.5; MS (ESI): *m/z* 333 $(M+H)^+$.

***N*-(5,7-Dichloroquinolin-8-yl)-4-methylbenzenesulfonamide (7b):**

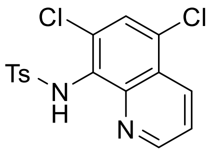

(12 mg, 8% yield) as a minor product obtained along with **6b**, R_f = 0.30 (1:3 EtOH/EtOAc in hexanes and two drops of AcOH, 20:80), R_f = 0.35 (1:3 EtOH/EtOAc in hexanes and two drops of NH_4OH, 20:80), ^{1}H NMR (400 MHz, $CDCl_3$ + DMSO-*d6*, 8:2): δ 8.86 (s, 1H), 8.74-8.56 (m, 1H), 8.47 (d, *J* = 8.5 Hz, 1H), 7.70 (s, 1H), 7.68-7.60 (m, 2H), 7.56-7.40 (m, 1H), 7.15 (d, *J* = 7.3 Hz, 2H), 2.37 (s, 3H); ^{13}C{^{1}H}NMR (100 MHz, $CDCl_3$ + DMSO-*d6*, 8:2): δ 150.6, 144.0, 143.2, 137.3, 133.0, 131.5, 130.9, 129.4, 128.8, 128.2, 127.3, 125.0, 122.1, 21.4; FTIR (neat): 3230, 3091, 3080, 1600, 1579, 1561, 1423, 1388, 1360, 1142, 1093, 897, 868, 793, 680 cm^{-1}; MS (ESI): *m/z* 367 $(M+H)^+$; HRMS (ESI): *m/z* calcd for $C_{16}H_{13}Cl_2N_2O_2S$ $(M+H)^+$: 367.0069, found: 367.0071.

***N*-(5-Bromoquinolin-8-yl)-4-methylbenzenesulfonamide (8b):**[26]

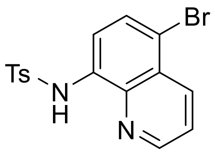

4-Methyl-*N*-(quinolin-8-yl)benzenesulfonamide (**1ak**, 119 mg, 0.40 mmol) and tribromoisocyanuric acid (53 mg, 0.145 mmol) in acetonitrile (3 mL), 30 min at room temperature. After column chromatography (gradient: EtOAc/hexanes 05:95 to 60:40) 123 mg (82%) of a white solid was obtained. R_f = 0.25 (1:3 EtOH/EtOAc in hexanes and two drops of AcOH, 20:80), R_f = 0.28 (1:3 EtOH/EtOAc in hexanes and two drops of NH_4OH, 20:80); ^{1}H NMR (400 MHz, $CDCl_3$): δ 9.21 (s, 1H), 8.77 (dd, *J* = 4.2, 1.1 Hz, 1H), 8.43 (d, *J* = 8.5 Hz, 1H), 7.79 (d, *J* = 8.3 Hz, 2H), 7.74-7.64 (m, 2H), 7.52 (dd, *J* = 8.5, 4.2 Hz, 1H), 7.17 (d, *J* = 8.3 Hz, 2H), 2.30 (s, 3H); ^{13}C{^{1}H}NMR

(100 MHz, $CDCl_3$): δ 149.1, 144.0, 139.0, 136.1, 135.8, 133.7, 130.3, 129.6, 127.4, 127.2, 123.0, 115.1, 114.8, 21.4; MS (ESI): *m/z* 377 $(M+H)^+$.

***N*-(5,7-Dibromoquinolin-8-yl)-4-methylbenzenesulfonamide (9b):**

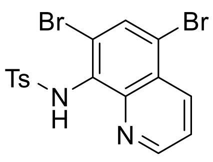

(11 mg, 6% yield) as a minor product obtained along with **8b**, R_f = 0.31 (1:3 EtOH/EtOAc in hexanes and two drops of AcOH, 20:80), R_f = 0.35 (1:3 EtOH/EtOAc in hexanes and two drops of NH_4OH, 20:80), ^{1}H NMR (400 MHz, $CDCl_3$ + DMSO-*d6*, 8:2): δ 9.35 (s, 1H), 8.51 (dd, *J* = 4.0, 1.2 Hz, 1H), 8.42 (dd, *J* = 8.5, 1.1 Hz, 1H), 8.05 (s, 1H), 7.71 (s, 1H), 7.58 (d, *J* = 8.2 Hz, 1H), 7.48 (dd, *J* = 8.5, 4.2 Hz, 1H), 7.15 (d, *J* = 8.0 Hz, 2H), 2.37 (s, 3H); ^{13}C{^{1}H}NMR (100 MHz, $CDCl_3$ + DMSO-*d6*, 8:2): δ 150.5, 144.8, 142.8, 137.8, 135.4, 133.9, 133.5, 128.7, 127.2, 126.8, 123.4, 122.5, 120.1, 21.3; FTIR (neat): 3227, 2983, 2917, 1695, 1574, 1558, 1480, 1332, 1276, 1072, 881, 789, 677 cm^{-1}; MS (ESI): *m/z* 455 $(M+H)^+$; HRMS (ESI): *m/z* calcd for $C_{16}H_{13}Br_2N_2O_2S$ $(M+H)^+$: 454.9059, found: 454.9060.

***N*-(5-Chloroquinolin-8-yl)benzenesulfonamide (6c):**

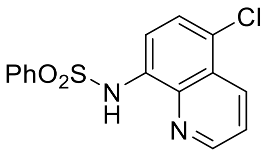

N-(Quinolin-8-yl)benzenesulfonamide (**1al**, 114 mg, 0.40 mmol) and trichloroisocyanuric acid (34 mg, 0.145 mmol) in acetonitrile (3 mL), 30 min at room temperature. After column chromatography (gradient: EtOAc/hexanes 05:95 to 60:40) 102 mg (80%) of a white solid was obtained. R_f = 0.25 (1:3 EtOH/EtOAc in hexanes and two drops of AcOH, 20:80), R_f = 0.28 (1:3 EtOH/EtOAc in hexanes and two drops of NH_4OH, 20:80); ^{1}H NMR (400 MHz, $CDCl_3$): δ 9.19 (s, 1H), 8.79 (dd, *J* = 4.2, 1.6 Hz, 1H), 8.46 (dd, *J* = 8.5, 1.6 Hz, 1H), 7.93-7.87 (m, 2H), 7.77 (d, *J* = 8.3 Hz, 1H), 7.54-7.48 (m, 2H), 7.48-7.42 (m, 1H), 7.40-7.34 (m, 2H); ^{13}C{^{1}H}NMR (100 MHz, $CDCl_3$): δ 149.2, 139.0, 138.9, 133.3, 133.0, 132.9, 128.9, 127.1, 126.6, 126.1, 125.2, 122.7, 114.9; FTIR (neat): 3254, 3098, 3063, 1613, 1589, 1499, 1463, 1413, 1364, 1302, 1157, 952, 845, 717, 685 cm^{-1}; MS (ESI): *m/z* 319 $(M+H)^+$; HRMS (ESI): *m/z* calcd for $C_{15}H_{12}ClN_2O_2S$ $(M+H)^+$: 319.0303, found: 319.0305.

***N*-(5,7-Dichloroquinolin-8-yl)benzenesulfonamide (7c):**

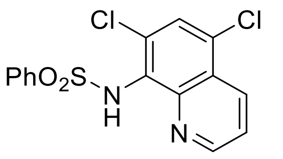

(10 mg, 7% yield) as a minor product obtained along with **6c**, R_f = 0.30 (1:3 EtOH/EtOAc in hexanes and two drops of AcOH, 20:80), R_f = 0.35 (1:3 EtOH/EtOAc in hexanes and two drops of NH_4OH, 20:80), ^1H NMR (400 MHz, $CDCl_3$): δ 8.64 (dd, *J* = 4.2, 1.6 Hz, 1H), 8.46 (dd, *J* = 8.5, 1.6 Hz, 1H), 8.17 (s, 1H), 7.85-7.80 (m, 2H), 7.69 (s, 1H), 7.52-7.42 (m, 2H), 7.40-7.31 (m, 2H); $^{13}C\{^1H\}$NMR (100 MHz, $CDCl_3$): δ 150.6, 143.3, 139.9, 133.3, 132.8, 130.9, 130.3, 129.3, 128.6, 128.5, 127.6, 125.1, 122.3; FTIR (neat): 3313, 3013, 2967, 1646, 1590, 1513, 1411, 1396, 1302, 1279, 1008, 899, 764 cm^{-1}; MS (ESI): *m/z* 353 $(M+H)^+$; HRMS (ESI): *m/z* calcd for $C_{15}H_{11}Cl_2O_2N_2S$ $(M+H)^+$: 352.9913, found: 352.9919.

***N*-(5-Bromoquinolin-8-yl)benzenesulfonamide (8c):**[2a]

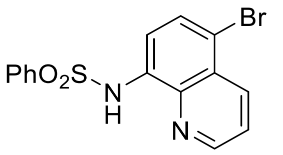

N-(Quinolin-8-yl)benzenesulfonamide (**1al**, 114 mg, 0.40 mmol) and tribromoisocyanuric acid (53 mg, 0.145 mmol) in acetonitrile (3 mL), 30 min at room temperature. After column chromatography (gradient: EtOAc/hexanes 5:95 to 60:40) 117 mg (81%) of a white solid was obtained. R_f = 0.25 (1:3 EtOH/EtOAc in hexanes and two drops of AcOH, 20:80), R_f = 0.28 (1:3 EtOH/EtOAc in hexanes and two drops of NH_4OH, 20:80); ^1H NMR (400 MHz, $CDCl_3$): δ 9.23 (s, 1H), 8.77 (dd, *J* = 4.2, 1.5 Hz, 1H), 8.43 (dd, *J* = 8.5, 1.5 Hz, 1H), 7.94-7.88 (m, 2H), 7.75-7.66 (m, 2H), 7.52 (dd, *J* = 8.5, 4.2 Hz, 1H), 7.49-7.43 (m, 1H), 7.41-7.35 (m, 2H); $^{13}C\{^1H\}$NMR (100 MHz, $CDCl_3$): δ 149.2, 139.0, 135.9, 133.6, 133.1, 130.3, 129.0, 127.4 (2), 127.1, 123.0, 115.4, 115.1; MS (ESI): *m/z* 363 $(M+H)^+$.

***N*-(5,7-Dibromoquinolin-8-yl)benzenesulfonamide (9c):**[2a]

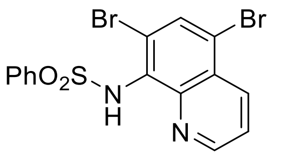

(9 mg, 5% yield) as a minor product obtained along with **8c**, R_f = 0.31 (1:3 EtOH/EtOAc in hexanes and two drops of AcOH, 20:80), R_f = 0.35 (1:3 EtOH/EtOAc in hexanes and two drops of NH_4OH, 20:80), ^1H NMR (400 MHz, $CDCl_3$ + DMSO-*d6*, 8:2): δ 9.26 (s, 1H), 8.43 (dd, *J* = 24.1, 6.2 Hz, 2H), 8.08 (s, 1H), 7.71 (d, *J* = 8.1 Hz, 2H), 7.55-7.40 (m, 2H), 7.35 (t, *J* = 7.4 Hz, 2H); $^{13}C\{^1H\}$NMR (100 MHz, $CDCl_3$ + DMSO-d6, 8:2): δ 144.1, 140.0, 134.9, 133.5, 132.8, 131.8, 127.7 (2), 126.7, 126.3, 122.8, 122.0, 119.7; MS (ESI): *m/z* 441 $(M+H)^+$.

***N*-Benzyl-5-chloroquinolin-8-amine (6d):**

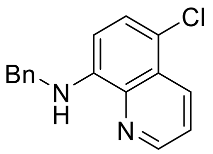

N-Benzylquinolin-8-amine (**1am**, 94 mg, 0.40 mmol) and trichloroisocyanuric acid (34 mg, 0.145 mmol) in acetonitrile (3 mL), 30 min at room temperature. After column chromatography (gradient: EtOAc/hexanes 05:90 to 40:60) 65 mg (61%) of a pale yellow oil was obtained. R_f = 0.30 (EtOAc/hexanes 20:80); ^{1}H NMR (400 MHz, $CDCl_3$): δ 8.75 (dd, *J* = 4.2, 1.6 Hz, 1H), 8.47 (dd, *J* = 8.5, 1.6 Hz, 1H), 7.50 (dd, *J* = 8.5, 4.2 Hz, 1H), 7.45-7.32 (m, 5H), 7.31-7.26 (m, 1H), 6.65 (s, 1H), 6.53 (d, *J* = 8.3 Hz, 1H), 4.55 (d, *J* = 5.8 Hz, 2H); ^{13}C{^{1}H}NMR (100 MHz, $CDCl_3$): δ 147.3, 143.8, 138.8, 138.6, 133.0, 128.7, 127.6, 127.3, 127.3, 126.3, 122.2, 116.5, 104.8, 47.6; FTIR (neat): 3411, 3067, 2812, 1608, 1576, 1517, 1472, 1200, 988, 936, 783, 697 cm^{-1}; MS (ESI): *m/z* 269 $(M+H)^+$; HRMS (ESI): *m/z* calcd for $C_{16}H_{14}ClN_2$ $(M+H)^+$: 269.0840, found: 269.0842.

***N*-Benzyl-5,7-dichloroquinolin-8-amine (7d):**

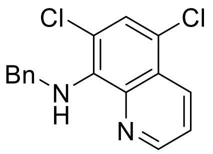

(16 mg, 13% yield) as a minor product obtained along with **6d**, R_f = 0.42 (EtOAc/hexanes 20:80), ^{1}H NMR (400 MHz, $CDCl_3$): δ 8.79 (dd, *J* = 4.2, 1.6 Hz, 1H), 8.44 (dd, *J* = 8.5, 1.6 Hz, 1H), 7.52-7.46 (m, 2H), 7.39 (dd, *J* = 7.8, 1.0 Hz, 2H), 7.33-7.20 (m, 3H), 6.61 (s, 1H), 4.88 (s, 2H); ^{13}C{^{1}H}NMR (100 MHz, $CDCl_3$): δ 148.5, 141.6, 141.3, 140.2, 133.1, 129.3, 128.5, 127.7, 127.1, 125.2, 122.0, 119.7, 116.9 50.5; FTIR (neat): 3310, 3063, 2861, 1601, 1580, 1492, 1361, 1350, 1074, 1048, 936, 784, 723 cm^{-1}; MS (ESI): *m/z* 303 $(M+H)^+$; HRMS (ESI): *m/z* calcd for $C_{16}H_{13}Cl_2N_2$ $(M+H)^+$: 303.0450, found: 303.0451.

***N*-Benzyl-5-bromoquinolin-8-amine (8d):**

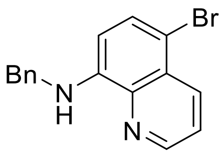

N-Benzylquinolin-8-amine (**1am**, 94 mg, 0.40 mmol) and tribromoisocyanuric acid (53 mg, 0.145 mmol) in acetonitrile (3 mL), 30 min at room temperature. After column chromatography (gradient: EtOAc/hexanes 05:95 to 50:50) 80 mg (64%) of a brown solid was obtained. R_f = 0.42 (EtOAc/hexanes 20:80), mp = 61-63 °C; ^{1}H NMR (400 MHz, $CDCl_3$): δ 8.71 (dd, *J* = 4.2, 1.6 Hz, 1H), 8.41 (dd, *J* = 8.5, 1.6 Hz, 1H), 7.54 (d, *J* = 8.3 Hz, 1H), 7.48 (dd, *J* = 8.5, 4.2 Hz, 1H), 7.43-7.38 (m, 2H), 7.37-7.31 (m, 2H), 7.31-7.23 (m, 1H), 6.69 (t, *J* = 5.1 Hz, 1H), 6.48 (d, *J* = 8.3 Hz, 1H), 4.53 (d, *J* = 5.8 Hz, 2H); ^{13}C{^{1}H}NMR (100 MHz, $CDCl_3$): δ 147.2, 144.4, 138.8, 138.7, 135.4, 131.1,

128.6, 127.5, 127.3, 127.2, 122.4, 105.7, 105.5, 47.5; FTIR (neat): 3331, 3062, 2895, 1559, 1487, 1453, 1346, 1209, 1104, 976, 912, 726, 695 cm^{-1}; MS (ESI): *m/z* 313 $(M+H)^+$; HRMS (ESI): *m/z* calcd for $C_{16}H_{14}BrN_2$ $(M+H)^+$: 313.0335, found: 313.0336.

***N*-Benzyl-5,7-dibromoquinolin-8-amine (9d):**

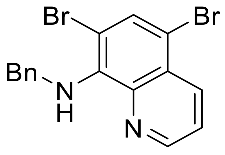

(19 mg, 12% yield) as a minor product obtained along with **8d**, R_f = 0.42 (EtOAc/hexanes 20:80), ^{1}H NMR (400 MHz, $CDCl_3$): δ 8.76 (dd, *J* = 4.2, 1.6 Hz, 1H), 8.40 (dd, *J* = 8.5, 1.6 Hz, 1H), 7.84 (s, 1H), 7.50 (dd, *J* = 8.5, 4.2 Hz, 1H), 7.42-7.37 (m, 2H), 7.33-7.27 (m, 2H), 7.26-7.20 (m, 1H), 6.55 (s, 1H), 4.89 (s, 2H); $^{13}C\{^1H\}$NMR (100 MHz, $CDCl_3$): δ 148.4, 144.0, 141.7, 140.0, 135.7, 135.1, 128.5, 127.8, 127.1, 126.9, 122.5, 109.6, 106.7, 51.0; FTIR (neat): 3405, 3029, 2846, 1605, 1574, 1516, 1453, 1284, 1111, 1076, 1028, 920, 805, 781, 696 cm^{-1}; MS (ESI): *m/z* 390 $(M+H)^+$; HRMS (ESI): *m/z* calcd for $C_{16}H_{13}Br_2N_2$ $(M+H)^+$: 390.9440, found: 390.9442.

***N,N*-Dibenzyl-5-chloroquinolin-8-amine (6e):**

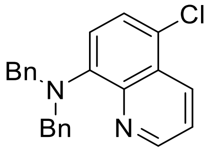

N,N-Dibenzylquinolin-8-amine (**1an**, 130 mg, 0.40 mmol) and trichloroisocyanuric acid (34 mg, 0.145 mmol) in acetonitrile (3 mL), 40 min at room temperature. After column chromatography (gradient: EtOAc/hexanes 05:95 to 50:50) 103 mg (72%) of a pale yellow solid was obtained. R_f = 0.35 (EtOAc/hexanes 20:80), mp = 76-78 °C; ^{1}H NMR (400 MHz, $CDCl_3$): δ 8.93 (dd, *J* = 4.1, 1.6 Hz, 1H), 8.03 (dd, *J* = 8.2, 1.8 Hz, 1H), 7.42 (s, 2H), 7.41-7.37 (m, 4H), 7.33 (dd, *J* = 8.2, 4.2 Hz, 1H), 7.24-7.10 (m, 6H), 4.63 (s, 4H); $^{13}C\{^1H\}$NMR (100 MHz, $CDCl_3$): δ 148.8, 148.1, 145.0, 139.8, 136.2, 134.3, 128.9, 128.6, 128.2, 127.8, 126.6, 125.2, 120.7, 57.6; FTIR (neat): 3061, 3025, 2842, 1599, 1576, 1449, 1392, 1252, 1044, 899, 874, 786, 742 cm^{-1}; MS (ESI): *m/z* 359 $(M+H)^+$; HRMS (ESI): *m/z* calcd for $C_{23}H_{20}ClN_2$ $(M+H)^+$: 359.1310, found: 359.1309.

***N,N*-Dibenzyl-5,7-dichloroquinolin-8-amine (7e):**

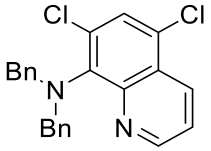

(20 mg, 13% yield) as a minor product obtained along with **6e**, R_f = 0.40 (EtOAc/hexanes 20:80), ^{1}H NMR (400 MHz, $CDCl_3$): δ 8.97 (dd, *J* = 4.1, 1.7 Hz, 1H), 8.46 (dd, *J* = 8.5, 1.7 Hz, 1H), 7.56 (s, 1H), 7.46 (dd, *J* = 8.5, 4.1 Hz, 1H), 7.39-7.33 (m, 4H), 7.24-7.12 (m, 6H), 4.60 (s, 4H); $^{13}C\{^1H\}$NMR (100 MHz, $CDCl_3$): δ 149.4, 148.2, 144.4, 139.5,

133.8, 133.3, 128.9, 128.3, 127.9, 127.5, 126.7, 126.0, 121.4, 57.6; FTIR (neat): 3085, 3025, 2866, 1604, 1586, 1487, 1302, 1117, 1039, 974, 825, 741, 696 cm^{-1}; MS (ESI): *m/z* 393 $(M+H)^+$; HRMS (ESI): *m/z* calcd for $C_{23}H_{19}Cl_2N_2$ $(M+H)^+$: 393.0920, found: 393.0920.

***N,N*-Dibenzyl-5-bromoquinolin-8-amine (8e):**

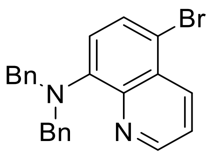

N,N-Dibenzylquinolin-8-amine (**1an**, 130 mg, 0.40 mmol) and tribromoisocyanuric acid (53 mg, 0.145 mmol) in acetonitrile (3 mL), 60 min at room temperature. After column chromatography (gradient: EtOAc/hexanes 5:95 to 50:50) 138 mg (86%) of a white solid was obtained as a sole product. R_f = 0.35 (EtOAc/hexanes 20:80), mp = 107-109 °C; ^{1}H NMR (400 MHz, $CDCl_3$): δ 8.85 (dd, *J* = 4.1, 1.6 Hz, 1H), 8.41 (dd, *J* = 8.5, 1.5 Hz, 1H), 7.45-7.38 (m, 2H), 7.24-7.08 (m, 10H), 6.68 (d, *J* = 8.3 Hz, 1H), 4.61 (s, 4H); $^{13}C\{^1H\}$NMR (100 MHz, $CDCl_3$): δ 148.0, 147.0, 143.7, 138.4, 135.8, 130.0, 128.5, 128.3, 128.2, 126.9, 121.9, 119.0, 112.8, 56.7; FTIR (neat): 3059, 3025, 2808, 1643, 1581, 1492, 1364, 1215, 1070, 891, 808, 730 cm^{-1}; MS (ESI): *m/z* 403 $(M+H)^+$; HRMS (ESI): *m/z* calcd for $C_{23}H_{20}BrN_2$ $(M+H)^+$: 403.0804, found: 403.0806.

Synthesis of *N,N*-dibenzyl-5,7-dibromoquinolin-8-amine (9e):

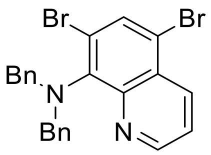

A 10 mL round bottom flask equipped with a magnetic stir bar was charged with *N,N*-dibenzylquinolin-8-amine (**1an**, 130 mg, 0.40 mmol) and tribromoisocyanuric acid (106 mg, 0.29 mmol) in acetonitrile (5 mL), 15 h at room temperature. The reaction mixture was then evaporated, and the crude residue was purified by flash chromatography (EtOAc: hexanes, 5:95 to 50:50) to yield the *N,N*-dibenzyl-5,7-dibromoquinolin-8-amine in 154 mg (80%) of a brown solid. R_f = 0.40 (EtOAc/hexanes 20:80), ^{1}H NMR (400 MHz, $CDCl_3$): δ 8.93 (dd, *J* = 4.1, 1.7 Hz, 1H), 8.43 (dd, *J* = 8.5, 1.7 Hz, 1H), 7.94 (s, 1H), 7.48 (dd, *J* = 8.5, 4.1 Hz, 1H), 7.37 (dd, *J* = 7.9, 1.5 Hz, 4H), 7.23-7.13 (m, 6H), 4.60 (s, 4H); $^{13}C\{^1H\}$NMR (100 MHz, $CDCl_3$): δ 149.2, 148.4, 146.7, 139.3, 136.0, 134.2, 129.2, 127.8, 127.8, 126.7, 125.7, 121.9, 118.2, 57.8; FTIR (neat): 3059, 3025, 2842, 1699, 1662, 1564, 1444, 1391, 1340, 1196, 1118, 1042, 868, 742, 696 cm^{-1}; MS (ESI): *m/z* 483 $(M+H)^+$; HRMS (ESI): *m/z* calcd for $C_{23}H_{19}Br_2N_2$ $(M+H)^+$: 482.9889, found: 482.9892.

5-Chloroquinolin-8-amine (6f):[23]

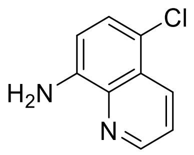

Quinolin-8-amine (**1ao**, 58 mg, 0.40 mmol) and trichloroisocyanuric acid (34 mg, 0.145 mmol) in acetonitrile (3 mL), 45 min at room temperature. After column chromatography (gradient: EtOAc/hexanes 05:95 to 50:50) 51 mg (71%) of a white solid was obtained. R_f = 0.30 (EtOAc/hexanes 20:80); ^{1}H NMR (400 MHz, $CDCl_3$): δ 8.80 (dd, J = 4.2, 1.6 Hz, 1H), 8.47 (dd, J = 8.5, 1.6 Hz, 1H), 7.49 (dd, J = 8.5, 4.2 Hz, 1H), 7.39 (d, J = 8.1 Hz, 1H), 6.84 (d, J = 8.1 Hz, 1H), 5.01 (s, 2H); ^{13}C{^{1}H}NMR (100 MHz, $CDCl_3$): δ 147.8, 143.3, 138.7, 132.9, 127.2, 126.5, 122.1, 118.0, 109.5; MS (ESI): *m/z* 179 $(M+H)^+$.

5,7-Dichloroquinolin-8-amine (7f):[27]

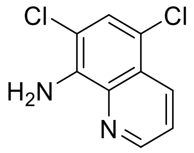

(9.4 mg, 11% yield) as a minor product obtained along with **6f**, R_f = 0.42 (EtOAc/hexanes 20:80); ^{1}H NMR (400 MHz, $CDCl_3$): δ 8.80 (dd, J = 4.2, 1.6 Hz, 1H), 8.43 (dd, J = 8.5, 1.6 Hz, 1H), 7.53-7.43 (m, 2H), 5.35 (s, 2H); ^{13}C{^{1}H}NMR (100 MHz, $CDCl_3$): δ 148.5, 140.0, 138.4, 133.0, 127.5, 125.2, 122.0, 117.6, 113.4; FTIR (neat): 3418, 3289, 3034, 1728, 1609, 1580, 1459, 1367, 1039, 943, 862, 806, 784 cm^{-1}; MS (ESI): *m/z* 213 $(M+H)^+$; HRMS (ESI): *m/z* calcd for $C_9H_7Cl_2N_2$ $(M+H)^+$: 212.9981, found: 212.9984.

5-Bromoquinolin-8-amine (8f):[25]

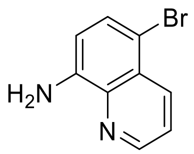

Quinolin-8-amine (**1ao**, 58 mg, 0.40 mmol) and tribromoisocyanuric acid (53 mg, 0.145 mmol) in acetonitrile (3 mL), 45 min at room temperature. After column chromatography (gradient: EtOAc/hexanes 5:95 to 50:50) 64 mg (72%) of a white solid was obtained. R_f = 0.32 (EtOAc/hexanes 20:80); ^{1}H NMR (400 MHz, $CDCl_3$): δ 8.75 (dd, J = 4.2, 1.6 Hz, 1H), 8.41 (dd, J = 8.5, 1.6 Hz, 1H), 7.55 (d, J = 8.1 Hz, 1H), 7.46 (dd, J = 8.5, 4.2 Hz, 1H), 6.78 (d, J = 8.1 Hz, 1H), 5.04 (s, 2H); ^{13}C{^{1}H}NMR (100 MHz, $CDCl_3$): δ 147.7, 143.9, 138.9, 135.3, 130. 127.7, 122.4, 110.1, 107.3; MS (ESI): *m/z* 223 $(M+H)^+$.

5,7-Dibromoquinolin-8-amine (9f):[2a]

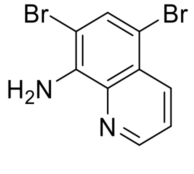

(8.4 mg, 7% yield) as a minor product obtained along with **8f**, R_f = 0.45 (EtOAc/hexanes 20:80), ^{1}H NMR (400 MHz, $CDCl_3$): δ 8.76 (dd, J = 4.2, 1.6 Hz, 1H), 8.38 (dd, J = 8.5,

1.6 Hz, 1H), 7.79 (s, 1H), 7.49 (dd, *J* = 8.5, 4.2 Hz, 1H), 5.45 (s, 2H); $^{13}C\{^1H\}$NMR (100 MHz, $CDCl_3$): δ 148.4, 142.1, 138.4, 135.6, 133.1, 126.7, 122.5, 106.8, 103.1; MS (ESI): *m/z* 301 $(M+H)^+$.

5-Chloroquinolin-8-ol (6g):[28]

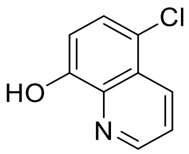

Quinolin-8-ol (**1ap**, 58 mg, 0.40 mmol) and trichloroisocyanuric acid (34 mg, 0.145 mmol) in acetonitrile (3 mL), 60 min at room temperature. After column chromatography (gradient: EtOAc/hexanes 2:98 to 30:70) 51 mg (71%) of a brown solid was obtained. R_f = 0.25 (3:1 EtOAc/EtOH in hexanes 20:80); ^1H NMR (400 MHz, $CDCl_3$): δ 8.83 (dd, *J* = 4.2, 1.5 Hz, 1H), 8.52 (dd, *J* = 8.5, 1.5 Hz, 1H), 8.27 (s, 1H), 7.56 (dd, *J* = 8.5, 4.2 Hz, 1H), 7.52 (d, *J* = 8.2 Hz, 1H), 7.11 (d, *J* = 8.2 Hz, 1H); $^{13}C\{^1H\}$NMR (100 MHz, $CDCl_3$): δ 151.3, 148.4, 138.6, 133.4, 127.5, 126.3, 122.6, 120.4, 109.9; MS (ESI): *m/z* 180 $(M+H)^+$.

5,7-Dichloroquinolin-8-ol (7g):[29]

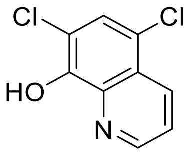

(11 mg, 13% yield) as a minor product obtained along with **6g**, R_f = 0.26 (3:1 EtOAc/EtOH in hexanes 20:80), ^1H NMR (400 MHz, $CDCl_3$): δ 8.86 (dd, *J* = 4.2, 1.4 Hz, 1H), 8.50 (dd, *J* = 8.5, 1.5 Hz, 1H), 7.60 (s, 1H), 7.58 (dd, *J* = 8.5, 4.3 Hz, 1H); $^{13}C\{^1H\}$NMR (100 MHz, $CDCl_3$): δ 149.3, 147.6, 138.6, 133.6, 128.3, 125.0, 122.6, 120.8, 115.2; MS (ESI): *m/z* 214 $(M+H)^+$.

5-Bromoquinolin-8-ol (8g):[30]

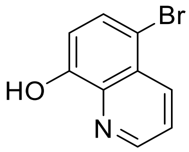

Quinolin-8-ol (**1ap**, 58 mg, 0.40 mmol) and tribromoisocyanuric acid (53 mg, 0.145 mmol) in acetonitrile (3 mL), 30 min at room temperature. After column chromatography (gradient: EtOAc/hexanes 02:98 to 40:60) 64 mg (72%) of a white solid was obtained. R_f = 0.26 (3:1 EtOAc/EtOH in hexanes 20:80); ^1H NMR (400 MHz, $CDCl_3$): δ 8.83 (dd, *J* = 4.2, 1.5 Hz, 1H), 8.53 (dd, *J* = 8.5, 1.5 Hz, 1H), 8.24 (s, 1H), 7.57 (dd, *J* = 8.5, 4.2 Hz, 1H), 7.52 (d, *J* = 8.2 Hz, 1H), 7.11 (d, *J* = 8.2 Hz, 1H); $^{13}C\{^1H\}$NMR (100 MHz, $CDCl_3$): δ 151.3, 148.4, 138.8, 133.4, 127.5, 126.3, 122.6, 120.4, 109.9; MS (ESI): *m/z* 224 $(M+H)^+$.

5,7-Dibromoquinolin-8-ol (9g):[31]

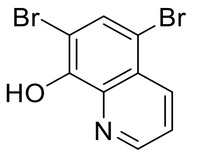

(13 mg, 11% yield) as a minor product obtained along with **8g**, R_f = 0.27 (3:1 EtOAc/EtOH in hexanes 20:80), ^{1}H NMR (400 MHz, $CDCl_3$): δ 8.86 (dd, *J* = 4.2, 1.5 Hz, 1H), 8.50 (dd, *J* = 8.5, 1.5 Hz, 1H), 7.60 (s, 1H), 7.58 (dd, *J* = 8.5, 4.3 Hz, 1H); ^{13}C{^{1}H}NMR (100 MHz, $CDCl_3$): δ 149.3, 147.6, 138.6, 133.6, 128.3, 125.0, 122.5, 120.8, 115.2; MS (ESI): *m/z* 302 $(M+H)^+$.

Ethyl 2-((5-chloroquinolin-8-yl)oxy)acetate (6h):

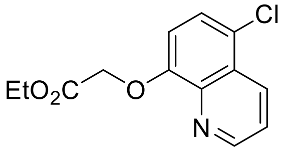

Ethyl 2-(quinolin-8-yloxy)acetate (**1aq**, 141 mg, 0.40 mmol) and trichloroisocyanuric acid (34 mg, 0.145 mmol) in acetonitrile (3 mL), 6 h at room temperature. After column chromatography (gradient: EtOAc/hexanes 5:95 to 50:50) 80 mg (75%) of a white solid was obtained. R_f = 0.30 (EtOAc/hexanes 20:80), mp = 133-135 ^{o}C; ^{1}H NMR (400 MHz, $CDCl_3$): δ 8.98 (dd, *J* = 4.2, 1.6 Hz, 1H), 8.49 (dd, *J* = 8.6, 1.6 Hz, 1H), 7.53 (dd, *J* = 8.6, 4.2 Hz, 1H), 7.48 (d, *J* = 8.4 Hz, 1H), 6.89 (d, *J* = 8.4 Hz, 1H), 4.96 (s, 2H), 4.27 (q, *J* = 7.1 Hz, 2H), 1.27 (t, *J* = 7.1 Hz, 3H); ^{13}C{^{1}H}NMR (100 MHz, $CDCl_3$): δ 168.2, 152.6, 149.7, 140.4, 132.7, 126.9, 125.8, 123.2, 122.3, 109.3, 66.0, 61.2, 13.9; FTIR (neat): 2990, 2898, 1764, 1613, 1590, 1498, 1409, 1197, 1109, 934, 803, 759 cm^{-1}; MS (ESI): *m/z* 266 $(M+H)^+$; HRMS (ESI): *m/z* calcd for $C_{13}H_{13}ClNO_3$ $(M+H)^+$: 266.0578, found: 266.0584.

Ethyl 2-((5-bromoquinolin-8-yl)oxy)acetate (8h):

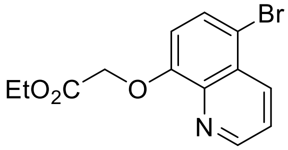

Ethyl 2-(quinolin-8-yloxy)acetate (**1aq**, 141 mg, 0.40 mmol) and tribromoisocyanuric acid (53 mg, 0.145 mmol) in acetonitrile (3 mL), 6 h at room temperature. After column chromatography (gradient: EtOAc/hexanes 5:95 to 50:50) 97 mg (78%) of a white solid was obtained. R_f = 0.31 (EtOAc/hexanes 20:80), mp = 122-124 ^{o}C; ^{1}H NMR (400 MHz, $CDCl_3$): δ 8.97 (dd, *J* = 4.2, 1.6 Hz, 1H), 8.47 (dd, *J* = 8.6, 1.6 Hz, 1H), 7.68 (d, *J* = 8.4 Hz, 1H), 7.54 (dd, *J* = 8.6, 4.2 Hz, 1H), 6.86 (d, *J* = 8.4 Hz, 1H), 4.96 (s, 2H), 4.27 (q, *J* = 7.1 Hz, 2H), 1.27 (t, *J* = 7.1 Hz, 3H); ^{13}C{^{1}H}NMR (100 MHz, $CDCl_3$): δ 168.2, 153.3, 149.8, 140.7, 135.4, 129.5, 128.2, 122.7, 113.1, 109.9, 66.1, 61.3, 14.0; FTIR (neat): 3071, 2985, 2933, 1765, 1609, 1588, 1497,

1433, 1359, 1198, 1015, 866, 781, 754 cm^{-1}; MS (ESI): *m/z* 312 (M+H)$^+$; HRMS (ESI): *m/z* calcd for $C_{13}H_{13}BrNO_3$ (M+H)$^+$: 312.0053, found: 312.0054.

***N*-(5-Iodoquinolin-8-yl)acetamide (10a):**[21]

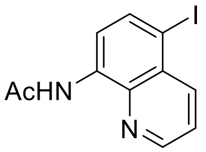

N-(Quinolin-8-yl)acetamide (**1a**, 75 mg, 0.40 mmol) and triiodoisocyanuric acid (74 mg, 0.145 mmol) in acetonitrile (3 mL), 90 min at room temperature. After column chromatography (gradient: EtOAc/hexanes 10:90 to 40:60) 120 mg (96%) of a white solid was obtained. R_f = 0.25 (EtOAc/hexanes 30:70); ^{1}H NMR (400 MHz, $CDCl_3$): δ 9.79 (s, 1H), 8.76 (dd, *J* = 4.2, 1.5 Hz, 1H), 8.53 (d, *J* = 8.3 Hz, 1H), 8.36 (dd, *J* = 8.5, 1.6 Hz, 1H), 8.06 (d, *J* = 8.3 Hz, 1H), 7.53 (dd, *J* = 8.5, 4.2 Hz, 1H), 2.35 (s, 3H); ^{13}C{^{1}H}NMR (100 MHz, $CDCl_3$): δ 168.8, 148.6, 140.8, 138.7, 138.2, 135.3, 129.5, 123.1, 117.8, 89.2, 25.2; MS (ESI): *m/z* 313 (M+H)$^+$.

***N*-(5-Iodoquinolin-8-yl)pivalamide (10b):**[21]

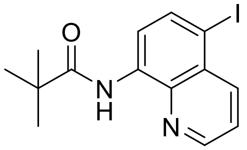

N-(Quinolin-8-yl)pivalamide (**1c**, 91 mg, 0.40 mmol) and triiodoisocyanuric acid (74 mg, 0.145 mmol) in acetonitrile (3 mL), 60 min at room temperature. After column chromatography (gradient: EtOAc/hexanes 5:95 to 30:70) 144 mg (97%) of a white solid was obtained. R_f = 0.35 (EtOAc/hexanes 20:80); ^{1}H NMR (400 MHz, $CDCl_3$): δ 10.24 (s, 1H), 8.75 (dd, *J* = 4.2, 1.6 Hz, 1H), 8.55 (d, *J* = 8.3 Hz, 1H), 8.29 (dd, *J* = 8.5, 1.6 Hz, 1H), 8.03 (d, *J* = 8.3 Hz, 1H), 7.48 (dd, *J* = 8.5, 4.2 Hz, 1H), 1.43 (s, 9H); ^{13}C{^{1}H}NMR (100 MHz, $CDCl_3$): δ 177.1, 148.6, 140.5, 139.1, 138.1, 135.4, 129.3, 122.9, 117.5, 88.9, 40.3, 27.6; MS (ESI): *m/z* 371 (M+H)$^+$; HRMS (ESI): *m/z* calcd for $C_{14}H_{16}IO_2N_2$ (M+H)$^+$: 371.0251, found: 371.0253.

***N*-(5-Iodoquinolin-8-yl)benzamide (10c):**[21]

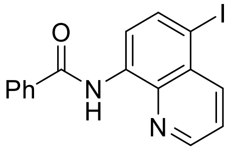

N-(Quinolin-8-yl)benzamide (**1n**, 99 mg, 0.40 mmol) and triiodoisocyanuric acid (74 mg, 0.145 mmol) in acetonitrile (3 mL), 120 min at room temperature. After column chromatography (gradient: EtOAc/hexanes 10:90 to 50:50) 145 mg (97%) of a white solid was obtained. R_f = 0.40 (EtOAc/hexanes 20:80); ^{1}H NMR (400 MHz, $CDCl_3$): δ 10.74 (s, 1H), 8.81 (dd, *J* = 4.2, 1.5 Hz, 1H), 8.71 (d, *J* = 8.3 Hz, 1H), 8.38 (dd, *J* = 8.5, 1.5 Hz, 1H), 8.12 (d, *J* = 8.3 Hz, 1H),

8.09-8.04 (m, 2H), 7.65-7.50 (m, 4H); $^{13}C\{^1H\}$NMR (100 MHz, $CDCl_3$): δ 165.4, 148.8, 140.7, 139.3, 138.3, 135.4, 134.8, 132.0, 129.6, 128.8, 127.3, 123.2, 117.9, 89.5; MS (ESI): *m/z* 375 $(M+H)^+$.

***N*-(5-Iodoquinolin-8-yl)cyclopentanecarboxamide (10d):**[12]

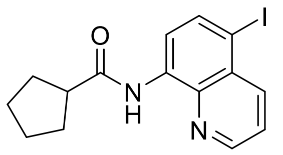

N-(Quinolin-8-yl)cyclopentanecarboxamide (**1e**, 96 mg, 0.40 mmol) and triiodoisocyanuric acid (74 mg, 0.145 mmol) in acetonitrile (3 mL), 120 min at room temperature. After column chromatography (gradient: EtOAc/hexanes 10:90 to 50:50) 138 mg (94%) of a white solid was obtained. R_f = 0.35 (EtOAc/hexanes 20:80); ^1H NMR (400 MHz, $CDCl_3$): δ 9.85 (s, 1H), 8.74 (dd, *J* = 4.2, 1.5 Hz, 1H), 8.55 (d, *J* = 8.3 Hz, 1H), 8.32 (dd, *J* = 8.5, 1.6 Hz, 1H), 8.04 (d, *J* = 8.3 Hz, 1H), 7.50 (dd, *J* = 8.5, 4.2 Hz, 1H), 2.94 (p, *J* = 8.1 Hz, 1H), 2.13-1.91 (m, 4H), 1.92-1.75 (m, 2H), 1.74-1.57 (m, 2H); $^{13}C\{^1H\}$NMR (100 MHz, $CDCl_3$): δ 175.1, 148.6, 140.6, 138.9, 138.2, 135.5, 129.4, 123.0, 117.7, 88.9, 47.4, 30.5, 26.0; MS (ESI): *m/z* 367 $(M+H)^+$.

2-Cyano-*N*-(5-iodoquinolin-8-yl)-2-methyl-3-phenylpropanamide (10e):

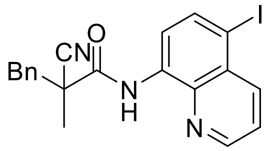

2-Cyano-2-methyl-3-phenyl-*N*-(quinolin-8-yl)propanamide (**1i**, 126 mg, 0.40 mmol) and triiodoisocyanuric acid (74 mg, 0.145 mmol) in acetonitrile (3 mL), 120 min at room temperature. After column chromatography (gradient: EtOAc/hexanes 10:90 to 50:50) 155 mg (88%) of a white solid was obtained. R_f = 0.31 (EtOAc/hexanes 20:80), mp = 188-190 °C; ^1H NMR (400 MHz, $CDCl_3$): δ 10.65 (s, 1H), 8.75 (dd, *J* = 4.2, 1.5 Hz, 1H), 8.48 (d, *J* = 8.3 Hz, 1H), 8.34 (dd, *J* = 8.5, 1.5 Hz, 1H), 8.09 (d, *J* = 8.2 Hz, 1H), 7.52 (dd, *J* = 8.5, 4.2 Hz, 1H), 7.36-7.31 (m, 2H), 7.28-7.17 (m, 3H), 3.42 (d, *J* = 13.5 Hz, 1H), 3.12 (d, *J* = 13.5 Hz, 1H), 1.77 (s, 3H); $^{13}C\{^1H\}$NMR (100 MHz, $CDCl_3$): δ 166.0, 149.3, 140.5, 139.1, 137.9, 134.4, 134.2, 130.0, 129.5, 128.6, 127.8, 123.4, 120.8, 118.0, 90.9, 47.0, 43.9, 23.7; FTIR (neat): 3313, 2923, 2853, 1713, 1590, 1493, 1379, 1343, 1153, 1088, 883, 762, 662 cm^{-1}; MS (ESI): *m/z* 442 $(M+H)^+$; HRMS (ESI): *m/z* calcd for $C_{20}H_{17}IN_3O$ $(M+H)^+$: 442.0411, found: 442.0415.

2-Benzyl-2-cyano-*N*-(5-iodoquinolin-8-yl)-3-phenylpropanamide (10f):

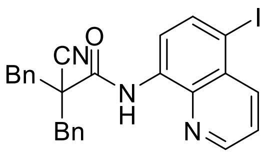

2-Benzyl-2-cyano-3-phenyl-*N*-(quinolin-8-yl)propanamide (**1h**, 156 mg, 0.40 mmol) and triiodoisocyanuric acid (74 mg, 0.145 mmol) in acetonitrile (3 mL), 120 min at room temperature. After column chromatography (gradient: EtOAc/hexanes 10:90 to 50:50) 184 mg (89%) of a white solid was obtained. R_f = 0.32 (EtOAc/hexanes 20:80), mp = 220-222 °C; ^{1}H NMR (400 MHz, $CDCl_3$): δ 10.30 (s, 1H), 8.58 (dd, *J* = 4.2, 1.5 Hz, 1H), 8.46 (d, *J* = 8.2 Hz, 1H), 8.22 (dd, *J* = 8.5, 1.5 Hz, 1H), 8.04 (d, *J* = 8.2 Hz, 1H), 7.39 (dd, *J* = 8.5, 4.2 Hz, 1H), 7.37-7.31 (m, 4H), 7.26-7.18 (m, 4H), 7.18-7.11 (m, 2H), 3.54 (d, *J* = 13.4 Hz, 2H), 3.16 (d, *J* = 13.4 Hz, 2H); ^{13}C{^{1}H}NMR (100 MHz, $CDCl_3$): δ 164.9, 149.1, 140.1, 138.9, 137.7, 134.3, 134.0, 130.0, 129.3, 128.5, 127.7, 123.2, 119.6, 117.8, 90.9, 54.6, 43.1; FTIR (neat): 3289, 3031, 2924, 1677, 1606, 1526, 1439, 1380, 1205, 1033, 916, 833, 739, 693 cm^{-1}; MS (ESI): *m/z* 518 $(M+H)^+$; HRMS (ESI): *m/z* calcd for $C_{26}H_{21}IN_3O$ $(M+H)^+$: 518.0724, found: 518.0727.

***tert*-Butyl (5-iodoquinolin-8-yl)carbamate (10g):**[24]

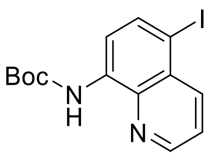

tert-Butyl quinolin-8-ylcarbamate (**1v**, 98 mg, 0.40 mmol) and triiodoisocyanuric acid (74 mg, 0.145 mmol) in acetonitrile (3 mL), 60 min at room temperature. After column chromatography (gradient: EtOAc/hexanes 5:95 to 50:50) 142 mg (96%) of a white solid was obtained. R_f = 0.35 (EtOAc/hexanes 20:80); ^{1}H NMR (400 MHz, $CDCl_3$): δ 8.93 (s, 1H), 8.64 (dd, *J* = 4.2, 1.6 Hz, 1H), 8.21 (dd, *J* = 8.5, 1.6 Hz, 1H), 8.09 (d, *J* = 8.3 Hz, 1H), 7.92 (d, *J* = 8.3 Hz, 1H), 7.38 (dd, *J* = 8.5, 4.2 Hz, 1H), 1.49 (s, 9H); ^{13}C{^{1}H}NMR (100 MHz, $CDCl_3$): δ 152.5, 148.4, 140.4, 138.7, 138.0, 136.1, 129.4, 123.0, 115.8, 87.5, 80.7, 28.3; MS (ESI): *m/z* 371 $(M+H)^+$.

***N*-(5-Iodoquinolin-8-yl)-*N*-methylbenzamide (10h):**

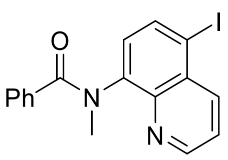

N-Methyl-*N*-(quinolin-8-yl)benzamide (**1x**, 105 mg, 0.40 mmol) and triiodoisocyanuric acid (74 mg, 0.145 mmol) in acetonitrile (3 mL), 6 h at room temperature. After column chromatography (gradient: EtOAc/hexanes 10:90 to 50:50) 123 mg (79%) of a white solid was obtained. R_f = 0.21 (EtOAc/hexanes 30:70), mp = 155-157 °C; ^{1}H NMR (400 MHz, $CDCl_3$): δ 8.87 (dd, *J* = 4.2, 1.5 Hz, 1H), 8.25 (dd, *J* = 8.5, 1.6 Hz, 1H), 7.88 (d, *J* =

7.9 Hz, 1H), 7.42 (dd, *J* = 8.5, 4.2 Hz, 1H), 7.28-7.18 (m, 2H), 7.13-7.00 (m, 2H), 6.95 (t, *J* = 7.5 Hz, 2H), 3.50 (s, 3H); $^{13}C\{^1H\}$NMR (100 MHz, $CDCl_3$): δ 172.0, 151.2, 144.2, 143.5, 140.6, 137.2, 136.4, 131.0, 129.9, 129.5, 128.0, 127.6, 123.3, 97.3, 38.6; FTIR (neat): 3313, 2922, 2857, 1678, 1634, 1518, 1489, 1393, 1286, 1033, 864, 760 cm^{-1}; MS (ESI): *m/z* 389 $(M+H)^+$; HRMS (ESI): *m/z* calcd for $C_{17}H_{14}IN_2O$ $(M+H)^+$: 389.0145, found: 389.0147.

3-(5-Iodoquinolin-8-yl)-1,1-dimethylurea (10i):

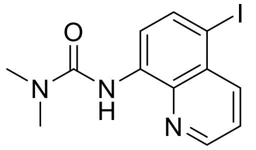

1,1-Dimethyl-3-(quinolin-8-yl)urea (**1ab**, 86 mg, 0.40 mmol) and triiodoisocyanuric acid (74 mg, 0.145 mmol) in acetonitrile (3 mL), 60 min at room temperature. After column chromatography (gradient: EtOAc/hexanes 10:90 to 50:50) 135 mg (99%) of a white solid was obtained. R_f = 0.22 (EtOAc/hexanes 30:70), mp = 104-106 °C; ^1H NMR (400 MHz, $CDCl_3$): δ 9.35 (s, 1H), 8.70 (dd, *J* = 4.2, 1.5 Hz, 1H), 8.35 (d, *J* = 8.4 Hz, 1H), 8.31 (dd, *J* = 8.5, 1.6 Hz, 1H), 8.02 (d, *J* = 8.4 Hz, 1H), 7.48 (dd, *J* = 8.5, 4.2 Hz, 1H), 3.15 (s, 6H); $^{13}C\{^1H\}$NMR (100 MHz, $CDCl_3$): δ 155.1, 148.2, 140.5, 139.0, 138.3, 136.9, 129.4, 122.8, 116.0, 86.5, 36.4; MS (ESI): *m/z* 342 $(M+H)^+$; HRMS (ESI): *m/z* calcd for $C_{12}H_{13}ION_3$ $(M+H)^+$: 342.0098, found: 342.0101.

Ethyl *P*-ethyl-*N*-(5-iodoquinolin-8-yl)phosphonamidate (10j):

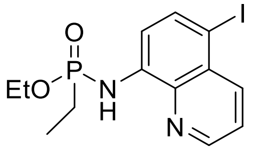

Ethyl *P*-ethyl-*N*-(quinolin-8-yl)phosphonamidate (**1ad**, 106 mg, 0.40 mmol) and triiodoisocyanuric acid (74 mg, 0.145 mmol) in acetonitrile (3 mL), 60 min at room temperature. After column chromatography (gradient: EtOAc/hexanes 10:90 to 60:40) 153 mg (98%) of a white solid was obtained. R_f = 0.25 (EtOAc/hexanes 30:70), mp = 161-163 °C; ^1H NMR (400 MHz, $CDCl_3$): δ 8.75 (dd, *J* = 4.1, 1.4 Hz, 1H), 8.32 (dd, *J* = 8.5, 1.5 Hz, 1H), 7.96 (d, *J* = 8.2 Hz, 1H), 7.68 (d, *J* = 8.3 Hz, 1H), 7.52 (dd, *J* = 8.5, 4.2 Hz, 1H), 7.33 (d, *J* = 8.2 Hz, 1H), 4.39-3.89 (m, 2H), 2.08-1.86 (m, 2H), 1.37 (t, *J* = 7.1 Hz, 3H), 1.18 (dt, *J* = 20.3, 7.7 Hz, 3H); $^{13}C\{^1H\}$NMR (100 MHz, $CDCl_3$): δ 148.5, 140.4, 139.0 (d, *J* = 8.0 Hz), 138.9 (d, *J* = 2.3 Hz), 137.7, 129.9, 123.3, 113.2, 85.9, 60.6 (d, *J* = 6.8 Hz), 19.6 (d, *J* = 130.9 Hz), 16.3 (d, *J* = 6.4 Hz), 6.2 (d, *J* = 5.9 Hz); FTIR (neat): 3310,

2933, 2976, 1680, 1587, 1498, 1353, 1318, 1244, 948, 855, 784 cm^{-1}; MS (ESI): *m/z* 391 $(M+H)^+$; HRMS (ESI): *m/z* calcd for $C_{13}H_{17}IN_2O_2P$ $(M+H)^+$: 391.0067, found: 391.0072.

***N*,*N*-Dibenzyl-5-iodoquinolin-8-amine (10k):**

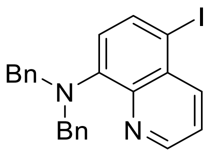

N,*N*-Dibenzylquinolin-8-amine (**1an**, 130 mg, 0.40 mmol) and triiodoisocyanuric acid (74 mg, 0.145 mmol) in acetonitrile (3 mL), 6 h at room temperature. After column chromatography (gradient: EtOAc/hexanes 5:95 to 50:50) 155 mg (86%) of a white solid was obtained as a sole product. R_f = 0.38 (EtOAc/hexanes 20:80), mp = 161-163 °C; ^{1}H NMR (400 MHz, $CDCl_3$): δ 8.87 (dd, J = 4.1, 1.7 Hz, 1H), 8.34 (dd, J = 8.5, 1.7 Hz, 1H), 7.78 (d, J = 8.2 Hz, 1H), 7.46 (dd, J = 8.5, 4.1 Hz, 1H), 7.33-7.15 (m, 10H), 6.66 (d, J = 8.3 Hz, 1H), 4.70 (s, 4H); $^{13}C\{^1H\}$NMR (100 MHz, $CDCl_3$): δ 148.0, 148.0, 143.7, 140.7, 138.4, 137.3, 130.9, 128.3, 128.2, 126.9, 122.4, 119.9, 87.8, 56.7; FTIR (neat): 3290, 2923, 2861, 1712, 1590, 1505, 1470, 1379, 1170, 1152, 1012, 833, 730, 664 cm^{-1}; MS (ESI): *m/z* 451 $(M+H)^+$; HRMS (ESI): *m/z* calcd for $C_{23}H_{20}IN_2$ $(M+H)^+$: 451.0666, found: 451.0671.

***N*-(5-Iodo-6-methoxyquinolin-8-yl)acetamide (10l):[18a]**

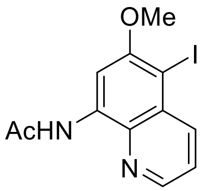

N-(6-Methoxyquinolin-8-yl)acetamide (**1ag**, 86 mg, 0.40 mmol) was treated with triiodoisocyanuric acid (73 mg, 0.145 mmol) in acetonitrile (3 mL) for 6 h at room temperature. After column chromatography (gradient: EtOAc/hexanes 20:80 to 60:40) 130 mg (95%) of a white solid was obtained. R_f = 0.25 (EtOAc/hexanes 30:70); ^{1}H NMR (400 MHz, $CDCl_3$): δ 9.85 (s, 1H), 8.75 (s, 1H), 8.59 (dd, J = 4.2, 1.5 Hz, 1H), 8.40 (dd, J = 8.6, 1.5 Hz, 1H), 7.46 (dd, J = 8.6, 4.2 Hz, 1H), 4.06 (s, 3H), 2.37 (s, 3H); $^{13}C\{^1H\}$NMR (100 MHz, $CDCl_3$): δ 169.1, 157.3, 146.2, 139.5, 136.3, 134.8, 130.4, 123.5, 103.7, 57.2, 25.3; MS (ESI): *m/z* 343 $(M+H)^+$.

***N*-(3-Bromo-5-iodoquinolin-8-yl)acetamide (10m):[18a]**

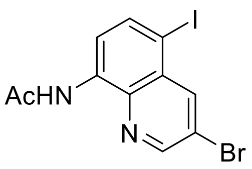

N-(3-Bromoquinolin-8-yl)acetamide (**1ai**, 106 mg, 0.40 mmol) was treated with triiodoisocyanuric acid (73 mg, 0.145 mmol) in acetonitrile (3 mL) for 6 h at room temperature. After column chromatography (gradient: EtOAc/hexanes 20:80 to 60:40) 127 mg (81%) of a pale yellow solid was obtained. R_f = 0.25 (EtOAc/hexanes 30:70); ^{1}H NMR (400

MHz, $CDCl_3$): δ 9.61 (s, 1H), 8.74 (s, 1H), 8.59 – 8.53 (m, 2H), 8.08 (d, J = 8.2 Hz, 1H), 2.35 (s, 3H); $^{13}C\{^1H\}$NMR (100 MHz, $CDCl_3$): δ 168.8, 149.8, 142.2, 139.5, 136.9, 135.6, 130.6, 119.9, 118.1, 87.4, 25.2; MS (ESI): m/z 393 $(M+H)^+$.

Ethyl 2-((5-iodoquinolin-8-yl)oxy)acetate (10n):

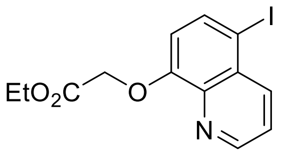

Ethyl 2-(quinolin-8-yloxy)acetate (**1aq**, 141 mg, 0.40 mmol) and triiodoisocyanuric acid (74 mg, 0.145 mmol) in acetonitrile (3 mL), 40 h at room temperature. After column chromatography (gradient: EtOAc/hexanes 5:95 to 50:50) 112 mg (54%) of a white solid was obtained. R_f = 0.35 (EtOAc/hexanes 20:80); ^{1}H NMR (400 MHz, $CDCl_3$): δ 8.94 (dd, J = 4.2, 1.6 Hz, 1H), 8.36 (dd, J = 8.6, 1.6 Hz, 1H), 7.99 (d, J = 8.3 Hz, 1H), 7.54 (dd, J = 8.6, 4.2 Hz, 1H), 6.77 (d, J = 8.3 Hz, 1H), 4.96 (s, 2H), 4.27 (q, J = 7.1 Hz, 2H), 1.28 (t, J = 7.1 Hz, 3H); $^{13}C\{^1H\}$NMR (100 MHz, $CDCl_3$): δ 168.4, 154.5, 150.1, 140.8, 140.4, 137.0, 130.9, 123.4, 111.0, 88.2, 66.1, 61.5, 14.1; FTIR (neat): 3056, 2993, 2891, 1752, 1601, 1587, 1447, 1301, 1098, 1001, 896, 754, 683 cm^{-1}; MS (ESI): m/z 358 $(M+H)^+$; HRMS (ESI): m/z calcd for $C_{13}H_{13}INO_3$ $(M+H)^+$: 357.9935, found: 357.9938.

7-Iodoquinolin-8-ol (11):[32]

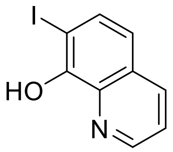

In a 50 mL round bottom flask equipped with reflux condenser, a solution of 8-hydroxyquinoline (**1ap**, 200 mg, 1.37 mmol) and N-iodosuccinimide (342 mg, 1.51 mmol) in $CHCl_3$ (12 mL) was vigorously stirred for 24 h at 40 °C. The resulting suspension was filtered and the organic phase was washed with a 5% aqueous solution of $Na_2S_2O_3$ (2 × 15 mL) and dried ($MgSO_4$). After removal of the solvent under reduced pressure, the product was isolated as a light-yellow solid by crystallization from MeOH:H_2O (268 mg, 72%). ^{1}H NMR (400 MHz, $CDCl_3$): δ 8.78 (dd, J = 4.3, 1.5 Hz, 1H), 8.16 (dd, J = 8.3, 1.5 Hz, 1H), 7.79 (d, J = 8.8 Hz, 1H), 7.49 (dd, J = 8.3, 4.3 Hz, 1H), 7.15 (d, J = 8.8 Hz, 1H); $^{13}C\{^1H\}$NMR (100 MHz, $CDCl_3$): δ 152.8, 148.4, 145.1, 137.2, 136.4, 136.3, 128.1, 122.3, 119.3; MS (ESI): m/z 272 $(M+H)^+$.

5-Chloro-7-iodoquinolin-8-ol (G):[33]

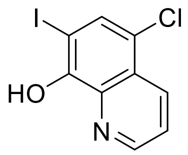

A 10 mL round bottom flask equipped with a magnetic stir bar was charged with 7-iodoquinolin-8-ol (**11**, 109 mg, 0.40 mmol), and acetonitrile (4 mL) under open air conditions at room temperature. To the stirred solution was added TCCA (34 mg, 0.145 mmol) in one portion, and the resulting solution was stirred at room temperature for 2 h. The reaction mixture was then evaporated, and the crude residue was purified by flash chromatography (EtOAc/hexanes, 05:95 to 60:40) to yield the corresponding C5 chlorinated derivative **G** in 79% yield as a brown solid. ^{1}H NMR (400 MHz, $CDCl_3$): *δ* 8.95 (dd, *J* = 4.5, 1.5 Hz, 1H), 8.47 (dd, *J* = 8.2, 1.6 Hz, 1H), 7.74 (dd, *J* = 8.2, 4.5 Hz, 1H), 7.50 (s, 1H); ^{13}C{^{1}H}NMR (100 MHz, $CDCl_3$): *δ* 174.8, 152.7, 141.2, 141.0, 139.6, 137.7, 132.0, 128.2, 76.1; MS (ESI): *m/z* 306 $(M+H)^+$.

***N*-(5,7-Dichloro-2-methylquinolin-8-yl)-4-fluorobenzenesulfonamide (15):**[34]

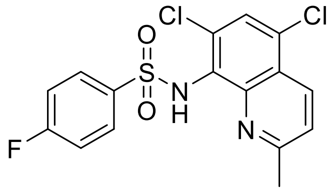

A 10 mL round bottom flask equipped with a magnetic stir bar was charged with 4-fluoro-*N*-(2-methylquinolin-8-yl)benzenesulfonamide (**12**, 126 mg, 0.40 mmol) and trichloroisocyanuric acid (65 mg, 0.29 mmol) in acetonitrile (5 mL) and stirred for 6 h at room temperature. The reaction mixture was then evaporated, and the crude residue was purified by flash chromatography (EtOAc/hexanes, 10:90 to 50:50) to yield the *N*-(5,7-dichloro-2-methylquinolin-8-yl)-4-fluorobenzenesulfonamide in 98 mg (86%) of a white powder. R_f = 0.20 (3:1 EtOAc:ethanol/hexanes 20:80); ^{1}H NMR (400 MHz, $CDCl_3$): *δ* 8.31 (d, *J* = 8.6 Hz, 1H), 8.10 (s, 1H), 7.79-7.70 (m, 2H), 7.63 (s, 1H), 7.30 (d, *J* = 8.6 Hz, 1H), 7.04-6.93 (m, 2H), 2.50 (s, 3H); ^{13}C{^{1}H}NMR (100 MHz, $CDCl_3$): *δ* 166.3, 163.8, 160.3, 142.7, 135.9 (d, *J* = 3.2 Hz), 133.4, 131.1, 130.3 (d, *J* = 9.4 Hz), 129.7 (d, *J* = 22.4 Hz), 127.7, 123.3, 123.2, 115.5 (d, *J* = 22.6 Hz), 24.8; FTIR (neat): 3285, 2967, 2857, 1732, 1575, 1497, 1363, 1276, 1183, 1001, 876, 699 cm^{-1}; MS (ESI): *m/z* 385 $(M+H)^+$; HRMS (ESI): *m/z* calcd for $C_{16}H_{12}Cl_2FN_2O_2S$ $(M+H)^+$: 384.9975, found: 384.9976.

***N*-(5,7-Dichloro-2-formylquinolin-8-yl)-4-fluorobenzenesulfonamide (A):**[34]

To a solution of *N*-(5,7-dichloro-2-methylquinolin-8-yl)-4-fluorobenzenesulfonamide **15** (115 mg, 0.3 mmol) in 1,4-dioxane (2 mL), selenium dioxide (45 mg, 0.4 mmol) was added, and the mixture was heated

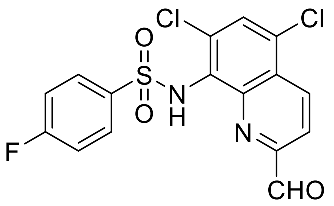

at 80 °C for 4 h. After cooling to room temperature, the solution was filtered through Celite and concentrated under reduced pressure and purified by column chromatography to afford the compound as a white solid (108 mg, 90%). ^{1}H NMR (400 MHz, CDCl$_3$): δ 9.84 (d, J = 0.6 Hz, 1H), 8.66 (dd, J = 8.6, 0.6 Hz, 1H), 8.05 (d, J = 8.6 Hz, 1H), 7.96 (s, 1H), 7.89-7.76 (m, 3H), 7.05 (t, J = 8.5 Hz, 2H); ^{13}C{^{1}H}NMR (100 MHz, CDCl$_3$): δ 191.7, 166.5, 163.9, 152.2, 143.3, 135.9 (d, J = 3.3 Hz), 135.4, 132.7, 131.1, 130.3 (d, J = 9.4 Hz), 127.0, 118.5, 116.0, 115.7; FTIR (neat): 3309, 2923, 2853, 1712, 1590, 1493, 1378, 1326, 1169, 908, 834, 709 cm^{-1}; MS (ESI): *m/z* 399 (M+H)$^+$; HRMS (ESI): *m/z* calcd for $C_{16}H_{10}Cl_2FN_2O_3S$ (M+H)$^+$: 398.9768, found: 398.9771.

***N*-(5-Bromoquinolin-8-yl)-3-(trifluoromethyl)benzenesulfonamide (16):**[26]

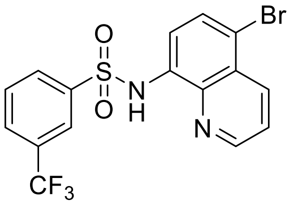

A 10 mL round bottom flask equipped with a magnetic stir bar was charged with quinoline derivative **13** (141 mg, 0.40 mmol) and acetonitrile (4 mL) under open air conditions at room temperature. To the stirred solution was added TBCA (53 mg, 0.145 mmol) in one portion, and the resulting solution was stirred at room temperature for 15 minutes. The reaction mixture was then evaporated, and the crude residue was purified by flash chromatography (EtOAc/hexanes, 10:90 to 60:40) to yield the C5-brominated quinoline derivative **16** in 86% yield as a white solid. ^{1}H NMR (400 MHz, CDCl$_3$): δ 9.25 (s, 1H), 8.77 (dd, J = 4.2, 1.5 Hz, 1H), 8.44 (dd, J = 8.5, 1.6 Hz, 1H), 8.18 (s, 1H), 8.05 (d, J = 7.9 Hz, 1H), 7.77-7.66 (m, 3H), 7.57-7.45 (m, 2H); ^{13}C{^{1}H}NMR (100 MHz, CDCl$_3$): δ 149.5, 140.2, 139.2, 136.1, 133.0, 131.6 (q, J = 33.6 Hz), 130.3, 130.3, 129.8, 129.5 (q, J = 3.6 Hz), 127.6, 124.3 (q, J = 3.9 Hz), 123.2, 123.1 (q, J = 273.0 Hz), 116.3, 116.1; MS (ESI): *m/z* 431 (M+H)$^+$.

***N*-(5-(Pyridin-3-yl)quinolin-8-yl)-3-(trifluoromethyl)benzenesulfonamide (B):**[12, 26]

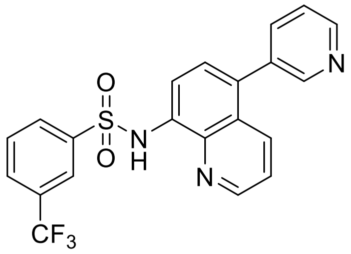

Bromo compound **16** (130 mg, 0.3 mmol, 1 equiv.) and pyridine-3-boronic acid (**17**, 56 mg, 0.45 mmol, 1.5 equiv.) were taken in a dried microwave tube with a magnetic stir bar under argon. Then 1,4-dioxane:water (3:1) mixture was added, and the resulting mixture was degassed for 1 min by bubbling argon gas through it. The catalyst $(Ph_3P)_2PdCl_2$ (10.5 mg, 0.015 mmol, 5 mol%) and Na_2CO_3 (64 mg, 0.6 mmol, 2 equiv.) were added, and the reaction was further degassed for 1 min before sealing and heating under microwave irradiation at 100 ºC for 20 min. After completion of the reaction (monitored by TLC), the mixture was diluted with EtOAc and washed with saturated $NaHCO_3$ solution. The organic layer were dried ($MgSO_4$) and concentrated under reduced pressure, and the crude was purified by silica gel column chromatography (EtOAc/hexanes, 30:70 to 100:0) to provide product **B** as a brown solid (114 mg, 88%). ^{1}H NMR (400 MHz, $CDCl_3$): δ 9.40 (s, 1H), 8.81 (dd, J = 4.2, 1.6 Hz, 1H), 8.72-8.63 (m, 2H), 8.20 (s, 1H), 8.17-8.09 (m, 2H), 7.93 (d, J = 7.9 Hz, 1H), 7.76-7.68 (m, 2H), 7.55 (t, J = 7.9 Hz, 1H), 7.48-7.40 (m, 3H); ^{13}C{^{1}H}NMR (100 MHz, $CDCl_3$): δ 150.6, 149.2, 149.1, 140.6, 138.8, 137.4, 134.5, 134.3, 133.4, 131.9, 131.8 (q, $J_{C\text{-}F}$ = 66.26 Hz), 130.5, 129.9, 129.7 (q, $J_{C\text{-}F}$ = 3.43 Hz), 128.0, 126.7, 124.5 (q, $J_{C\text{-}F}$ = 3.71 Hz), 123.6, 122.2 (q, $J_{C\text{-}F}$ = 271.22 Hz), 122.6, 115.4; MS (ESI): *m/z* 430 $(M+H)^+$.

5-Chloro-8-methylquinoline (18a):[35]

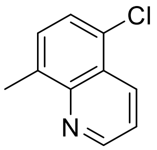

White solid, ^{1}H NMR (400 MHz, $CDCl_3$): δ 8.98 (dd, J = 4.2, 1.7 Hz, 1H), 8.56 (dd, J = 8.5, 1.7 Hz, 1H), 7.54-7.43 (m, 3H), 2.78 (s, 3H); ^{13}C{^{1}H}NMR (100 MHz, $CDCl_3$): δ 149.6, 147.7, 136.4, 133.0, 129.1, 128.7, 126.2, 126.1, 121.5, 18.0; MS (ESI): *m/z* 178 $(M+H)^+$.

5-Chloro-8-(chloromethyl)quinoline (18b):[35]

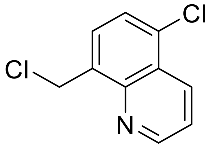

White solid, ^{1}H NMR (400 MHz, $CDCl_3$): δ 9.03 (dd, J = 4.2, 1.7 Hz, 1H), 8.60 (dd, J = 8.6, 1.7 Hz, 1H), 7.78 (d, J = 7.8 Hz, 1H), 7.63 (d, J = 7.8 Hz, 1H), 7.56 (dd, J = 8.5, 4.2 Hz, 1H), 5.30 (s, 2H); ^{13}C{^{1}H}NMR (100 MHz, $CDCl_3$): δ 150.6, 146.3, 135.0, 133.2, 131.9, 129.7, 126.3 (2), 122.2, 42.0; MS (ESI): *m/z* 212 $(M+H)^+$.

8-(Bromomethyl)quinoline (19a):[36]

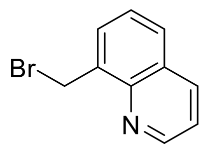

White solid, ^{1}H NMR (400 MHz, $CDCl_3$): δ 9.00 (dd, J = 4.2, 1.8 Hz, 1H), 8.14 (dd, J = 8.3, 1.8 Hz, 1H), 7.80 (ddd, J = 9.6, 7.7, 1.3 Hz, 2H), 7.49 (dd, J = 8.2, 7.2 Hz, 1H), 7.42 (dd, J = 8.3, 4.2 Hz, 1H), 5.24 (s, 2H); $^{13}C\{^1H\}$NMR (100 MHz, $CDCl_3$): δ 150.1, 145.7, 136.2, 136.0, 130.5, 128.8, 128.4, 126.3, 121.5, 29.6; MS (ESI): *m/z* 222 $(M+H)^+$.

5-Bromo-8-(bromomethyl)quinoline (21):[37]

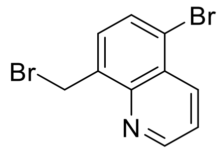

White solid, ^{1}H NMR (400 MHz, $CDCl_3$): δ 9.03 (dd, J = 4.2, 1.7 Hz, 1H), 8.57 (dd, J = 8.6, 1.7 Hz, 1H), 7.81 (d, J = 7.7 Hz, 1H), 7.70 (d, J = 7.7 Hz, 1H), 7.56 (dd, J = 8.6, 4.2 Hz, 1H), 5.20 (s, 2H); $^{13}C\{^1H\}$NMR (100 MHz, $CDCl_3$): δ 150.7, 146.3, 136.2, 135.8, 130.7, 130.2, 127.9, 122.7, 122.6, 28.9; MS (ESI): *m/z* 300 $(M+H)^+$.

4) References:

1. S. H. Combe, A. Hosseini, A. Parra and P. R. Schreiner, *J. Org. Chem.*, 2017, **82**, 2407-2413.
2. (a) J. Chen, T. Wang, Y. Liu, T. Wang, A. Lin, H. Yao and J. Xu, *Org. Chem. Front.*, 2017, **4**, 622-626; (b) Y. Li, L. Zhu, X. Cao, C.-T. Au, R. Qiu and S.-F. Yin, *Adv. Synth. Catal.*, 2017, **359**, 2864-2873.
3. (a) L. Grigorjeva and O. Daugulis, *Angew. Chem. Int. Ed.*, 2014, **53**, 10209-10212; (b) C. J. Whiteoak, O. Planas, A. Company and X. Ribas, *Adv. Synth. Catal.*, 2016, **358**, 1679-1688; (c) M. D. Reddy, F. R. Fronczek and E. B. Watkins, *Org. Lett.*, 2016, **18**, 5620-5623; (d) S. Mondal, A. Maity, S. Naskar, R. Paira, A. Hazra, K. B. Sahu, P. Saha, S. Das, S. Banerjee and N. B. Mondal, *Synthesis*, 2011, **2011**, 2079-2084.
4. M. D. Reddy and E. B. Watkins, *J. Org. Chem.*, 2015, **80**, 11447-11459.
5. (a) H. Yi, H. Chen, C. Bian, Z. Tang, A. K. Singh, X. Qi, X. Yue, Y. Lan, J.-F. Lee and A. Lei, *Chem. Commun.*, 2017, **53**, 6736-6739; (b) Y. Kuninobu, M. Nishi and M. Kanai, *Org. Biomol. Chem.*, 2016, **14**, 8092-8100; (c) Z.-Y. Huang, J.-F. Yang, K. Song, Q. Chen, S.-L. Zhou, G.-F. Hao and G.-F. Yang, *J. Org. Chem.*, 2016, **81**, 9647-9657; (d) C. R. Shugrue and S. J. Miller, *Angew. Chem. Int. Ed.*, 2015, **54**, 11173-11176.
6. T. T. Nguyen and O. Daugulis, *Chem. Commun.*, 2017, **53**, 4609-4611.
7. M. Rouffet, C. A. F. de Oliveira, Y. Udi, A. Agrawal, I. Sagi, J. A. McCammon and S. M. Cohen, *J. Am. Chem. Soc.*, 2010, **132**, 8232-8233.
8. G. Brancatelli, D. Drommi, G. Femino, M. Saporita, G. Bottari and F. Faraone, *New J. Chem.*, 2010, **34**, 2853-2860.
9. G. Desai, A. Asrondkar, V. Patil, A. Bobade and A. Chowdhary, *World J. Pharm. Res.*, 2015, **4**, 1487-1494.
10. M. Liu, Y. Niu, Y.-F. Wu and X.-S. Ye, *Org. Lett.*, 2016, **18**, 1836-1839.
11. Y. Wang, Y. Wang, K. Jiang, Q. Zhang and D. Li, *Org. Biomol. Chem.*, 2016, **14**, 10180-10184.
12. H. Sahoo, I. Ramakrishna and M. Baidya, *ChemistrySelect.*, 2016, **1**, 1949-1953.
13. J. Zhang, H. Chen, C. Lin, Z. Liu, C. Wang and Y. Zhang, *J. Am. Chem. Soc.*, 2015, **137**, 12990-12996.
14. S.-Y. Zhang, Q. Li, G. He, W. A. Nack and G. Chen, *J. Am. Chem. Soc.*, 2015, **137**, 531-539.

15. J.-Y. Jiao, Y.-J. Mao, A.-W. Feng, X.-F. Li, M.-T. Li and X.-H. Zhang, *Tetrahedron.*, 2017, **73**, 1482-1488.
16. M. G. Ferlin, G. Chiarelotto and I. Castagliuolo, *J. Heterocycl. Chem.*, 2002, **39**, 631-638.
17. H. Qiao, S. Sun, F. Yang, Y. Zhu, W. Zhu, Y. Dong, Y. Wu, X. Kong, L. Jiang and Y. Wu, *Org. Lett.*, 2015, **17**, 6086-6089.
18. (a) X. He, Y.-z. Xu, L.-x. Kong, H.-h. Wu, D.-z. Ji, Z.-b. Wang, Y.-g. Xu and Q.-h. Zhu, *Org. Chem. Front.*, 2017, **4**, 1046-1050; (b) H. Chen, P. Li, M. Wang and L. Wang, *Org. Lett.*, 2016, **18**, 4794-4797.
19. K. Canto, R. da Silva Ribeiro, A. F. P. Biajoli and C. R. D. Correia, *Eur. J. Org. Chem.*, 2013, **2013**, 8004-8013.
20. R. d. S. Ribeiro, P. M. Esteves and M. C. S. de Mattos, *Tetrahedron Lett.*, 2007, **48**, 8747-8751.
21. X. He, Y.-z. Xu, L.-x. Kong, H.-h. Wu, D.-z. Ji, Z.-b. Wang, Y.-g. Xu and Q.-h. Zhu, *Org. Chem. Front.*, 2017, **4**, 1046-1050.
22. A. M. Suess, M. Z. Ertem, C. J. Cramer and S. S. Stahl, *J. Am. Chem. Soc.*, 2013, **135**, 9797-9804.
23. Y. Aihara, M. Tobisu, Y. Fukumoto and N. Chatani, *J. Am. Chem. Soc.*, 2014, **136**, 15509-15512.
24. C. Sen, T. Sahoo and S. C. Ghosh, *ChemistrySelect.*, 2017, **2**, 2745-2749.
25. H. Qiao, S. Sun, F. Yang, Y. Zhu, J. Kang, Y. Wu and Y. Wu, *Adv. Synth. Catal.*, 2017, **359**, 1976-1980.
26. R. Singh, A. Sran, D. C. Carroll, J. Huang, L. Tsvetkov, X. Zhou, J. Sheung, J. McLaughlin, S. D. Issakani, D. G. Payan and S. J. Shaw, *Bioorg. Med. Chem. Lett.*, 2015, **25**, 5199-5202.
27. Y.-S. Xie, B. V. D. Vijaykumar, K. Jang, H.-H. Shin, H. Zuo and D.-S. Shin, *Tetrahedron Lett.*, 2013, **54**, 5151-5154.
28. R. S. Talekar, G. S. Chen, S.-Y. Lai and J.-W. Chern, *J. Org. Chem.*, 2005, **70**, 8590-8593.
29. A. J. Middleton, W. J. Marshall and N. S. Radu, *J. Am. Chem. Soc.*, 2003, **125**, 880-881.
30. K. Li, Y. Li, D. Zhou, Y. Fan, H. Guo, T. Ma, J. Wen, D. Liu and L. Zhao, *Biorg. Med. Chem.*, 2016, **24**, 1889-1897.
31. L. Kumar, T. Mahajan and D. D. Agarwal, *Ind. Eng. Chem. Res.*, 2012, **51**, 2227-2234.
32. F. Babudri, A. Cardone, C. T. Cioffi, G. M. Farinola, F. Naso and R. Ragni, *Synthesis*, 2006, 1325-1332.
33. A. Deshmukh, B. Gore, H. V. Thulasiram and V. P. Swamy, *RSC Adv.*, 2015, **5**, 88311-88315.
34. E. C. L. Gautier, K. J. Barnham, P. J. Huggins and J. G. Parsons, WO2008074068A1, 2008.
35. B. Liu, P. Hu, X. Zhou, D. Bai, J. Chang and X. Li, *Org. Lett.*, 2017, **19**, 2086-2089.
36. (a) A. N. Dwyer, M. C. Grossel and P. N. Horton, *Supramol. Chem.*, 2004, **16**, 405-410; (b) P. G. Gildner, A. A. S. Gietter, D. Cui and D. A. Watson, *J. Am. Chem. Soc.*, 2012, **134**, 9942-9945.
37. (a) A. I. Tochilkin, I. N. Gracheva, I. R. Kovel'man and E. P. Prokof'ev, *Khim. Geterotsikl. Soedin.*, 1983, 1373-1376; (b) J. Jiao, H. Fang, H. Zhu, L. Su, L. Jiang and W. Xu, *Zhongguo Yaowu Huaxue Zazhi*, 2008, **18**, 250-253.

^{1}H NMR spectrum of compound **1g** ($CDCl_3$, 400 MHz)**:**

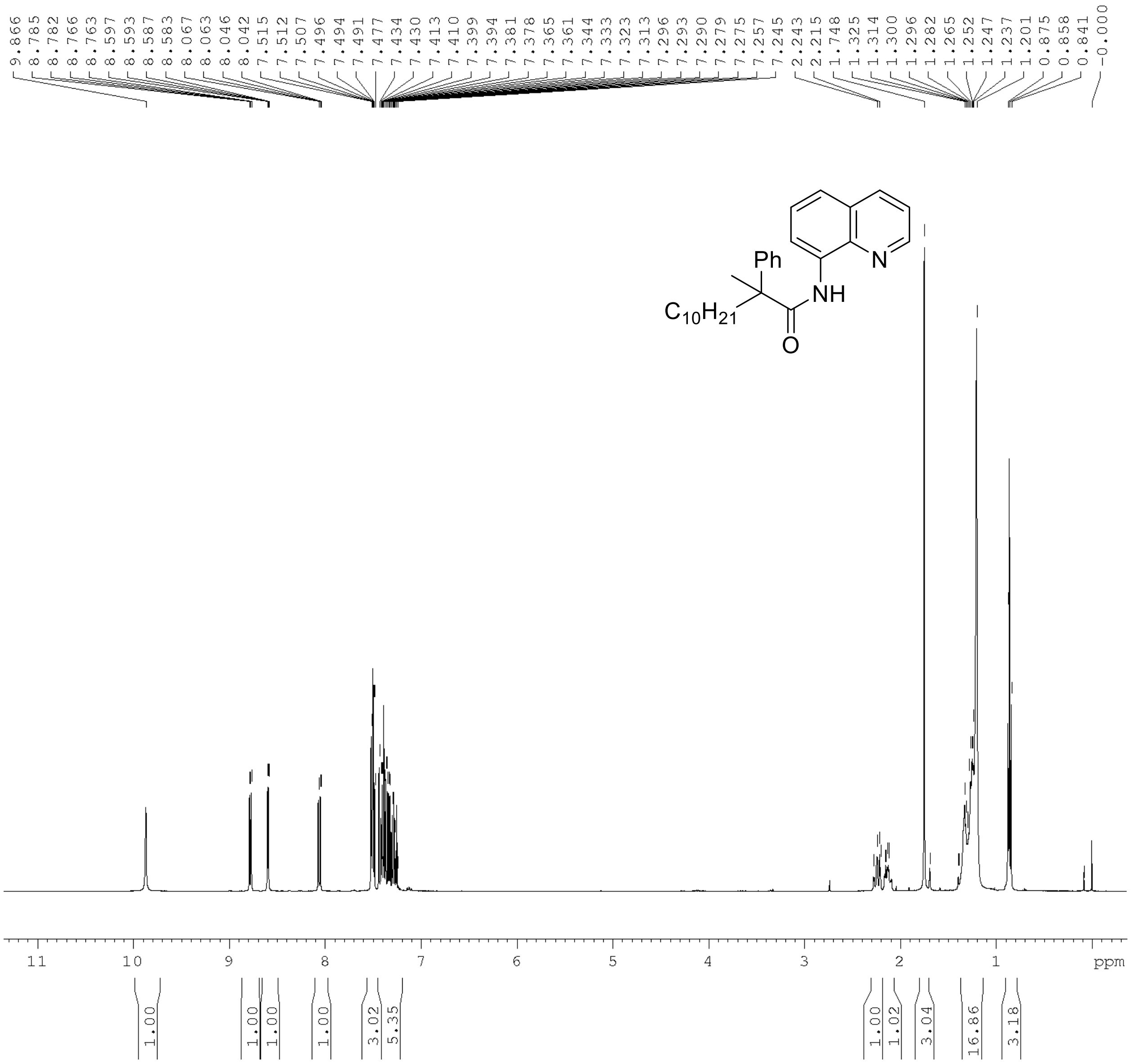

$^{13}C\{^{1}H\}$NMR spectrum of compound **1g** ($CDCl_3$, 100 MHz)**:**

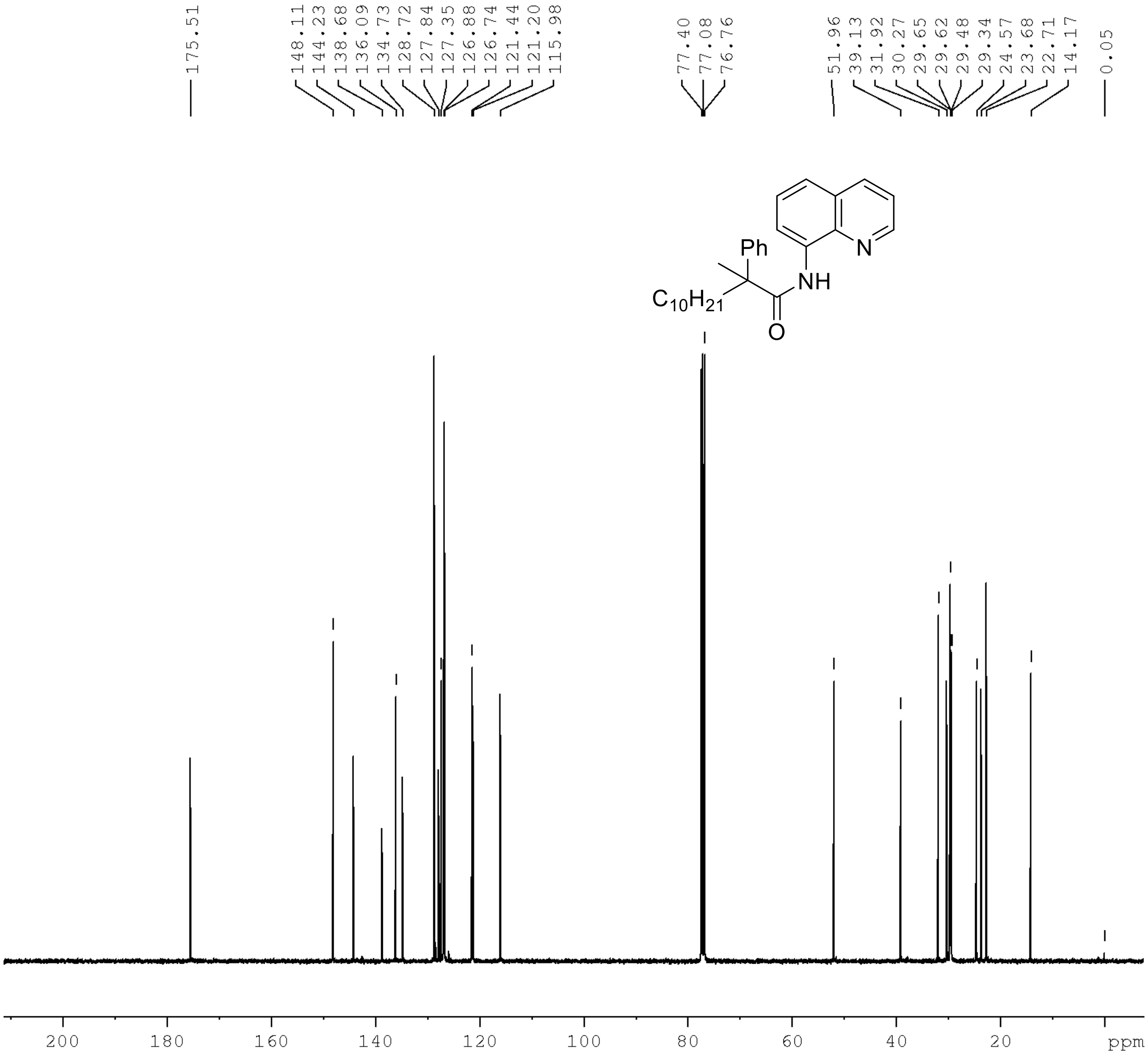

DEPT135 spectrum of compound **1g** ($CDCl_3$, 100 MHz)**:**

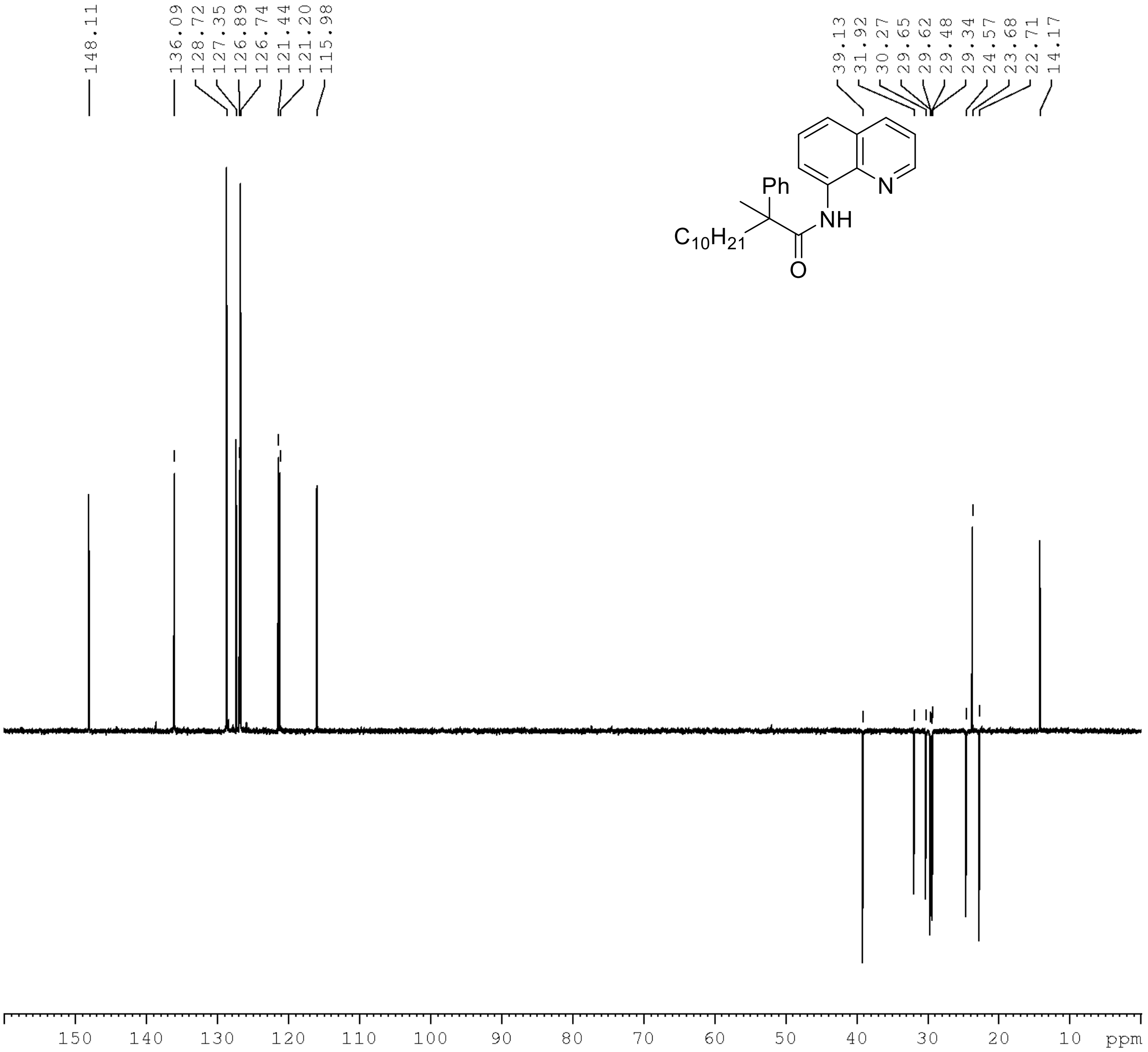

^{1}H NMR spectrum of compound **1y** ($CDCl_3$, 400 MHz)**:**

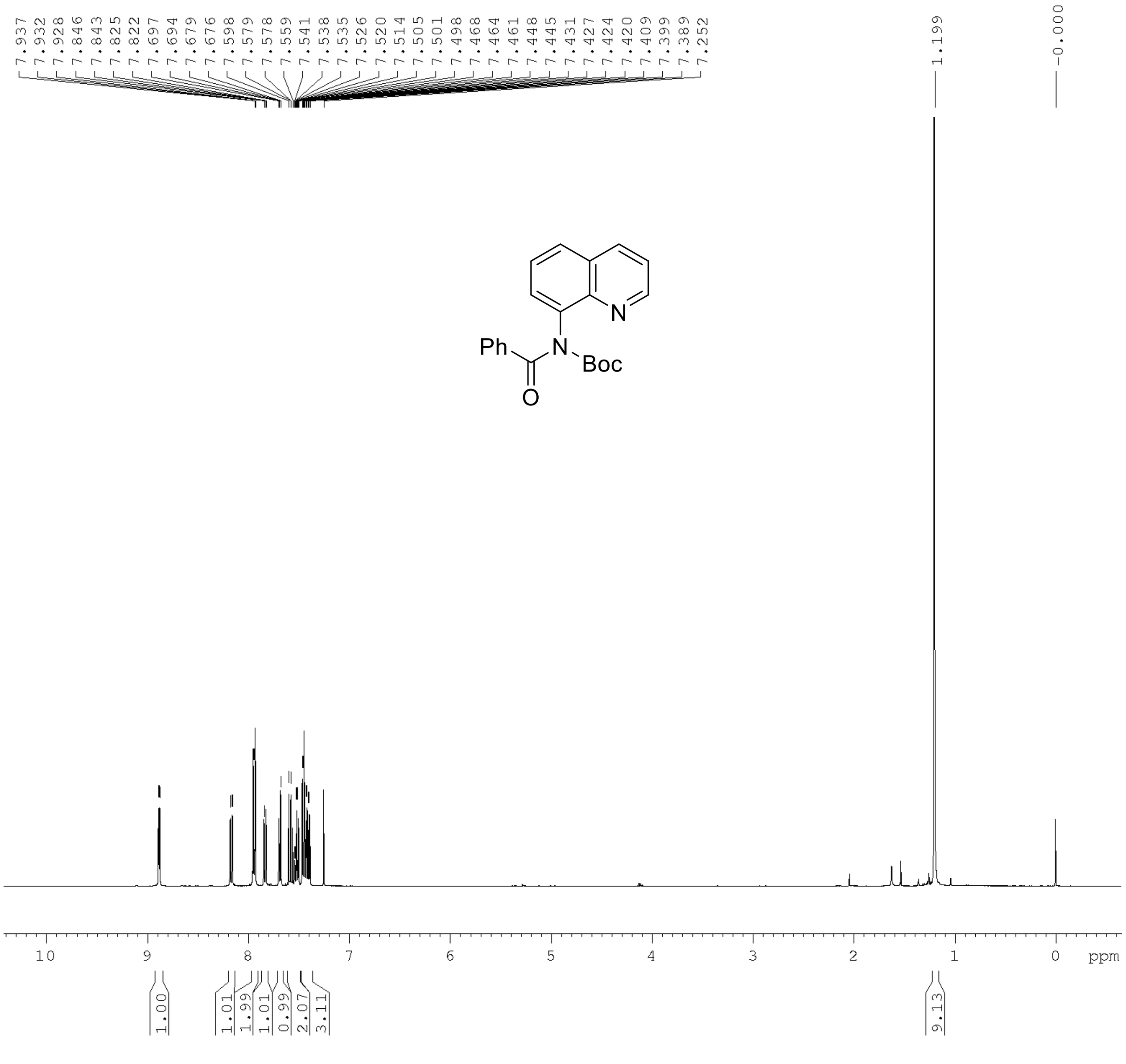

$^{13}C\{^{1}H\}$NMR spectrum of compound **1y** ($CDCl_3$, 100 MHz)**:**

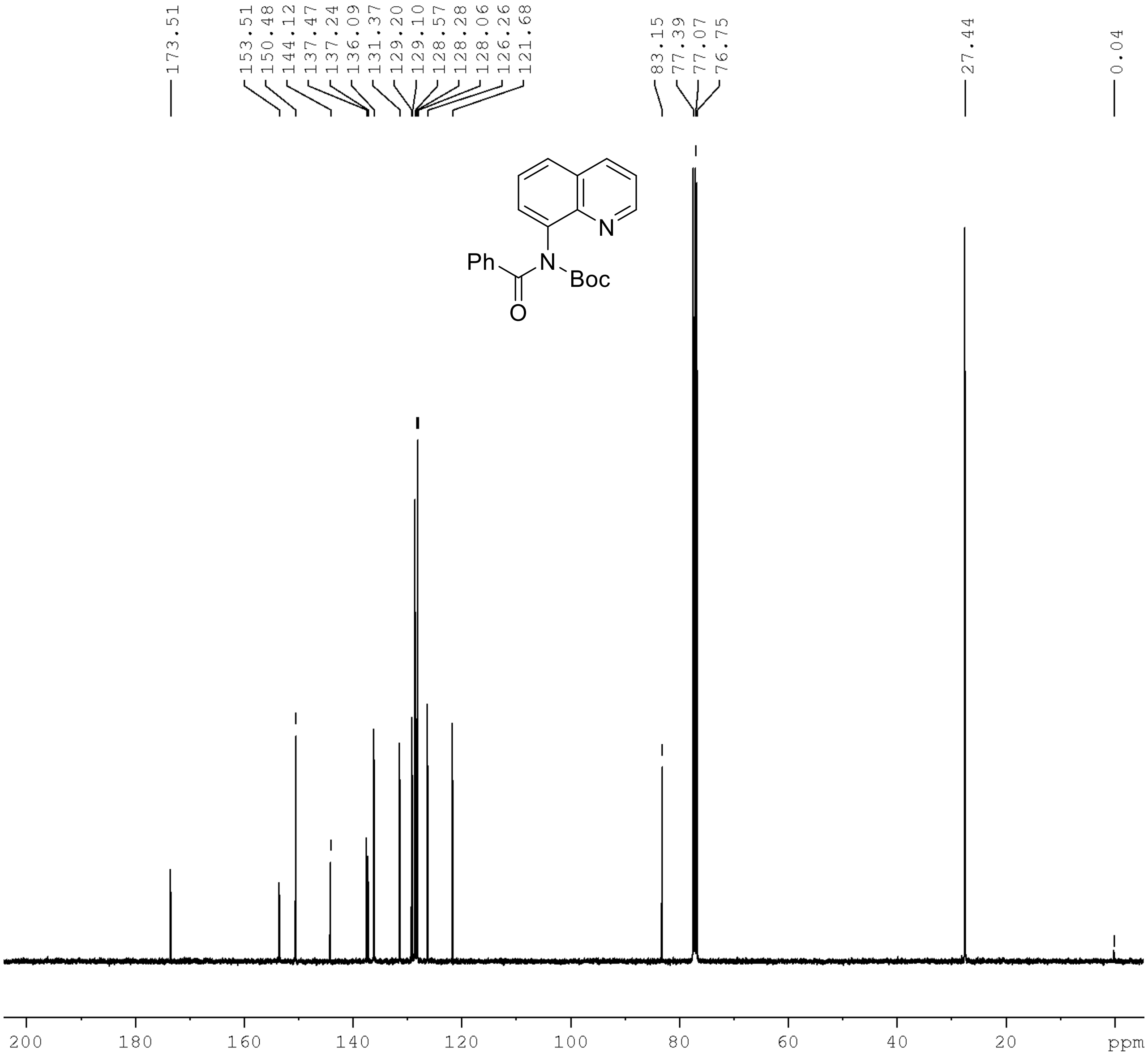

DEPT135 spectrum of compound **1y** ($CDCl_3$, 100 MHz)**:**

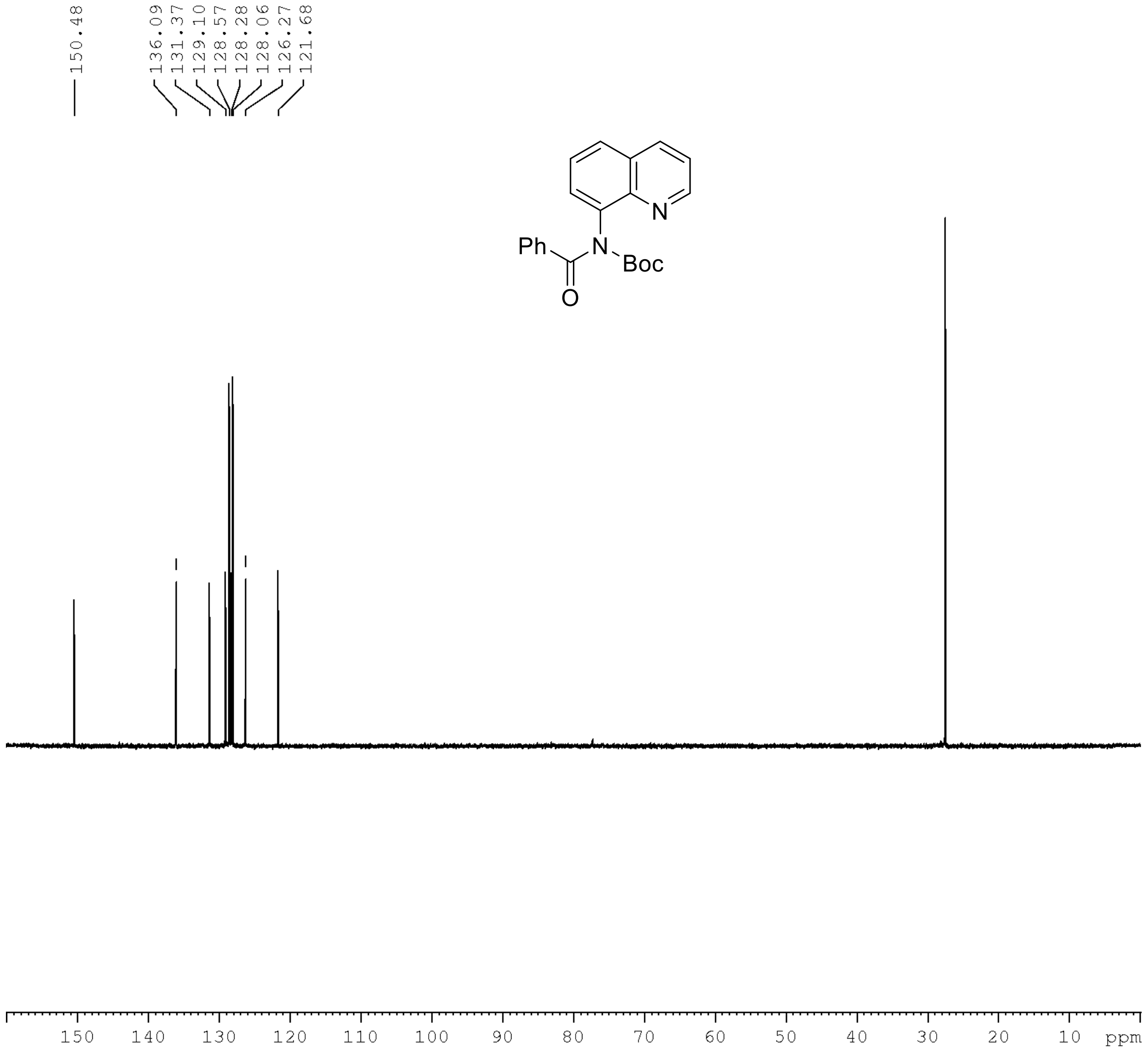

^{1}H NMR spectrum of compound **1z** ($CDCl_3$, 400 MHz)**:**

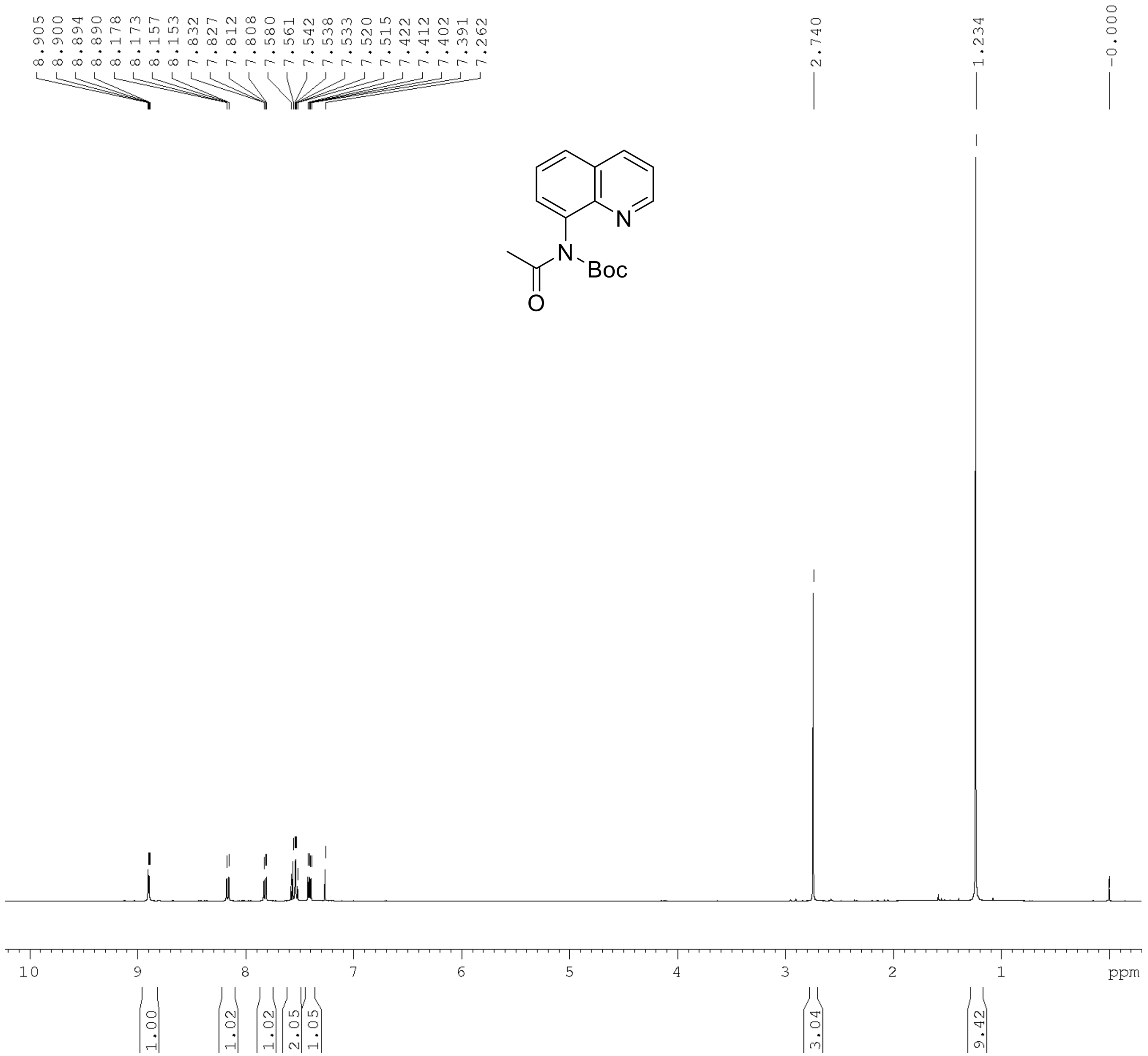

$^{13}C\{^{1}H\}$NMR spectrum of compound **1z** ($CDCl_3$, 100 MHz)**:**

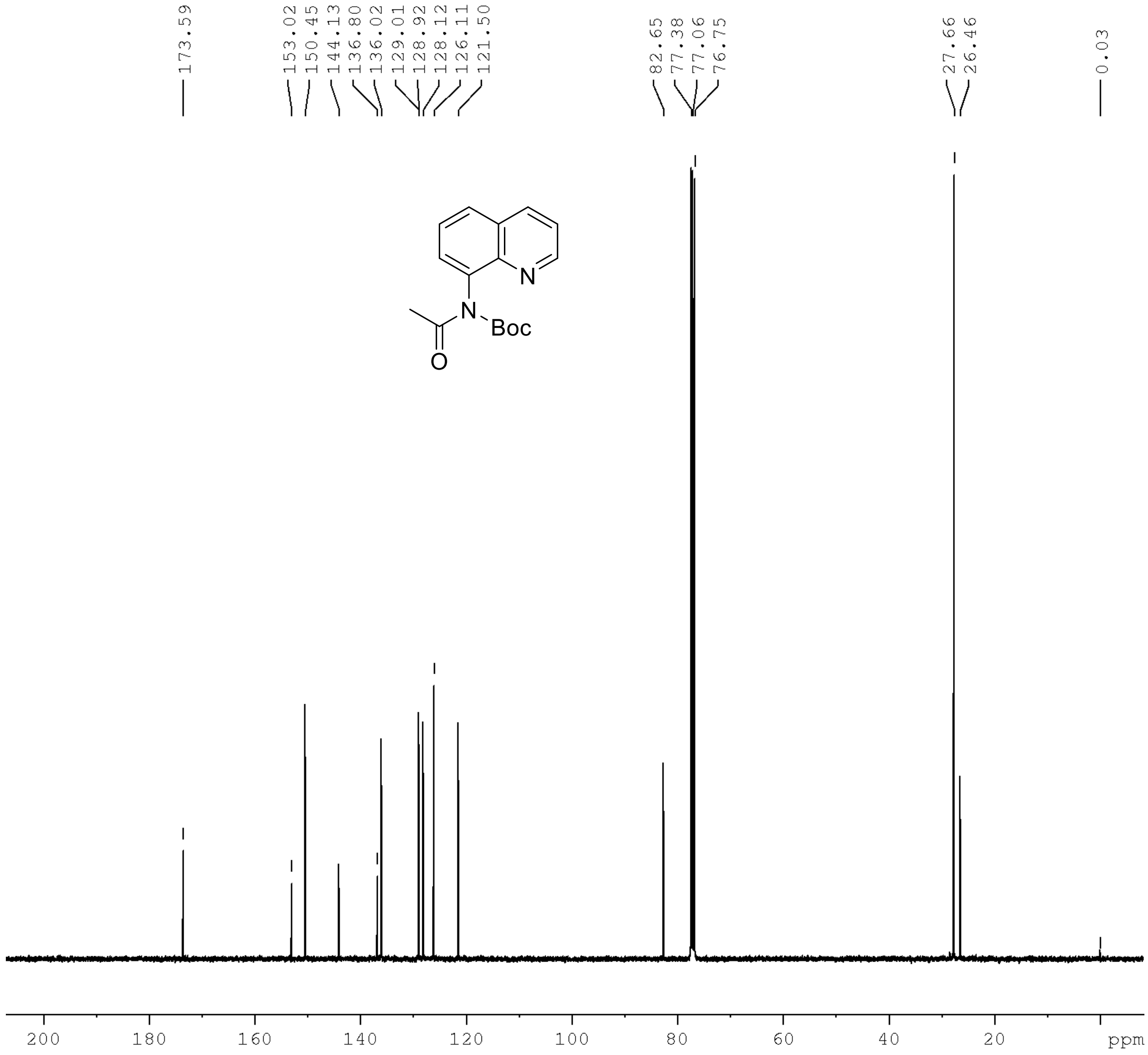

DEPT135 spectrum of compound **1z** ($CDCl_3$, 100 MHz)**:**

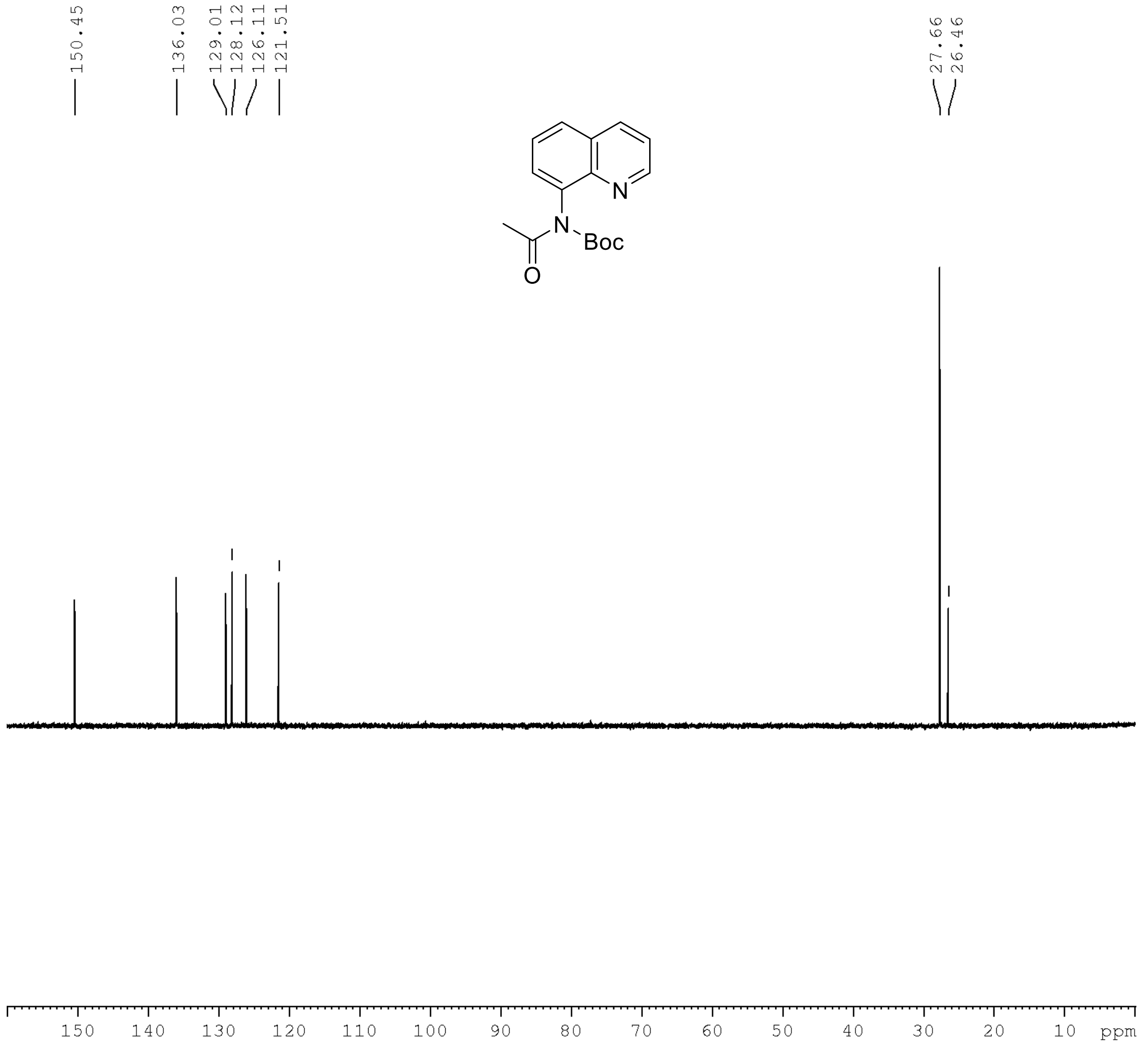

^{1}H NMR spectrum of compound **1ac** (8:2, $CDCl_3$ + DMSO-*d6*, 400 MHz)**:**

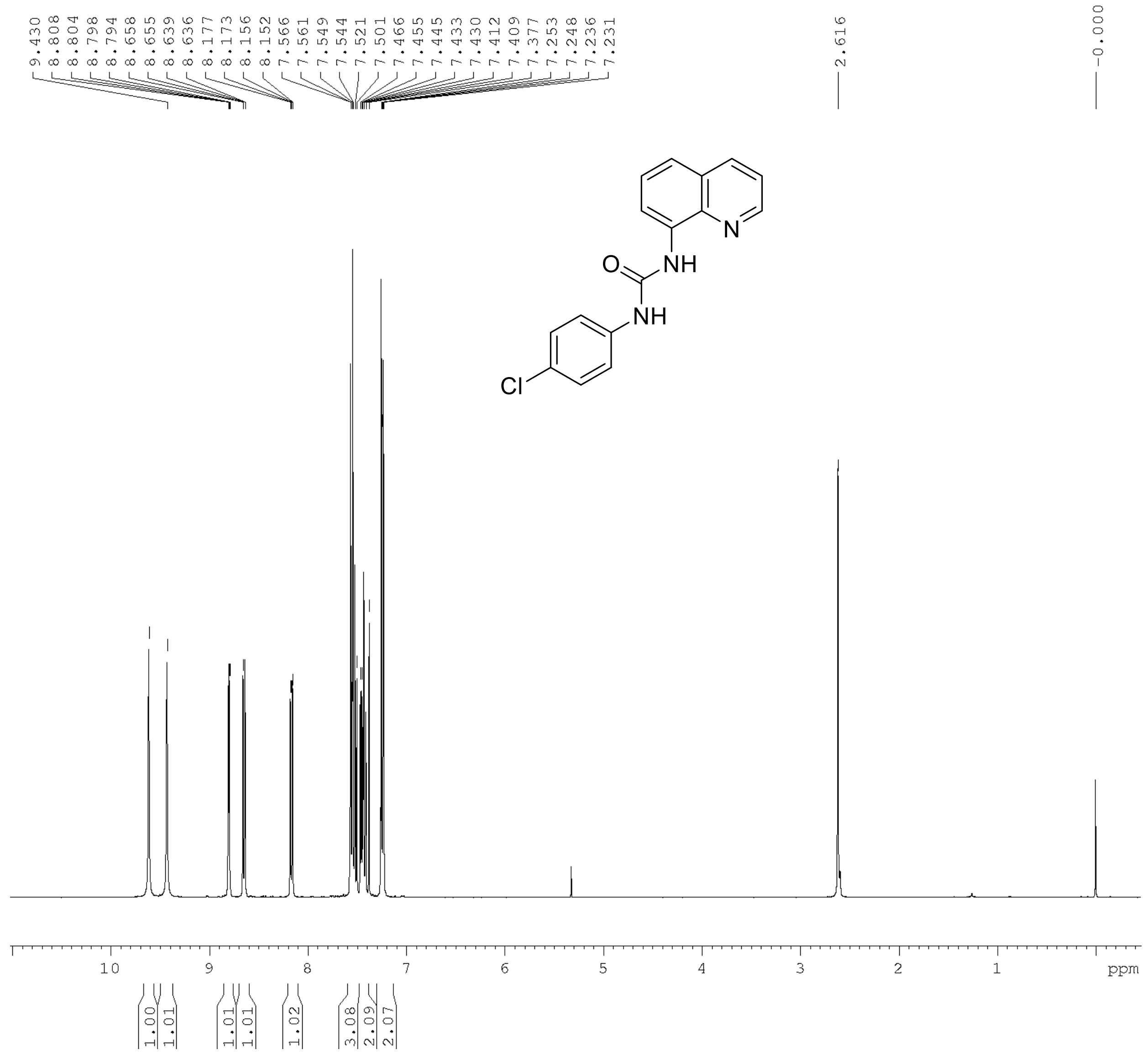

$^{13}C\{^1H\}$NMR spectrum of compound **1ac** ($CDCl_3$, 100 MHz)**:**

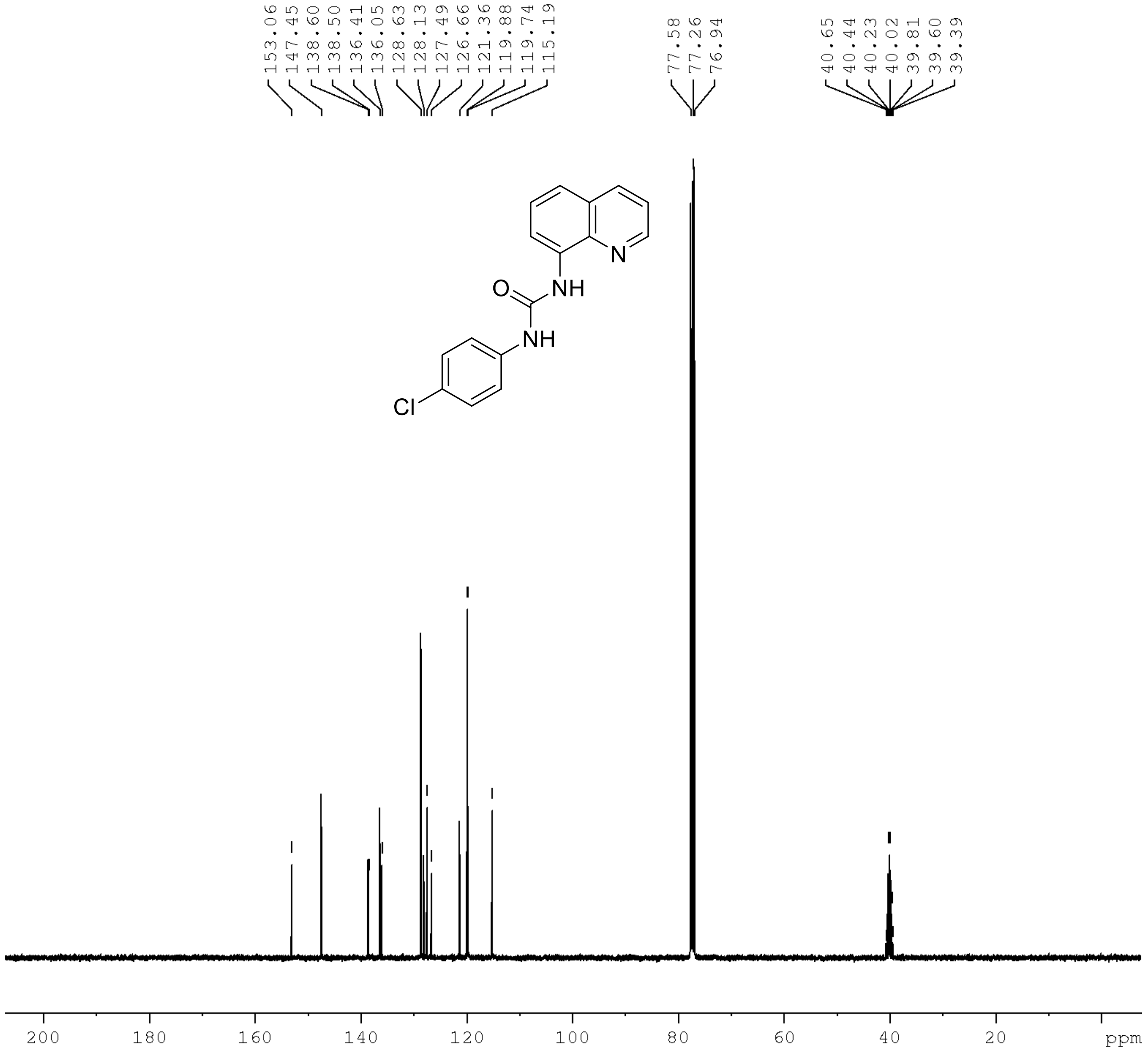

DEPT135 spectrum of compound **1ac** ($CDCl_3$, 100 MHz)**:**

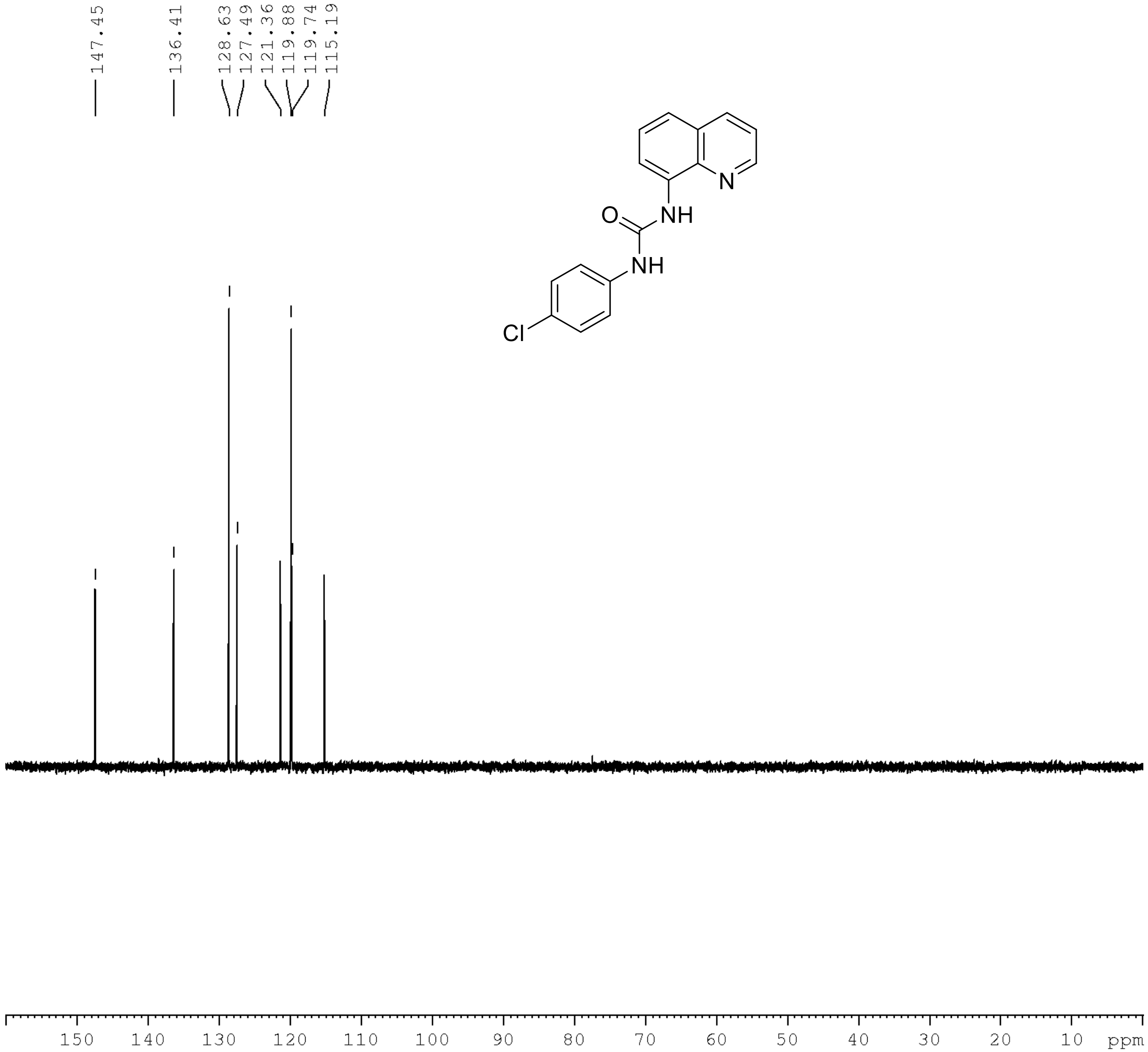

^{1}H NMR spectrum of compound **1ae** ($CDCl_3$, 400 MHz)**:**

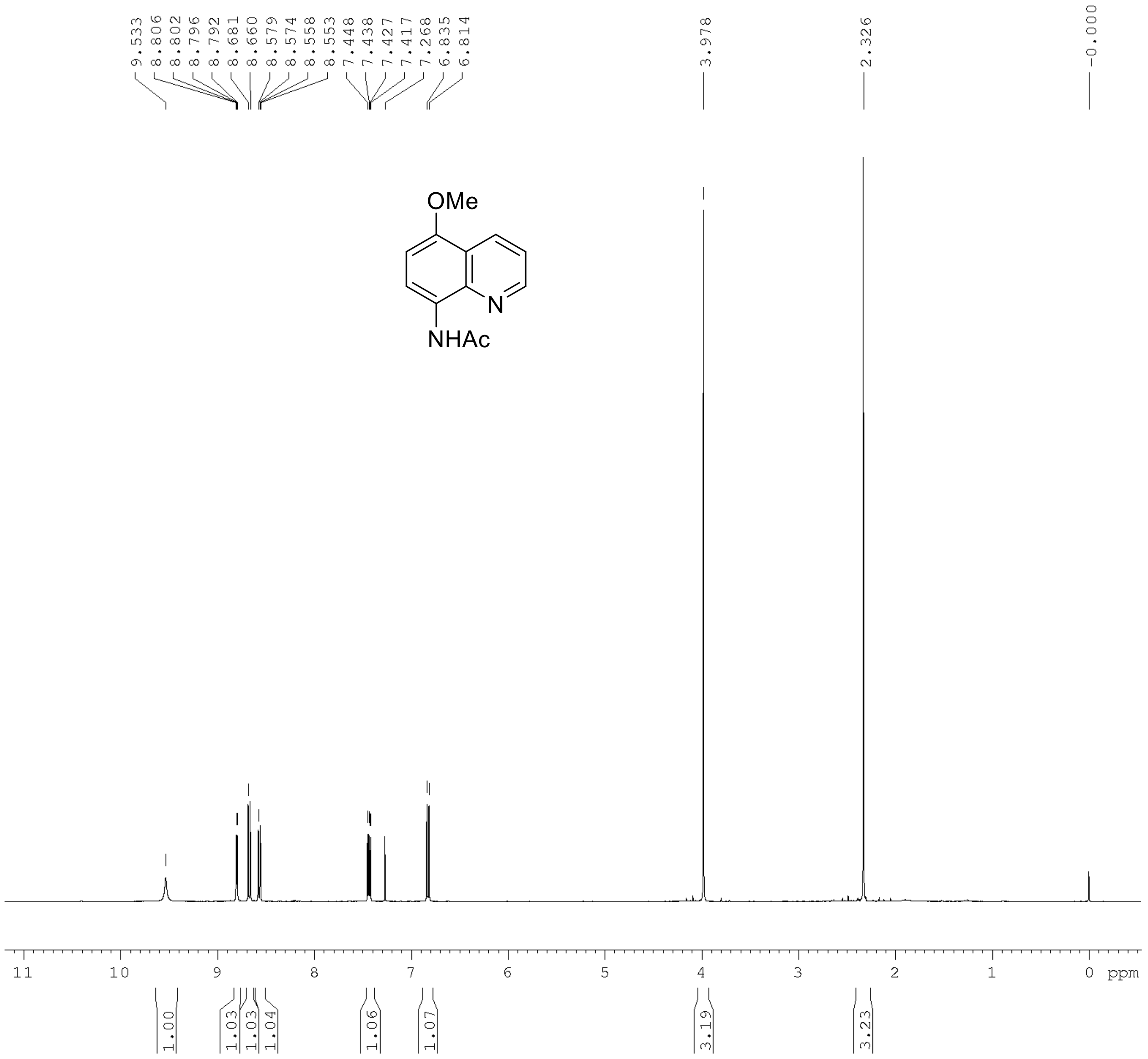

$^{13}C\{^1H\}$NMR spectrum of compound **1ae** ($CDCl_3$, 100 MHz):

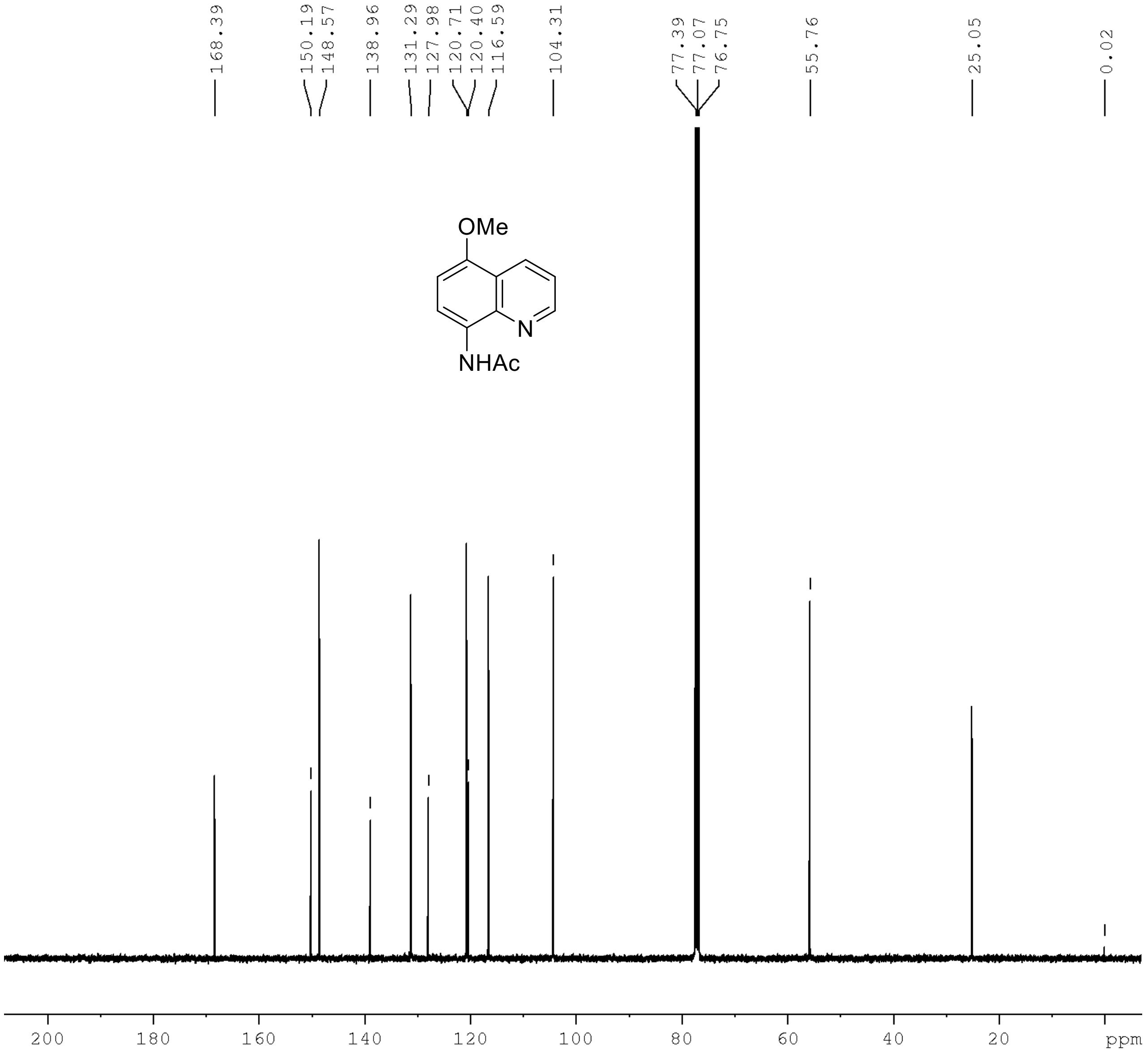

DEPT135 spectrum of compound **1ae** ($CDCl_3$, 100 MHz)**:**

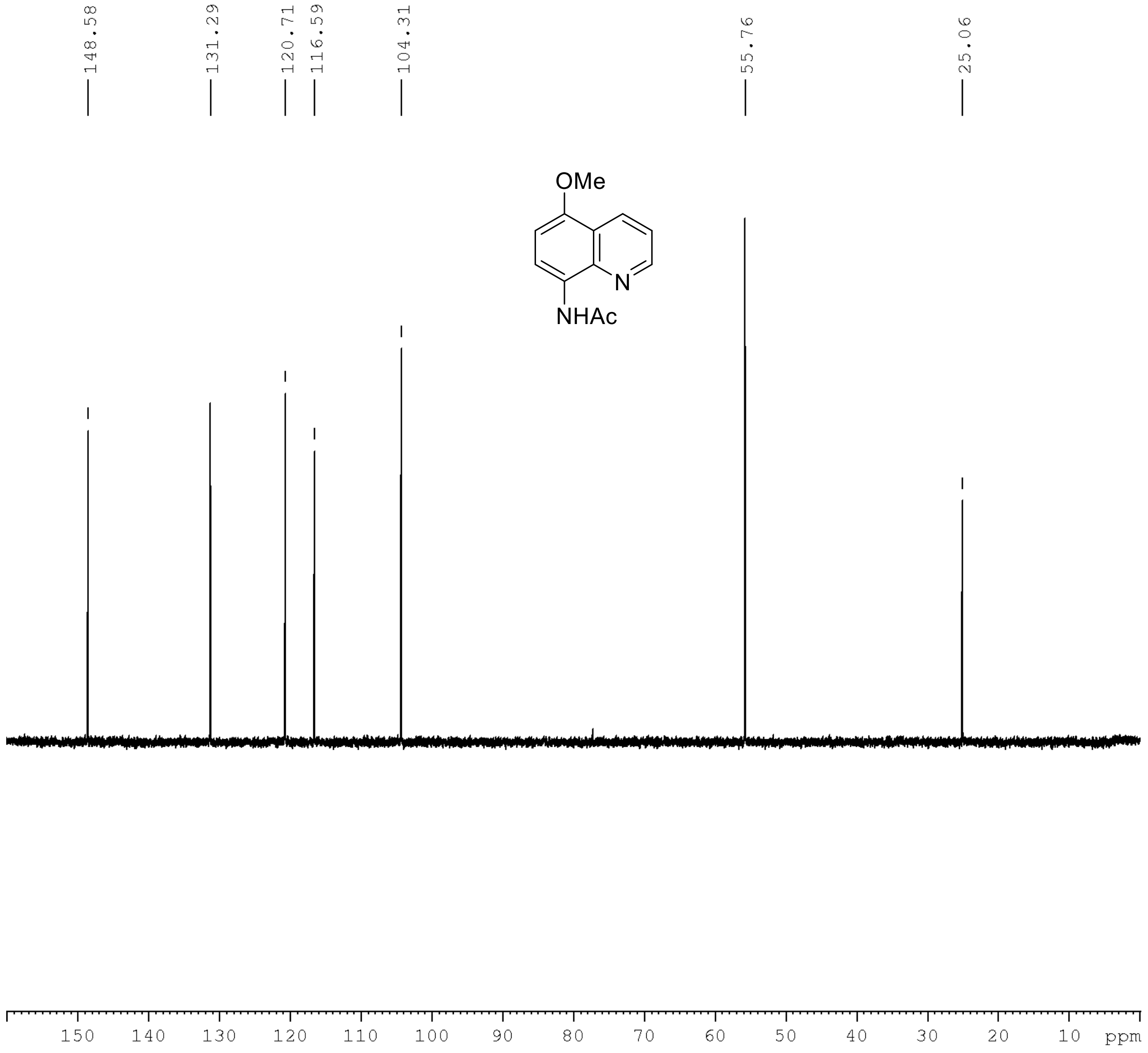

^{1}H NMR spectrum of compound **2a** ($CDCl_3$, 400 MHz)**:**

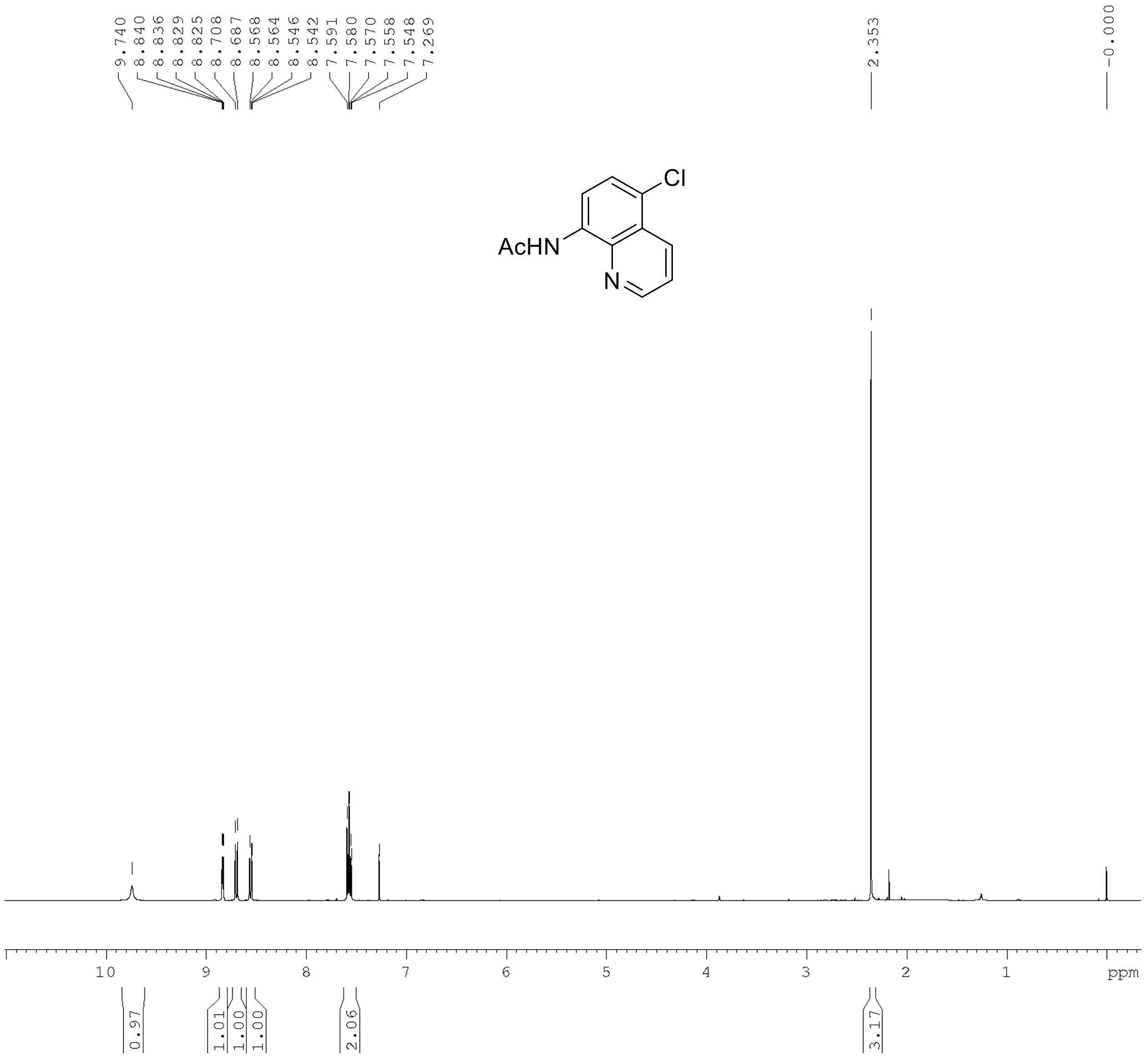

$^{13}C\{^{1}H\}$NMR spectrum of compound **2a** ($CDCl_3$, 100 MHz):

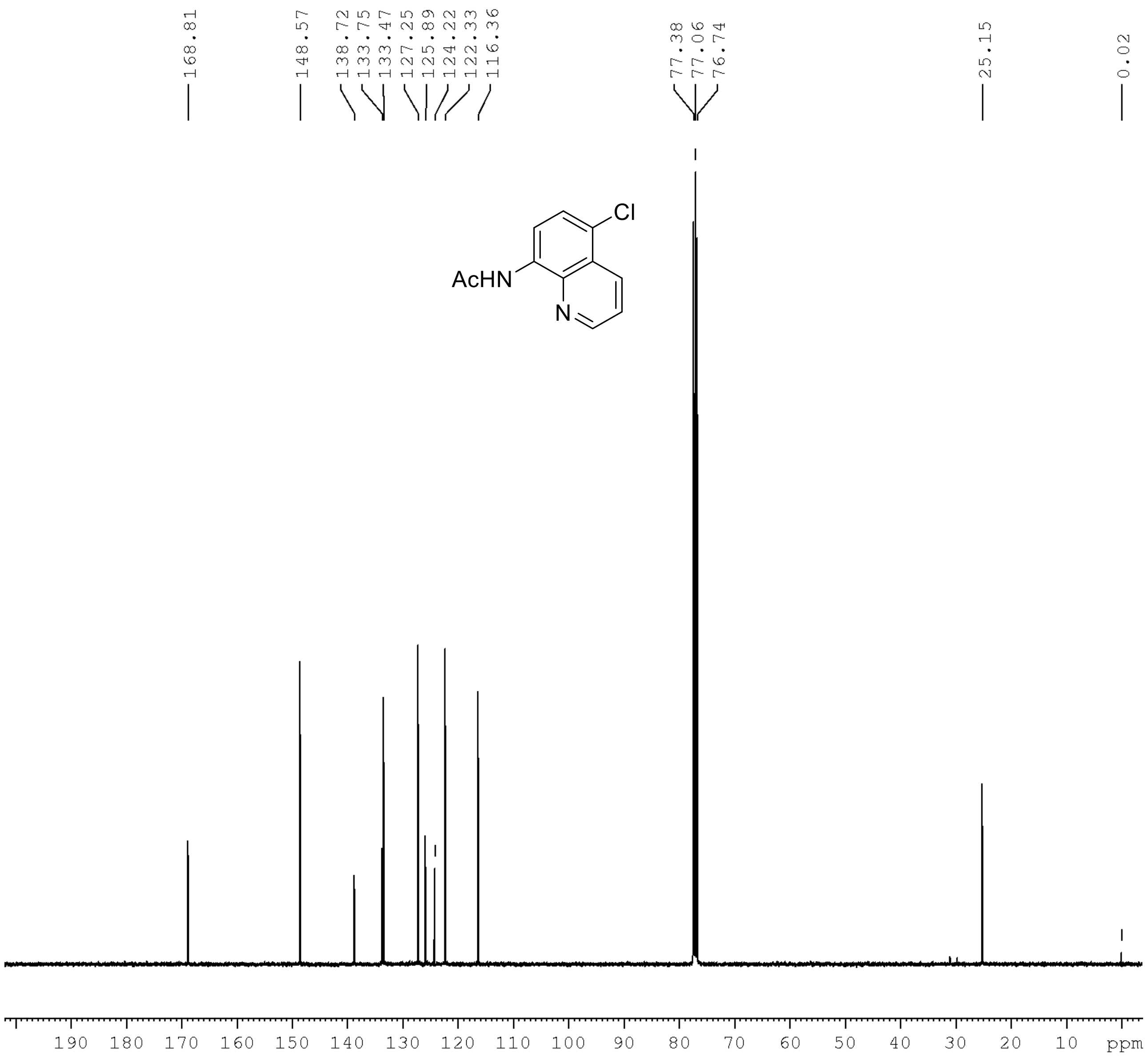

DEPT135 spectrum of compound **2a** ($CDCl_3$, 100 MHz)**:**

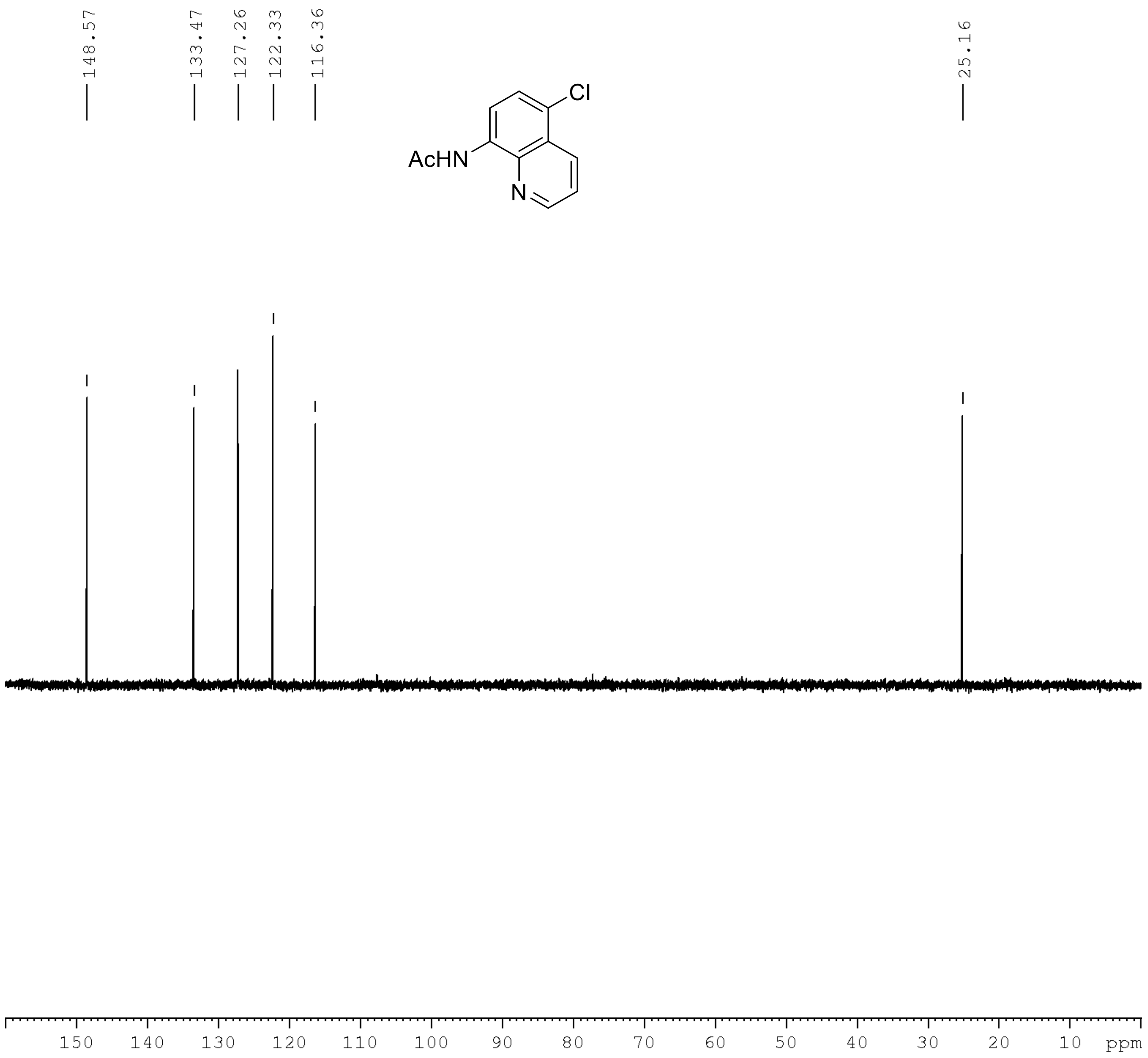

^{1}H NMR spectrum of compound **3a** ($CDCl_3$, 400 MHz)**:**

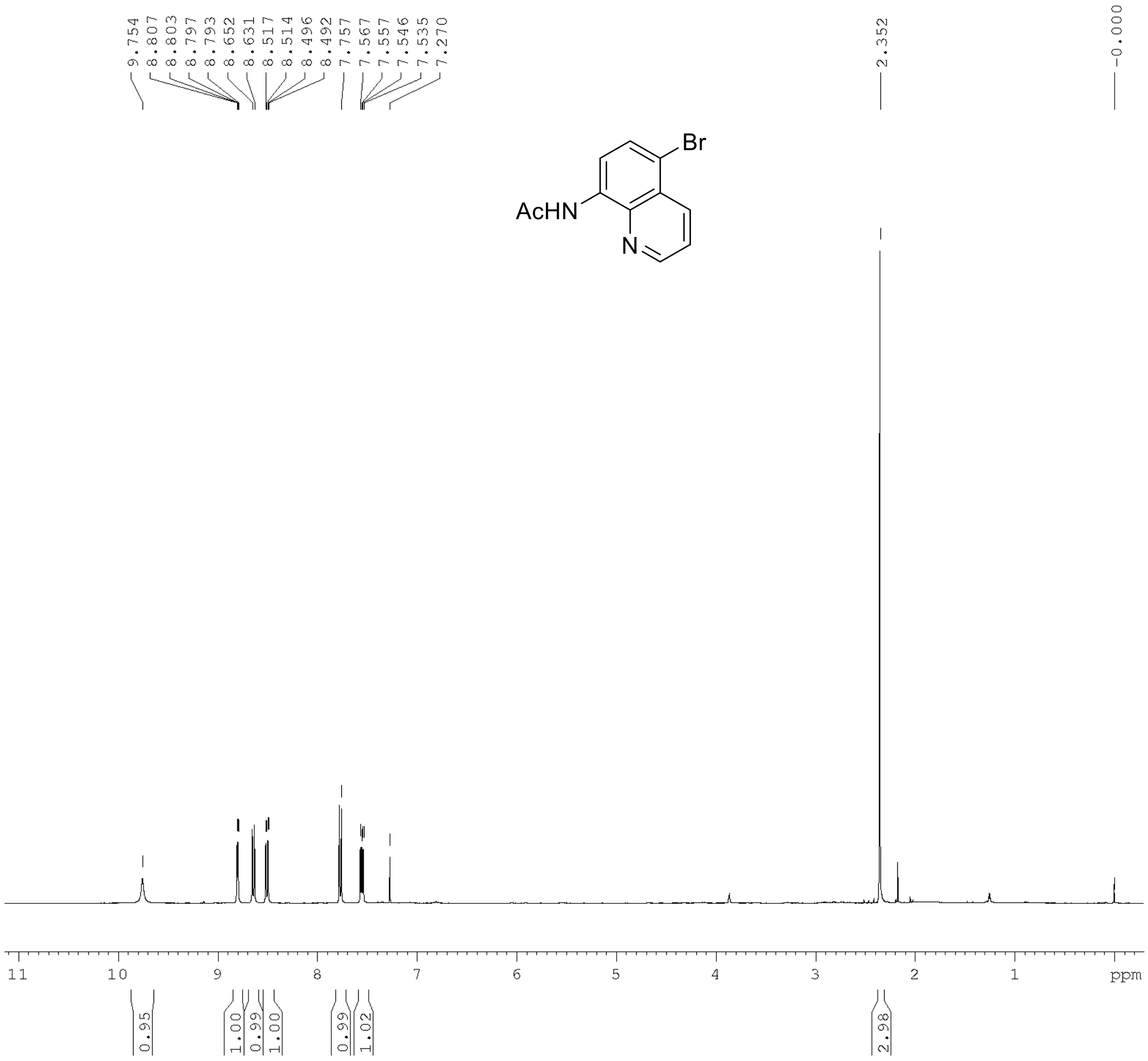

$^{13}C\{^{1}H\}$NMR spectrum of compound **3a** ($CDCl_3$, 100 MHz)**:**

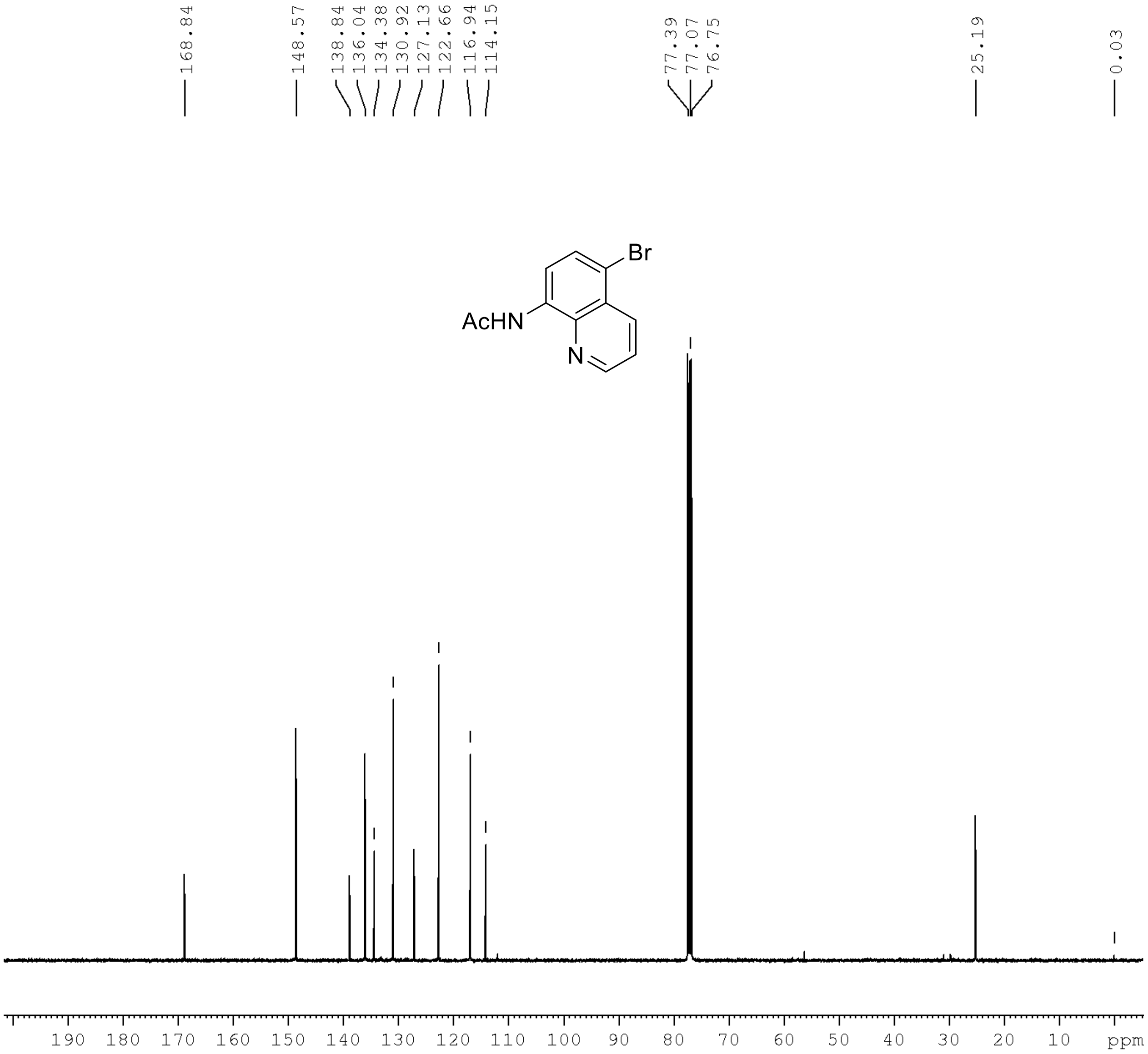

DEPT135 spectrum of compound **3a** ($CDCl_3$, 100 MHz)**:**

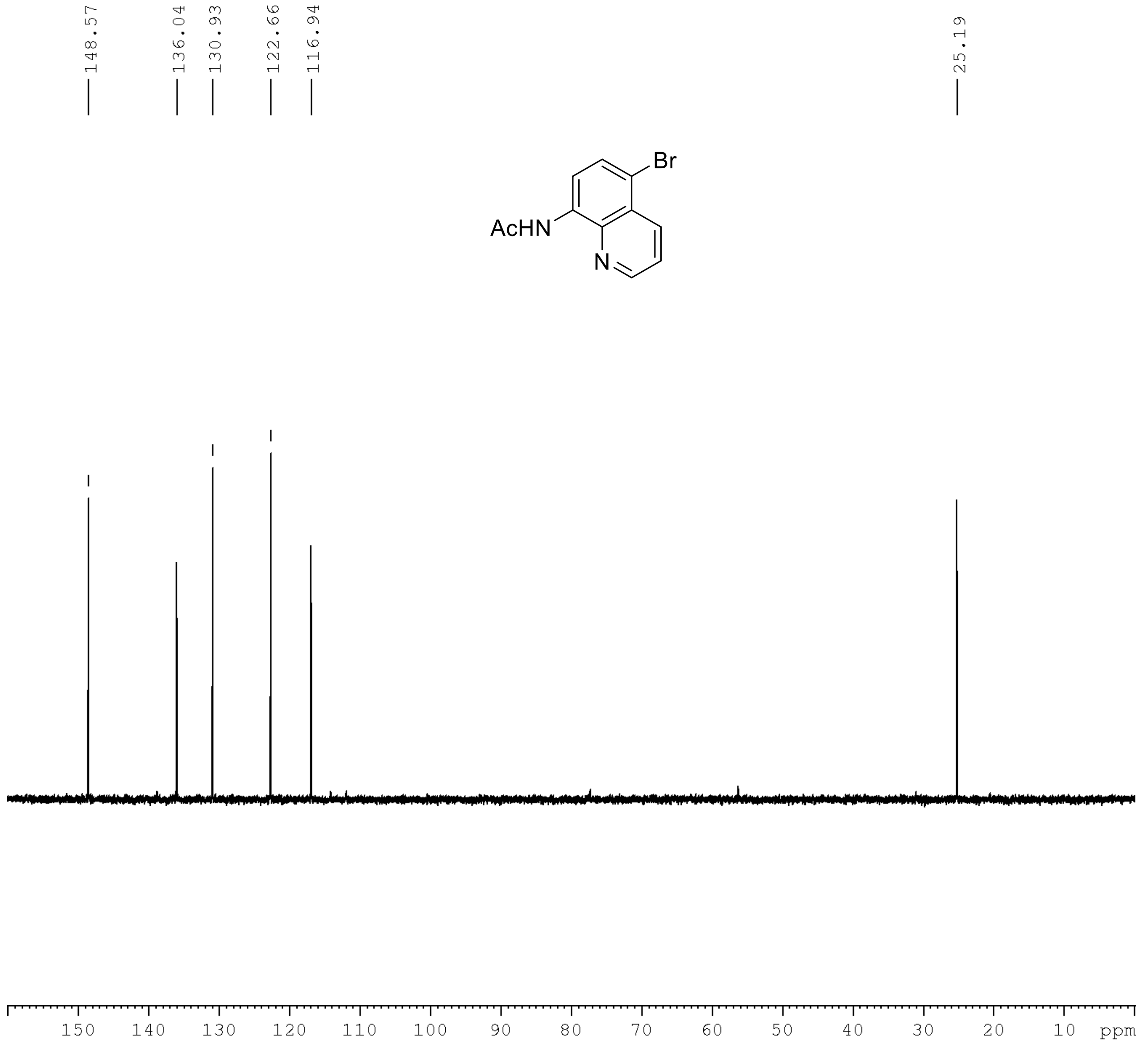

^{1}H NMR spectrum of compound **2b** ($CDCl_3$, 400 MHz)**:**

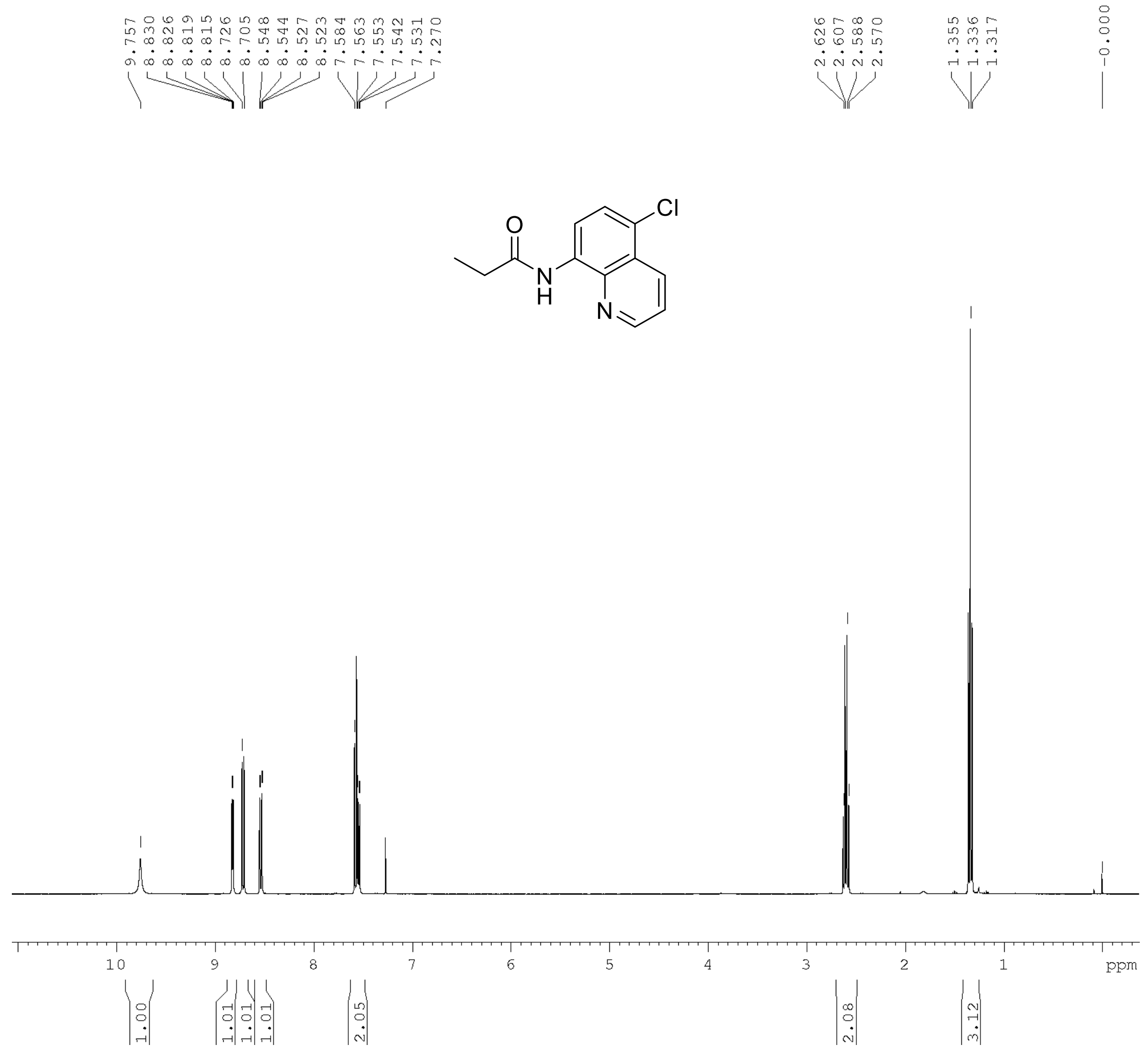

$^{13}C\{^{1}H\}$NMR spectrum of compound **2b** ($CDCl_3$, 100 MHz)**:**

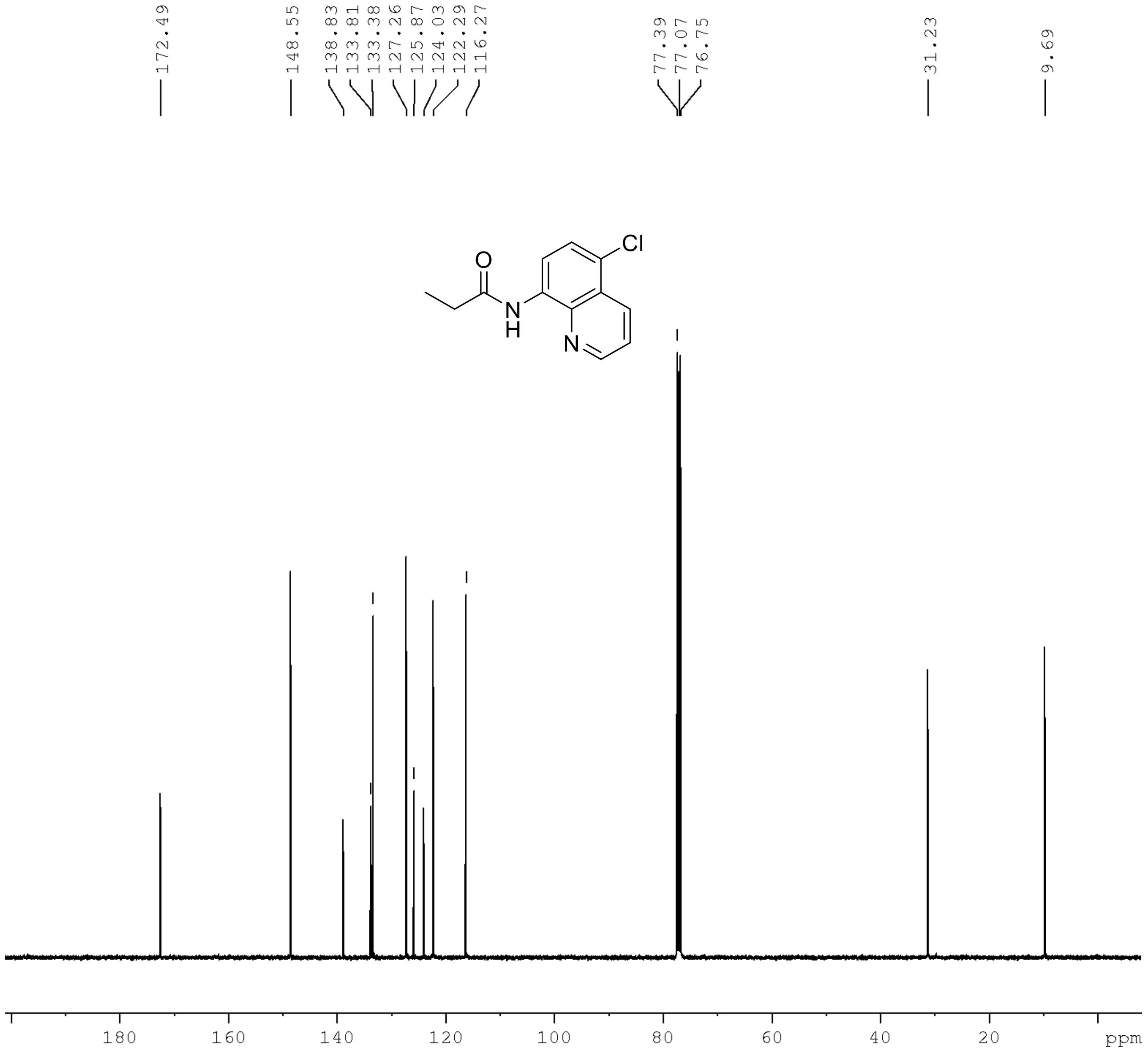

DEPT135 spectrum of compound **2b** ($CDCl_3$, 100 MHz)**:**

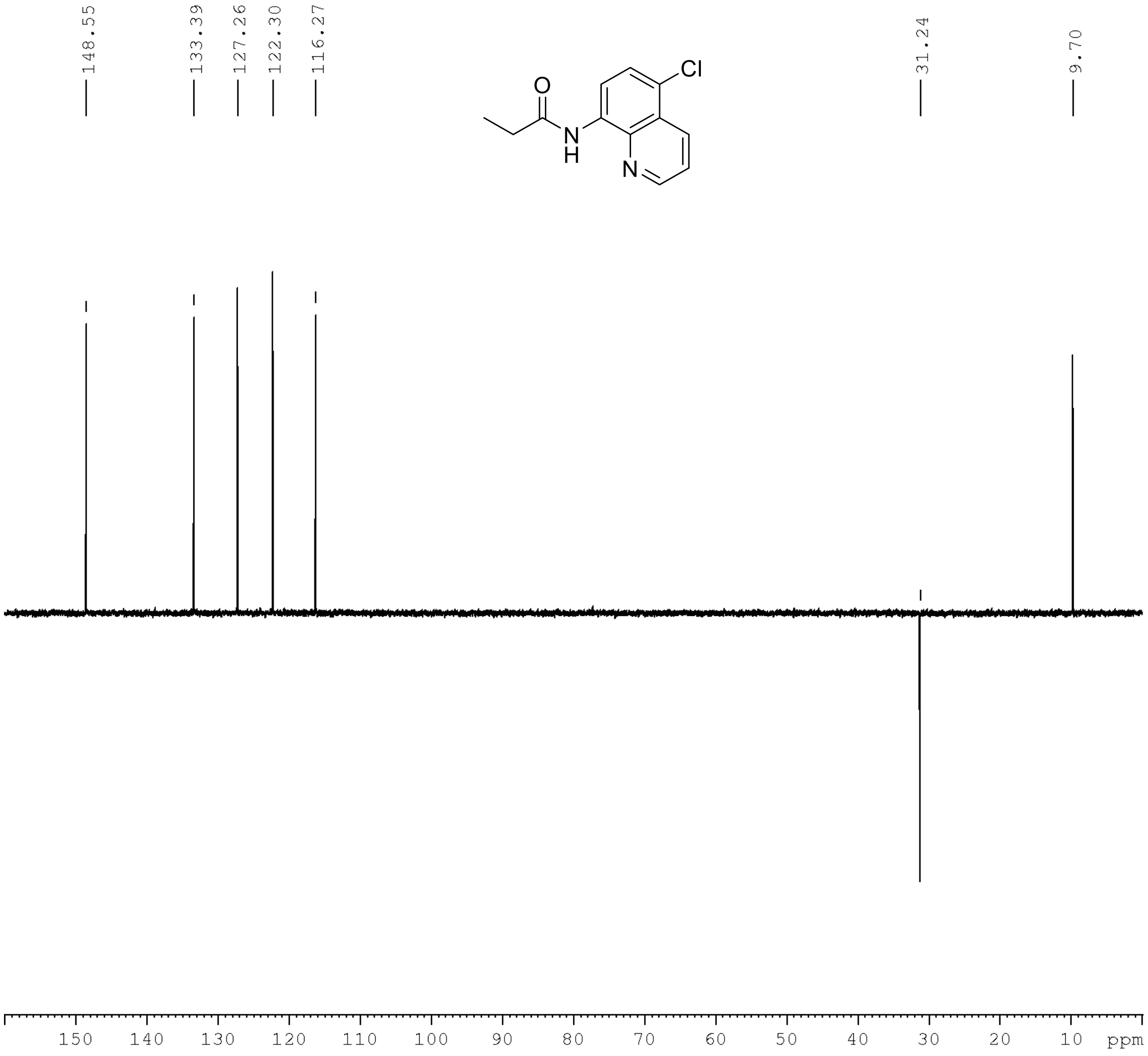

^{1}H NMR spectrum of compound **3b** ($CDCl_3$, 400 MHz)**:**

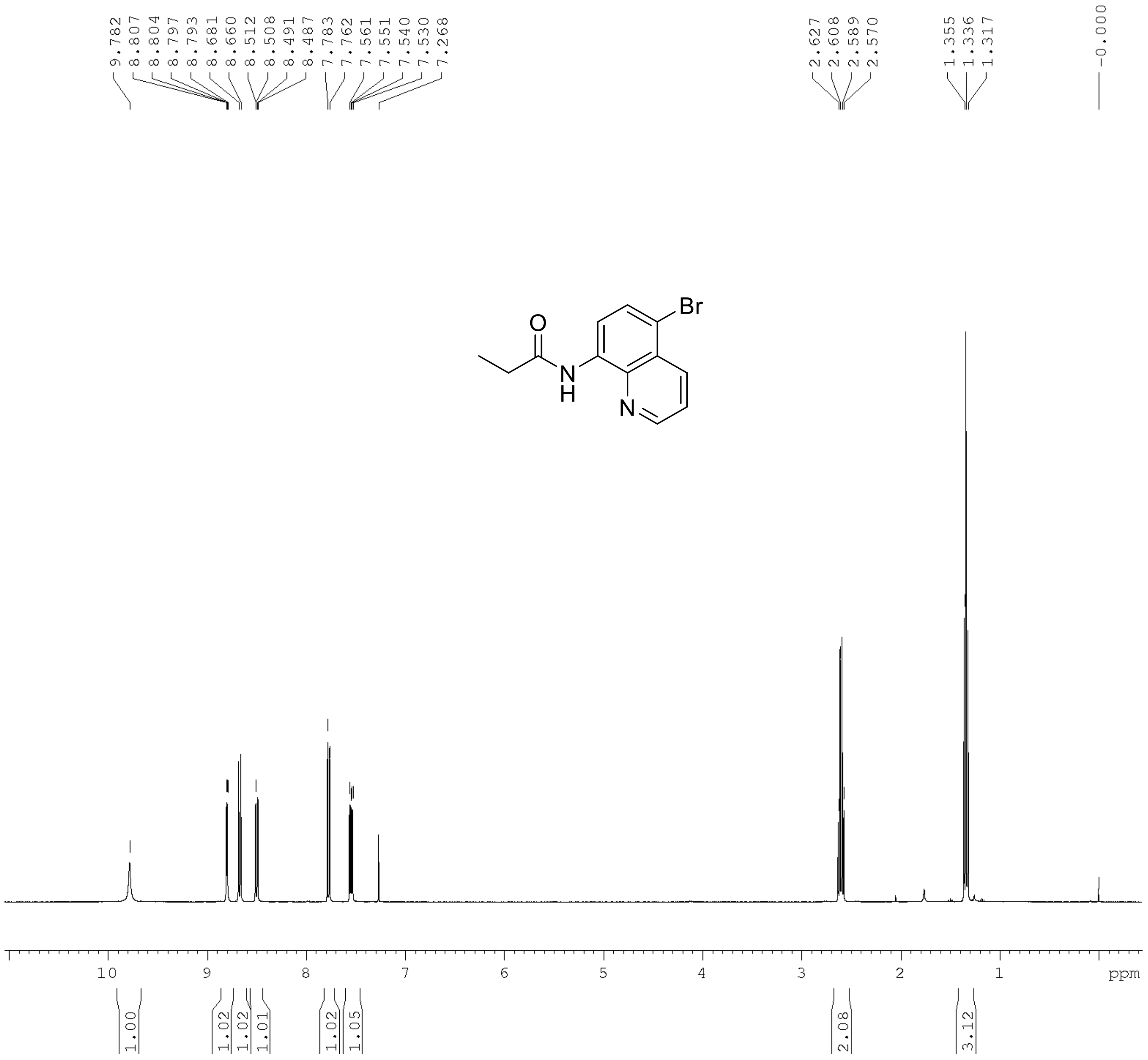

$^{13}C\{^{1}H\}$NMR spectrum of compound **3b** ($CDCl_3$, 100 MHz)**:**

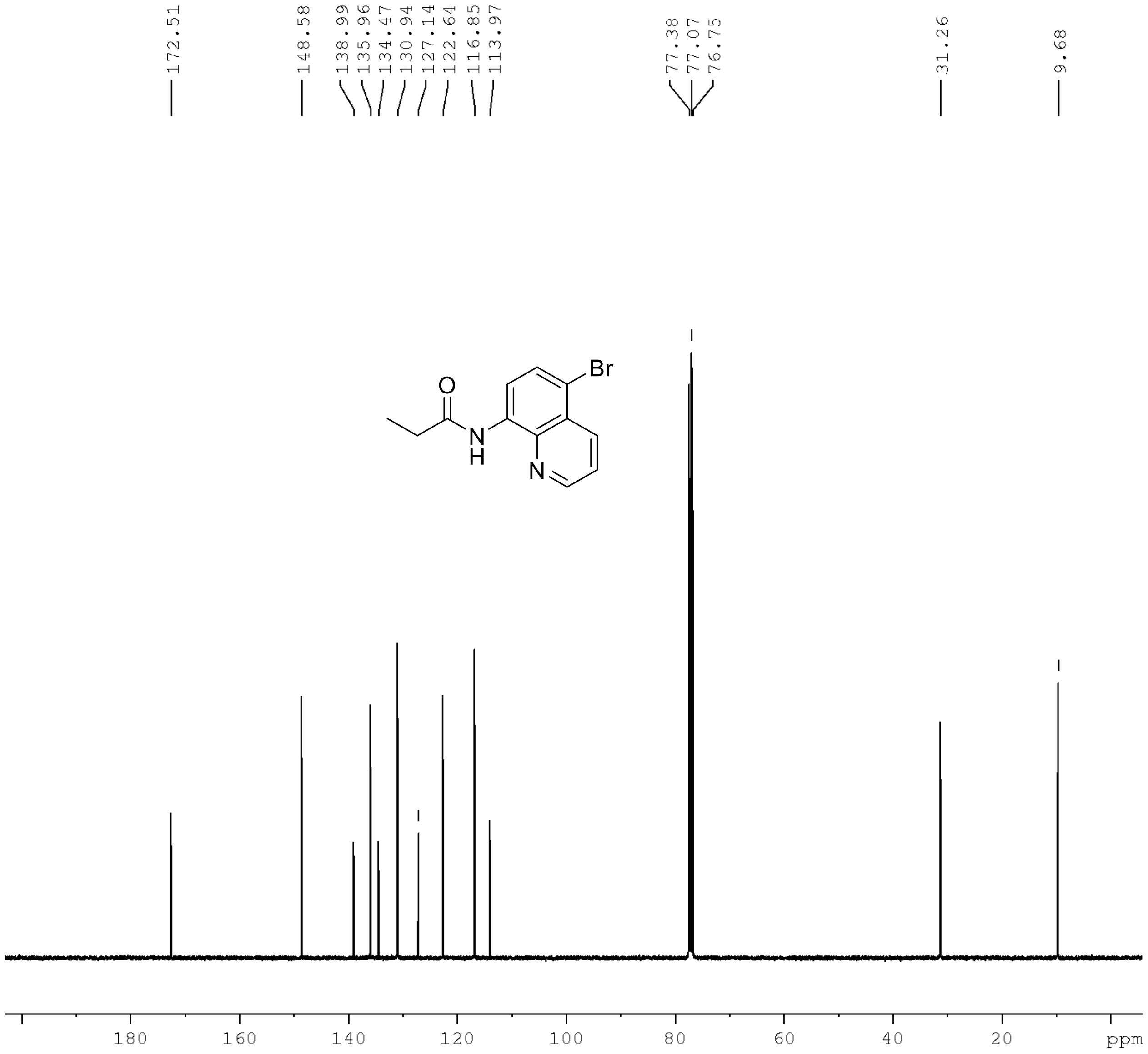

DEPT135 spectrum of compound **3b** ($CDCl_3$, 100 MHz)**:**

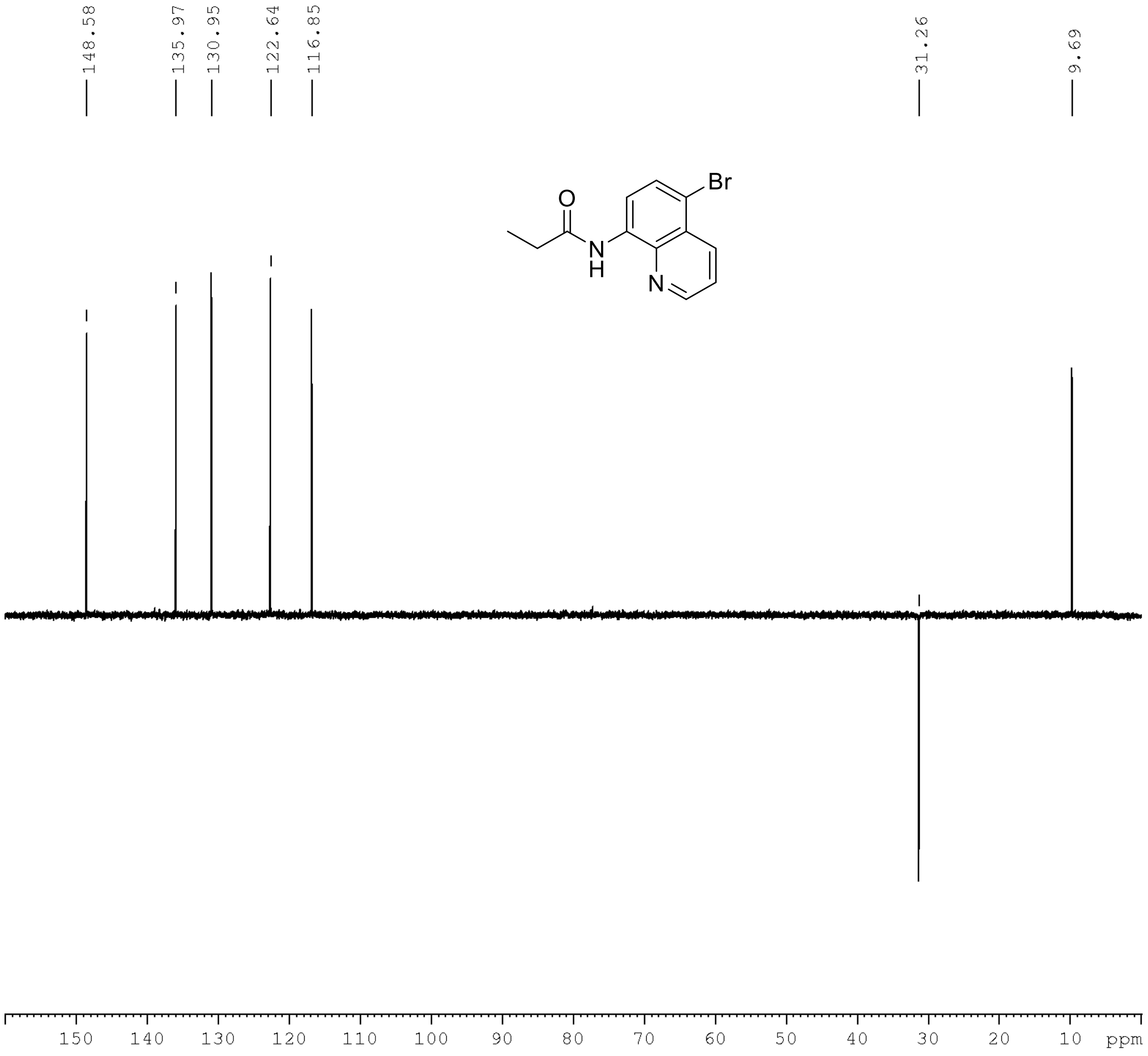

^{1}H NMR spectrum of compound **2c** ($CDCl_3$, 400 MHz)**:**

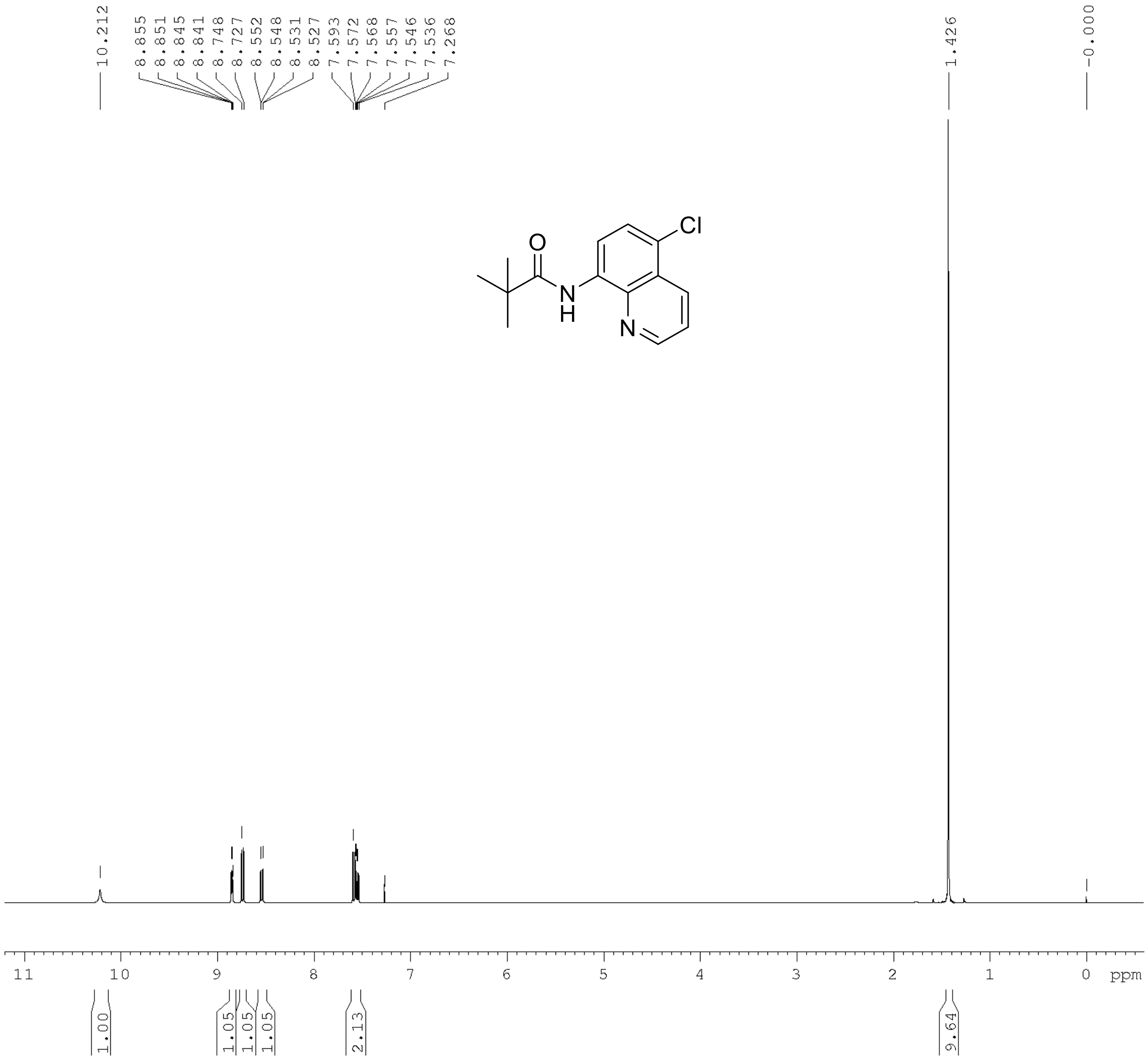

$^{13}C\{^1H\}$NMR spectrum of compound **2c** ($CDCl_3$, 100 MHz)**:**

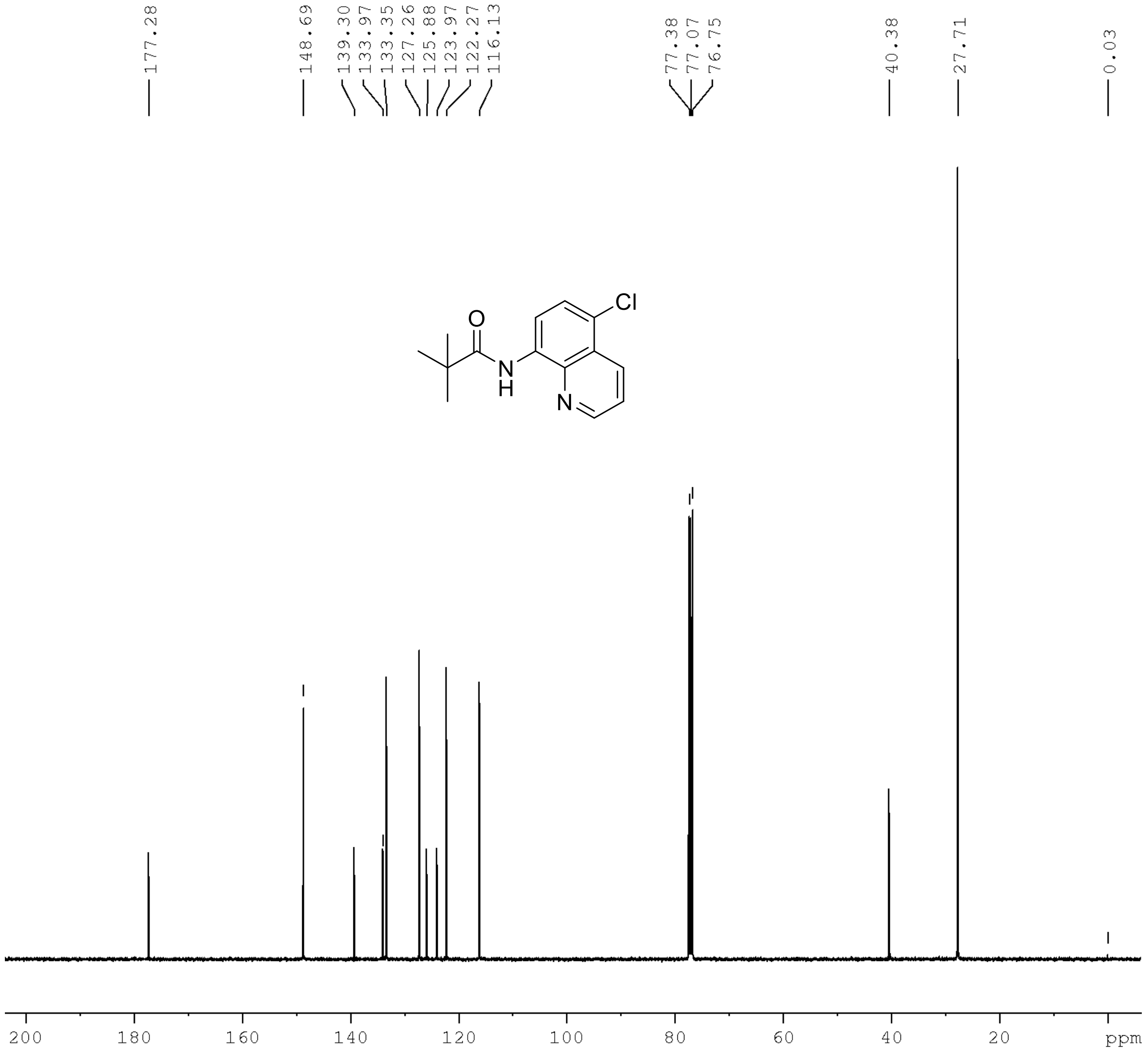

DEPT135 spectrum of compound **2c** ($CDCl_3$, 100 MHz)**:**

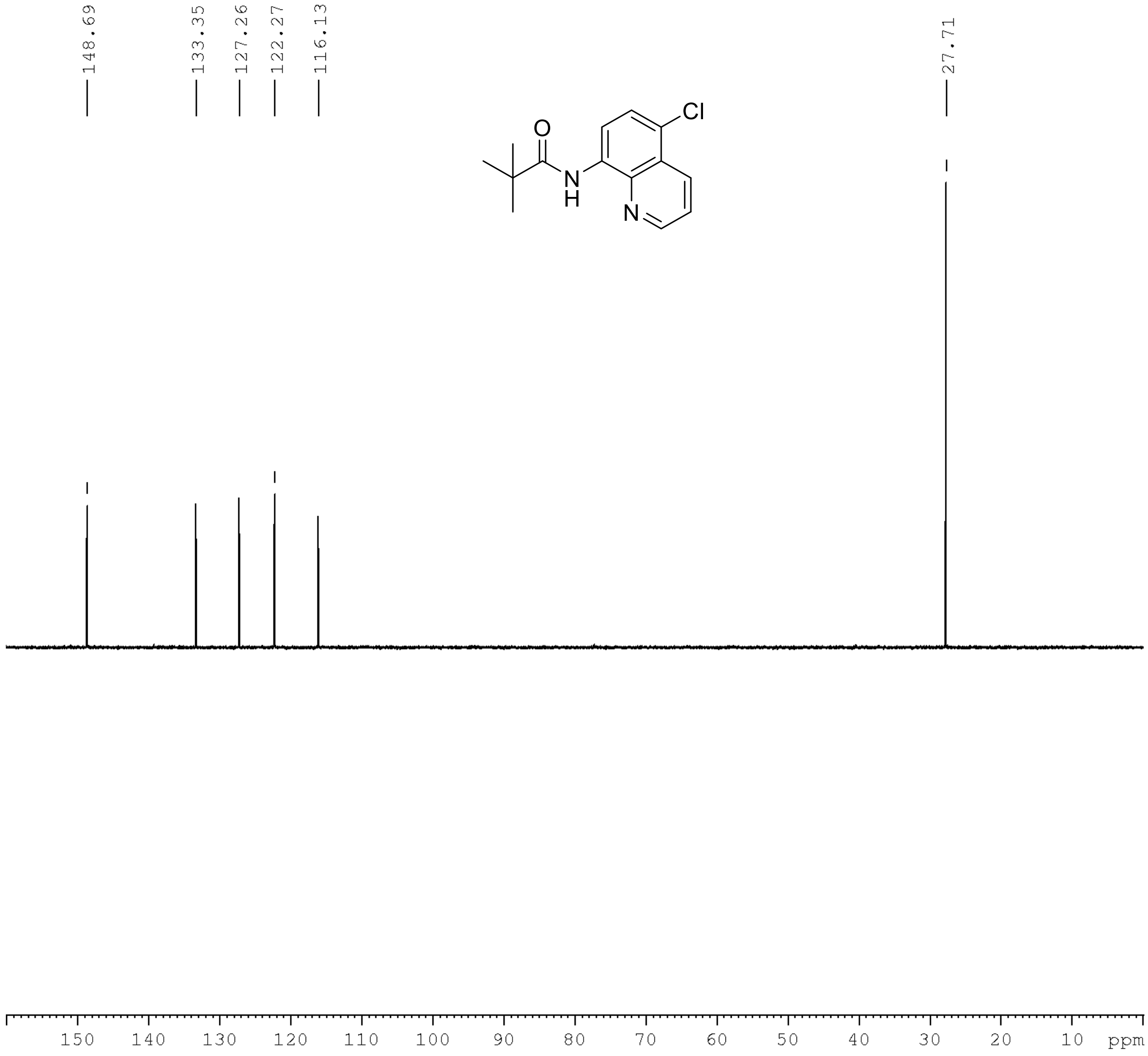

^{1}H NMR spectrum of compound **3c** ($CDCl_3$, 400 MHz)**:**

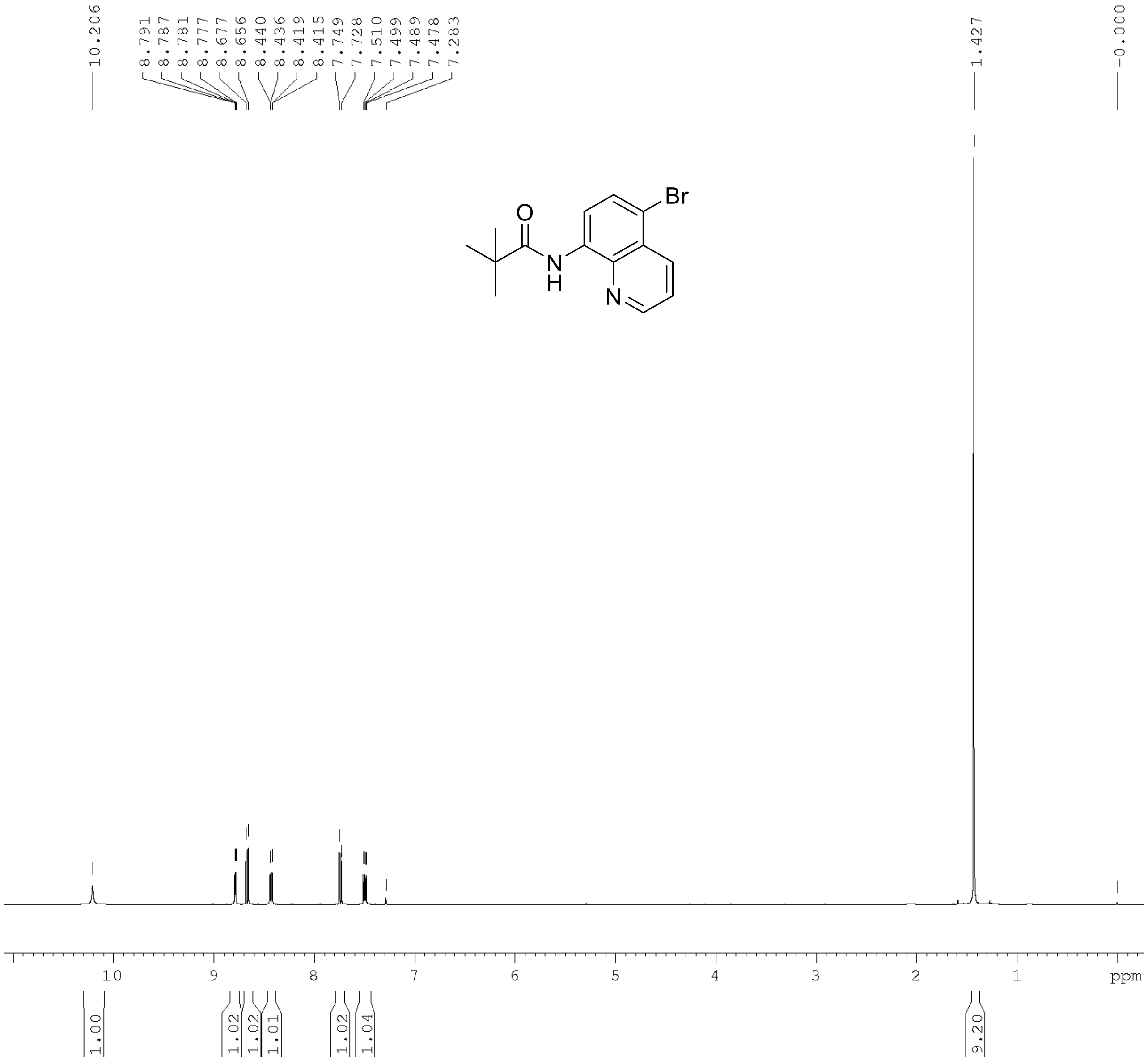

$^{13}C\{^{1}H\}$NMR spectrum of compound **3c** ($CDCl_3$, 100 MHz)**:**

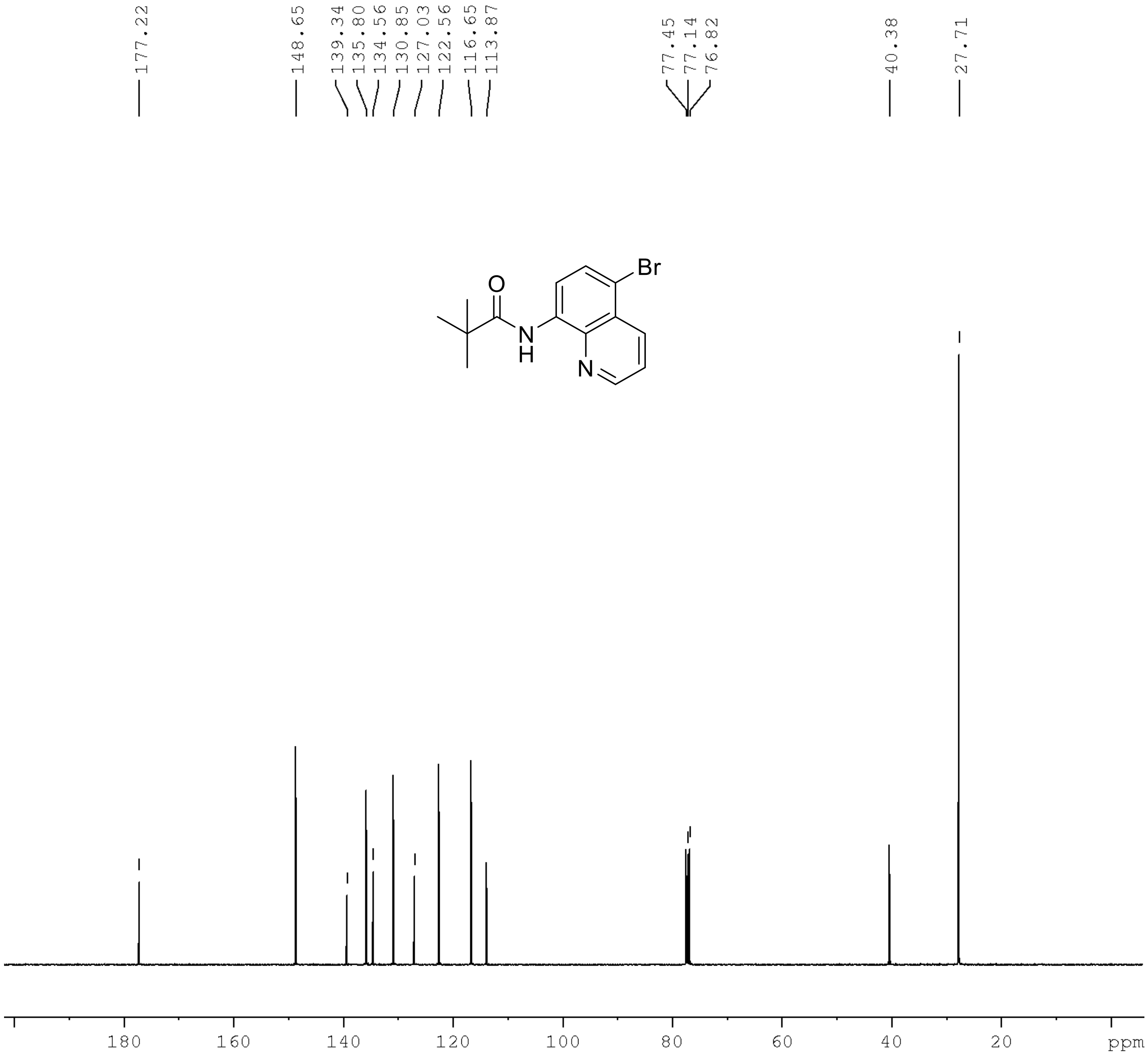

DEPT135 spectrum of compound **3c** ($CDCl_3$, 100 MHz)**:**

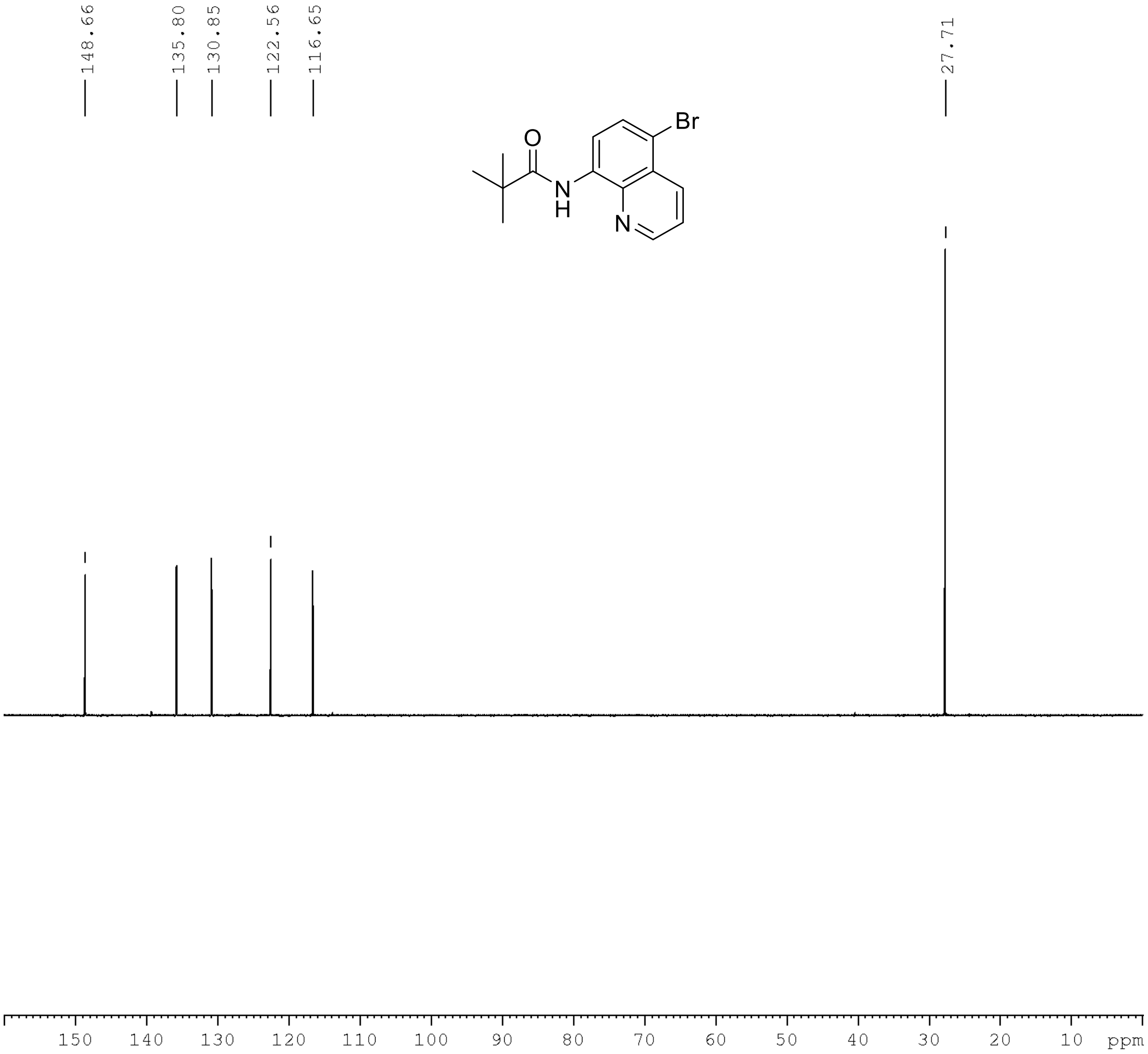

^{1}H NMR spectrum of compound **2d** ($CDCl_3$, 400 MHz)**:**

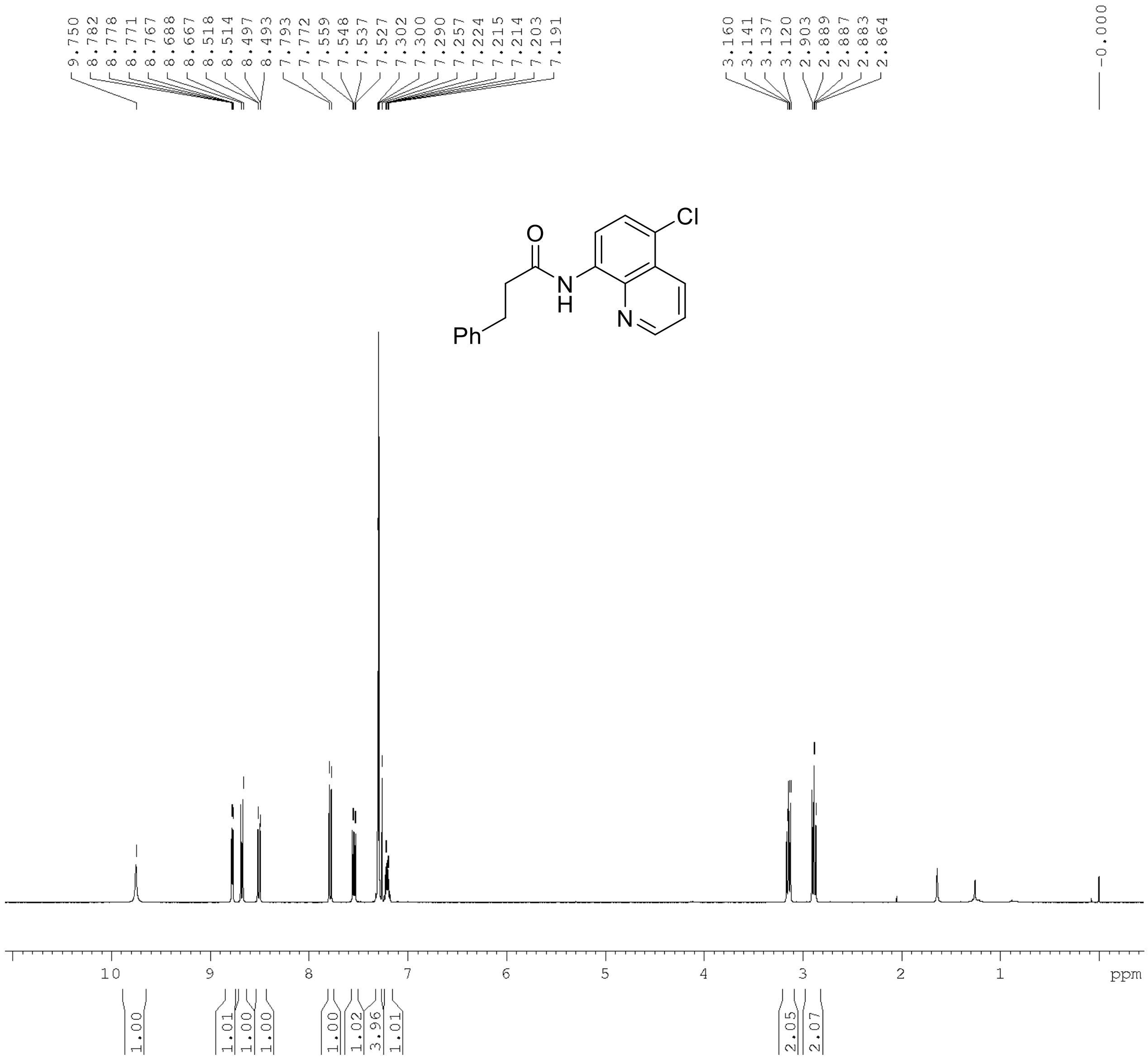

$^{13}C\{^1H\}$NMR spectrum of compound **2d** ($CDCl_3$, 100 MHz)**:**

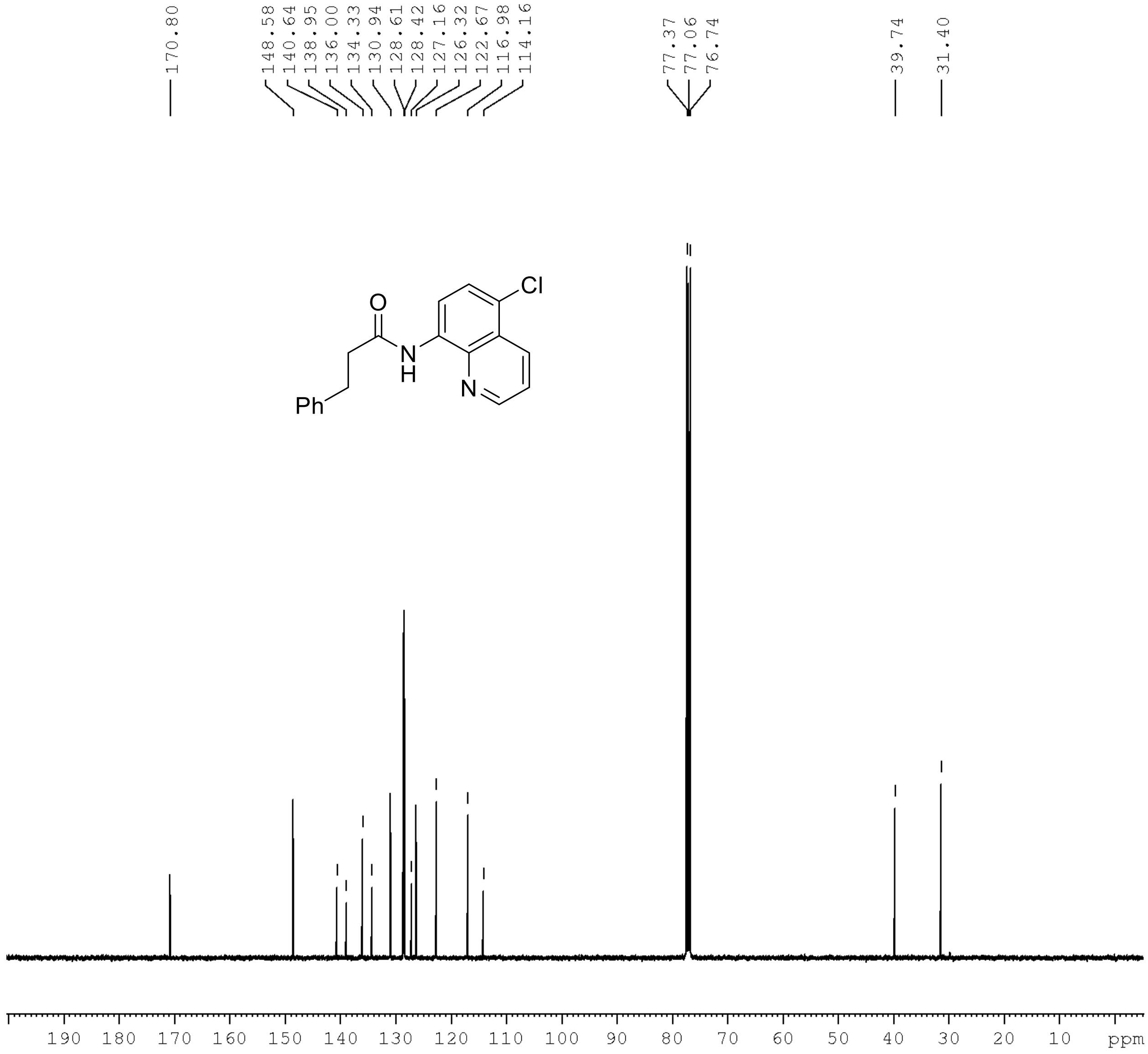

^{1}H NMR spectrum of compound **3d** ($CDCl_3$, 400 MHz):

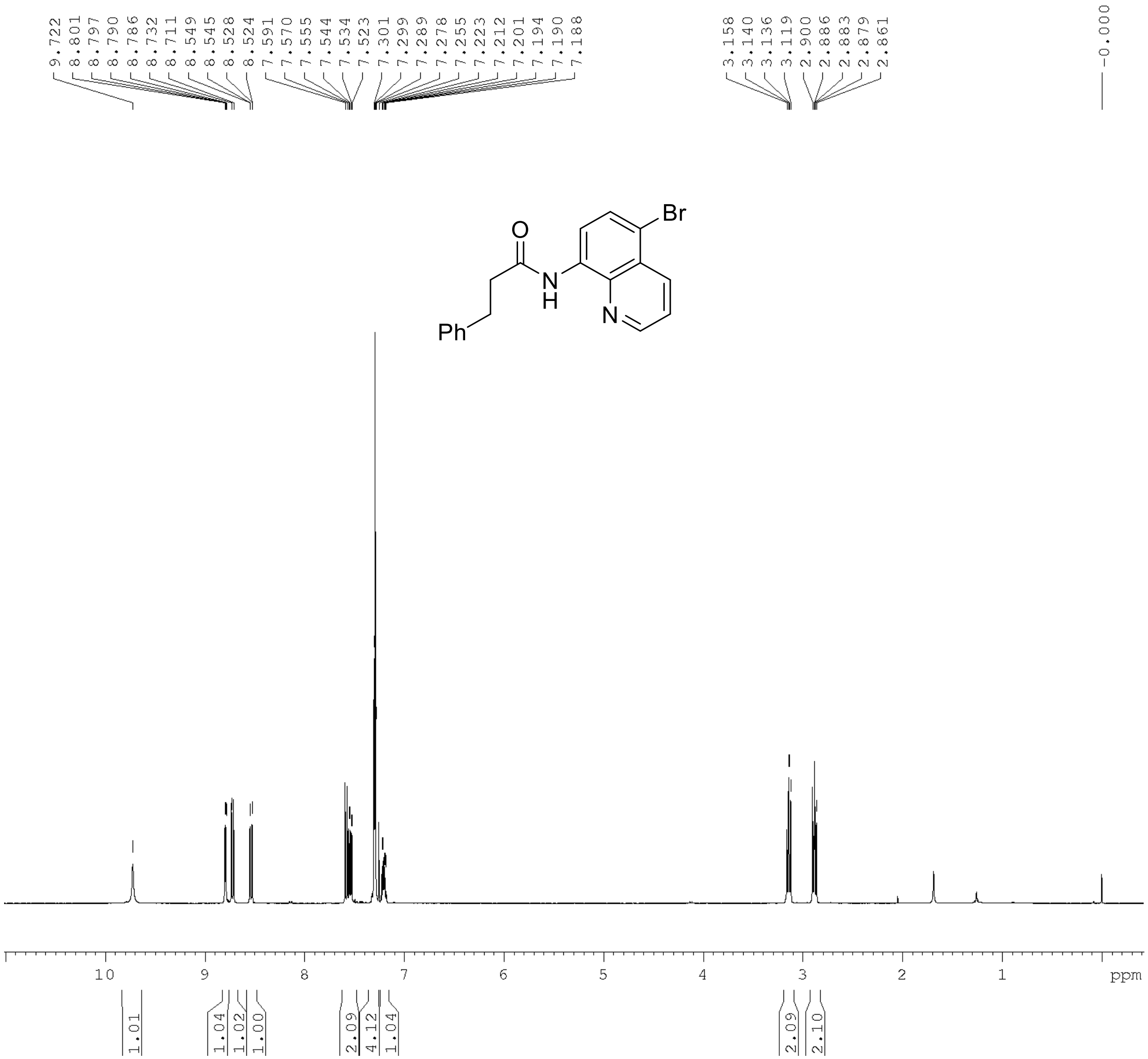

$^{13}C\{^1H\}$NMR spectrum of compound **3d** ($CDCl_3$, 100 MHz):

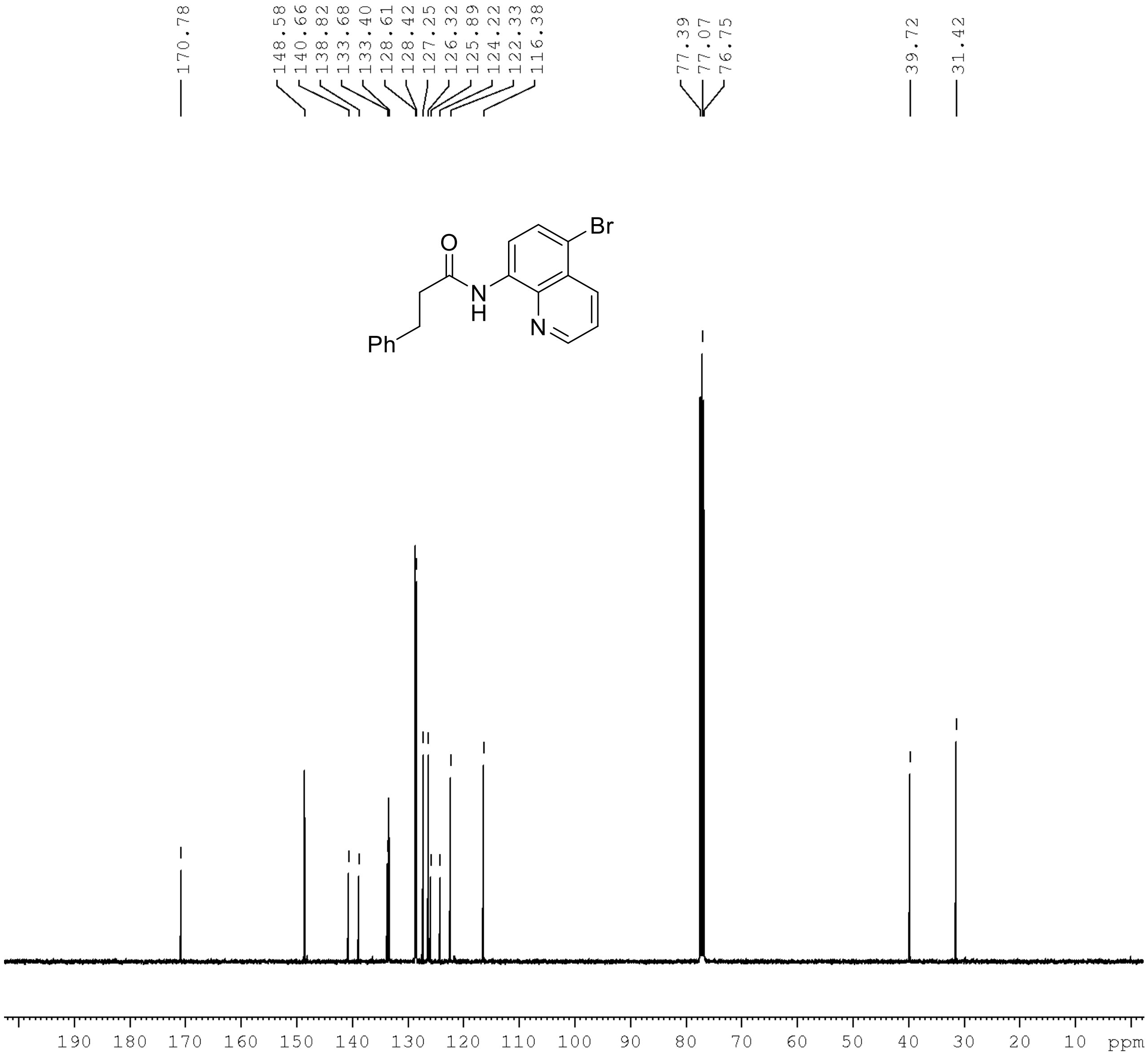

^{1}H NMR spectrum of compound **2e** ($CDCl_3$, 400 MHz):

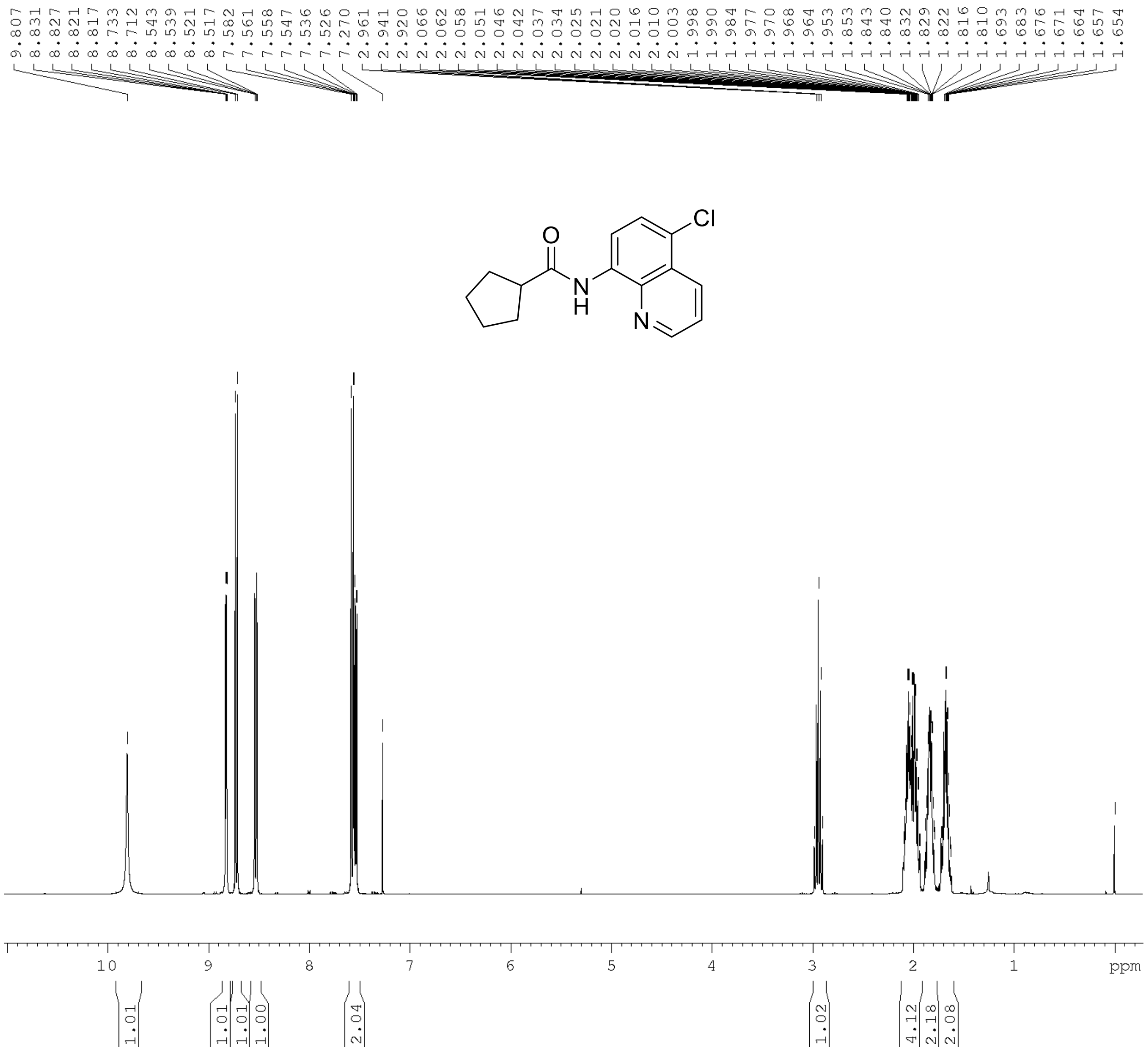

$^{13}C\{^{1}H\}$NMR spectrum of compound **2e** ($CDCl_3$, 100 MHz)**:**

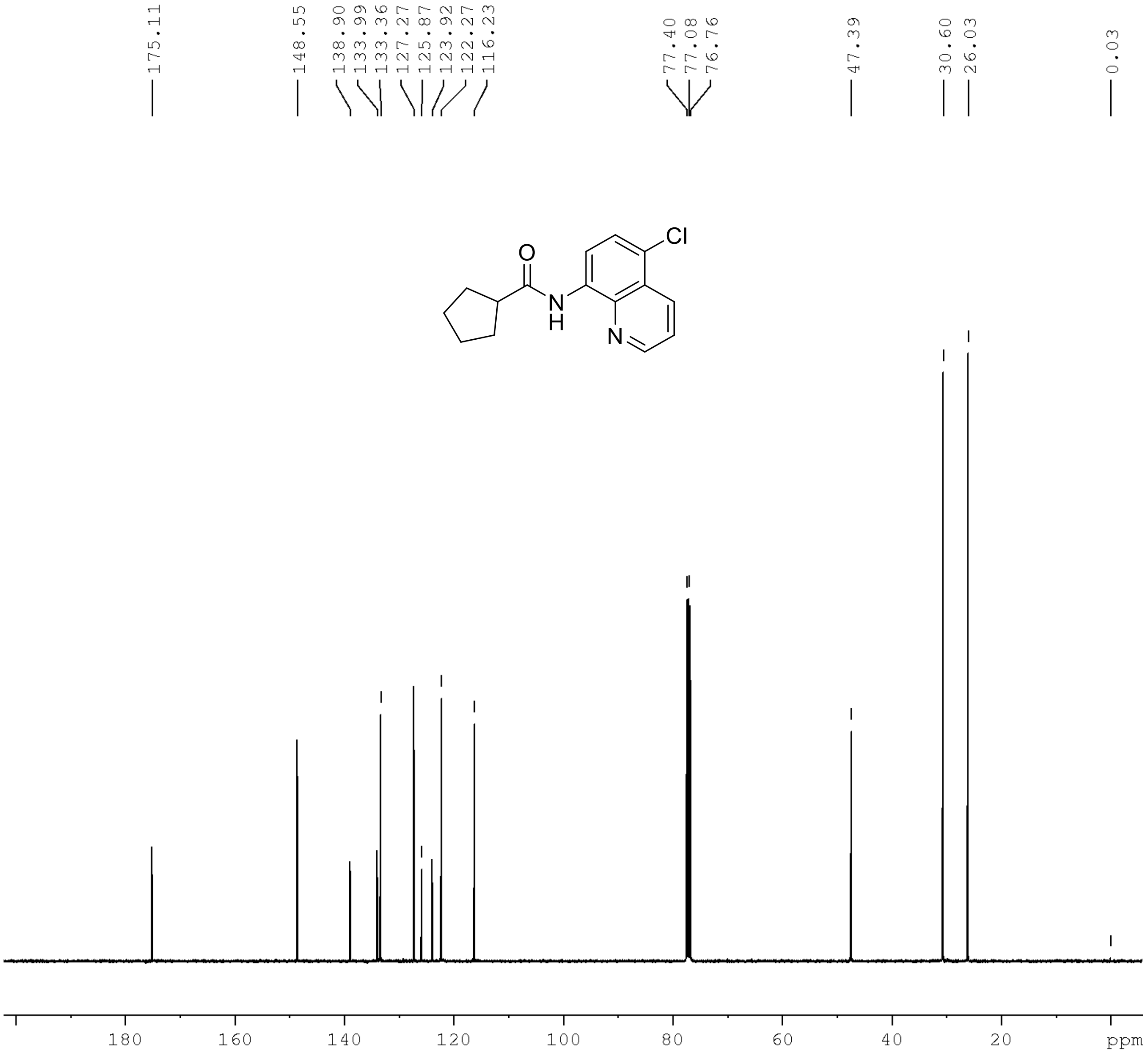

DEPT135 spectrum of compound **2e** ($CDCl_3$, 100 MHz)**:**

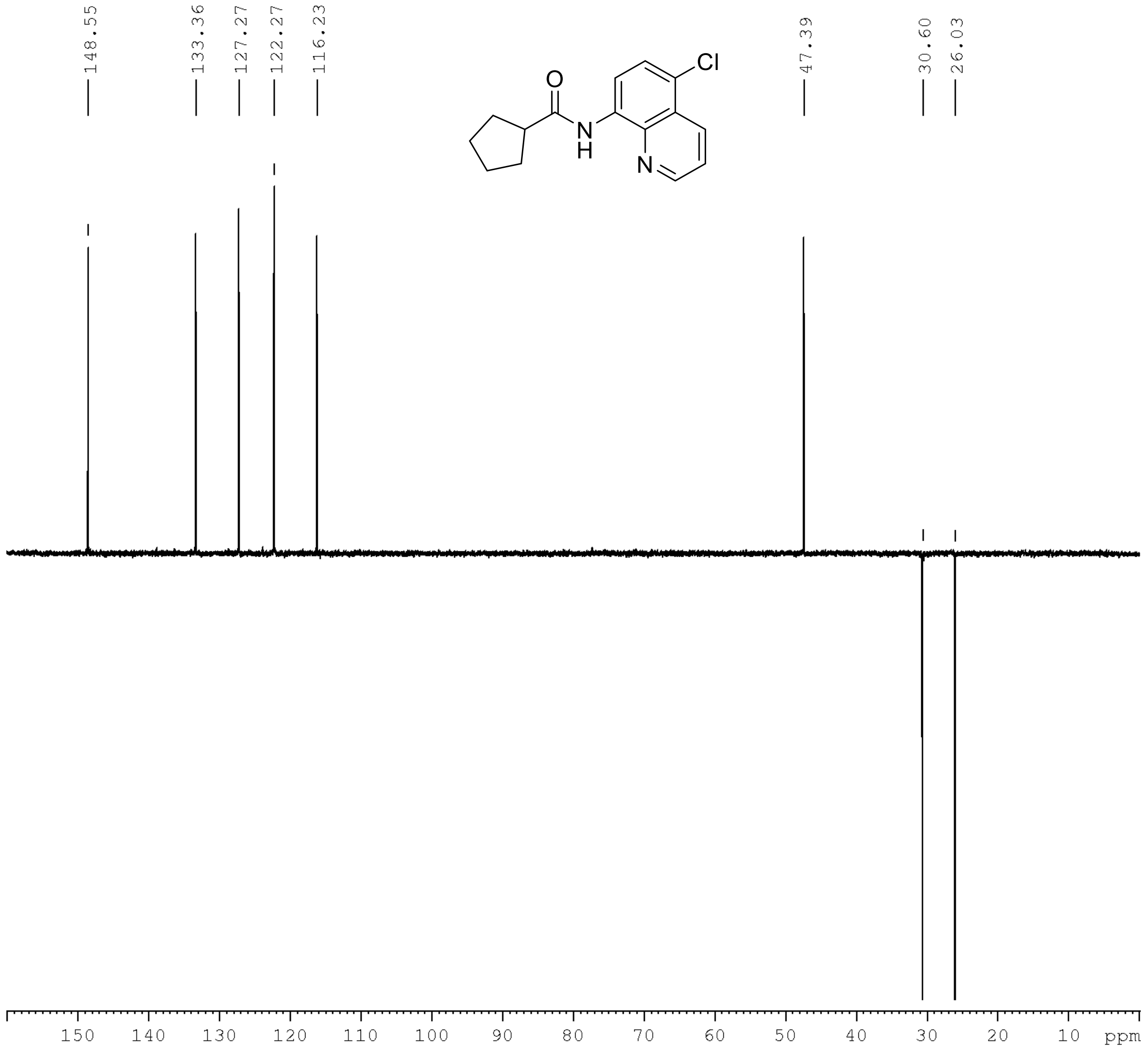

HRMS spectrum of compound **2e:**

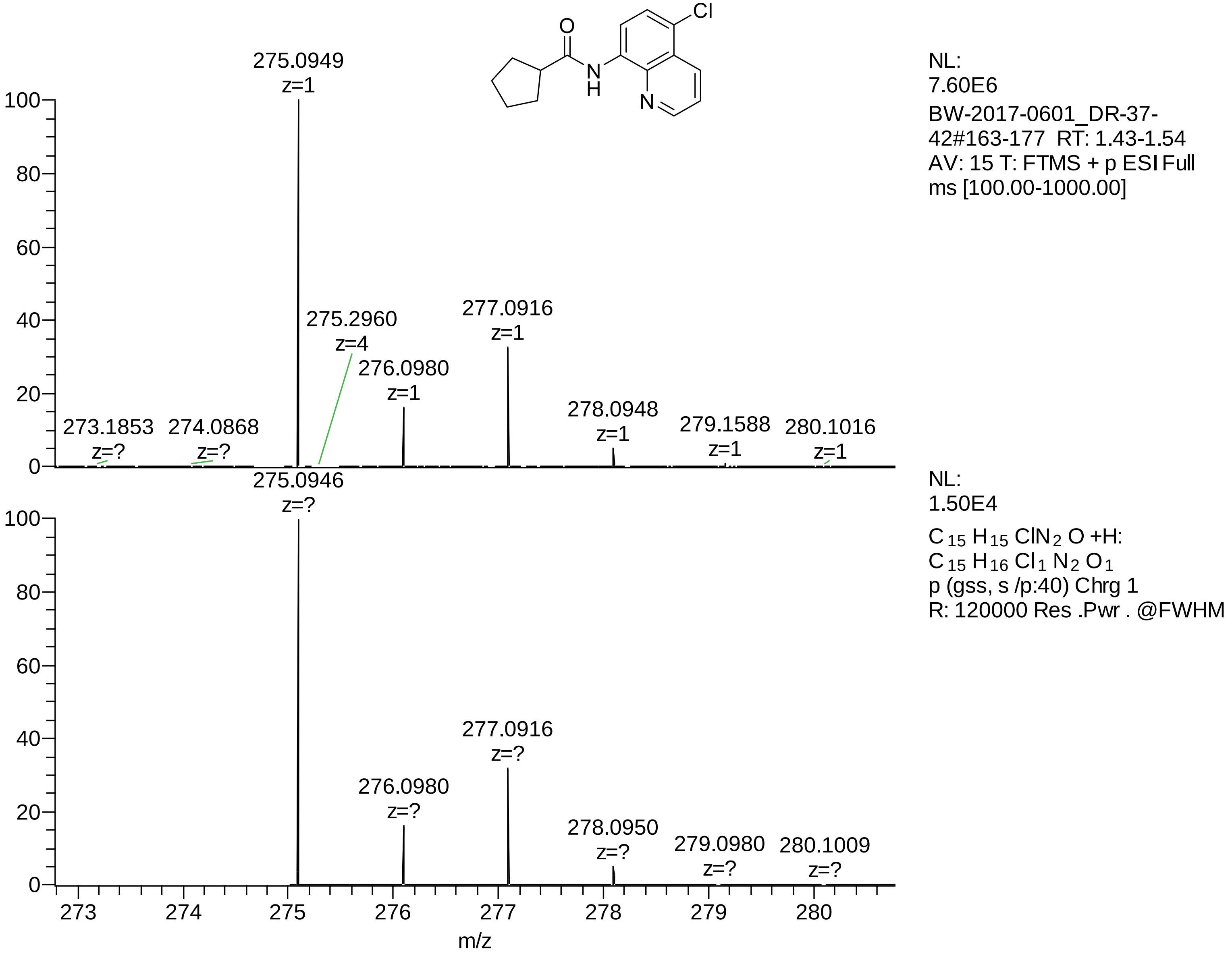

^{1}H NMR spectrum of compound **3e** ($CDCl_3$, 400 MHz):

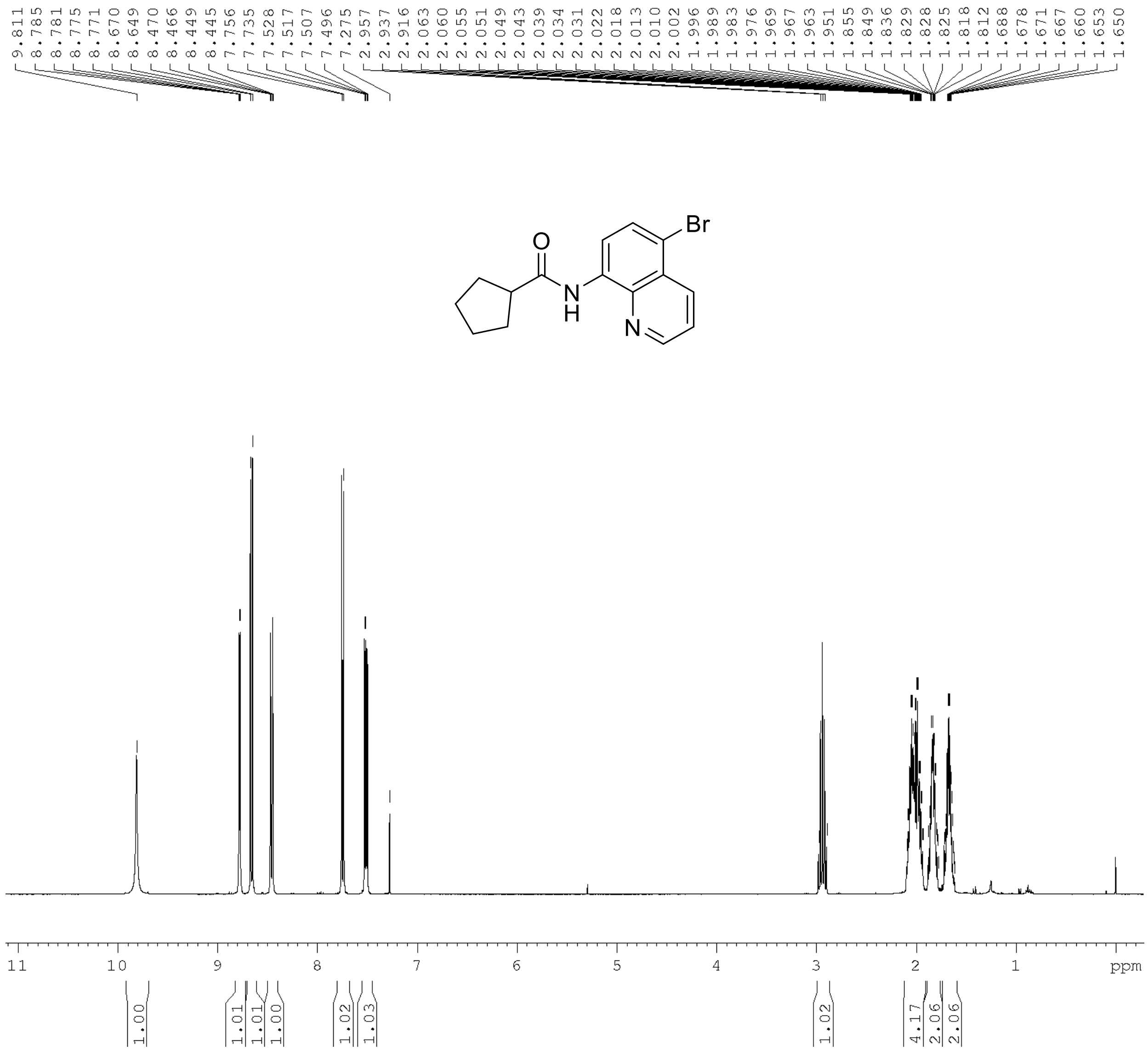

$^{13}C\{^1H\}$NMR spectrum of compound **3e** ($CDCl_3$, 100 MHz)**:**

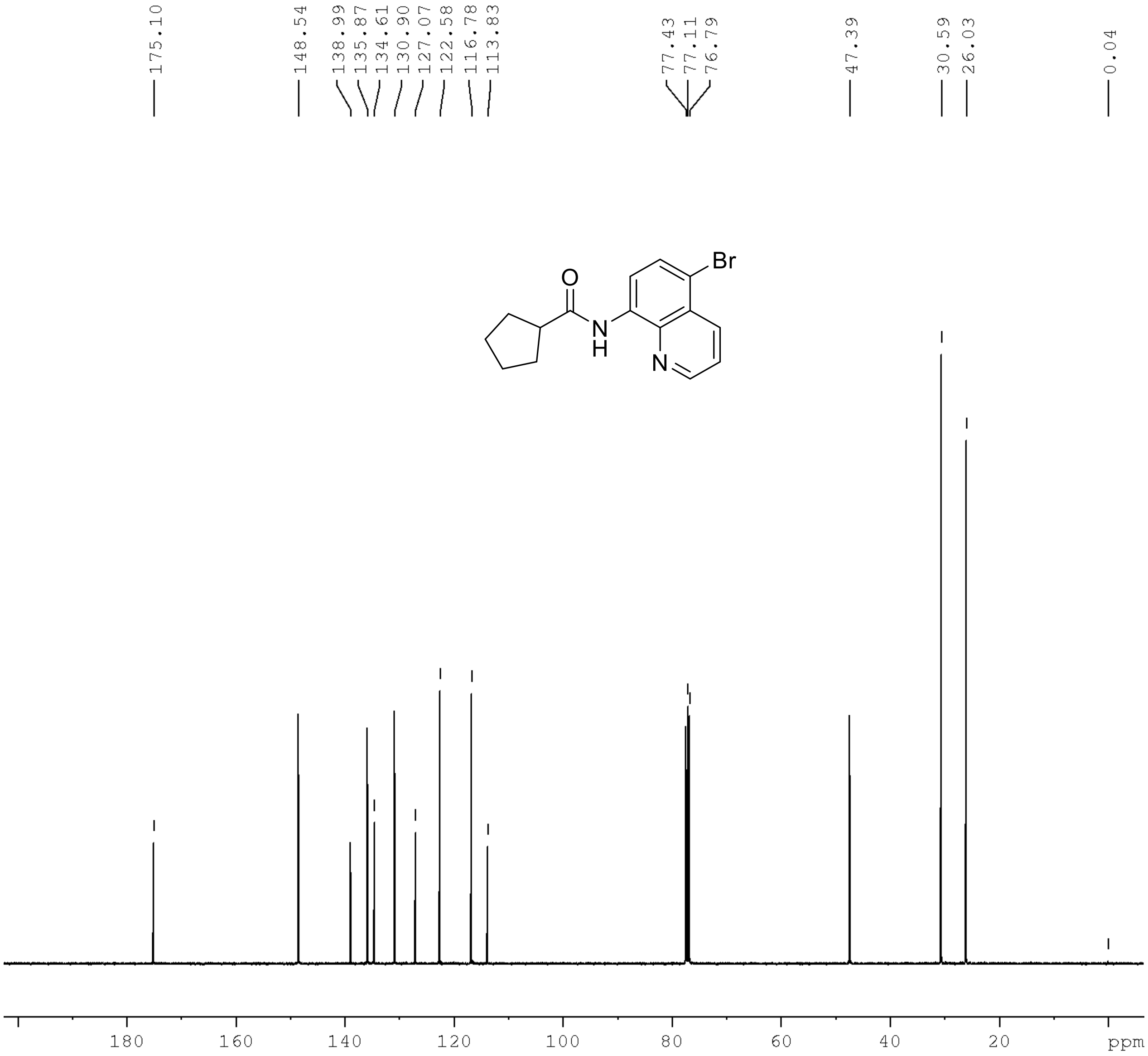

DEPT135 spectrum of compound **3e** ($CDCl_3$, 100 MHz)**:**

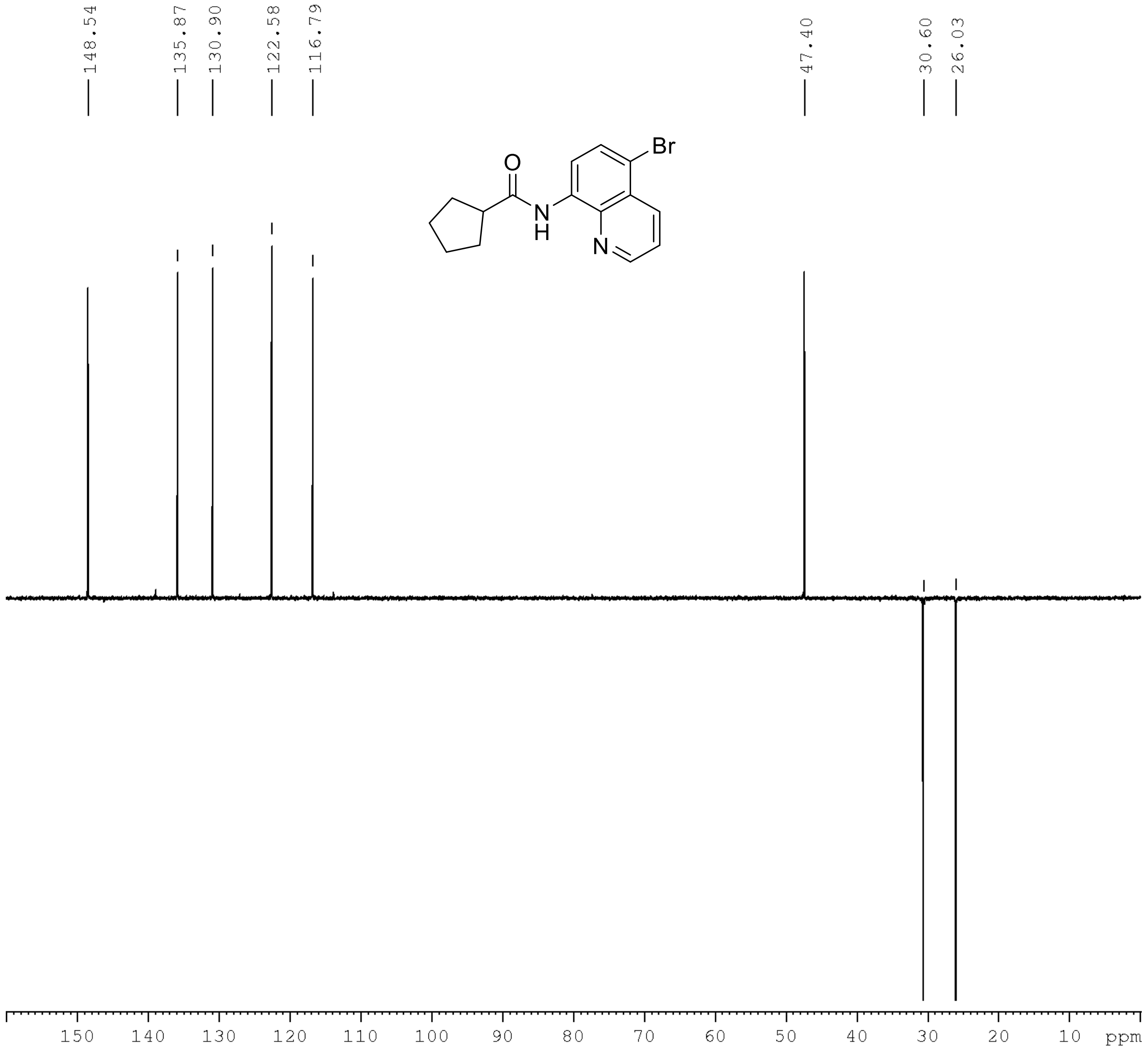

^{1}H NMR spectrum of compound **2f** ($CDCl_3$, 400 MHz)**:**

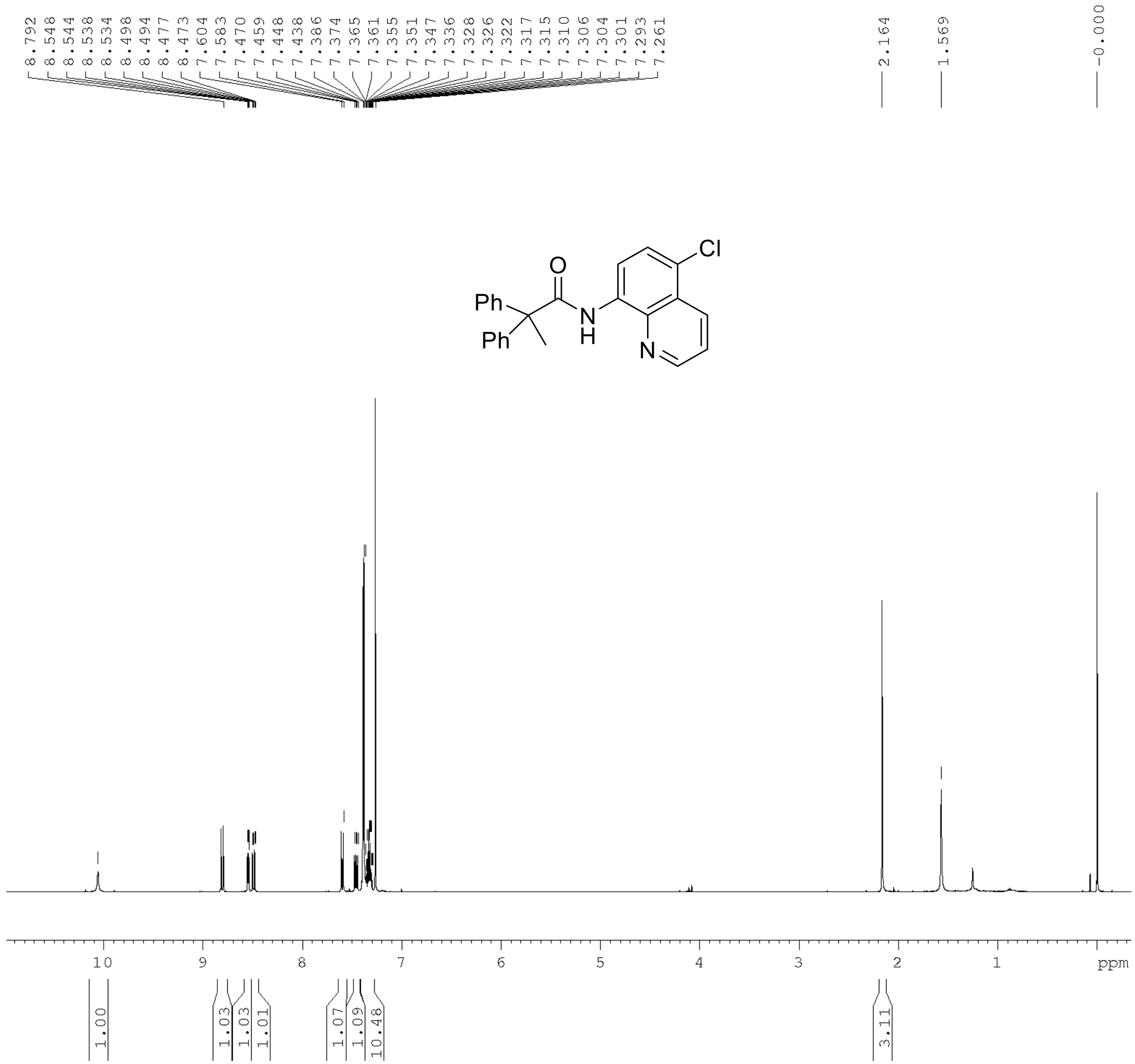

$^{13}C\{^{1}H\}$NMR spectrum of compound **2f** ($CDCl_3$, 100 MHz)**:**

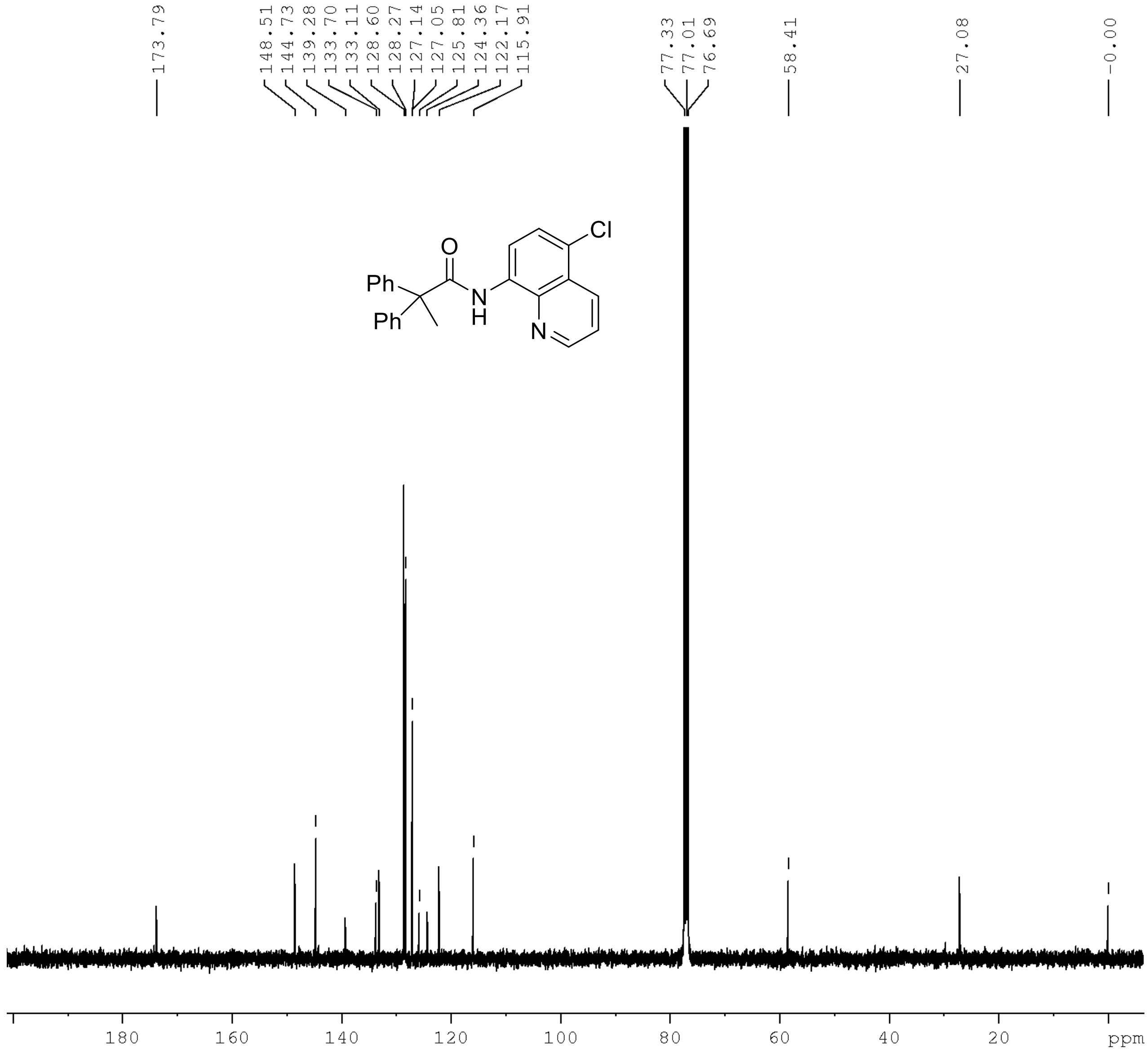

^{1}H NMR spectrum of compound **3f** ($CDCl_3$, 400 MHz):

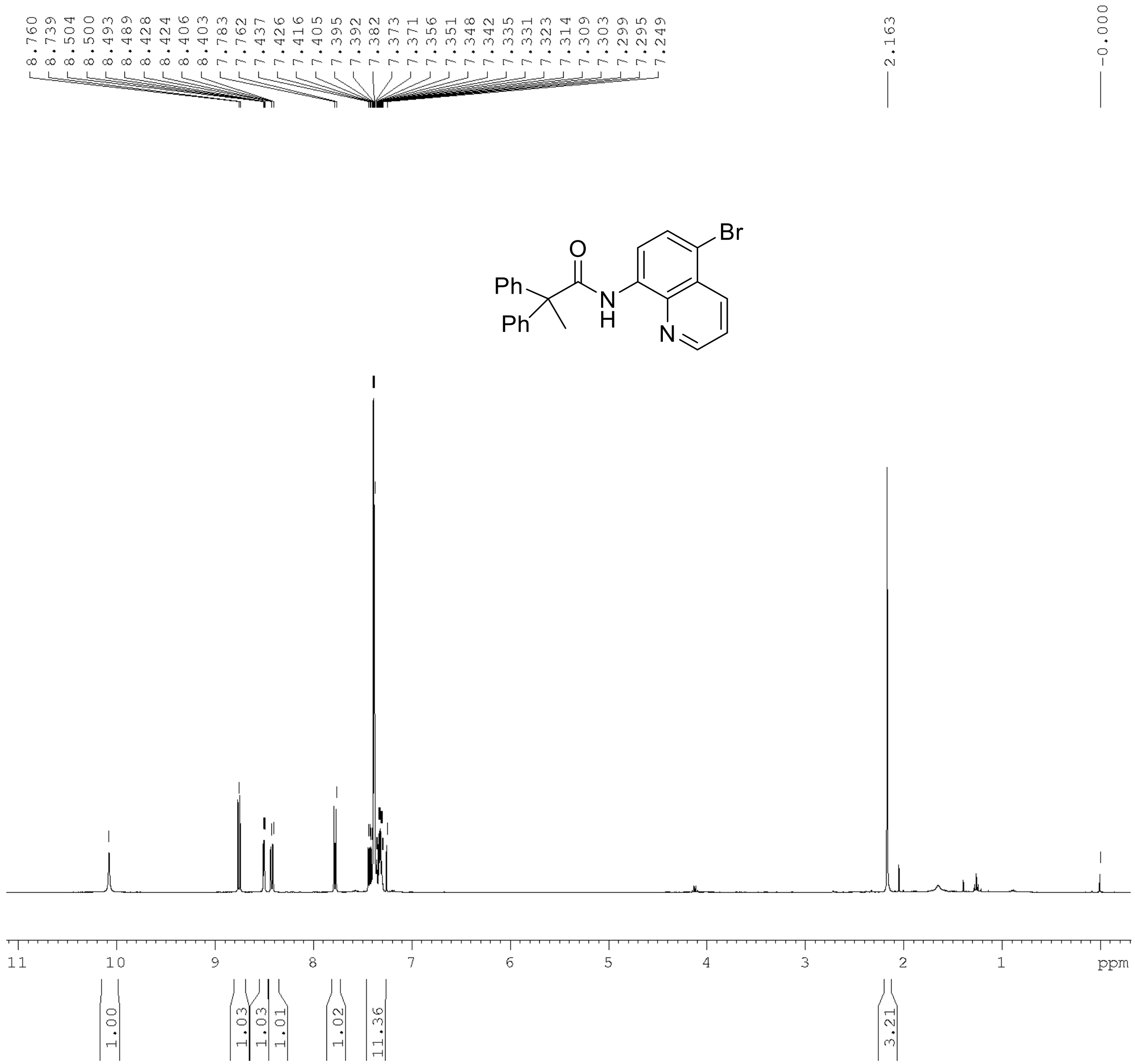

HRMS spectrum of compound **3f:**

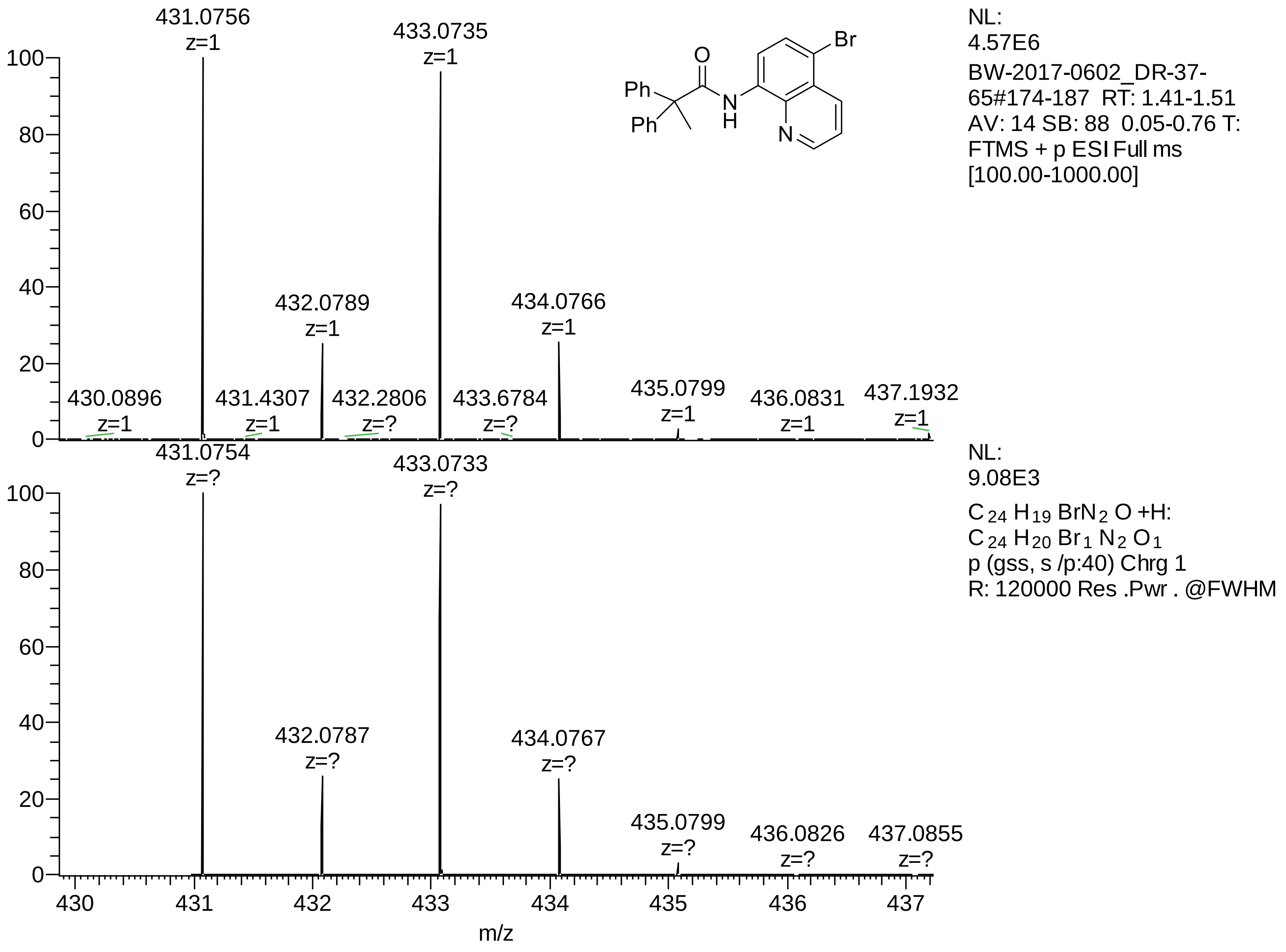

$^{13}C\{^1H\}$NMR spectrum of compound **3f** ($CDCl_3$, 100 MHz)**:**

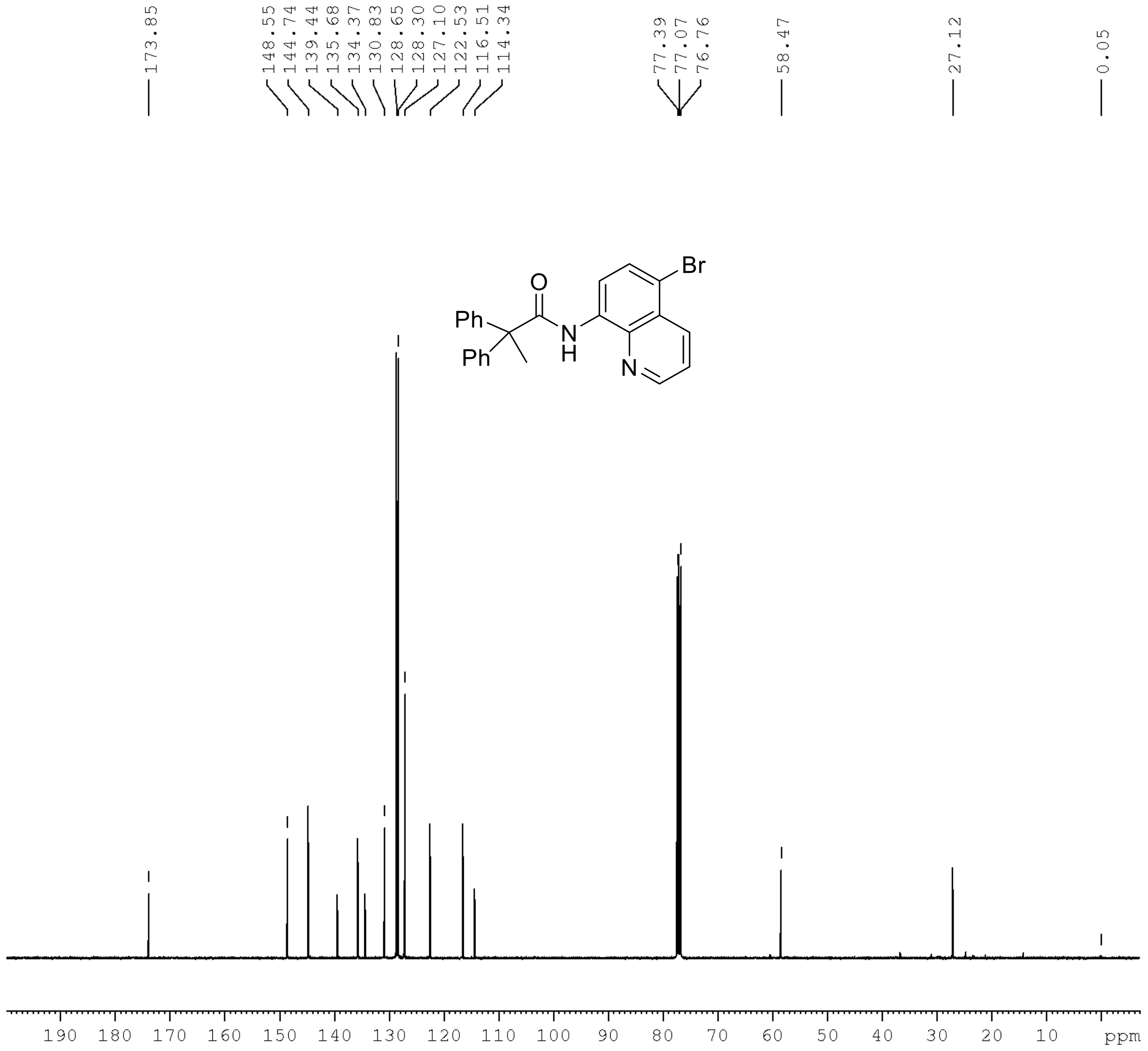

^{1}H NMR spectrum of compound **2g** ($CDCl_3$, 400 MHz)**:**

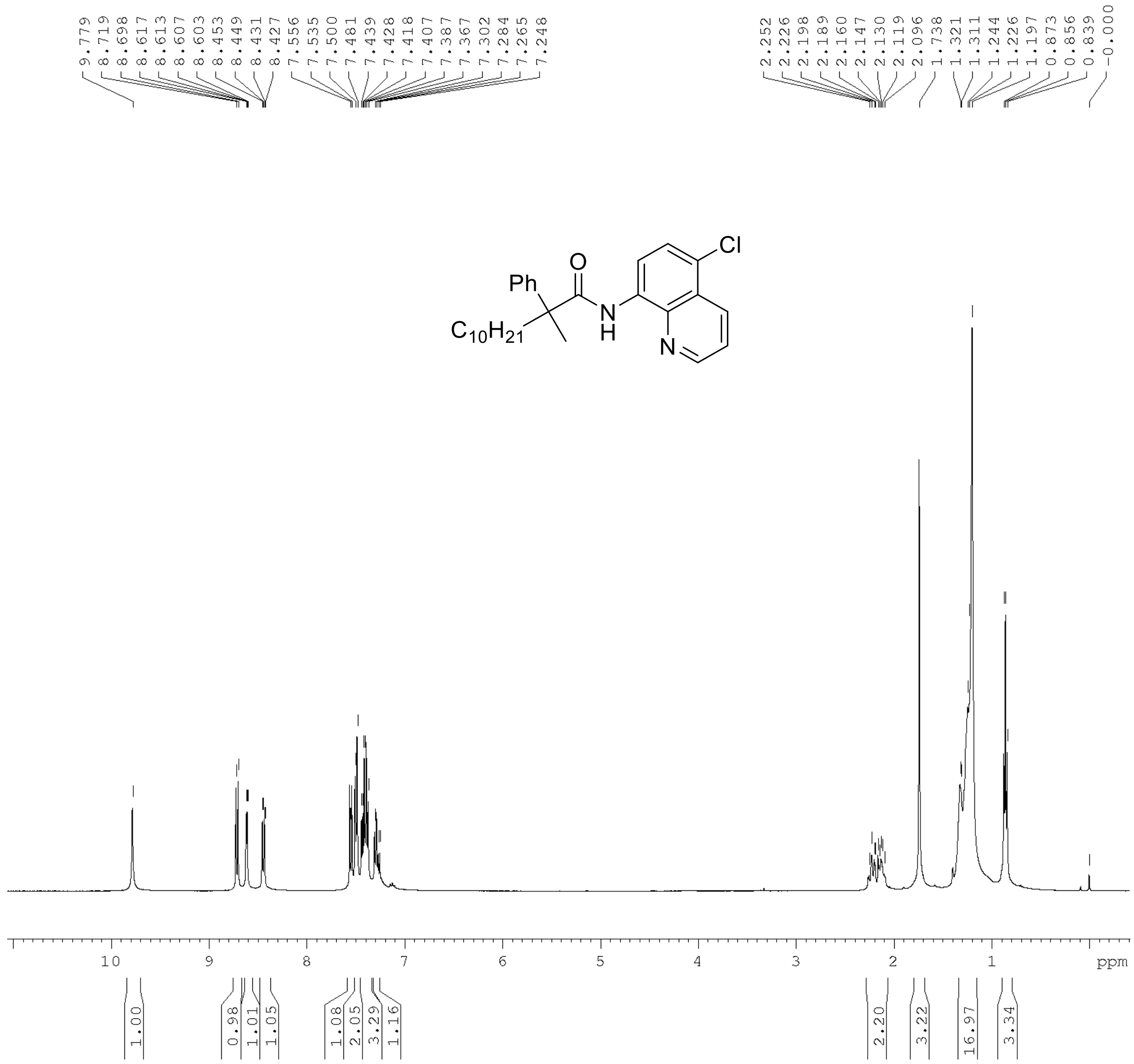

$^{13}C\{^{1}H\}$NMR spectrum of compound **2g** ($CDCl_3$, 100 MHz)**:**

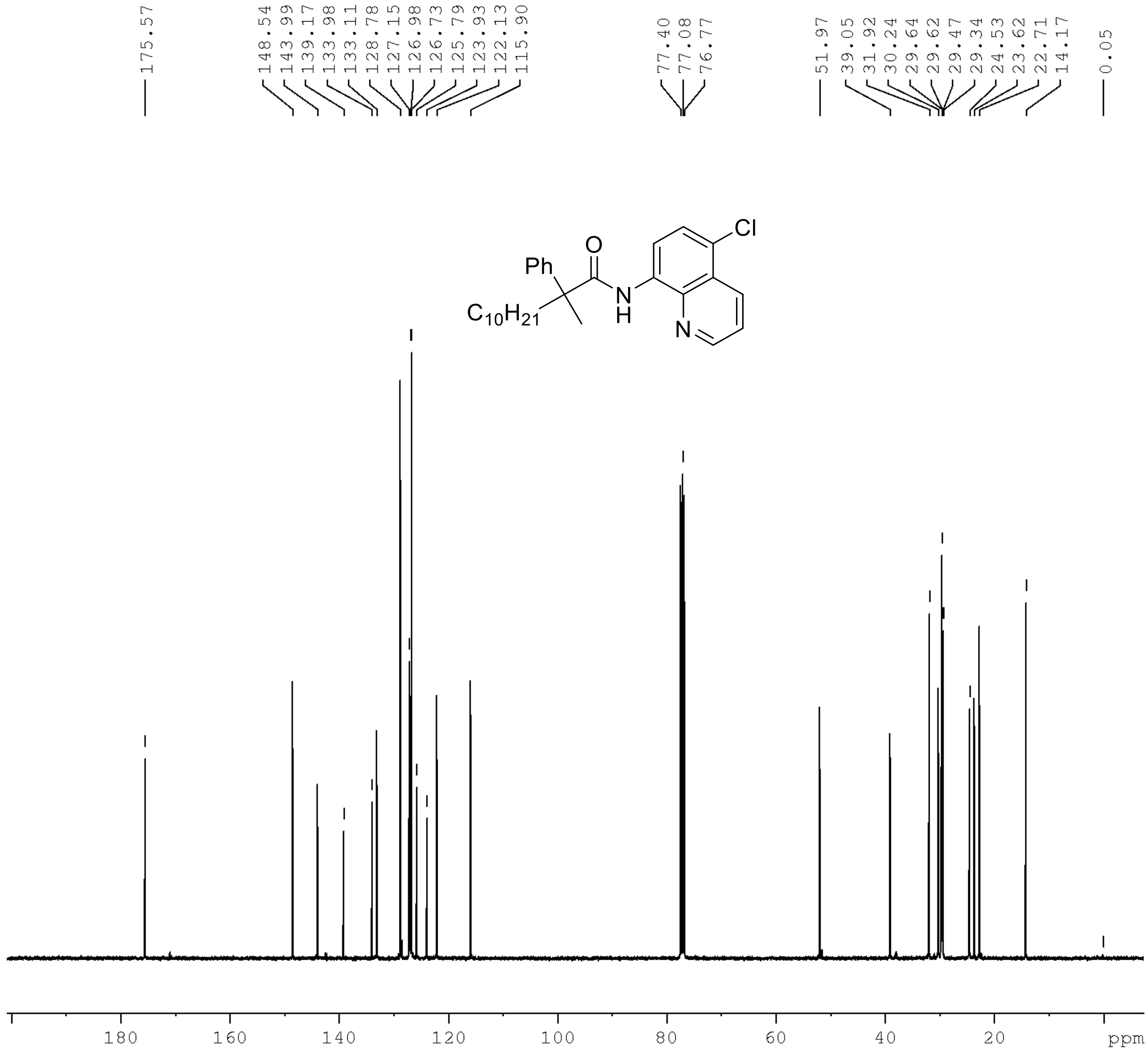

DEPT135 spectrum of compound **2g** ($CDCl_3$, 100 MHz)**:**

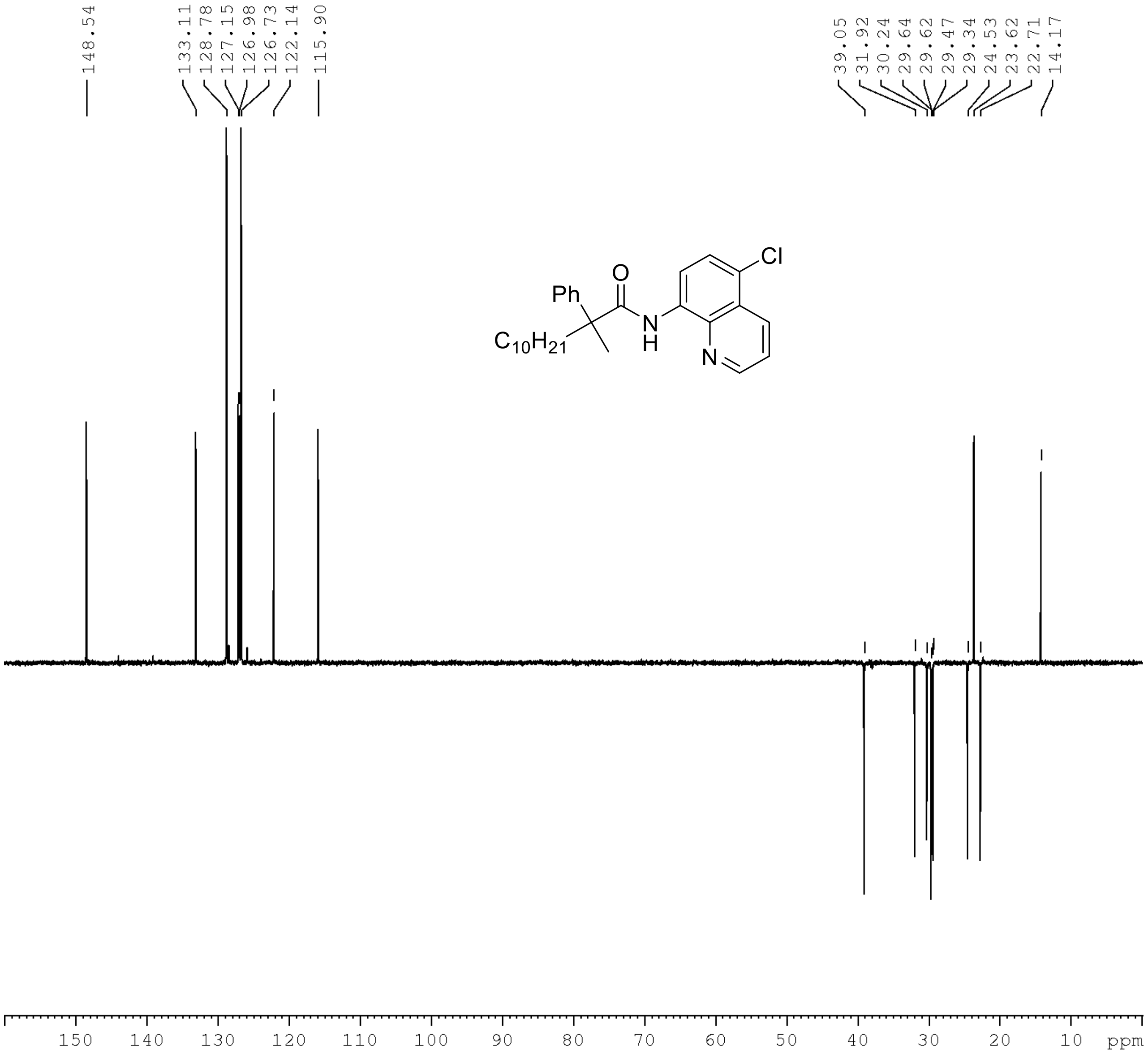

HRMS spectrum of compound **2g:**

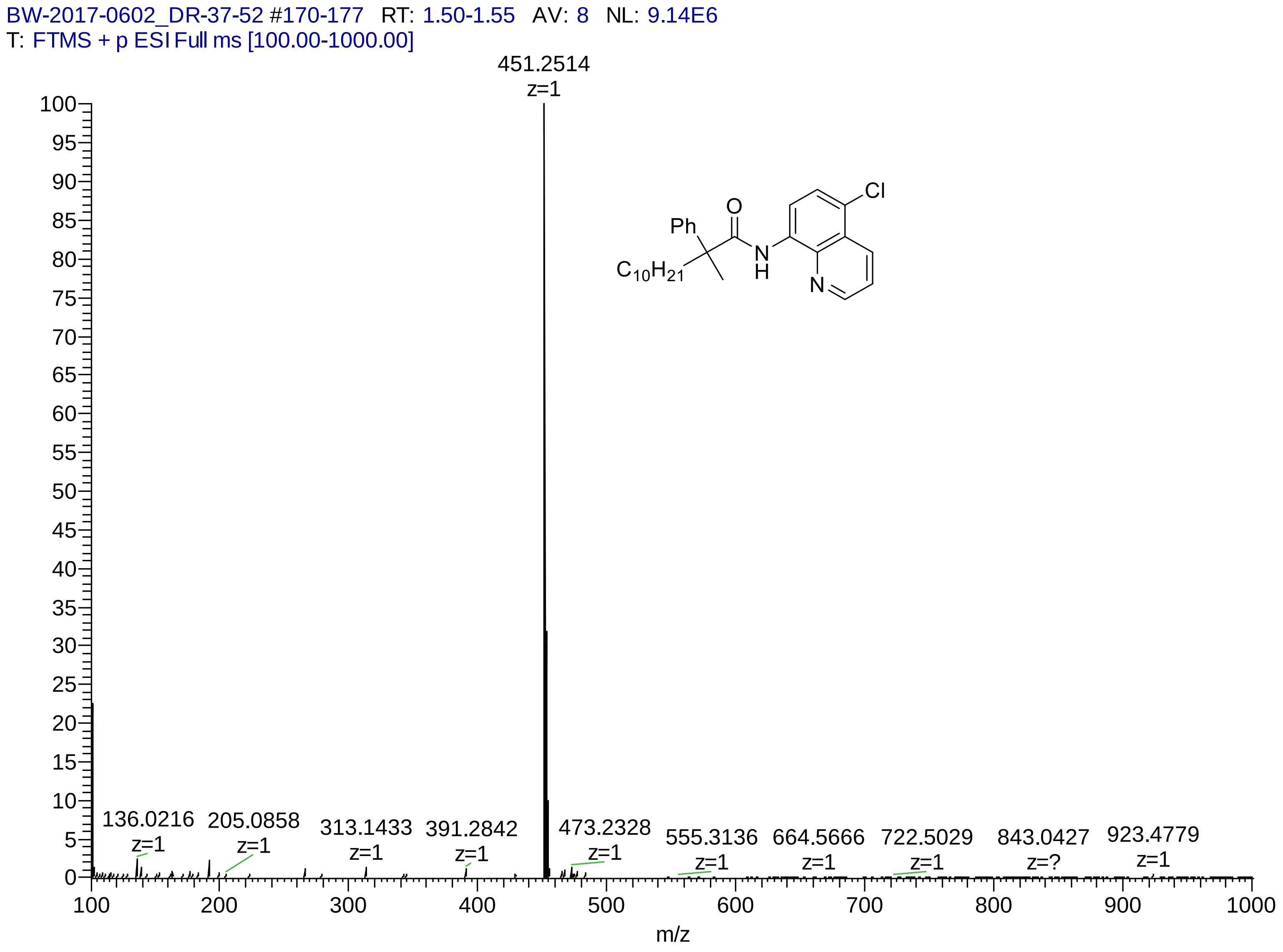

^{1}H NMR spectrum of compound **3g** ($CDCl_3$, 400 MHz):

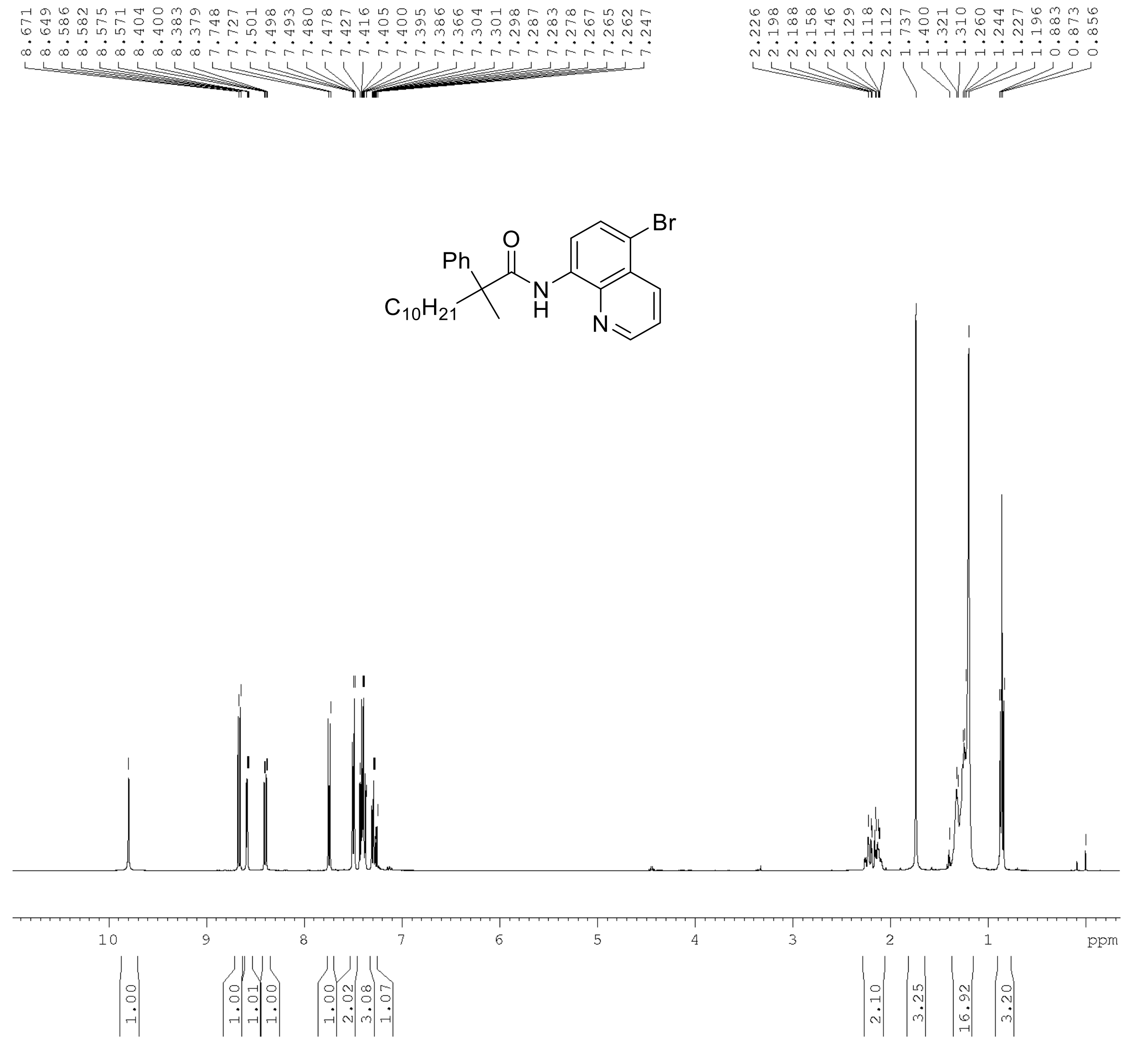

$^{13}C\{^{1}H\}$NMR spectrum of compound **3g** ($CDCl_3$, 100 MHz)**:**

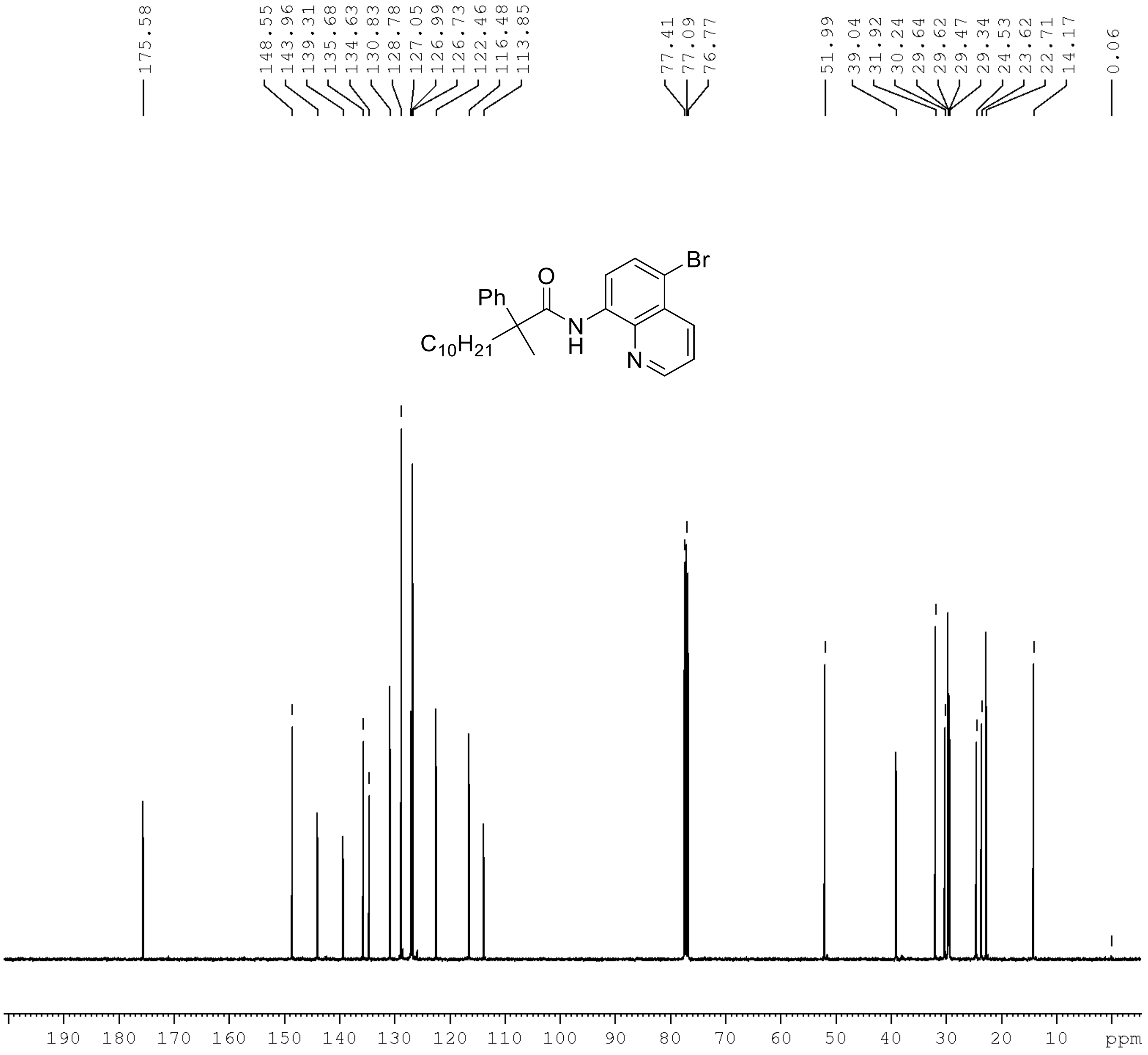

DEPT135 spectrum of compound **3g** ($CDCl_3$, 100 MHz)**:**

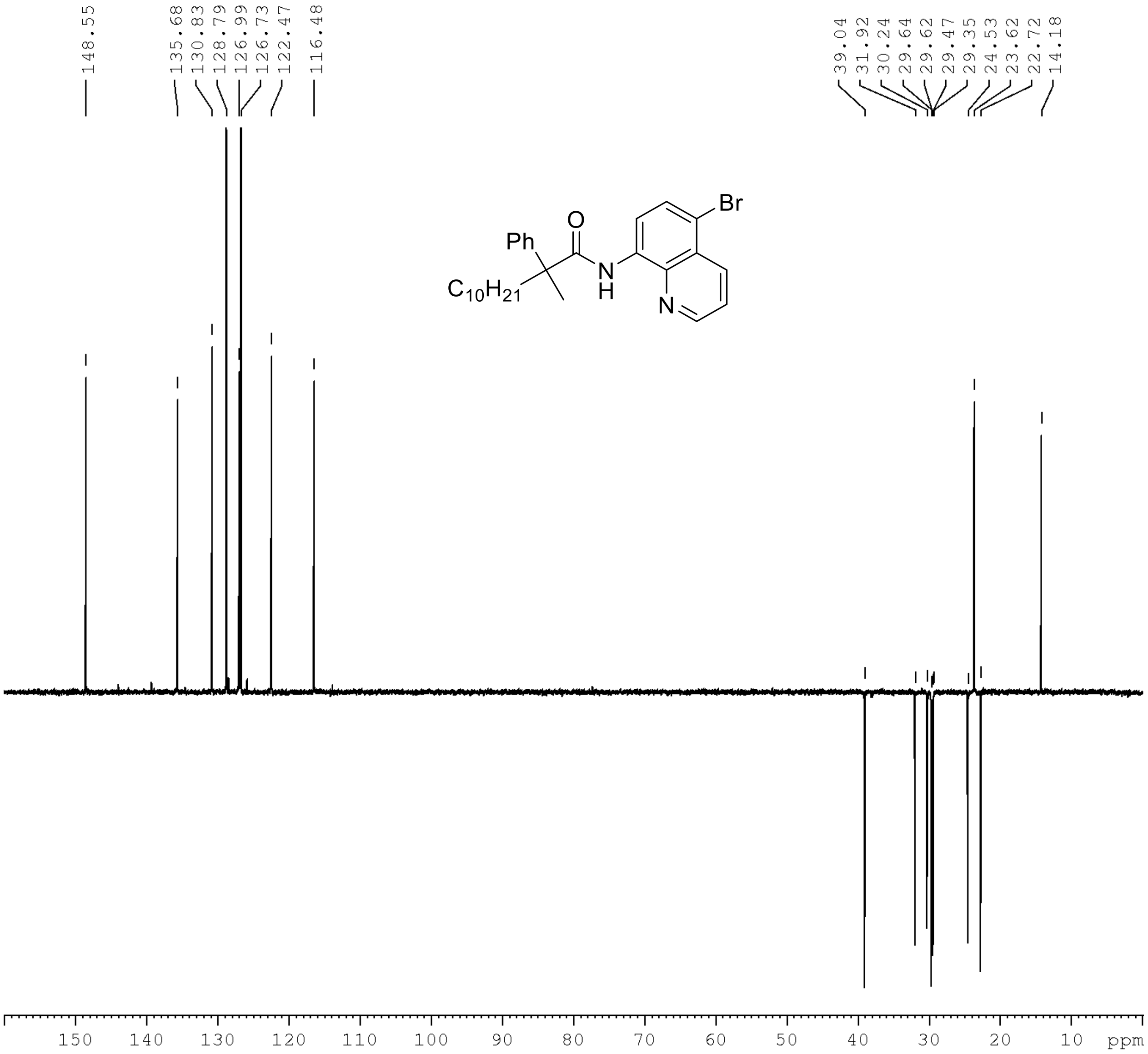

HRMS spectrum of compound **3g:**

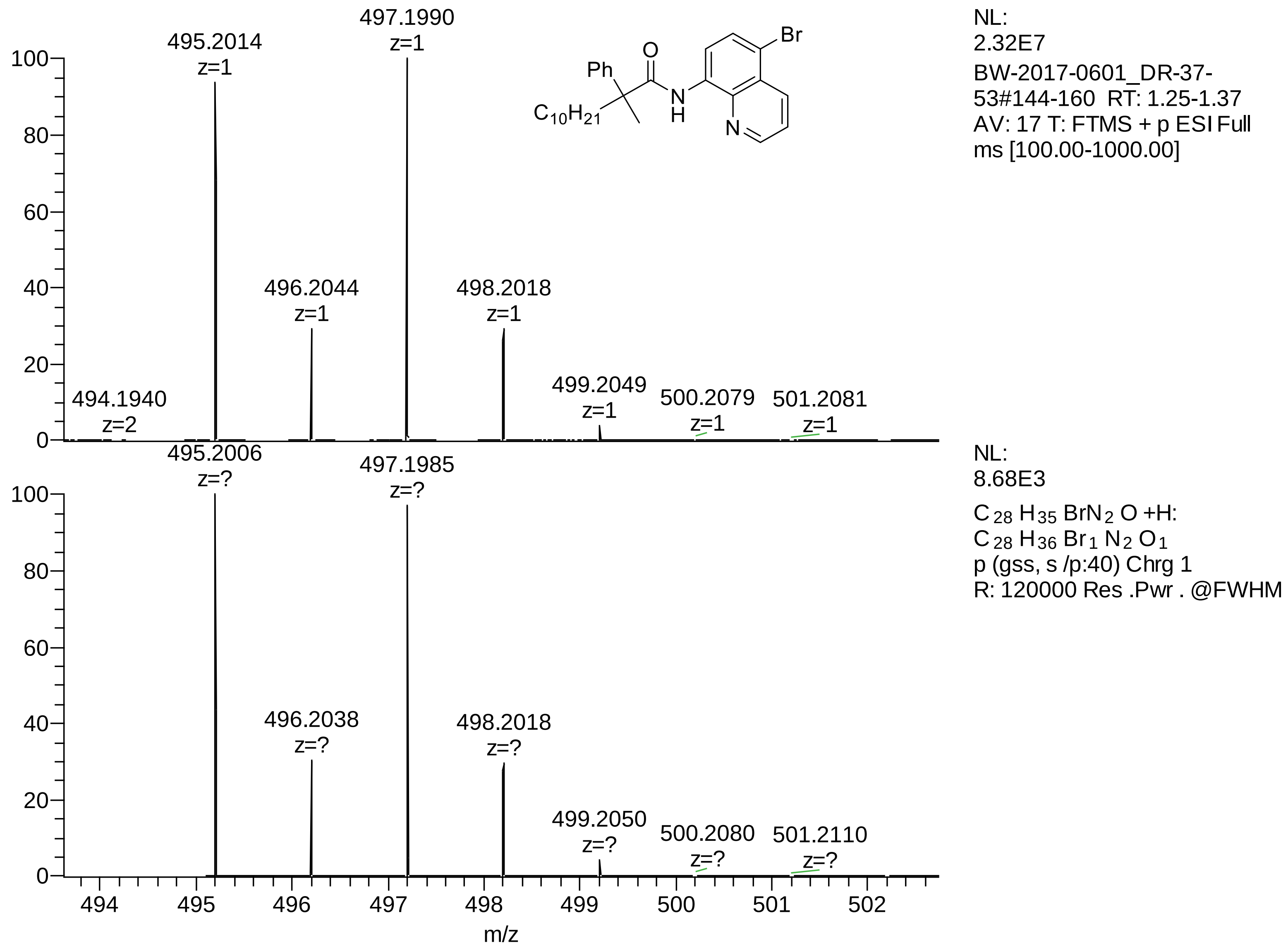

^{1}H NMR spectrum of compound **2h** ($CDCl_3$, 400 MHz):

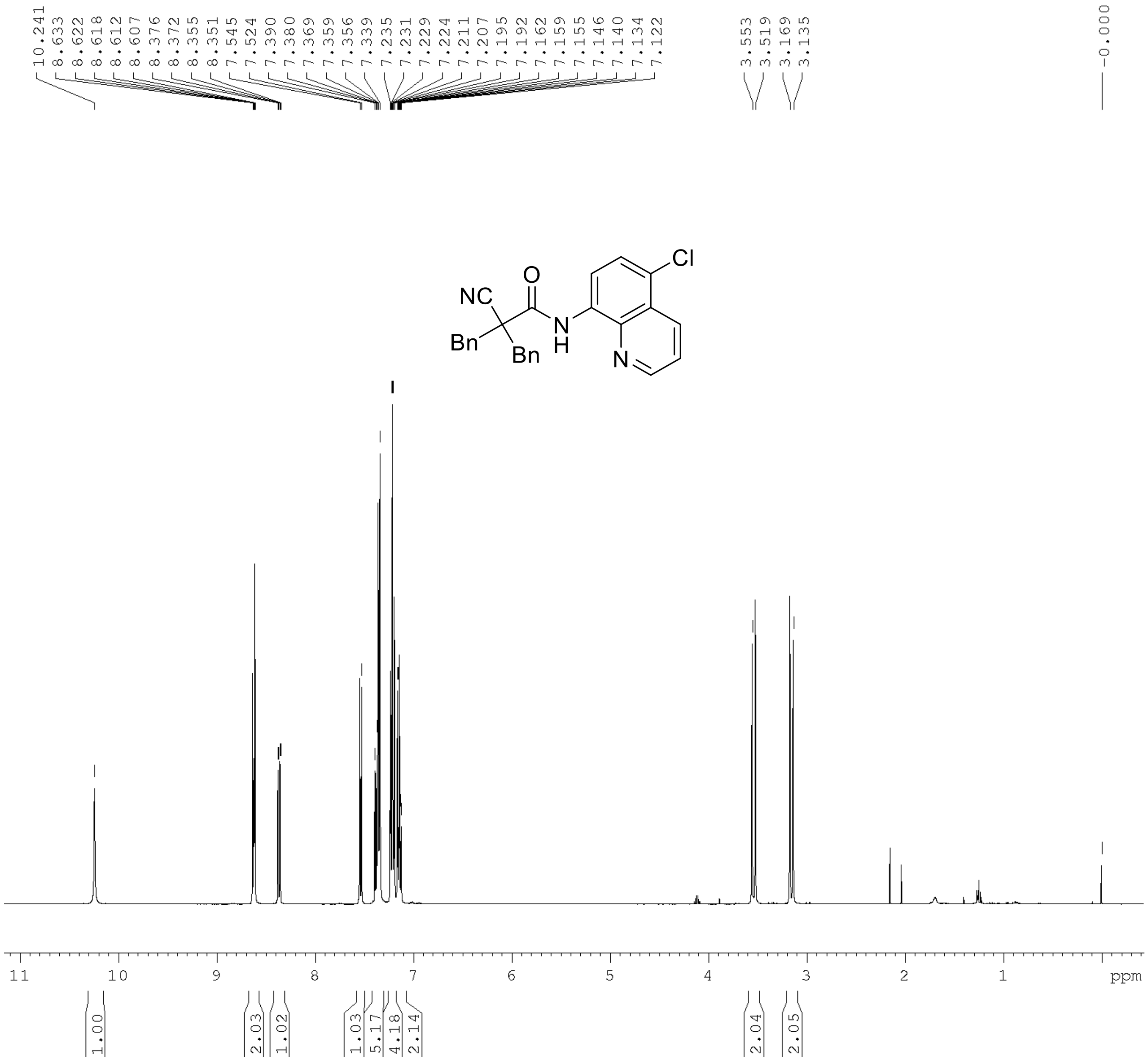

$^{13}C\{^1H\}$NMR spectrum of compound **2h** ($CDCl_3$, 100 MHz)**:**

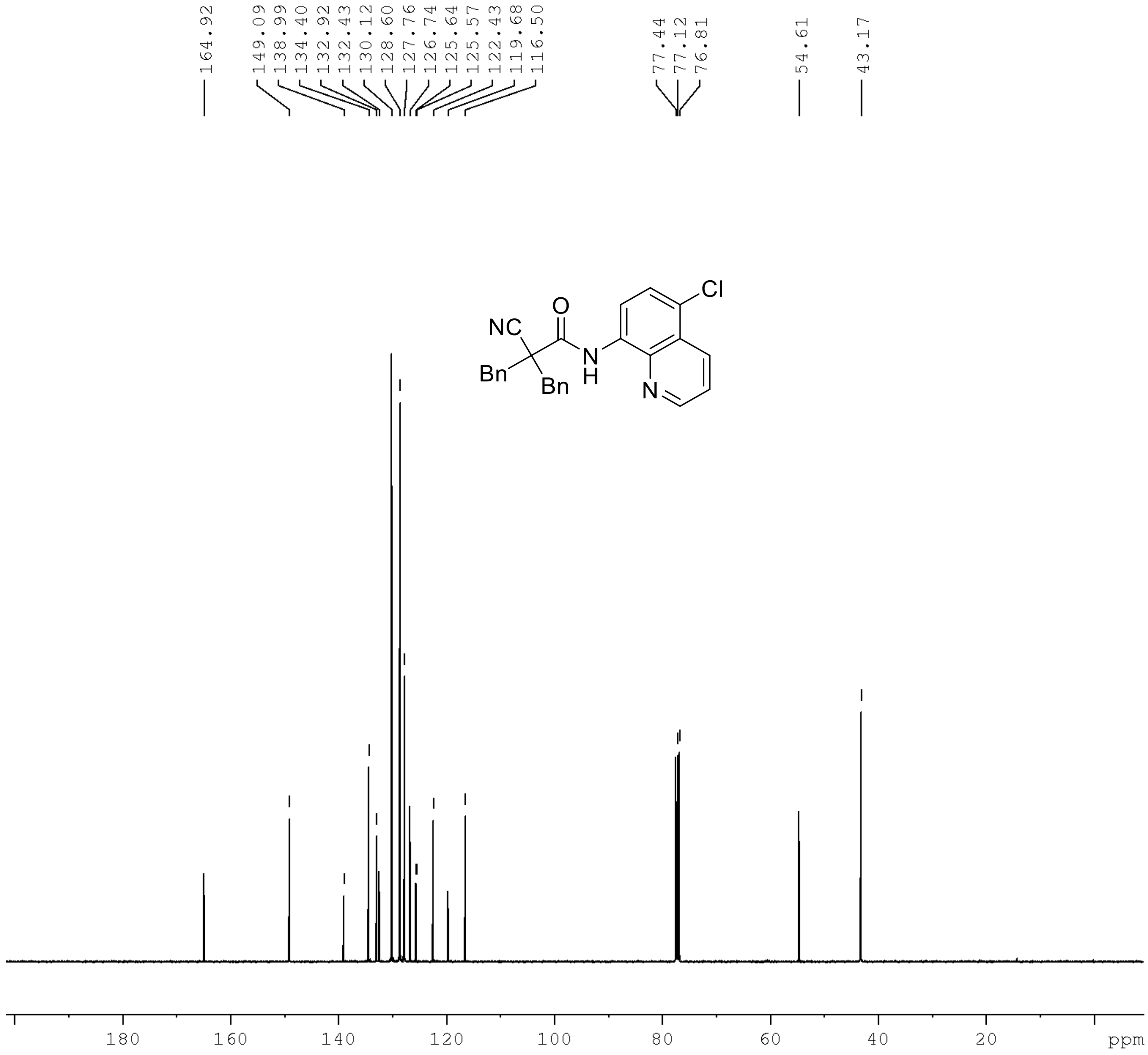

DEPT135 spectrum of compound **2h** ($CDCl_3$, 100 MHz)**:**

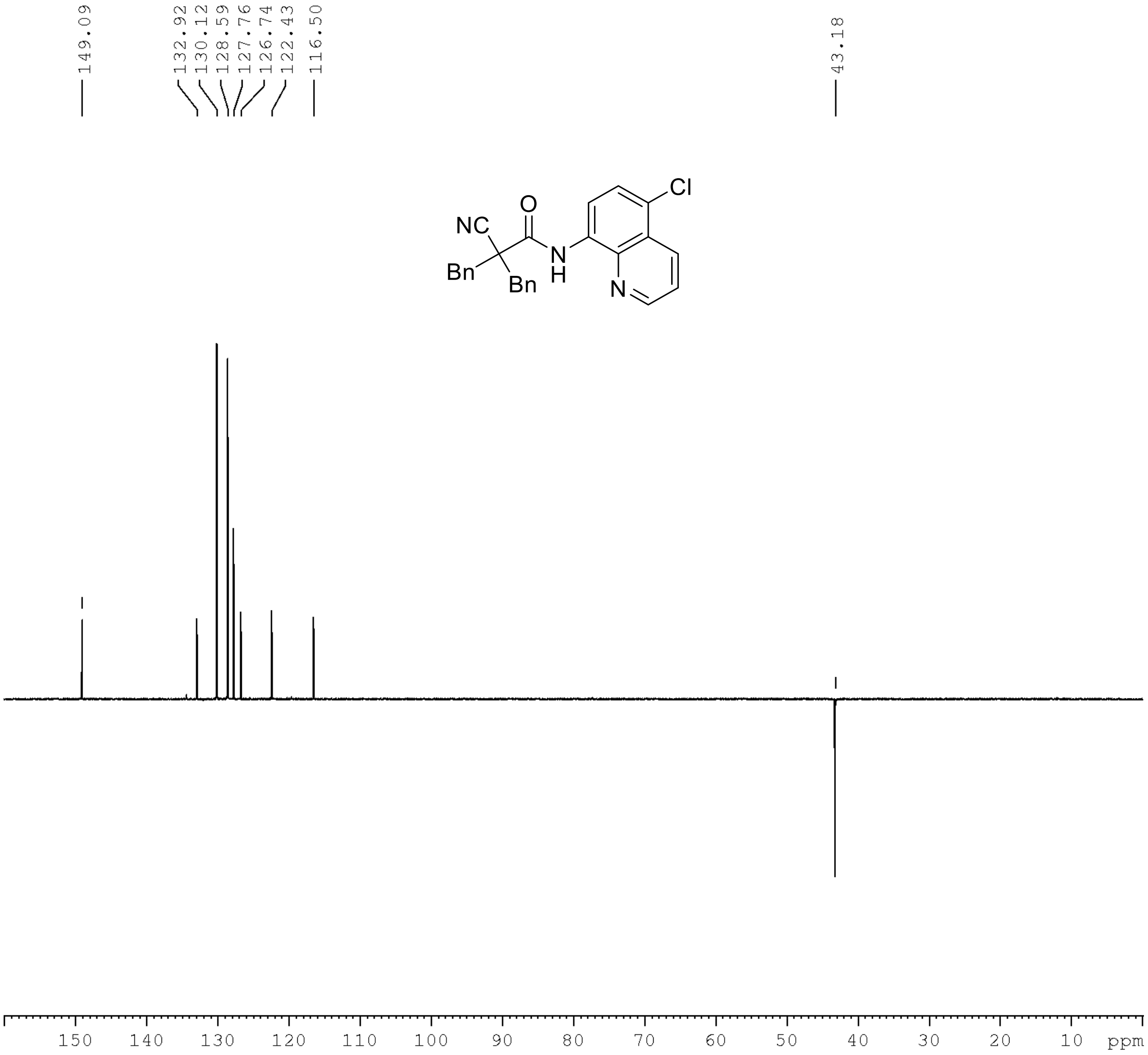

^{1}H NMR spectrum of compound **3h** ($CDCl_3$, 400 MHz)**:**

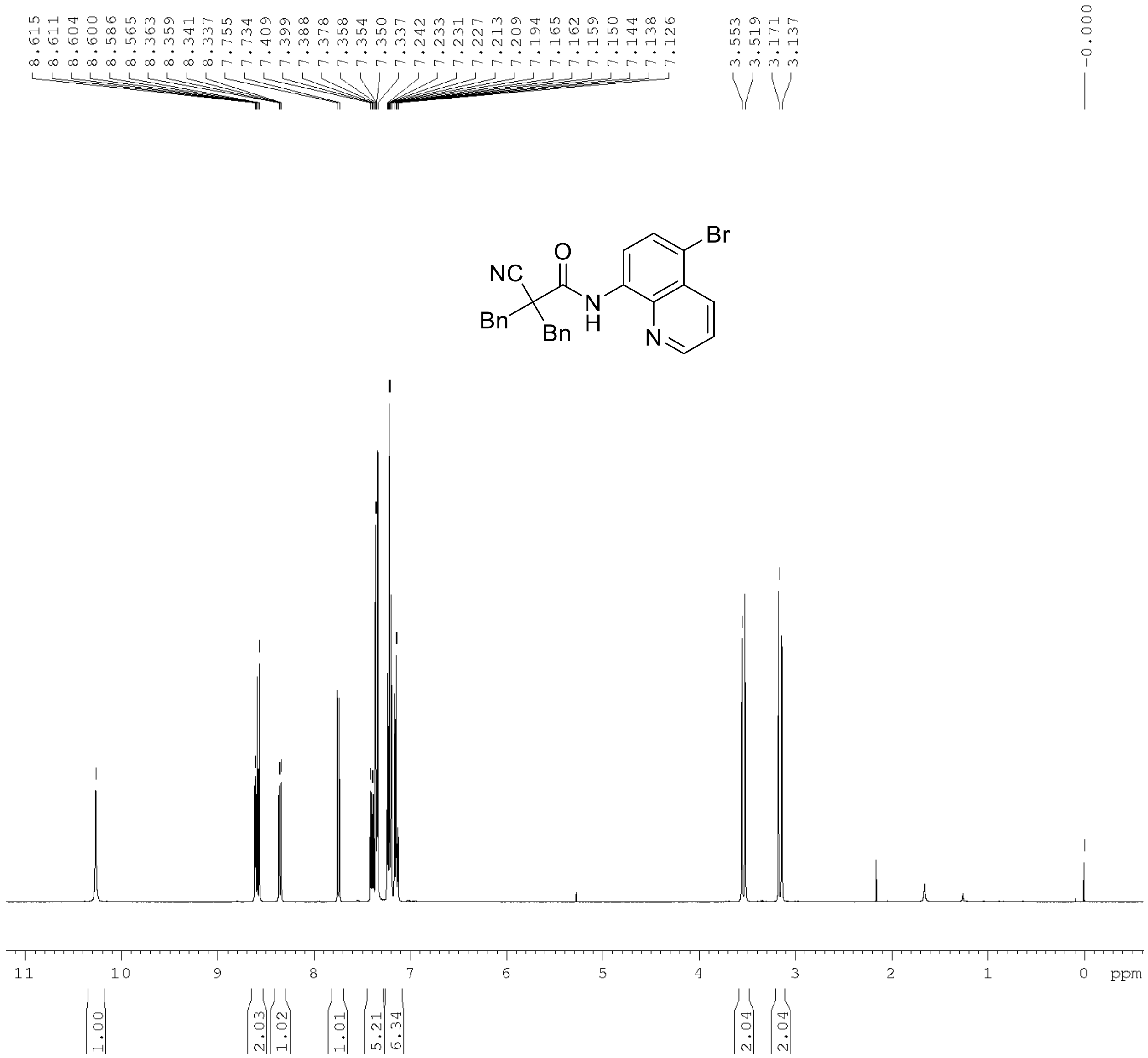

^{13}C{^{1}H}NMR spectrum of compound **3h** ($CDCl_3$, 100 MHz)**:**

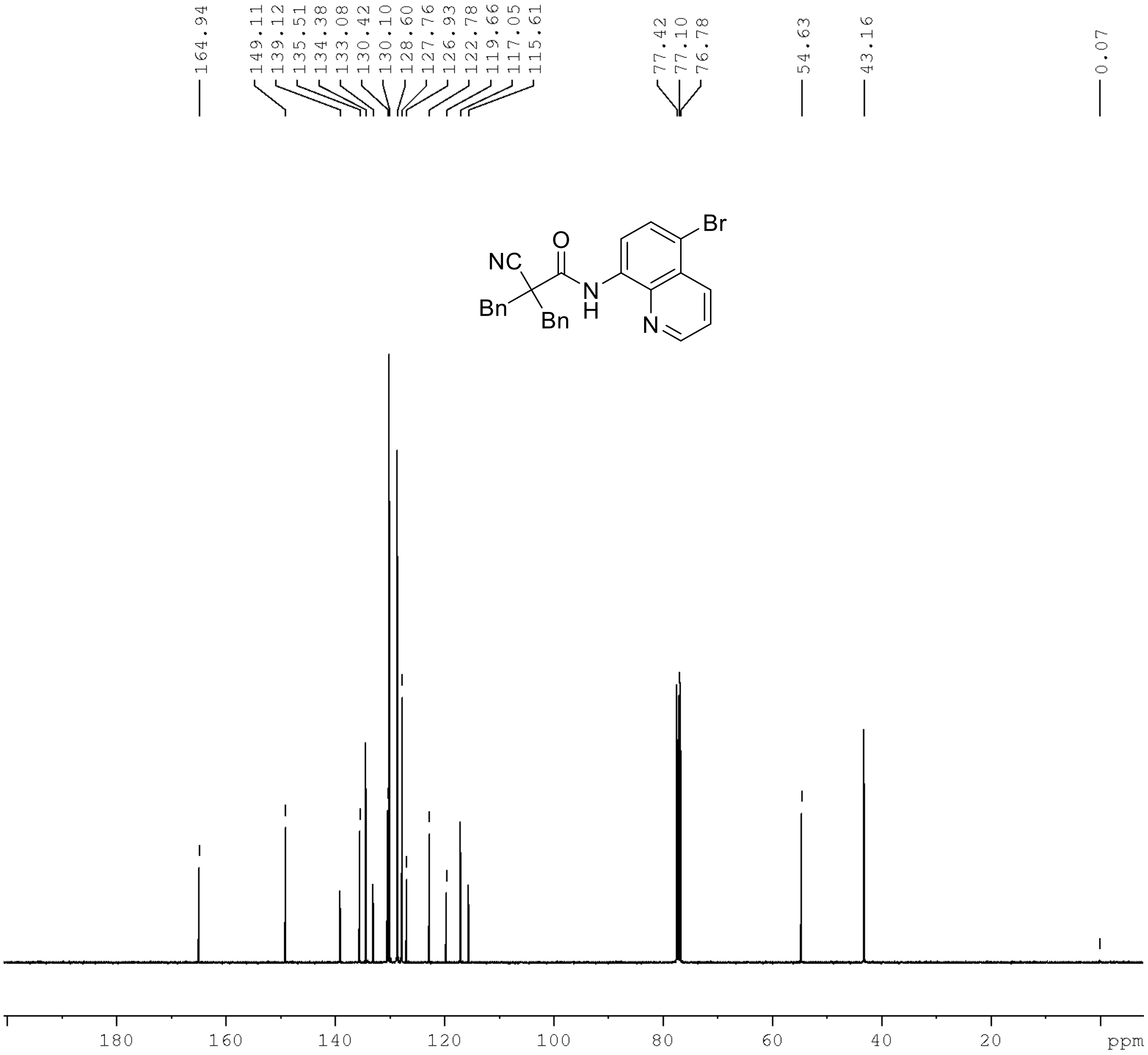

DEPT135 spectrum of compound **3h** ($CDCl_3$, 100 MHz)**:**

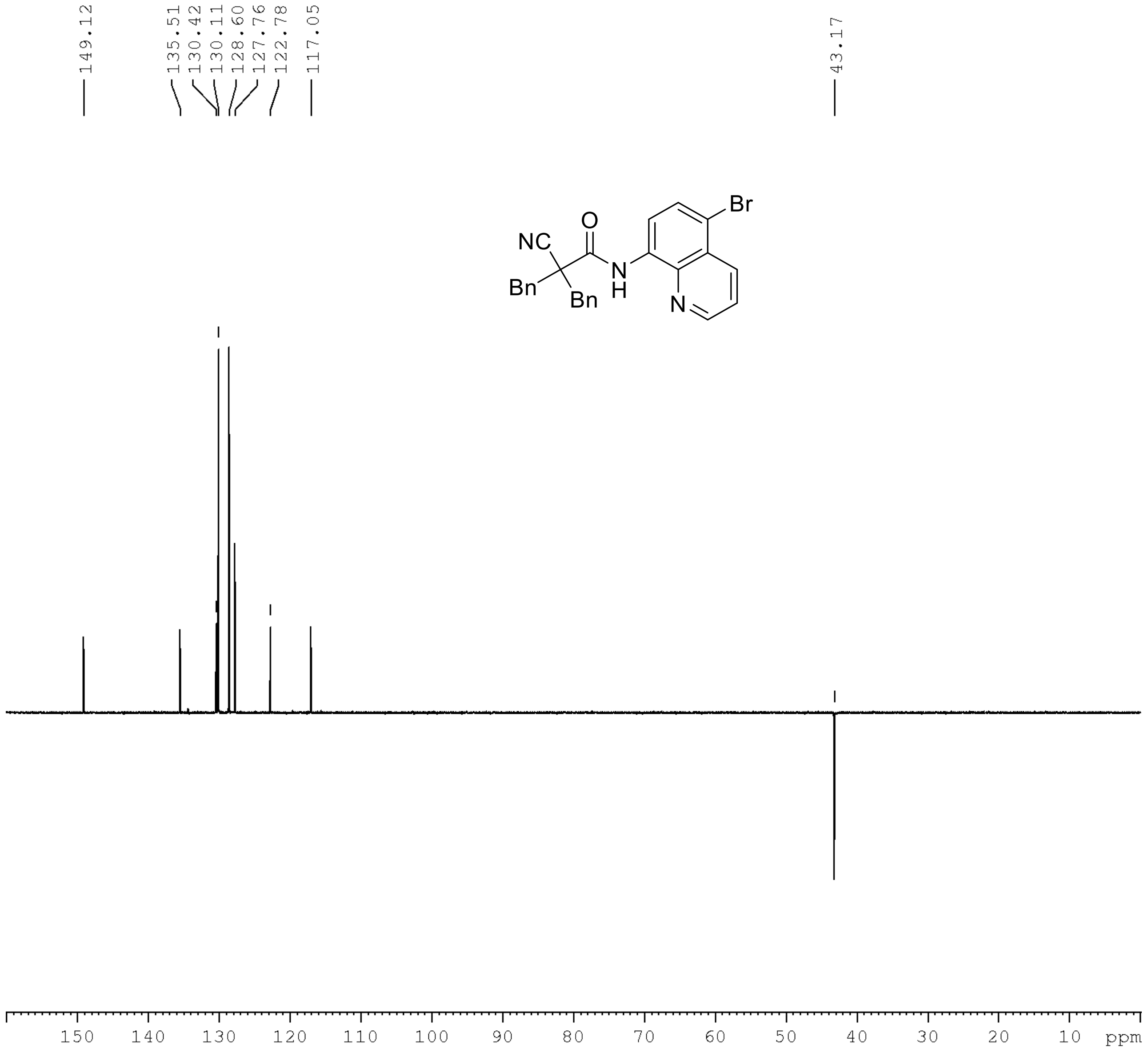

^{1}H NMR spectrum of compound **2i** ($CDCl_3$, 400 MHz)**:**

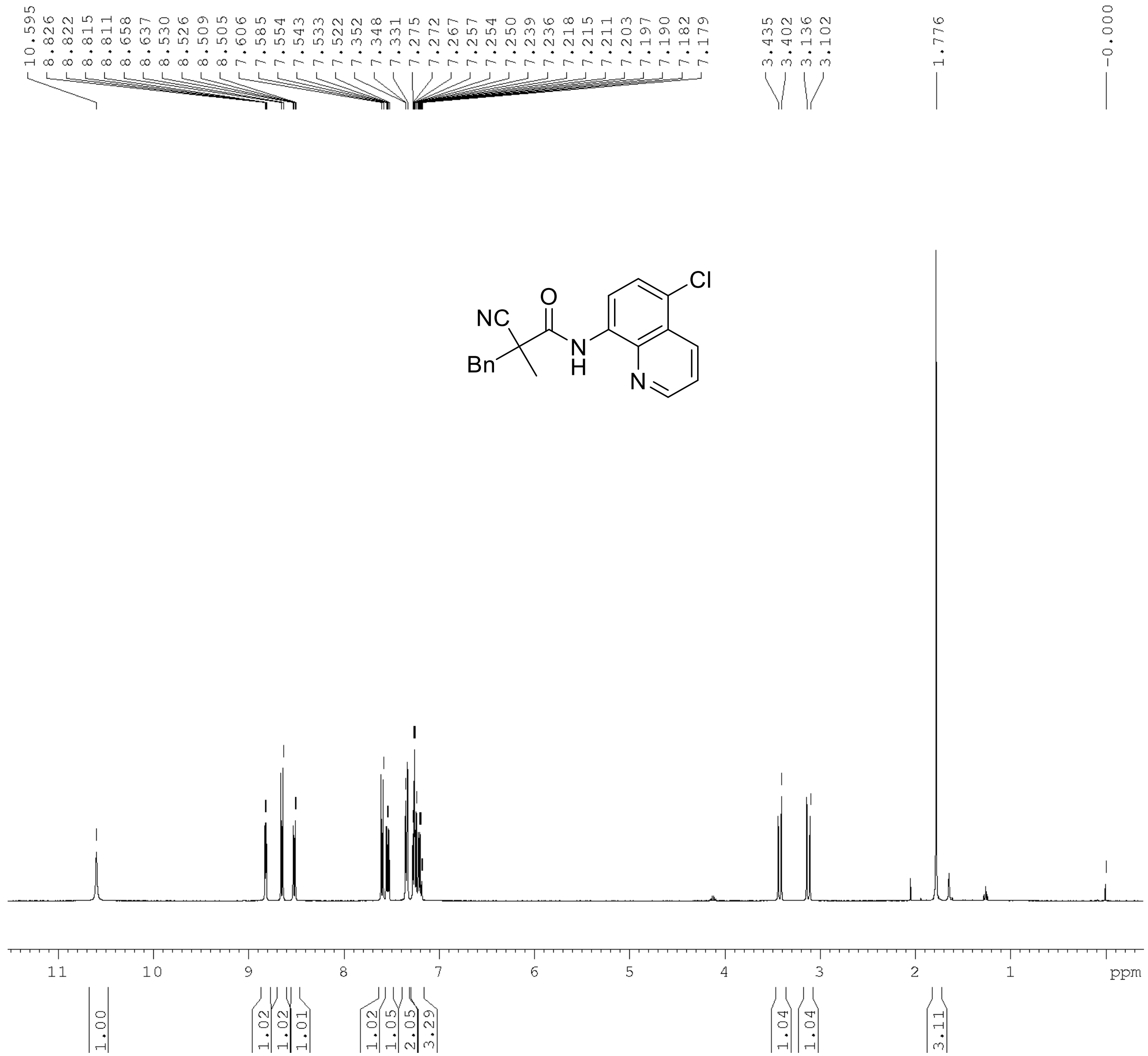

$^{13}C\{^1H\}$NMR spectrum of compound **2i** ($CDCl_3$, 100 MHz)**:**

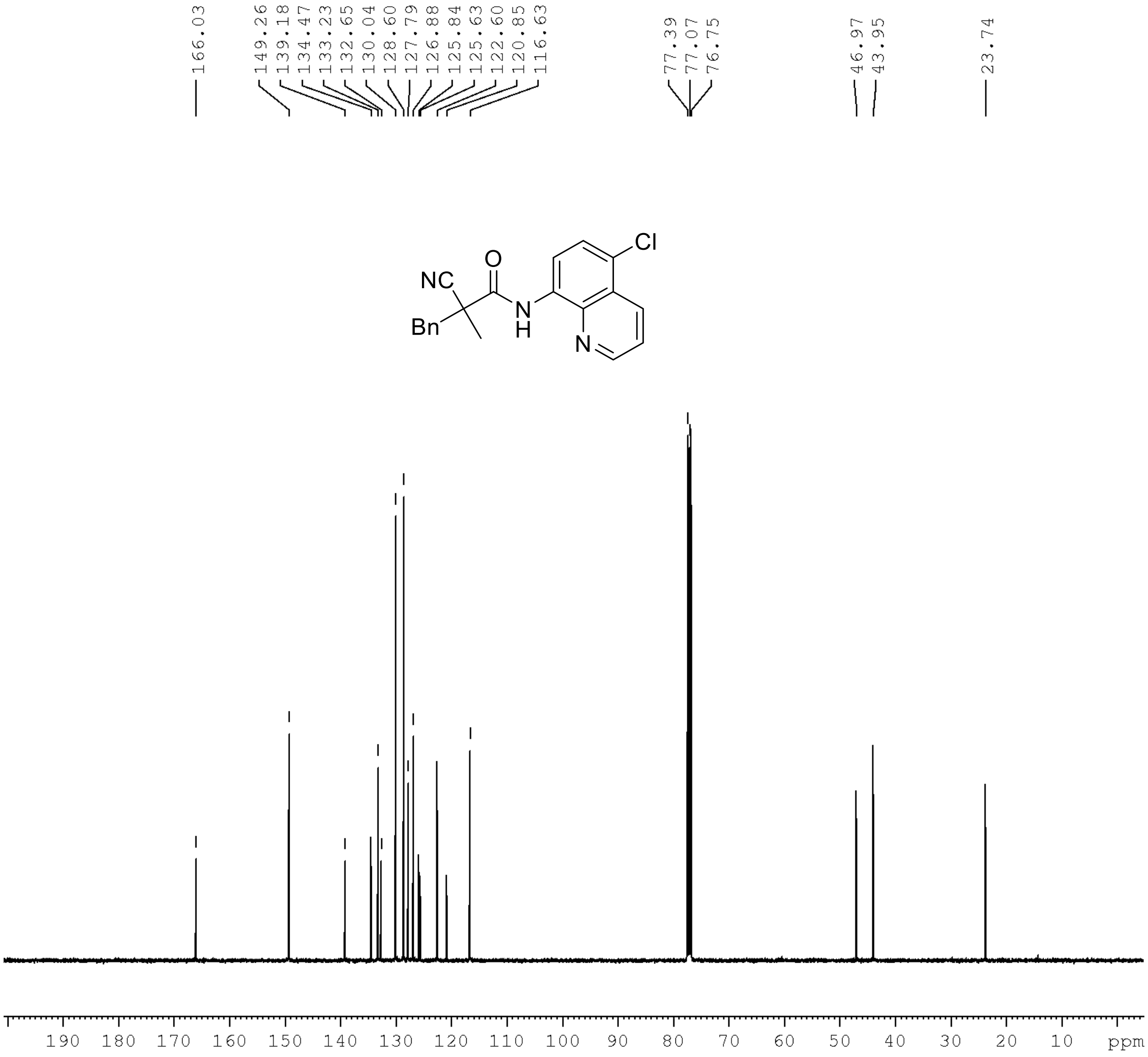

DEPT135 spectrum of compound **2i** ($CDCl_3$, 100 MHz)**:**

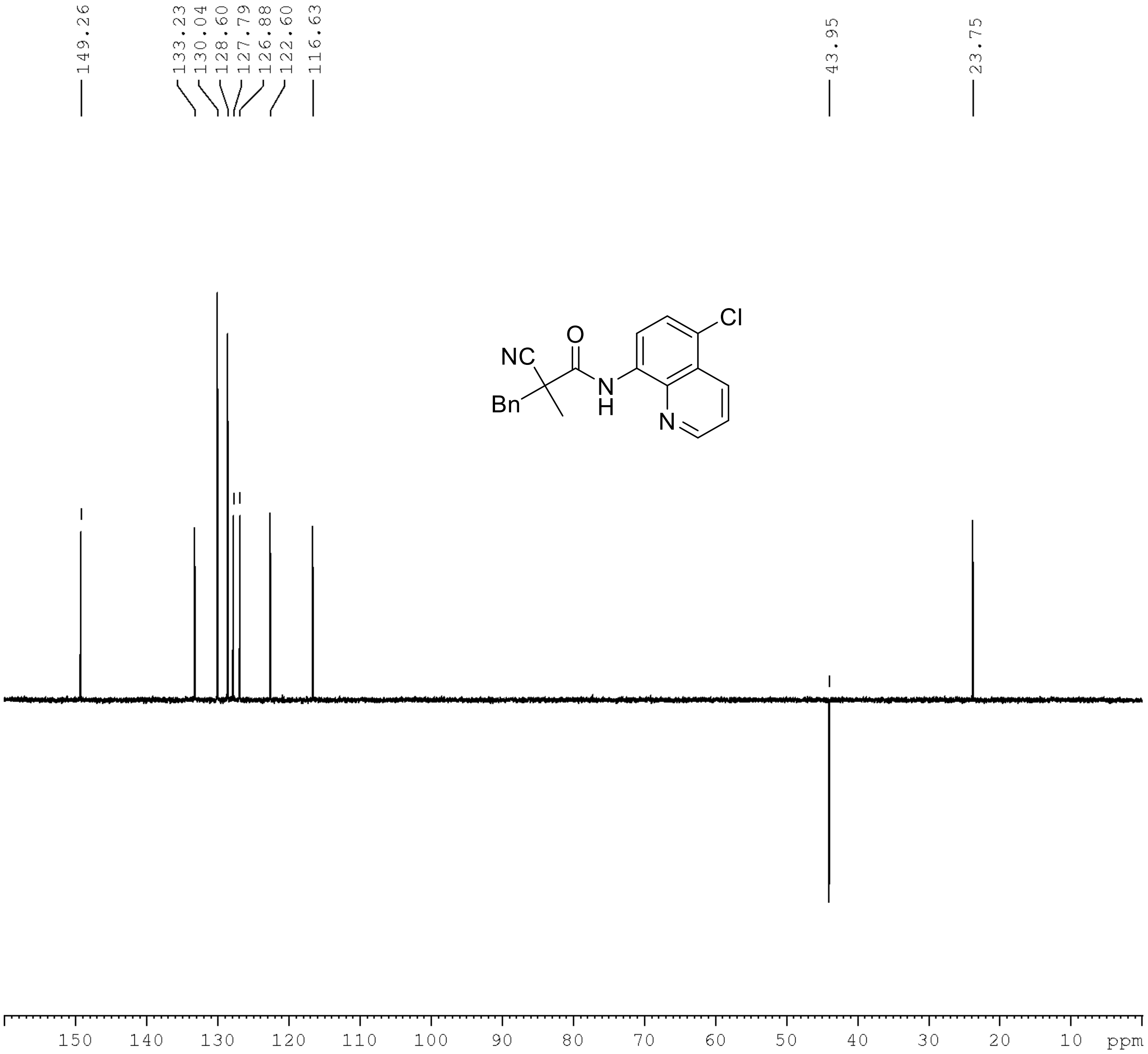

HRMS spectrum of compound **2i:**

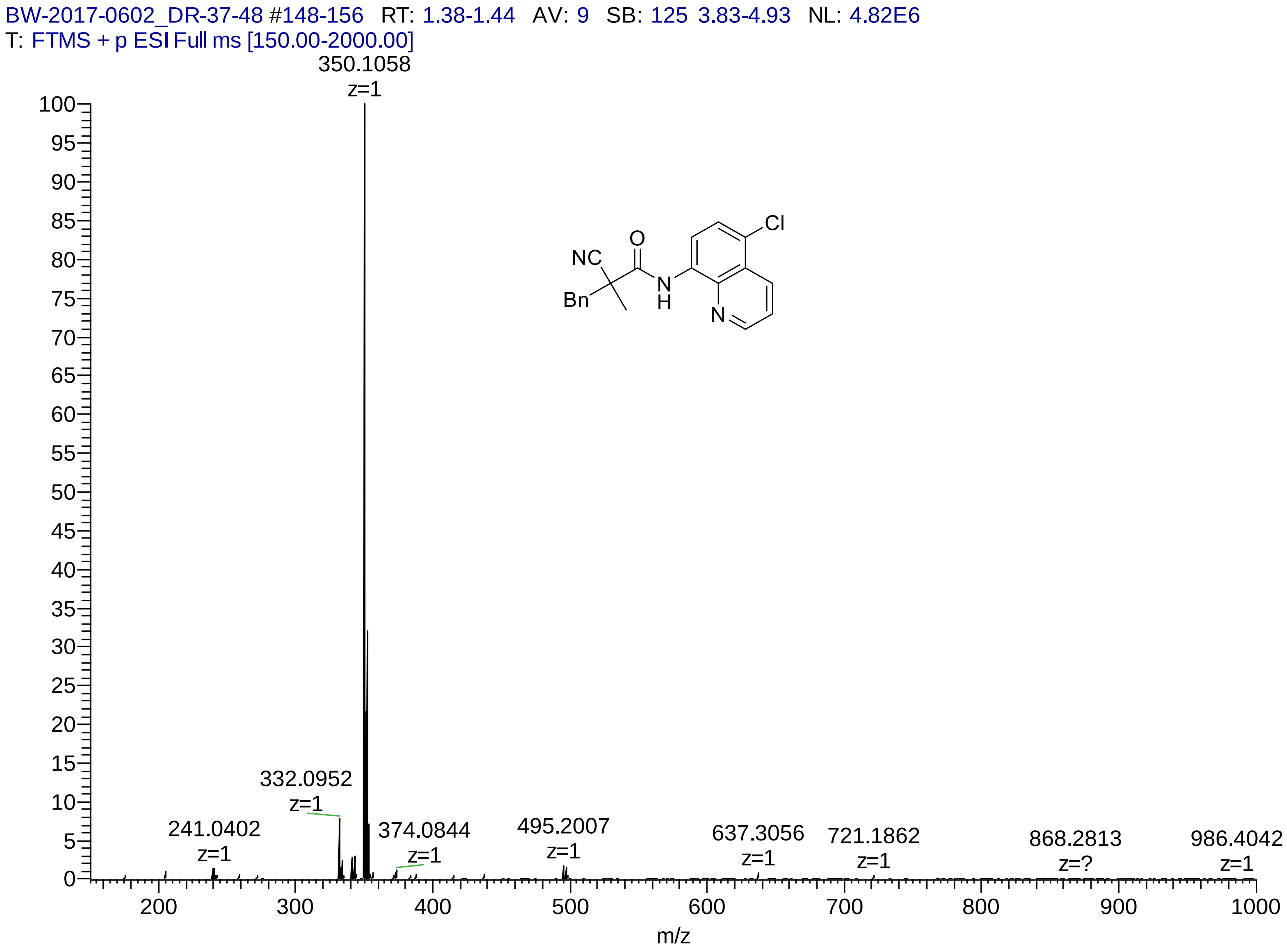

^{1}H NMR spectrum of compound **3i** ($CDCl_3$, 400 MHz)**:**

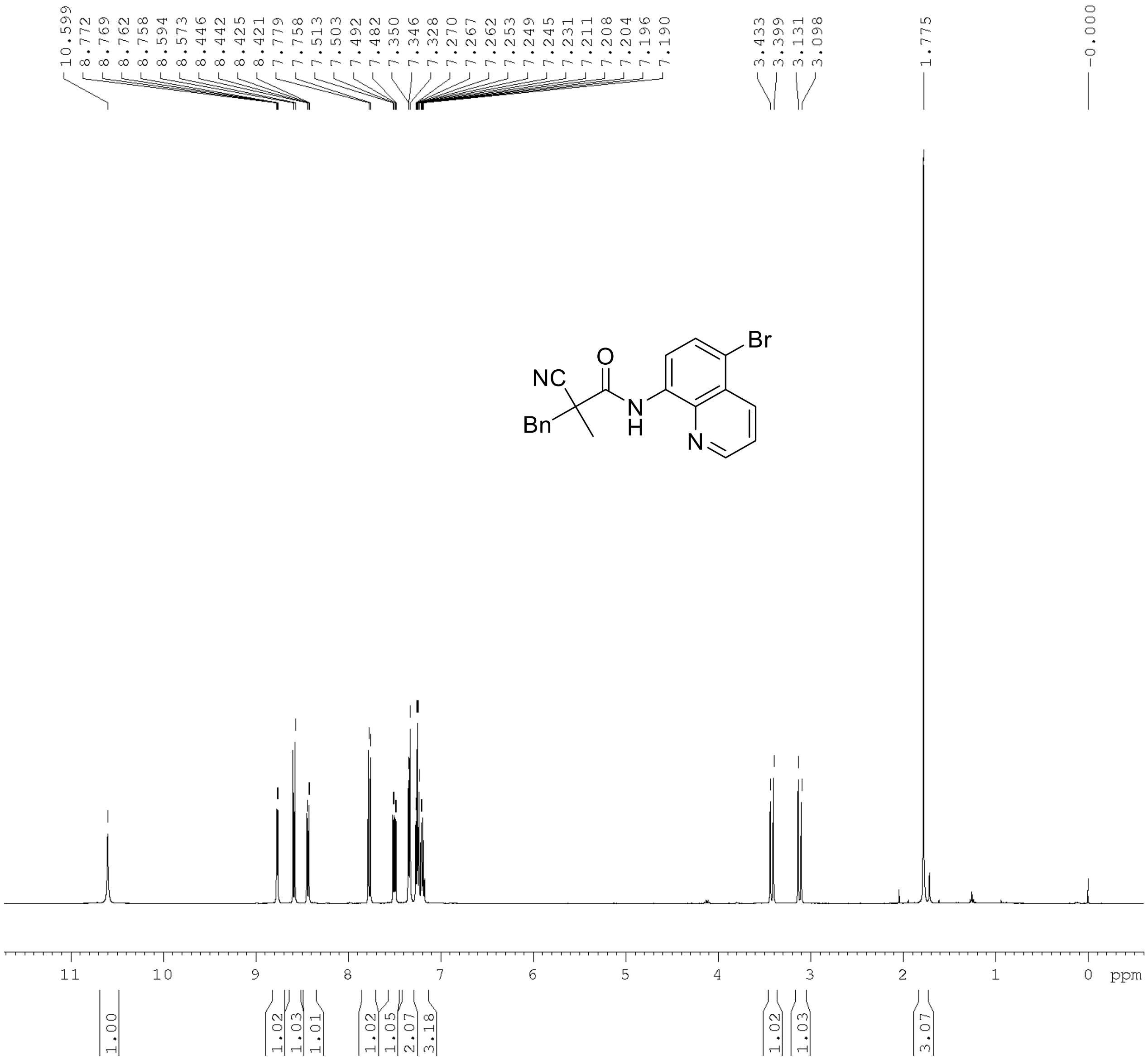

$^{13}C\{^{1}H\}$NMR spectrum of compound **3i** ($CDCl_3$, 100 MHz)**:**

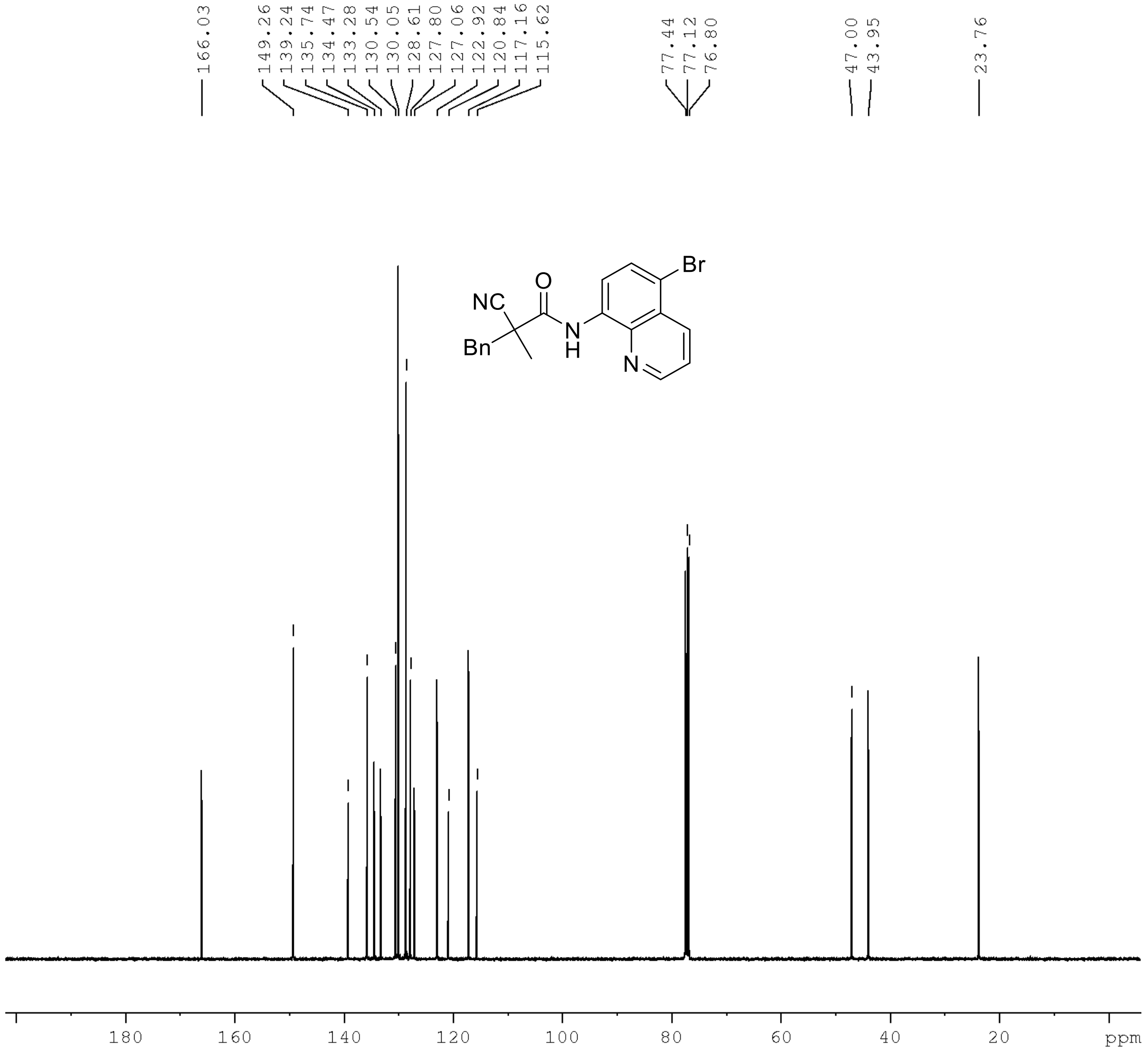

DEPT135 spectrum of compound **3i** ($CDCl_3$, 100 MHz)**:**

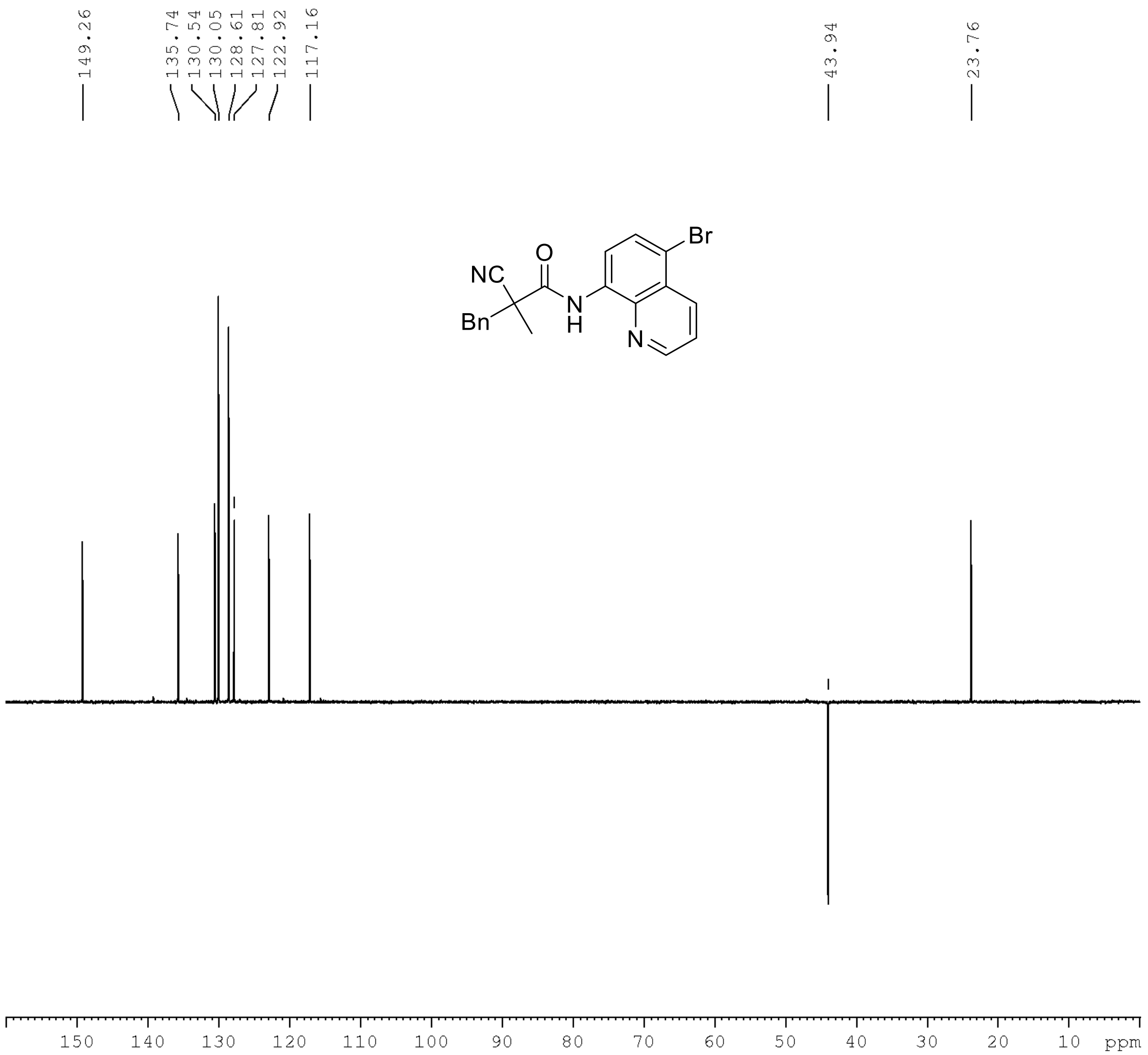

HRMS spectrum of compound **3i:**

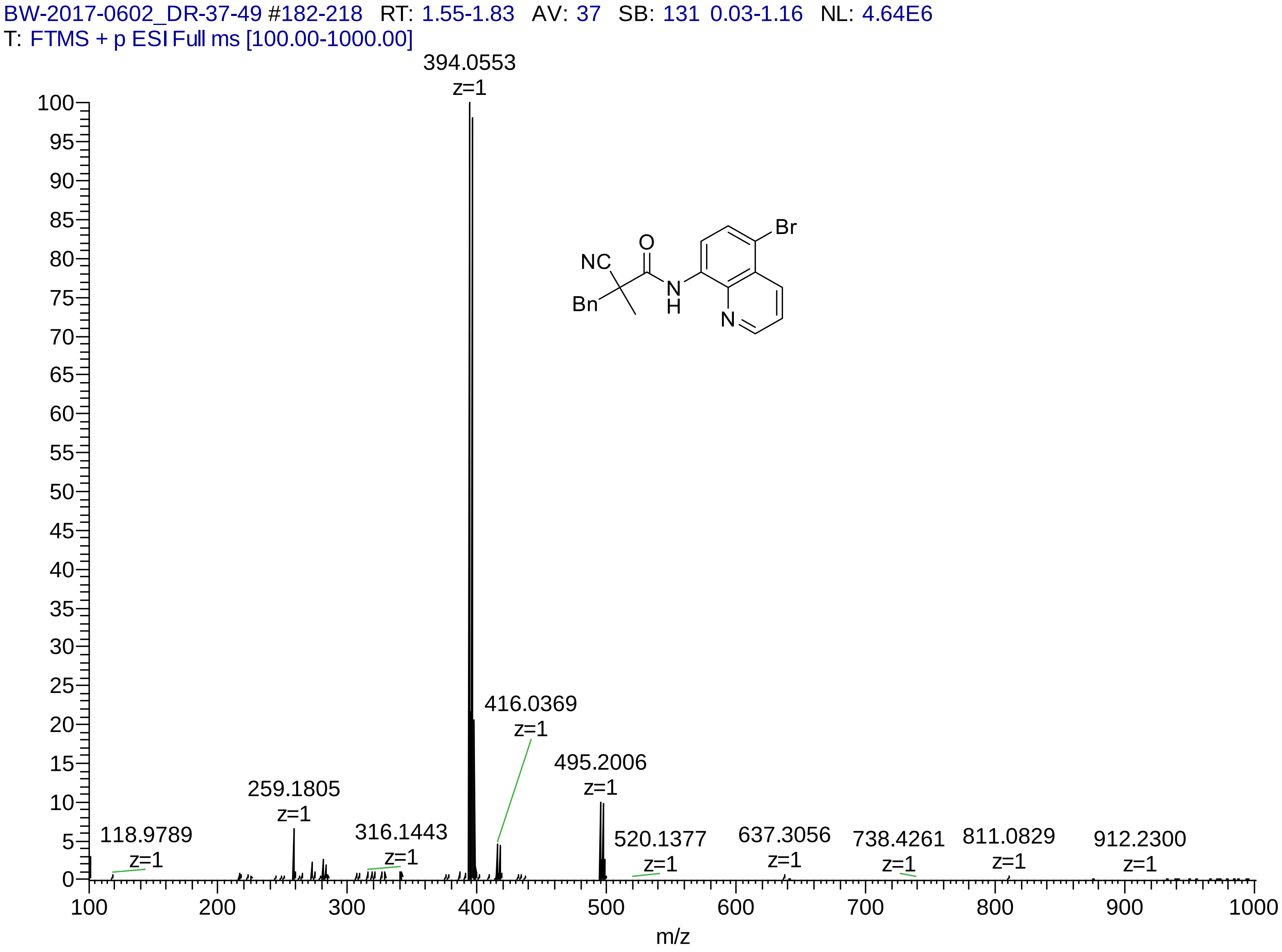

^{1}H NMR spectrum of compound **2j** ($CDCl_3$, 400 MHz)**:**

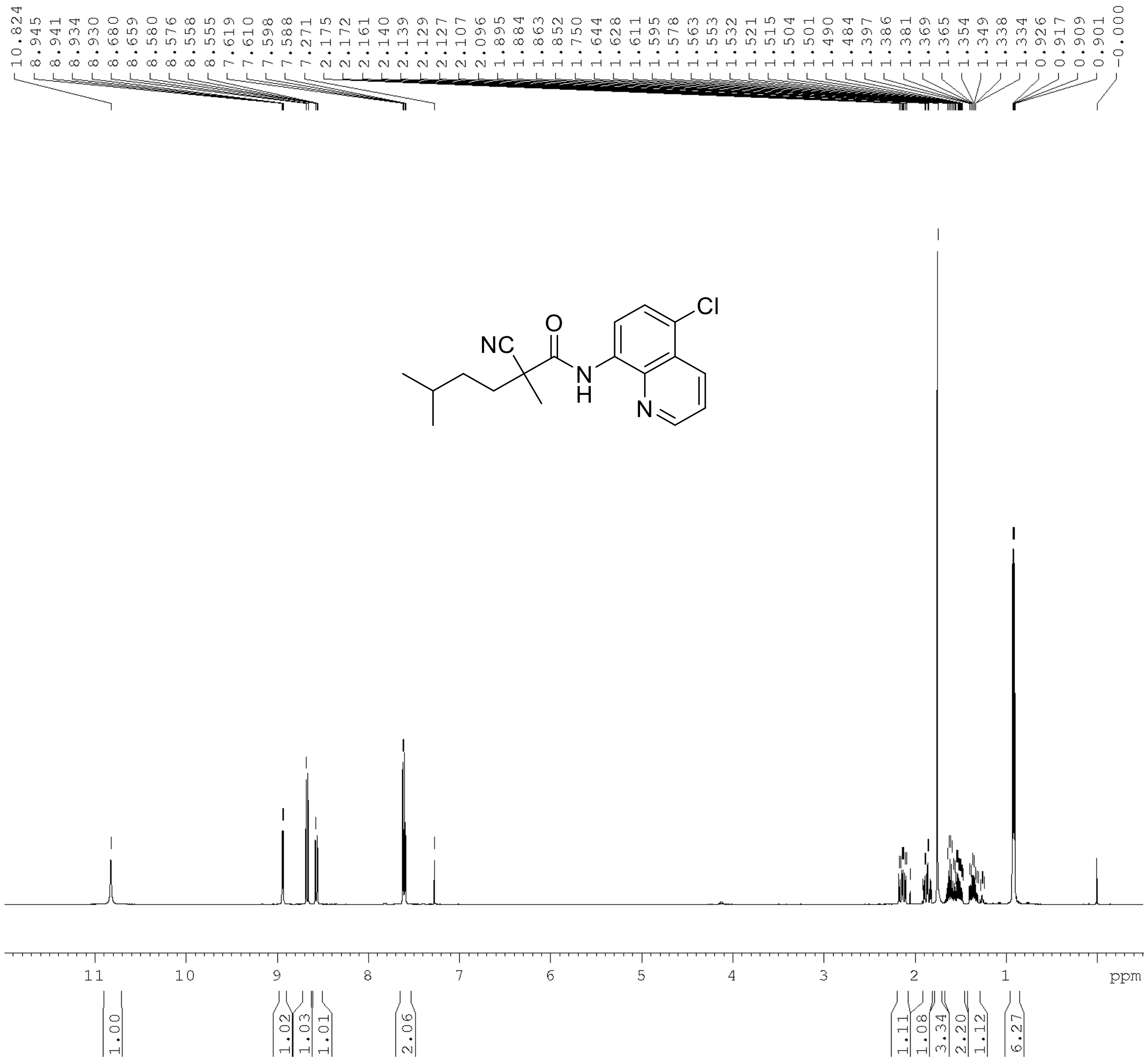

$^{13}C\{^1H\}$NMR spectrum of compound **2j** ($CDCl_3$, 100 MHz)**:**

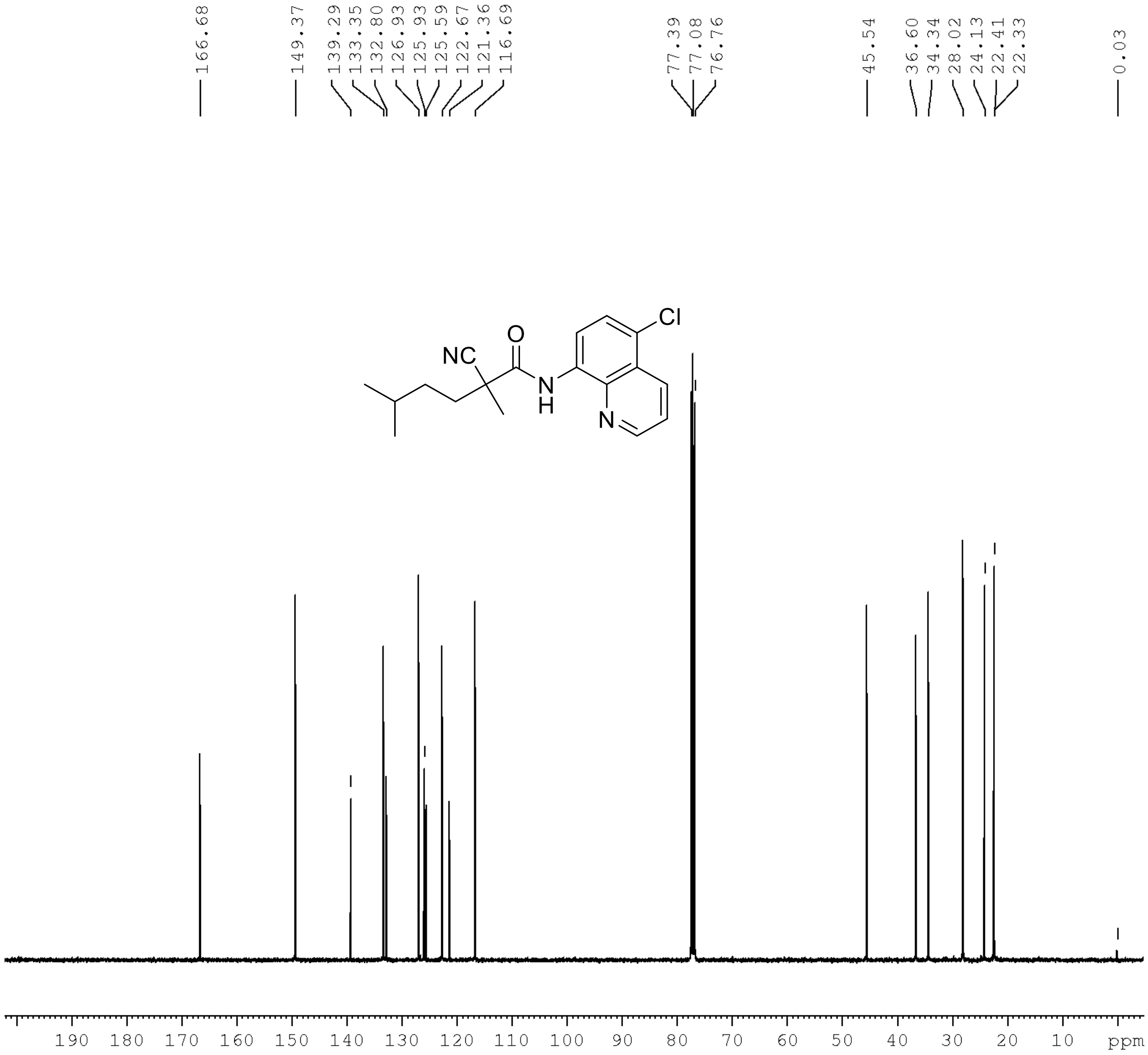

DEPT135 spectrum of compound **2j** ($CDCl_3$, 100 MHz):

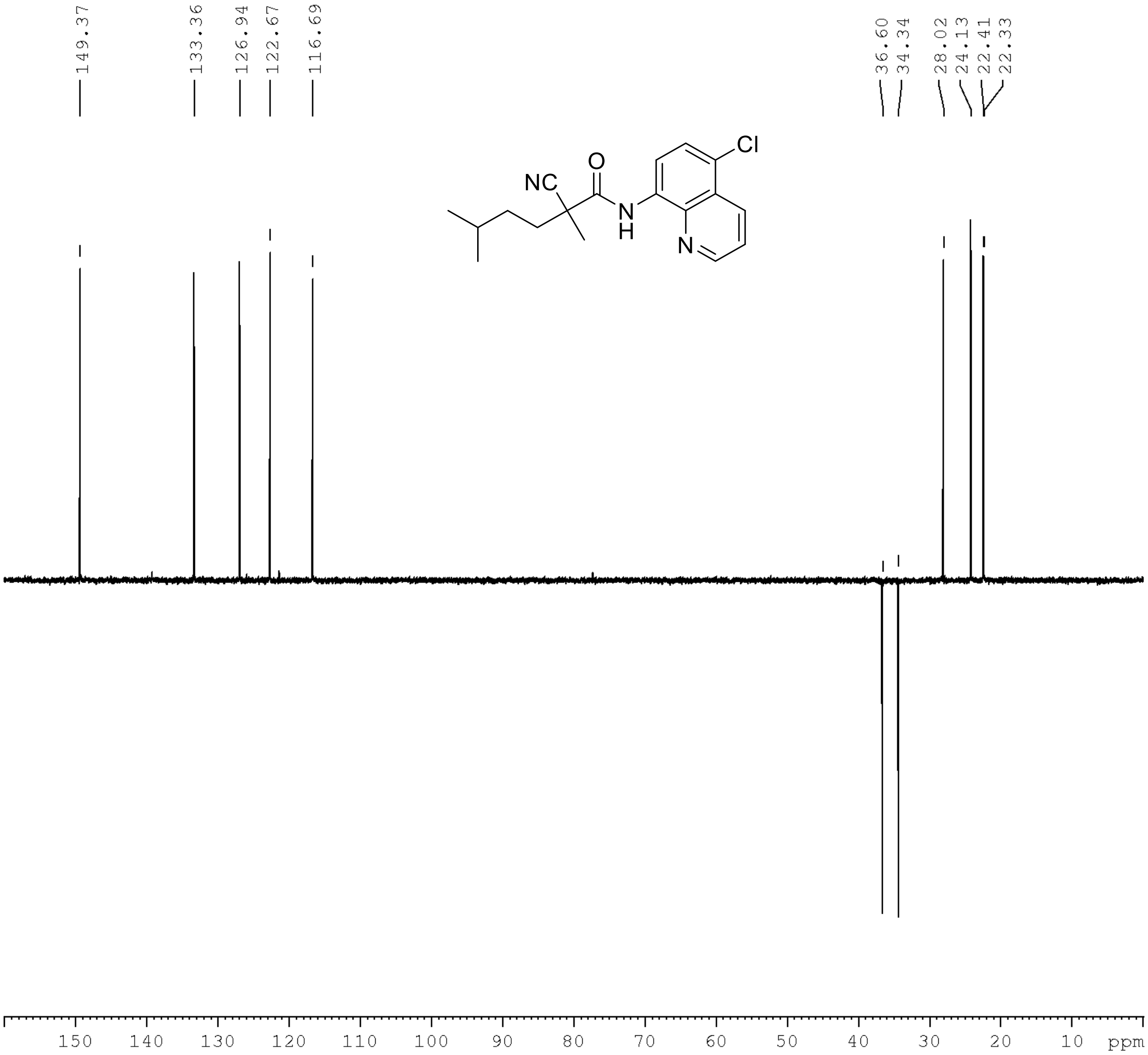

^{1}H NMR spectrum of compound **3j** ($CDCl_3$, 400 MHz)**:**

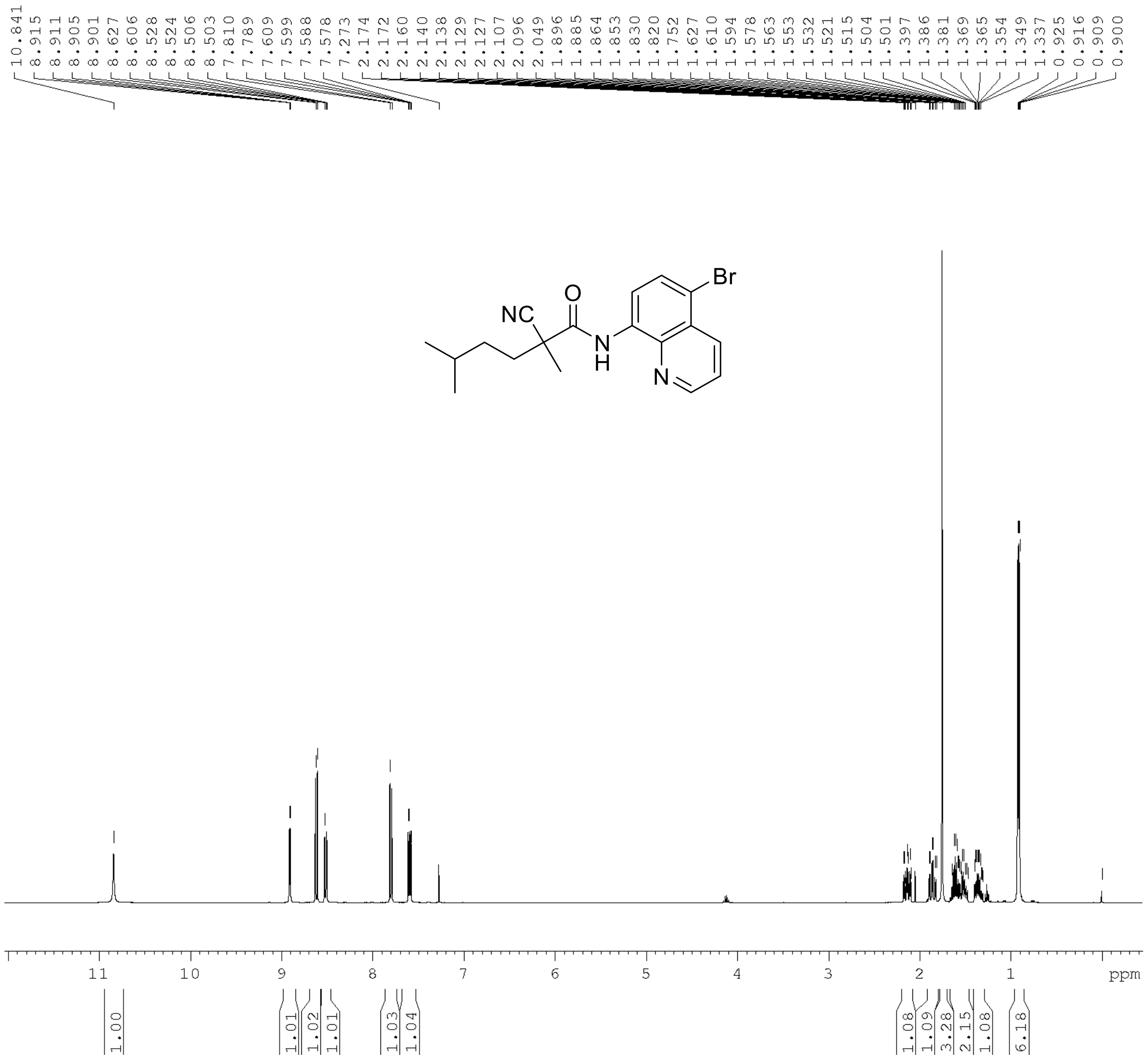

$^{13}C\{^{1}H\}$NMR spectrum of compound **3j** ($CDCl_3$, 100 MHz)**:**

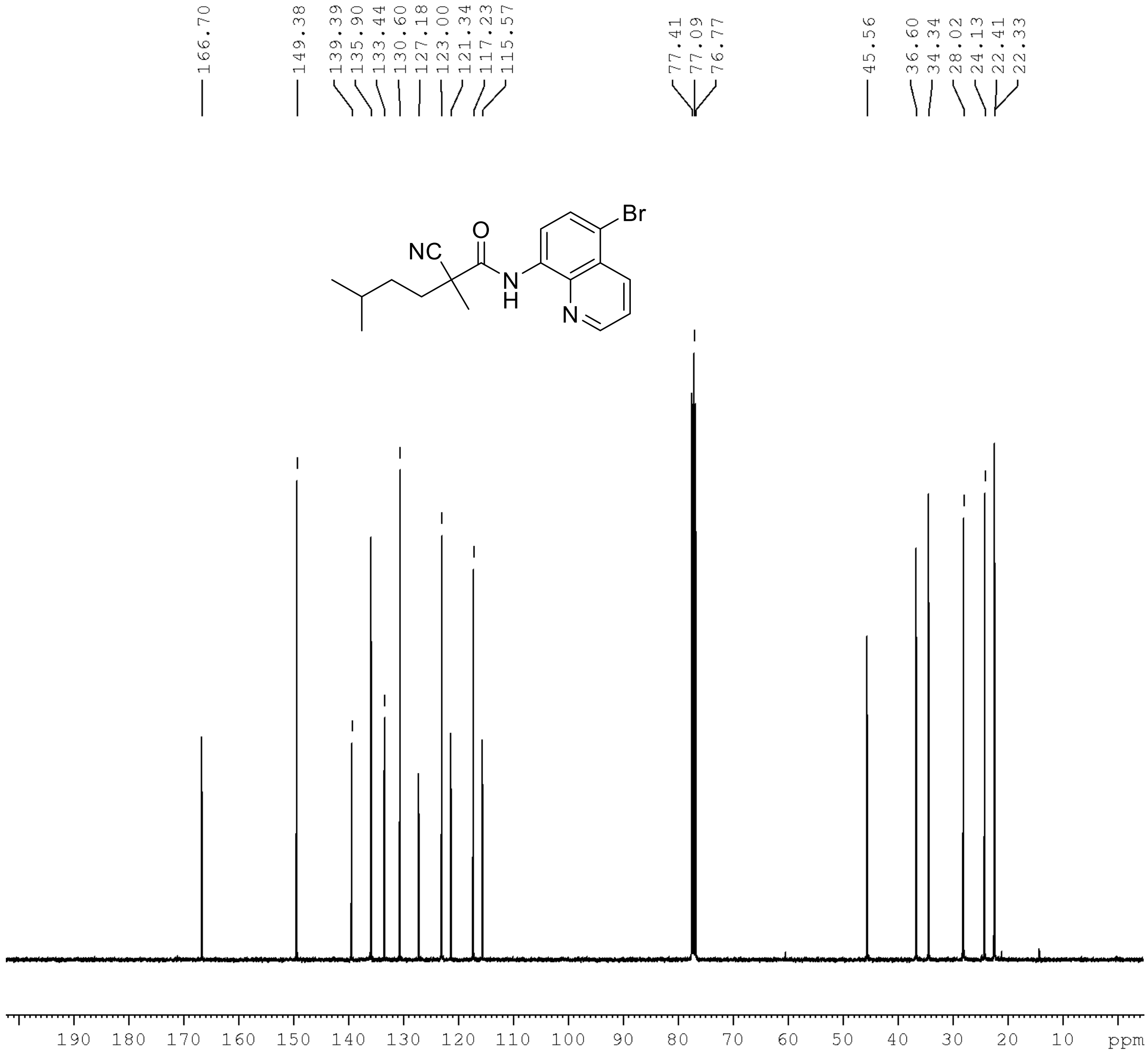

DEPT135 spectrum of compound **3j** ($CDCl_3$, 100 MHz)**:**

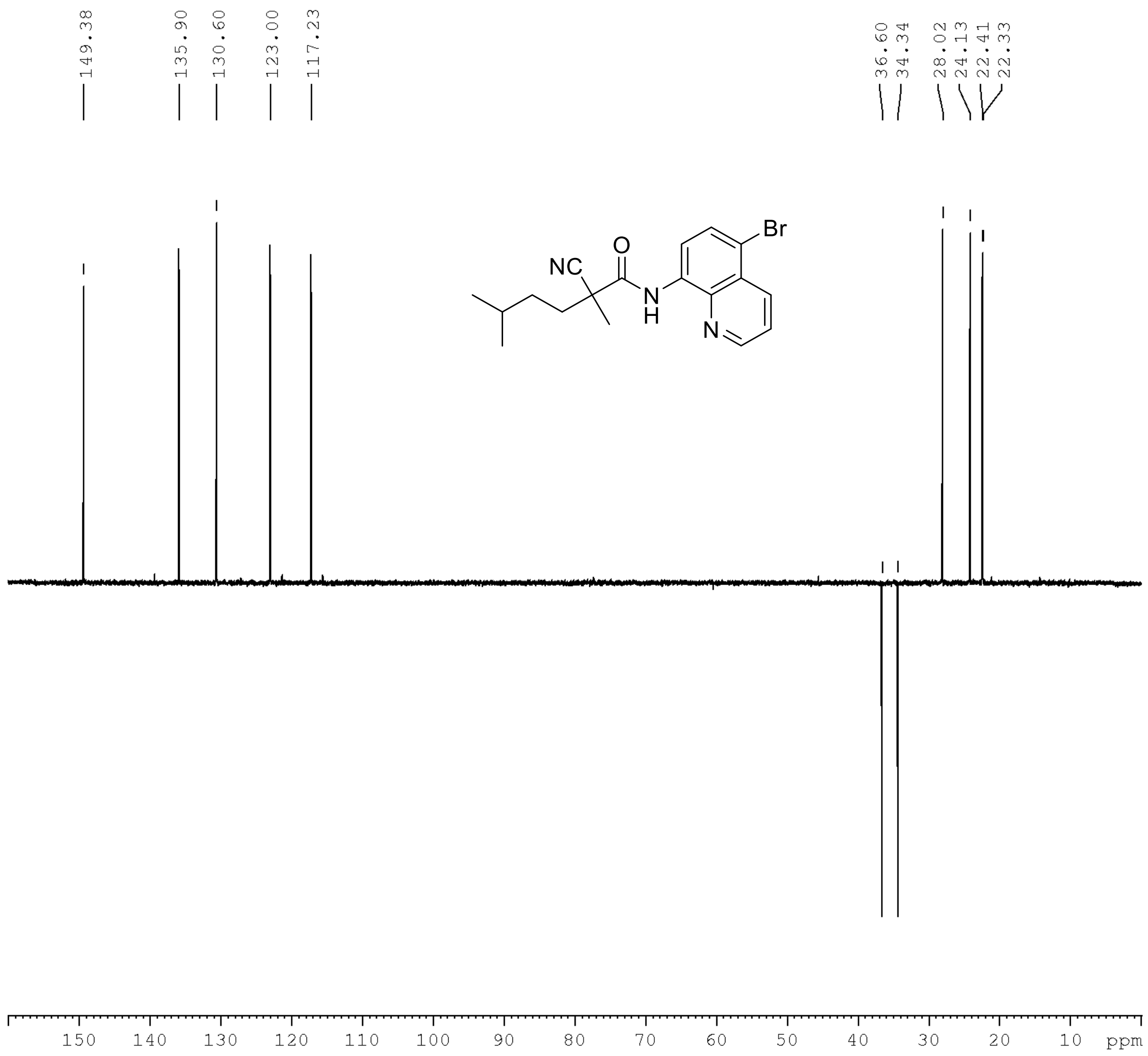

HRMS spectrum of compound **3j:**

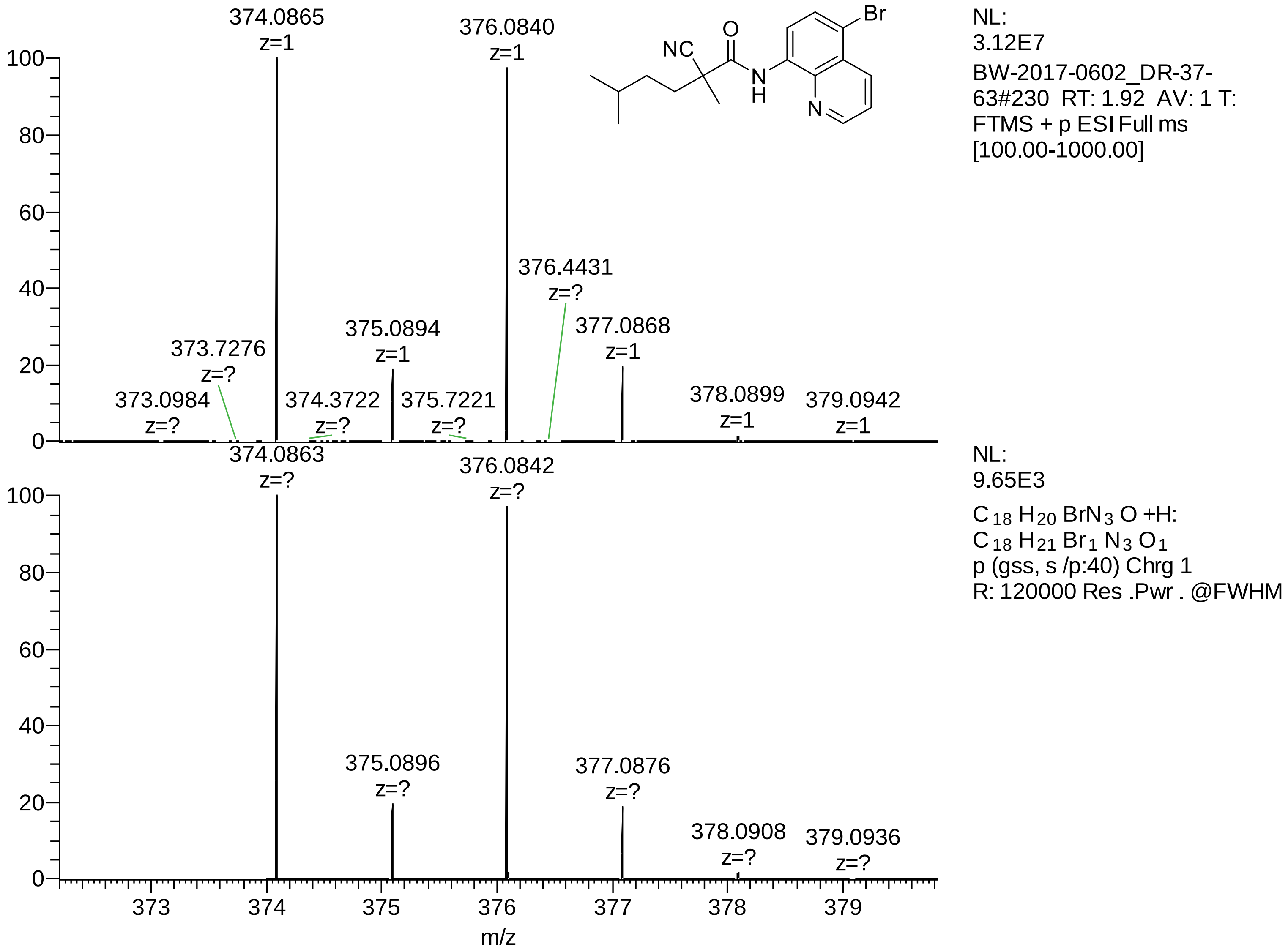

^{1}H NMR spectrum of compound **2k** ($CDCl_3$, 400 MHz)**:**

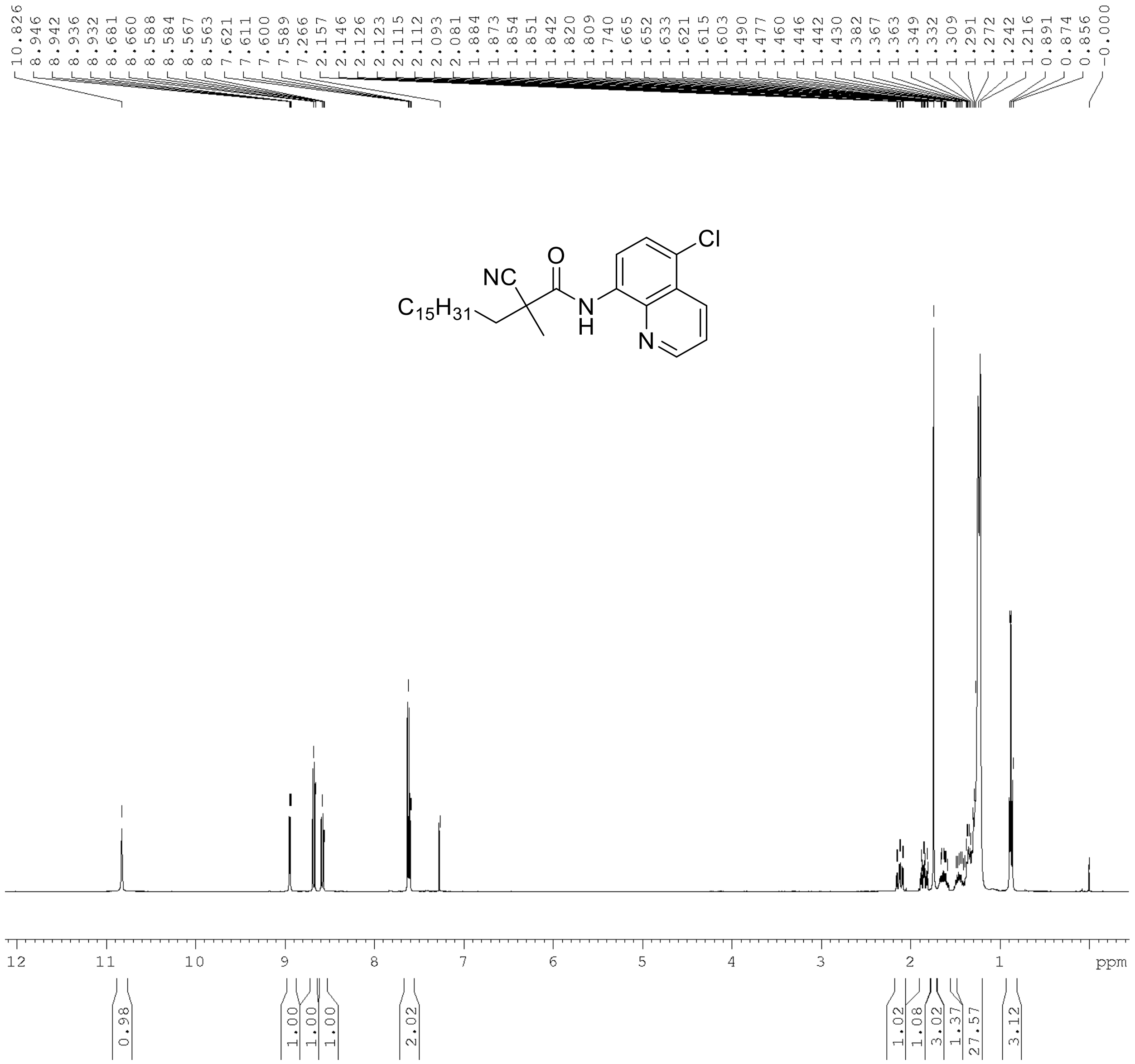

$^{13}C\{^{1}H\}$NMR spectrum of compound **2k** ($CDCl_3$, 100 MHz)**:**

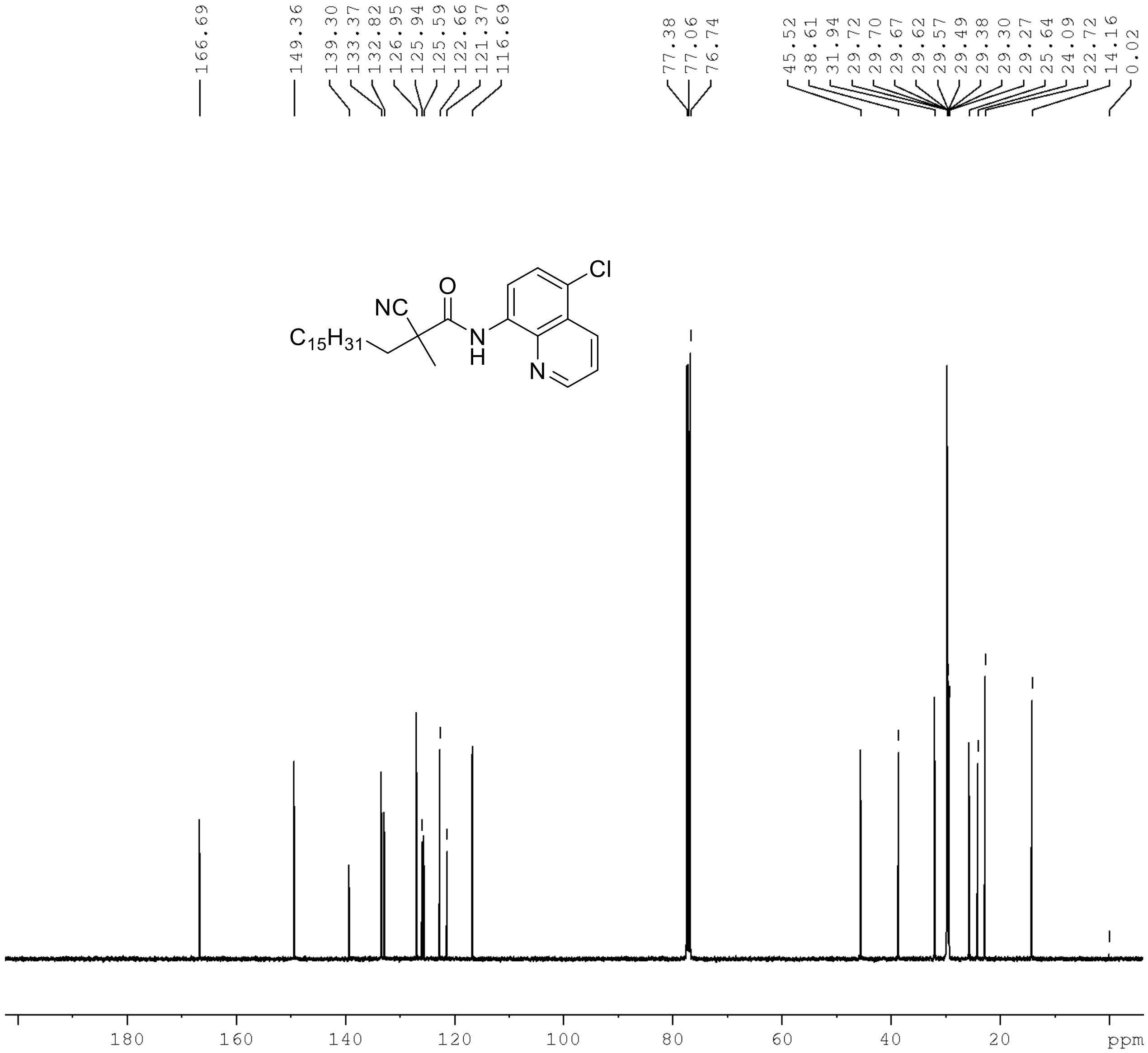

HRMS spectrum of compound **2k:**

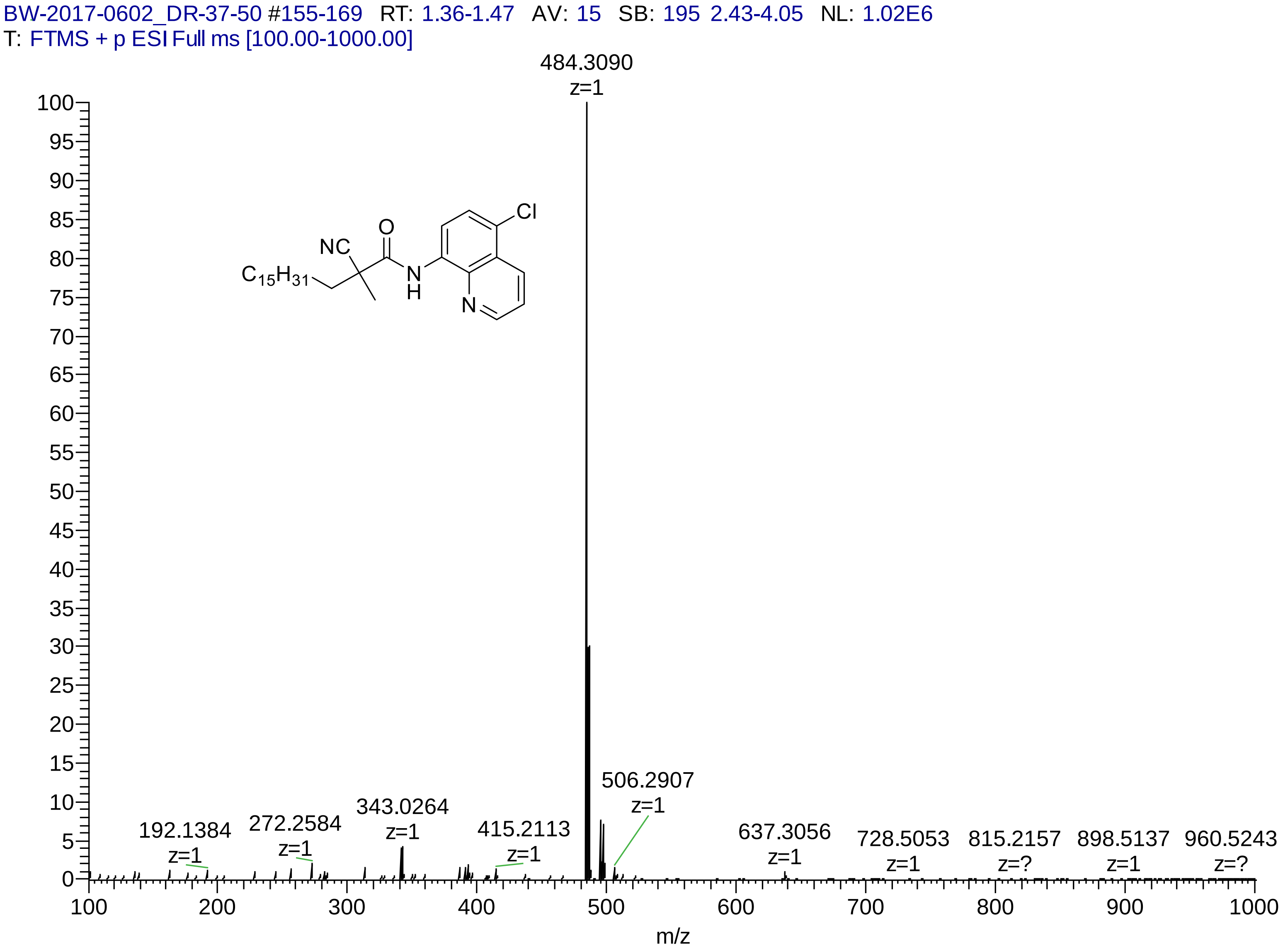

^{1}H NMR spectrum of compound **3k** ($CDCl_3$, 400 MHz)**:**

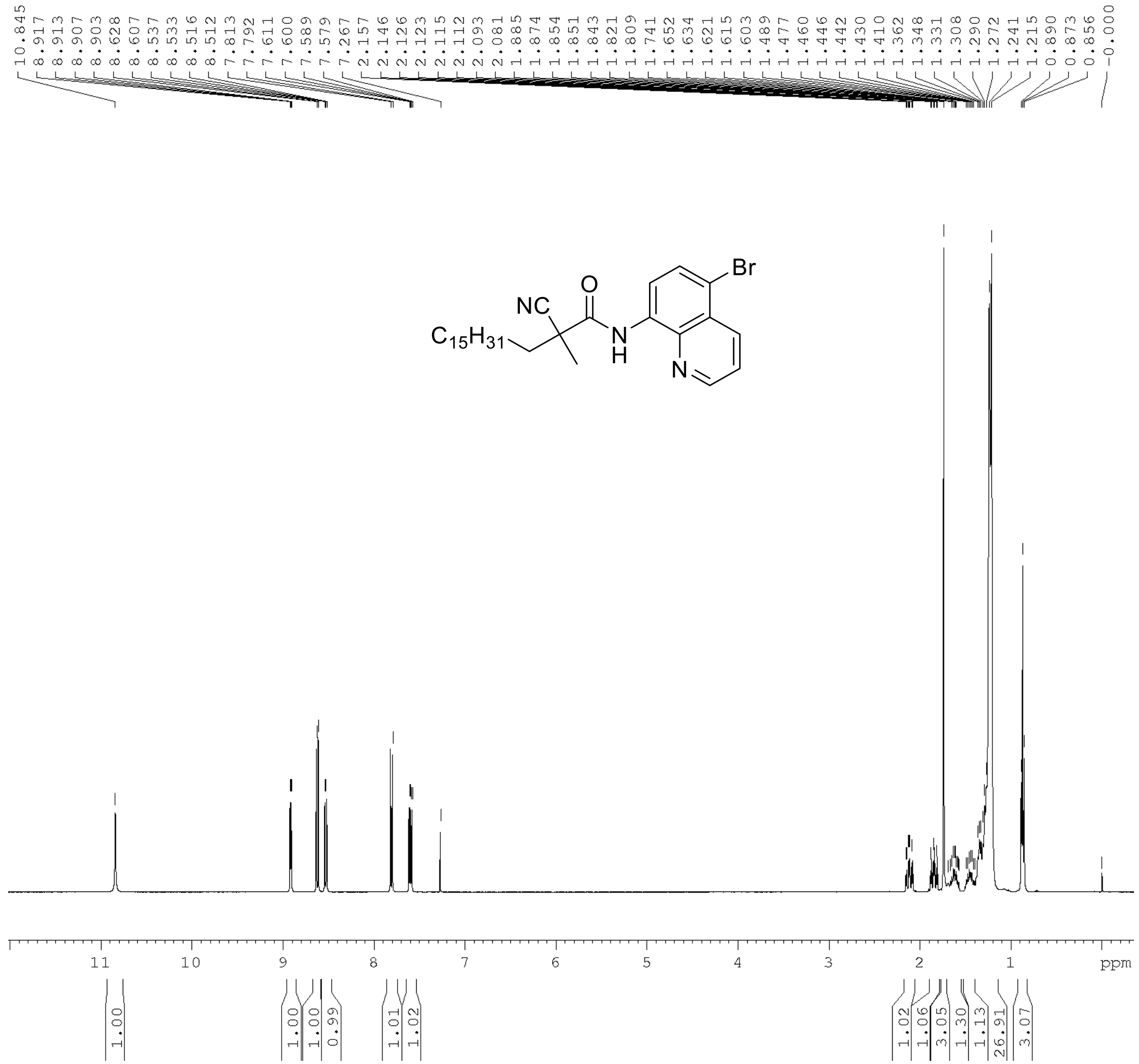

$^{13}C\{^1H\}$NMR spectrum of compound **3k** ($CDCl_3$, 100 MHz)**:**

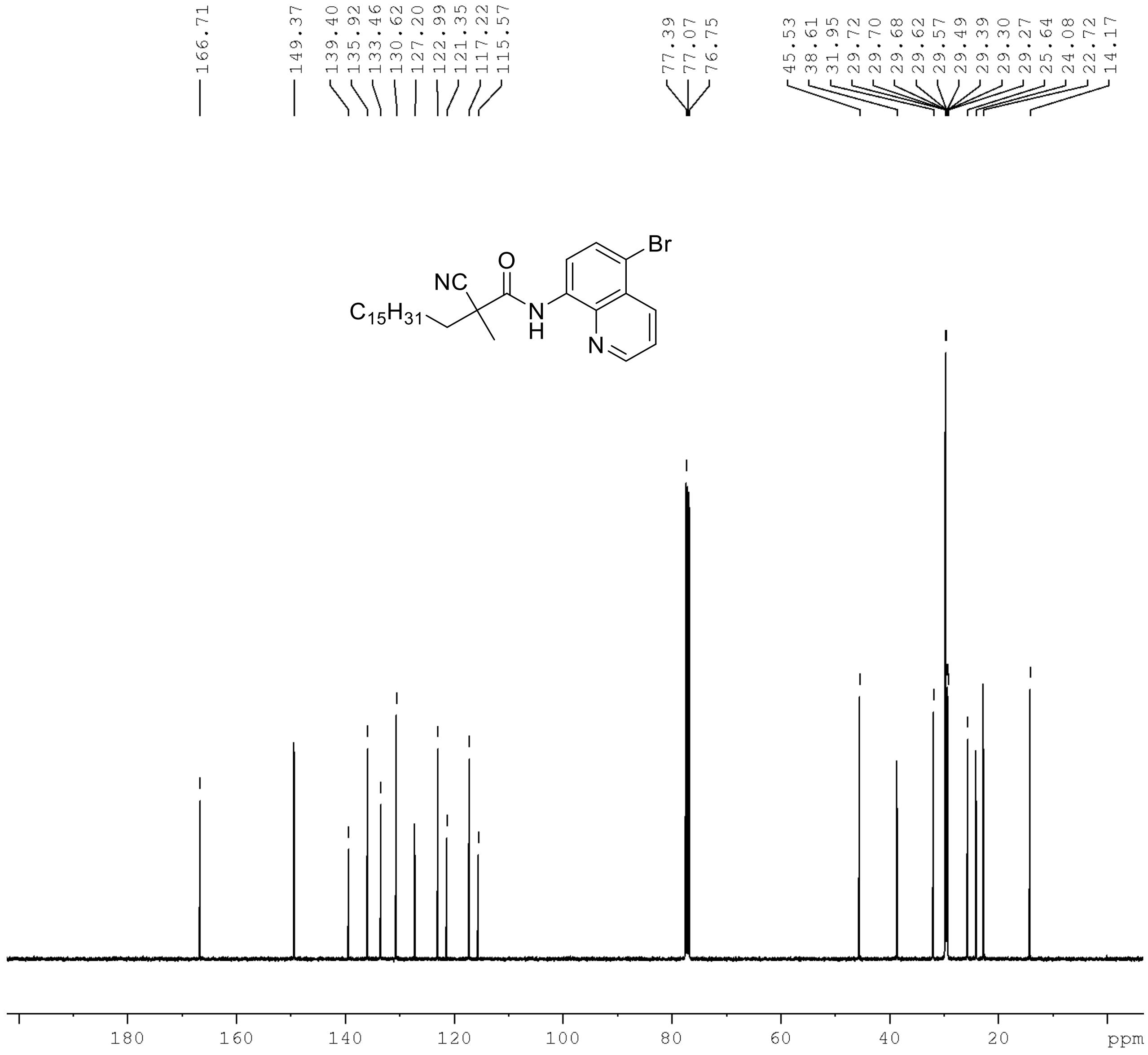

HRMS spectrum of compound **3k:**

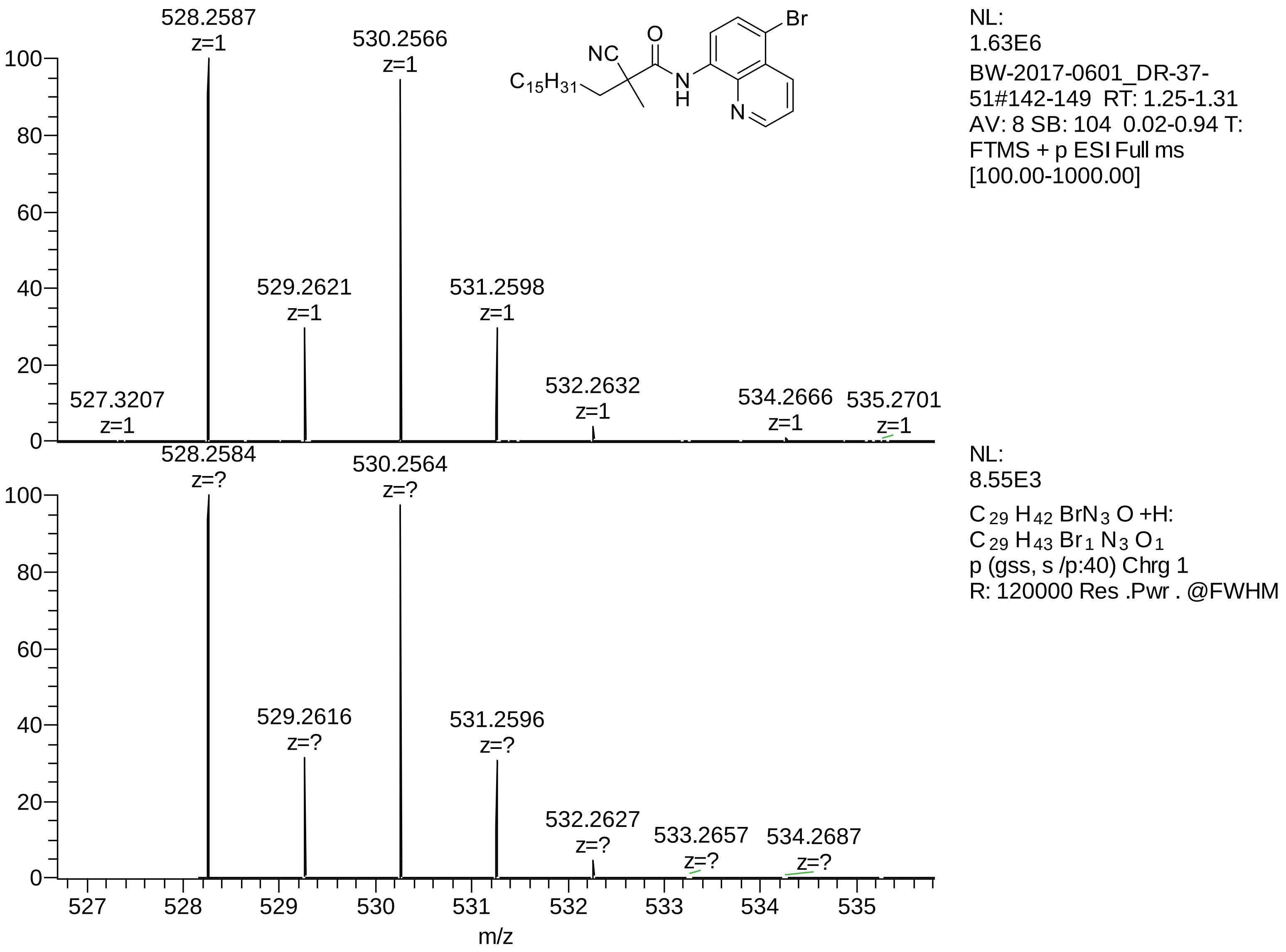

^{1}H NMR spectrum of compound **2l** ($CDCl_3$, 400 MHz)**:**

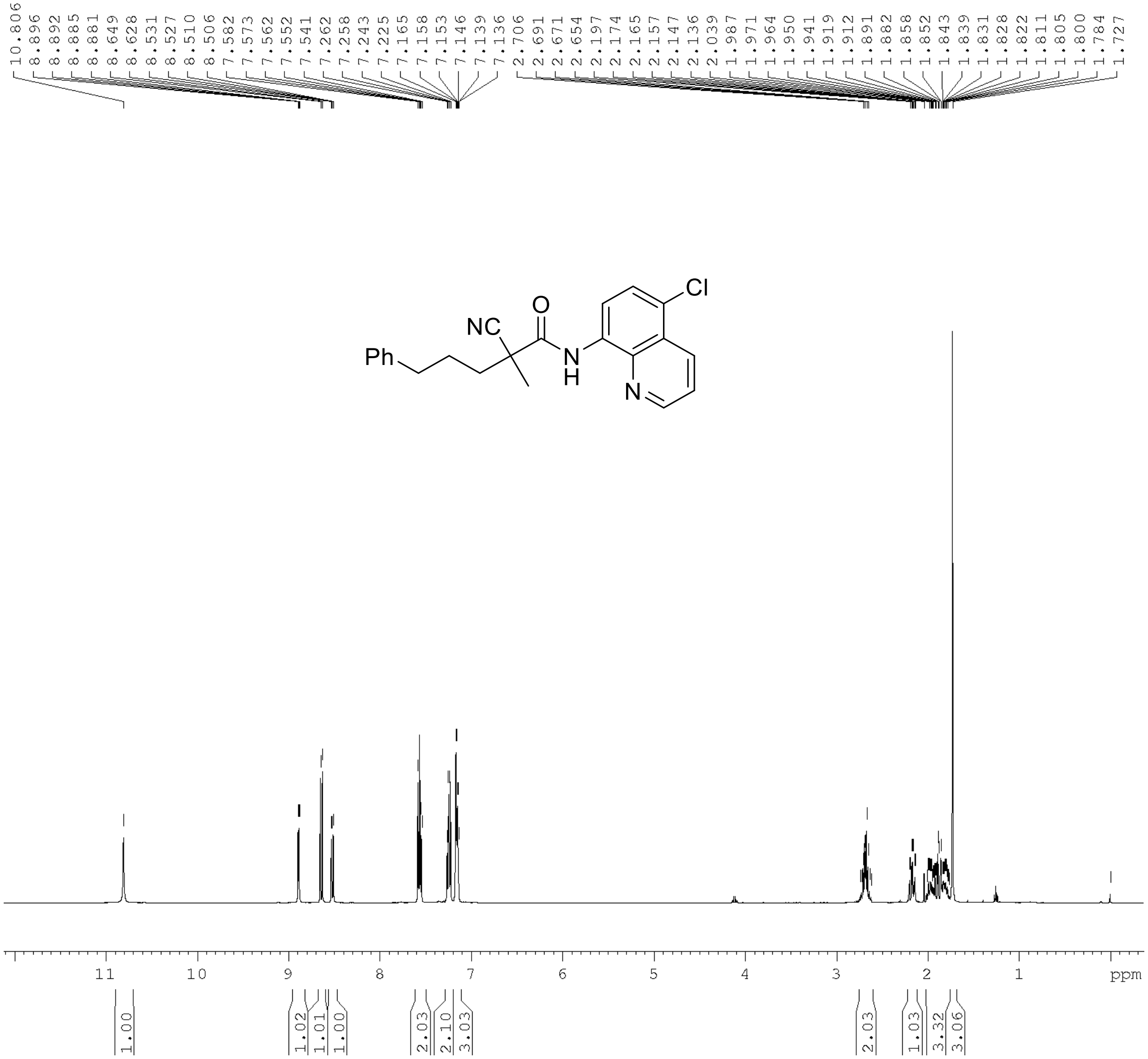

$^{13}C\{^1H\}$NMR spectrum of compound **2l** ($CDCl_3$, 100 MHz)**:**

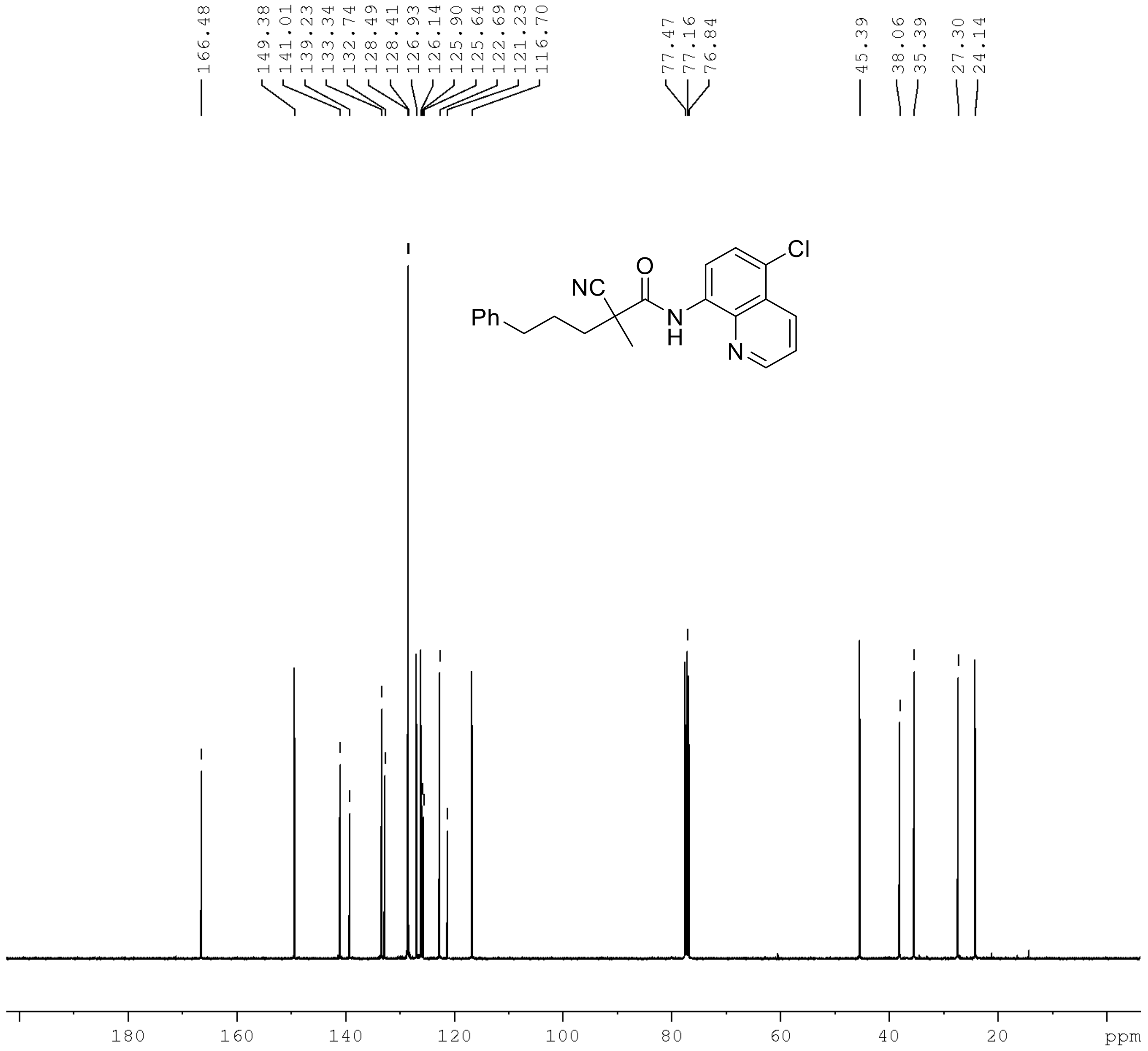

DEPT135 spectrum of compound **2l** ($CDCl_3$, 100 MHz)**:**

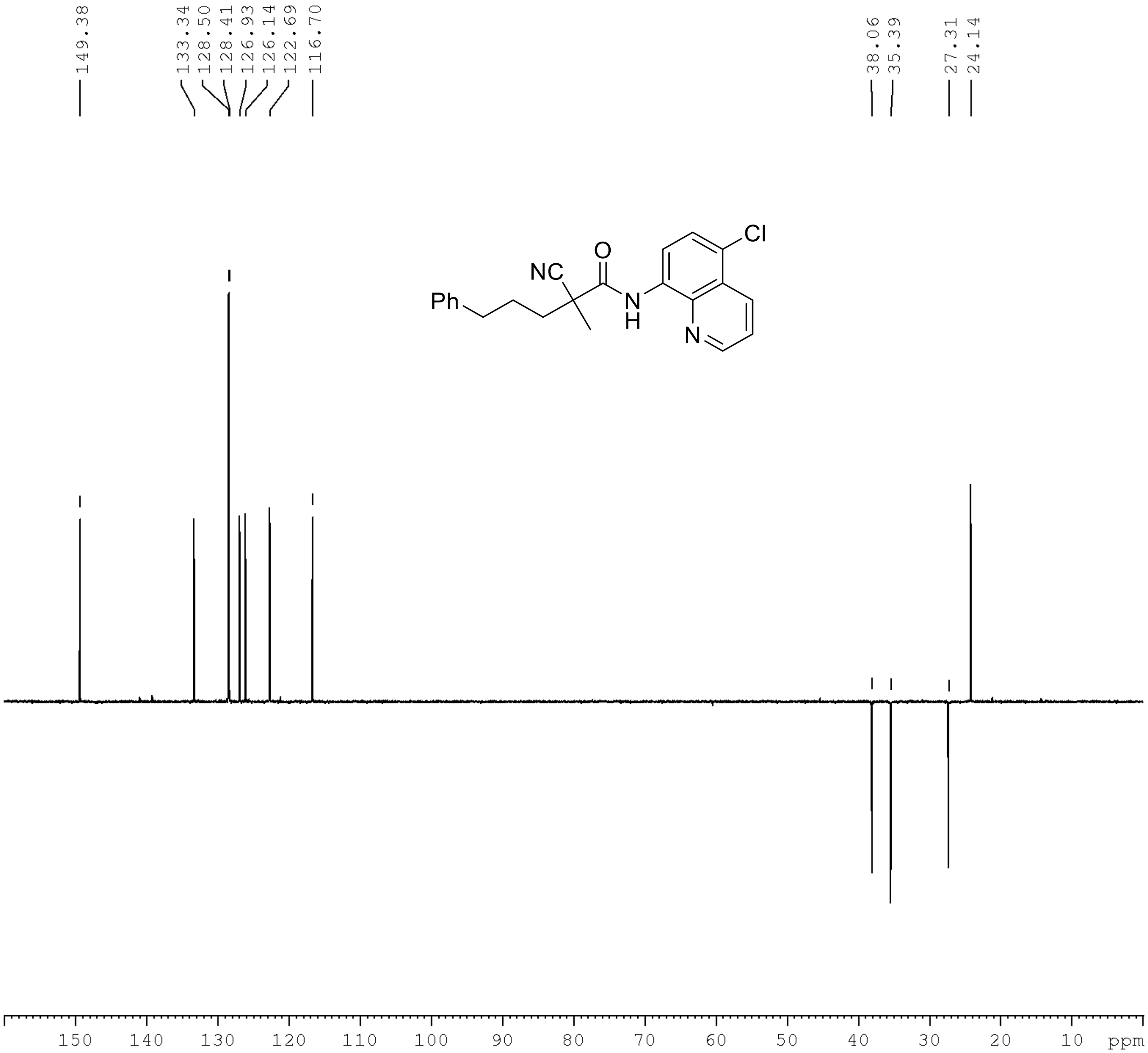

^{1}H NMR spectrum of compound **3l** ($CDCl_3$, 400 MHz)**:**

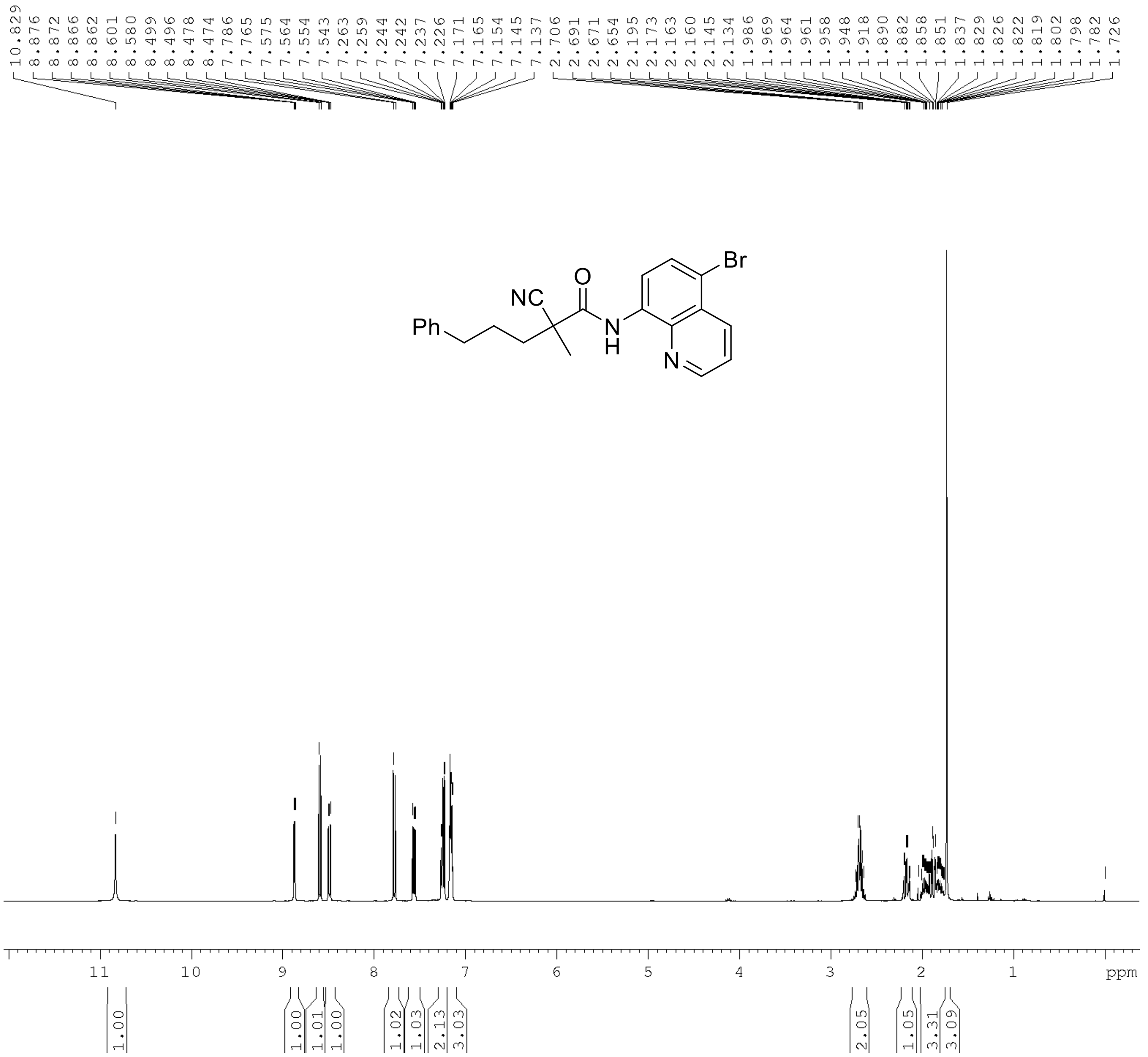

$^{13}C\{^1H\}$NMR spectrum of compound **3l** ($CDCl_3$, 100 MHz)**:**

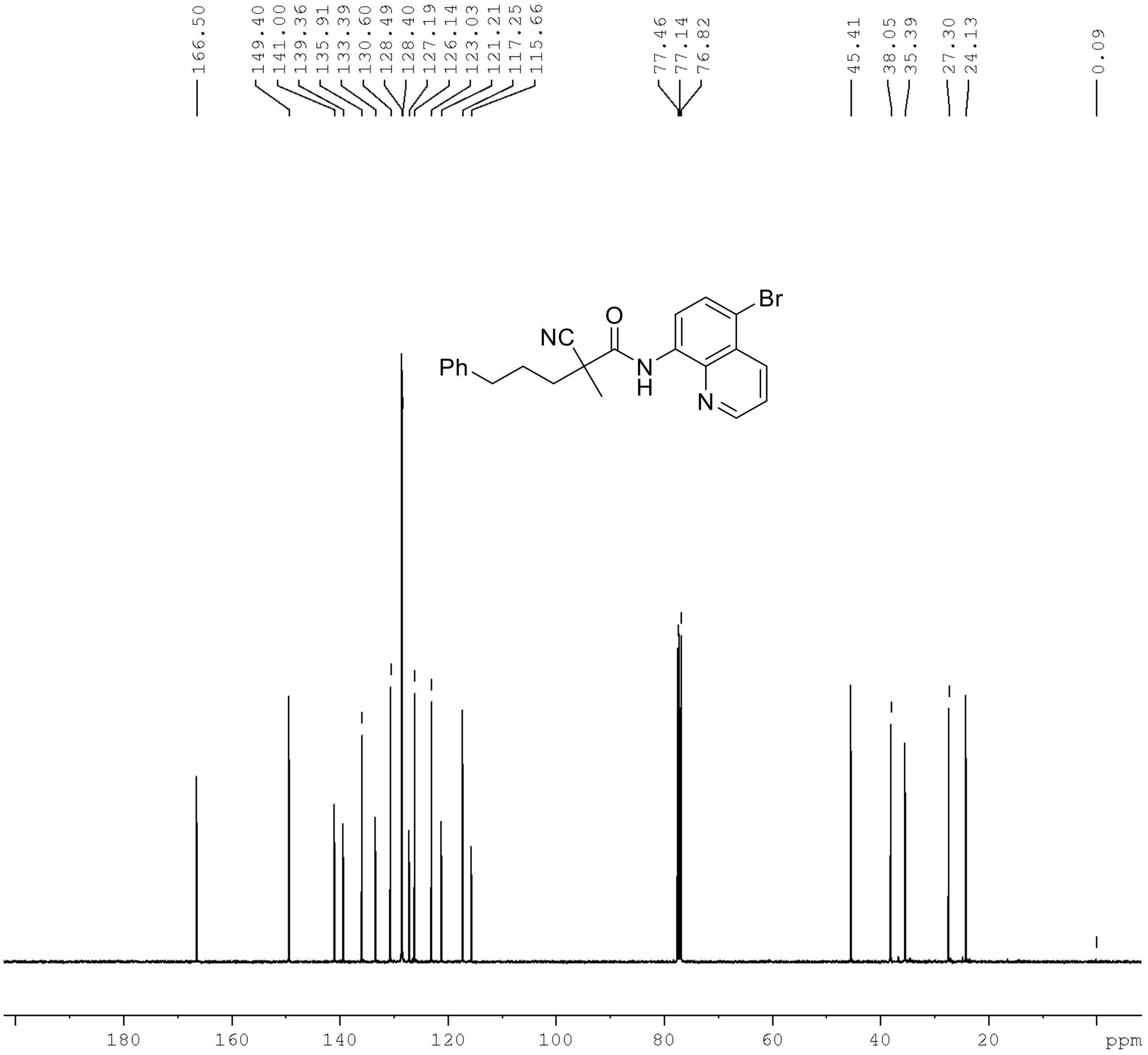

DEPT135 spectrum of compound **3l** ($CDCl_3$, 100 MHz)**:**

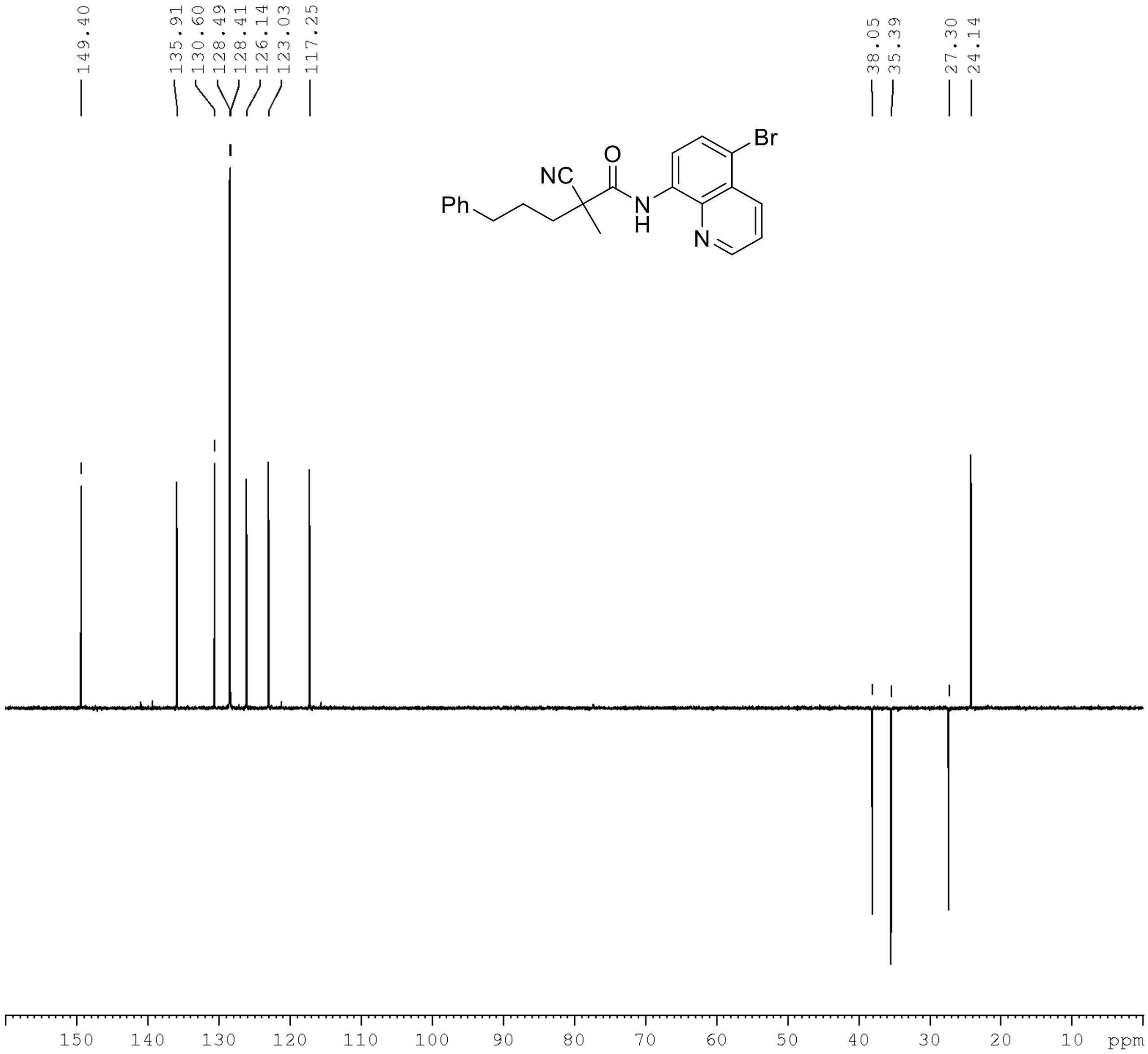

^{1}H NMR spectrum of compound **2m** ($CDCl_3$, 400 MHz)**:**

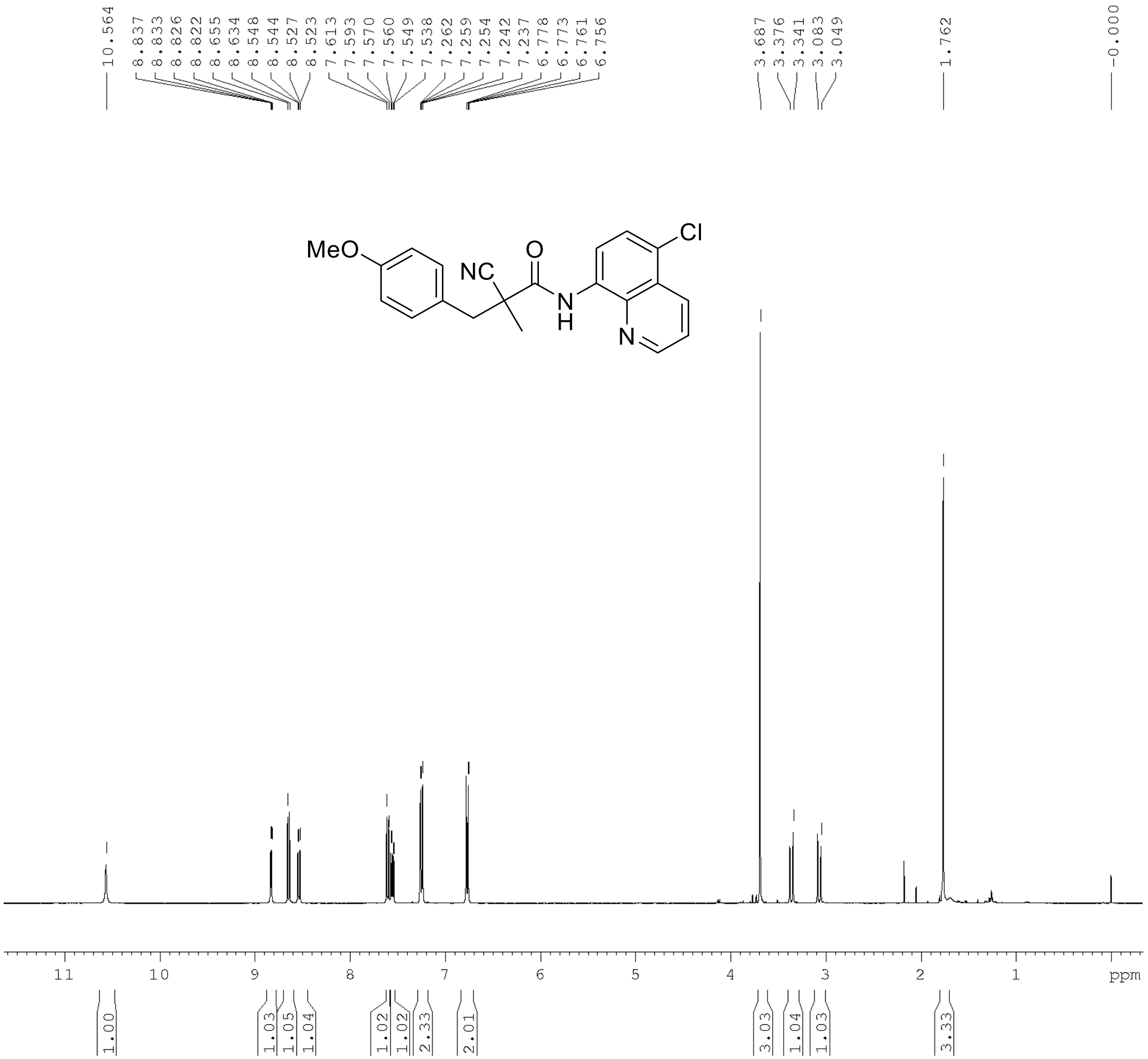

$^{13}C\{^{1}H\}$NMR spectrum of compound **2m** ($CDCl_3$, 100 MHz)**:**

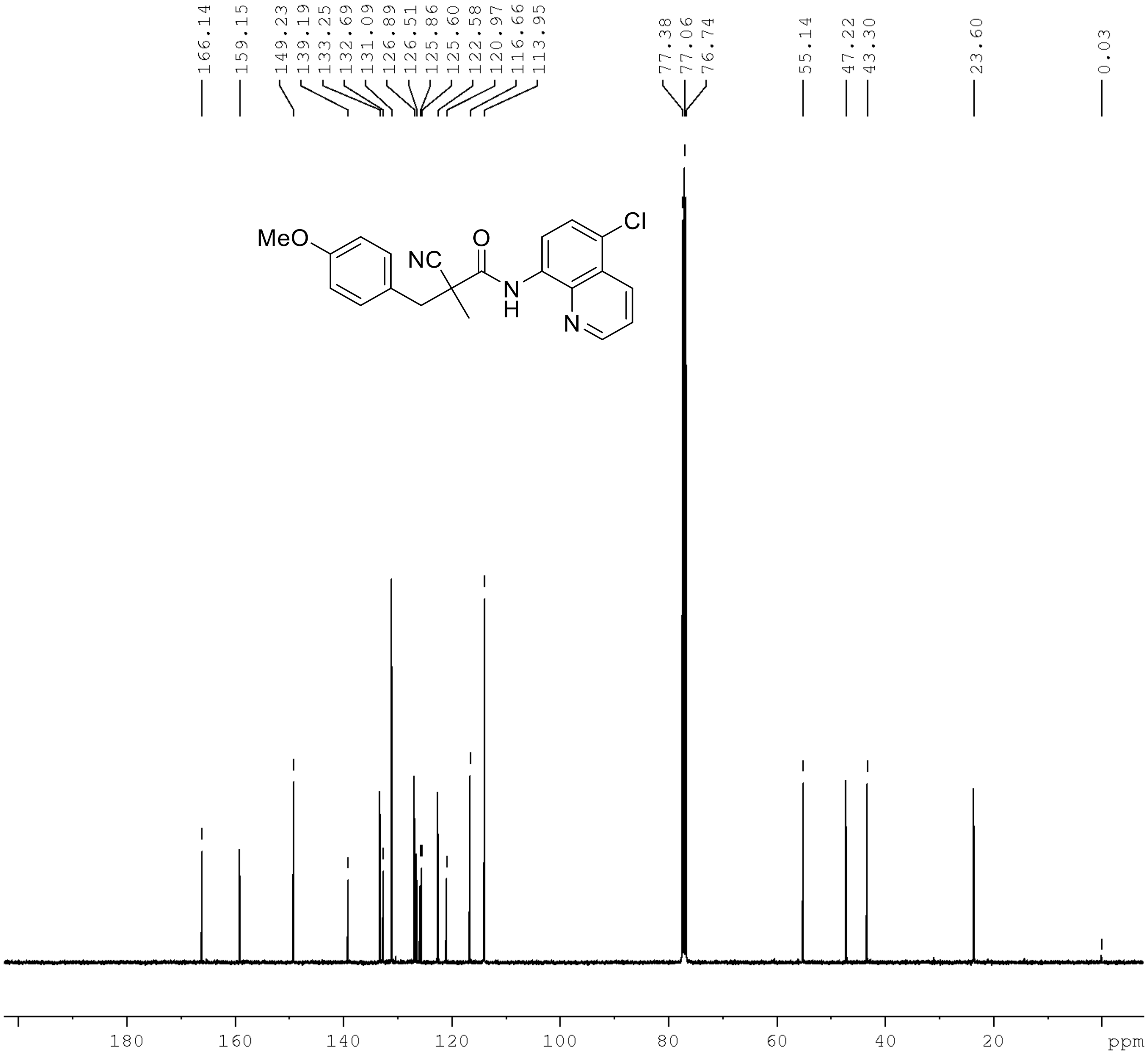

DEPT135 spectrum of compound **2m** ($CDCl_3$, 100 MHz)**:**

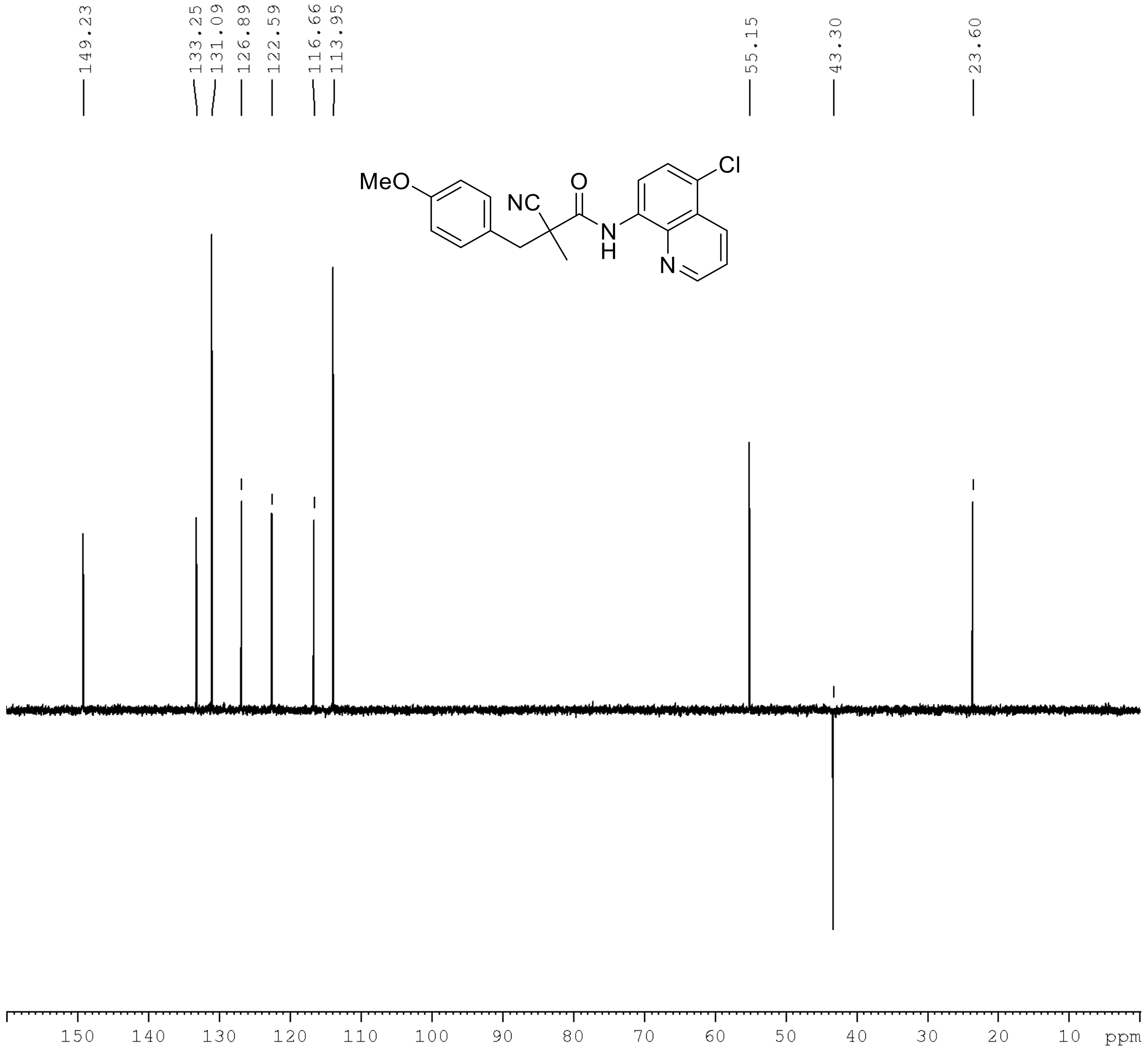

^{1}H NMR spectrum of compound **3m** ($CDCl_3$, 400 MHz)**:**

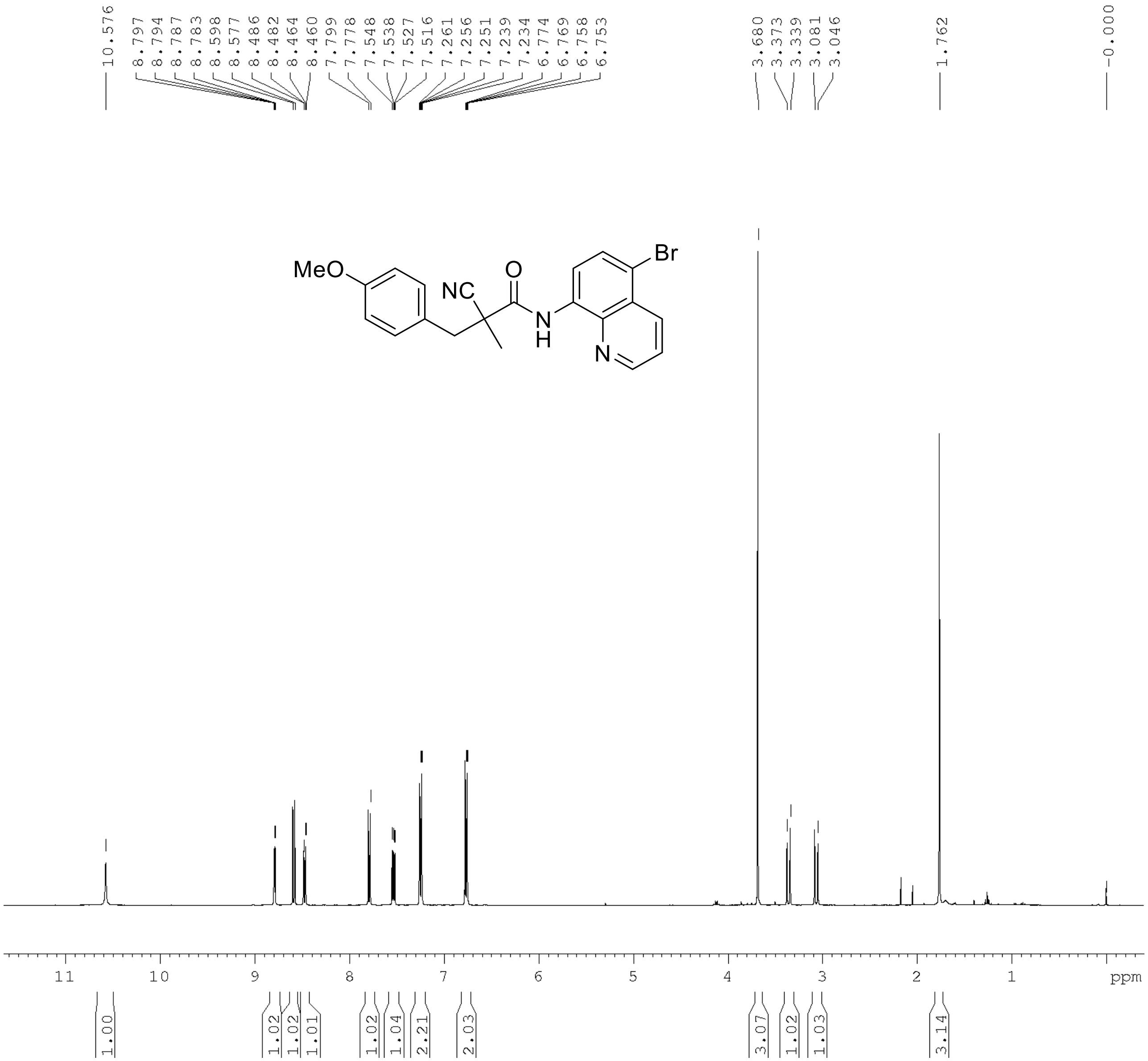

$^{13}C\{^{1}H\}$NMR spectrum of compound **3m** ($CDCl_3$, 100 MHz)**:**

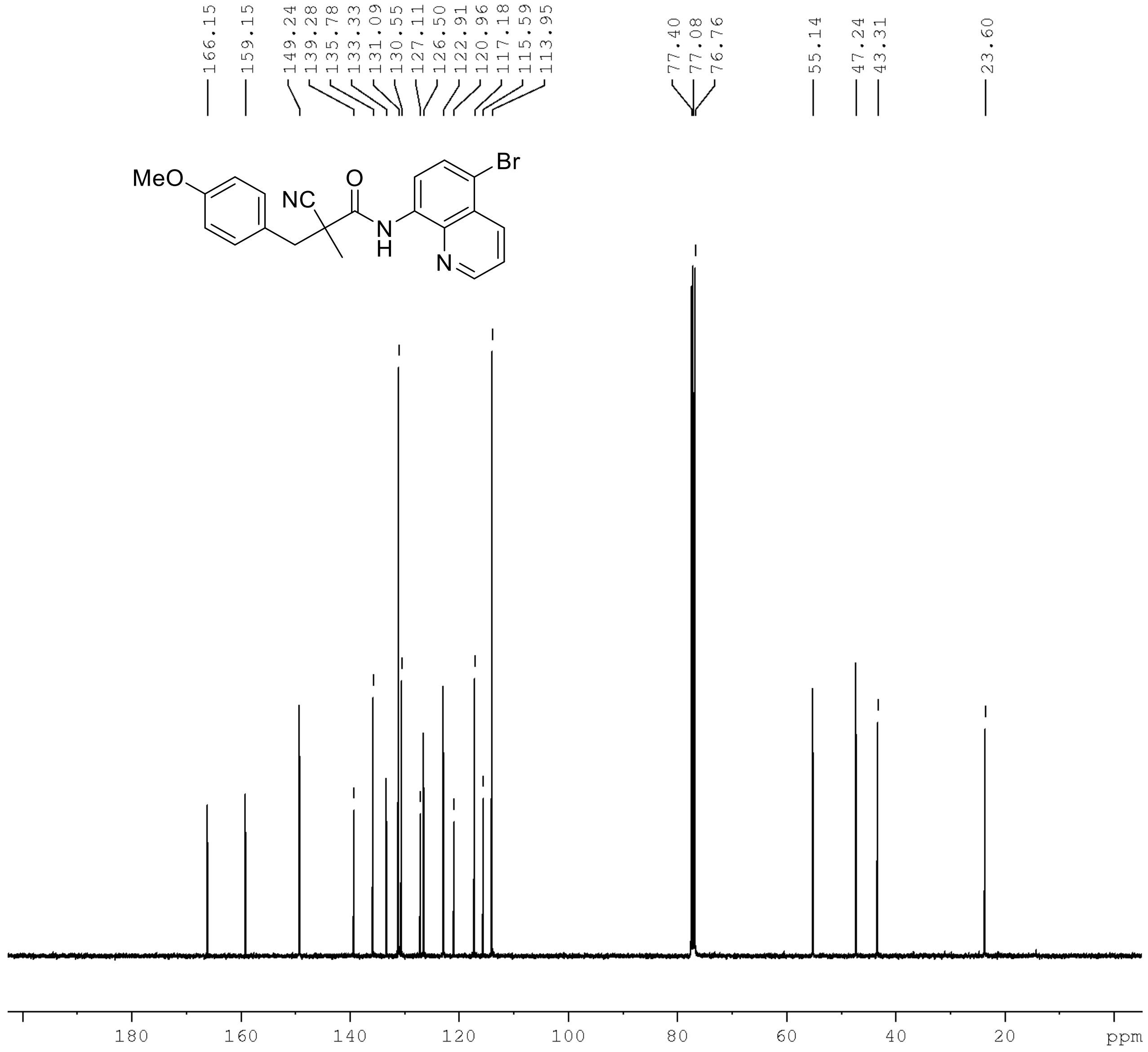

DEPT135 spectrum of compound **3m** ($CDCl_3$, 100 MHz)**:**

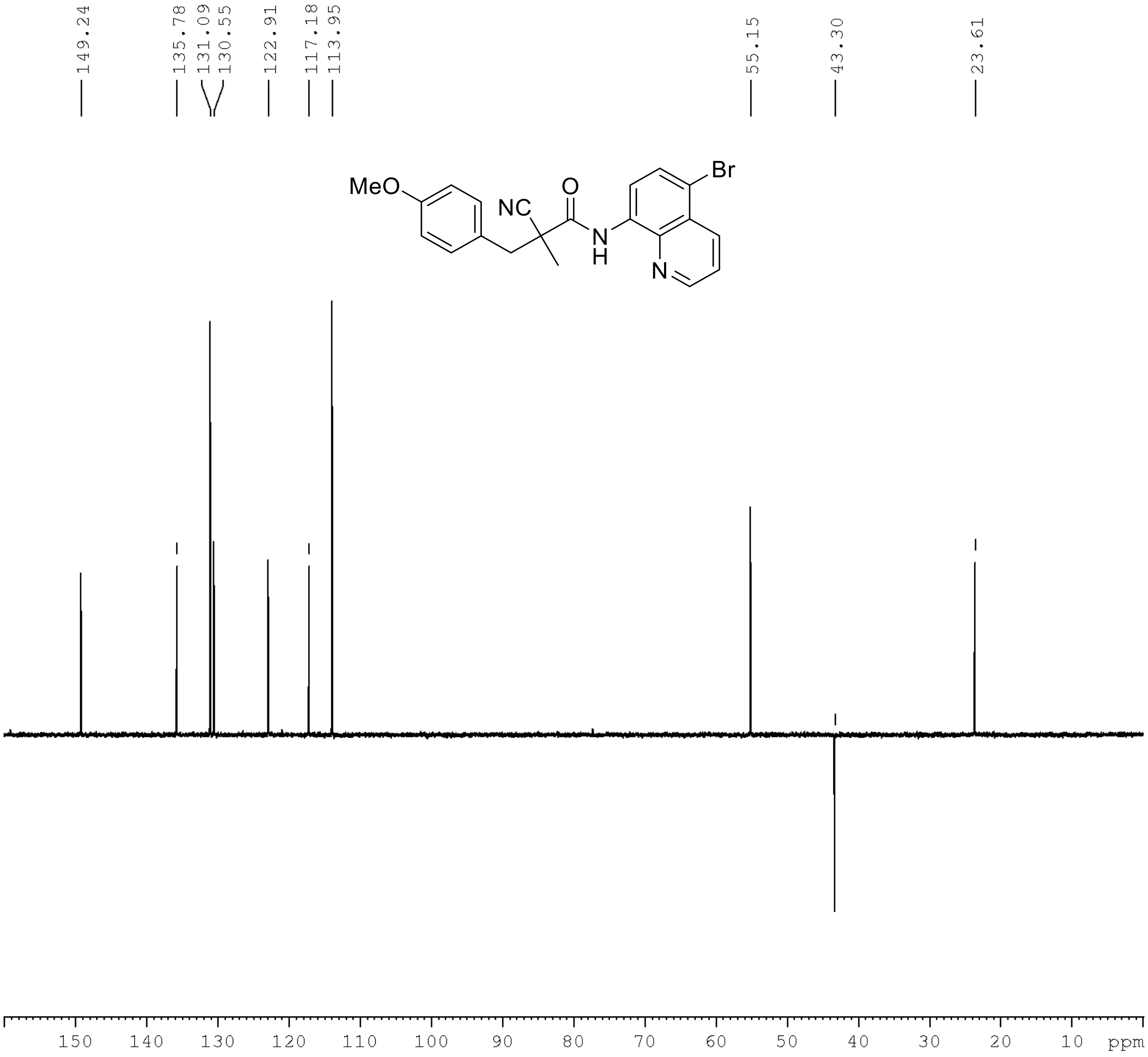

^{1}H NMR spectrum of compound **2n** ($CDCl_3$, 400 MHz)**:**

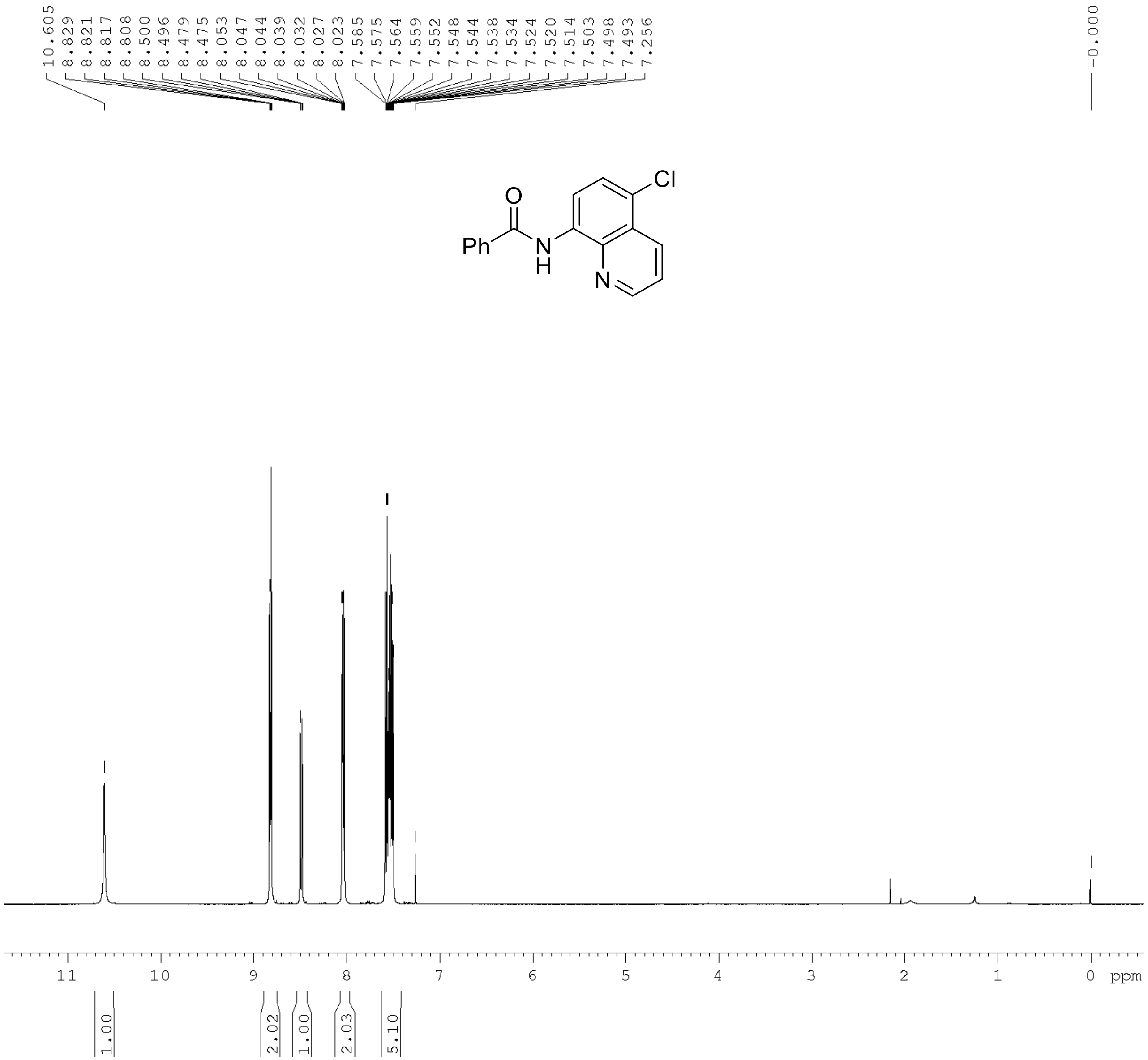

$^{13}C\{^{1}H\}$NMR spectrum of compound **2n** ($CDCl_3$, 100 MHz)**:**

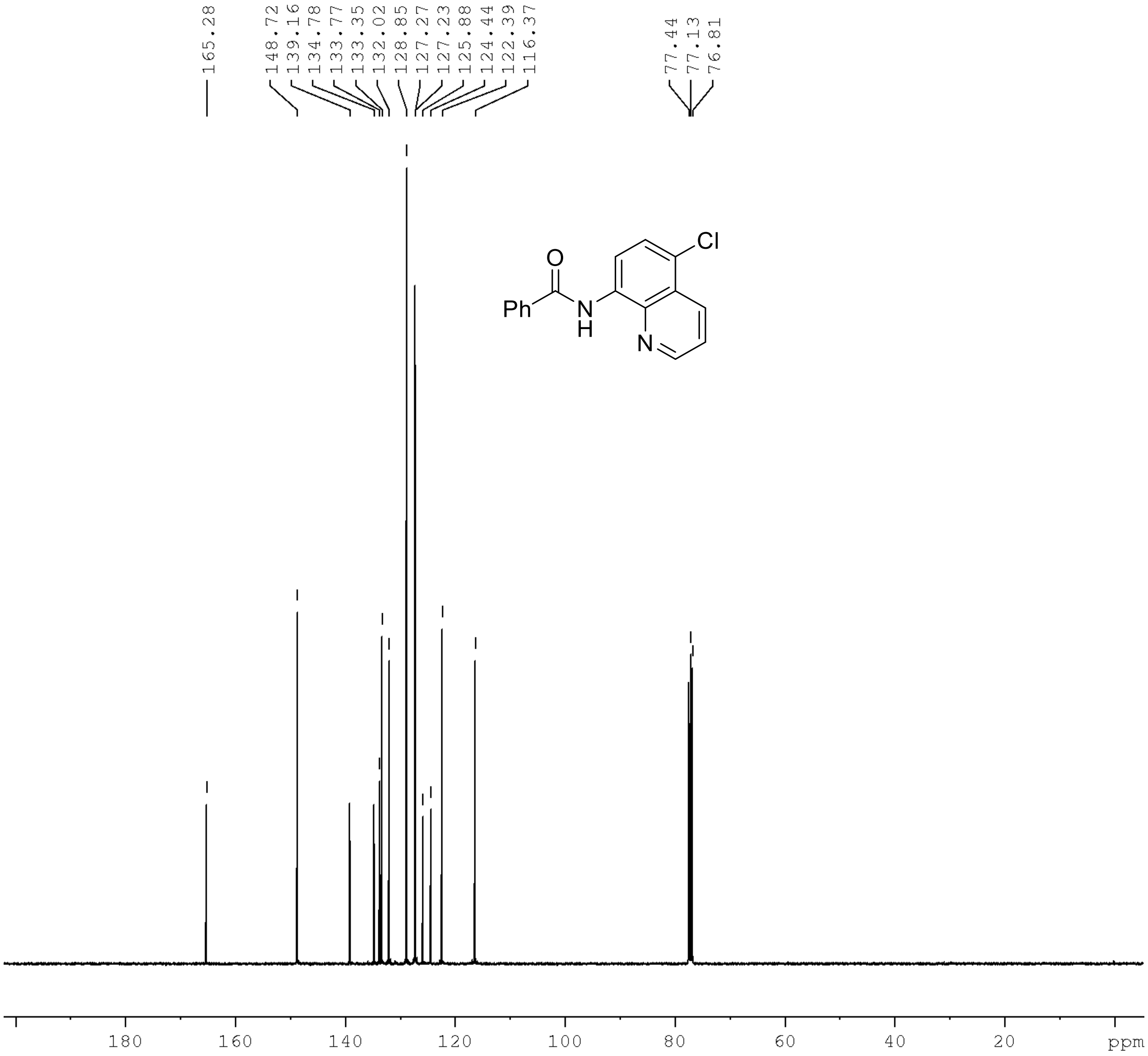

DEPT135 spectrum of compound **2n** ($CDCl_3$, 100 MHz)**:**

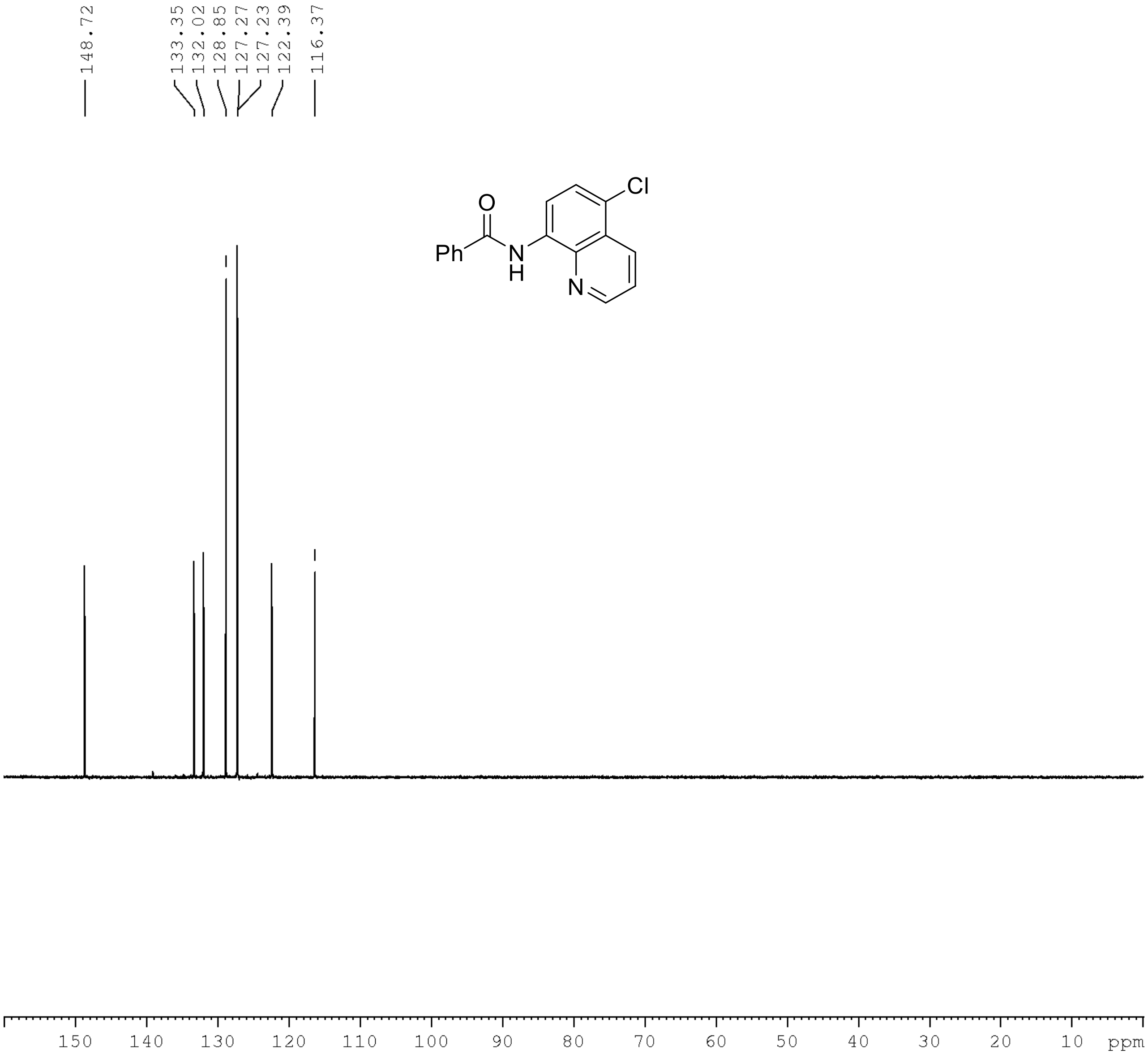

^{1}H NMR spectrum of compound **3n** ($CDCl_3$, 400 MHz)**:**

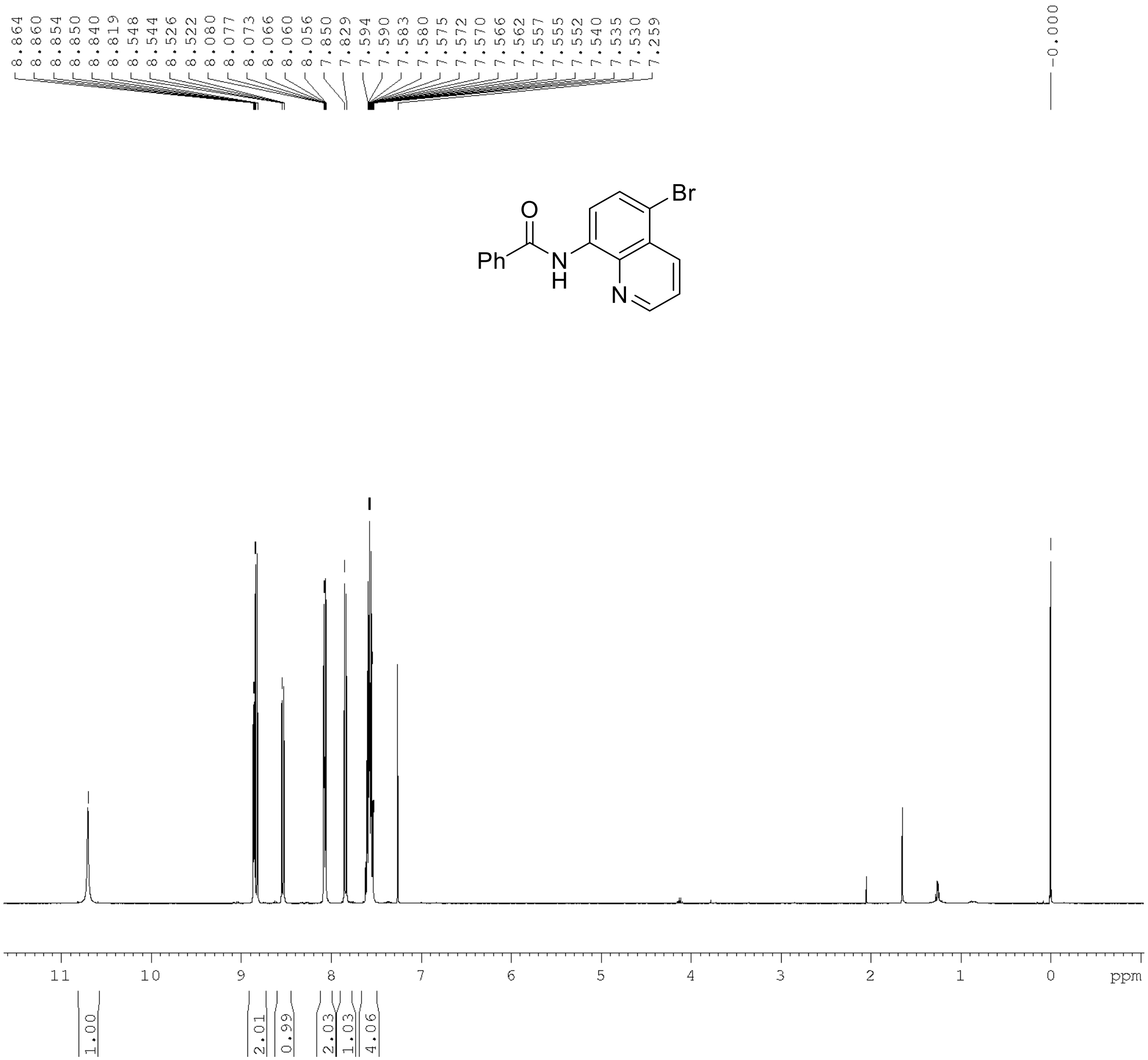

$^{13}C\{^1H\}$NMR spectrum of compound **3n** ($CDCl_3$, 100 MHz)**:**

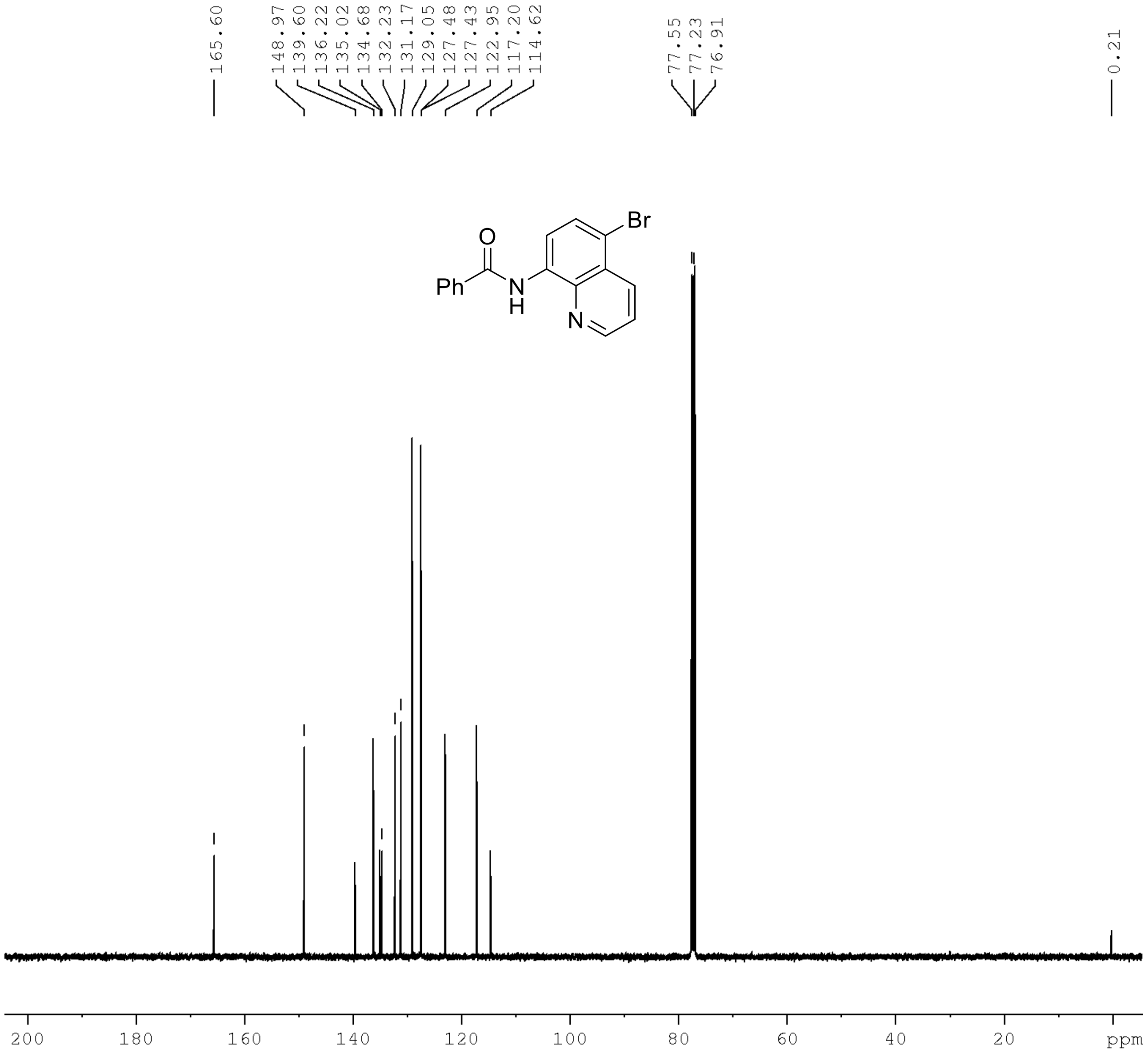

^{1}H NMR spectrum of compound **2o** ($CDCl_3$, 400 MHz)**:**

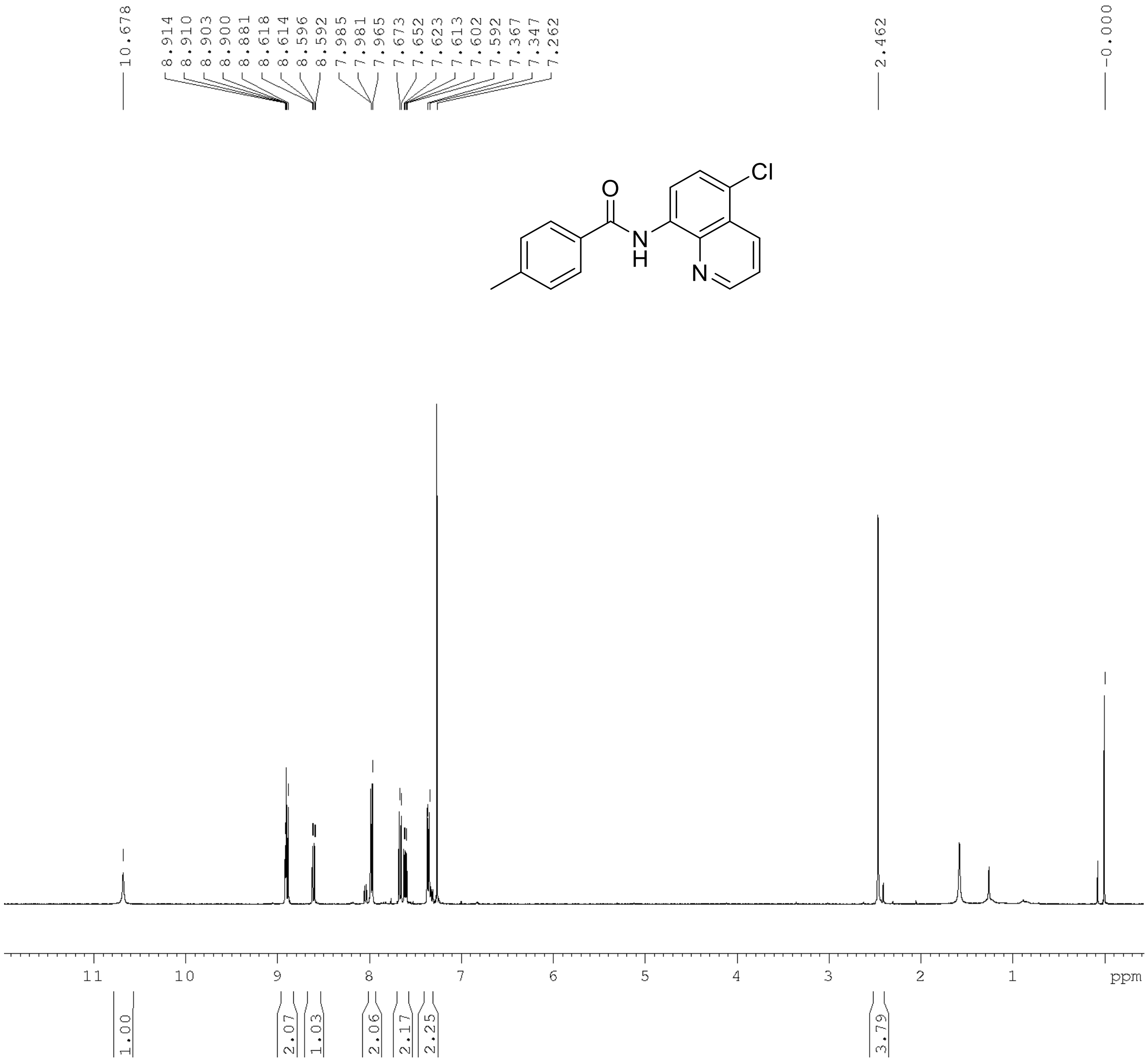

$^{13}C\{^1H\}$NMR spectrum of compound **2o** ($CDCl_3$, 100 MHz)**:**

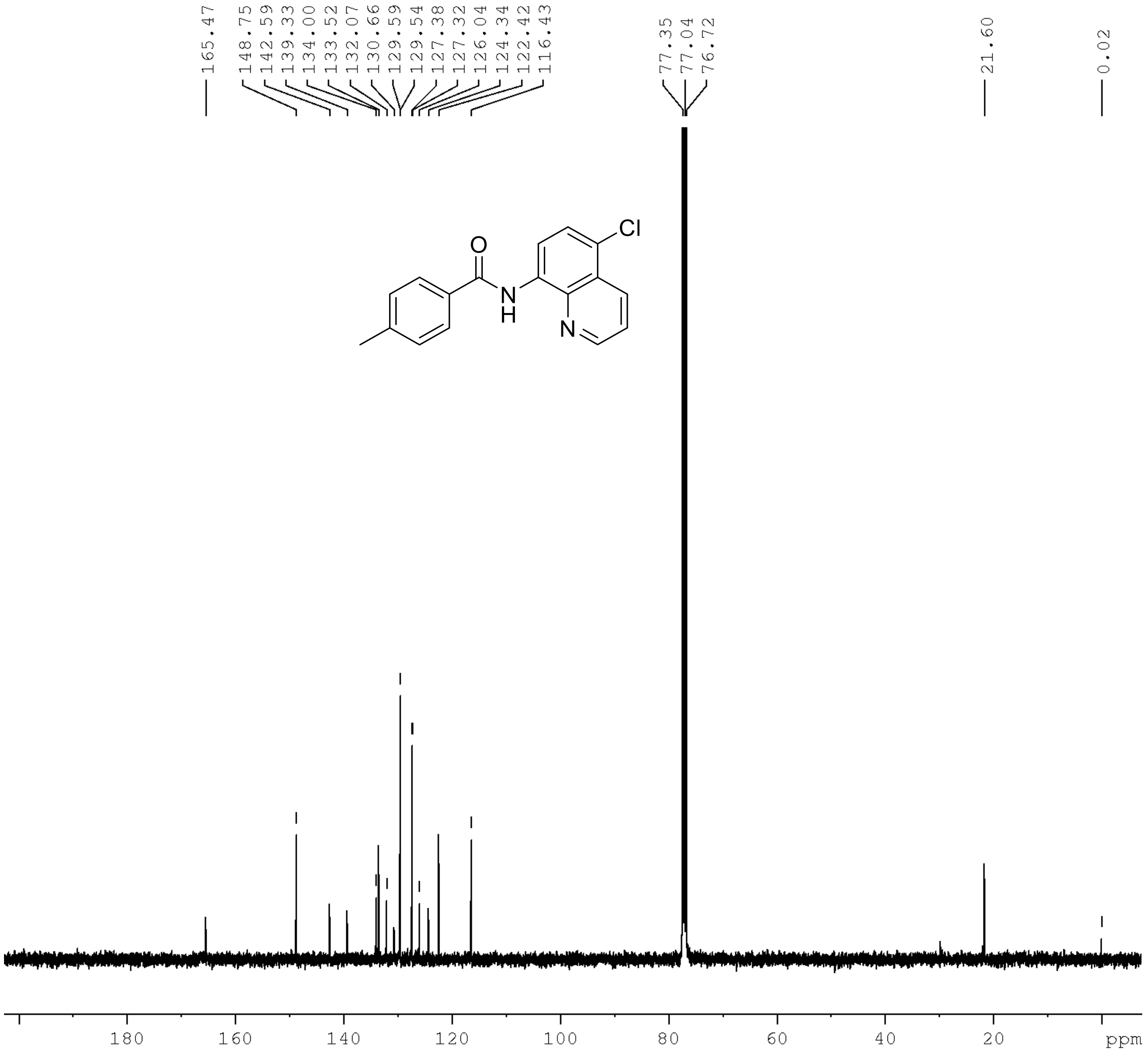

^{1}H NMR spectrum of compound **3o** ($CDCl_3$, 400 MHz)**:**

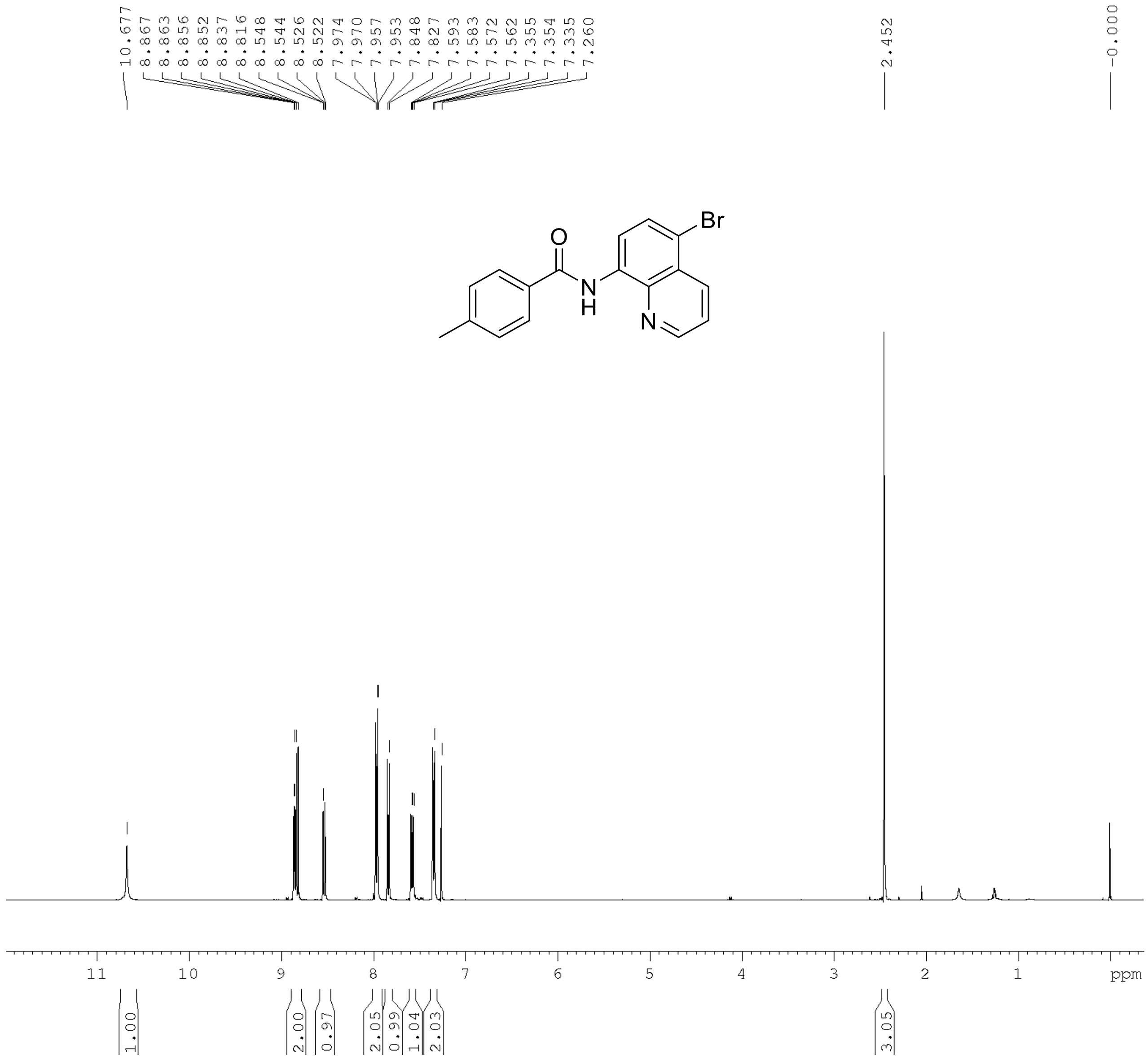

$^{13}C\{^1H\}$NMR spectrum of compound **3o** ($CDCl_3$, 100 MHz)**:**

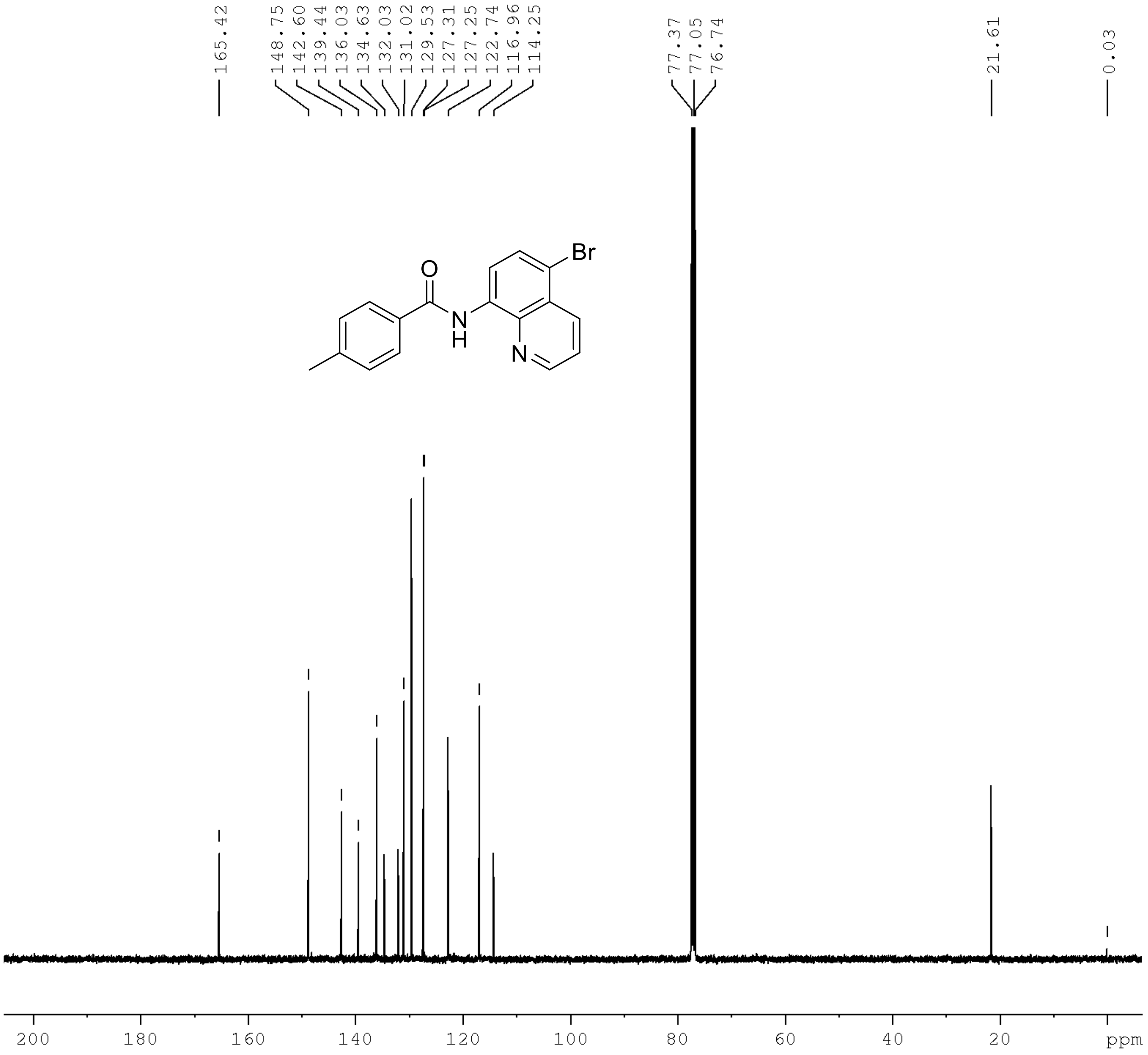

^{1}H NMR spectrum of compound **2p** ($CDCl_3$, 400 MHz)**:**

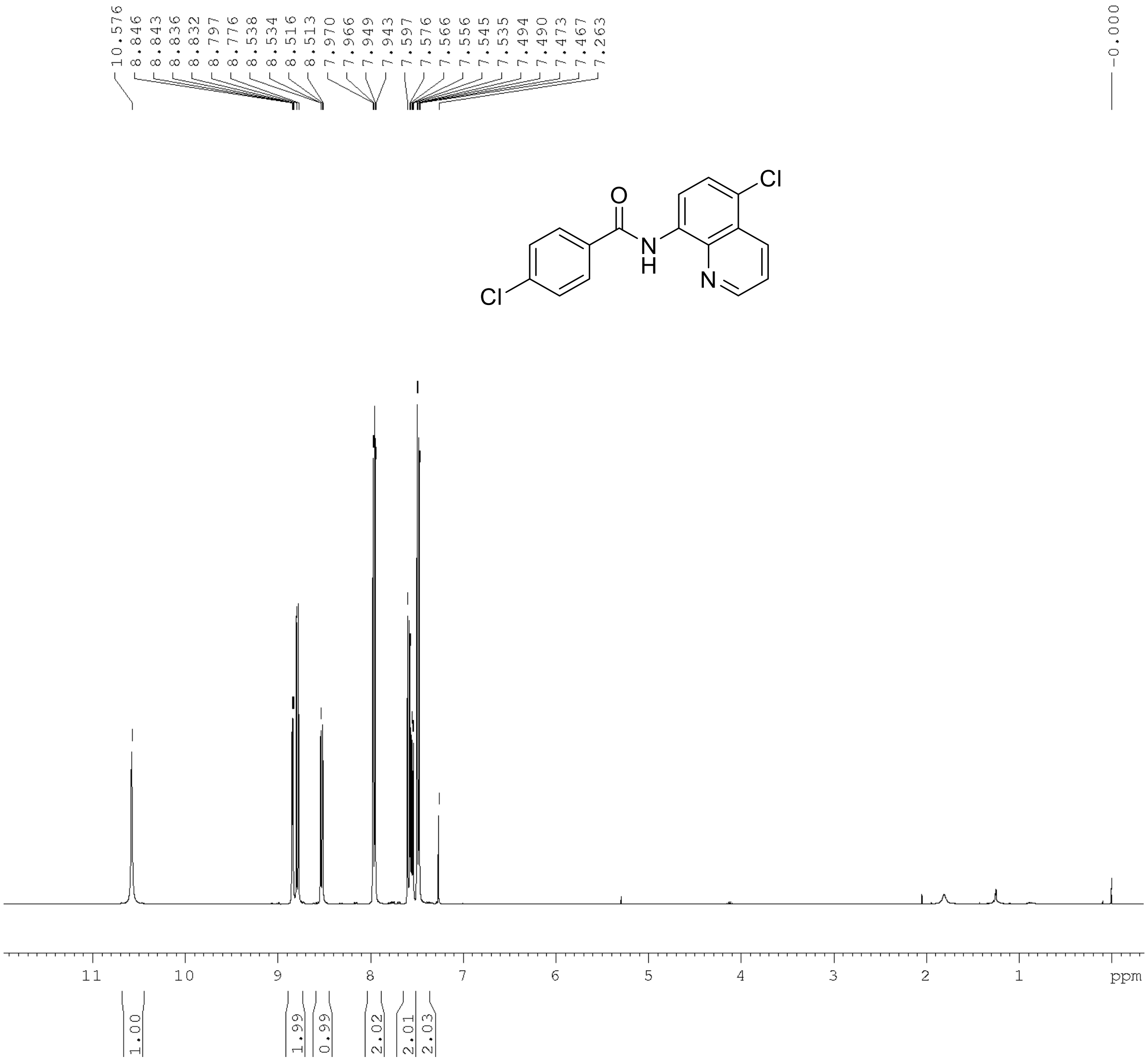

$^{13}C\{^1H\}$NMR spectrum of compound **2p** ($CDCl_3$, 100 MHz):

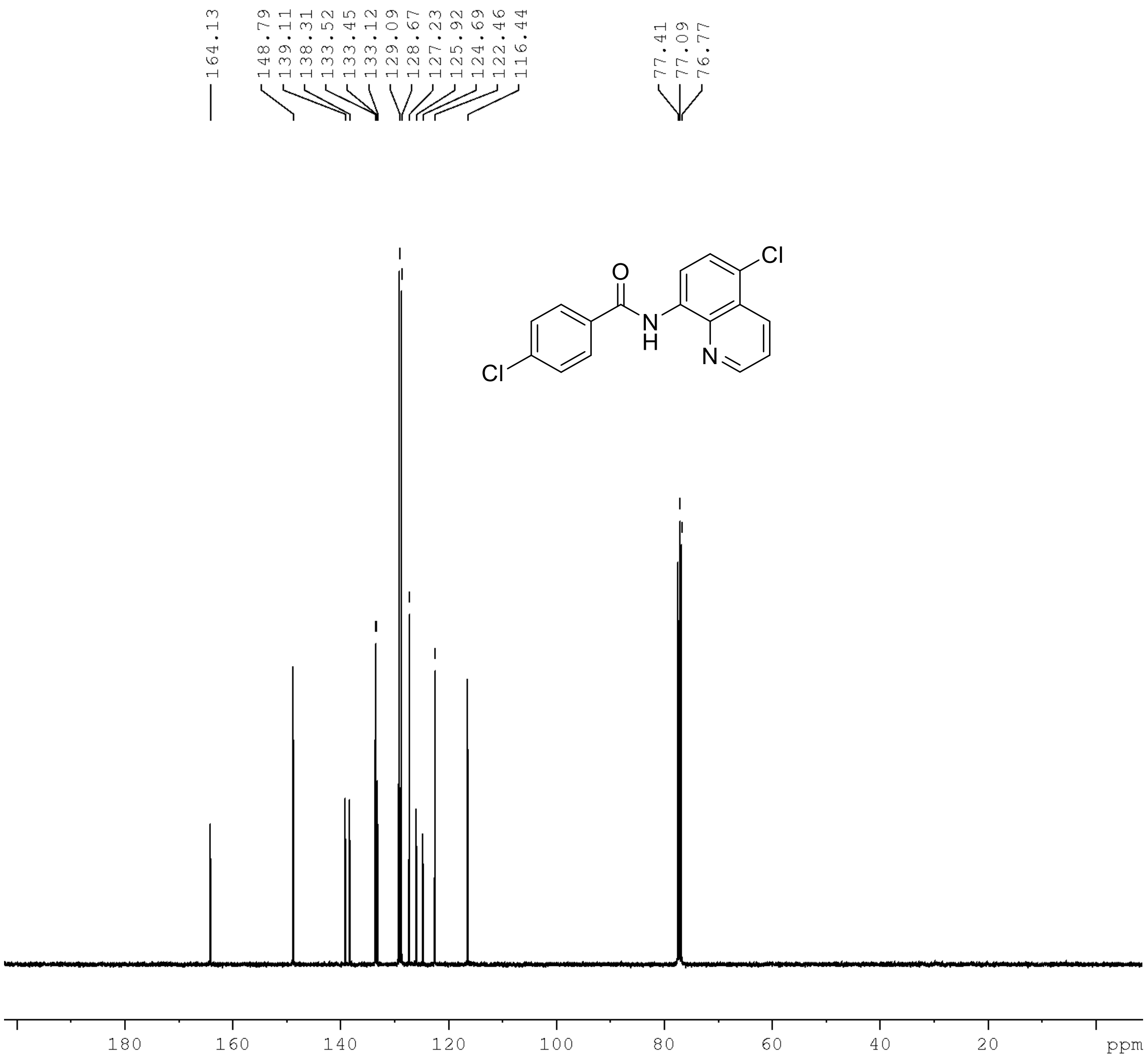

^{1}H NMR spectrum of compound **3p** ($CDCl_3$, 400 MHz)**:**

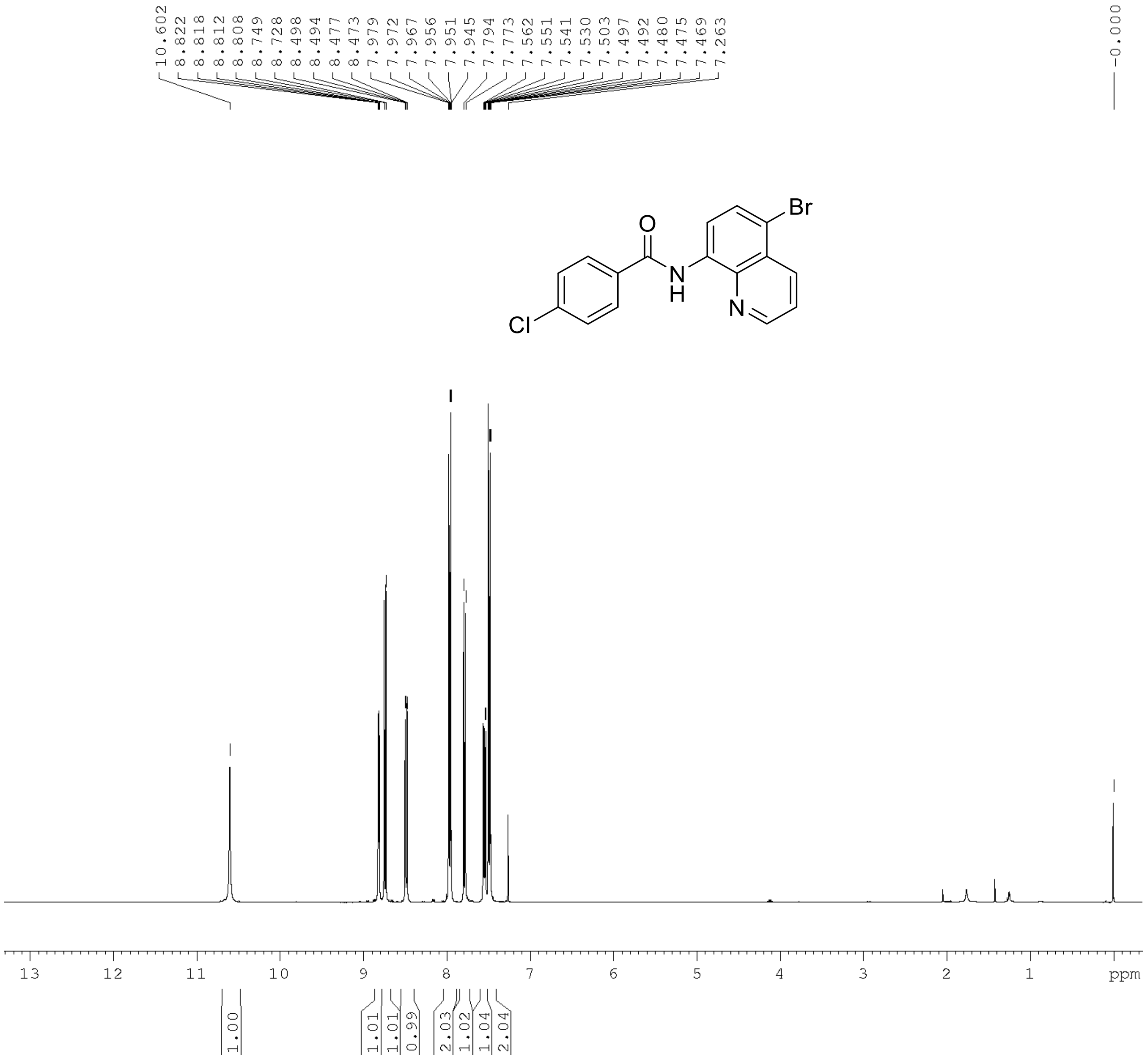

$^{13}C\{^1H\}$NMR spectrum of compound **3p** ($CDCl_3$, 100 MHz)**:**

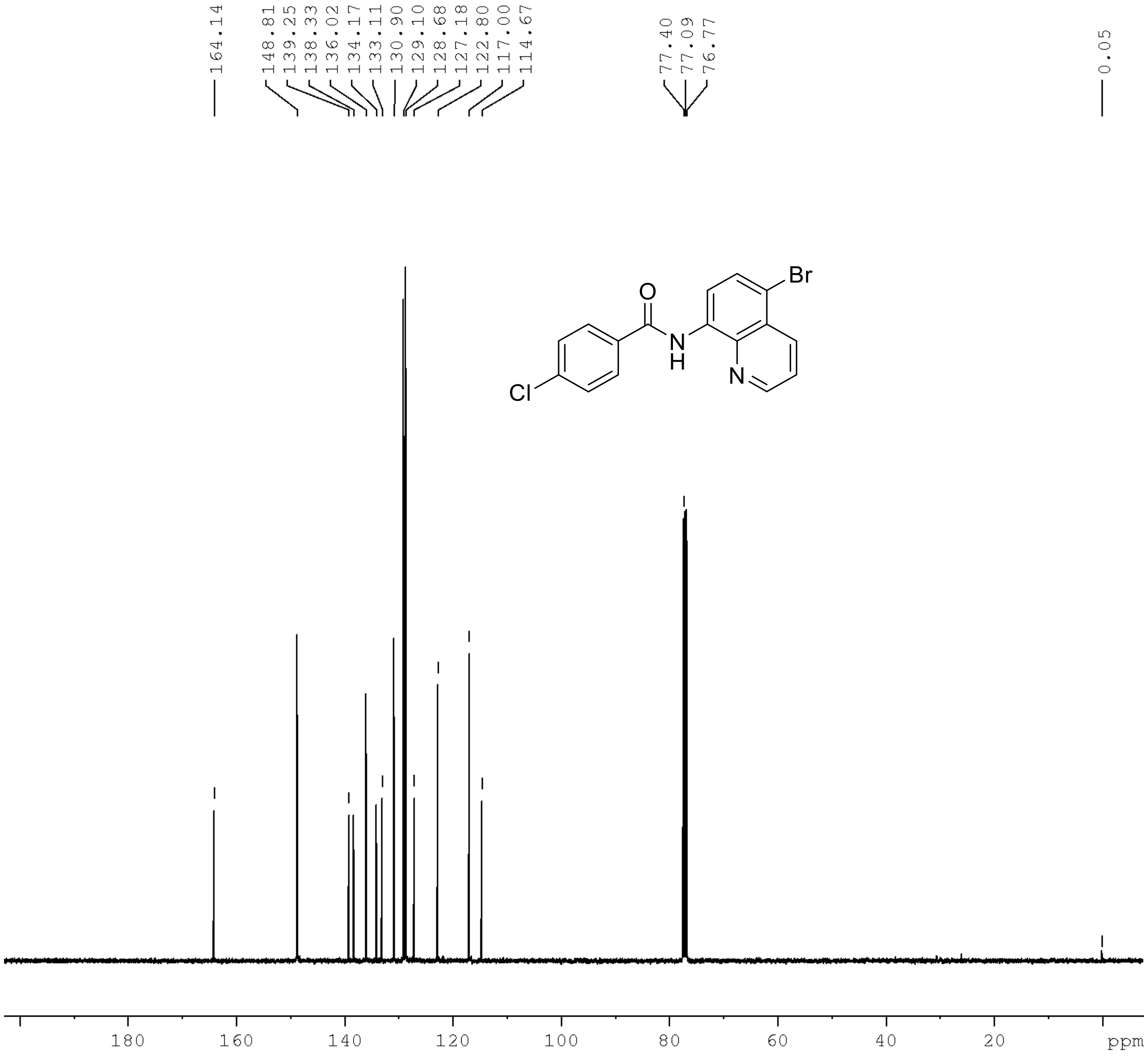

^{1}H NMR spectrum of compound **2q** ($CDCl_3$, 400 MHz)**:**

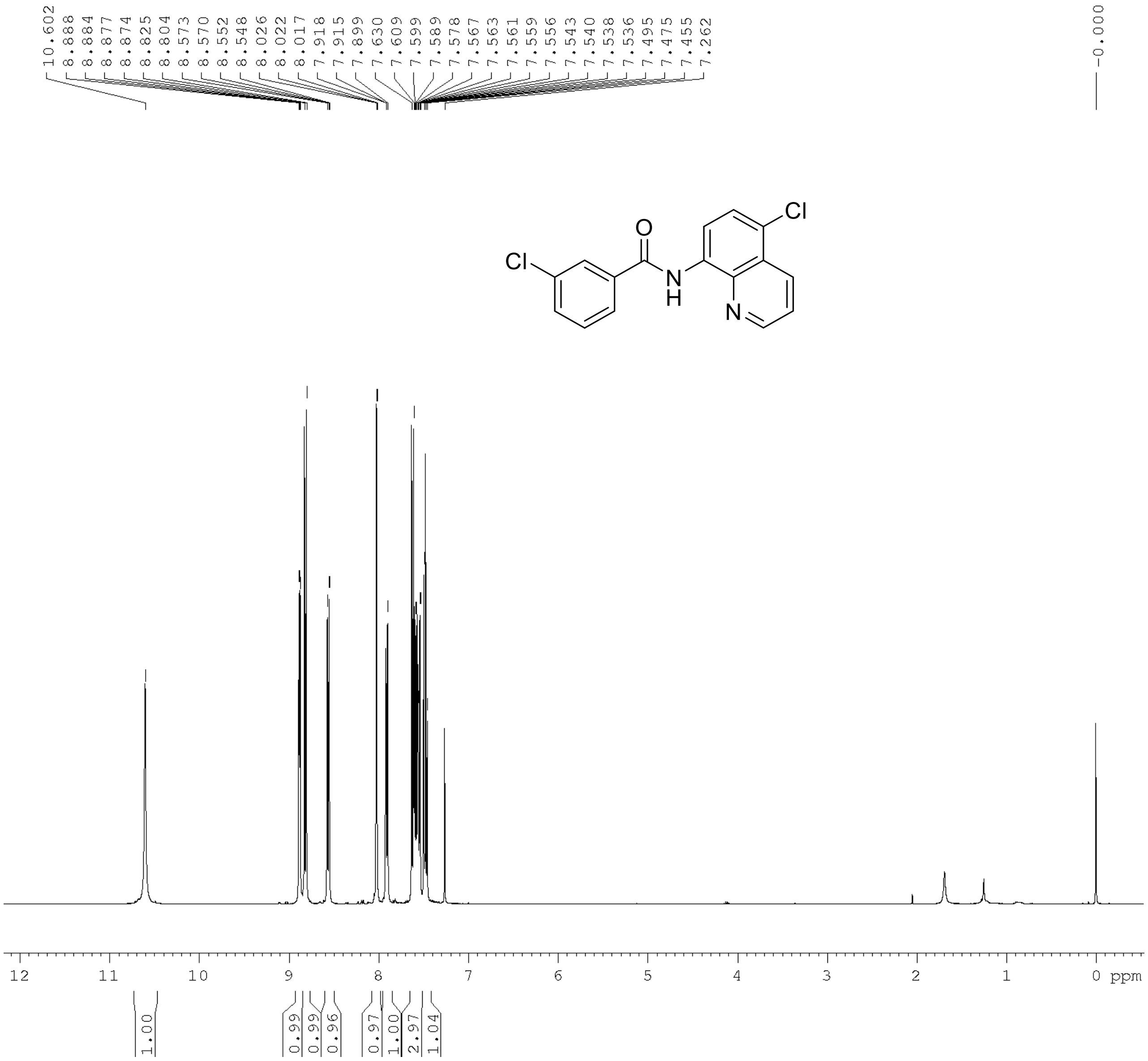

$^{13}C\{^{1}H\}$NMR spectrum of compound **2q** ($CDCl_3$, 100 MHz)**:**

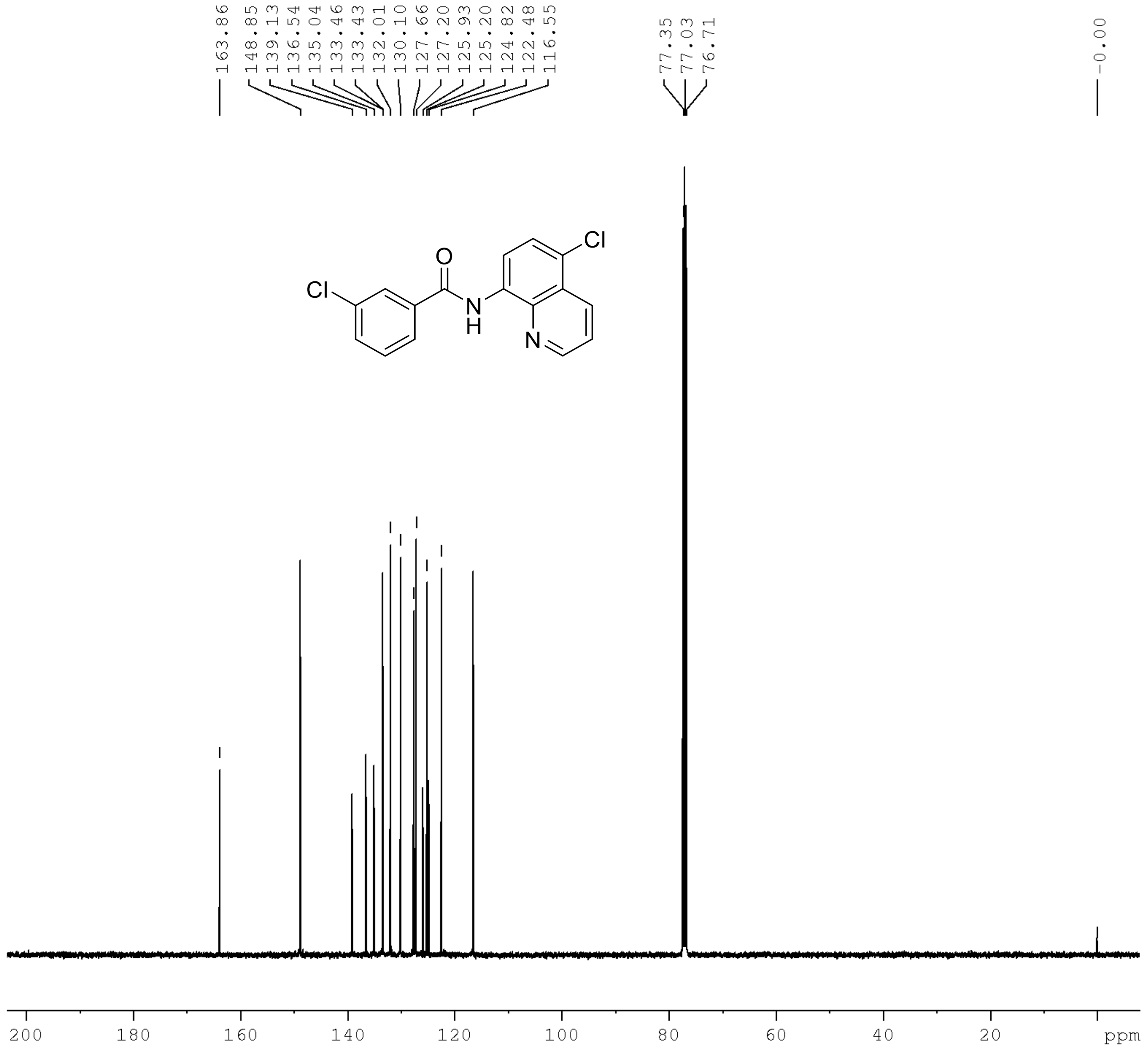

^{1}H NMR spectrum of compound **3q** ($CDCl_3$, 400 MHz)**:**

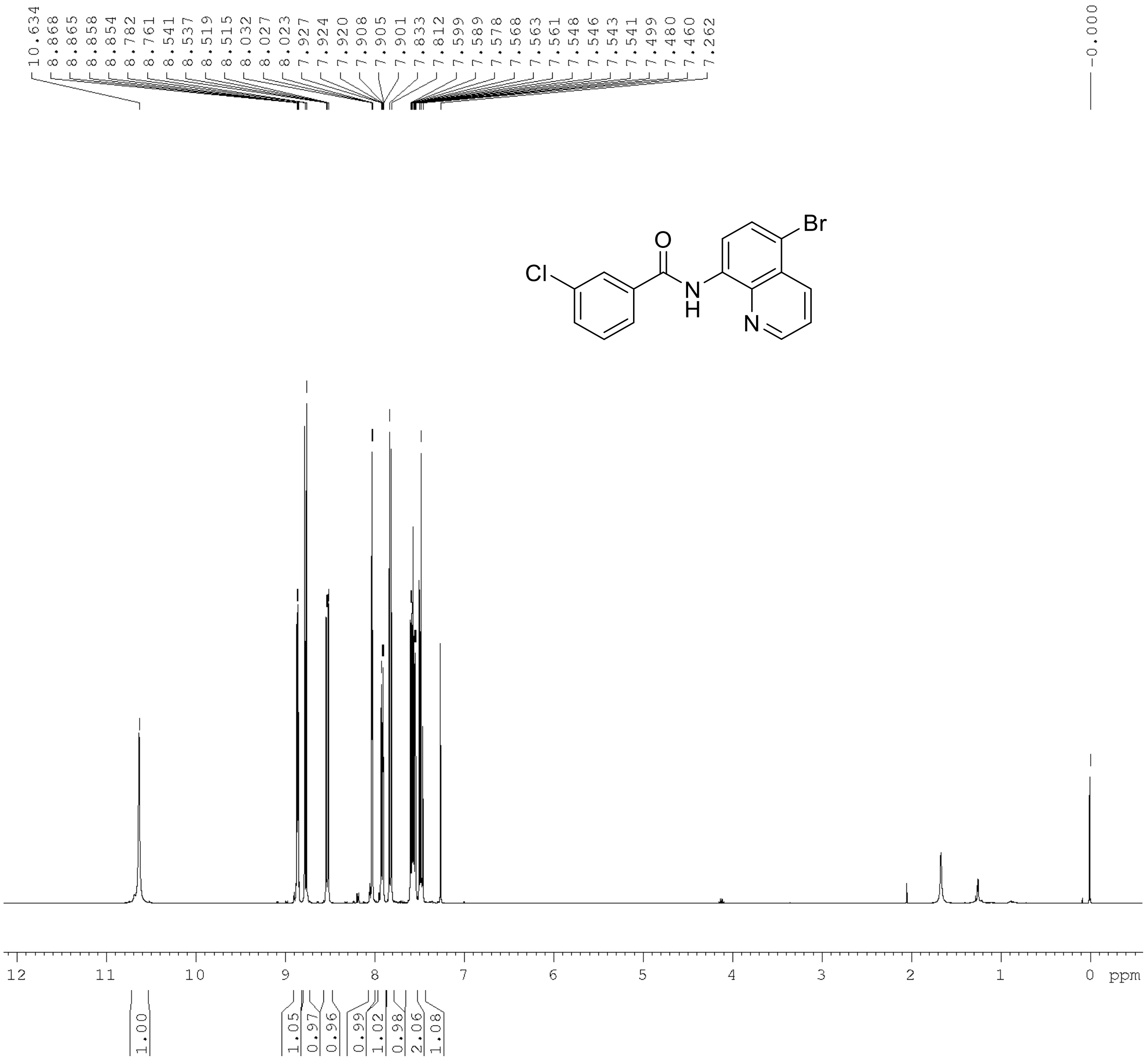

$^{13}C\{^{1}H\}$NMR spectrum of compound **3q** ($CDCl_3$, 100 MHz):

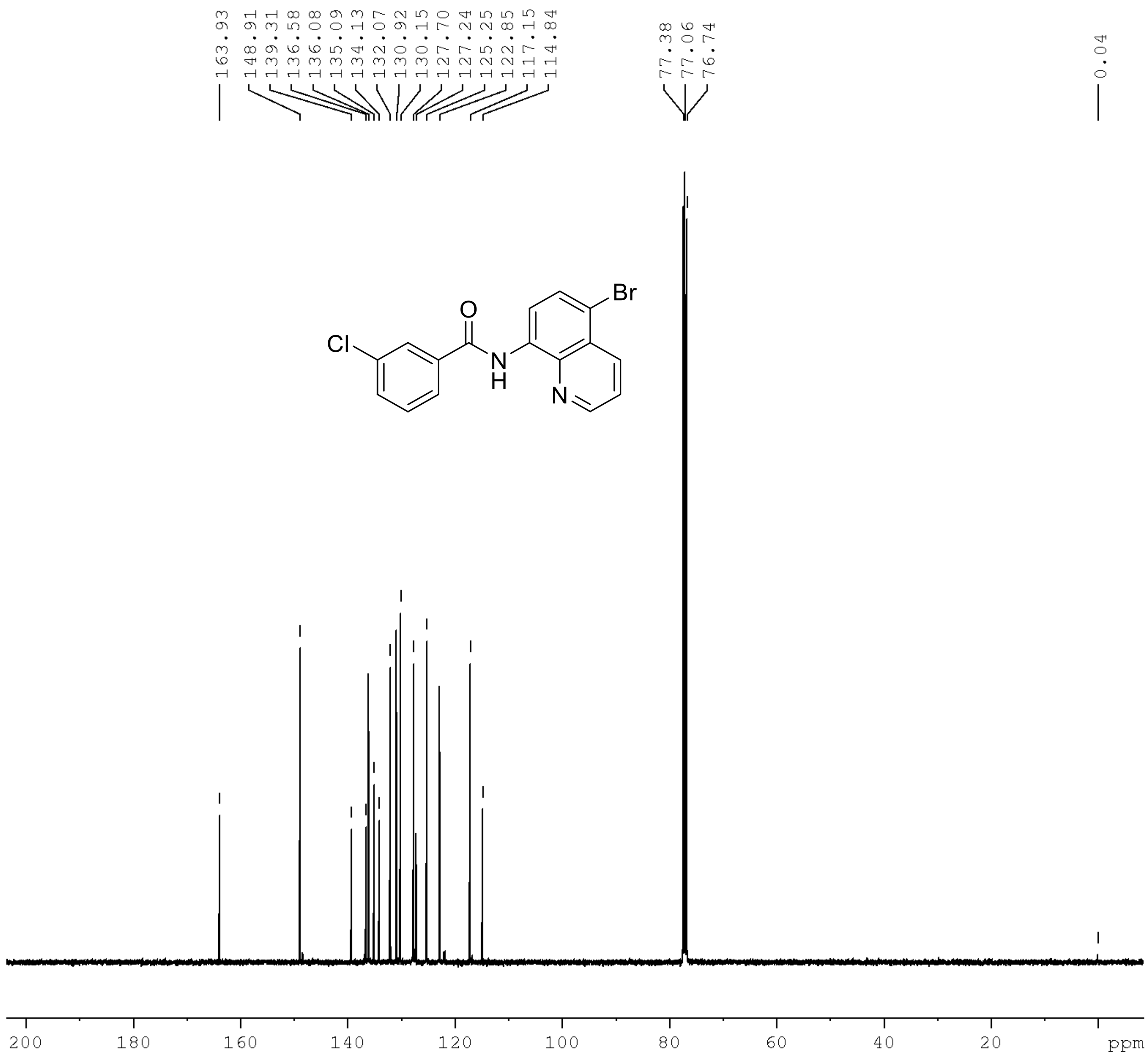

^{1}H NMR spectrum of compound **2r** ($CDCl_3$, 400 MHz)**:**

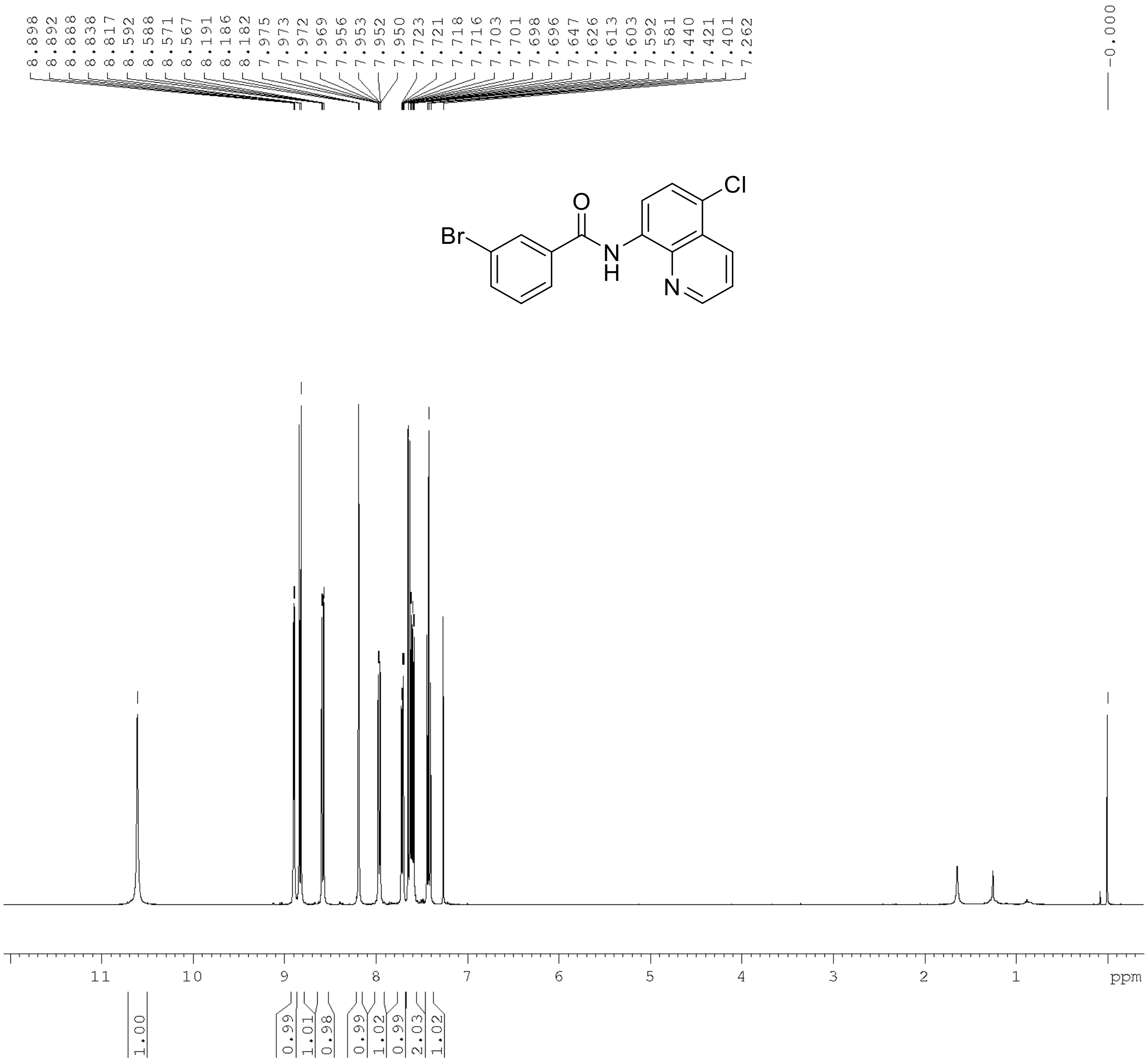

$^{13}C\{^{1}H\}$NMR spectrum of compound **2r** ($CDCl_3$, 100 MHz)**:**

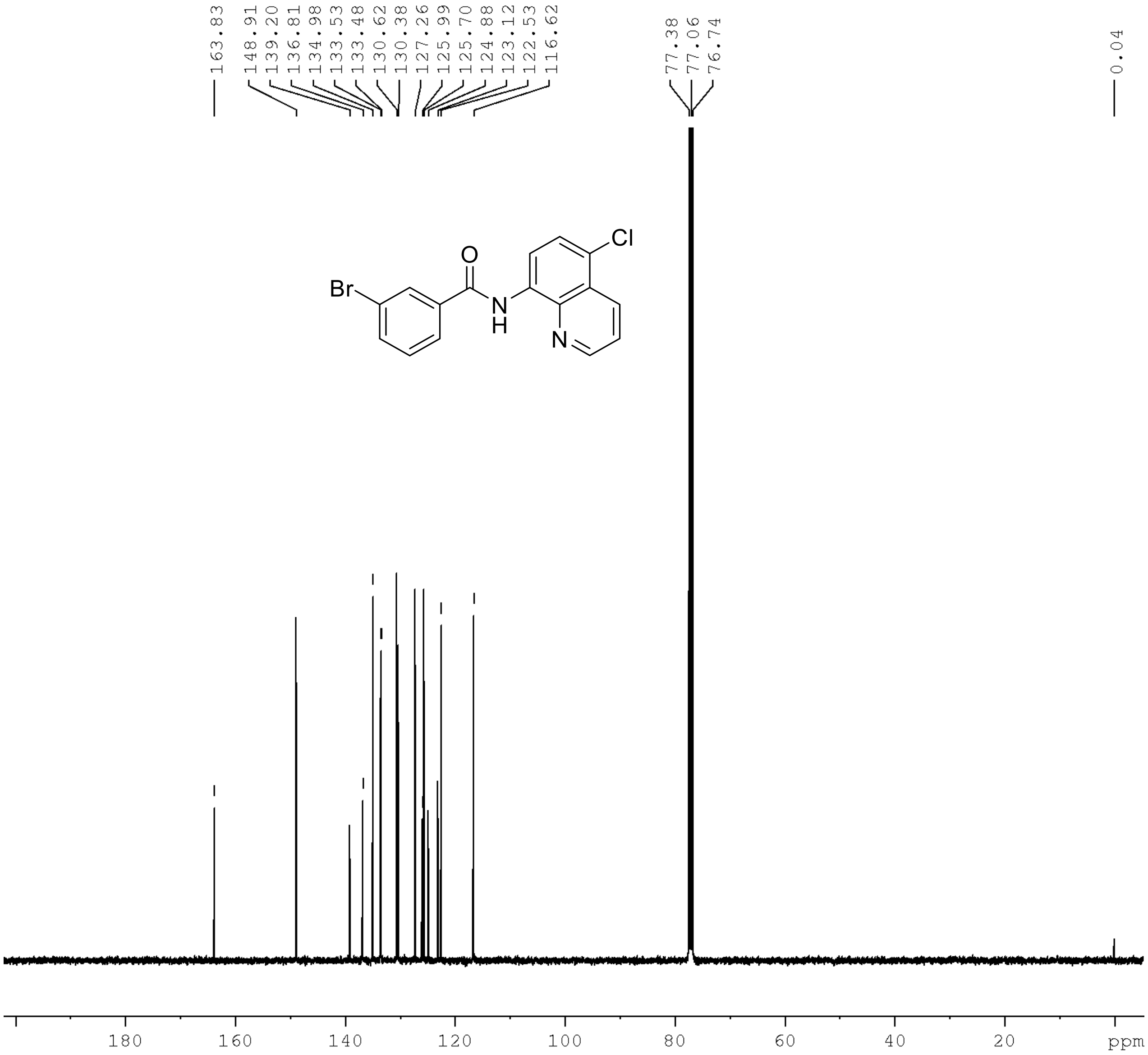

^{1}H NMR spectrum of compound **3r** ($CDCl_3$, 400 MHz)**:**

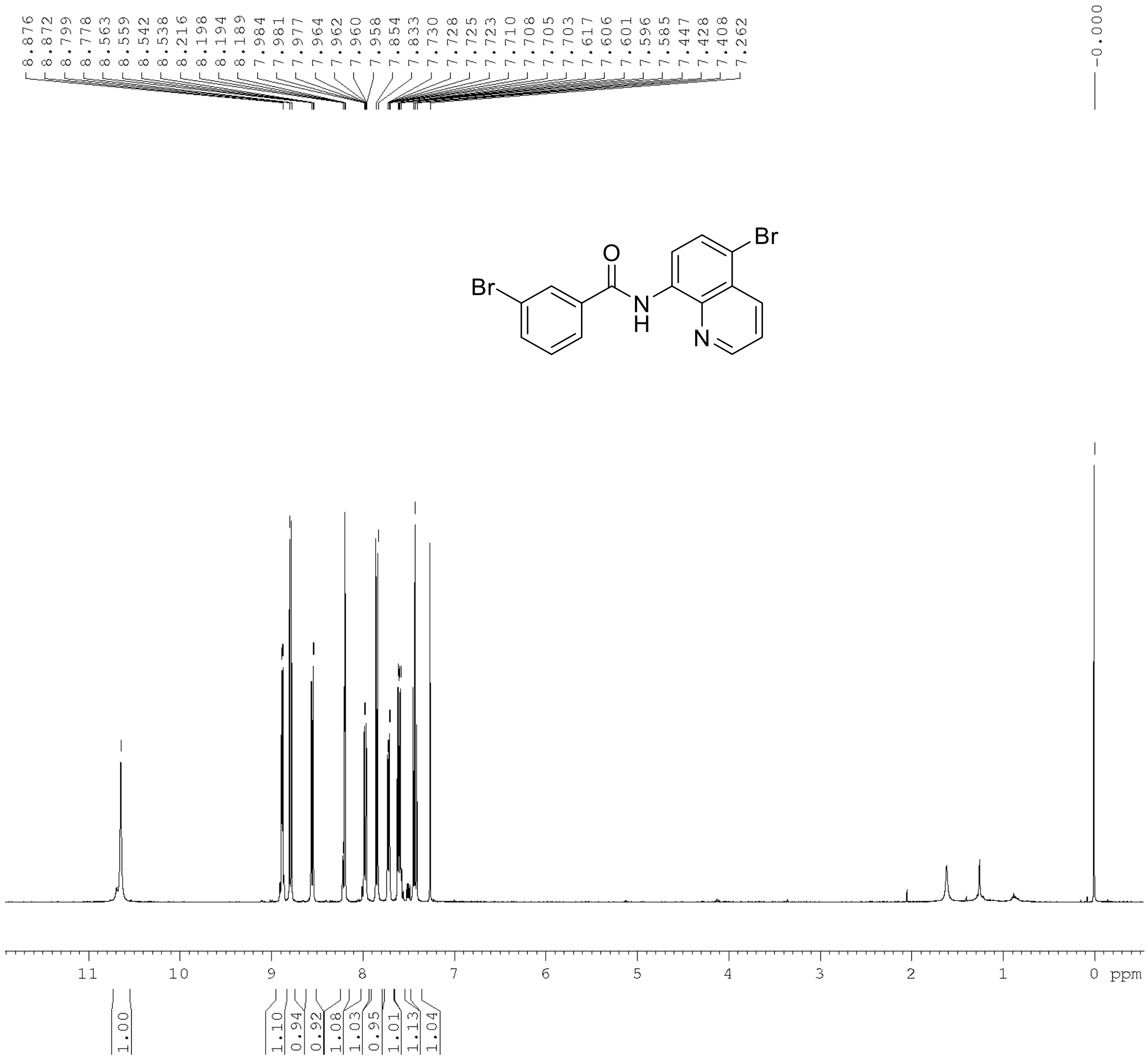

$^{13}C\{^1H\}$NMR spectrum of compound **3r** ($CDCl_3$, 100 MHz)**:**

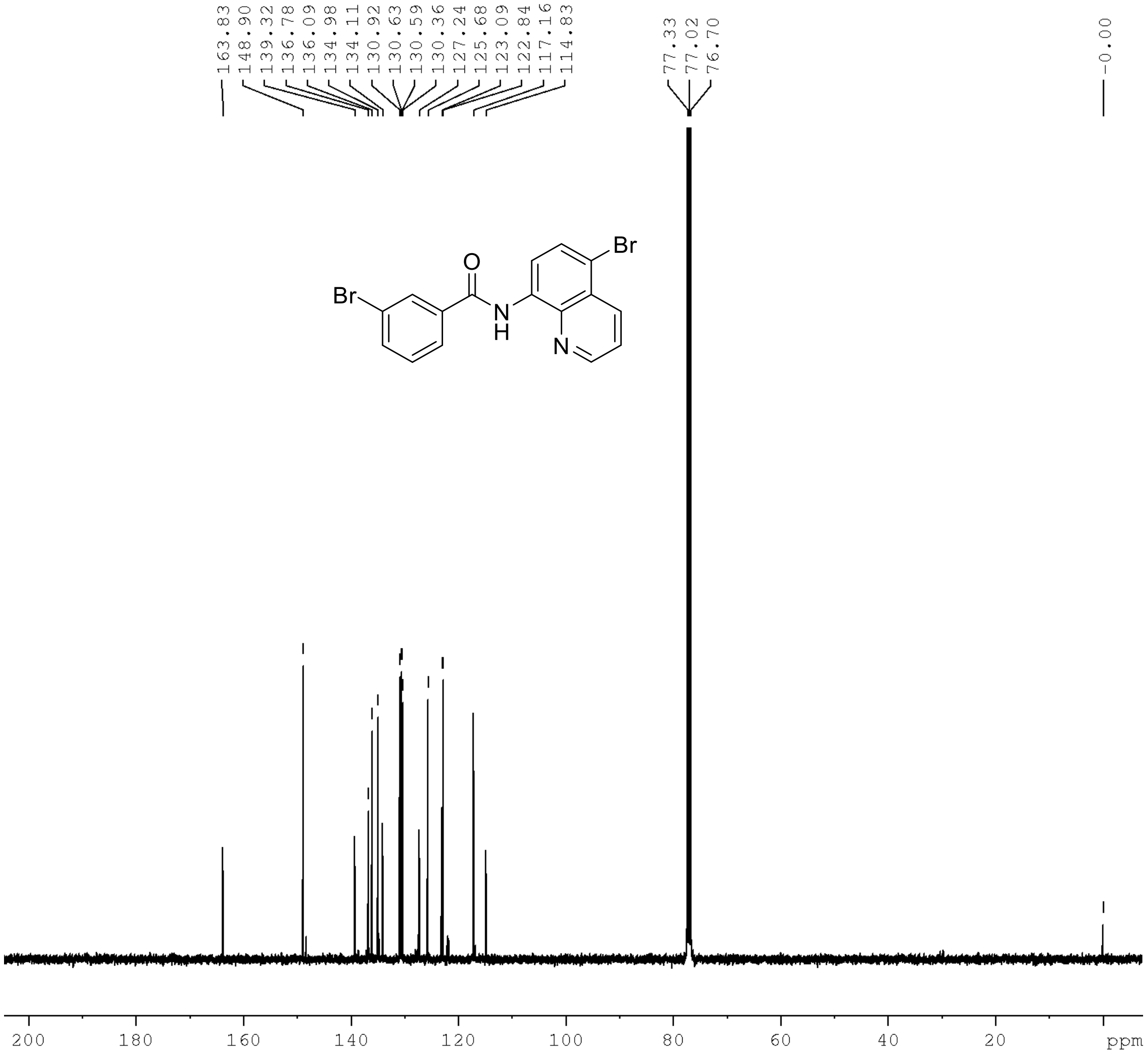

HRMS spectrum of compound **3r:**

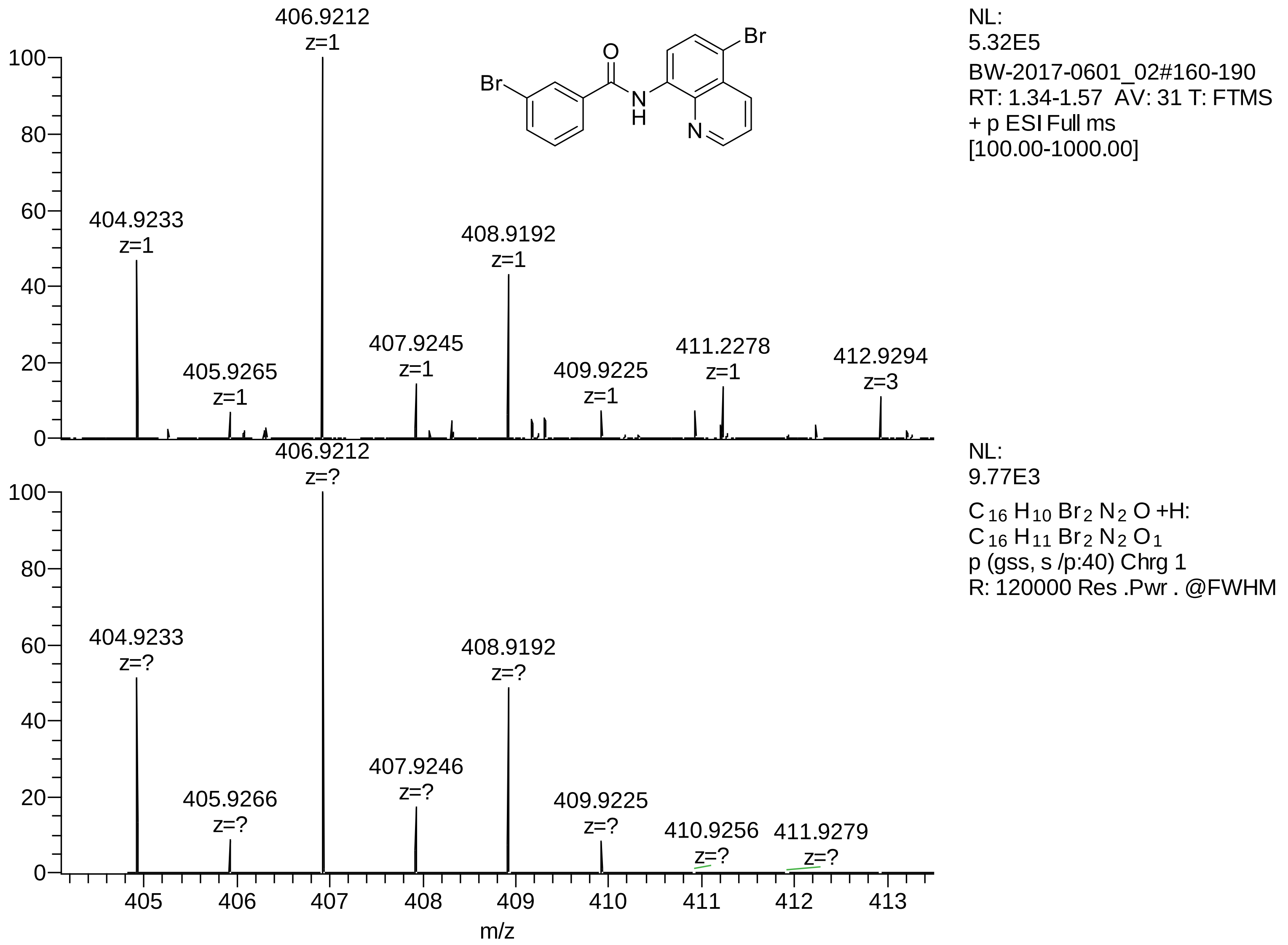

^{1}H NMR spectrum of compound **2s** ($CDCl_3$, 400 MHz)**:**

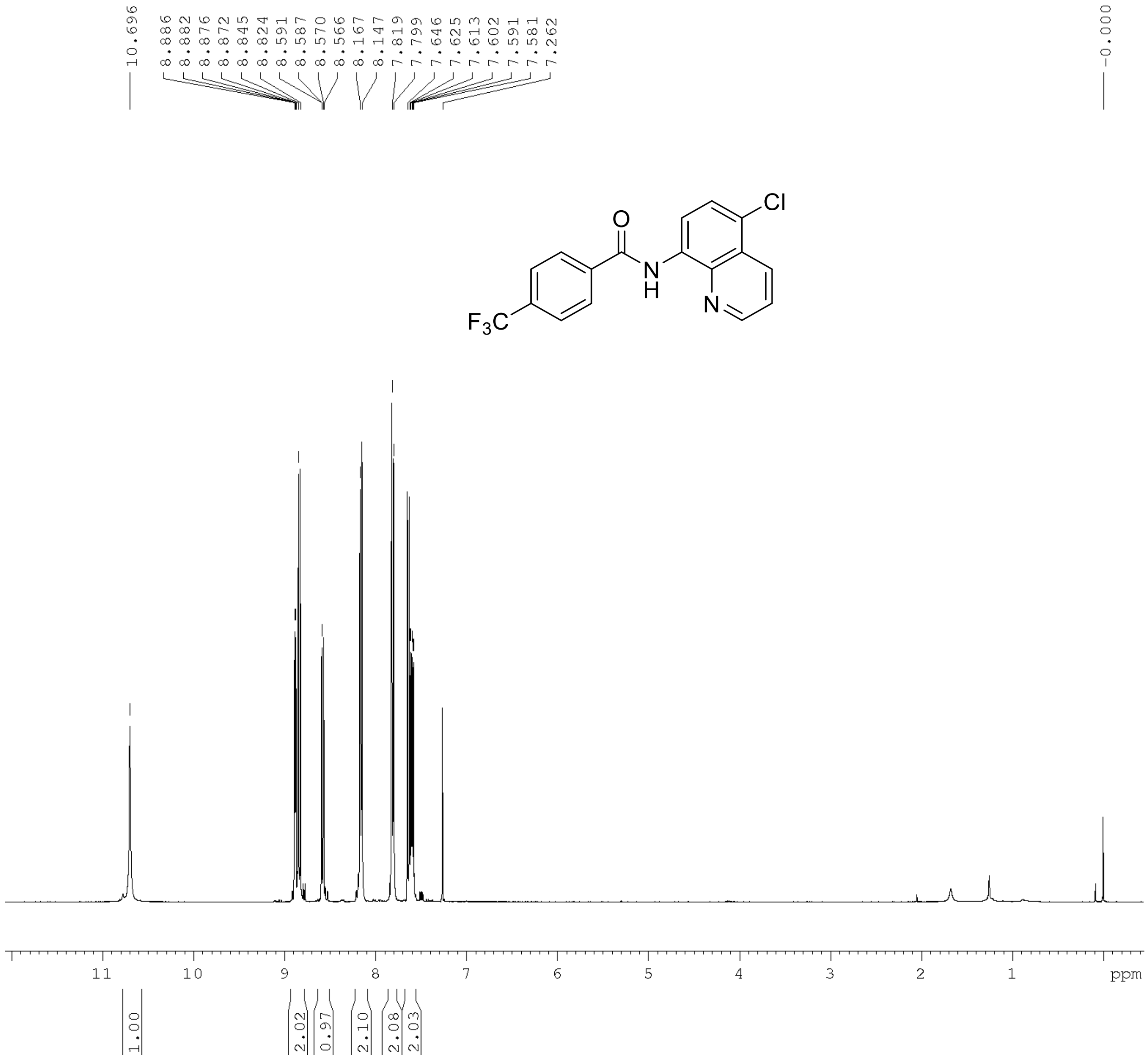

$^{13}C\{^1H\}$NMR spectrum of compound **2s** ($CDCl_3$, 100 MHz)**:**

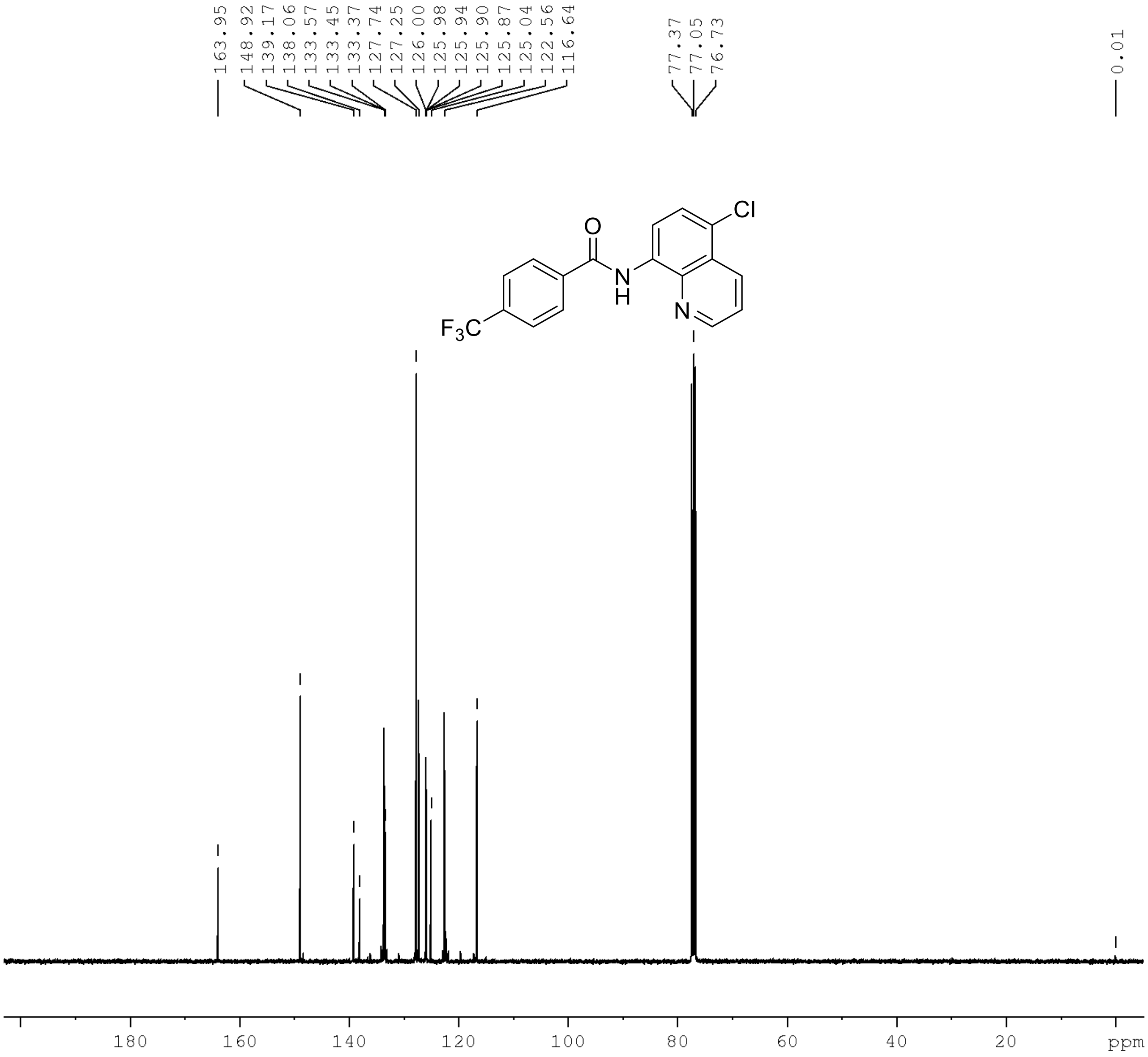

^{1}H NMR spectrum of compound **3s** ($CDCl_3$, 400 MHz)**:**

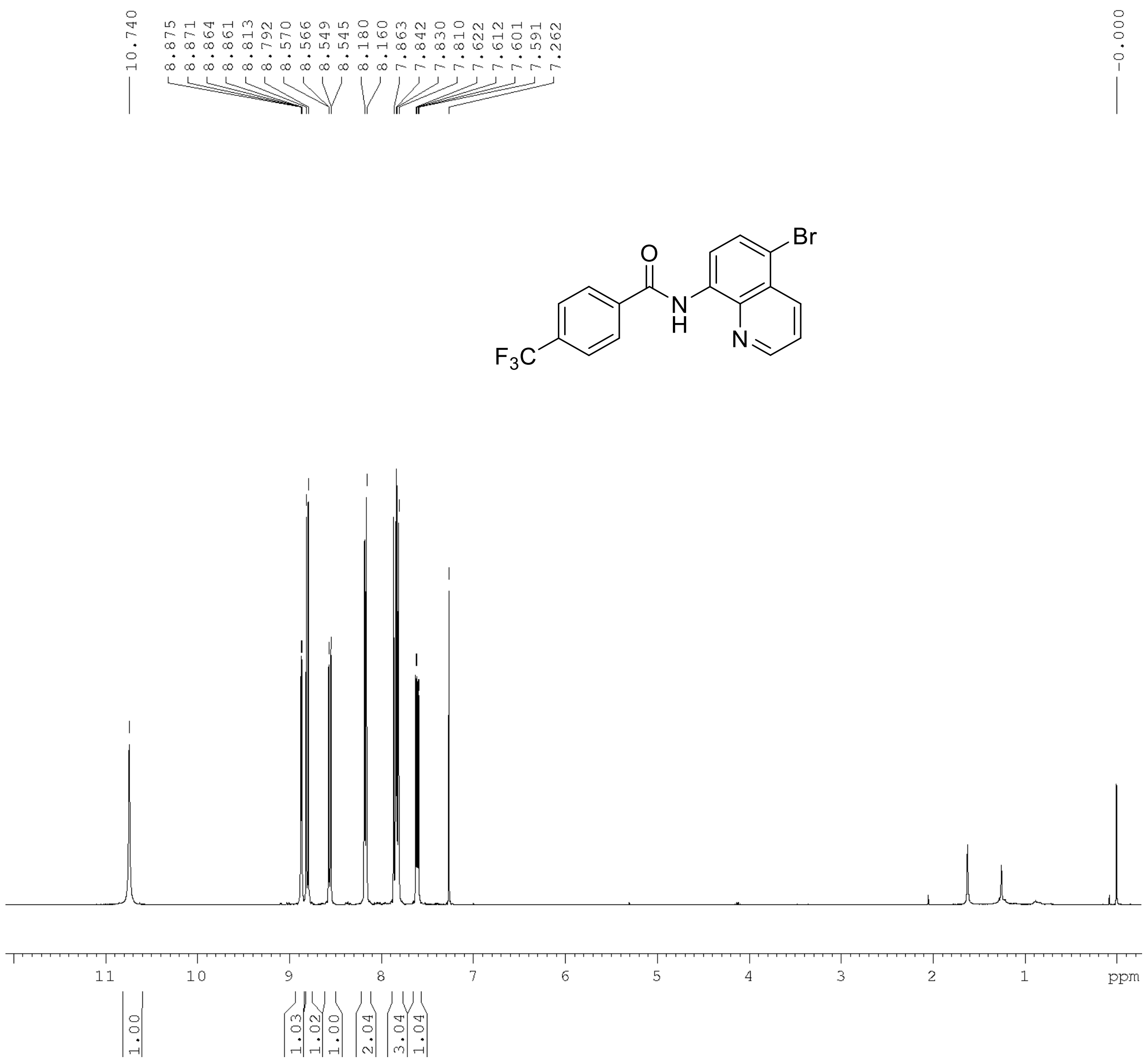

$^{13}C\{^1H\}$NMR spectrum of compound **3s** ($CDCl_3$, 100 MHz)**:**

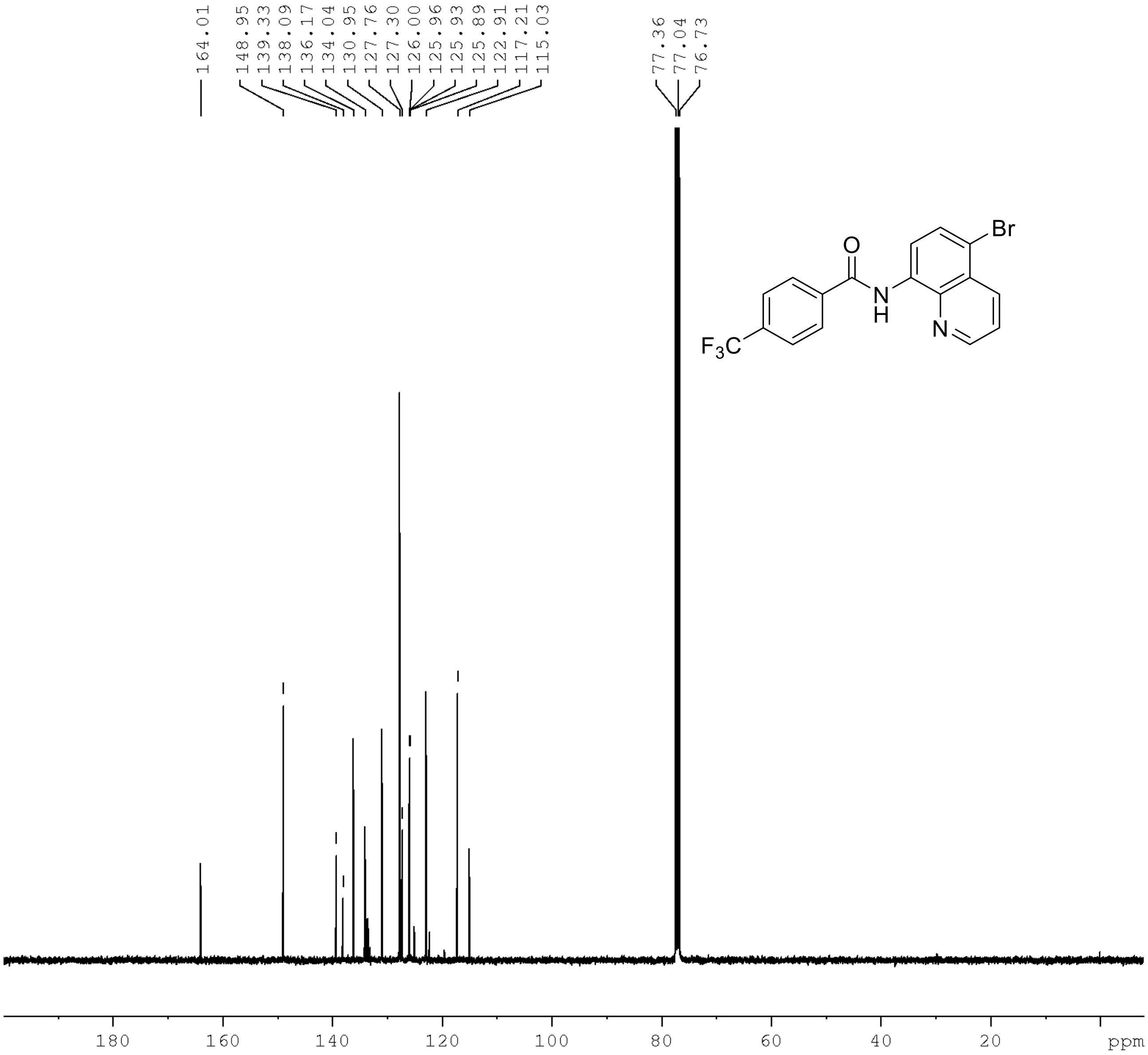

^{1}H NMR spectrum of compound **2t** ($CDCl_3$, 400 MHz)**:**

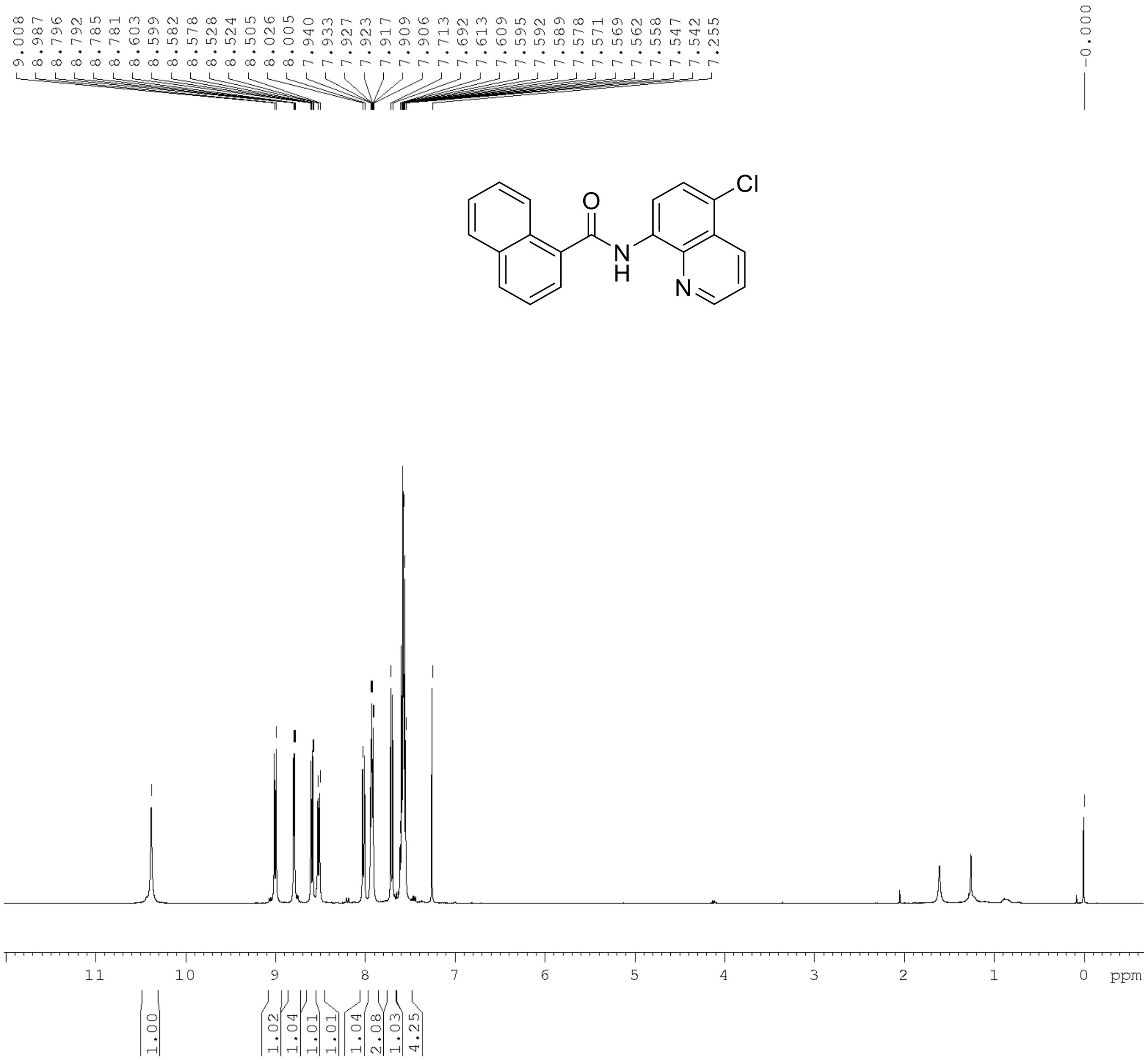

$^{13}C\{^{1}H\}$NMR spectrum of compound **2t** ($CDCl_3$, 100 MHz)**:**

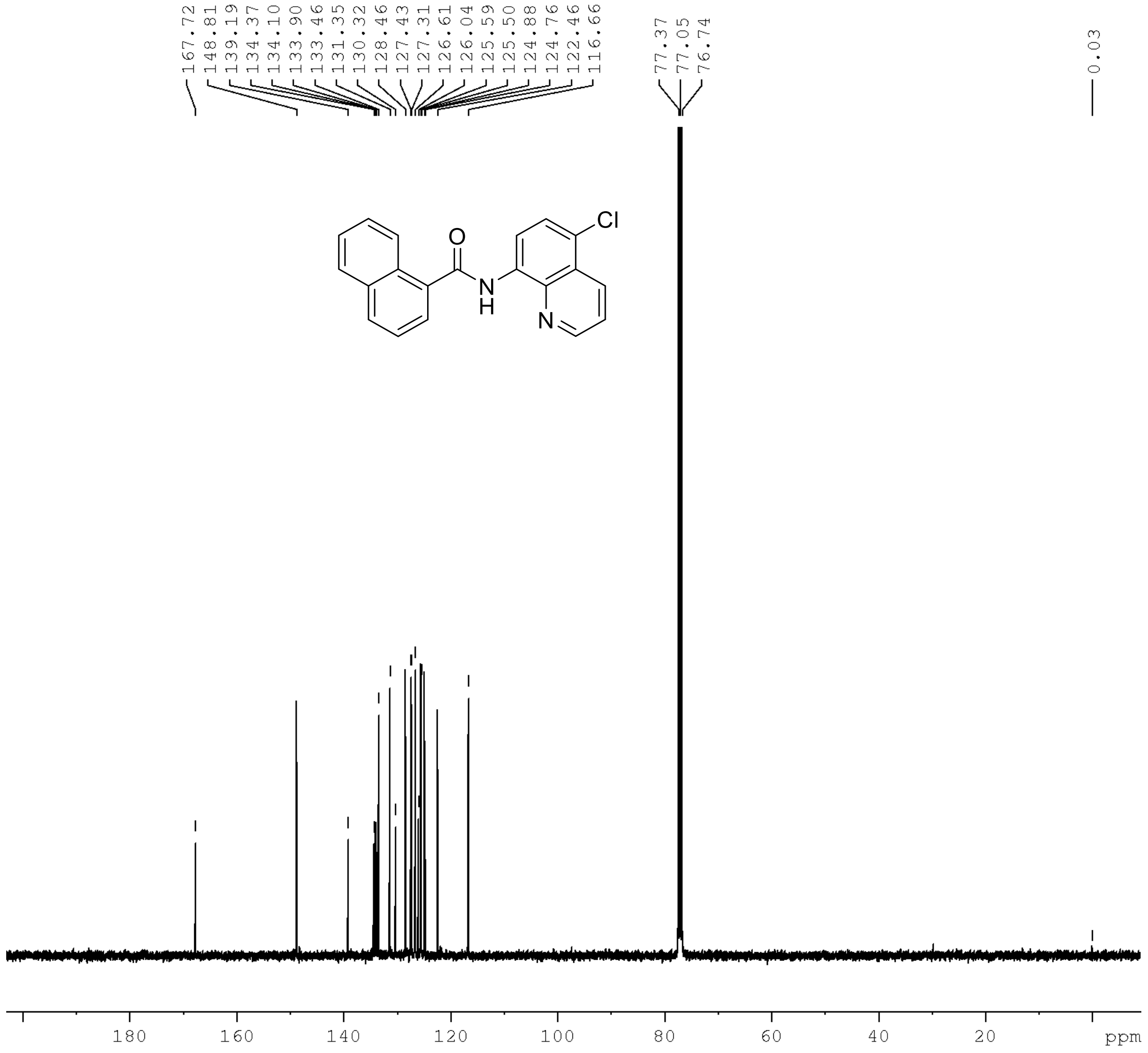

^{1}H NMR spectrum of compound **3t** ($CDCl_3$, 400 MHz)**:**

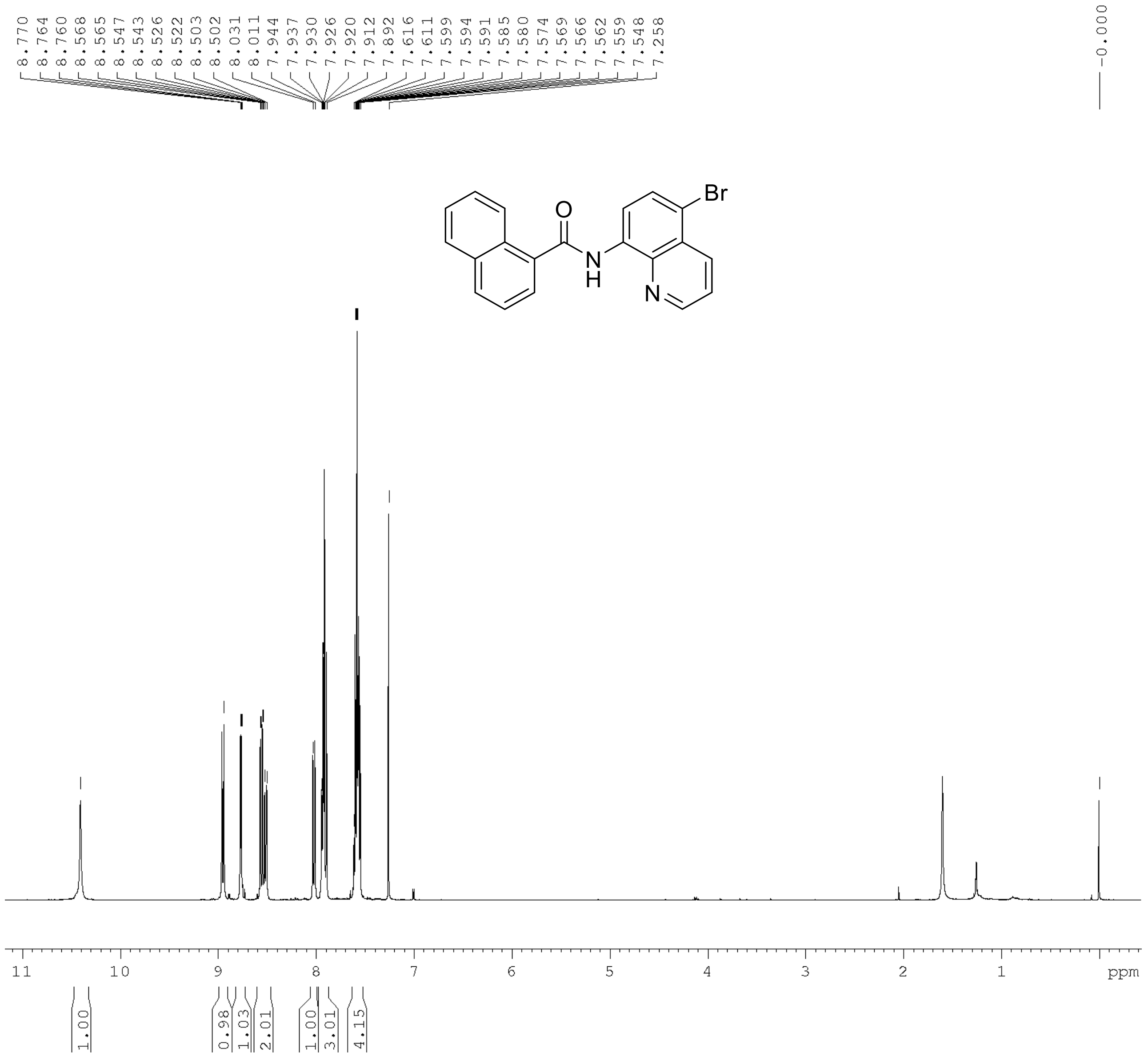

$^{13}C\{^1H\}$NMR spectrum of compound **3t** ($CDCl_3$, 100 MHz)**:**

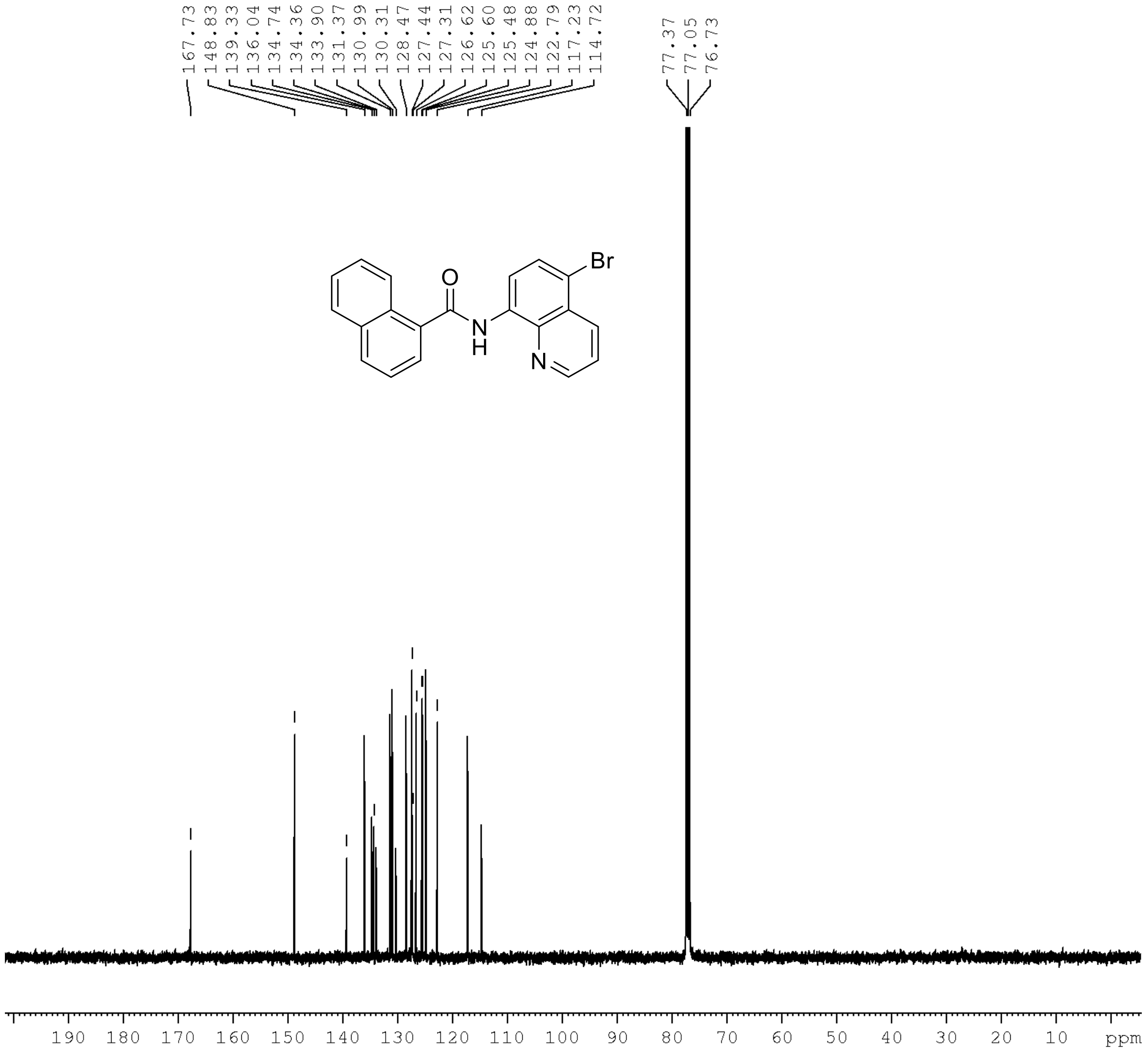

HRMS spectrum of compound **3t:**

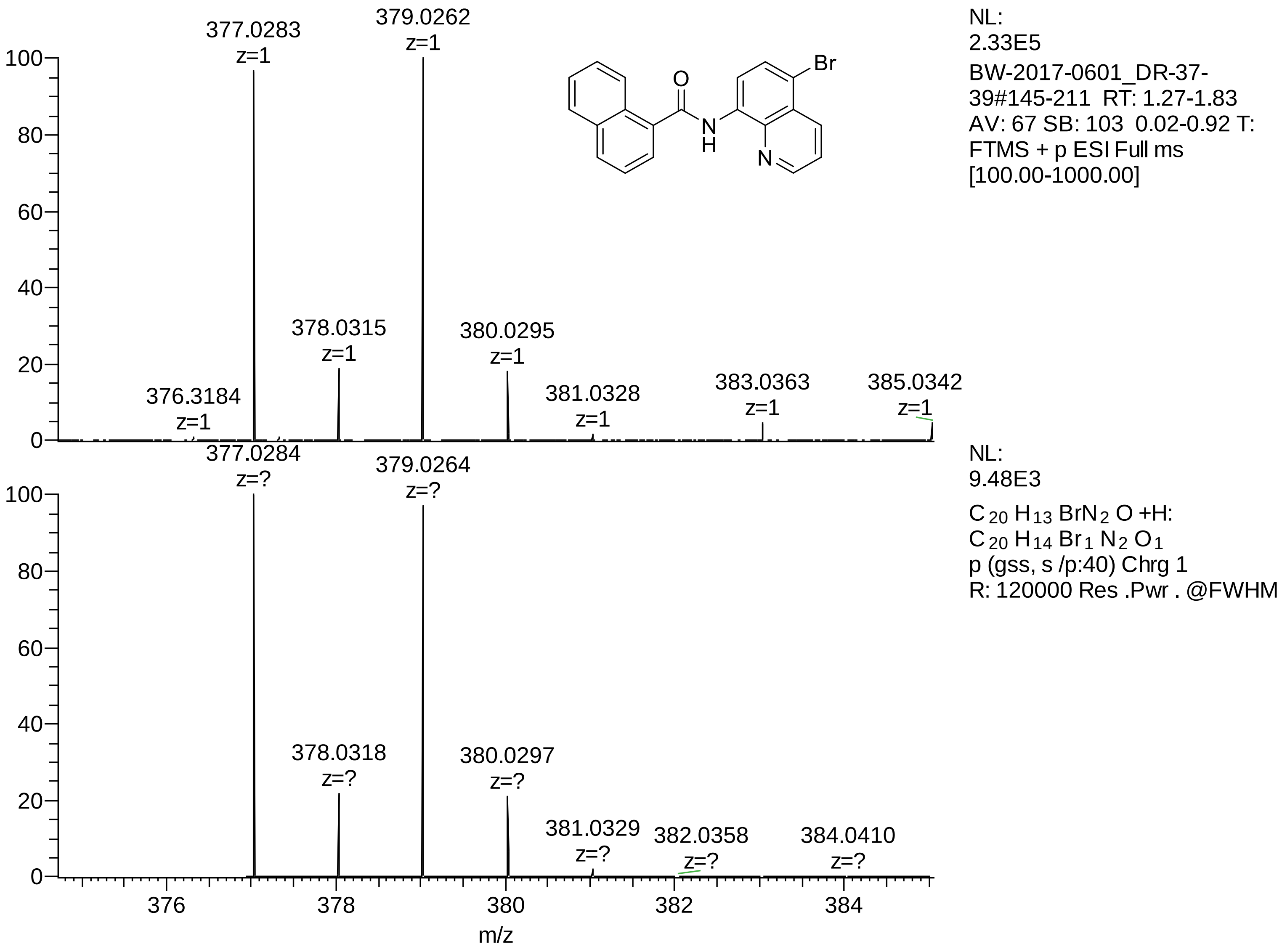

^{1}H NMR spectrum of compound **2u** ($CDCl_3$, 400 MHz)**:**

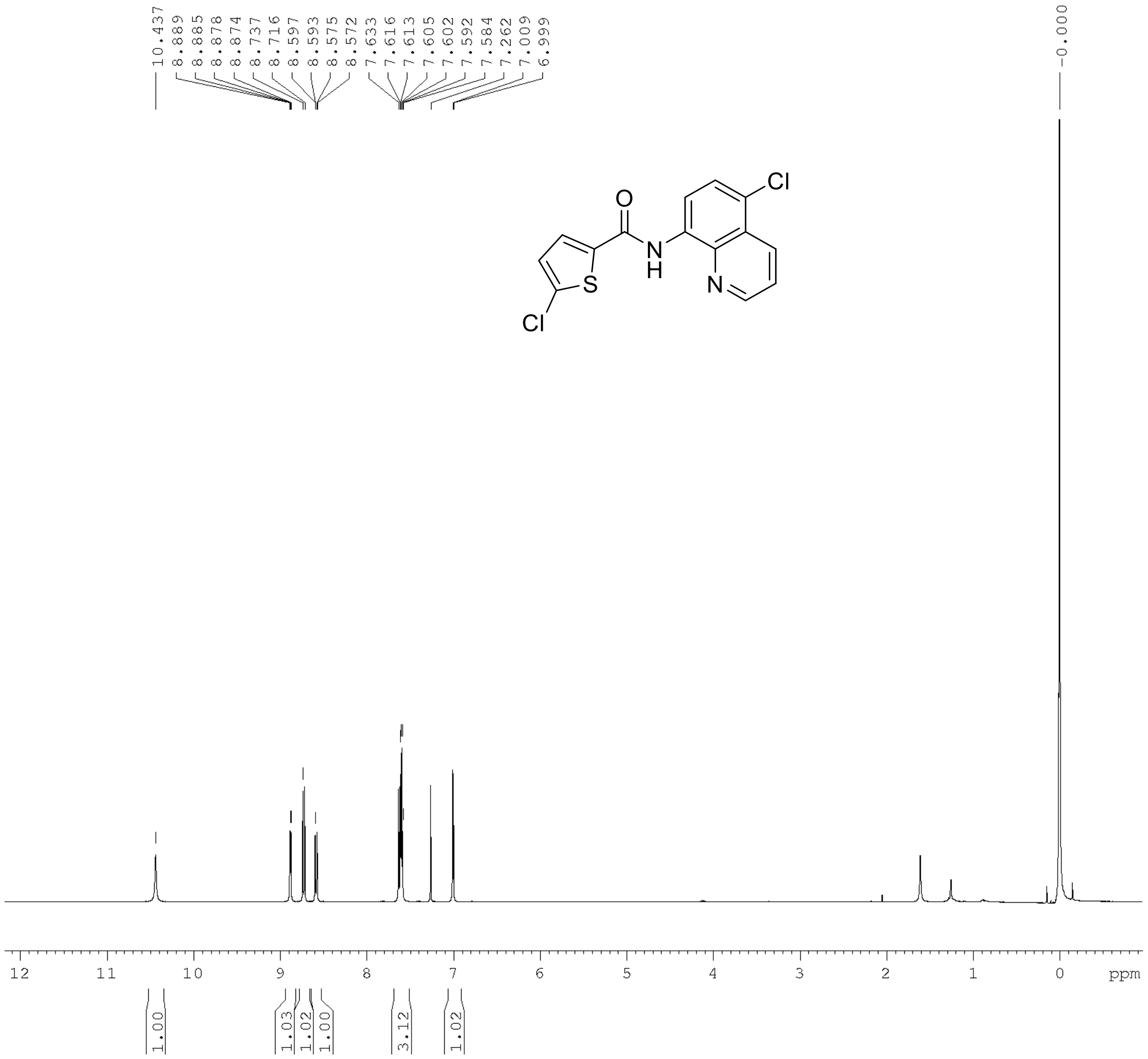

$^{13}C\{^{1}H\}$NMR spectrum of compound **2u** ($CDCl_3$, 100 MHz):

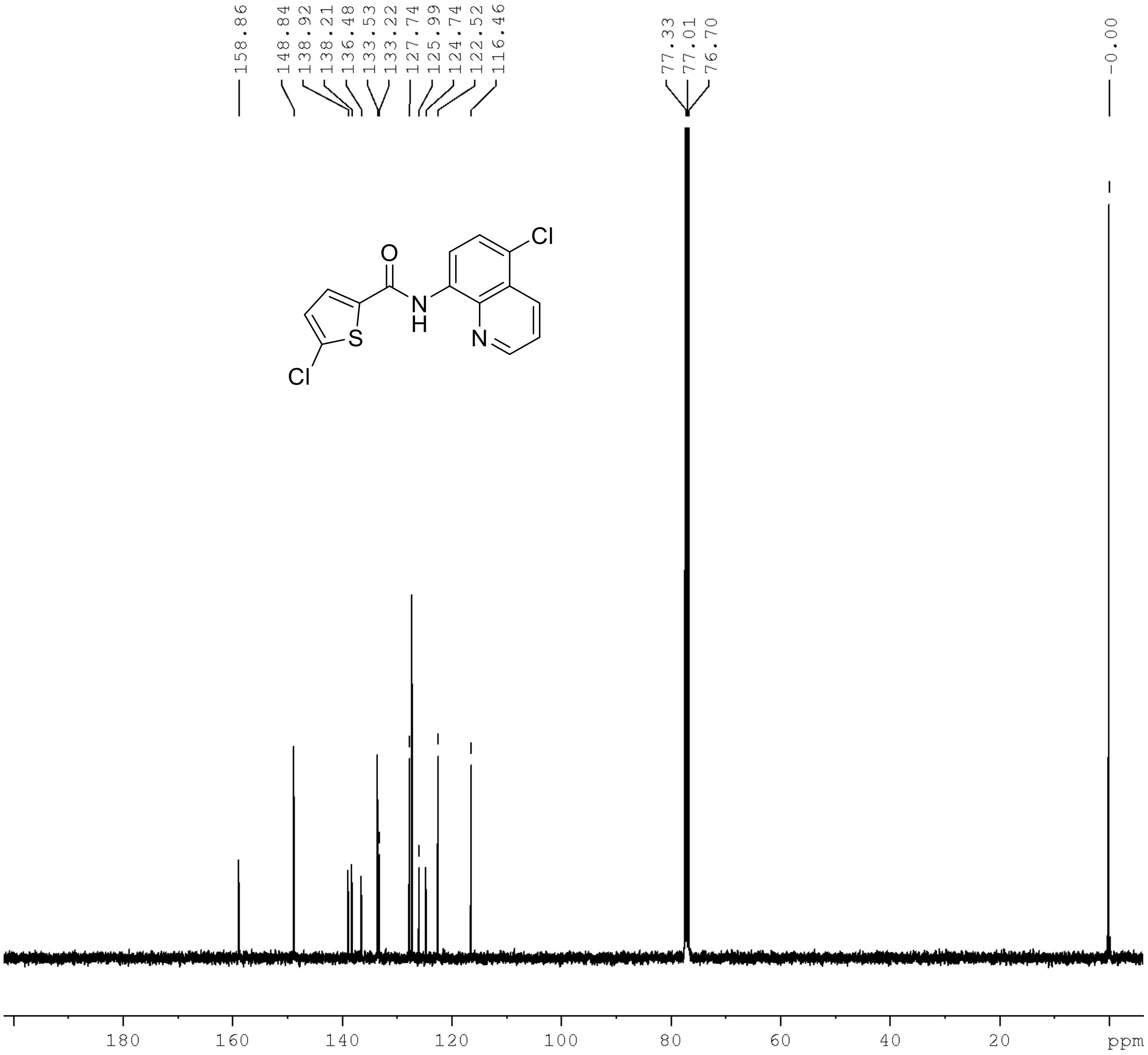

HRMS spectrum of compound **2u:**

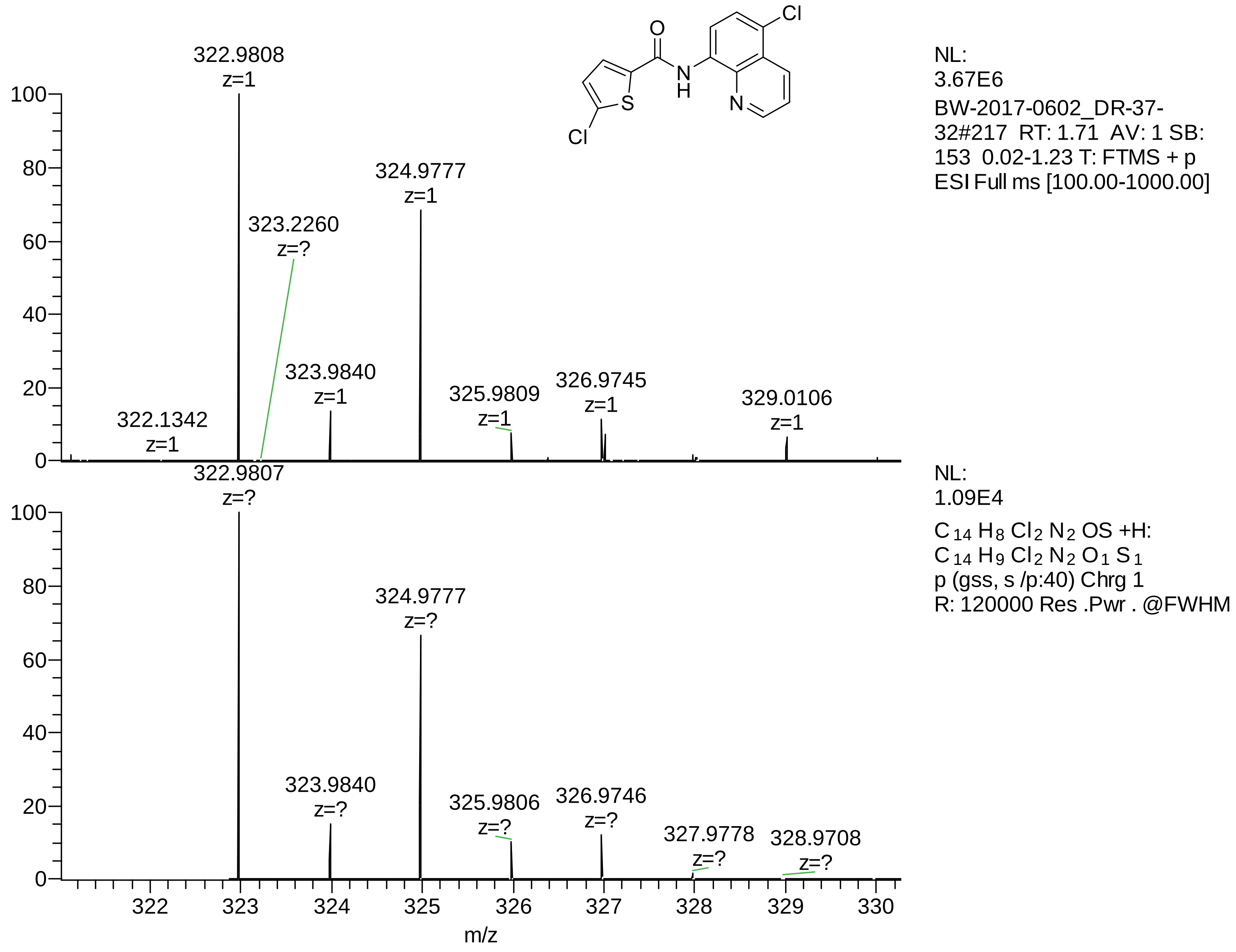

^{1}H NMR spectrum of compound **3u** ($CDCl_3$, 400 MHz)**:**

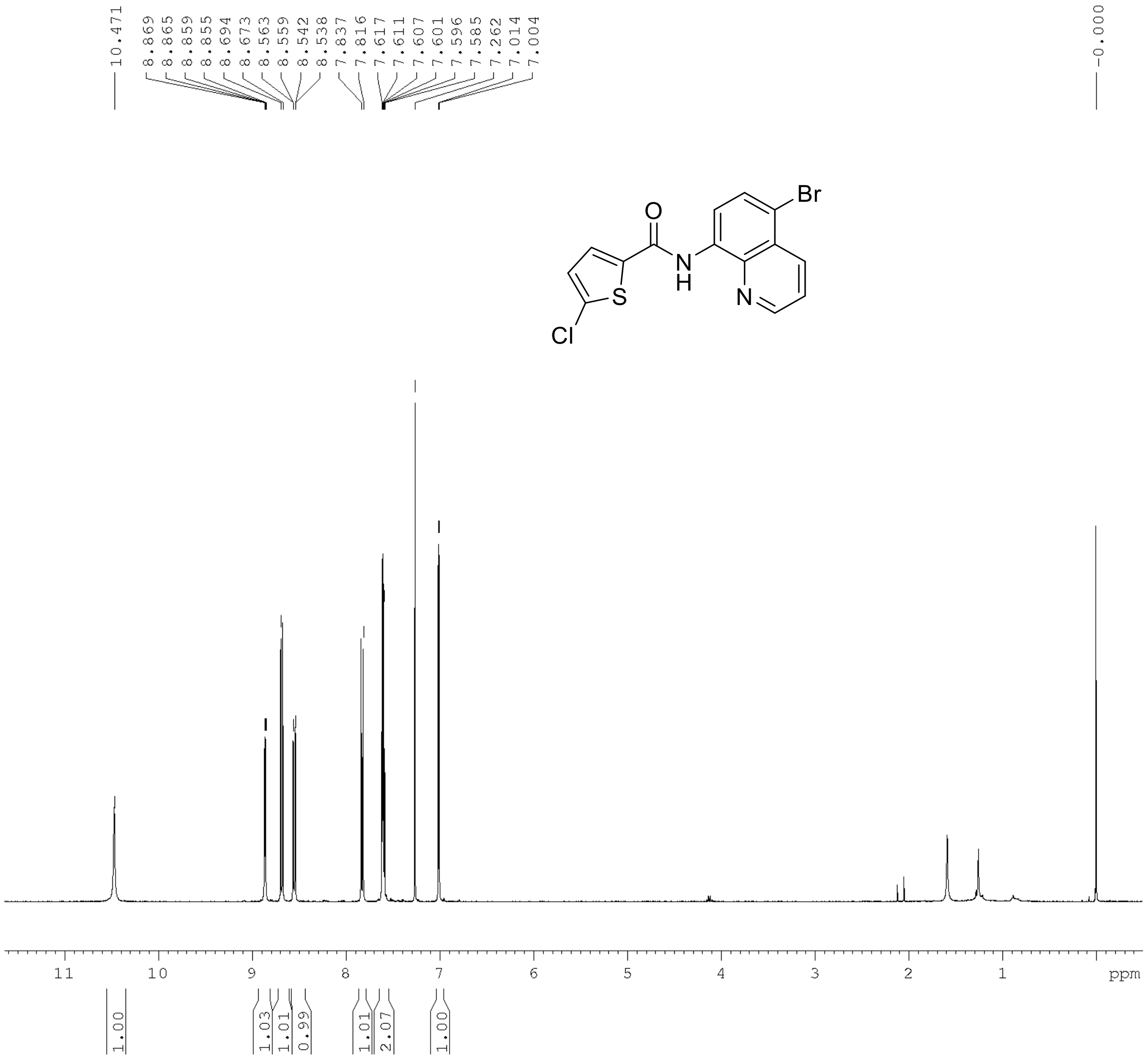

$^{13}C\{^{1}H\}$NMR spectrum of compound **3u** ($CDCl_3$, 100 MHz)**:**

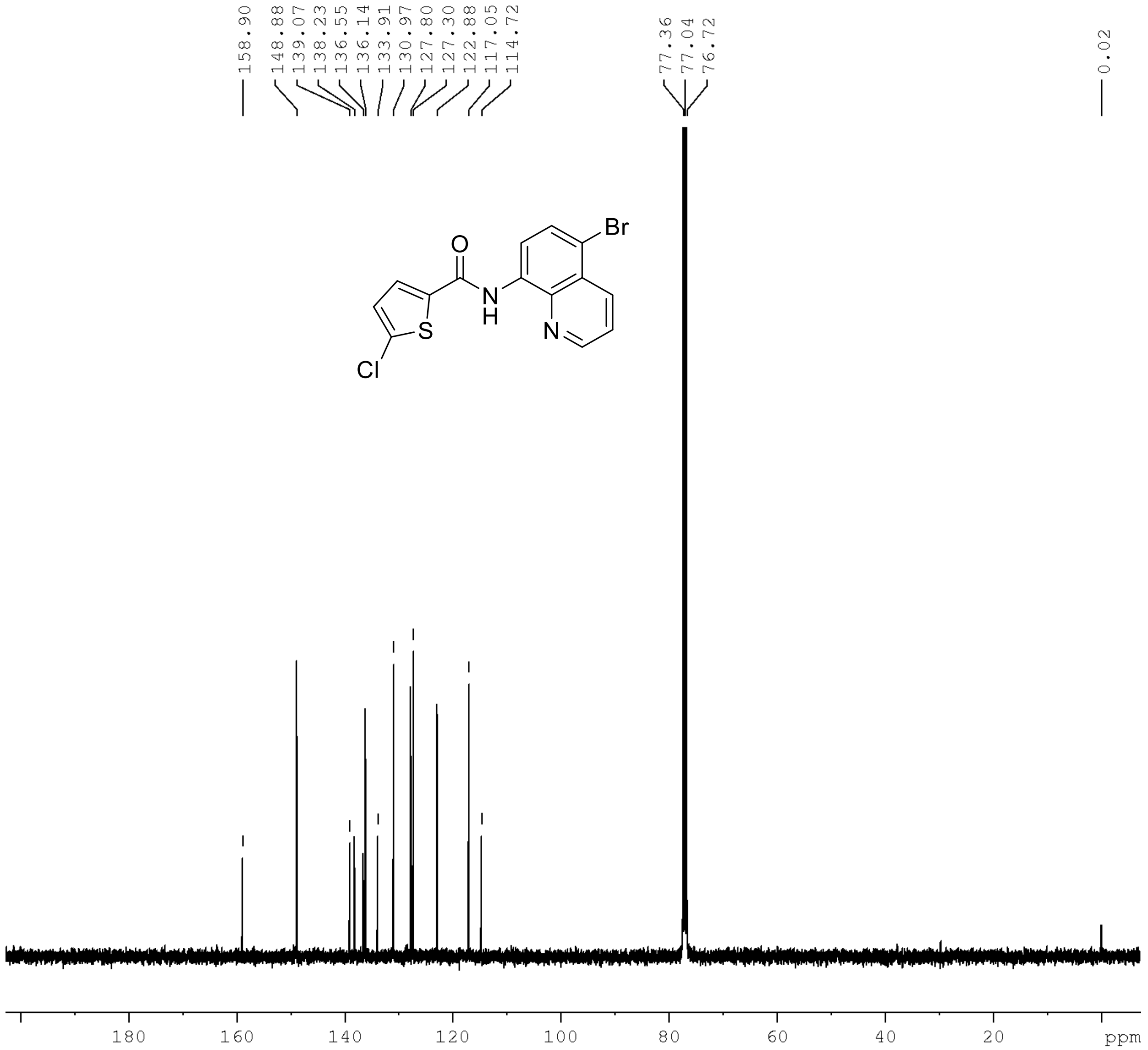

HRMS spectrum of compound **3u:**

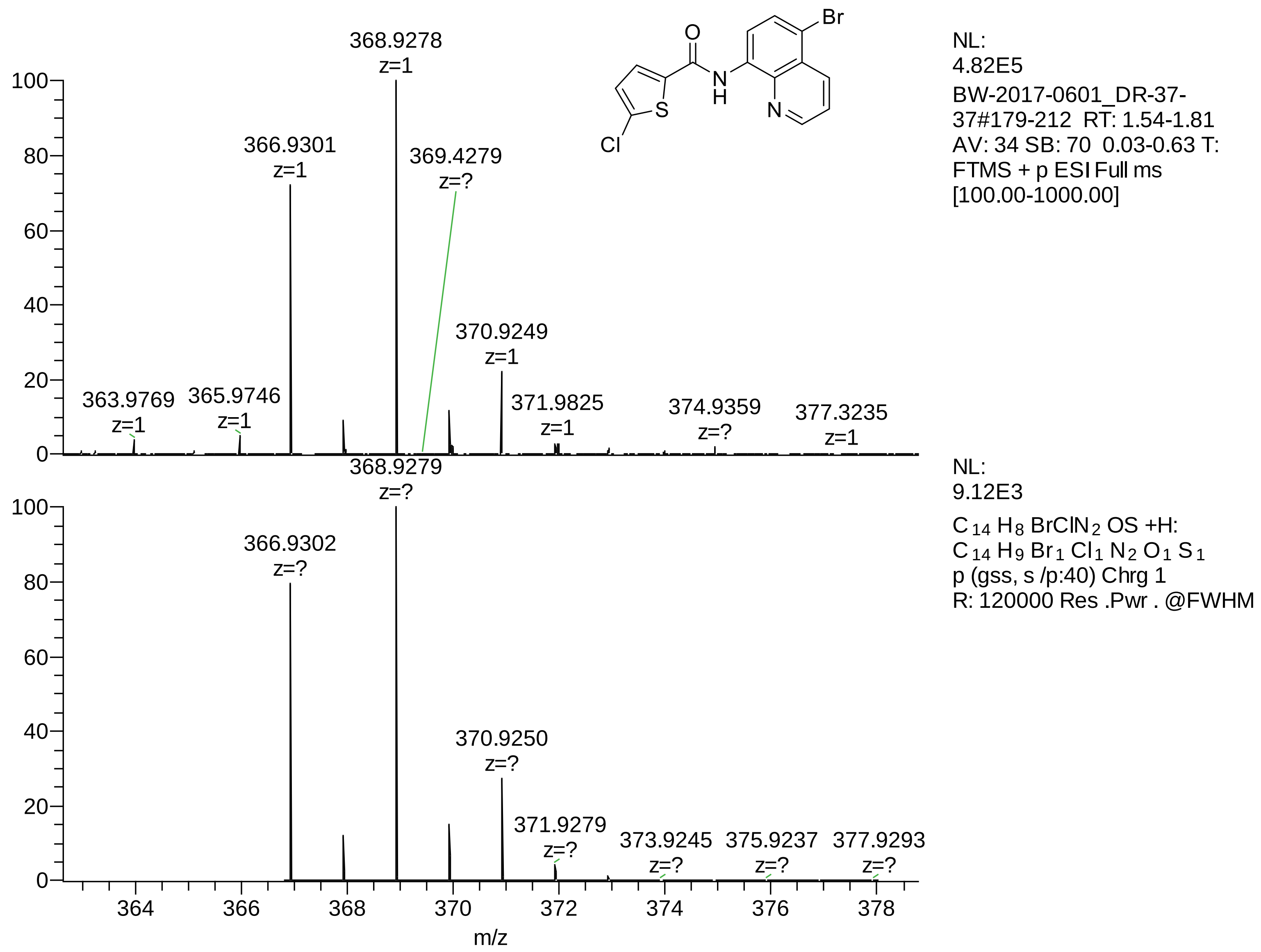

^{1}H NMR spectrum of compound **2v** ($CDCl_3$, 400 MHz)**:**

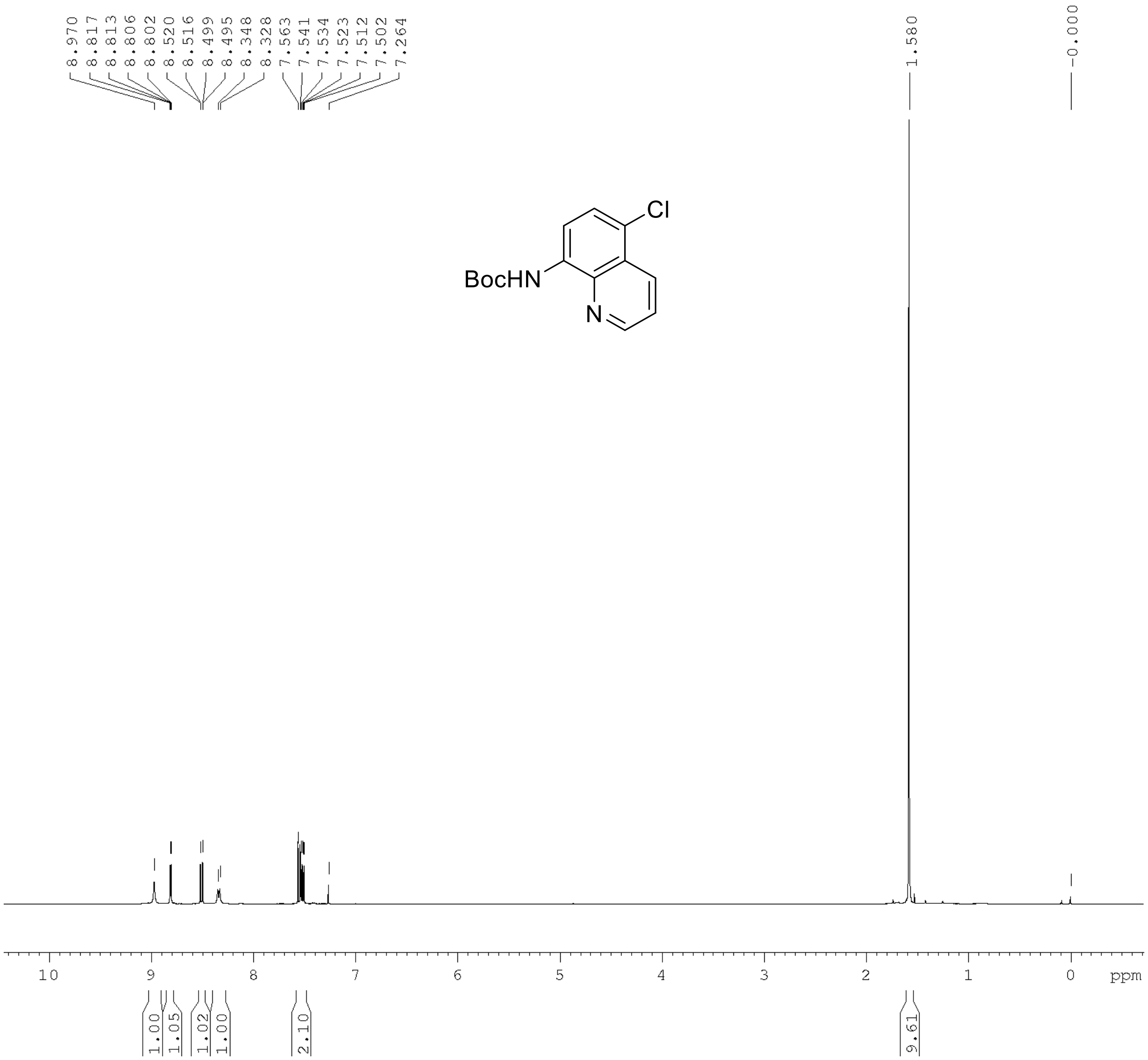

$^{13}C\{^{1}H\}$NMR spectrum of compound **2v** ($CDCl_3$, 100 MHz)**:**

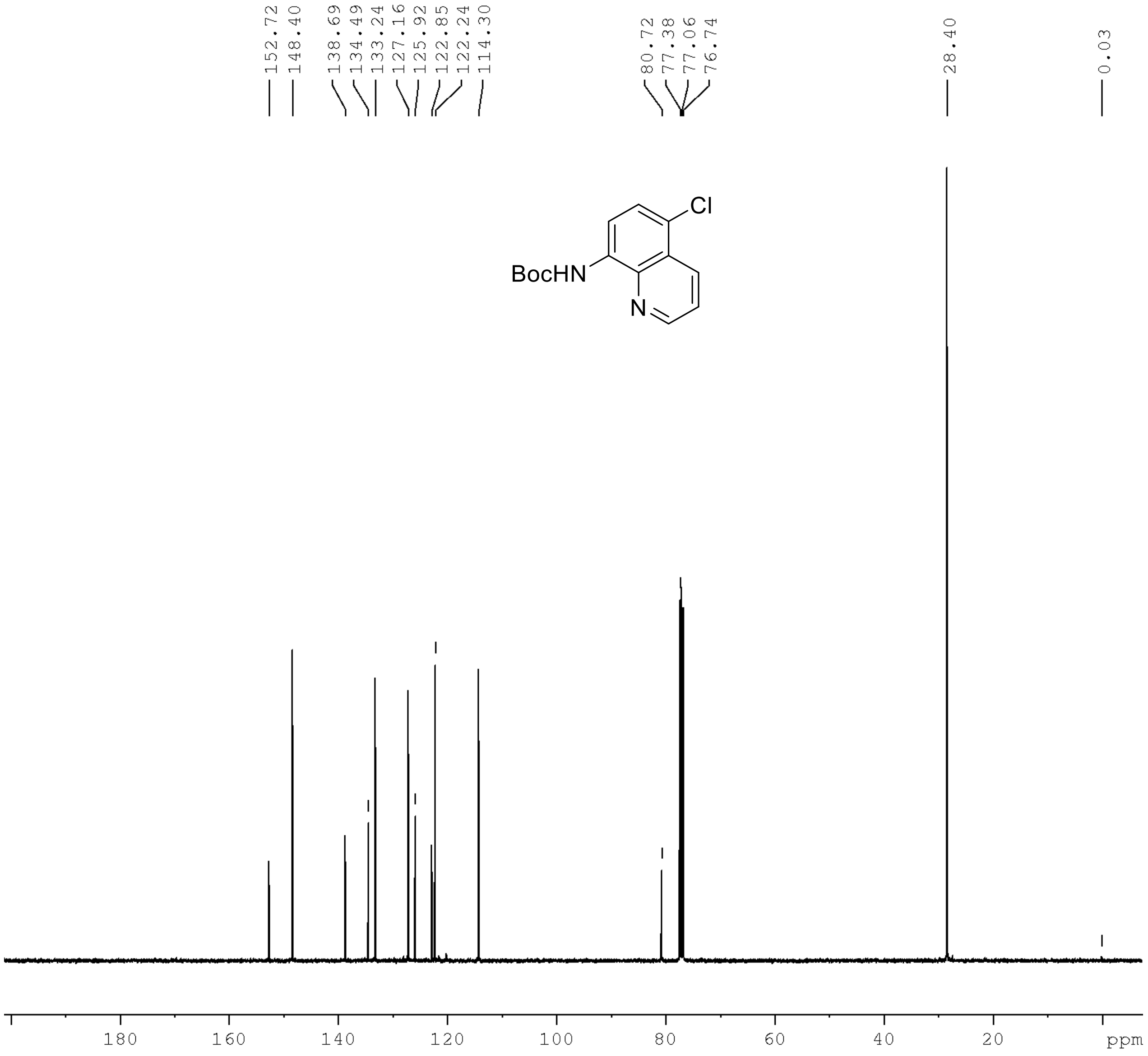

DEPT135 spectrum of compound **2v** ($CDCl_3$, 100 MHz)**:**

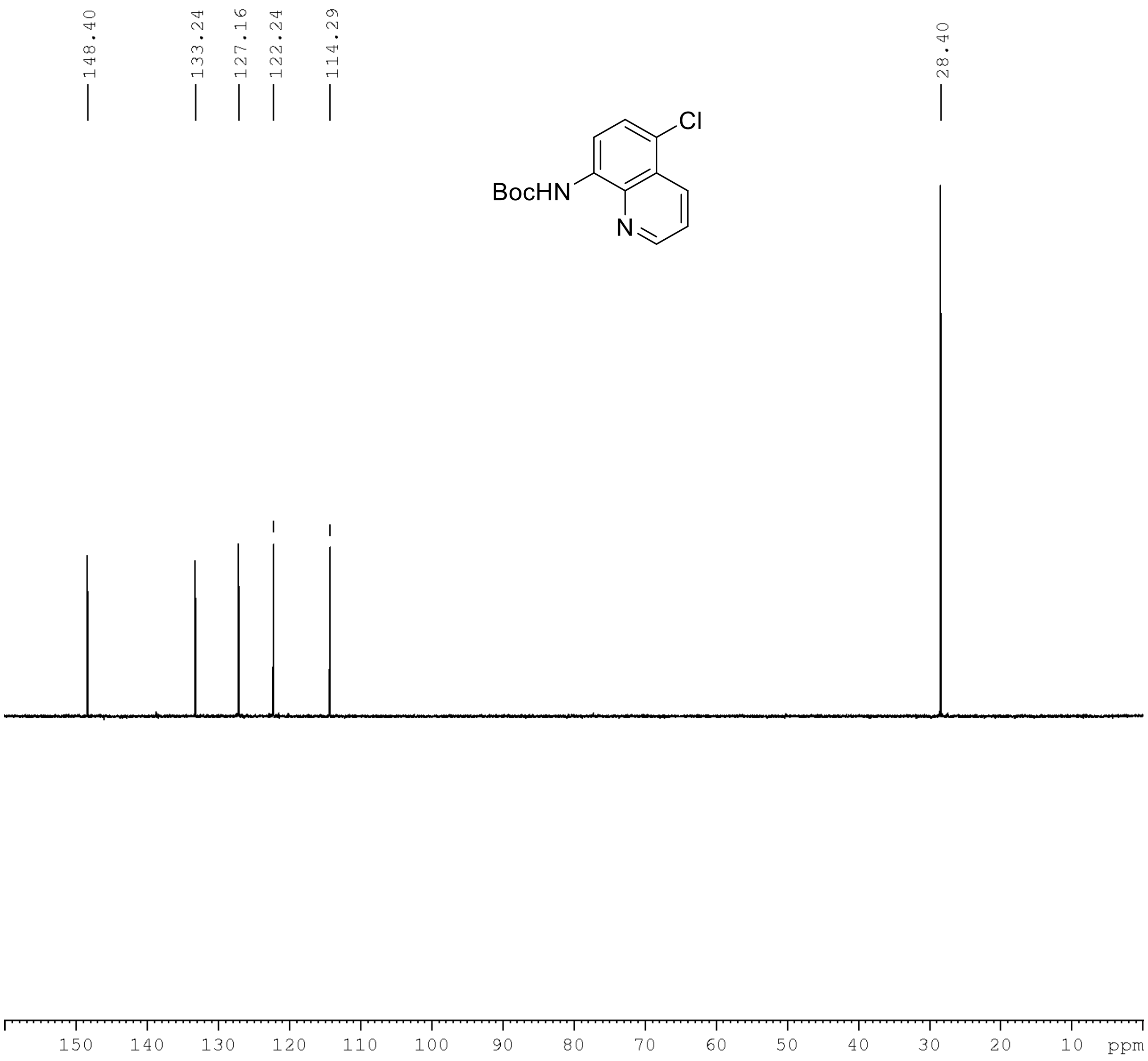

HRMS spectrum of compound **2v:**

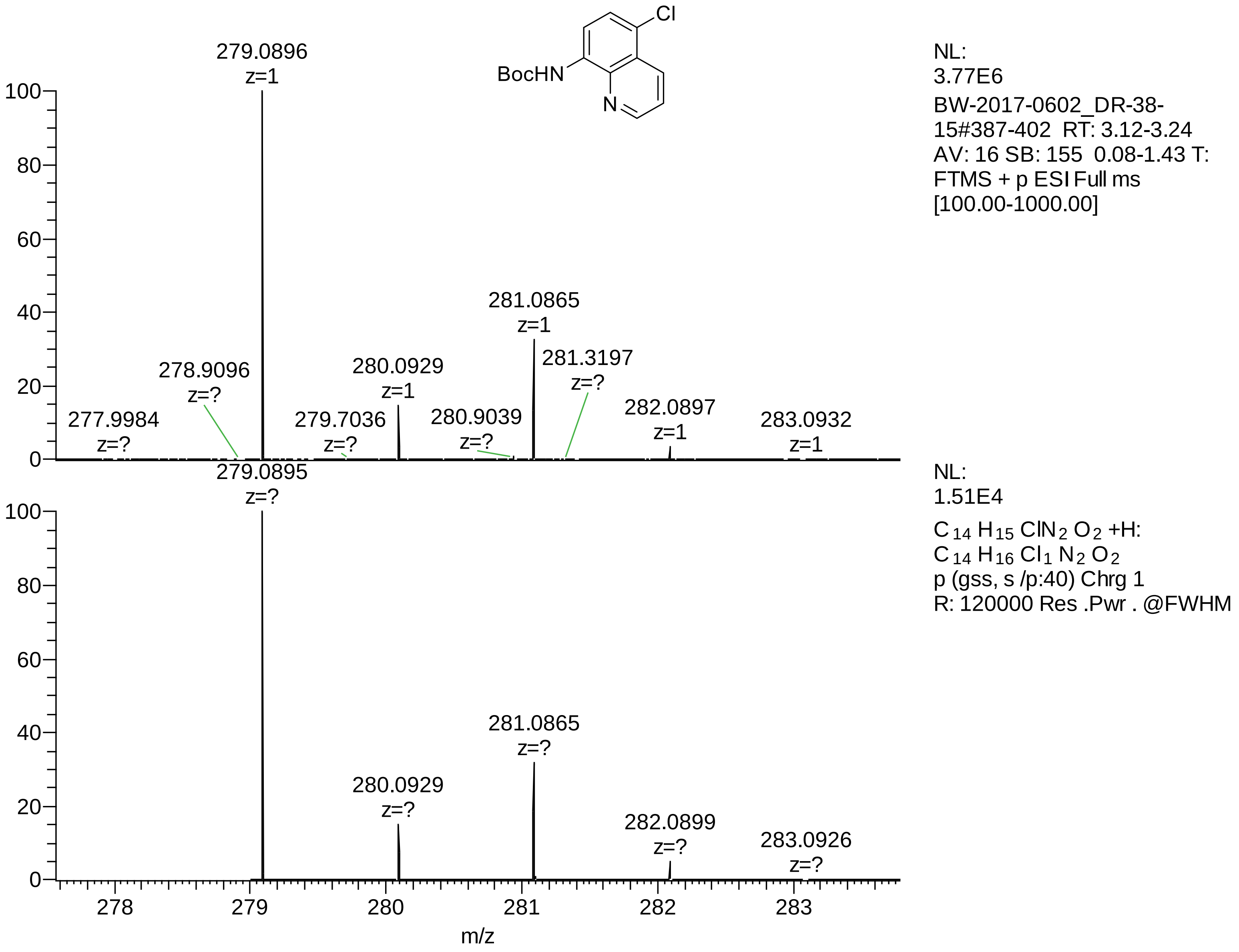

^{1}H NMR spectrum of compound **3v** ($CDCl_3$, 400 MHz)**:**

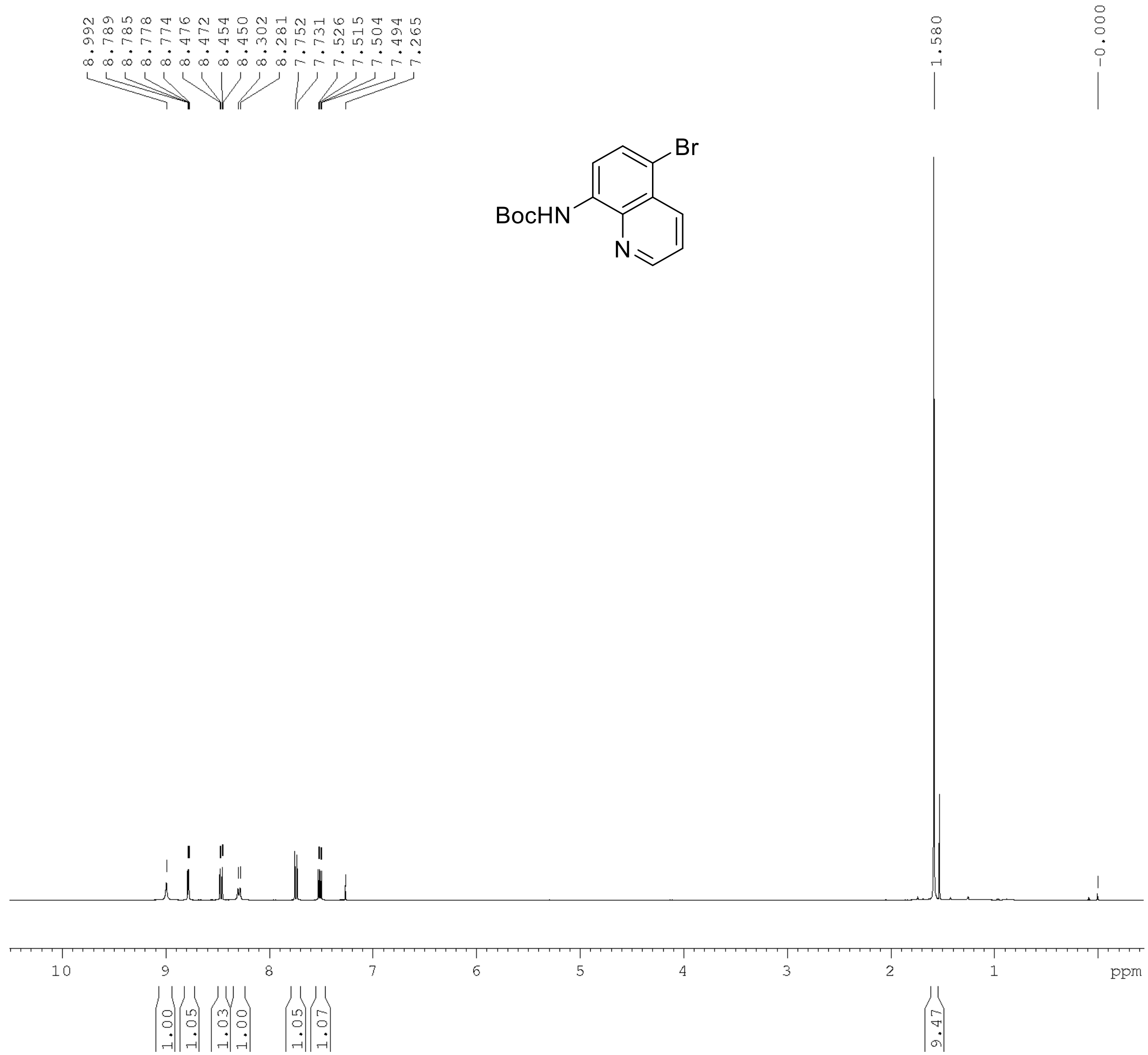

$^{13}C\{^{1}H\}$NMR spectrum of compound **3v** ($CDCl_3$, 100 MHz)**:**

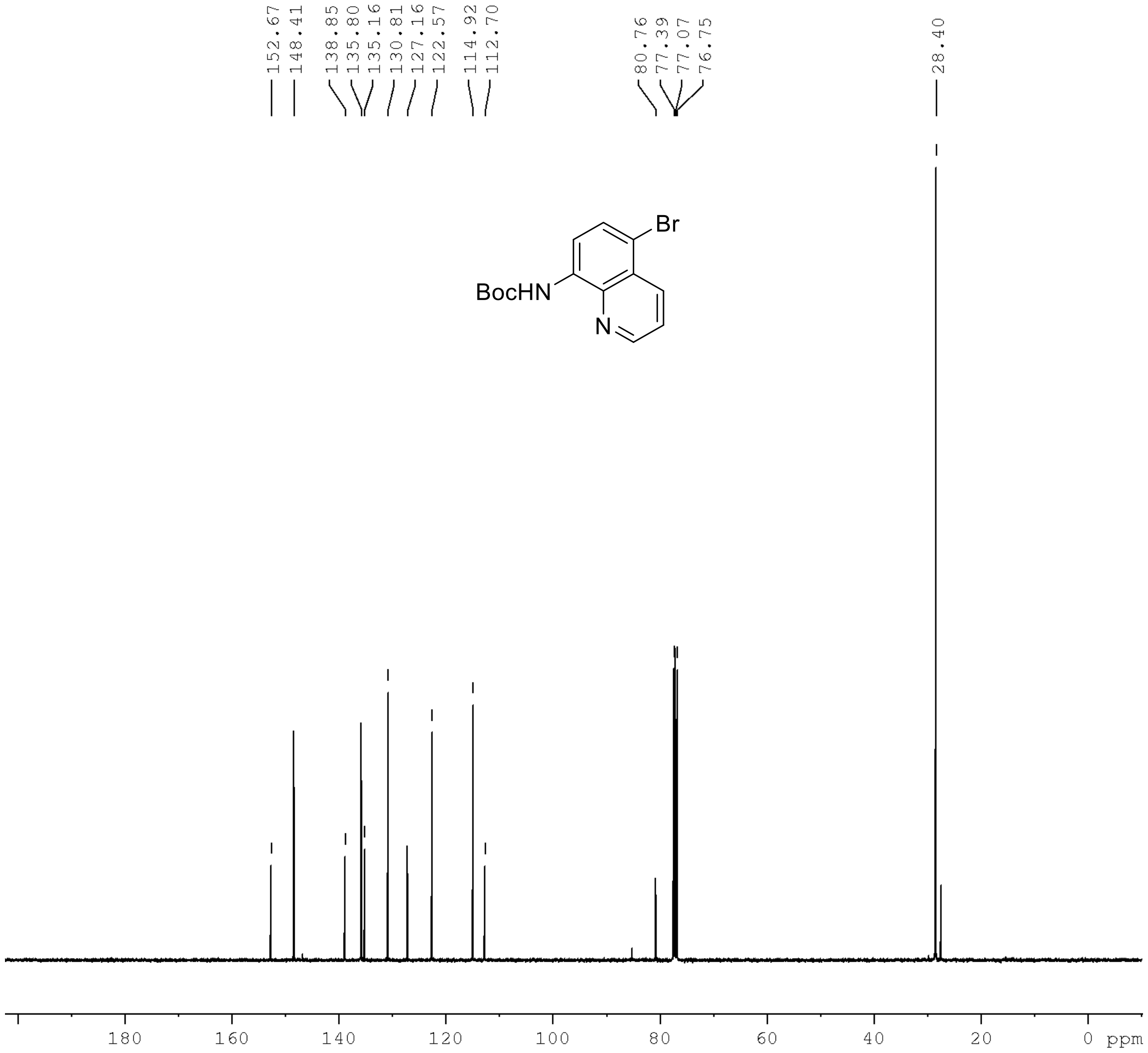

DEPT135 spectrum of compound **3v** ($CDCl_3$, 100 MHz)**:**

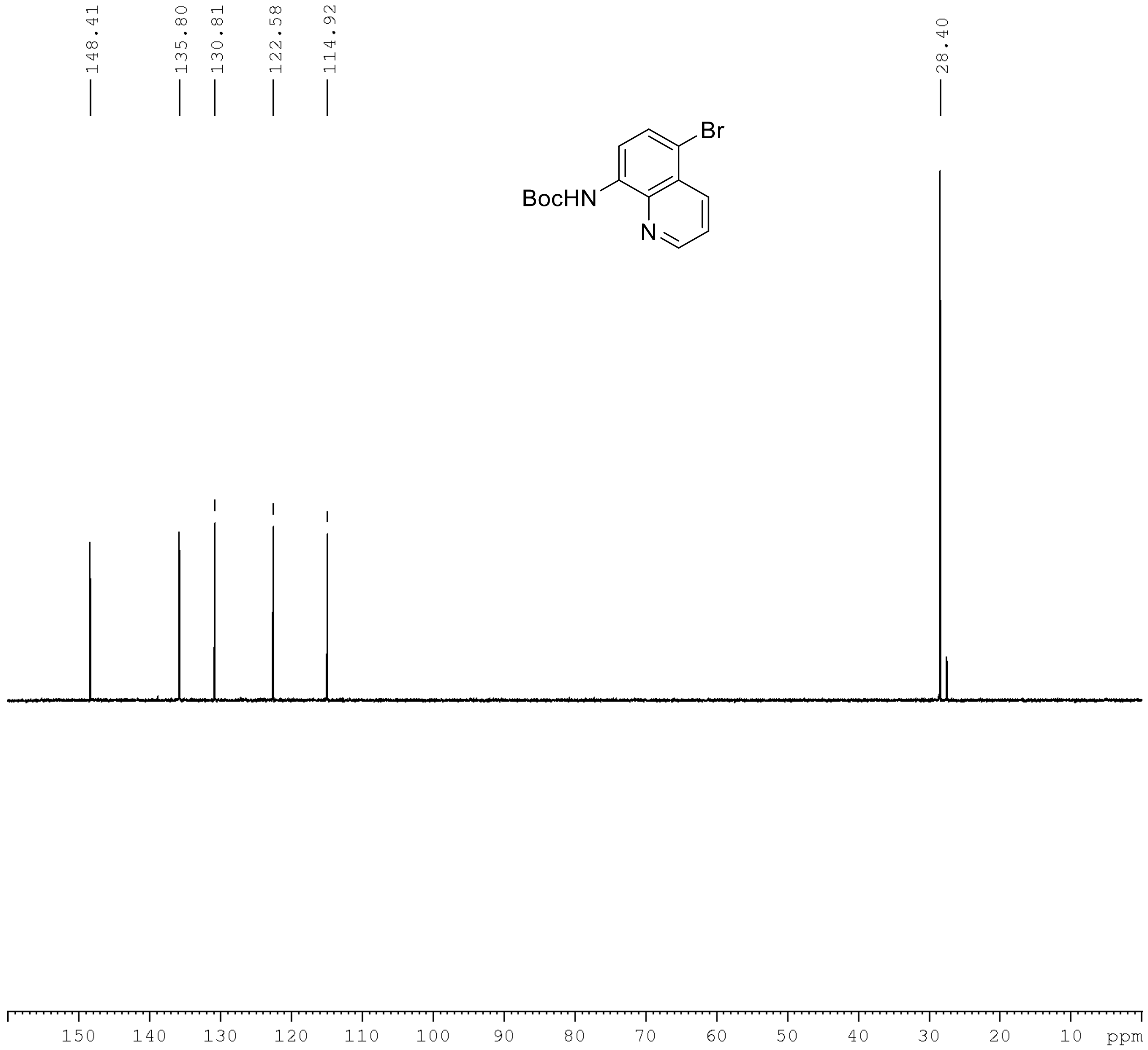

^{1}H NMR spectrum of compound **2w** ($CDCl_3$, 400 MHz)**:**

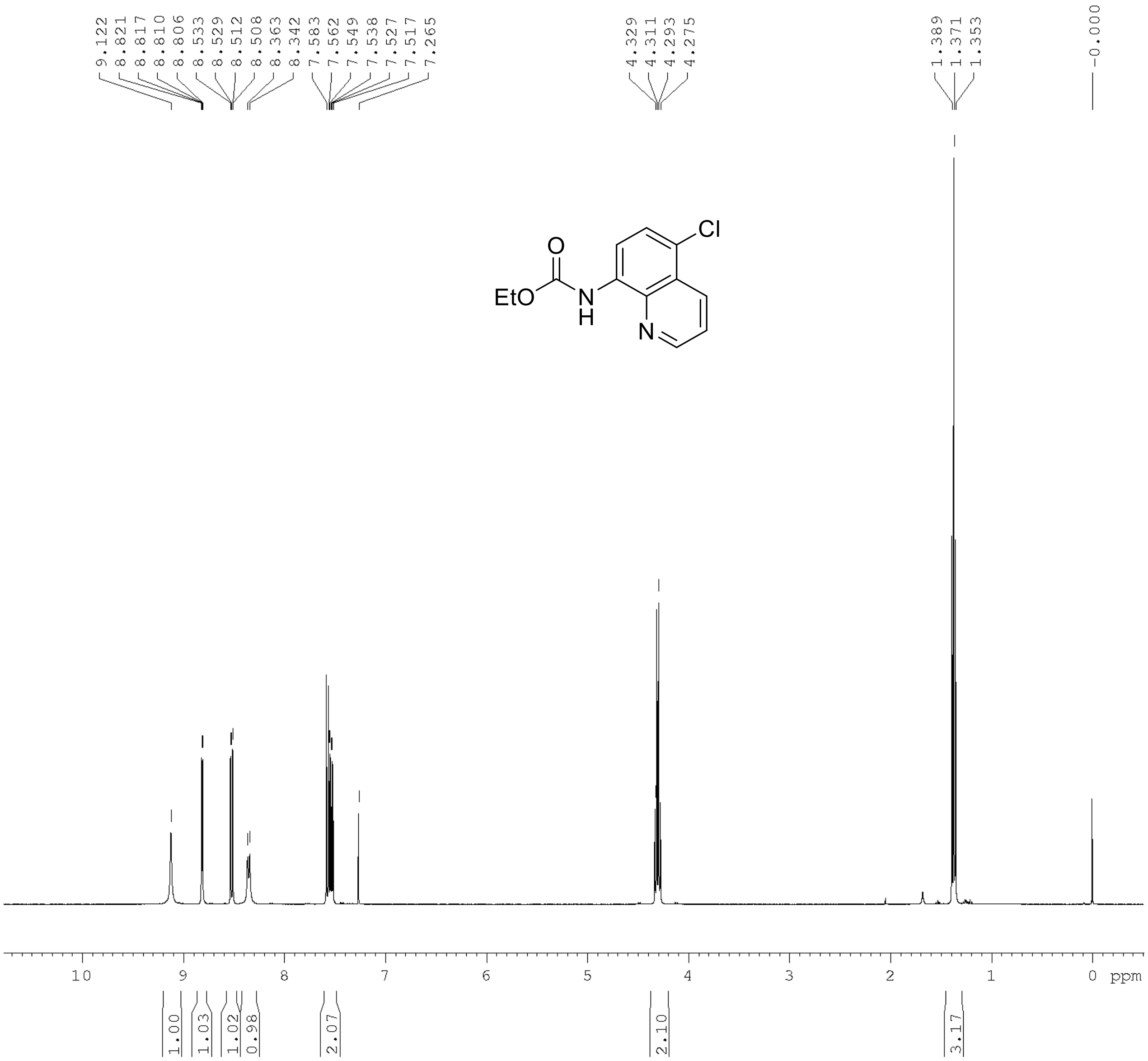

$^{13}C\{^{1}H\}$NMR spectrum of compound **2w** ($CDCl_3$, 100 MHz)**:**

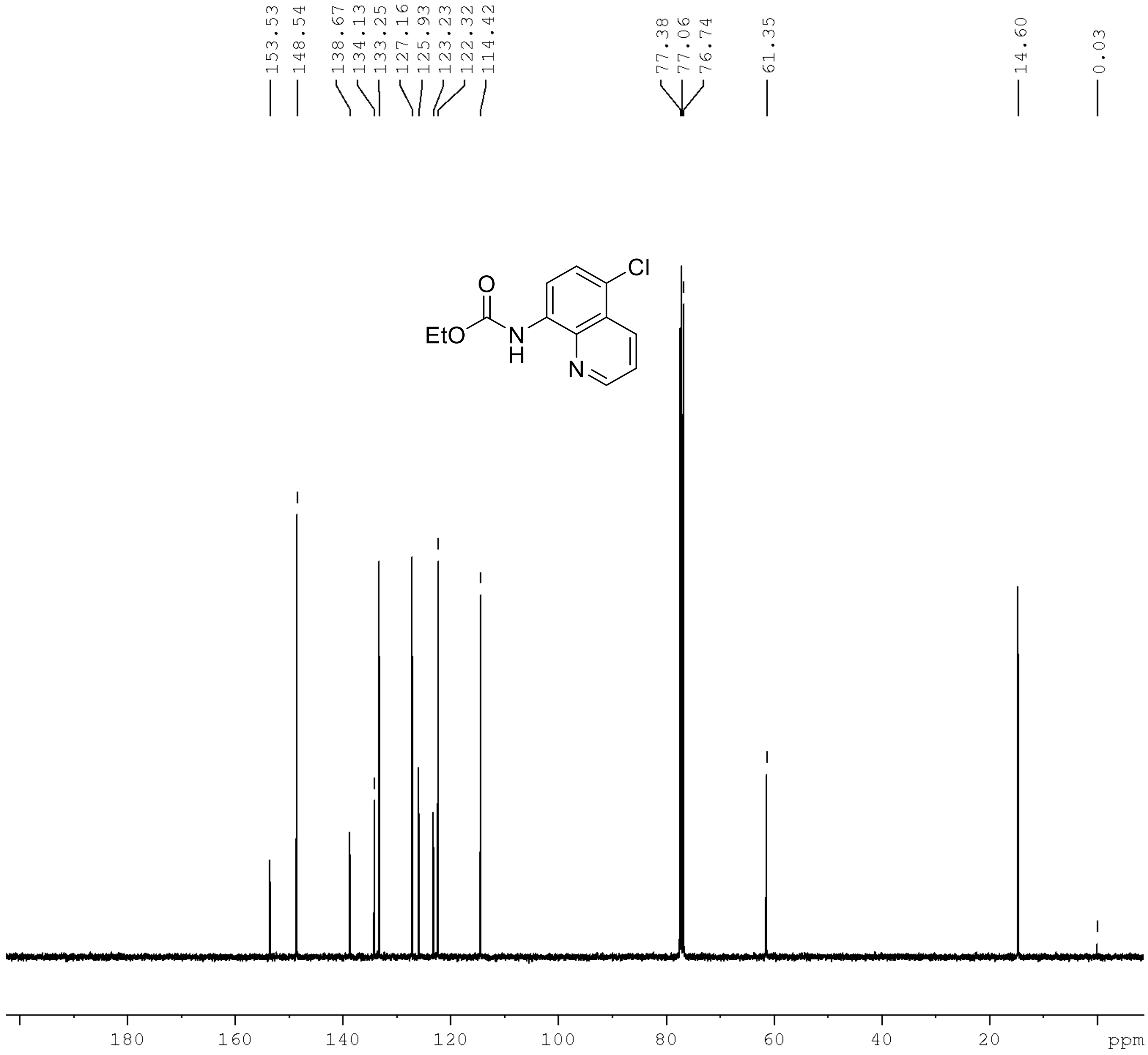

DEPT135 spectrum of compound **2w** ($CDCl_3$, 100 MHz)**:**

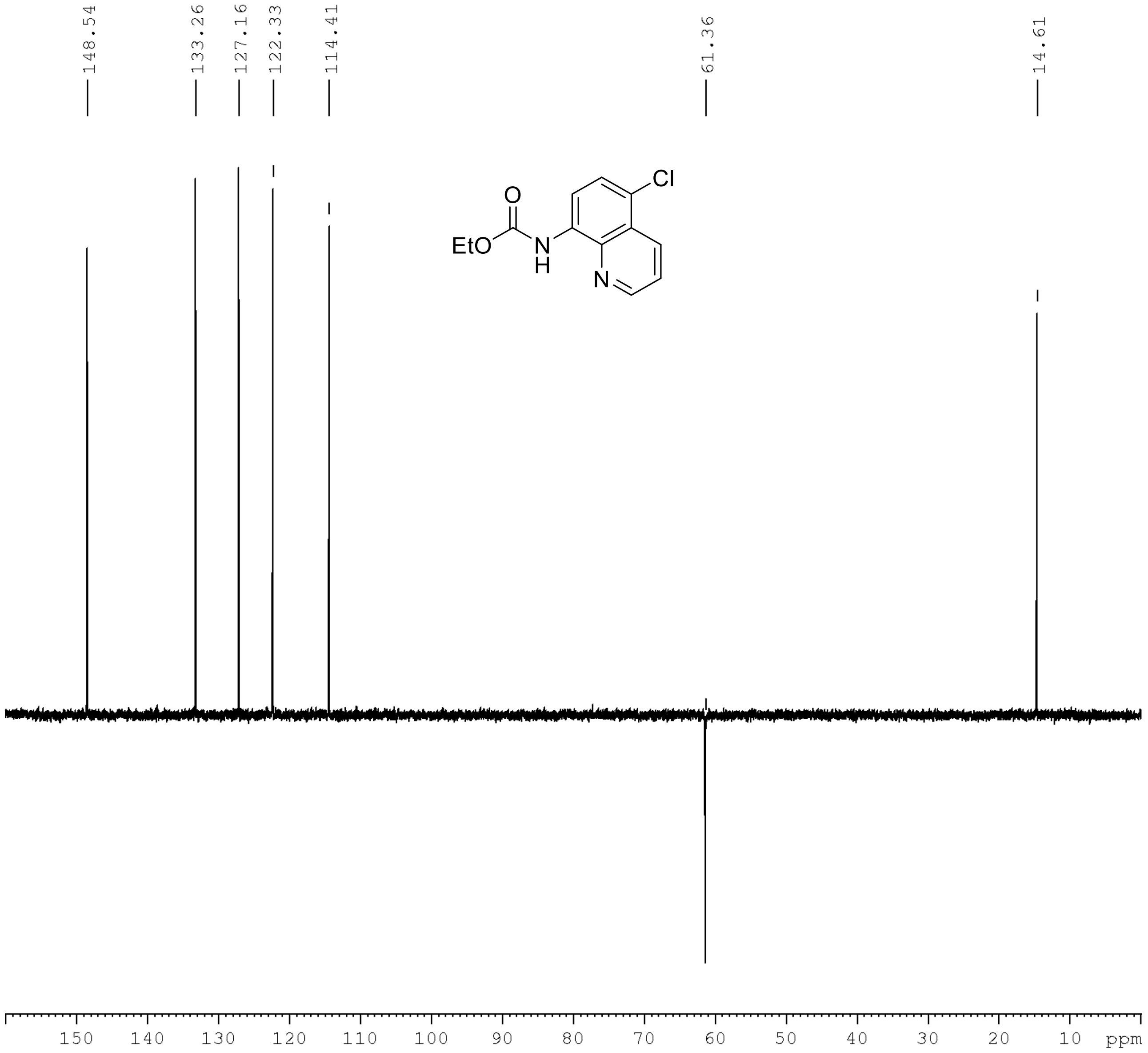

HRMS spectrum of compound **2w:**

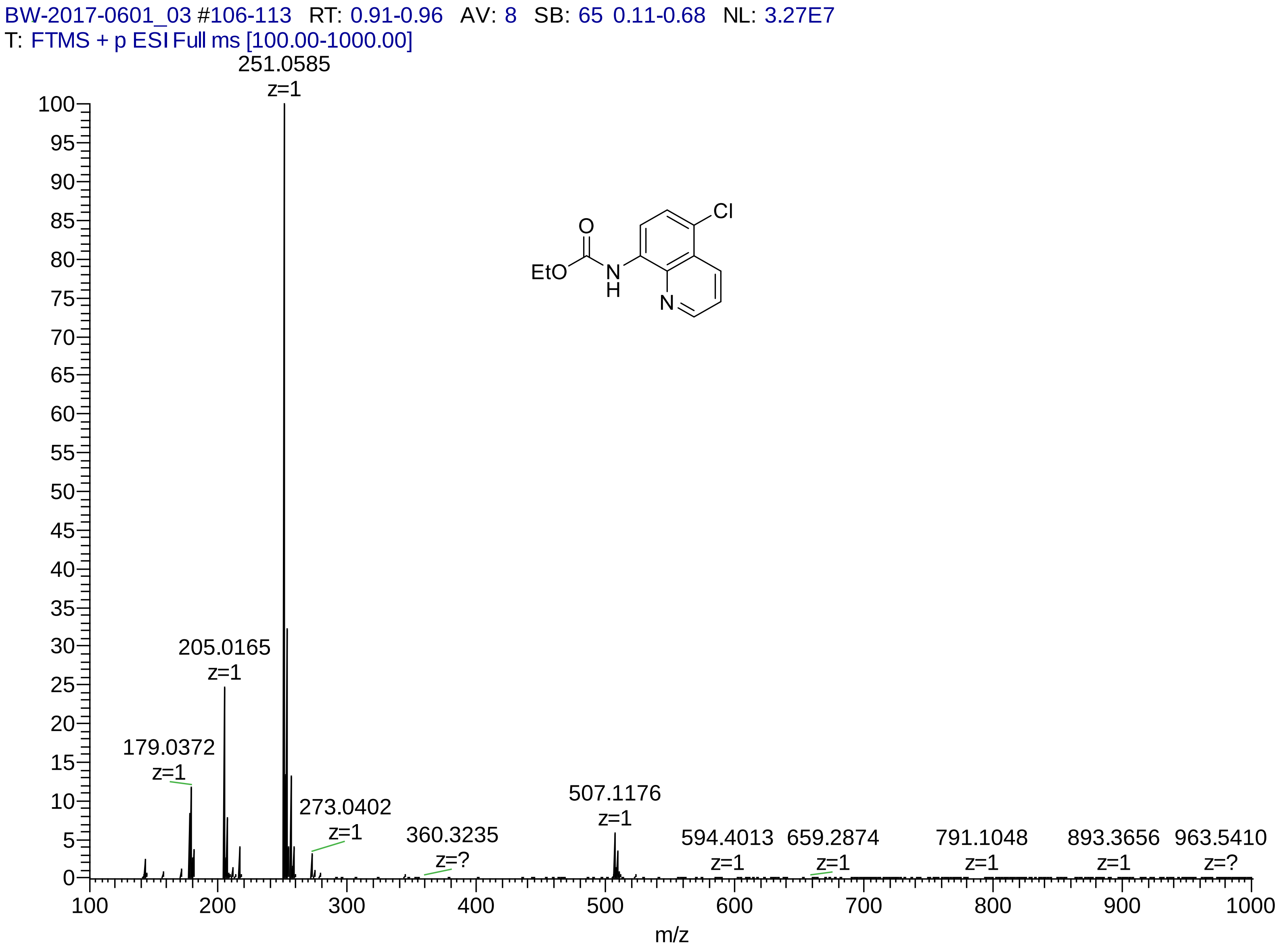

^{1}H NMR spectrum of compound **3w** ($CDCl_3$, 400 MHz)**:**

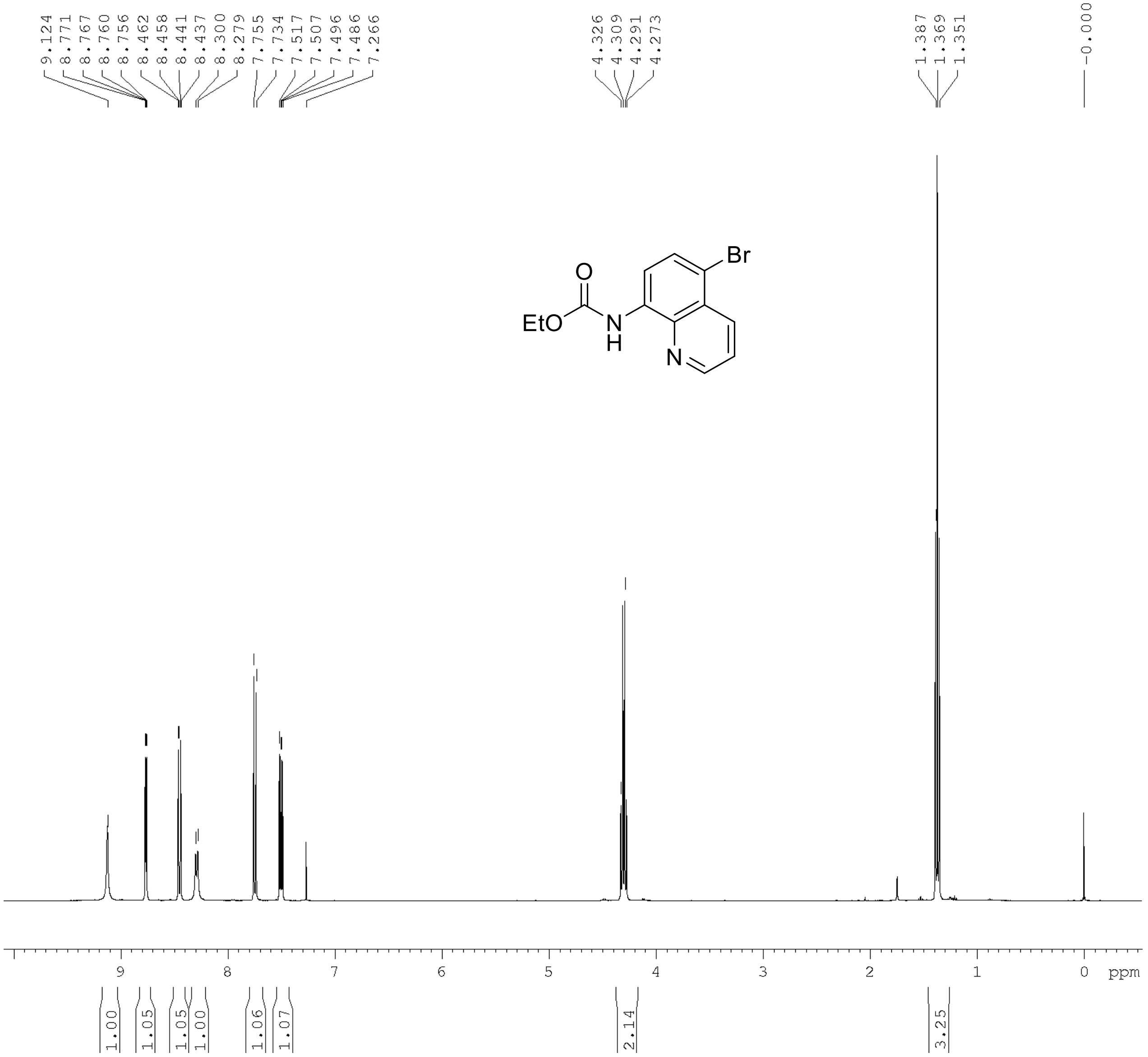

$^{13}C\{^1H\}$NMR spectrum of compound **3w** ($CDCl_3$, 100 MHz):

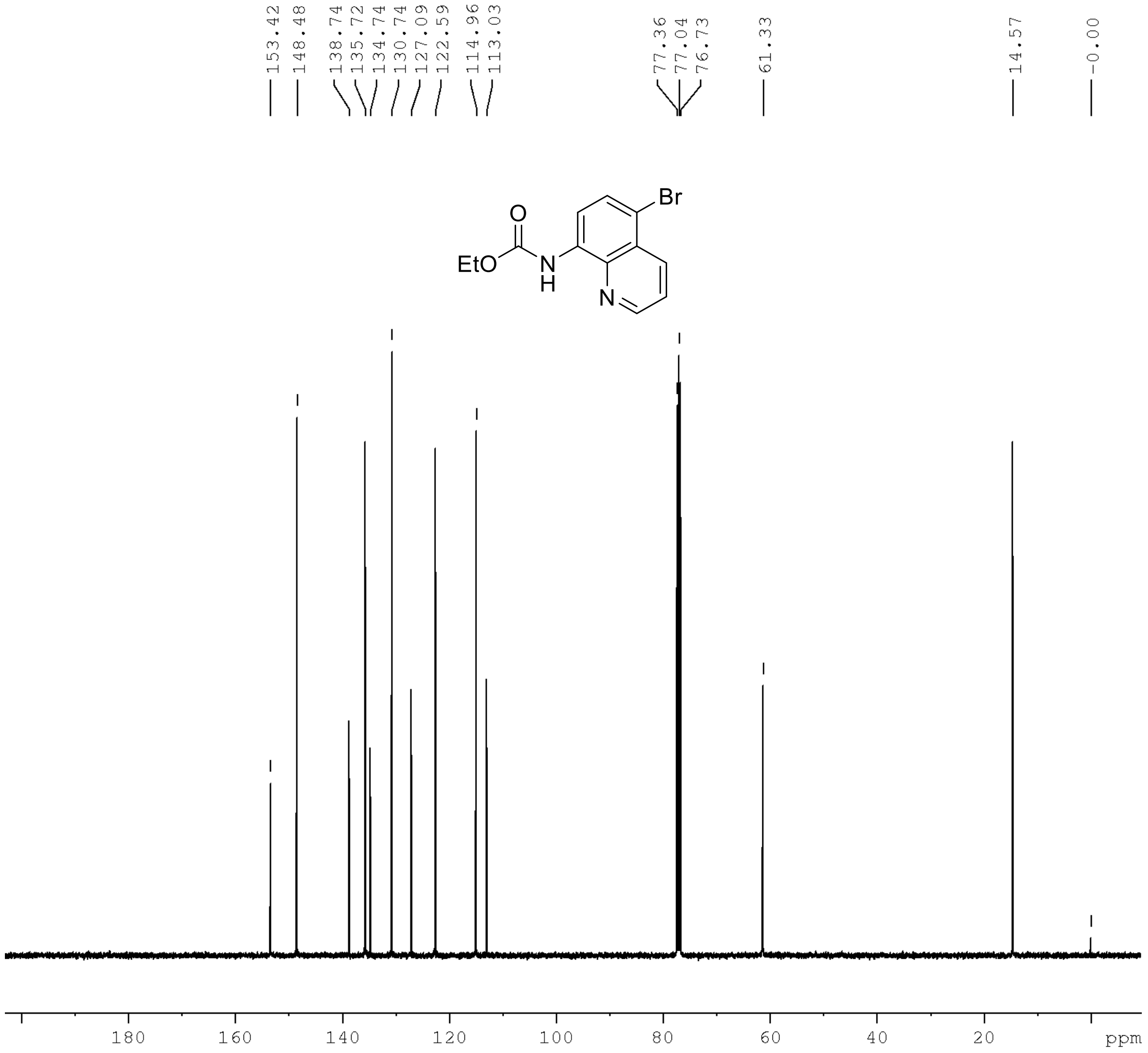

DEPT135 spectrum of compound **3w** ($CDCl_3$, 100 MHz)**:**

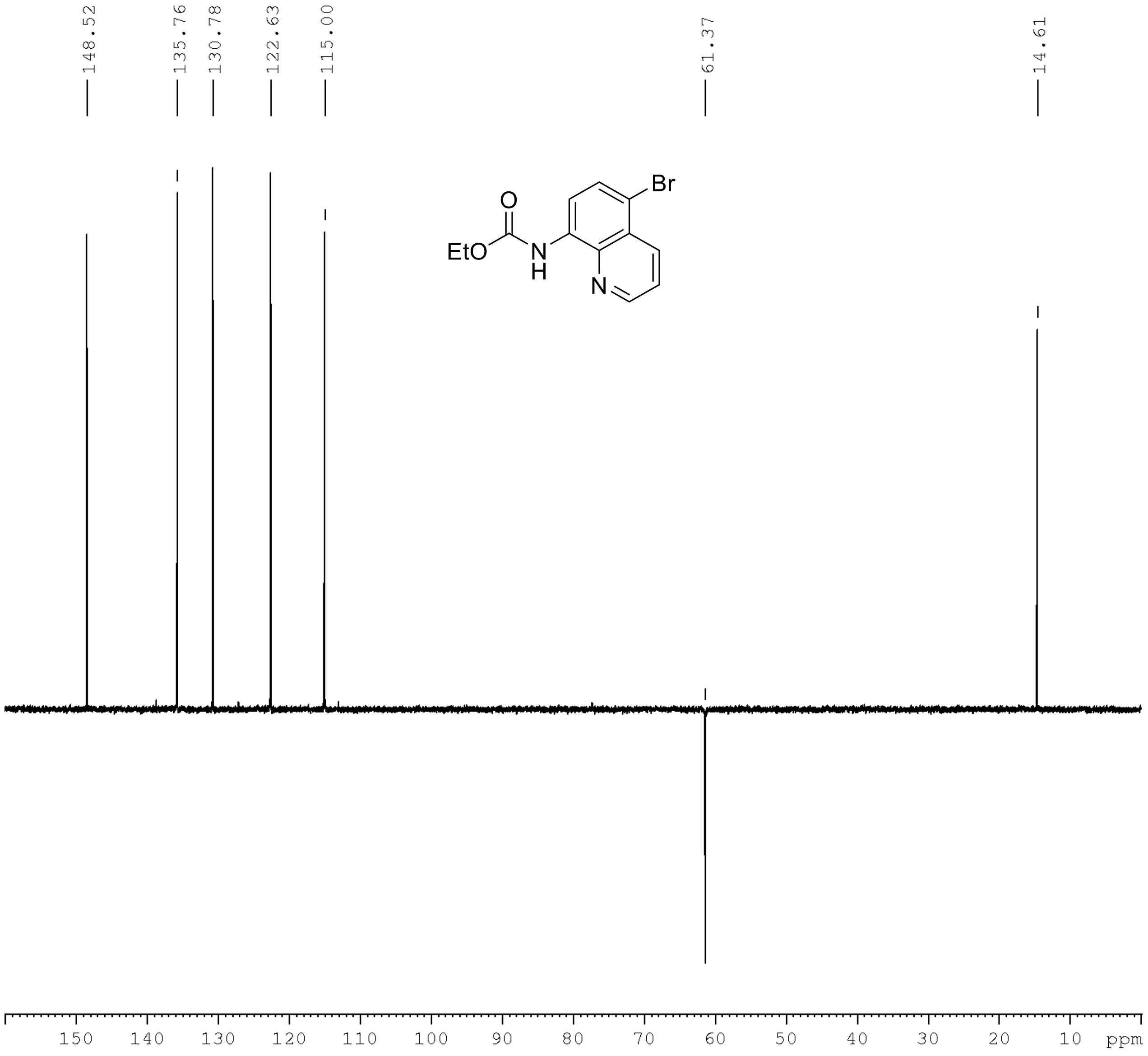

HRMS spectrum of compound **3w:**

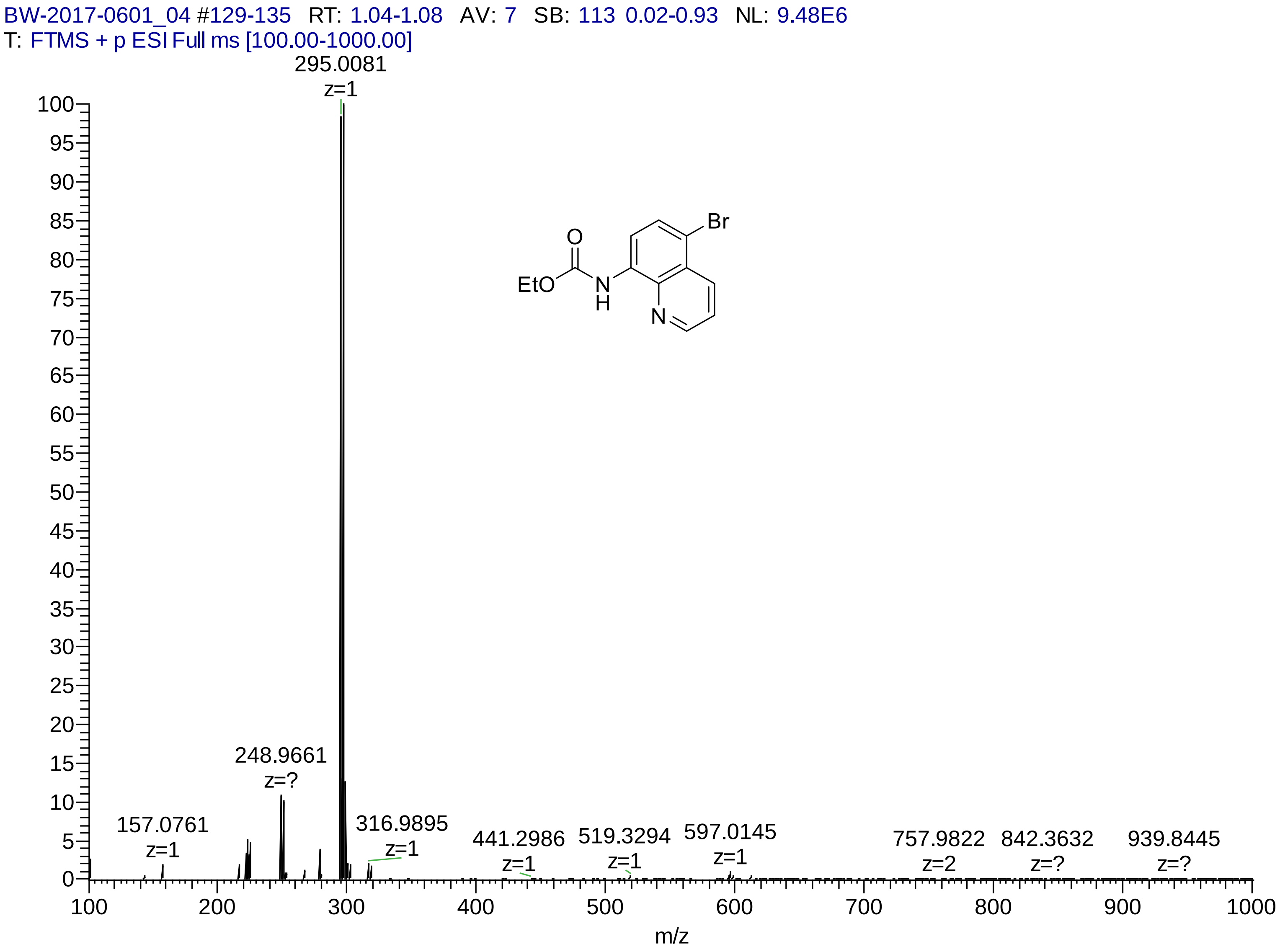

^{1}H NMR spectrum of compound **2x** ($CDCl_3$, 400 MHz)**:**

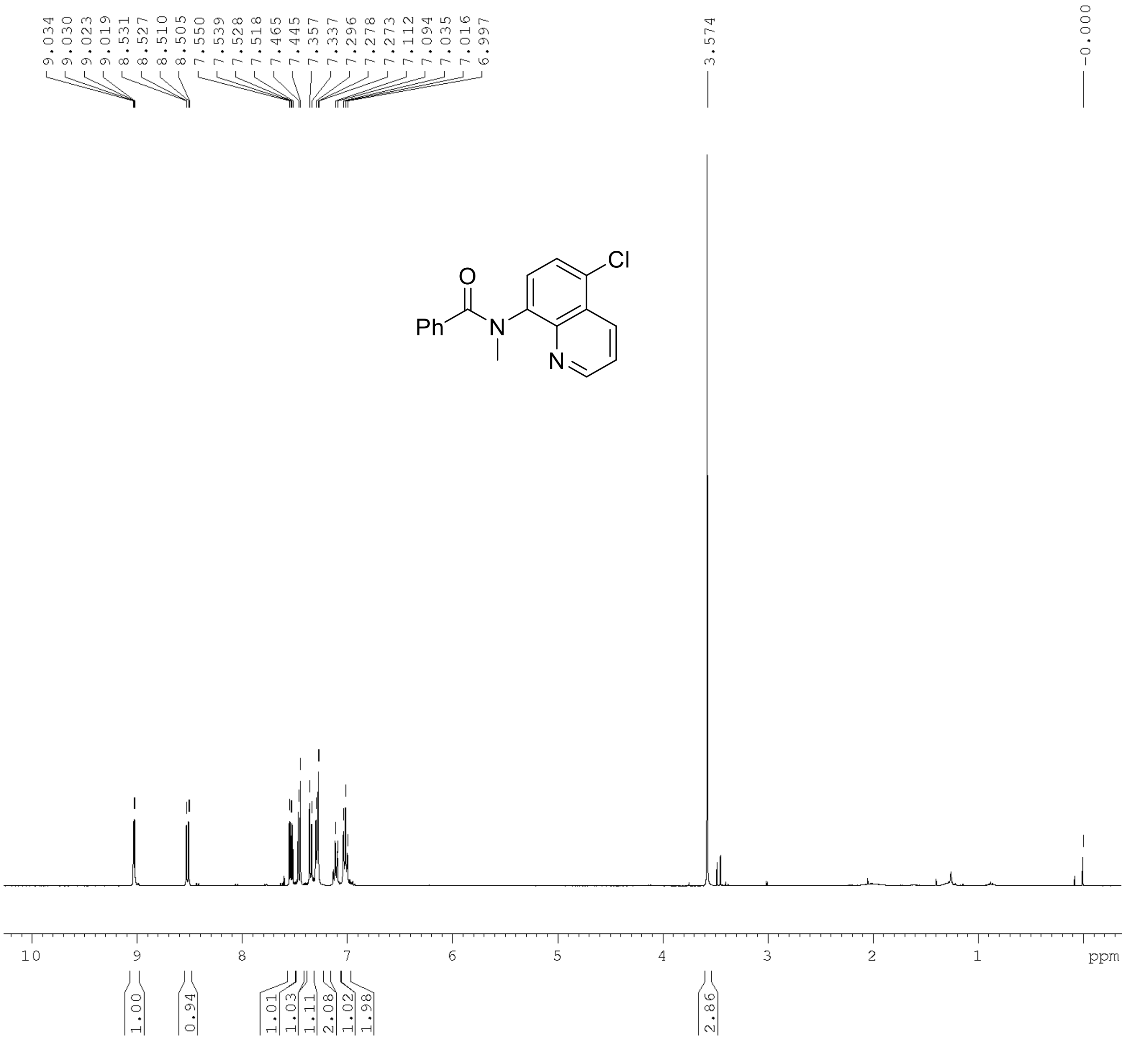

$^{13}C\{^{1}H\}$NMR spectrum of compound **2x** ($CDCl_3$, 100 MHz)**:**

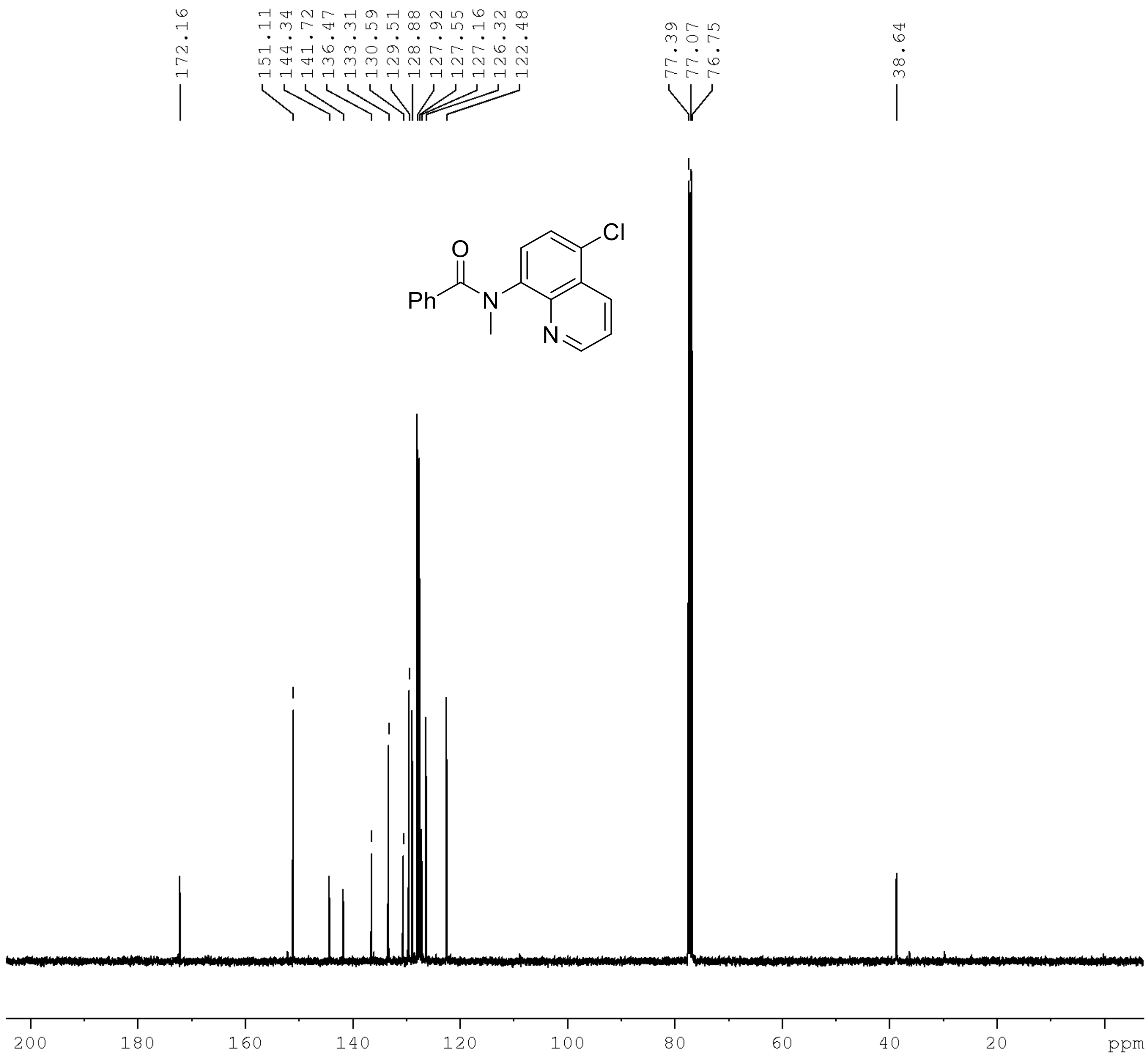

DEPT135 spectrum of compound **2x** ($CDCl_3$, 100 MHz):

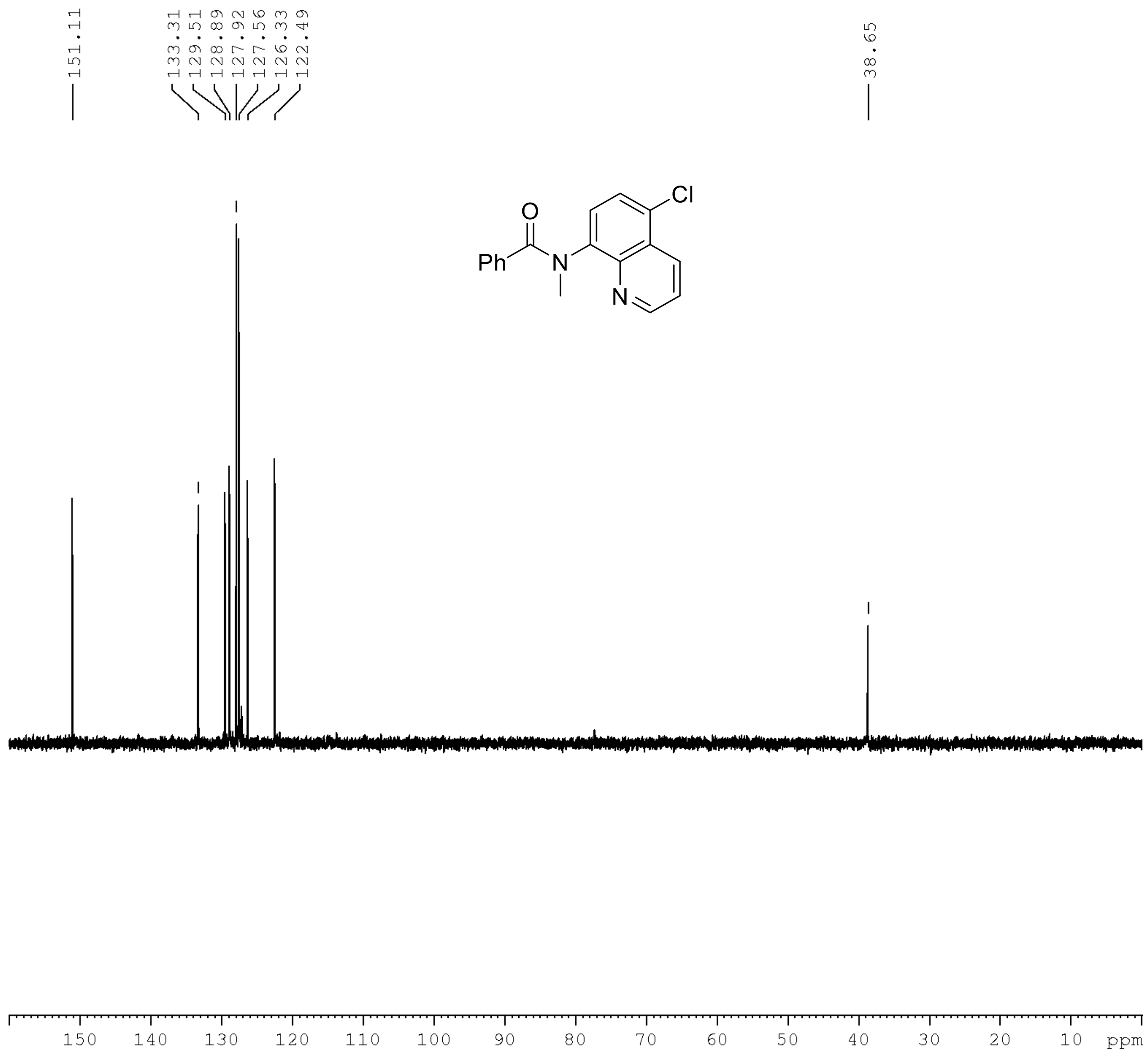

HRMS spectrum of compound **2x:**

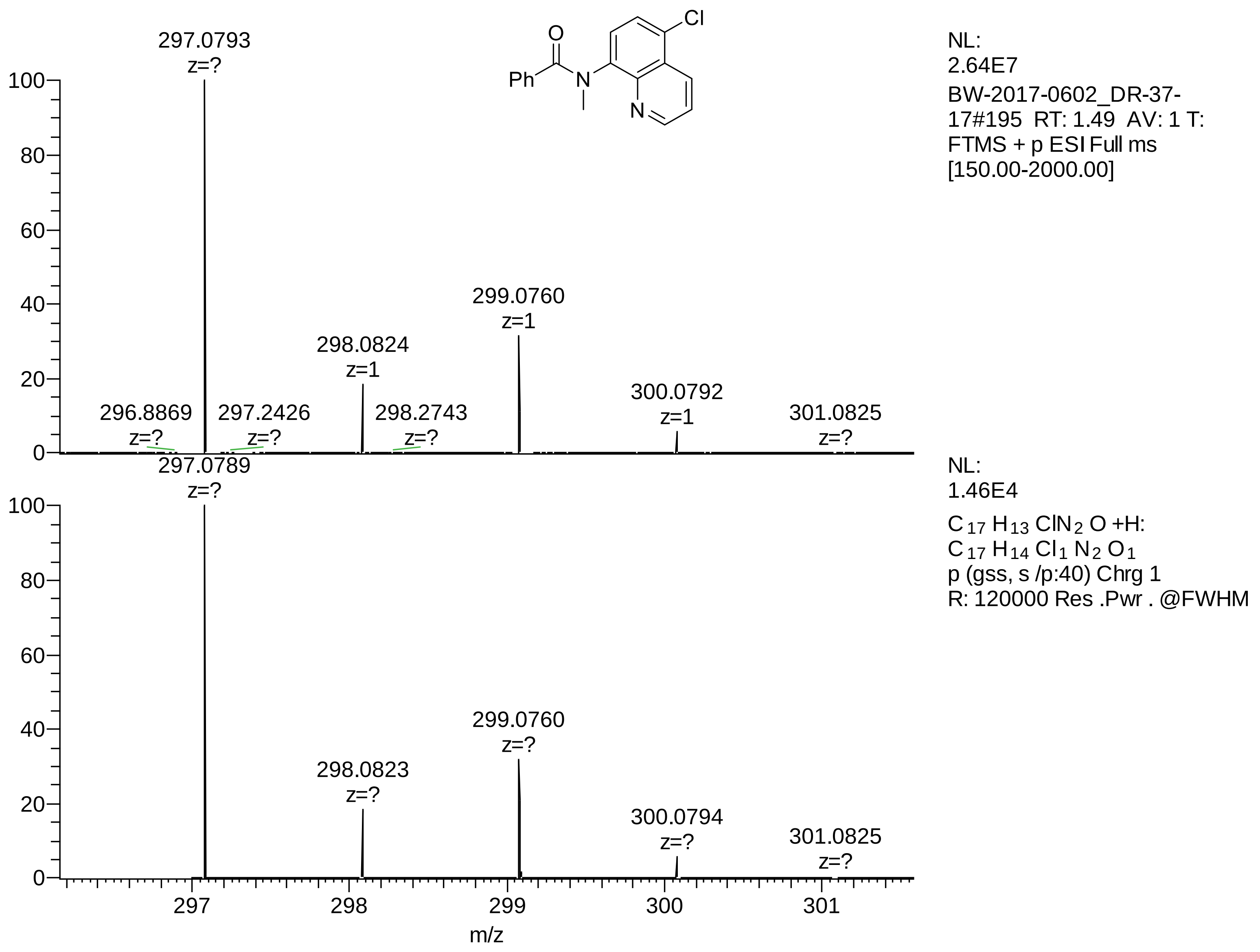

^{1}H NMR spectrum of compound **3x** ($CDCl_3$, 400 MHz)**:**

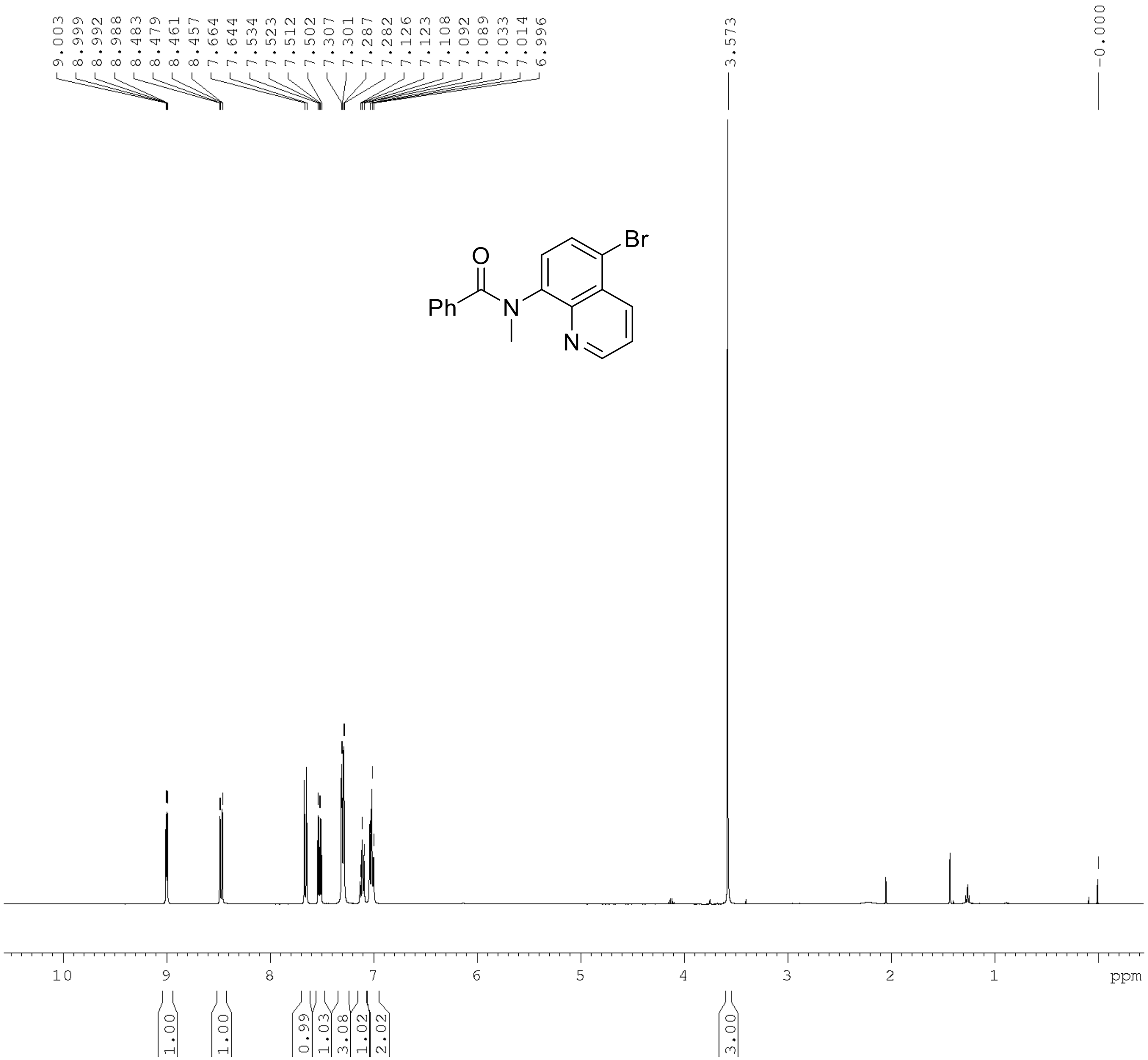

$^{13}C\{^{1}H\}$NMR spectrum of compound **3x** ($CDCl_3$, 100 MHz):

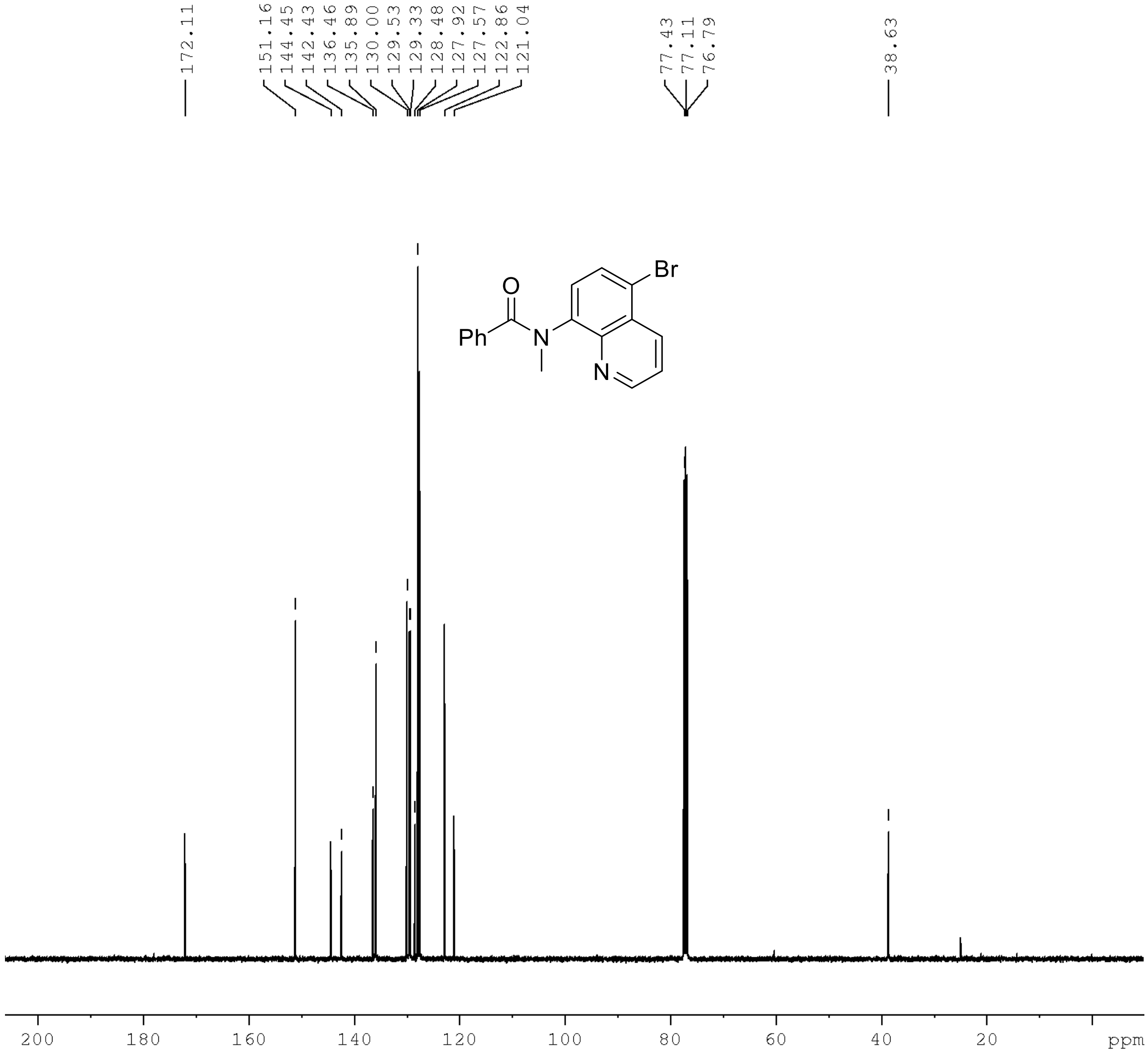

DEPT135 spectrum of compound **3x** ($CDCl_3$, 100 MHz)**:**

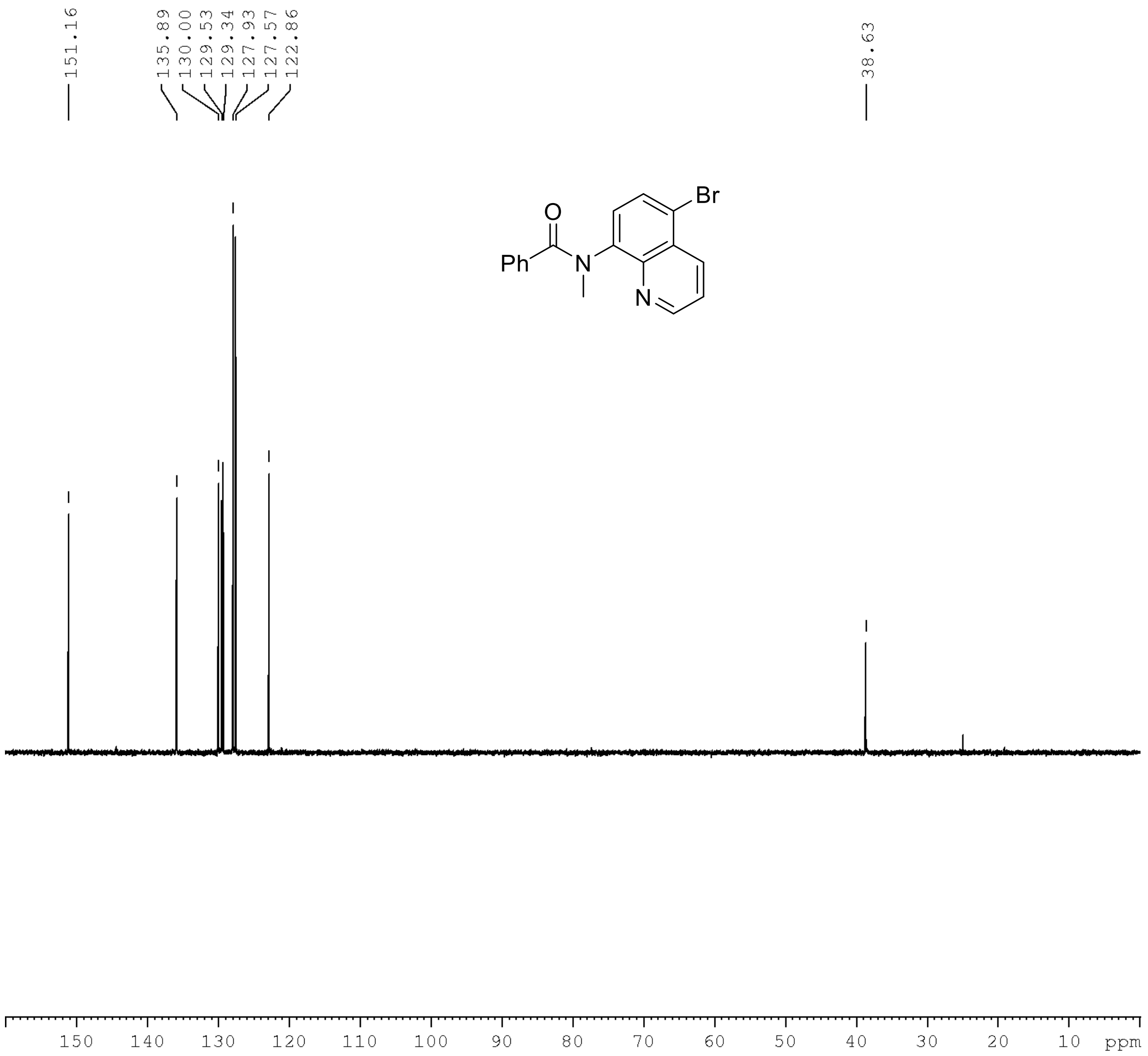

^{1}H NMR spectrum of compound **2y** ($CDCl_3$, 400 MHz)**:**

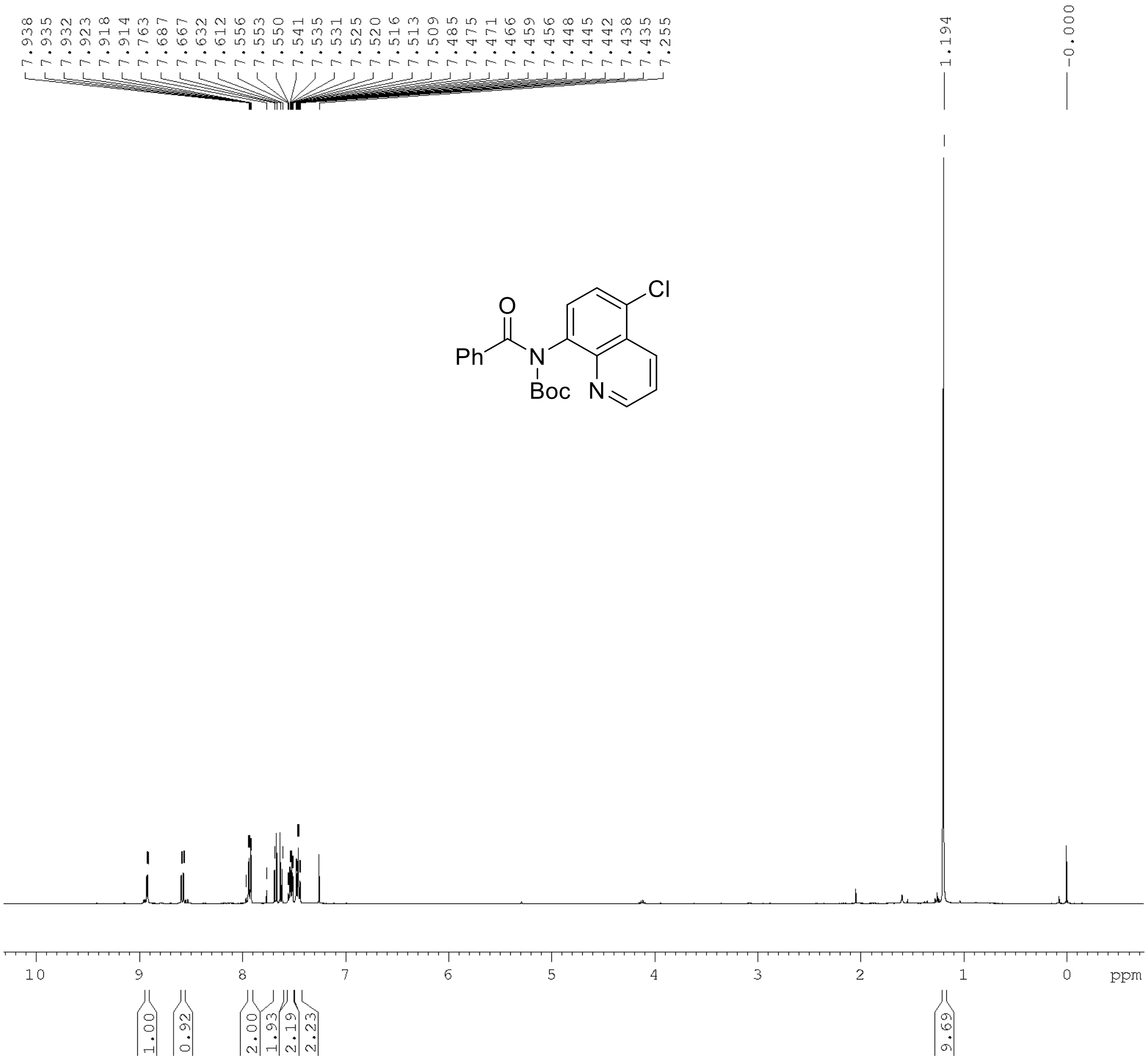

$^{13}C\{^1H\}$NMR spectrum of compound **2y** ($CDCl_3$, 100 MHz)**:**

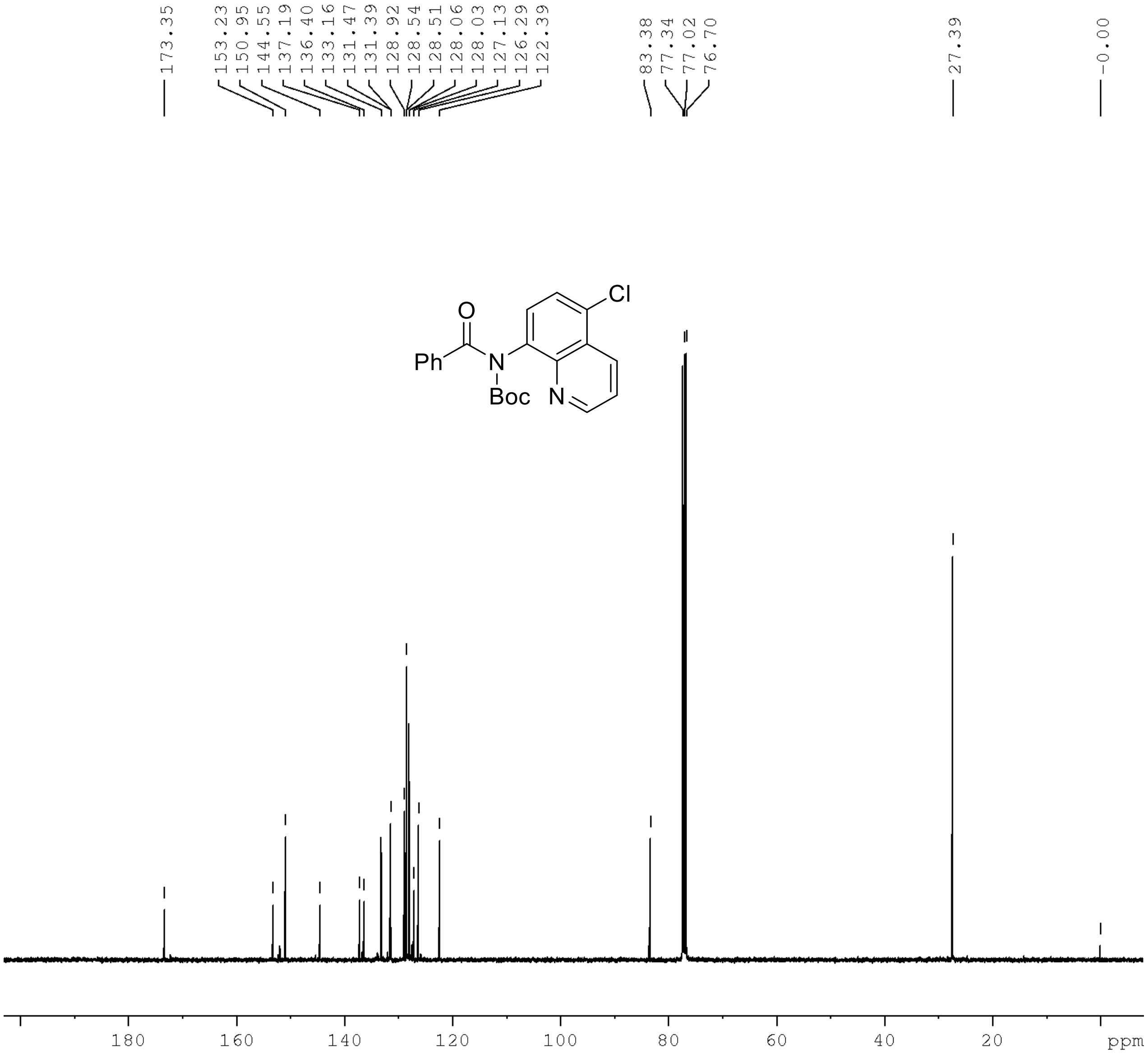

DEPT135 spectrum of compound **2y** ($CDCl_3$, 100 MHz)**:**

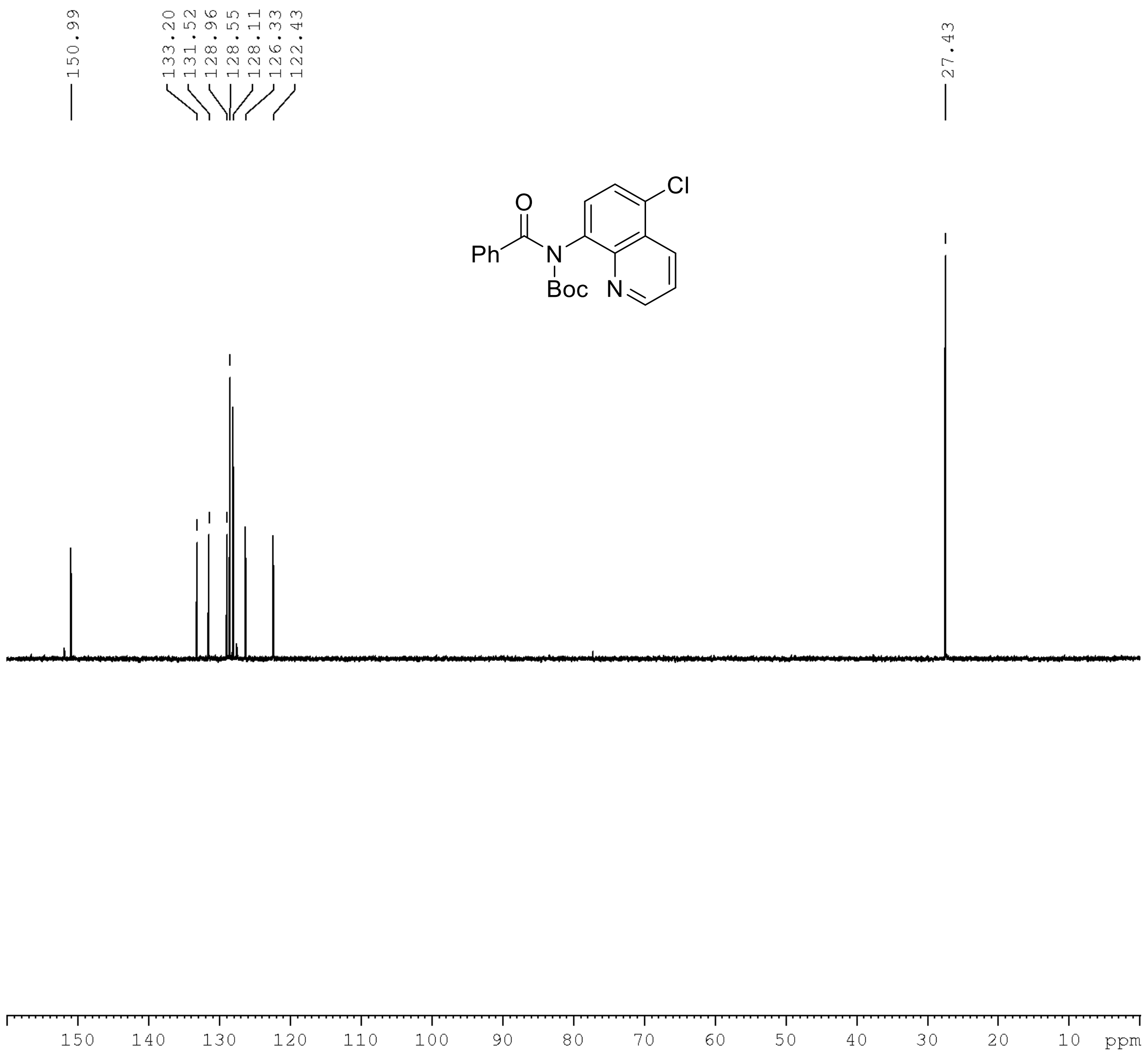

HRMS spectrum of compound **2y:**

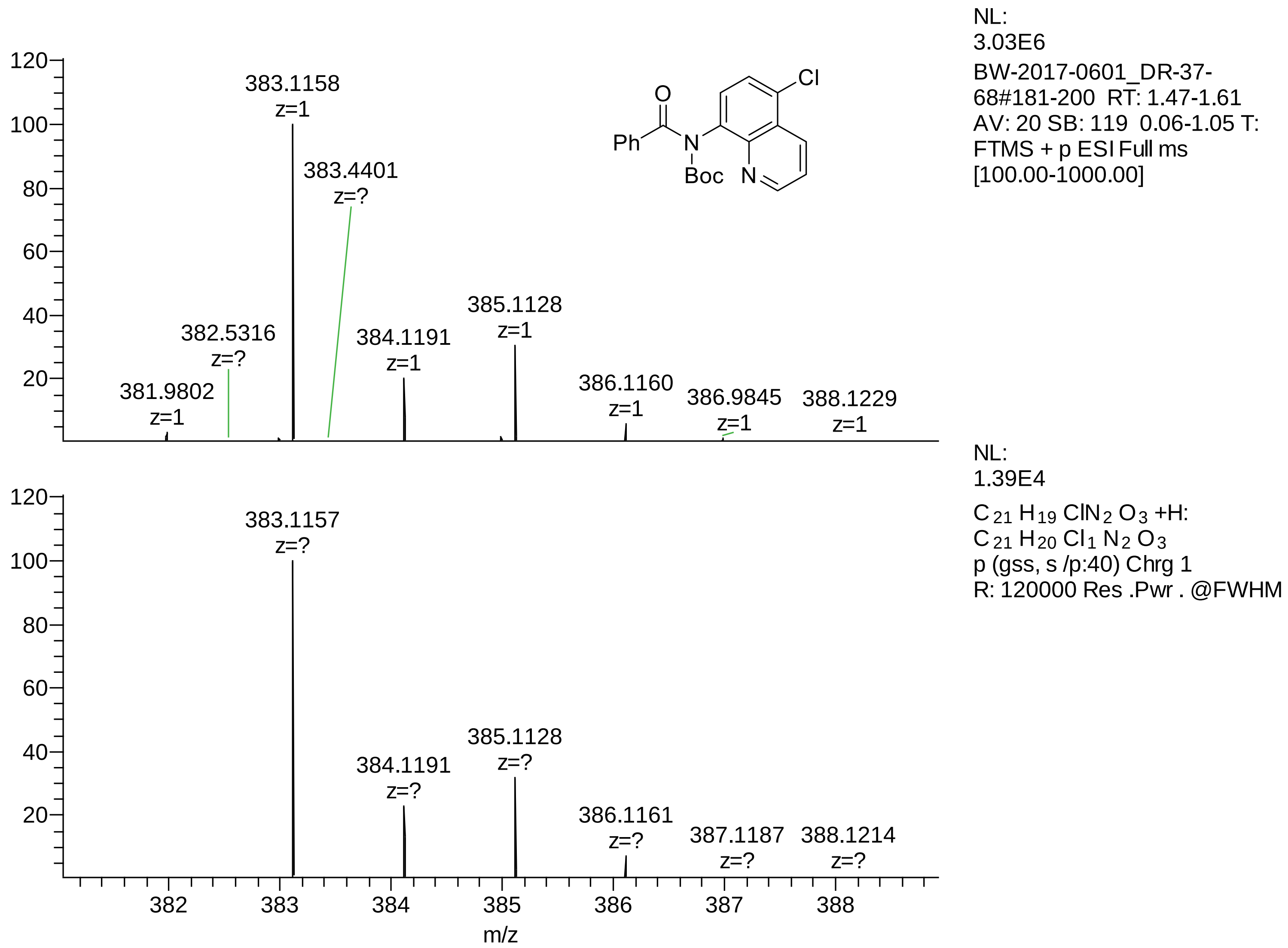

^{1}H NMR spectrum of compound **3y** ($CDCl_3$, 400 MHz)**:**

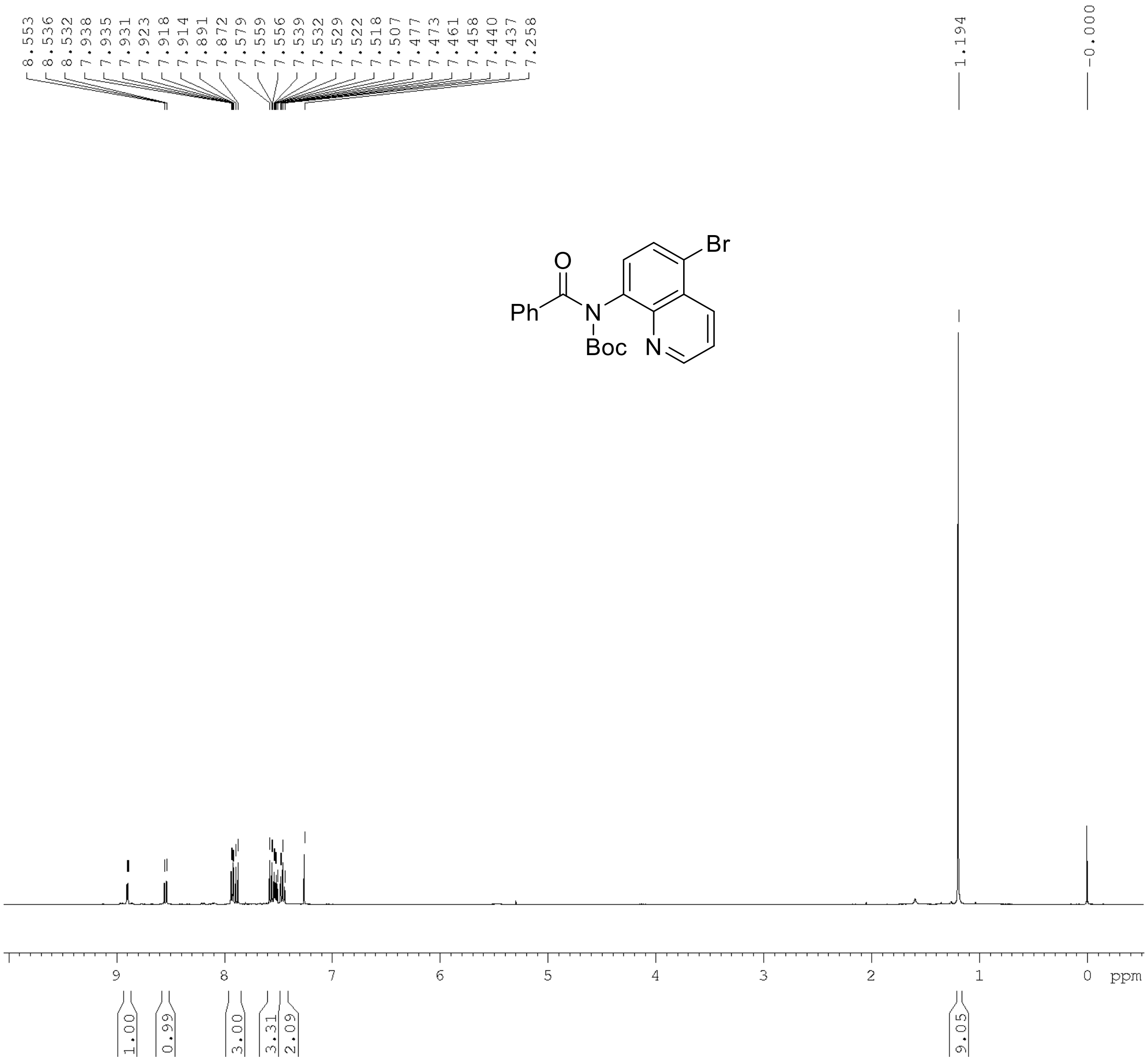

$^{13}C\{^{1}H\}$NMR spectrum of compound **3y** ($CDCl_3$, 100 MHz)**:**

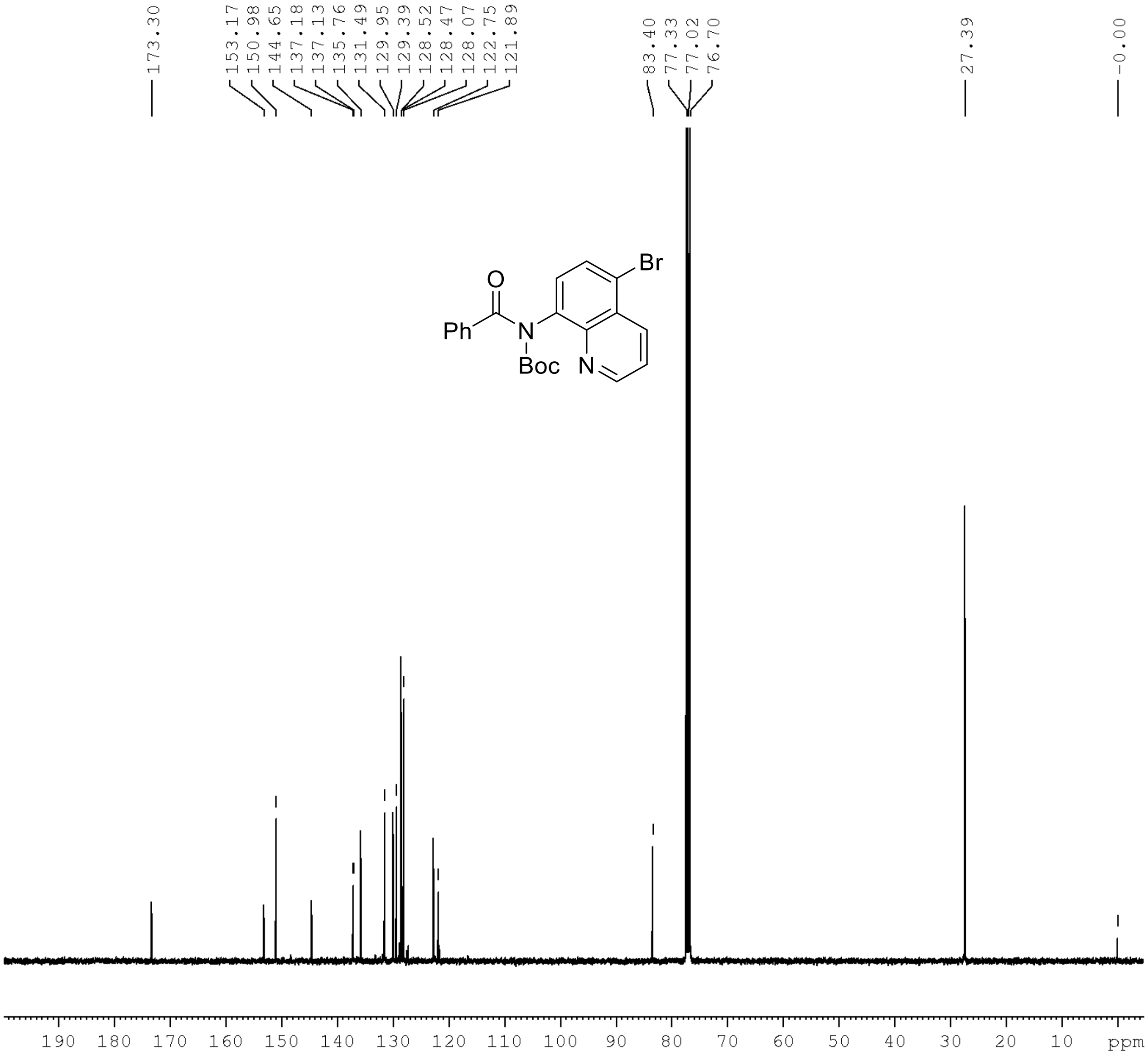

DEPT135 spectrum of compound **3y** ($CDCl_3$, 100 MHz)**:**

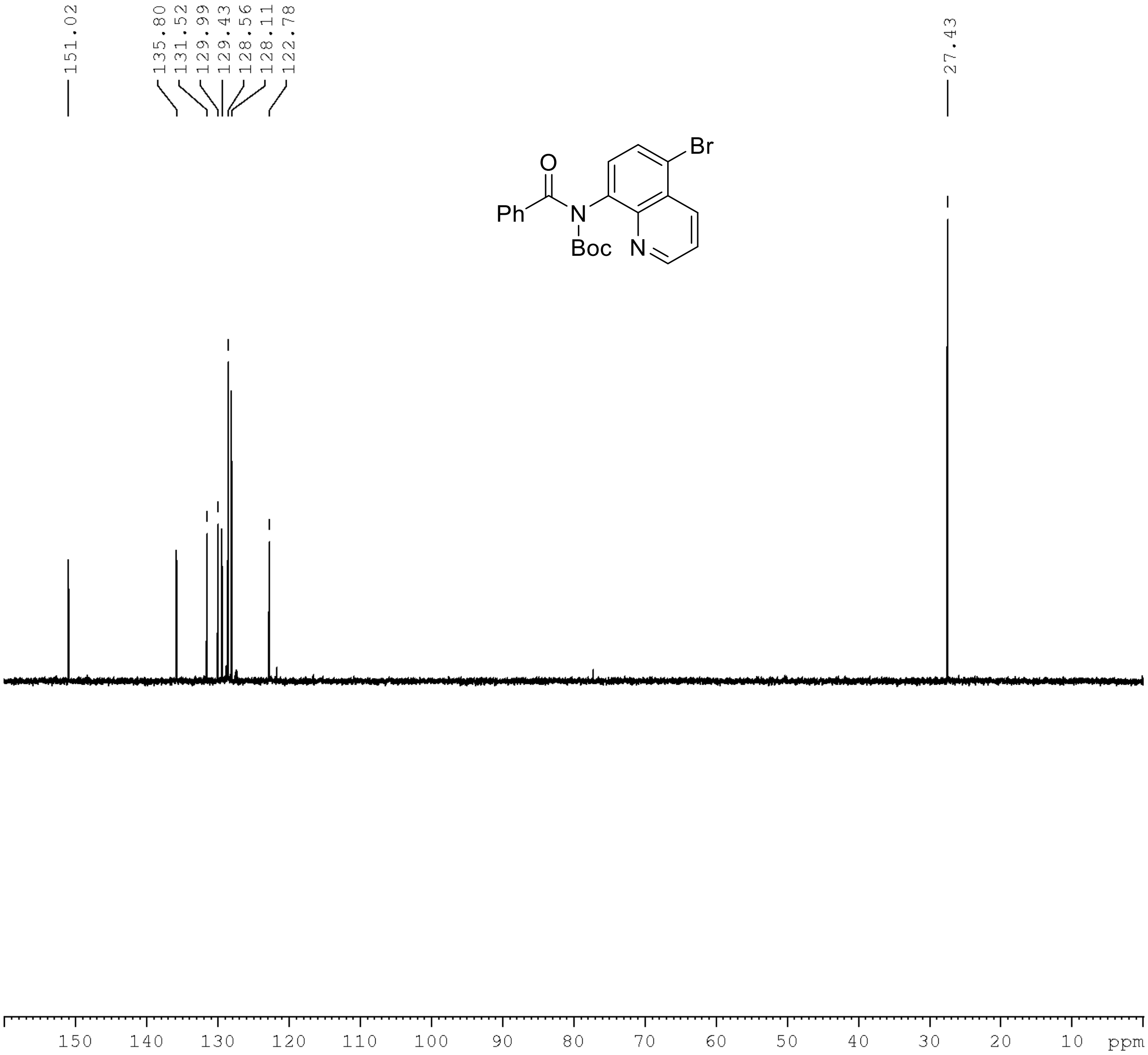

HRMS spectrum of compound **3y:**

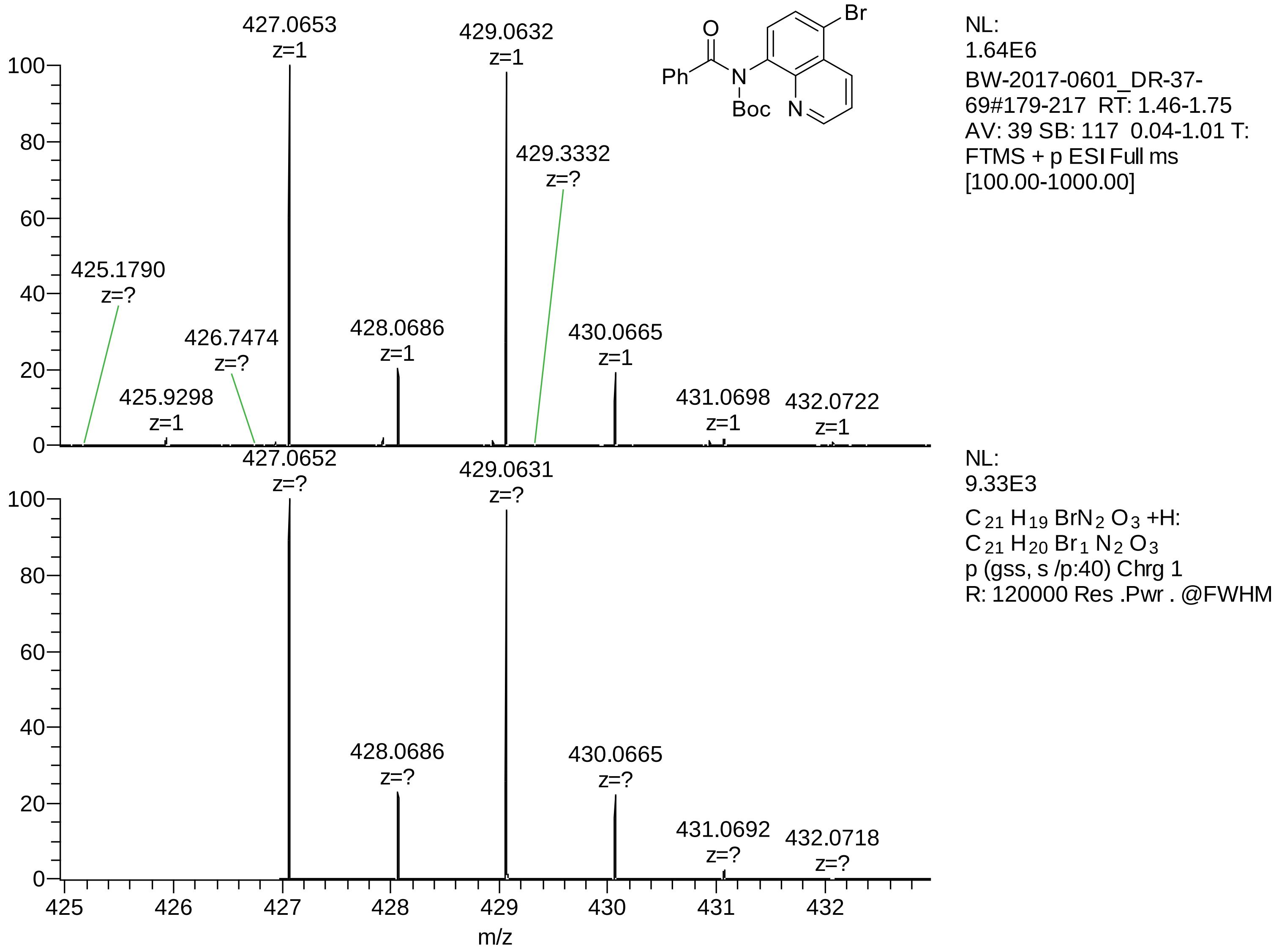

^{1}H NMR spectrum of compound **2z** ($CDCl_3$, 400 MHz)**:**

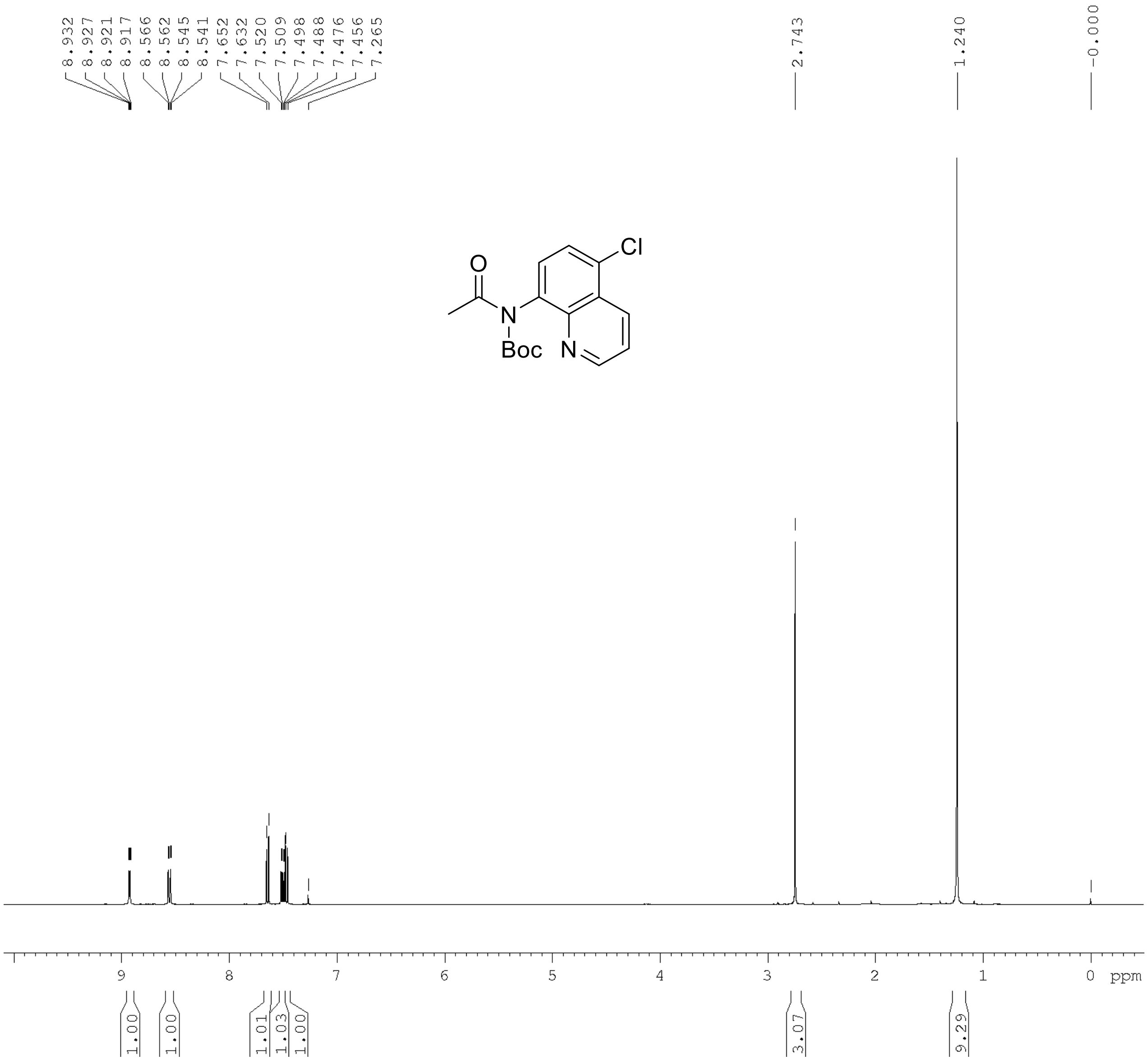

$^{13}C\{^1H\}$NMR spectrum of compound **2z** ($CDCl_3$, 100 MHz)**:**

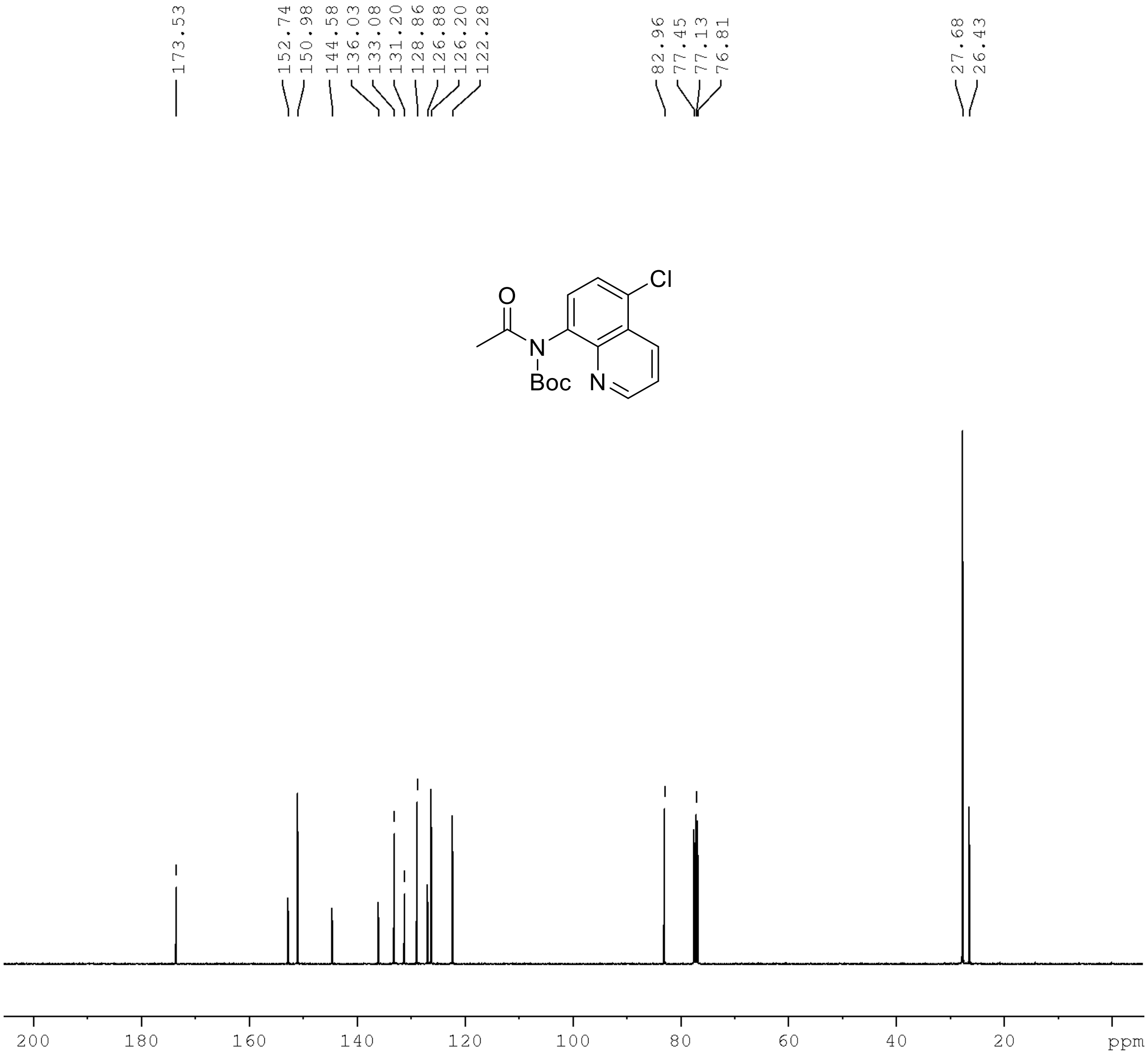

DEPT135 spectrum of compound **2z** ($CDCl_3$, 100 MHz)**:**

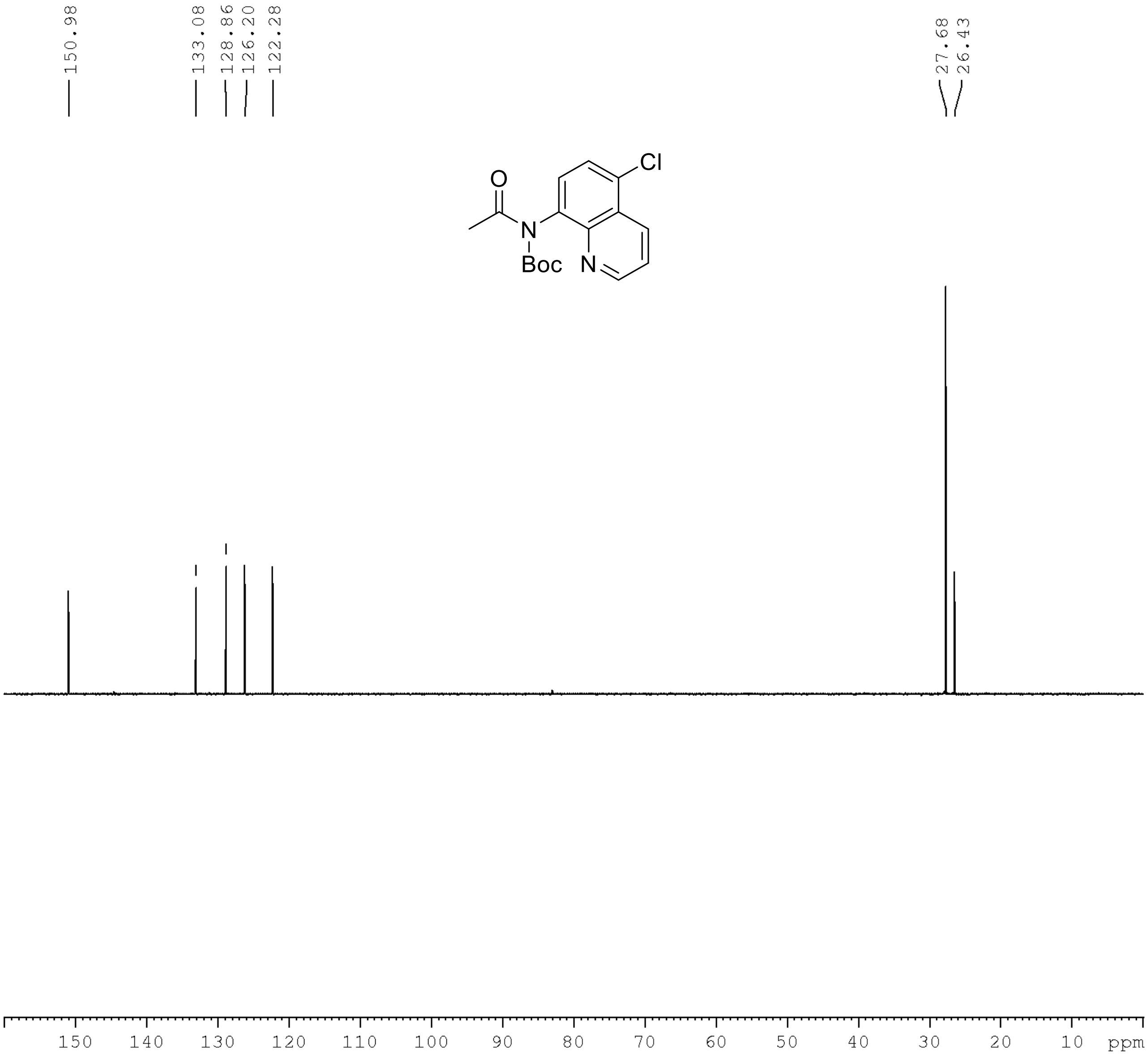

^{1}H NMR spectrum of compound **3z** ($CDCl_3$, 400 MHz)**:**

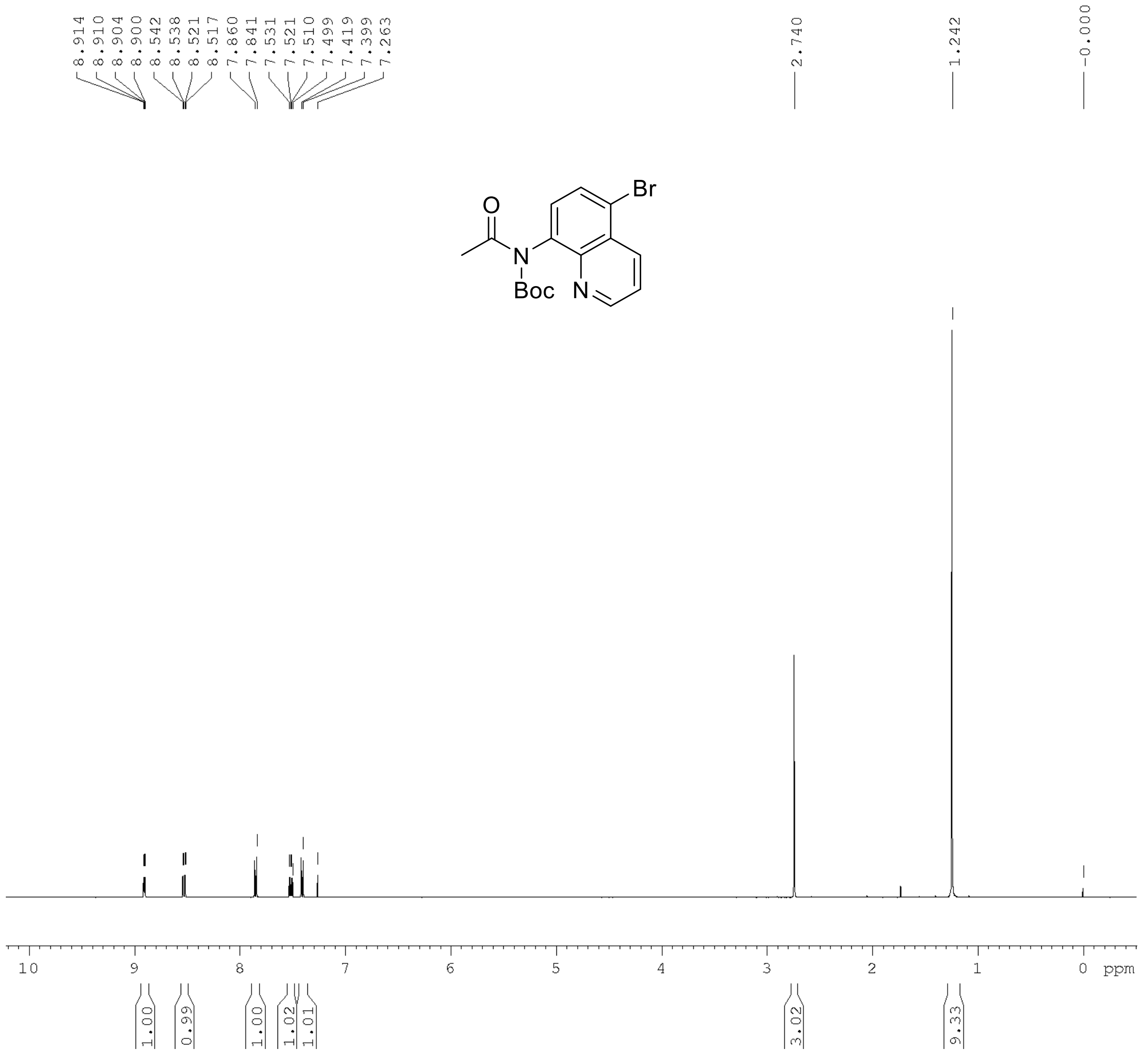

^{13}C{^{1}H}NMR spectrum of compound **3z** ($CDCl_3$, 100 MHz)**:**

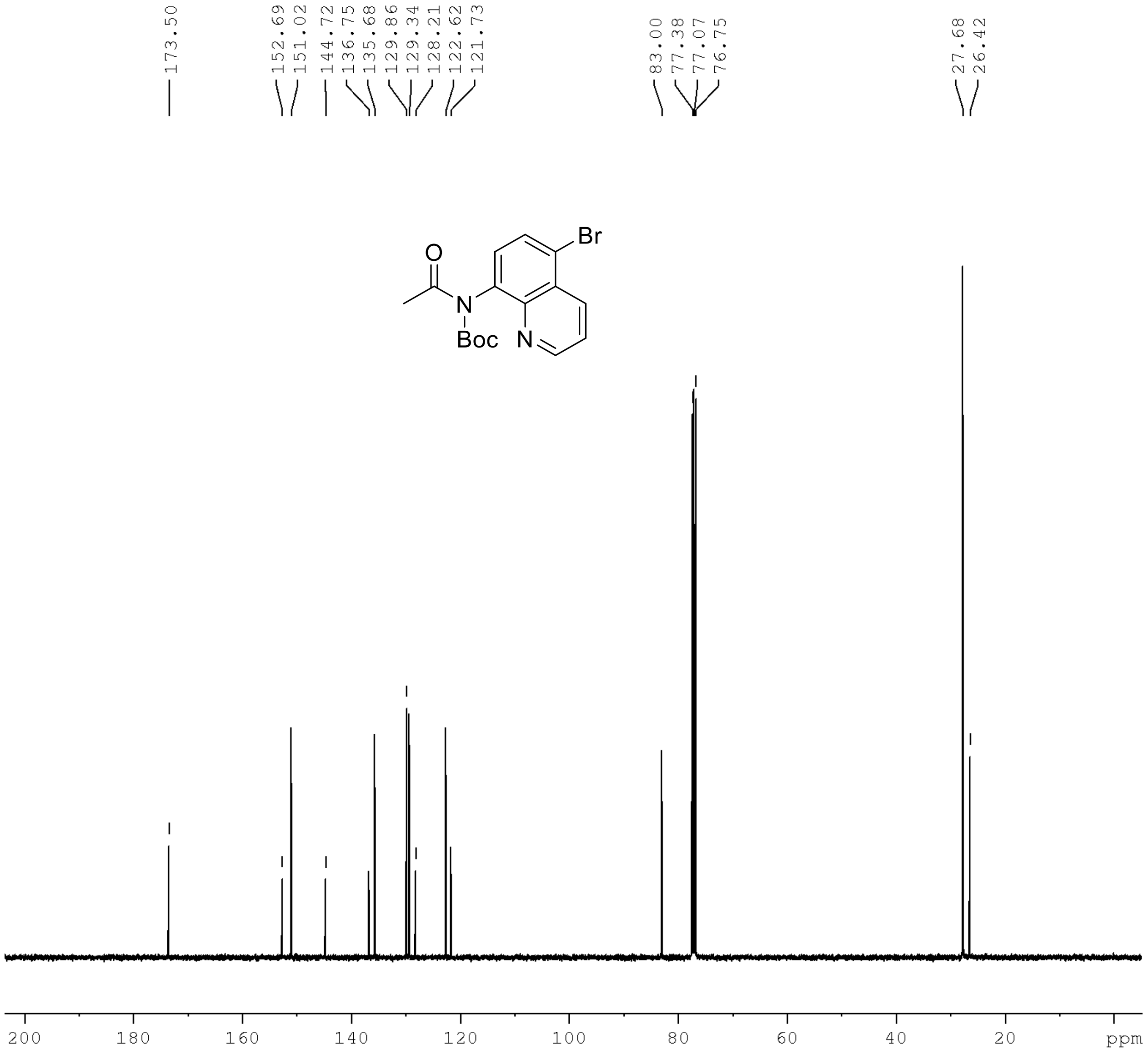

DEPT135 spectrum of compound **3z** ($CDCl_3$, 100 MHz)**:**

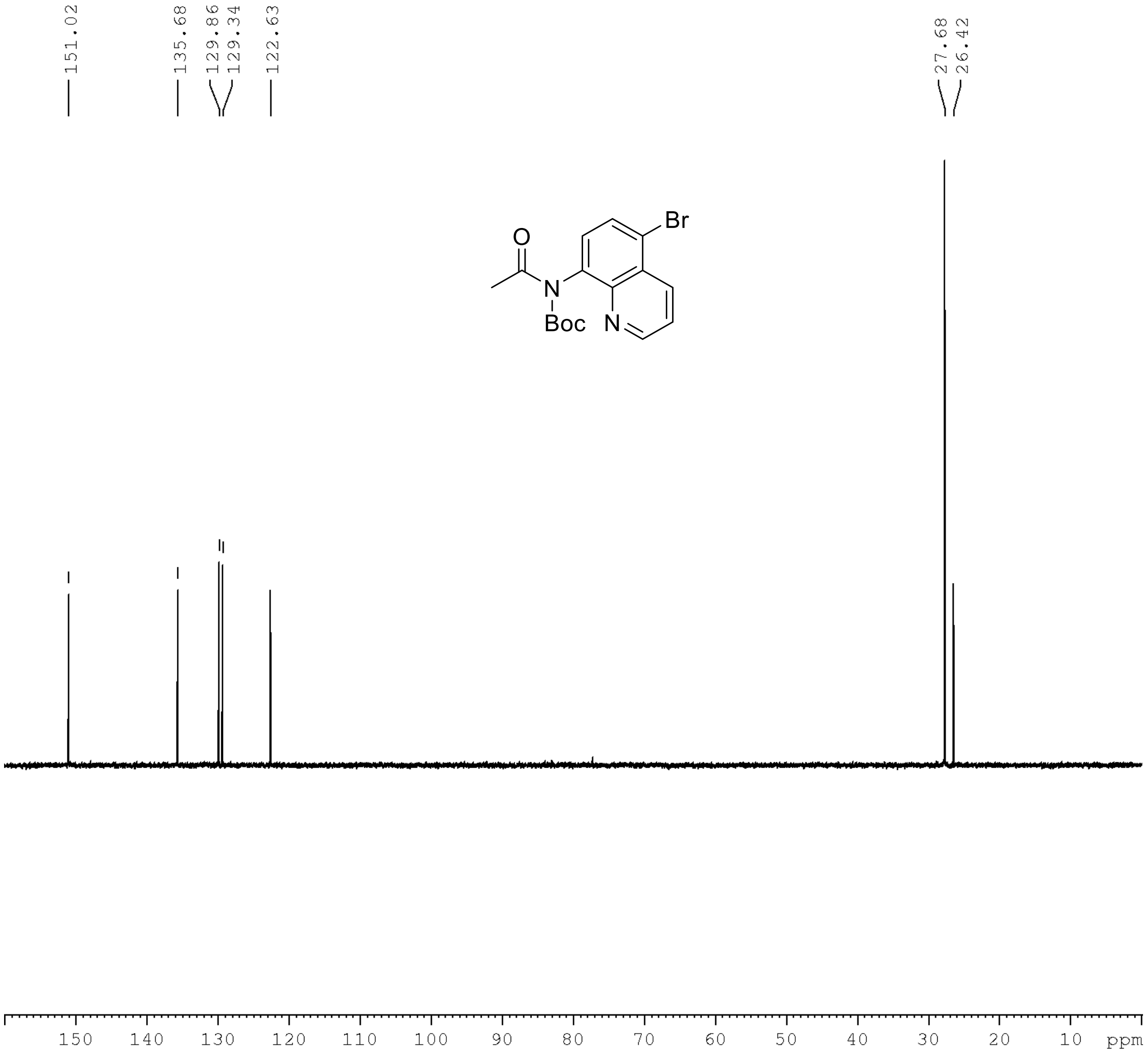

^{1}H NMR spectrum of compound **4a** ($CDCl_3$, 400 MHz)**:**

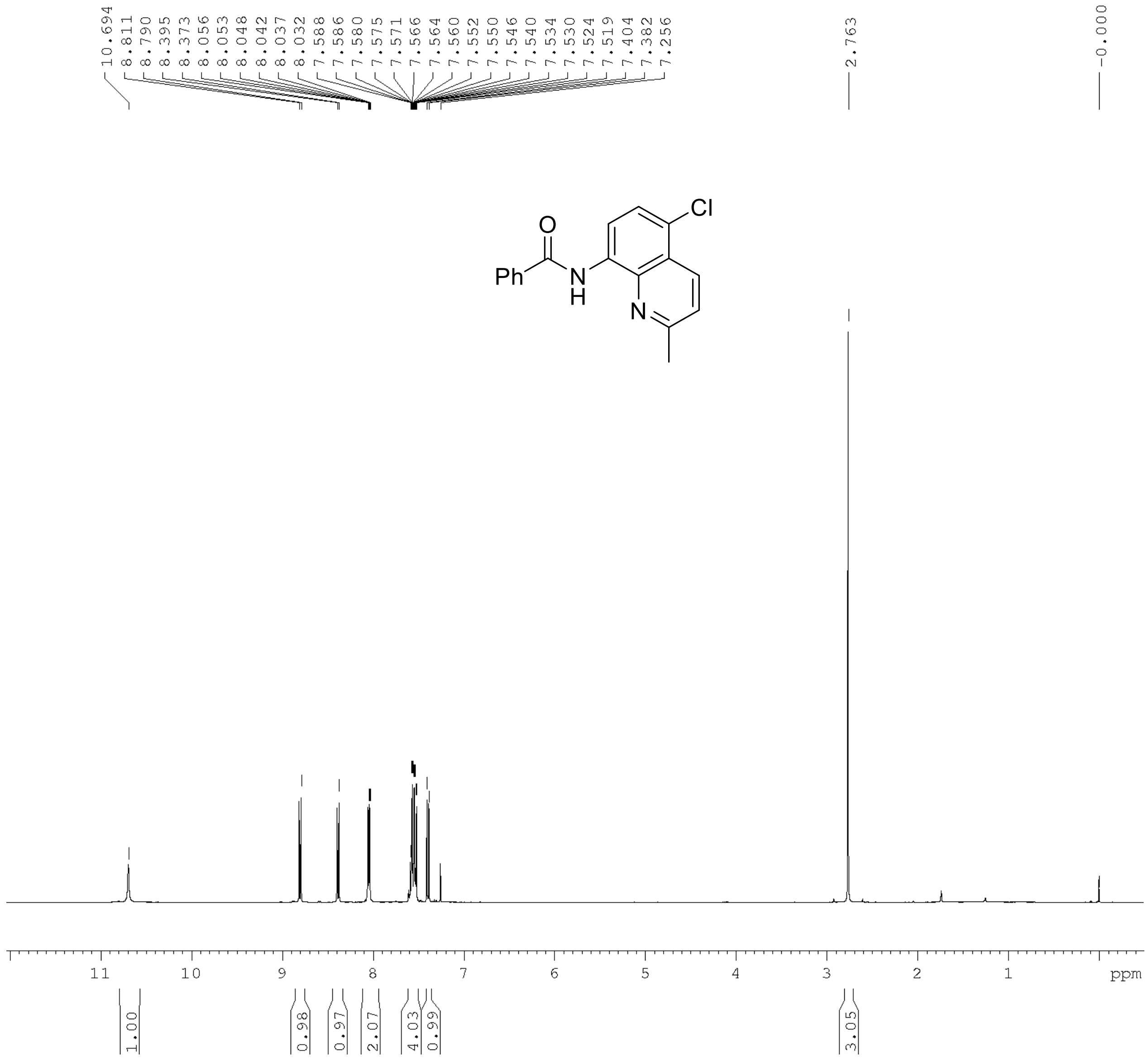

$^{13}C\{^{1}H\}$NMR spectrum of compound **4a** ($CDCl_3$, 100 MHz)**:**

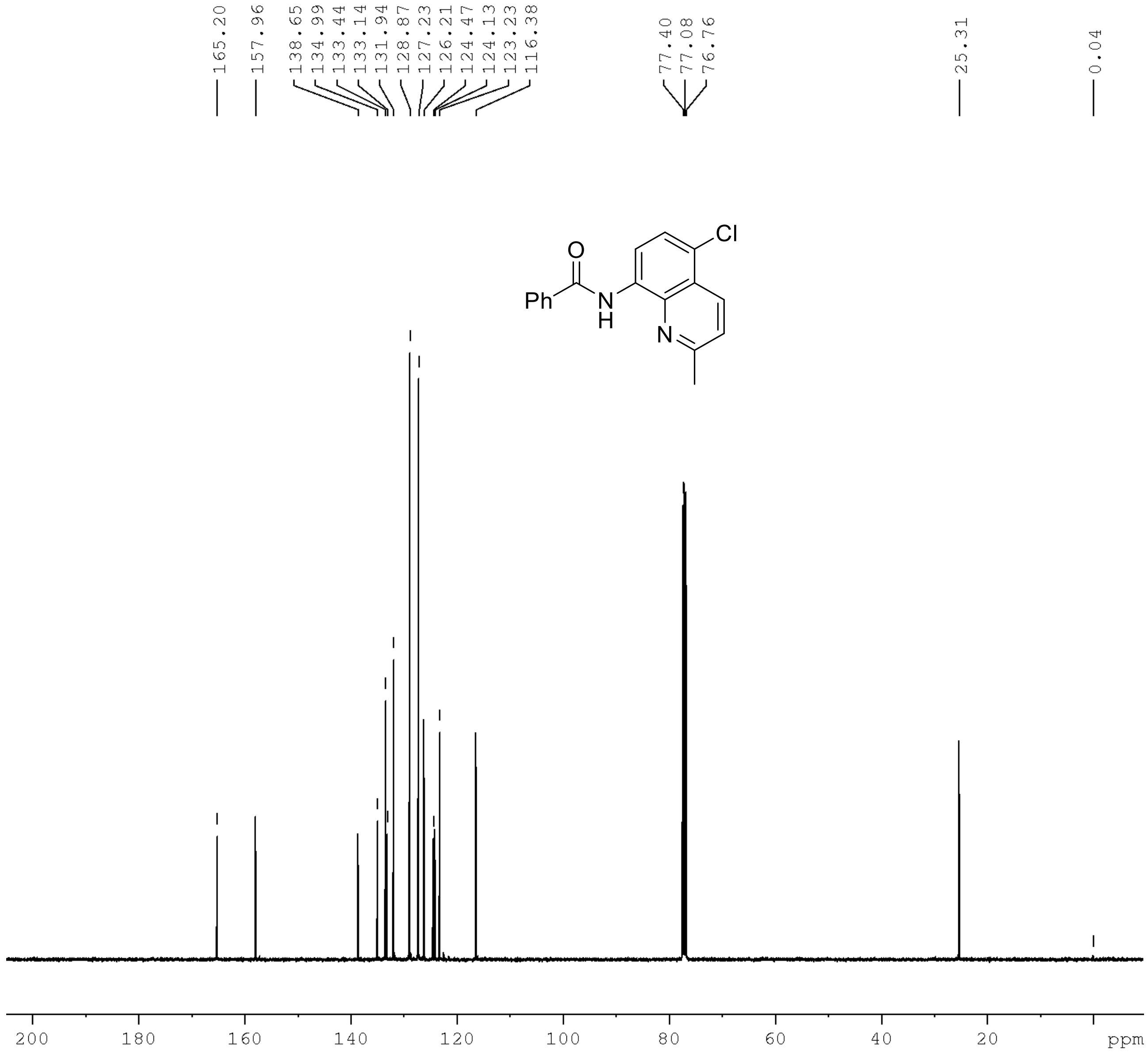

^{1}H NMR spectrum of compound **5a** ($CDCl_3$, 400 MHz)**:**

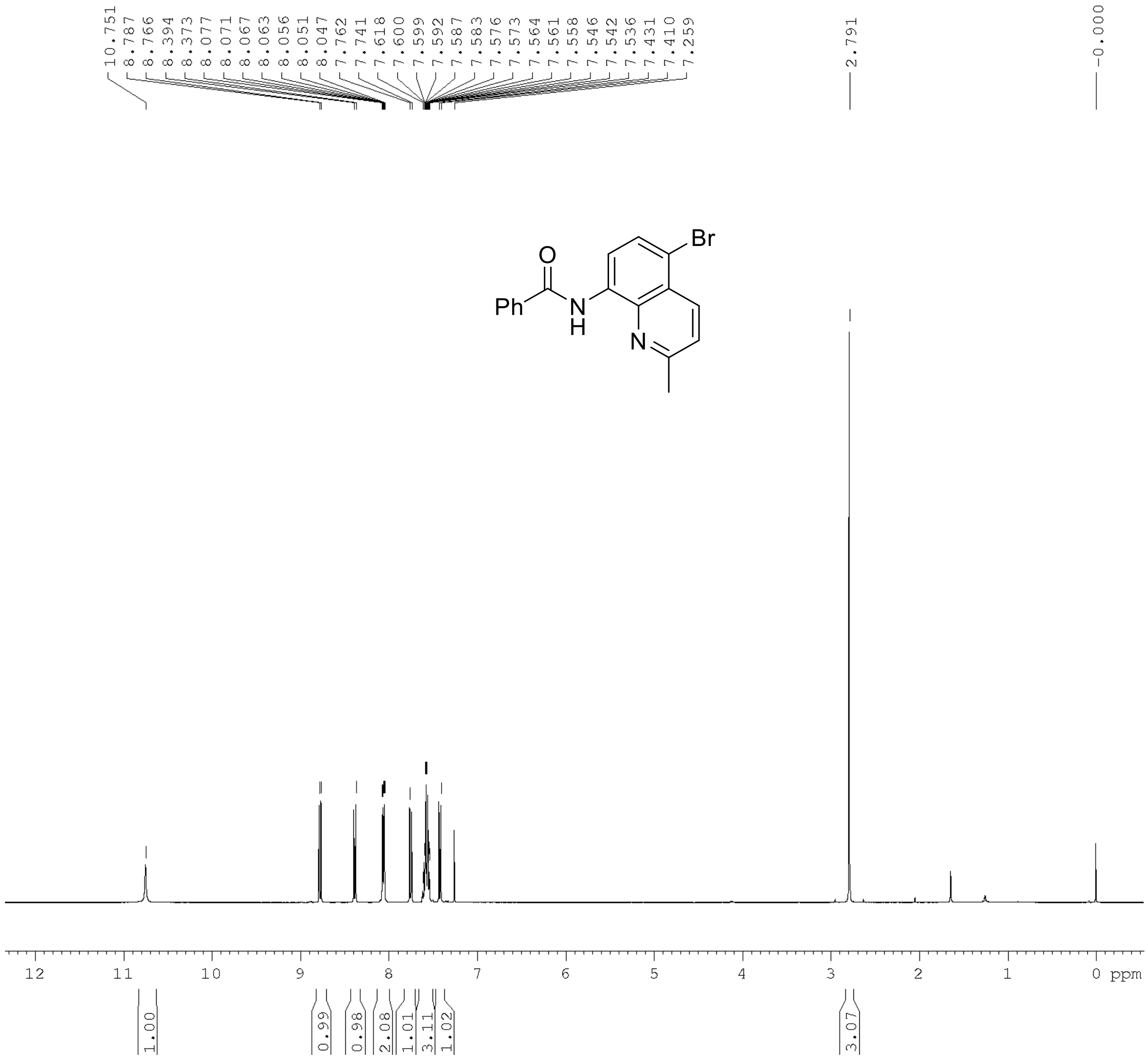

$^{13}C\{^{1}H\}$NMR spectrum of compound **5a** ($CDCl_3$, 100 MHz)**:**

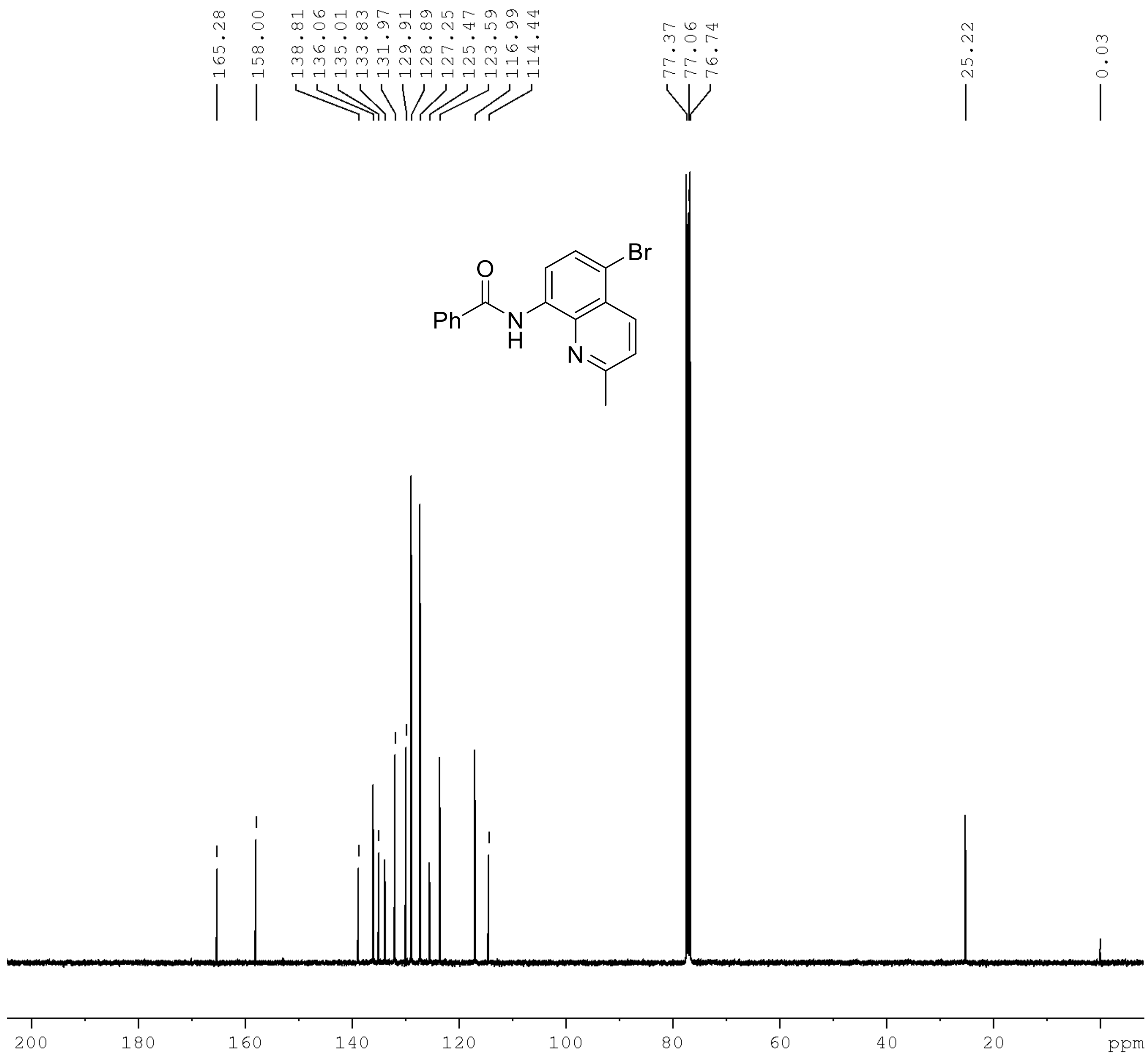

HRMS spectrum of compound **5a:**

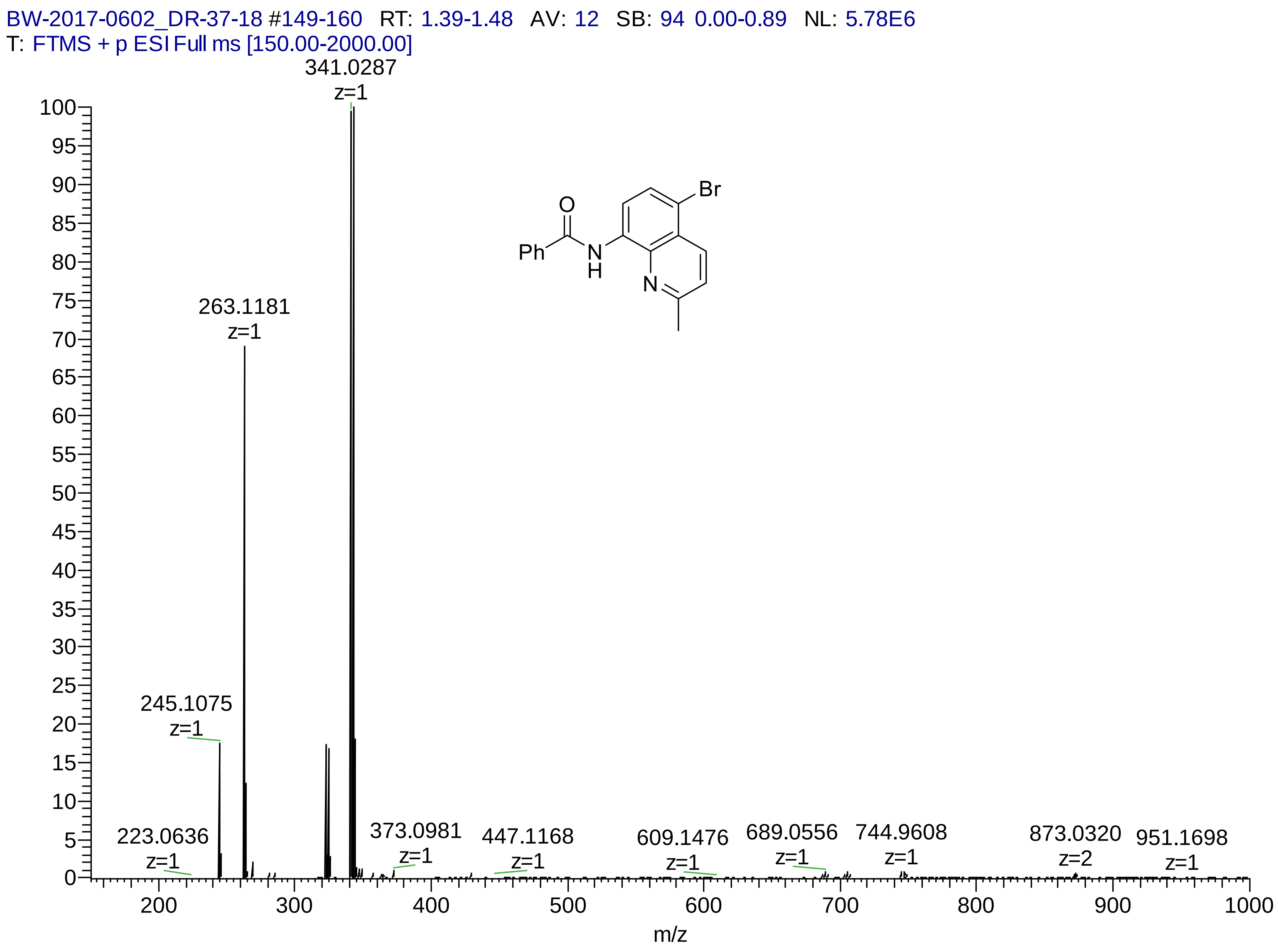

^{1}H NMR spectrum of compound **4b** ($CDCl_3$, 400 MHz)**:**

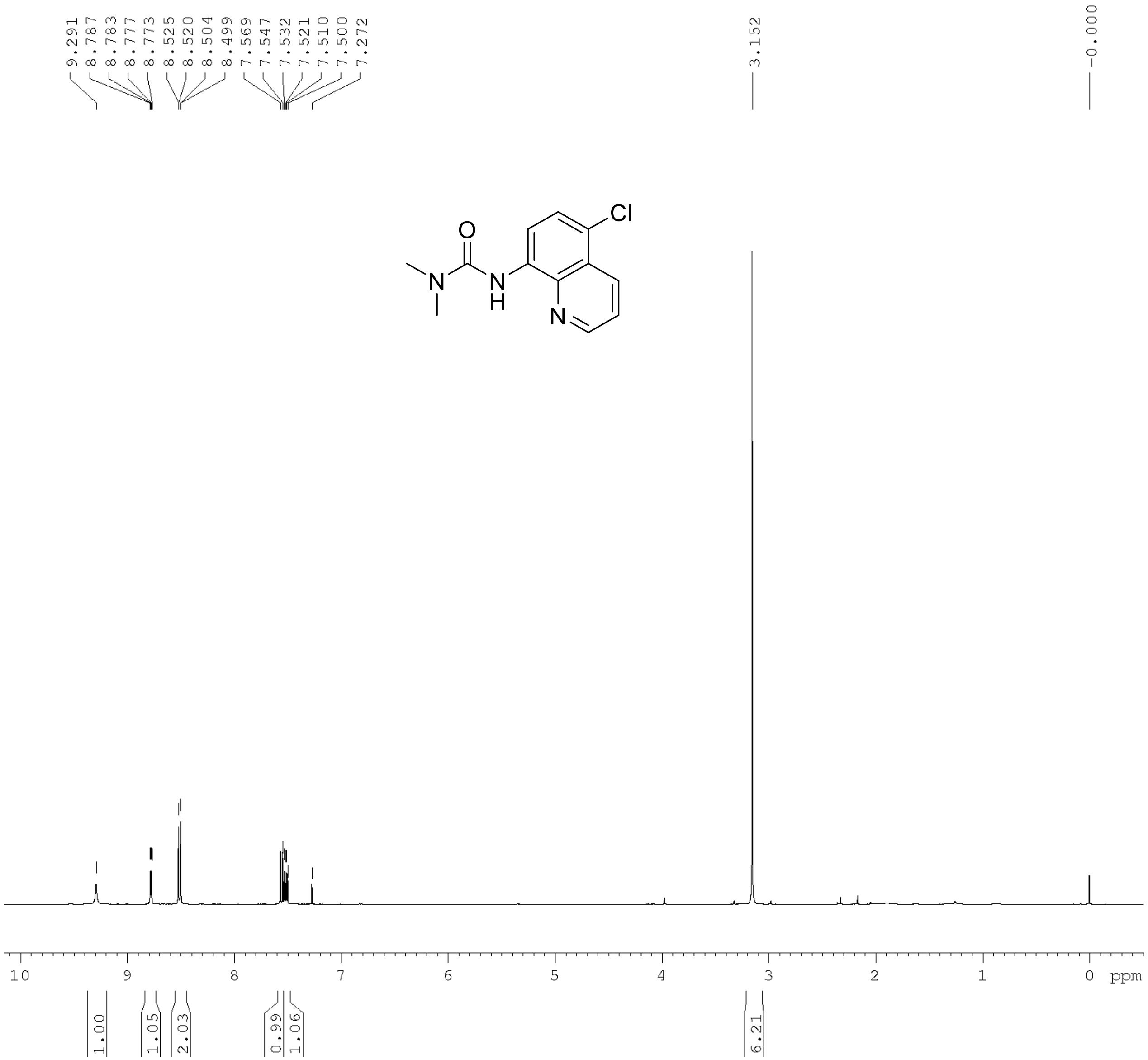

$^{13}C\{^1H\}$NMR spectrum of compound **4b** ($CDCl_3$, 100 MHz)**:**

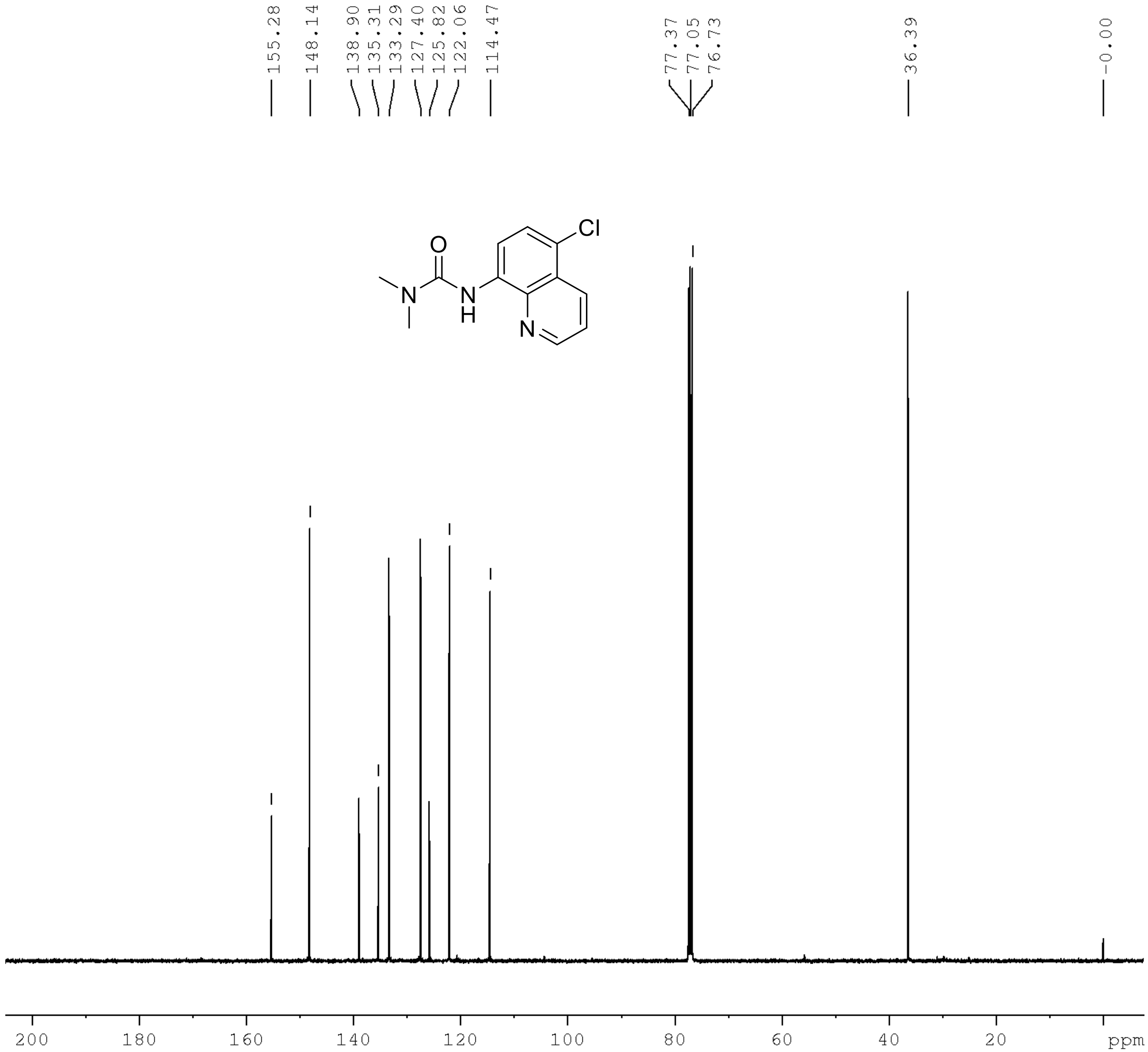

DEPT135 spectrum of compound **4b** ($CDCl_3$, 100 MHz)**:**

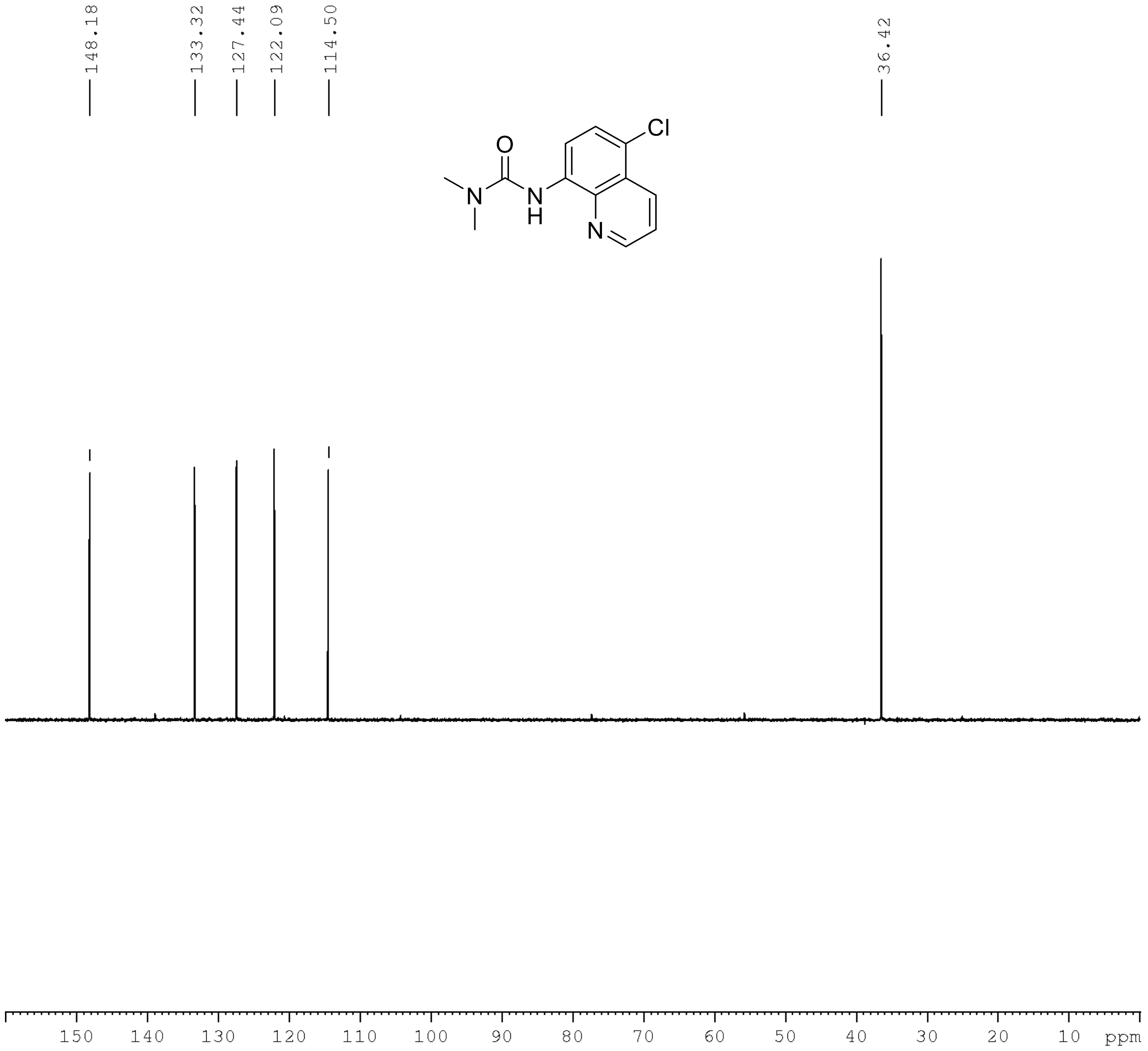

HRMS spectrum of compound **4b:**

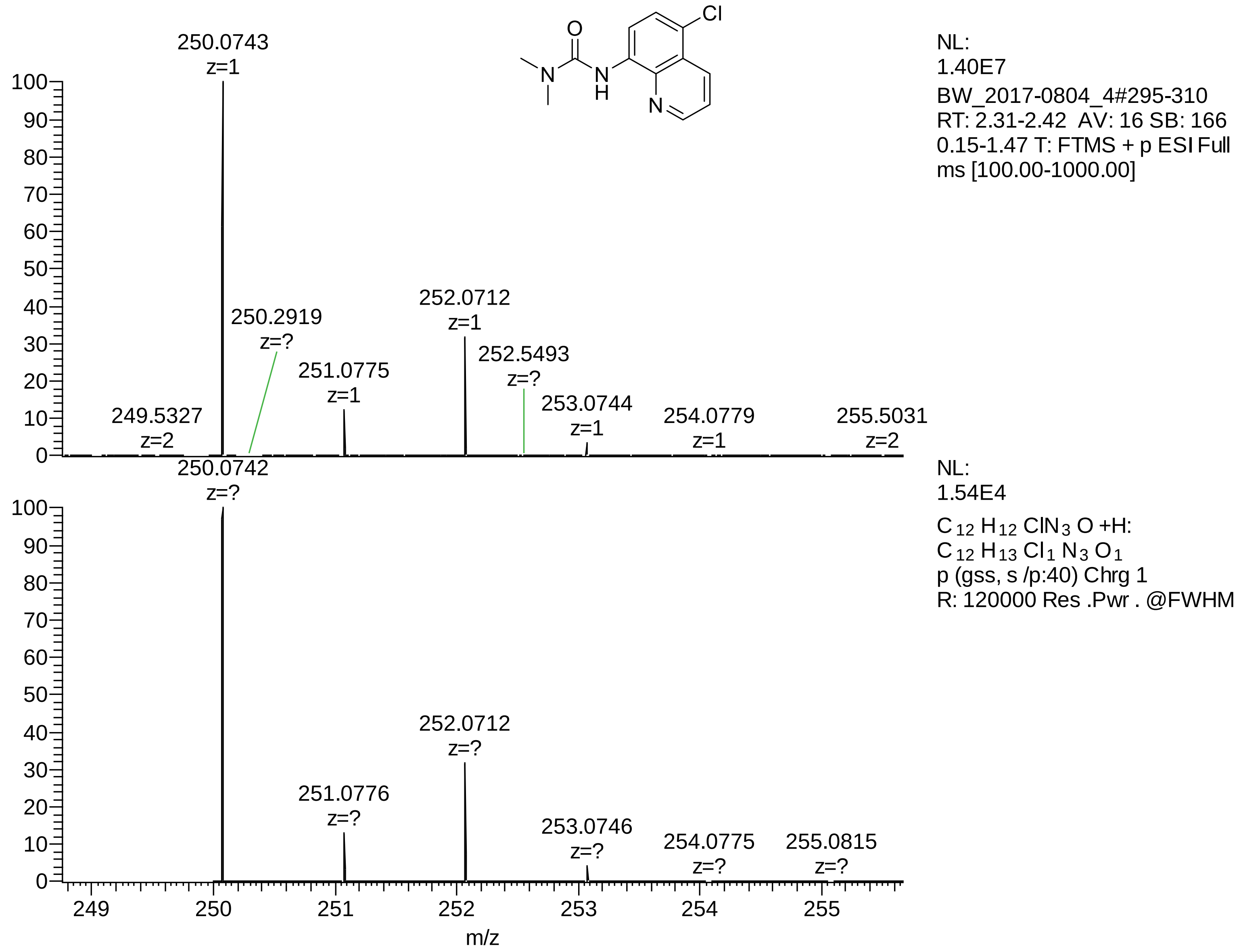

^{1}H NMR spectrum of compound **5b** ($CDCl_3$, 400 MHz)**:**

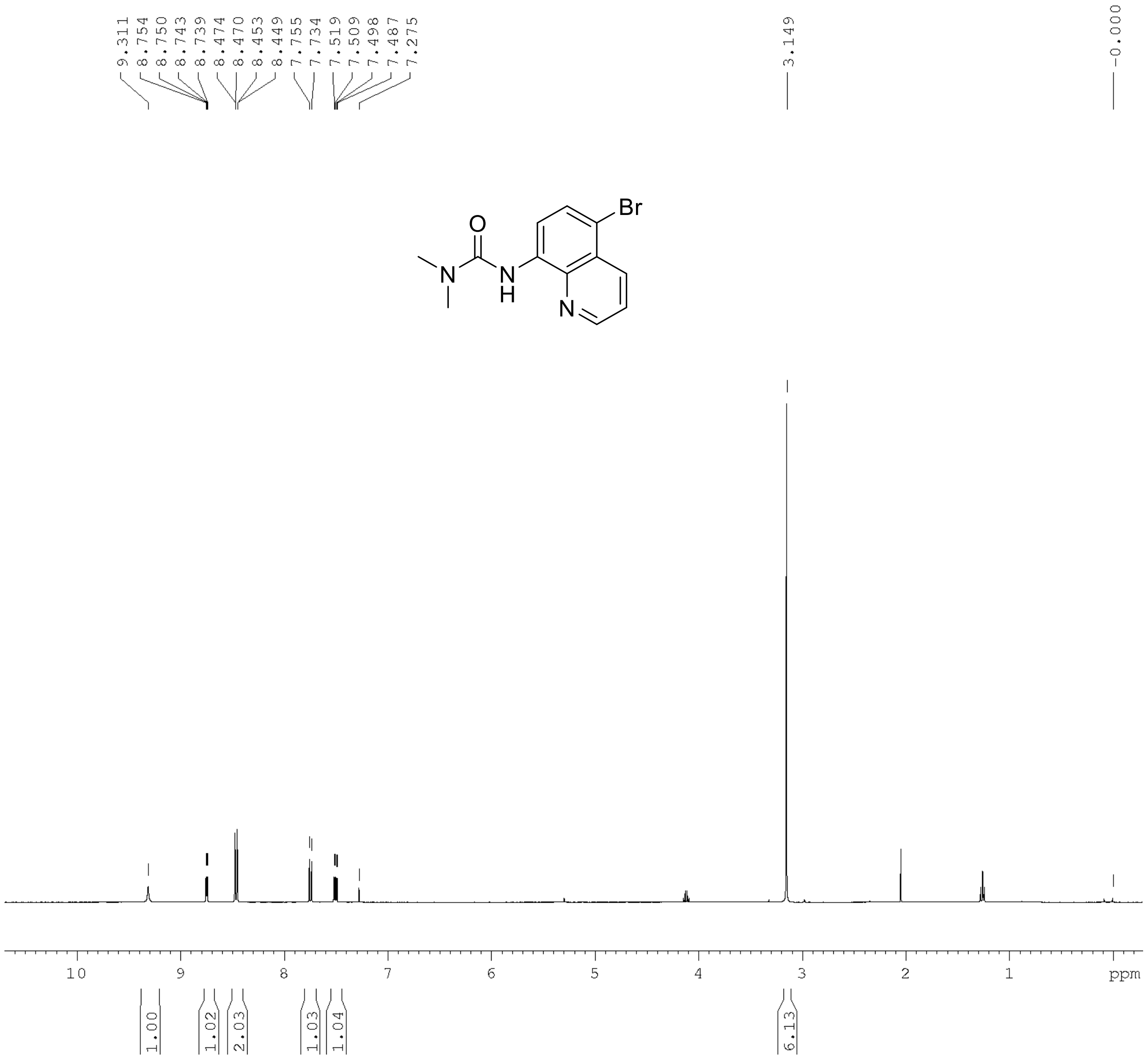

$^{13}C\{^1H\}$NMR spectrum of compound **5b** ($CDCl_3$, 100 MHz):

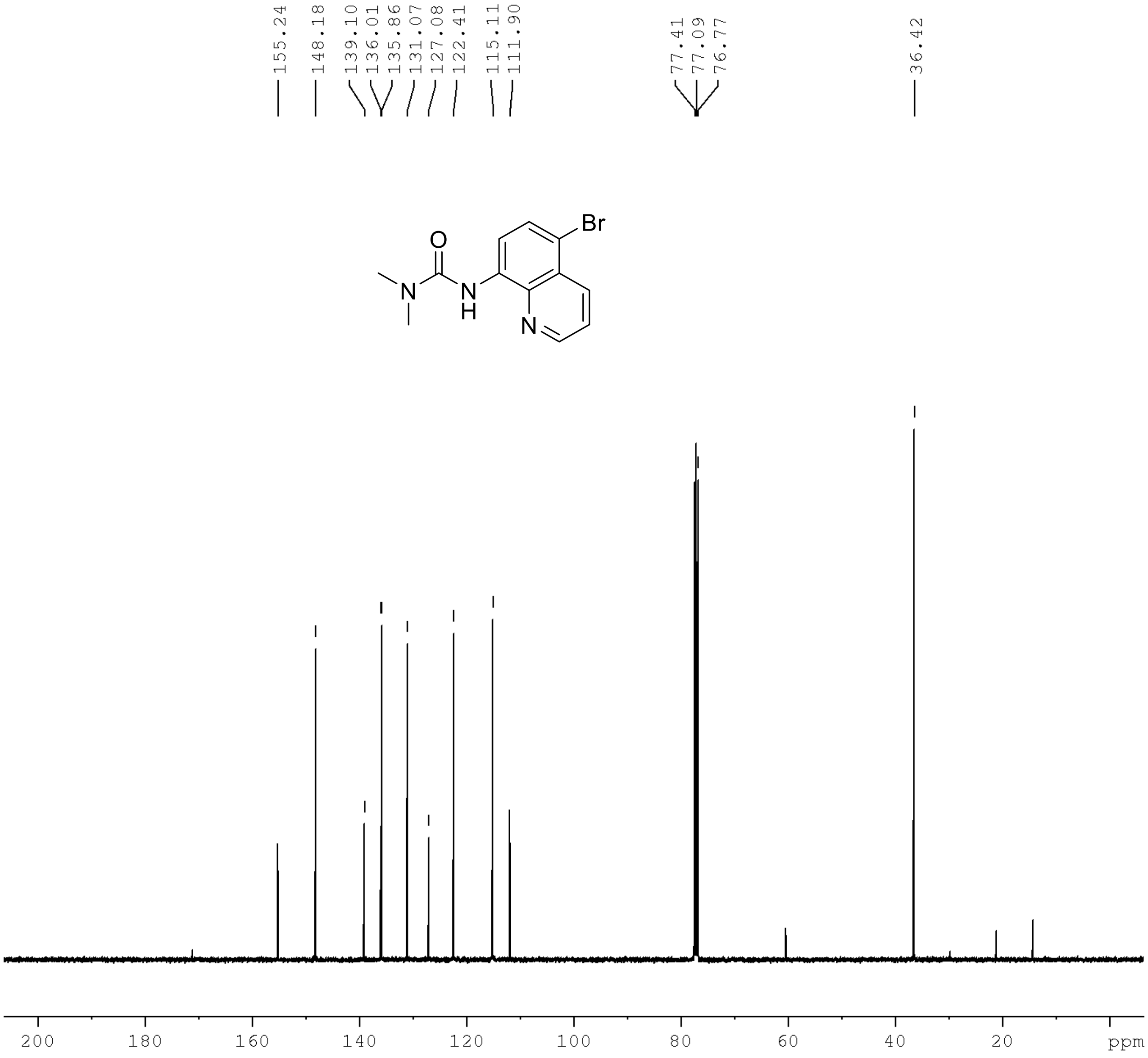

DEPT135 spectrum of compound **5b** ($CDCl_3$, 100 MHz)**:**

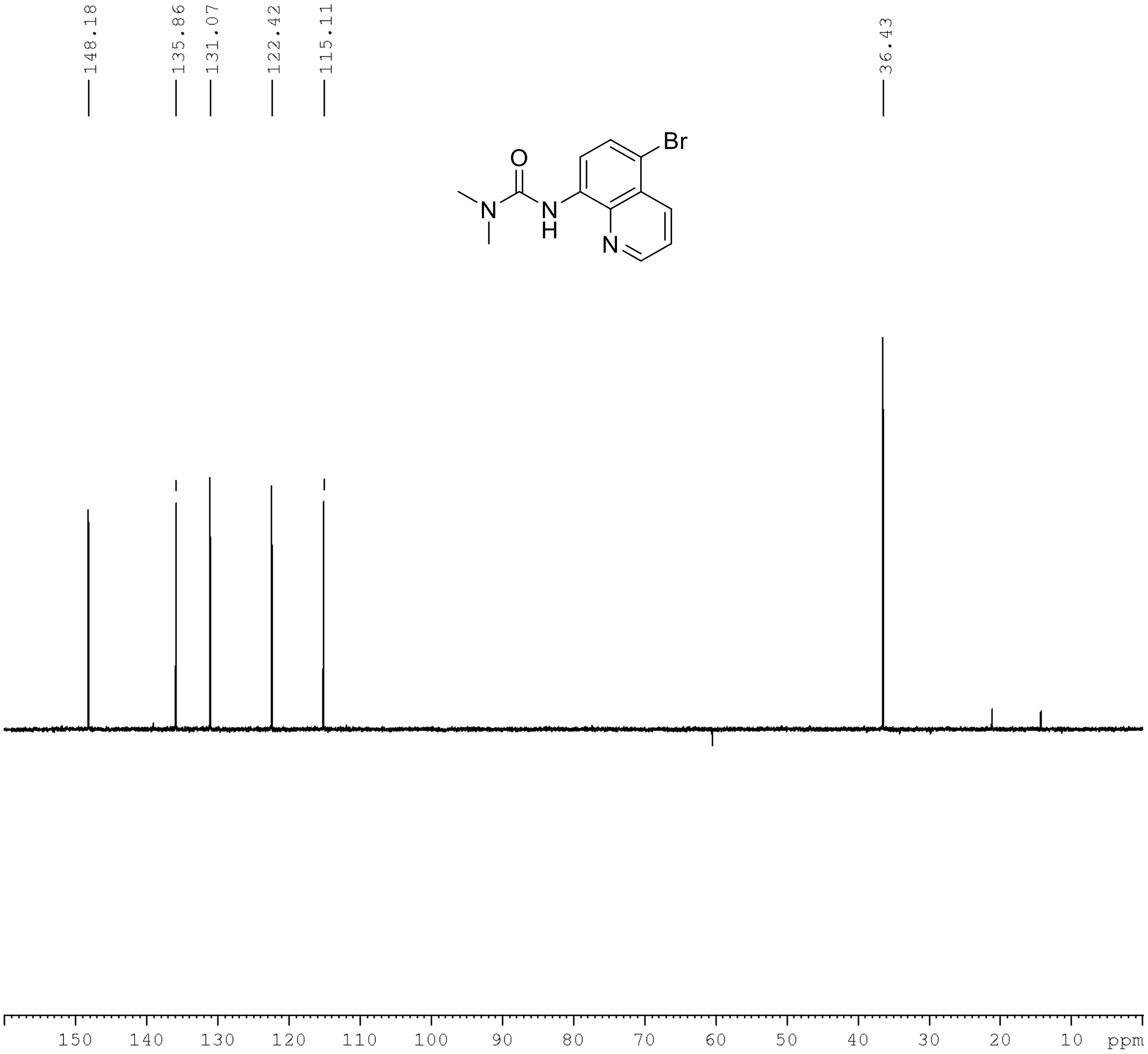

HRMS spectrum of compound **5b:**

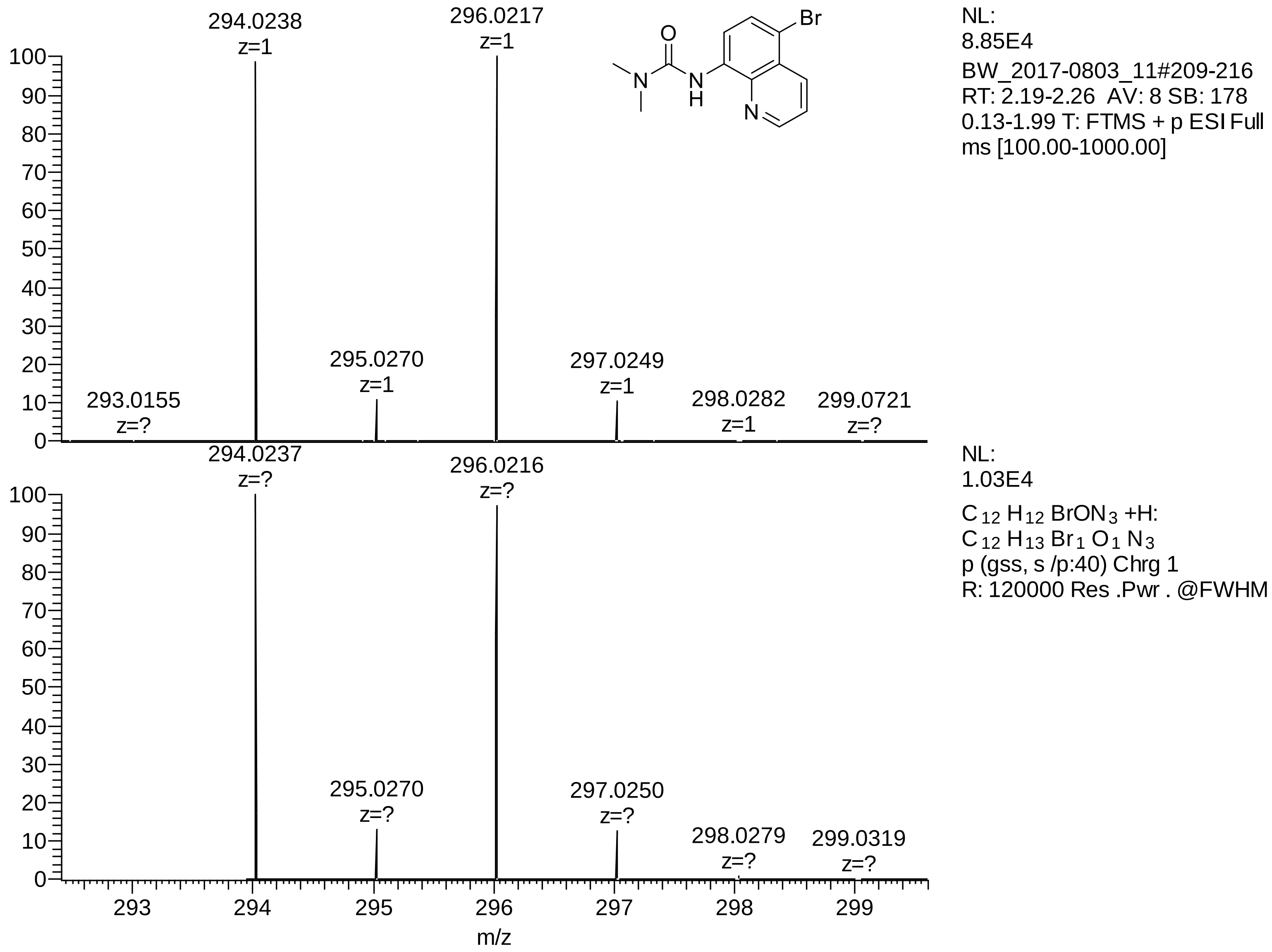

^{1}H NMR spectrum of compound **4c** (8:2, $CDCl_3$ + DMSO-*d6*, 400 MHz)**:**

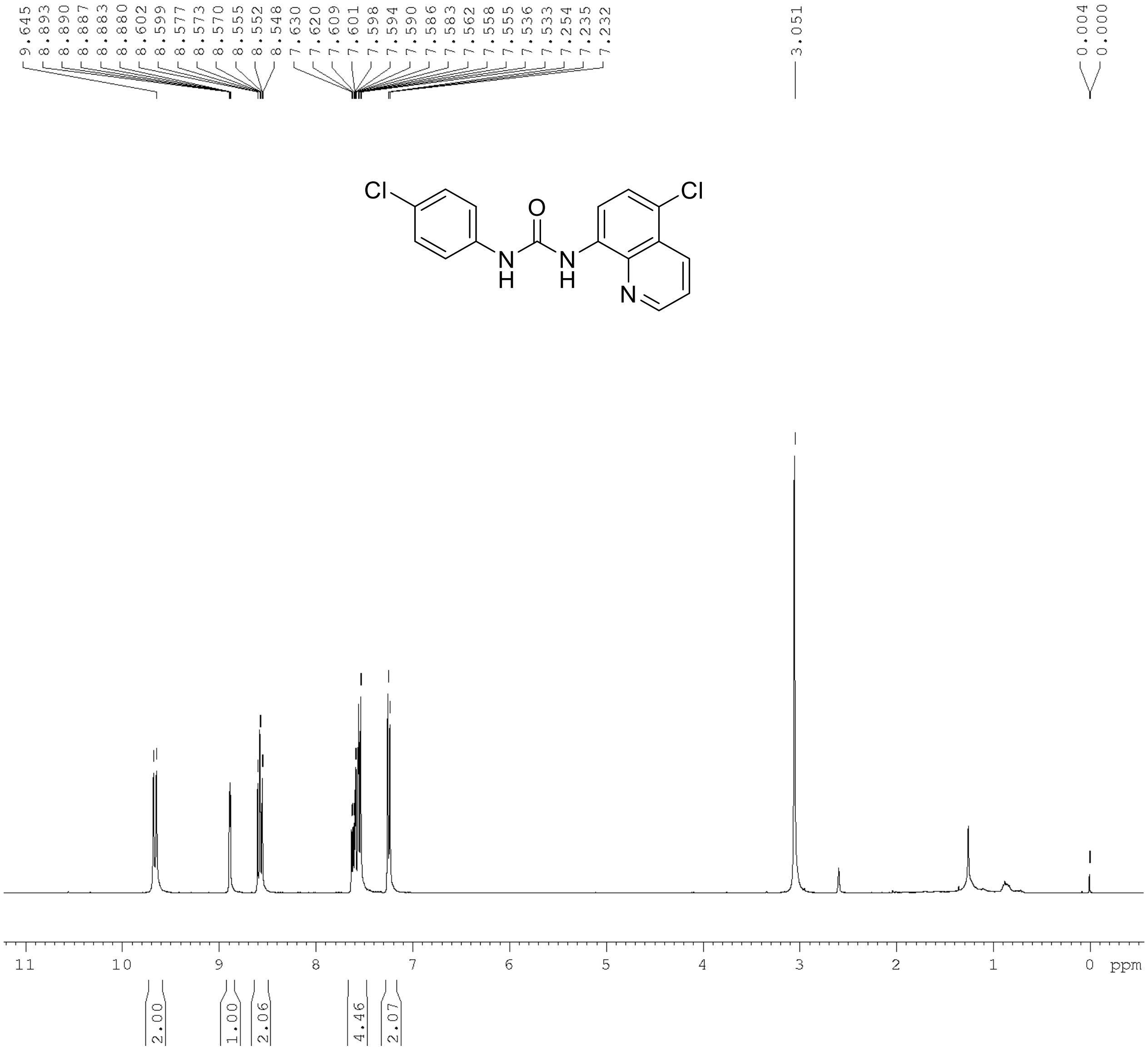

$^{13}C\{^{1}H\}$NMR spectrum of compound **4c** (8:2, $CDCl_3$ + DMSO-*d6*, 100 MHz)**:**

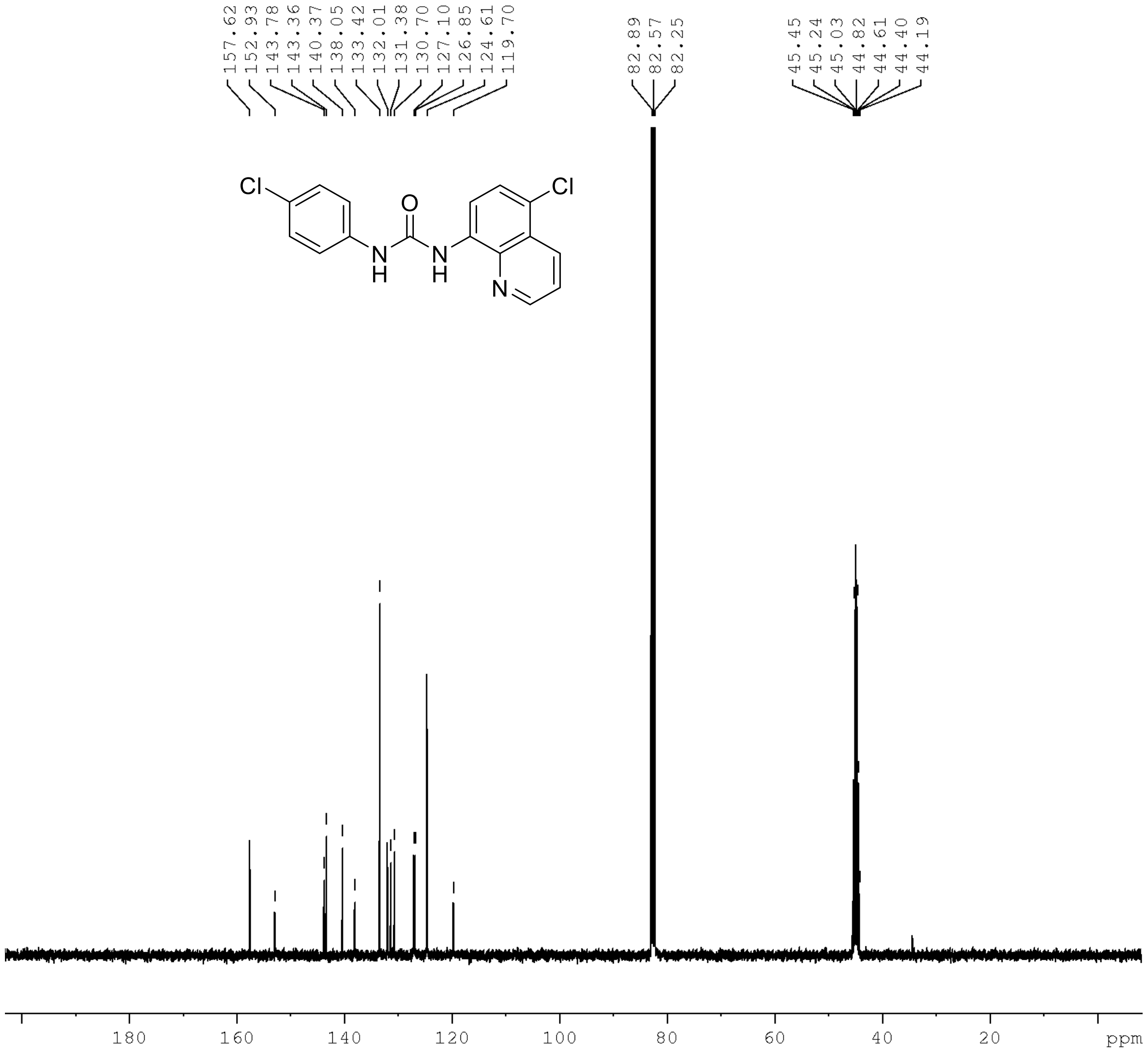

HRMS spectrum of compound **4c:**

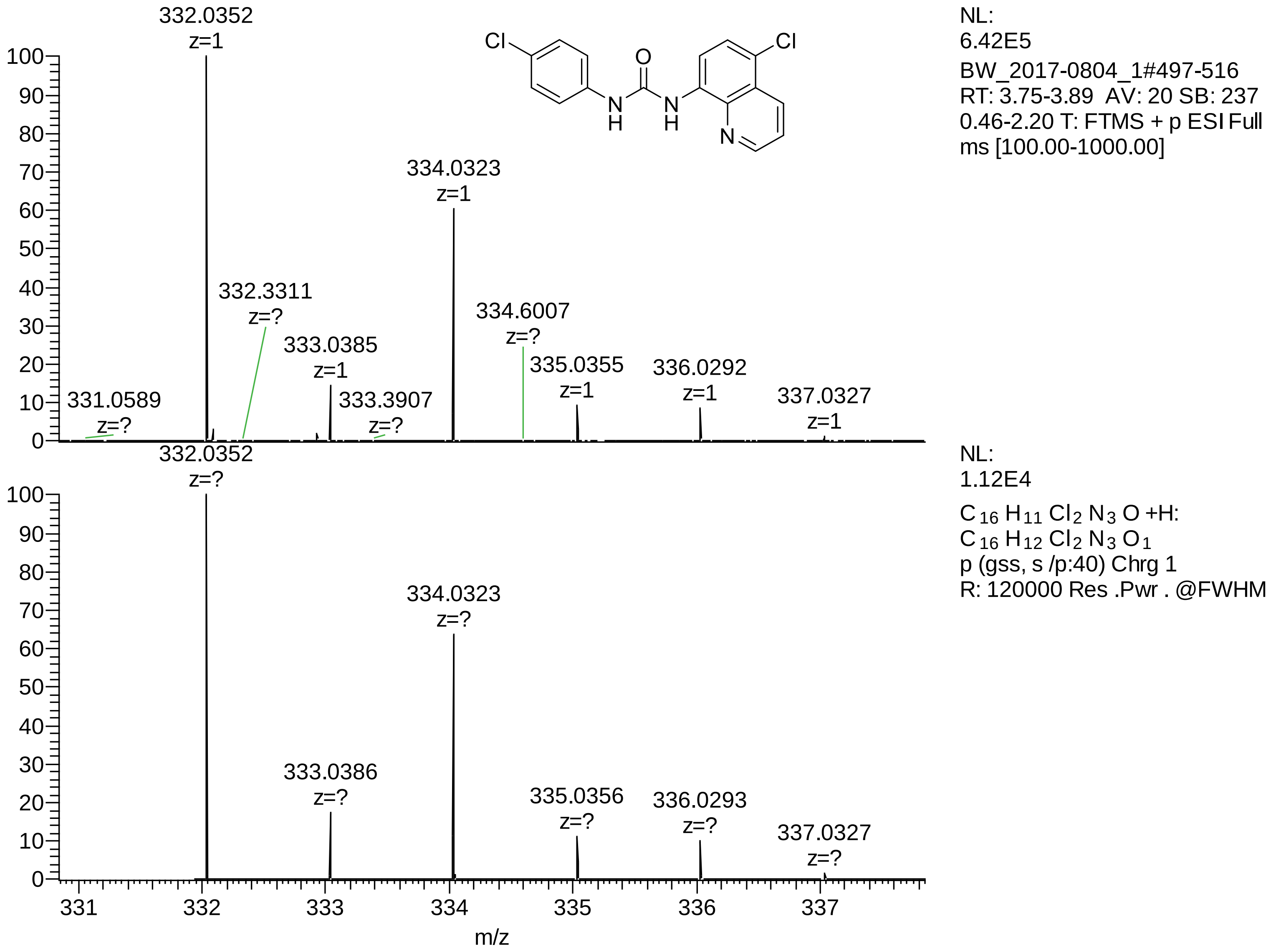

^{1}H NMR spectrum of compound **5c** (8:2, $CDCl_3$ + DMSO-*d6*, 400 MHz)**:**

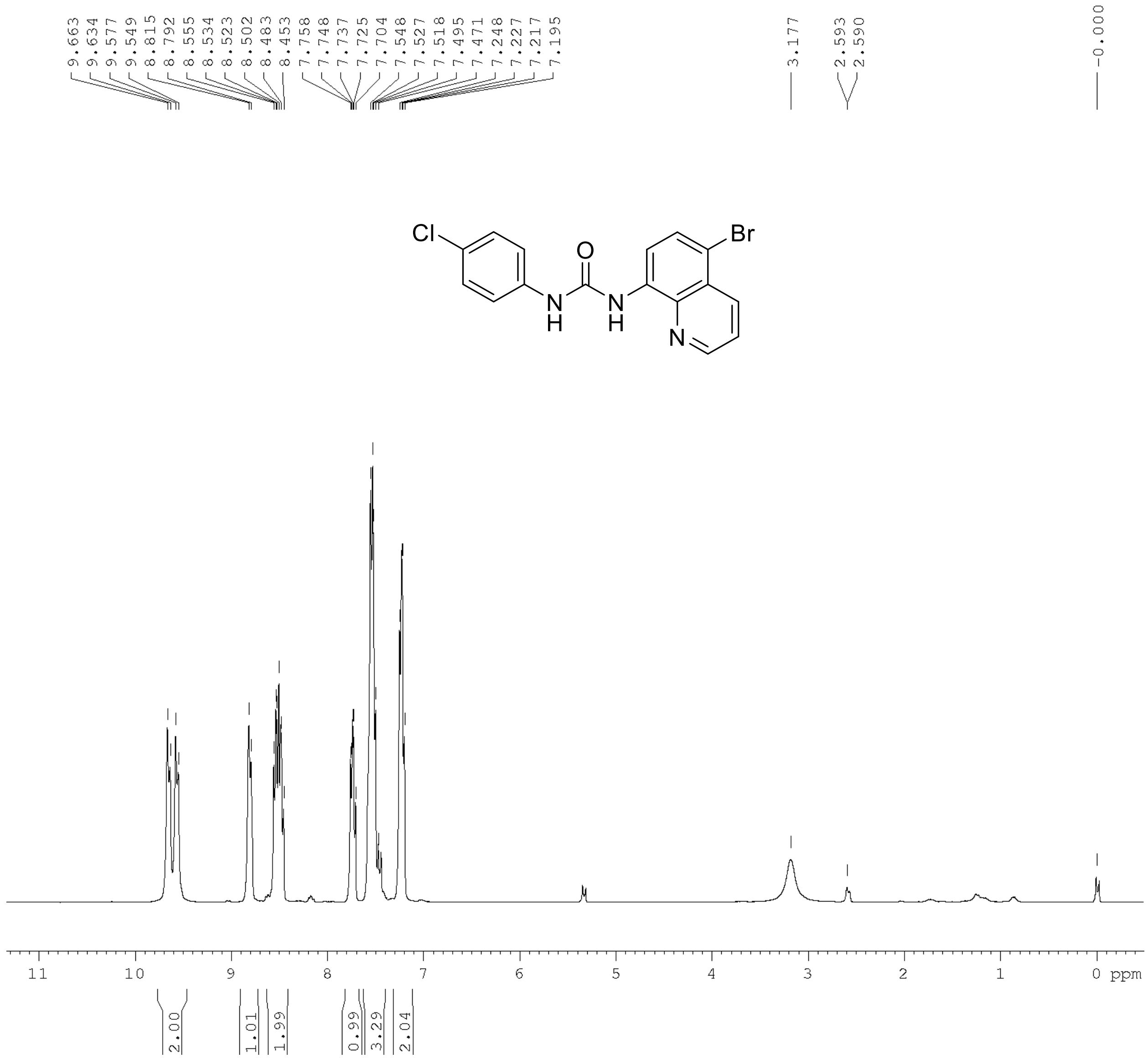

$^{13}C\{^{1}H\}$NMR spectrum of compound **5c** (8:2, $CDCl_3$ + DMSO-*d6*, 100 MHz)**:**

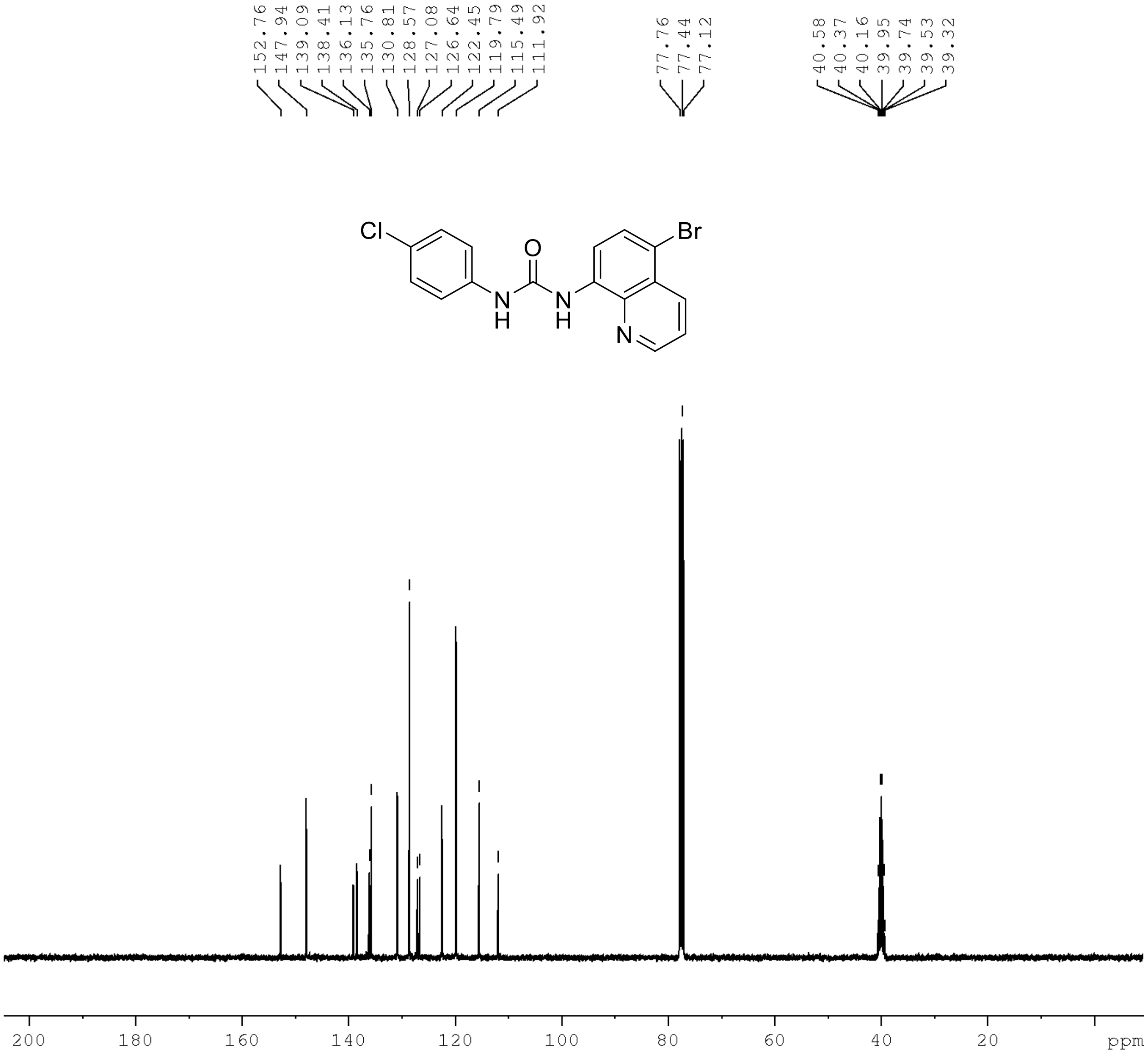

^{1}H NMR spectrum of compound **4d** ($CDCl_3$, 400 MHz)**:**

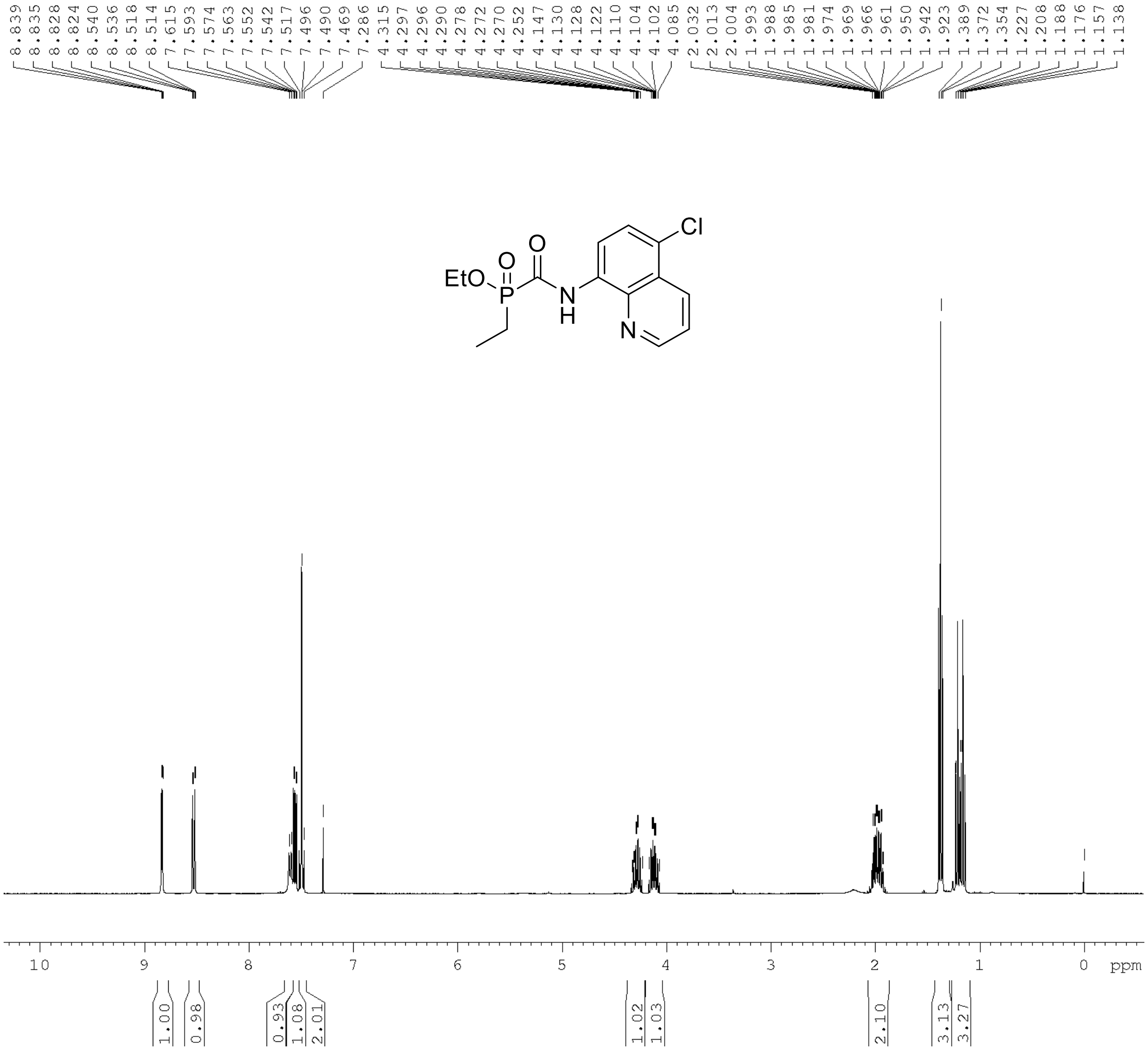

$^{13}C\{^{1}H\}$NMR spectrum of compound **4d** ($CDCl_3$, 100 MHz)**:**

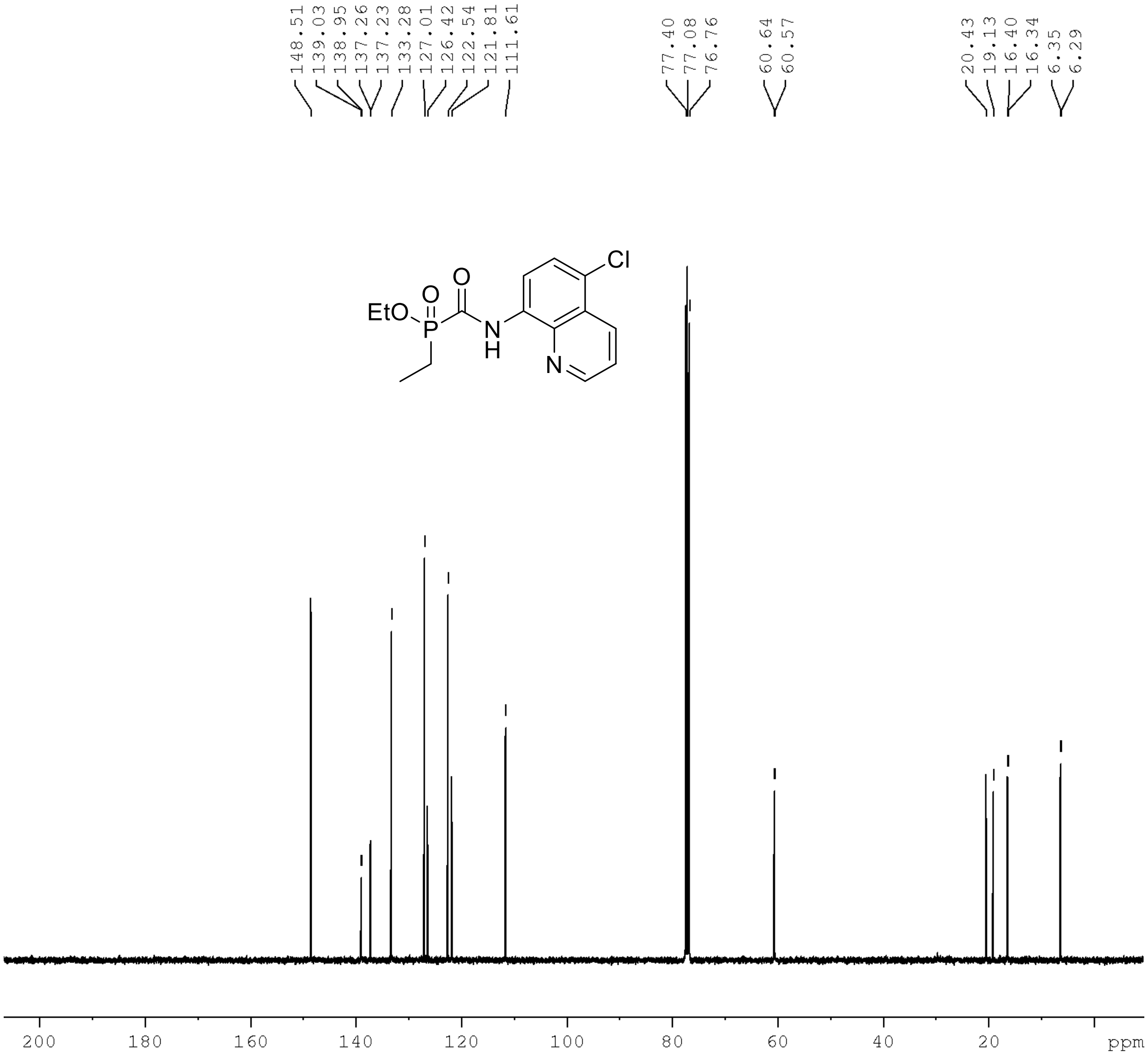

DEPT135 spectrum of compound **4d** ($CDCl_3$, 100 MHz)**:**

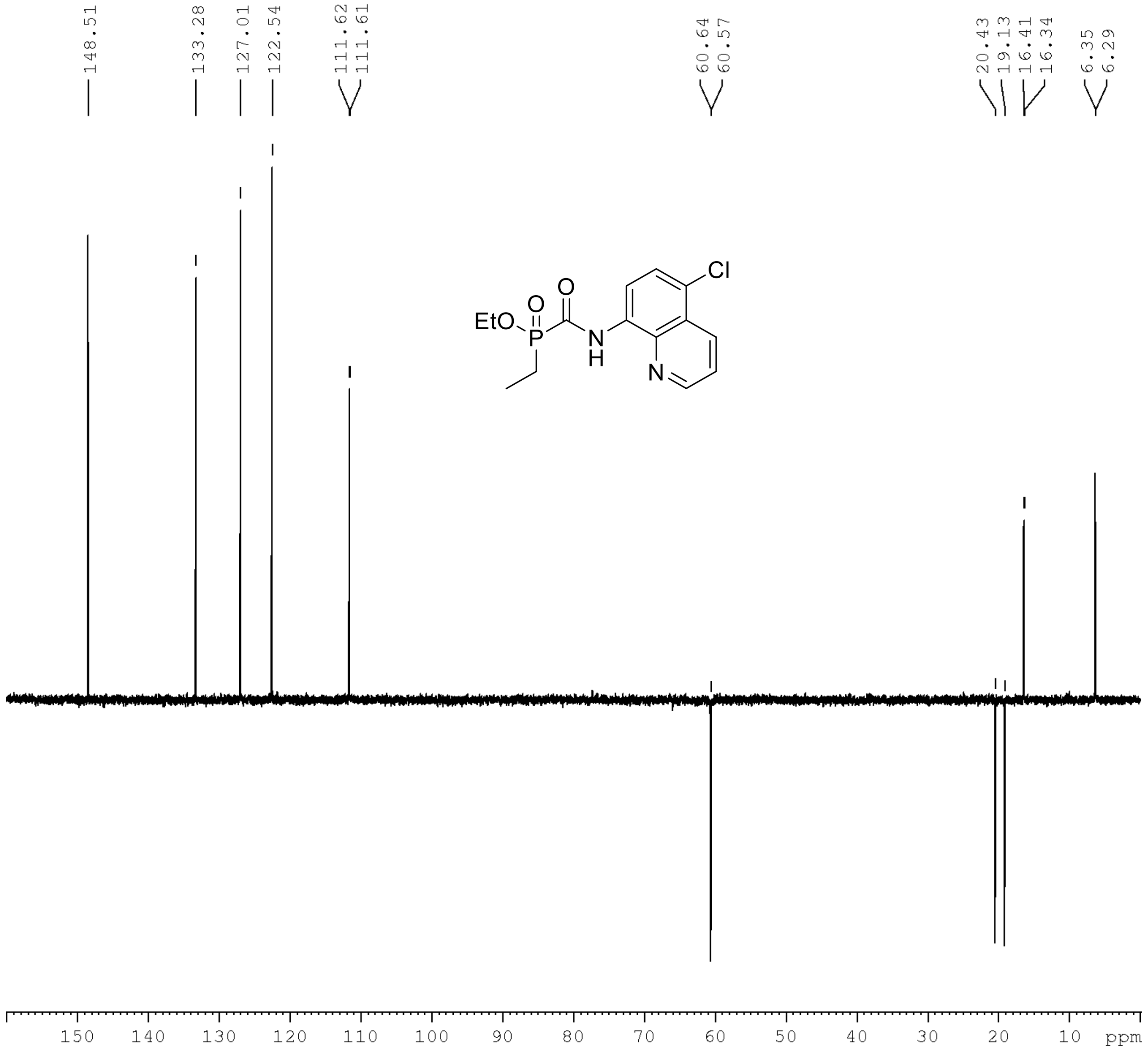

HRMS spectrum of compound **4d:**

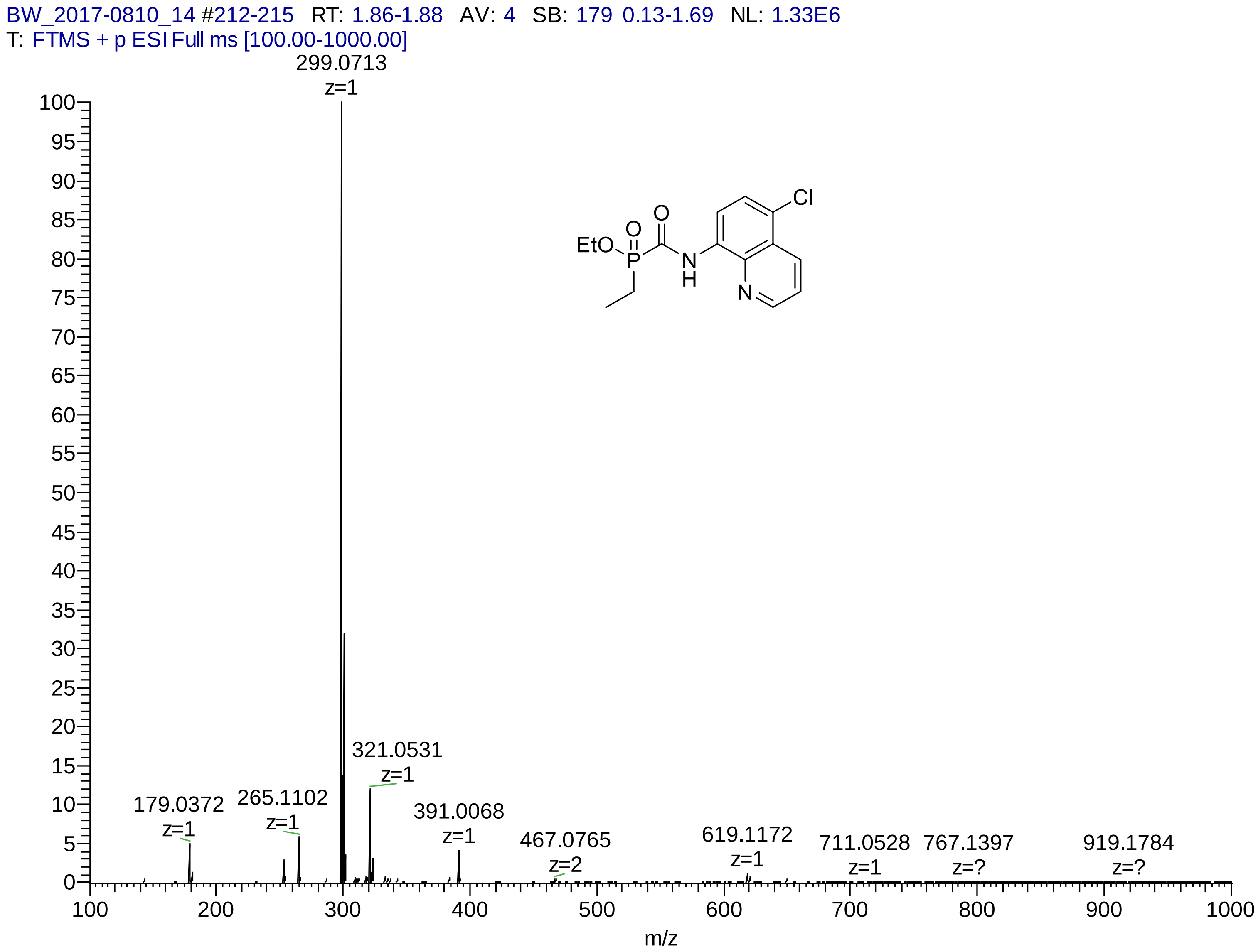

^{1}H NMR spectrum of compound **5d** ($CDCl_3$, 400 MHz)**:**

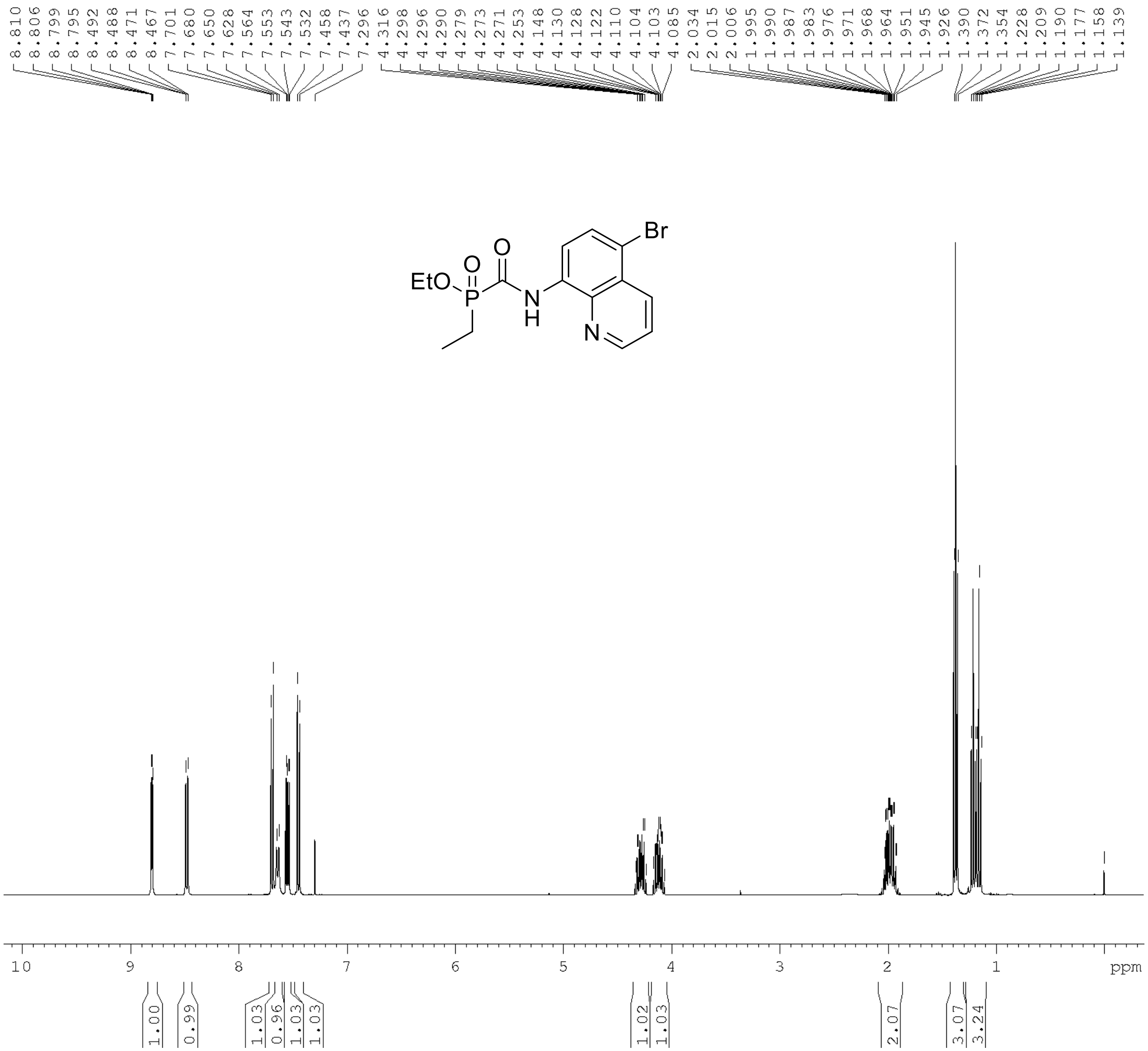

$^{13}C\{^1H\}$NMR spectrum of compound **5d** ($CDCl_3$, 100 MHz)**:**

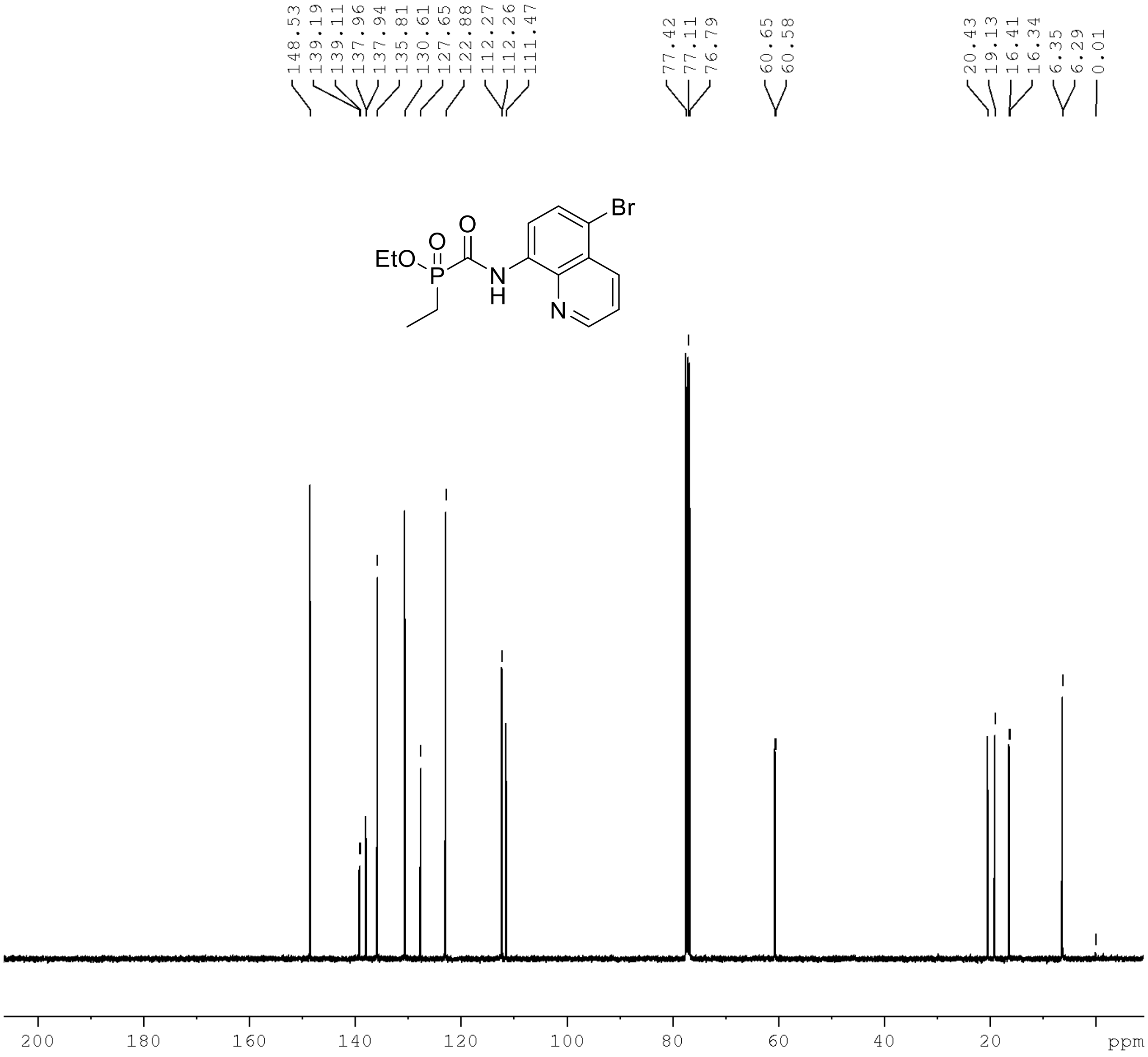

DEPT135 spectrum of compound **5d** ($CDCl_3$, 100 MHz)**:**

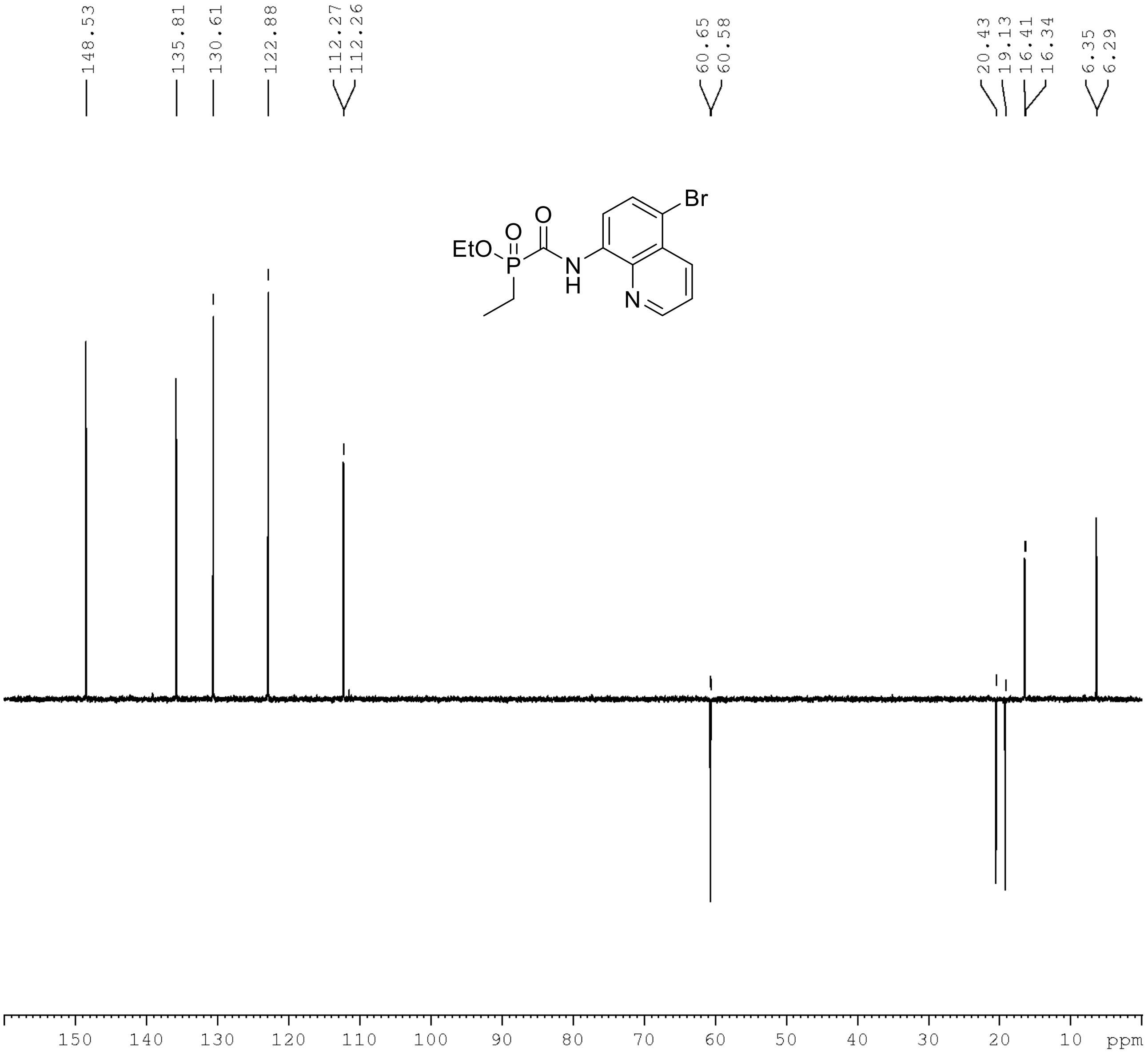

HRMS spectrum of compound **5d:**

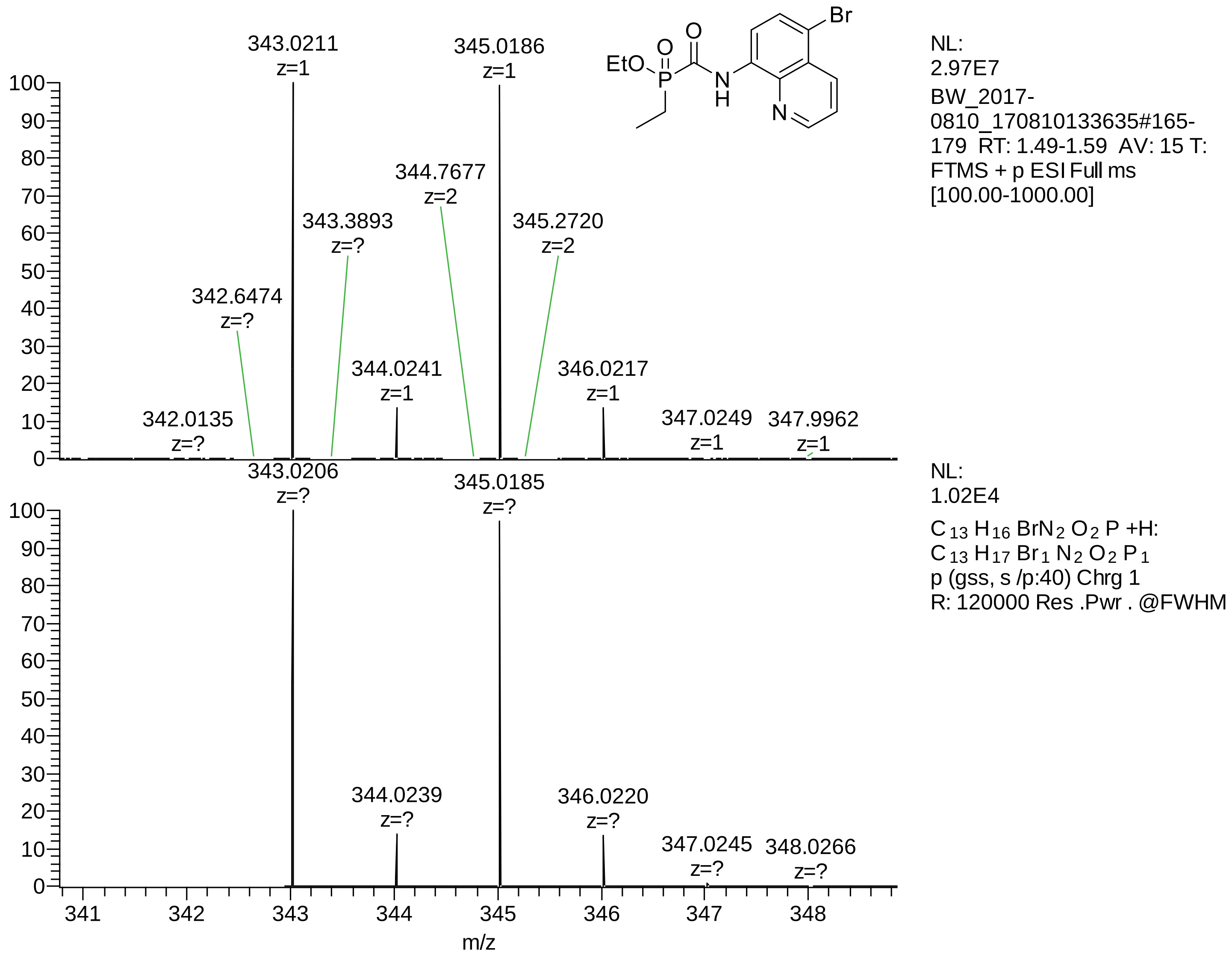

^{1}H NMR spectrum of compound **4e** ($CDCl_3$, 400 MHz)**:**

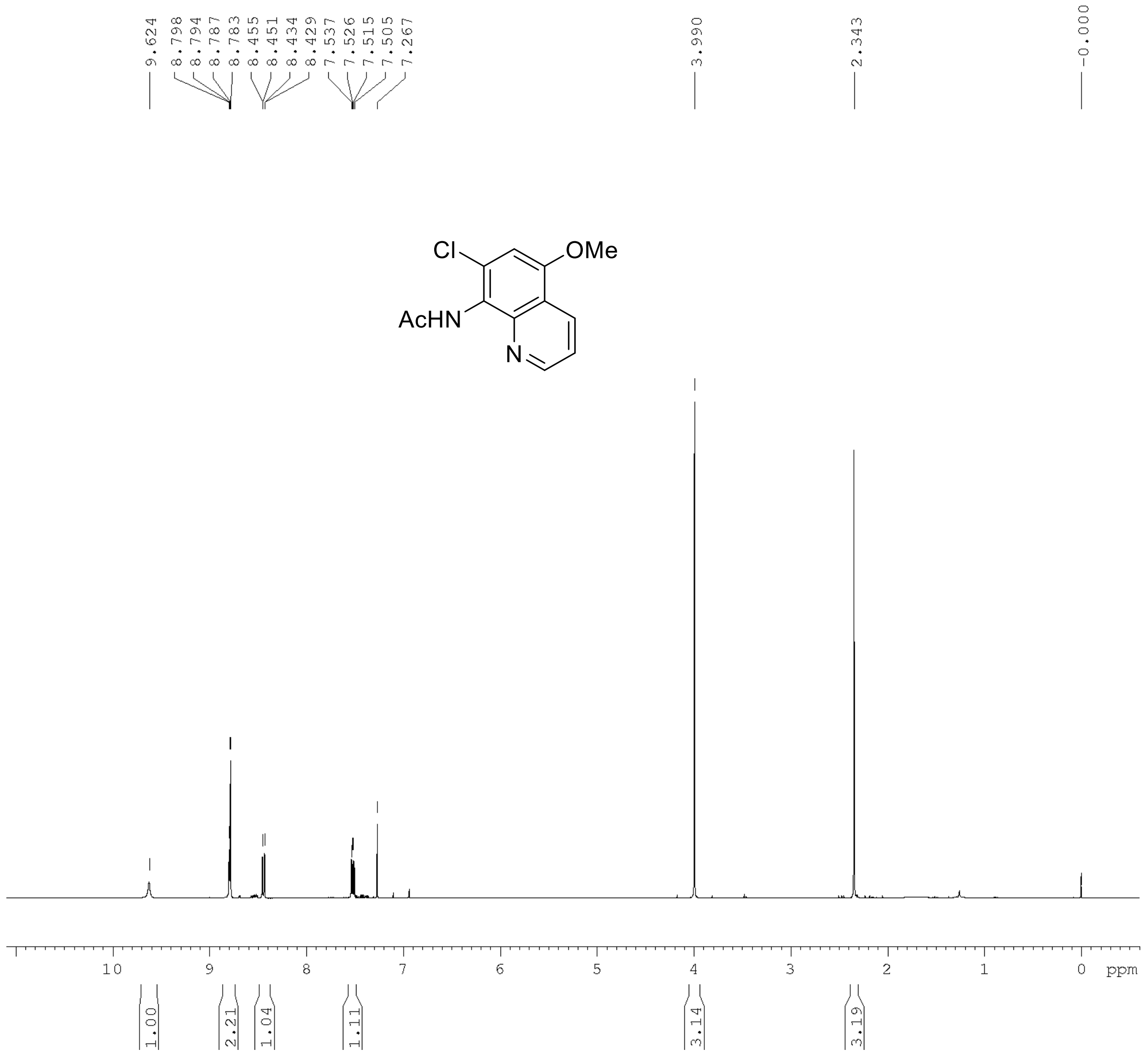

$^{13}C\{^1H\}$NMR spectrum of compound **4e** ($CDCl_3$, 100 MHz):

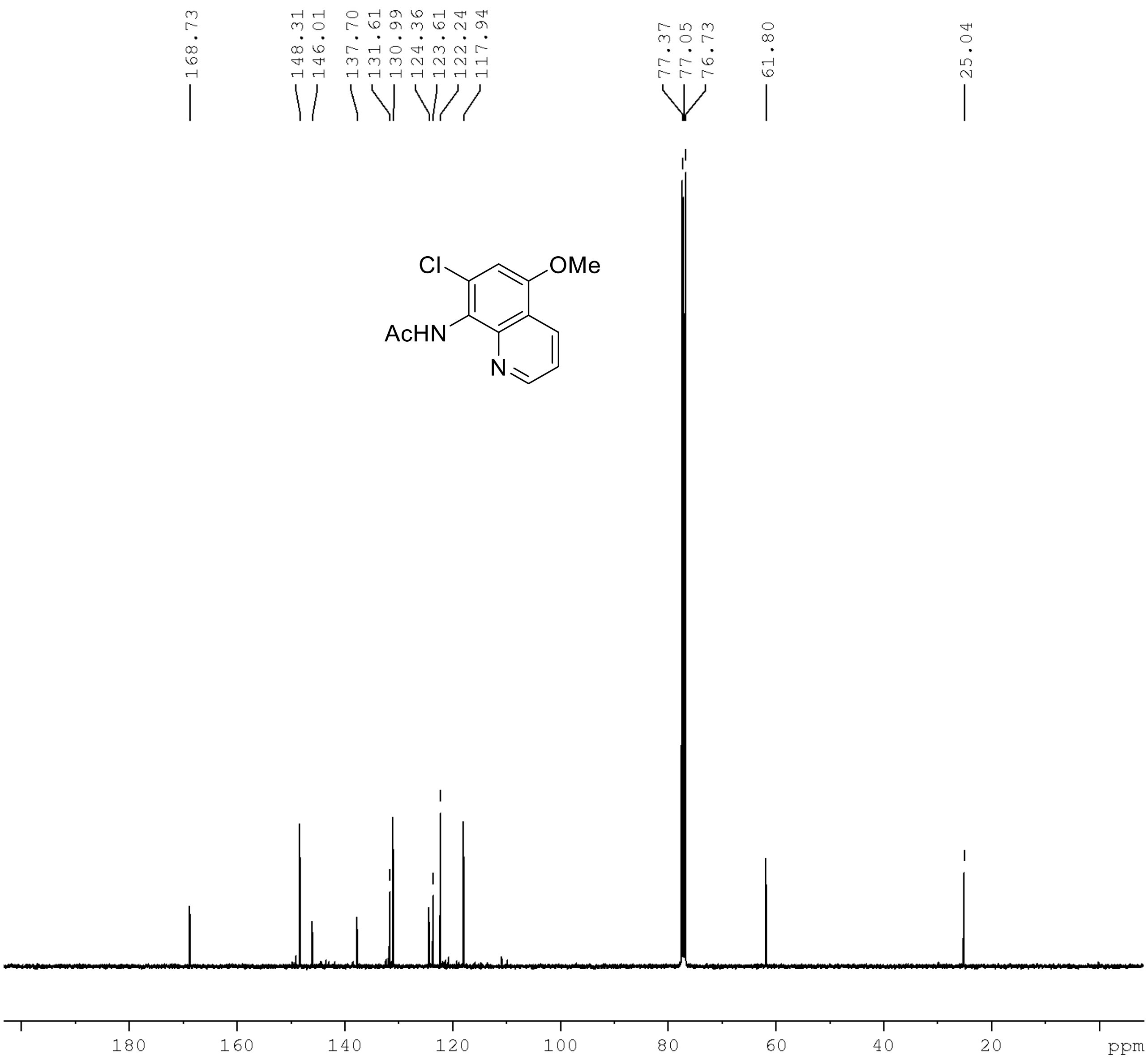

^{1}H NMR spectrum of compound **4f** ($CDCl_3$, 400 MHz)**:**

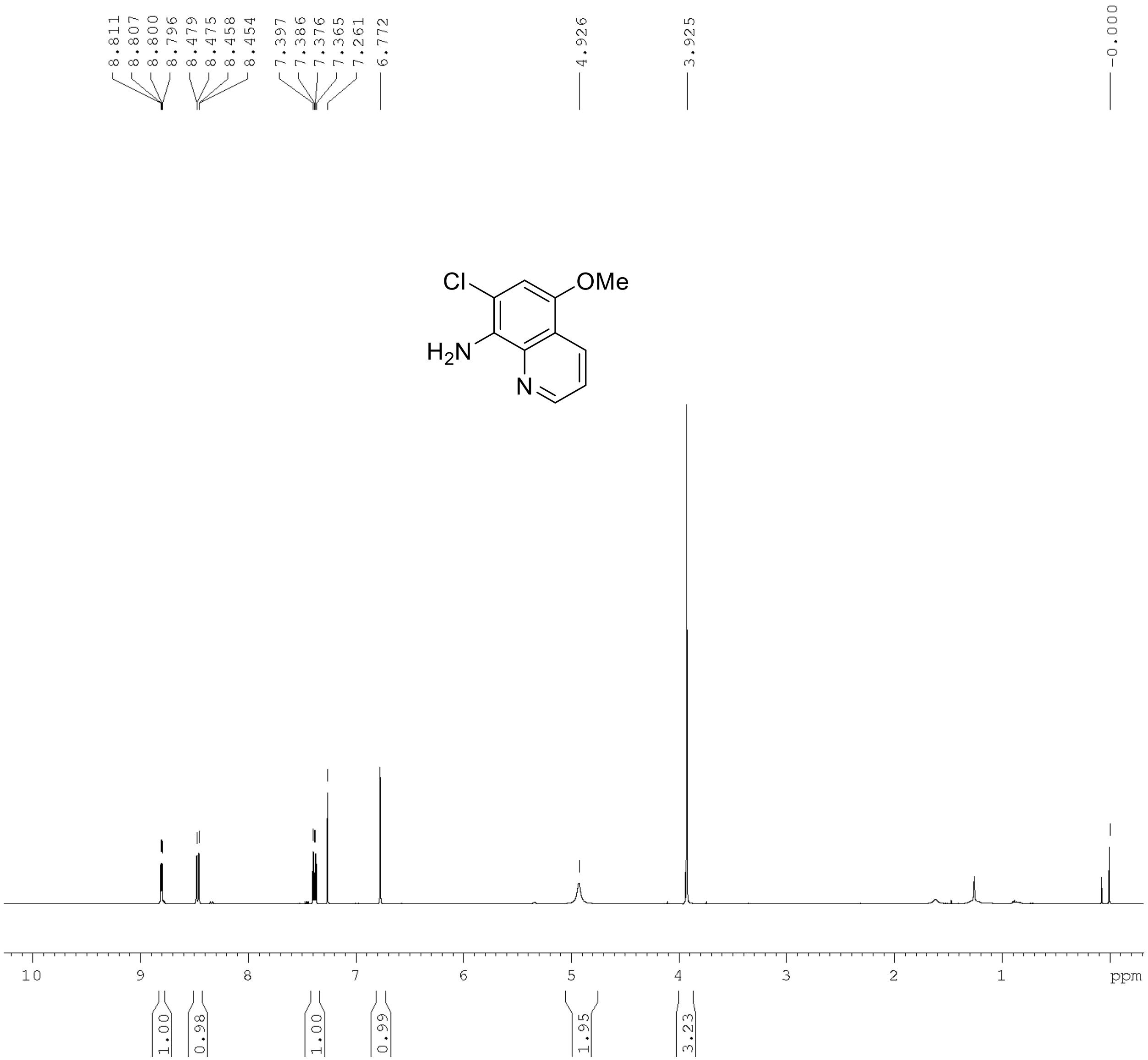

$^{13}C\{^{1}H\}$NMR spectrum of compound **4f** ($CDCl_3$, 100 MHz)**:**

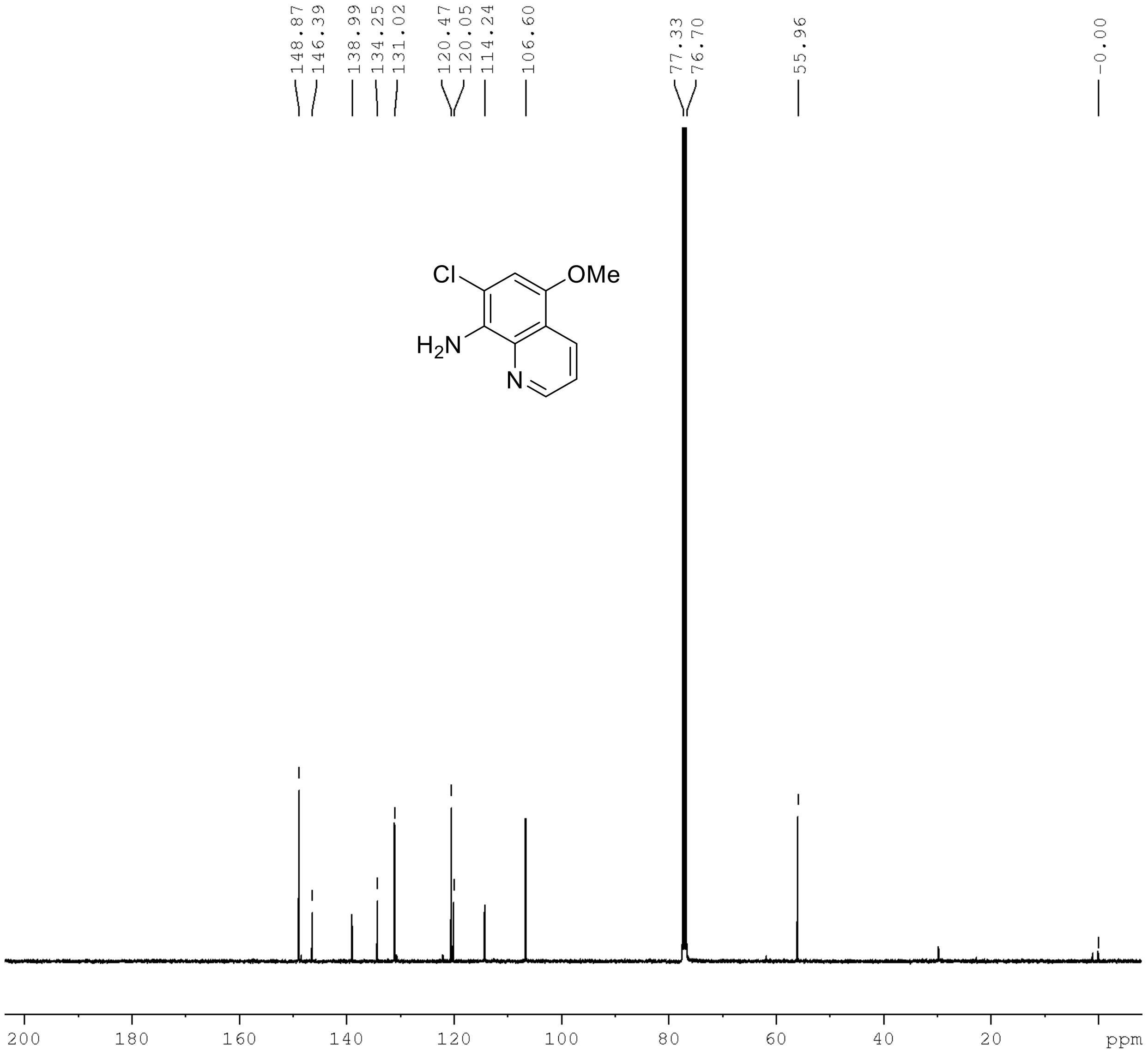

DEPT135 spectrum of compound **4f** ($CDCl_3$, 100 MHz)**:**

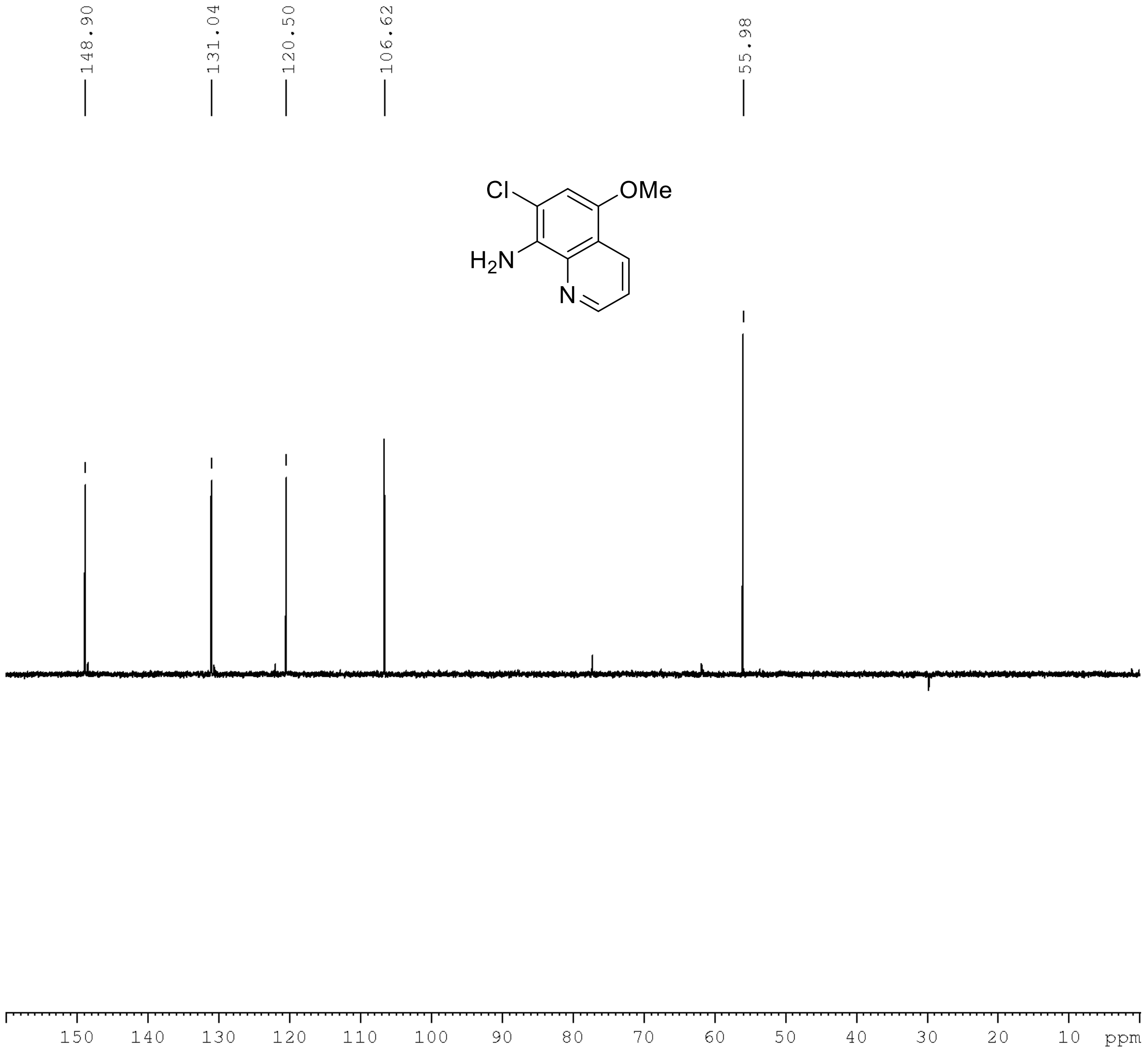

^{1}H NMR spectrum of compound **5f** ($CDCl_3$, 400 MHz):

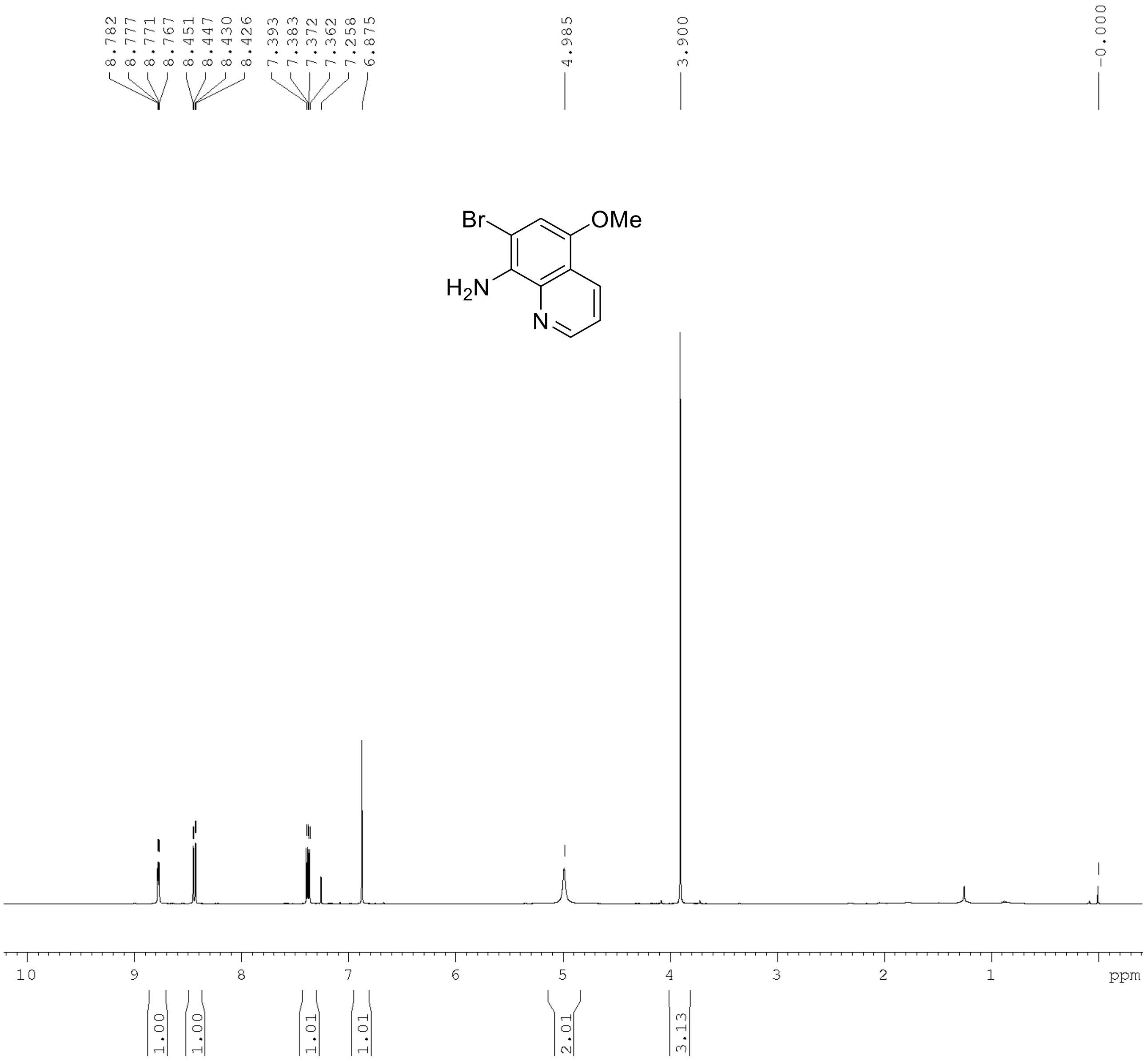

$^{13}C\{^{1}H\}$NMR spectrum of compound **5f** ($CDCl_3$, 100 MHz)**:**

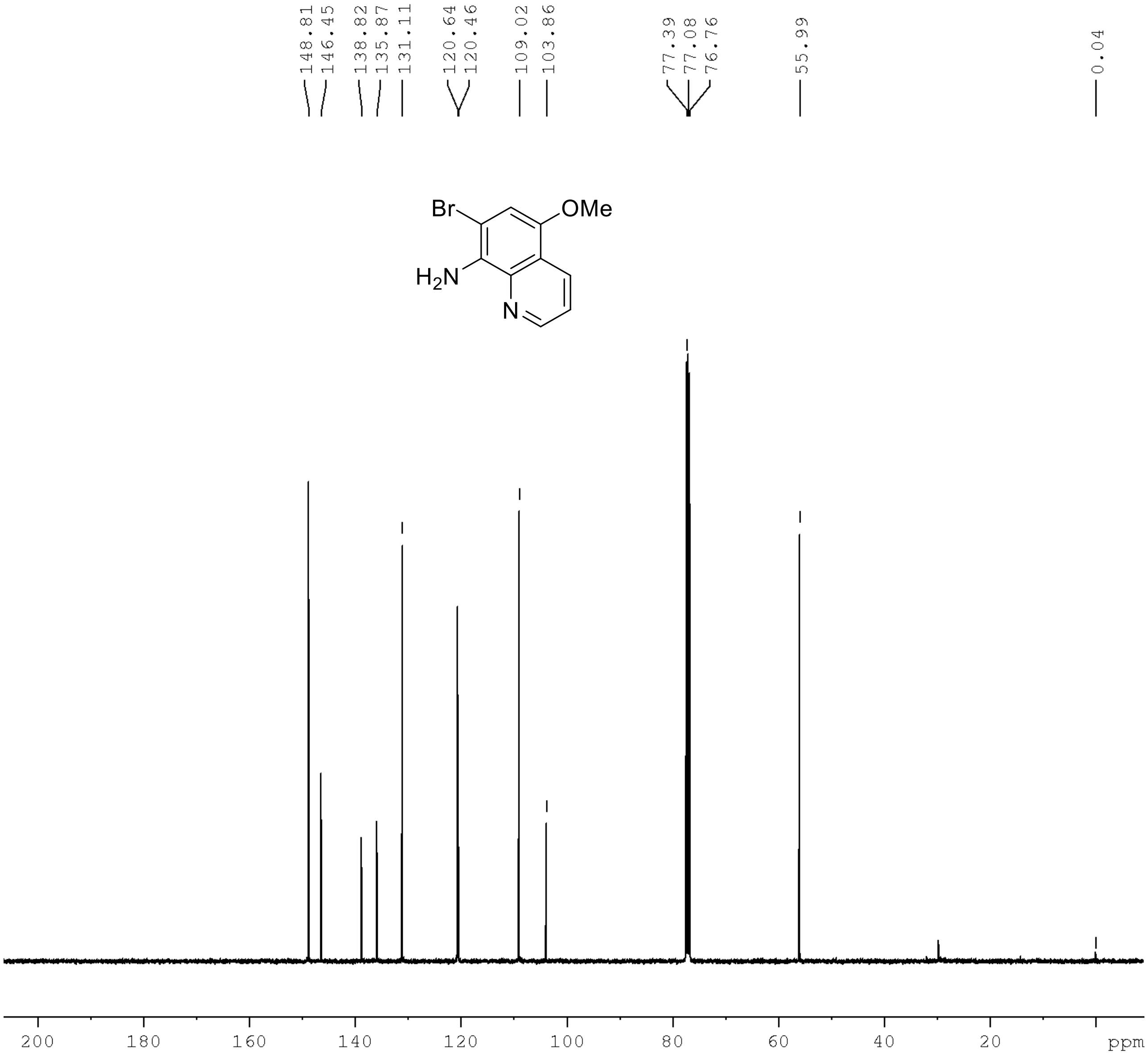

DEPT135 spectrum of compound **5f** ($CDCl_3$, 100 MHz)**:**

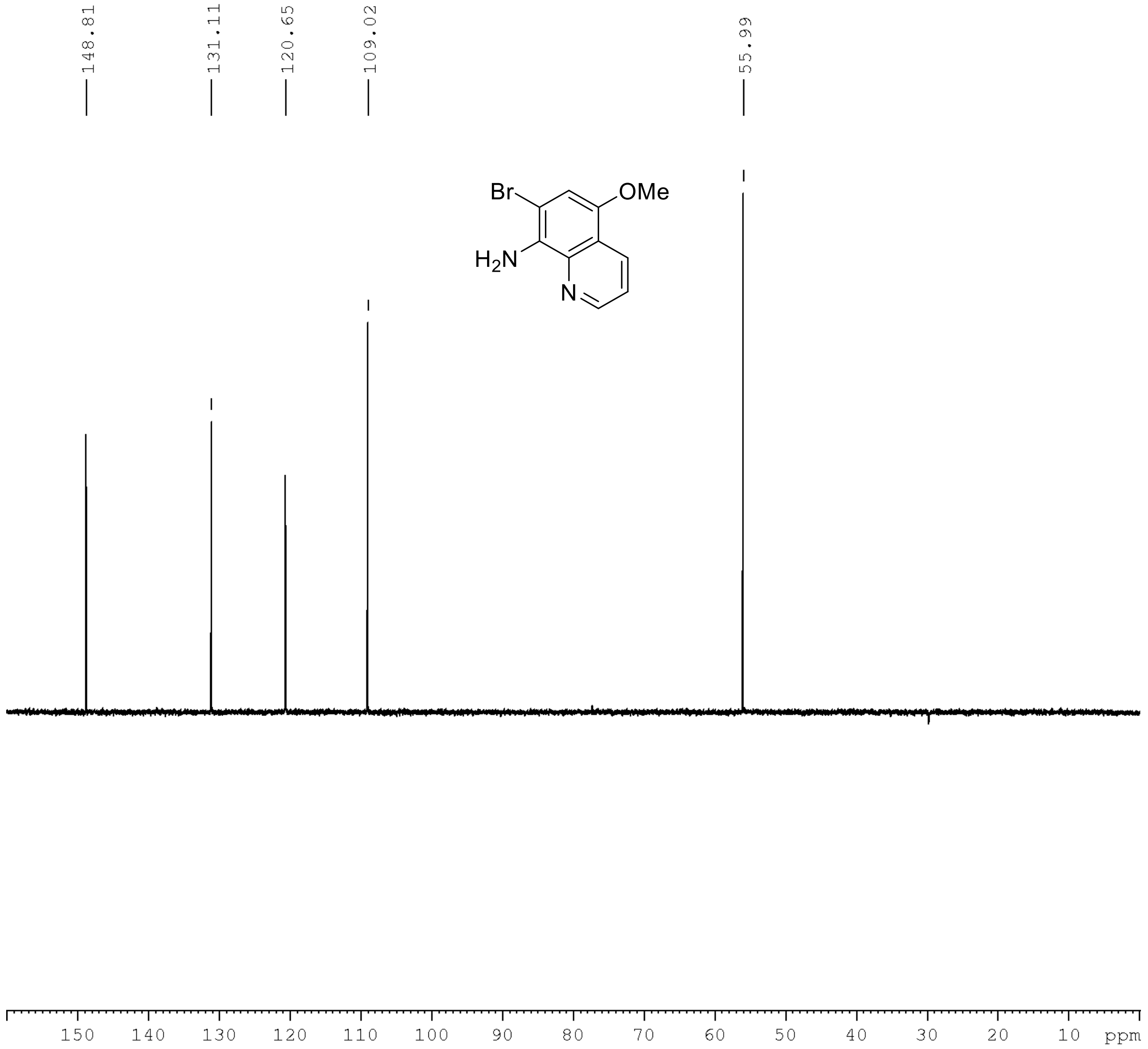

HRMS spectrum of compound **5f:**

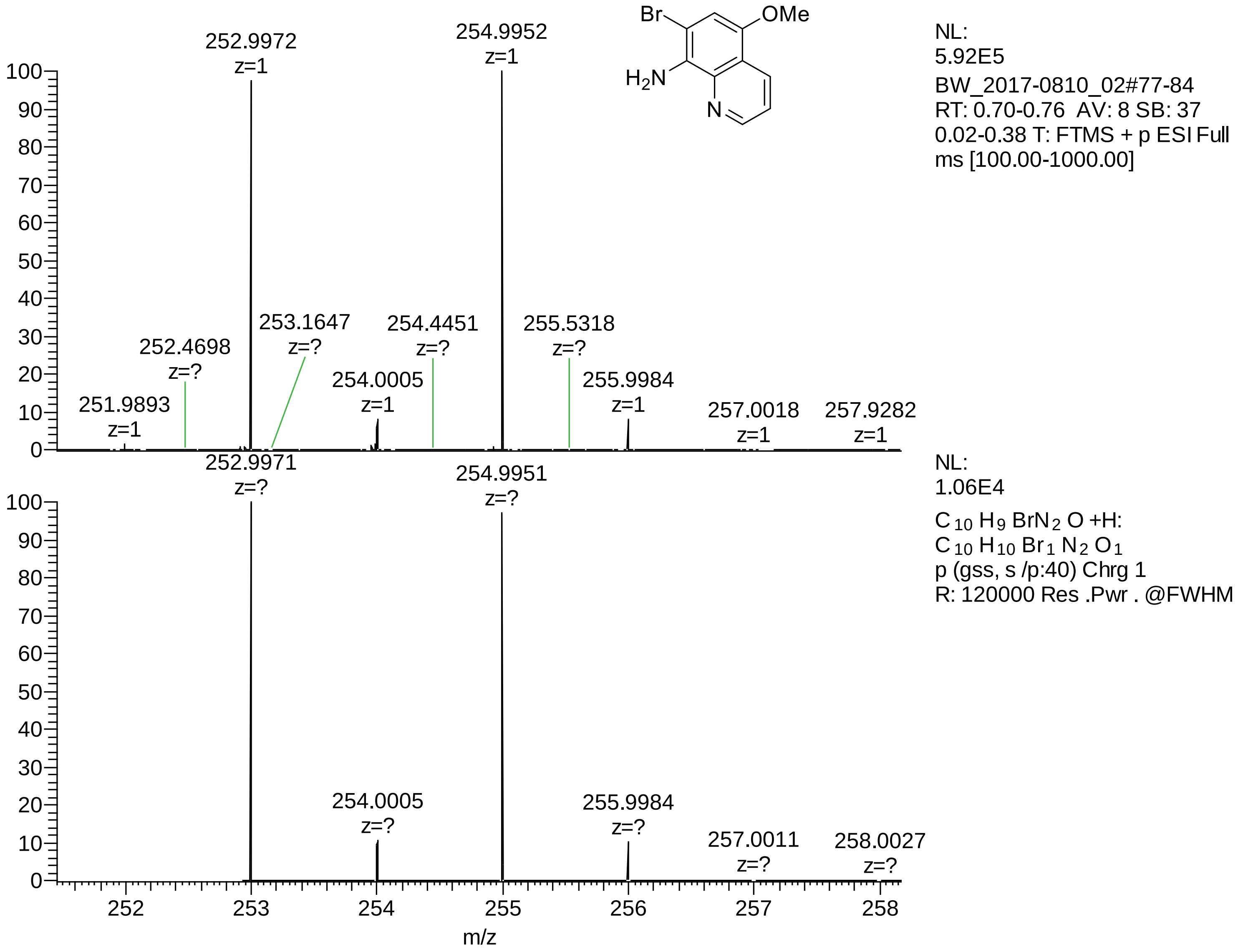

^{1}H NMR spectrum of compound **4g** ($CDCl_3$, 400 MHz):

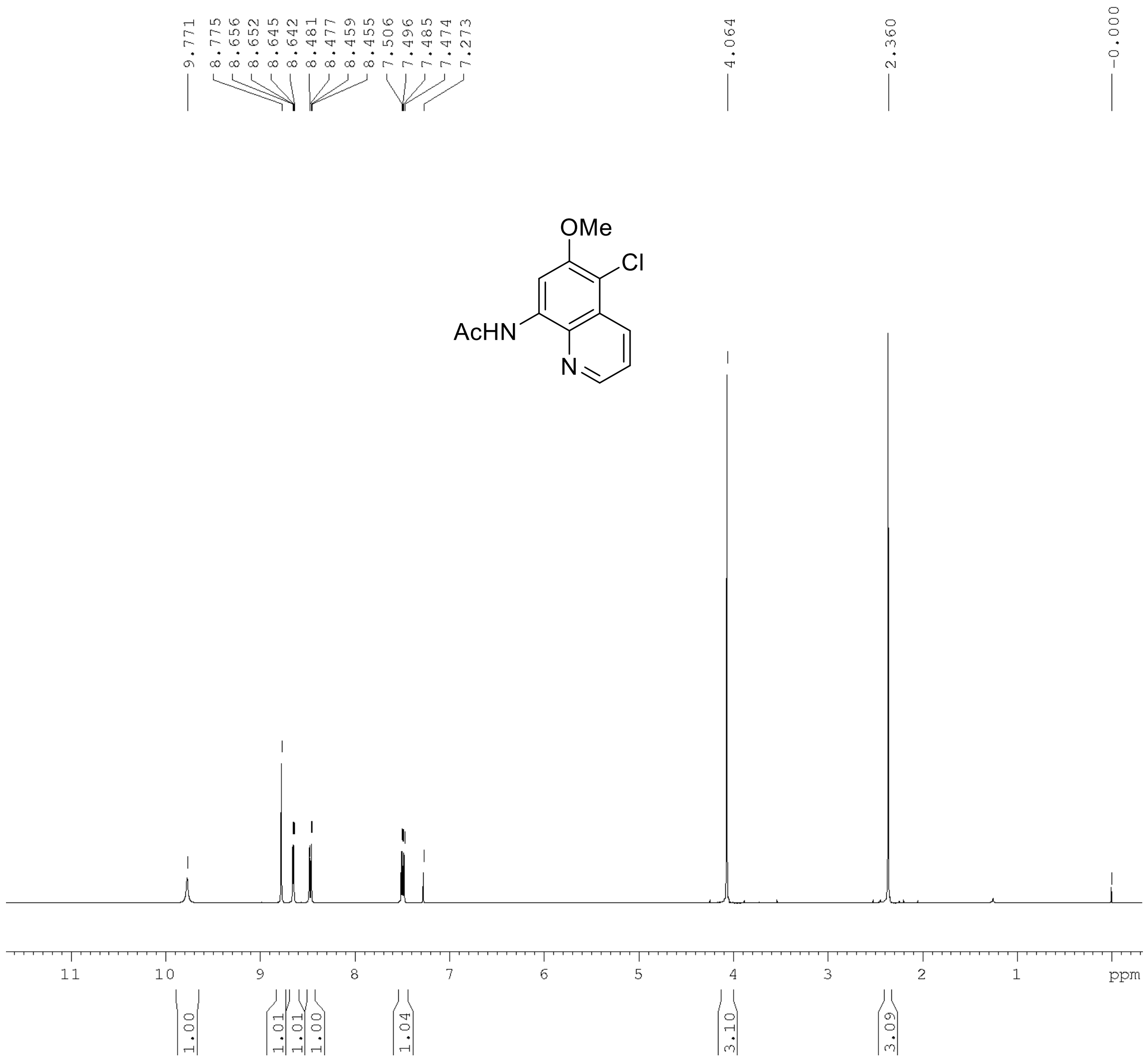

$^{13}C\{^{1}H\}$NMR spectrum of compound **4g** ($CDCl_3$, 100 MHz):

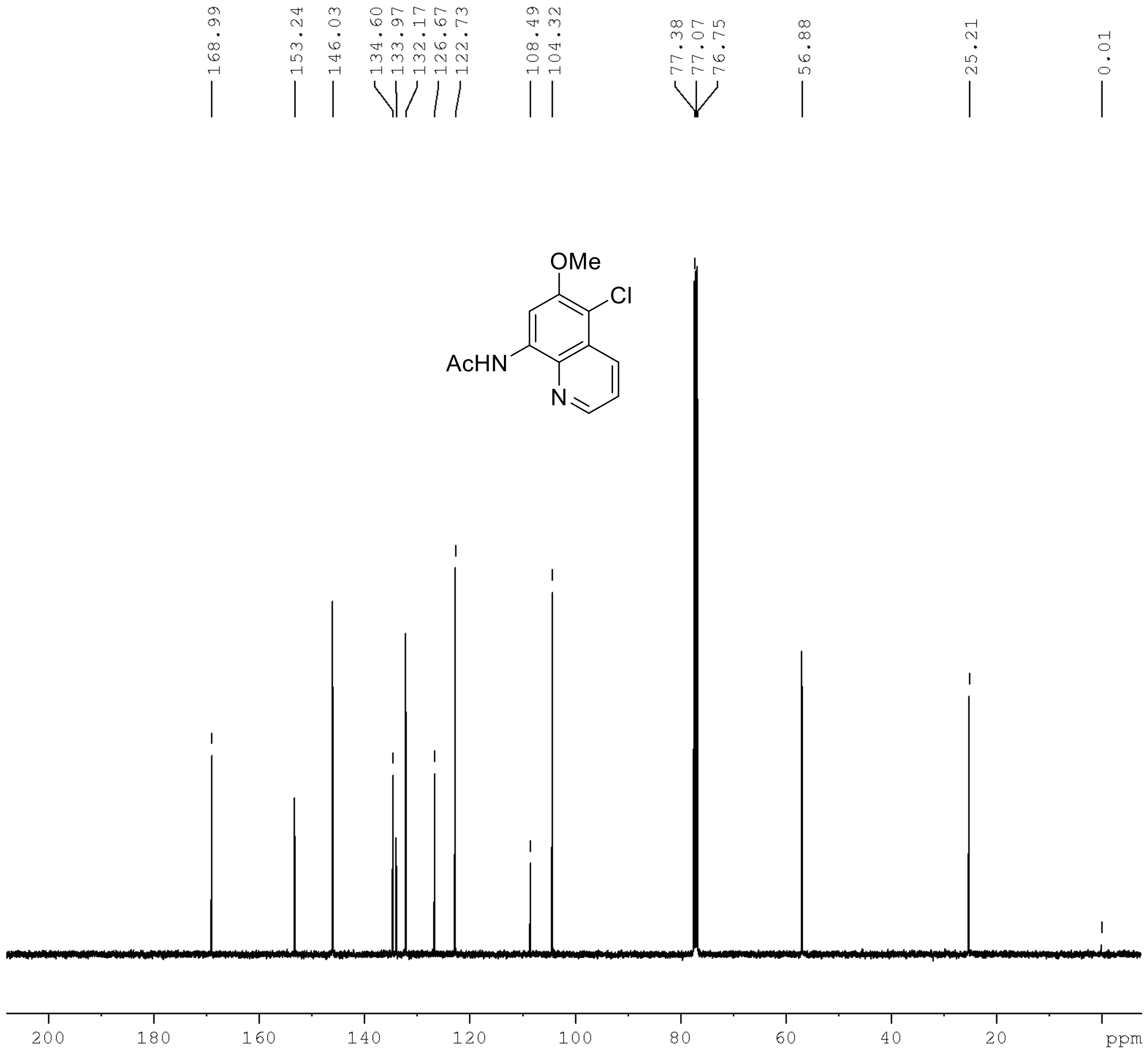

DEPT135 NMR spectrum of compound **4g** ($CDCl_3$, 100 MHz):

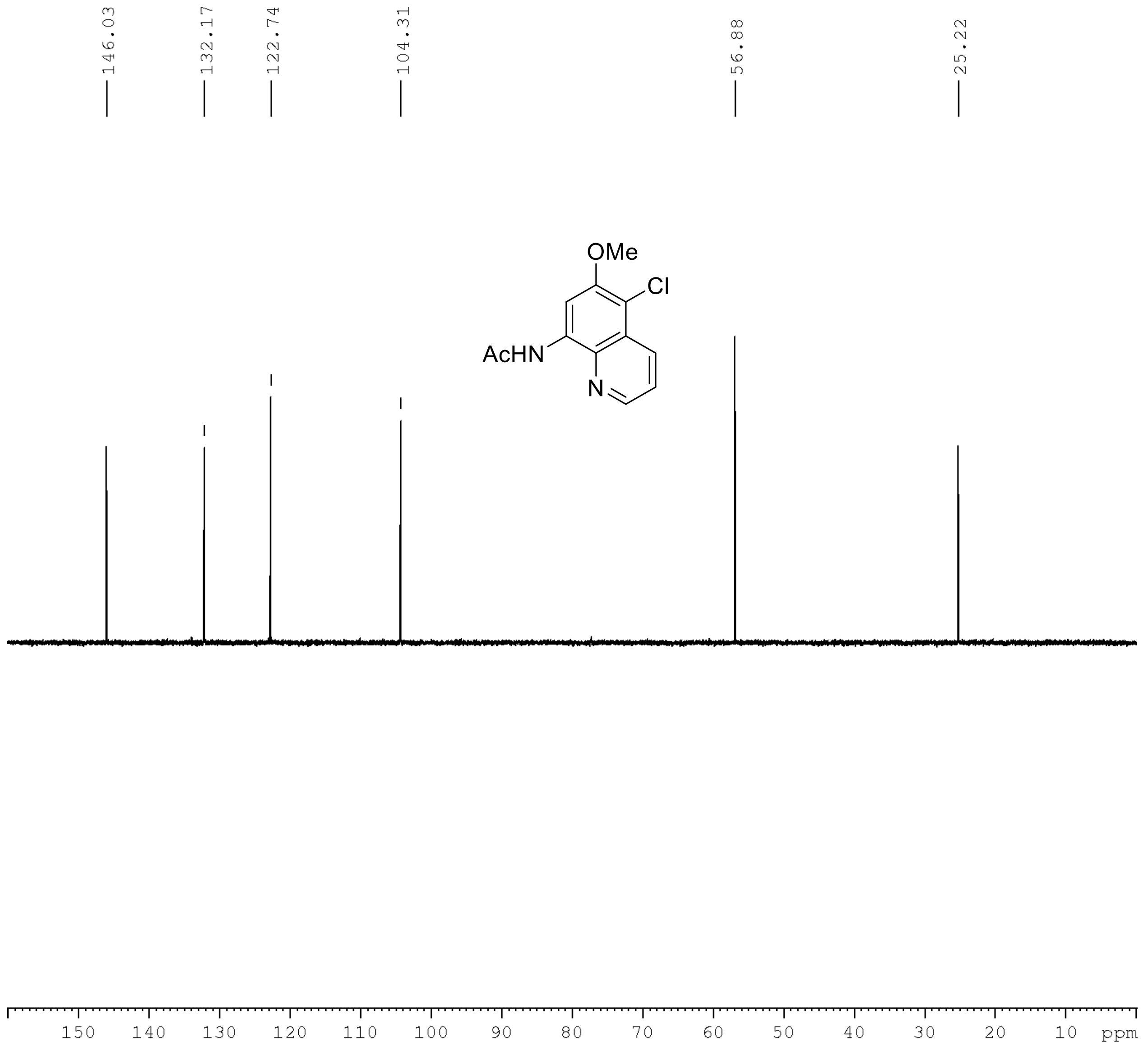

^{1}H NMR spectrum of compound **5g** ($CDCl_3$, 400 MHz):

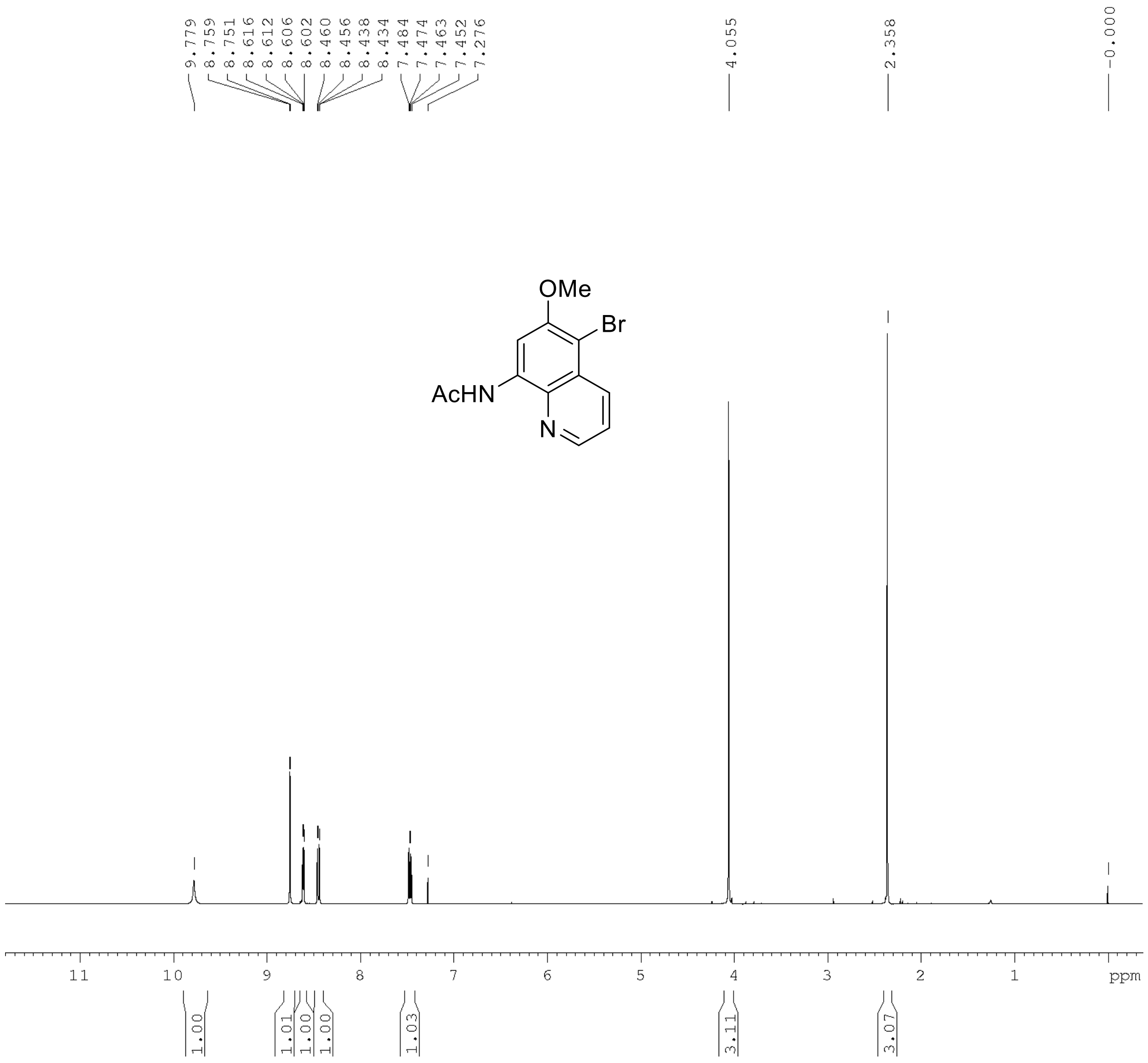

$^{13}C\{^{1}H\}$NMR spectrum of compound **5g** ($CDCl_3$, 100 MHz):

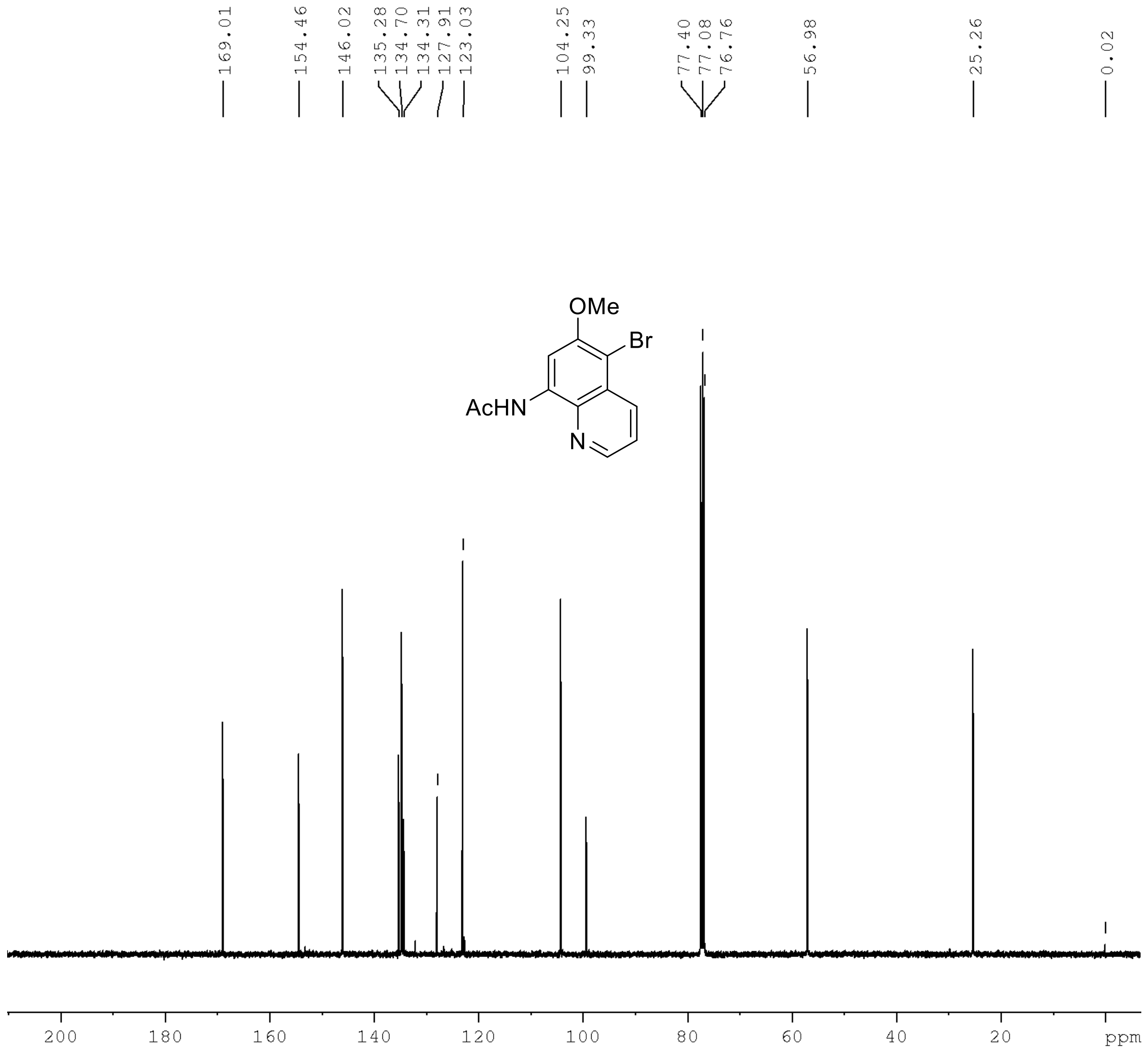

DEPT135 NMR spectrum of compound **5g** ($CDCl_3$, 100 MHz):

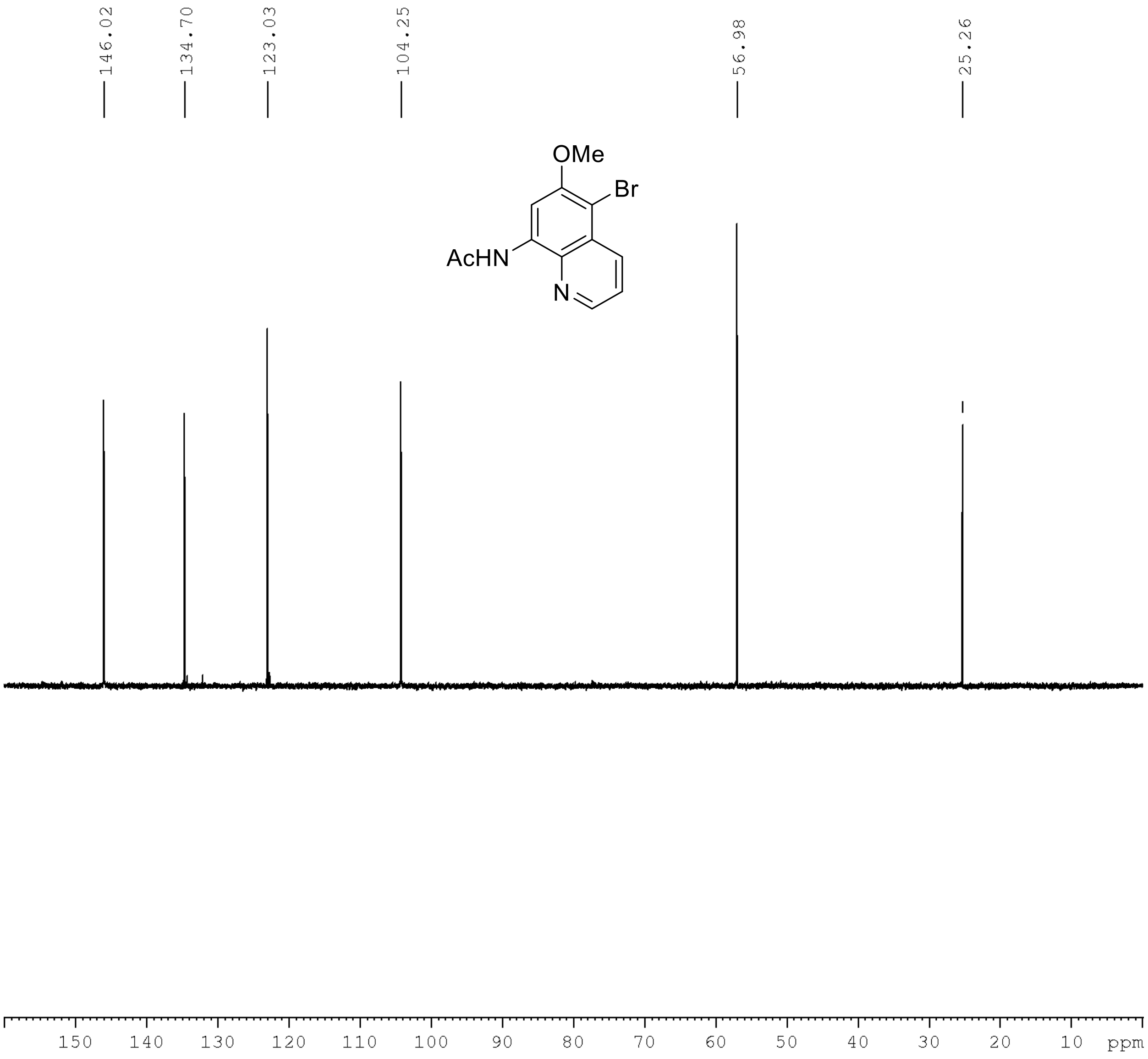

^{1}H NMR spectrum of compound **4h** ($CDCl_3$, 400 MHz):

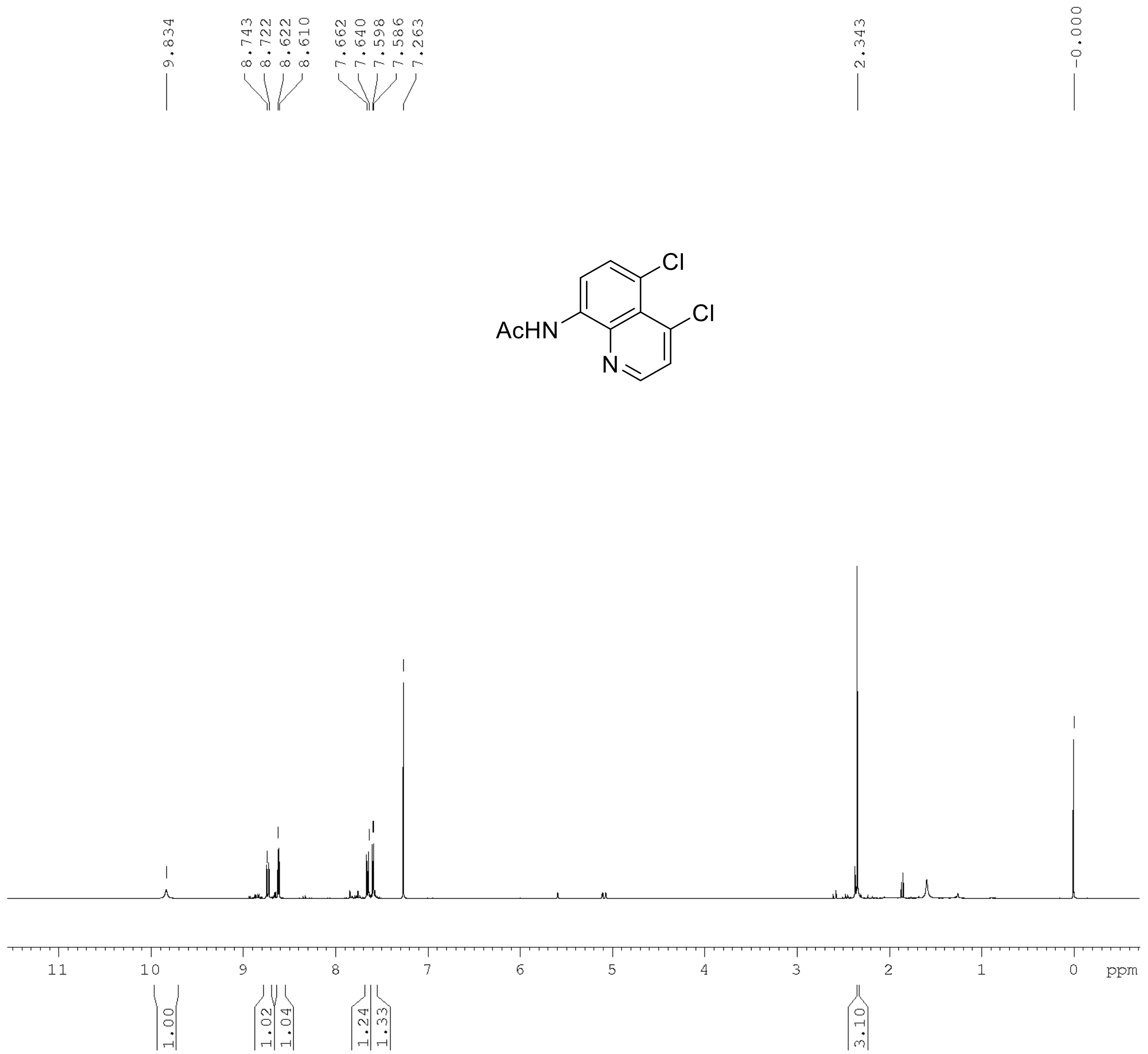

$^{13}C\{^{1}H\}$NMR spectrum of compound **4h** ($CDCl_3$, 100 MHz):

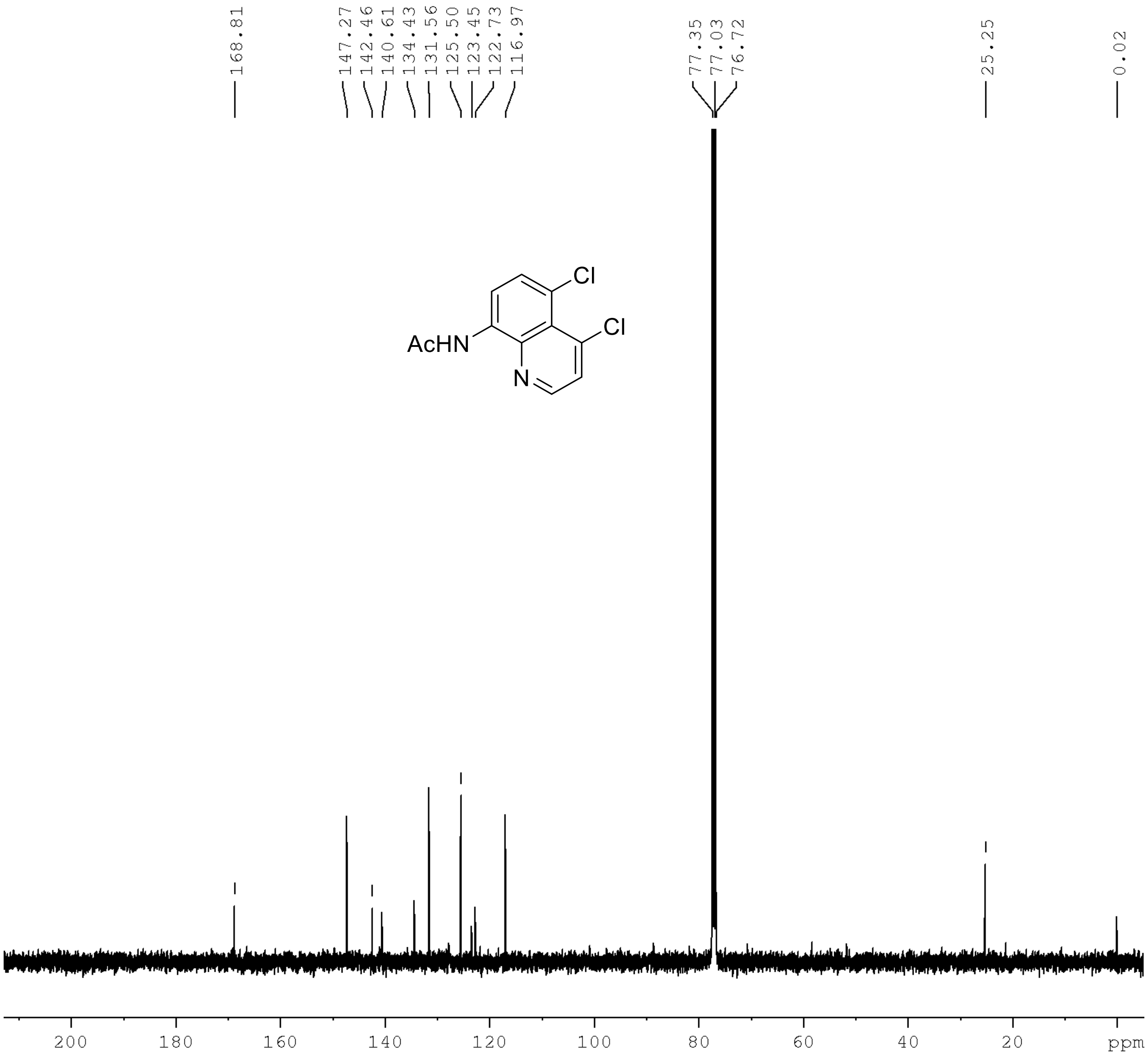

DEPT135 NMR spectrum of compound **4h** ($CDCl_3$, 100 MHz):

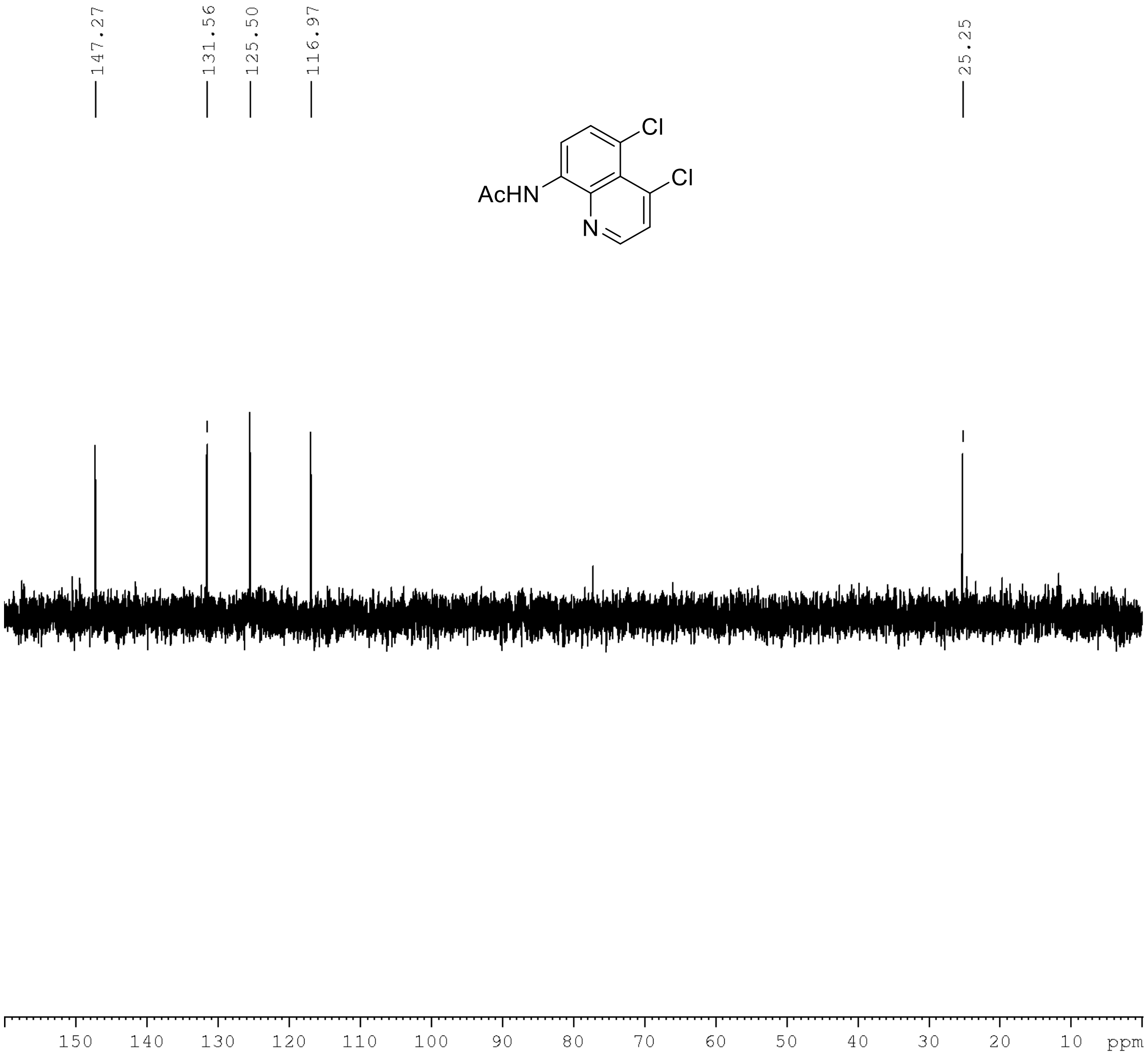

^{1}H NMR spectrum of compound **5h** ($CDCl_3$, 400 MHz):

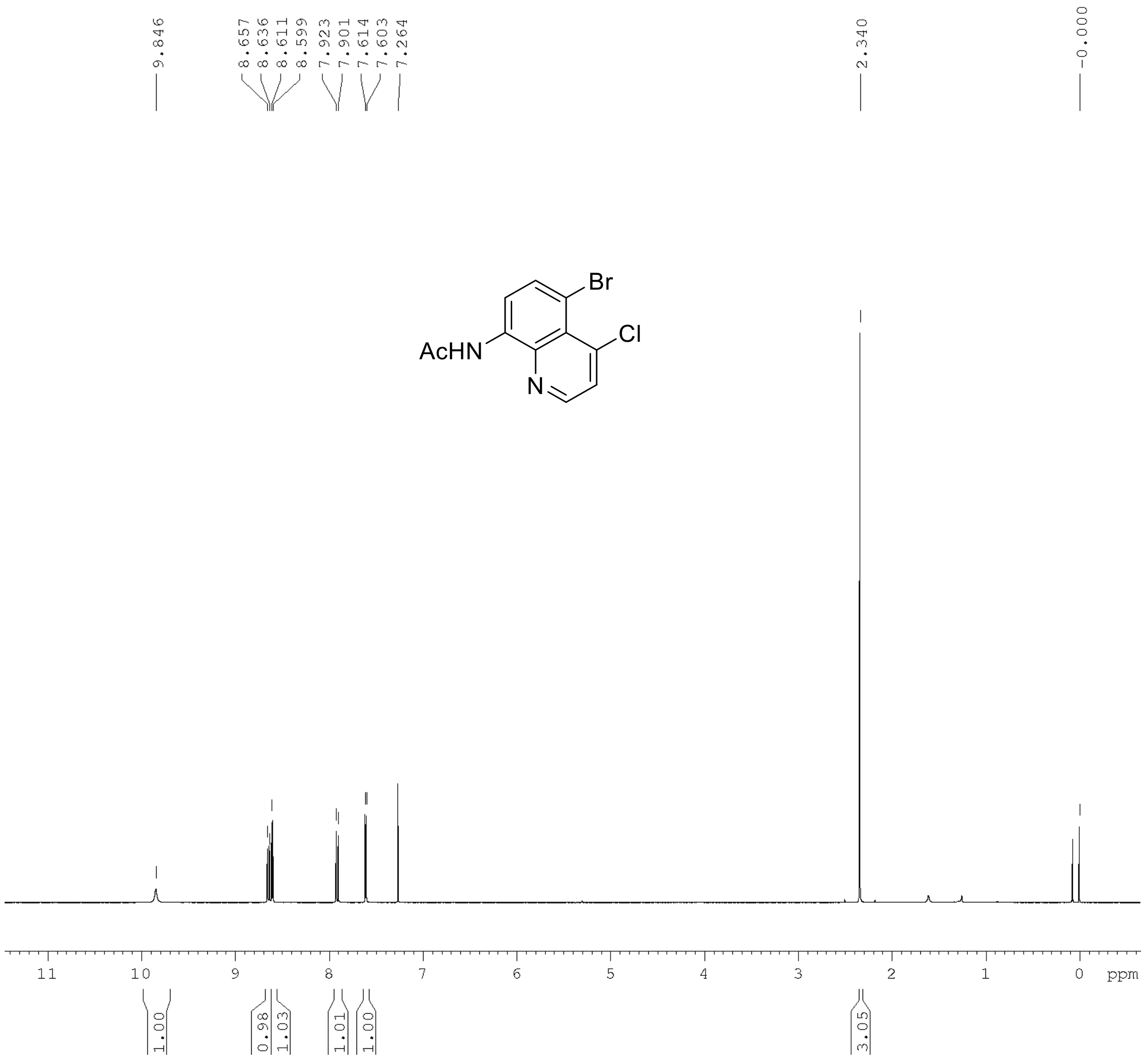

$^{13}C\{^{1}H\}$NMR spectrum of compound **5h** ($CDCl_3$, 100 MHz):

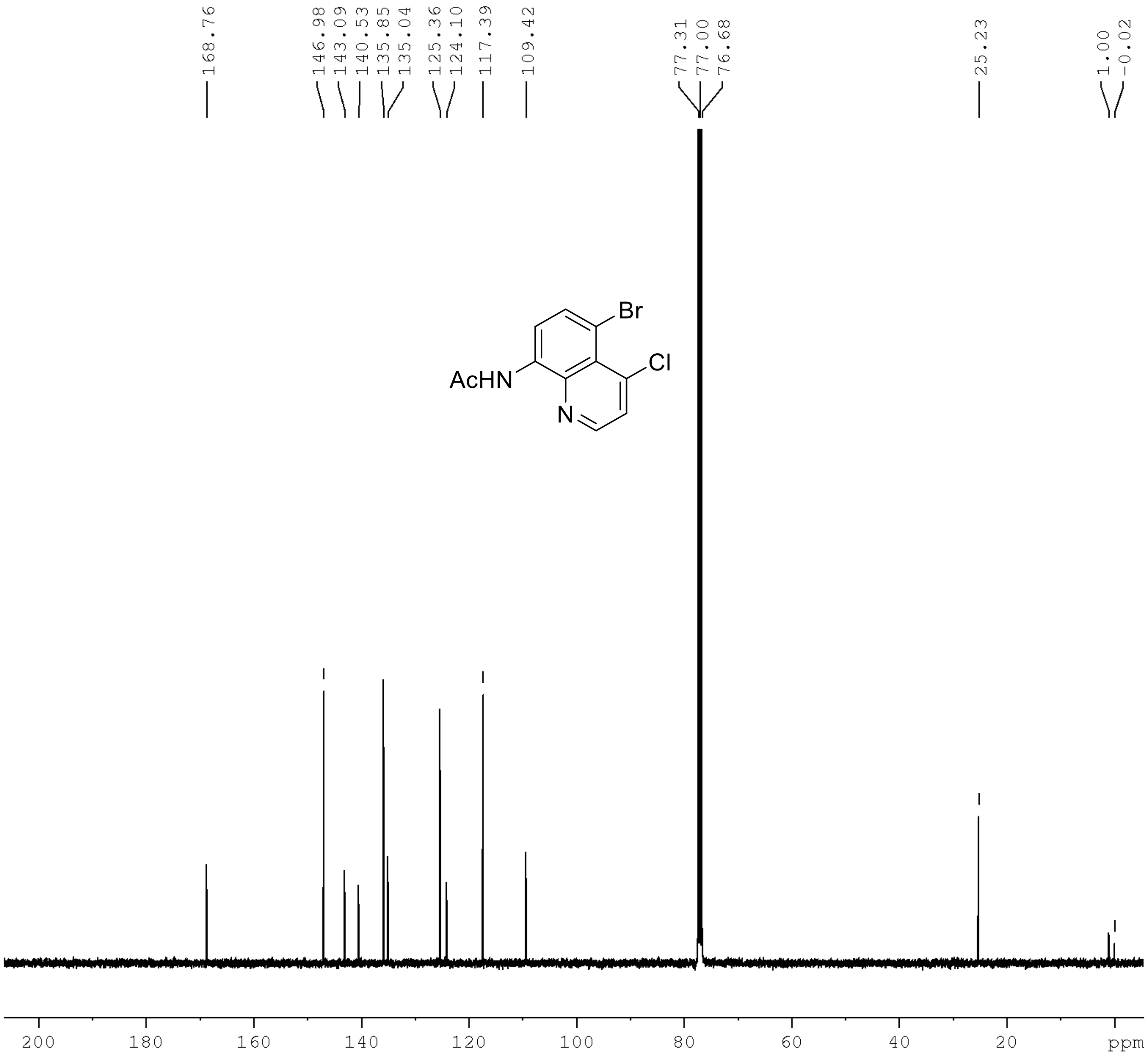

DEPT135 NMR spectrum of compound **5h** ($CDCl_3$, 100 MHz):

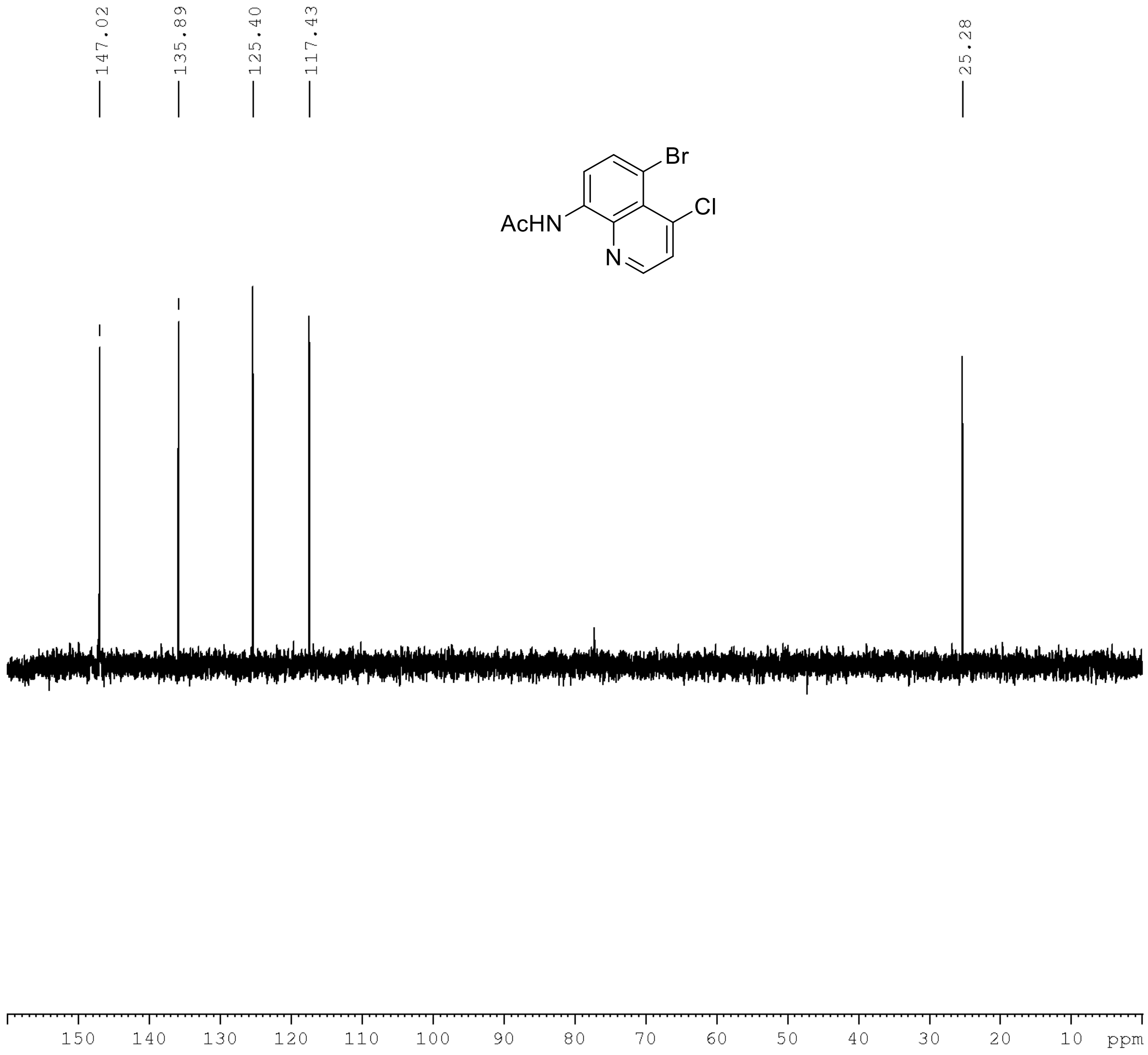

^{1}H NMR spectrum of compound **4i** ($CDCl_3$, 400 MHz):

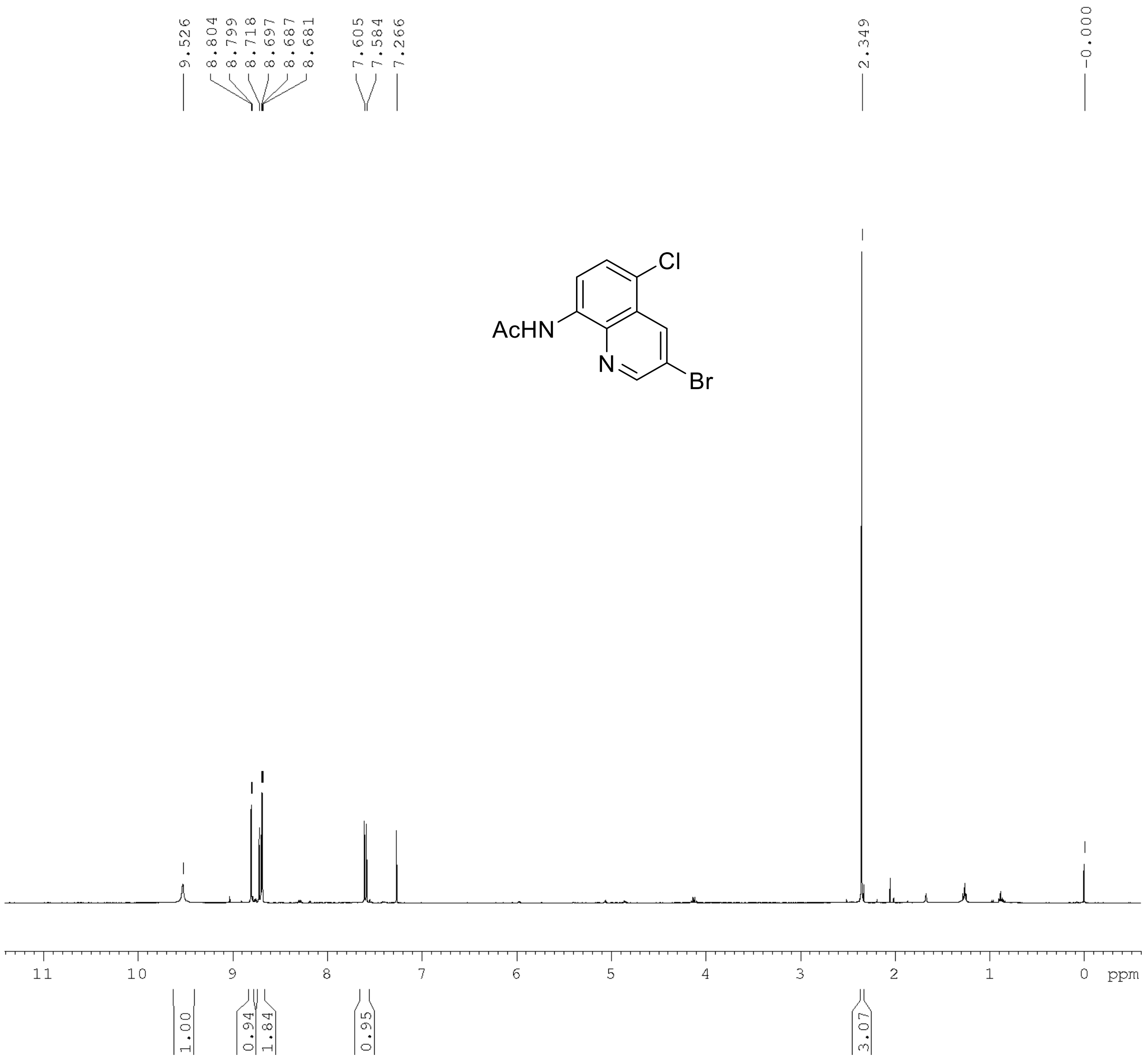

$^{13}C\{^1H\}$NMR spectrum of compound **4i** ($CDCl_3$, 100 MHz):

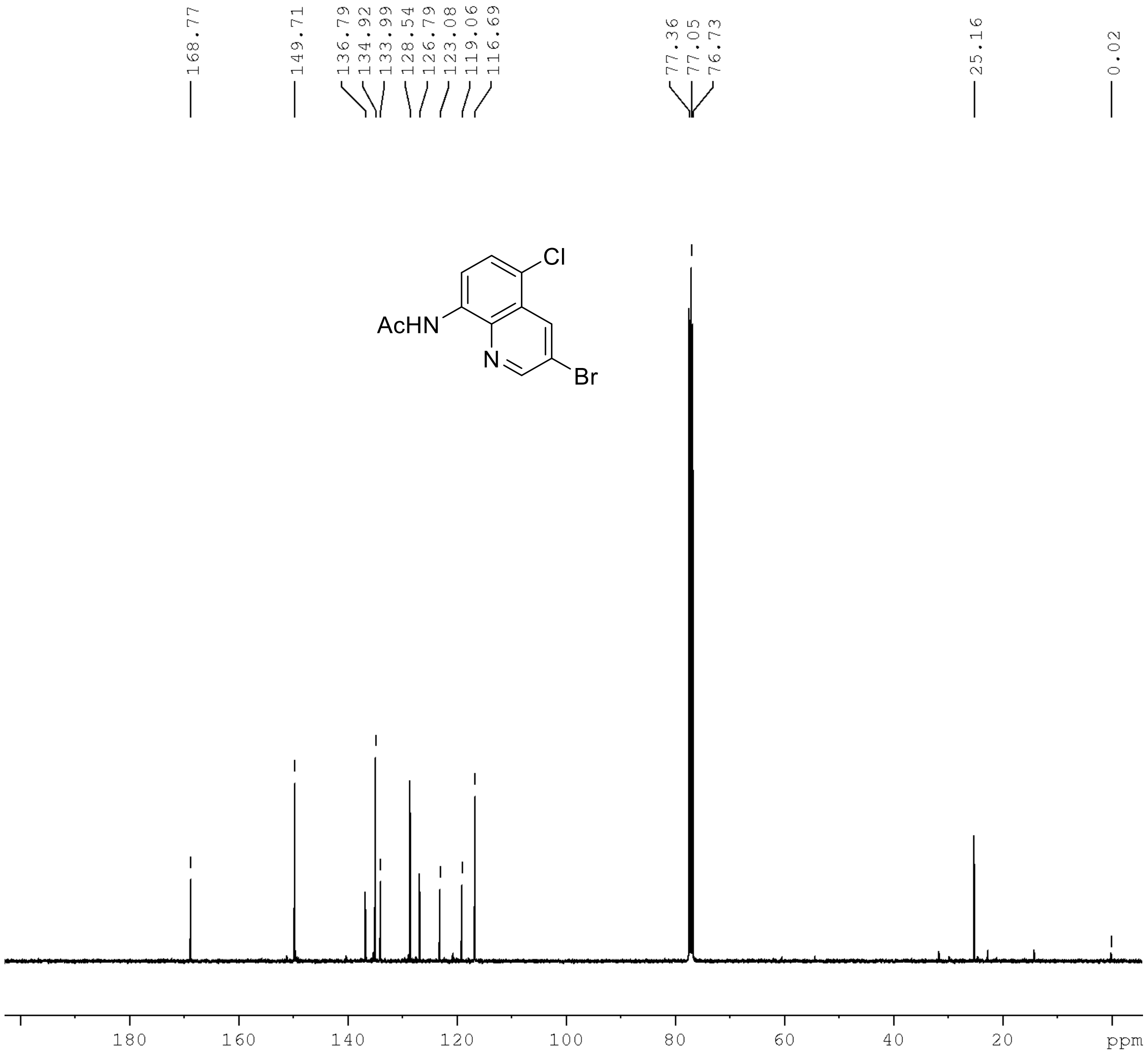

DEPT135 NMR spectrum of compound **4i** ($CDCl_3$, 100 MHz):

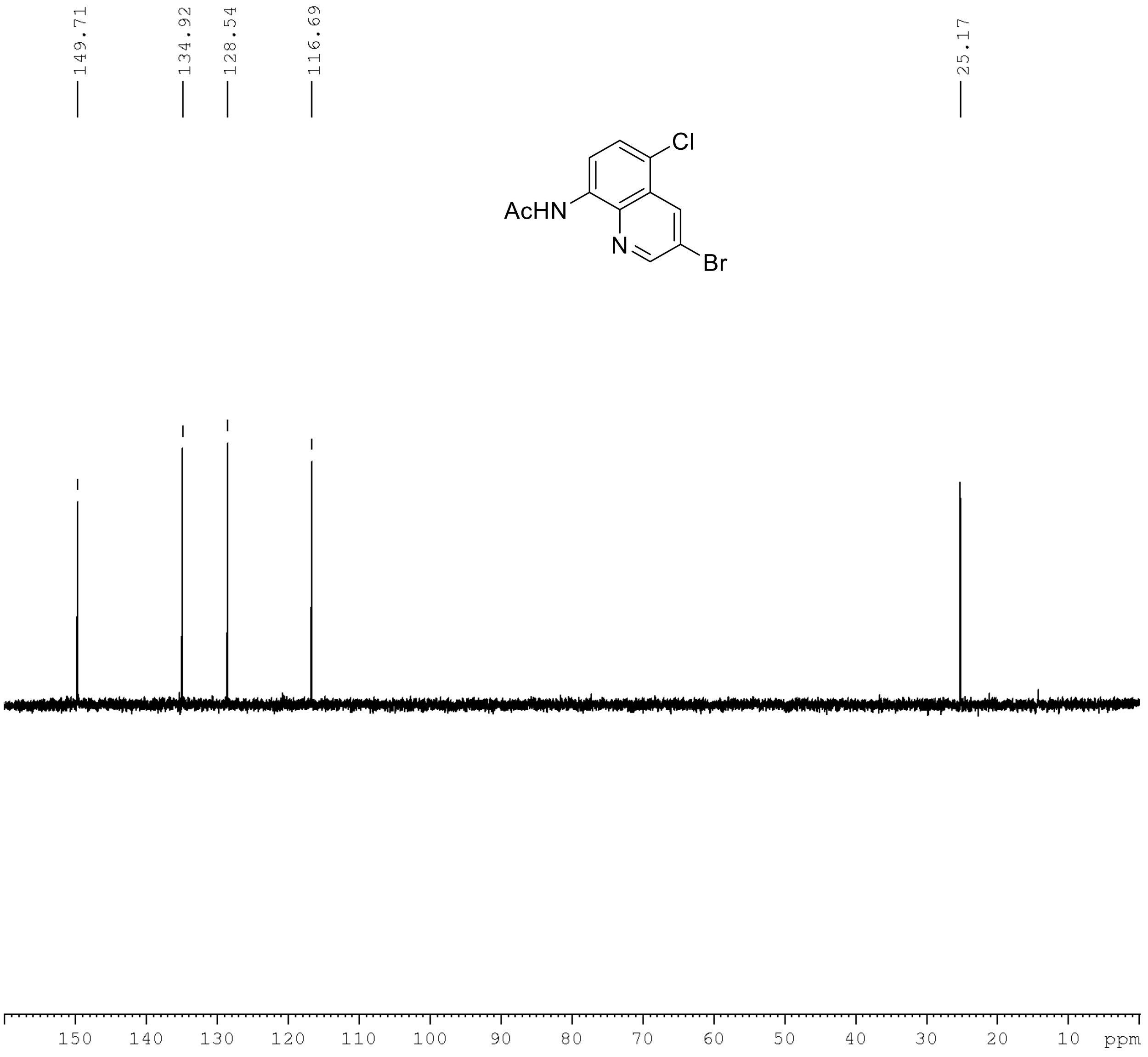

^{1}H NMR spectrum of compound **5i** ($CDCl_3$, 400 MHz):

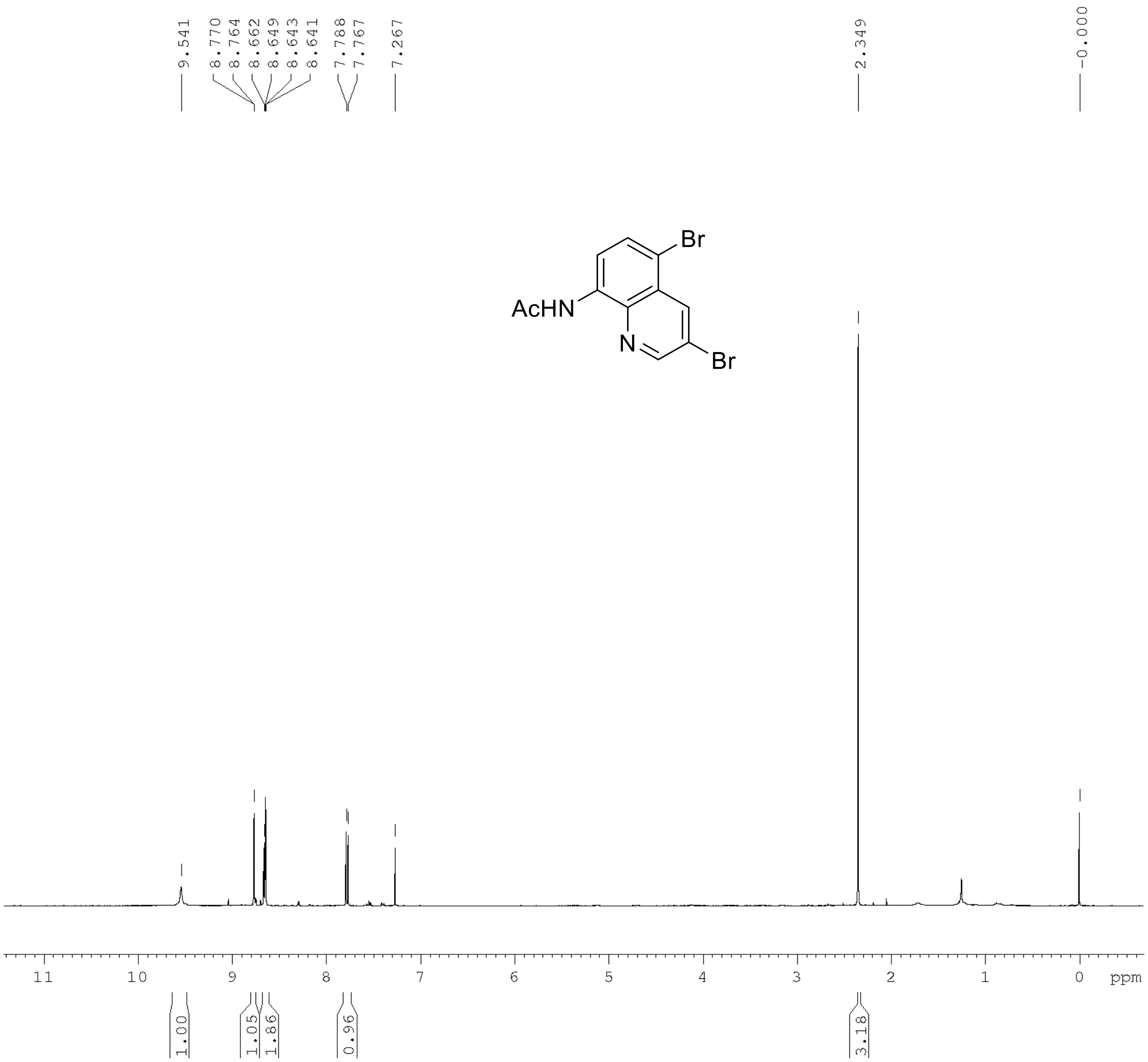

$^{13}C\{^{1}H\}$NMR spectrum of compound **5i** ($CDCl_3$, 100 MHz):

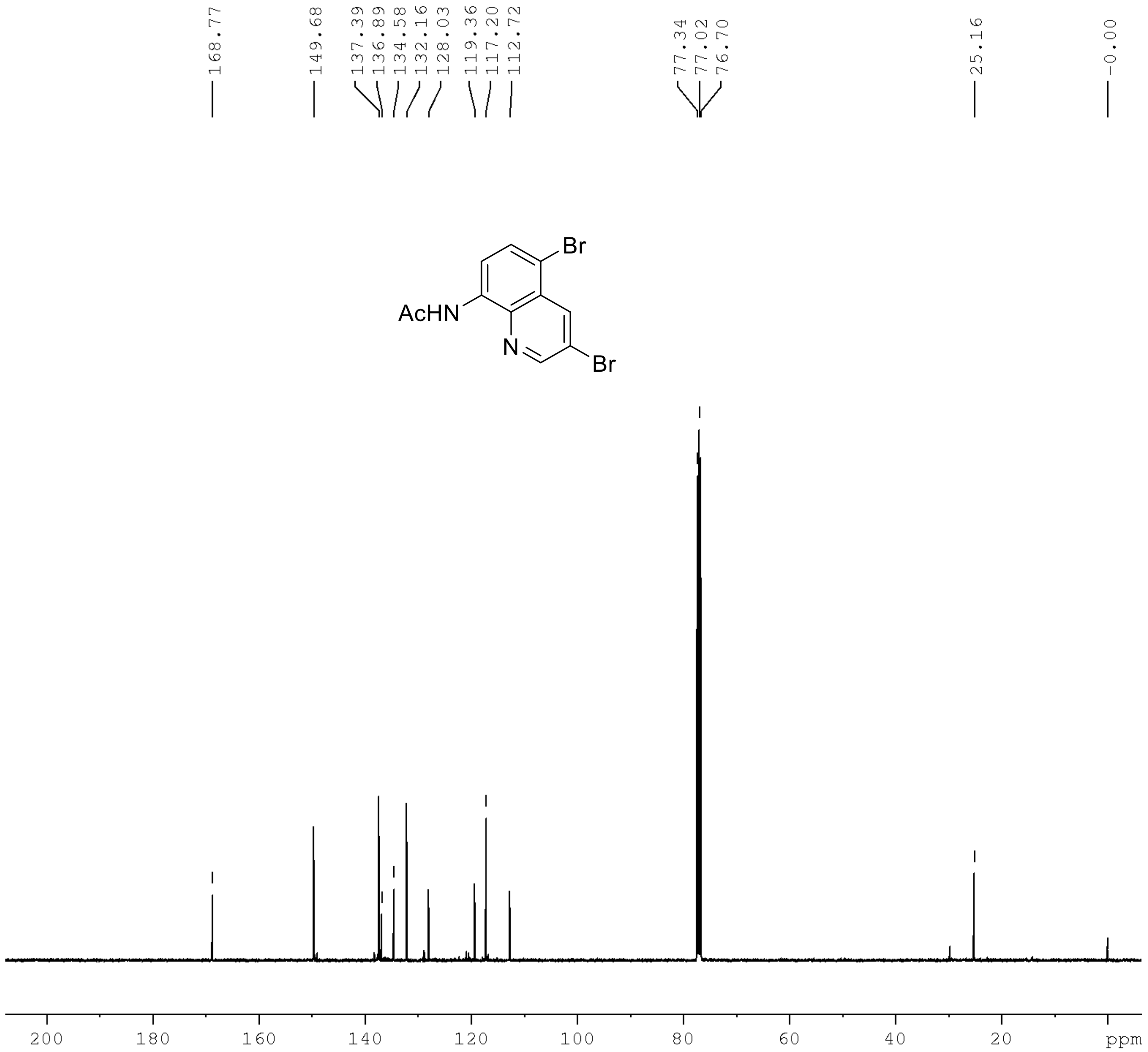

DEPT135 NMR spectrum of compound **5i** ($CDCl_3$, 100 MHz):

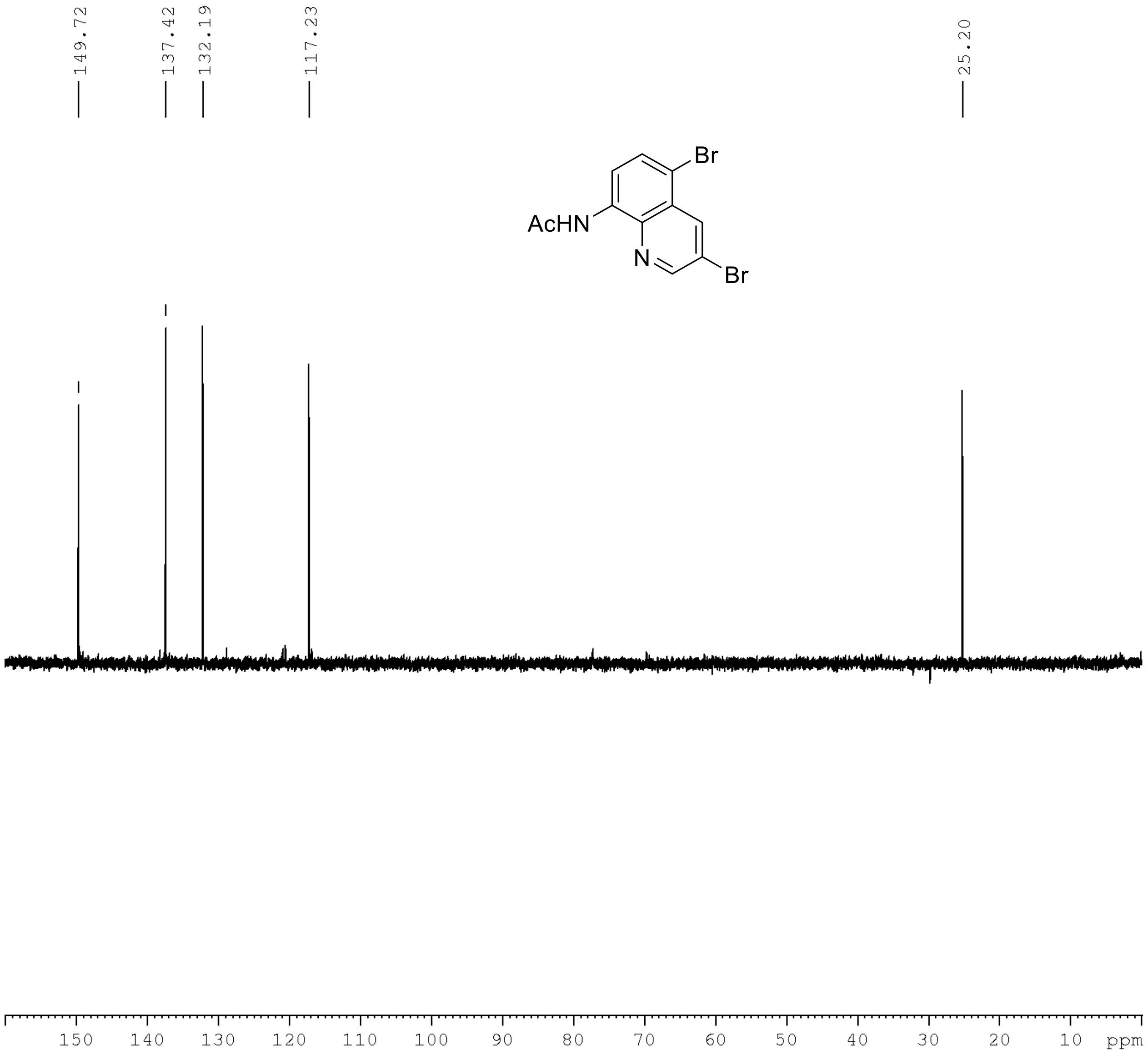

^{1}H NMR spectrum of compound **6a** ($CDCl_3$, 400 MHz)**:**

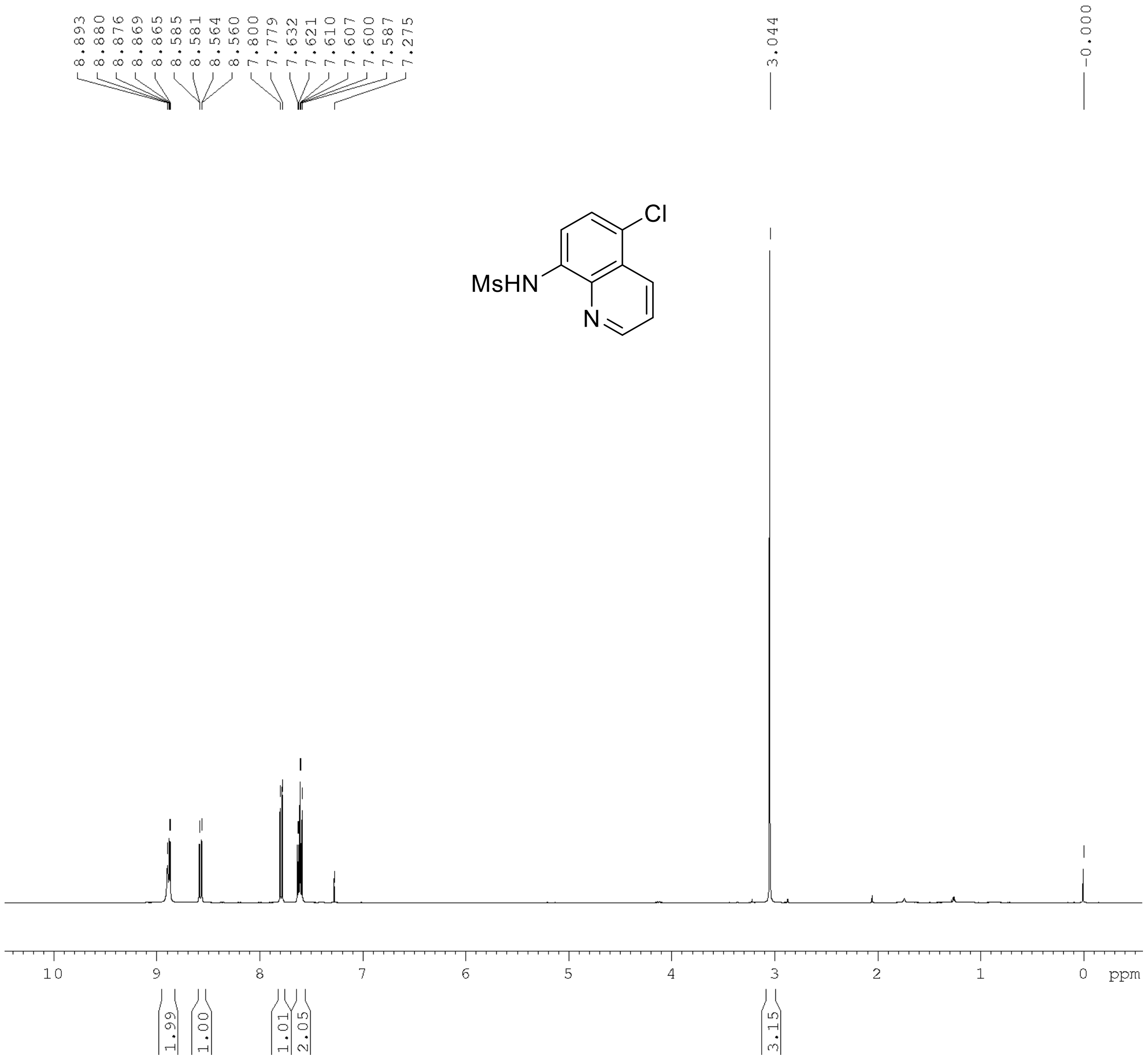

$^{13}C\{^1H\}$NMR spectrum of compound **6a** ($CDCl_3$, 100 MHz)**:**

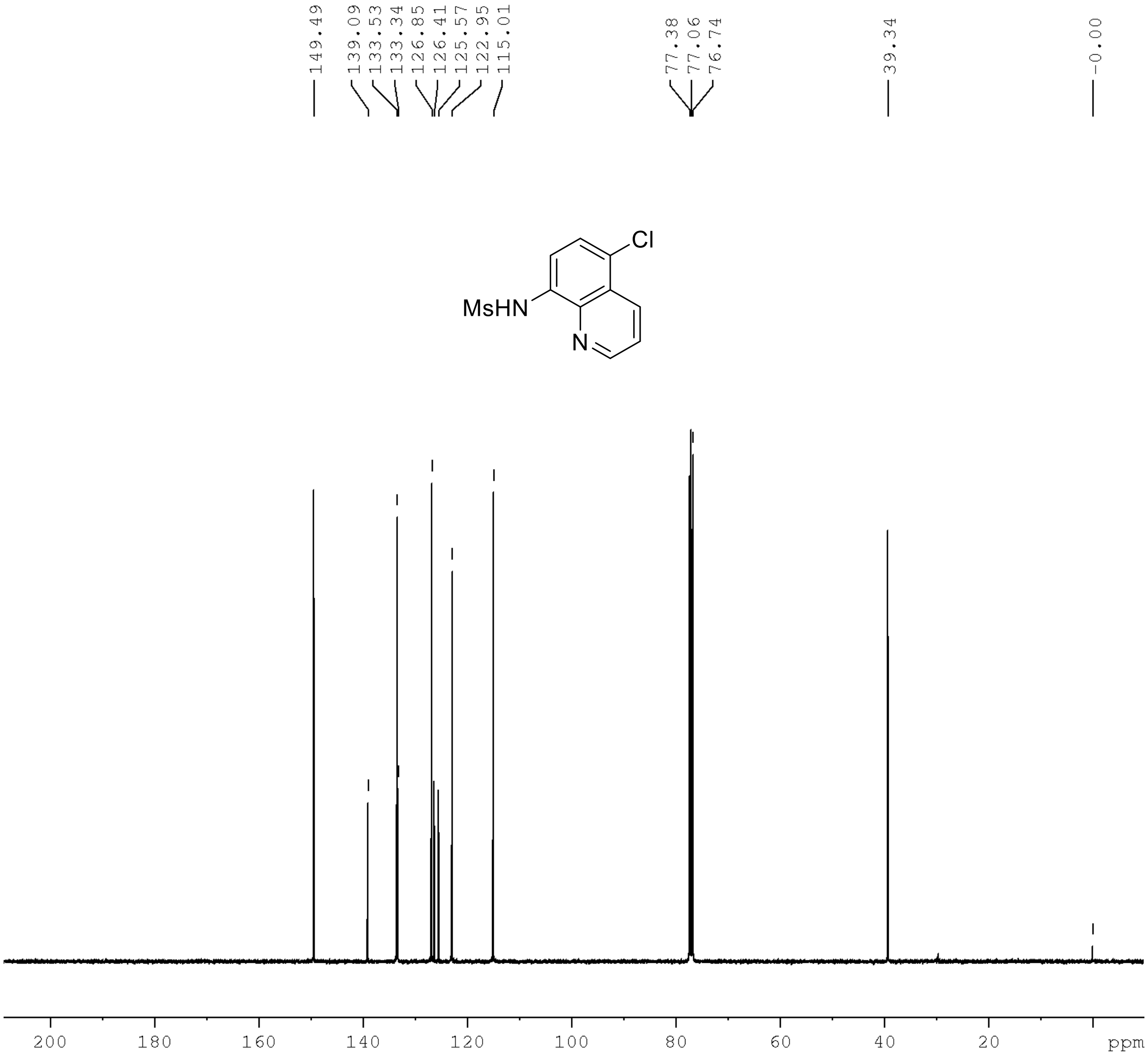

DEPT135 spectrum of compound **6a** ($CDCl_3$, 100 MHz)**:**

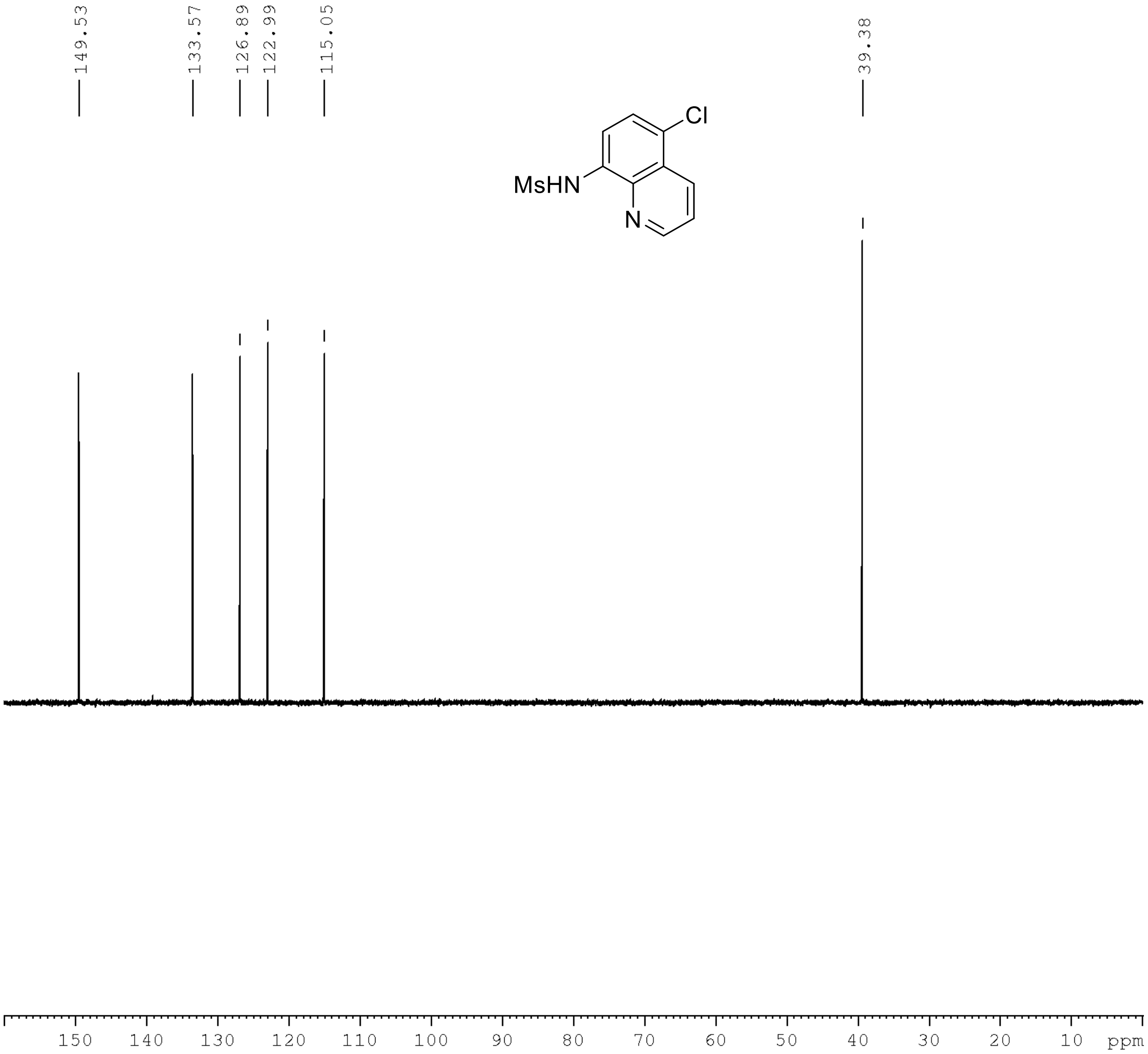

^{1}H NMR spectrum of compound **7a** ($CDCl_3$, 400 MHz)**:**

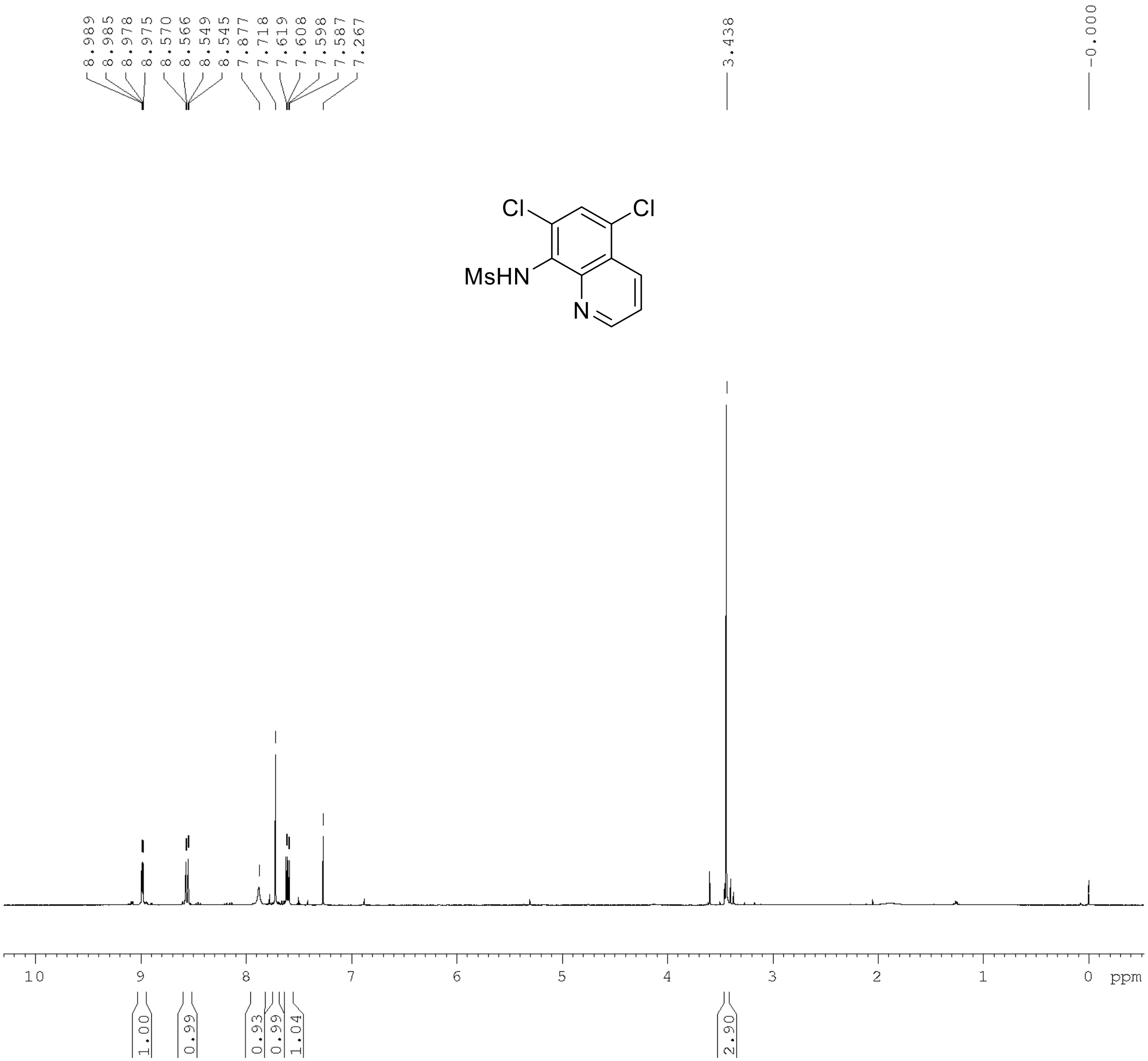

$^{13}C\{^1H\}$NMR spectrum of compound **7a** ($CDCl_3$, 100 MHz)**:**

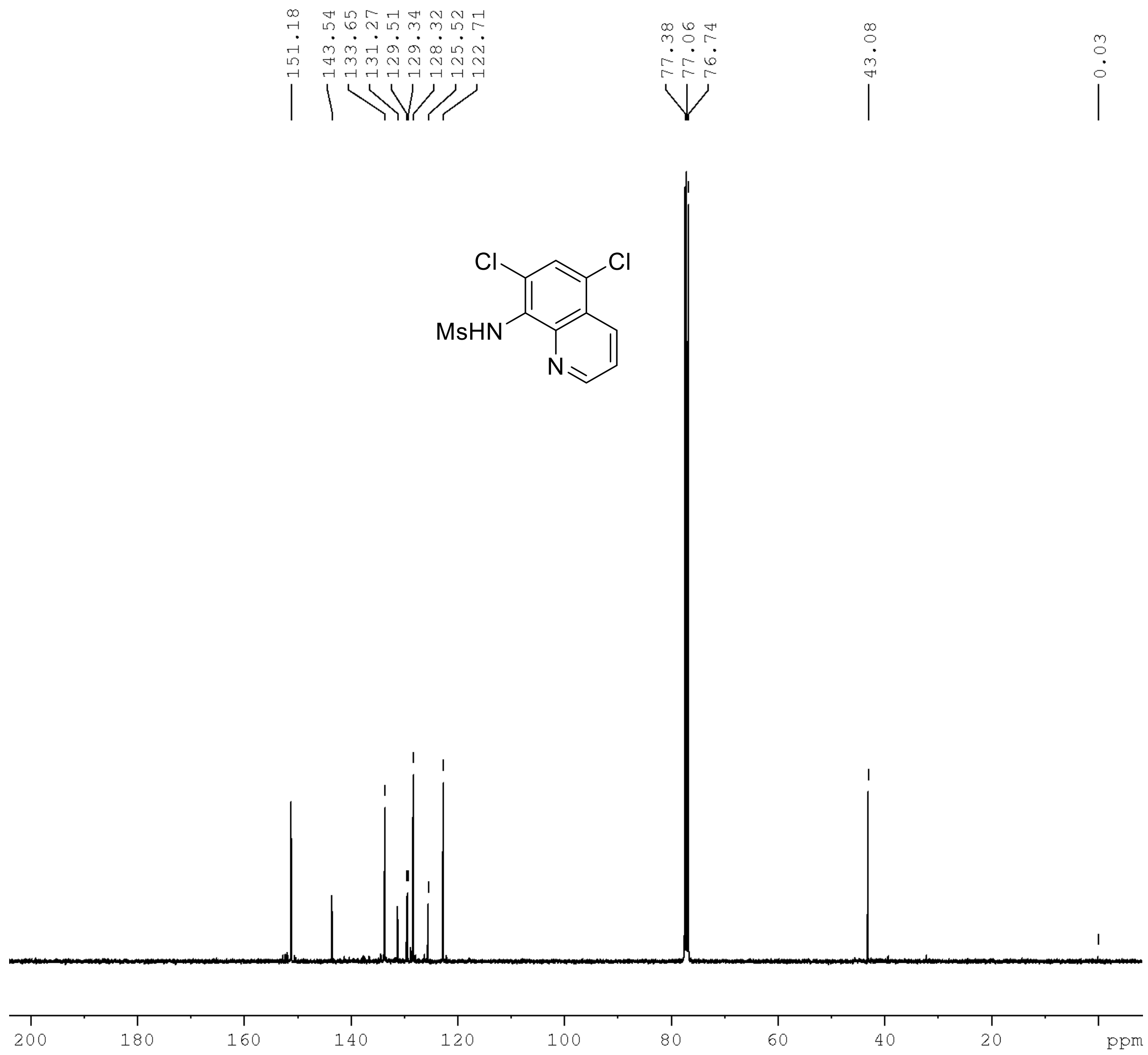

DEPT135 spectrum of compound **7a** ($CDCl_3$, 100 MHz)**:**

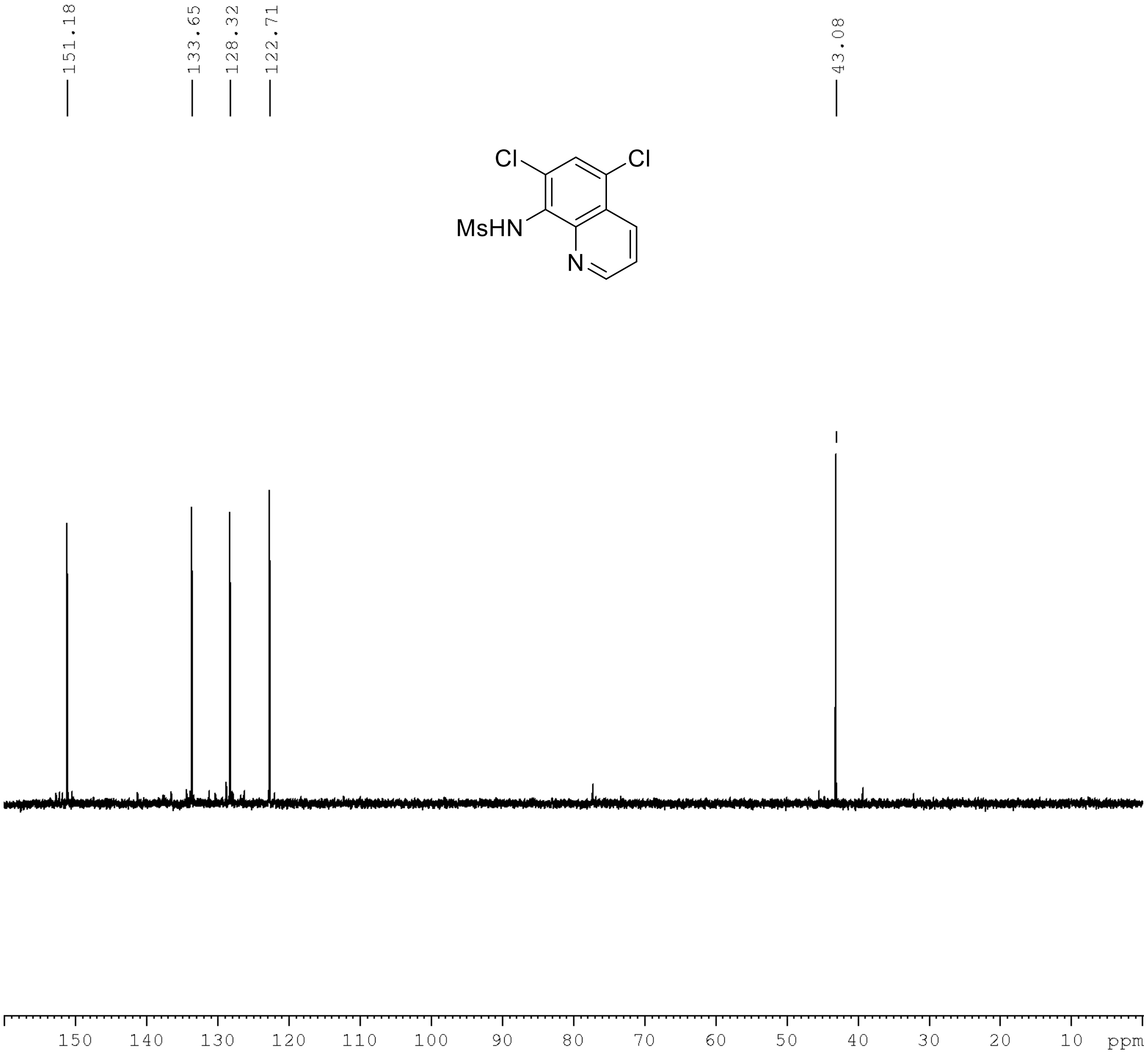

^{1}H NMR spectrum of compound **8a** ($CDCl_3$, 400 MHz)**:**

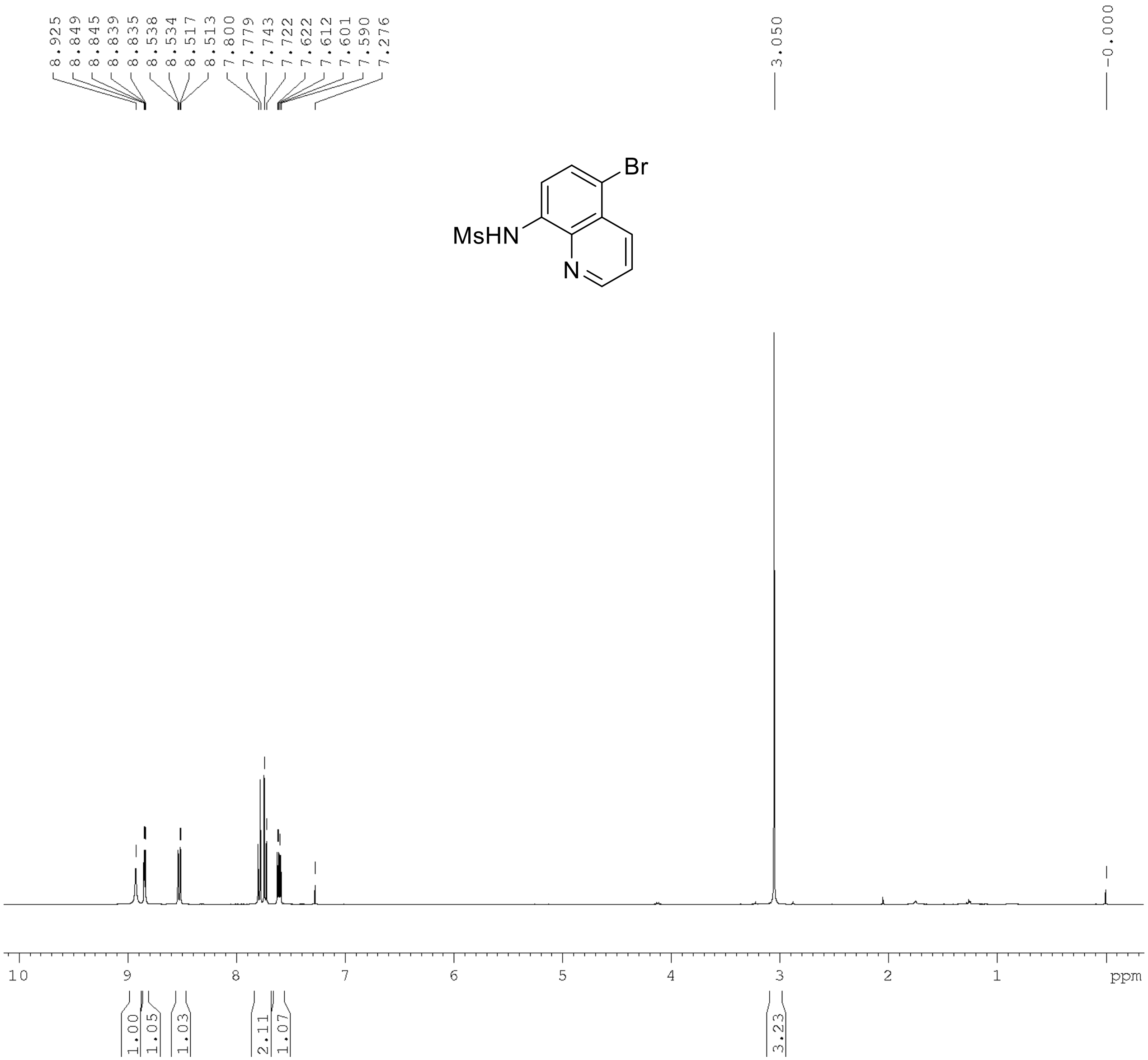

$^{13}C\{^1H\}$NMR spectrum of compound **8a** ($CDCl_3$, 100 MHz):

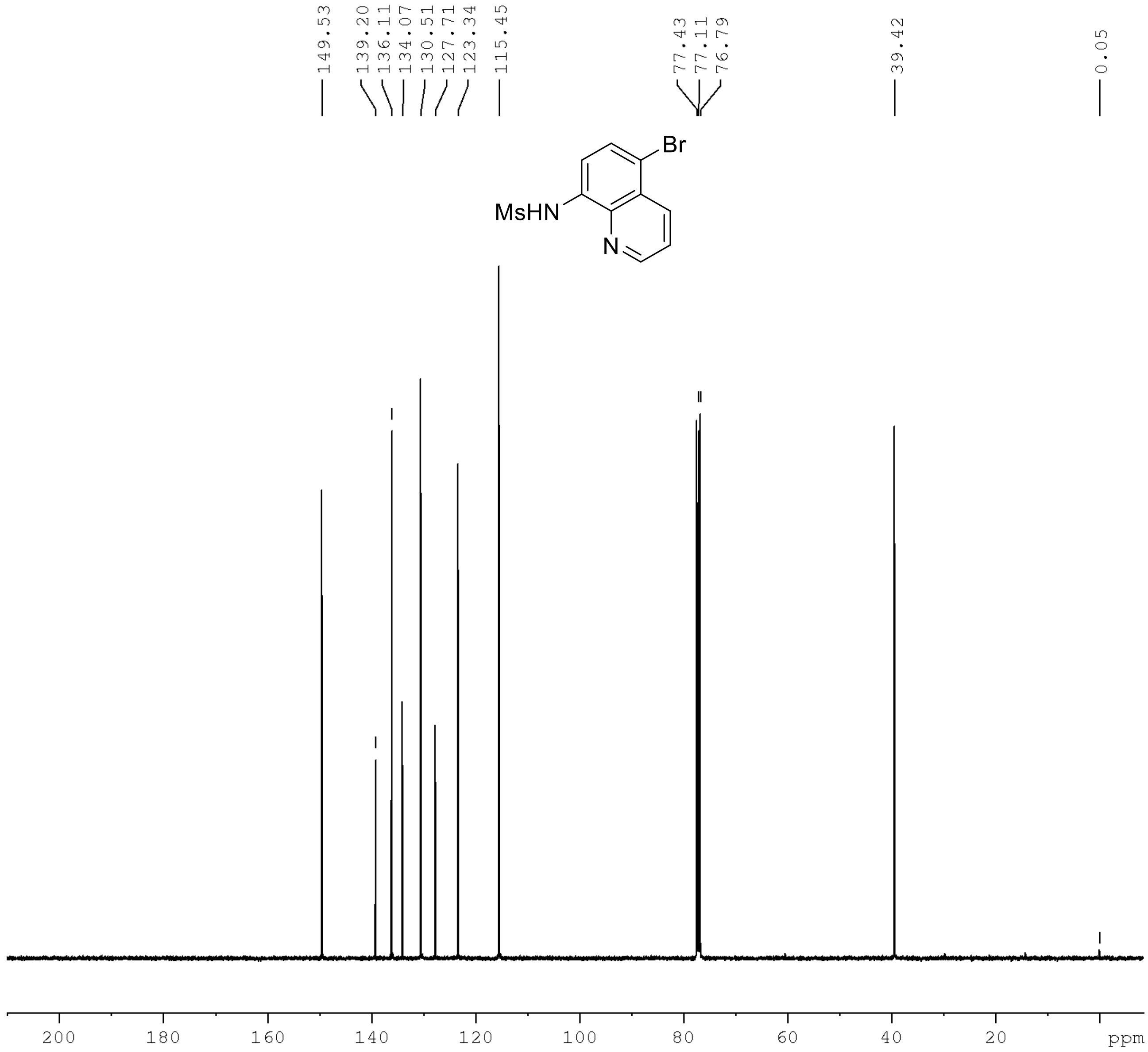

DEPT135 spectrum of compound **8a** ($CDCl_3$, 100 MHz):

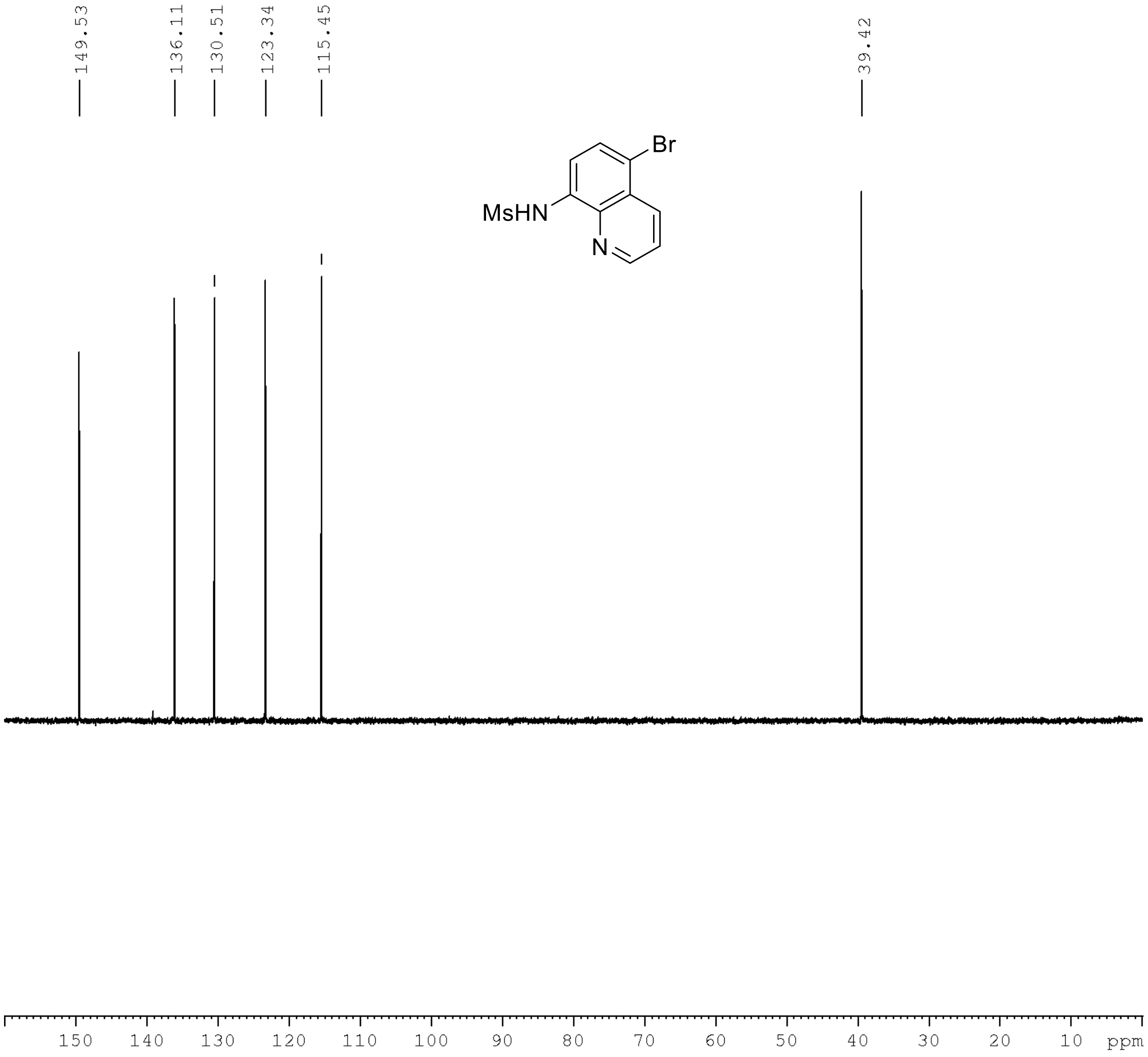

^{1}H NMR spectrum of compound **9a** ($CDCl_3$, 400 MHz)**:**

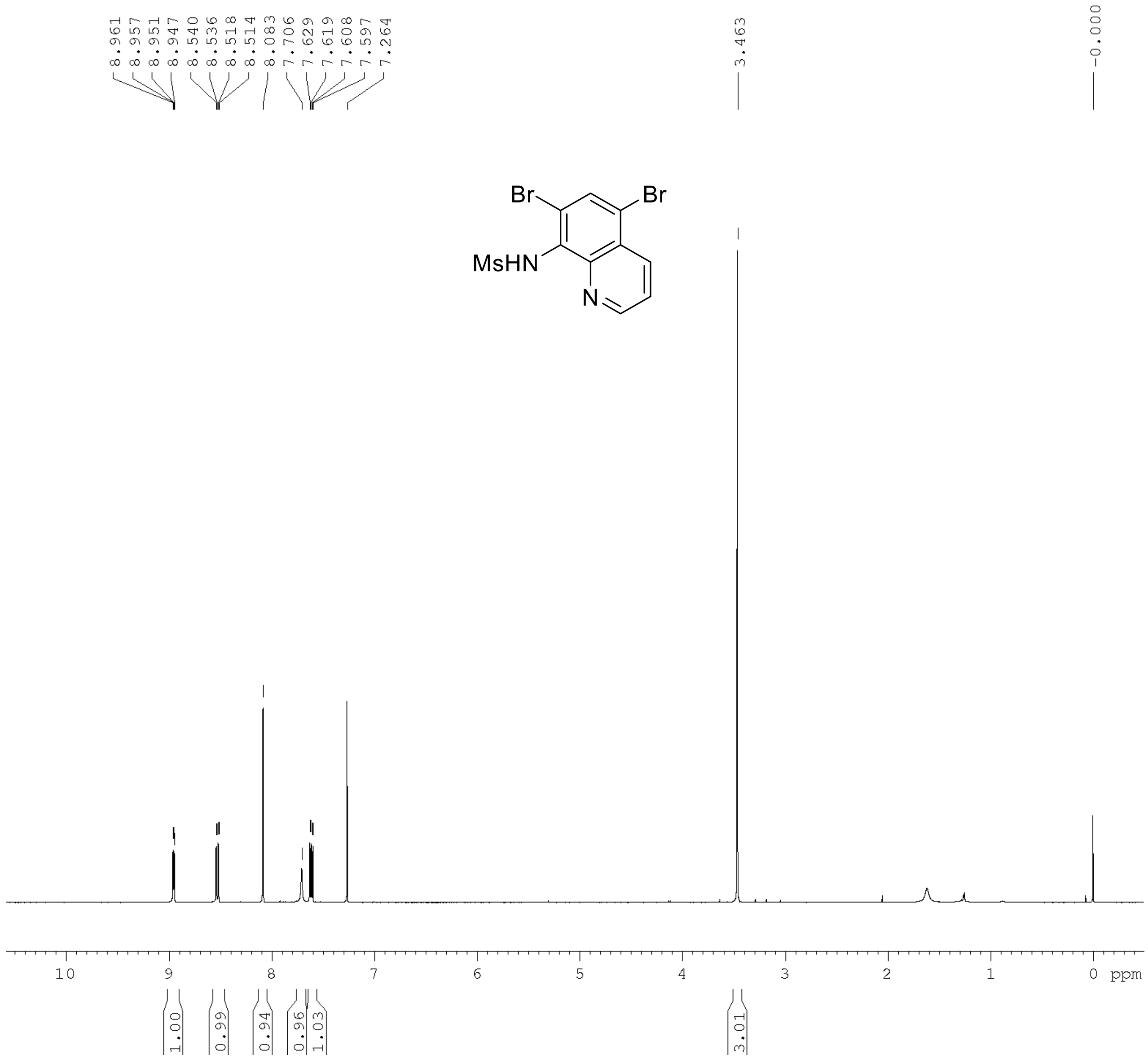

$^{13}C\{^1H\}$NMR spectrum of compound **9a** ($CDCl_3$, 100 MHz)**:**

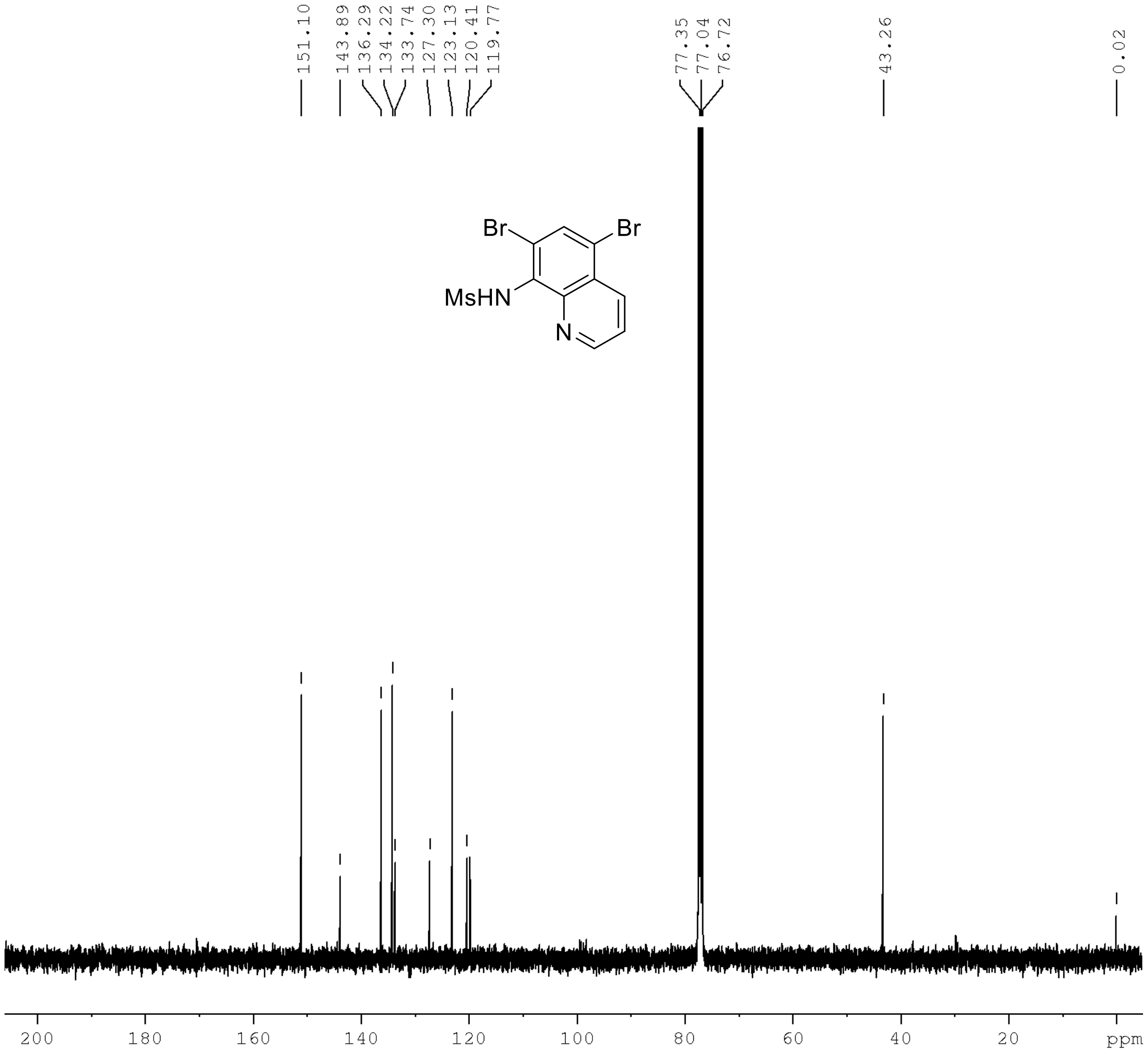

DEPT135 spectrum of compound **9a** ($CDCl_3$, 100 MHz)**:**

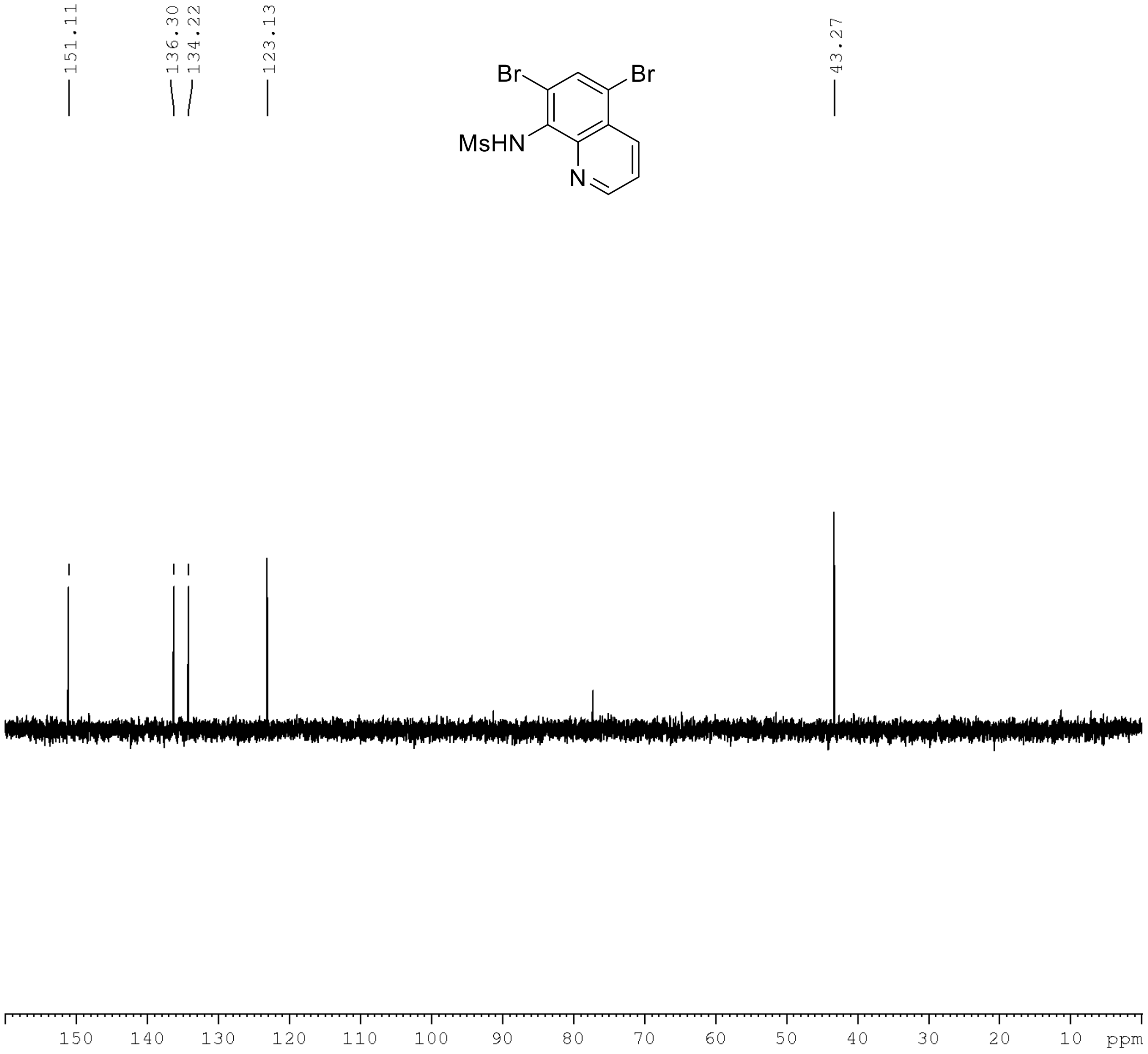

^{1}H NMR spectrum of compound **6b** ($CDCl_3$, 400 MHz):

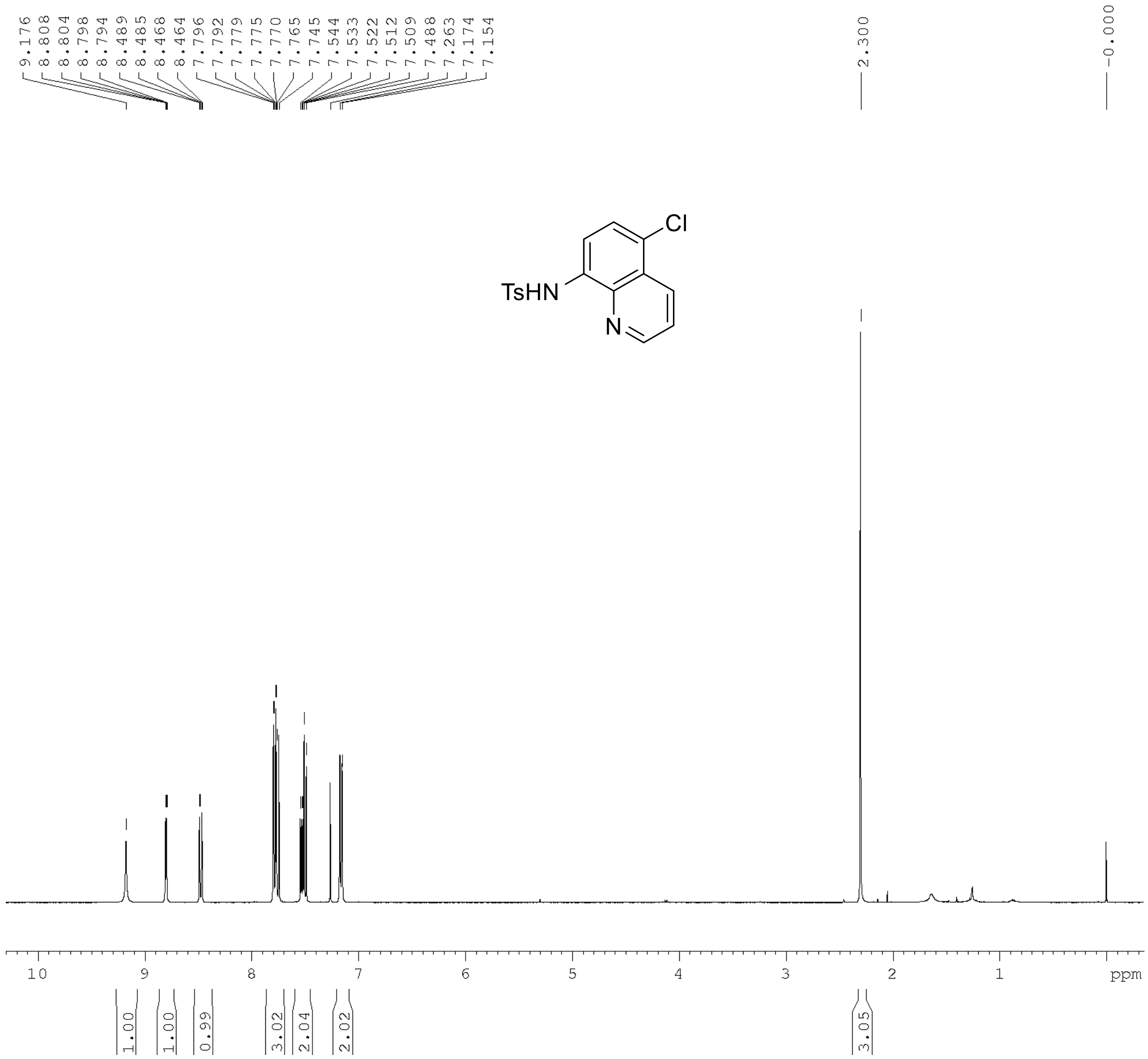

$^{13}C\{^1H\}$NMR spectrum of compound **6b** ($CDCl_3$, 100 MHz)**:**

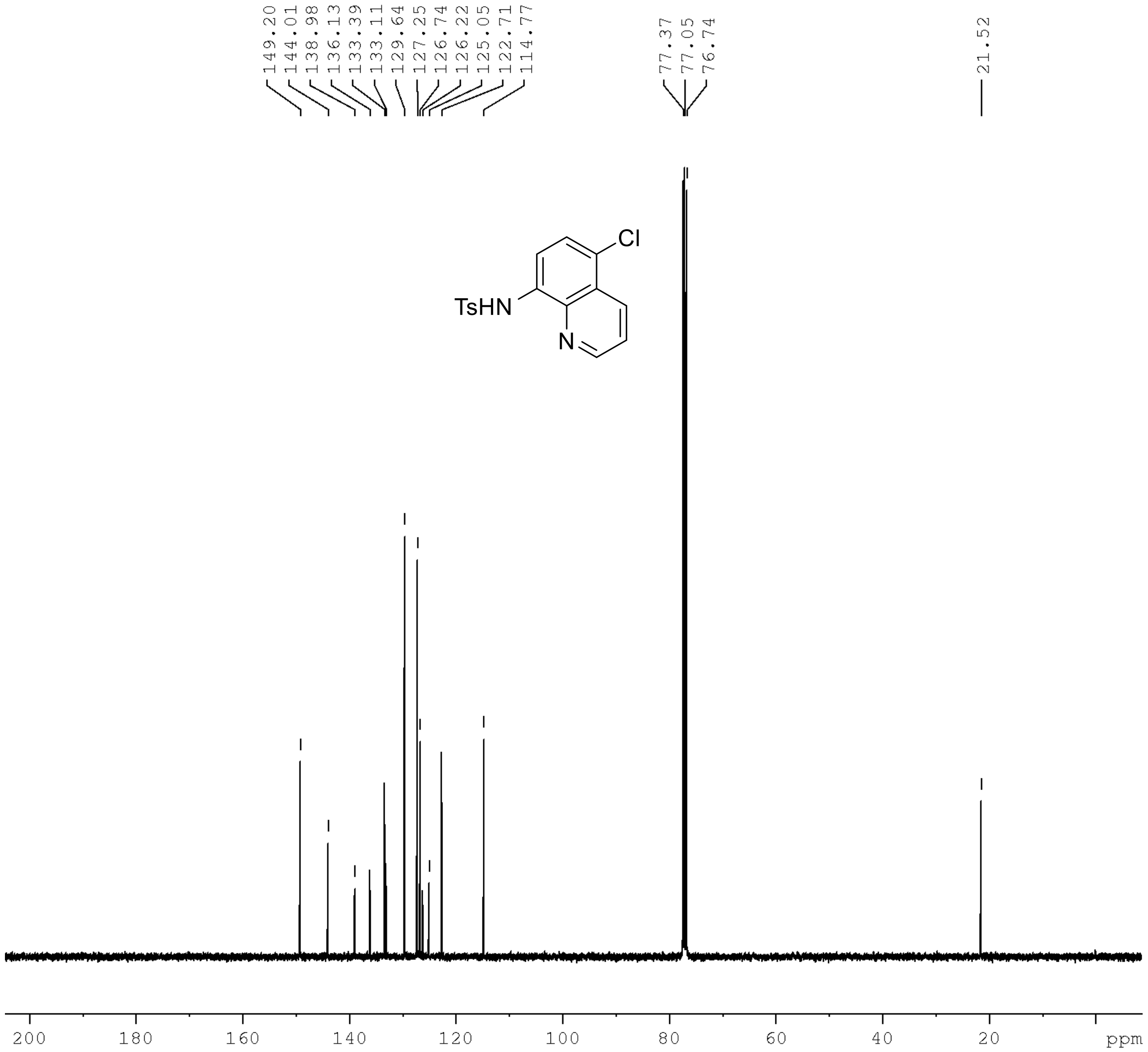

DEPT135 spectrum of compound **6b** ($CDCl_3$, 100 MHz)**:**

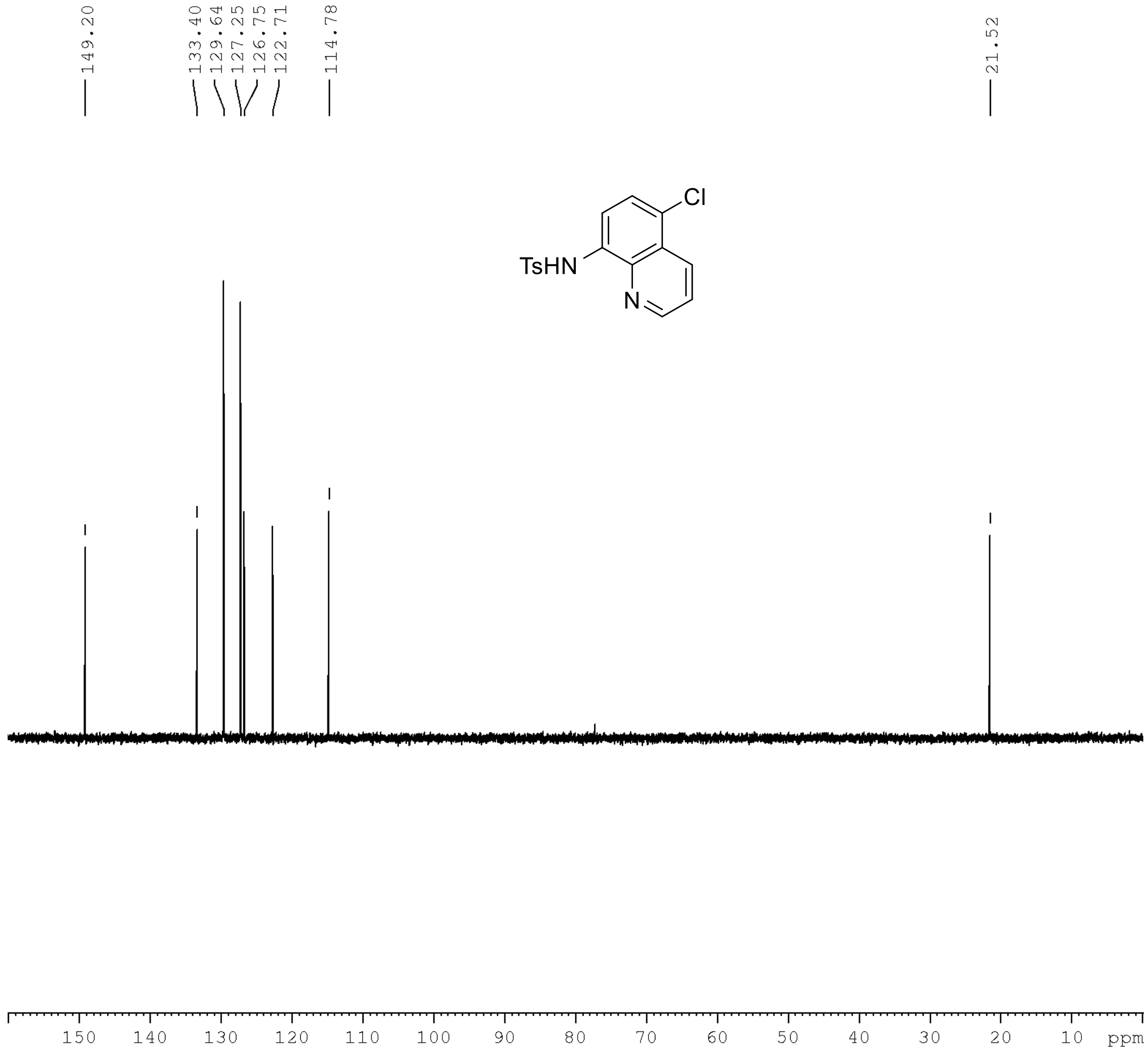

^{1}H NMR spectrum of compound **7b** (8:2, $CDCl_3$ + DMSO-*d6*, 400 MHz)**:**

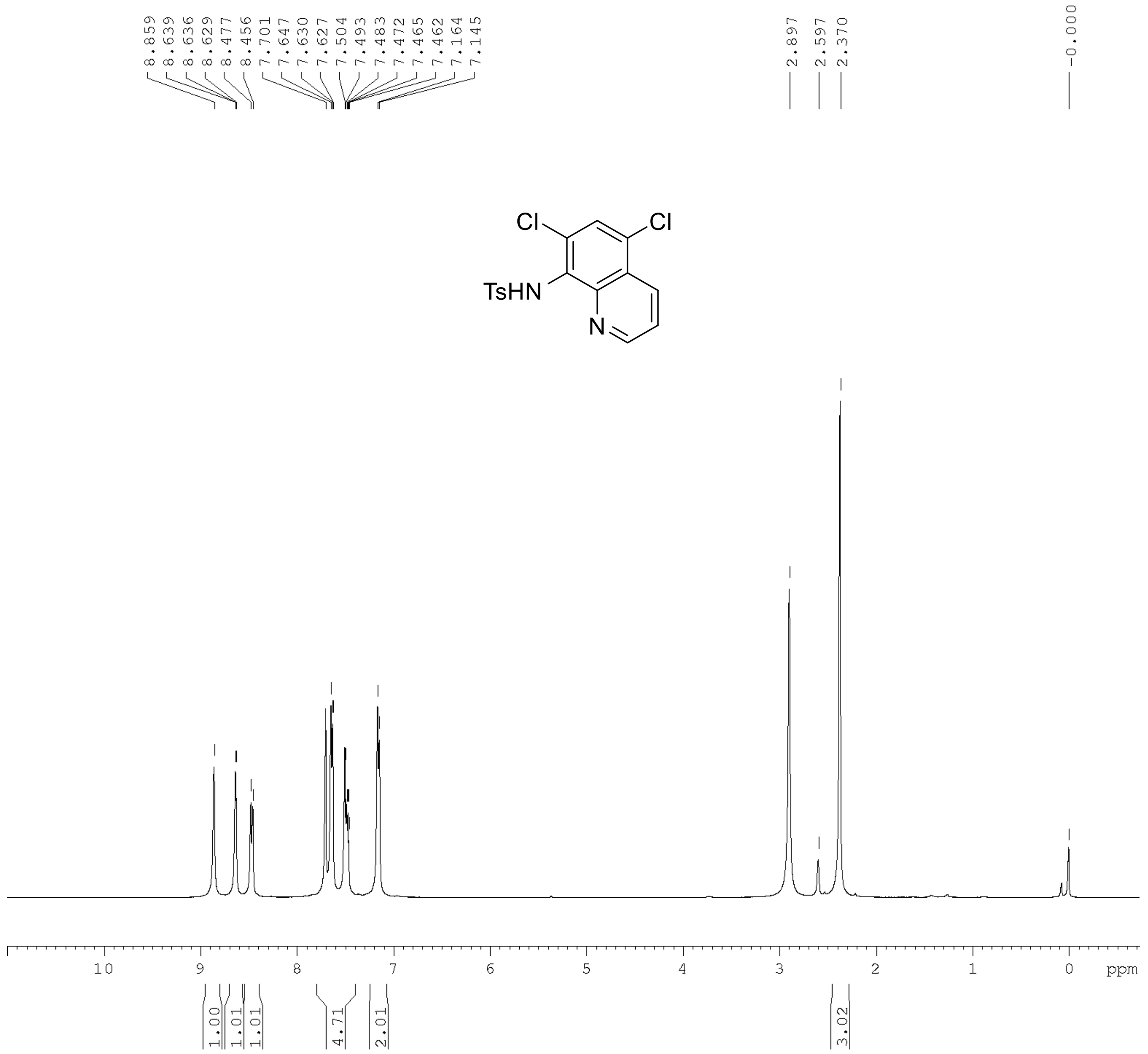

$^{13}C\{^1H\}$NMR spectrum of compound **7b** (2:8, DMSO-*d6* + $CDCl_3$, 100 MHz)**:**

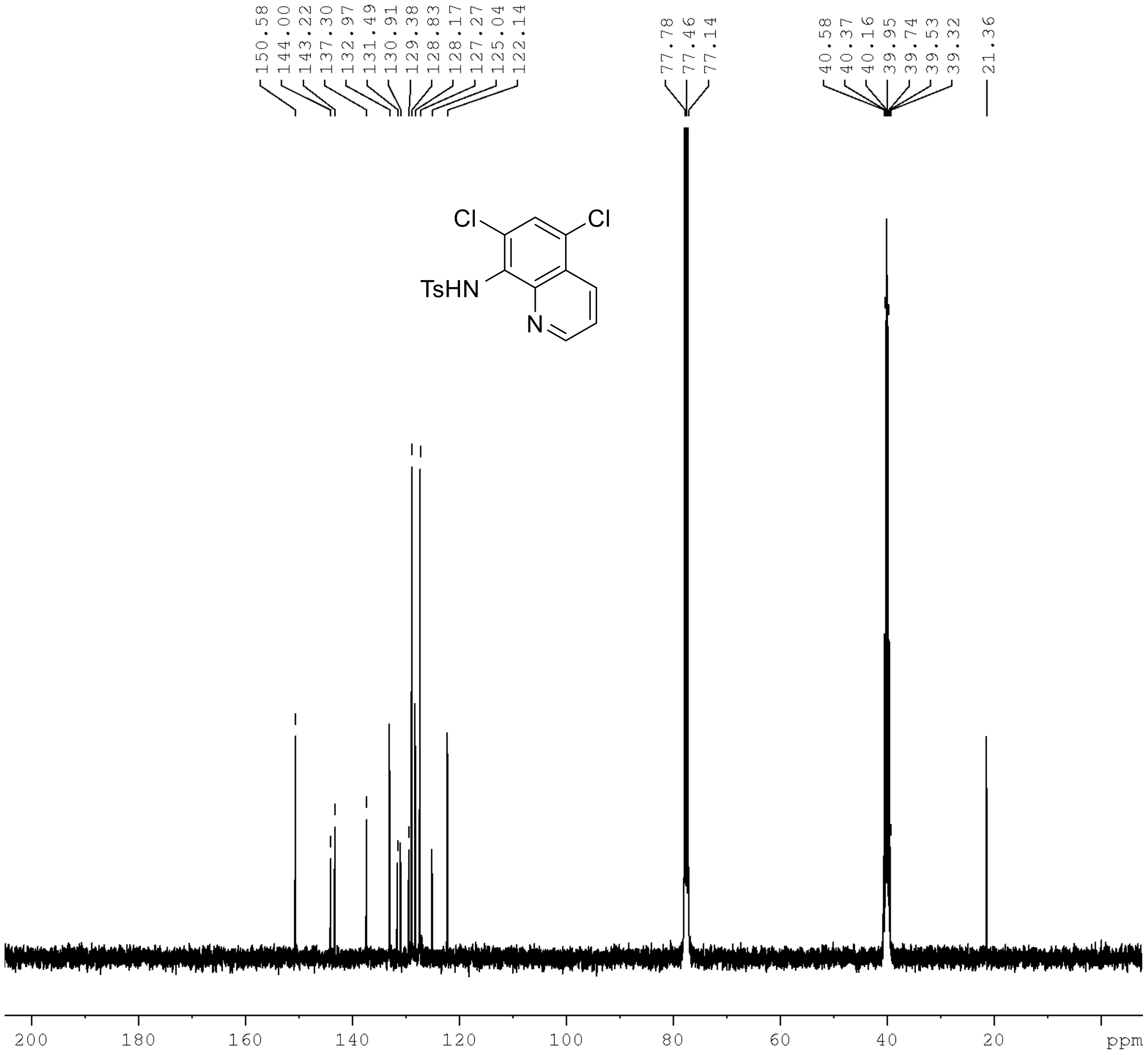

DEPT135 spectrum of compound **7b** (2:8, DMSO-*d6* + $CDCl_3$, 100 MHz)**:**

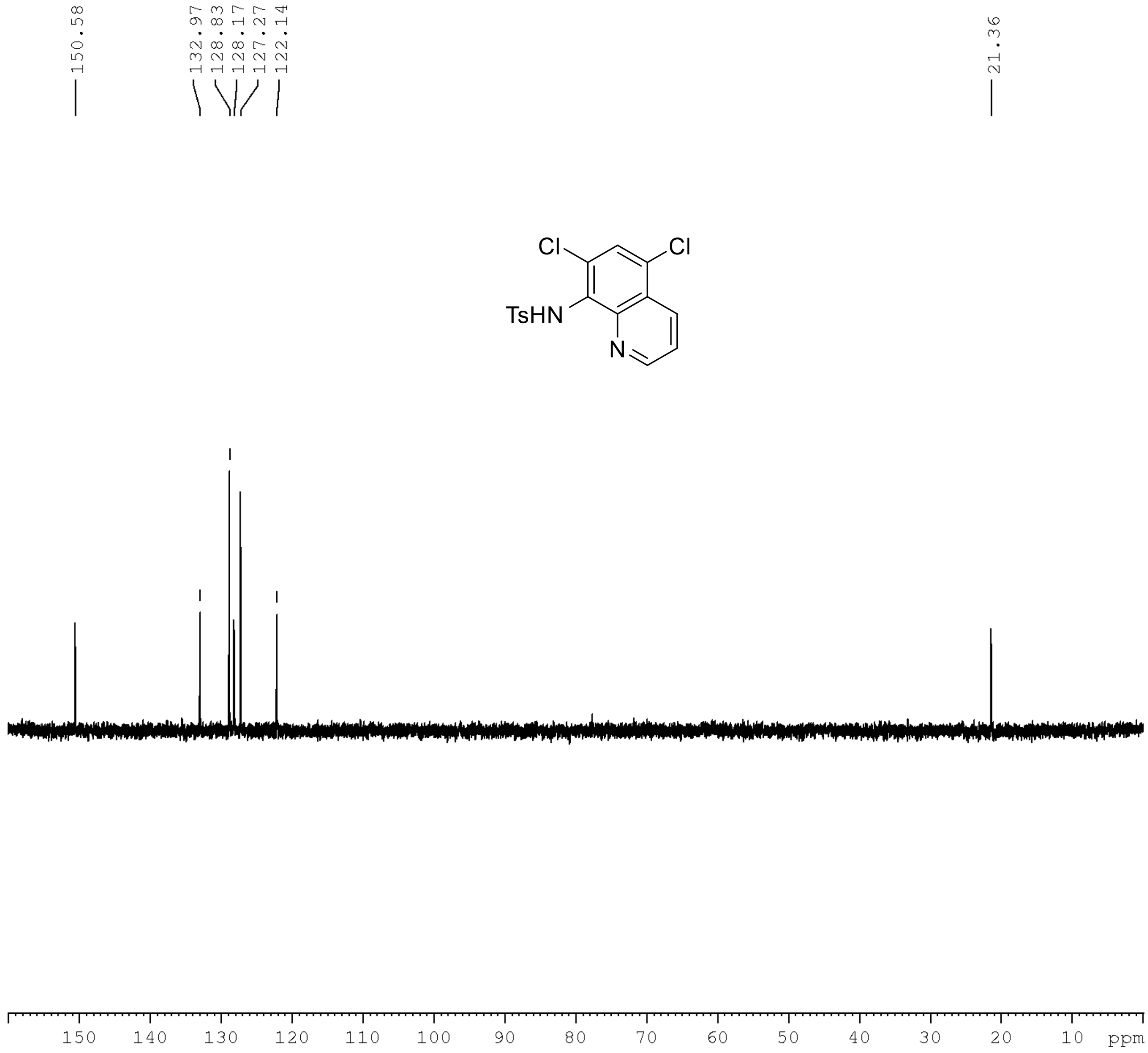

HRMS spectrum of compound **7b:**

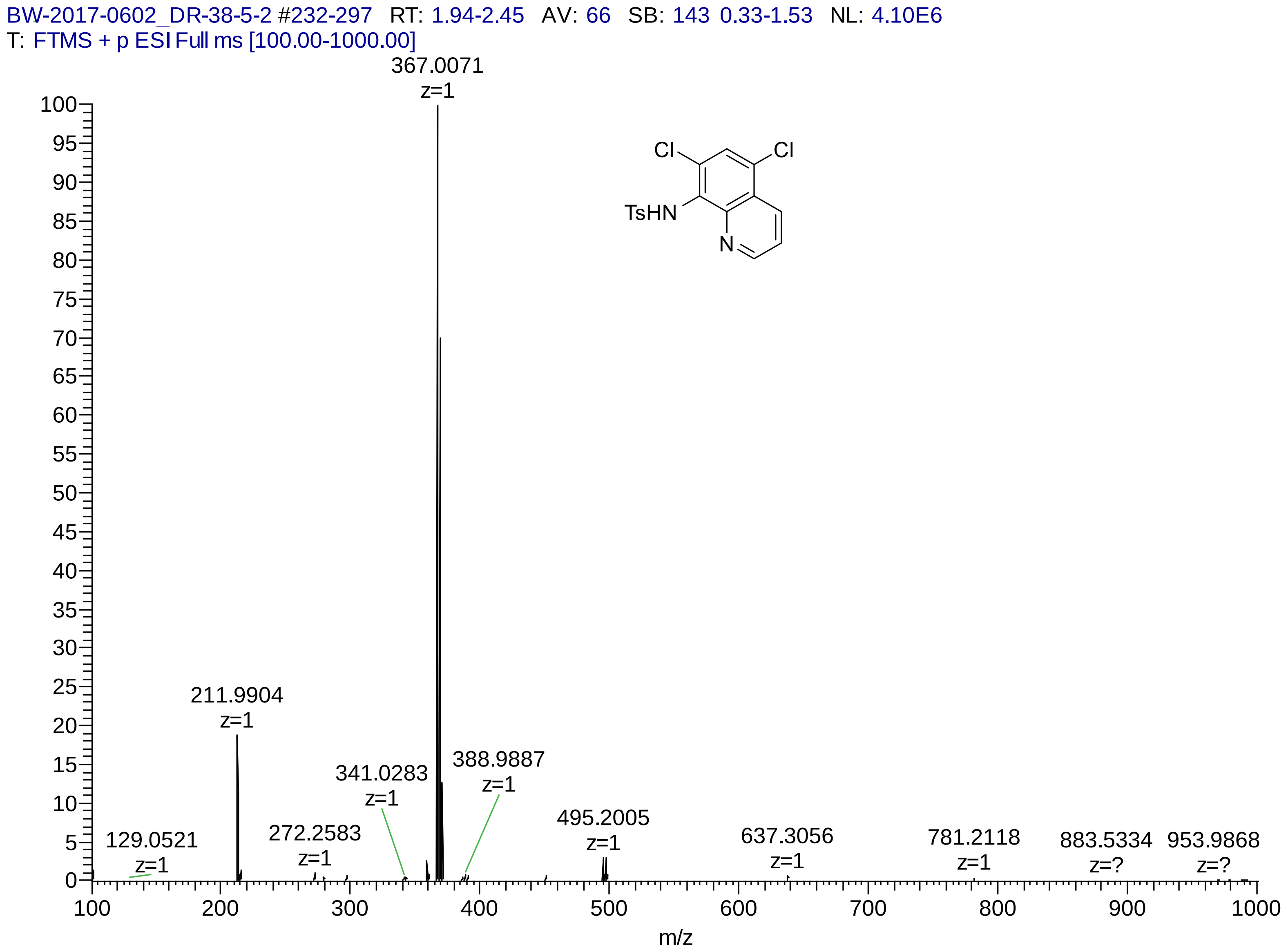

^{1}H NMR spectrum of compound **8b** ($CDCl_3$, 400 MHz)**:**

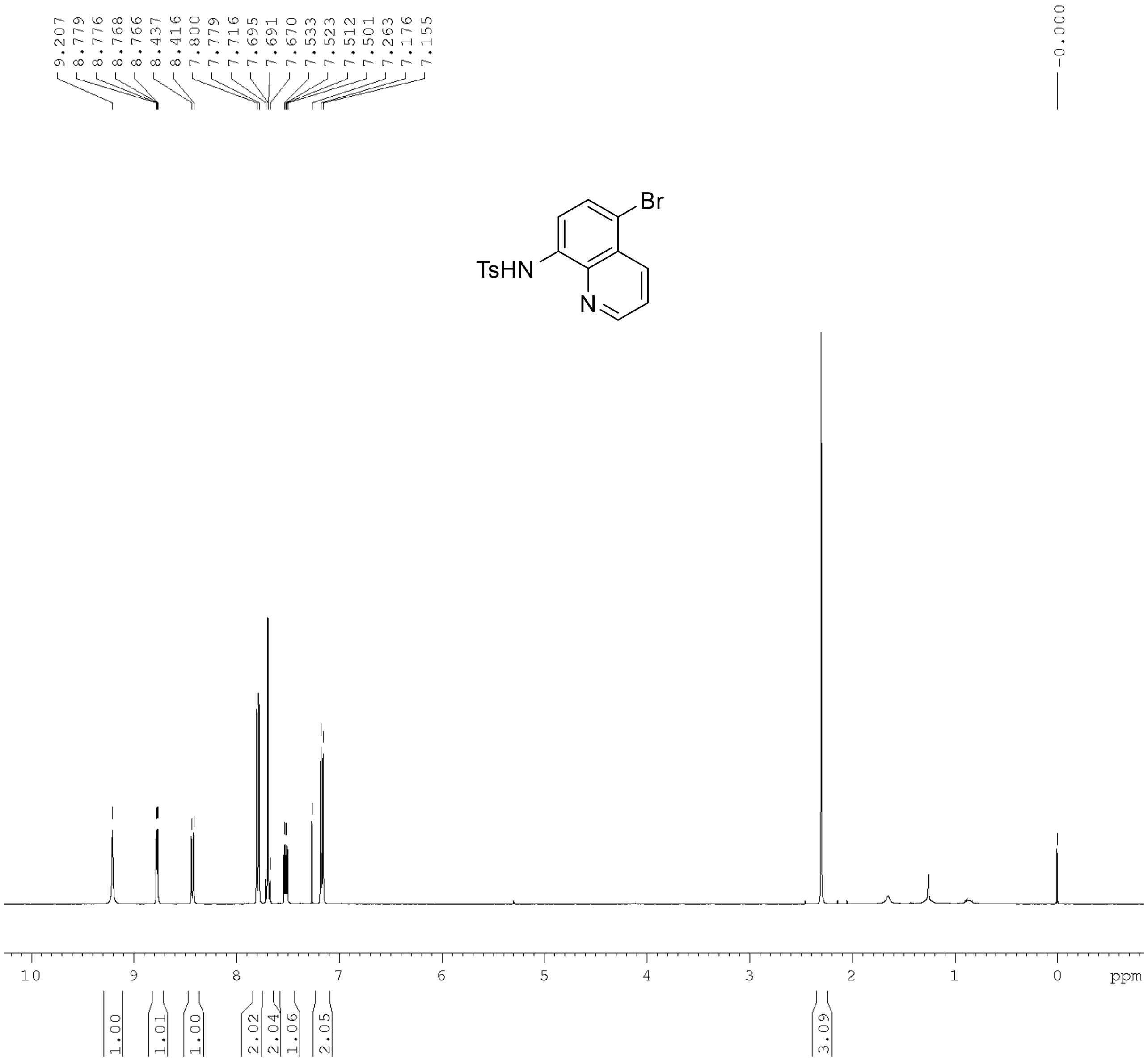

$^{13}C\{^1H\}$NMR spectrum of compound **8b** ($CDCl_3$, 100 MHz)**:**

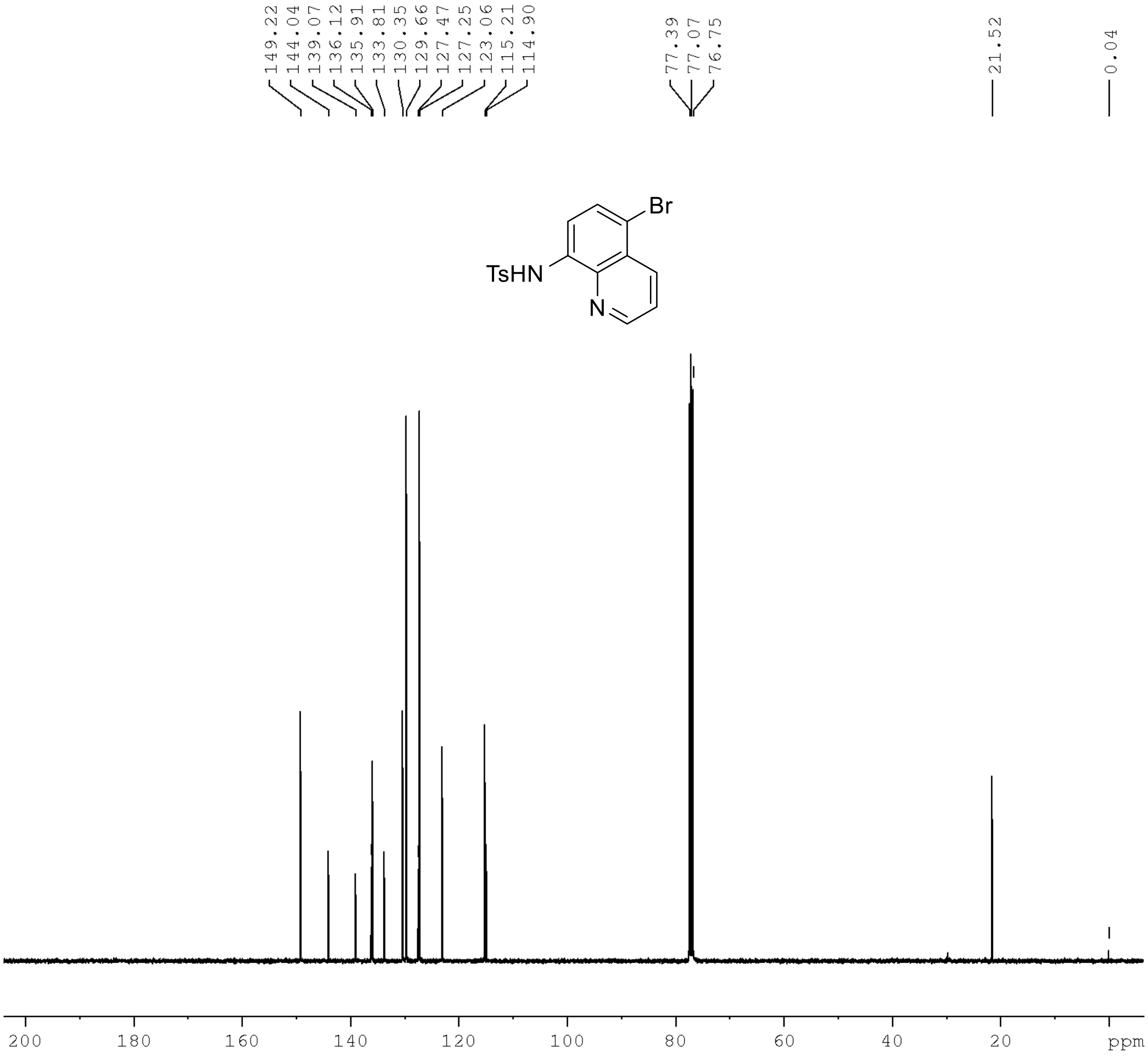

DEPT135 spectrum of compound **8b** ($CDCl_3$, 100 MHz)**:**

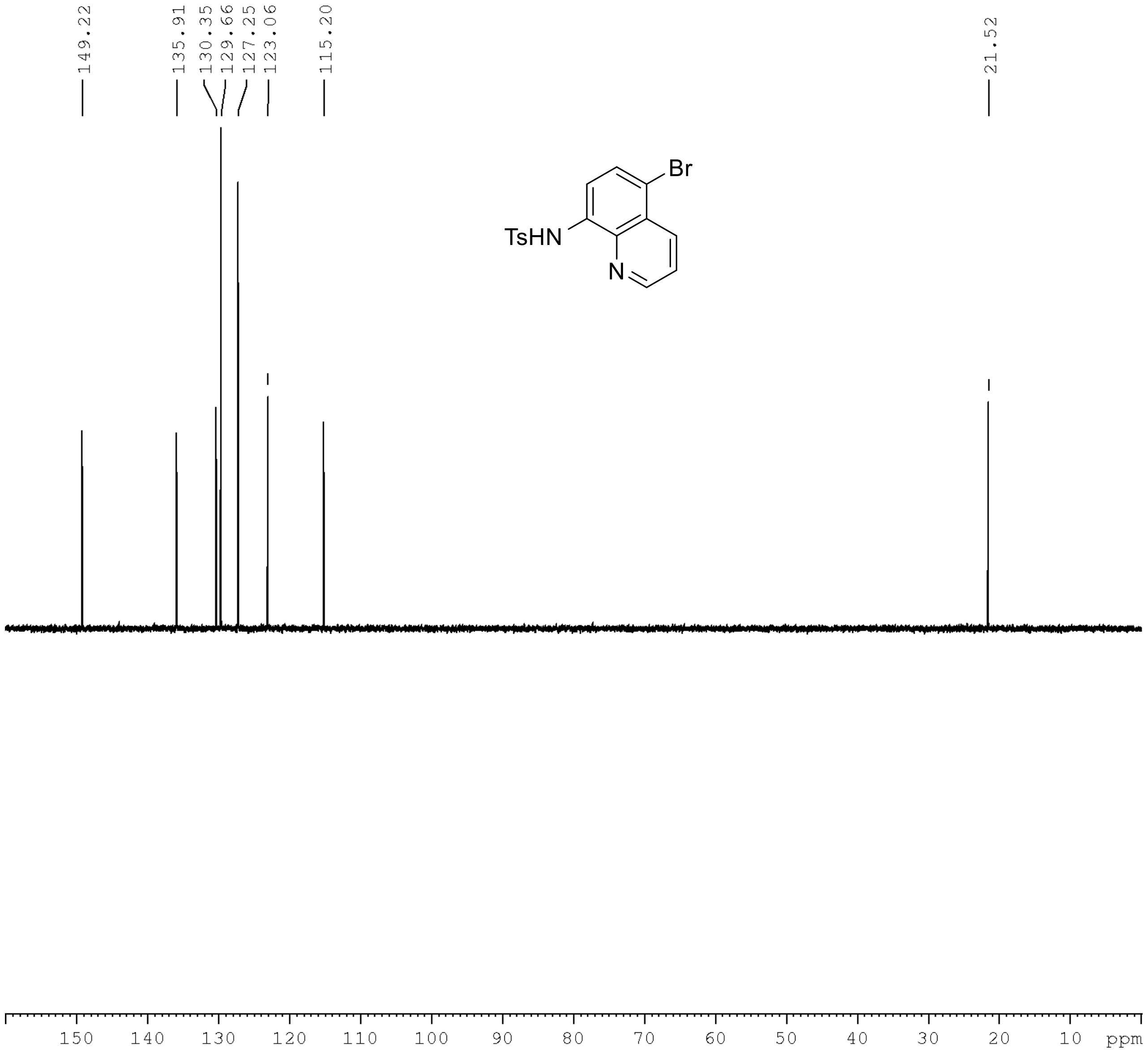

^{1}H NMR spectrum of compound **9b** (8:2, DMSO-*d6* + $CDCl_3$, 400 MHz)**:**

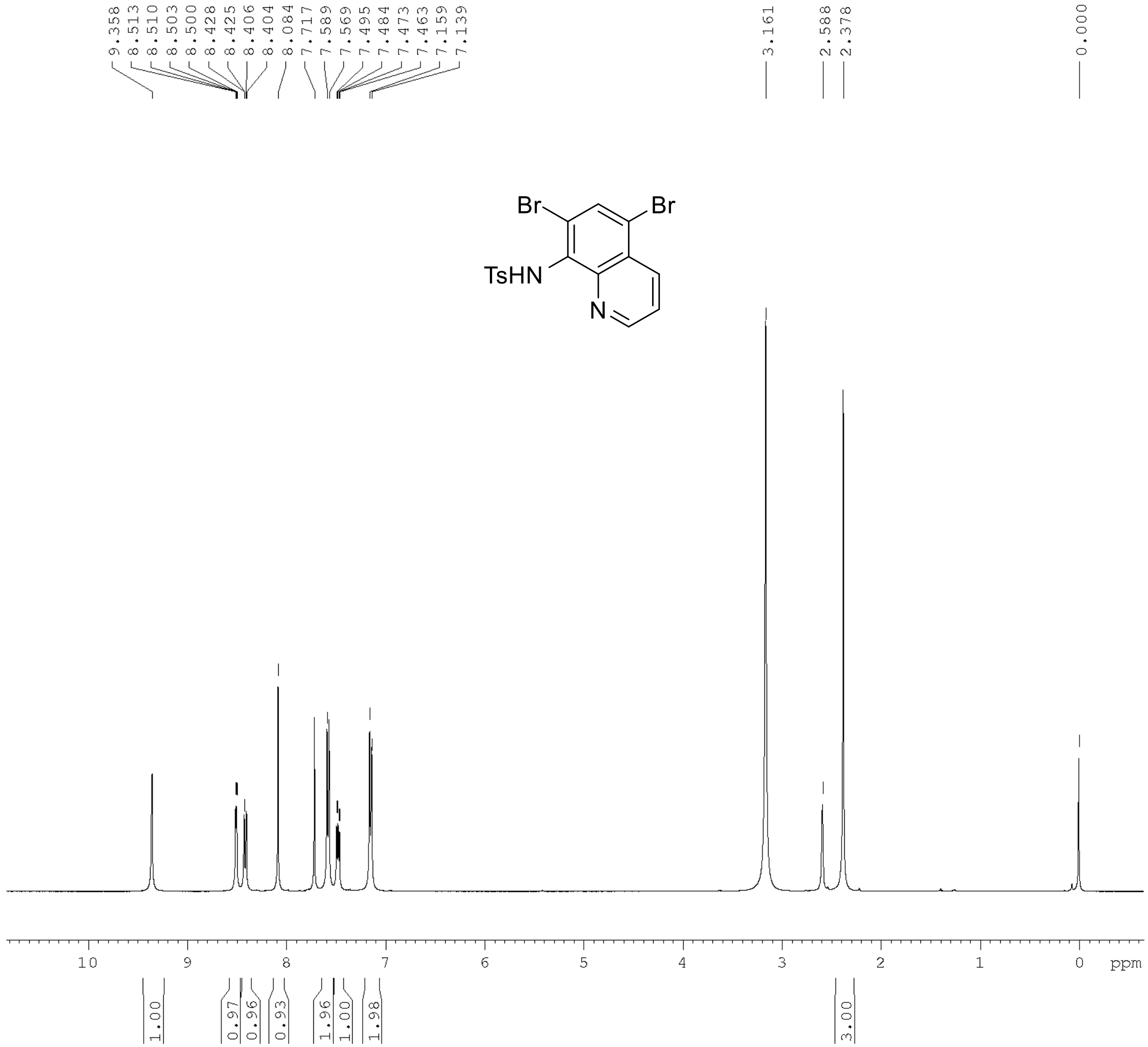

$^{13}C\{^{1}H\}$NMR spectrum of compound **9b** (2:8, DMSO-*d6* + $CDCl_3$, 100 MHz)**:**

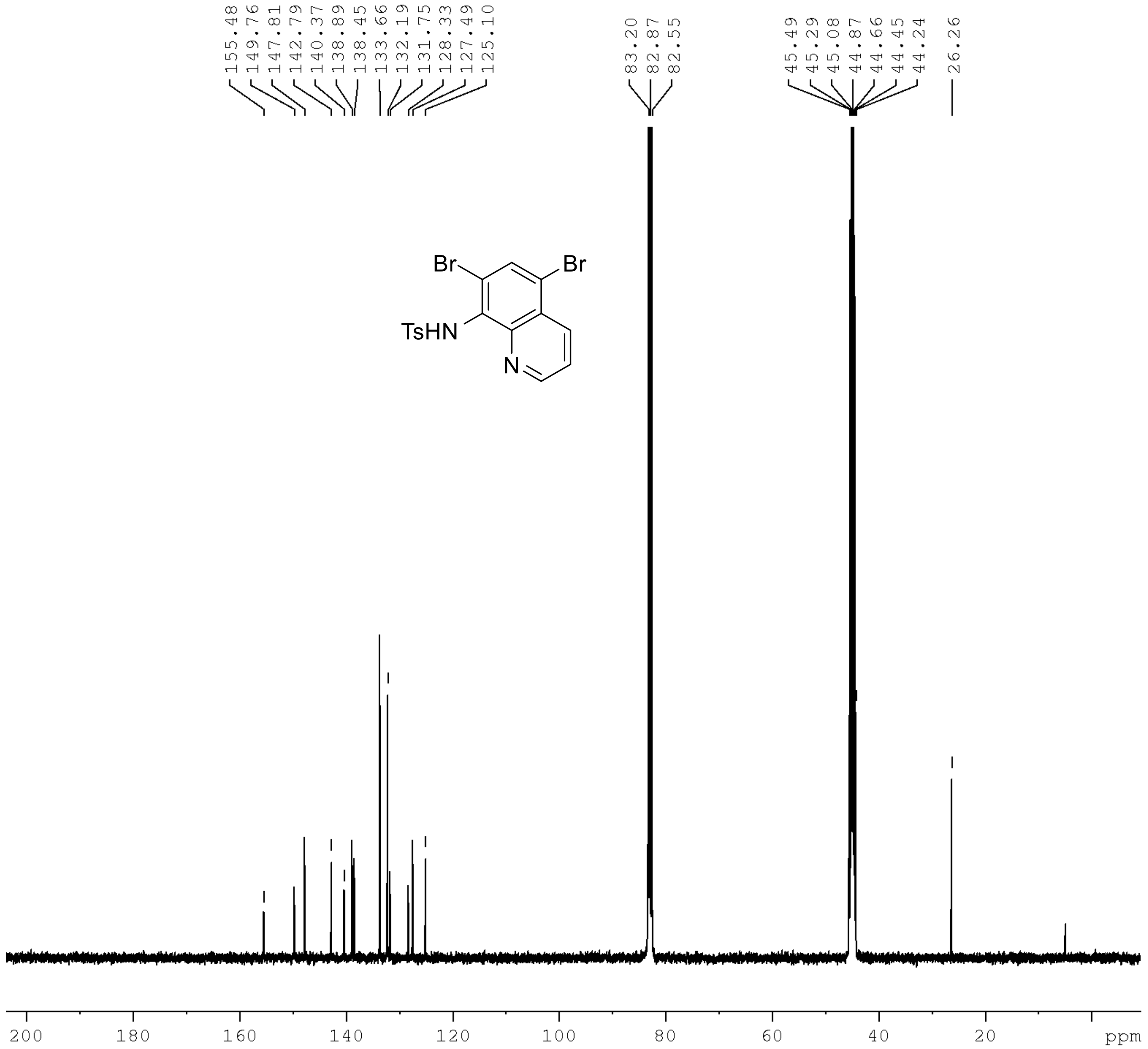

DEPT135 spectrum of compound **9b** (8:2, DMSO-*d6* + $CDCl_3$, 100 MHz)**:**

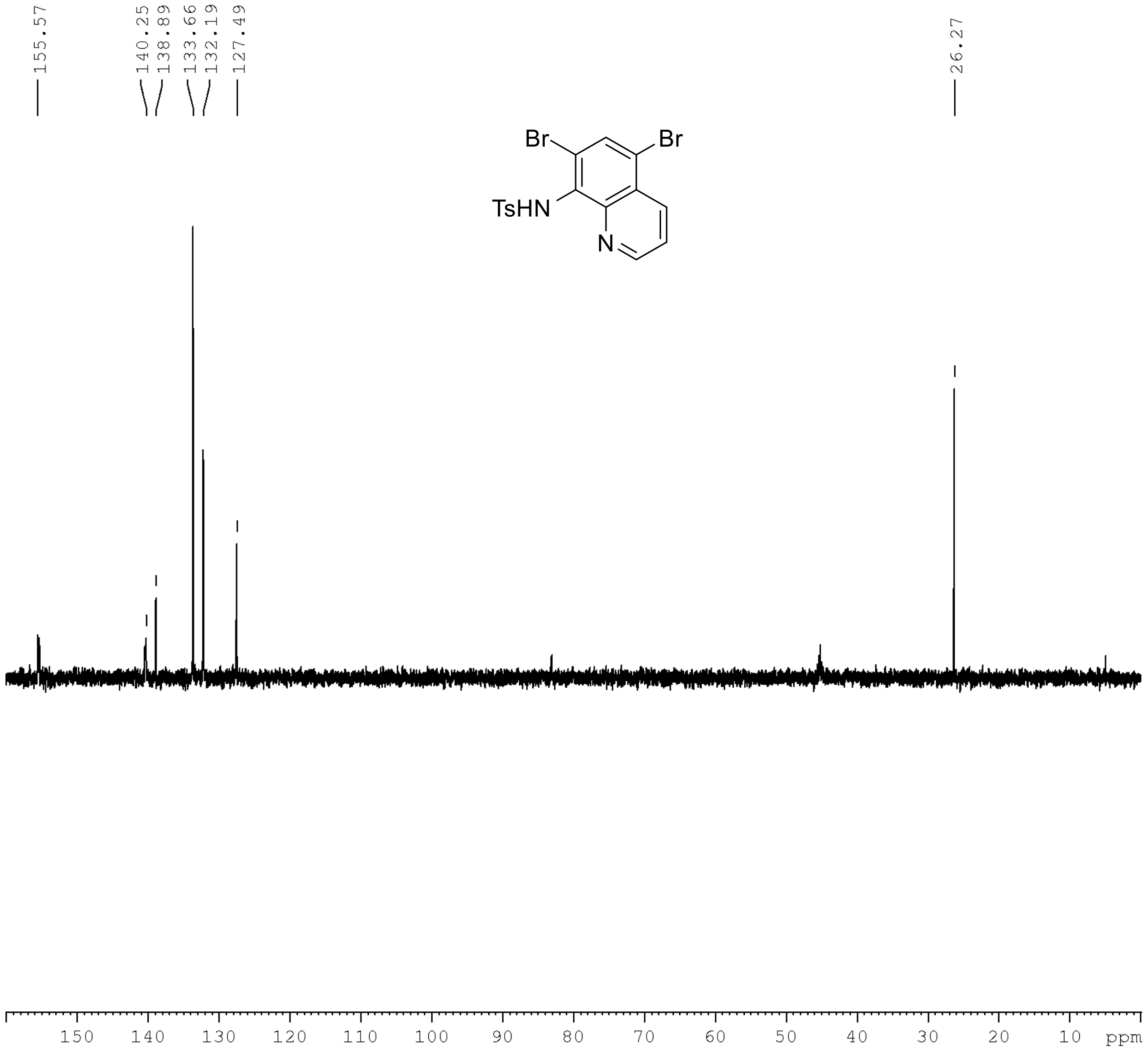

^{1}H NMR spectrum of compound **6c** ($CDCl_3$, 400 MHz)**:**

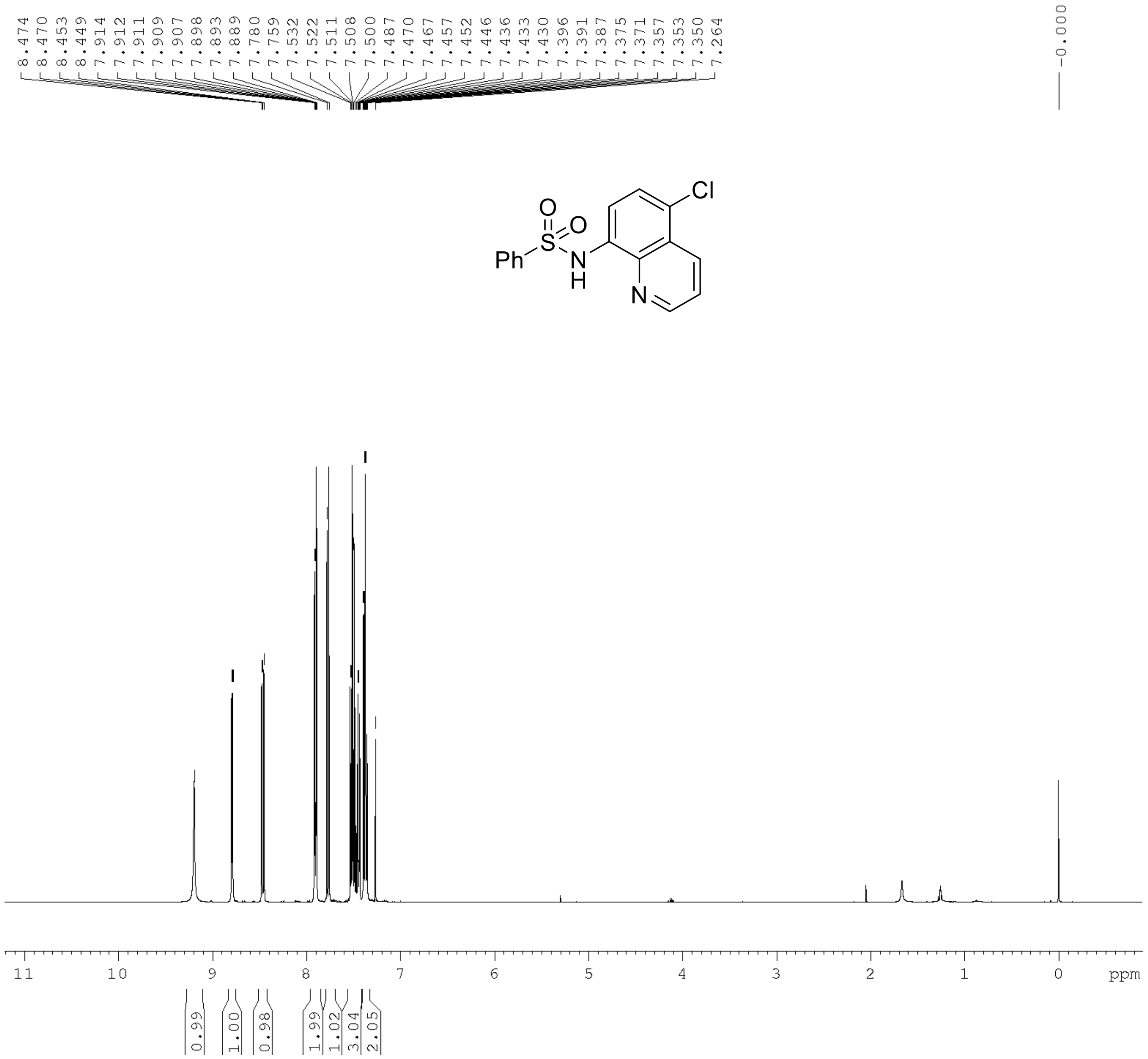

$^{13}C\{^{1}H\}$NMR spectrum of compound **6c** ($CDCl_3$, 100 MHz)**:**

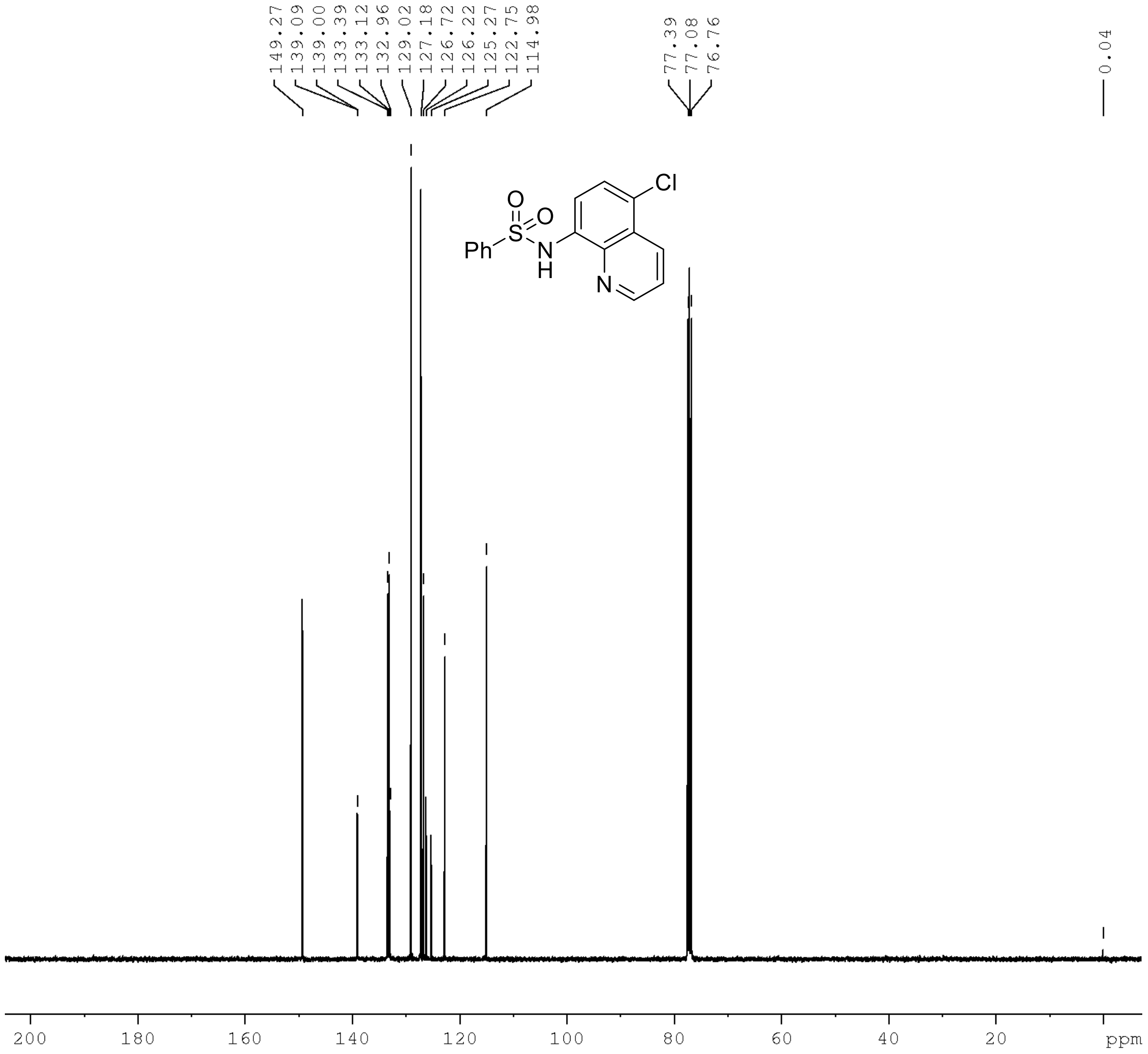

DEPT135 spectrum of compound **6c** ($CDCl_3$, 100 MHz)**:**

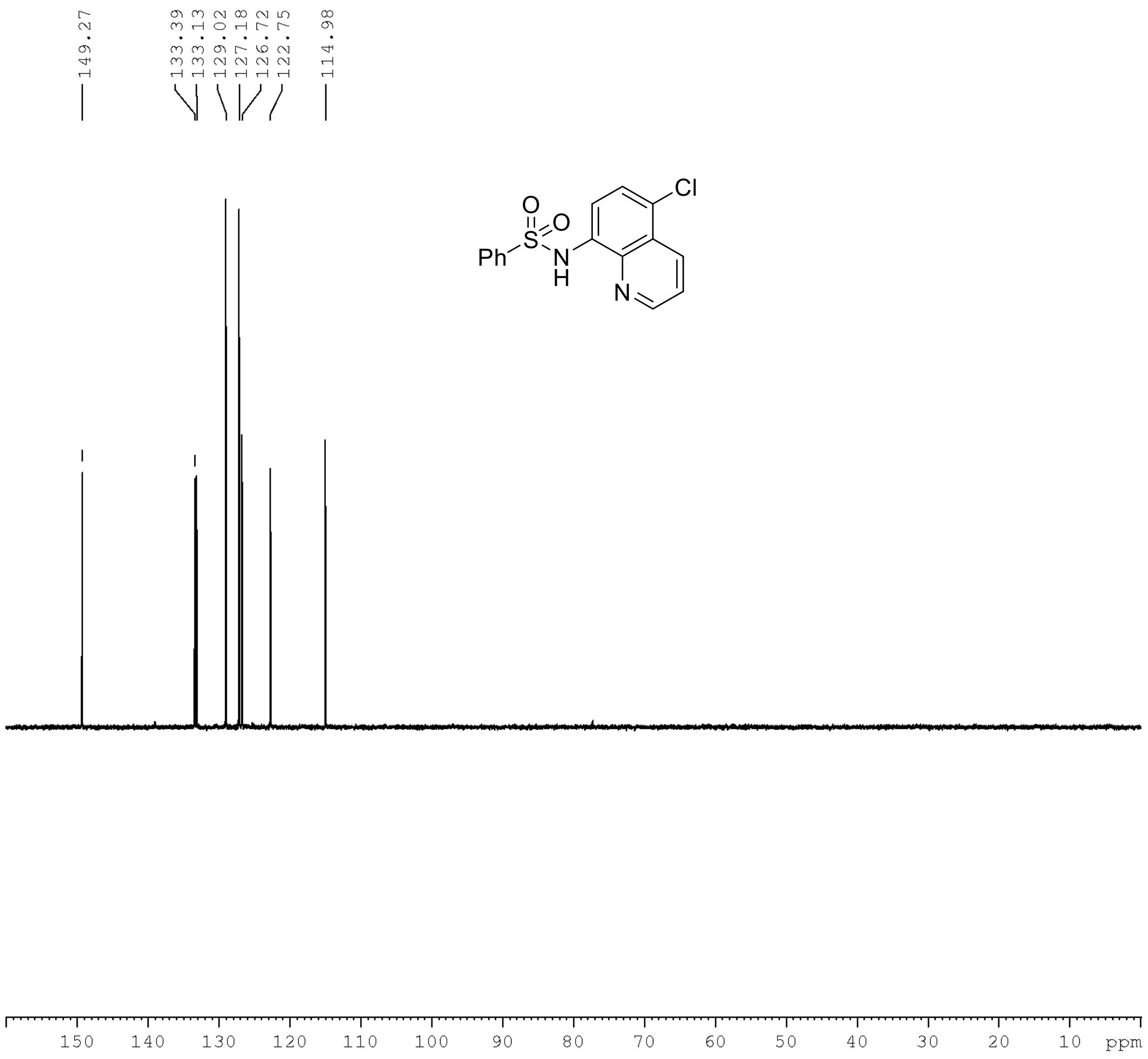

^{1}H NMR spectrum of compound **7c** ($CDCl_3$, 400 MHz)**:**

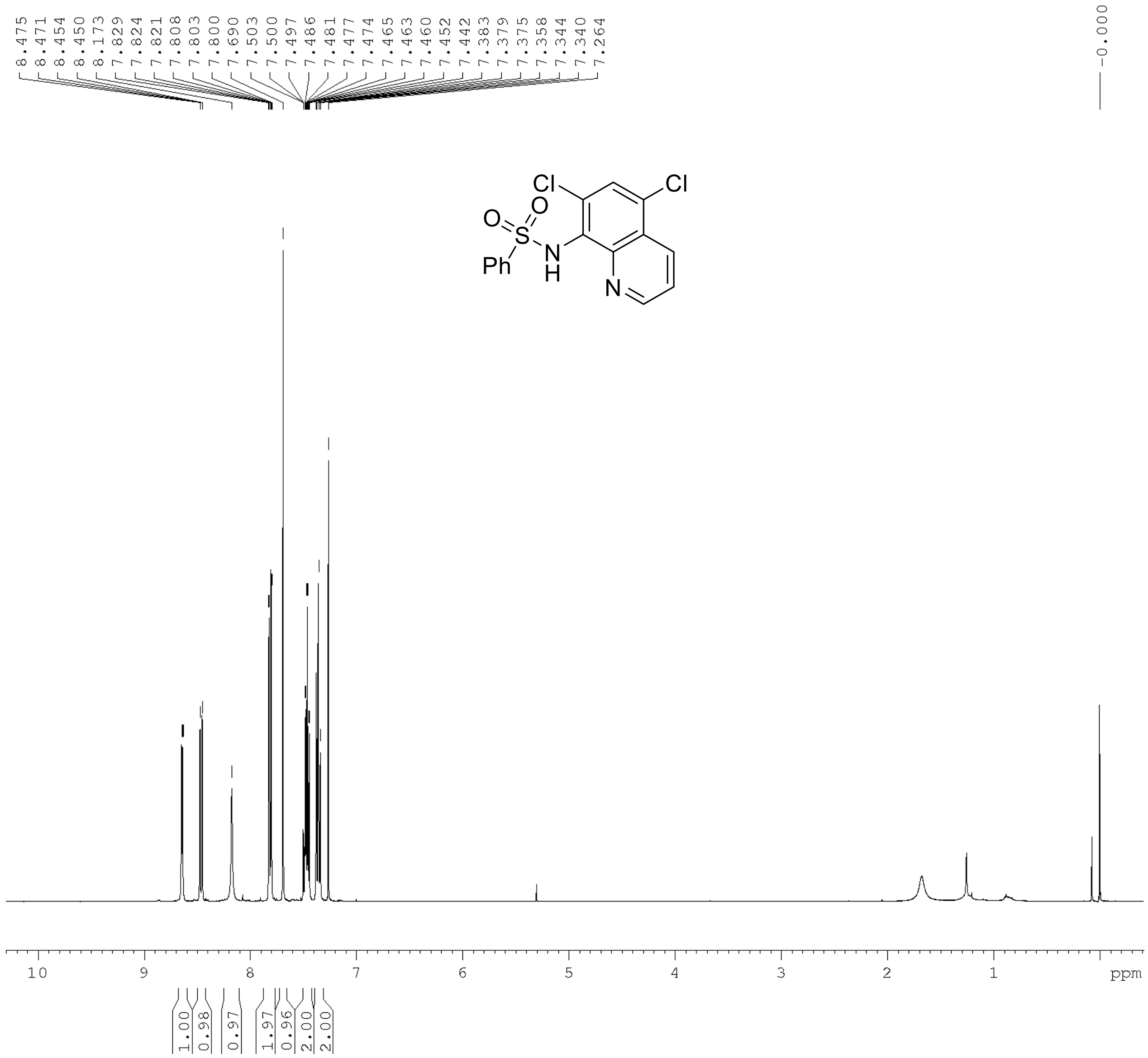

$^{13}C\{^1H\}$NMR spectrum of compound **7c** ($CDCl_3$, 100 MHz)**:**

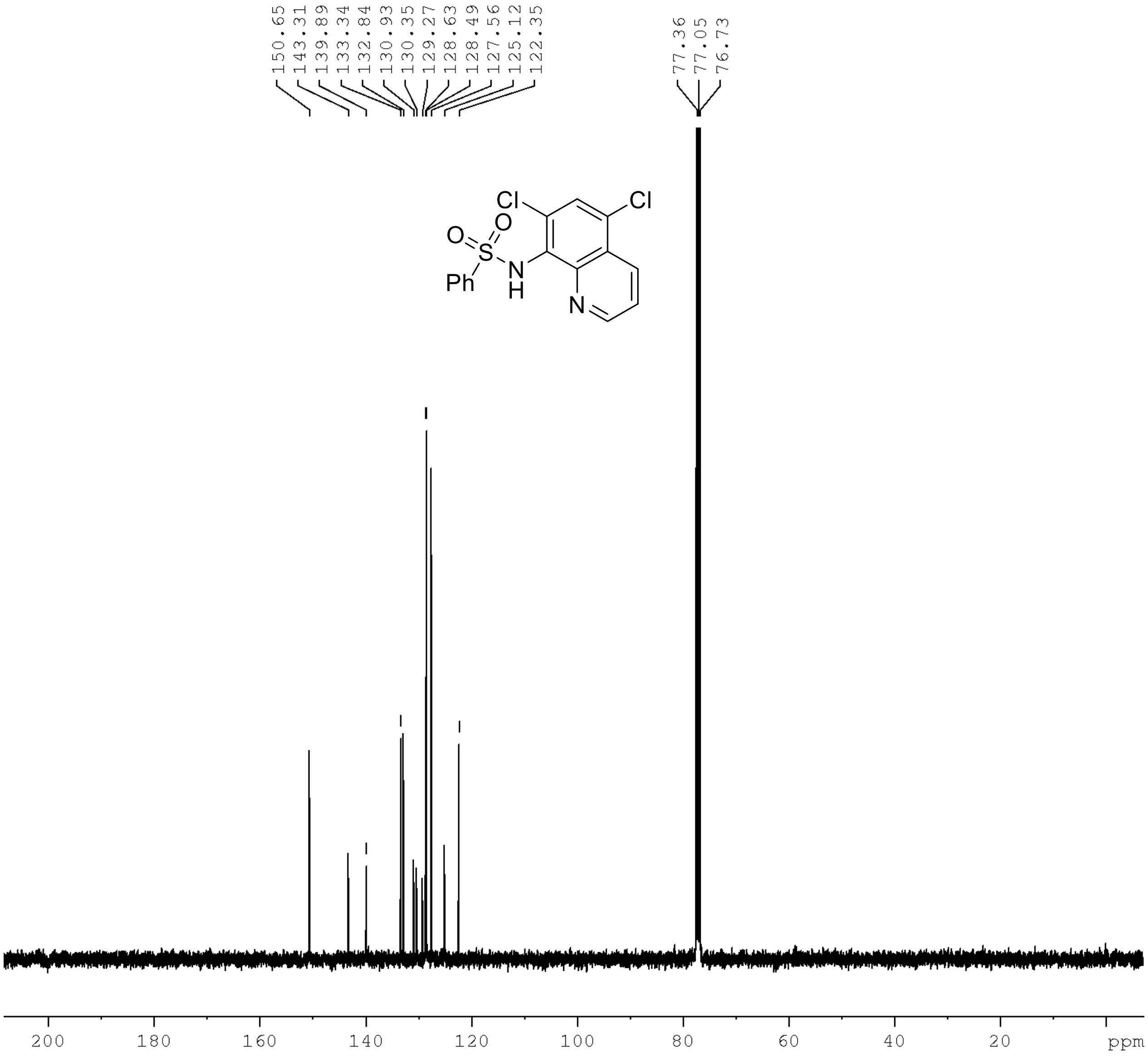

DEPT135 spectrum of compound **7c** ($CDCl_3$, 100 MHz):

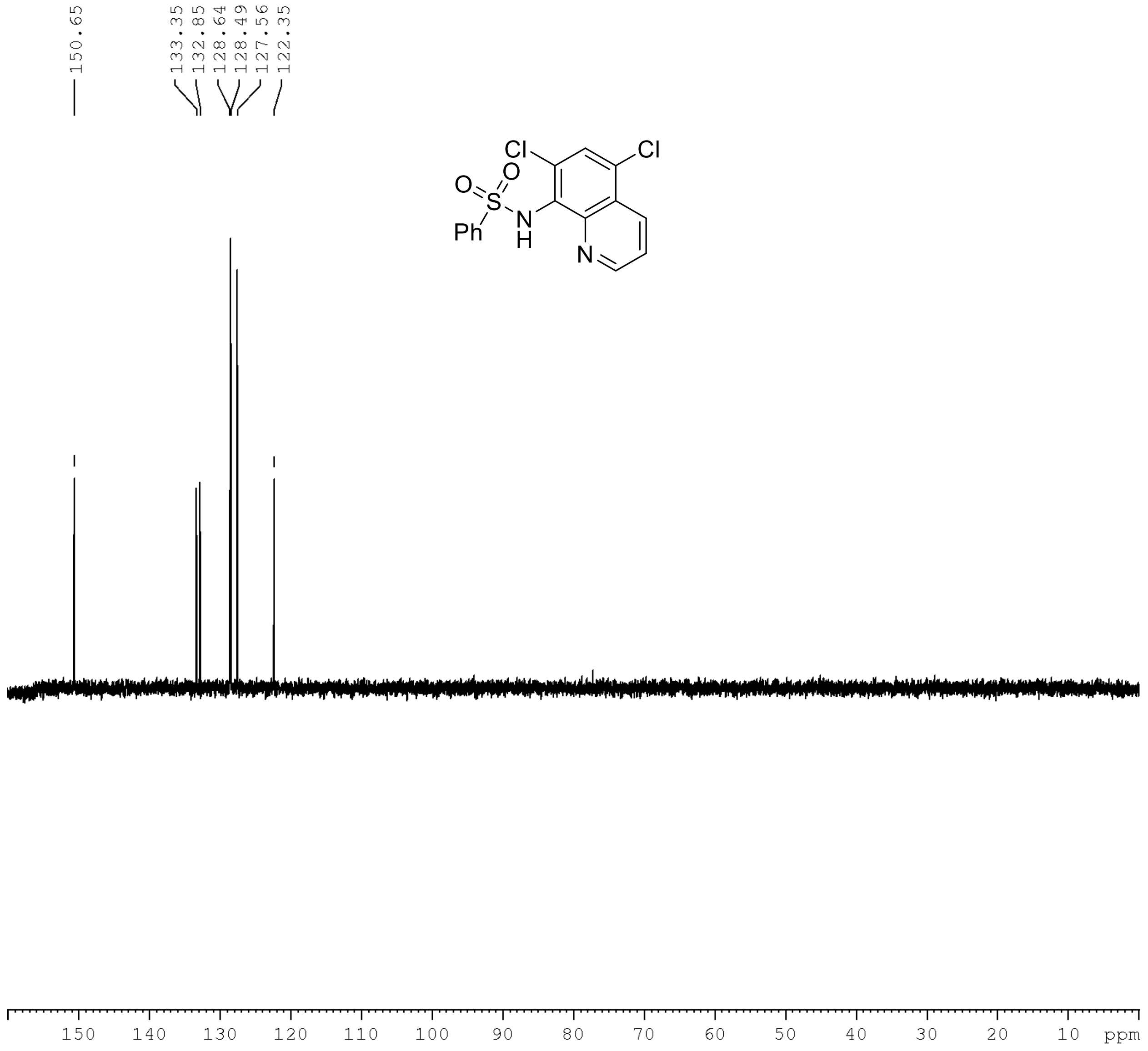

^{1}H NMR spectrum of compound **8c** ($CDCl_3$, 400 MHz)**:**

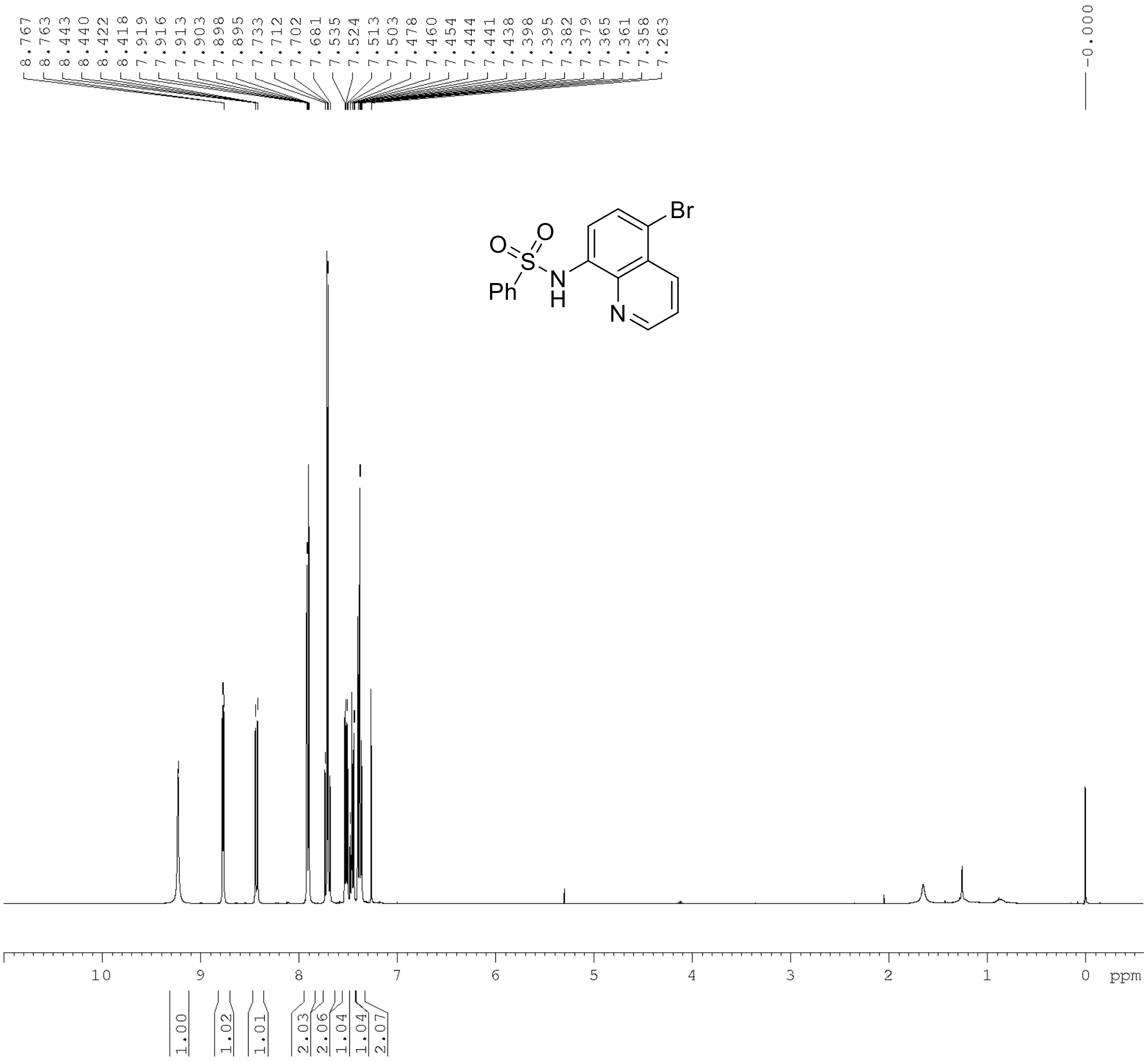

$^{13}C\{^{1}H\}$NMR spectrum of compound **8c** ($CDCl_3$, 100 MHz)**:**

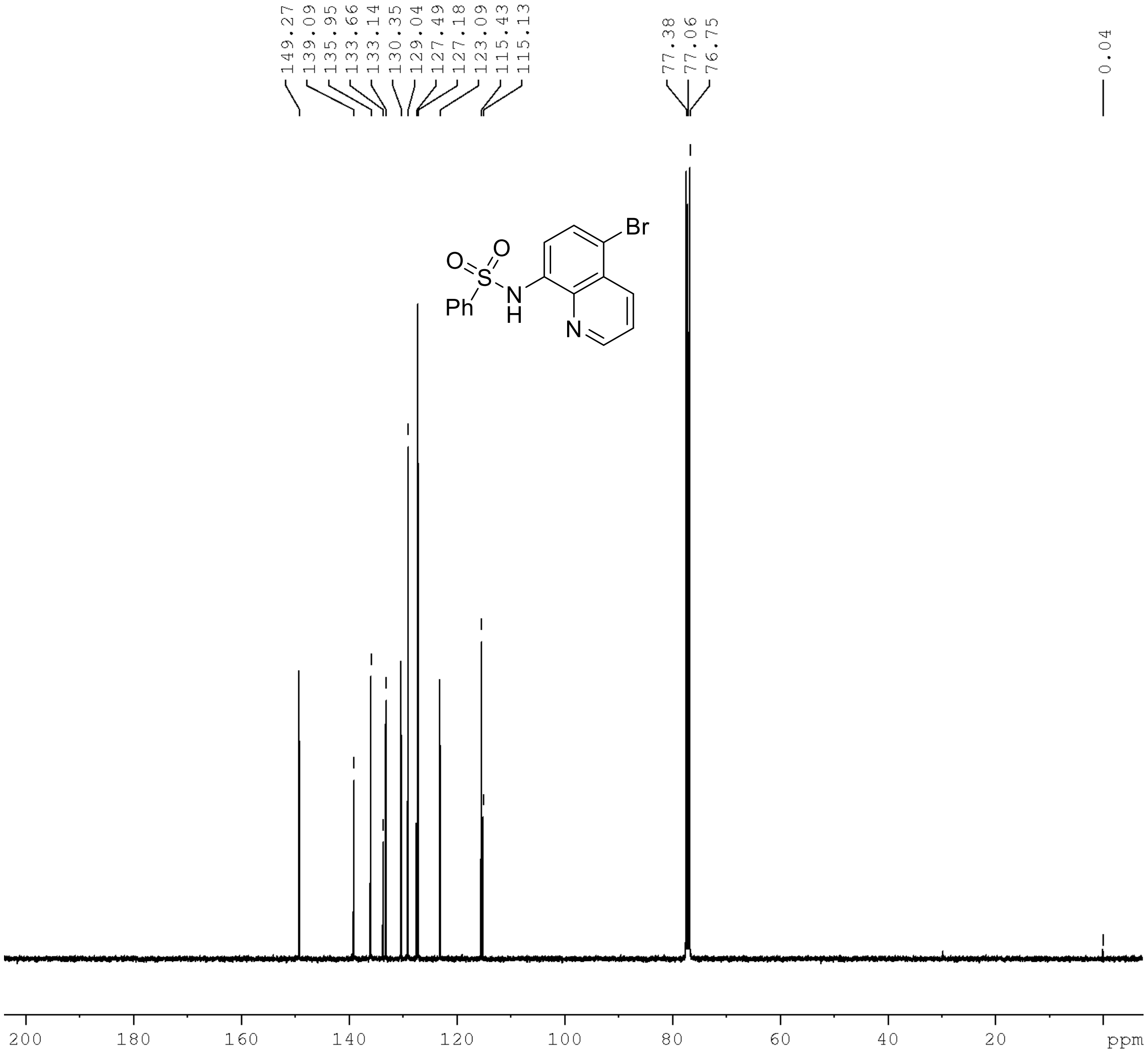

DEPT135 spectrum of compound **8c** ($CDCl_3$, 100 MHz)**:**

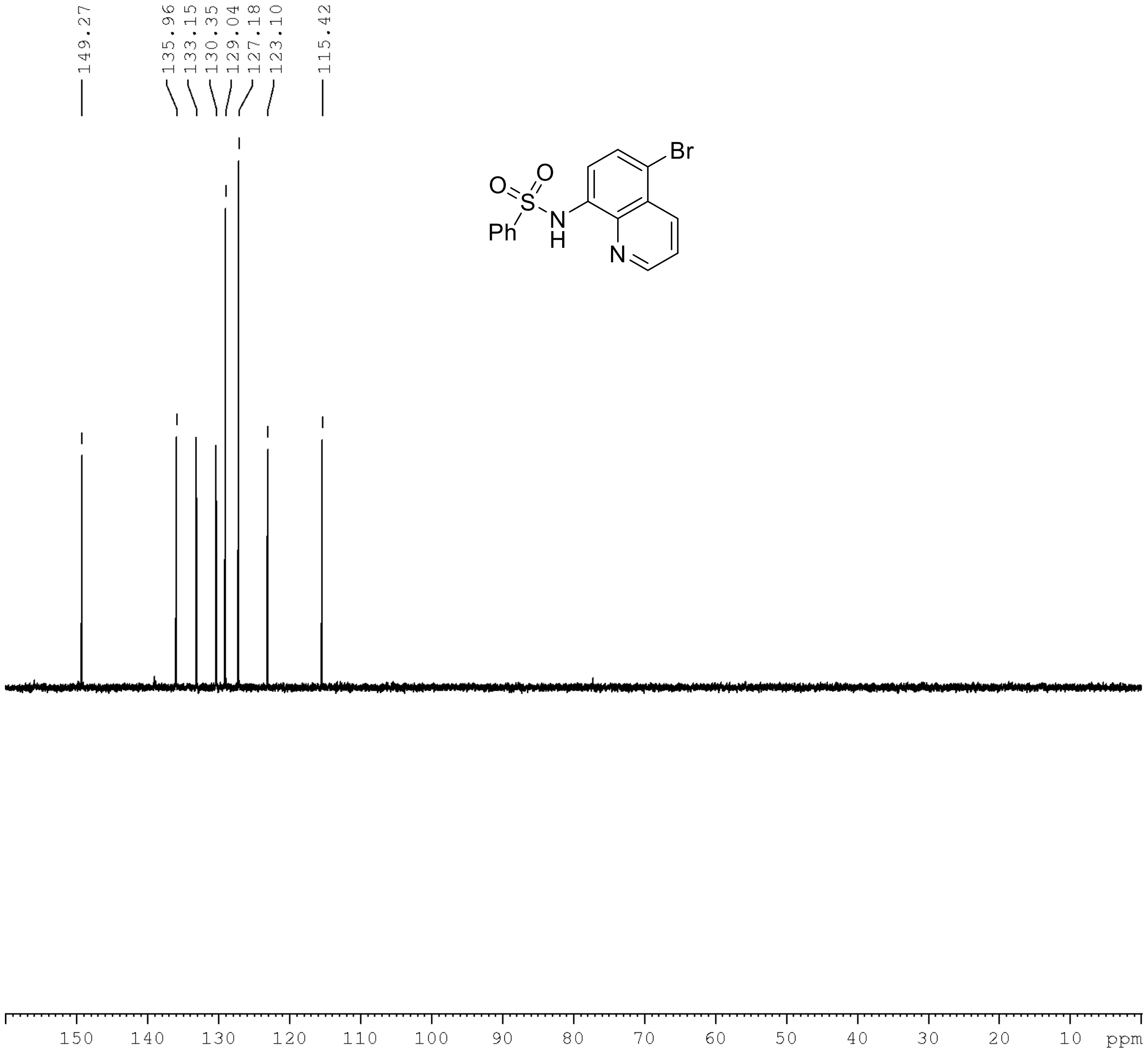

^{1}H NMR spectrum of compound **9c** (2:8, DMSO-*d6* + $CDCl_3$, 400 MHz)**:**

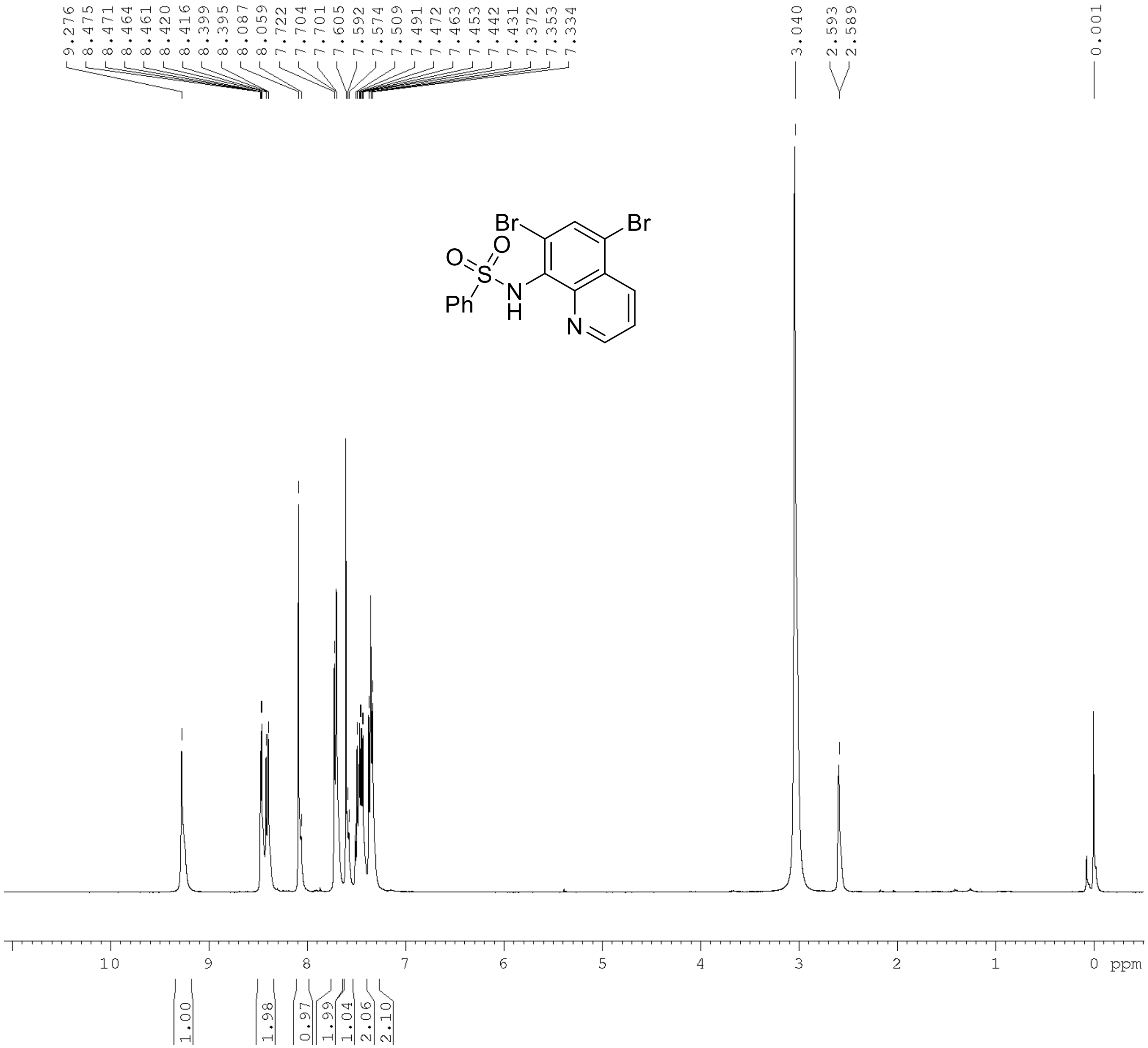

$^{13}C\{^1H\}$NMR spectrum of compound **9c** (2:8, DMSO-*d6* + $CDCl_3$, 100 MHz)**:**

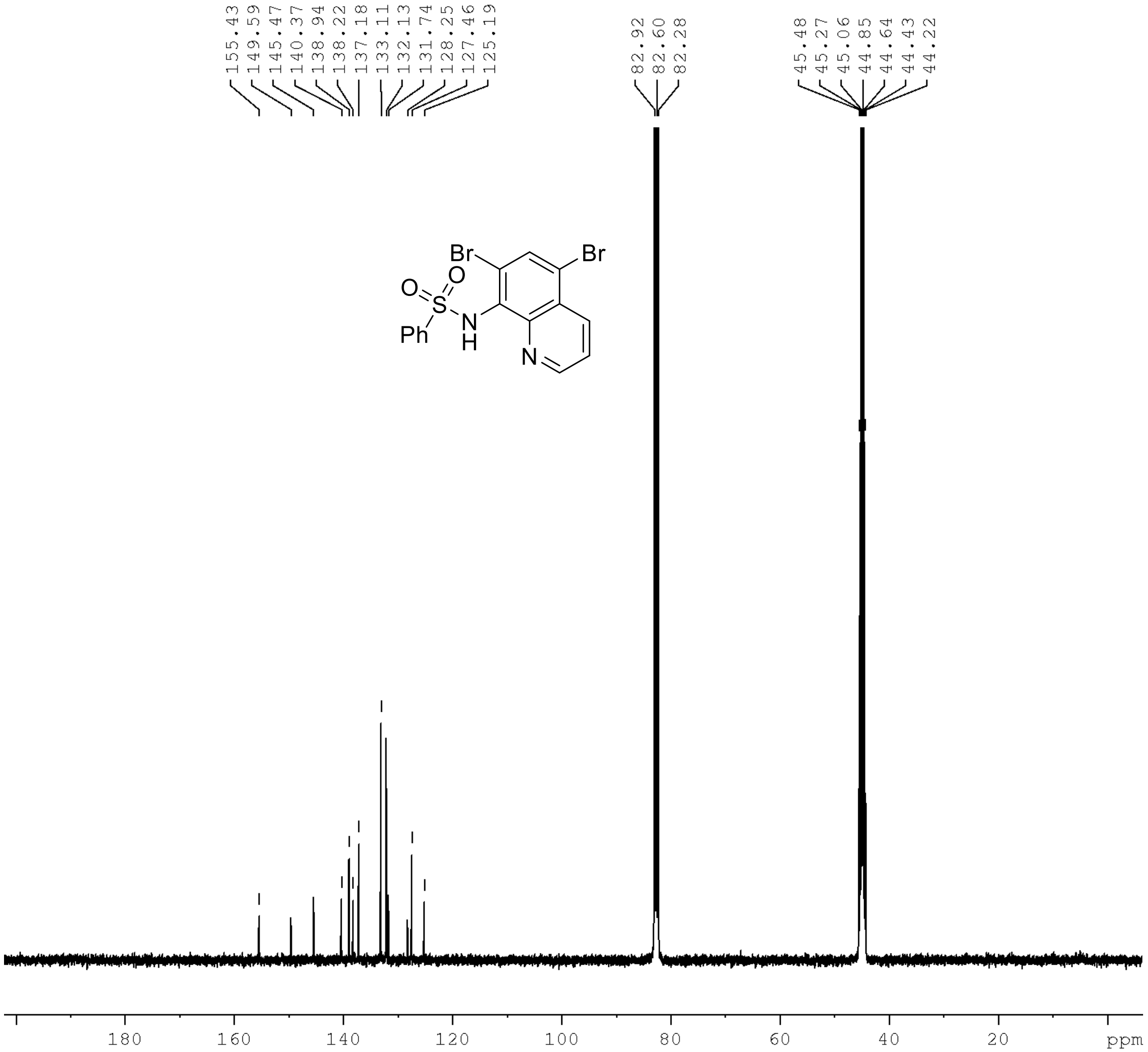

DEPT135 spectrum of compound **9c** (2:8, DMSO-*d6* + $CDCl_3$,100 MHz)**:**

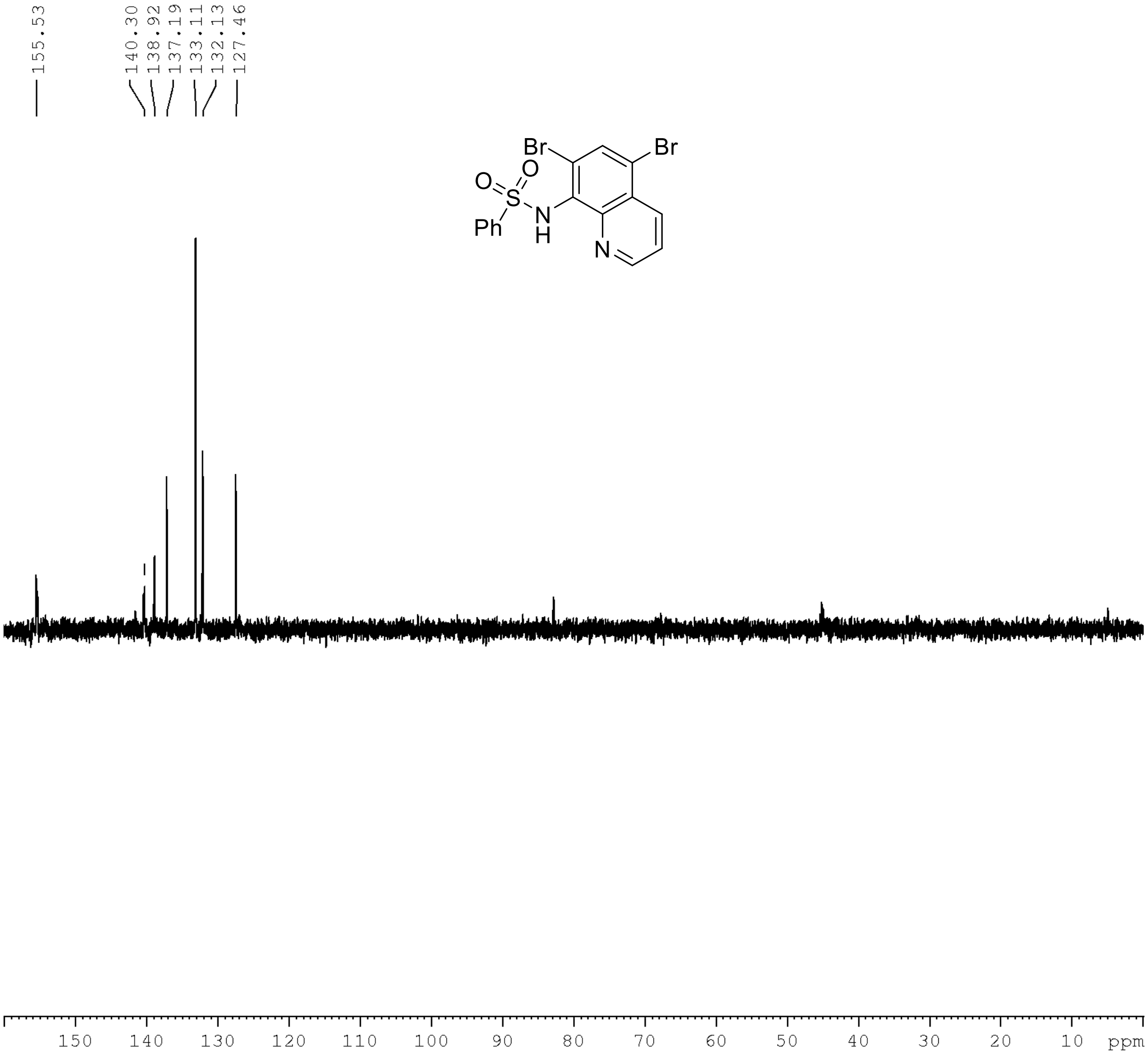

^{1}H NMR spectrum of compound **6d** ($CDCl_3$, 400 MHz)**:**

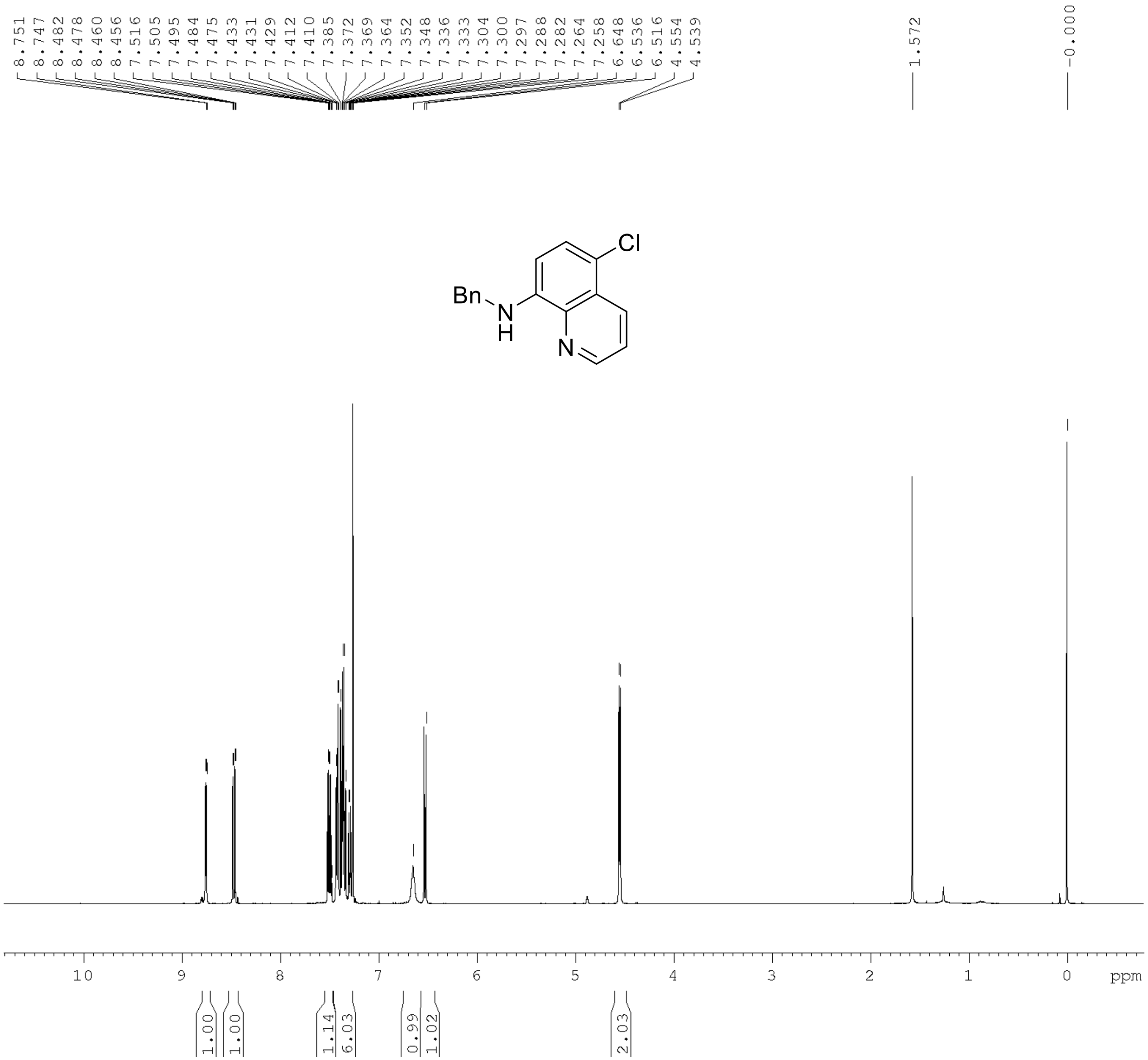

$^{13}C\{^{1}H\}$NMR spectrum of compound **6d** ($CDCl_3$, 100 MHz)**:**

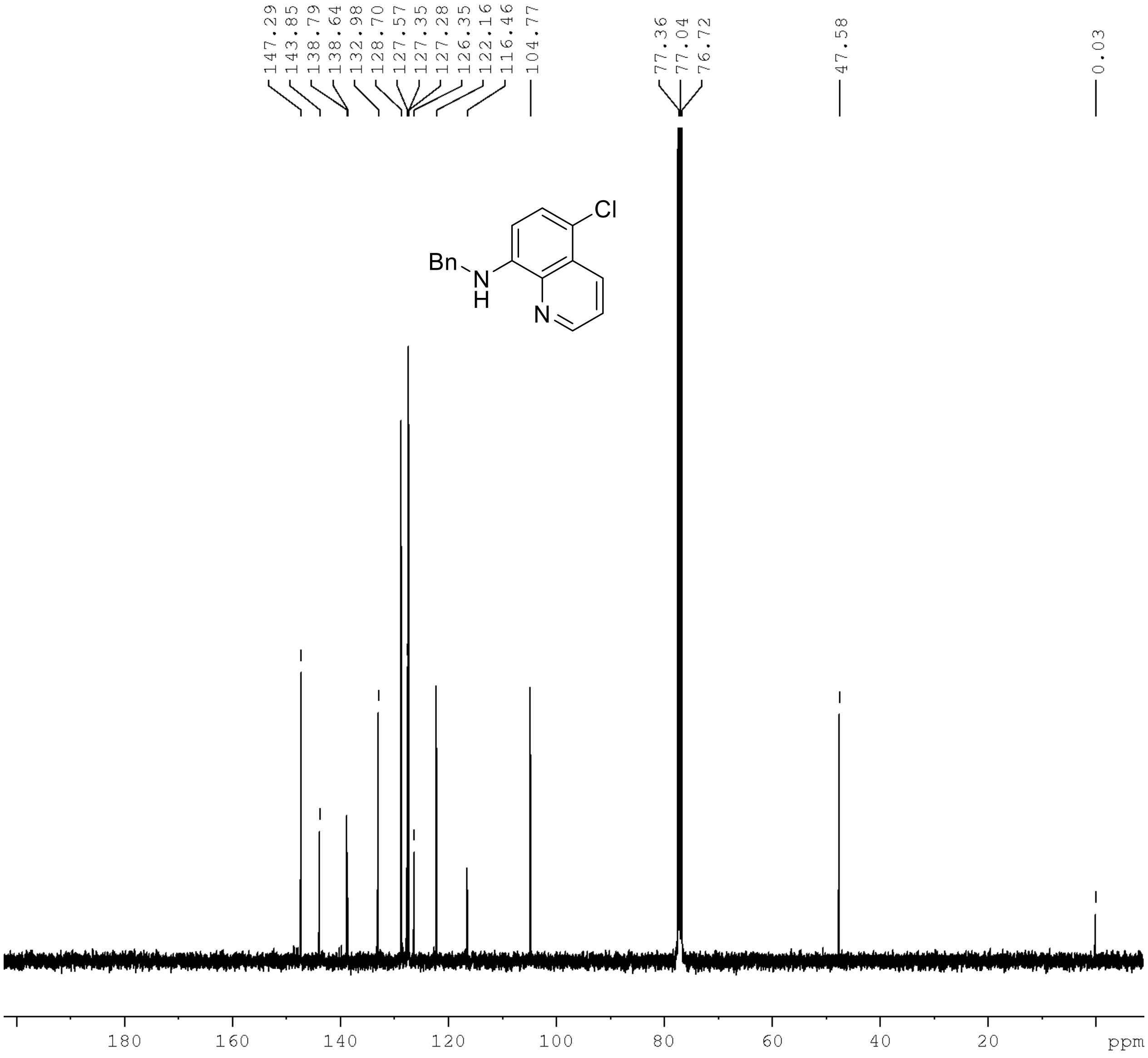

DEPT135 spectrum of compound **6d** ($CDCl_3$, 100 MHz)**:**

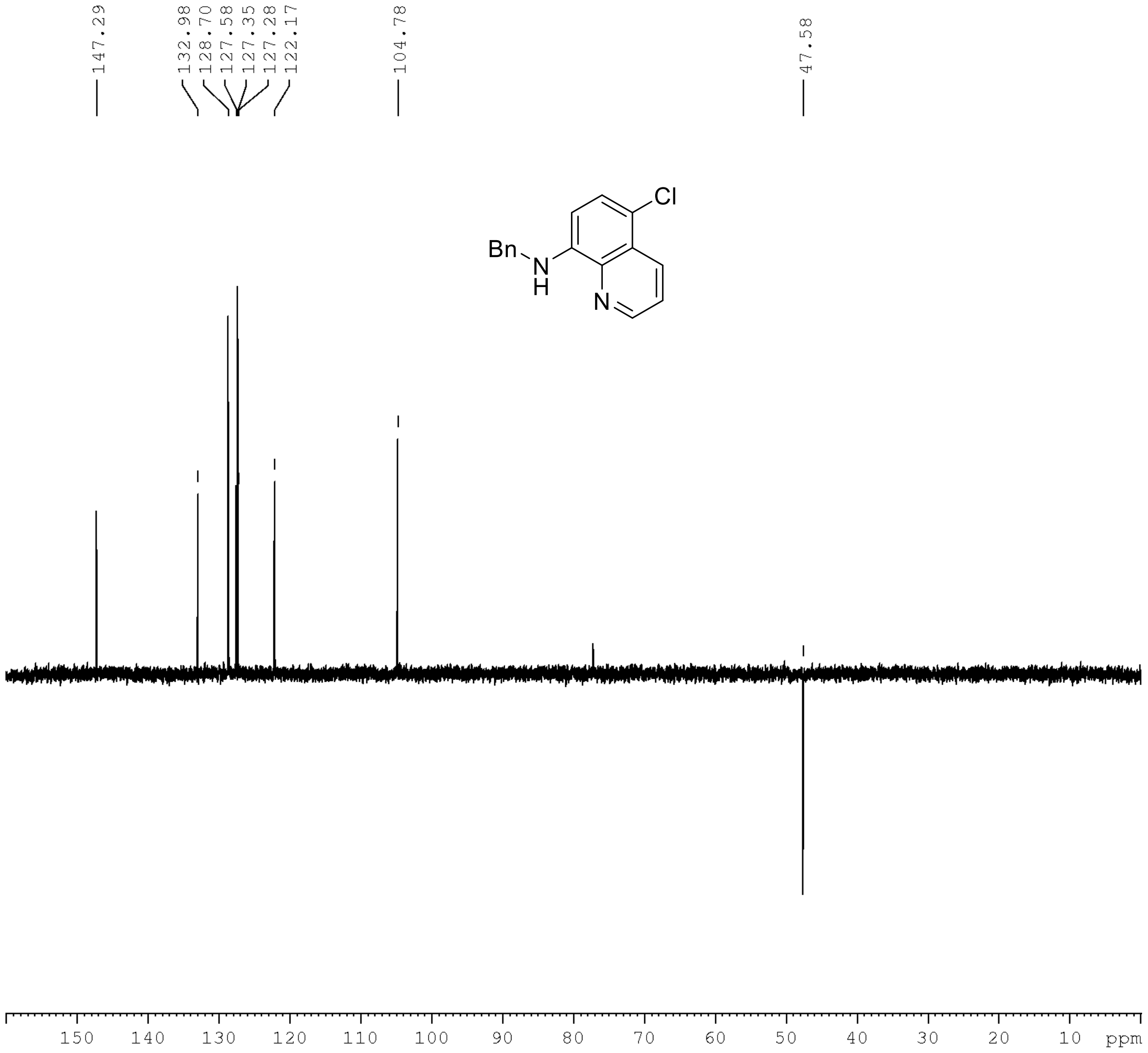

HRMS spectrum of compound **6d:**

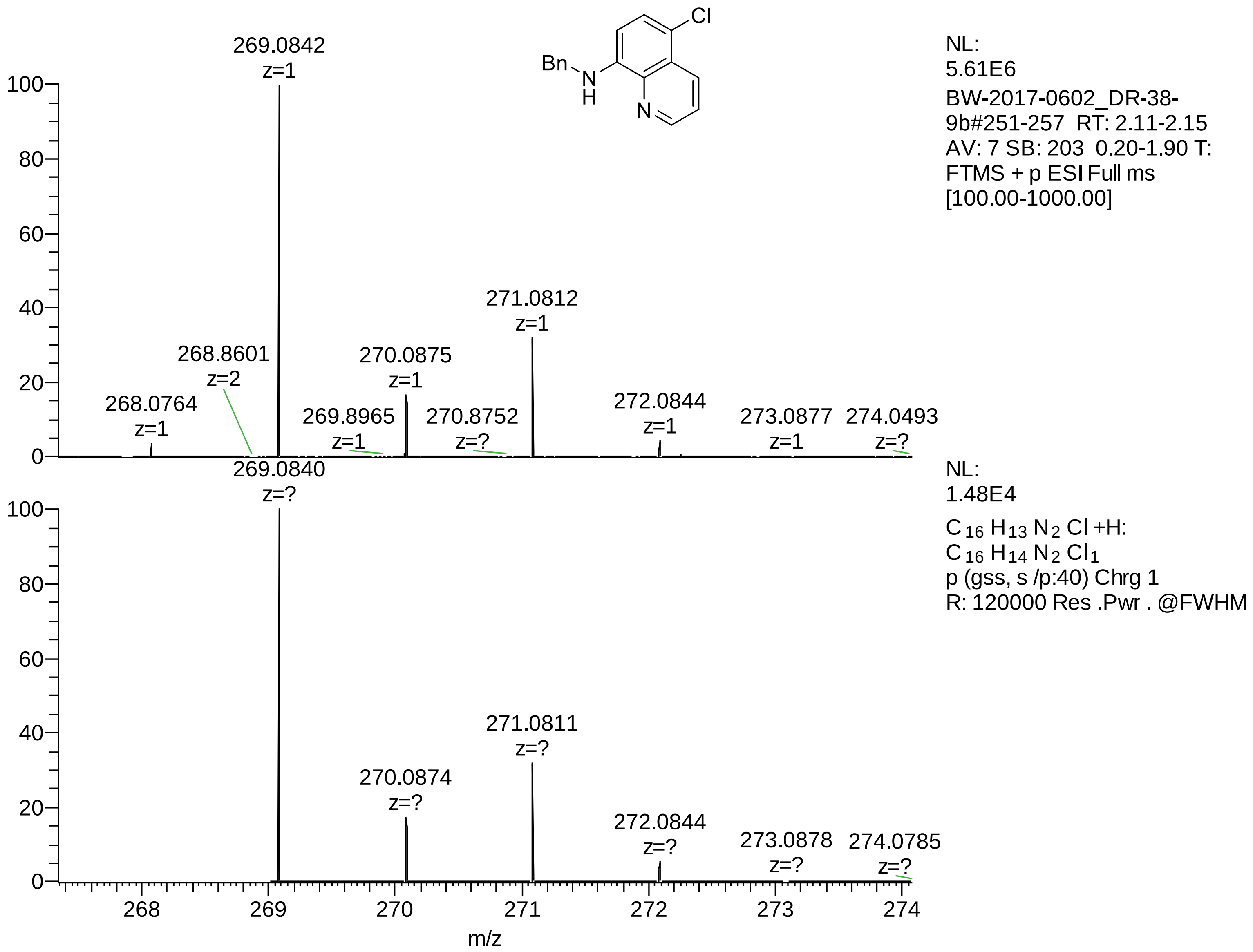

^{1}H NMR spectrum of compound **7d** ($CDCl_3$, 400 MHz)**:**

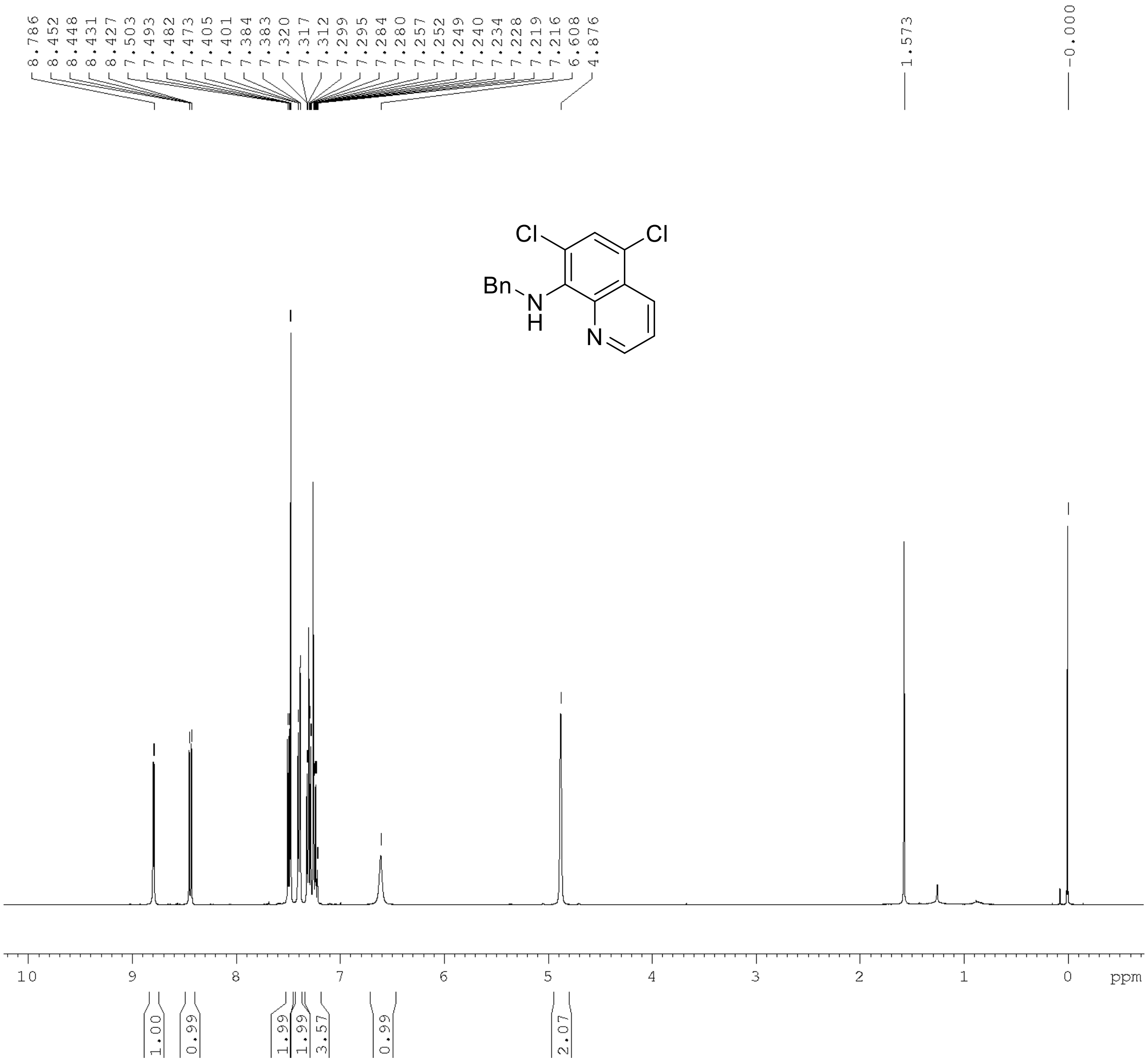

$^{13}C\{^{1}H\}$NMR spectrum of compound **7d** ($CDCl_3$, 100 MHz)**:**

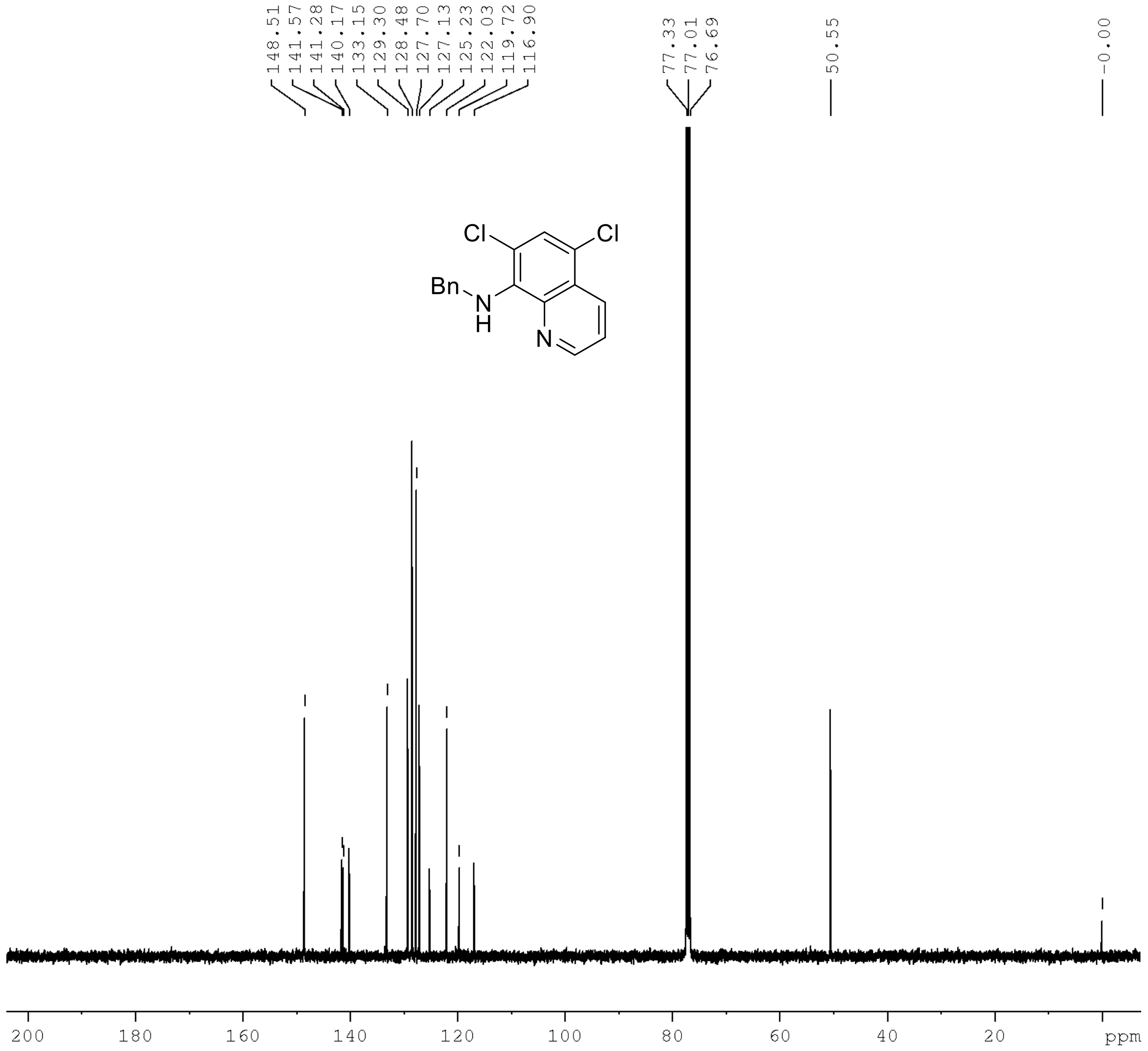

DEPT135 spectrum of compound **7d** ($CDCl_3$, 100 MHz)**:**

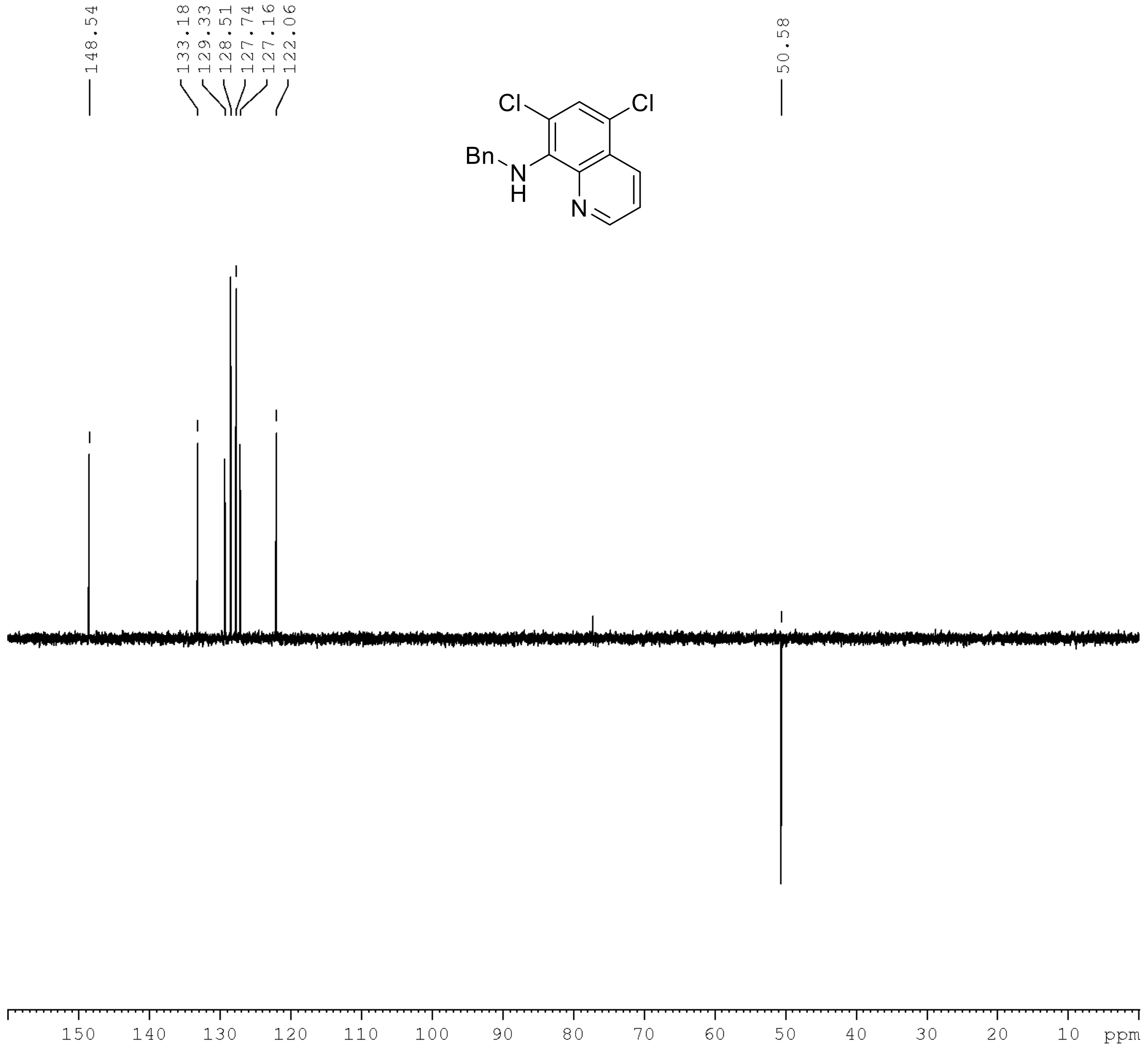

HRMS spectrum of compound **7d:**

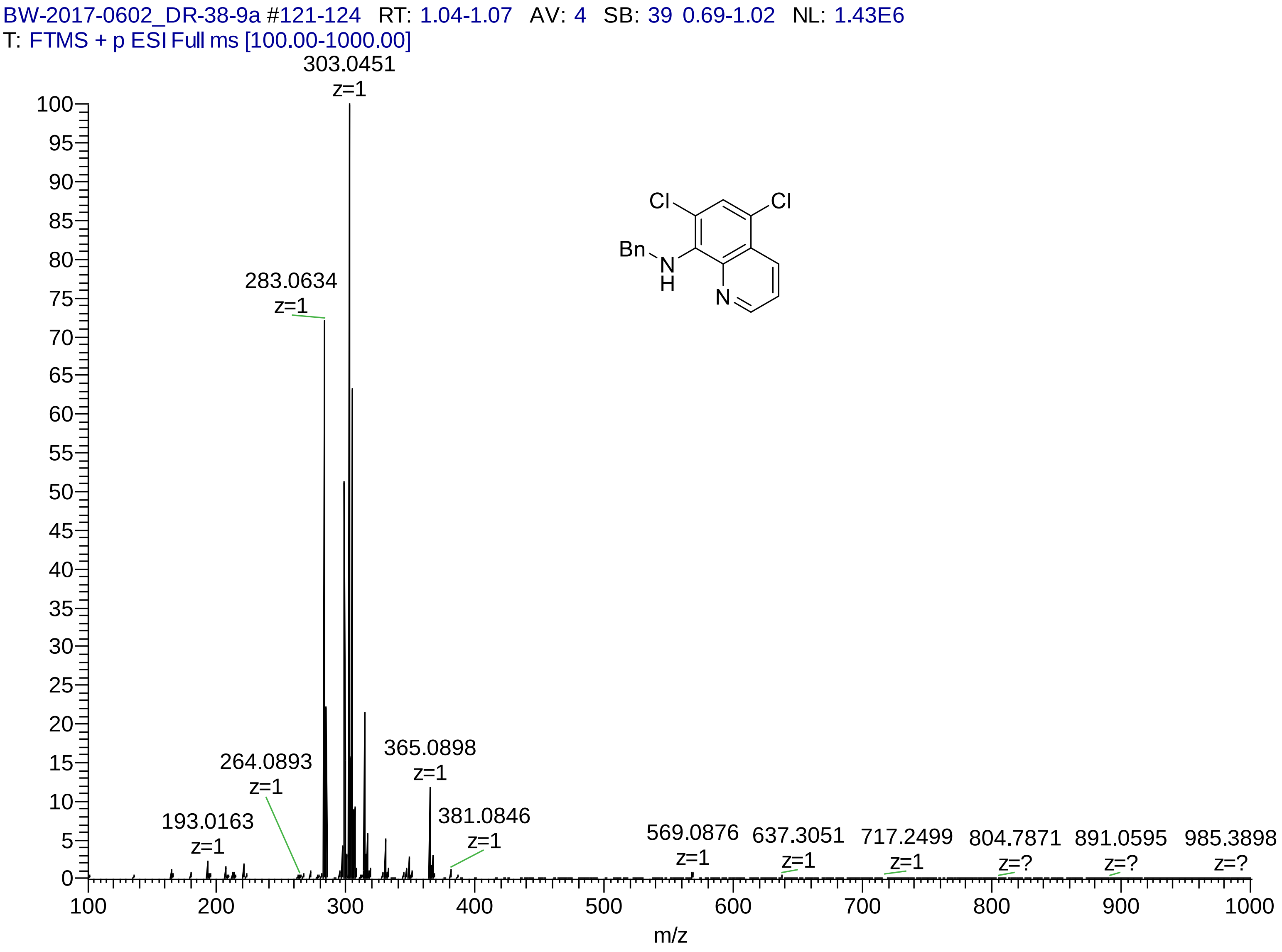

^{1}H NMR spectrum of compound **8d** ($CDCl_3$, 400 MHz)**:**

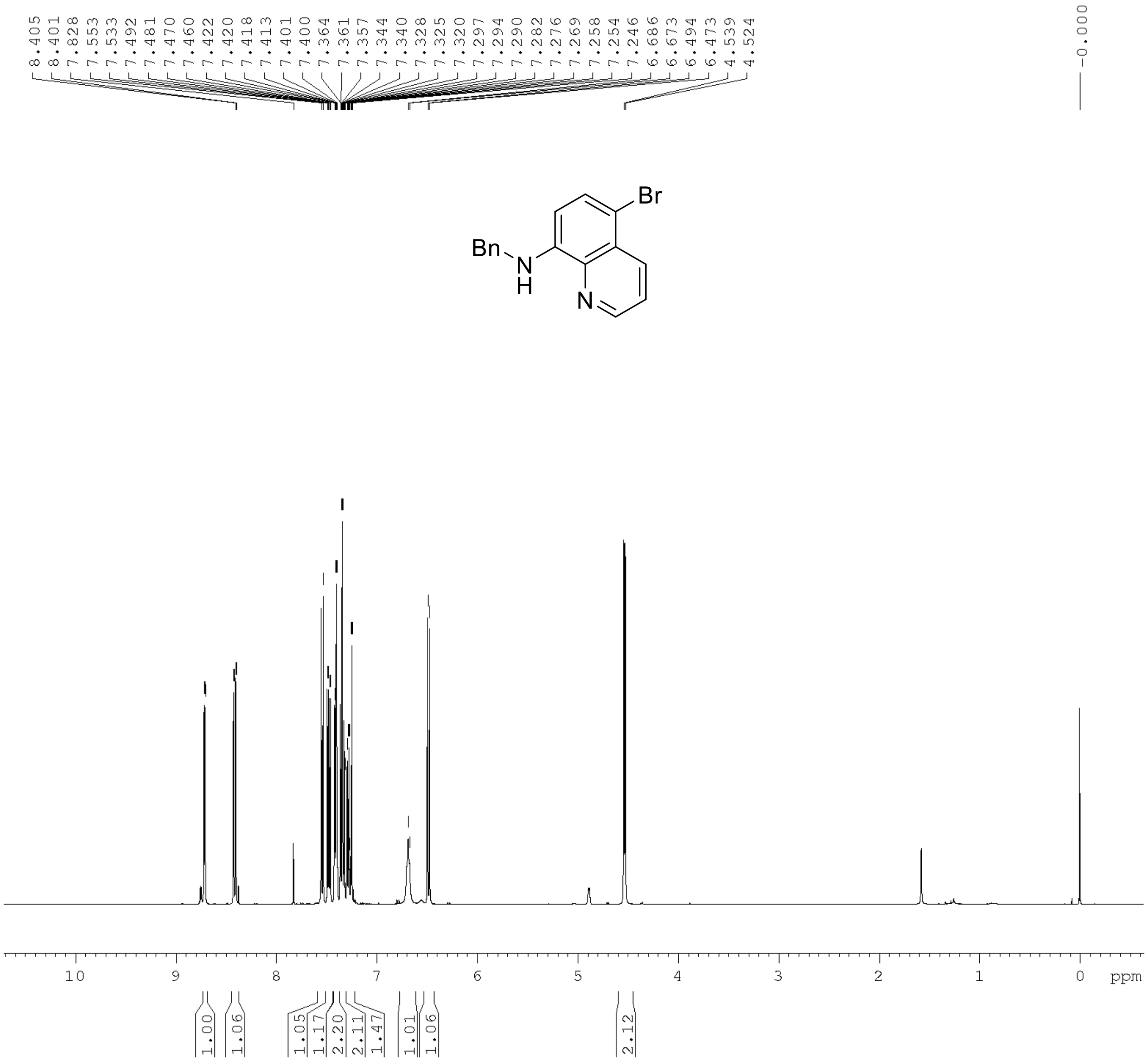

$^{13}C\{^{1}H\}$NMR spectrum of compound **8d** ($CDCl_3$, 100 MHz)**:**

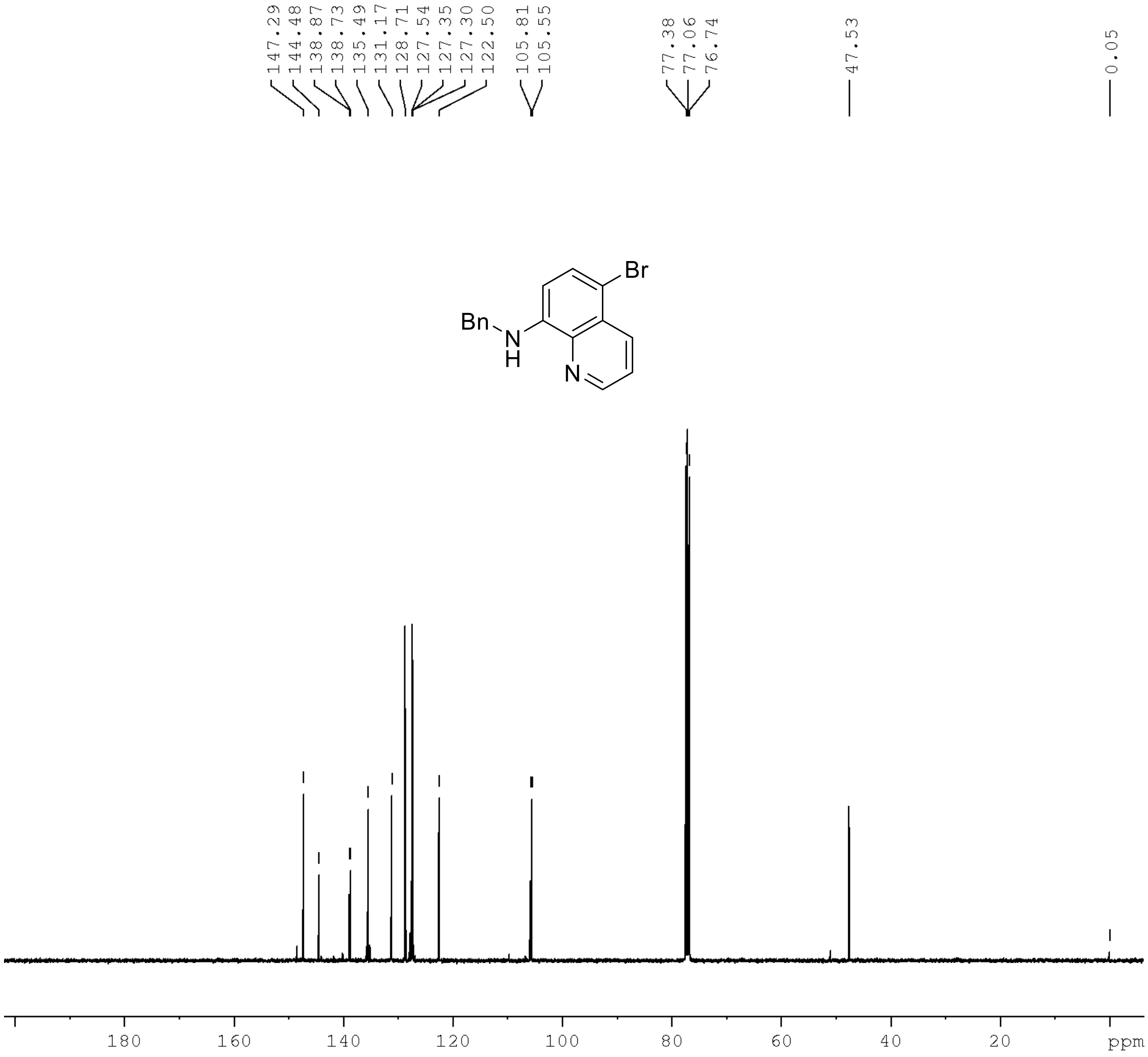

DEPT135 spectrum of compound **8d** ($CDCl_3$, 100 MHz)**:**

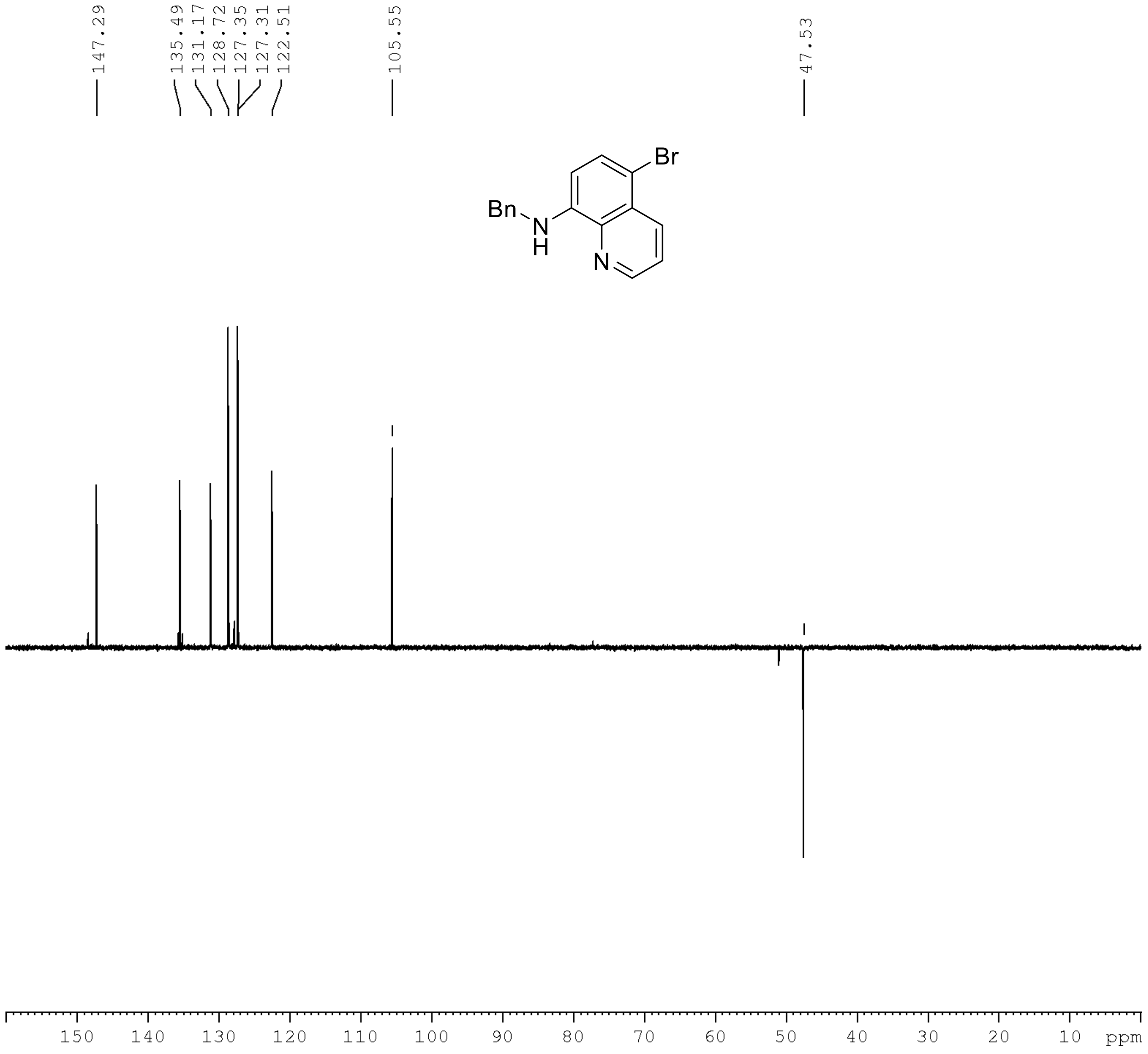

HRMS spectrum of compound **8d:**

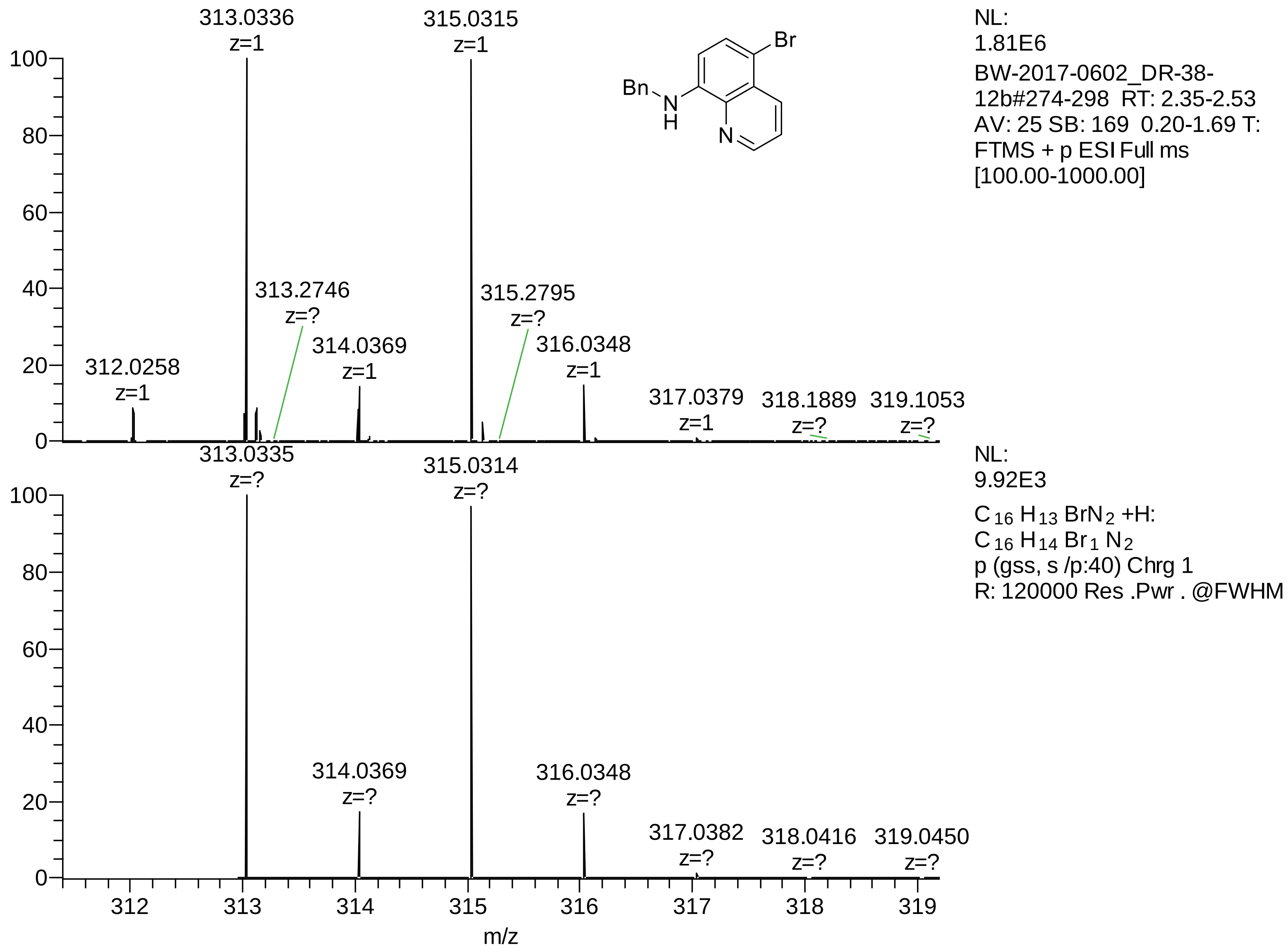

^{1}H NMR spectrum of compound **9d** ($CDCl_3$, 400 MHz)**:**

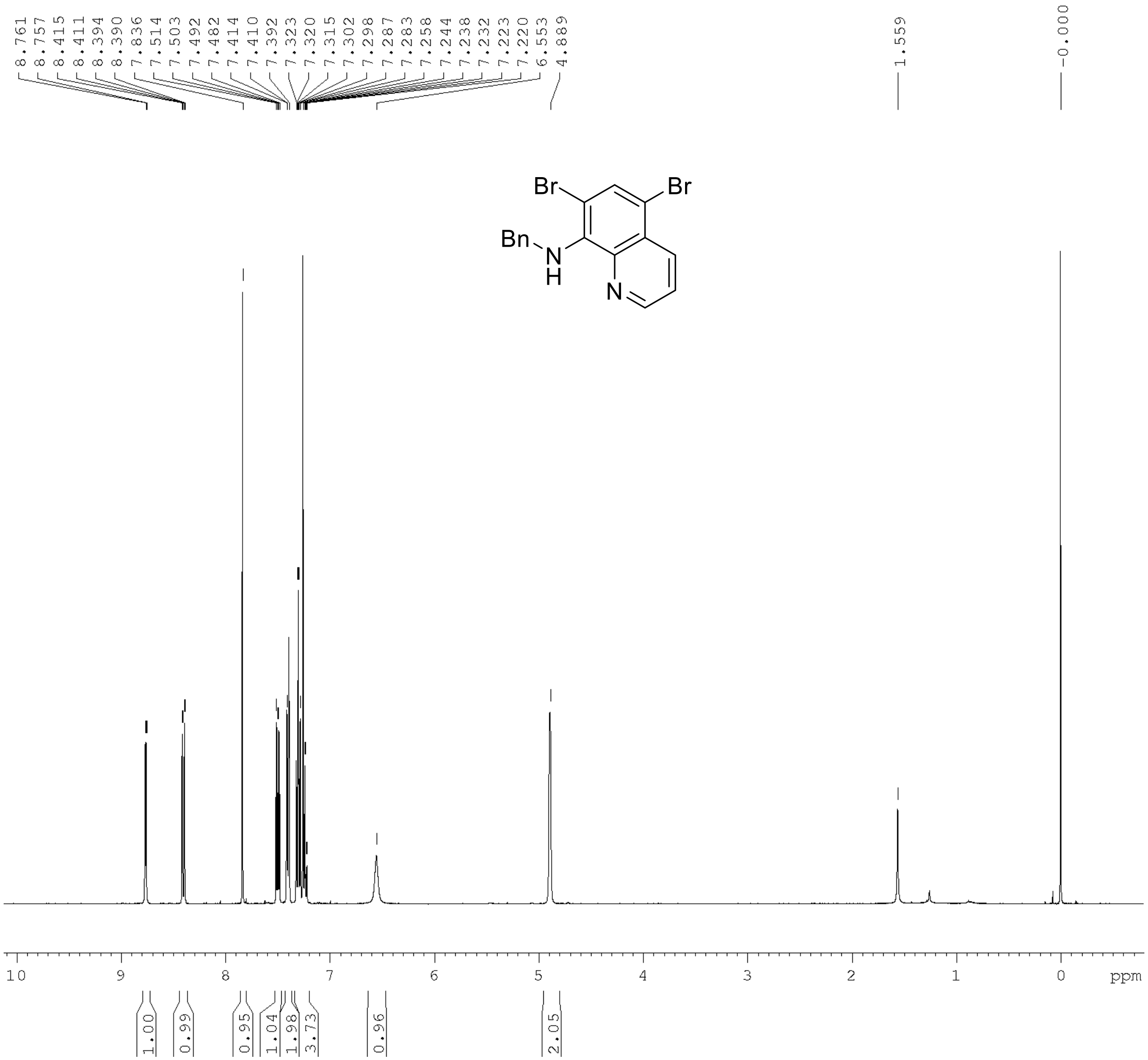

$^{13}C\{^{1}H\}$NMR spectrum of compound **9d** ($CDCl_3$, 100 MHz)**:**

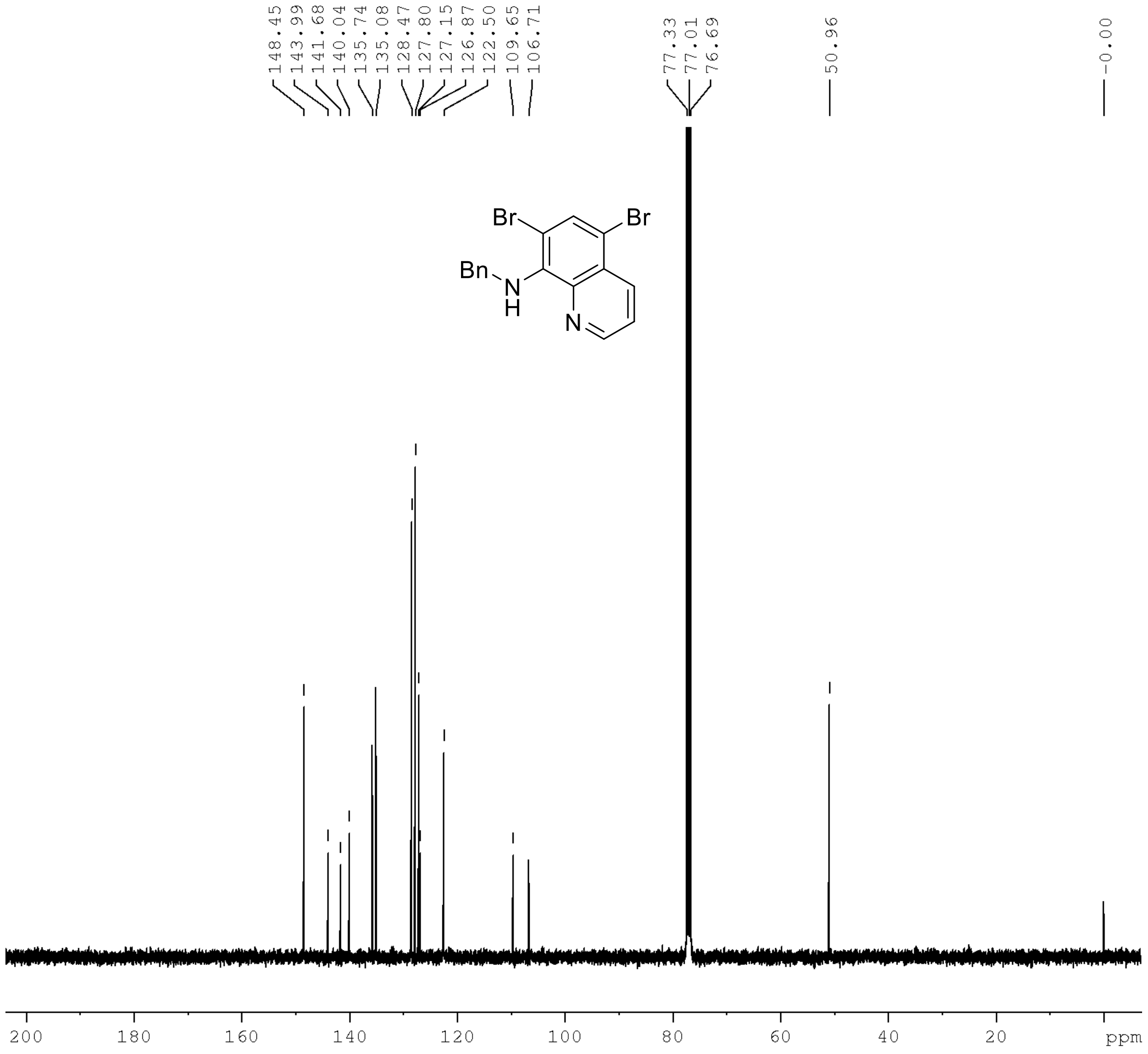

DEPT135 spectrum of compound **9d** ($CDCl_3$, 100 MHz)**:**

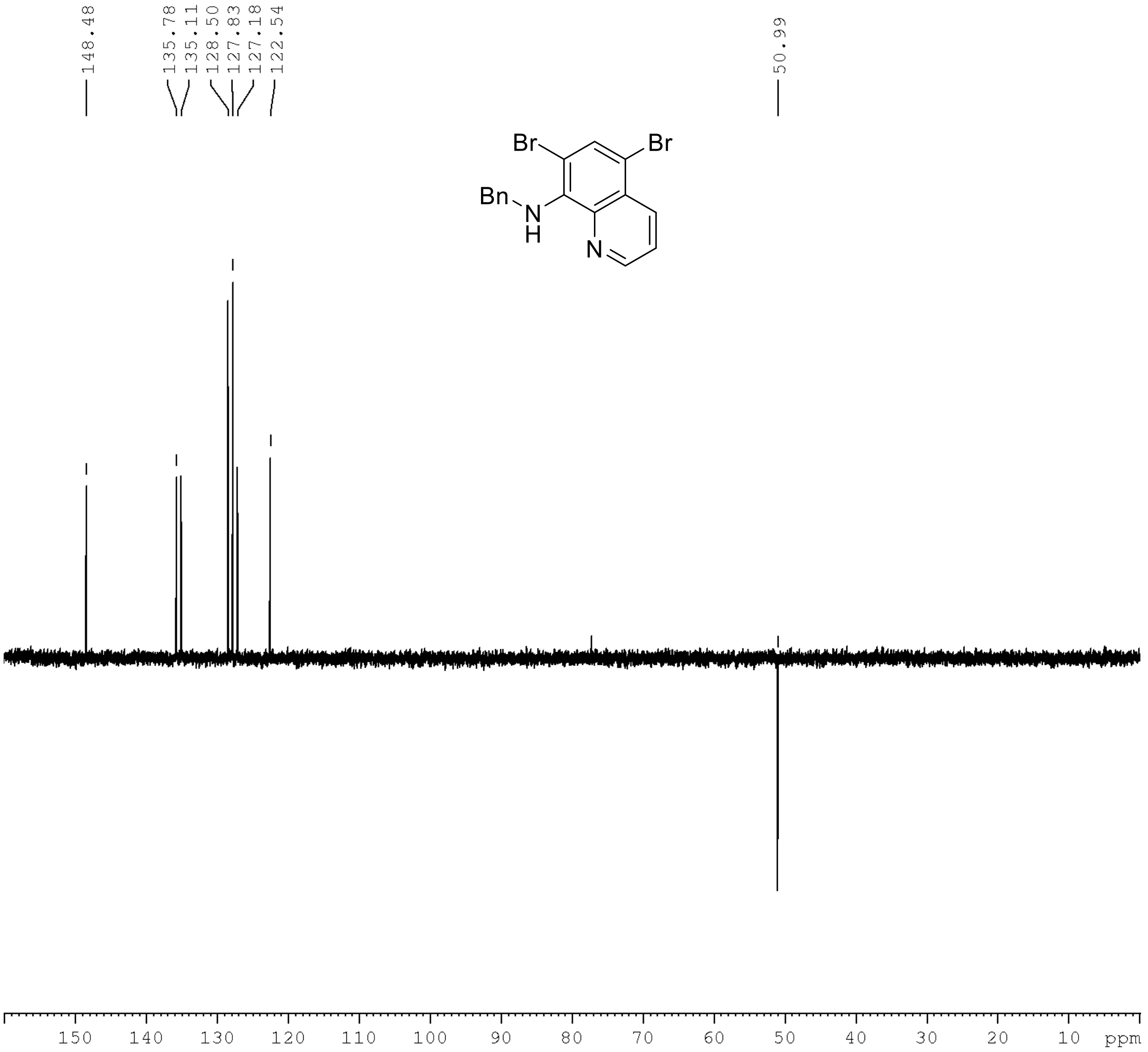

^{1}H NMR spectrum of compound **6e** ($CDCl_3$, 400 MHz)**:**

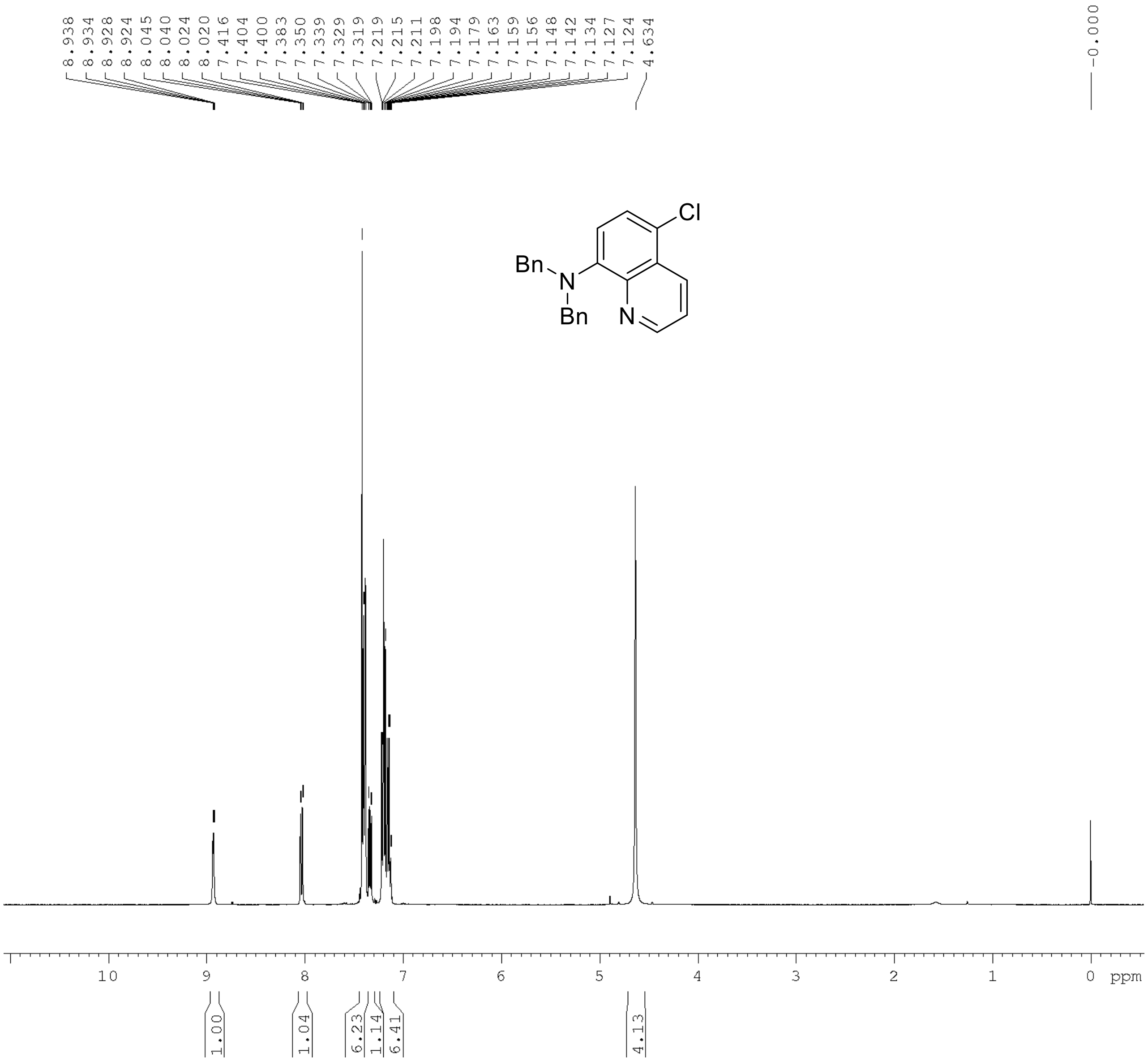

$^{13}C\{^{1}H\}$NMR spectrum of compound **6e** ($CDCl_3$, 100 MHz):

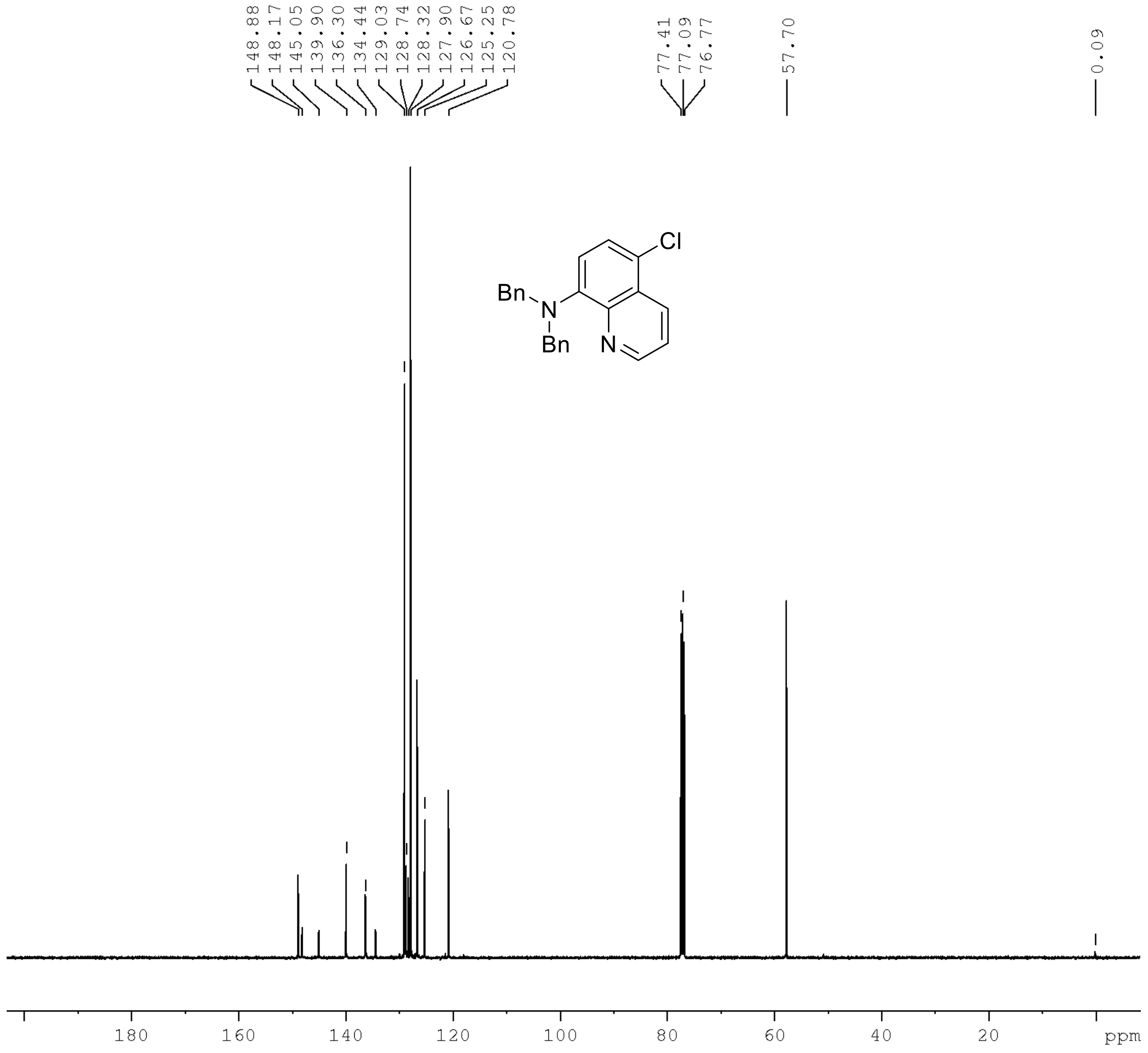

DEPT135 spectrum of compound **6e** ($CDCl_3$, 100 MHz)**:**

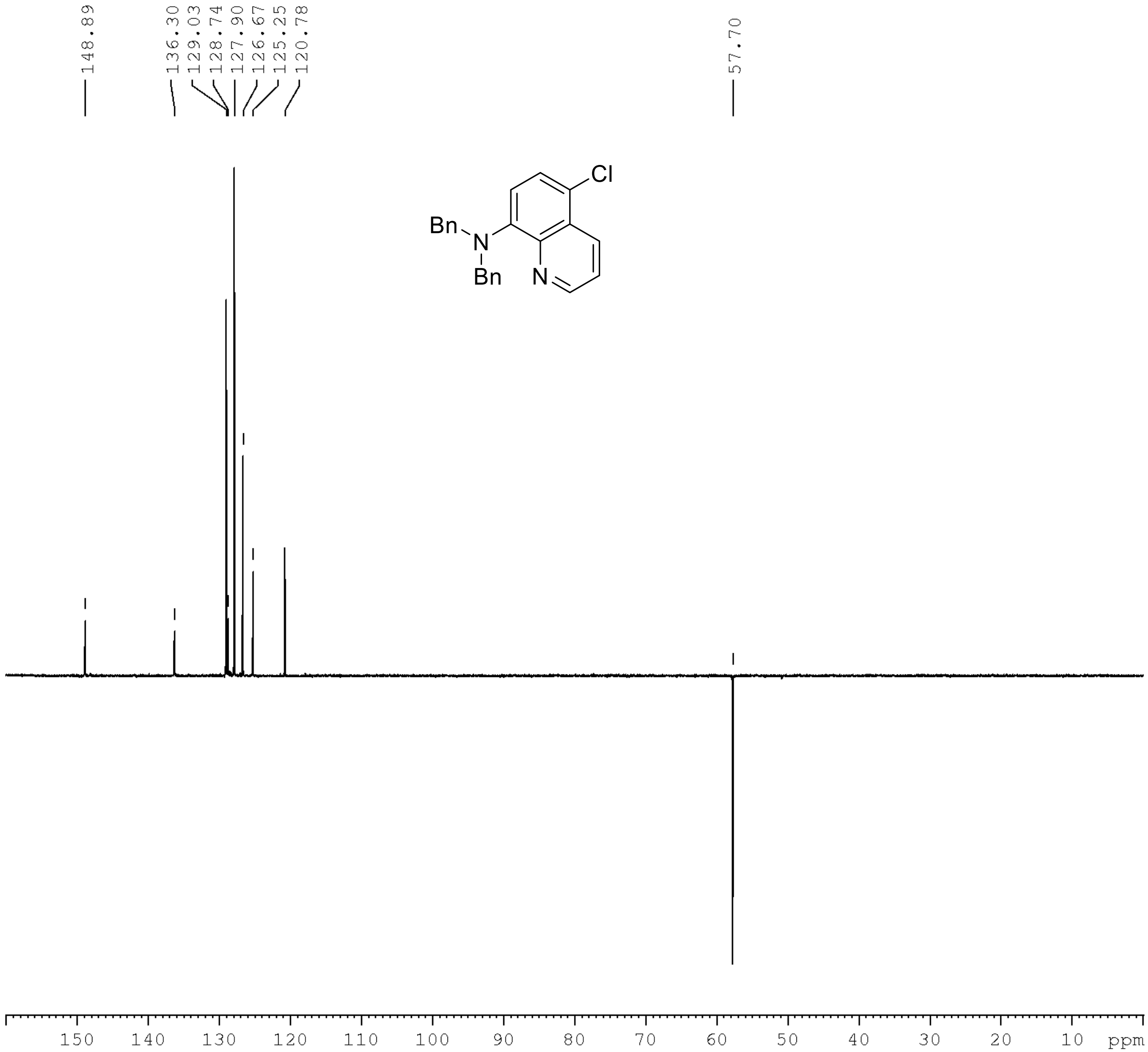

HRMS spectrum of compound **6e:**

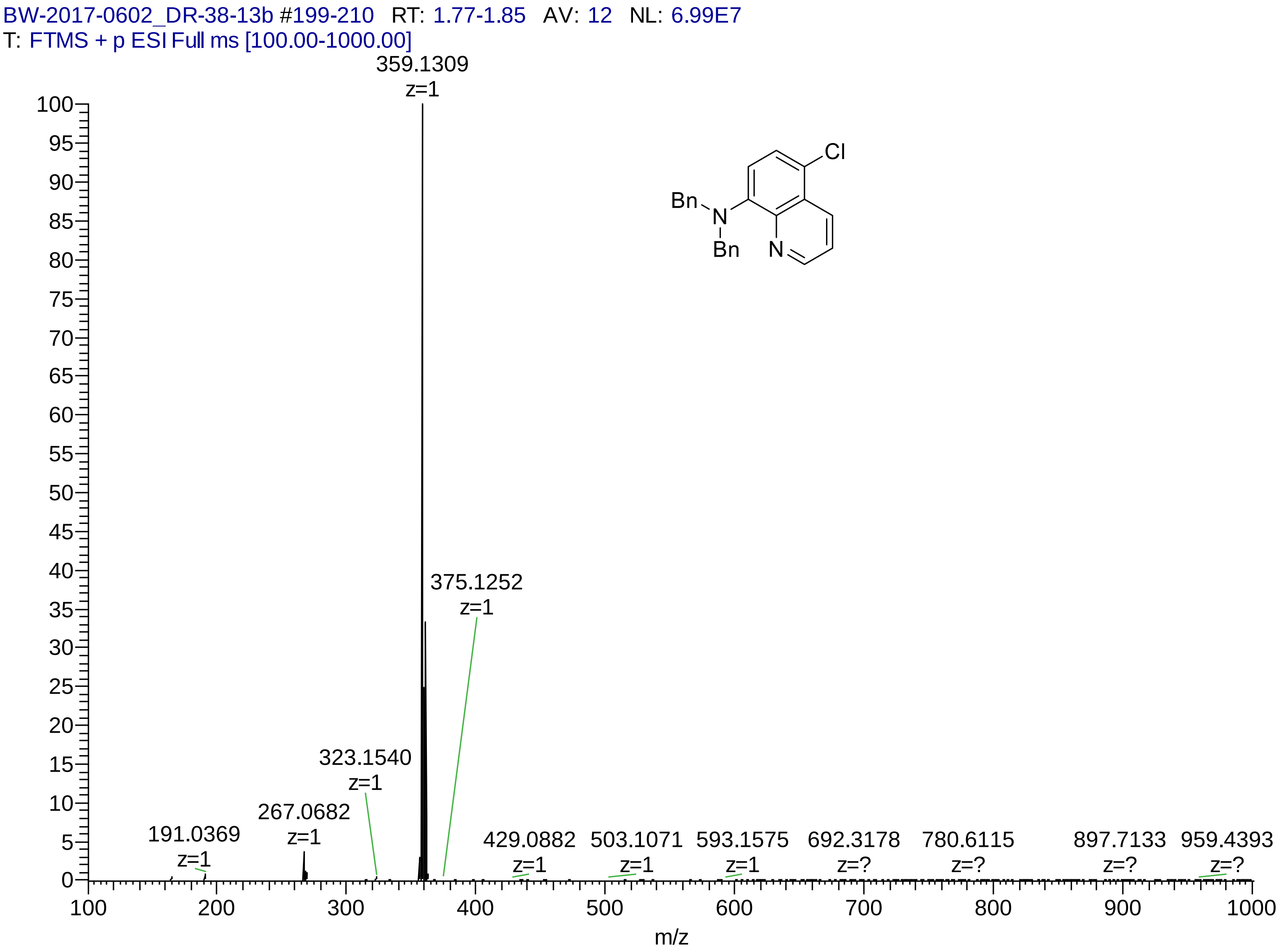

^{1}H NMR spectrum of compound **7e** ($CDCl_3$, 400 MHz)**:**

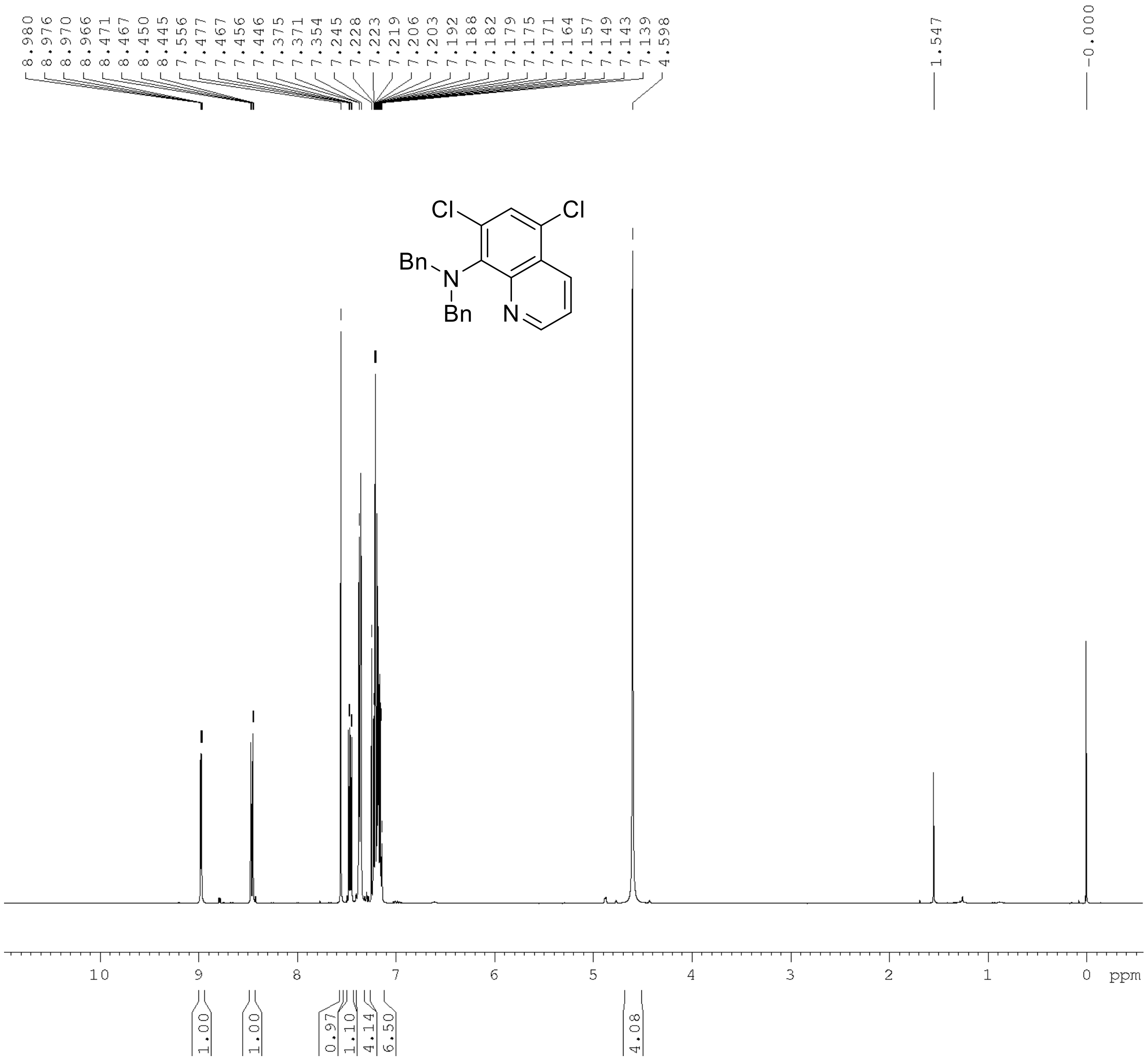

$^{13}C\{^1H\}$NMR spectrum of compound **7e** ($CDCl_3$, 100 MHz)**:**

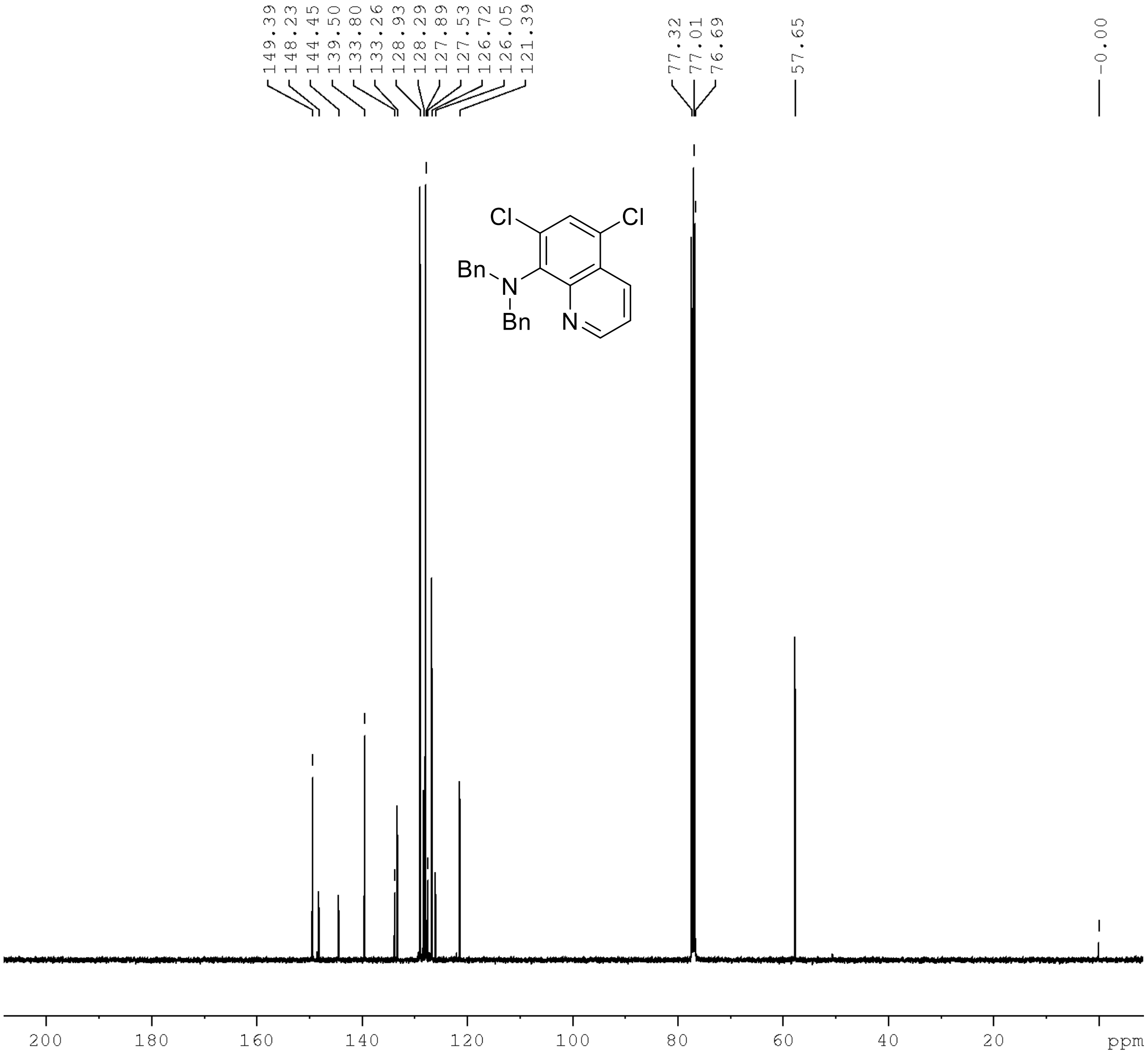

DEPT135 spectrum of compound **7e** ($CDCl_3$, 100 MHz)**:**

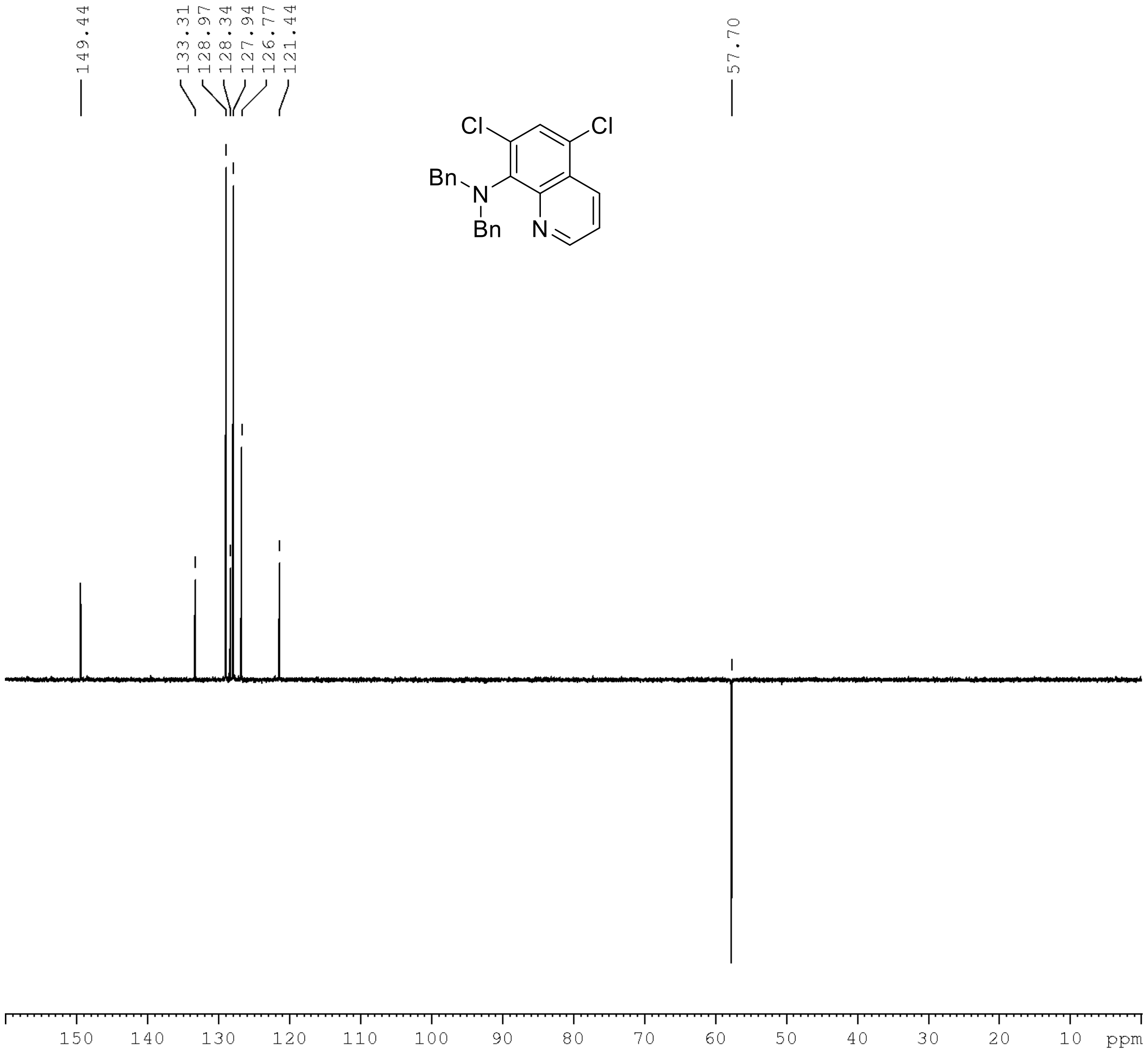

HRMS spectrum of compound **7e:**

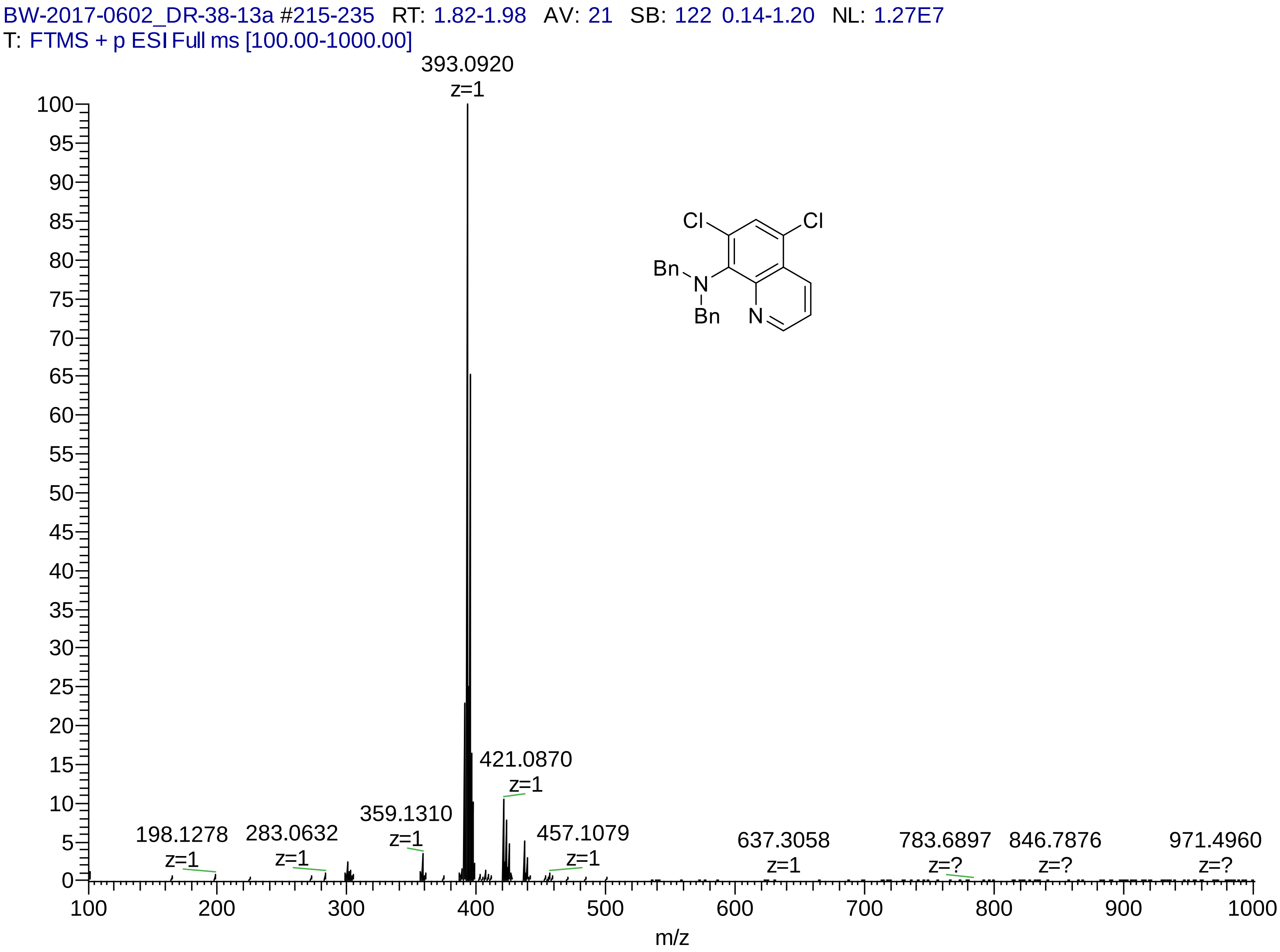

^{1}H NMR spectrum of compound **8e** ($CDCl_3$, 400 MHz)**:**

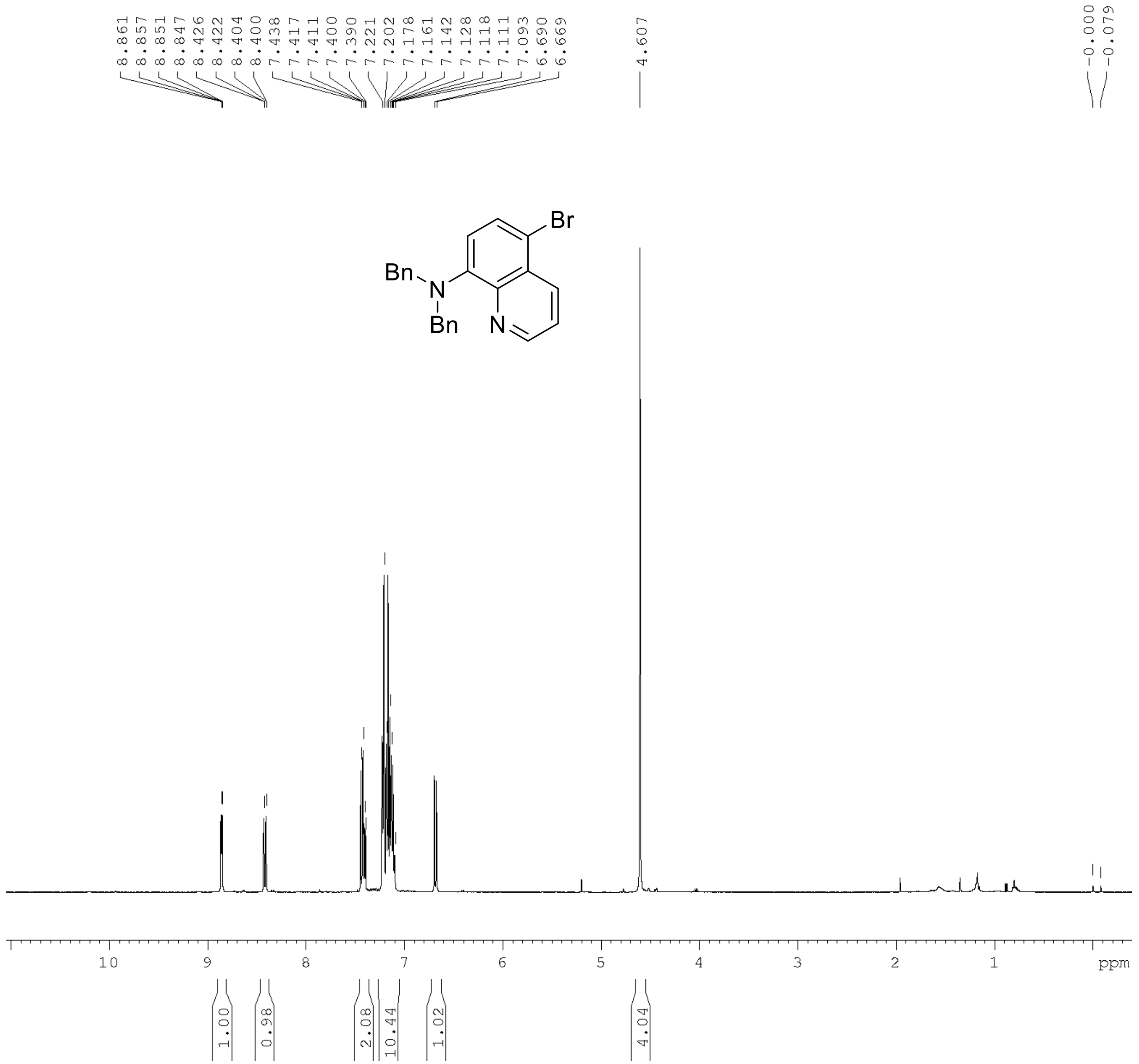

$^{13}C\{^1H\}$NMR spectrum of compound **8e** ($CDCl_3$, 100 MHz):

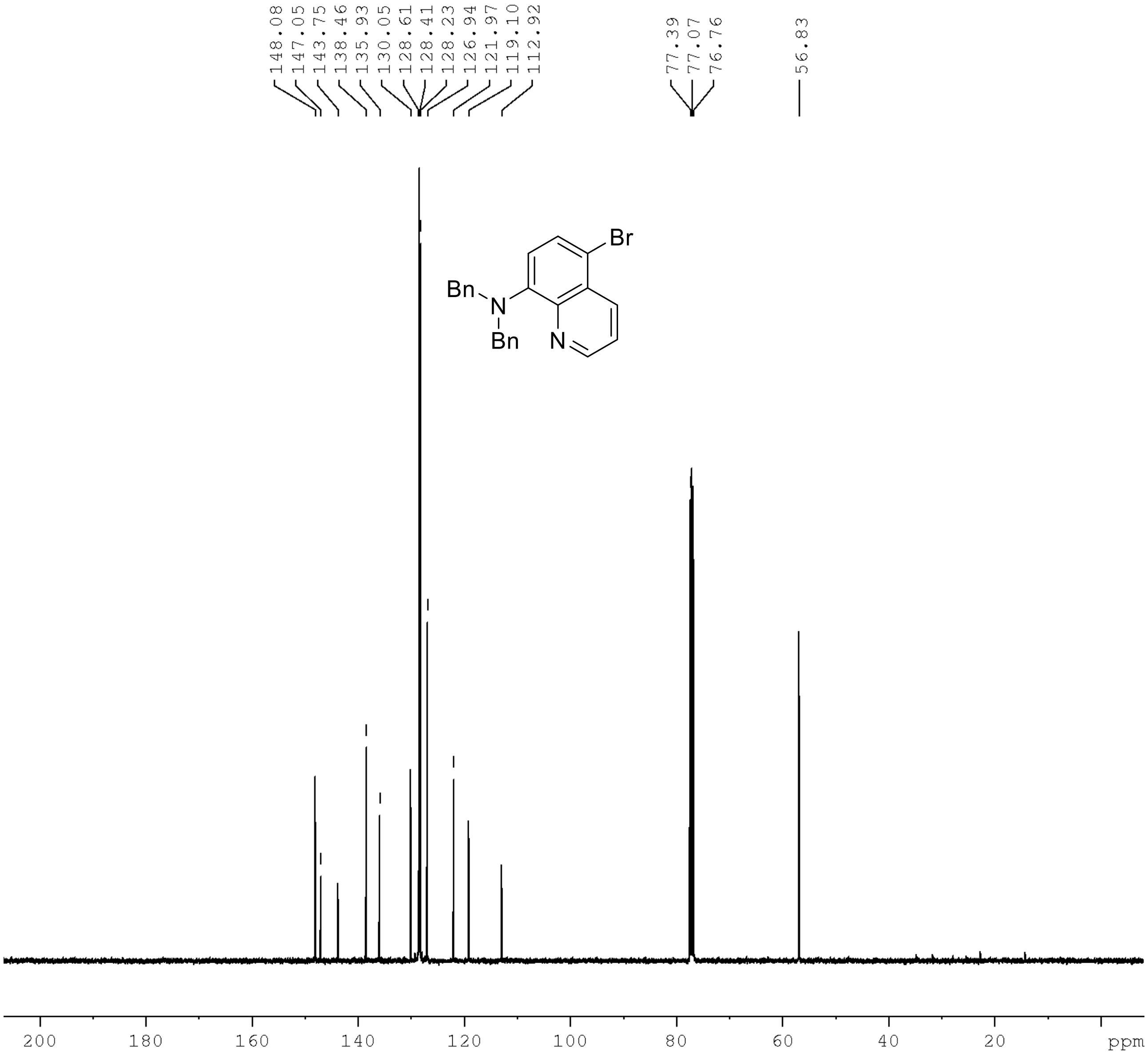

DEPT135 spectrum of compound **8e** ($CDCl_3$, 100 MHz):

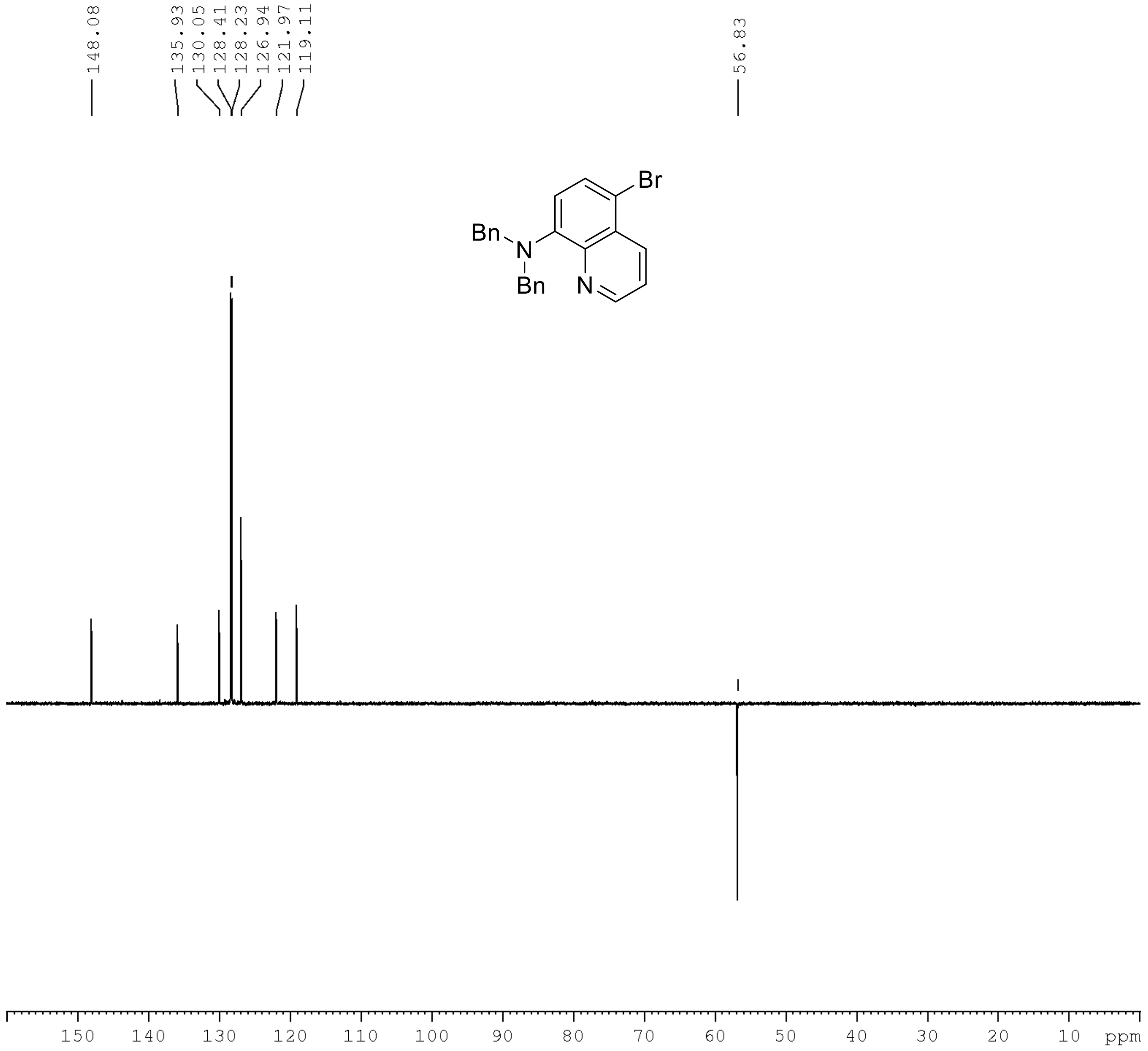

^{1}H NMR spectrum of compound **9e** ($CDCl_3$, 400 MHz)**:**

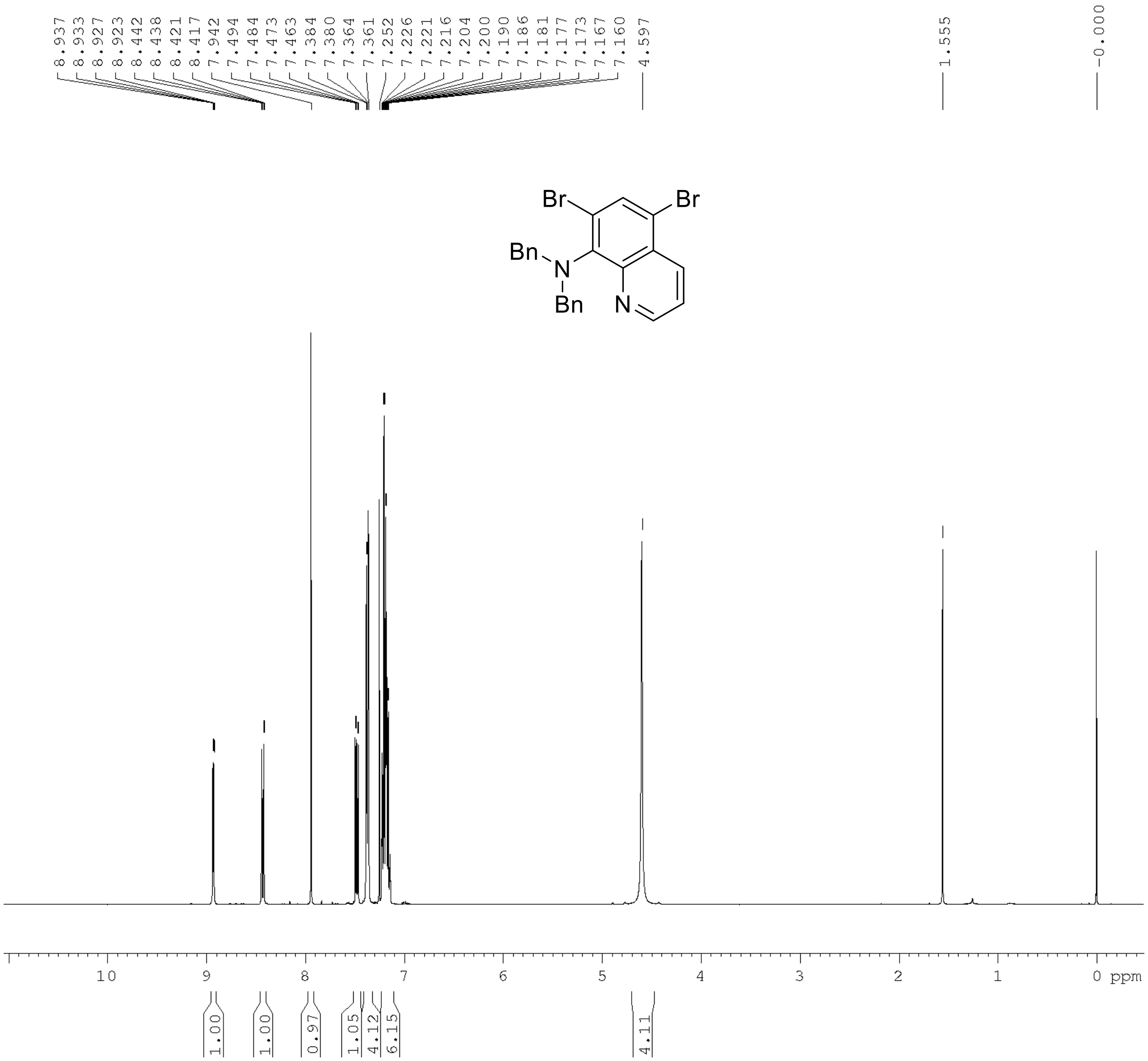

$^{13}C\{^1H\}$NMR spectrum of compound **9e** ($CDCl_3$, 100 MHz):

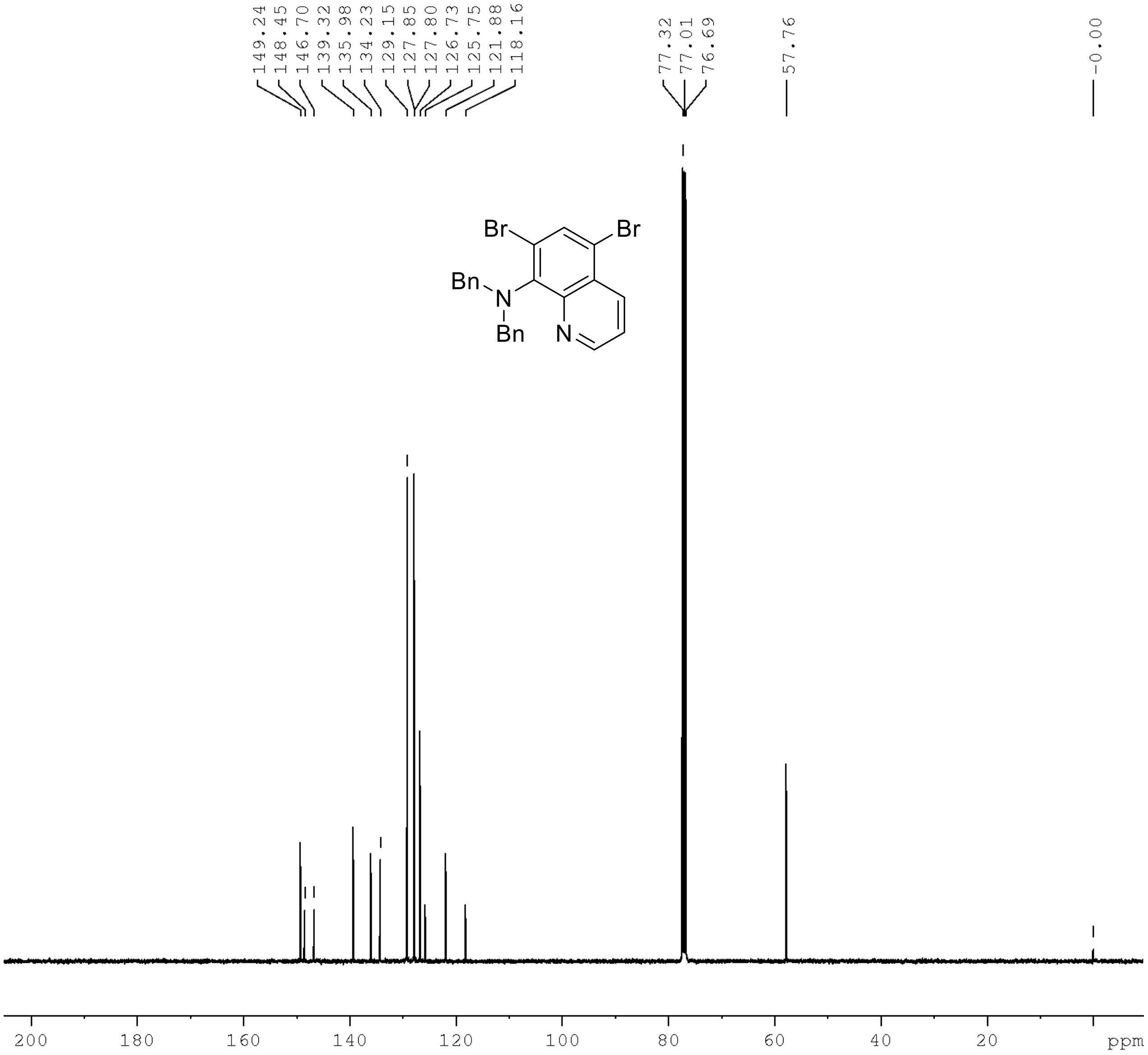

DEPT135 spectrum of compound **9e** ($CDCl_3$, 100 MHz)**:**

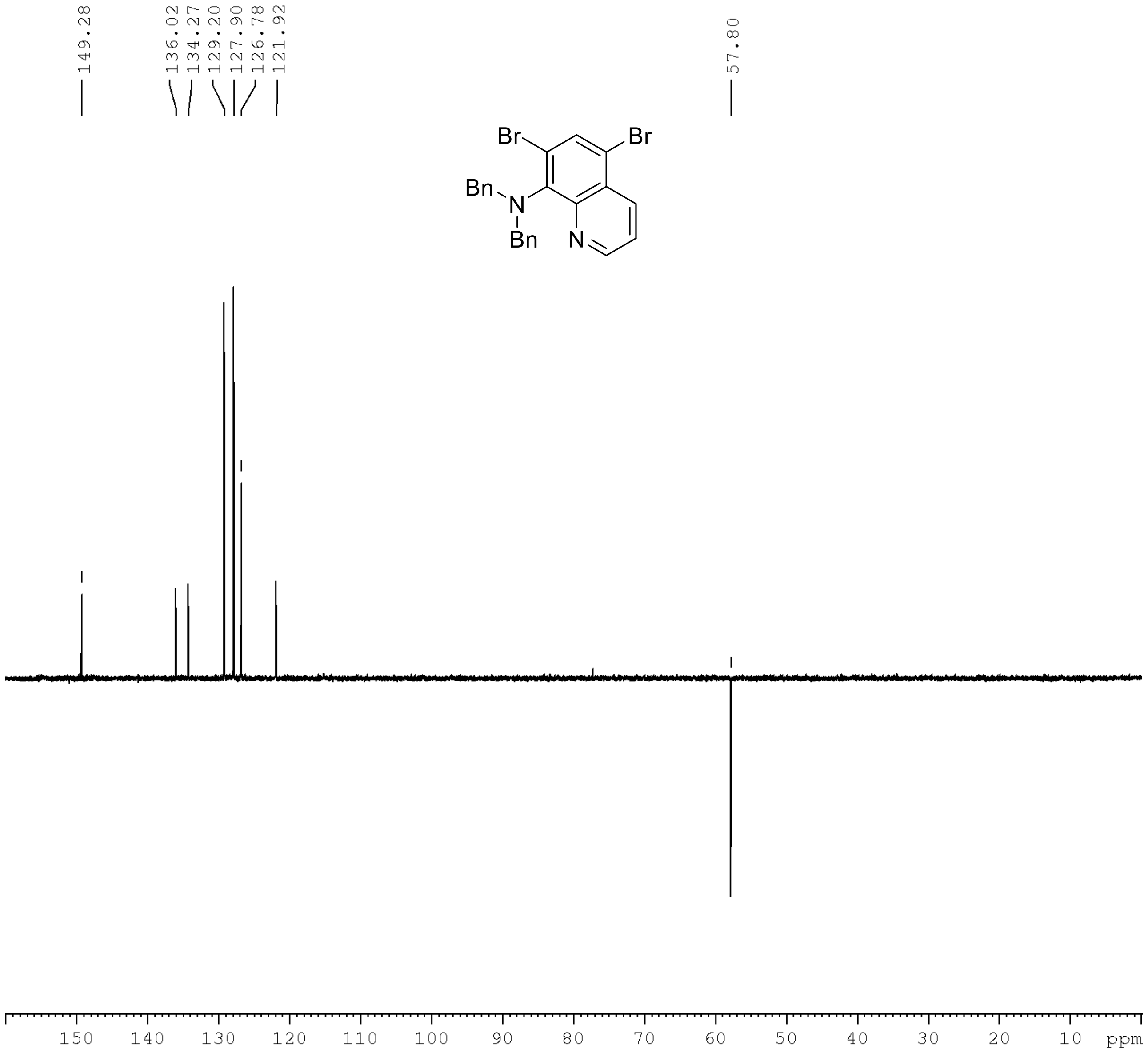

HRMS spectrum of compound **9e:**

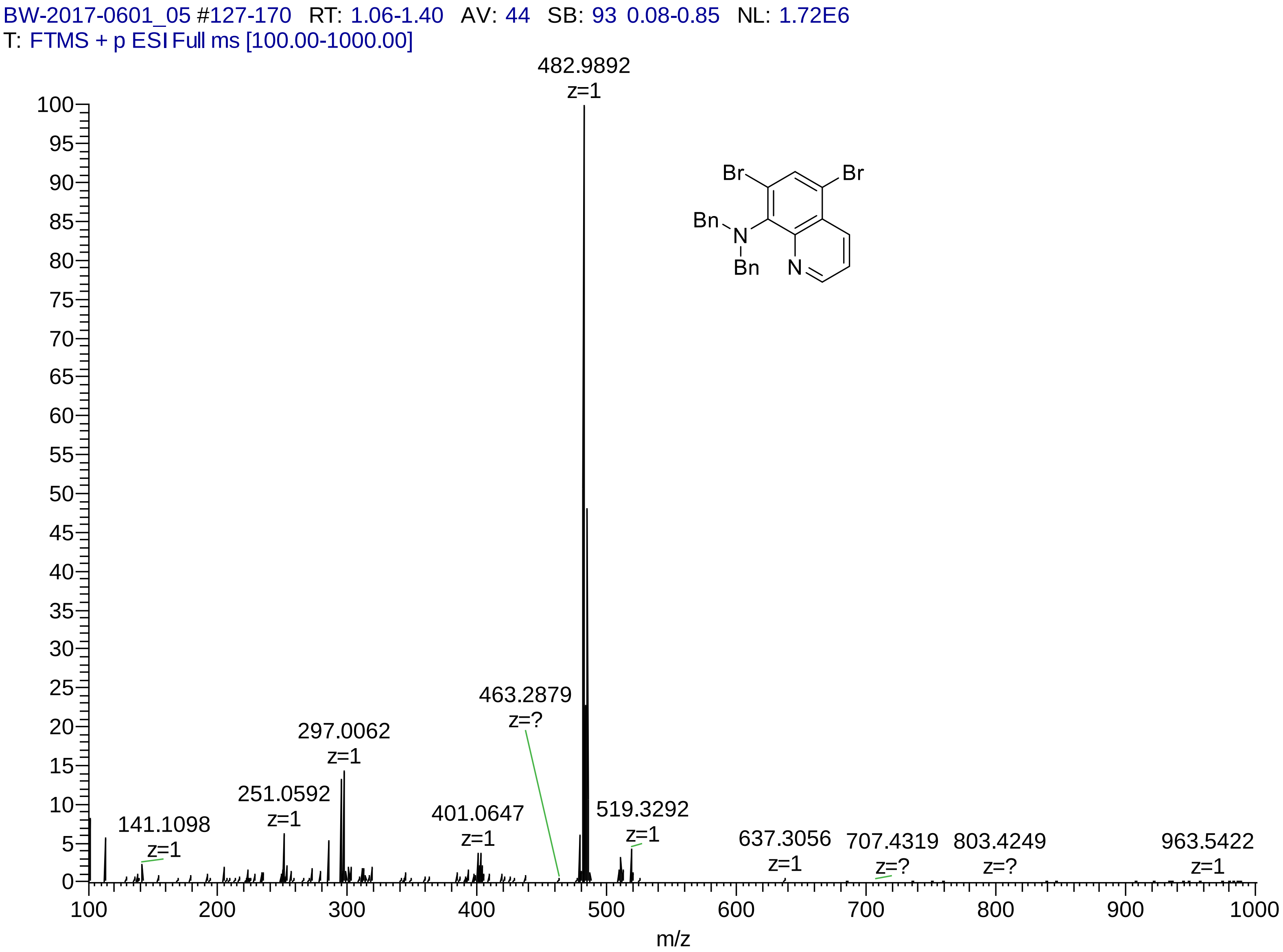

^{1}H NMR spectrum of compound **6f** ($CDCl_3$, 400 MHz)**:**

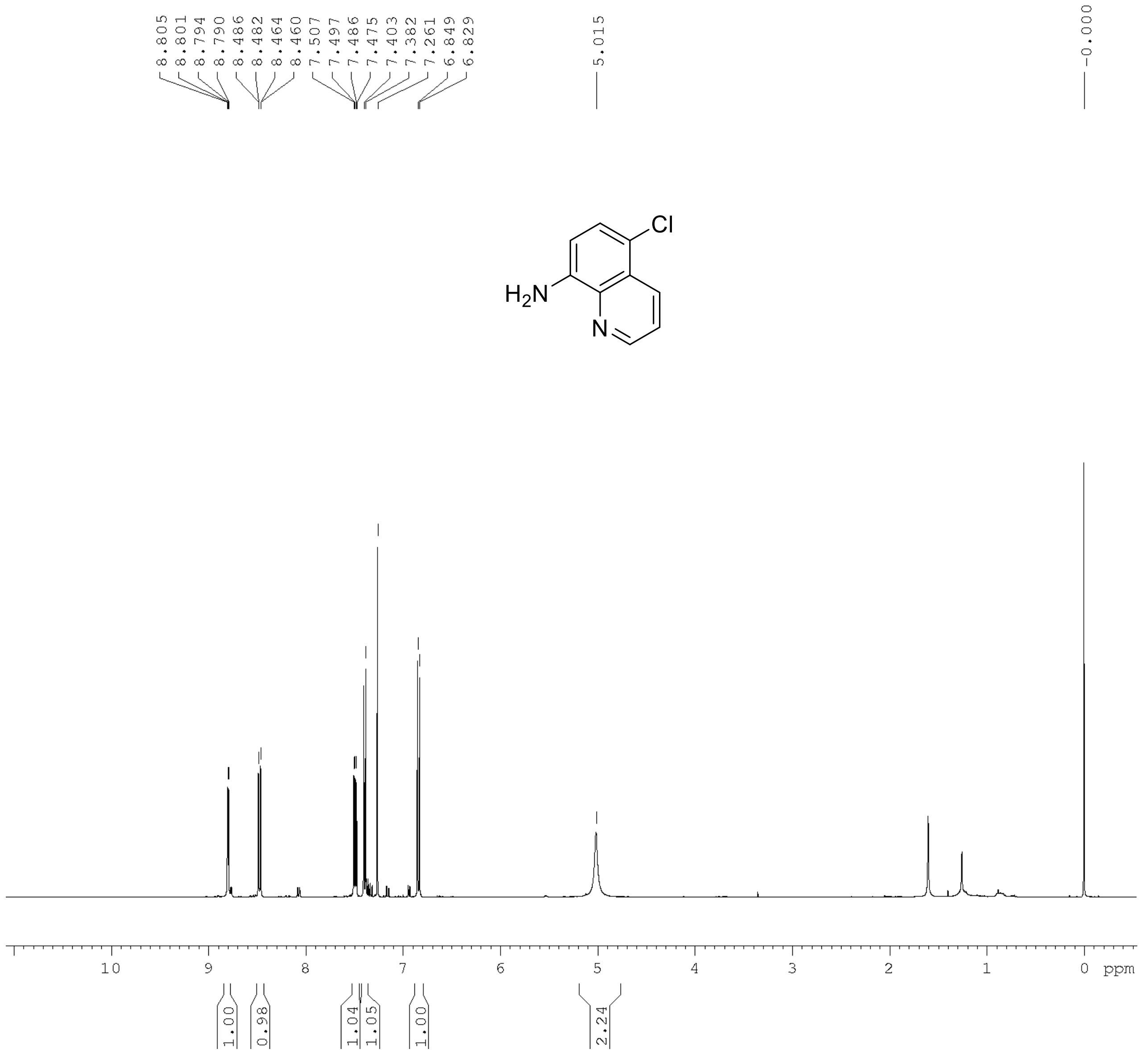

$^{13}C\{^1H\}$NMR spectrum of compound **6f** ($CDCl_3$, 100 MHz)

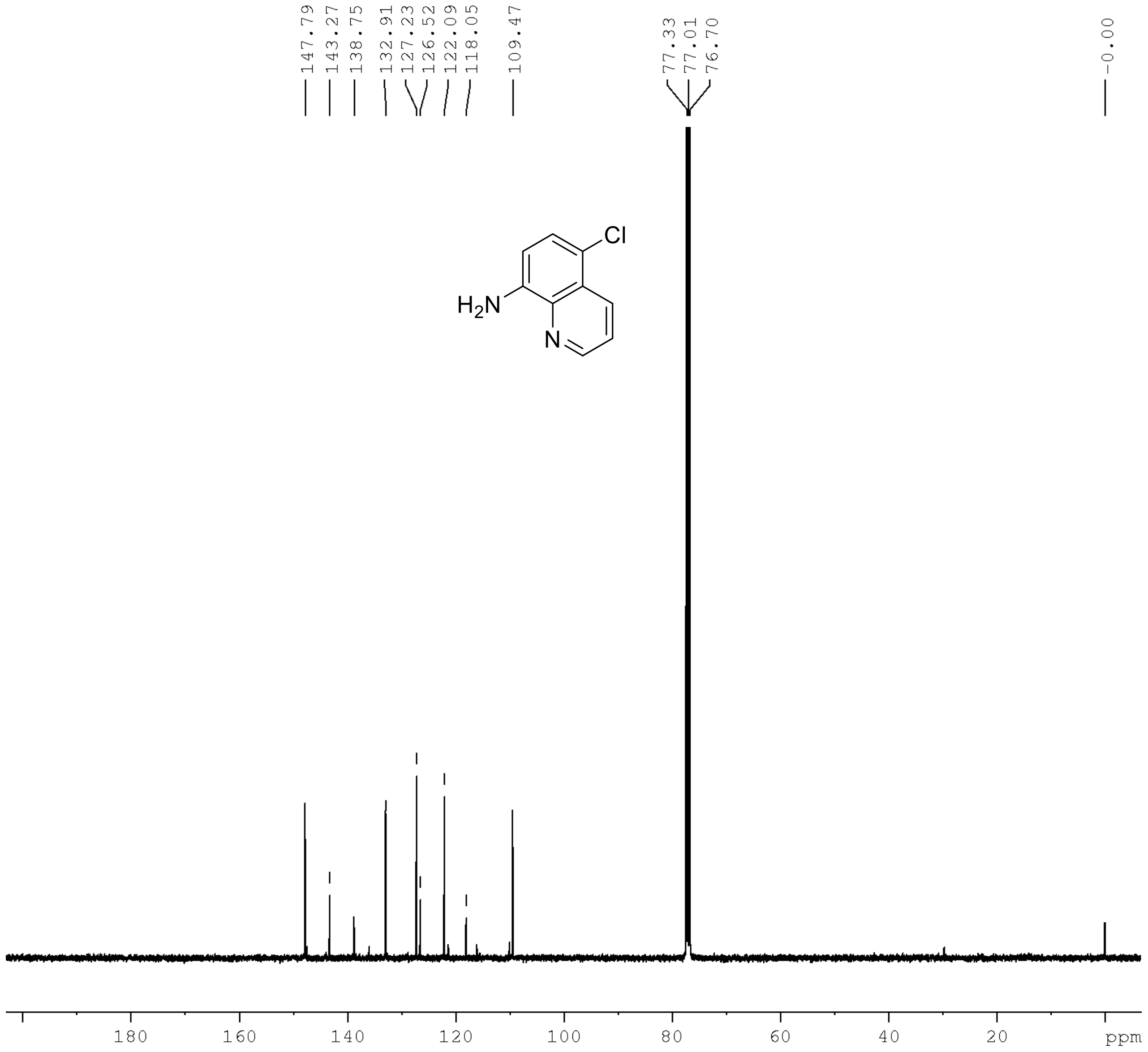

DEPT135 spectrum of compound **6f** ($CDCl_3$, 100 MHz)**:**

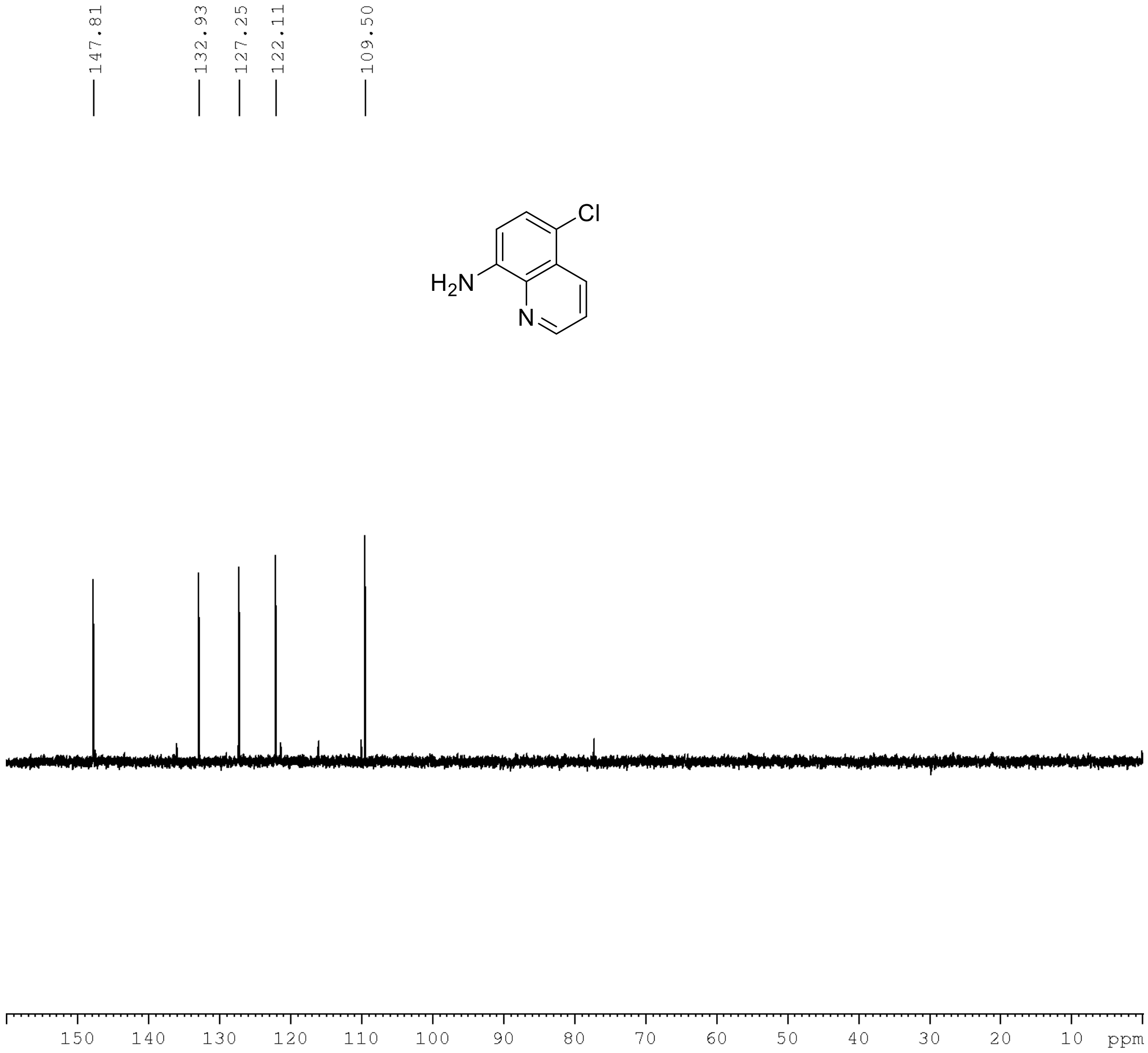

^{1}H NMR spectrum of compound **7f** ($CDCl_3$, 400 MHz)**:**

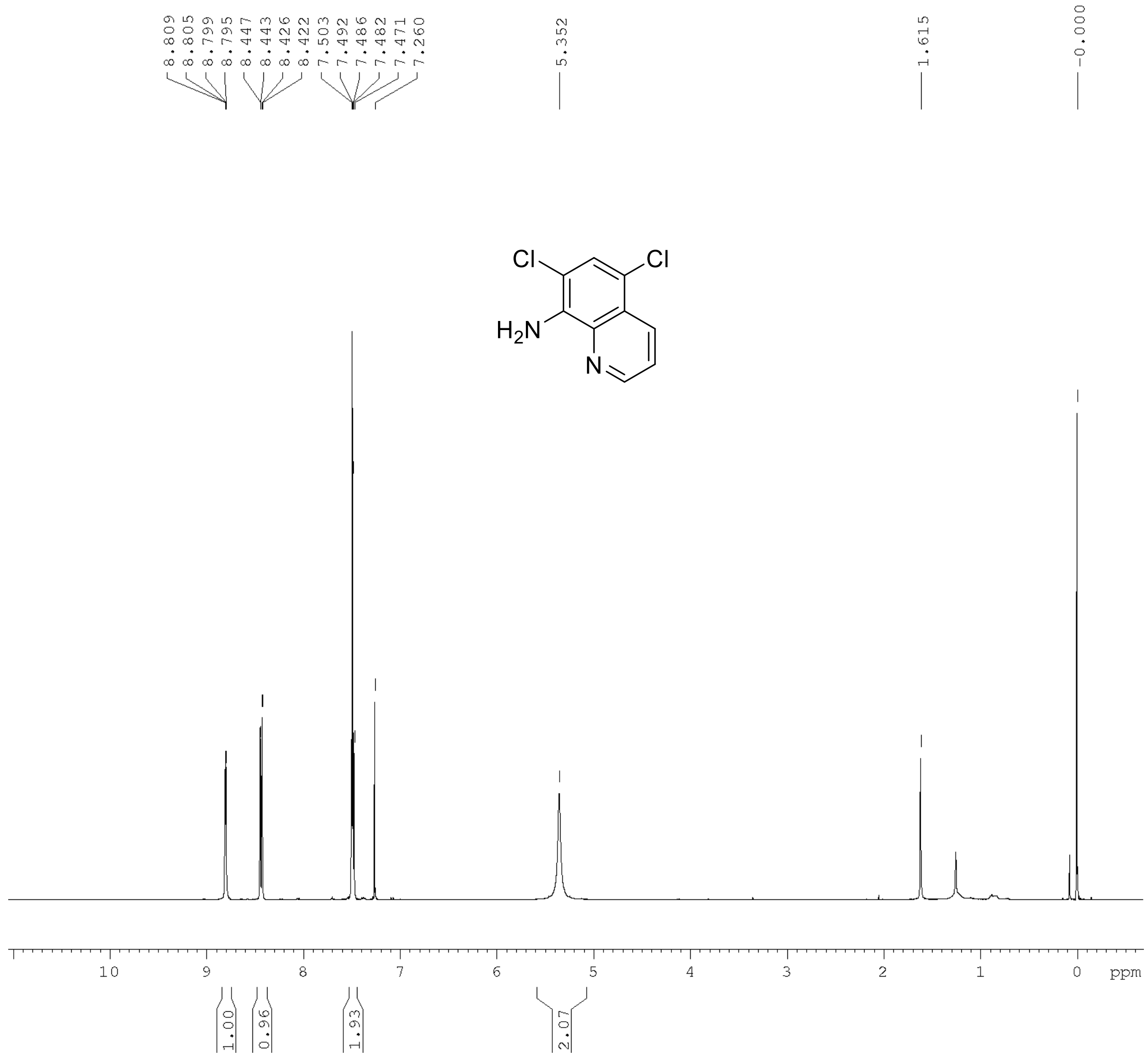

$^{13}C\{^{1}H\}$NMR spectrum of compound **7f** ($CDCl_3$, 100 MHz)**:**

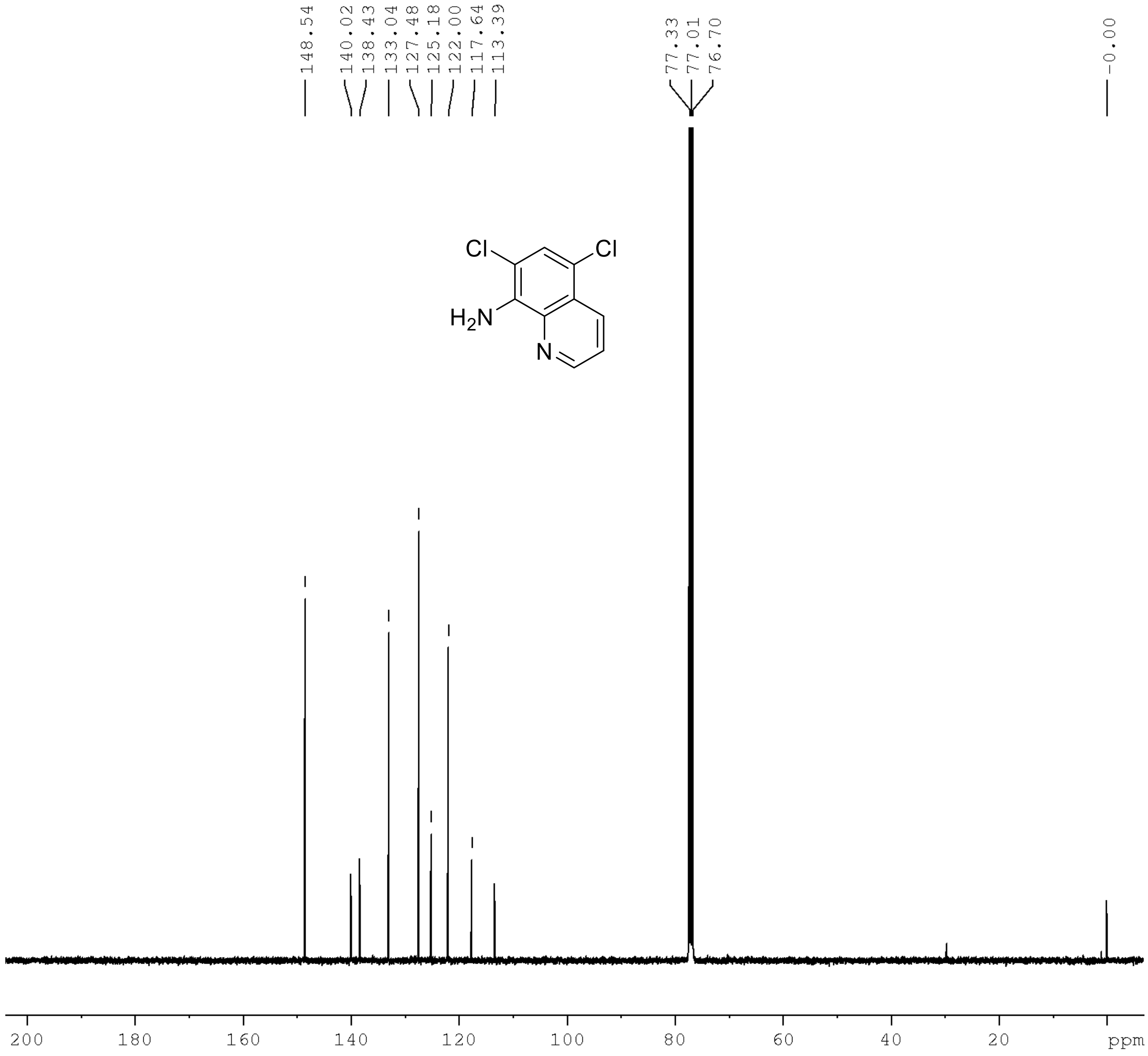

DEPT135 spectrum of compound **7f** ($CDCl_3$, 100 MHz)**:**

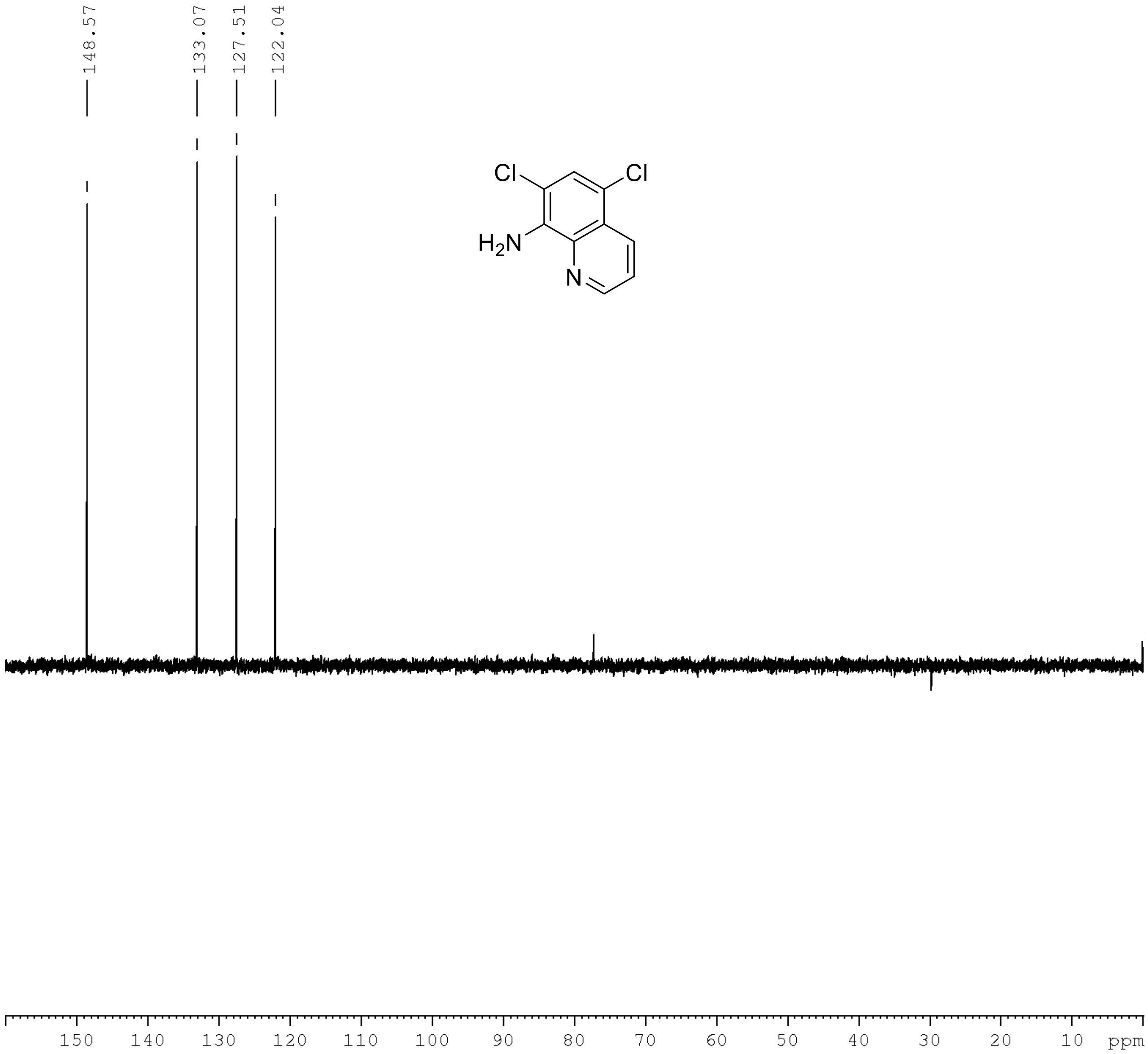

^{1}H NMR spectrum of compound **8f** ($CDCl_3$, 400 MHz)**:**

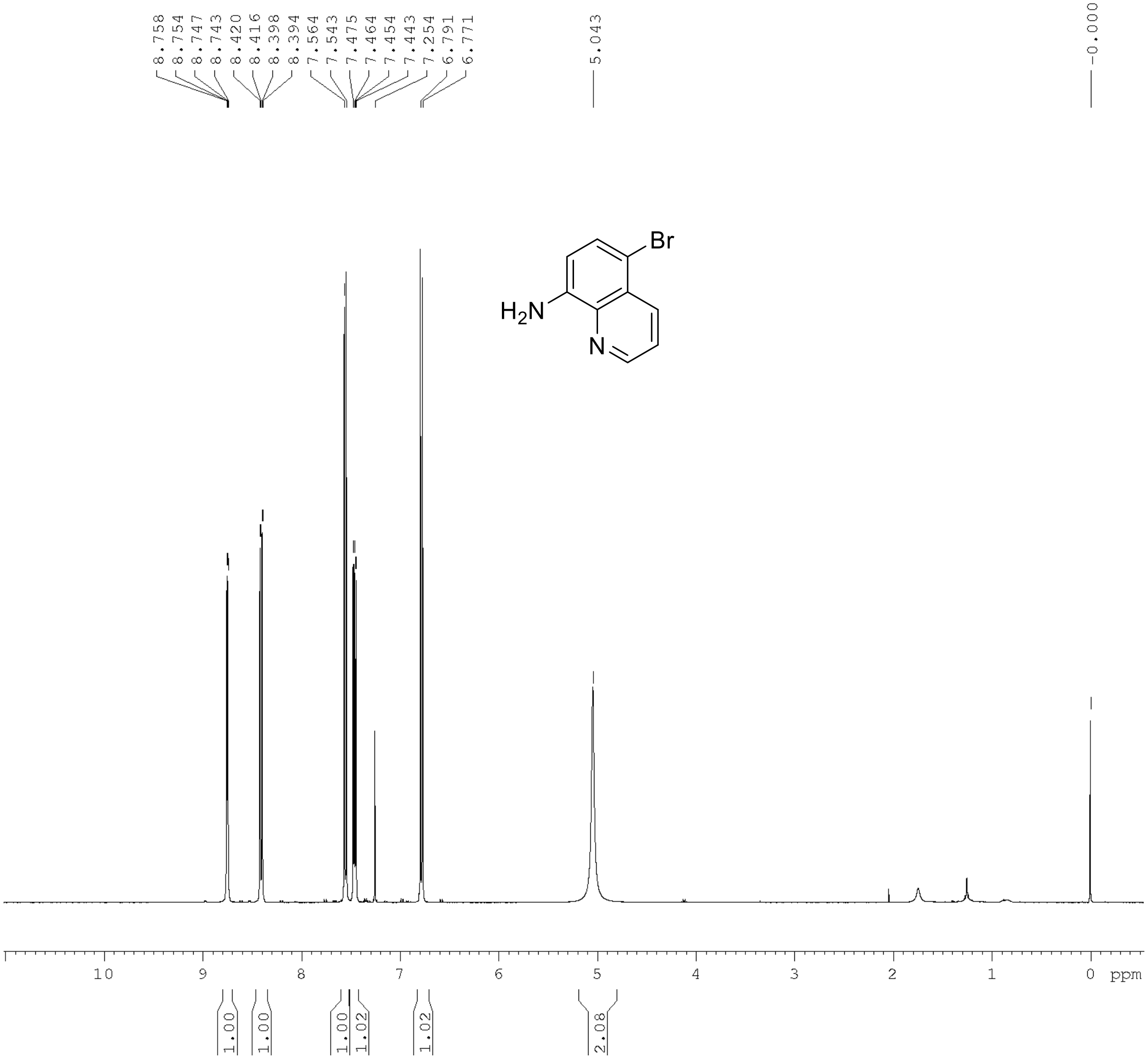

$^{13}C\{^{1}H\}$NMR spectrum of compound **8f** ($CDCl_3$, 100 MHz)**:**

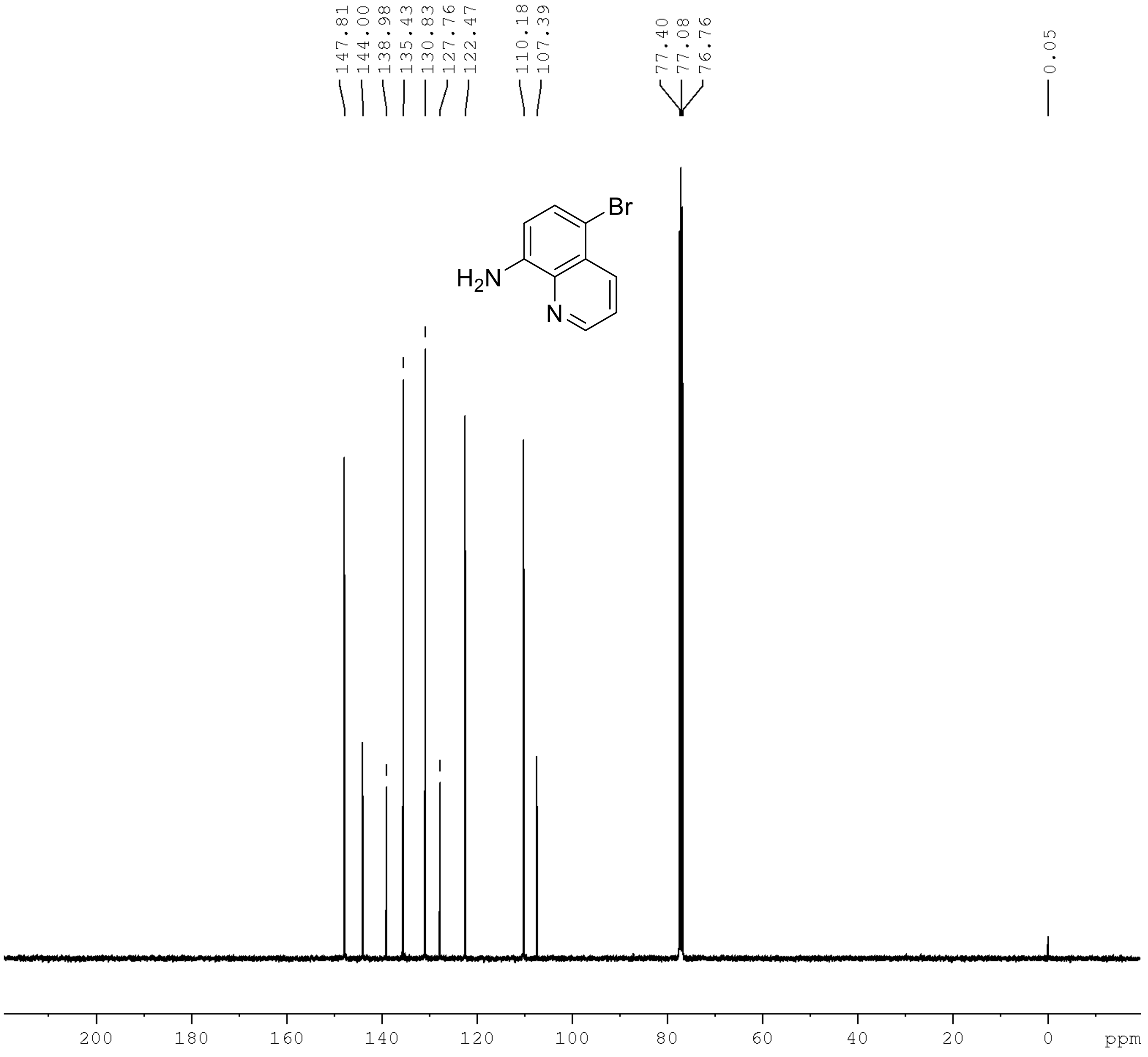

DEPT135 spectrum of compound **8f** ($CDCl_3$, 100 MHz)**:**

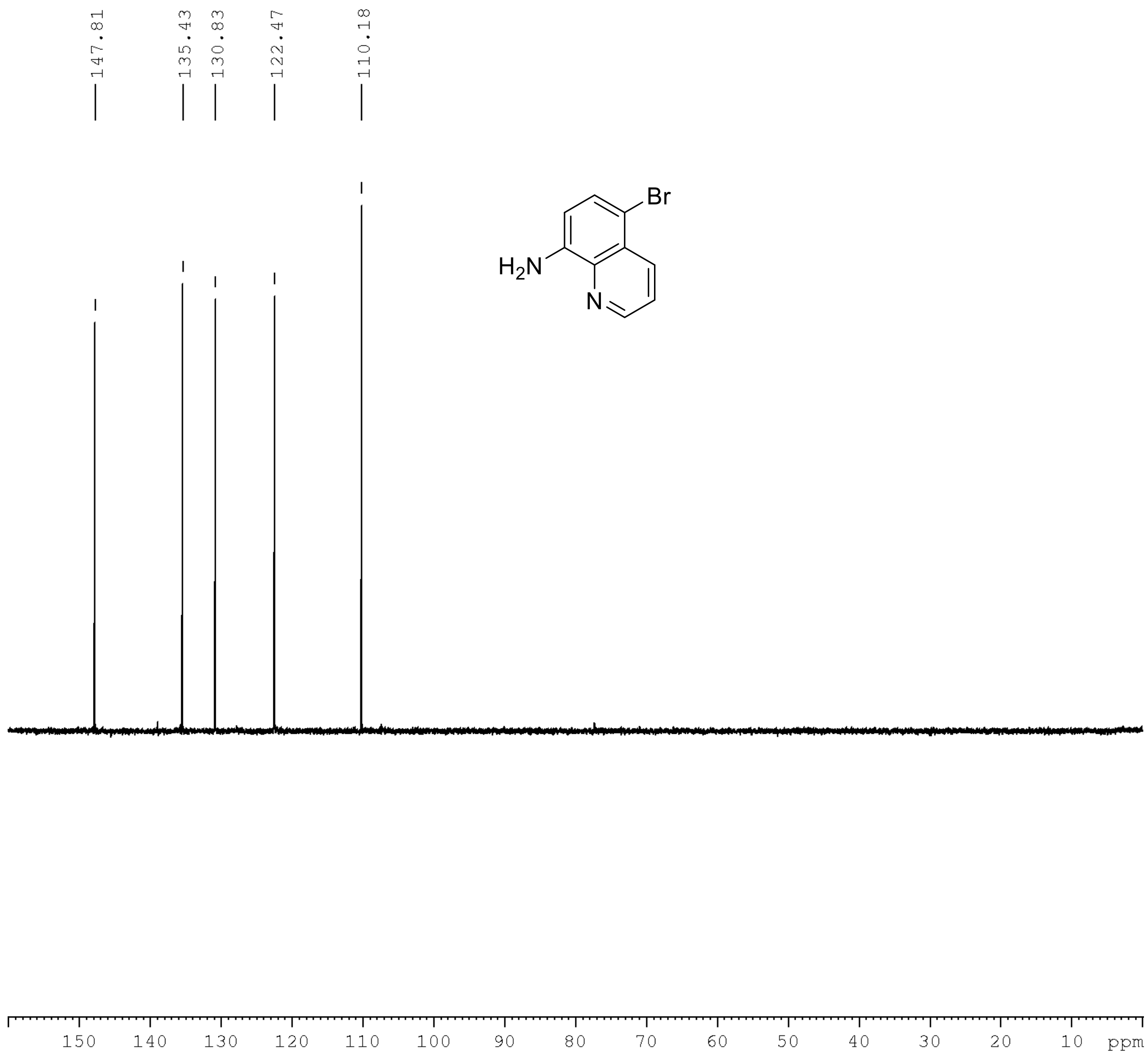

^{1}H NMR spectrum of compound **9f** ($CDCl_3$, 400 MHz)**:**

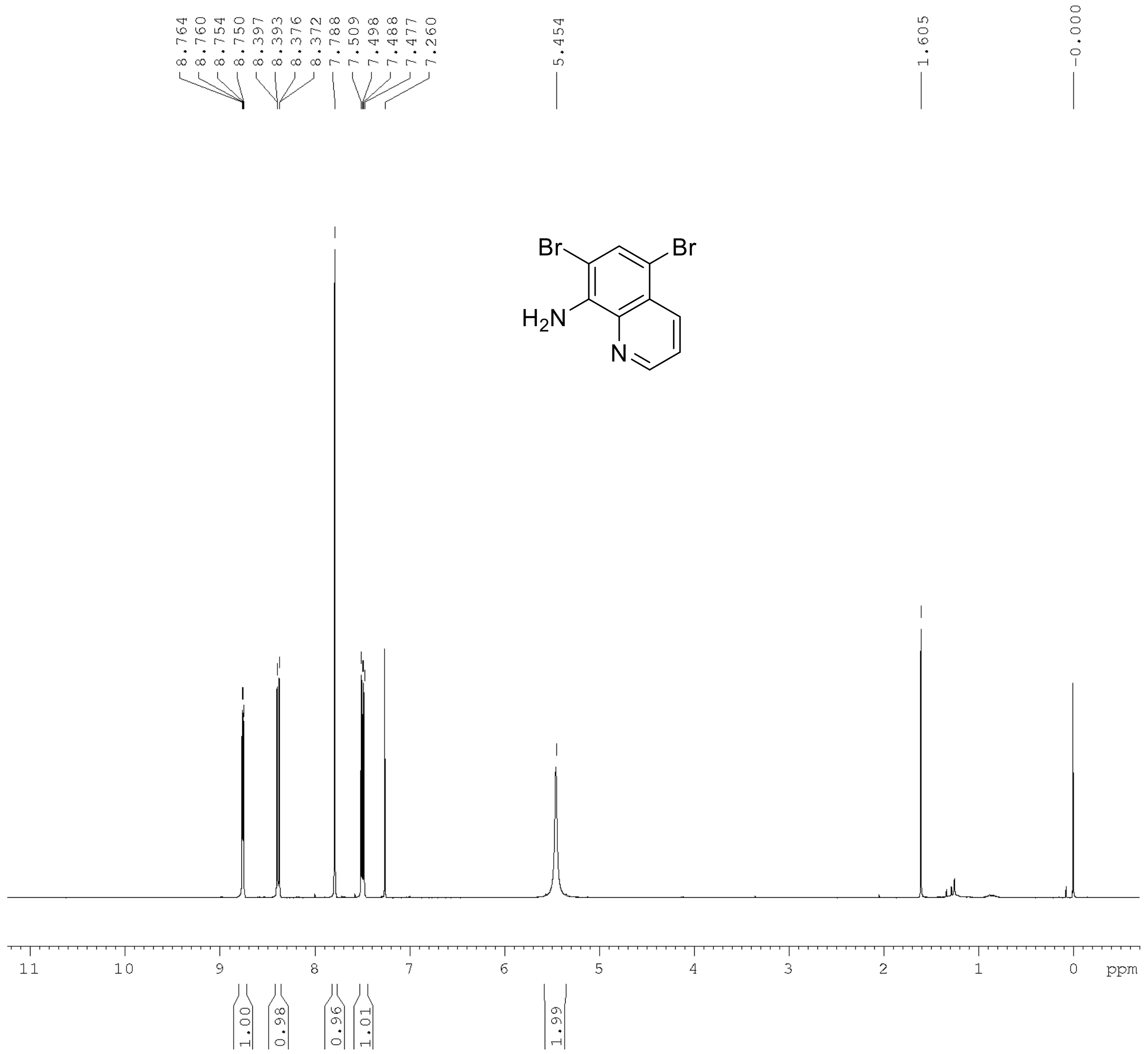

$^{13}C\{^{1}H\}$NMR spectrum of compound **9f** ($CDCl_3$, 100 MHz)**:**

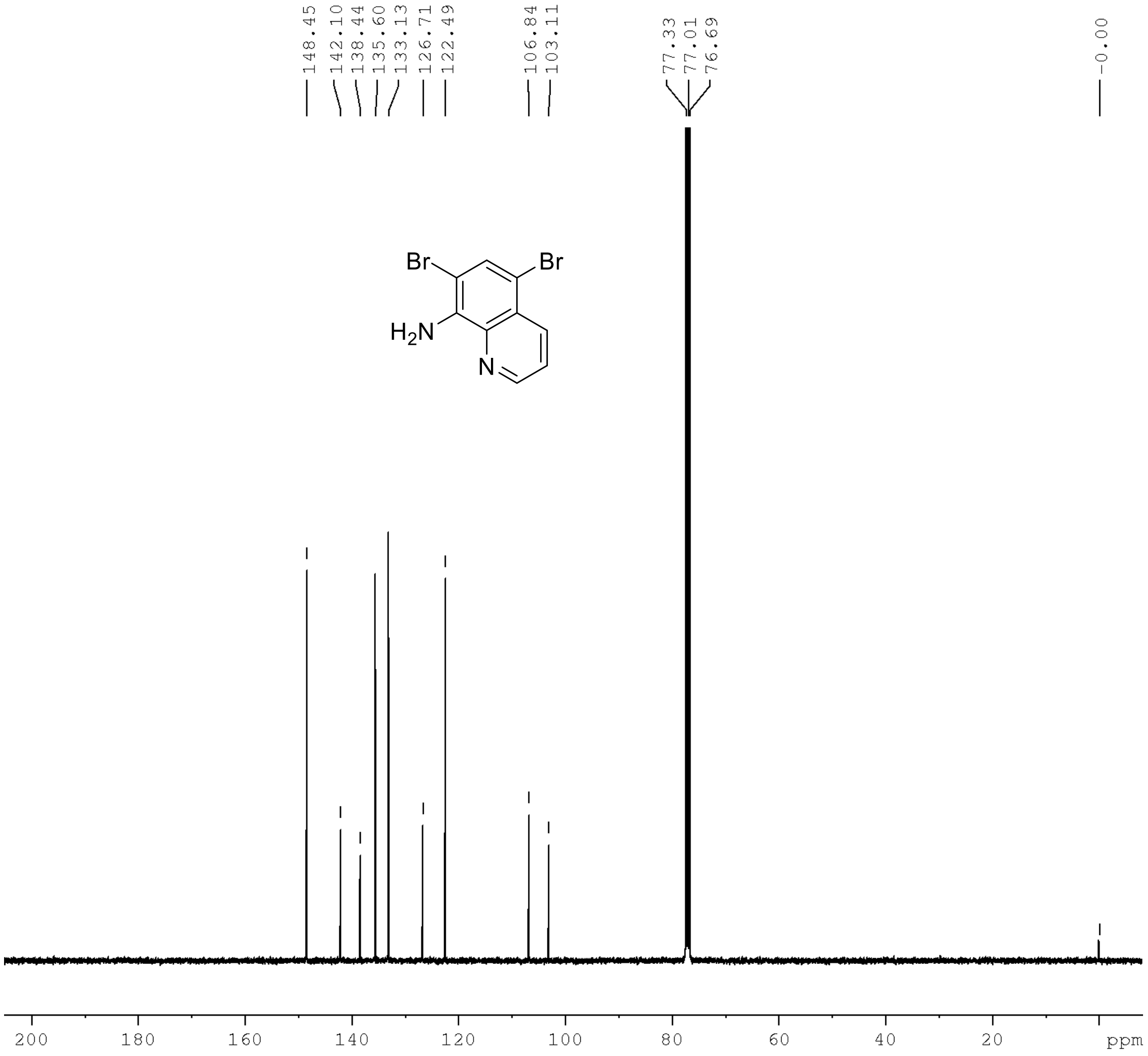

DEPT135 spectrum of compound **9f** ($CDCl_3$, 100 MHz)**:**

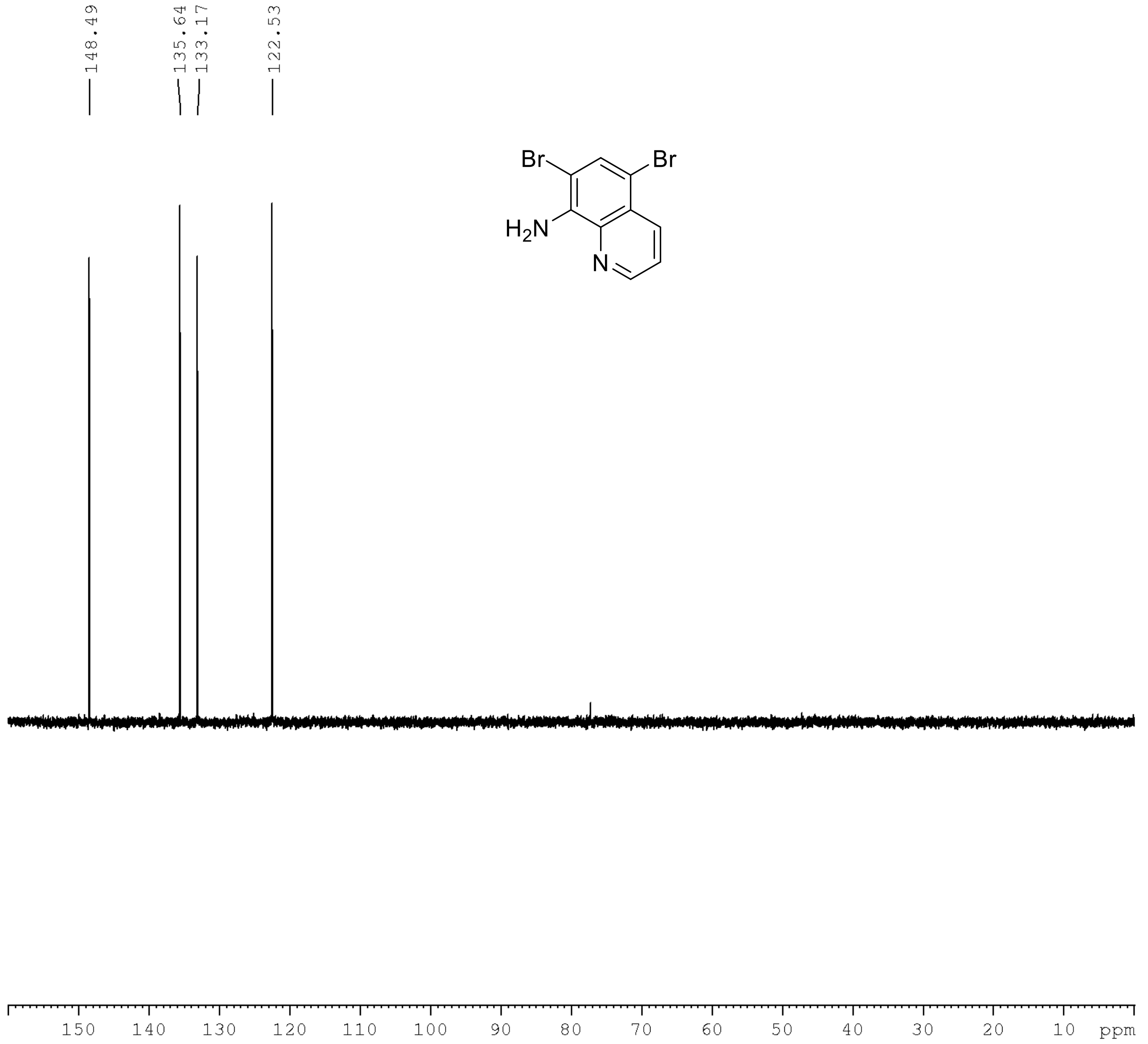

^{1}H NMR spectrum of compound **6g** ($CDCl_3$, 400 MHz)**:**

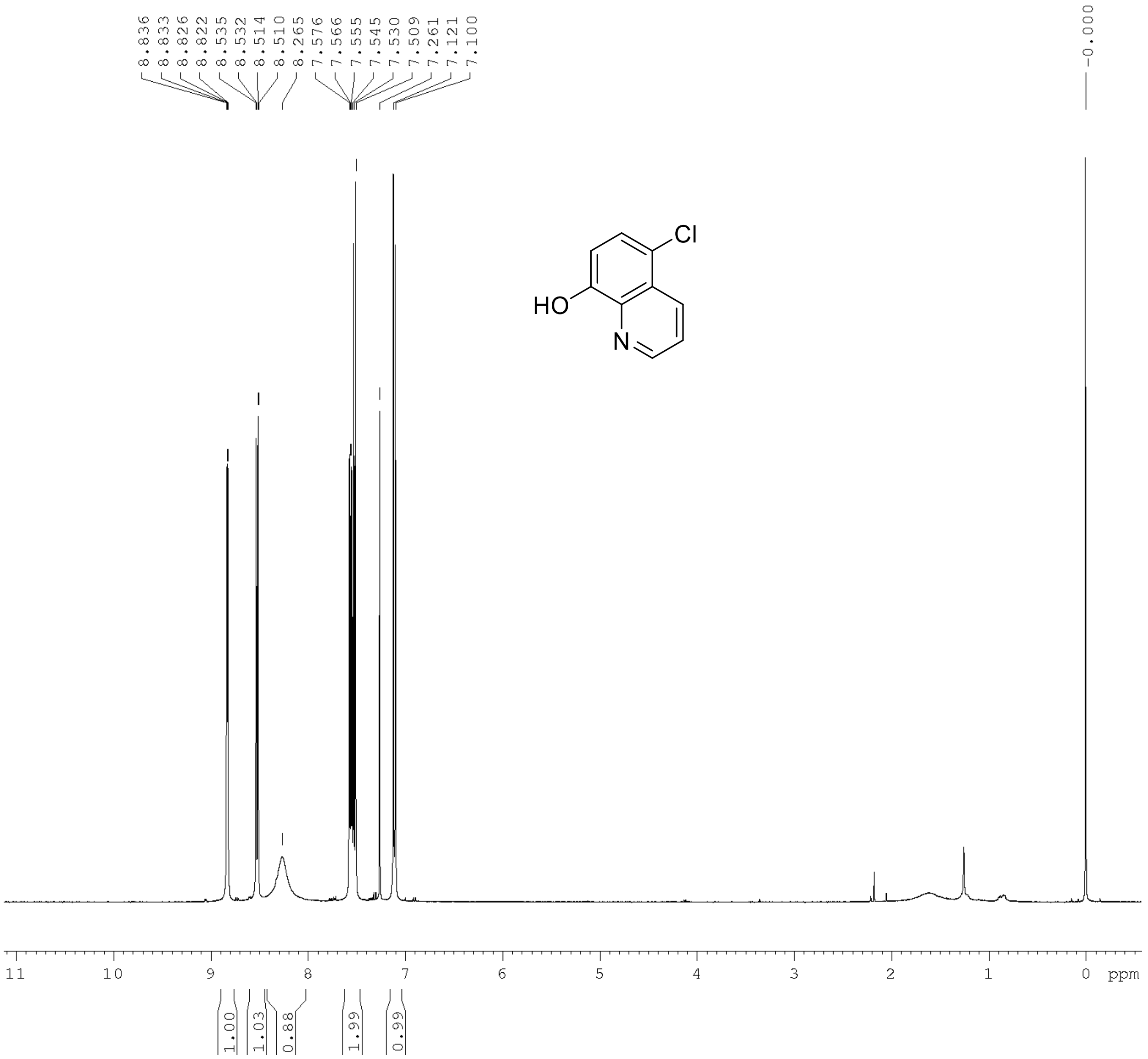

$^{13}C\{^{1}H\}$NMR spectrum of compound **6g** ($CDCl_3$, 100 MHz)**:**

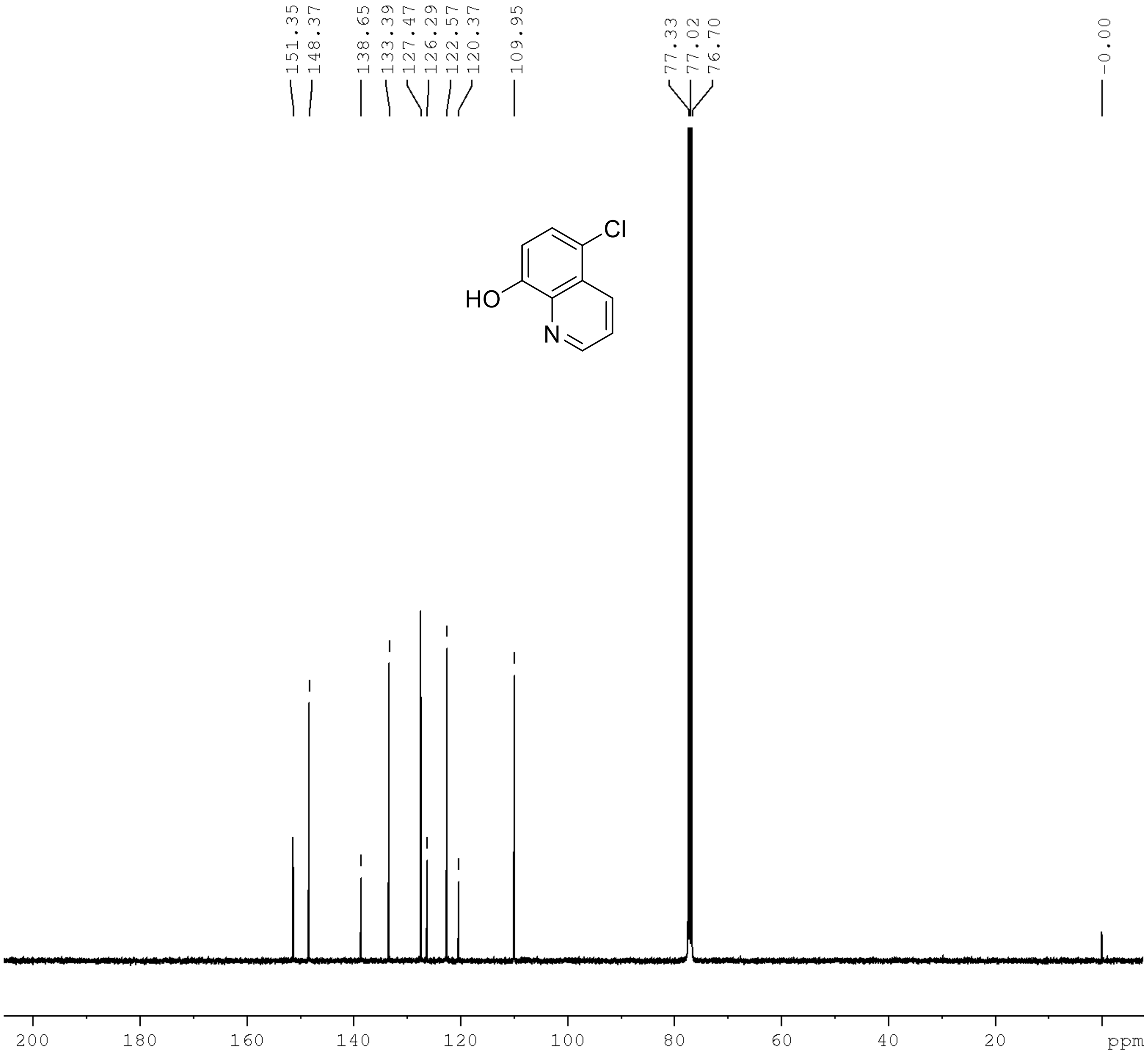

DEPT135 spectrum of compound **6g** ($CDCl_3$, 100 MHz)**:**

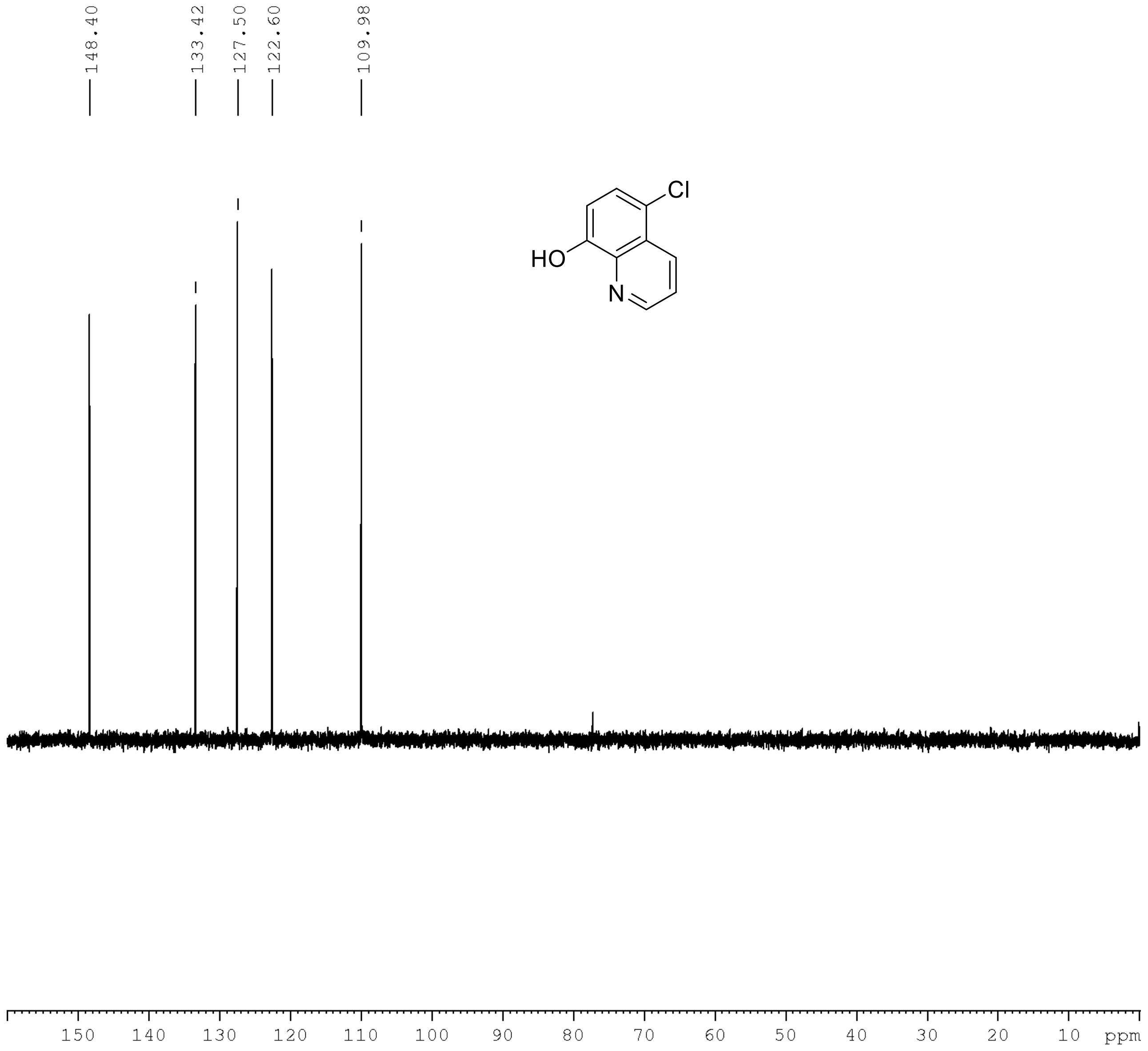

^{1}H NMR spectrum of compound **7g** ($CDCl_3$, 400 MHz)**:**

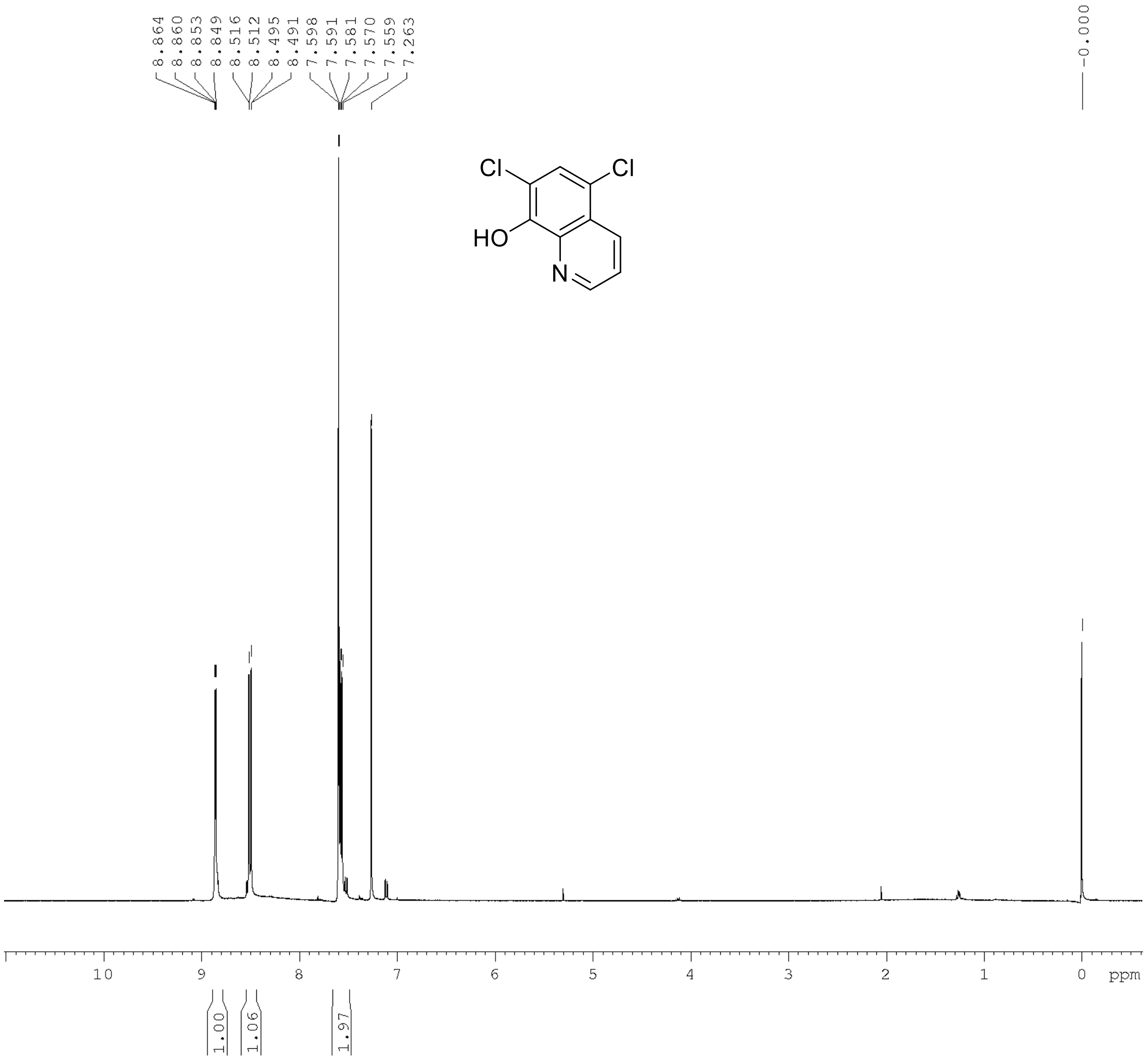

$^{13}C\{^{1}H\}$NMR spectrum of compound **7g** ($CDCl_3$, 100 MHz)**:**

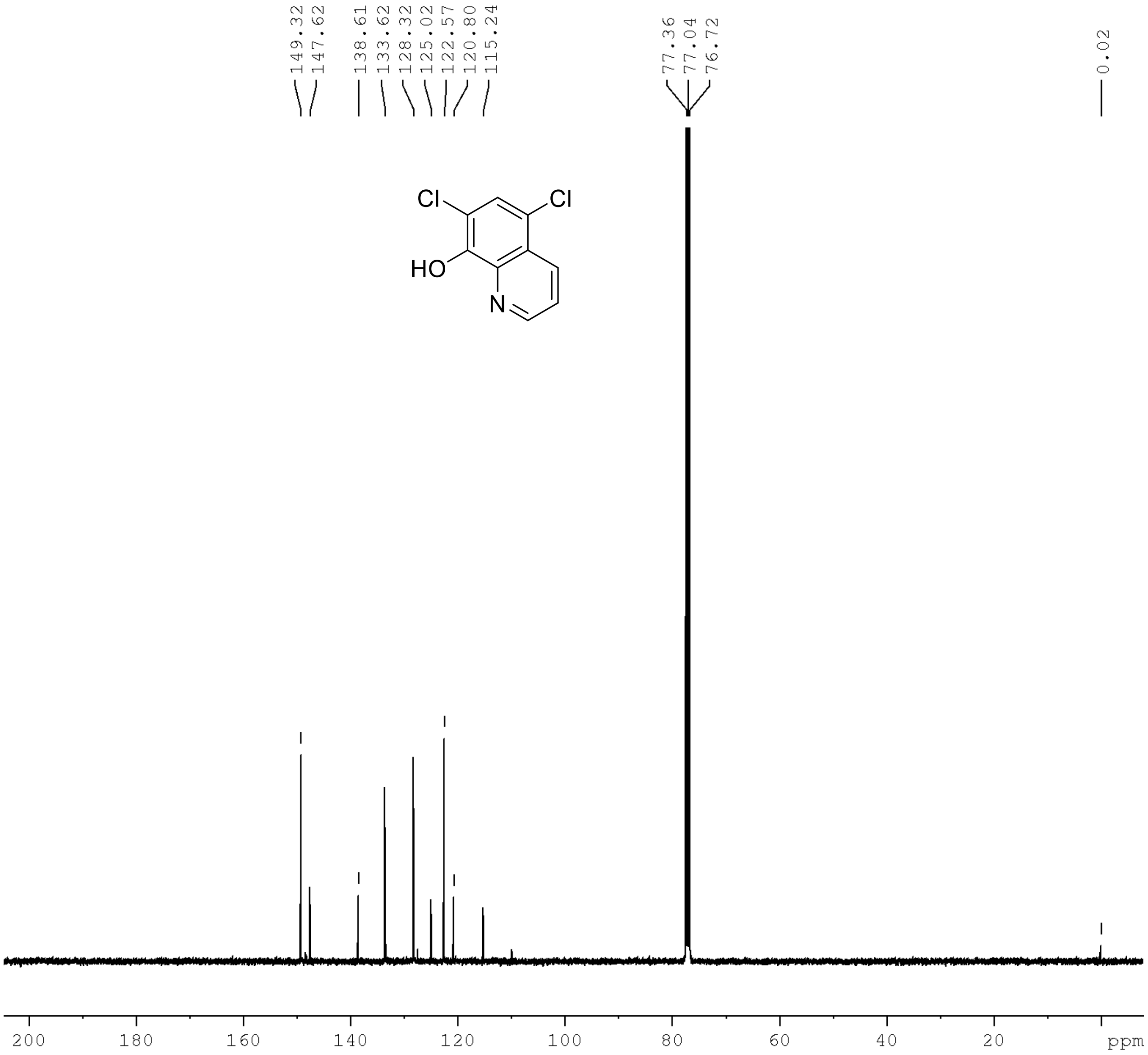

DEPT135 spectrum of compound **7g** ($CDCl_3$, 100 MHz)**:**

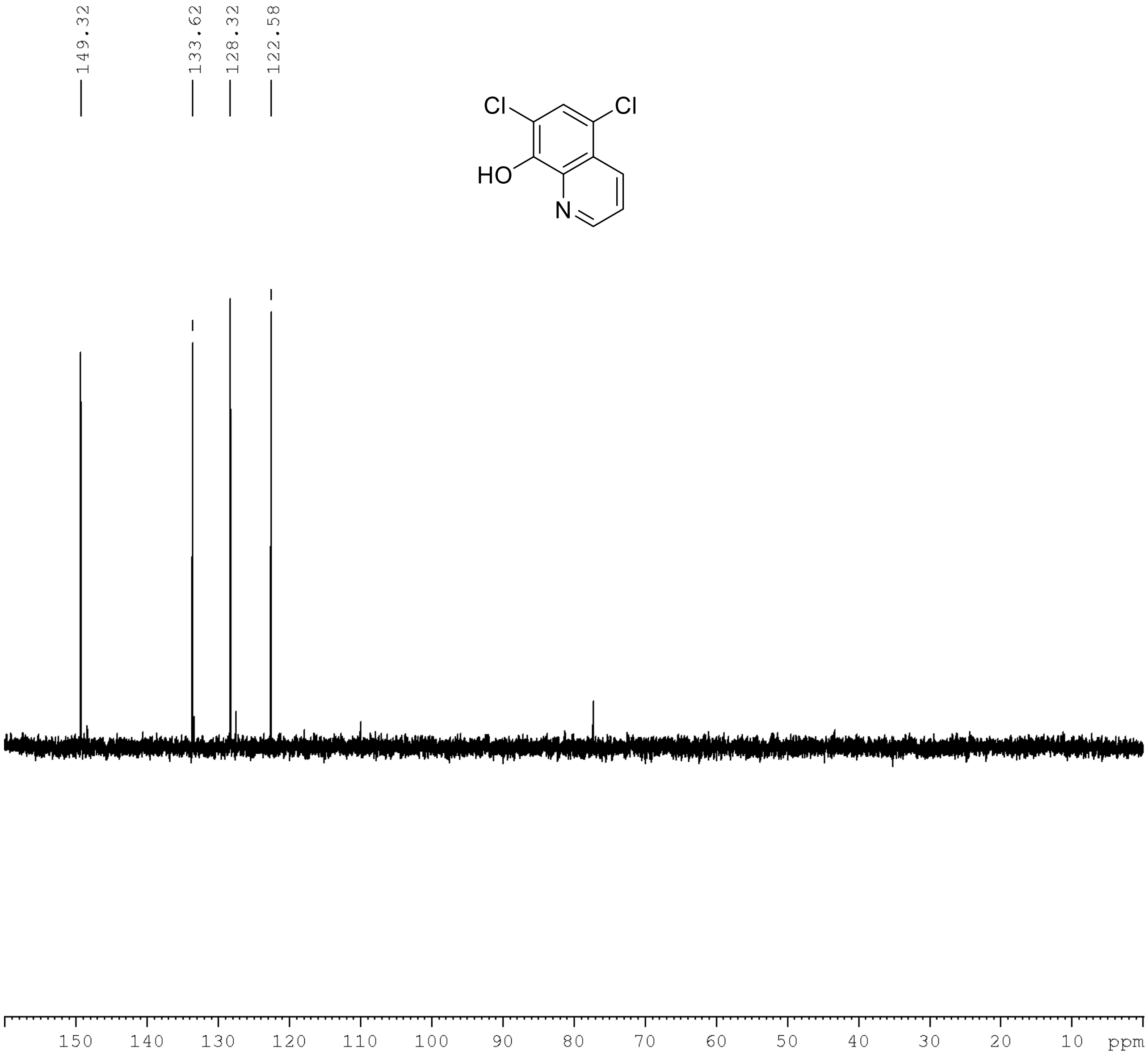

^{1}H NMR spectrum of compound **8g** ($CDCl_3$, 400 MHz)**:**

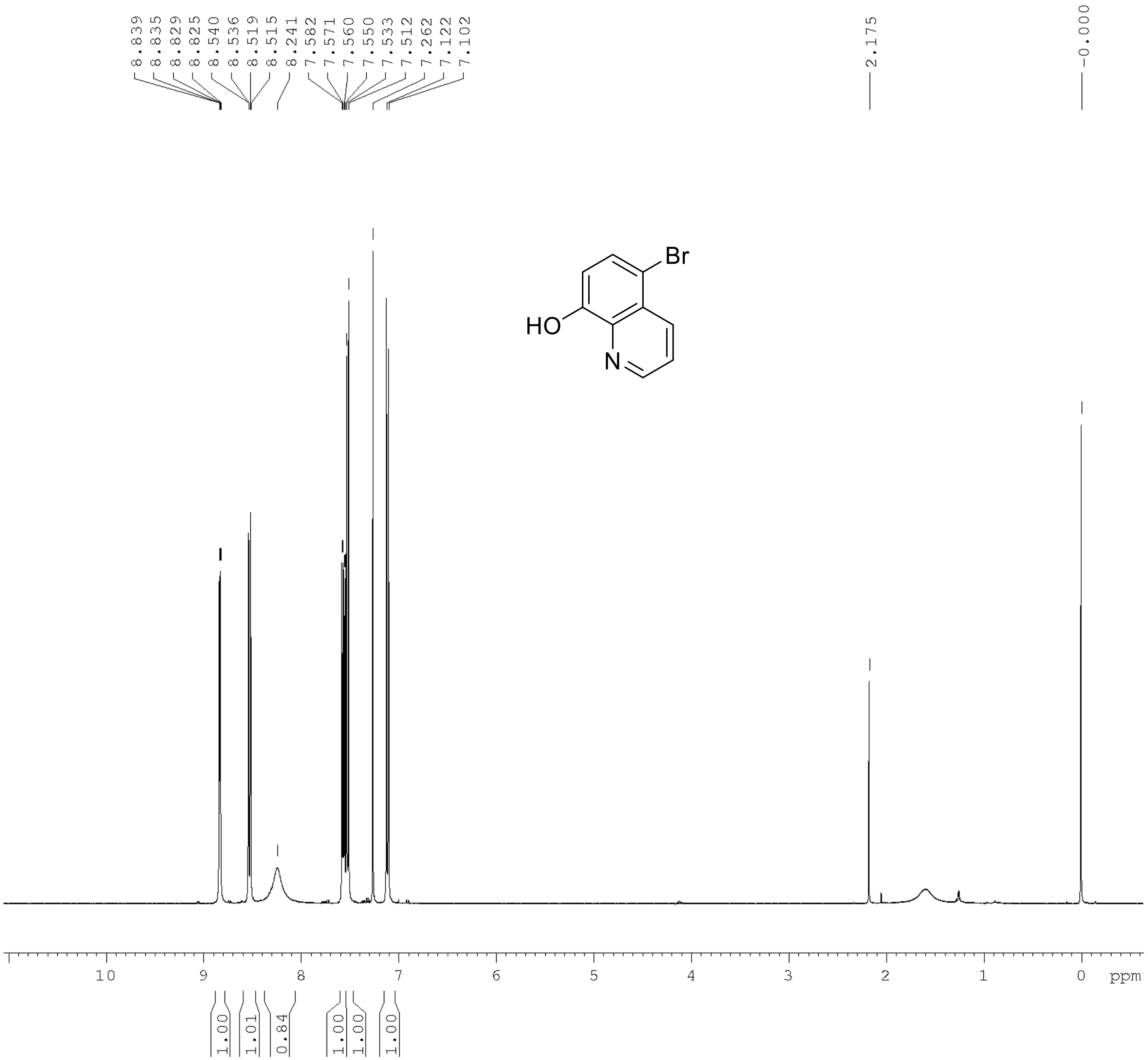

$^{13}C\{^{1}H\}$NMR spectrum of compound **8g** ($CDCl_3$, 100 MHz)**:**

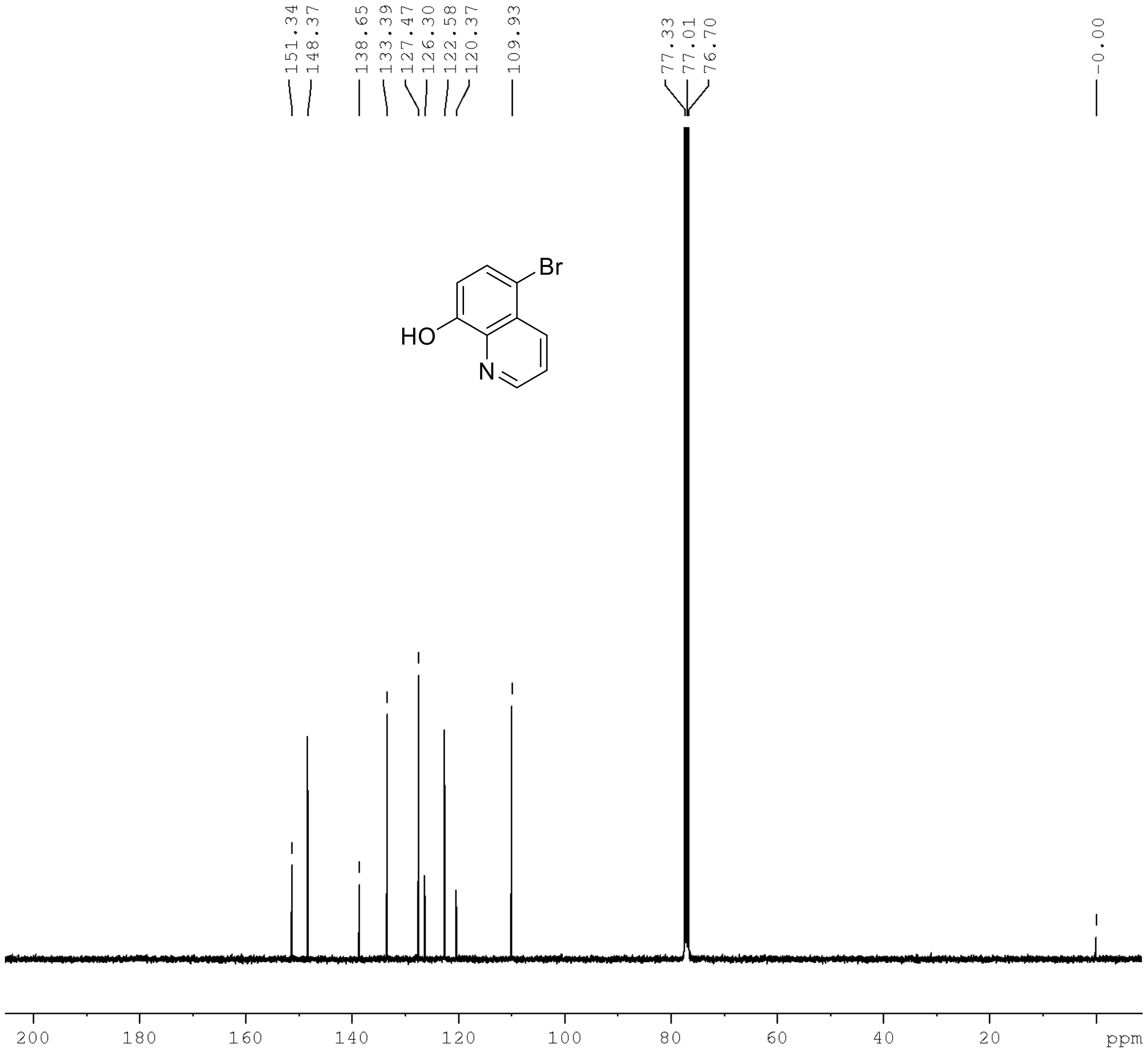

DEPT135 spectrum of compound **8g** ($CDCl_3$, 100 MHz)**:**

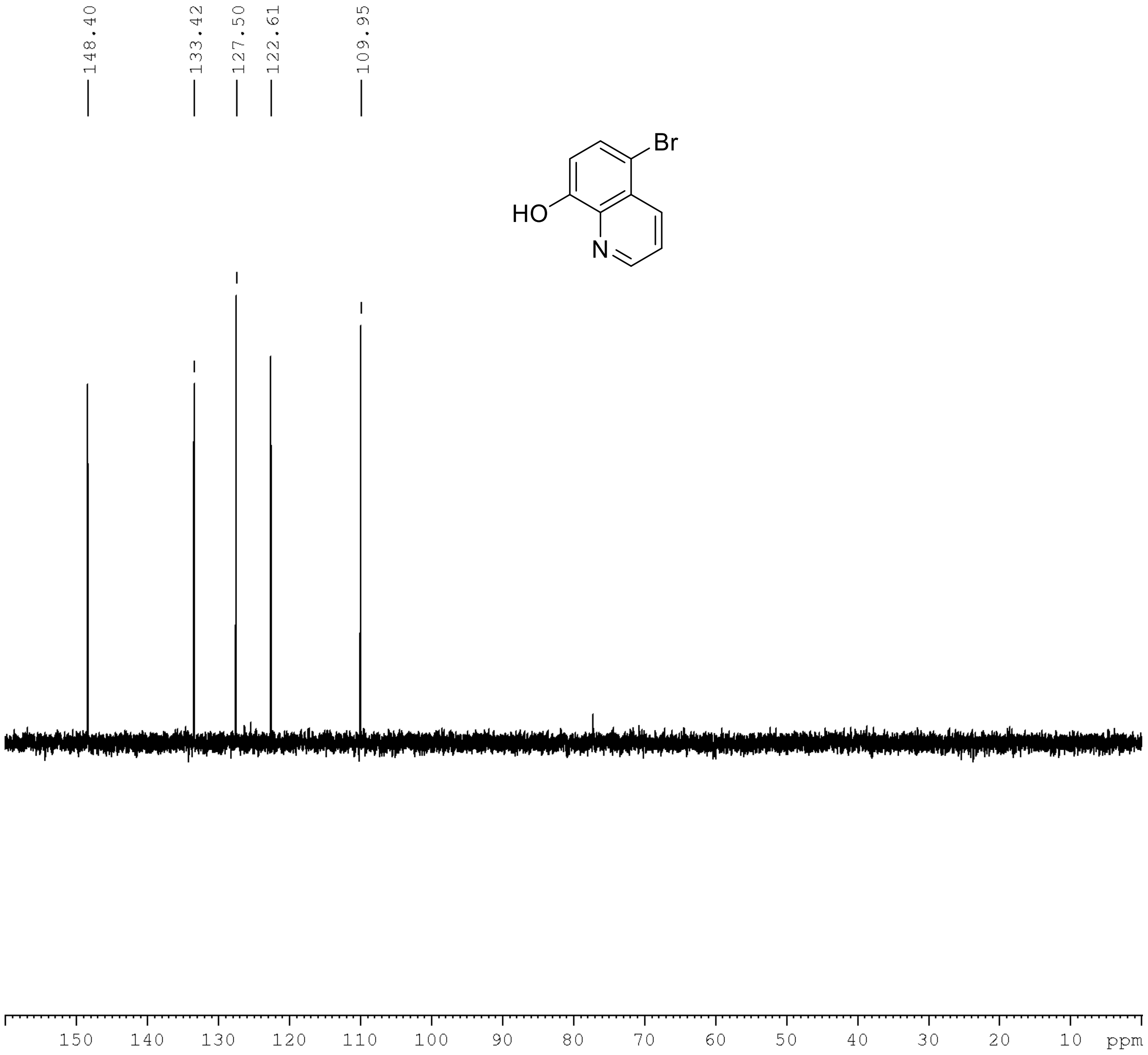

^{1}H NMR spectrum of compound **9g** ($CDCl_3$, 400 MHz)**:**

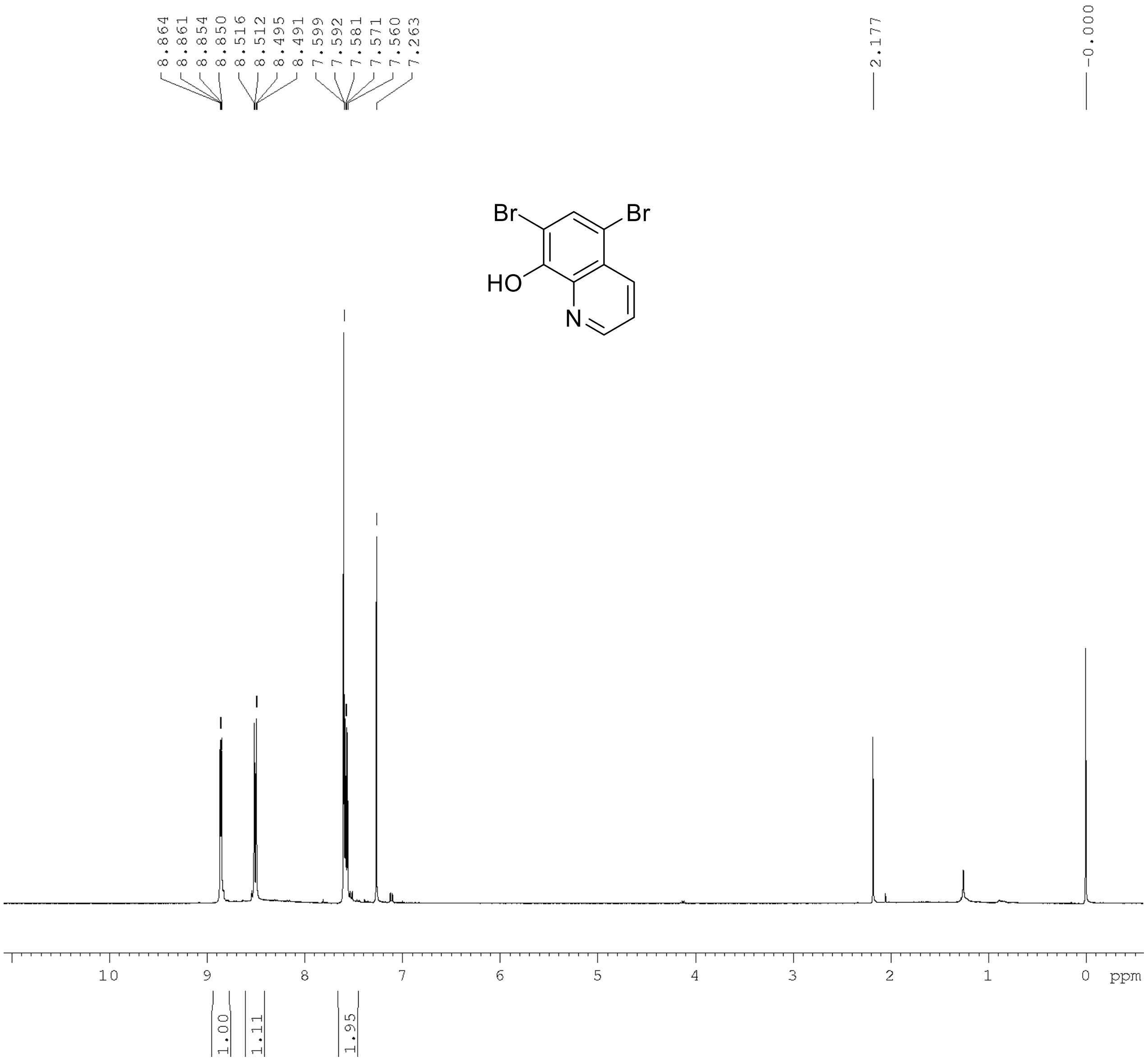

$^{13}C\{^1H\}$NMR spectrum of compound **9g** ($CDCl_3$, 100 MHz)**:**

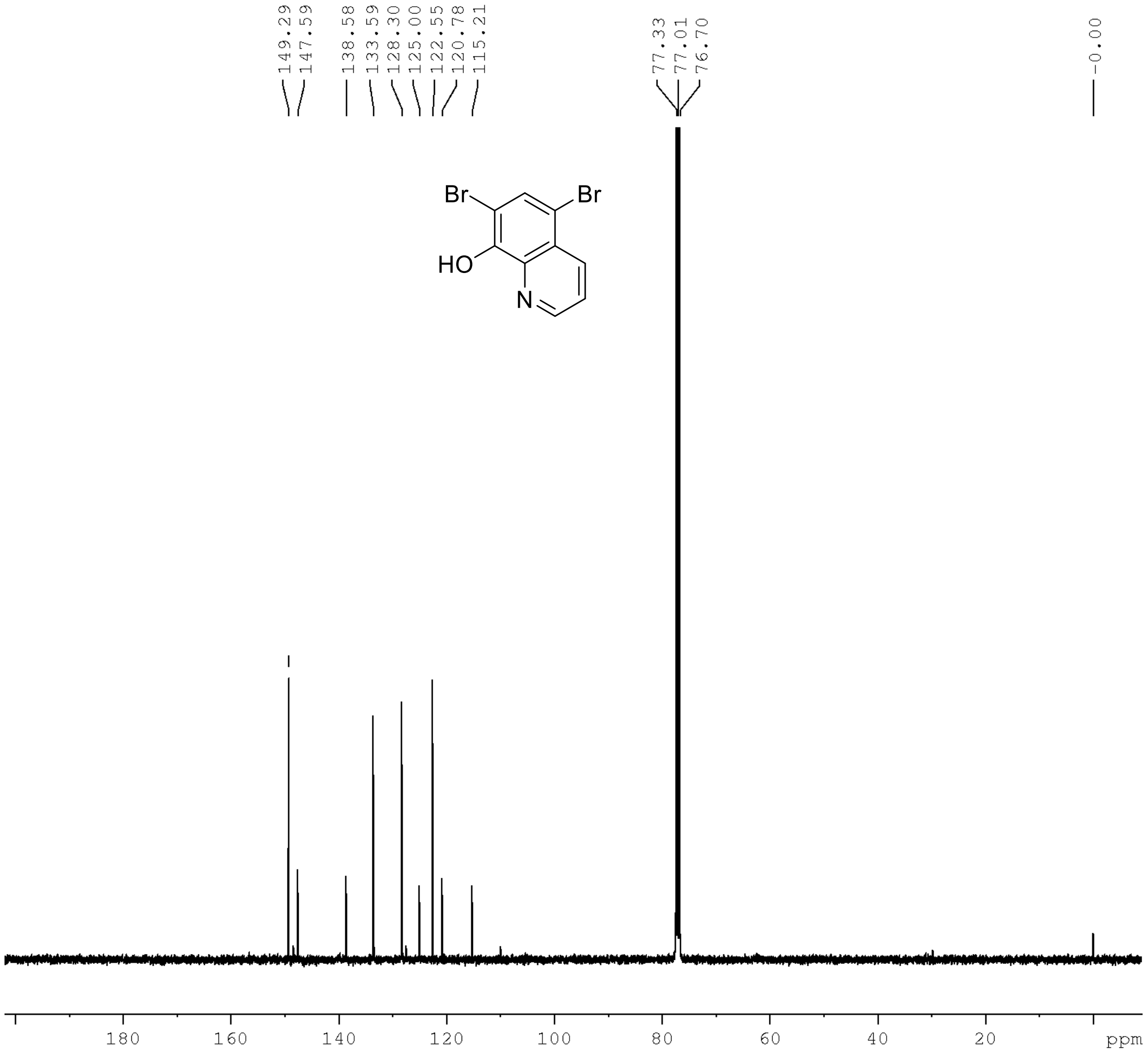

DEPT135 spectrum of compound **9g** ($CDCl_3$, 100 MHz)**:**

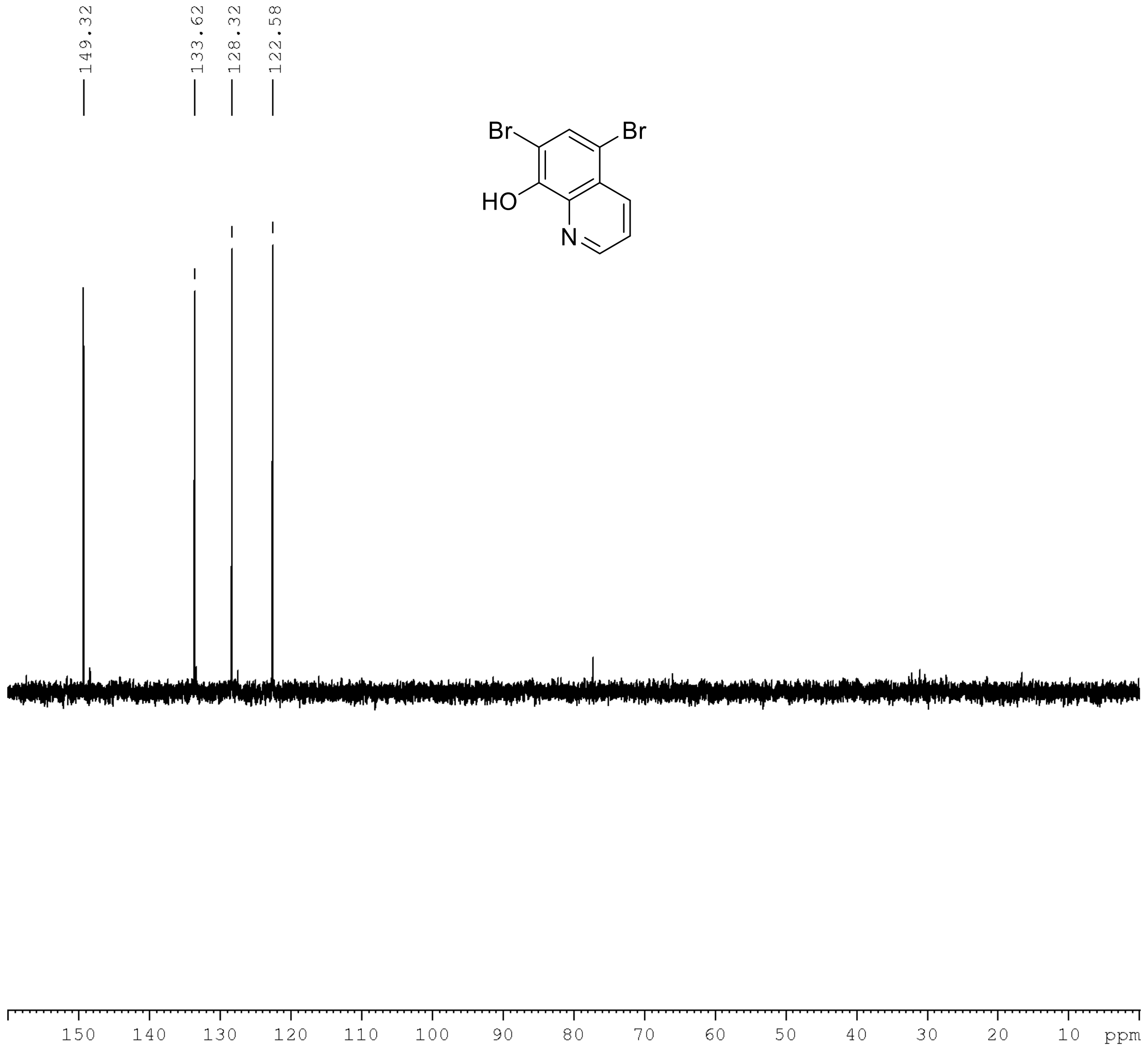

^{1}H NMR spectrum of compound **6h** ($CDCl_3$, 400 MHz)**:**

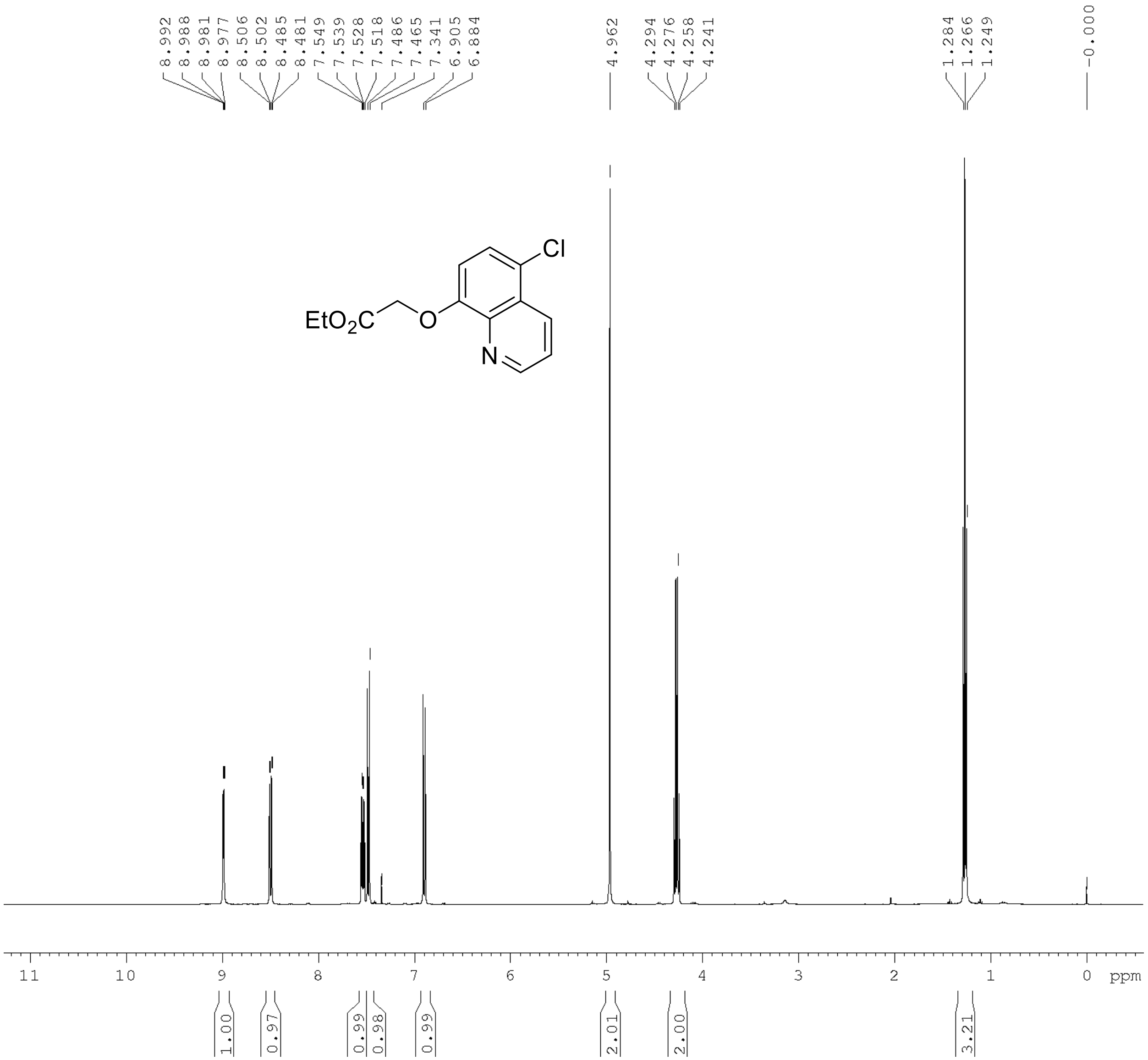

$^{13}C\{^{1}H\}$NMR spectrum of compound **6h** ($CDCl_3$, 100 MHz)**:**

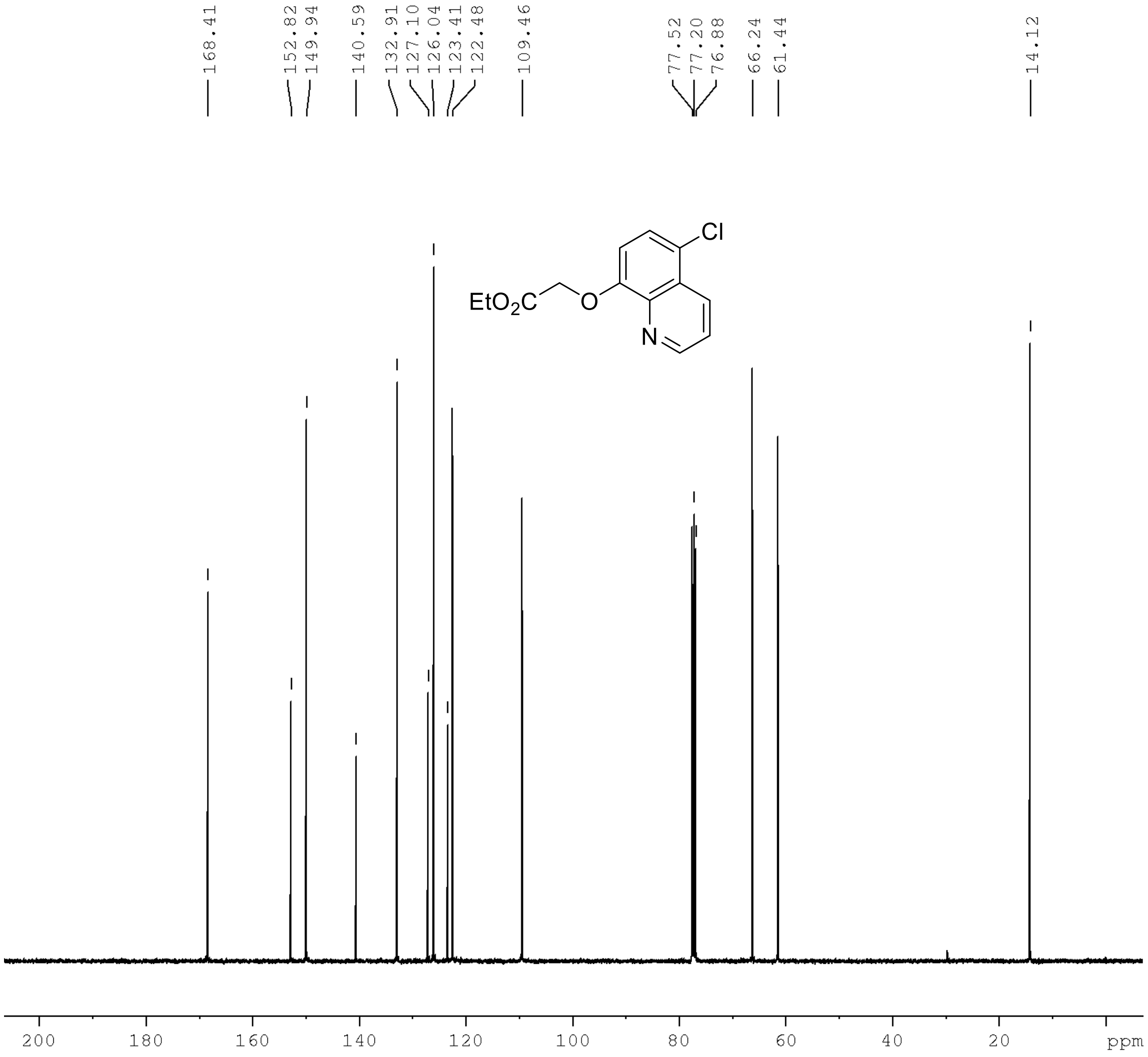

DEPT135 spectrum of compound **6h** ($CDCl_3$, 100 MHz)**:**

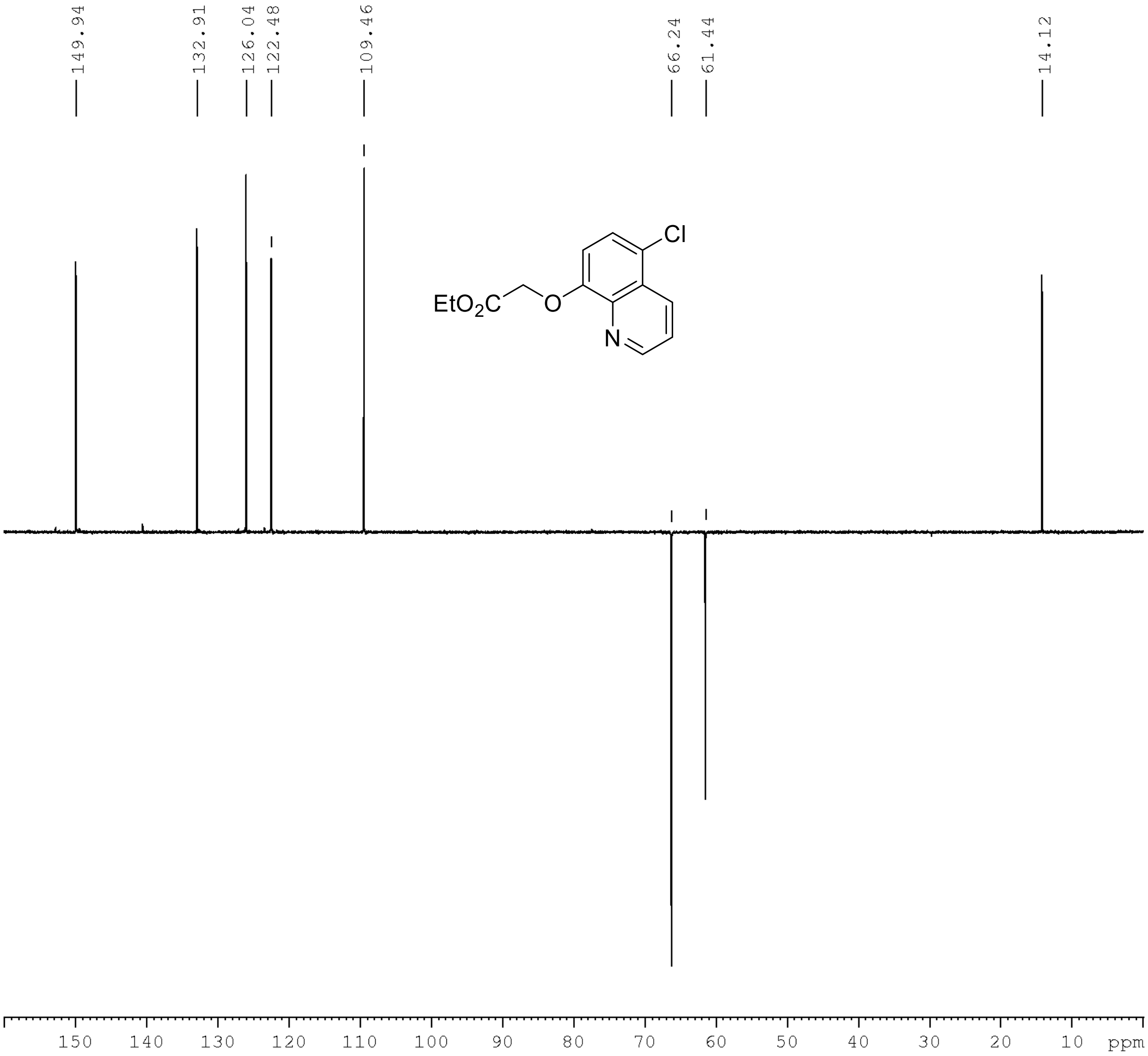

HRMS spectrum of compound **6h:**

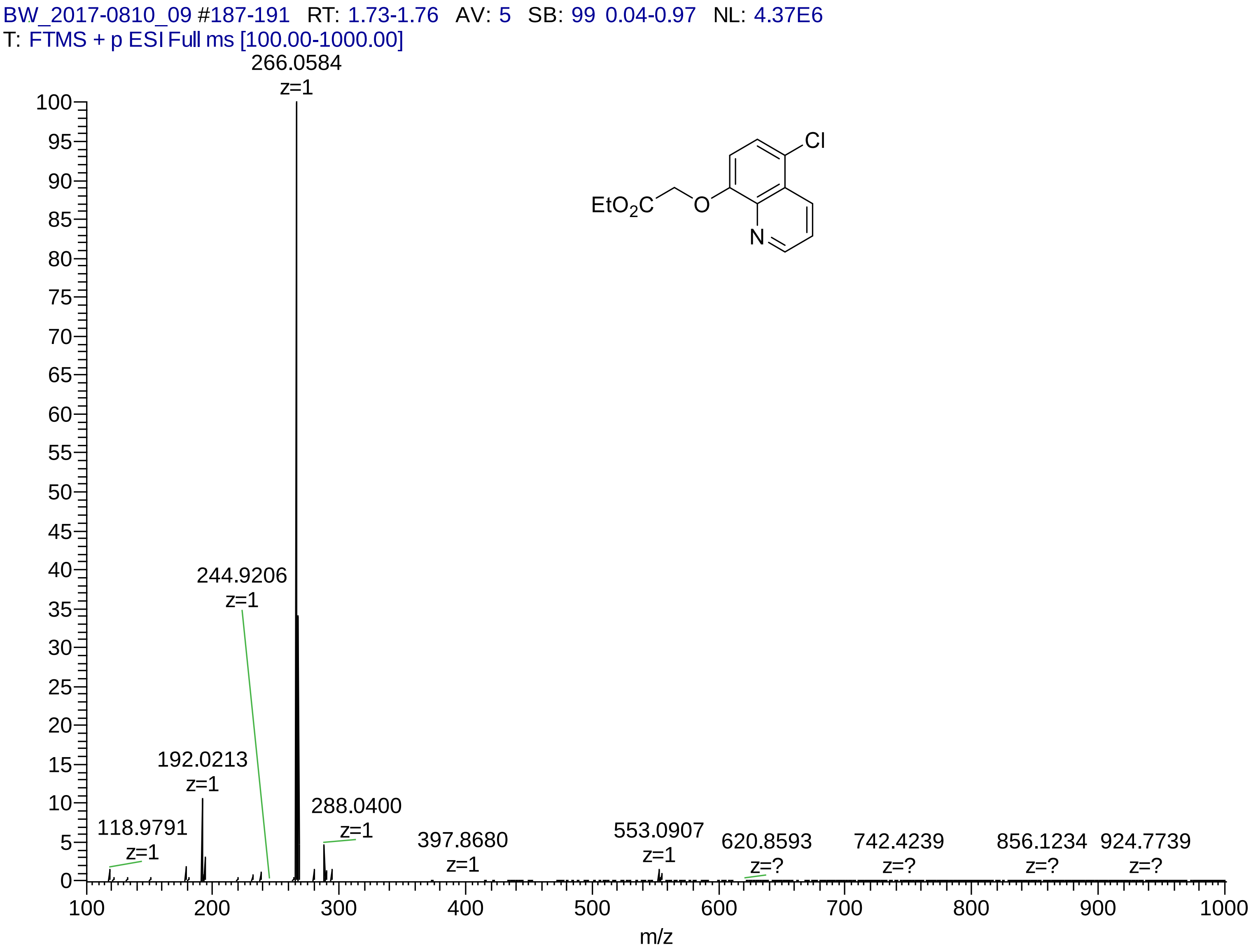

^{1}H NMR spectrum of compound **8h** ($CDCl_3$, 400 MHz):

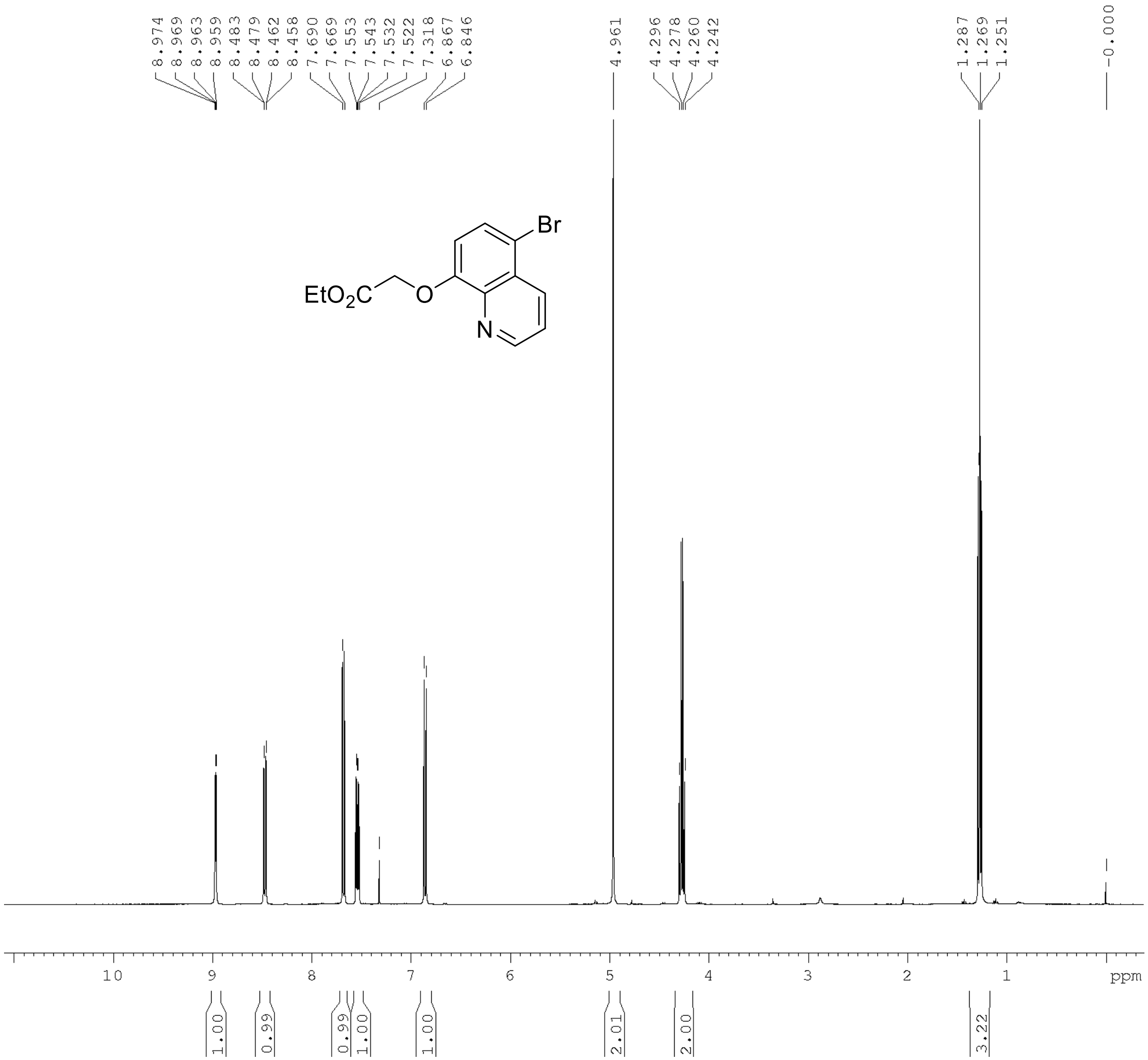

^{13}C{^{1}H}NMR spectrum of compound **8h** ($CDCl_3$, 100 MHz)**:**

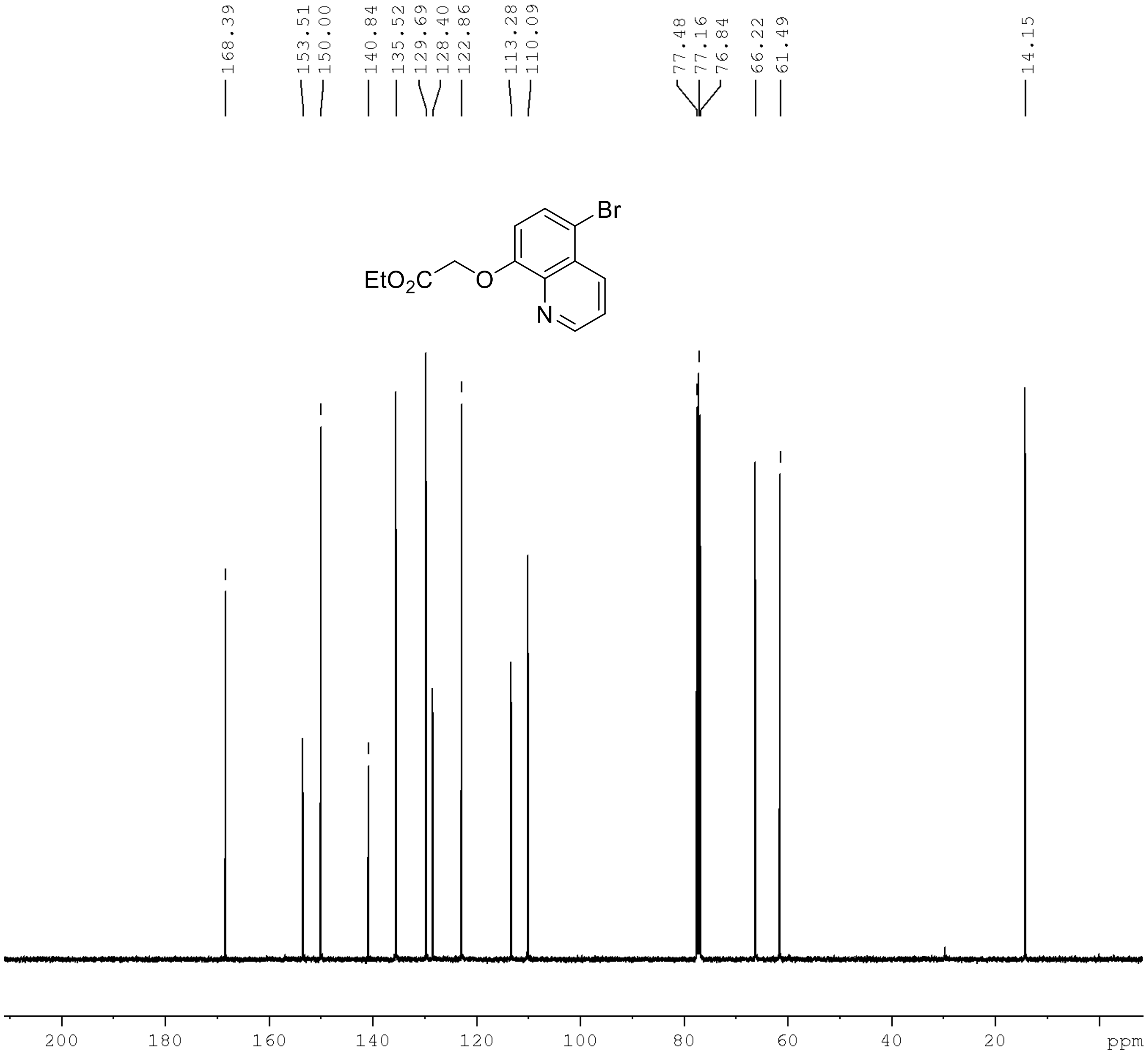

DEPT135 spectrum of compound **8h** ($CDCl_3$, 100 MHz)**:**

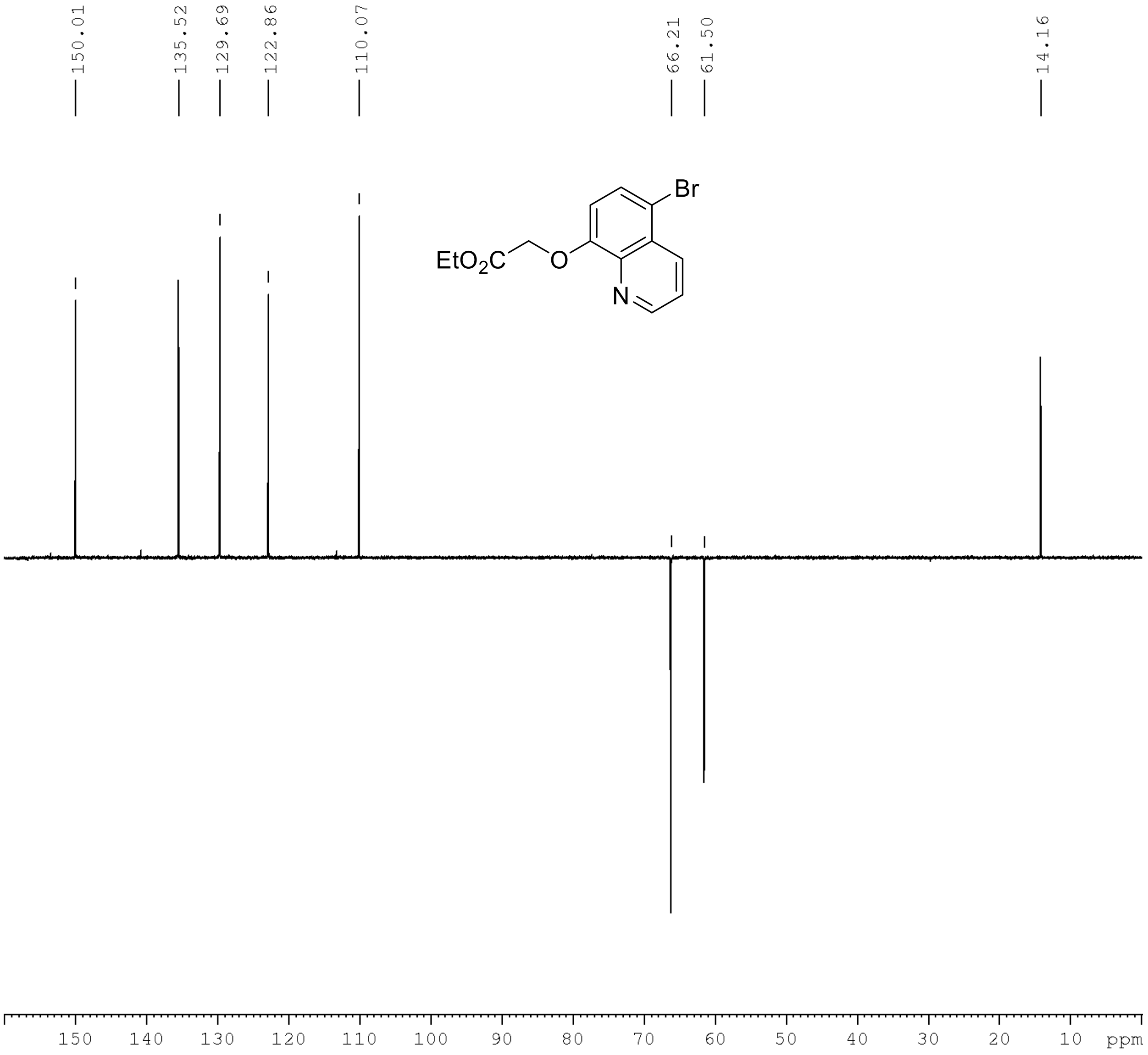

HRMS spectrum of compound **8h:**

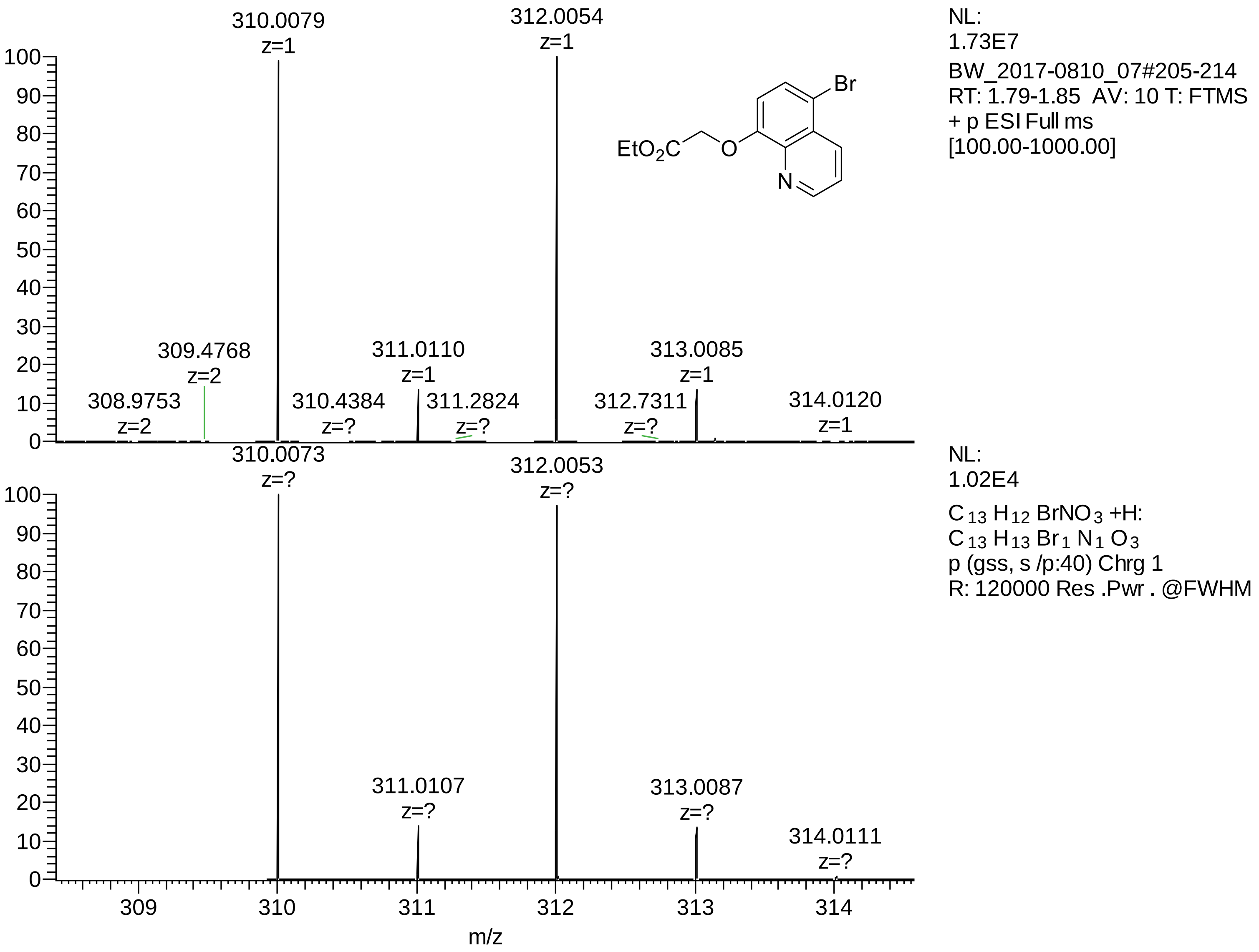

^{1}H NMR spectrum of compound **10a** ($CDCl_3$, 400 MHz)**:**

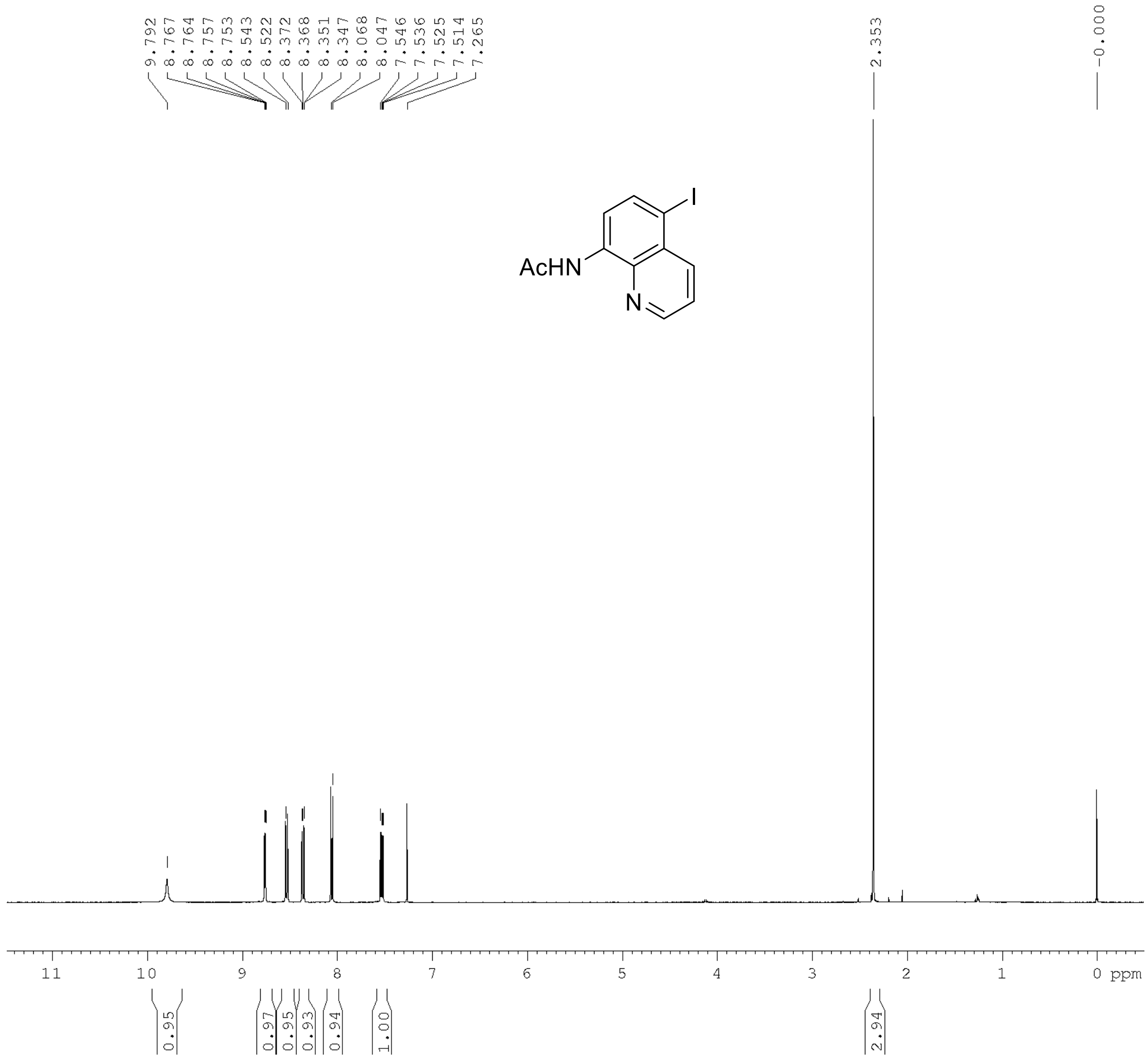

$^{13}C\{^{1}H\}$NMR spectrum of compound **10a** ($CDCl_3$, 100 MHz)**:**

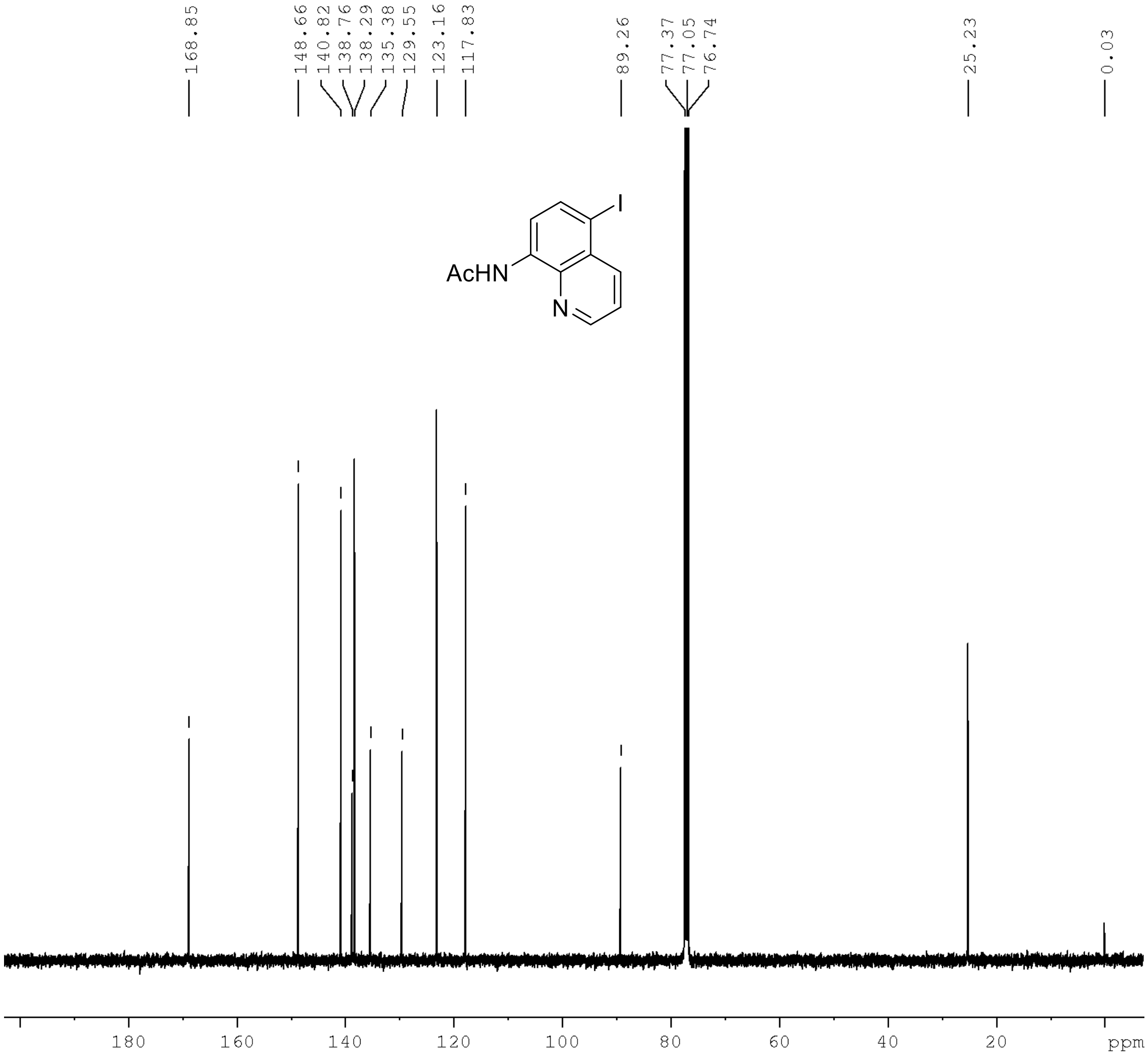

DEPT135 spectrum of compound **10a** ($CDCl_3$, 100 MHz)**:**

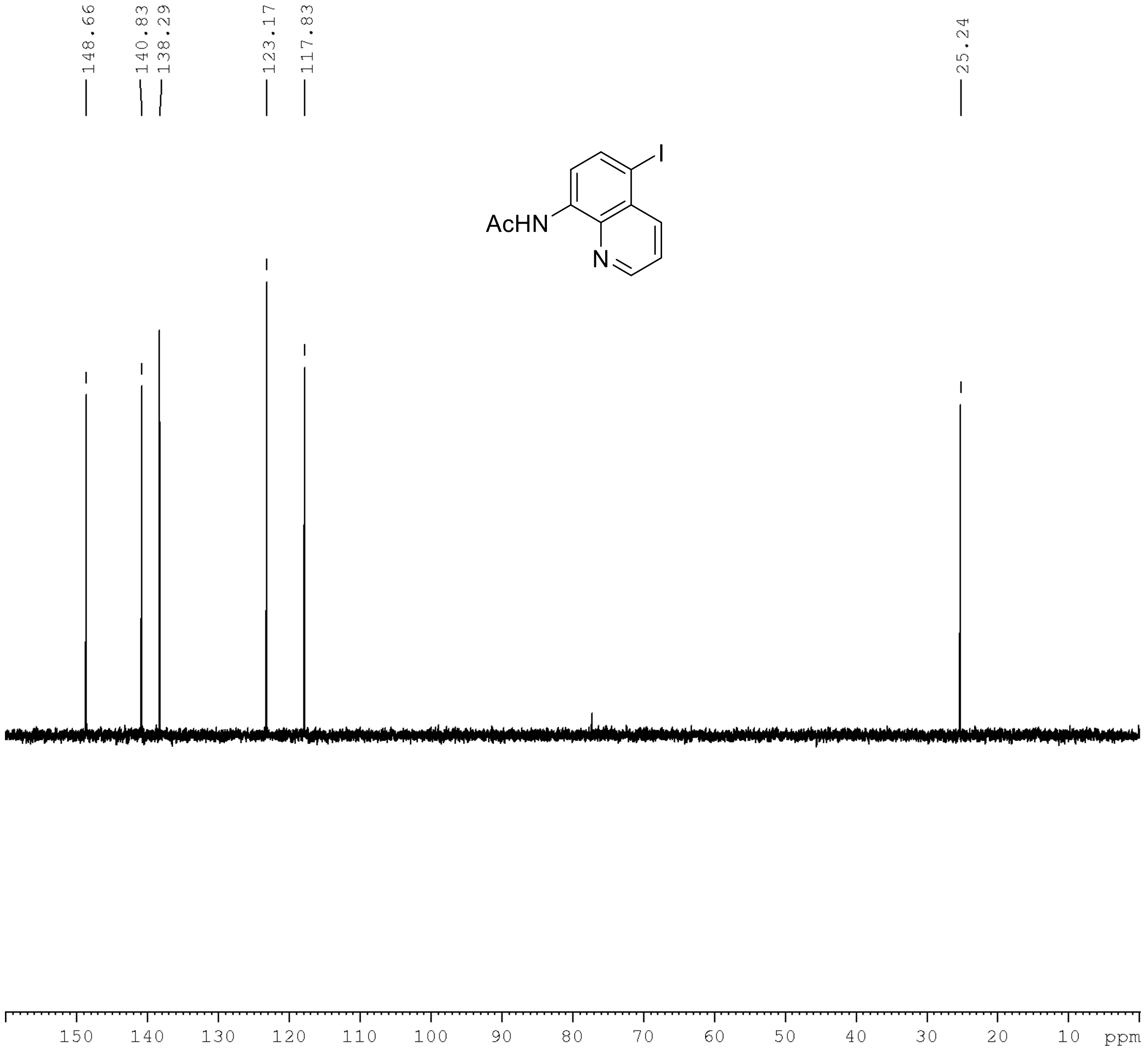

^{1}H NMR spectrum of compound **10b** ($CDCl_3$, 400 MHz)**:**

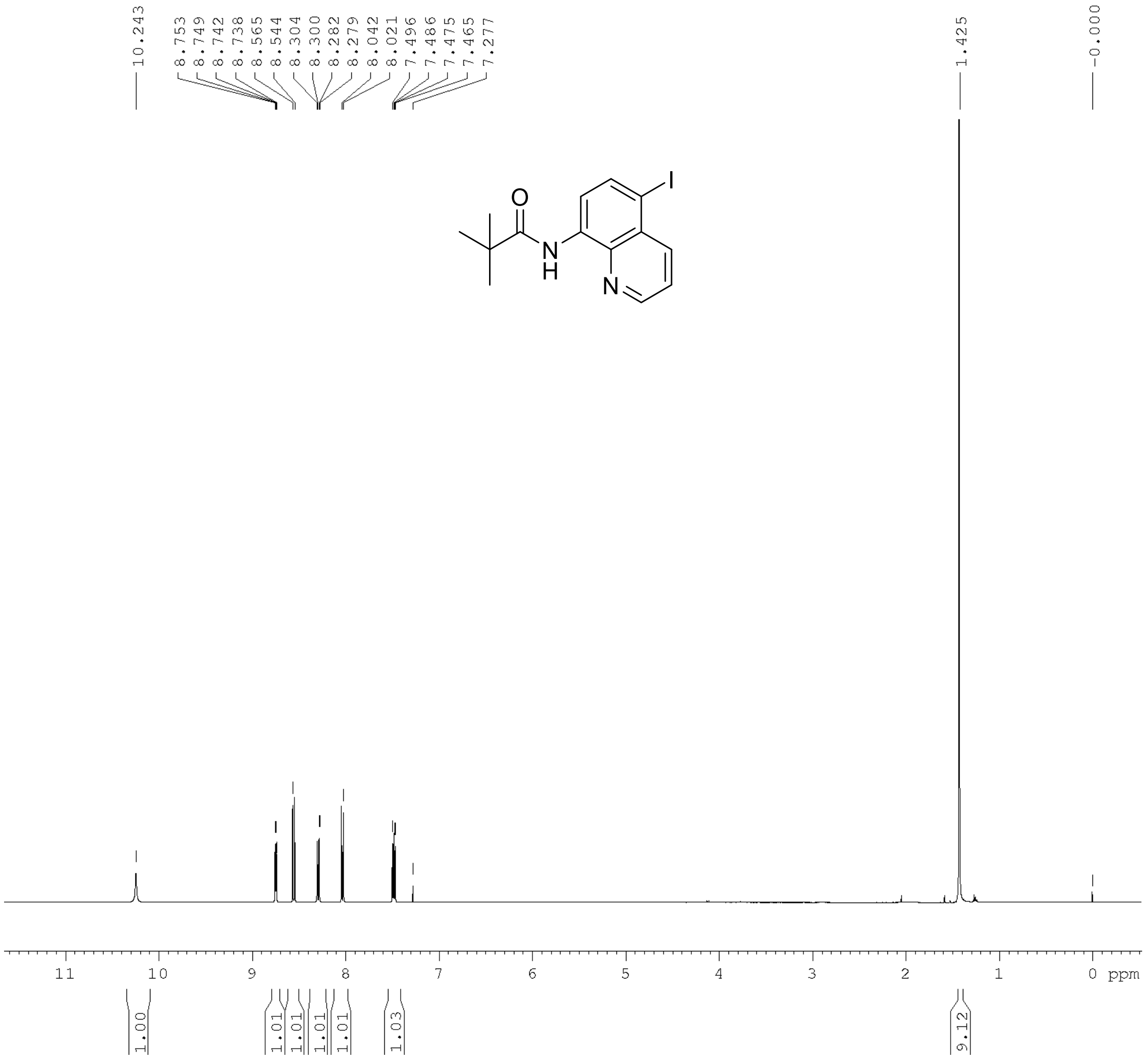

$^{13}C\{^{1}H\}$NMR spectrum of compound **10b** ($CDCl_3$, 100 MHz)**:**

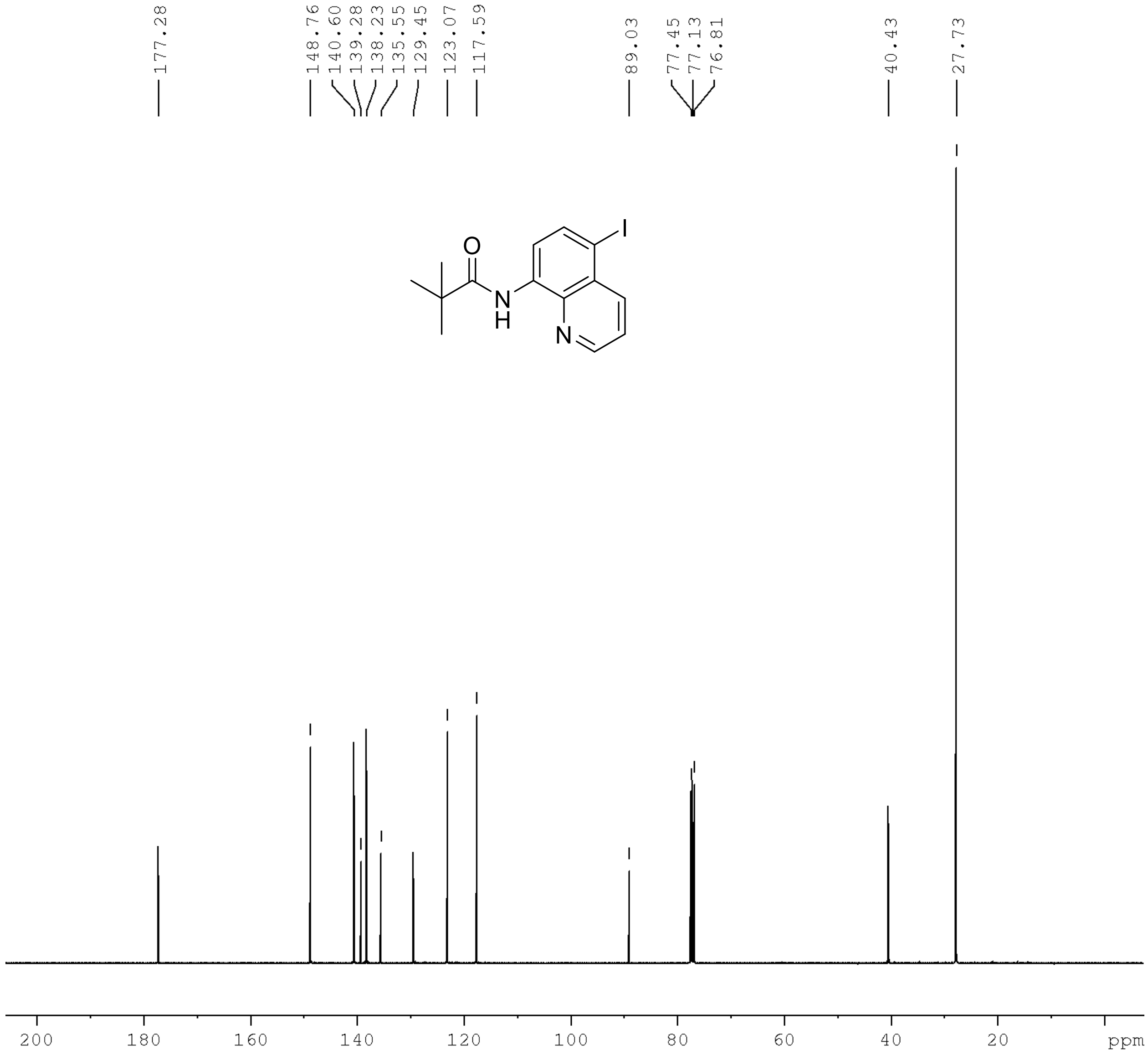

DEPT135 spectrum of compound **10b** ($CDCl_3$, 100 MHz)**:**

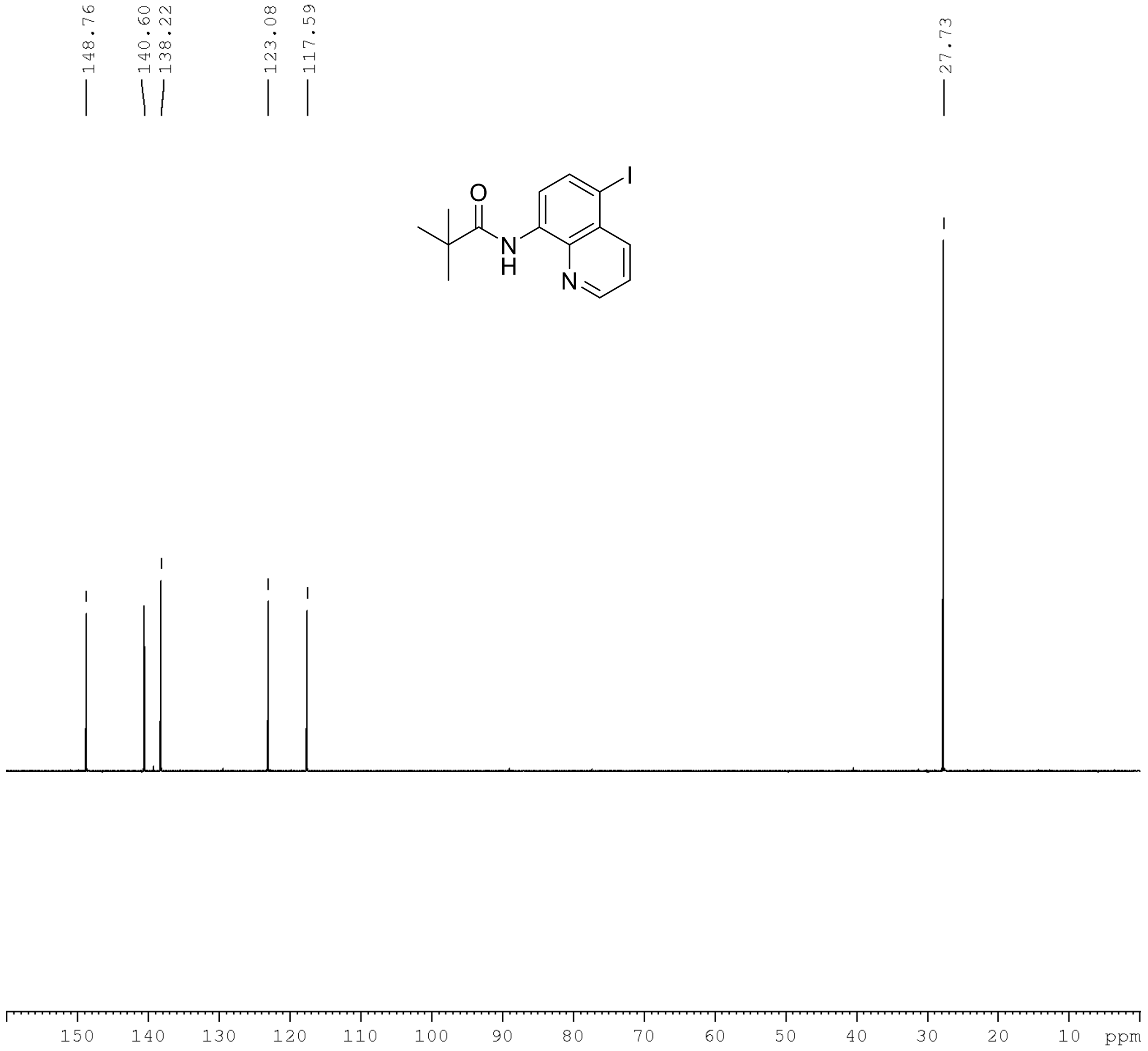

^{1}H NMR spectrum of compound **10c** ($CDCl_3$, 400 MHz):

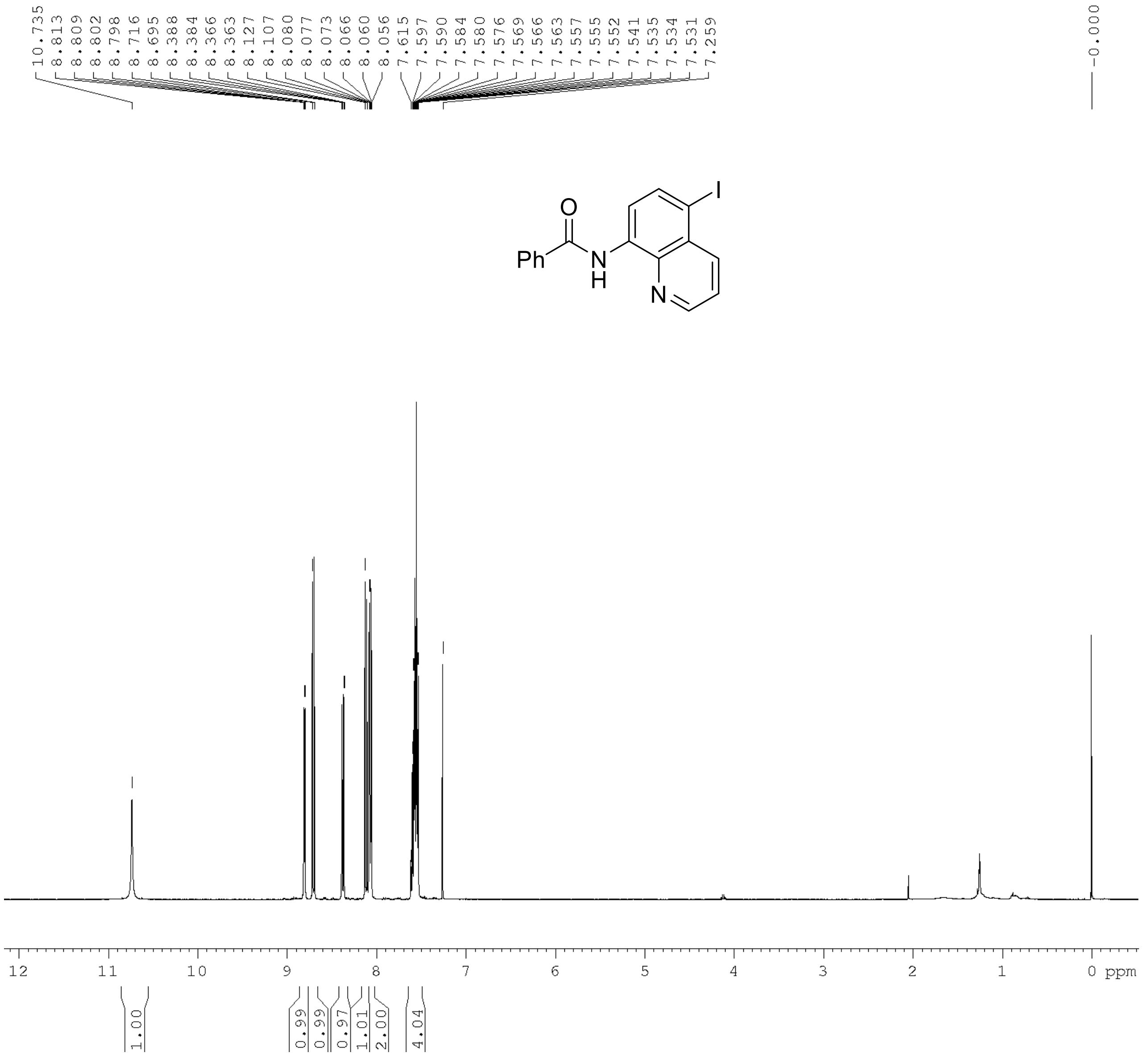

$^{13}C\{^{1}H\}$NMR spectrum of compound **10c** ($CDCl_3$, 100 MHz):

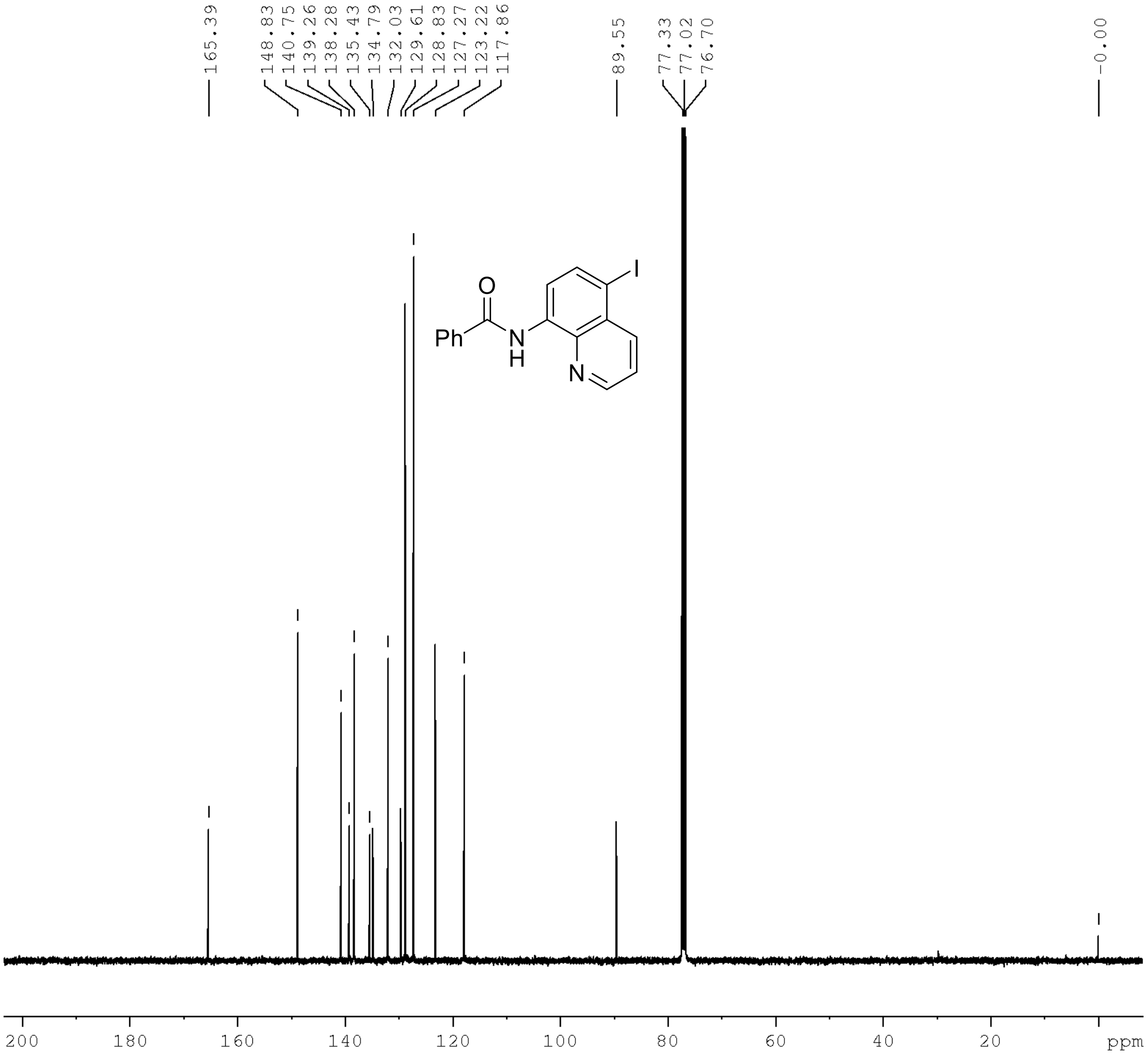

DEPT135 spectrum of compound **10c** ($CDCl_3$, 100 MHz)**:**

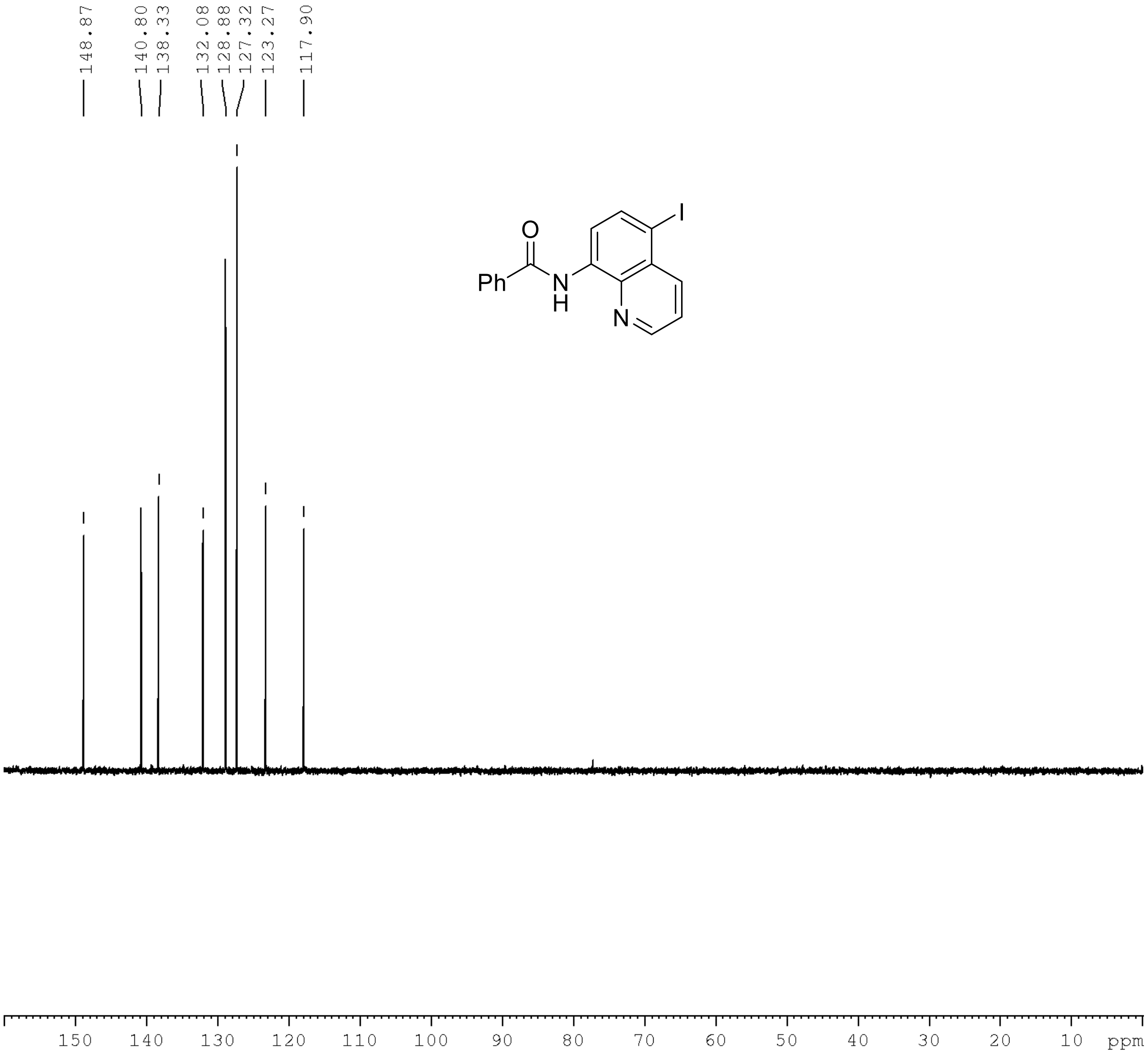

^{1}H NMR spectrum of compound **10d** ($CDCl_3$, 400 MHz)**:**

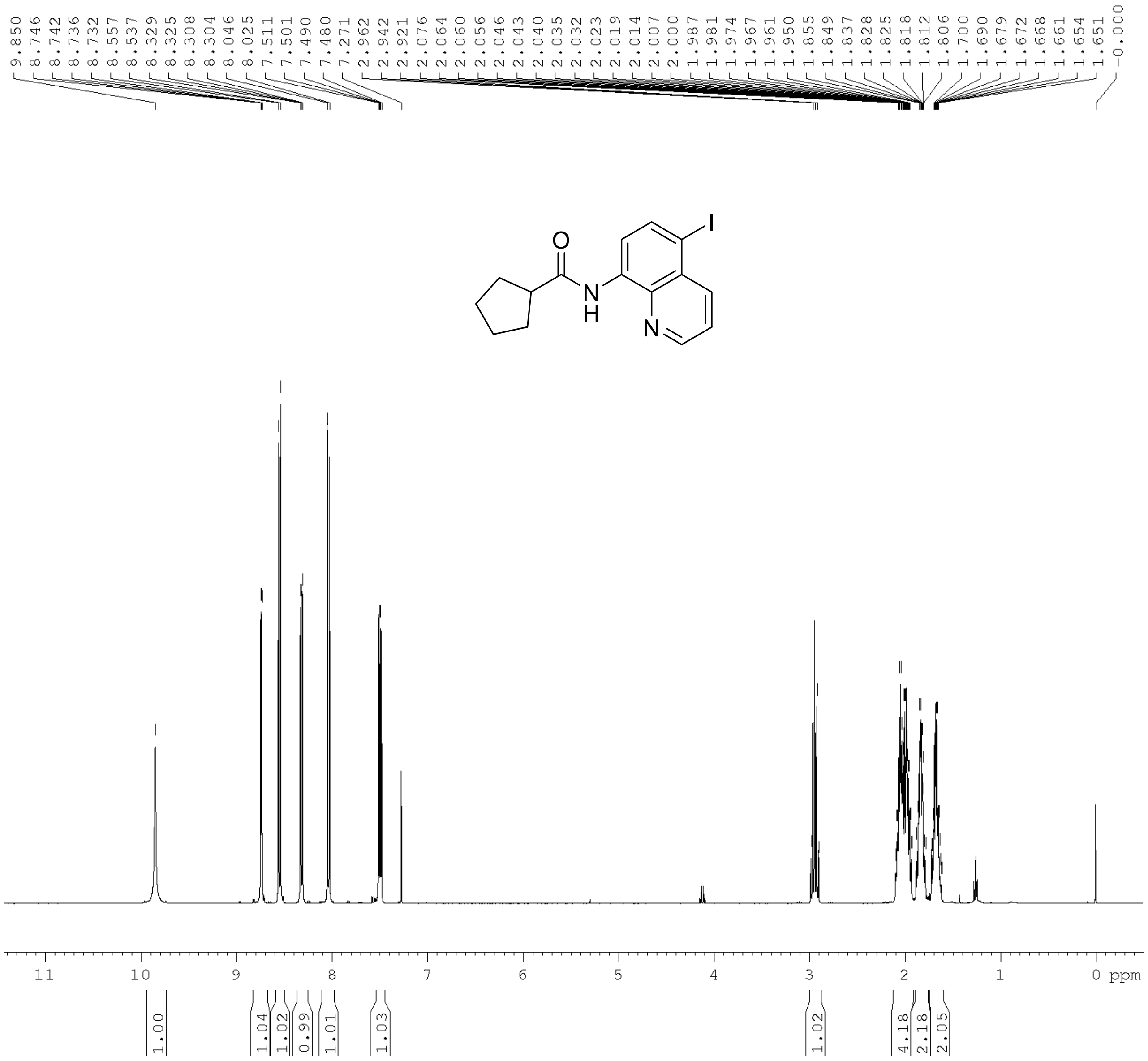

$^{13}C\{^{1}H\}$NMR spectrum of compound **10d** ($CDCl_3$, 100 MHz)**:**

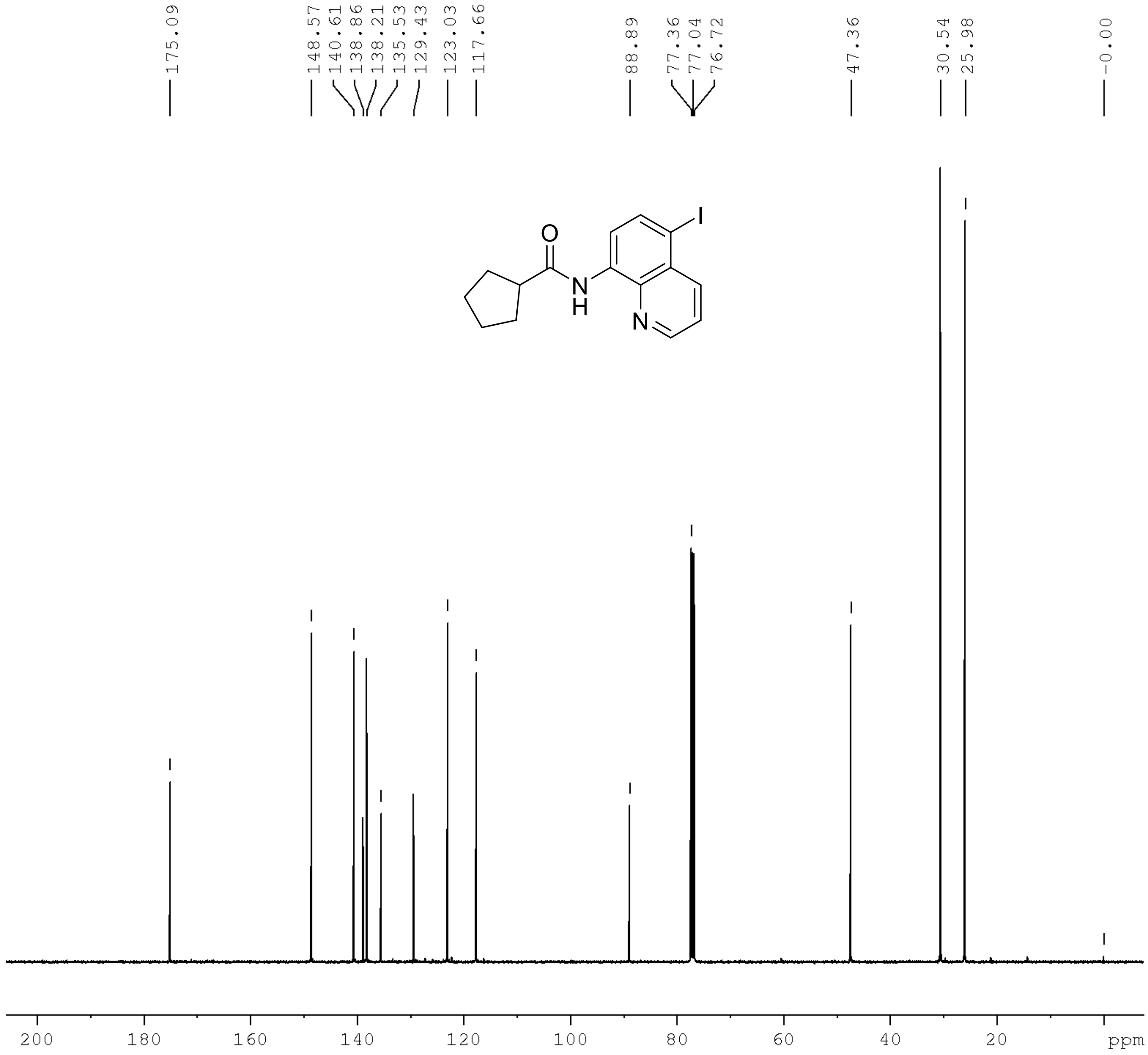

DEPT135 spectrum of compound **10d** ($CDCl_3$, 100 MHz)**:**

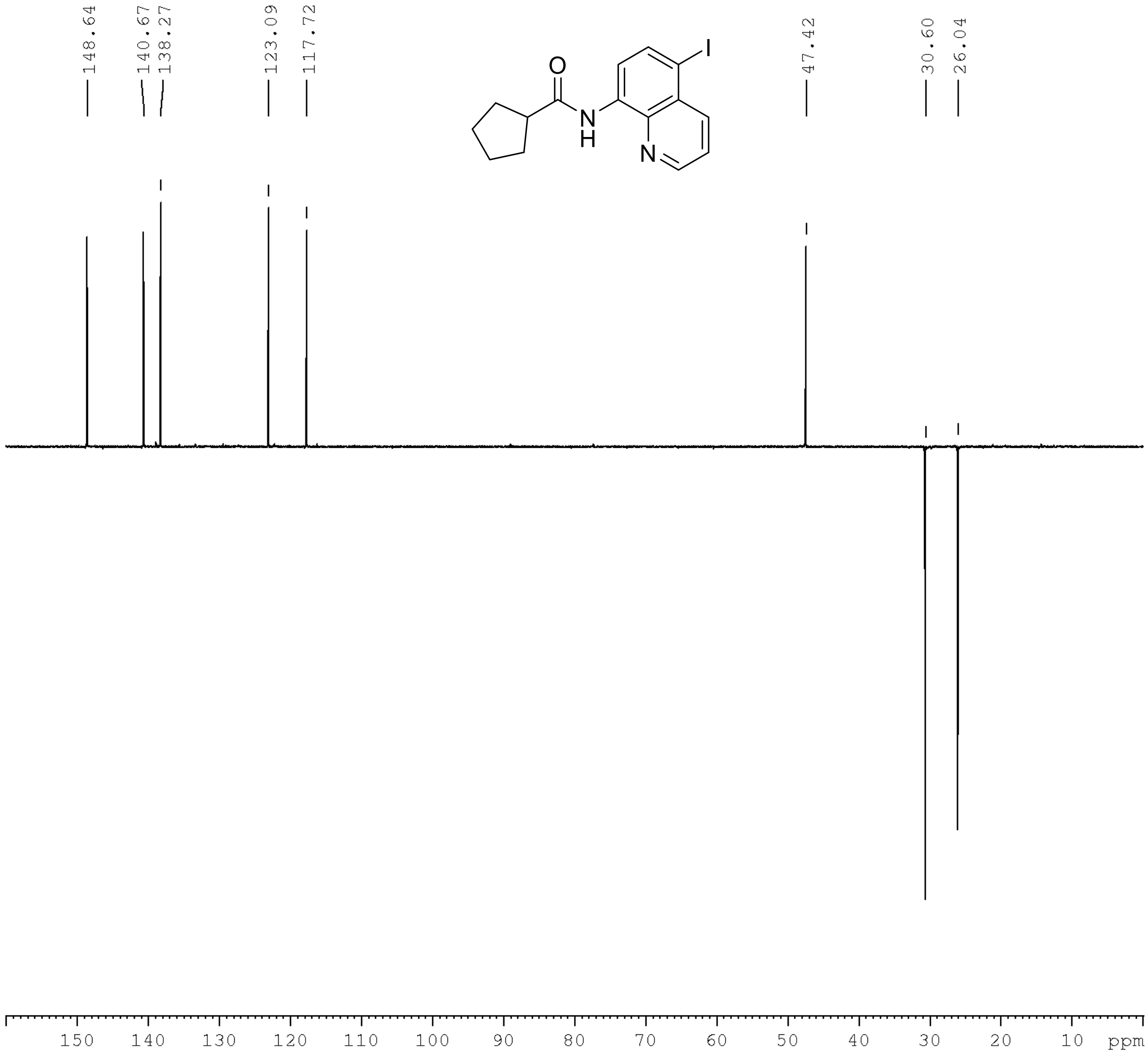

^{1}H NMR spectrum of compound **10e** ($CDCl_3$, 400 MHz)**:**

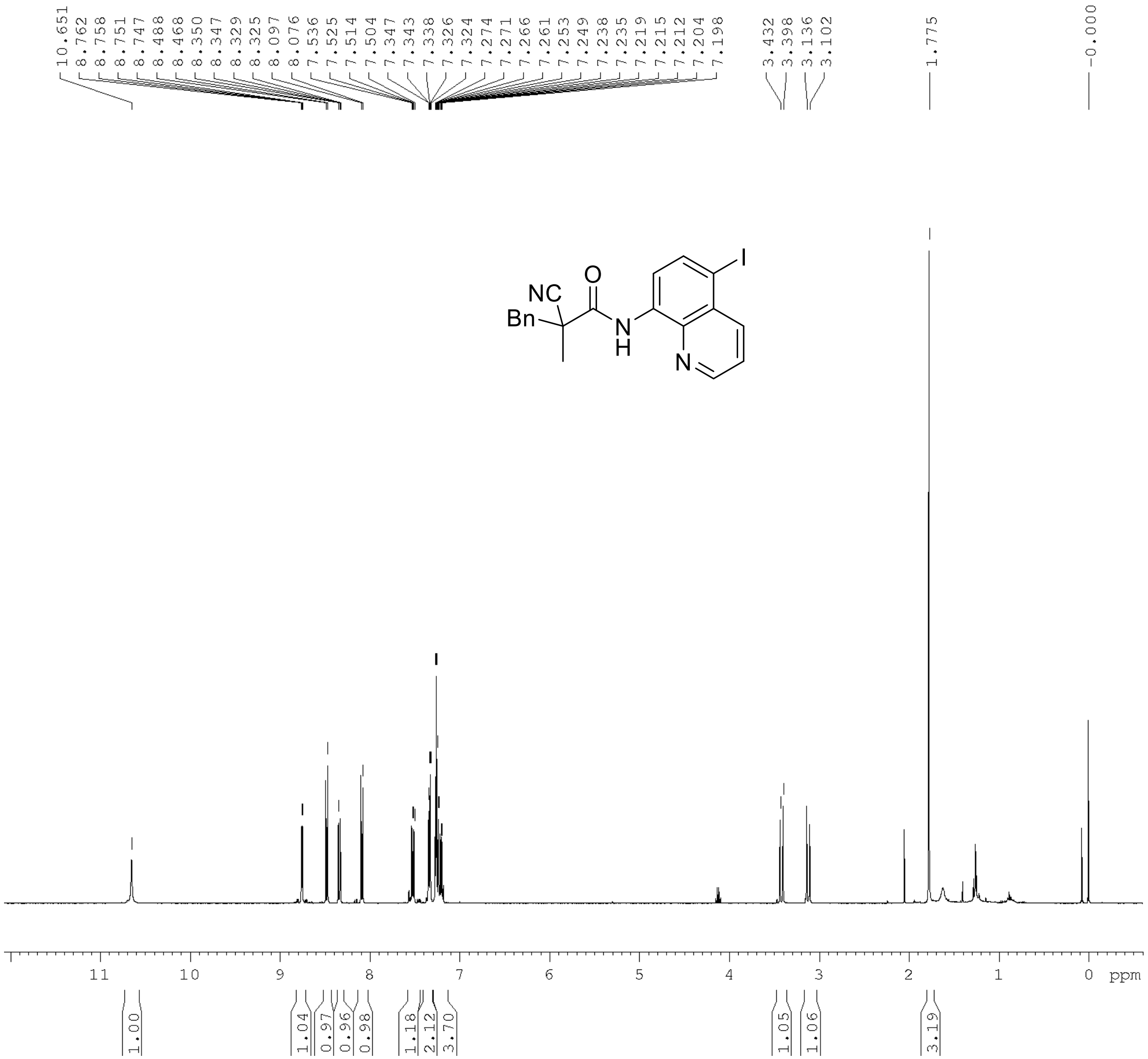

$^{13}C\{^{1}H\}$NMR spectrum of compound **10e** ($CDCl_3$, 100 MHz)**:**

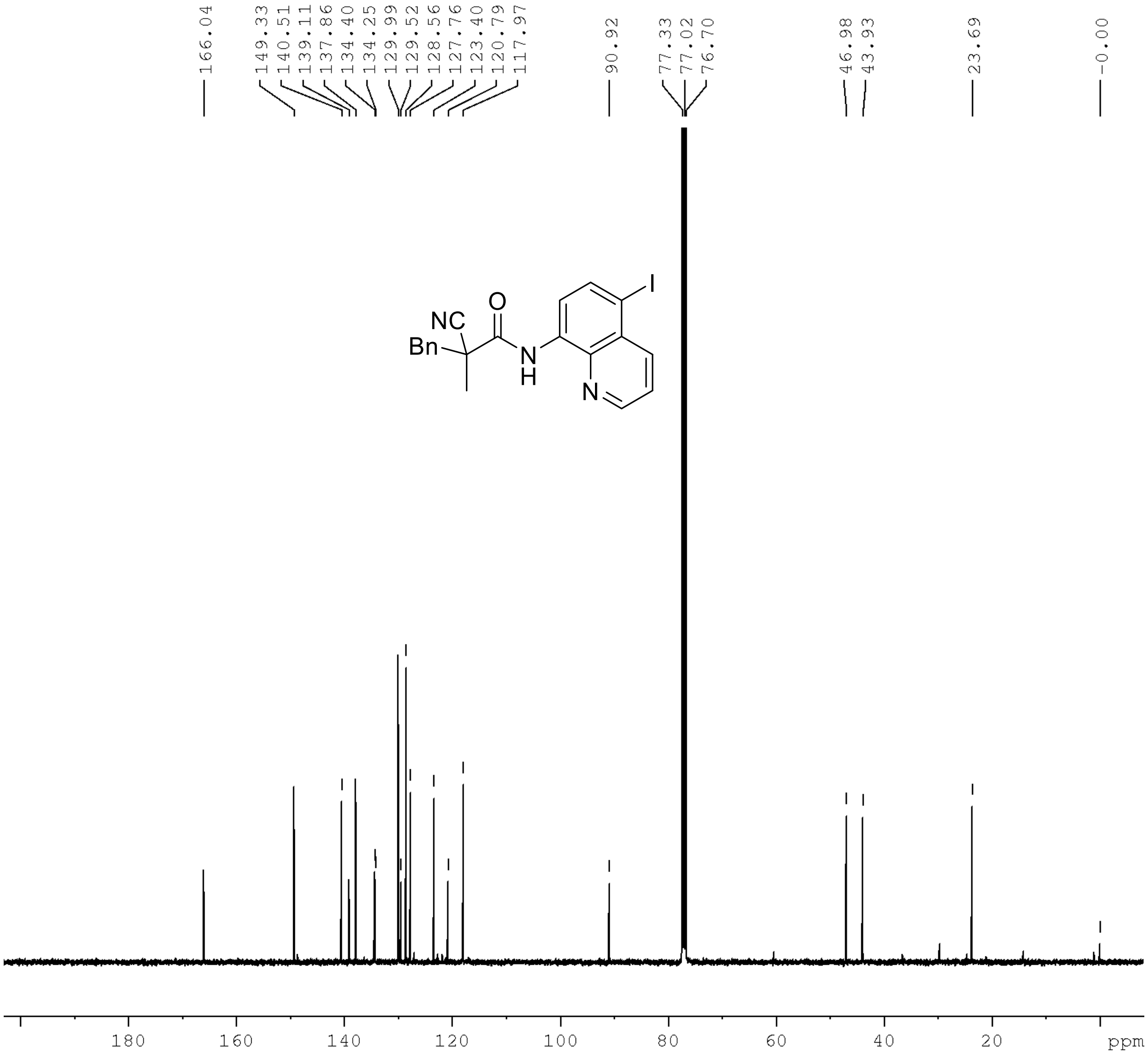

DEPT135 spectrum of compound **10e** ($CDCl_3$, 100 MHz)**:**

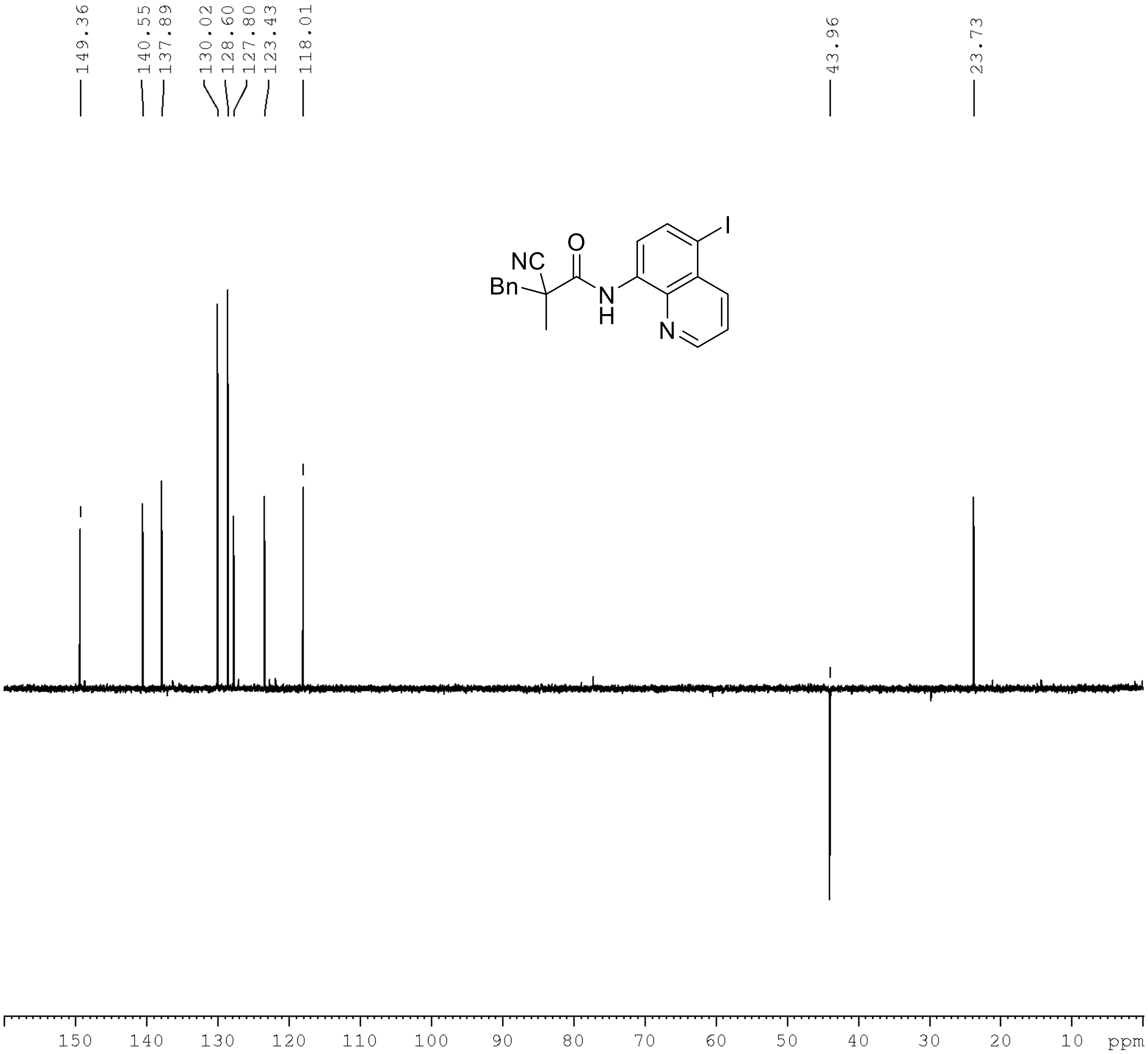

^{1}H NMR spectrum of compound **10f** ($CDCl_3$, 400 MHz)**:**

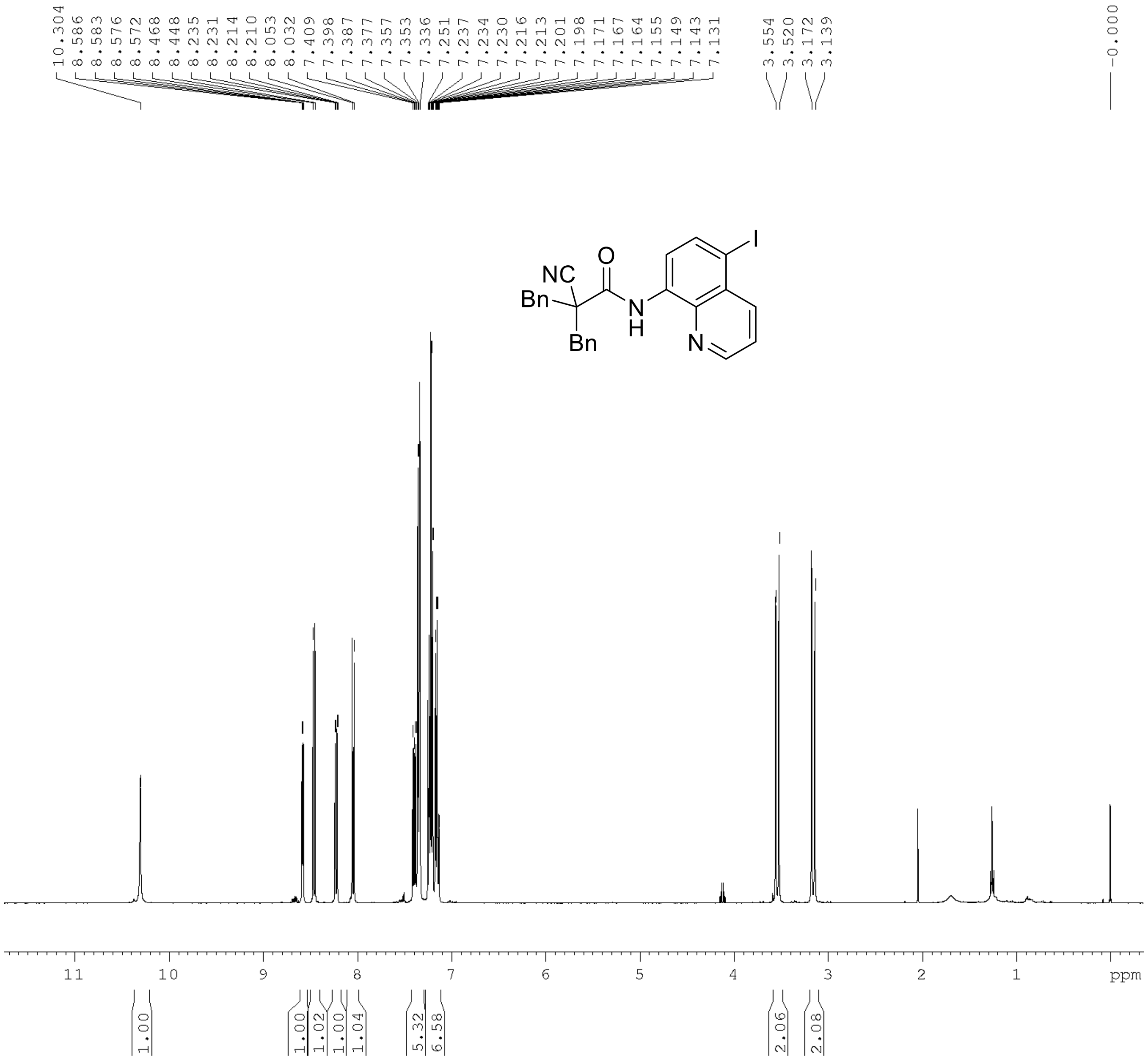

$^{13}C\{^{1}H\}$NMR spectrum of compound **10f** ($CDCl_3$, 100 MHz)**:**

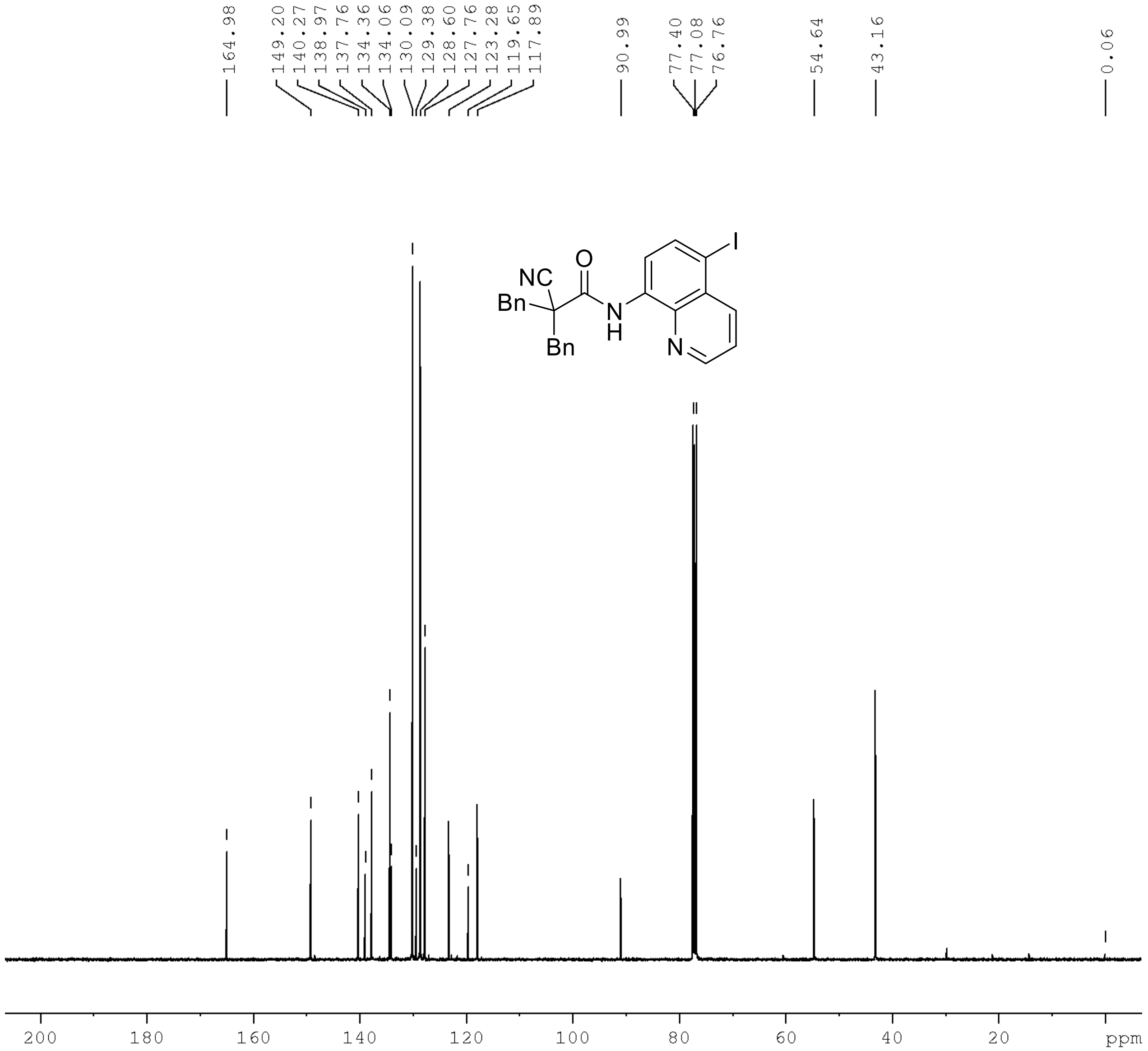

DEPT135 spectrum of compound **10f** ($CDCl_3$, 100 MHz):

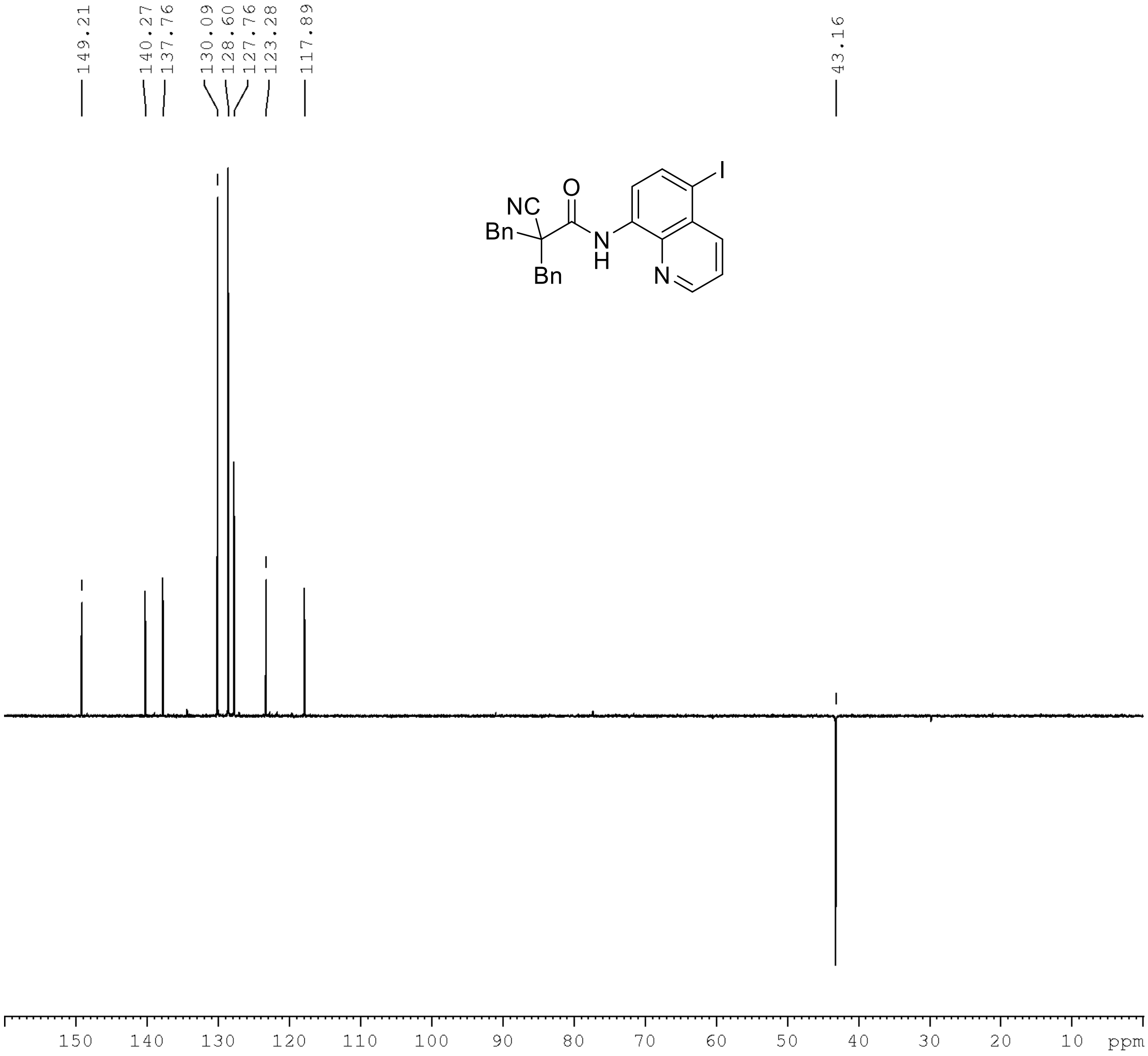

HRMS spectrum of compound **10f:**

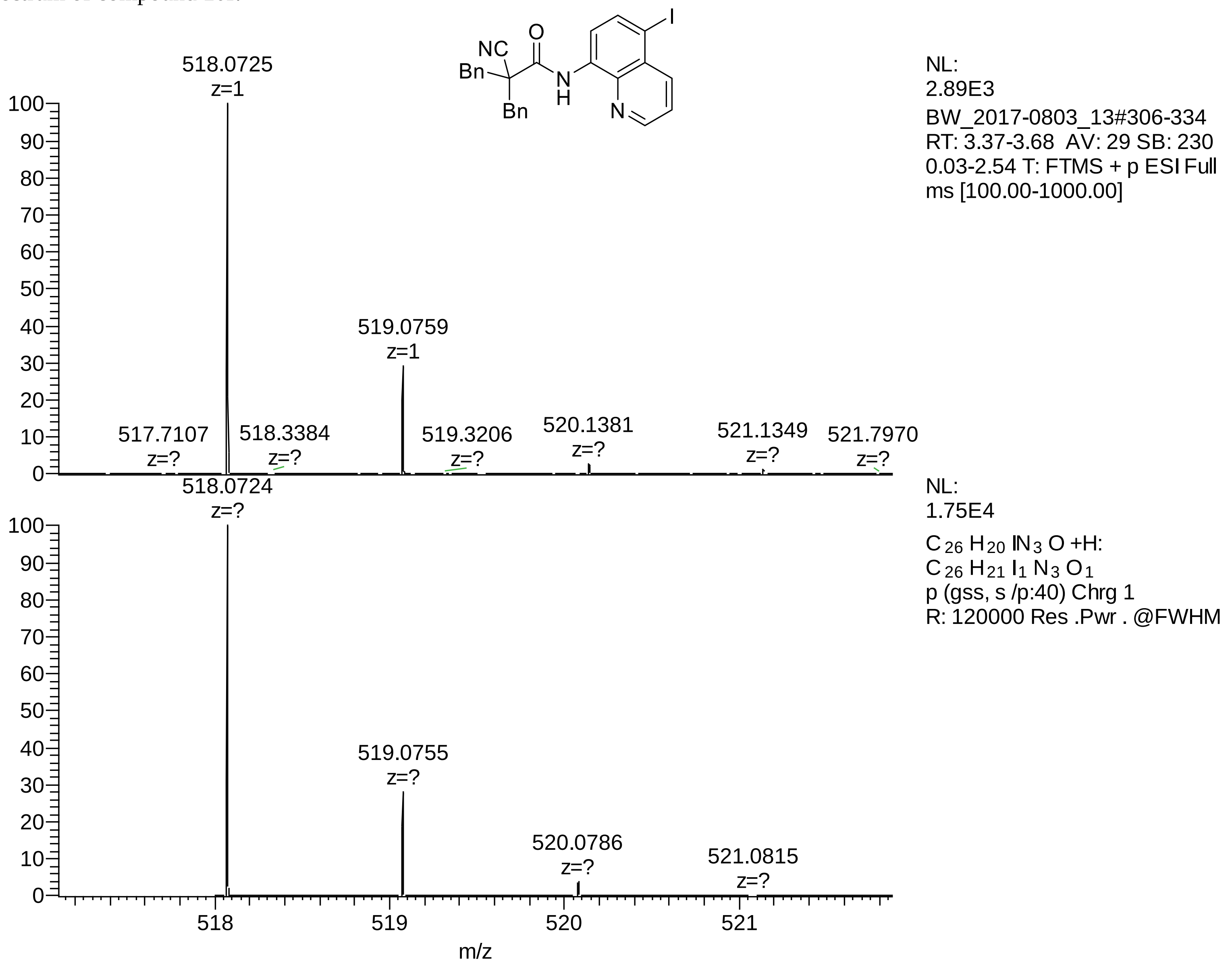

^{1}H NMR spectrum of compound **10g** ($CDCl_3$, 400 MHz)**:**

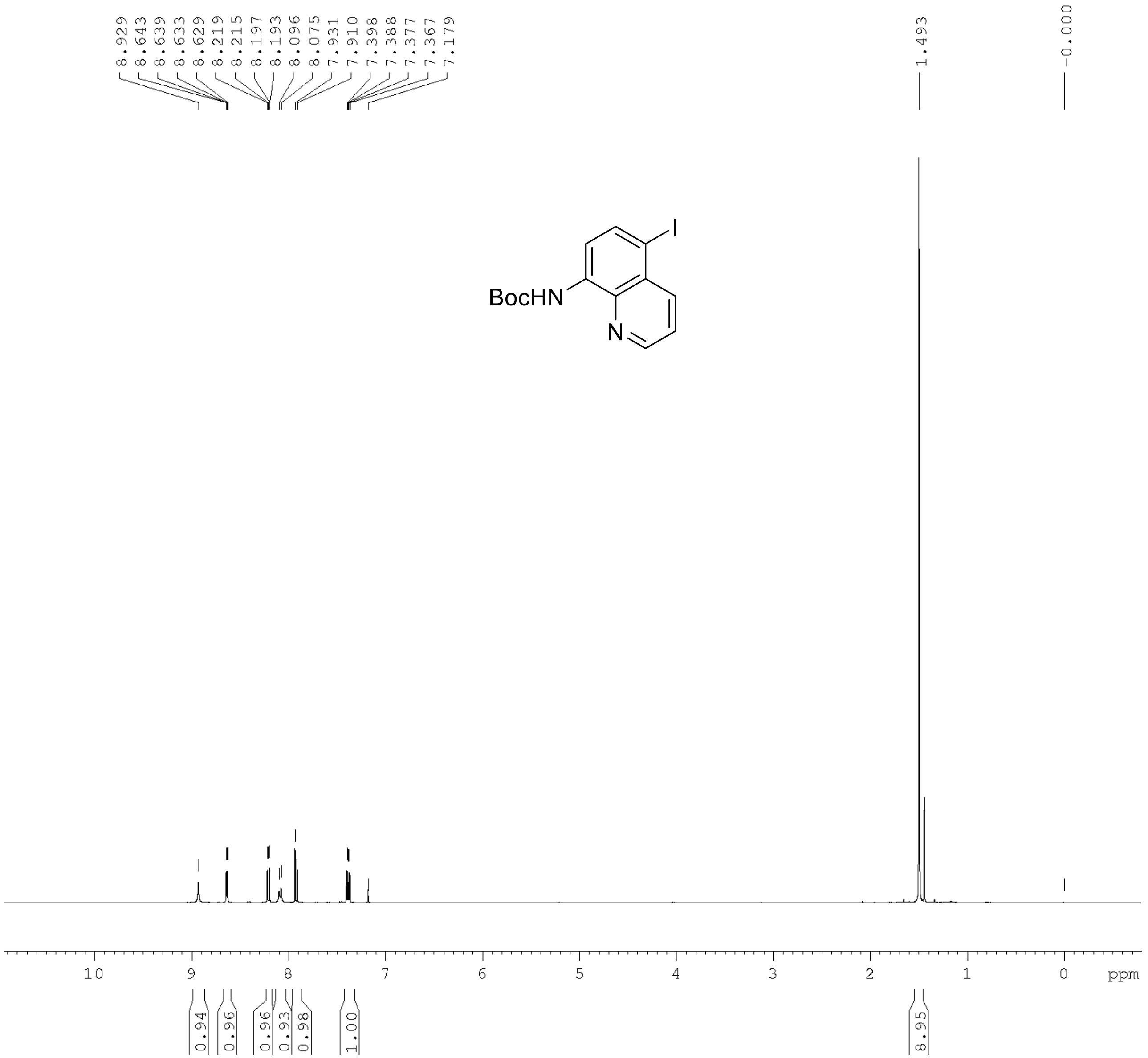

$^{13}C\{^1H\}$NMR spectrum of compound **10g** ($CDCl_3$, 100 MHz)**:**

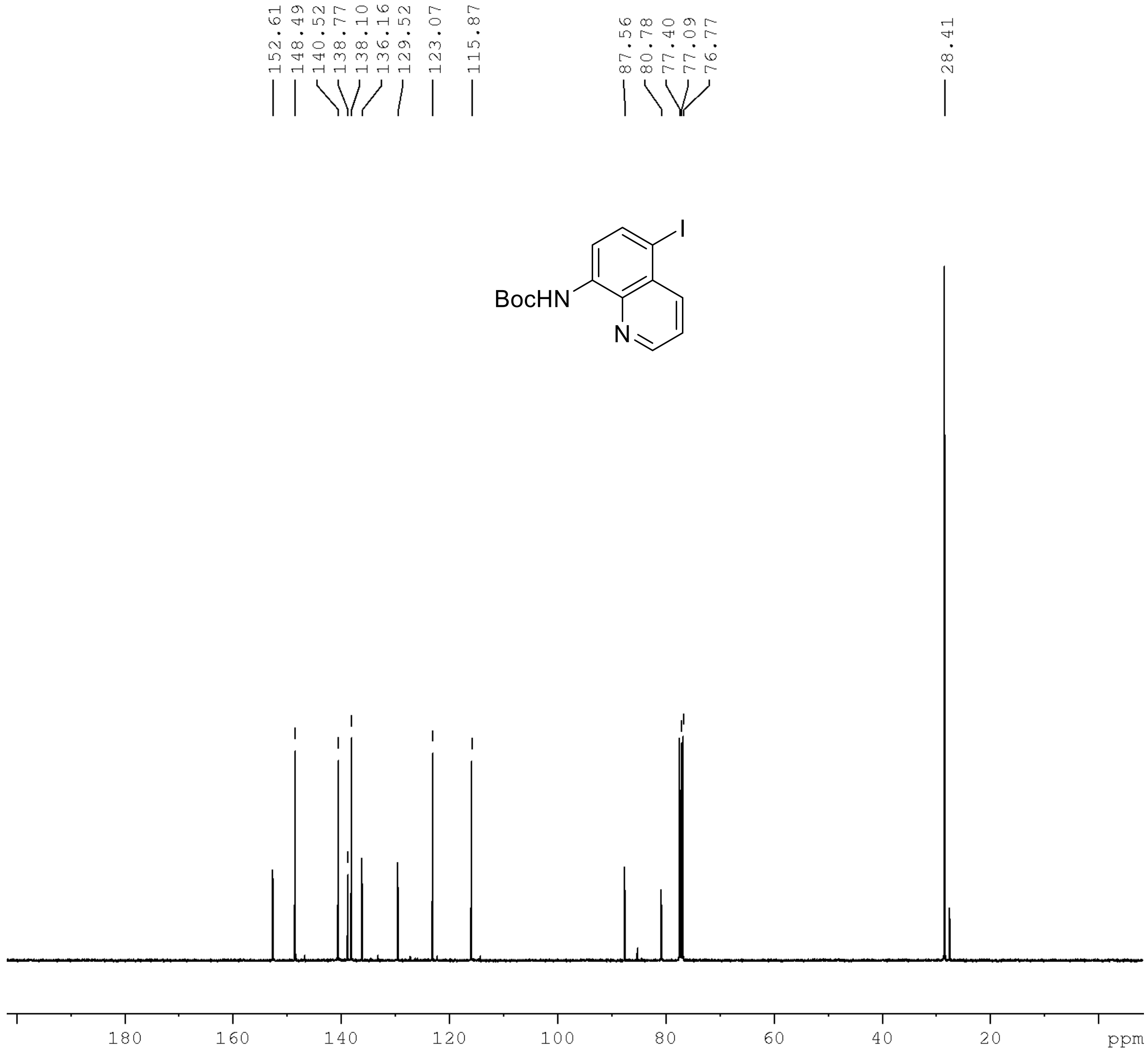

^1H NMR spectrum of compound **10h** ($CDCl_3$, 400 MHz)**:**

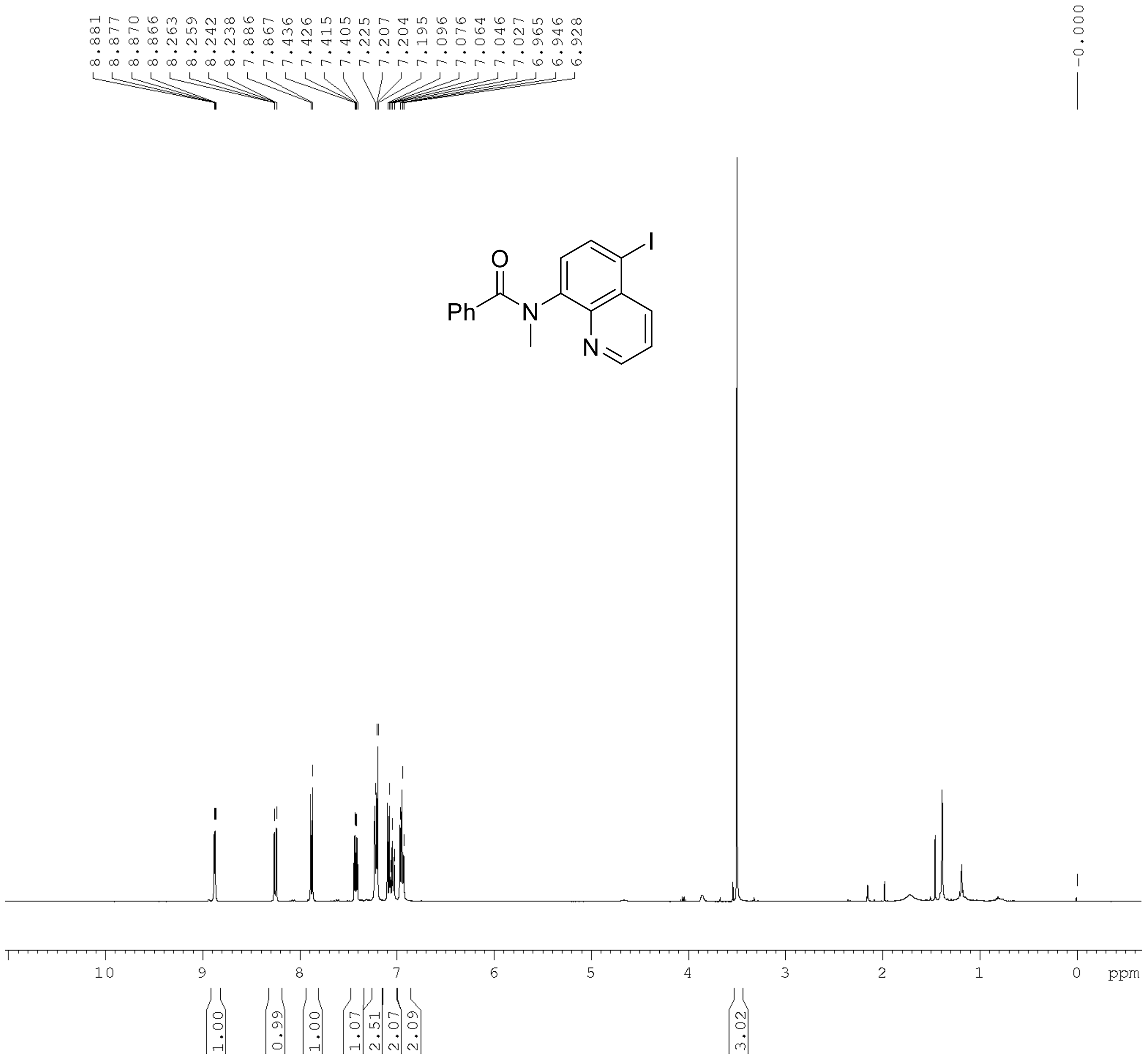

$^{13}C\{^{1}H\}$NMR spectrum of compound **10h** ($CDCl_3$, 100 MHz):

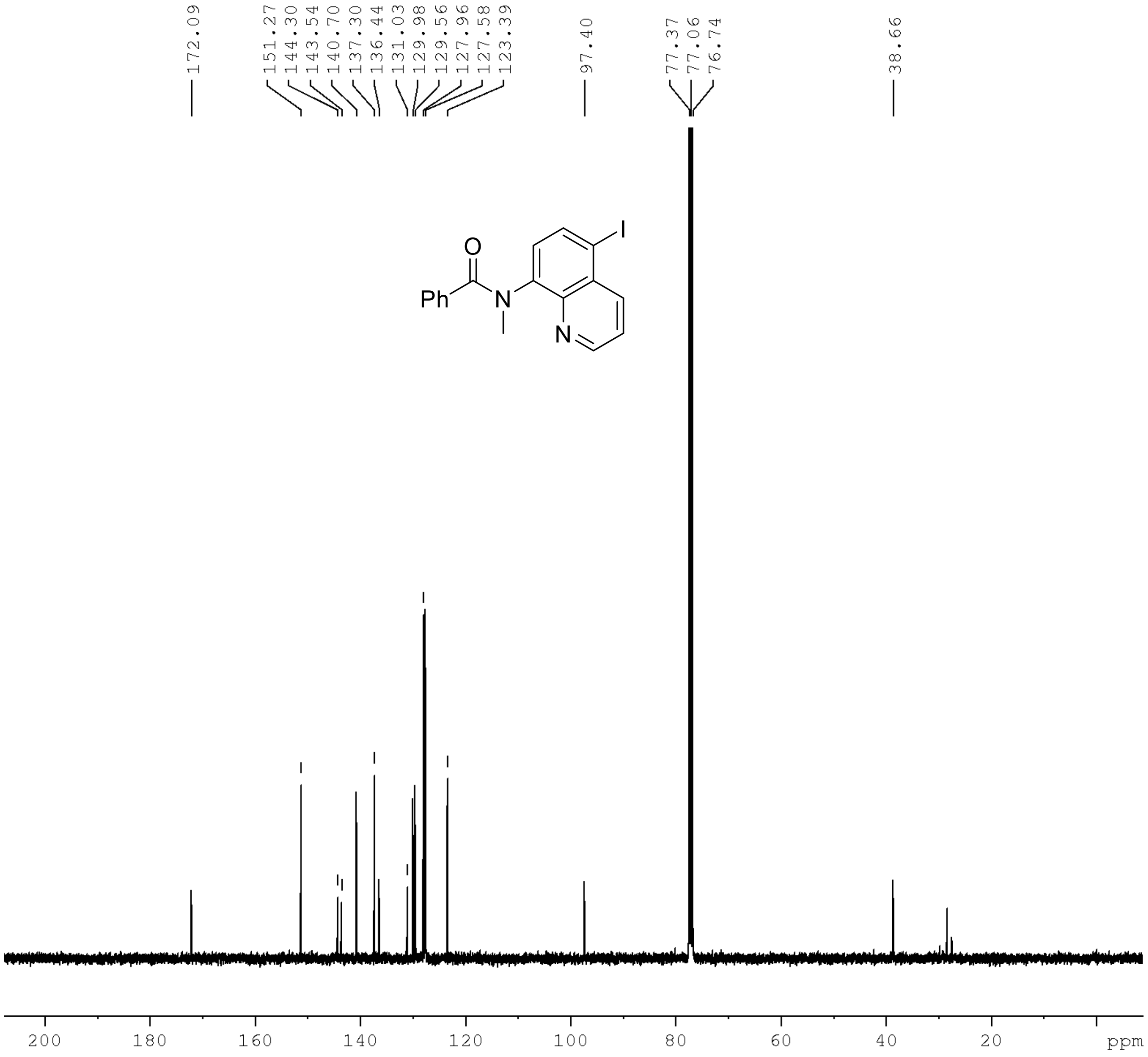

HRMS spectrum of compound **10h:**

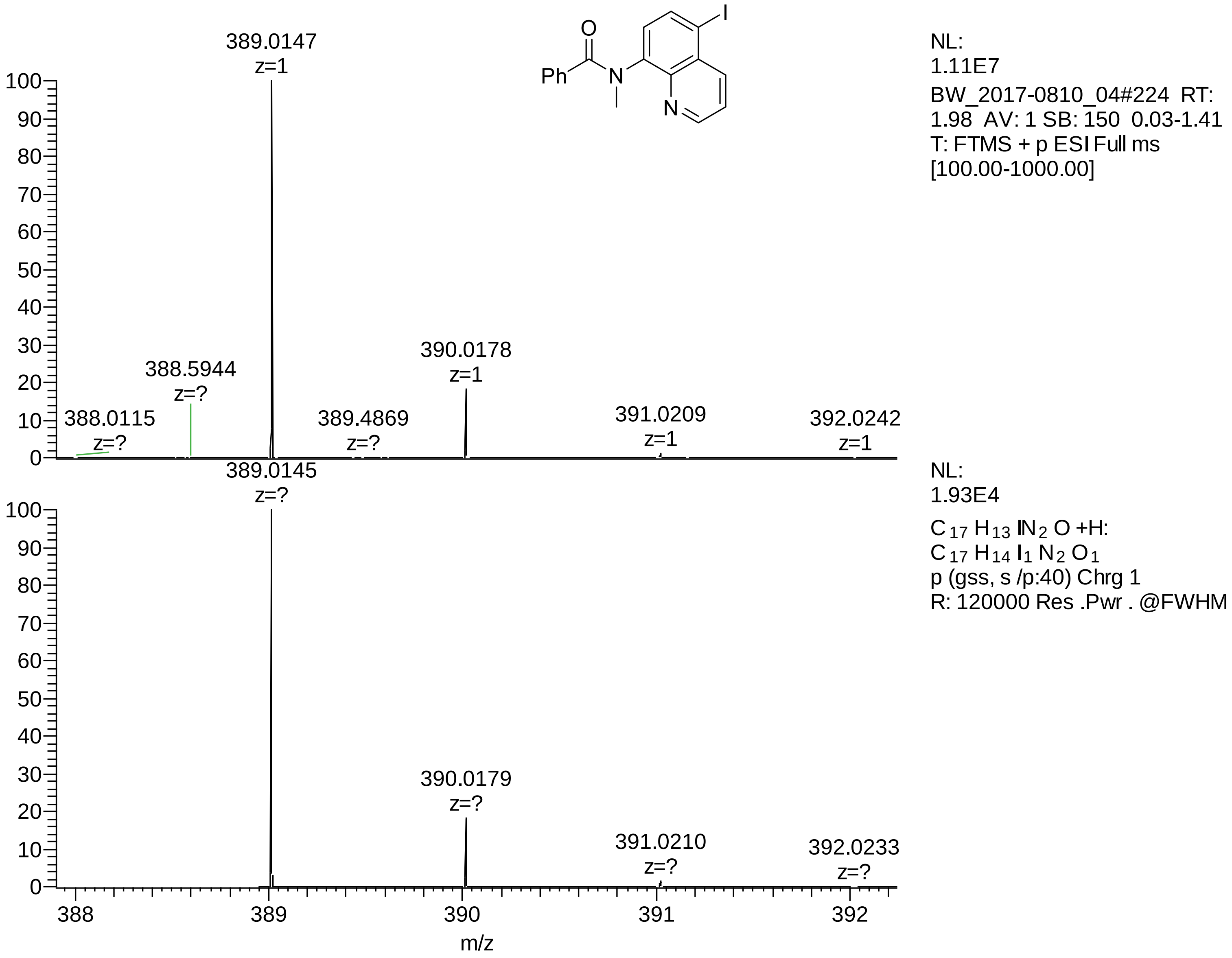

^{1}H NMR spectrum of compound **10i** ($CDCl_3$, 400 MHz)**:**

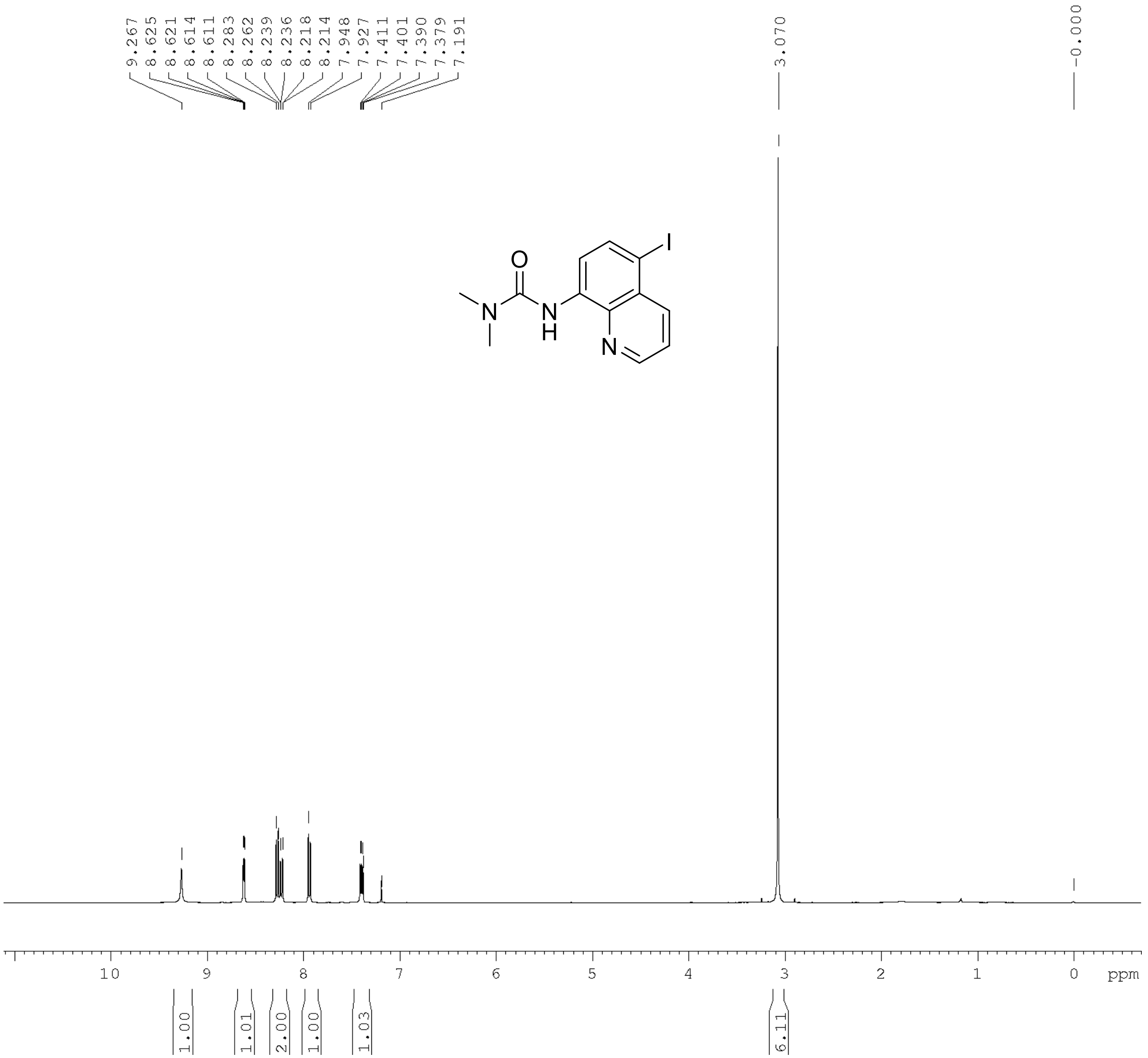

$^{13}C\{^1H\}$NMR spectrum of compound **10i** ($CDCl_3$, 100 MHz)**:**

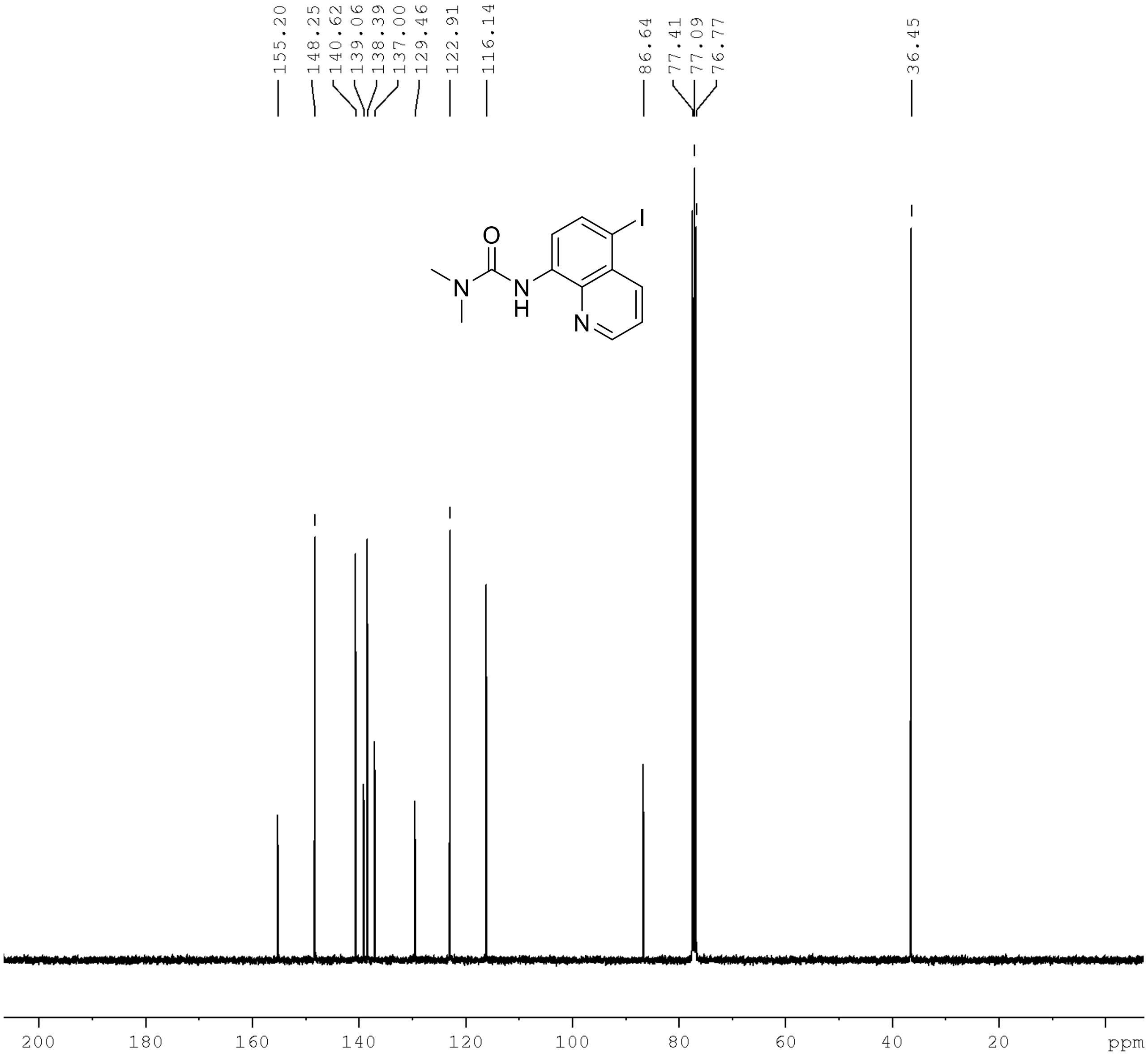

HRMS spectrum of compound **10i:**

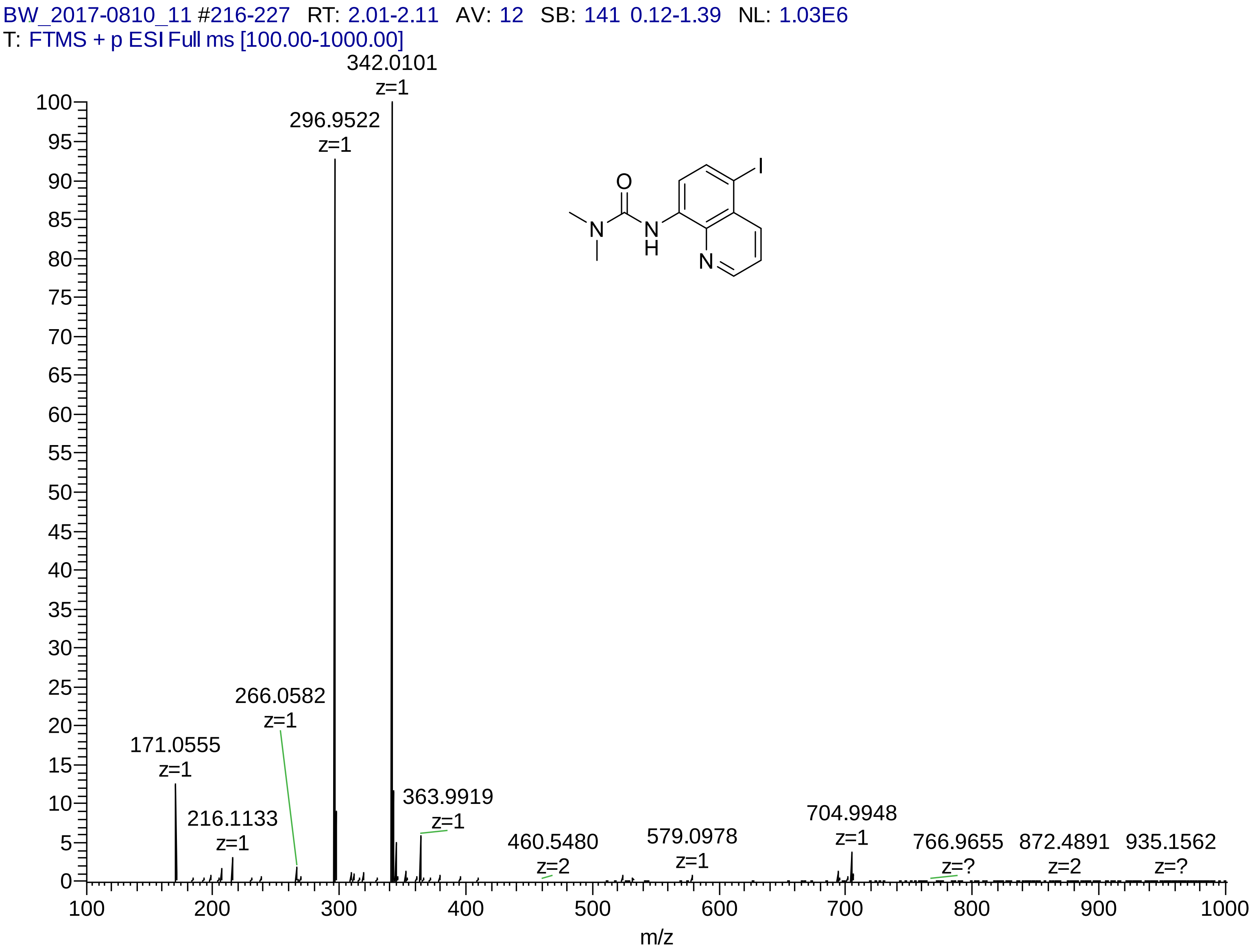

^{1}H NMR spectrum of compound **10j** ($CDCl_3$, 400 MHz)**:**

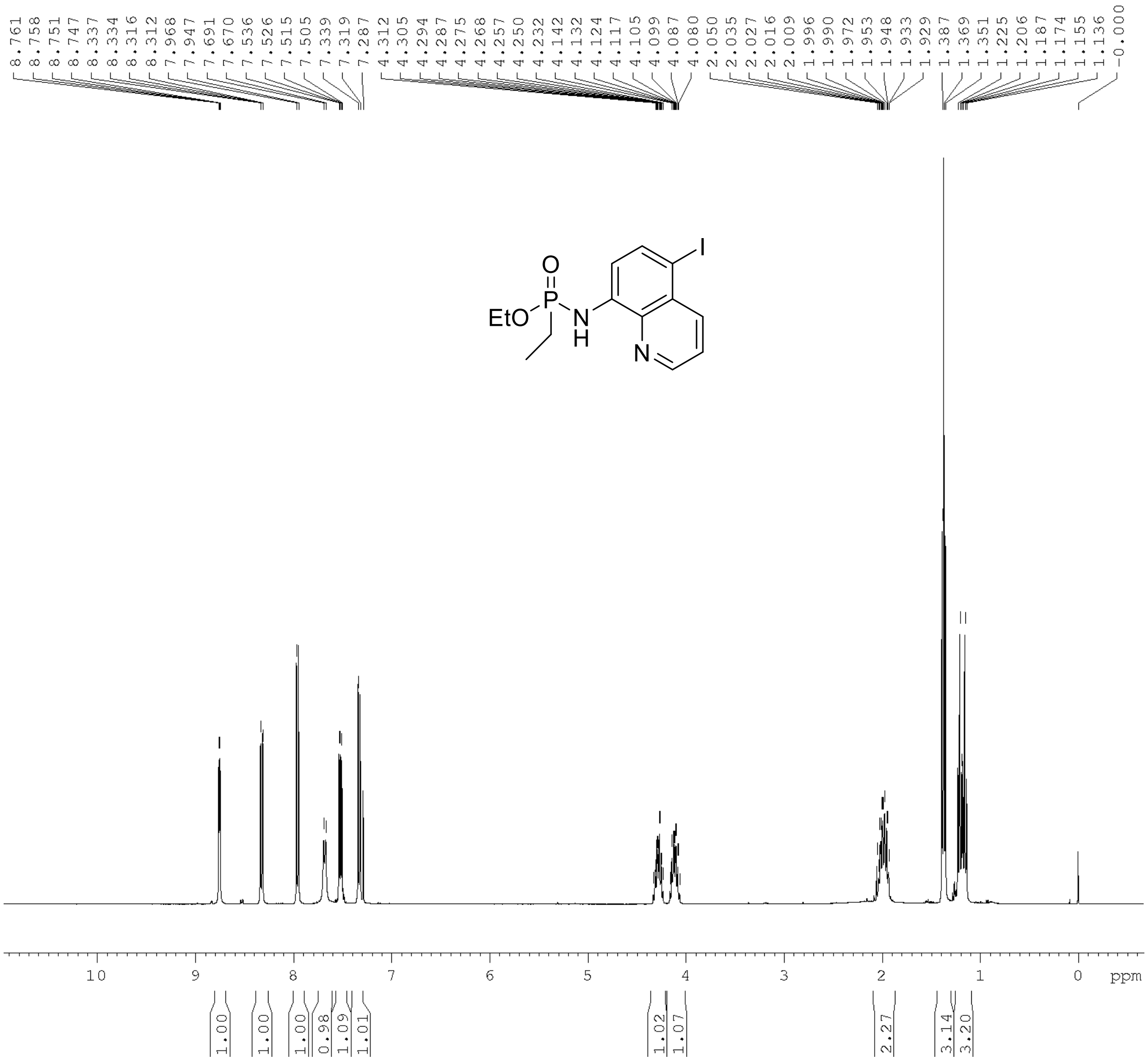

^{13}C{^{1}H}NMR spectrum of compound **10j** ($CDCl_3$, 100 MHz)**:**

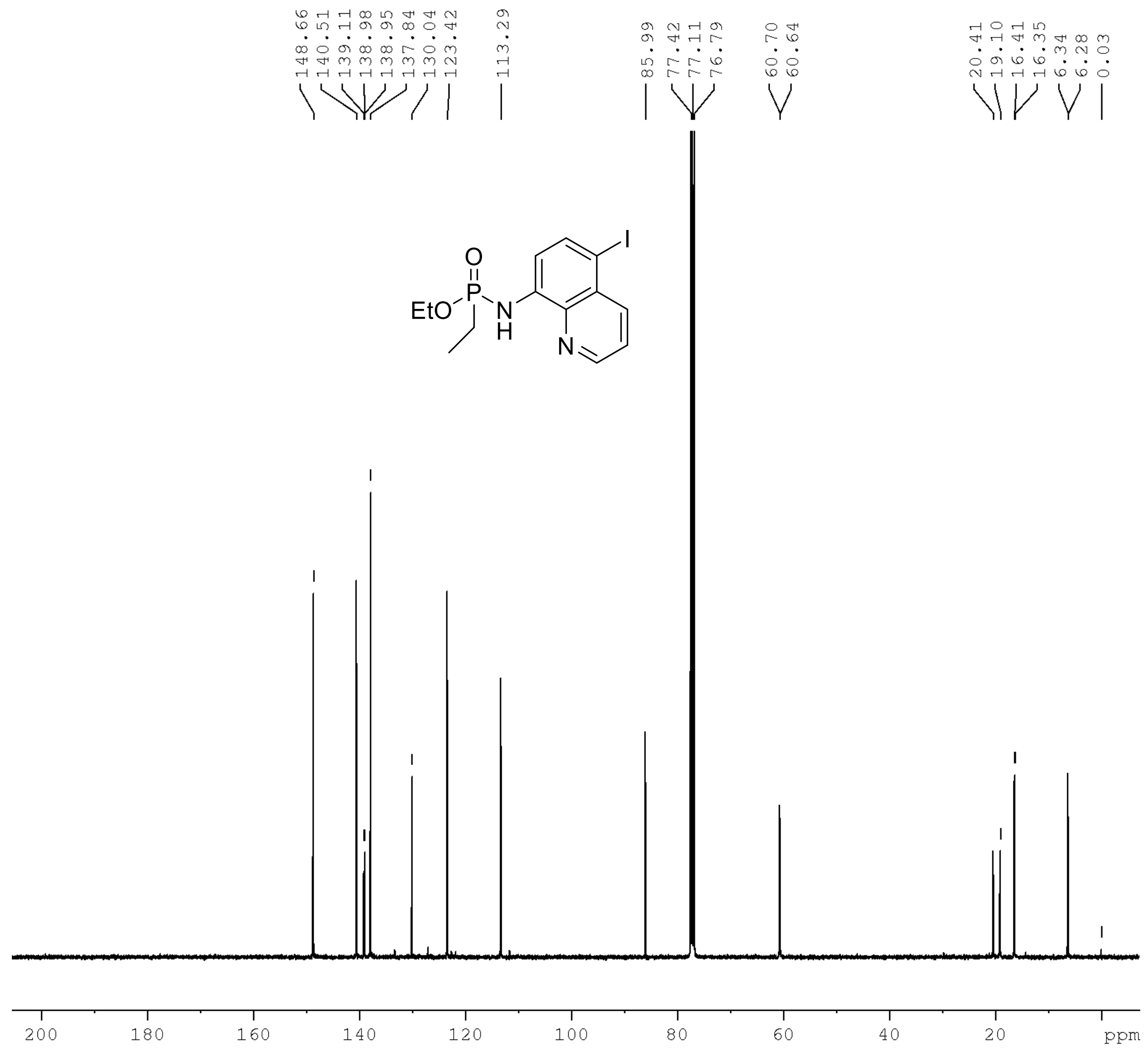

DEPT135 spectrum of compound **10j** ($CDCl_3$, 100 MHz)**:**

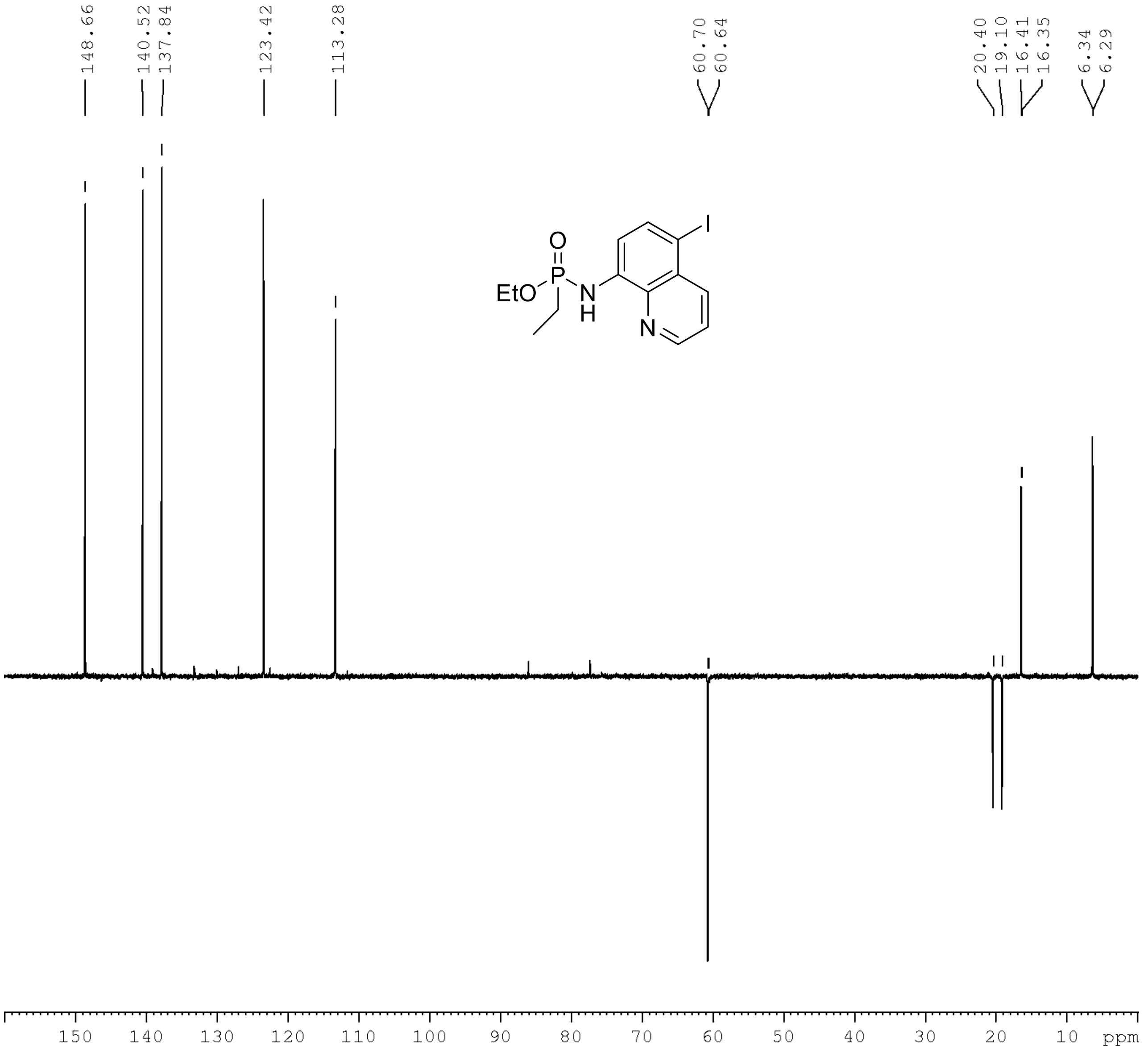

HRMS spectrum of compound **10j:**

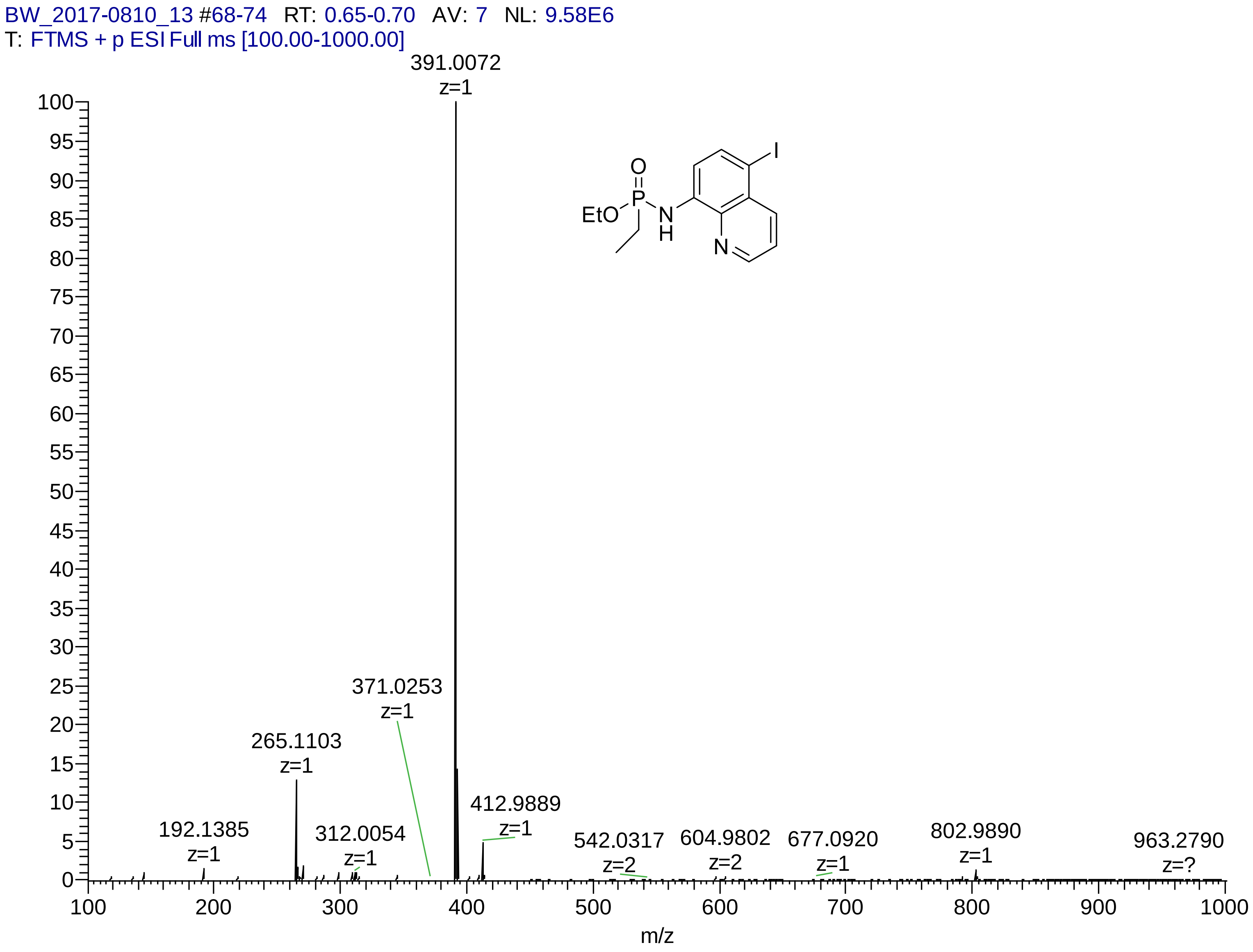

^{1}H NMR spectrum of compound **10k** ($CDCl_3$, 400 MHz)**:**

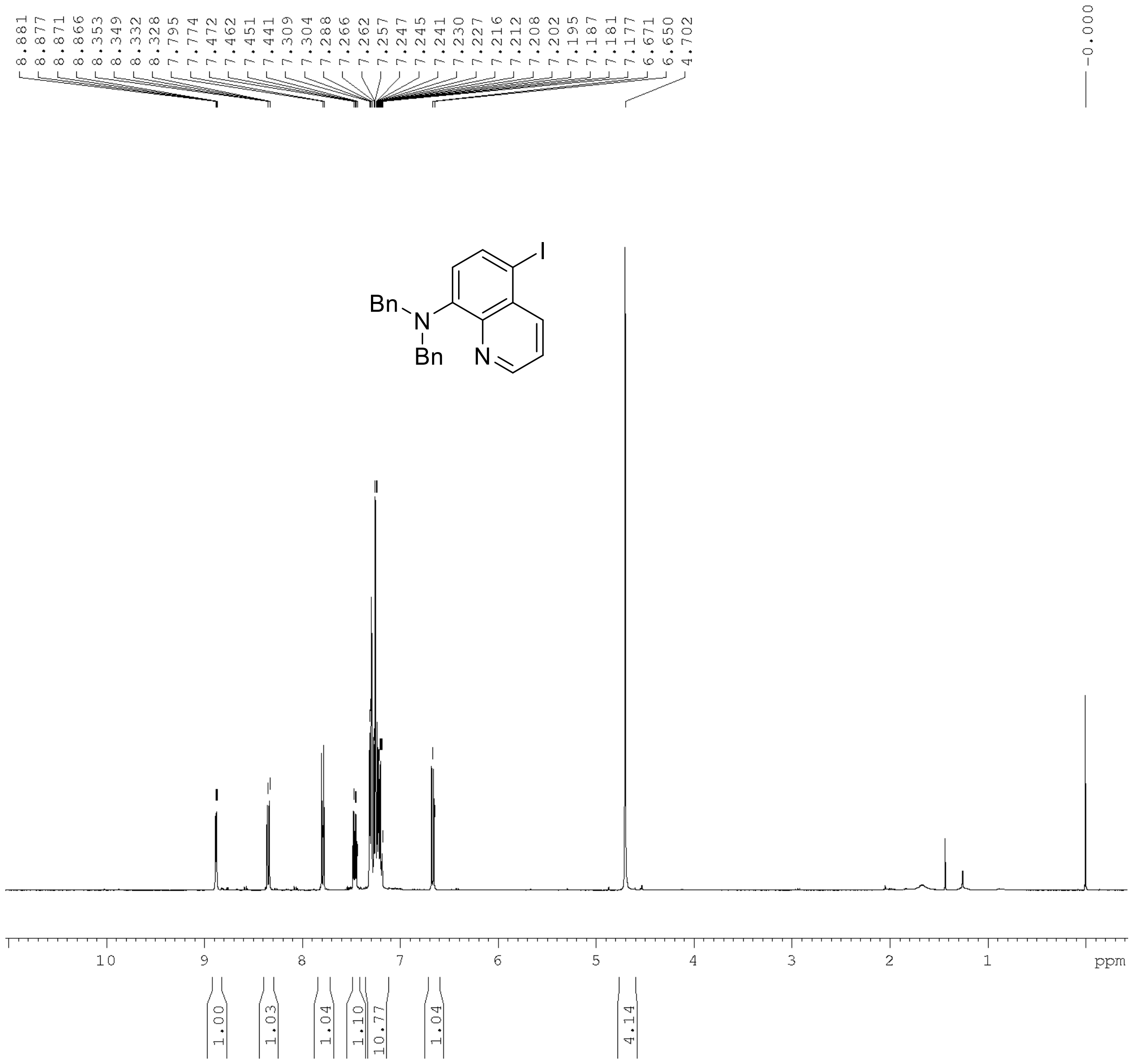

$^{13}C\{^{1}H\}$NMR spectrum of compound **10k** ($CDCl_3$, 100 MHz)**:**

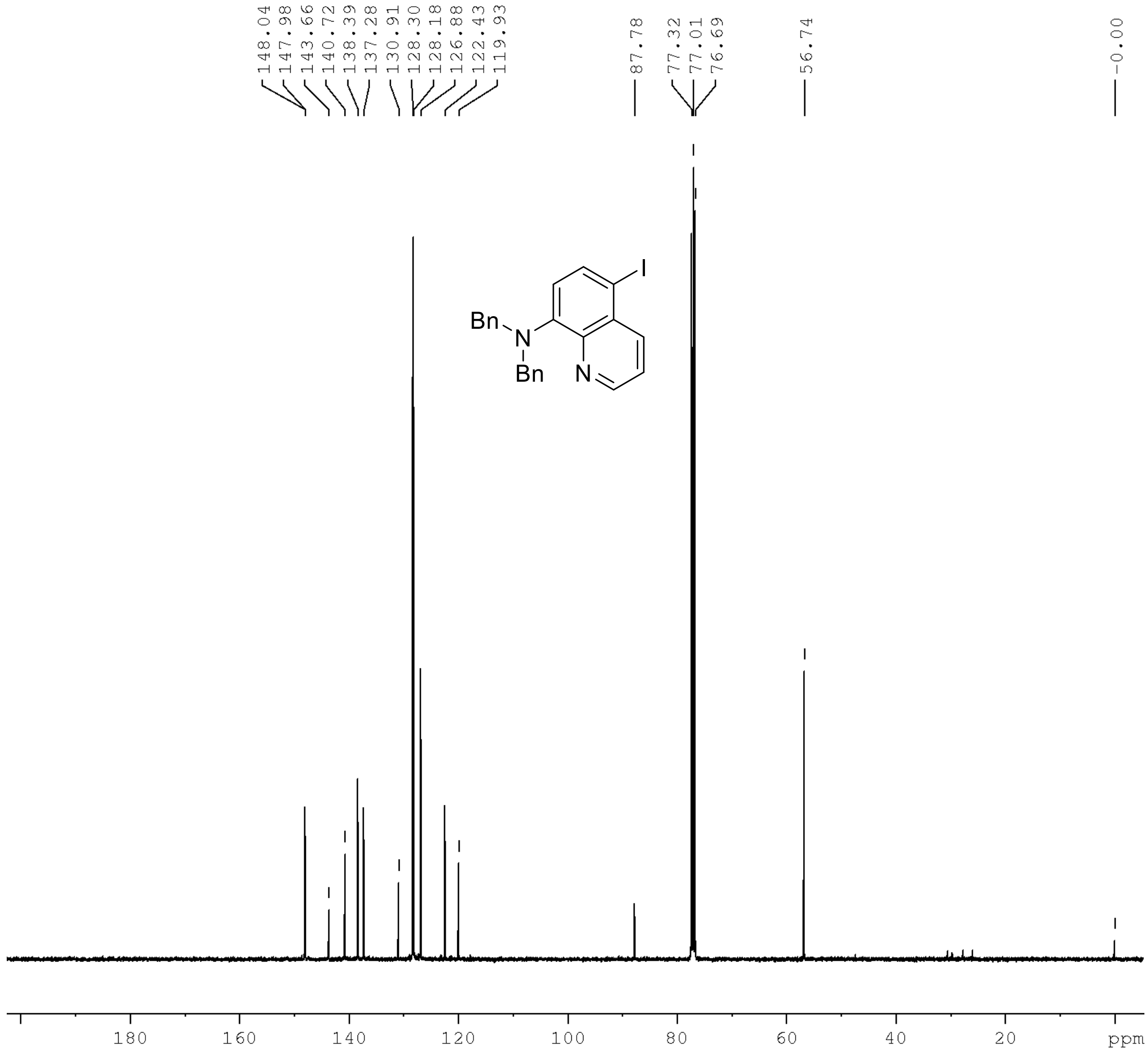

DEPT135 spectrum of compound **10k** ($CDCl_3$, 100 MHz)**:**

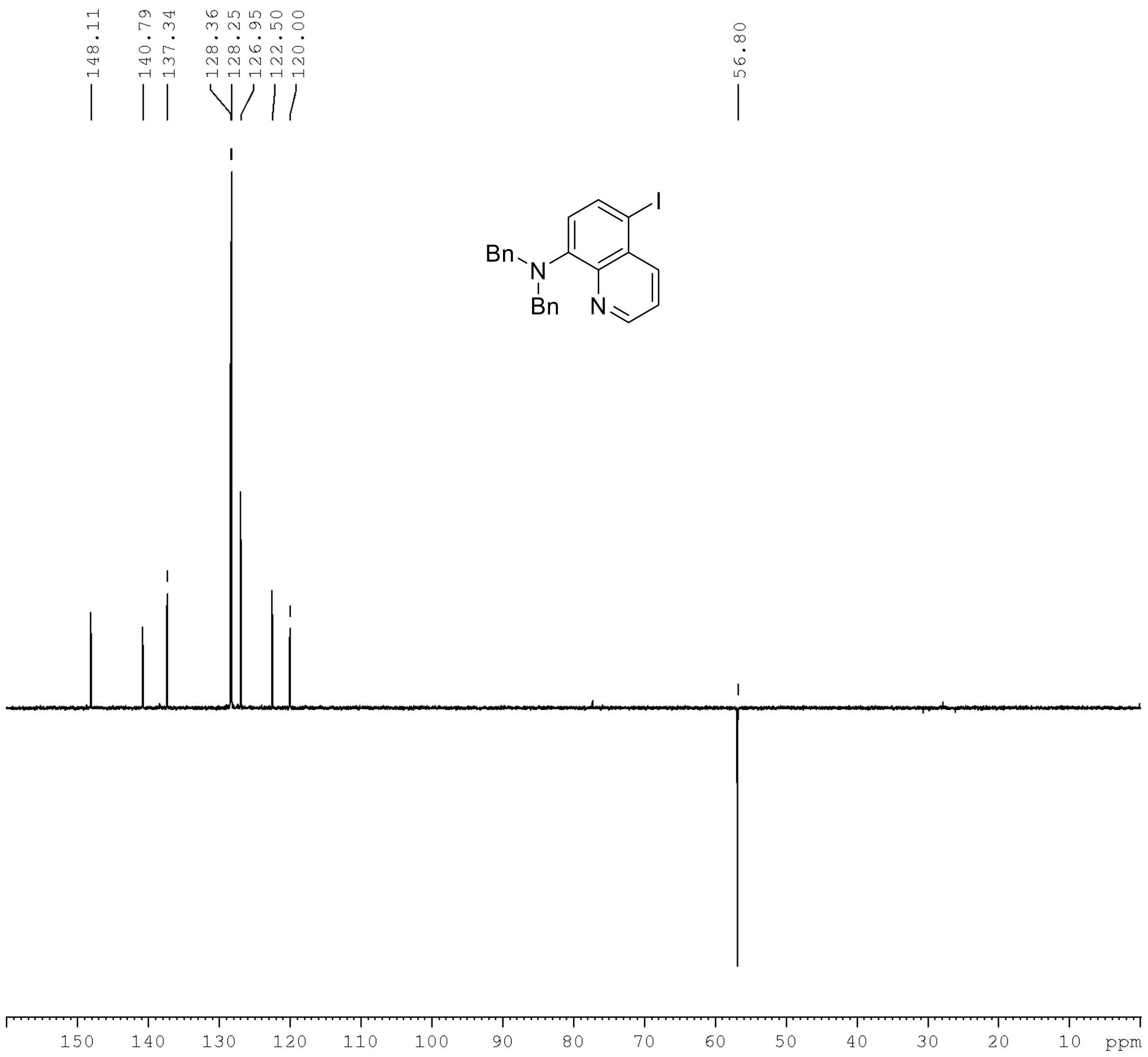

HRMS spectrum of compound **10k:**

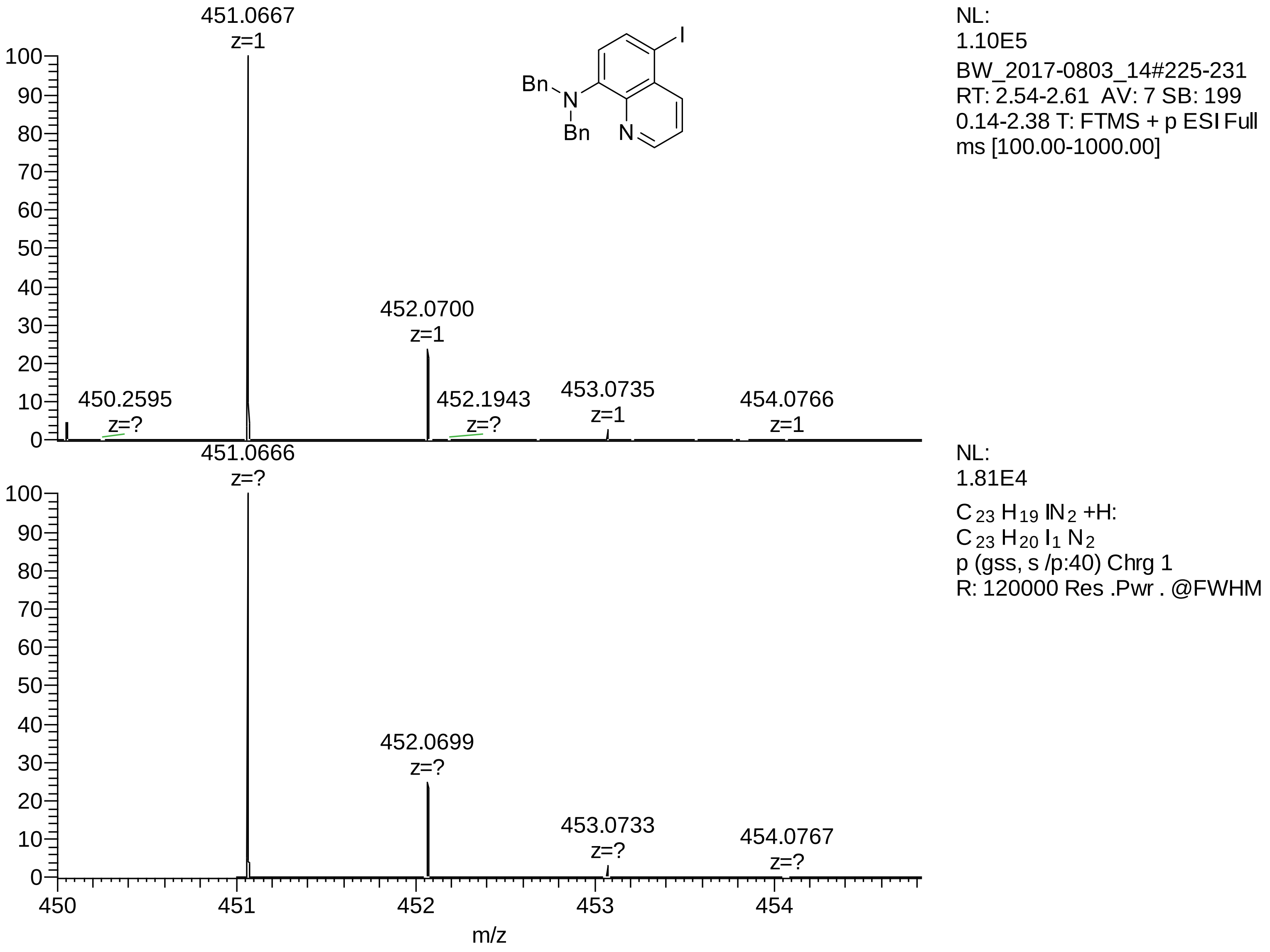

^{1}H NMR spectrum of compound **10l** ($CDCl_3$, 400 MHz):

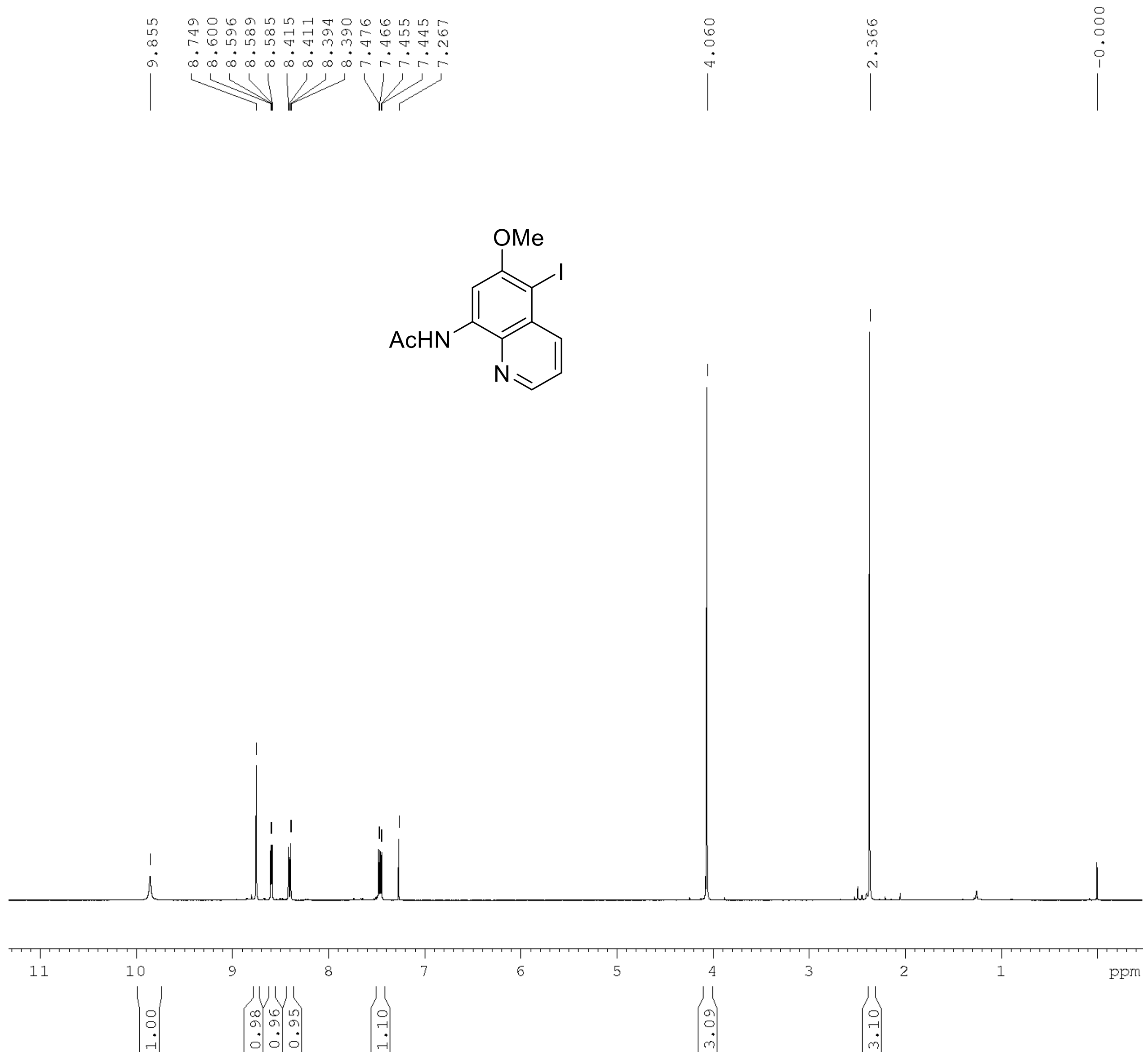

$^{13}C\{^1H\}$NMR spectrum of compound **10l** ($CDCl_3$, 100 MHz):

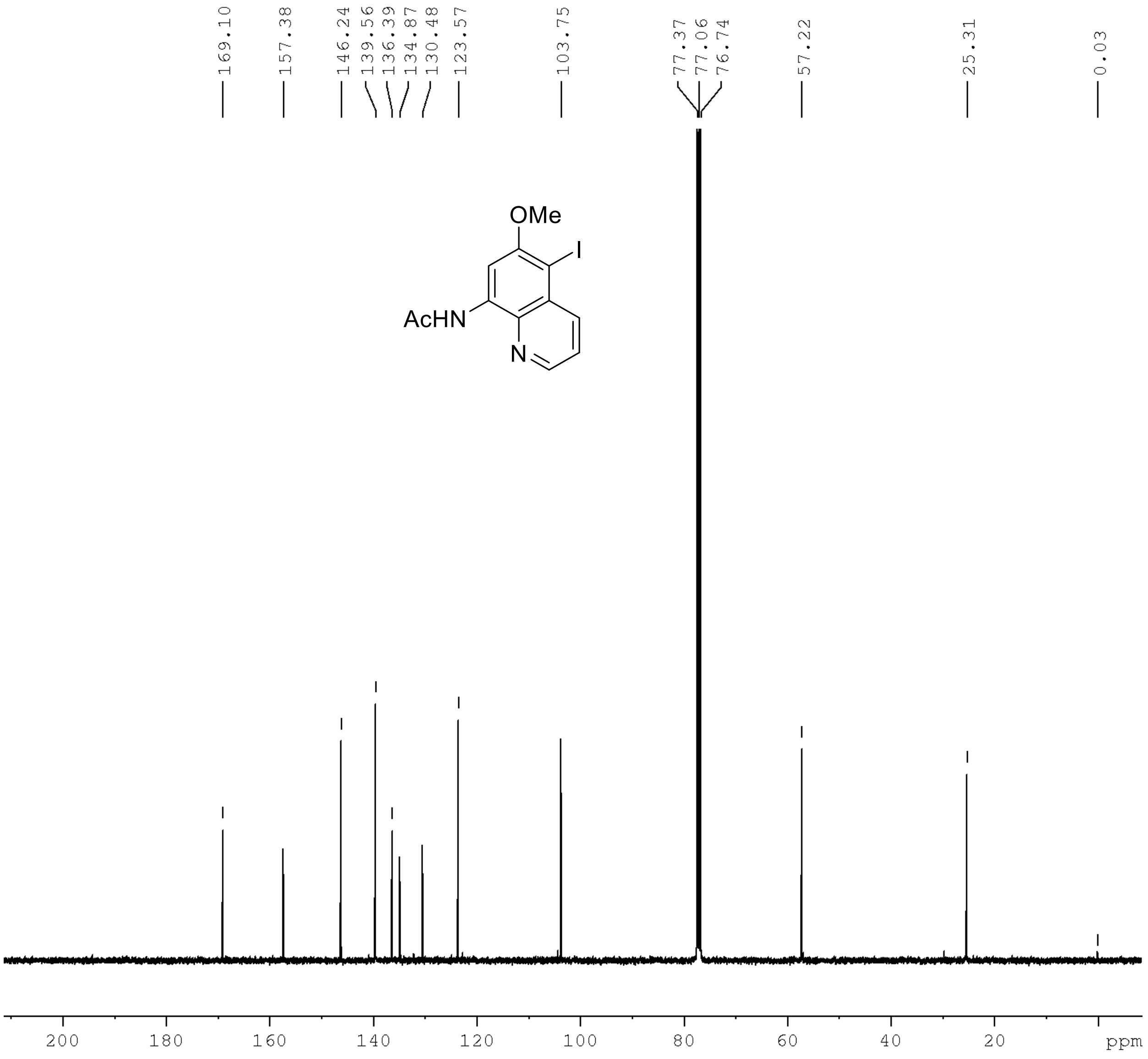

DEPT135 NMR spectrum of compound **10l** ($CDCl_3$, 100 MHz):

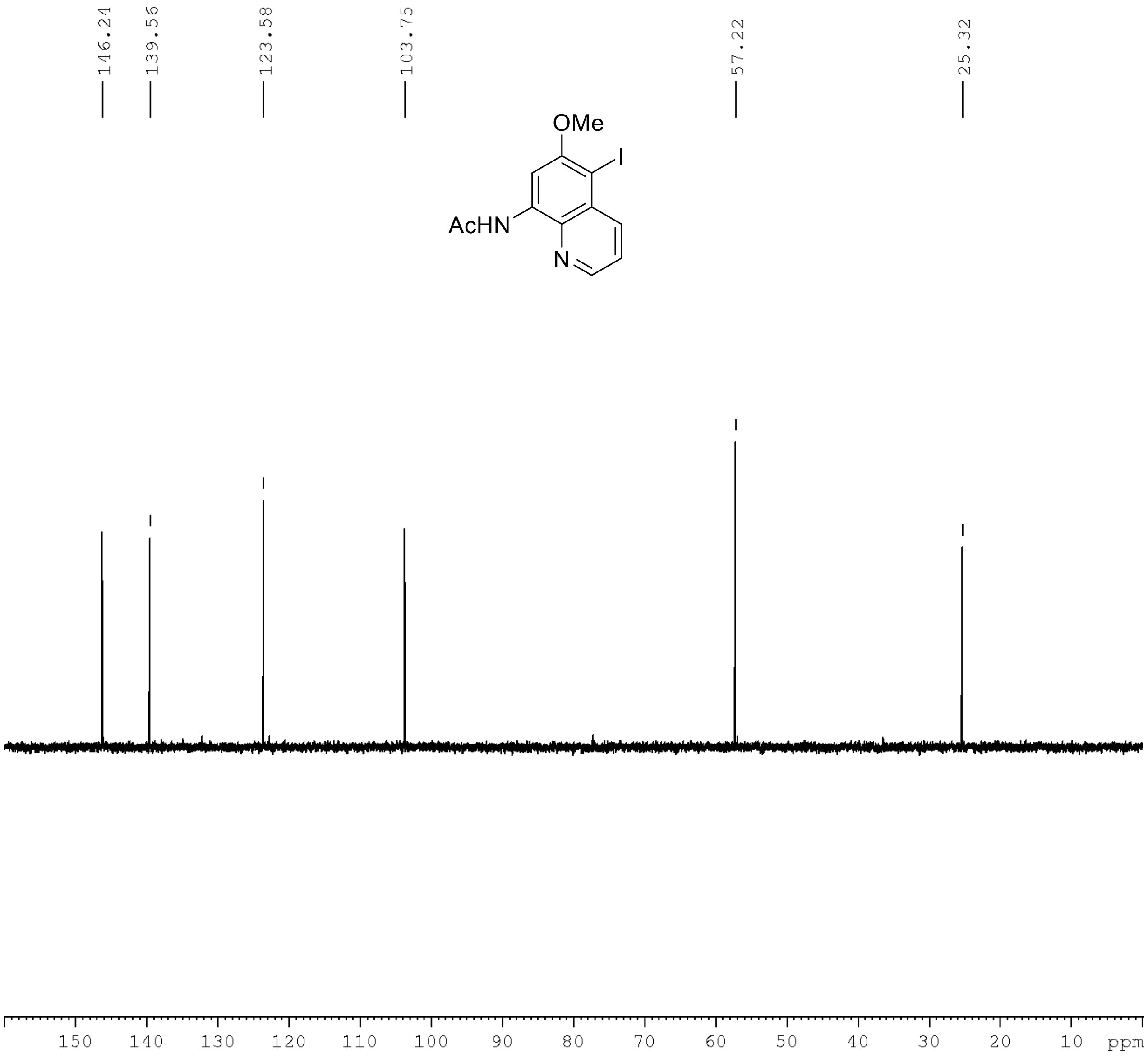

^{1}H NMR spectrum of compound **10m** ($CDCl_3$, 400 MHz):

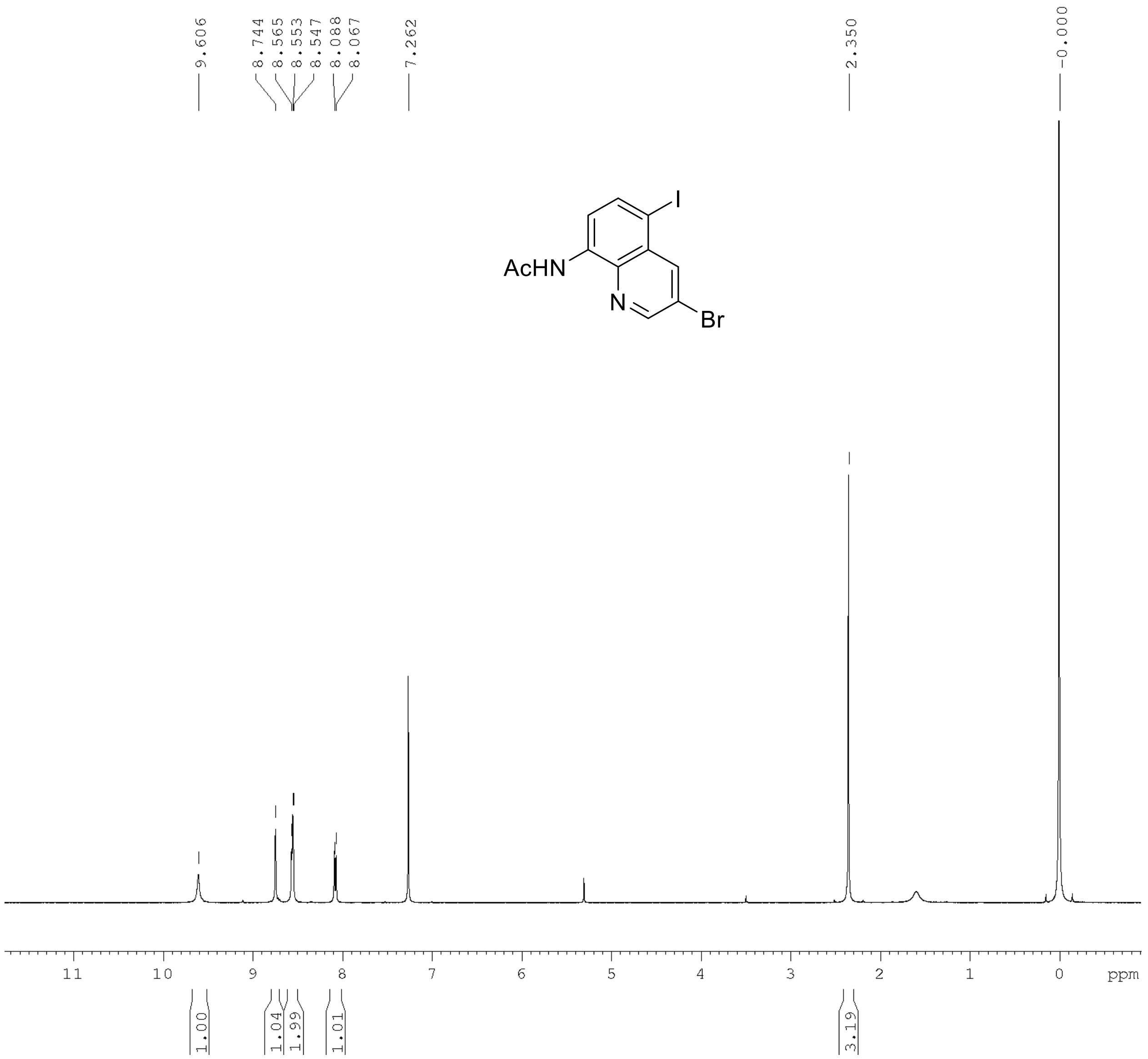

$^{13}C\{^{1}H\}$NMR spectrum of compound **10m** ($CDCl_3$, 100 MHz):

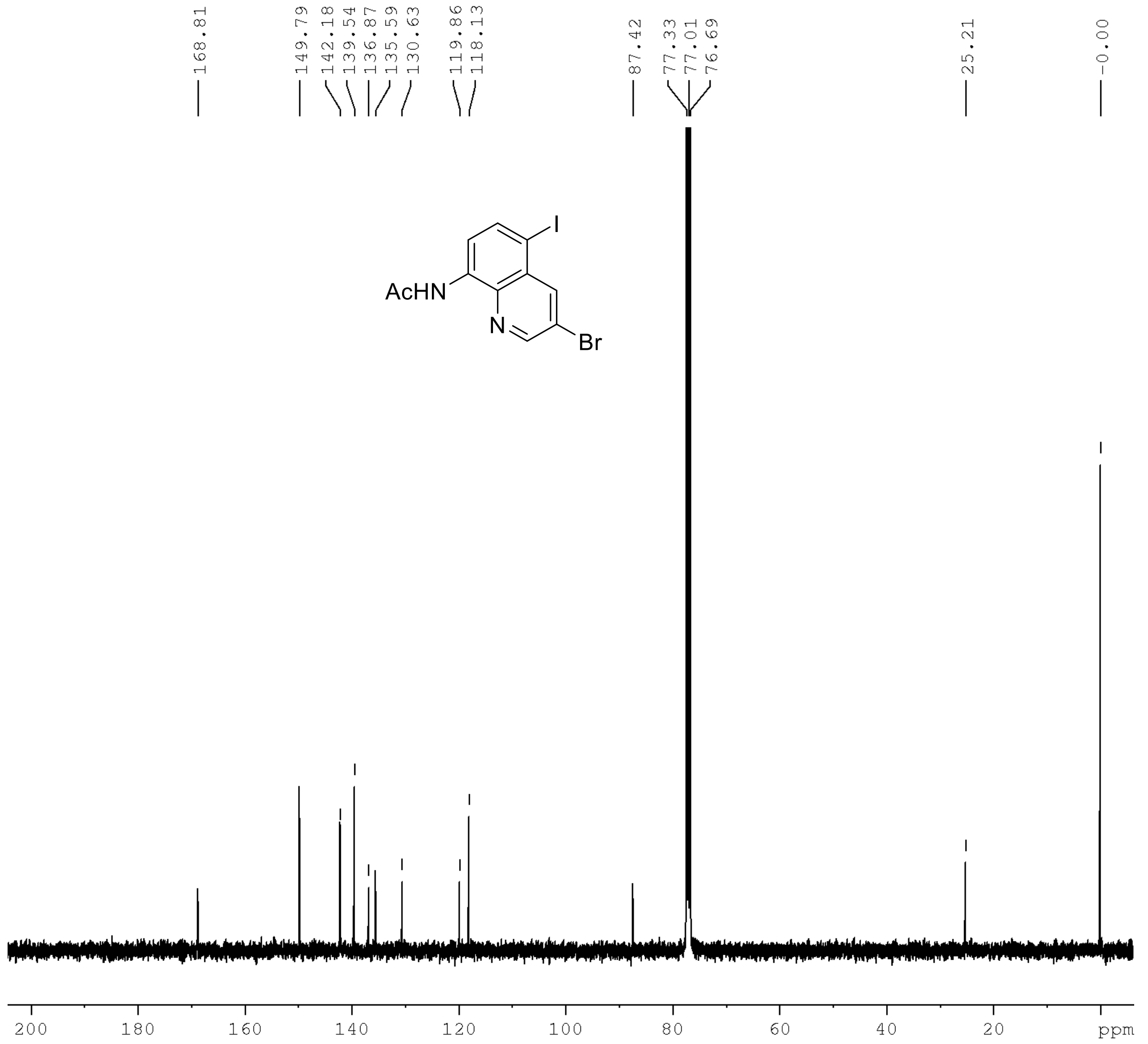

DEPT135 NMR spectrum of compound **10m** ($CDCl_3$, 100 MHz):

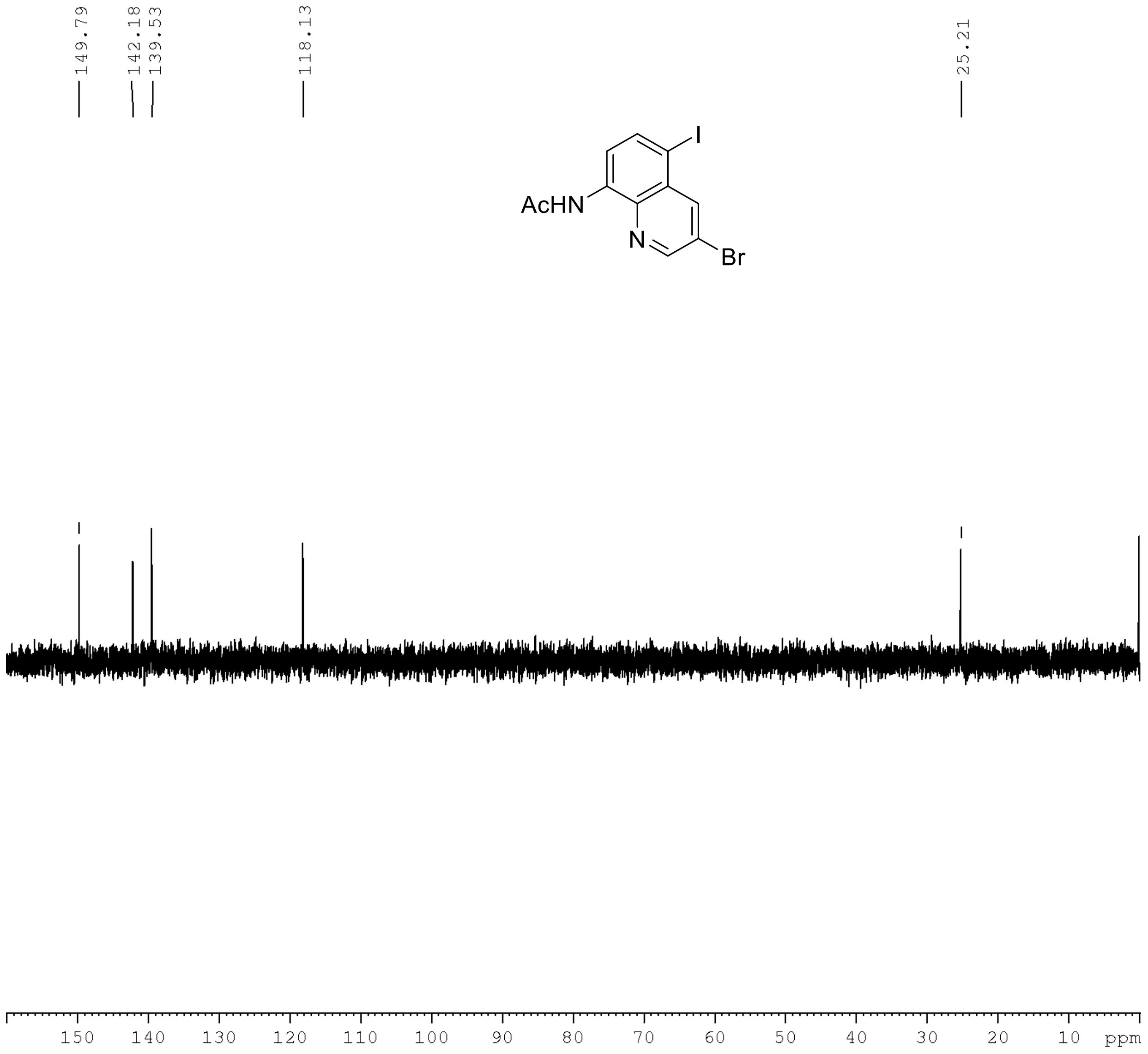

^{1}H NMR spectrum of compound **10n** ($CDCl_3$, 400 MHz)**:**

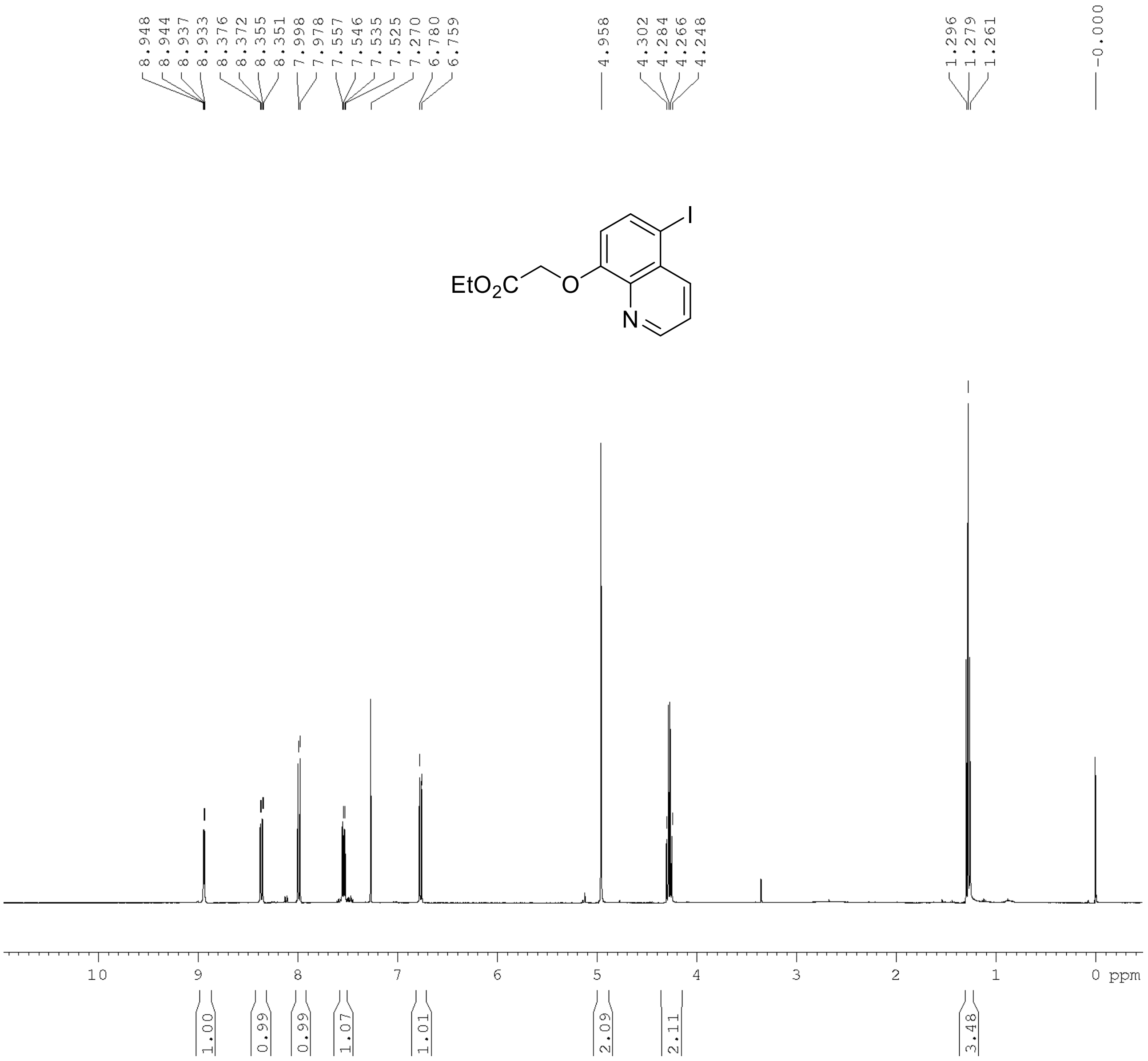

$^{13}C\{^1H\}$NMR spectrum of compound **10n** ($CDCl_3$, 100 MHz)**:**

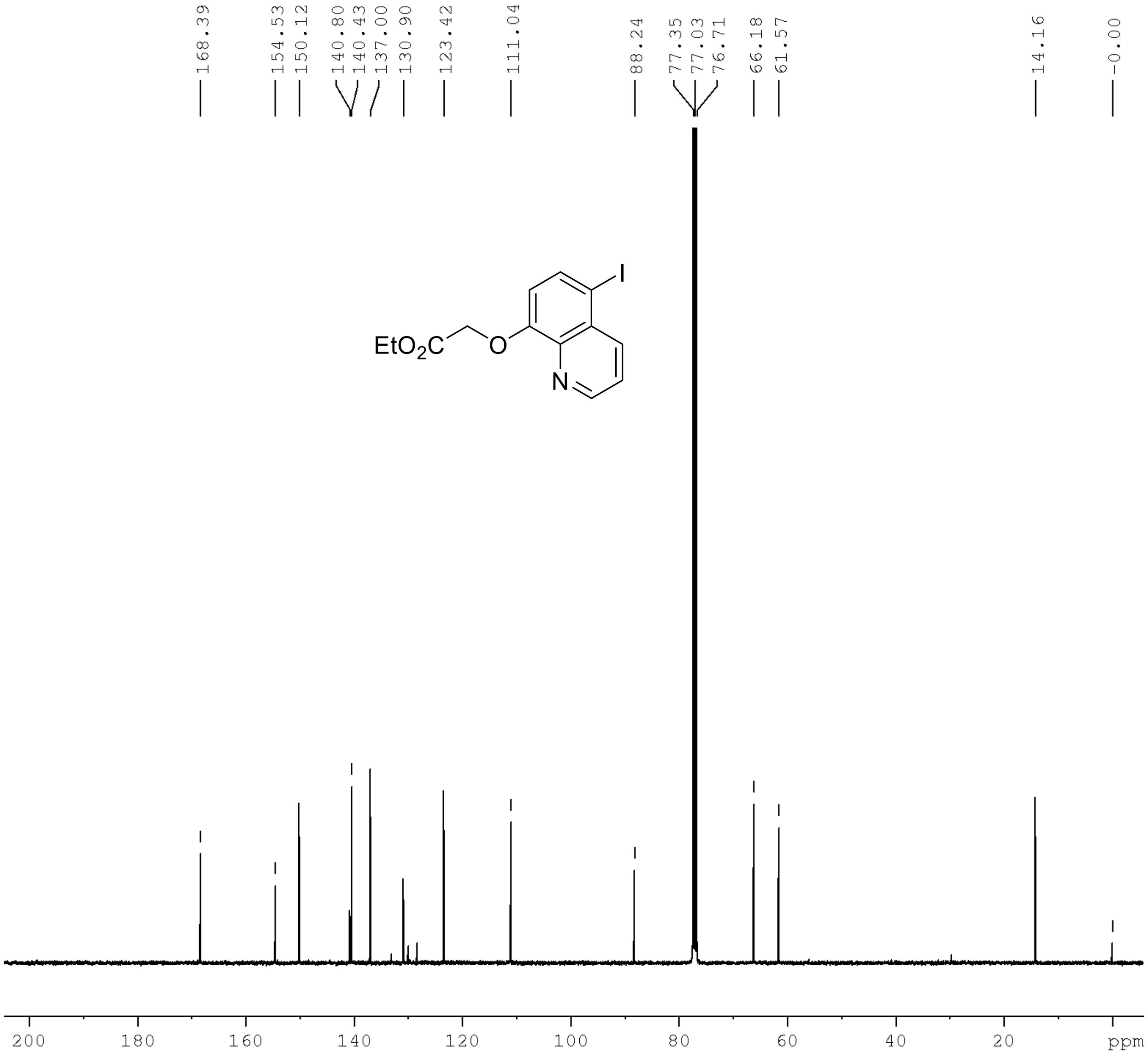

DEPT135 spectrum of compound **10n** ($CDCl_3$, 100 MHz)**:**

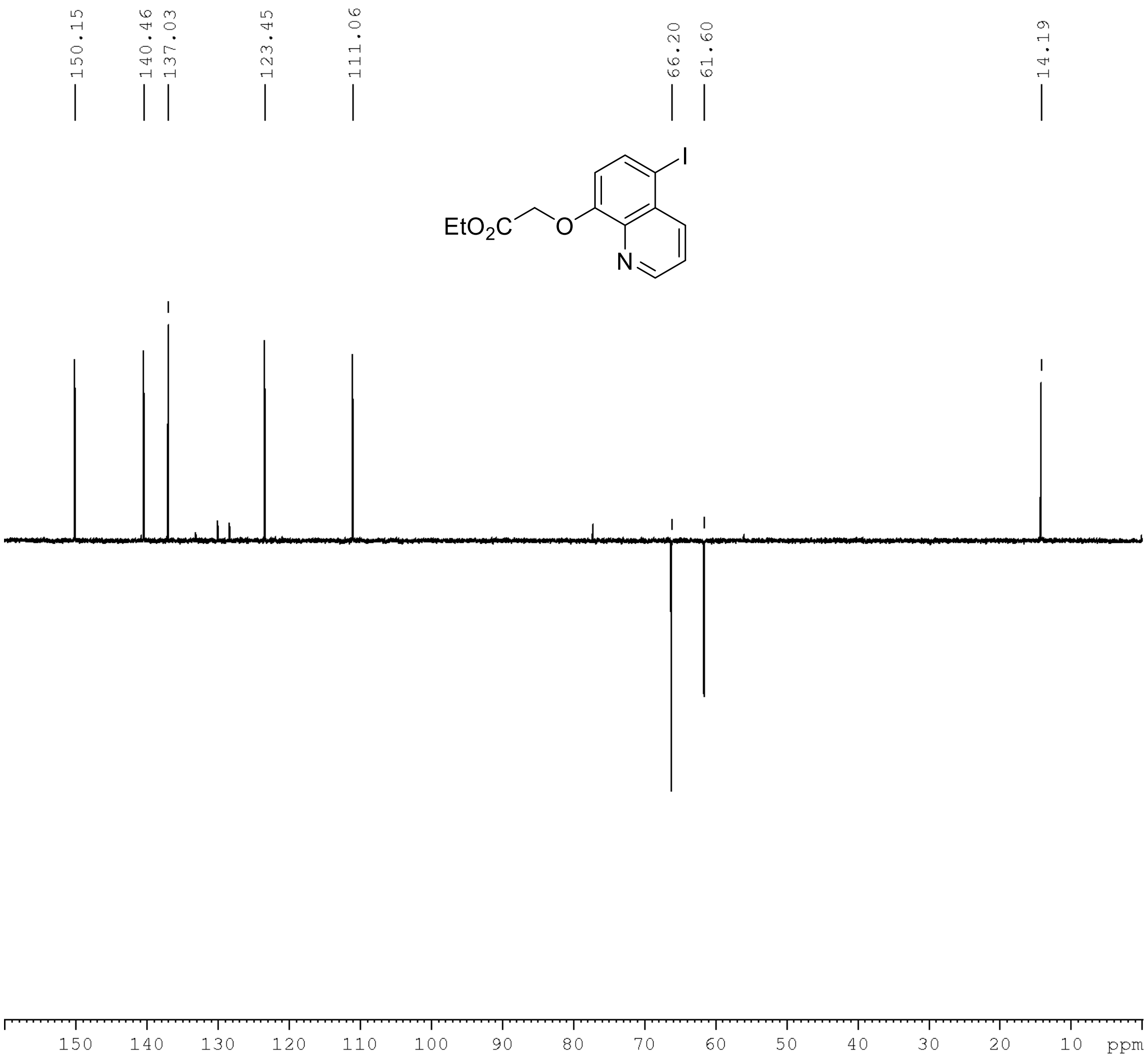

HRMS spectrum of compound **10n:**

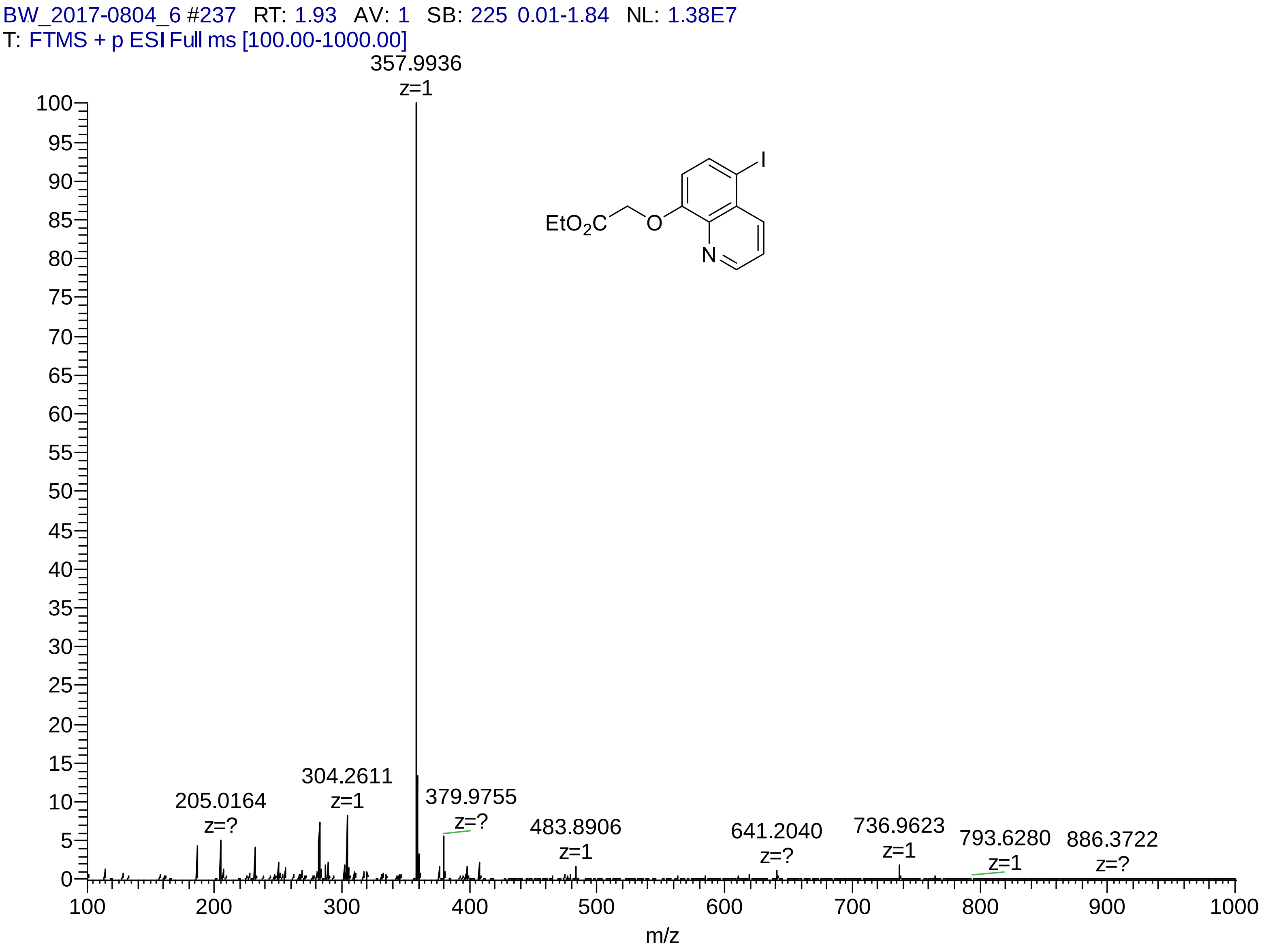

^{1}H NMR spectrum of compound **14** ($CDCl_3$, 400 MHz)**:**

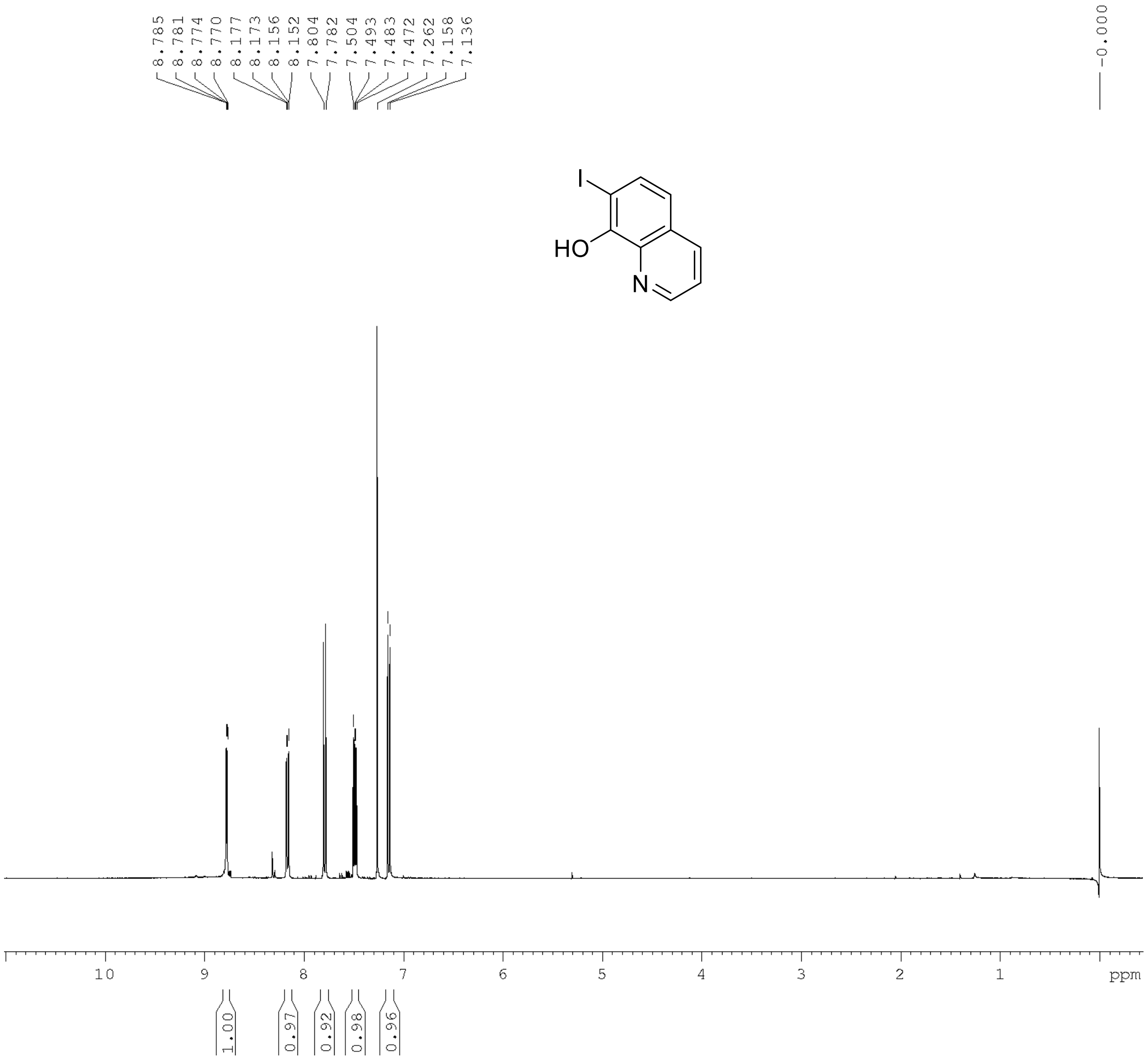

$^{13}C\{^1H\}$NMR spectrum of compound **14** ($CDCl_3$, 100 MHz)**:**

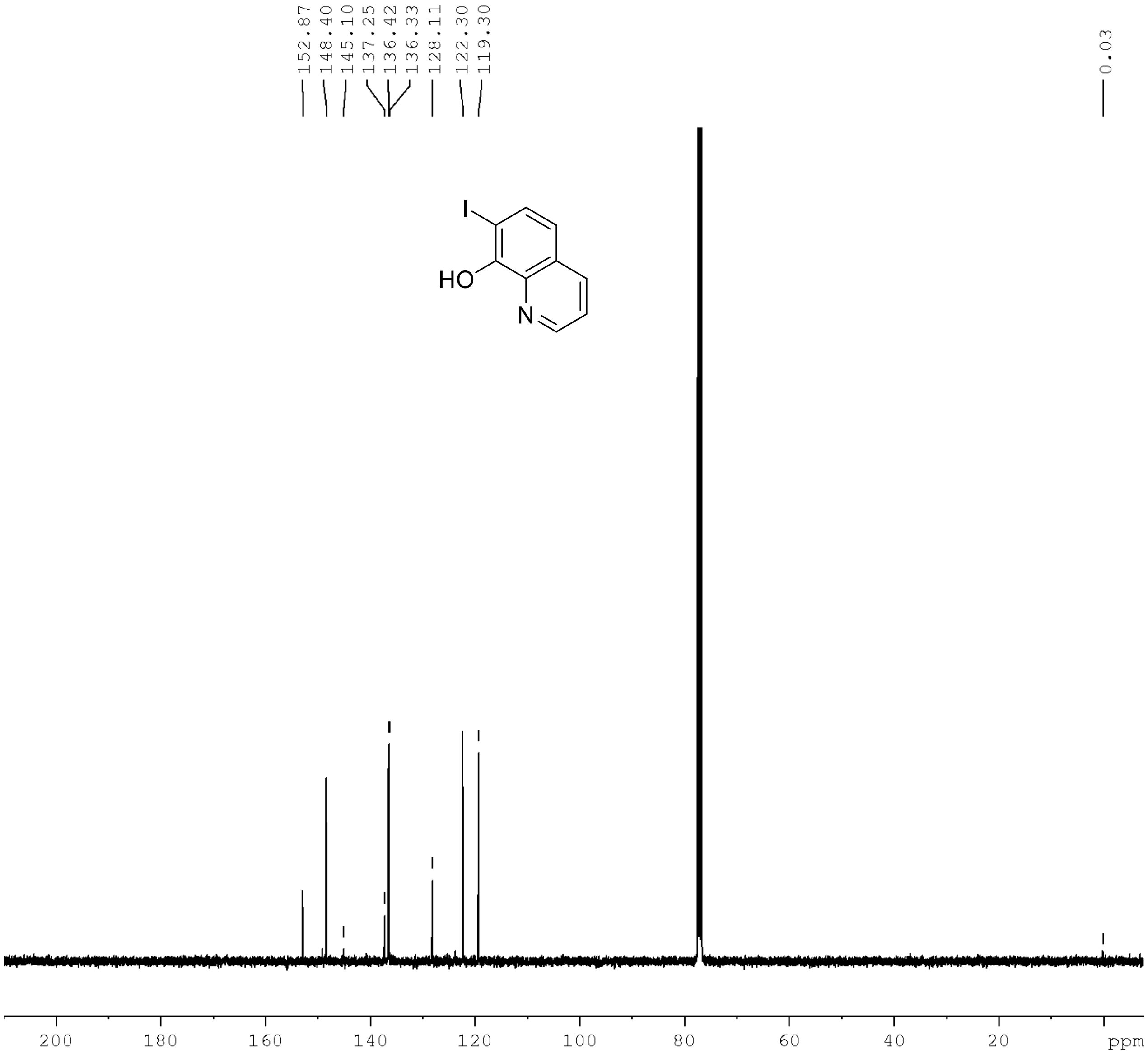

DEPT135 spectrum of compound **14** ($CDCl_3$, 100 MHz)**:**

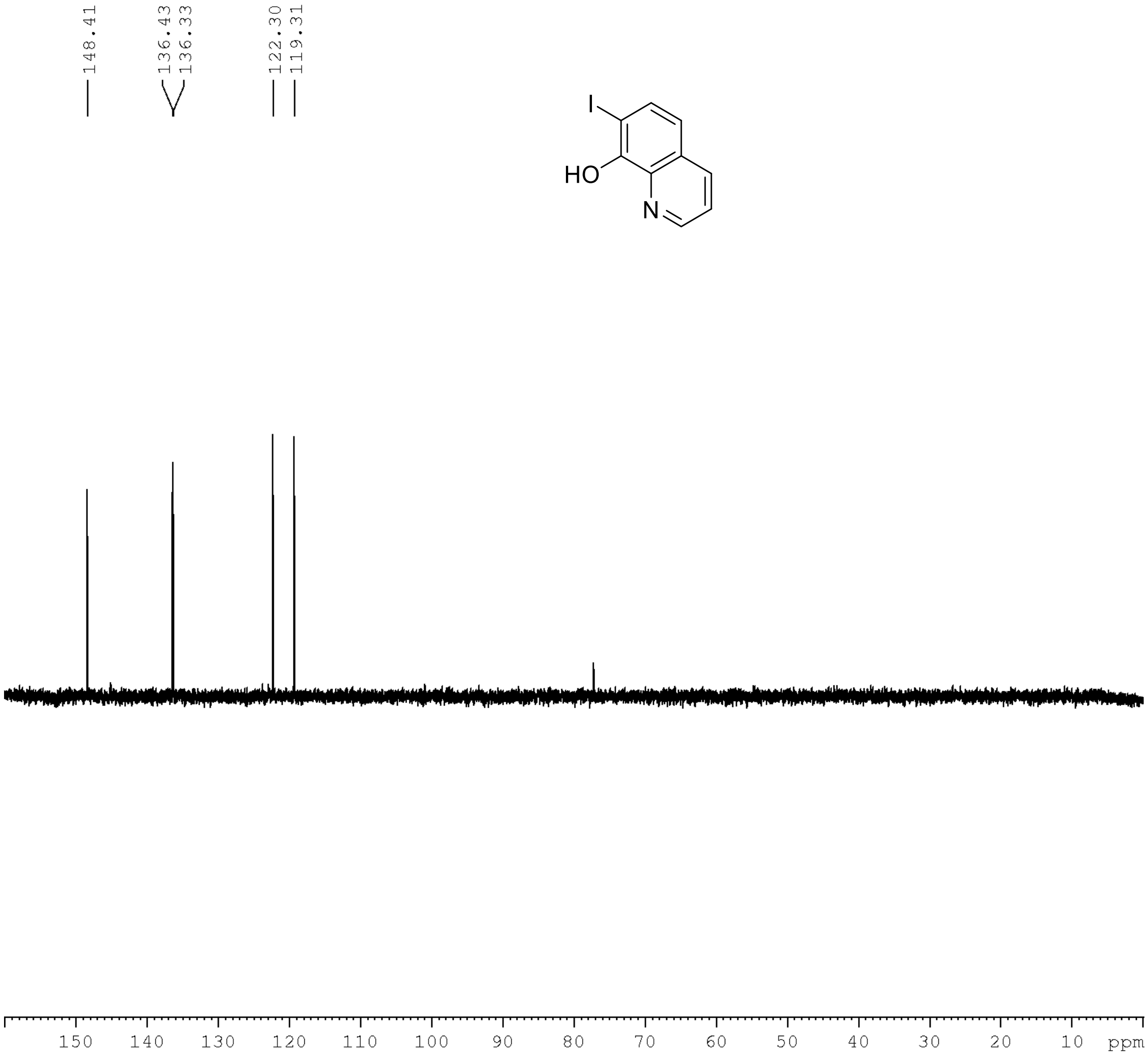

^{1}H NMR spectrum of compound **G** ($CDCl_3$, 400 MHz)**:**

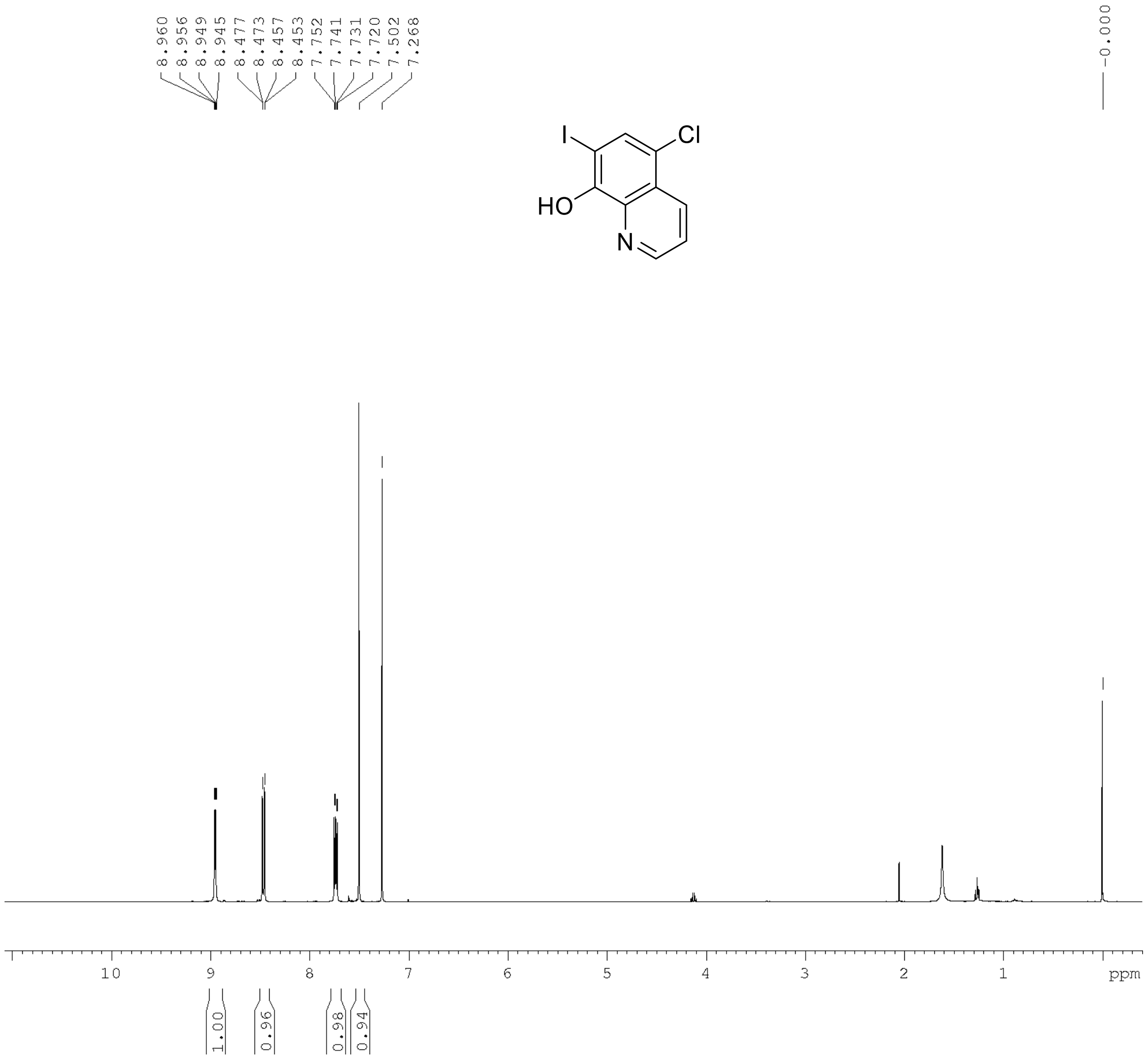

$^{13}C\{^{1}H\}$NMR spectrum of compound **G** ($CDCl_3$, 100 MHz)**:**

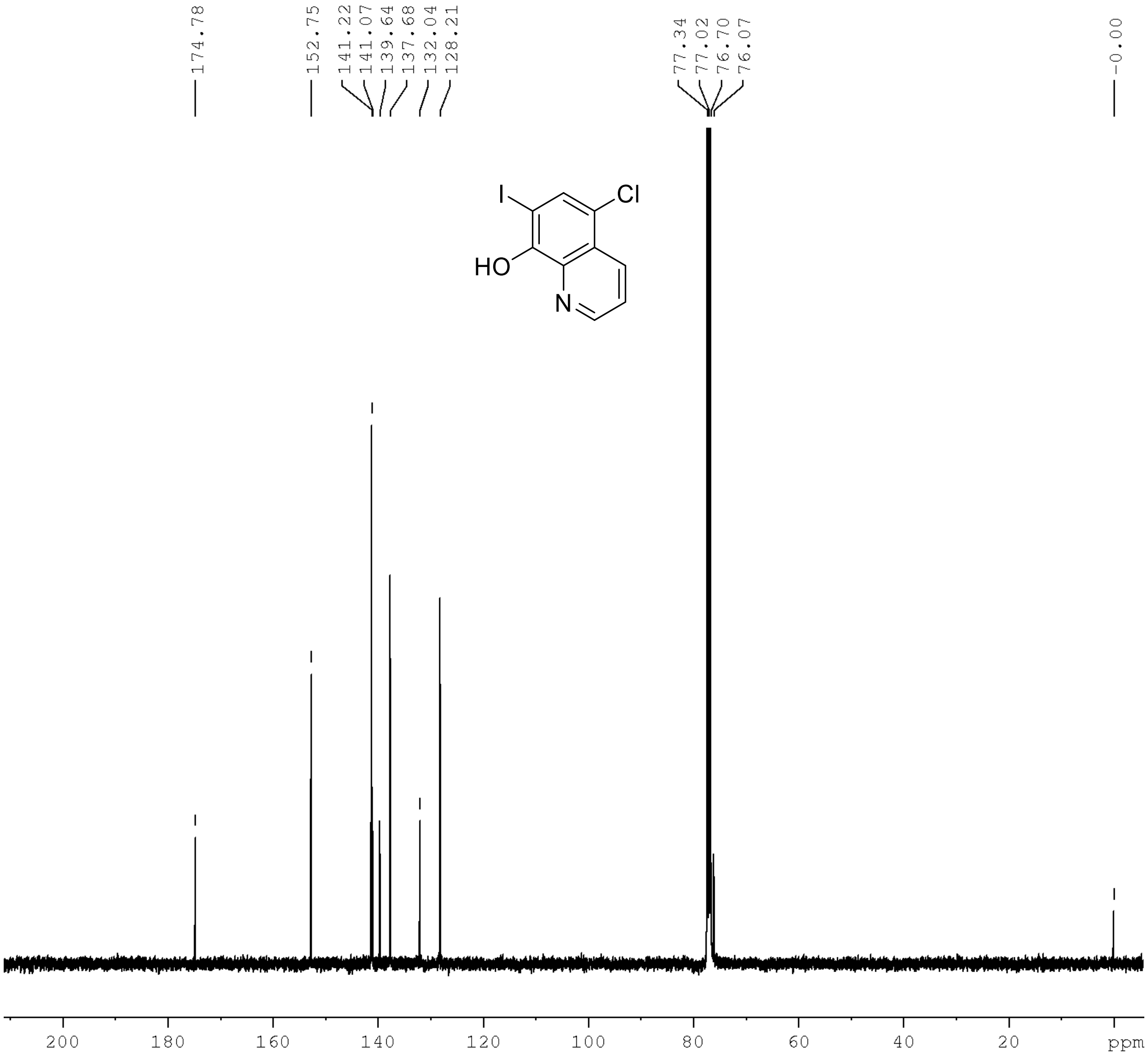

DEPT135 spectrum of compound **G** ($CDCl_3$, 100 MHz)**:**

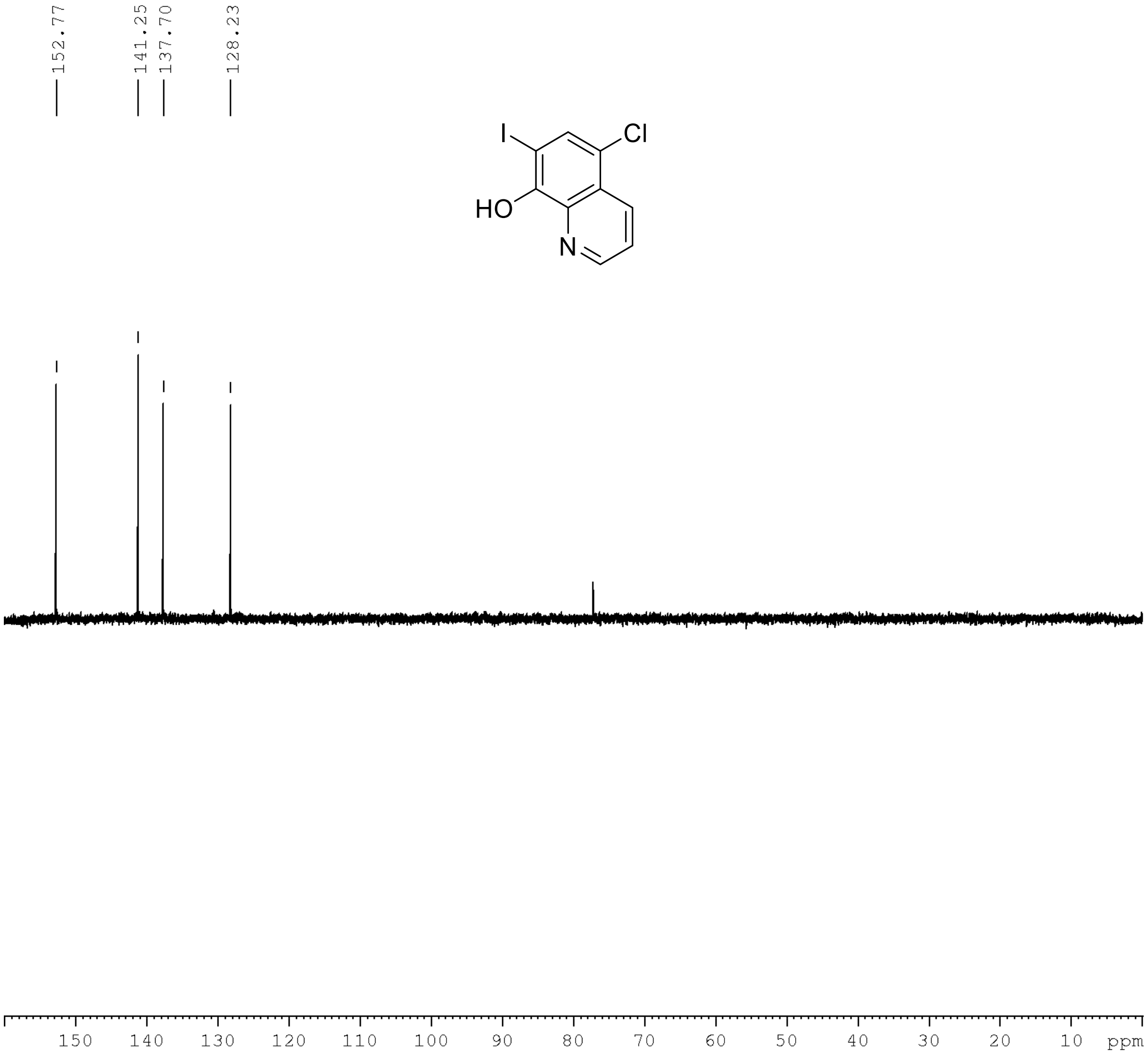

^{1}H NMR spectrum of compound **12** ($CDCl_3$, 400 MHz)**:**

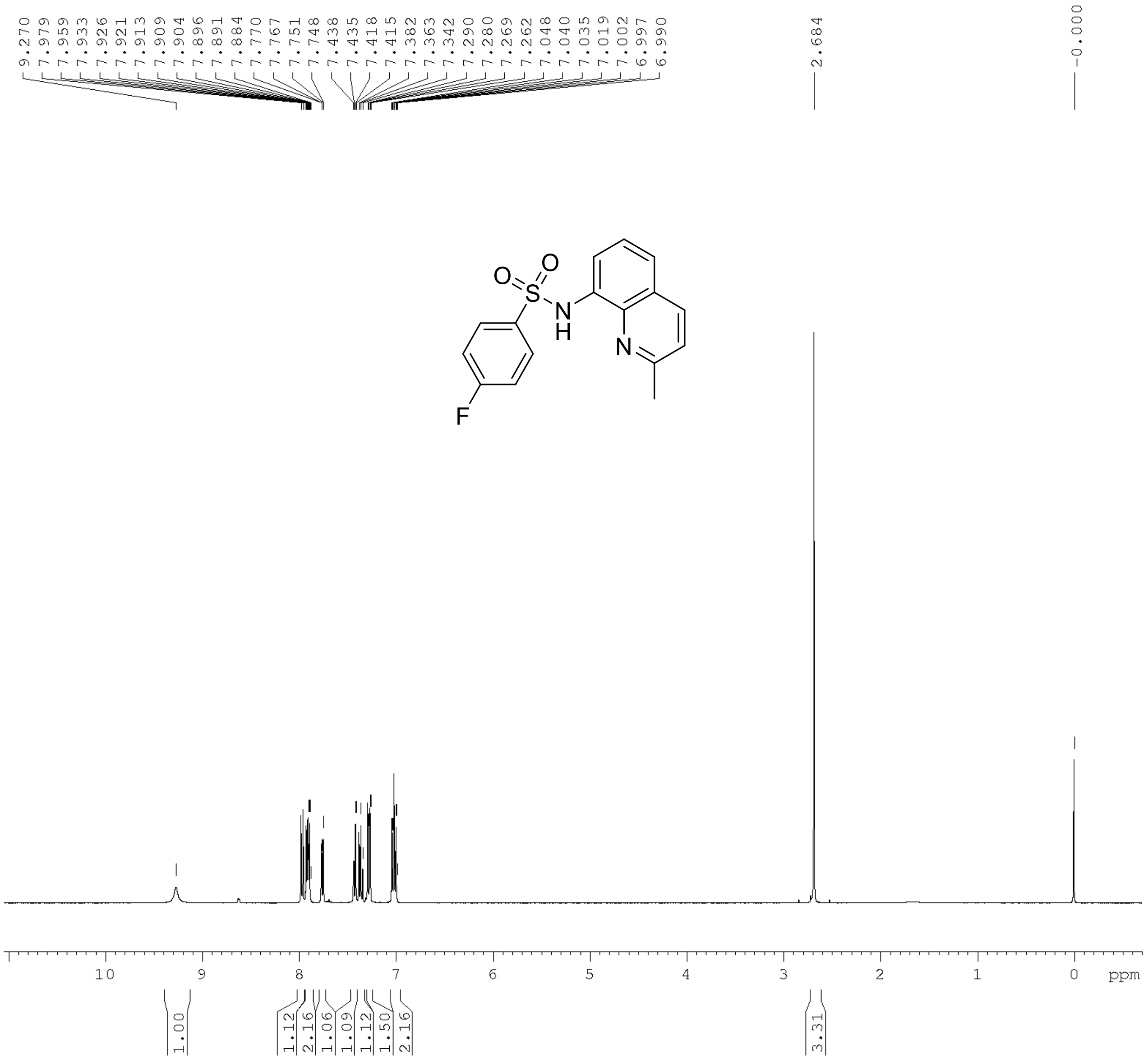

$^{13}C\{^{1}H\}$NMR spectrum of compound **12** ($CDCl_3$, 100 MHz)**:**

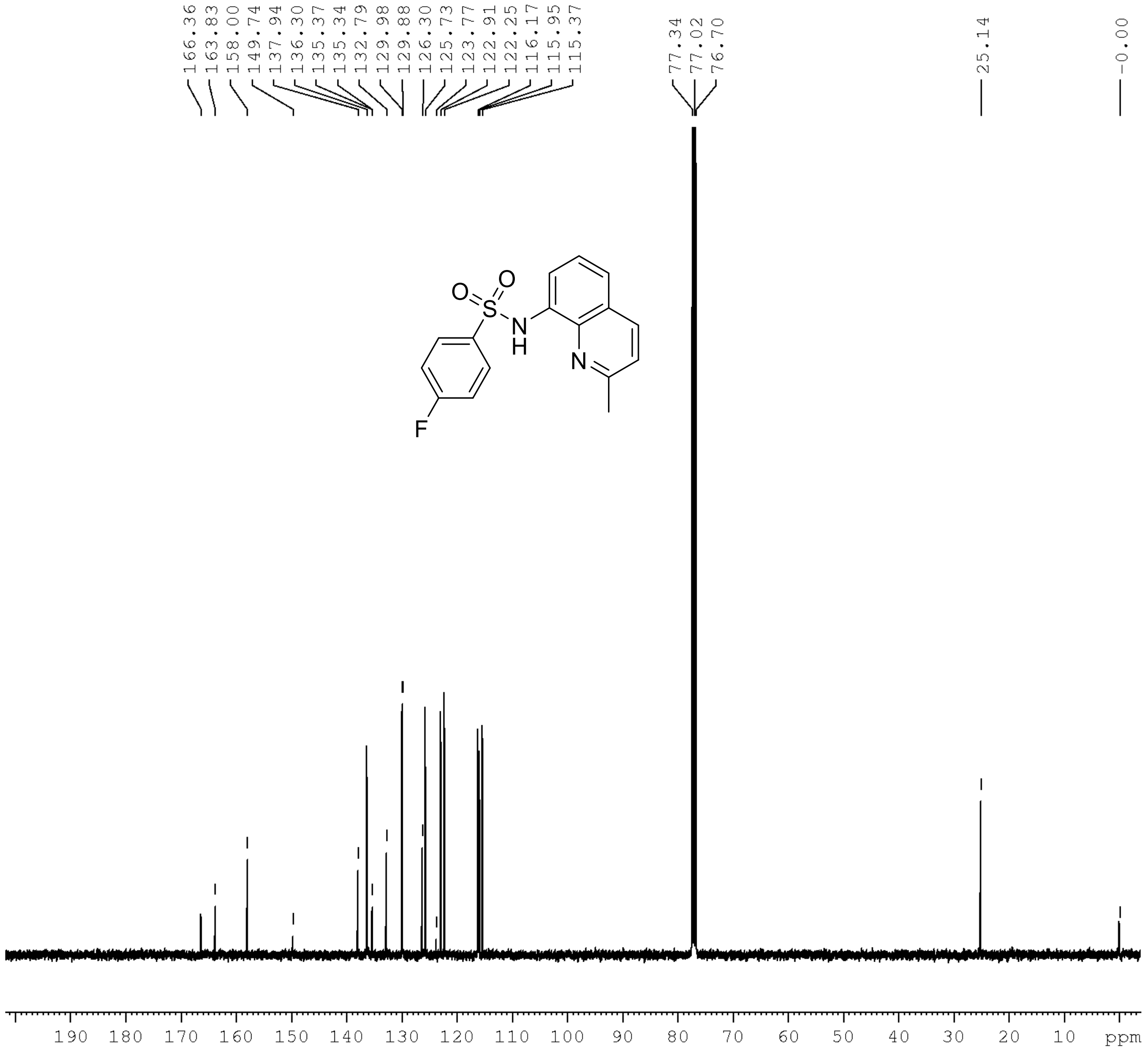

DEPT135 spectrum of compound **12** ($CDCl_3$, 100 MHz)**:**

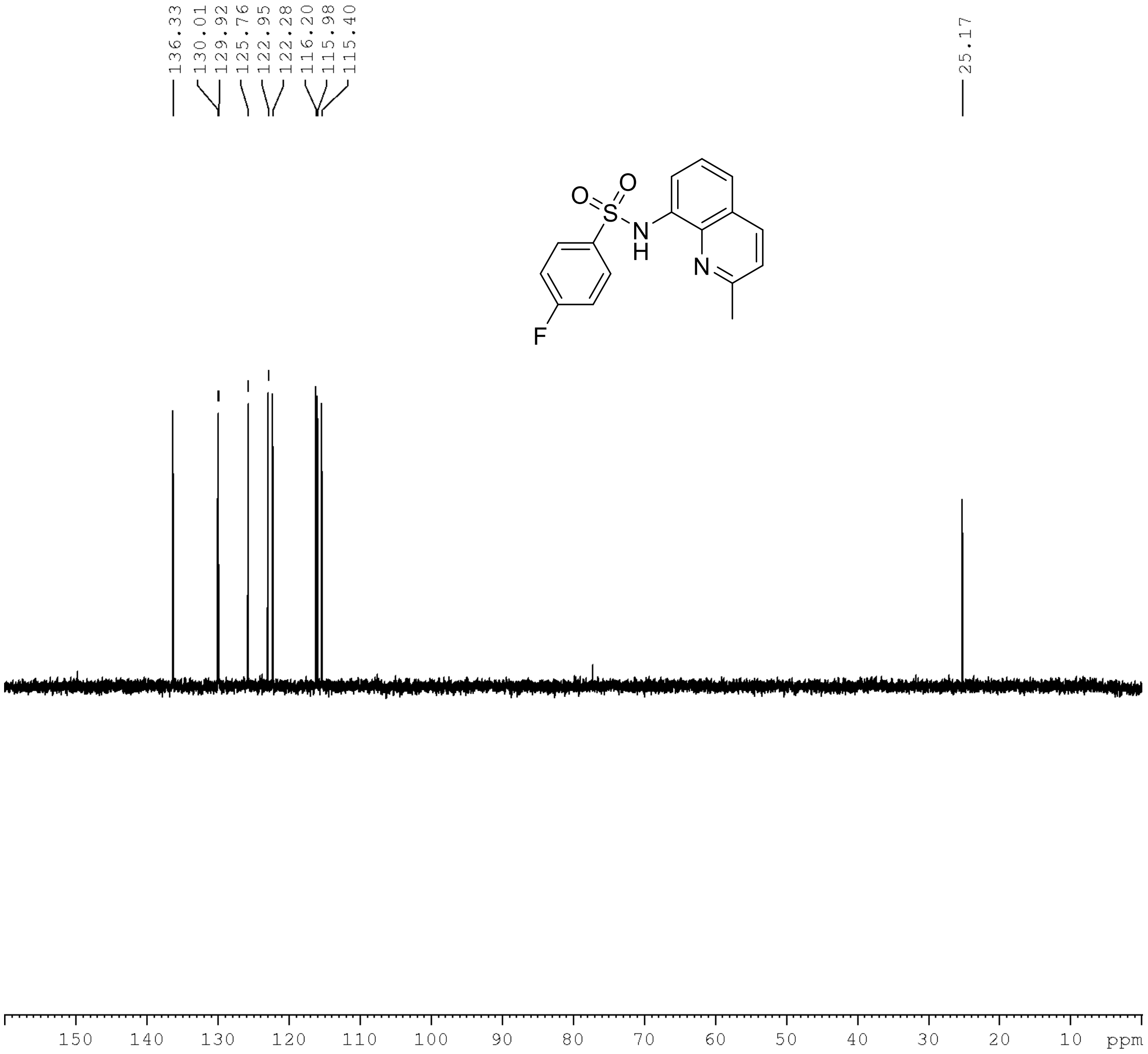

^{1}H NMR spectrum of compound **13** ($CDCl_3$, 400 MHz)**:**

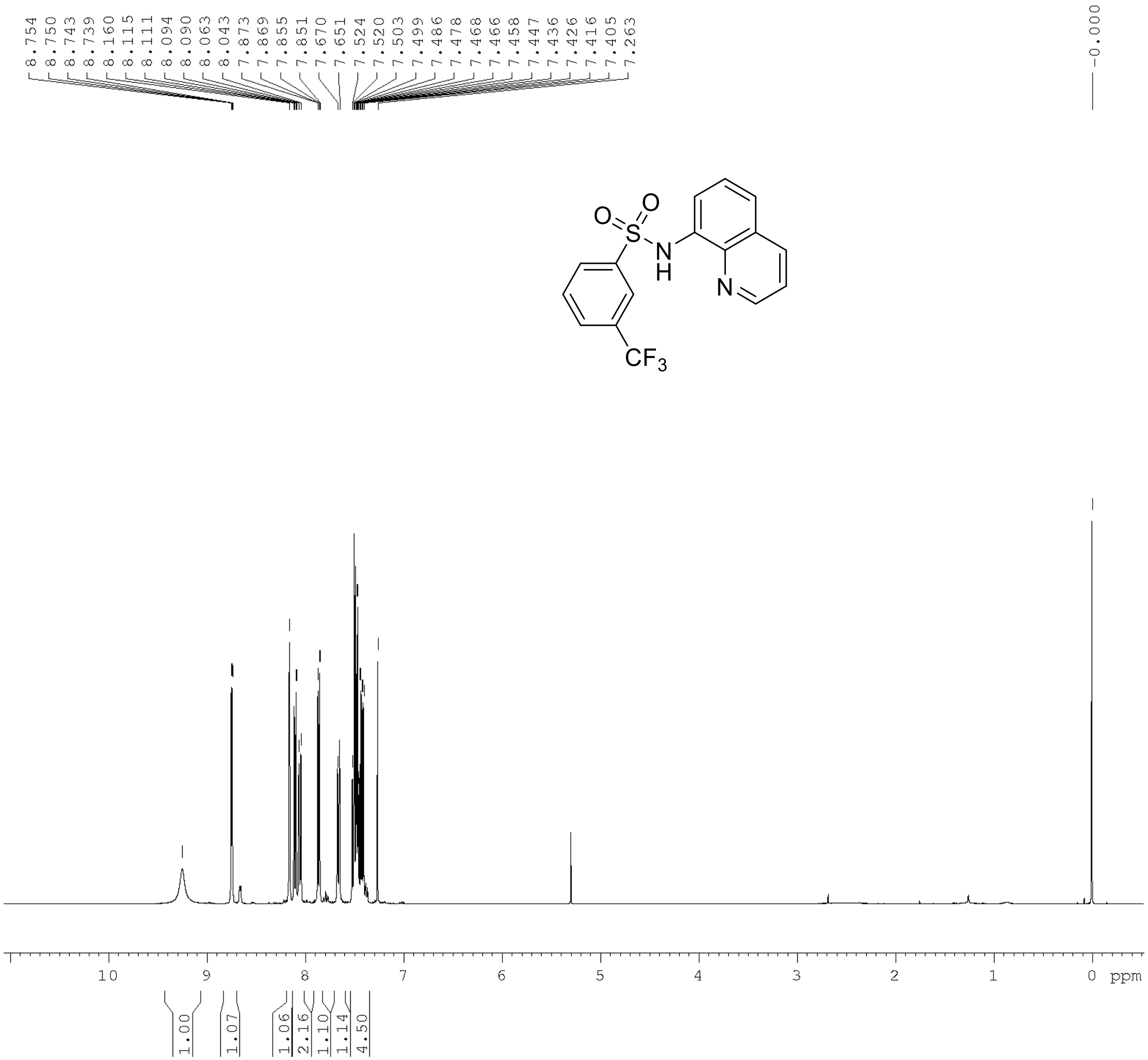

$^{13}C\{^1H\}$NMR spectrum of compound **13** ($CDCl_3$, 100 MHz):

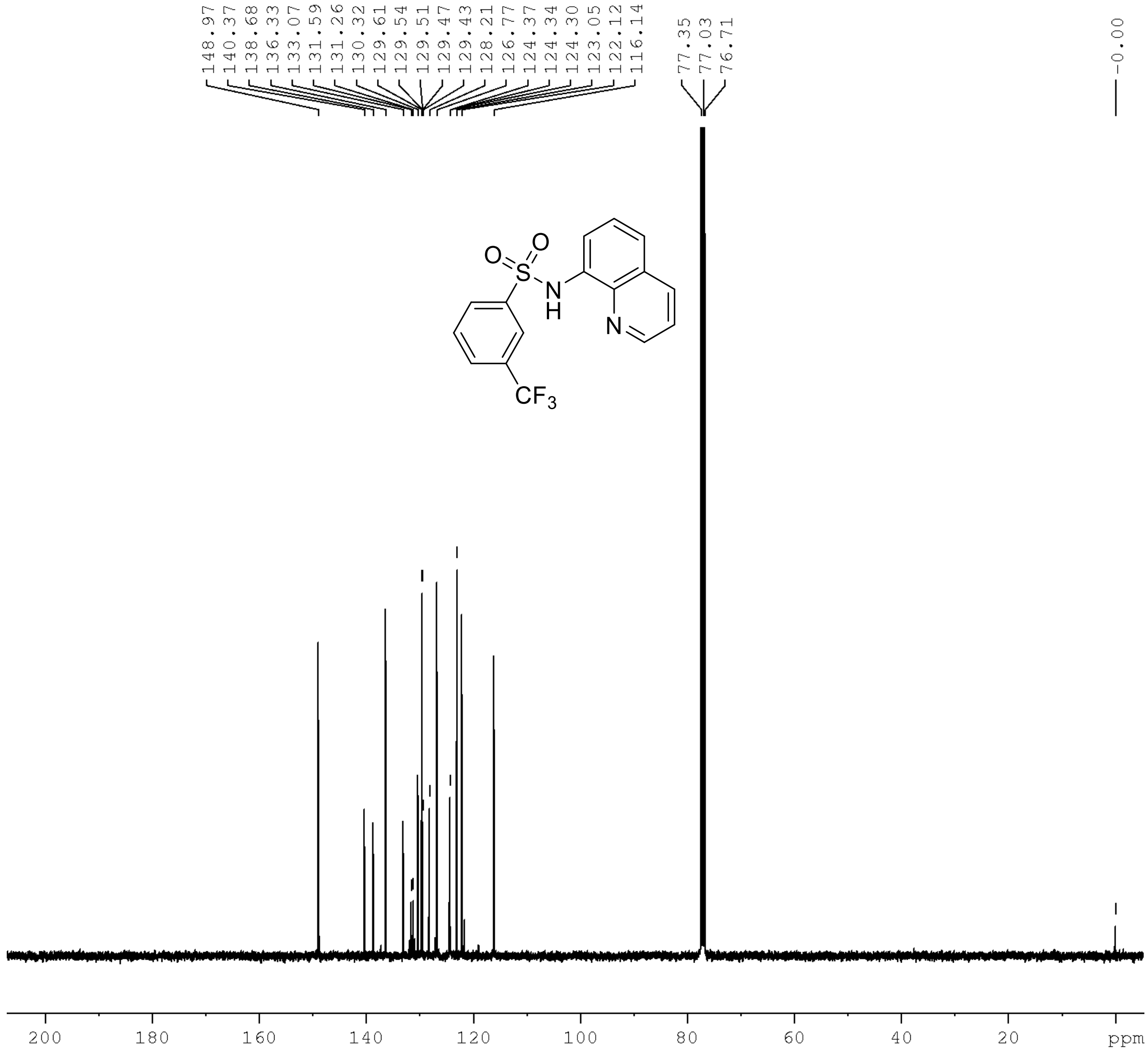

DEPT135 spectrum of compound **13** ($CDCl_3$, 100 MHz)**:**

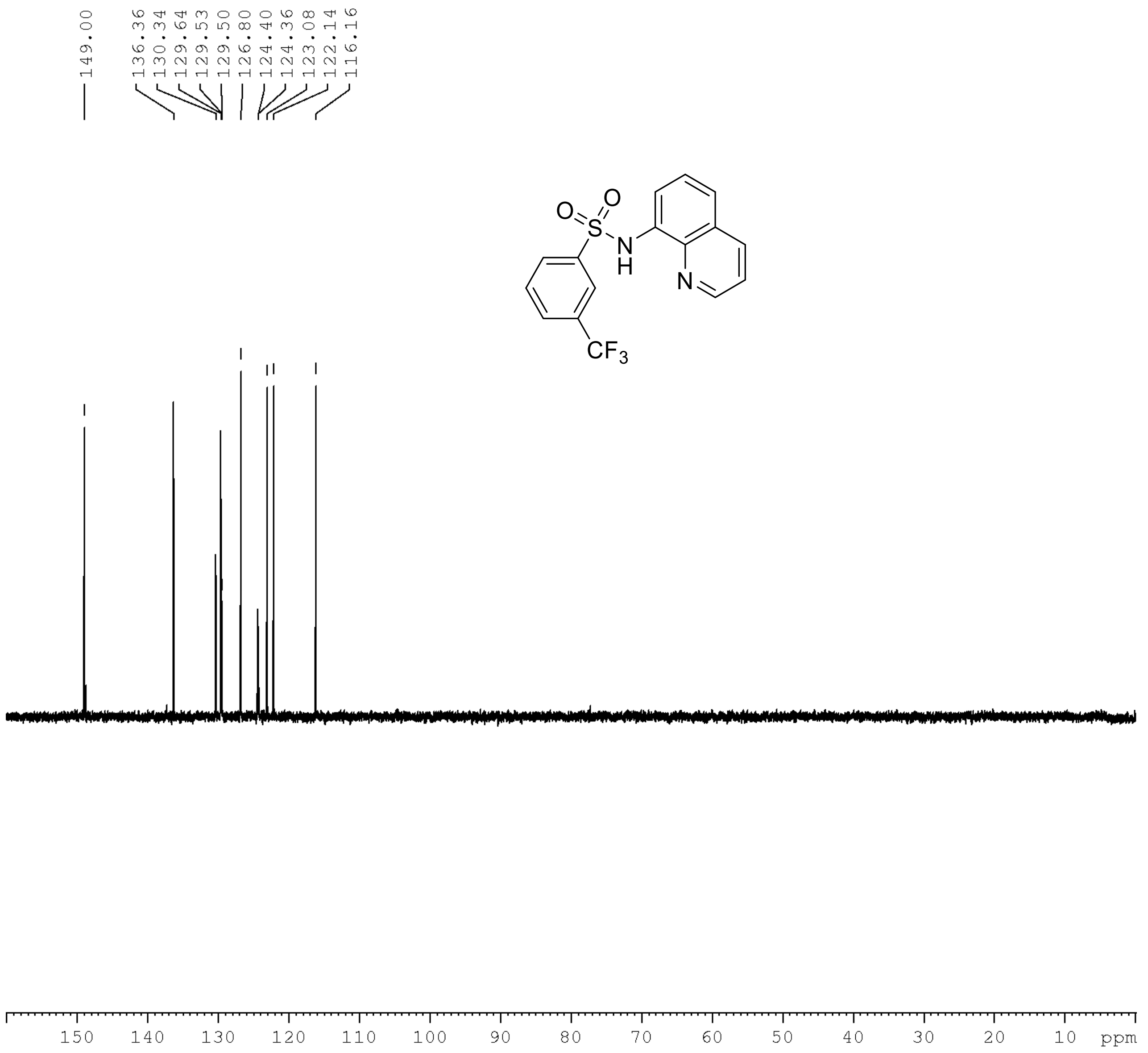

^{1}H NMR spectrum of compound **15** ($CDCl_3$, 400 MHz)**:**

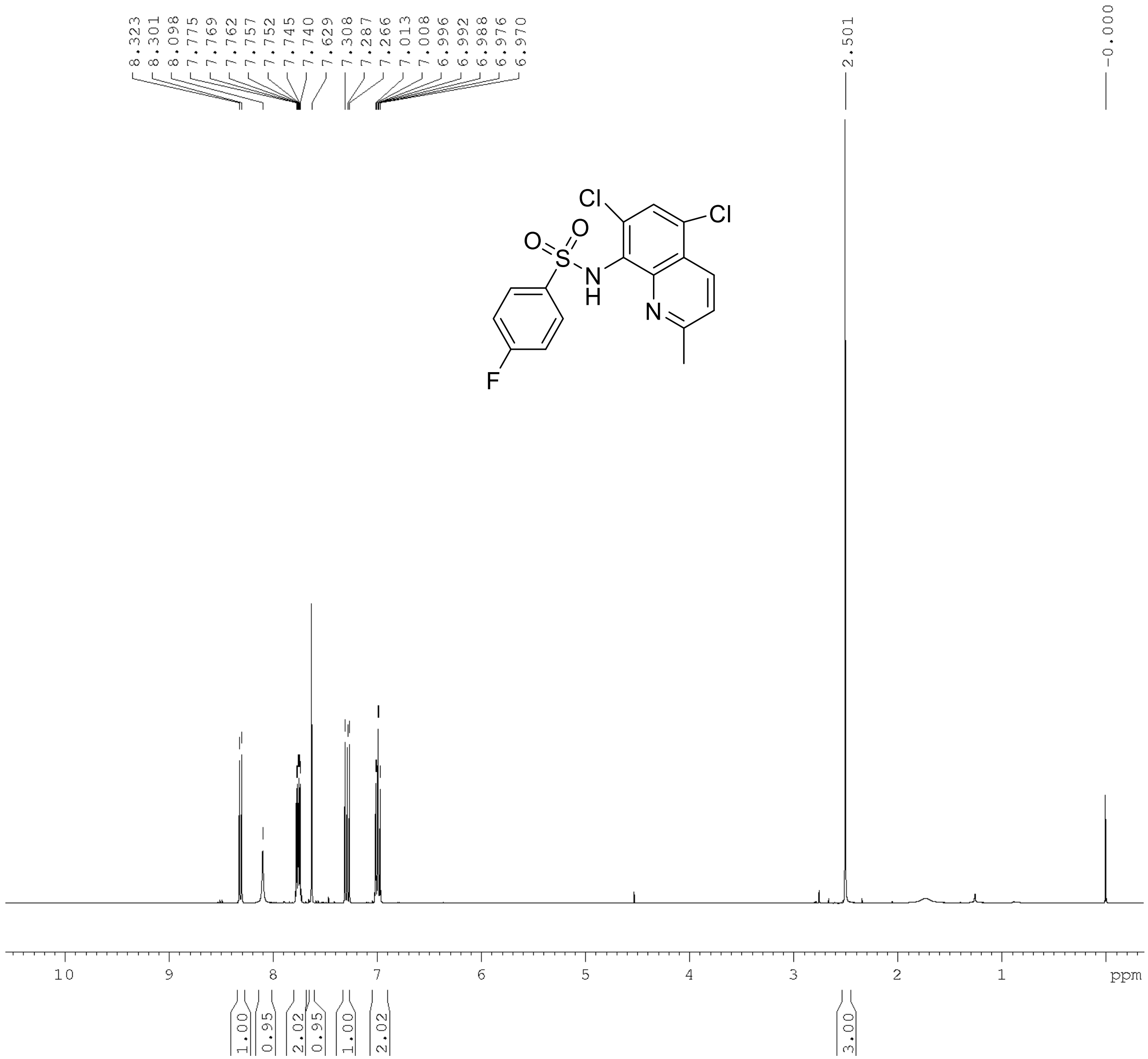

$^{13}C\{^1H\}$NMR spectrum of compound **15** ($CDCl_3$, 100 MHz)**:**

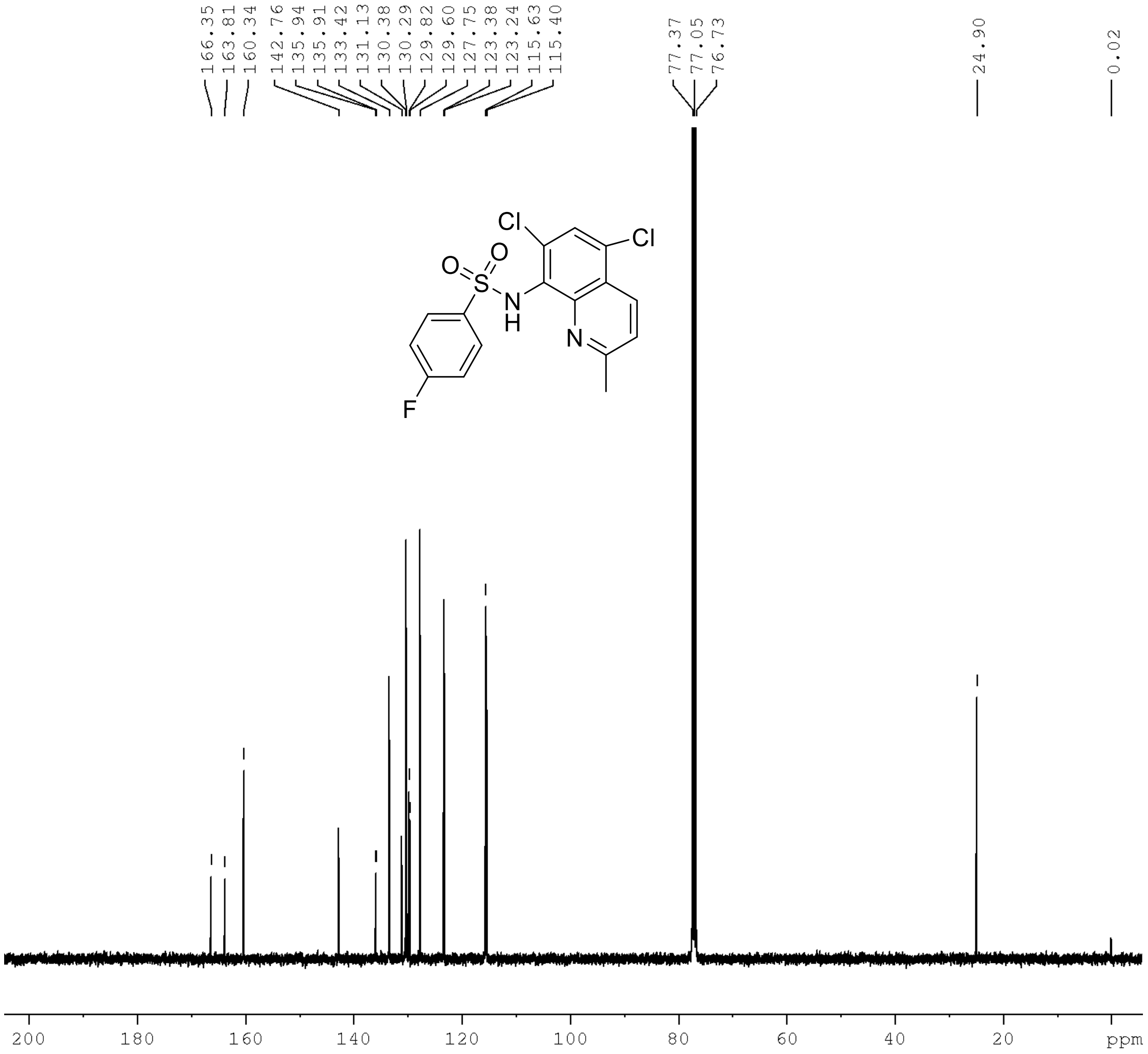

DEPT135 spectrum of compound **15** ($CDCl_3$, 100 MHz):

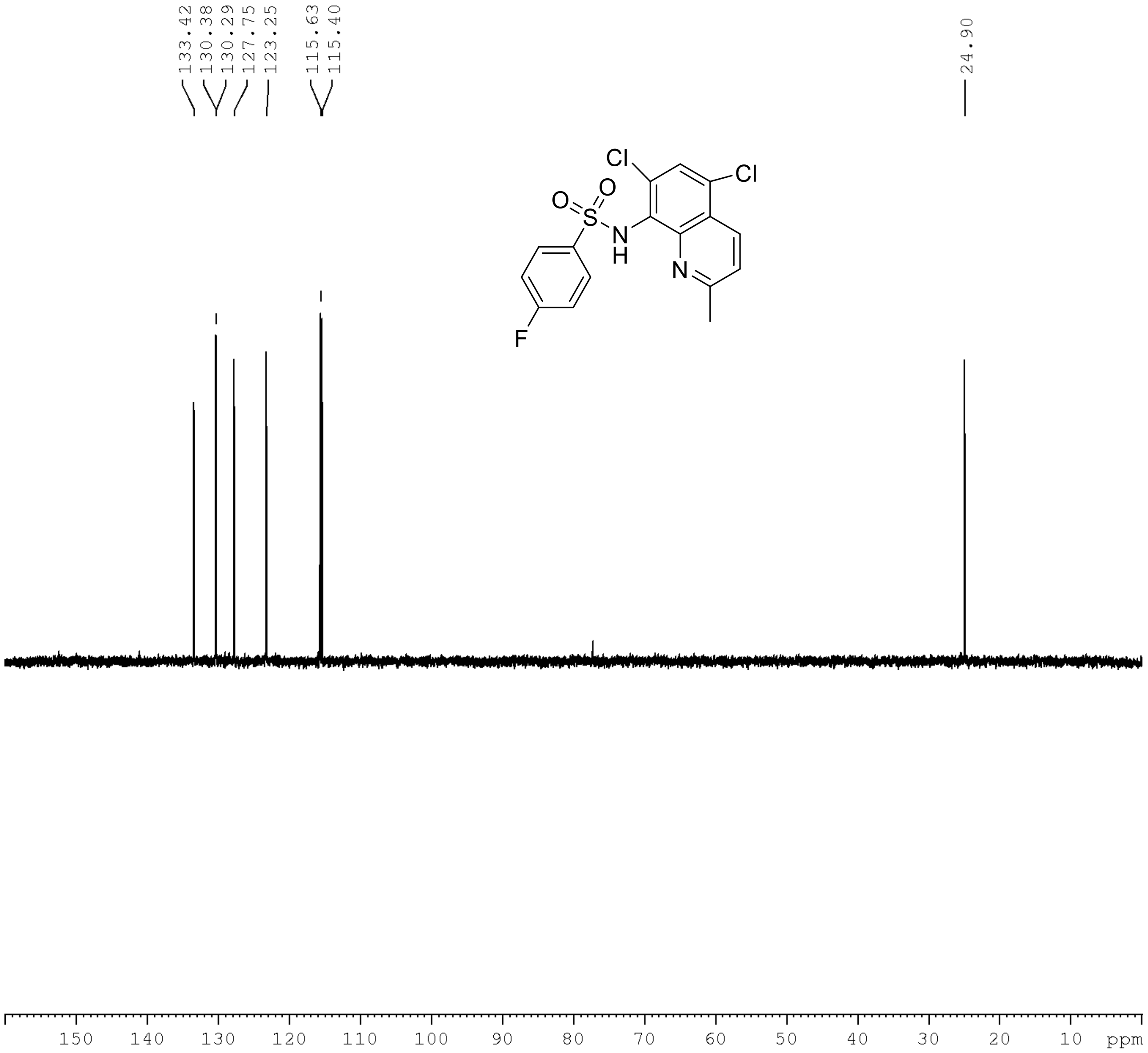

^{1}H NMR spectrum of compound **A** ($CDCl_3$, 400 MHz)**:**

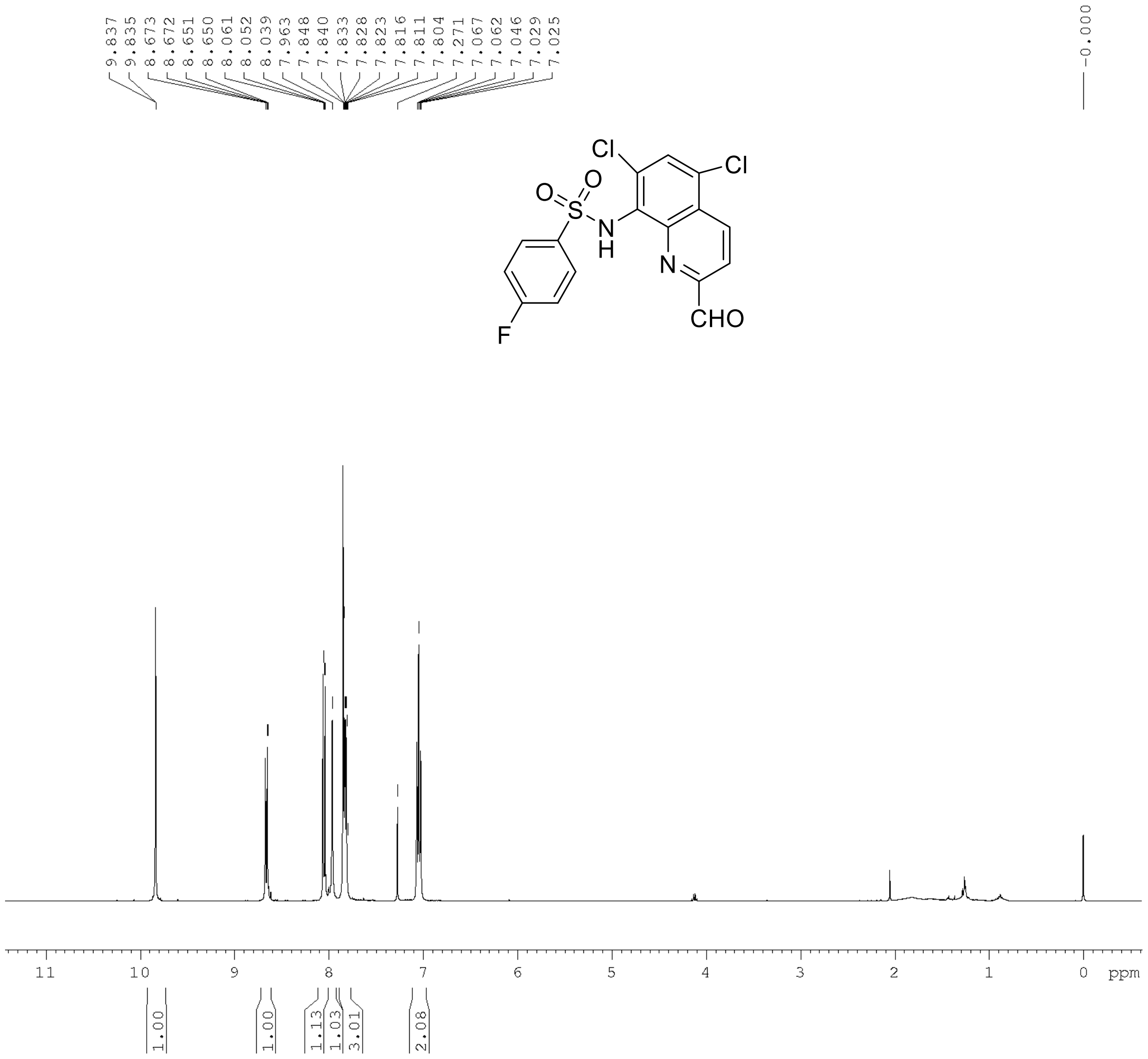

$^{13}C\{^{1}H\}$NMR spectrum of compound **A** ($CDCl_3$, 100 MHz)**:**

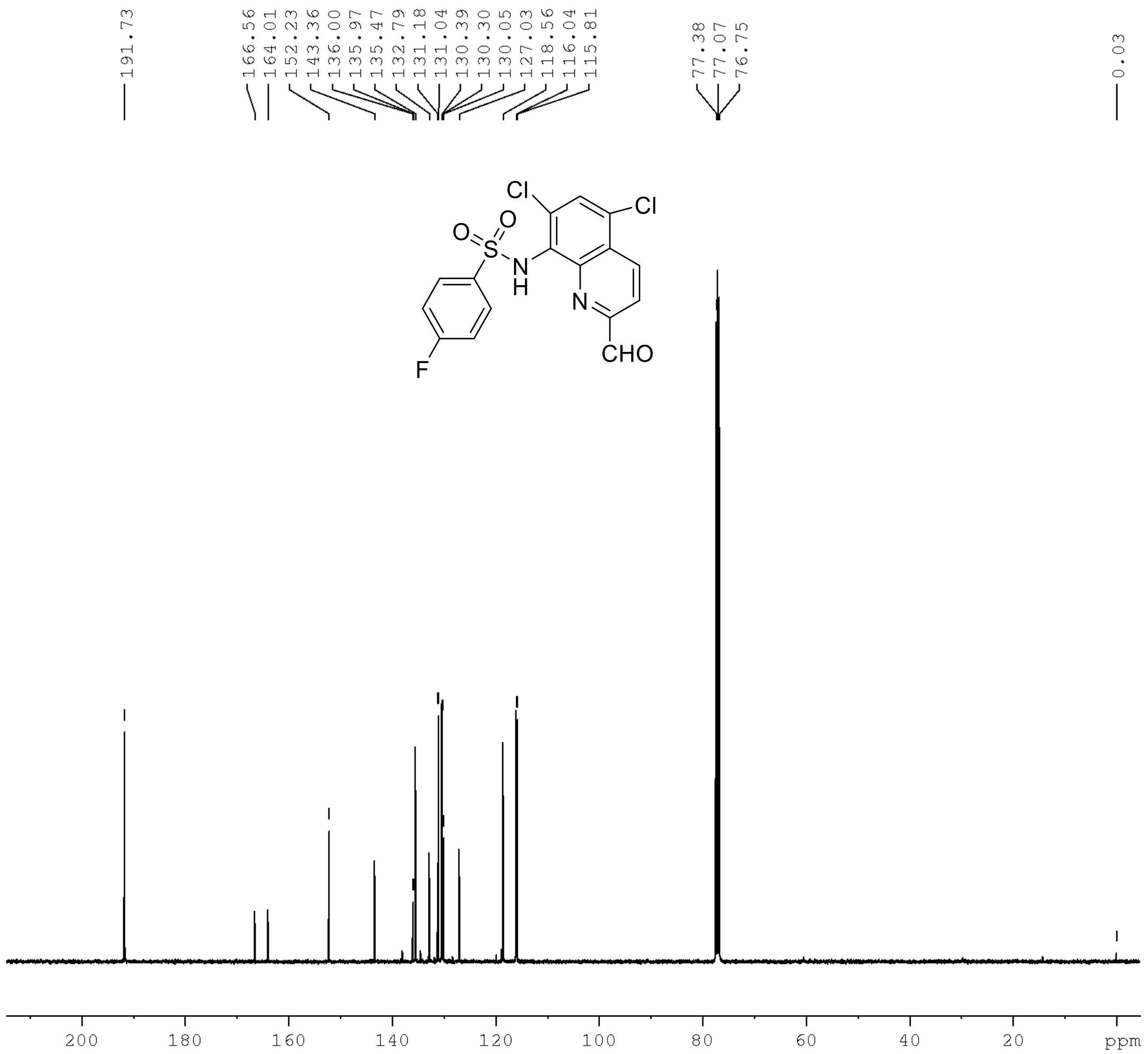

DEPT135 spectrum of compound **A** ($CDCl_3$, 100 MHz)**:**

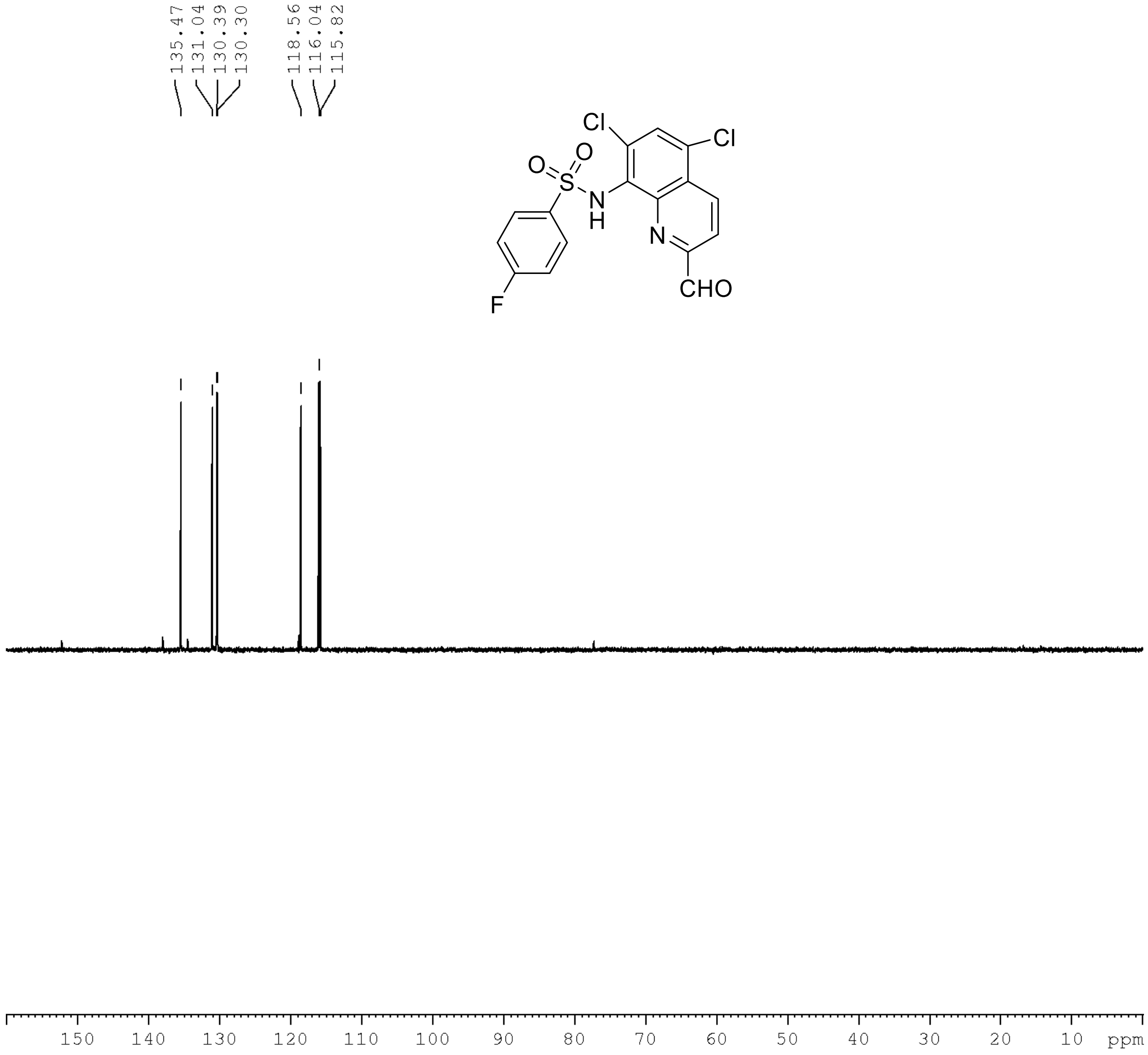

^{19}F spectrum of compound **A** ($CDCl_3$, 100 MHz)**:**

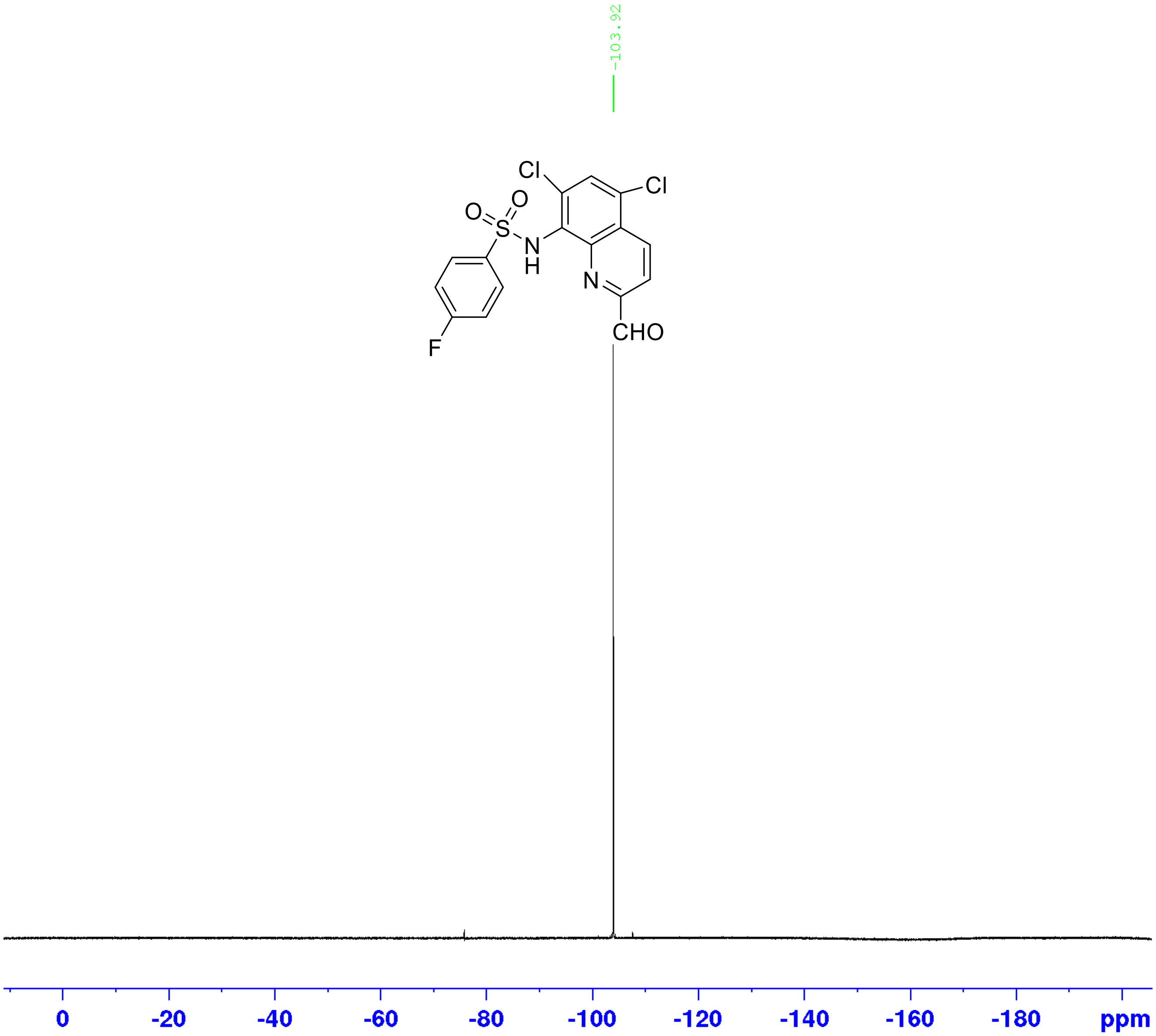

HRMS spectrum of compound **A:**

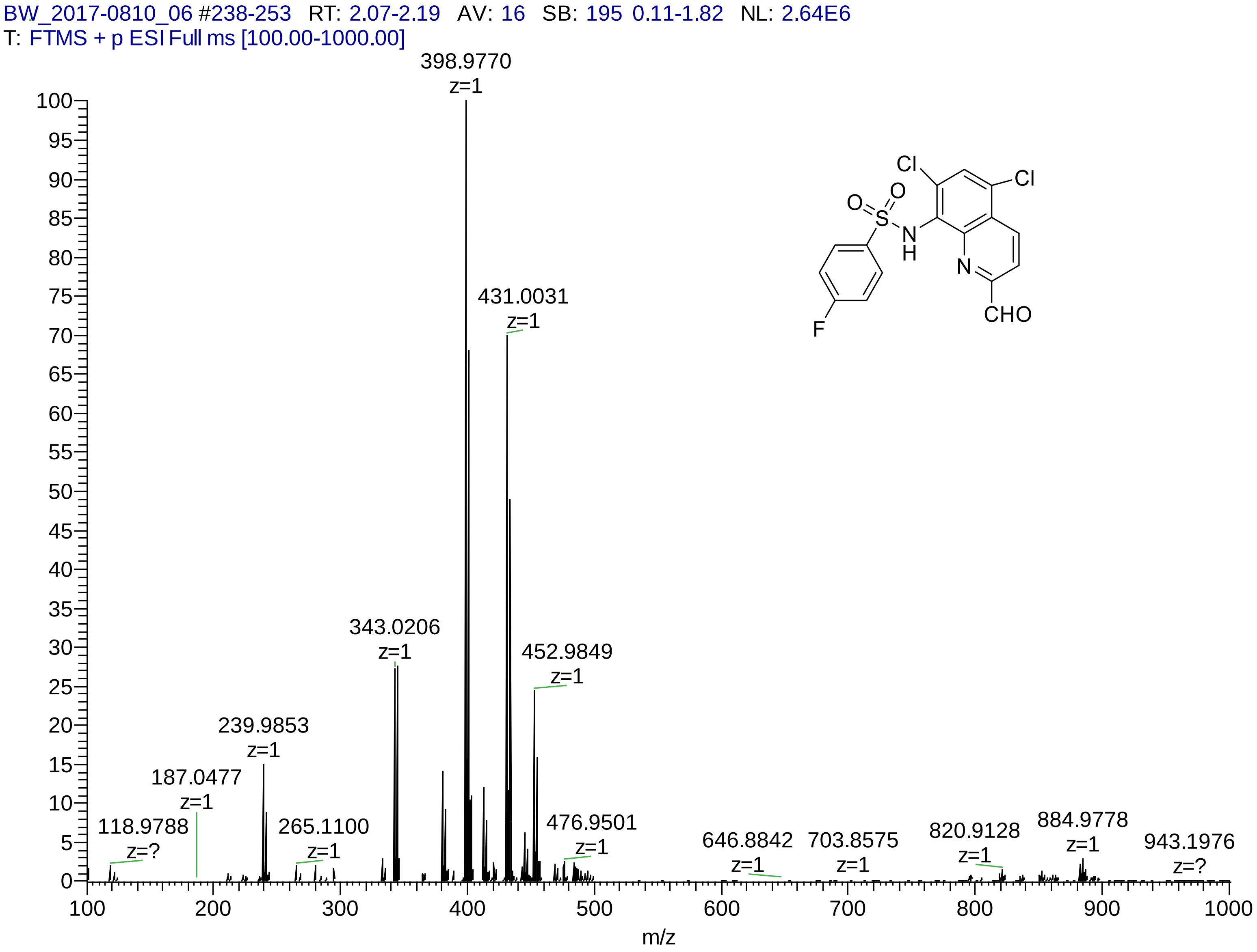

^{1}H NMR spectrum of compound **16** ($CDCl_3$, 400 MHz)**:**

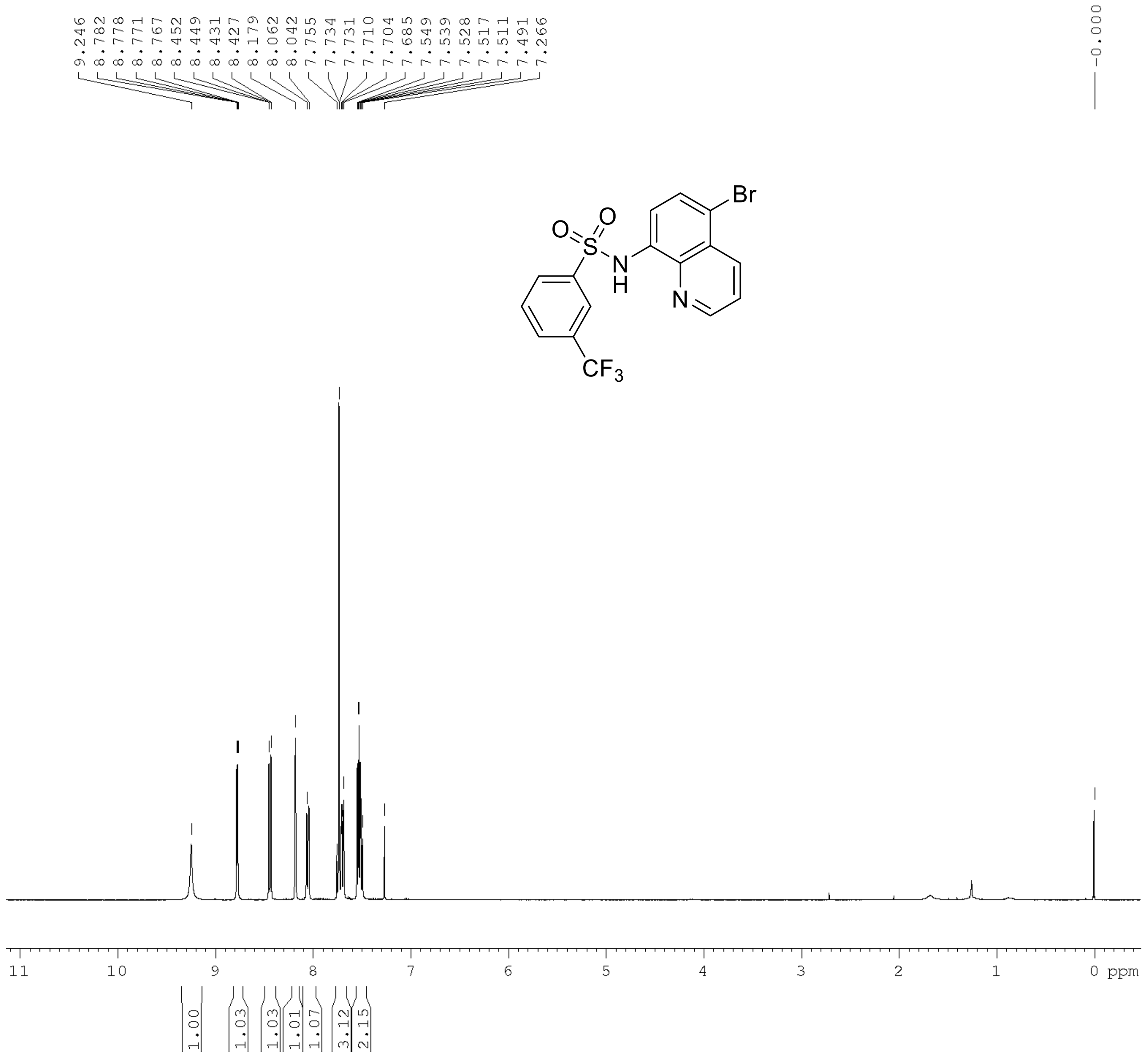

$^{13}C\{^{1}H\}$NMR spectrum of compound **16** ($CDCl_3$, 100 MHz)**:**

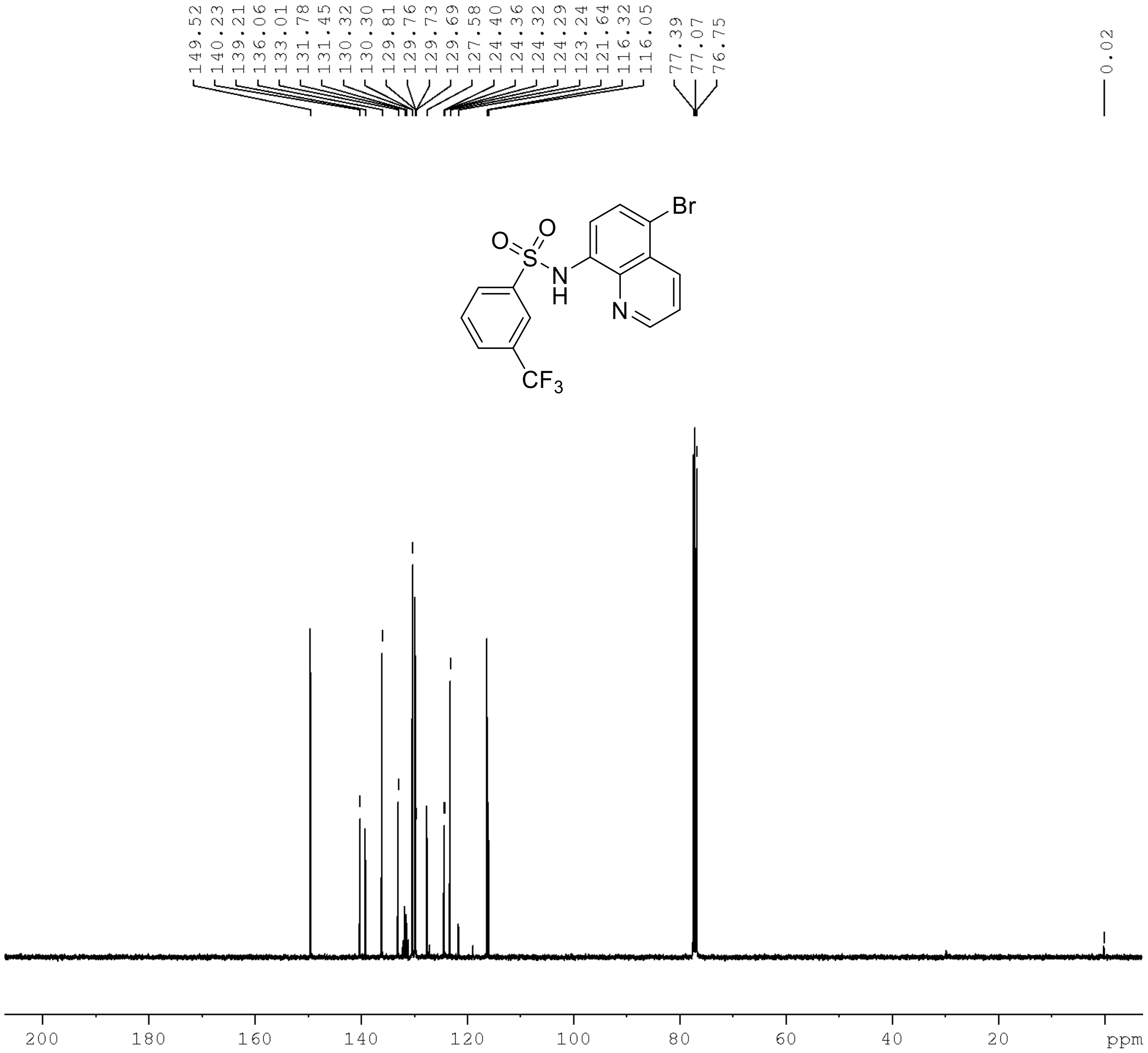

DEPT135 spectrum of compound **16** ($CDCl_3$, 100 MHz)**:**

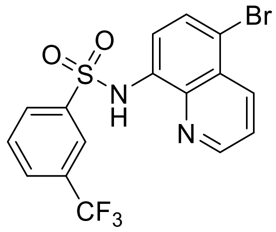

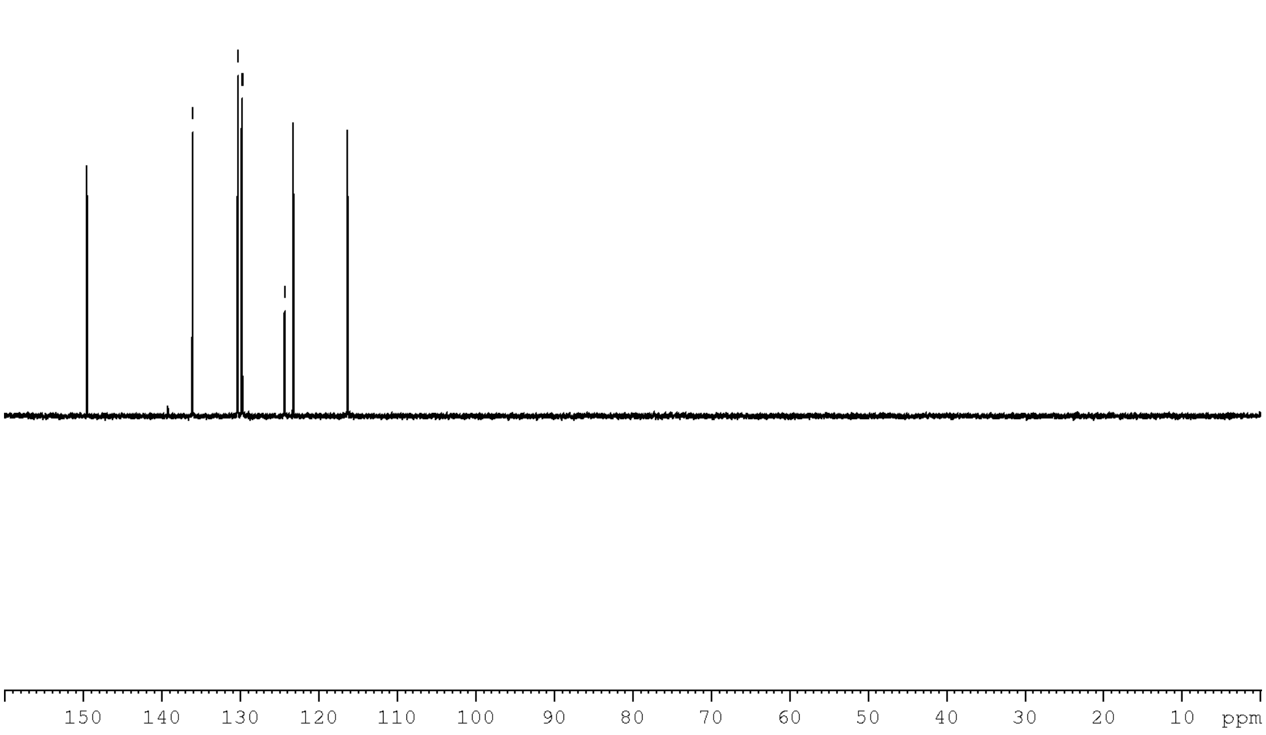

^{1}H NMR spectrum of compound **B** ($CDCl_3$, 400 MHz):

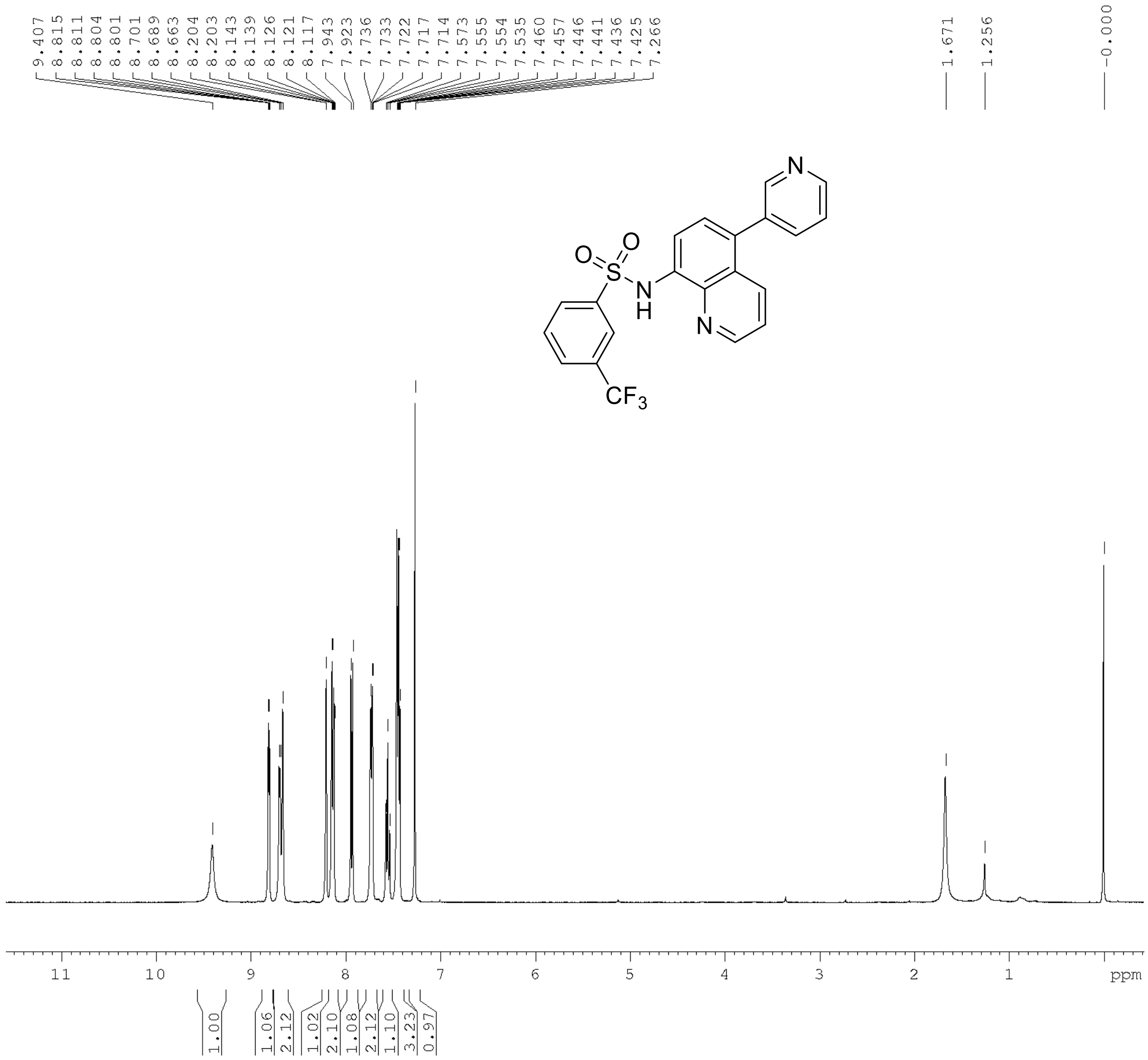

$^{13}C\{^{1}H\}$NMR spectrum of compound **B** ($CDCl_3$, 100 MHz):

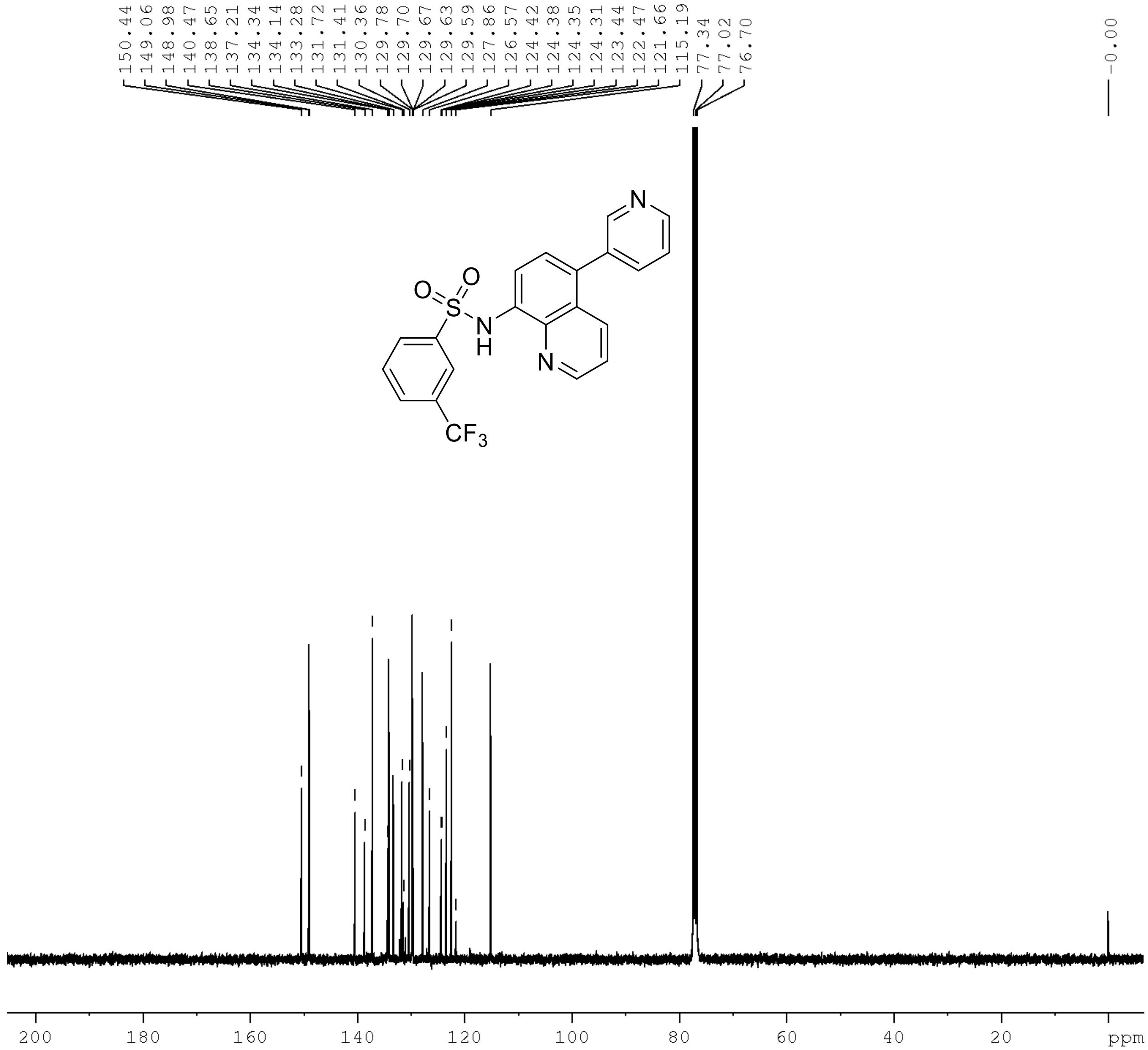

DEPT135 spectrum of compound **B** ($CDCl_3$, 100 MHz):

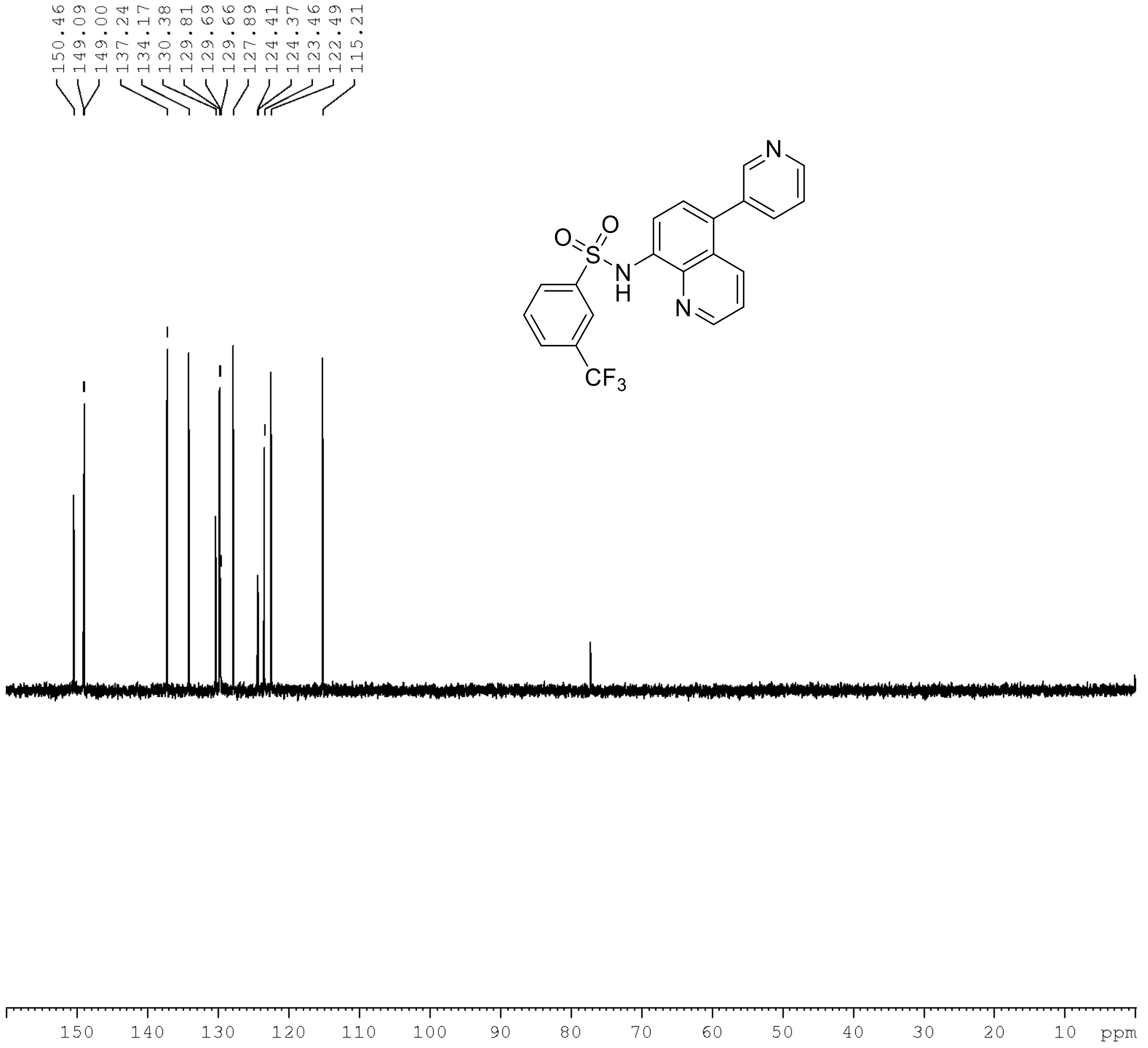

^{1}H NMR spectrum of compound **18a** ($CDCl_3$, 400 MHz)**:**

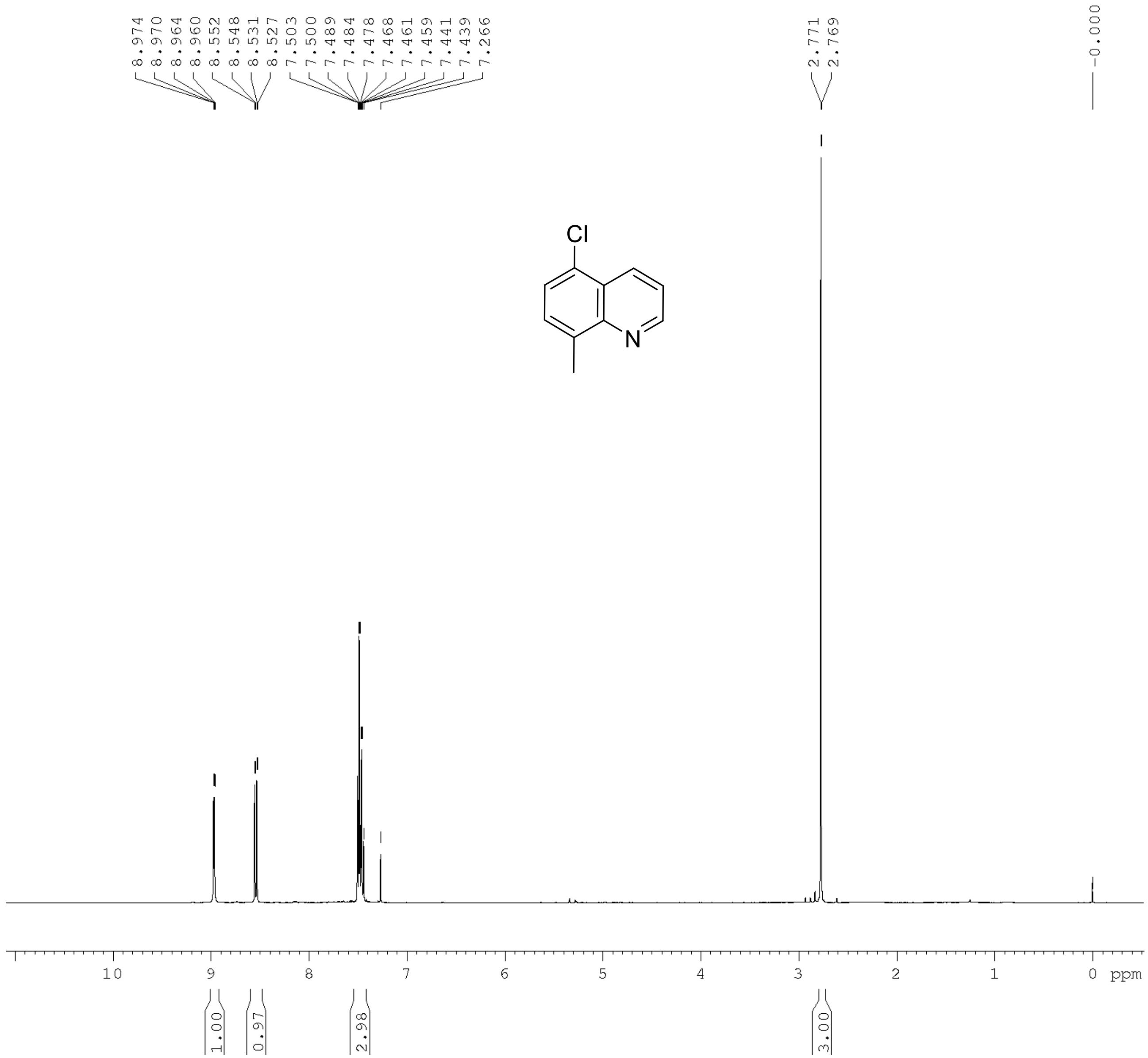

$^{13}C\{^1H\}$NMR spectrum of compound **18a** ($CDCl_3$, 100 MHz)**:**

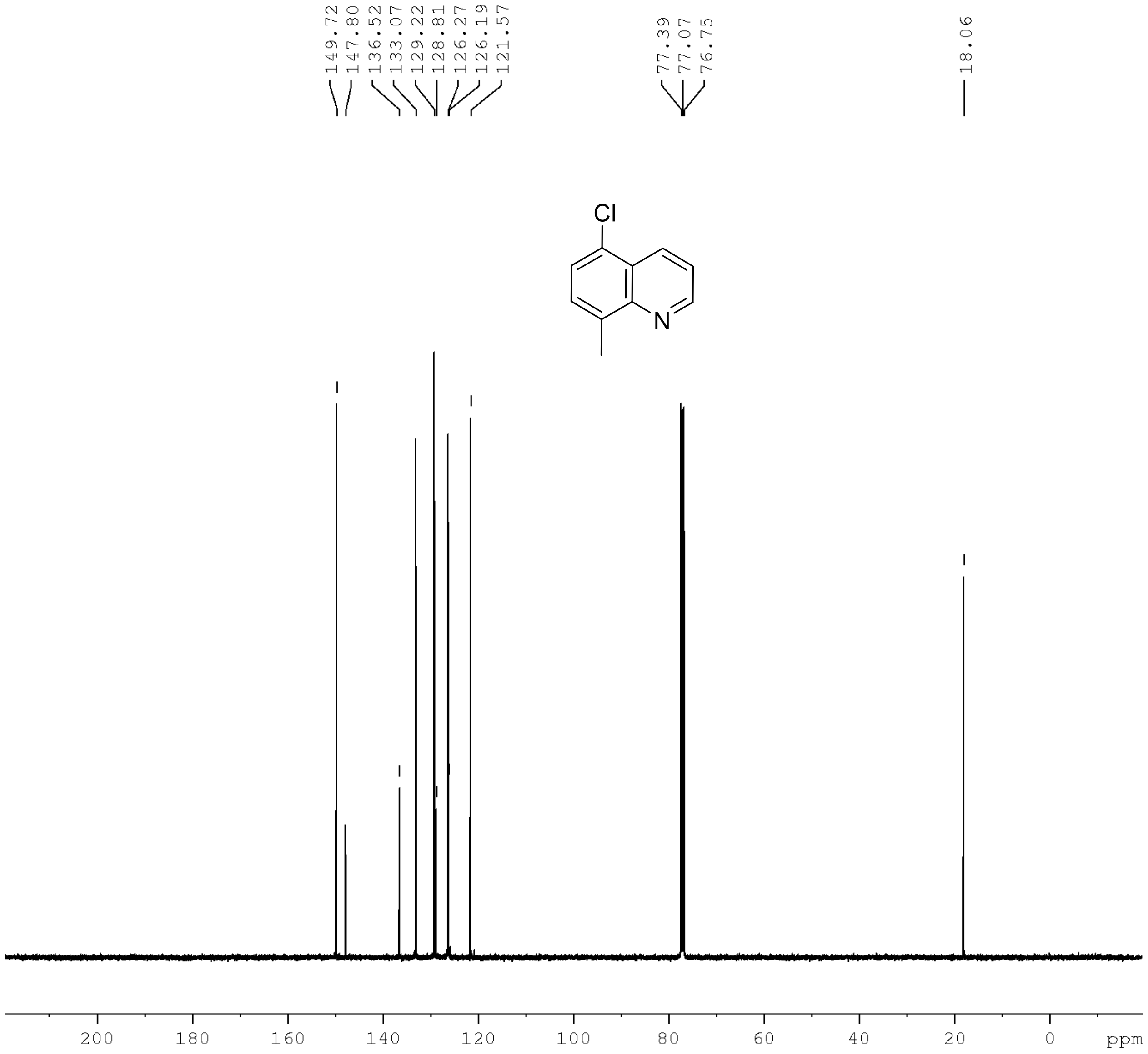

^{1}H NMR spectrum of compound **18b** ($CDCl_3$, 400 MHz)**:**

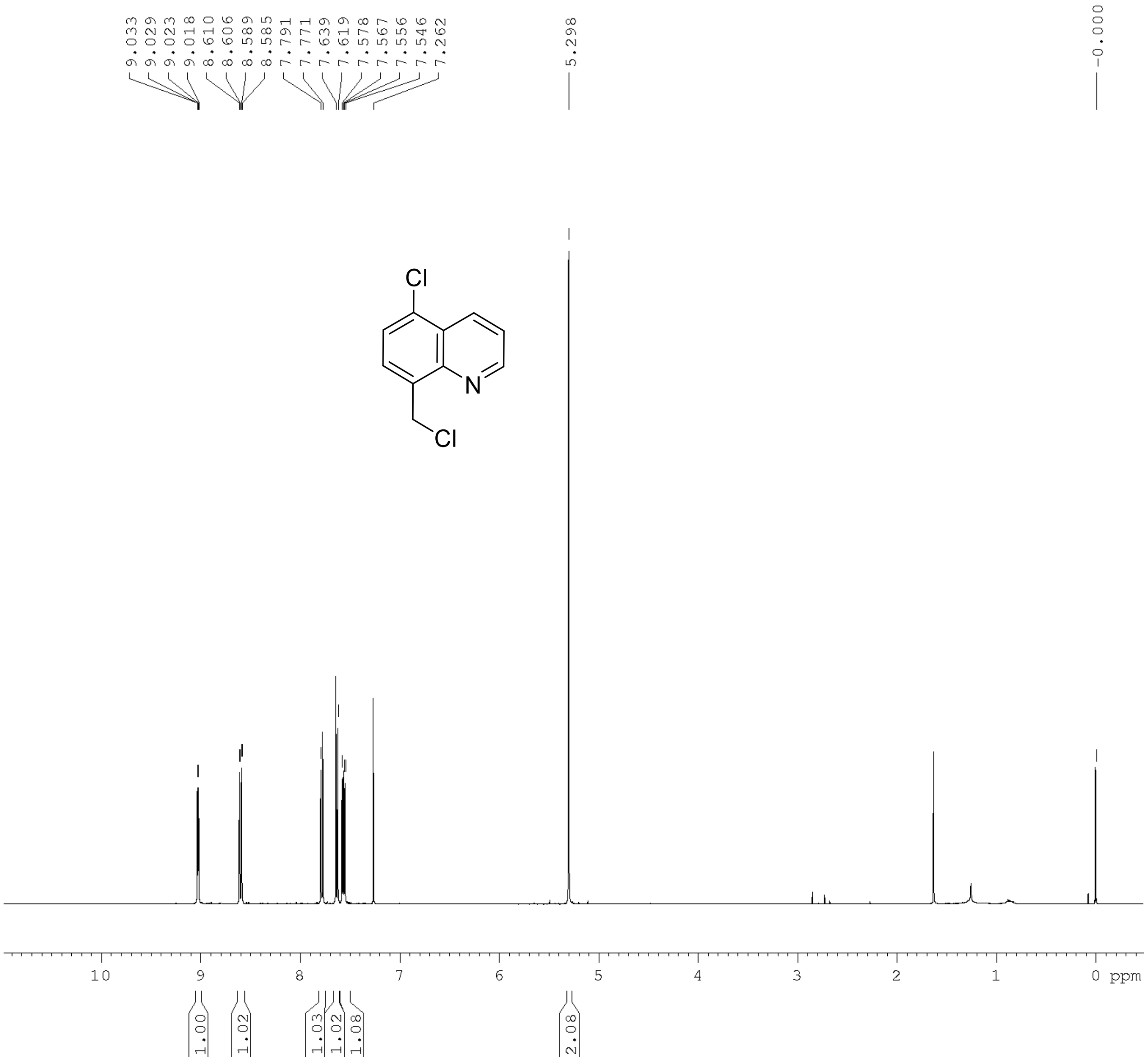

$^{13}C\{^{1}H\}$NMR spectrum of compound **18b** ($CDCl_3$, 100 MHz)**:**

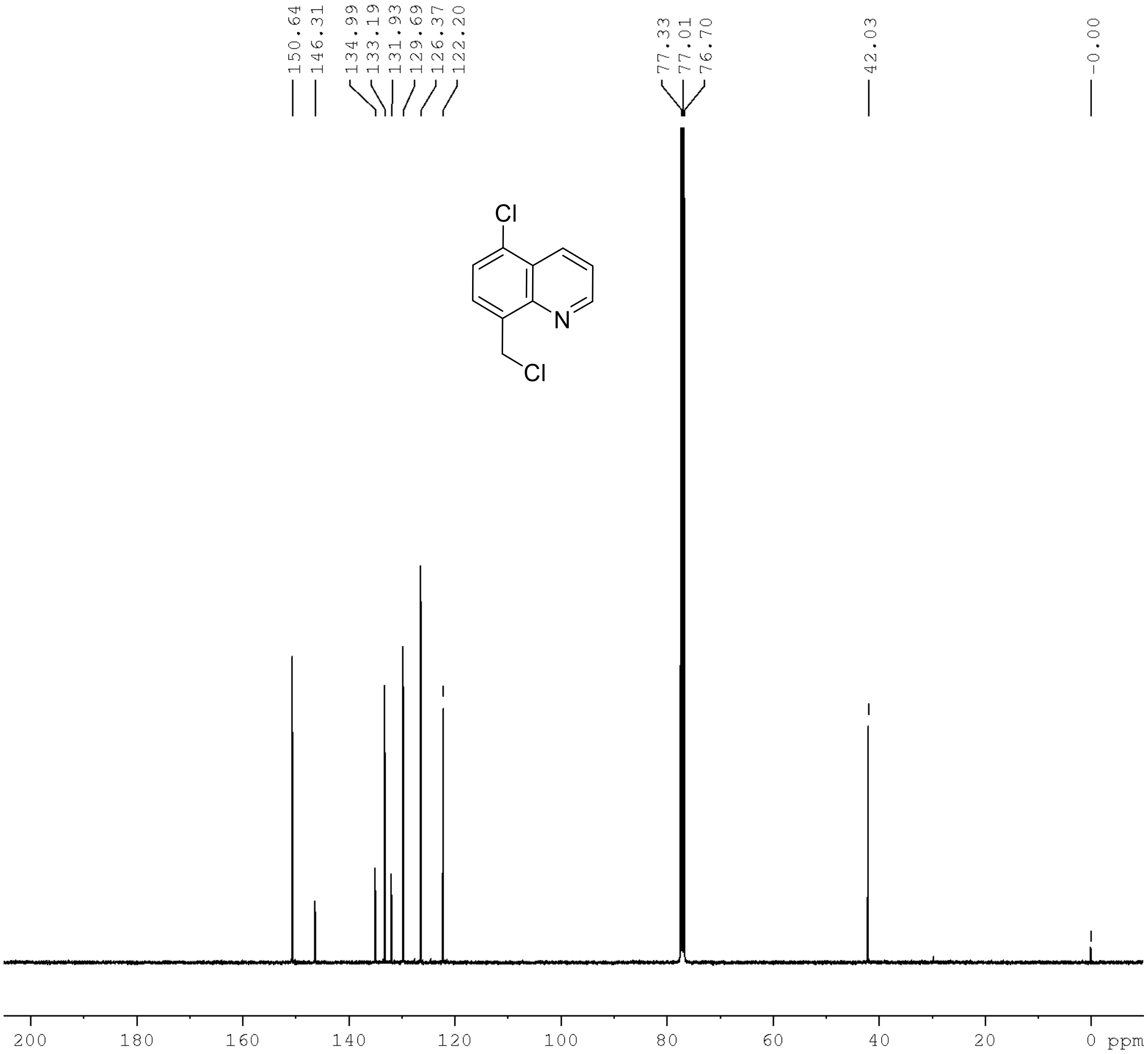

DEPT135 spectrum of compound **18b** ($CDCl_3$, 100 MHz)**:**

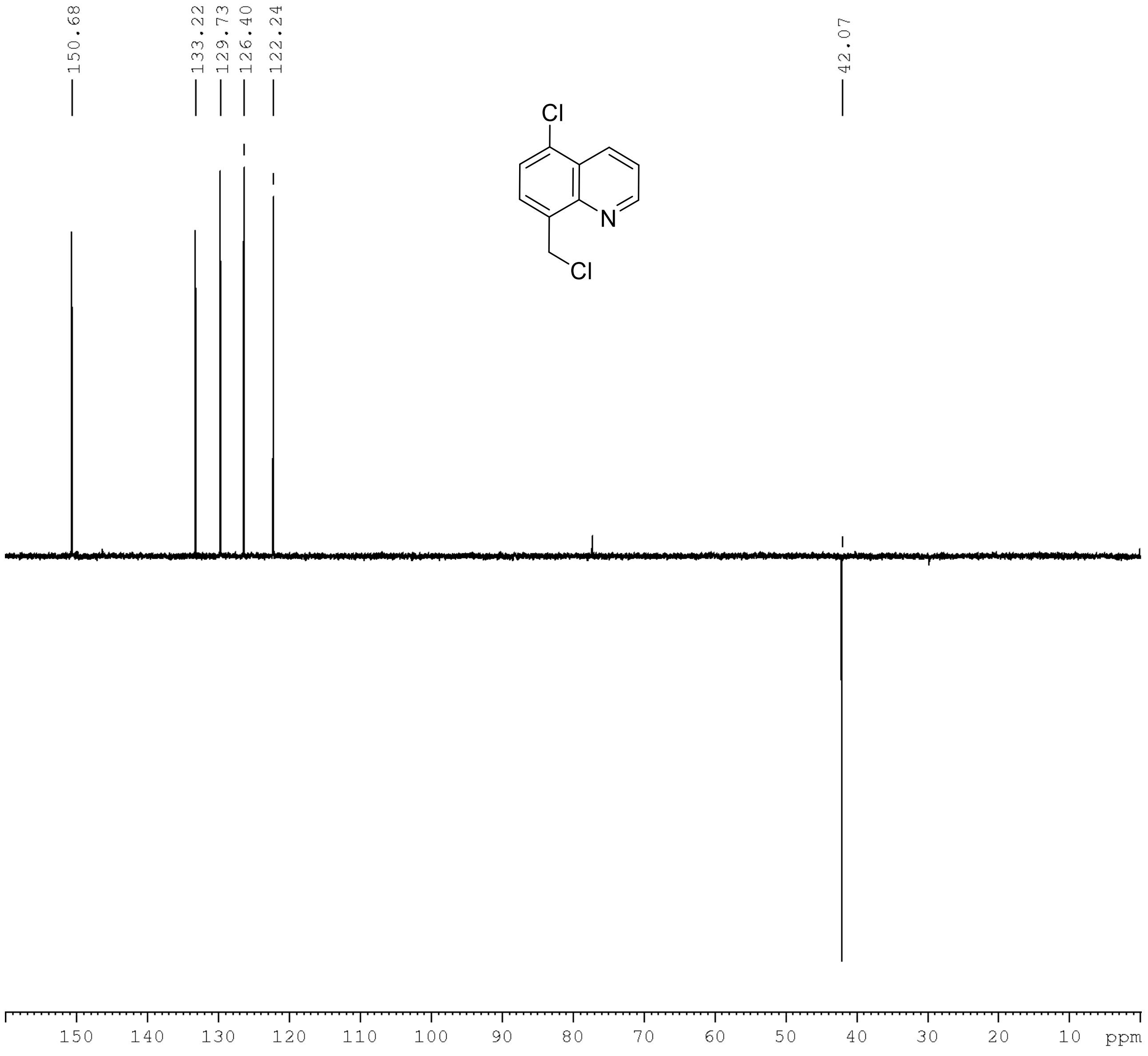

^{1}H NMR spectrum of compound **19a** ($CDCl_3$, 400 MHz)**:**

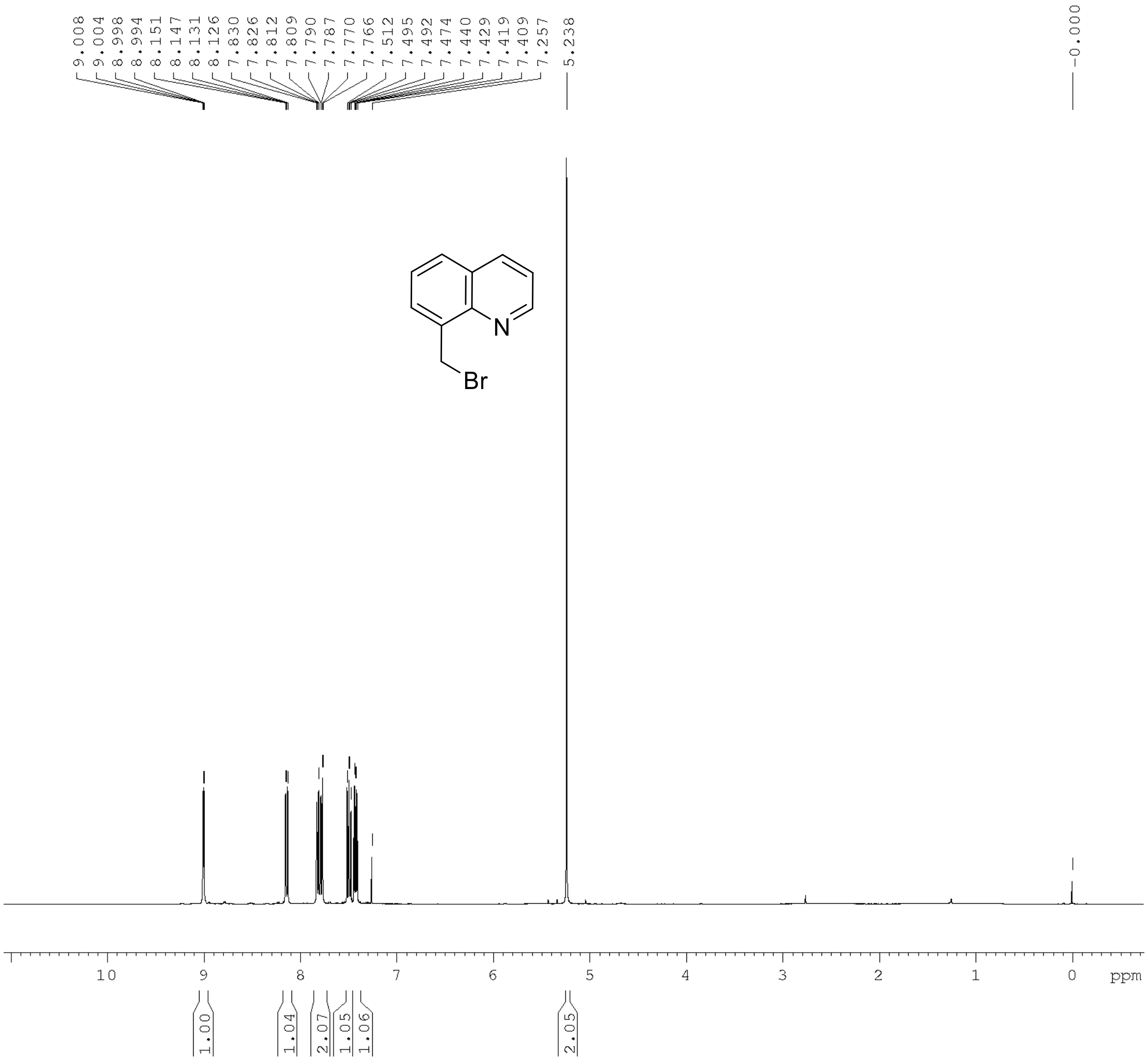

$^{13}C\{^1H\}$NMR spectrum of compound **19a** ($CDCl_3$, 100 MHz)**:**

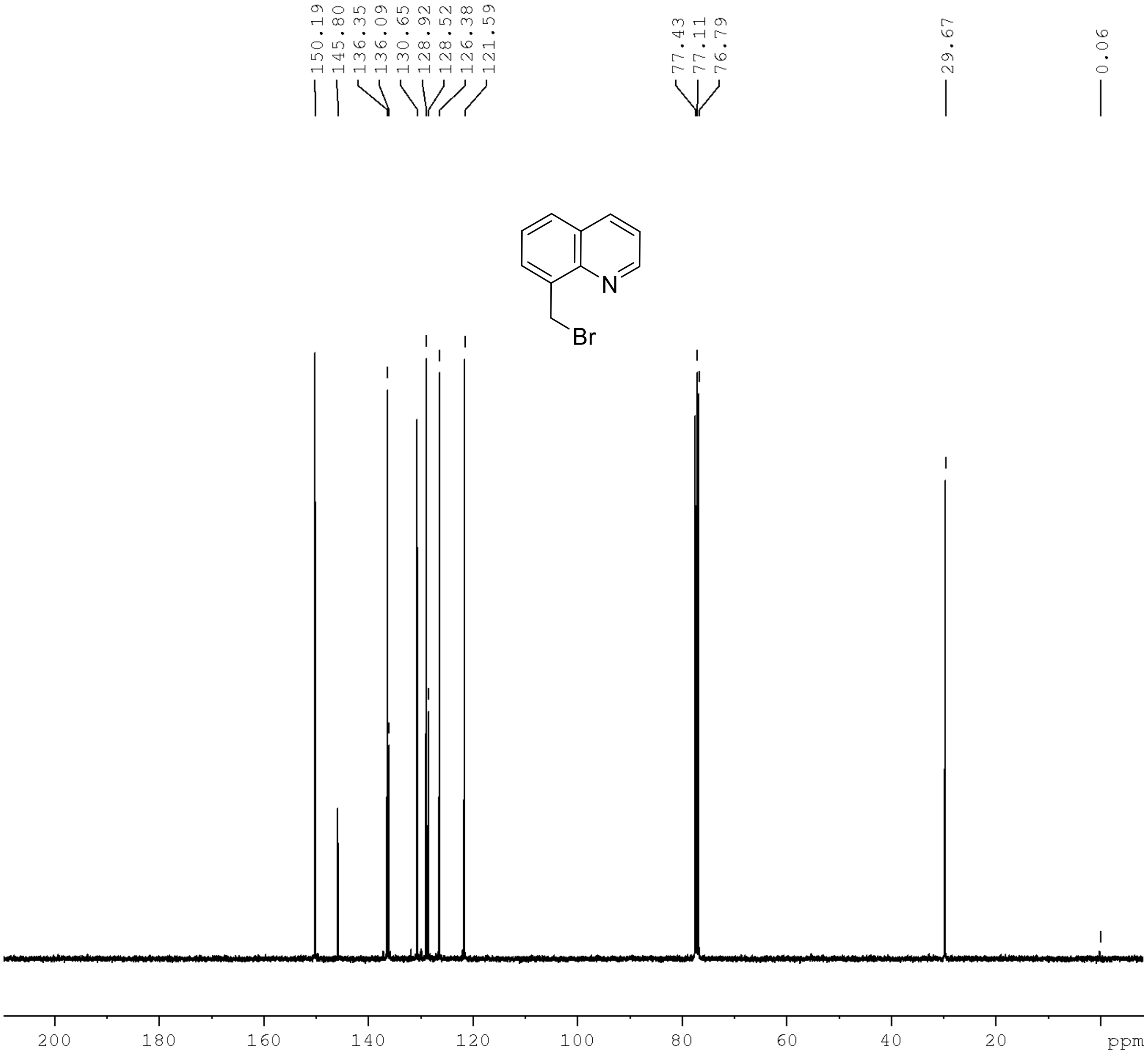

DEPT135 spectrum of compound **19a** ($CDCl_3$, 100 MHz)**:**

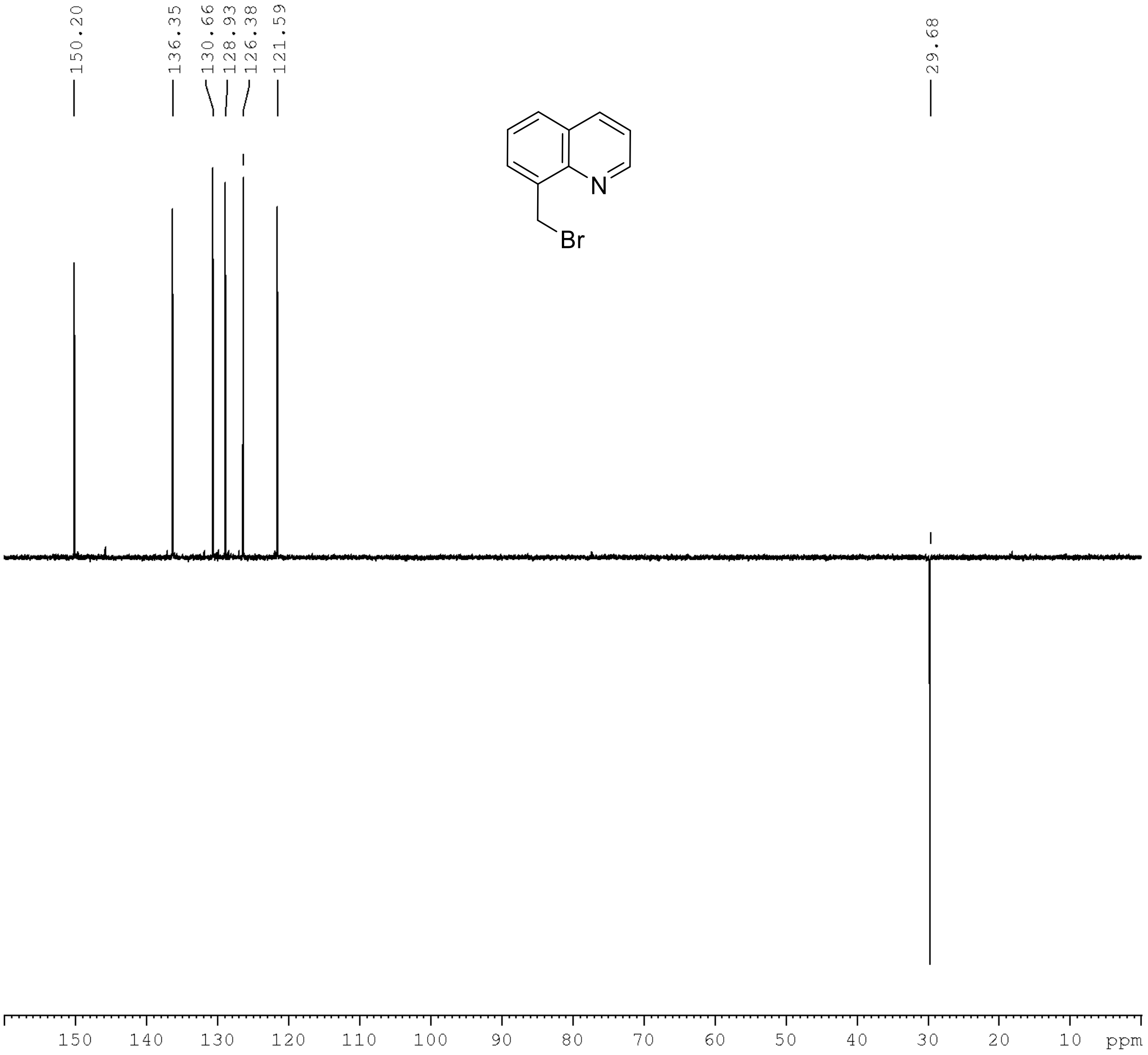

^{1}H NMR spectrum of compound **19b** ($CDCl_3$, 400 MHz)**:**

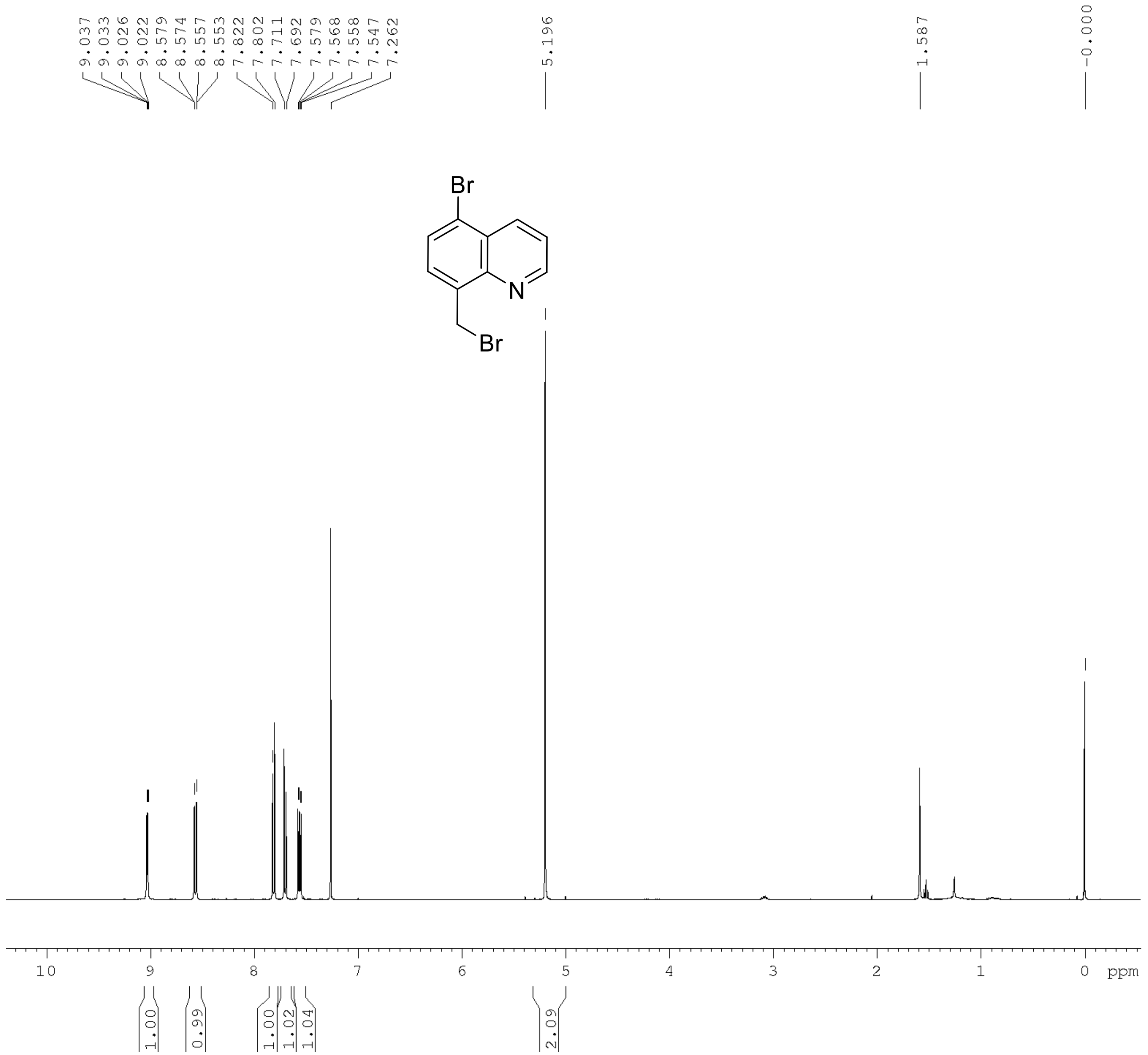

$^{13}C\{^1H\}$NMR spectrum of compound **19b** ($CDCl_3$, 100 MHz)**:**

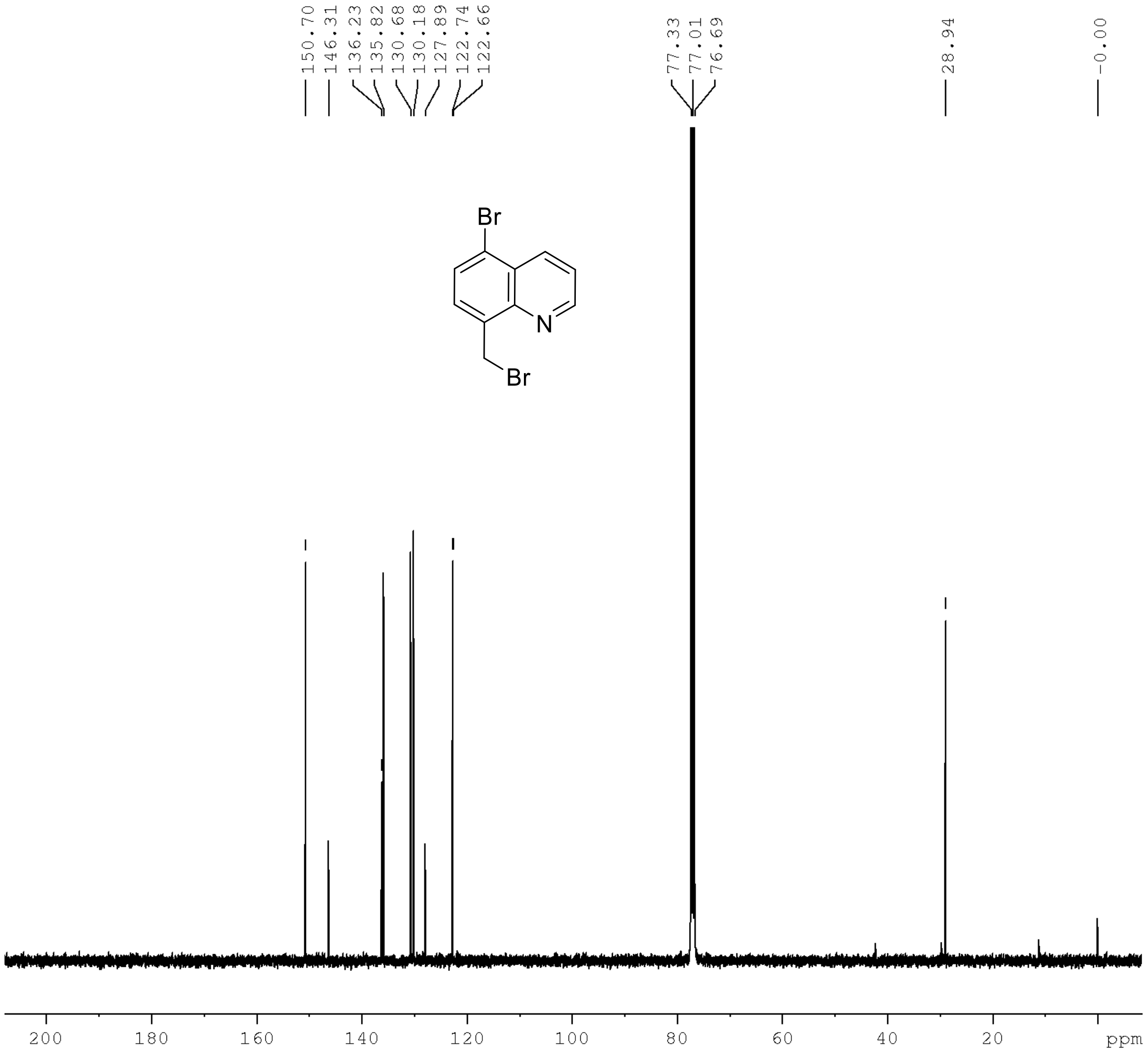

DEPT135 spectrum of compound **19b** ($CDCl_3$, 100 MHz)**:**

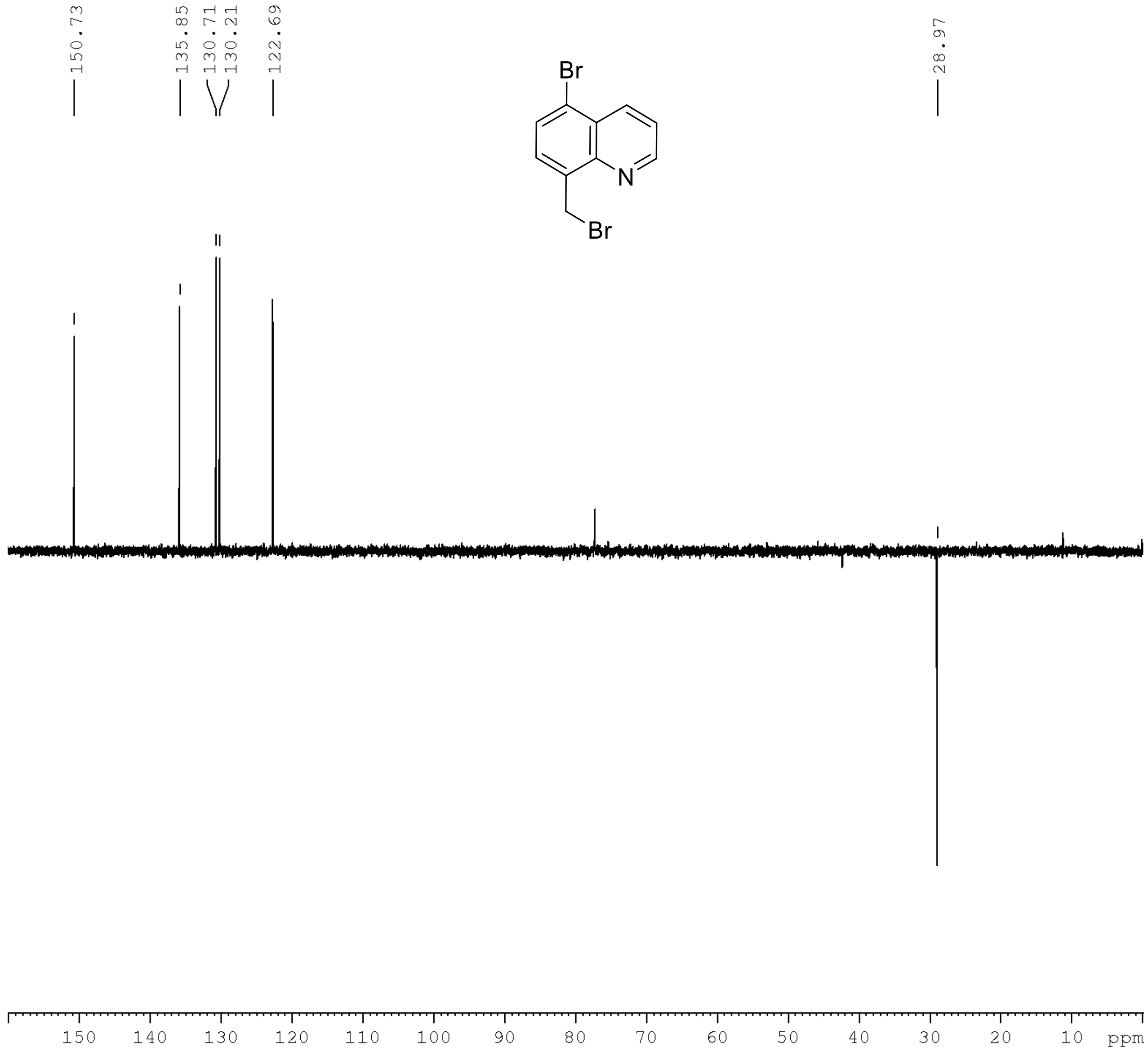

Supplement: Supplementary file 1 [file SC-009-C7SC04107A-s001.pdf]
